# Estatística Cardiovascular – Brasil 2021

**DOI:** 10.36660/abc.20211012

**Published:** 2022-01-01

**Authors:** Gláucia Maria Moraes de Oliveira, Luisa Campos Caldeira Brant, Carisi Anne Polanczyk, Deborah Carvalho Malta, Andreia Biolo, Bruno Ramos Nascimento, Maria de Fatima Marinho de Souza, Andrea Rocha De Lorenzo, Antonio Aurélio de Paiva Fagundes, Beatriz D. Schaan, Fábio Morato de Castilho, Fernando Henpin Yue Cesena, Gabriel Porto Soares, Gesner Francisco Xavier, Jose Augusto Soares Barreto, Luiz Guilherme Passaglia, Marcelo Martins Pinto, M. Julia Machline-Carrion, Marcio Sommer Bittencourt, Octavio M. Pontes, Paolo Blanco Villela, Renato Azeredo Teixeira, Roney Orismar Sampaio, Thomaz A. Gaziano, Pablo Perel, Gregory A. Roth, Antonio Luiz Pinho Ribeiro

**Affiliations:** 1 Instituto do Coração Edson Saad Universidade Federal do Rio de Janeiro Rio de Janeiro RJ Brasil - Instituto do Coração Edson Saad da Universidade Federal do Rio de Janeiro (UFRJ), Rio de Janeiro , RJ – Brasil; 2 Universidade Federal do Rio de Janeiro Rio de Janeiro RJ Brasil - Universidade Federal do Rio de Janeiro (UFRJ), Rio de Janeiro , RJ – Brasil; 3 Universidade Federal de Minas Gerais Belo Horizonte MG Brasil - Universidade Federal de Minas Gerais (UFMG) Belo Horizonte , MG – Brasil; 4 Hospital das Clínicas Universidade Federal de Minas Gerais Belo Horizonte MG Brasil - Hospital das Clínicas da Universidade Federal de Minas Gerais (UFMG) Belo Horizonte , MG – Brasil; 5 Universidade Federal do Rio Grande do Sul Porto Alegre RS Brasil - Universidade Federal do Rio Grande do Sul (UFRS), Porto Alegre , RS – Brasil; 6 Hospital Moinhos de Vento Porto Alegre RS Brasil - Hospital Moinhos de Vento , Porto Alegre , RS – Brasil; 7 Hospital de Clínicas de Porto Alegre Porto Alegre RS Brasil - Hospital de Clínicas de Porto Alegre (HCPA), Porto Alegre , RS – Brasil; 8 Vital Strategies Nova York EUA - Vital Strategies , Nova York – EUA; 9 Instituto Nacional de Cardiologia Rio de Janeiro RJ Brasil - Instituto Nacional de Cardiologia , Rio de Janeiro , RJ – Brasil; 10 Brigham and Women’s Hospital Boston EUA - Brigham and Women’s Hospital , Boston – EUA; 11 Hospital Israelita Albert Einstein São Paulo SP Brasil - Hospital Israelita Albert Einstein , São Paulo , SP – Brasil; 12 Curso de Medicina Universidade de Vassouras Vassouras RJ Brasil - Curso de Medicina da Universidade de Vassouras , Vassouras , RJ – Brasil; 13 Universidade Federal de Sergipe Aracaju SE Brasil - Universidade Federal de Sergipe , Aracaju , SE – Brasil; 14 Hospital São Lucas Rede São Luiz D’Or Aracaju SE Brasil - Hospital São Lucas Rede São Luiz D’Or , Aracaju , SE – Brasil; 15 ePHealth Primary Care Solutions Santo Antônio SC Brasil - ePHealth Primary Care Solutions , Santo Antônio , SC – Brasil; 16 Department of Medicine and Radiology University of Pittsburgh Pittsburgh EUA - Department of Medicine and Radiology University of Pittsburgh , Pittsburgh – EUA; 17 Faculdade de Medicina de Ribeirão Preto Universidade de São Paulo São Paulo SP Brasil - Faculdade de Medicina de Ribeirão Preto da Universidade de São Paulo (USP), São Paulo , SP – Brasil; 18 Faculdade de Medicina Universidade de São Paulo São Paulo SP Brasil - Faculdade de Medicina da Universidade de São Paulo (USP), São Paulo , SP – Brasil; 19 Instituto do Coração Hospital das Clínicas Universidade de São Paulo São Paulo SP Brasil - Instituto do Coração (Incor) do Hospital das Clínicas da Faculdade de Medicina da Universidade de São Paulo (HCFMUSP), São Paulo , SP – Brasil; 20 Brigham and Women’s Hospital Boston EUA - Brigham and Women’s Hospital , Boston – EUA; 21 Department of Medicine, Cardiovascular Harvard Medical School Boston EUA - Department of Medicine, Cardiovascular , Harvard Medical School , Boston – EUA; 22 World Heart Federation Geneva Switzerland - World Heart Federation , Geneva – Switzerland; 23 Centre for Global Chronic Conditions, London School of Hygiene & Tropical Medicine Londres Inglaterra - Centre for Global Chronic Conditions, London School of Hygiene & Tropical Medicine , Londres – Inglaterra; 24 Division of Cardiology, Department of Medicine University of Washington Washington EUA - Division of Cardiology, Department of Medicine , University of Washington , Washington – EUA

**Keywords:** Doenças Cardiovasculares, Doença das Coronárias, Cardiomiopatias, Insuficiência Cardíaca, Doenças das Valvas Cardíacas, Fibrilação Atrial, Flutter Atrial, Estatística, Brasil

## Sobre estas estatísticas

**Table:** Abreviaturas usadas em “Sobre Estas Estatísticas” e *Highlights*

AVC	Acidente Vascular Cerebral
AVCH	Acidente Vascular Cerebral Hemorrágico
AVCI	Acidente Vascular Cerebral Isquêmico
CID	Classificação Estatística Internacional de Doenças e Problemas Relacionados à Saúde
CRVM	Cirurgia de Revascularização do Miocárdio
CV	Cardiovascular
DAC	Doença Arterial Coronariana
DALYs	Anos de vida perdidos ajustados por incapacidade (do inglês, *Disability-Adjusted Life-Year* )
DCR	Doença Cardíaca Reumática
DCV	Doença Cardiovascular
DVNR	Doença Valvar do Coração Não Reumática
FA	Fibrilação Atrial
GBD	Global Burden of Doença
HR	Hazard Ratio
HSA	Hemorragia Subaracnóidea
IAM	Infarto Agudo do Miocárdio
IBGE	Instituto Brasileiro de Geografia e Estatística
IC	Intervalo de Confiança
ICP	Intervenção Coronária Percutânea
IHME	Institute for Health Metrics and Evaluation
II	Intervalo de Incerteza
Int$	Dólares internacionais
IPCA	Índice de Preços ao Consumidor Amplo
IRR	Razão da taxa de incidência (do inglês, *Incidence Rate Ratio* )
OR	Odds Ratio
PIB	Produto Interno Bruto
PNS	Pesquisa Nacional de Saúde
PPC	Paridade do Poder de Compra
SDI	Índice Sociodemográfico (do inglês, *Sociodemographic Index* )
SIH	Sistema de Informações Hospitalares
SIM	Sistema de Informações sobre Mortalidade
SUS	Sistema Único de Saúde
UF	Unidade Federativa
US$	Dólares americanos
YLDs	Anos vividos com incapacidade (do inglês, *Years Lived with Disability* )
YLLs	Anos potenciais de vida perdidos (do inglês, *Years of Life Lost* )

Esta é a edição de 2021 da **Estatística Cardiovascular – Brasil** , um esforço multi-institucional para fornecer periodicamente informação atualizada sobre a epidemiologia das DCV e AVC no Brasil. Este relatório incorpora estatísticas oficiais fornecidas pelo Ministério da Saúde brasileiro e outros órgãos governamentais, pelo projeto GBD liderado pelo IHME da Universidade de Washington, além de dados gerados por outras fontes e estudos científicos, como coortes e registros, sobre as DCV e seus fatores de risco. Este documento é dirigido aos pesquisadores, médicos, pacientes, formuladores de políticas de saúde, profissionais da mídia, o público em geral e todos que buscam dados nacionais abrangentes sobre as DCV e o AVC.

A **Estatística Cardiovascular – Brasil 2021** é uma versão atualizada e expandida da **Estatística Cardiovascular – Brasil 2020** , ^1^ publicada no ano passado no *ABC Cardiol.* Inclui os dados mais recentes sobre DCV como um grupo de condições e as cinco DCV específicas abordadas no documento de 2020, além de novos capítulos sobre comportamentos e fatores de risco CV, em especial hipertensão, diabetes, dislipidemia, obesidade e tabagismo e uso de tabaco. O trabalho foi conduzido por pesquisadores voluntários de várias universidades brasileiras e instituições de pesquisa liderados por um *steering committee* de cinco membros (ALPR, CAP, DCM, GMMO e LCCB), com o suporte da Sociedade Brasileira de Cardiologia e a colaboração da Rede GBD Brasil ^2^ e de um comitê internacional (GAR, PP e TAG). Este relatório seguiu a metodologia usada pela *American Heart Association* para produzir anualmente o documento **Heart Disease & Stroke Statistics Update** , ^3^ que enfatiza dados epidemiológicos e de saúde pública. A **Estatística Cardiovascular – Brasil** não fornece informação sobre mecanismos fisiopatológicos nem recomenda tratamentos. Diferentemente de diretrizes e ‘ *position papers* ’, pretende apresentar as melhores e mais recentes métricas relacionadas às estatísticas sobre DCV na população brasileira.

Os dados usados no presente documento são provenientes de quatro diferentes fontes: (a) os sistemas de informações sobre mortalidade e de informações hospitalares no Brasil, disponibilizados pelo governo; (b) as estimativas do GBD 2019; ^4^ (c) a revisão sistemática da literatura com ênfase nas publicações dos últimos dez anos; (d) os custos de utilização da atenção à saúde, com base nas tabelas de reembolso do Sistema Público de Saúde, ajustados para inflação e reportados nas duas unidades monetárias originais (Reais ou dólares americanos em um ano específico) e em dólares internacionais. Os dólares internacionais foram convertidos em PPC ajustados para US$ 2019 (Int$ 2019), usando-se o conversor de custo do *Campbell and Cochrane Economics Methods Group* e do *Evidence for Policy and Practice Information and Coordinating Centre* . ^5^ Além disso, uma melhor explicação de como as estimativas da taxa de mortalidade podem variar dependendo da fonte usada (sistema de informação sobre mortalidade ou conjunto de dados do GBD) pode ser encontrada em outras publicações. ^6^

Como esperado, métricas diferentes ou discordantes são por vezes apresentadas para uma mesma condição, considerando-se que os estudos podem ter metodologias distintas ou ser conduzidos em diferentes períodos de tempo, locais e grupos etários. Tais diferenças são inevitáveis e suas possíveis razões foram sempre discutidas neste documento. Como muitos estudos cobrem um longo período de tempo e a expectativa de vida no Brasil aumentou nas últimas décadas, decidimos usar taxas padronizadas por idade, i.e., uma média ponderada de taxas específicas para idade por 100 mil pessoas, onde os pesos são as proporções de pessoas nos grupos etários correspondentes de uma população padrão. A padronização por idade do GBD utiliza um padrão global de idade, embora outras fontes possam ter usado diferentes populações de referência. Para a maioria dos estudos, raça/cor da pele foi usada de acordo com a definição do IBGE, i.e., branca, preta, parda, amarela (oriental) ou indígena (nativa).

A DCV ainda é responsável por quase um terço das mortes no Brasil e afeta desproporcionalmente o estrato mais vulnerável da população, que tem grande dificuldade no acesso a cuidados de saúde de alta qualidade. ^7^ ^,^ ^8^ Possuir dados nacionais representativos, confiáveis e abrangentes sobre DCV, comportamentos e fatores de risco é uma etapa obrigatória para a superação dessas desigualdades e para a oferta do melhor cuidado CV possível para todos os brasileiros. Este estudo reúne essa informação, essencial para o cuidado individual e para o planejamento dos próximos passos da política de saúde no Brasil, ^9^ além de indicar as lacunas de conhecimento a serem preenchidas em estudos futuros. Desejamos que as pessoas vivam mais e melhor e a compreensão das estatísticas CV para uma melhor abordagem das DCV pode contribuir para isso.

## Principais Fontes de Dados Brasileiros

Na presente versão da **Estatística Cardiovascular – Brasil** , as principais fontes de dados brasileiros são o sistema de informações sobre mortalidade, o sistema de informações hospitalares, as pesquisas de saúde periódicas, como a PNS, e as estimativas populacionais oficiais, especificados a seguir:

A. Sistema de Informações sobre Mortalidade: O SIM foi criado em 1975 pelo Ministério da Saúde brasileiro, sendo responsável por coletar, armazenar, gerenciar e divulgar dados nacionais de mortalidade. Esse sistema de informação em saúde representou um grande avanço na vigilância epidemiológica do país, pois sua principal atribuição é registrar todas as mortes ocorridas no território brasileiro. O Ministério da Saúde implementou um modelo de declaração de óbito padrão para coletar informação sobre morte, que utiliza a CID para codificar as causas de morte. Além disso, um fluxo para coletar, processar e distribuir a informação sobre morte foi implementado em todos os 5.570 municípios do país. ^10^ ^,^ ^11^ A qualidade da estatística sobre causas de morte no Brasil, baixa no início dos anos 2000, em especial em algumas partes do país, melhorou significativamente nas duas últimas décadas. ^12^ Por conhecer a heterogeneidade desses indicadores no Brasil, o relatório **Estatística Cardiovascular – Brasil** tratou os dados para ter uma estimativa mais próxima do real, realizando a correção para subnotificação e a redistribuição das causas de morte mal definidas. Mais detalhes podem ser obtidos no artigo de Malta *et al* . ^6^

B. Sistema de Informações Hospitalares: O objetivo da base de dados do SIH é registrar todas as hospitalizações financiadas pelo SUS. O SIH-SUS armazena dados sobre as hospitalizações em nível municipal através da Autorização de Internação Hospitalar, que contém informação sobre as doenças que levaram a hospitalização (usando a CID-10), o tempo de permanência, os procedimentos e os custos. ^13^ A informação do SIH-SUS permite o desenvolvimento de metodologias e a definição de indicadores para identificar disparidades geográficas relacionadas aos recursos hospitalares. ^14^

C. Pesquisa Nacional de Saúde: Quando da citação das estatísticas para fatores de risco CV, deu-se preferência à PNS, que é um inquérito epidemiológico de base domiciliar representativo do Brasil, de suas grandes regiões e UF, regiões metropolitanas, capitais e outros municípios em cada UF. A amostragem da PNS 2013 foi composta por 64.348 domicílios. A pesquisa foi conduzida pelo IBGE em parceria com o Ministério da Saúde, tendo incluído a maioria dos tópicos de saúde, como doenças não transmissíveis, fatores de risco, idosos, mulheres, crianças, utilização dos serviços de saúde, desigualdades em saúde, características antropométricas, exames laboratoriais, além da aferição de pressão arterial. ^15^ Os dados da PNS são usados pelo GBD em suas estimativas para o Brasil.

D. Para as estimativas populacionais, utilizaram-se no denominador as estimativas populacionais mais atualizadas geradas pelo IBGE ( www.ibge.gov.br ). Para as hospitalizações e análises de custo, utilizou-se a população residente estimada para o Tribunal de Contas da União anualmente, de 2008 a 2019.

## GBD 2019

O Estudo GBD ( http://www.healthdata.org/gbd ) é o mais abrangente estudo epidemiológico observacional de âmbito mundial até o momento. Descreve a mortalidade e a morbidade decorrentes das principais doenças, injúrias e fatores de risco em níveis global, nacional e regional. O exame das tendências a partir de 1990 até o presente, assim como as comparações entre populações, permite compreender os desafios em saúde enfrentados pelas pessoas em todo o mundo no século 21. O GBD 2019 é o último conjunto de dados disponibilizado publicamente. ^4,16–19^ A Rede GBD Brasil tem colaborado com o IHME, que lidera o projeto em âmbito mundial, para a identificação e a provisão de conjuntos de dados, a revisão de modelos e estimativas, bem como a validação e a publicação de resultados para o Brasil. ^20^ ^,^ ^21^ Detalhes de como as estimativas são calculadas podem ser obtidos nas publicações de base do Estudo GBD e no *website* do IHME ( http://www.healthdata.org/acting-data/what-we-measure-and-why ). As principais estimativas usadas neste documento estão resumidas abaixo:

A. Estimativas de mortes e de causas de morte. A principal fonte de informação é o SIM, uma base de dados do Ministério da Saúde, ajustada para outras fontes nacionais e internacionais. O IHME corrigiu a subnotificação de mortes e as mortes com “código *garbage* ” através da utilização de metodologia com algoritmos previamente publicada, ^22^ atualizada nas versões mais recentes do estudo ( http://www.healthdata.org/acting-data/determining-causes-death-how-we-reclassify-miscoded-deaths ).

B. Os YLLs são os anos perdidos em razão de mortalidade prematura, sendo calculados subtraindo-se a idade à época da morte da maior expectativa de vida possível para uma pessoa. Por exemplo, se a maior expectativa de vida para homens em certo país for de 75 anos e se um homem morre de câncer aos 65 nesse país, tem-se 10 anos potenciais de vida perdidos para o câncer.

C. Os YLDs também podem ser descritos como os anos vividos com saúde inferior à ideal. Estão aqui incluídas condições como influenza, que pode durar apenas uns poucos dias, ou epilepsia, que pode durar uma vida inteira. Os YLDs podem ser calculados ao se multiplicar a prevalência da condição pelo peso da incapacidade por ela gerada. Os pesos da incapacidade refletem a gravidade de diferentes condições e são desenvolvidos através de pesquisas com o público em geral.

D. Os DALYs são uma métrica universal que permite que pesquisadores e formuladores de políticas comparem populações e condições de saúde muito diferentes ao longo do tempo. Os DALYs correspondem à soma de YLLs e YLDs, sendo 1 DALY igual a 1 ano de vida saudável perdido. Esse índice permite que se estime o número total de anos perdidos devido a causas específicas e fatores de risco em níveis global, nacional e regional.

## Revisão Sistemática da Literatura

Os descritores para a elaboração das estratégias de busca foram selecionados no MeSH e no DeCS, os vocabulários controlados da MEDLINE e da LILACS, respectivamente. O plano da Embase foi desenhado com descritores Emtree em associação com MeSH. Além disso, termos livres foram usados, i.e., palavras-chave significativas e seus sinônimos, variações ortográficas e acrônimos essenciais para a busca no domínio pesquisado, mas que não são descritores controlados (ou não estão na lista de sinônimo desses descritores). É importante lembrar que, para manter a uniformidade, os mesmos descritores foram usados em todas as estratégias de busca. Entretanto, as estratégias foram customizadas conforme as especificidades de cada base de dados. Vale ainda lembrar que o grupo de termos relacionados a “Brasil” foi em geral utilizado em todos os campos de pesquisa (assunto, autor, título, afiliação institucional, nome do periódico, etc.).

As bases selecionadas para busca foram a MEDLINE através da PubMed, Embase, LILACS, CINAHL, *Cochrane Library Scopus* e *Web of Science* . Os seguintes filtros e limites da pesquisa bibliográfica foram utilizados: período de publicação (2004-2020); línguas (português, inglês e espanhol); tipo de estudo/publicação (Revisão, Meta-Análise, Ensaio Clínico, Ensaio Randomizado Controlado, Estudo Comparativo, Diretriz de Prática, Diretriz, Revisão Sistemática, Estudo de Avaliação, Publicação Governamental e Estudo Multicêntrico). Todas as referências foram organizadas usando-se *EndNote Web* . A partir da busca, os artigos foram incluídos se os estudos fossem de base populacional ou comunitária. Deu-se preferência aos estudos de âmbito nacional ou estadual. Os estudos conduzidos em serviços de saúde ou hospitais foram incluídos caso fossem multicêntricos e possuíssem tamanho amostral adequado (> 200 participantes foi o ponto de corte sugerido). Além dos artigos identificados na busca sistemática, os autores puderam incluir outros encontrados nas referências dos artigos buscados ou outros de que tivessem conhecimento em suas áreas de especialidade, caso os estudos atendessem aos critérios acima mencionados. Por fim, a decisão de quais estudos incluir em cada capítulo coube principalmente aos especialistas designados para o tema em questão.

## Utilização da Atenção à Saúde

Os estudos sobre o custo da atenção à saúde apresentam grande variabilidade metodológica e precisam ser interpretados com cautela. No presente documento, a maior parte dos dados sobre custo foi obtida das tabelas de reembolso do Sistema Público de Saúde de 2008 a 2019. Durante esse período, o reajuste pela inflação não foi realizado de maneira regular nem homogênea nos grupos e procedimentos de DCV, de modo que os valores brutos apresentados não foram ajustados para a verdadeira inflação.

Para minimizar o viés na notificação e na interpretação dos dados de custo, aplicou-se uma abordagem sistemática em todos os capítulos. Nas análises de custo geral, foram utilizadas as unidades monetárias originais [ *Reais* ou dólares americanos em um determinado ano] e dólares internacionais. Os dólares internacionais foram convertidos em PPC ajustados para US$ 2019 (Int$ 2019), usando-se o conversor de custo do *Campbell and Cochrane Economics Methods Group* e do *Evidence for Policy and Practice Information and Coordinating Centre* (https://eppi.ioe.ac.uk/costconversion/default.aspx). Nesse método, aplicou-se uma abordagem em duas etapas. Na primeira, ajustou-se a estimativa original de custo no preço-ano original para o preço-ano alvo, usando-se o índice de deflação do PIB (valores PIBD). Na segunda, houve conversão dessa estimativa ajustada da moeda original para a moeda alvo, usando-se as taxas de conversão baseadas em PPC para o PIB (valores PPC). ^5^ Para estudos econômicos originais, quando o ano-base da moeda não foi informado ou não pôde ser inferido a partir do manuscrito (p. e., coleta de dados do ano passado), recomendou-se adotar o ano anterior ao da publicação do manuscrito.

## Highlights

### Doença Cardiovascular Total

•De acordo com o Estudo GBD 2019 e a base de dados do SUS, as DCV são a causa número 1 de morte no Brasil. Entre as DCV, a DAC foi a principal causa de morte no país, seguida pelo AVC em 1990 e 2019.

•De acordo com o Estudo GBD 2019, a prevalência de DCV foi estimada em 6,1% da população e vem crescendo desde 1990 devido ao crescimento e envelhecimento populacional. No entanto, as taxas de prevalência e incidência de DCV padronizadas por idade no Brasil diminuíram no mesmo período.

•Observou-se redução na taxa de mortalidade ajustada por idade de 1990 a 2019 em todas as UF, embora menos significativa no Norte e Nordeste em comparação às outras regiões.

•As taxas de DALYs padronizadas por idade no Brasil caíram de 1990 a 2019, tendo havido correlação entre a redução percentual nas taxas de DALYs e o aumento no SDI: quanto maior o SDI, maior a redução nos DALYs por DCV.

•Dados do SUS mostraram um número significativo de procedimentos CV clínicos e cirúrgicos pagos, em especial para insuficiência cardíaca, doenças cerebrovasculares e síndrome coronariana aguda. Hospitalizações para ICP aumentaram significativamente nas últimas décadas, enquanto os procedimentos cirúrgicos permaneceram estáveis.

### AVC

•De acordo com um estudo de base comunitária realizado na cidade de Matão em 2003-2004 e 2015-2016, a incidência de AVC ajustada por idade diminuiu em 39% (IRR 0,61; IC 95%, 0,46–0,79) e a mortalidade, em 50% (IRR 0,50; IC 95%, 0,31– 0,94). A idade média de pacientes com AVC aumentou em 9%, passando de 65,2 (IC 95%, 62,6–67,8) para 71,0 (IC 95%, 68,1–73,8) anos. A taxa de letalidade de 1 ano foi 26%. Aproximadamente 56% dos pacientes eram funcionalmente independentes, enquanto 7% tiveram um AVC recorrente.

•Com relação à distribuição dos subtipos de AVC, de acordo com o Registro Joinvasc realizado na cidade de Joinville, de 1995 a 2013, a proporção de AVCI aumentou 12%, enquanto a de AVCH diminuiu 16%. Entretanto, a proporção de HSA permaneceu relativamente estável, variando de 7,5% em 1995 a 6% em 2012-2013. Nos últimos 8 anos, as incidências de AVCI e de AVCH apresentaram reduções significativas de 15% (IC 95%, 1-28) e de 60% (IC 95%, 13-86), respectivamente.

•Segundo dados do Estudo GBD 2019, as taxas de mortalidade por AVC padronizadas por idade por 100 mil foram 137,8 (127,8 a 144) em 1990 e 58,1 (52,6 a 61,8) em 2019, representando variação percentual de -57,8 (-60,4 a -0,6). A maior variação percentual ocorreu em Goiás, -65,9 (-71,8 a -0,6), e a menor, no Maranhão, -22,7 (-37,2 a 0). Para os adultos, a maior variação percentual foi observada no grupo etário de 50-69 anos, -61 (-63,6 a -0,6).

•Considerando a carga de AVC no Brasil, o Estudo GBD 2019 observou redução marcada nos YLL: as taxas de YLL por AVC padronizadas por idade por 100 mil foram 2.778,6 (2.659,5 a 2.879,2) em 1990 e 1.098,7 (1.025,8 a 1.153,7) em 2019, representando uma variação percentual de -60,5 (-62,7 a -0,6). Para adultos, a maior variação percentual foi observada no grupo etário de 50-69 anos, -61,7 (-64,3 a -0,6).

### Doença Arterial Coronariana Aguda e Crônica

•De acordo com dados do estudo GBD 2019, no Brasil, o número de portadores de DAC (IAM, angina estável e insuficiência cardíaca isquêmica) aumentou de 1,48 milhão em 1990 para mais de 4 milhões em 2019, e a prevalência bruta de DAC passou de 0,99% para 1,85% no período, embora a taxa de prevalência padronizada por idade tenha permanecido estável.

•Em 2019, houve 171.246 mortes atribuídas a DAC no Brasil, correspondendo a 12% do total de mortes no país e a 43% de todas as mortes por DCV. A DAC foi a primeira causa de morte no Brasil e em quase todas as suas UF, exceto duas. De 1990 a 2019, observou-se redução na taxa de mortalidade ajustada por idade em todas as UF, embora menos significativa no Nordeste em comparação às demais regiões.

•De acordo com dados do SUS, o número de hospitalizações por IAM no sistema público aumentou 54% de 2008 a 2019, ajustado para a população. Os procedimentos de ICP não primária por habitante dobraram, enquanto os de ICP primária aumentaram 31%. Enquanto isso, o número total de CRVM permaneceu estável no período. A taxa de mortalidade hospitalar por IAM diminuiu de 15,9% em 2008 para 12,9% em 2019. Para a síndrome coronariana aguda, as taxas permaneceram estáveis no período, assim como as de ICP e CRVM.

### Cardiomiopatia e Insuficiência Cardíaca

•De acordo com as estimativas do Estudo GBD 2019, a prevalência padronizada por idade de cardiomiopatia e miocardite diminuiu no Brasil, passando de 76,6 (II 95%, 53,4-107,2) em 1990 para 73,0 (II 95%, 51,1-100,1) em 2019, uma redução de 4,7% (II 95%, - 9,5 a 0,8) no período. Em números absolutos, as estimativas de prevalência de cardiomiopatia e miocardite no Brasil passaram de menos de 60 mil em 1990 para mais de 160 mil em 2019, principalmente devido ao crescimento e envelhecimento da população. A prevalência de cardiomiopatia e miocardite foi maior nos homens (98,9; II 95%, 69,5-137,2) do que nas mulheres (54,1; II 95%, 38,4-73,8) em 2019.

•No estudo de coorte retrospectivo sobre Chagas dos *National Institutes of Health* , REDS-II, doadores de sangue inicialmente saudáveis com uma doação-índice soropositiva para *T. cruzi* pareados por idade, sexo e período com doadores soronegativos foram acompanhados por 20 anos.

A incidência diferencial de cardiomiopatia atribuível à infecção por *T. cruzi* foi 1,85 por 100 pessoas-ano nos 10 primeiros anos de seguimento e 0,9 por 100 pessoas-ano nos 10 anos seguintes. O nível de anticorpo anti- *T. cruzi* na segunda visita foi associado com o desenvolvimento de cardiomiopatia (OR ajustado, 1,4; IC 95%, 1,1-1,8) na última visita.

•De acordo com o Estudo GBD 2019, o número de mortes por doença de Chagas no Brasil diminuiu de 7.903 (II 95%, 2.438-10.073) em 1990 para 6.523 (II 95%, 3.350-11.226) em 2019. A taxa de mortalidade padronizada por idade apresentou redução mais marcante (variação de -67,5%), passando de 8,6 (II 95%, 2,8-10,9) mortes por 100 mil habitantes em 1990 para 2,8 (II 95%, 1,8-4,8) por 100 mil habitantes em 2019, correspondendo a 1,6% de todas as mortes CV no país.

•De acordo com dados do SUS, houve 3.085.359 hospitalizações por insuficiência cardíaca de 2008 a 2019. Esse número representa um terço do total de hospitalizações clínicas relacionadas às condições CV no período estudado. Nesse período, houve redução no número de hospitalizações clínicas por insuficiência cardíaca, que passaram de 298.474 (157 por 100 mil) em 2008 para 222.620 (105 por 100 mil) em 2019, sendo a redução uniforme ao longo dos anos. A despeito dessa redução no número de admissões, os gastos em saúde não ajustados estimados a partir do pagamento direto por assistência a pacientes com insuficiência cardíaca aumentaram de 2008 para 2019 em quase 32%, passando de R$ 272.280.662 (2019 Int$ 267.102.469) em 2008 para R$ 359.301.691 (2019 Int$ 173.659.589) em 2019. Insuficiência cardíaca foi responsável pela maioria dos custos relacionados às hospitalizações clínicas por DCV.

### Doença Valvar do Coração

•De acordo com o Estudo GBD 2019, o padrão de doença valvar cardíaca tem mudado no Brasil: a prevalência de DCR padronizada por idade ficou estável de 1990 a 2019, mas houve um aumento significativo de mais de 50% na DVNR, especialmente para homens e grupos etários mais avançados. Entre as doenças valvares específicas, houve marcado aumento de 201,8% de doença valvar aórtica calcífica, reforçando o impacto do envelhecimento populacional.

•As taxas de mortalidade padronizadas por idade atribuíveis à DCR diminuíram significativamente em 59,4% de 1990 a 2019, enquanto as atribuíveis a DVNR apresentaram leve redução, 16,2% (II 95%, 10,3-22,5). Entretanto, a taxa bruta de mortalidade aumentou significativamente para as idades mais avançadas (>70 anos), em associação com doença valvar aórtica degenerativa, sugerindo uma carga crescente para os sistemas de saúde, requerendo ações específicas para minimizar os impactos.

•A carga atribuível à doença valvar cardíaca no Brasil permanece ligada a fatores socioeconômicos, com significativas correlações negativas entre o SDI e as variações nas taxas de mortalidade associada a DCR padronizadas por idade em 1990 e 2019, além de correlações positivas entre o SDI e a taxa de mortalidade por DVNR nos dois anos.

•Despesas com doença valvar cardíaca no sistema público de saúde brasileiro diminuíram proporcionalmente de 2008 a 2019 (-6,3% e -28% para admissões clínicas e intervencionais, respectivamente), devido a inflação e correção monetária. Essa redução demanda discussões de revisão orçamentária, evitando o adiamento ou restrição de procedimentos intervencionais e cirúrgicos e permitindo a incorporação de novas tecnologias e dispositivos.

•A despeito da melhoria nas últimas décadas, a DCR permanece importante causa de morbimortalidade no Brasil: dados do SUS mostram que, além de ser a etiologia de quase metade das cirurgias valvares no sistema público de saúde, associada com maior mortalidade hospitalar, a prevalência de doença subclínica entre escolares mostrou-se alta (4,5%), com a implementação de programas de rastreio em larga escala em 2014. Portanto, ações coordenadas para diagnóstico precoce e profilaxia são necessárias para evitar progressão da doença e sequelas tardias.

### Fibrilação Atrial

•De acordo com as estimativas do Estudo GBD 2019, as taxas de prevalência de FA e *flutter* atrial padronizadas por idade apresentaram um pequeno aumento no Brasil: de 519 (II 95%, 393-669) em 1990 para 537 (II 95%, 409-692) em 2019, por 100 mil habitantes, para ambos os sexos, com variação de 3,5% (II 95%, 1,8-5,1) no período.

•Em seguimento de 10 anos de 1.462 indivíduos com idade ≥ 60 anos (idade média, 69 anos; 61% mulheres) incluídos no estudo de coorte de Bambuí em 1997, FA ou *flutter* mostrou associação independente com um aumento na mortalidade por todas as causas (HR, 2,35; IC 95%, 1,53-3,62) entre pacientes com e sem doença de Chagas.

•Dados do sistema de telessaúde de Minas Gerais, que incluiu 1.558.421 indivíduos (idade média, 51±18 anos; 40,2% homens) com ECG realizado entre 2010 e 2017 revelaram, em modelos multivariados ajustados para idade e sexo, os seguintes fatores de risco e comorbidades autorrelatados relacionados à presença de FA: doença de Chagas (OR 3,08; IC 95%, 2,91-3,25), infarto do miocárdio prévio (OR 1,74; IC 95%, 1,56-1,93), doença pulmonar obstrutiva crônica (OR 1,48; IC 95%, 1,33-1,66), hipertensão (OR 1,31; IC 95%, 1,27-1,34) e dislipidemia (OR 1,09; IC 95%, 1,03-1,16). Tabagismo atual e diabetes não se associaram a FA prevalente.

•De todos os 429 casos de AVC (87,2% dos quais isquêmicos) que ocorreram na cidade de Joinville em 2015 e foram incluídos em um registro, FA foi detectada em 11,4% deles e em 58% daqueles com AVC cardioembólico. Da mesma forma, detectou-se FA em 58% dos 359 pacientes com AVC cardioembólico de uma amostra consecutiva proveniente de um único centro na cidade de Curitiba.

### Hipertensão

•Análise dos dados da PNS 2013 mostraram que a prevalência de hipertensão aferida a partir dos 18 anos de idade foi 22,8% (IC 95%, 22,1 - 23,4%) em uma amostra de 59.402 indivíduos. Naqueles com mais de 75 anos, a prevalência estimada foi de 47,1%. No grupo etário de 18 a 74 anos, a prevalência foi maior entre os homens, tendo as mulheres apresentado leve predominância apenas a partir dos 75 anos de idade. A análise por região mostrou que o Sudeste (25%) e o Sul (25%) apresentaram as maiores prevalências em ambos os sexos.

•Dados da PNS 2013 mostraram que 36% da população brasileira incluída tinha diagnóstico prévio e/ou medida da pressão arterial igual ou superior a 140/90 mmHg. Desses, 89% haviam contatado o sistema de saúde nos 2 anos anteriores, mas apenas 65% estavam cientes de sua condição. Entre os conhecedores de sua condição de hipertensos, 62% procuravam assistência com regularidade, 92% dos quais tendo recebido prescrição de medicamentos. Daqueles que informaram receber medicamentos, apenas 56% relataram que a assistência à sua condição não apresentava problemas e incluía orientação sobre importantes fatores de risco e comportamento. De toda a população hipertensa, cerca de 33% apresentavam controle de sua pressão arterial.

•O Estudo de Riscos Cardiovasculares em Adolescentes (ERICA) avaliou 73.399 estudantes com idade média de 14,7 ± 1,6 anos, sendo 55,4% do sexo feminino. A prevalência de hipertensão foi 9,6%, sendo mais baixa nas regiões Norte (8,4%) e Nordeste (8,4%) e mais alta na região Sul (12,5%). Adolescentes obesos apresentaram maior prevalência de hipertensão (28,4%) em comparação àqueles com sobrepeso (15,4%) ou eutróficos (6,3%). A proporção de hipertensão atribuída a obesidade foi estimada em 17,8%.

•De acordo com dados do estudo de coorte ELSA-Brasil, que incluiu 7.063 pacientes, com idade média de 58,9 anos na linha de base (2008-2010), hipertensão foi associada com maiores declínios de memória, fluência e escore cognitivo global. Além disso, pré-hipertensão foi preditor independente de maior declínio no teste de fluência verbal e no escore cognitivo global. Entre indivíduos tratados, o controle da pressão arterial na linha de base associou-se inversamente com o declínio no escore cognitivo global e no teste de memória.

### Diabetes mellitus

•Segundo dados da Federação Internacional de Diabetes publicados em 2019, o Brasil ocupava o quinto lugar no mundo em quantidade de adultos com diabetes, totalizando 16,8 milhões (IC 95% 15,0 - 18,7) de indivíduos, 46% dos quais desconhecia ser portador da condição. A prevalência de pré-diabetes foi de 9,5% (15,1 milhões de pessoas).

•Segundo dados do Estudo GBD 2019, mortalidade por DCV atribuível ao diabetes para todas as idades no Brasil aumentou em termos absolutos, passando de 50.812 mortes (II 95%, 35.649 - 73.137) em 1990 para 80.754 (II 95%, 55.922 - 118.175) em 2019. No entanto, as taxas de mortalidade padronizadas por idade por 100 mil habitantes diminuíram de 70,4 (II 95%, 47,4 - 106,1) em 1990 para 35,9 (II 95% 24,5 - 53,0) em 2019, uma redução de -49,0% (II 95%, -53,4 a -43,9).

•Quanto à carga de DCV atribuível ao diabetes, as taxas de DALYs padronizadas por idade por 100 mil habitantes diminuíram 47,4% (II 95%, -52,2 a -41,9) entre 1990 e 2019, apesar de ter ocorrido um aumento no número total de DALYs de 1.072.309 (II 95%, 784.276 -1.484.959) para 1.571.116 (II 95%, 1.140.912 - 2.203.188) no período. Houve redução heterogênea das taxas de DALYs padronizadas por idade por diabetes entre as diversas UF e regiões do Brasil.

### Dislipidemia

•De acordo com a PNS 2014-2015, a prevalência de dislipidemia no Brasil ainda é alta: colesterol total ≥ 200 mg/dl em 32,7% (IC 95%, 31,5 – 34,1) da população geral; HDLc baixo em 31,8% (IC 95%, 30,5 – 33,1); e LDLc alto em 18,6% (IC 95%, 17,5 – 19,7). Nível educacional mais alto foi relacionado a menor prevalência de colesterol total elevado, de LDLc alto e de HDLc baixo.

•De acordo com o Estudo GBD 2019, ao analisar as tendências de 1990 a 2019, observa-se um aumento no número absoluto de mortes, YLLs e DALYs, com redução nas taxas padronizadas por idade dessas métricas em todos os estados e em âmbito nacional.

•O estudo ELSA-Brasil, uma coorte brasileira, avaliou hipercolesterolemia familiar e mostrou prevalência de 1 em 263 indivíduos, mas ainda faltam dados de carga de doença e impacto nos custos.

•Segundo o estudo ELSA-Brasil, a conscientização a respeito de dislipidemia é baixa (58,1% dos indivíduos com LDLc elevado), sendo que apenas 42,3% deles recebem tratamento medicamentoso. Além disso, só 58,3% dos indivíduos em uso de algum hipolipemiante atinge os níveis-alvo de lipídios séricos.

### Obesidade e sobrepeso

•De acordo com dados do IBGE, no Brasil, o percentual de adultos (≥18 anos de idade) com excesso de peso e obesidade em 2019 foi de 57,5% (IC 95%, 54,8 - 60,2) e 21,8 % (IC 95%, 19,2 - 24,7) para os homens, e de 62,6% (IC 95%, 59,1 - 66,0) e 29,5% (IC 95%, 25,4 - 34,0) para as mulheres, respectivamente. Houve aumento progressivo de obesidade com a idade, variando de 10,7% (IC 95%, 7,7 - 14,7) [masculino: 7,9% (IC 95%, 4,8 - 12,8); feminino: 13,5% (IC 95%, 8,8 - 20,4)] no grupo etário de 18-24 anos a 34,4% (IC 95%, 29,7 - 39,4) [masculino: 30,2% (IC 95%, 24,8 - 36,3); feminino: 38,0% (IC 95%, 32,3 - 44,0)] no grupo etário de 40-59 anos. Nota-se maior prevalência de excesso de peso e obesidade no sexo feminino em todos os grupos etários.

•De 1990 a 2019, houve uma variação negativa nas taxas de mortalidade por DCV atribuível a índice de massa corporal elevado para as mulheres [-33,9 (-43,7;-16,7)] maior do que para os homens [-22,8 (-35,9;6,2)]. As maiores reduções no percentual de mortalidade ocorreram nas UF de maior renda no Brasil.

•A maioria das UF apresentou redução nas taxas padronizadas por idade de DALYs por DCV atribuível a índice de massa corporal elevado para mulheres no período. Comportamento similar foi observado para os homens, com redução percentual de obesidade de 1990 a 2019.

•A maioria das políticas públicas falhou em reduzir a obesidade em adultos e crianças, provavelmente por se tratar de condição multifatorial e envolver muitos aspectos socioeconômicos. A obesidade é pandêmica e tem impacto nos países tanto desenvolvidos quanto em desenvolvimento, com consequências nos níveis individual, social, familiar e financeiro. Registros nacionais de obesidade devem ser elaborados para permitir o desenvolvimento de políticas públicas mais efetivas para controle da obesidade, que vem aumentando no Brasil nos dois sexos e nos diferentes grupos etários.

### Tabagismo e Uso de Tabaco

•Dados da PNS 2019 indicam que 12,8% (IC 95%, 12,4 – 13,2%) dos adultos usam algum derivado do tabaco, sendo o uso maior entre homens (16,2%; IC 95%, 15,6 – 16,9%) do que entre mulheres (9,9%; IC 95%, 9,3 – 10,3%). Segundo a pesquisa domiciliar Vigitel, houve significativa redução na prevalência de tabagismo na população adulta, de 37,6% de 2006 a 2019. Entretanto, houve aumento de 0,5% na prevalência de 2019 em comparação à de 2018, sugerindo uma mudança na tendência.

•De acordo com o Estudo GBD 2019, houve uma redução de 58,8% (II 95%, 56,2 - 61,1) na taxa de mortalidade total atribuída ao tabagismo no Brasil de 1990 a 2019.

A tendência foi a mesma para homens e mulheres e em todas as UF brasileiras. Da mesma forma, a mortalidade CV atribuível ao tabagismo diminuiu em quase 70% no mesmo período.

•A carga de doença CV atribuída ao tabaco diminuiu de 1990 a 2019, com redução de 69% (95% UI, 56 - 61) nas taxas de DALYs padronizadas por idade. Houve redução heterogênea na taxa de DALYs padronizada por idade atribuída ao tabaco nas diferentes UF e regiões do Brasil, mais pronunciada no Sudeste, Sul e Centro-Oeste, com modesta redução nas UF do Norte e ainda mais discreta na maioria das UF do Nordeste.

•Em estudo utilizando modelo probabilístico de microssimulação econômica de Markov, o custo direto total do tabaco foi estimado em US$ 11,8 bilhões ao ano, 70% correspondendo ao custo direto associado com a assistência à saúde e o restante associado com o custo indireto por perda de produtividade por morte prematura e incapacidade.

O tabaco representou 22% dos custos diretos de DCV no Brasil e 17% dos custos diretos de AVC.

## 1. DOENÇA CARDIOVASCULAR TOTAL

### CID-9 390 a 459; CID-10 I00 a I99

**Ver Tabelas 1-1 até 1-13 e Figuras 1-1 até 1-16**

**Table:** Abreviaturas usadas no Capítulo 1

AHA	American Heart Association
AVC	Acidente Vascular Cerebral
CID	Classificação Estatística Internacional de Doenças e Problemas Relacionados à Saúde
CRVM	Cirurgia de Revascularização do Miocárdio
DALYs	Anos de vida perdidos ajustados por incapacidade (do inglês, *Disability-Adjusted Life-Year* )
DATASUS	Departamento de Informática do Sistema Único de Saúde
DCNT	Doenças Crônicas Não Transmissíveis
DCV	Doença Cardiovascular
DIC	Doença Isquêmica do Coração
ELSA-Brasil	Estudo Longitudinal de Saúde do Adulto
GBD	Global Burden of Disease
IAM	Infarto Agudo do Miocárdio
IBGE	Instituto Brasileiro de Geografia e Estatística
IC	Intervalo de Confiança
IDH	Índice de Desenvolvimento Humano
IDHm	Índice de Desenvolvimento Humano Municipal
II	Intervalo de Incerteza
OR	Odds Ratio
PIB	Produto Interno Bruto
PSF	Programa Saúde da Família
RAP	Risco Atribuível na População
RR	Risco Relativo
SDI	Índice Sociodemográfico (do inglês, *Sociodemographic Index* )
SIDRA	Sistema IBGE de Recuperação Automática
SIM	Sistema de Informações sobre Mortalidade
SNS	Sistema Nacional de Saúde
SUS	Sistema Único de Saúde
UF	Unidade Federativa

**Tabela 1-1:** – Número de casos prevalentes e taxas de prevalência de doença cardiovascular padronizadas por idade, por 100 mil habitantes, e variação percentual das taxas, por grupo etário e sexo, no Brasil, em 1990 e 2019.

Causa de morte e grupo etário	1990		2019		Variação percentual (II 95%)
	Número (II 95%)	Taxa (II 95%)	Número (II 95%)	Taxa (II 95%)	
**Ambos**					
15-49 anos	164612,9 (144806,1;186400)	214,8 (188,9;243,2)	215729,8 (190813,1;243619,6)	186,8 (165,2;210,9)	-13 (-15,8;-10,1)
50-69 anos	218837,1 (196759,4;243342,6)	1395 (1254,2;1551,2)	450185,8 (406572,8;498758)	1115,9 (1007,8;1236,3)	-20 (-22,6;-17,2)
5-14 anos	50036,9 (33169,3;71176,5)	141,6 (93,9;201,4)	45954,6 (30182,4;65586,3)	142,5 (93,6;203,4)	0,6 (-1,7;3,1)
70+ anos	147631,3 (132225,3;164119,3)	3490 (3125,8;3879,8)	372585,5 (336268;412493)	2846,7 (2569,2;3151,6)	-18,4 (-21;-15,7)
Padronizada por idade	593711,1 (555922,5;636828,7)	593,7 (558,1;636,4)	1095891 (1030085,3;1166807,2)	474,9 (447,1;506,5)	-20 (-21,7;-18,4)
Todas as idades	593711,1 (555922,5;636828,7)	398,9 (373,5;427,9)	1095891 (1030085,3;1166807,2)	505,8 (475,4;538,5)	26,8 (23,3;30,4)
Abaixo de 5	12592,9 (9098,3;17056,8)	74,3 (53,7;100,7)	11435,3 (8280,9;15433,2)	73,8 (53,4;99,6)	-0,7 (-3,6;2,2)
**Mulheres**					
15-49 anos	81840,1 (71524,9;92783,6)	210,2 (183,7;238,3)	105700 (92430,3;120336,2)	180,7 (158,1;205,8)	-14 (-16,8;-10,9)
50-69 anos	102496,1 (91526,7;114514,7)	1255,7 (1121,3;1402,9)	208399,3 (186607,1;232143,7)	973,2 (871,4;1084)	-22,5 (-25;-19,8)
5-14 anos	26514,1 (17557,2;37666,8)	151,7 (100,4;215,5)	24476,1 (16014,6;34617,6)	154,6 (101,1;218,6)	1,9 (-1,4;5,7)
70+ anos	77895,9 (69702,8;86822,5)	3321,7 (2972,3;3702,4)	200343,1 (180509,1;222408)	2653,3 (2390,6;2945,5)	-20,1 (-22,9;-17,2)
Padronizada por idade	294962,9 (275518,3;317426,8)	557,5 (523,8;597,3)	544515,2 (512491,4;581529,1)	437,4 (411;468,6)	-21,5 (-23,3;-20)
Todas as idades	294962,9 (275518,3;317426,8)	391,9 (366;421,7)	544515,2 (512491,4;581529,1)	491,1 (462,3;524,5)	25,3 (21,7;29,1)
Abaixo de 5	6216,7 (4434;8521,4)	74,5 (53,1;102,1)	5596,8 (3974;7644)	73,8 (52,4;100,8)	-1 (-4;2,2)
**Homens**					
15-49 anos	82772,8 (72882;93235)	219,5 (193,3;247,2)	110029,8 (97839,1;123670,6)	193 (171,6;216,9)	-12,1 (-15,2;-8,8)
50-69 anos	116341 (104908;129351,1)	1546 (1394,1;1718,9)	241786,5 (218258,2;268733,5)	1277,4 (1153,1;1419,7)	-17,4 (-20,2;-14,2)
5-14 anos	23522,8 (15573,9;33681,3)	131,8 (87,2;188,7)	21478,5 (14174,7;31018,6)	130,9 (86,4;189)	-0,7 (-3,4;2)
70+ anos	69735,4 (62169,2;77641,2)	3699,5 (3298,1;4118,9)	172242,4 (155338,1;190212,2)	3110,3 (2805,1;3434,8)	-15,9 (-18,7;-12,9)
Padronizada por idade	298748,2 (279373,3;320526,9)	635,3 (595,3;681,3)	551375,8 (517313,3;587391,2)	520,8 (489,5;554,7)	-18 (-19,8;-16,3)
Todas as idades	298748,2 (279373,3;320526,9)	406,1 (379,8;435,7)	551375,8 (517313,3;587391,2)	521,2 (489;555,2)	28,3 (24,6;32,2)
Abaixo de 5	6376,2 (4668,2;8561,1)	74,2 (54,3;99,6)	5838,5 (4282,6;7786)	73,8 (54,1;98,4)	-0,5 (-4,1;3,5)
Total	8978586,3 (7687971,2;10466915,3)	46326,6 (38506,4;55504,7)	15575150,5 (13460371,5;18007447,8)	37817,9 (31606;45148,7)	3435,8 (1527,7;5473,2)

*Fonte: Dados derivados do estudo Global Burden of Disease 2019, Institute for Health Metrics and Evaluation, University of Washington. ^46^*

**Tabela 1-2:** – Número de casos incidentes, taxas de incidência de doença cardiovascular padronizadas por idade (por 100 mil) e variação percentual das taxas no Brasil e unidades federativas, em 1990 e 2019.

Causa de morte e local	1990		2019		Variação percentual (II 95%)
	Número (II 95%)	Taxa (II 95%)	Número (II 95%)	Taxa (II 95%)	
**B.2-Doenças Cardiovasculares**					
Brasil	593711.1 (555922.5;636828.7)	593.7 (558.1;636.4)	1095891 (1030085.3;1166807.2)	474.9 (447.1;506.5)	-20(-21.7;-18.4)
Acre	1206.9 (1109.3;1324)	549.1 (513.6;588)	3266 (3049.1;3501.1)	471.7 (441.9;504.1)	-14.1(-16.4;-11.8)
Alagoas	9183 (8506.9;9969.2)	581.1 (541.6;625.5)	15952 (14961.2;17073.7)	484.3 (454.2;517.6)	-16.7(-19;-14.3)
Amapá	716.8 (656.5;788.6)	520.5 (486.1;559)	2653.7 (2476.6;2862.4)	440.7 (411.6;473.2)	-15.3(-17.5;-13.2)
Amazonas	5629.9 (5174.7;6164.5)	531.1 (496.3;569.2)	14473.2 (13534.3;15528.2)	451.3 (422.1;482.1)	-15(-17.2;-12.7)
Bahia	44112.1 (41105.1;47717.9)	562.8 (525.9;603.5)	75928.2 (71378.4;80995.6)	473.7 (444.5;506.6)	-15.8(-18.2;-13.8)
Ceará	24076.6 (22398.9;25978.9)	524.8 (491.1;560.8)	45521.1 (42602.7;48610)	458.7 (429.6;490.7)	-12.6(-14.8;-10.4)
Distrito Federal	4505.6 (4168.8;4888.6)	550.4 (515.5;590.1)	11843.1 (11093.6;12666.6)	433.2 (406.9;463.9)	-21.3(-23.6;-19.1)
Espírito Santo	10124.4 (9476.6;10907.6)	597 (558.7;639.6)	20329.8 (19077.6;21677.9)	475.9 (446.4;508.7)	-20.3(-22.7;-17.8)
Goiás	14164.8 (13166.4;15345)	559.3 (522.7;600.2)	30538.9 (28602.7;32638.9)	440.8 (412.6;470.9)	-21.2(-23.5;-19)
Maranhão	16322.6 (15071;17739.8)	528.8 (493.8;568.2)	31679.3 (29575.4;34020.2)	453.8 (424.3;486.7)	-14.2(-16.3;-11.9)
Mato Grosso	5861.4 (5416;6376.9)	560.1 (523.8;603.2)	15572.9 (14640.8;16594.5)	462.1 (433.4;493.7)	-17.5(-19.8;-15.3)
Mato Grosso do Sul	6310.2 (5890.9;6820.4)	582.4 (545;624.2)	14125.2 (13252.9;15040.8)	483.8 (453;515.7)	-16.9(-19.2;-14.7)
Minas Gerais	71837.8 (67116.6;77197)	662.2 (620.6;708.3)	134221.7 (125539.7;143761.7)	526.5 (492.9;563)	-20.5(-22.8;-18.4)
Pará	14537.4 (13368.9;15902.3)	540 (505.1;579.9)	33318.7 (31088.5;35782.7)	443.4 (414;475.7)	-17.9(-20.2;-15.7)
Paraíba	13732.3 (12775.8;14788.3)	549.2 (513.2;590)	20836.7 (19578.6;22302.1)	449.3 (420.7;482.1)	-18.2(-20.8;-16)
Paraná	33688.5 (31469.4;36286)	607.4 (567.4;652)	61307.5 (57604;65450.1)	476.9 (448.7;509.1)	-21.5(-23.7;-19.2)
Pernambuco	29154.2 (27148.1;31474)	575.5 (540.2;616.7)	48609.7 (45664.2;51831.3)	486.1 (455.6;518.8)	-15.5(-17.8;-13.5)
Piaui	9035.6 (8358.6;9805.9)	543.1 (506.4;584.1)	17353.8 (16310.2;18496.1)	464.3 (436.1;495.3)	-14.5(-16.9;-12)
Rio de Janeiro	64591.8 (60558.2;69272.2)	635.4 (597.7;678.7)	100862 (94708.7;107521.3)	473.2 (445;504.7)	-25.5(-27.7;-23.5)
Rio Grande do Norte	9424.3 (8784.4;10171.1)	530.4 (495.3;570.5)	17234 (16146;18374.5)	450.5 (422;481.3)	-15.1(-17.3;-12.6)
Rio Grande do Sul	43143.4 (40538.5;46255.1)	615.4 (577.1;659.5)	70448.5 (66035.9;75279.1)	482.5 (454.4;515.7)	-21.6(-23.8;-19.4)
Rondônia	2951.5 (2701.4;3248.2)	565.1 (526.5;608.3)	7150.6 (6699;7664.9)	449.4 (419.3;481)	-20.5(-23;-17.9)
Roraima	502 (459.5;551.6)	544 (508.2;584.8)	1917.1 (1781.8;2065.3)	445.2 (414.8;476.5)	-18.2(-20.4;-15.9)
Santa Catarina	16812.6 (15702.4;18087.7)	573.7 (537.3;613.9)	37225 (35046.3;39624.9)	470.9 (442.5;502.4)	-17.9(-20.4;-15.2)
São Paulo	133961.2 (125479.2;143345)	604.8 (567.2;644.6)	245976.9 (231441.1;261645.1)	470.8 (442.9;502.5)	-22.2(-24.3;-20.1)
Sergipe	5348.7 (4961.9;5795.4)	572 (534.5;614.3)	11043.8 (10386.9;11787.7)	485.8 (456;518.7)	-15.1(-17.4;-12.5)
Tocantins	2775.6 (2556.8;3025.5)	533.9 (499.9;572.8)	6501.8 (6086.7;6952.9)	440.7 (412.2;470.6)	-17.5(-19.7;-15.2)

*Fonte: Dados derivados do estudo Global Burden of Disease 2019, Institute for Health Metrics and Evaluation, University of Washington. ^46^*

**Tabela 1-3:** – Número de mortes e taxas de mortalidade padronizadas por idade por doenças cardiovasculares (por 100 mil habitantes) e variação percentual das taxas no Brasil e suas unidades federativas, em 1990 e 2019.

Causa de morte e local	1990		2019		Variação percentual (II 95%)
	Número (II 95%)	Taxa (II 95%)	Número (II 95%)	Taxa (II 95%)	
**B.2-Doenças Cardiovasculares**					
Brasil	269722,7 (257743,7;277272,1)	355,4 (332,5;367,6)	397993 (361776,4;417773,2)	175,7 (159;184,8)	-50,6(-52,7;-48,8)
Acre	349,6 (326,1;369)	279,6 (255,4;295,7)	912,7 (824,2;991,4)	171,2 (151,8;187)	-38,8(-44,1;-33)
Alagoas	4147,2 (3861,4;4448,1)	345,1 (316,4;370,7)	6951,8 (6104,9;7772,3)	224,9 (196,8;250,8)	-34,8(-42,4;-26,7)
Amapá	188,5 (174,2;200,2)	238,8 (214,9;254,2)	704 (637,8;762,4)	154 (137,2;167,9)	-35,5(-40,4;-30,7)
Amazonas	1720,8 (1593,1;1851,7)	289,9 (266,6;311,4)	3750,6 (3301,4;4208,2)	143 (124,9;160,1)	-50,7(-55,9;-44,6)
Bahia	17822,2 (16003,5;19631,3)	288,4 (255,5;317,4)	28572,6 (24486,2;32622,6)	176 (151,4;201,1)	-39(-48,2;-28,5)
Ceará	9061,7 (7840,2;10176,8)	231,2 (198,8;260,9)	17908,4 (15189,3;20616,8)	182,6 (154,9;210,3)	-21(-34,2;-4,9)
Distrito Federal	1643,6 (1503,3;1806,1)	444,2 (408,3;477,6)	3389,8 (3043,6;3756,2)	185,8 (165,1;204,6)	-58,2(-62,4;-53,3)
Espírito Santo	4410,6 (4203,1;4562,4)	374,8 (348,4;389,6)	7850,8 (6919,7;8701,8)	191,8 (167,7;212,9)	-48,8(-54,2;-43,7)
Goiás	6519,1 (5763,1;7447,3)	389,1 (347;440,7)	11384,2 (9683,2;13202,3)	176,2 (149,7;204,1)	-54,7(-62;-46,4)
Maranhão	6817,6 (5871,1;7739,5)	288,3 (245,9;327,4)	15483,5 (13437,5;17842,1)	242,1 (210,4;278,9)	-16(-29,3;1,4)
Mato Grosso	1985,3 (1769,3;2199,9)	319,4 (284,1;349)	4651,3 (4170,1;5171,1)	155,6 (137,5;172,7)	-51,3(-56,6;-44,7)
Mato Grosso do Sul	2631,9 (2474,4;2769,5)	366 (338,5;386,8)	4856,1 (4328,6;5378,6)	177,3 (157,9;196,2)	-51,6(-56,5;-46,5)
Minas Gerais	30599,1 (28786,2;32249,8)	375,8 (347,3;396,3)	38760 (34341,1;42744,9)	147,8 (130,9;163)	-60,7(-64,4;-57)
Pará	5594,9 (5019,4;6163)	337 (300,4;370,7)	10746,4 (9457,6;11880,5)	163,8 (144;181,1)	-51,4(-57;-45)
Paraíba	5822,3 (5293,6;6316)	264,8 (239,1;287,5)	8913,3 (7679,8;10085,3)	180,7 (157,5;204,5)	-31,7(-40,4;-22,8)
Paraná	16189,6 (15581,2;16614,3)	423,1 (399,2;437,6)	22072,6 (19565;24449,1)	179,4 (158,4;198,7)	-57,6(-61,6;-53,4)
Pernambuco	13939,5 (13198,3;14460)	352,1 (327,8;367,1)	21121,2 (18791,5;23336,3)	219,9 (195;243,1)	-37,5(-43,7;-31,4)
Piaui	3829,3 (3497,7;4109,5)	331,2 (296,9;355,1)	6848,1 (5908,3;7614,1)	177,6 (154,4;197,1)	-46,4(-52;-40,6)
Rio de Janeiro	36000,2 (34654,8;36880,4)	441,3 (417,7;454,5)	41989,3 (37764,1;46009)	192,5 (172,8;211,1)	-56,4(-60;-52,4)
Rio Grande do Norte	3713,9 (3297,2;4074,3)	245,4 (216,6;269,9)	6158,7 (5180,9;7181,3)	154,5 (130,7;180,1)	-37(-46,6;-26,1)
Rio Grande do Sul	19771,7 (18934,4;20371)	360,4 (338,1;373,8)	25731,6 (22928,2;28470,1)	168,3 (150,1;186,3)	-53,3(-57,3;-49)
Rondônia	933,5 (827,7;1027,6)	461,4 (425,8;493,9)	2447,7 (2140,3;2781,4)	178,2 (155,3;201,6)	-61,4(-66,2;-55,8)
Roraima	145,1 (132,4;156,2)	382,9 (355,3;404,4)	526,7 (481,9;570,5)	186,5 (166,8;201,7)	-51,3(-55,3;-47,1)
Santa Catarina	7804,4 (7403,4;8174,6)	388,7 (361,6;408,4)	12033,1 (10731,6;13309,4)	164,6 (145,7;181,3)	-57,7(-61,6;-53,6)
São Paulo	65063,6 (61927;67978,8)	403,5 (373,9;423,1)	87751,7 (77569,4;96216,6)	171,5 (150,9;187,8)	-57,5(-61,2;-53,8)
Sergipe	2033,8 (1875,3;2190,6)	319 (292,8;343,3)	3755,8 (3258,8;4282,6)	173,6 (150,1;197,5)	-45,6(-52,8;-37,6)
Tocantins	983,6 (877,3;1090,3)	355,3 (318,1;389,4)	2721 (2382,9;3072)	204 (178,1;230,4)	-42,6(-50,2;-34,5)

*Fonte: Dados derivados do estudo Global Burden of Disease 2019, Institute for Health Metrics and Evaluation, University of Washington. ^46^*

**Tabela 1-4:** – Mortalidade proporcional por doenças cardiovasculares (DCV), doença isquêmica do coração (DIC) e acidente vascular cerebral (AVC) por região brasileira, unidade federativa e no Brasil, em 2019.

Regiões/UF	DCV/Total	DIC/DCV	AVC/DCV
	%	%	%
Norte	22,9	30,8	33,1
RO	23,4	28,9	27,0
AC	22,6	36,5	30,0
AM	18,5	28,8	36,9
RR	22,0	27,2	35,7
PA	23,8	31,9	34,2
AP	20,2	30,5	32,8
TO	29,0	30,0	29,3
Nordeste	27,3	32,5	29,9
MA	30,5	32,0	32,5
PI	31,3	30,1	33,7
CE	27,4	34,2	28,8
RN	28,2	41,1	24,4
PB	28,3	35,3	25,7
PE	27,8	37,9	28,5
AL	31,0	30,1	31,0
SE	23,3	27,4	32,4
BA	24,0	26,2	31,5
Sudeste	27,7	32,6	25,3
MG	25,2	23,9	27,6
ES	28,8	33,7	29,2
RJ	26,9	33,8	24,6
SP	29,2	35,5	24,3
Sul	26,3	30,9	30,1
PR	27,2	29,4	30,2
SC	27,1	31,0	26,6
RS	25,1	32,1	31,7
Centro-Oeste	26,1	33,1	27,6
MS	29,1	39,2	25,8
MT	24,2	30,2	28,0
GO	26,2	32,1	26,6
DF	24,5	31,4	32,9
Brasil	27,0	32,3	27,8

*Fonte: Sistema de Informações sobre Mortalidade – SIM/DATASUS. ^43^*

**Tabela 1-5:** – Número de DALYs e taxas de DALYs padronizadas por idade por doenças cardiovasculares (por 100 mil habitantes) e variação percentual das taxas no Brasil e suas unidades federativas, em 1990 e 2019.

Causa de morte e local	1990		2019		Variação percentual (II 95%)
	Número (II 95%)	Taxa (II 95%)	Número (II 95%)	Taxa (II 95%)	
**B.2-Doenças Cardiovasculares**					
Brasil	7006215,8 (6793265,2;7175766,7)	7496,4 (7214,2;7700,4)	8861401,5 (8394308,2;9258967,5)	3769,7 (3563,7;3941,2)	-49,7(-51,7;-47,9)
Acre	9825,4 (9146,4;10435,1)	5453,4 (5104,7;5755)	22488,6 (20552;24398,9)	3486,4 (3186,5;3773,4)	-36,1(-41,5;-29,9)
Alagoas	110461,2 (103870,2;118711,8)	7466,1 (6979,1;8003,9)	163441,5 (145907,2;181349,4)	5003,3 (4464,2;5545,6)	-33(-40,9;-24,4)
Amapá	5067,3 (4685,6;5415,9)	4630,7 (4327,3;4912,7)	18522,5 (17183,3;19974,3)	3307,7 (3047;3555,6)	-28,6(-34,2;-23)
Amazonas	45754,5 (42072,2;49330,8)	5541,4 (5130,9;5952,4)	89897,2 (81155,2;99494,8)	2991,8 (2679,2;3314,4)	-46(-51,9;-39,4)
Bahia	452812,5 (412140,4;496636)	6276,5 (5696,4;6892,4)	637197,3 (552757,5;722888,3)	3926,1 (3415,5;4453,8)	-37,4(-47;-26,7)
Ceará	220789,6 (195330,6;246721,7)	5001,5 (4427,7;5606,9)	378371,9 (324694,5;435882,1)	3785,3 (3251,5;4360,4)	-24,3(-37,3;-8,7)
Distrito Federal	52059,2 (47758,5;57091,7)	8173,4 (7548,7;8867,2)	81469,6 (73653,4;89826,9)	3262,3 (2942,3;3586,6)	-60,1(-64,4;-55,1)
Espírito Santo	113555,9 (109578,7;117427)	7455 (7140,5;7713,2)	176105,4 (156866,3;194211,4)	4041,5 (3597,1;4452,3)	-45,8(-51,3;-40,3)
Goiás	184416,2 (163600,3;210275,7)	8106 (7232,6;9221,2)	271049,9 (232375,2;312410,9)	3824,8 (3296,7;4398,5)	-52,8(-60,8;-43,6)
Maranhão	201000,2 (172783,1;228799,1)	6794,1 (5859,4;7722,4)	335711,6 (289713,2;391241,8)	4982,5 (4297,2;5816,9)	-26,7(-39,4;-9,2)
Mato Grosso	57476,4 (50382,9;64092,5)	6506,9 (5833,5;7149,3)	113821 (103871,1;125263,6)	3361,9 (3064,4;3696,7)	-48,3(-54,4;-40,7)
Mato Grosso do Sul	72483,1 (68659,5;76315,7)	7521,8 (7098,7;7914,8)	111484,1 (100917,1;123161,6)	3765,4 (3415,8;4150)	-49,9(-55;-44,4)
Minas Gerais	816075,2 (772344,5;861709,5)	7931,8 (7484,4;8365)	861429,4 (783971,7;940221,9)	3293,7 (2995,9;3591,5)	-58,5(-62,3;-54,5)
Pará	142720,7 (128334,6;157462)	6482,5 (5843,6;7126,1)	254515,4 (229761,6;279907,4)	3522,1 (3175,7;3867,1)	-45,7(-52,5;-38,1)
Paraíba	135924,9 (125409;147002)	5725,1 (5271,7;6197,9)	186763,1 (165980,5;210012,9)	3955,7 (3519,7;4448,8)	-30,9(-39,7;-22,1)
Paraná	415852,5 (404061,8;427856,4)	8363,5 (8061,3;8600)	479641,4 (431236,2;529867,9)	3653,3 (3287,2;4026,1)	-56,3(-60,4;-51,9)
Pernambuco	341781,1 (329624,5;354781,3)	7291 (6985,5;7564,9)	482460,4 (433602,6;531932,7)	4764,3 (4292,5;5249,2)	-34,7(-41,2;-28,3)
Piaui	97460,7 (90810,1;104026,2)	6600,2 (6091;7040)	141273 (126999,6;154951,2)	3743,8 (3367,7;4104,8)	-43,3(-49,3;-37)
Rio de Janeiro	945339,2 (918332,6;969826)	9470,4 (9161,4;9714,6)	946417,6 (860018,3;1034911,6)	4280,1 (3886,7;4676,3)	-54,8(-58,6;-50,6)
Rio Grande do Norte	84287 (76592,3;91924,1)	5086,1 (4601,8;5568,9)	132013,6 (113876,1;152147,5)	3382,5 (2917;3896)	-33,5(-43,7;-21,8)
Rio Grande do Sul	487134,2 (472994;500925,1)	7306,3 (7045;7526,8)	521946,9 (472984,7;572058,4)	3430,1 (3115,3;3752,2)	-53,1(-57;-48,6)
Rondônia	29859,8 (25915;33218,3)	8384,3 (7681,5;9083,1)	58381,7 (51876,1;66033,5)	3711,8 (3304;4188)	-55,7(-61,7;-48,5)
Roraima	4589 (4110,6;5005,9)	7068,2 (6561,2;7526,1)	13684,7 (12591,8;14854,5)	3543,2 (3254,2;3826,2)	-49,9(-54,3;-44,8)
Santa Catarina	194673,4 (185924,3;203639,1)	7562 (7195,5;7917,7)	262278,8 (236530;291520,9)	3277,3 (2965,5;3629,3)	-56,7(-60,8;-52,2)
São Paulo	1707145 (1633495,5;1778765,1)	8196,3 (7805,8;8547,4)	1975598 (1798088,4;2156089,9)	3684,9 (3354,2;4015,8)	-55(-58,9;-51,1)
Sergipe	49627,8 (45878,5;53350,1)	6106,6 (5653,9;6589,7)	84931,7 (74523,6;96498,9)	3726,6 (3282,2;4226,8)	-39(-47,1;-29,7)
Tocantins	28043,7 (25082,9;31090,9)	6599,3 (5948,1;7273,5)	60505,7 (53333,3;68340,1)	4170,5 (3667,3;4705,6)	-36,8(-45,7;-27,2)

*Fonte: Dados derivados do estudo Global Burden of Disease 2019, Institute for Health Metrics and Evaluation, University of Washington. ^46^*

**Tabela 1-6:** – Número de YLLs e taxas de YLLs padronizadas por idade por doenças cardiovasculares (por 100 mil habitantes) e variação percentual das taxas, de acordo com o grupo etário, no Brasil, em 1990 e 2019.

Causa de morte e grupo etário	1990		2019		Variação percentual (II 95%)
	Número (II 95%)	Taxa (II 95%)	Número (II 95%)	Taxa (II 95%)	
**B.2-Doenças cardiovasculares**					
Abaixo de 5	161337,8 (139792,5;192936,7)	952,5 (825,3;1139,1)	57843,6 (45826,5;71192,8)	373,3 (295,7;459,4)	-60,8(-70,6;-48,9)
5-14 anos	62158,5 (57376;66402,3)	175,9 (162,4;187,9)	30871,4 (26559,7;34588,1)	95,7 (82,4;107,3)	-45,6(-52,8;-38,4)
15-49 anos	1730356,8 (1689454,9;1774210,6)	2257,6 (2204,2;2314,8)	1478471,7 (1422955,7;1538181,6)	1280,2 (1232,1;1331,9)	-43,3(-45,8;-40,6)
50-69 anos	2840819,3 (2775664,6;2909002,7)	18108,6 (17693,3;18543,3)	3565725,8 (3424289;3705718,6)	8838,5 (8487,9;9185,5)	-51,2(-53,3;-49)
70+ anos	1841603,2 (1729243,4;1906089,7)	43536 (40879,8;45060,5)	2997320,6 (2653382,7;3166395,5)	22900,4 (20272,6;24192,2)	-47,4(-50,3;-45,3)
Todas as idades	6636275,6 (6454323,9;6792691)	4458,8 (4336,5;4563,9)	8130233,2 (7701177,6;8447854,9)	3752,4 (3554,4;3899)	-15,8(-19,8;-12,5)
Padronizada por idade	6636275,6 (6454323,9;6792691)	7122,2 (6860,4;7309,1)	8130233,2 (7701177,6;8447854,9)	3454,4 (3260,8;3591,7)	-51,5(-53,4;-49,7)

*Fonte: Dados derivados do estudo Global Burden of Disease 2019, Institute for Health Metrics and Evaluation, University of Washington. ^46^*

**Tabela 1-7:** – Número de YLDs e taxas de YLDs padronizadas por idade por doenças cardiovasculares (por 100 mil habitantes) e variação percentual das taxas, de acordo com o grupo etário, no Brasil, em 1990 e 2019.

Causa de morte e grupo etário	1990		2019		Variação percentual (II 95%)
	Número (II 95%)	Taxa (II 95%)	Número (II 95%)	Taxa (II 95%)	
**B.2-Doenças cardiovasculares**					
Abaixo de 5	1565,1 (1007,5;2281,2)	9,2 (5,9;13,5)	1412,2 (917,3;2069,4)	9,1 (5,9;13,4)	-1,4(-6,5;4,4)
5-14 anos	17436,6 (10966,3;26603,3)	49,3 (31;75,3)	16072,8 (9982,1;24508,3)	49,8 (31;76)	1(-2,9;4,9)
15-49 anos	124044,8 (86673,5;165579,4)	161,8 (113,1;216)	185532 (128907,5;249696,9)	160,6 (111,6;216,2)	-0,7(-3,9;2,5)
50-69 anos	125877,6 (90345,3;162729,8)	802,4 (575,9;1037,3)	255231,2 (183302,9;334295,6)	632,6 (454,4;828,6)	-21,2(-24,4;-18,1)
70+ anos	101016,2 (73759,2;129937,3)	2388 (1743,7;3071,8)	272920,1 (198393,9;353367,6)	2085,2 (1515,8;2699,8)	-12,7(-16;-9,2)
Todas as idades	369940,2 (272305,8;476273,8)	248,6 (183;320)	731168,4 (532797,5;954320,6)	337,5 (245,9;440,5)	35,8(31,6;40,3)
Padronizada por idade	369940,2 (272305,8;476273,8)	374,3 (276,1;480,5)	731168,4 (532797,5;954320,6)	315,3 (230,1;411,1)	-15,8(-18,2;-13,6)

*Fonte: Dados derivados do estudo Global Burden of Disease 2019, Institute for Health Metrics and Evaluation, University of Washington.* *^46^*

**Tabela 1-8:** – Número total de hospitalizações para procedimentos clínicos para doenças cardiovasculares por ano de competência, no Brasil, de 2008 a 2019.

	2008	2009	2010	2011	2012	2013	2014	2015	2016	2017	2018	2019	Total
CARDIOPATIA ISQUÊMICA CRÔNICA	12.393	9.743	9.300	8.497	8.000	7.197	7.581	6.403	6.317	6.171	6.292	6.703	94.597
DOENÇA CEREBROVASCULAR	159.545	176.047	181.035	184.751	182.065	183.043	187.110	191.678	195.787	198.068	203.066	211.149	2.253.344
DOENÇA VALVAR	3.237	4.156	3.526	3.637	3.285	2.996	2.753	2.400	2.244	2.231	2.330	2.289	35.084
FIBRILAÇÃO ATRIAL	29.034	28.174	28.382	28.583	28.760	28.268	29.799	29.754	29.889	30.265	30.958	32.753	354.619
INFARTO - CLÍNICO	47.358	50.987	55.513	58.194	59.562	58.552	62.809	66.647	70.441	71.835	74.569	80.614	757.081
INSUFICIÊNCIA CARDÍACA	298.474	297.763	289.110	284.844	264.469	254.285	243.913	240.832	236.358	230.297	222.394	222.620	3.085.359
MIOCARDIOPATIAS	2.092	2.363	2.459	2.302	2.357	2.293	2.370	2.230	2.250	1.997	2.251	2.390	27.354
SÍNDROME CORONARIANA AGUDA	63.300	68.833	72.912	71.523	75.734	73.432	76.945	72.686	70.430	70.713	68.413	70.204	855.125
Total	615.433	638.066	642.237	642.331	624.232	610.066	613.280	612.630	613.716	611.577	610.273	628.722	7.462.563

*Fonte: Sistema de Informações sobre Mortalidade – SIM/DATASUS. ^43^*

**Tabela 1-9:** – Número total de hospitalizações para procedimentos cirúrgicos para doenças cardiovasculares por ano de competência, no Brasil, de 2008 a 2019.

	2008	2009	2010	2011	2012	2013	2014	2015	2016	2017	2018	2019	Total
Ablação de fibrilação atrial	68	72	90	85	123	139	143	161	124	120	125	163	1.413
Angioplastia coronariana	38.635	45.648	49.492	55.931	60.959	63.838	66.492	66.550	69.802	73.971	78.575	85.518	755.411
Cirurgia de revascularização miocárdica	20.515	22.077	21.225	23.187	23.900	23.249	22.997	22.559	22.248	21.474	20.674	21.018	265.123
Cirurgia valvar	12.201	12.664	12.169	13.181	13.435	13.067	12.993	12.624	12.432	12.277	12.088	12.771	151.902
Infarto - angioplastia	7.648	6.362	6.262	6.033	5.865	6.055	7.135	8.524	10.195	10.774	10.811	11.099	96.763
Miocardiopatias	15	43	13	21	28	23	20	18	32	29	26	24	292
Outras valvoplastias	451	477	445	486	456	527	515	513	399	427	391	450	5.537
Valvoplastia mitral	477	551	478	473	403	431	408	341	206	236	200	195	4.399
Total	80.010	87.894	90.174	99.397	105.169	107.329	110.703	111.290	115.438	119.308	122.890	131.238	1.280.840

*Fonte: Sistema de Informações sobre Mortalidade – SIM/DATASUS. ^43^*

**Tabela 1-10:** – Valor total (em Reais) dos procedimentos clínicos para doenças cardiovasculares por ano de competência, no Brasil, de 2008 a 2019.

	2008	2009	2010	2011	2012	2013	2014	2015	2016	2017	2018	2019	Total
CARDIOPATIA ISQUÊMICA CRÔNICA	7.798.577,82	6.860.711,25	6.593.971,61	6.250.858,75	5.689.946,25	5.248.239,82	6.213.647,76	5.140.625,95	5.326.842,16	5.532.381,71	5.672.327,20	6.475.643,96	72.803.774,24
DOENÇA CEREBROVASCULAR	142.061.641,70	188.450.404,06	198.812.522,26	205.447.974,55	218.628.105,94	228.140.748,46	242.663.800,69	252.440.767,47	263.770.567,49	272.140.330,46	286.293.302,84	303.838.674,47	2.802.688.840,39
DOENÇA VALVAR	1.051.959,34	1.589.247,64	1.439.424,14	1.606.640,13	1.509.338,04	1.509.785,49	1.584.222,50	1.672.410,55	1.675.284,06	1.678.874,30	2.043.385,62	1.999.540,54	19.360.112,35
FIBRILAÇÃO ATRIAL	13.790.984,47	17.396.138,42	18.537.031,69	18.858.448,37	20.371.040,61	19.968.864,00	22.636.728,19	23.329.786,10	23.929.183,91	26.060.564,58	26.971.390,07	28.743.440,36	260.593.600,77
INFARTO - CLÍNICO	65.019.330,51	84.308.216,56	92.969.057,67	97.323.922,15	104.897.640,92	106.246.319,66	119.582.977,33	128.723.964,23	134.911.984,37	136.437.974,90	143.349.385,91	151.123.021,25	1.364.893.795,46
INSUFICIÊNCIA CARDÍACA	272.280.662,78	322.849.486,97	327.913.746,49	330.492.446,60	317.585.920,25	321.711.992,20	326.140.931,65	337.610.340,87	345.565.633,18	346.841.126,90	348.832.330,32	359.301.690,55	3.957.126.308,76
MIOCARDIOPATIAS	1.287.646,38	1.901.574,84	2.143.534,84	1.899.610,88	2.110.498,65	2.301.691,59	2.696.303,97	2.681.816,09	3.065.112,69	2.556.468,20	3.119.717,43	3.173.903,30	28.937.878,86
SÍNDROME CORONARIANA AGUDA	44.710.681,49	57.921.695,01	64.611.984,70	65.586.337,38	75.210.291,07	74.619.170,87	83.606.992,07	82.094.816,54	80.185.274,88	82.072.225,98	80.036.822,54	81.167.004,50	871.823.297,03
**Total**	**548.001.484,49**	**681.277.474,75**	**713.021.273,40**	**727.466.238,81**	**746.002.781,73**	**759.746.812,09**	**805.125.604,16**	**833.694.527,80**	**858.429.882,74**	**873.319.947,03**	**896.318.661,93**	**935.822.918,93**	**9.378.227.607,86**

*Fonte: Sistema de Informações sobre Mortalidade – SIM/DATASUS.* ^*43*^

**Tabela 1-11:** – Valor total (em Int$2019) dos procedimentos clínicos para doenças cardiovasculares por ano de competência, no Brasil, de 2008 a 2019.

	Int$2019	Int$2019	Int$2019	Int$2019	Int$2019	Int$2019	Int$2019	Int$2019	Int$2019	Int$2019	Int$2019	Int$2019	Int$2019
	**2008**	**2009**	**2010**	**2011**	**2012**	**2013**	**2014**	**2015**	**2016**	**2017**	**2018**	**2019**	**Total**
CARDIOPATIA ISQUÊMICA CRÔNICA	$ 7.650.265,61	$ 6.274.085,62	$ 5.560.158,75	$ 4.864.286,14	$ 4.101.608,01	$ 3.519.939,65	$ 3.863.867,68	$ 2.971.845,26	$ 2.849.164,90	$ 2.859.497,35	$ 2.845.701,28	$ 3.129.842,42	$ 50.490.262,67
DOENÇA CEREBROVASCULAR	$ 139.359.934,26	$ 172.336.938,05	$ 167.642.393,83	$ 159.875.270,76	$ 157.598.464,21	$ 153.011.617,97	$ 150.897.001,54	$ 145.938.433,52	$ 141.082.806,42	$ 140.659.953,49	$ 143.628.036,74	$ 146.852.911,78	$ 1.818.883.762,57
DOENÇA VALVAR	$ 1.031.953,33	$ 1.453.358,90	$ 1.213.749,04	$ 1.250.253,39	$ 1.088.009,05	$ 1.012.597,36	$ 985.126,02	$ 966.836,61	$ 896.058,19	$ 867.752,24	$ 1.025.128,64	$ 966.428,49	$ 12.757.251,26
FIBRILAÇÃO ATRIAL	$ 13.528.709,55	$ 15.908.680,29	$ 15.630.767,78	$ 14.675.245,87	$ 14.684.501,34	$ 13.392.908,59	$ 14.076.324,52	$ 13.487.173,53	$ 12.798.988,35	$ 13.469.807,27	$ 13.531.045,80	$ 13.892.431,30	$ 169.076.584,19
INFARTO - CLÍNICO	$ 63.782.802,43	$ 77.099.436,14	$ 78.393.227,96	$ 75.735.418,85	$ 75.615.653,52	$ 71.258.297,28	$ 74.360.958,09	$ 74.416.560,70	$ 72.160.292,77	$ 70.520.084,88	$ 71.915.726,50	$ 73.041.576,24	$ 878.300.035,36
INSUFICIÊNCIA CARDÍACA	$ 267.102.469,11	$ 295.244.217,24	$ 276.502.932,54	$ 257.182.235,55	$ 228.932.383,01	$ 215.768.874,17	$ 202.806.057,29	$ 195.175.782,35	$ 184.832.484,51	$ 179.270.219,50	$ 175.002.706,17	$ 173.659.589,44	$ 2.651.479.950,88
MIOCARDIOPATIAS	$ 1.263.158,11	$ 1.738.980,54	$ 1.807.468,20	$ 1.478.237,03	$ 1.521.356,76	$ 1.543.720,52	$ 1.676.657,92	$ 1.550.383,65	$ 1.639.435,00	$ 1.321.350,27	$ 1.565.104,34	$ 1.534.027,69	$ 18.639.880,03
SÍNDROME CORONARIANA AGUDA	$ 43.860.380,31	$ 52.969.096,11	$ 54.482.019,85	$ 51.037.901,30	$ 54.215.473,87	$ 50.046.298,80	$ 51.989.808,02	$ 47.459.802,34	$ 42.888.650,24	$ 42.420.303,78	$ 40.152.988,47	$ 39.230.065,00	$ 570.752.788,09
Total	$537.579.672,71	$623.024.792,89	$ 601.232.717,95	$566.098.848,89	$537.757.449,77	$509.554.254,34	$ 500.655.801,08	$ 481.966.817,96	$ 459.147.880,38	$ 451.388.968,78	$ 449.666.437,94	$ 452.306.872,36	$ 6.170.380.515,05

*Fonte: Sistema de Informações sobre Mortalidade – SIM/DATASUS.* ^*43*^

**Tabela 1-12:** – Valor total (em Reais) dos procedimentos cirúrgicos para doenças cardiovasculares por ano de competência, no Brasil, de 2008 a 2019.

	2008	2009	2010	2011	2012	2013	2014	2015	2016	2017	2018	019	Total
ABLAÇÃO DE FIBRILAÇÃO ATRIAL	360.473,27	376.837,24	471.138,73	456.926,51	690.004,54	788.451,61	770.674,50	906.324,09	707.088,34	690.689,60	732.003,78	961.948,99	7.912.561,20
ANGIOPLASTIA CORONARIANA	210.528.789,87	266.654.421,74	295.641.177,27	337.972.226,29	372.062.717,78	388.919.617,05	411.251.867,70	412.073.165,85	433.590.492,36	466.696.227,86	495.885.258,42	546.132.198,85	4.637.408.161,04
CIRURGIA DE REVASCULARIZAÇÃO MIOCÁRDICA	176.032.311,77	208.585.452,82	214.483.618,24	287.851.733,82	297.843.568,00	290.540.953,96	294.854.279,38	289.638.019,51	286.159.517,75	282.175.032,32	275.110.233,84	278.544.223,76	3.181.818.945,17
CIRURGIA VALVAR	125.954.499,60	140.683.968,83	142.383.177,39	179.111.011,96	183.271.263,12	178.563.635,59	180.088.277,85	176.813.774,47	175.318.559,92	176.135.832,05	177.583.849,27	187.382.032,83	2.023.289.882,88
INFARTO - ANGIOPLASTIA	45.267.283,32	37.887.853,46	37.112.662,15	35.576.928,51	35.545.437,05	37.287.935,69	45.883.433,02	56.100.757,09	66.515.237,46	71.624.171,14	73.429.322,18	74.907.756,33	617.138.777,40
MIOCARDIOPATIAS	168.992,82	544.719,64	192.352,38	325.920,92	436.100,76	353.814,17	305.552,03	298.288,99	526.912,73	451.789,68	426.075,02	404.878,61	4.435.397,75
OUTRAS VALVOPLASTIAS	1.518.843,49	1.661.544,32	1.717.544,82	1.918.678,81	1.870.621,37	2.051.540,92	2.128.294,09	2.085.967,07	1.594.213,11	1.888.744,84	1.689.593,28	1.959.571,29	22.085.157,41
VALVOPLASTIA MITRAL	3.115.254,91	3.585.355,62	3.147.310,18	3.227.816,88	2.718.391,46	2.970.343,43	2.808.556,55	2.392.670,47	1.377.571,02	1.720.524,66	1.461.666,40	1.430.166,52	29.955.628,10
Total	562.946.449,05	659.980.153,67	695.148.981,16	846.441.243,70	894.438.104,08	901.476.292,42	938.090.935,12	940.308.967,54	965.789.592,69	1.001.383.012,15	1.026.318.002,19	1.091.722.777,18	10.524.044.510,95

*Fonte: Sistema de Informações sobre Mortalidade – SIM/DATASUS. ^43^*

**Tabela 1-13:** – Valor total (em Int$2019) dos procedimentos cirúrgicos para doenças cardiovasculares por ano de competência, no Brasil, de 2008 a 2019.

	Int$ 2019	Int$ 2019	Int$ 2019	Int$ 2019	Int$ 2019	Int$ 2019	Int$ 2019	Int$ 2019	Int$ 2019	Int$ 2019	Int$ 2019	Int$ 2019	Int$ 2019
	**2008**	**2009**	**2010**	**2011**	**2012**	**2013**	**2014**	**2015**	**2016**	**2017**	**2018**	**2019**	**Total**
ABLAÇÃO DE FIBRILAÇÃO ATRIAL	$ 353.617,84	$ 344.615,74	$ 397.272,89	$ 355.570,55	$ 497.391,02	528.806,26	479.232,88	523.954,67	378.199,92	356.993,60	367.232,71	464.934,26	5.047.822,34
ANGIOPLASTIA CORONARIANA	$ 206.524.984,25	$ 243.854.115,30	$ 249.290.105,61	$ 263.002.841,99	$ 268.202.080,69	260.844.326,45	255.731.071,29	238.223.457,02	231.914.288,54	241.219.188,62	248.776.431,05	263.959.496,79	2.971.542.387,60
CIRURGIA DE REVASCULARIZAÇÃO MIOCÁRDICA	$ 172.684.555,11	$ 190.750.337,95	$ 180.856.551,64	$ 224.000.134,26	$ 214.701.072,80	194.862.784,28	183.350.901,63	167.442.522,38	153.057.970,91	145.846.544,88	138.017.698,57	134.627.464,36	2.100.198.538,77
CIRURGIA VALVAR	$ 123.559.115,42	$ 128.654.775,47	$ 120.060.127,13	$ 139.380.403,22	$ 132.111.420,33	119.760.766,01	111.985.310,80	102.217.742,13	93.772.533,78	91.038.538,47	89.090.521,41	90.566.473,09	1.342.197.727,26
INFARTO - ANGIOPLASTIA	$ 44.406.396,77	$ 34.648.249,69	$ 31.294.082,75	$ 27.685.213,69	$ 25.622.992,36	25.008.629,14	28.531.954,26	32.432.386,78	35.576.965,46	37.020.064,48	36.838.128,17	36.204.812,15	395.269.875,70
MIOCARDIOPATIAS	$ 165.778,94	$ 498.143,35	$ 162.195,08	$ 253.624,77	$ 314.364,02	237.299,47	190.003,14	172.443,73	281.829,50	233.514,51	213.753,93	195.688,07	2.918.638,51
OUTRAS VALVOPLASTIAS	$ 1.489.958,35	$ 1.519.473,85	$ 1.448.265,54	$ 1.493.075,29	$ 1.348.440,70	1.375.947,07	1.323.449,10	1.205.917,61	852.695,82	976.227,08	847.637,59	947.110,34	14.828.198,34
VALVOPLASTIA MITRAL	$ 3.056.009,45	$ 3.278.789,52	$ 2.653.870,12	$ 2.511.818,86	$ 1.959.557,38	1.992.178,33	1.746.460,58	1.383.225,79	736.820,50	889.279,87	733.290,91	691.235,63	21.632.536,94
Total	$ 552.240.416,13	$ 603.548.500,87	$ 586.162.470,76	$ 658.682.682,63	$ 644.757.319,30	$ 604.610.737,01	$ 583.338.383,68	$ 543.601.650,11	$ 516.571.304,43	$ 517.580.351,51	$ 514.884.694,34	$ 527.657.214,69	$ 6.853.635.725,46

**Figura 1-1:**
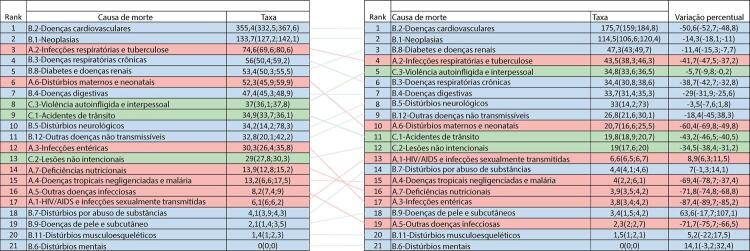
– Ranking das causas de morte no Brasil, 1990 e 2019, de acordo com as taxas de mortalidade padronizadas por idade por 100 mil habitantes, ambos os sexos, 1990 e 2019, e variação percentual das taxas.

Fonte: Dados derivados do estudo Global Burden of Disease 2019, Institute for Health Metrics and Evaluation, University of Washington. ^46^

**Figura 1-2:**
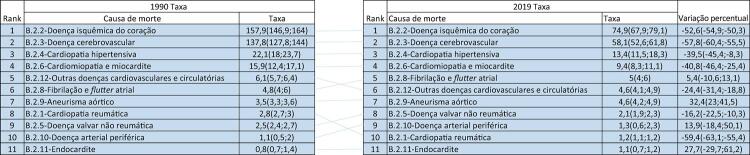
– Ranking das causas de morte cardiovascular no Brasil, 1990 e 2019, de acordo com as taxas de mortalidade padronizadas por idade por 100 mil habitantes, ambos os sexos, 1990 e 2019, e variação percentual das taxas.

Fonte: Dados derivados do estudo Global Burden of Disease 2019, Institute for Health Metrics and Evaluation, University of Washington. ^46^

**Figura 1-3:**
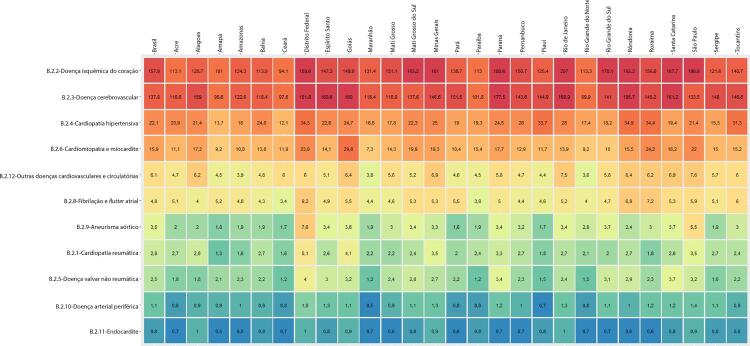
– Ranking das causas de morte cardiovascular por unidade federativa brasileira em 1990, de acordo com as taxas de mortalidade padronizadas por idade por 100 mil habitantes, ambos os sexos.

Fonte: Dados derivados do estudo Global Burden of Disease 2019, Institute for Health Metrics and Evaluation, University of Washington. ^46^

**Figura 1-4:**
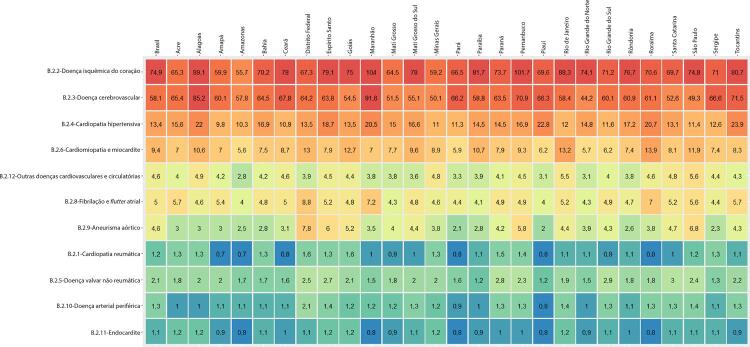
– Ranking das causas de morte cardiovascular por unidade federativa brasileira em 2019, de acordo com as taxas de mortalidade padronizadas por idade por 100 mil habitantes, ambos os sexos.

Fonte: Dados derivados do estudo Global Burden of Disease 2019, Institute for Health Metrics and Evaluation, University of Washington. ^46^

**Figura 1-5:**
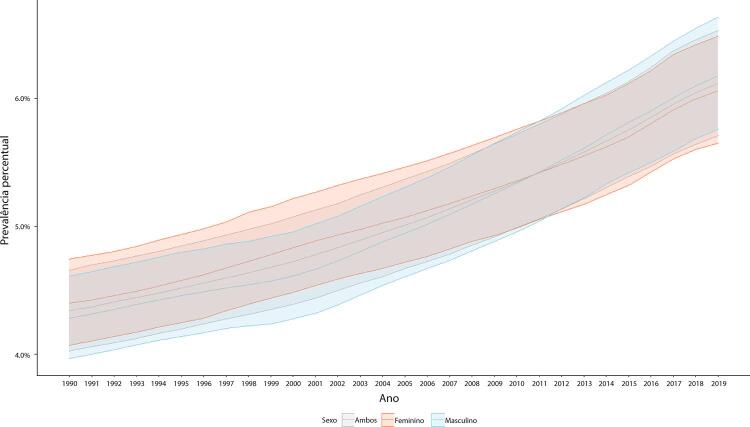
– Prevalência percentual de doença cardiovascular, por sexo, no Brasil, 1990-2019.

Fonte: Dados derivados do estudo Global Burden of Disease 2019, Institute for Health Metrics and Evaluation, University of Washington. ^46^

**Figura 1-6:**
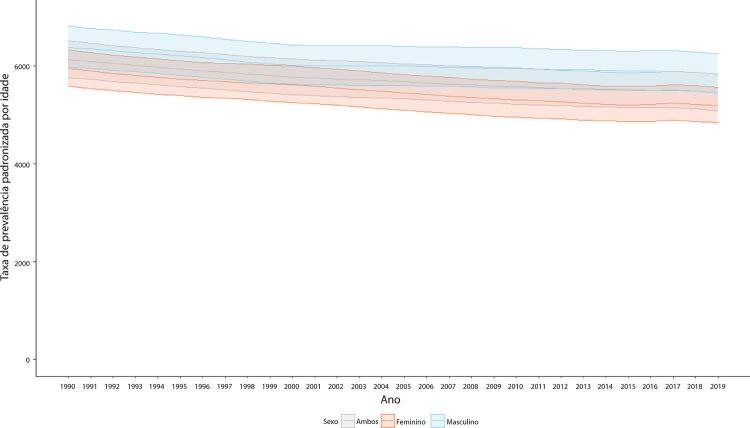
– Taxas de prevalência de doença cardiovascular padronizadas por idade, por 100 mil habitantes, por sexo, Brasil, 1990-2019.

Fonte: Dados derivados do estudo Global Burden of Disease 2019, Institute for Health Metrics and Evaluation, University of Washington. ^46^

**Figura 1-7:**
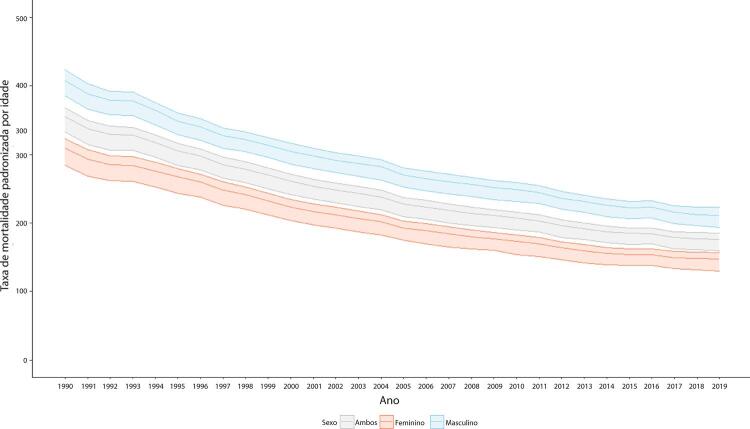
– Taxas de mortalidade por doença cardiovascular padronizadas por idade, por 100 mil habitantes, por sexo, Brasil, 1990-2019.

Fonte: Dados derivados do estudo Global Burden of Disease 2019, Institute for Health Metrics and Evaluation, University of Washington. ^46^

**Figura 1-8:**
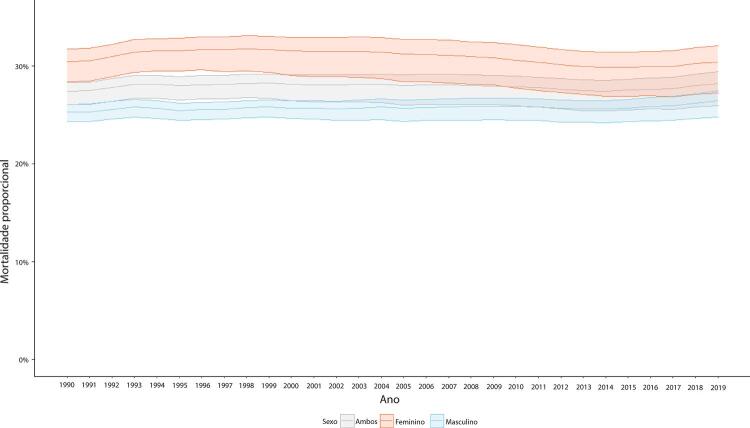
– Mortalidade proporcional por doença cardiovascular, por sexo, Brasil, 1990-2019.

Fonte: Dados derivados do estudo Global Burden of Disease 2019, Institute for Health Metrics and Evaluation, University of Washington. ^46^

**Figura 1-9:**
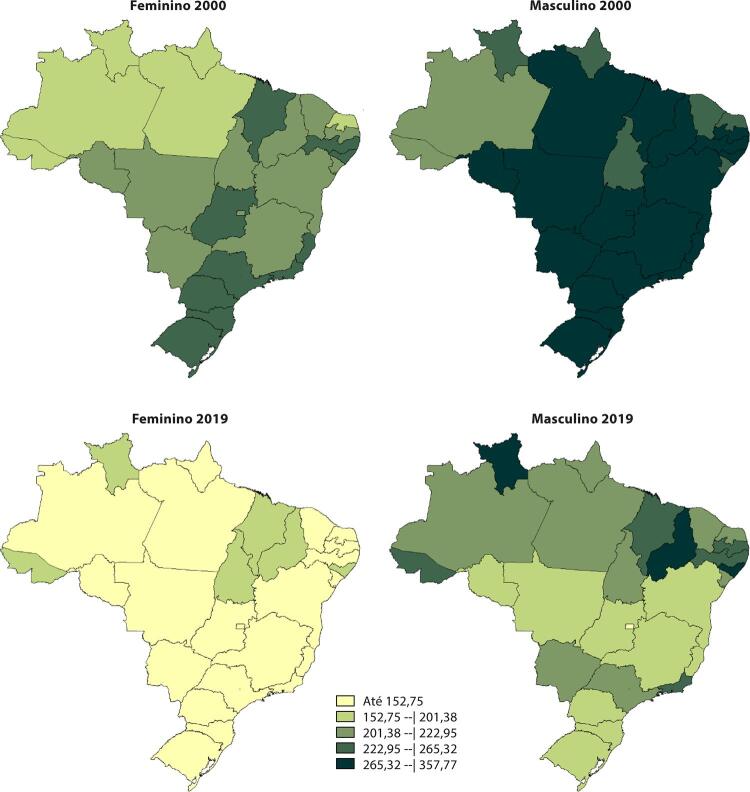
– Distribuição geográfica das taxas de mortalidade padronizadas por idade, por 100 mil habitantes, nas unidades federativas do Brasil, de acordo com sexo, 2000 e 2019.

Fonte: Sistema de Informações sobre Mortalidade – SIM/DATASUS, com redistribuição de causas mal definidas e correção para subnotificação (de acordo com os coeficientes do GBD 2017), e população IBGE. ^43^

**Figura 1-10:**
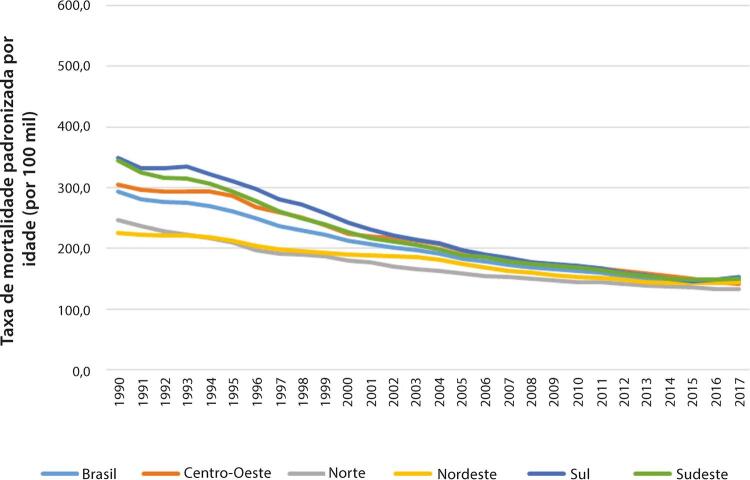
– Taxa de mortalidade por doença cardiovascular padronizada por idade, por 100 mil habitantes, por regiões brasileiras, para mulheres, 1990-2019.

Fonte: Dados derivados do estudo Global Burden of Disease 2019, Institute for Health Metrics and Evaluation, University of Washington. ^46^

**Figura 1-11:**
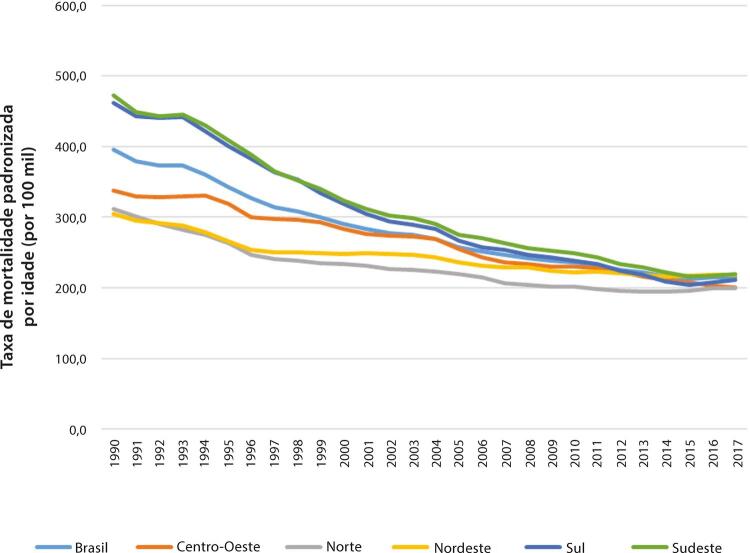
– Taxa de mortalidade por doença cardiovascular padronizada por idade, por 100 mil habitantes, por regiões brasileiras, para homens, 1990-2019.

Fonte: Dados derivados do estudo Global Burden of Disease 2019, Institute for Health Metrics and Evaluation, University of Washington. ^46^

**Figura 1-12:**
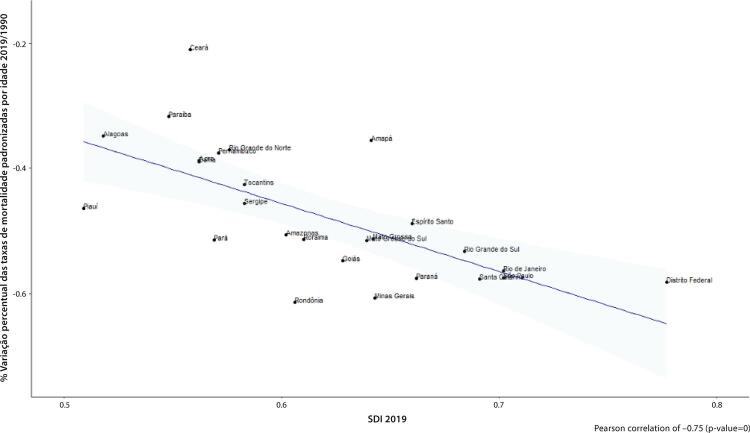
– Correlação entre a variação percentual das taxas de mortalidade padronizadas por idade 2019/1990 e o índice sociodemográfico 2019 (SDI 2019)

Fonte: Dados derivados do estudo Global Burden of Disease 2019, Institute for Health Metrics and Evaluation, University of Washington. ^46^

**Figura 1-13:**
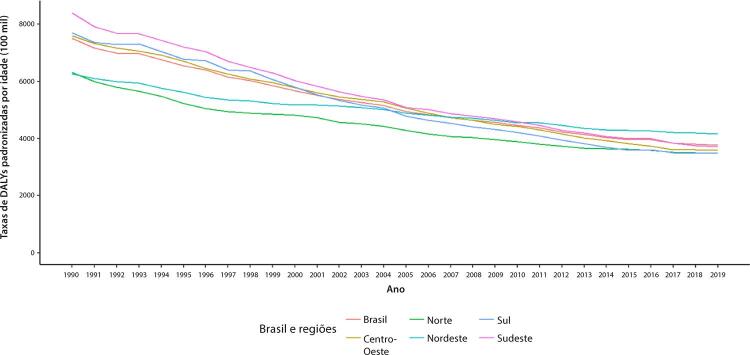
– Taxas de DALYs padronizadas por idade para doença cardiovascular, por 100 mil habitantes, 1990-2017, Brasil e suas regiões.

Fonte: Dados derivados do estudo Global Burden of Disease 2019, Institute for Health Metrics and Evaluation, University of Washington. ^46^

**Figura 1-14:**
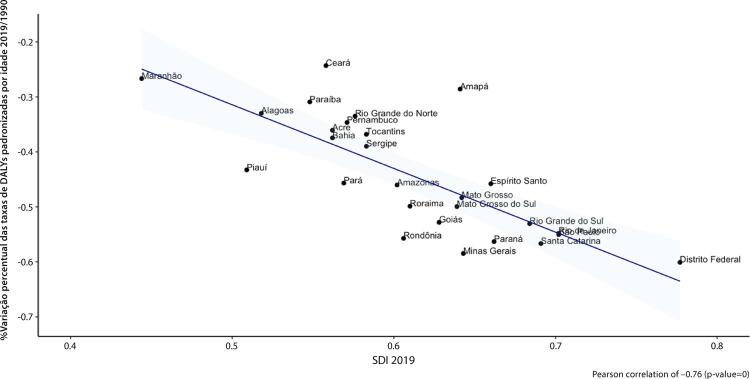
– Correlação entre a variação percentual das taxas de DALYs padronizadas por idade 2019/1990 e o índice sociodemográfico 2019 (SDI 2019).

Fonte: Dados derivados do estudo Global Burden of Disease 2019, Institute for Health Metrics and Evaluation, University of Washington. ^46^

**Figura 1-15:**
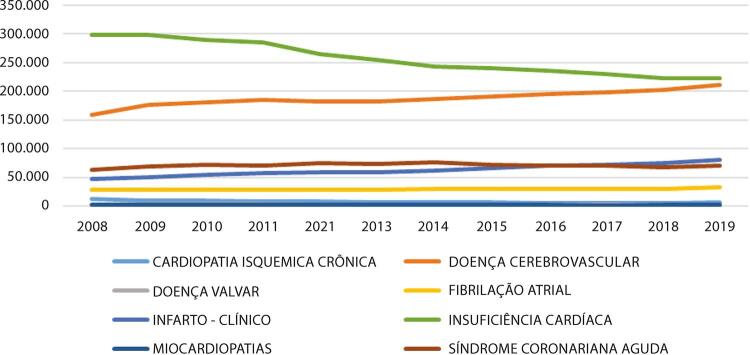
– Total de hospitalizações para procedimentos clínicos para doenças cardiovasculares por ano de competência, Brasil, 2008 a 2019.

Fonte: Sistema de Informações sobre Mortalidade – SIM/DATASUS. ^43^

**Figura 1-16:**
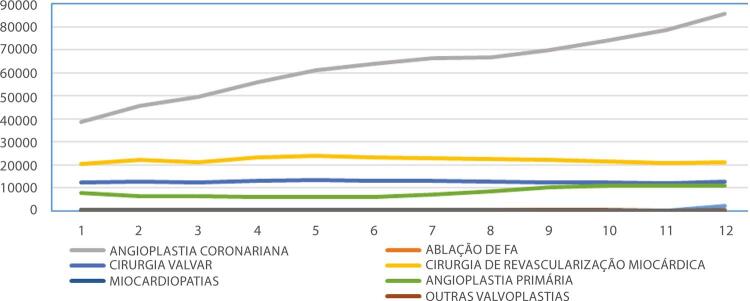
– Total de hospitalizações para procedimentos cirúrgicos para doenças cardiovasculares por ano de competência, Brasil, 2008 a 2019.

Fonte: Sistema de Informações sobre Mortalidade – SIM/DATASUS. ^43^

### Panorama

•As DCNT constituem o principal grupo de causa de morte em todo o mundo, sendo responsáveis por mortes prematuras, perda de qualidade de vida, além de impactos adversos econômicos e sociais. As DCNT são responsáveis por cerca de 70% das mortes globais, equivalendo a mais de 38 milhões de mortes por ano, excedendo significativamente as mortes por causas externas e por doenças infecciosas. ^^23^ – ^26^^ Cerca de 45% de todas as mortes por DCNT no mundo, mais de 17 milhões, são causadas por DCV. Distribuição similar é observada no Brasil, onde 72% das mortes resultam de DCNT, sendo 30% devidas a DCV e 16% a neoplasias ( Figura 1-1 ). ^21^ ^,^ ^27^ ^,^ ^28^

•A definição de DCV pode variar de acordo com o estudo, desde a inclusão de todas as doenças listadas no Capítulo IX da CID-10 até o simples agrupamento das 3 principais causas (DIC, AVC e insuficiência cardíaca). Para o GBD, a definição de DCV total engloba 10 causas: cardiopatia reumática, DIC, doença cerebrovascular, cardiopatia hipertensiva, cardiomiopatia, miocardite, fibrilação e *flutter* atrial, aneurisma aórtico, doença vascular periférica e endocardite. ^29^

•As DCV são a principal causa de morte no Brasil. De acordo com as estimativas do Estudo GBD 2019, entre as DCV, a DIC era a causa número 1 de morte no país, seguida por AVC, em 1990 e 2019 ( Figura 1-2 ). Na verdade, em 2019, a DIC foi a principal causa de morte em todas as UF brasileiras, exceto no Amazonas, na região Norte. Três estados nessa região, Acre, Amapá e Pará, não apresentaram diferença significativa quanto às taxas de mortalidade por DIC e AVC (Figuras 1-3 e 1-4 ).

### Prevalência

•Gonçalves *et al.* publicaram em 2019 um estudo transversal que analisou informação da Pesquisa Nacional de Saúde conduzida em 2013 em uma amostra de 60.202 adultos com mais de 18 anos, estratificados por sexo e grupos etários, usando um modelo de regressão logística binário e hierárquico. O diagnóstico autorreferido de doença cardíaca no Brasil foi de 4,2% (IC 95%: 4,0-4,3 ) e associado com as seguintes características: sexo feminino (OR = 1,1; IC 95%: 1,1-1,1), indivíduos de 65 anos ou mais (OR = 4,7; IC 95%, 3,3-5), hipertensão (OR = 2,4; IC 95%: 2,2-2,7), colesterol elevado (OR = 1,6; IC 95%: 1,5-1,8), sobrepeso (OR = 1,5; IC 95%: 1,4-1,8) ou obesidade (OR = 2,0; IC 95%: 1,7-2,2), sedentarismo (OR = 1,5; IC 95%: 1,02-2,1) e tabagismo (OR = 1,2; IC 95%: 1,03-1,3). ^30^

•No estudo ELSA-Brasil, uma coorte que incluiu 15.105 funcionários públicos de 6 instituições acadêmicas (54% mulheres, 35-74 anos, com avaliação basal entre 2008 e 2010), a prevalência autorreferida foi a seguinte: DIC, 4,7% (homens=5,7%, mulheres=4,0%); insuficiência cardíaca, 1,5% (homens=1,9%, mulheres=1,5%); AVC, 1,3% para ambos os sexos; febre reumática, 2,9% (homens=2,2%, mulheres=3,4%); e doença de Chagas, 0,4% para ambos os sexos. ^31^

•A prevalência de DCV aumenta significativamente com a idade. Em um estudo longitudinal com idosos a partir dos 60 anos, do estado de São Paulo, em 2000, 2006 e 2010, a prevalência de DCV foi definida como resposta positiva à pergunta: “Algum médico ou enfermeiro já lhe disse que você teve um ataque cardíaco, doença isquêmica do coração, angina, doença congestiva ou outros problemas cardíacos?”. A prevalência de DCV foi de 17,9%, 22,2% e 22,9% em 2000, 2006 e 2010, respectivamente. A presença de DCV foi associada a idade mais avançada, história de tabagismo, presença de diabetes e hipertensão. ^32^

•De acordo com o Estudo GBD 2019, a prevalência de DCV foi 6,1% da população em 2019, tendo aumentado desde 1990 devido a crescimento e envelhecimento populacional. No entanto, a taxa de prevalência de DCV padronizada por idade no Brasil diminuiu no mesmo período, passando de 6.138 (II 95%, 5.762 – 6.519) para 5.454 (II 95%, 5.082 – 5.838) por 100 mil habitantes ( Tabela 1-1 ).

•Os homens apresentaram maior taxa de prevalência padronizada por idade do que as mulheres em 2019 (Figuras 1-5 e 1-6 ) e, de 1990 a 2019, uma redução da taxa de prevalência de -8,7 (-10,2 a -7,2), menor do que a das mulheres (-12,8, II 95%, -14,1 a -11,3) no mesmo período ( Figura 1-6 e Tabela 1-1 ). Considerando-se o número total em 2019, 12.946.932 (II 95%, 11.899.752 – 13.617.524) indivíduos apresentaram DCV prevalente no Brasil, 51% dos quais eram homens. A taxa de prevalência diminuiu entre os idosos e subiu entre homens e mulheres de 15-49 anos de idade ( Tabela 1-1 ).

### Incidência

•De acordo com o Estudo GBD 2019, a taxa de incidência de DCV padronizada por idade no Brasil em 2019 foi de 475 (II 95%, 447-507) por 100 mil habitantes. De 1990 a 2019, essa taxa diminuiu -20% (-22 a -18) ( Tabela 1-2 ).

•A taxa de incidência de DCV padronizada por idade não diferiu significativamente entre as UF em 2019, variando de 441 no Piauí a 486 em Pernambuco. De 1990 a 2019, todas as UF apresentaram redução na taxa de incidência de DCV padronizada por idade, tendo o Rio de Janeiro apresentado a maior variação percentual (-25,5%; II 95%, -27,7 a -23,5) ( Tabela 1-2 ).

### Mortalidade

•No Brasil, Mansur *et al* . relataram que a taxa de mortalidade por DCV padronizada por idade diminuiu significativamente nas últimas décadas. Um estudo de 2016 analisou as taxas de mortalidade por DCV a partir dos 30 anos de idade, por sexo, por 100 mil habitantes. As variações anuais na mortalidade cardiovascular nos períodos 1980-2006 e 2007-2012 foram, respectivamente: -1,5% e -0,8%, para ambos os sexos; -1,4% e -0,6%, para homens; -1,7% e -1,0%, para mulheres. ^33^

•Baptista *et al* . investigaram como a composição etária e as taxas de mortalidade específicas por idade se relacionam à diferença observada nas mortes por DCV na população adulta, por sexo, nas microrregiões brasileiras de 1996 a 2015. Aqueles autores sugeriram, após correção para subnotificação das mortes, que houvesse uma redução nas taxas de morte por DCV no período estudado. Entretanto, o principal motivo da mudança nas taxas de mortalidade foi heterogêneo nas microrregiões brasileiras. Em geral, nas áreas mais desenvolvidas socioeconomicamente, a estrutura etária relacionou-se de maneira mais importante às taxas de mortalidade, com as populações mais idosas morrendo por DCV. É interessante notar que os principais motivos de mudança nas taxas de mortalidade por DCV diferiram ainda dentro das regiões e das UF brasileiras. ^34^

•Dados do Estudo GBD 2019 revelam que, embora as taxas de mortalidade por DCV no Brasil tenham diminuído significativamente nos últimos anos, o número total de mortes por DCV aumentou, provavelmente como resultado do crescimento e envelhecimento da população. Houve 269.722,7 (II 95%, 257.743,7 - 277.272,1) e 397.993 (II 95%, 361.776,4 – 417.773,2) mortes por DCV no país em 1990 e 2019, respectivamente. A taxa de mortalidade padronizada por idade por 100 mil habitantes foi 355,4 (332,5 - 367,6) em 1990 e 175,7 (159 - 184,8) em 2019, diminuindo -50,6% (-52,7 a -0,5) no período ( Figura 1-7 ). Embora as taxas de mortalidade padronizadas por idade fossem maiores nos homens em todo o período, a redução percentual foi similar para ambos os sexos, 48% para homens e 52% para mulheres ( Figura 1-8 ).

•A Tabela 1-3 mostra o número de mortes por DCV, a taxa de mortalidade padronizada por idade por 100 mil habitantes e a variação percentual das taxas, por UF e no Brasil, em 1990-2019. Maranhão e Alagoas apresentaram o maior risco de mortalidade, acima da média do país. As maiores reduções percentuais no período foram observadas em Rondônia, Minas Gerais, Distrito Federal, Paraná, Santa Catarina e São Paulo, em ordem decrescente.

•A Figura 1-9 mostra a distribuição geográfica das taxas de mortalidade por 100 mil habitantes, padronizadas por idade nas UF brasileiras, de acordo com o sexo, em 2000 e 2019, conforme dados do SIM e utilizando a população do IBGE. Houve diminuição nas taxas de mortalidade padronizadas em ambos os sexos, exceto para os homens de Roraima, Piauí e Alagoas, a despeito da redistribuição de causas mal definidas e a correção para subnotificação de acordo com os coeficientes do GBD 2019. Malta *et al.* compararam uma série histórica de taxa de mortalidade por DCV no Brasil, usando a base de dados do SIM com e sem correção e as estimativas do GBD 2017 entre 2000 e 2017. Os autores indicaram que o aumento na taxa de mortalidade observado em 2017 em algumas UF do Norte e Nordeste deveu-se às melhorias nos registros de morte e na definição das causas básicas de morte nos últimos anos. ^6^

•Ao analisarem dados do GBD 2015, Brant *et al.* observaram uma redução na taxa de mortalidade por DCV padronizada por idade de 429,5 (1990) para 256,0 (2015) por 100 mil habitantes (-40,4%), com acentuadas diferenças entre as UF. Essa redução foi mais pronunciada nas UF do Sudeste e Sul e no Distrito Federal, regiões que concentram as maiores populações e renda, sendo mais modesta na maioria dos estados do Norte e Nordeste. ^29^

•Importante salientar ainda que a redução anual nas taxas de mortalidade por DCV no Brasil foi menor nos últimos anos ao se considerar o período de 1990-2019, para homens e mulheres.

•Quanto à tendência por grupo etário, as maiores reduções nas taxas de mortalidade por DCV por 100 mil, entre 1990 e 2017, foram observadas no grupo ‘abaixo de 5’ [-60,9 (-70,7 a -48,9)], seguido pelo grupo etário de 50-69 anos [-50,6 (-52,7 a -48,4)].

•A cobertura do PSF foi associada a redução nas hospitalizações e na mortalidade por DCV que foram incluídas na Lista de Condições Sensíveis à Atenção Primária no Brasil, tendo seu efeito aumentado de acordo com a duração da implementação do PSF no município. Rasella *et al* . relataram reduções nas mortalidades por doença cerebrovascular e doença cardíaca de 0,82 (IC 95%: 0,79-0,86) e 0,79 (IC 95%: 0,75-0,80), respectivamente, chegando a 0,69 (IC 95%: 0,66-0,73) e 0,64 (IC 95%: 0.59-0.68), respectivamente, quando a cobertura do PSF foi consolidada no total dos 8 anos estudados. ^35^

•De acordo com o banco de dados do SIM, em 2019, as DCV corresponderam a 27,0% do total de mortes, com a maior proporção na região Sudeste e a menor na região Norte. A DIC foi responsável por 32,3% do total de mortes por DCV no Brasil e o AVC, por 27,8%. A maior proporção de mortalidade por DIC ocorreu no Mato Grosso do Sul, Pernambuco e São Paulo, enquanto a maior proporção de mortes por AVC ocorreu no Amazonas, Amapá e Distrito Federal ( Tabela 1-4 ).

•A proporção de mortes por DCV diminuiu entre homens (de 30,1% para 27,6%) e mulheres (31,1% para 29,9%) de 2000-2002 a 2015-2017. Além disso, Lotufo notou um constante excesso de mortes prematuras por DCV entre os homens naquele período, com uma razão homem/mulher de 2:1. ^36^

•Há significativa correlação do SDI, uma estimativa do nível socioeconômico, com a carga de DCV. A Figura 1-12 mostra a correlação entre a maior redução na variação percentual das taxas de mortalidade por DCV padronizadas por idade, entre 1990 e 2019, e o maior SDI de 2019, sugerindo que a diminuição da mortalidade por DCV seguiu-se a uma melhora nas condições socioeconômicas locais, como observado em outros estudos. ^5,37–39^

•Lotufo *et al.* compararam 3 diferentes níveis de renda domiciliar (alto, médio e baixo) com taxas de mortalidade por DCV, na cidade de São Paulo, de 1996 a 2010. As variações percentuais anuais e os IC 95% para homens residentes em áreas de renda alta, média e baixa foram -4,1 (-4,5 a -3,8), -3,0 (-3,5 a -2,6) e -2,5 (-2,8 a -2,1), respectivamente. As tendências para as taxas de mulheres residentes em áreas de renda alta foram -4,4 (-4,8 a -3,9) em 1996-2005 e -2,6 (-3,8 a -1,4) em 2005-2010. A redução nas mortes por DCV foi mais significativa para homens e mulheres residentes em áreas mais abastadas, com um gradiente decrescente para risco de morte maior para os residentes de áreas mais abastadas em comparação àqueles de áreas mais carentes. ^40^

•Observou-se associação inversa do IDHm e da cobertura de saúde suplementar com a mortalidade por DCV, sugerindo uma relação entre fatores socioeconômicos e DCV. ^37^ O IDHm aumentou entre 2000 e 2010 em todas as UF, sendo 0,7 ou maior na metade das UF. A cobertura de saúde suplementar aumentou no país durante o período estudado e associou-se inversamente com mortalidade por DCV entre 2004 e 2013. ^37^

•Soares *et al.* observaram uma diminuição na mortalidade por DCV nos estados do Rio de Janeiro, São Paulo e Rio Grande do Sul que precedeu a melhoria no índice socioeconômico. A evolução do PIB per capita, o declínio da mortalidade infantil, o maior nível educacional (representado pela escolaridade, em anos, dos indivíduos com idade superior a 25 anos) e o IDHm mostraram uma grande correlação com a redução na taxa de mortalidade por DCV. A redução nas taxas de mortalidade por DCV, AVC e DIC no estado do Rio de Janeiro nas últimas décadas foi precedida por um aumento no IDH. Um acréscimo de 0,1 no IDH correlacionou-se com as seguintes reduções no número de mortes por 100 mil habitantes: 53,5 por DCV; 30,2 por AVC; e 10,0 por DIC. ^38^ ^,^ ^39^

•Baptista *et al* . investigaram a relação entre a taxa de mortalidade por DCV e o desenvolvimento econômico no tempo e no espaço, medido pelo PIB per capita, nas microrregiões brasileiras, de 2001 a 2015. Os autores, usando as bases de dados SIM-DATASUS e SIDRA do IBGE, observaram um rápido declínio na mortalidade por DCV nas regiões Sul e Sudeste, assim como um declínio mais lento na região Centro-Oeste. Por outro lado, as regiões Norte e Nordeste apresentaram um aumento nas taxas de mortalidade por DCV ao longo do tempo, talvez em decorrência do menor acesso aos cuidados em saúde e dos fatores socioeconômicos. ^41^

•Silveira *et al.* , estudando o efeito da temperatura ambiente na mortalidade cardiovascular em 27 cidades brasileiras, observaram maior número de mortes cardiovasculares associado com temperaturas baixas e altas na maioria das cidades brasileiras e nas regiões Centro-Oeste, Norte, Sul e Sudeste. O RR geral para o Brasil foi 1,26 (IC 95%, 1,17–1,35) para o percentil 1 de temperatura e 1,07 (IC 95%, 1,01–1,13) para o percentil 99 de temperatura em comparação ao percentil 79 (27,7 °C), cujo RR foi o menor. ^42^

### Carga de Doença

•As taxas de DALYs padronizadas por idade no Brasil foram 6.907 (II 95%, 6.783-7.039) por 100 mil habitantes em 1990, caindo para 3.735 (II 95%, 3.621-3.849) por 100 mil habitantes em 2019. As taxas de DALYs caíram nas cinco regiões, com padrões diferentes, mais rapidamente no Sul e Sudeste e mais lentamente no Nordeste ( Figura 1-13 ). Houve uma correlação entre a redução percentual nas taxas de DALYs e o aumento no SDI: quanto maior o SDI, maior o declínio nos DALYs por DCV. Distrito Federal, Rio de Janeiro e Santa Catarina apresentaram o maior SDI e um grande declínio nas taxas de DALYs, enquanto Alagoas, Piauí e Ceará apresentaram um pequeno declínio nas taxas de DALYs e baixo SDI ( Figura 1-14 e Tabela 1-5 ).

•Com relação a YLLs, 8.130.233 anos de vida foram perdidos em 2019 devido a mortalidade por DCV. Esse número foi maior entre os indivíduos de 50-69 anos em comparação aos outros grupos etários. As taxas de YLLs caíram desde 1990 para todos os grupos etários ( Tabela 1-6 ). As taxas de YLLs padronizadas por idade diminuíram 51,5% (II 95%, -53,4 a -49,7) de 1990 a 2019 ( Tabela 1-6 ).

•A incapacidade causada por DCV não diminuiu como observado para mortalidade. As taxas de YLDs padronizadas por idade diminuíram 15% de 1990 a 2019 ( Tabela 1-7 ).

O grupo etário ‘50-69 anos’ apresentou o maior número de YLDs, sendo seguido de perto pelo grupo de ‘15-49 anos’. Todos os grupos etários apresentaram uma pequena redução nas taxas padronizadas por idade, tendo o grupo de ‘15-49 anos’ apresentado a menor (-0,7%) ( Tabela 1-7 ).

### Utilização e Custo da Atenção à Saúde

•No Brasil, de 2008 a 2019, os principais grupos de procedimentos cardiovasculares clínicos e cirúrgicos perfizeram 8.743.403 procedimentos pagos pelo SUS. Desses, 7.462.563 foram clínicos, devidos principalmente a insuficiência cardíaca, que representou 41,3% (3.085.359) das admissões, seguida pelas doenças cerebrovasculares, 30,2% (2.253.344), síndrome coronariana aguda, 11,5% (855.125) e IAM com abordagem clínica, 10,1% (757.081) (Tabelas 1-8 e 1-9 ).

•As hospitalizações por condições clínicas de DCV diminuíram em 13.289 de 2008 a 2019 ( Tabela 1-8 ), embora os números absolutos tenham permanecido estáveis ao longo dos anos. Em 2008, cada admissão clínica custou, em média, R$890 e, em 2019, R$1.488, um aumento de 67% ( Tabela 1-10 ).

•Dos 1.280.840 procedimentos cardiovasculares cirúrgicos realizados de 2008 a 2019, 755.411 (58,9%) foram angioplastias coronarianas, seguidas por 265.123 (20,1%) CRVM e 151.902 (11,9%) cirurgias valvares. A relação angioplastia/CRVM em 2008 foi 1,8, tendo aumentado em 2019 para 4,1.

•As hospitalizações para procedimentos cirúrgicos por DCV de 2008 a 2019 aumentaram 64% ( Tabela 1-9 ).

Em média, cada procedimento cirúrgico foi reembolsado em R$7.036 em 2008, mostrando um aumento não ajustado de 18% em 2019 em comparação a 2008, ao custo de R$8.319 por procedimento ( Tabela 1-12 ).

•Nos últimos 12 anos, no Brasil, houve significativa redução nas hospitalizações por insuficiência cardíaca e aumento nas hospitalizações anuais por IAM e doenças cerebrovasculares, enquanto os outros grupos de procedimentos clínicos tenderam à estabilidade ( Figura 1-15 ). Quanto às abordagens cirúrgicas nos mesmos anos, houve grande aumento no número anual de angioplastias coronarianas e tendência à estabilidade no número dos outros procedimentos cirúrgicos ( Figura 1-16 ).

•As Tabelas 1-10 e 1-11 mostram os valores em Reais e Dólares Internacionais de 2019 (Int$2019), respectivamente, pagos pelo sistema público de saúde para admissões clínicas cardiovasculares no Brasil de 2008 a 2019.

O total gasto nessas hospitalizações foi de R$ 9.378.278, correspondendo a Int$ 6.170.381 em 2019. Insuficiência cardíaca, doenças cerebrovasculares e síndromes coronarianas foram responsáveis pela maior parte desses valores.

•Os valores pagos por procedimentos cirúrgicos devidos a DCV são apresentados em Reais e em Int$2019 nas Tabelas 1-12 e 1-13 , respectivamente. Embora em menor número do que os procedimentos clínicos, são responsáveis pelas maiores despesas, com R$ 10.524.044 gastos, equivalendo a Int$2019 6.853.635. Os procedimentos usados para o tratamento de DIC, incluindo angioplastia coronariana e CRVM, representaram a maior parte dessas despesas.

### Pesquisa Futura

•O SIM, implementado em 1975, é uma ferramenta essencial para monitorar as estatísticas de mortalidade no Brasil, pois o registro de todas as mortes é obrigatório nas UF, sendo que, em 2017, a cobertura do território nacional foi de 98%, menor na região Norte do que na Sul. A região Nordeste apresenta a menor cobertura, ainda inferior a 95%. ^43^ Embora o SIM tenha melhorado através de projetos específicos do Ministério da Saúde, ^44^ ^,^ ^45^ ainda persistem problemas, como as causas mal definidas (cerca de 6%), ‘códigos *garbage* ’ e subnotificação de mortes, que geram vieses que podem comprometer a métrica apresentada. Portanto, pesquisa adicional é necessária para promover ajustes metodológicos para cobertura e redistribuição de causas mal definidas, especialmente nos primeiros anos da série histórica. Por outro lado, as estimativas do Estudo GBD requerem mais pesquisa para a implementação de modelos com melhor distribuição de ‘códigos *garbage* ’ adaptados às realidades locais.

•Vale mencionar que, devido à falta de dados de incidência primária (coortes) no Brasil, há necessidade de pesquisa que permita compreender como enfrentar a DCV nos estados e nas populações com baixos índices socioeconômicos.

•Devido à redução da tendência de declínio da mortalidade por DCV padronizada por idade nos últimos 5 anos, novas estratégias de combate a essa mortalidade devem ser estudadas. É fundamental que se compreendam os motivos para tal redução para que se implementem políticas efetivas, em particular ante o envelhecimento da população, que vai aumentar o número de indivíduos com DCV no país.

## 2. ACIDENTE VASCULAR CEREBRAL (DOENÇAS CEREBROVASCULARES)

### CID-9 430 a 438; CID-10 I60 a I69

**Ver Tabelas 2-1 a 2-12 e Figuras 2-1 a 2-3**

**Table:** Abreviaturas Usadas no Capítulo 2

AIT	Ataque Isquêmico Transitório
AVC	Acidente Vascular Cerebral
AVCH	Acidente Vascular Cerebral Hemorrágico
AVCI	Acidente Vascular Cerebral Isquêmico
CID	Classificação Estatística Internacional de Doenças e Problemas Relacionados à Saúde
CID-9	Classificação Estatística Internacional de Doenças e Problemas Relacionados à Saúde, 9 ^a^ Revisão
CID-10	Classificação Estatística Internacional de Doenças e Problemas Relacionados à Saúde, 10 ^a^ Revisão
DAC	Doença Arterial Coronariana
DALYs	Anos de vida perdidos ajustados por incapacidade (do inglês, *Disability-Adjusted Life-Year* )
GBD	Global Burden of Disease
HIC	Hemorragia Intracerebral
HSA	Hemorragia Subaracnóidea
IC	Intervalo de Confiança
IECA/BRA	Inibidor da Enzima de Conversão da Angiotensina/ Bloqueador do Receptor de Angiotensina
II	Intervalo de Incerteza
IMPACT-AF	Estudo *Improve* *Treatment with Anticoagulants in Patients with Atrial Fibrillation*
INR	Índice Internacional Normalizado (do inglês, *International Normalized Ratio* )
IRR	Razão da taxa de incidência (do inglês, *Incidence Rate Ratio* )
MAPS	Matão Preventing Stroke Study
MELAS	Encefalomiopatia mitocondrial, acidose lática e episódios *stroke-like*
NOAC	Anticoagulante Oral Não Antagonista da Vitamina K
OMS	Organização Mundial da Saúde
OR	Odds Ratio
PNS	Pesquisa Nacional de Saúde
PSF	Programa Saúde da Família
PURE	Estudo *Prospective Urban Rural Epidemiological Study*
RR	Risco Relativo
SDI	Índice Sociodemográfico (do inglês, *Sociodemographic Inde* *x* )
SIM	Sistema de Informações sobre Mortalidade
SSQOL	Escala Específica de Qualidade de Vida no AVC (em inglês, *Stroke Specific Quality of Life Scale* )
YLDs	Anos vividos com incapacidade (do inglês, *Years Lived with Disability* )
YLLs	Anos potenciais de vida perdidos (do inglês, *Years of Life Lost* )

**Tabela 2-1:** – Número de casos, taxas de prevalência padronizadas por idade (por 100 mil) de AVC isquêmico, hemorragia subaracnóidea e hemorragia intracerebral em 1990 e 2019, e variação percentual das taxas, no Brasil e suas unidades federativas.

Causa de morte e localização	1990		2019		Variação percentual (II 95%)
	Número (II 95%)	Taxa (II 95%)	Número (II 95%)	Taxa (II 95%)	
**B.2.3.1-AVC isquêmico**					
Acre	2337 (2005;2670,8)	1179,3 (1021,6;1368)	5959,2 (5165,9;6781,1)	876,1 (762,2;998)	-25,7(-30,7;-20,7)
Alagoas	20840,6 (17963,5;24003,8)	1415,7 (1222,3;1668,6)	33233,7 (29180,3;38341,4)	1016,1 (895,6;1175,5)	-28,2(-33,5;-22,9)
Amapá	1384 (1196,3;1573,2)	1124,1 (970,4;1298,2)	5077,4 (4424,8;5767,2)	861,2 (746,8;984,5)	-23,4(-28,6;-18)
Amazonas	11728,4 (10152,6;13440,3)	1240 (1069,8;1424,9)	26360,1 (23112,3;29848,5)	832,7 (729,8;950,6)	-32,8(-37,7;-27,4)
Bahia	92983,6 (80433,9;106701,5)	1252,7 (1072,5;1448,2)	140578,2 (122673,2;160762,3)	870,8 (760,2;1001,2)	-30,5(-35,3;-25,6)
Brasil	1287969,4 (1118323,7;1460716)	1327,6 (1151,2;1516)	2040376,9 (1784219,6;2330526)	870,1 (761,1;992,8)	-34,5(-36,7;-32,2)
Ceará	45261 (38660,3;51639)	1031,5 (880,1;1192,4)	85094,6 (74109,4;97715,8)	851,1 (740,3;977,2)	-17,5(-23;-11,4)
Distrito Federal	9491,3 (8108,7;10933,6)	1301,3 (1110,6;1516,4)	24286,9 (20992,9;28153,4)	885,1 (765,4;1030,9)	-32(-36,7;-26,9)
Espírito Santo	22957,7 (19897,4;26104,4)	1423 (1222,2;1642,3)	39703,5 (34433,2;45287,7)	913,4 (795,3;1041,4)	-35,8(-40,2;-31,2)
Goiás	30398,4 (26103,6;34903,4)	1266,7 (1089,5;1464)	57813,2 (50438,7;65757,5)	813,4 (711,9;925,3)	-35,8(-40,6;-31,2)
Maranhão	33415,7 (28606,1;38354,7)	1174,6 (998,4;1372,4)	57772,9 (50605,5;65580,5)	839,2 (735,7;956,6)	-28,6(-35;-20,2)
Mato Grosso	11695,9 (10093,3;13434,1)	1207,4 (1043,7;1397)	28745,8 (25006,5;32932)	838 (727,9;961,7)	-30,6(-36;-25)
Mato Grosso do Sul	12959,8 (11279,9;14803,9)	1255,5 (1093,2;1450,7)	25831,6 (22483,5;29207,6)	870,8 (758,4;984,9)	-30,6(-36,5;-25,4)
Minas Gerais	145398,6 (125793,7;168354,4)	1356,8 (1168,7;1571,8)	217642,4 (189023,2;251547)	843,5 (732,9;971,6)	-37,8(-42,8;-32,7)
Pará	32096,8 (27597,1;36767,6)	1325,8 (1132;1527,9)	64670,6 (56252,5;73679,5)	869 (753,8;991,8)	-34,5(-39,3;-29,4)
Paraíba	26607,1 (22881,2;30854,6)	1096,5 (940,9;1284,1)	37478 (32824,1;42797,6)	804,4 (704;921,6)	-26,6(-32;-21,3)
Paraná	81476,8 (70660,4;93825,4)	1534,4 (1322,5;1786,7)	126272,5 (109037,2;145179,1)	960,2 (830,4;1100)	-37,4(-42;-32,1)
Pernambuco	63426,2 (54597,3;73733,5)	1300,5 (1112,3;1536,8)	89359,9 (77947,2;102237,8)	884,3 (769,8;1015)	-32(-36,4;-26,9)
Piaui	17859,3 (15361,8;20573,6)	1143,9 (986,6;1328,8)	31595 (27482,8;36104)	843,1 (733,5;963)	-26,3(-31,3;-20,4)
Rio de Janeiro	151283,8 (130994,6;173336,9)	1489 (1283,3;1706,2)	197147,1 (170574,1;228003,1)	902,7 (783;1039,9)	-39,4(-44,1;-34,7)
Rio Grande do Norte	18111,6 (15616,7;20695,6)	1056,5 (908,6;1217,1)	29607,2 (25837,3;34049,1)	766,9 (670,6;883,4)	-27,4(-31,6;-22,4)
Rio Grande do Sul	98174.7 (84791.3;112264.8)	1410.7 (1218.8;1619.6)	138340.7 (118660.6;159686.5)	926.6 (799.2;1061.2)	-34.3(-39.1;-29.5)
Rondônia	6438.9 (5505.5;7461.4)	1477.7 (1270.8;1735.5)	13891.4 (11998.5;15994.2)	861.5 (741.1;998.2)	-41.7(-46.2;-36.8)
Roraima	980.8 (844.6;1128.1)	1199.9 (1033.2;1385.3)	3514.8 (3044.9;4034.3)	830.3 (719.9;952.8)	-30.8(-35.5;-25.7)
Santa Catarina	38488 (33075.1;43771.1)	1392.8 (1183.7;1603)	68595.6 (59198.3;78591.2)	848.8 (733.7;970.5)	-39.1(-43.9;-34.1)
São Paulo	295180.2 (255885.5;336598.3)	1344.1 (1162.1;1524.7)	458484.1 (395229.9;529532.9)	857 (741.3;982.9)	-36.2(-40.8;-31.8)
Sergipe	11546.5 (9966.9;13183)	1306.7 (1118.5;1507.3)	21060.5 (18405.7;24150.7)	920.1 (799.5;1060.9)	-29.6(-34.9;-25)
Tocantins	5446.7 (4686.9;6267.8)	1148 (974.7;1327.9)	12260 (10740.9;14034.4)	827.6 (721.1;948.7)	-27.9(-32.9;-21.9)
**B.2.3.2-Hemorragia intracerebral**					(;)
Acre	954.6 (816;1099.4)	423.3 (363.8;486.7)	2357.5 (2036.2;2708.6)	312.6 (271.5;357.9)	-26.1(-30.5;-21.2)
Alagoas	7759.2 (6563.3;8947.2)	487.7 (412.6;564.5)	12212.5 (10587.5;14019.8)	352.5 (305.3;404.1)	-27.7(-32.4;-23)
Amapá	580.2 (496.8;667.5)	412.4 (356.1;474.8)	2046.9 (1773;2347.9)	303.5 (264;346.5)	-26.4(-30.5;-21.6)
Amazonas	5190.3 (4456.4;5996.5)	481.6 (414.4;554.5)	10793.9 (9317.7;12412.6)	309.3 (268;353.8)	-35.8(-39.8;-31.4)
Bahia	37762.4 (32554.4;43452.4)	480.3 (409.2;551.3)	53634.2 (46593.5;61495.6)	323.4 (281.1;370.3)	-32.7(-37.5;-28)
Brasil	541445.3 (466619.8;621909.3)	507.5 (438.9;584.1)	757903 (659245.3;867100.5)	315.9 (275;361.4)	-37.7(-40.5;-34.9)
Ceará	17834.5 (15383.5;20700.6)	390.6 (335.7;452.2)	31985.9 (27865.3;36677)	313.1 (273.4;358.8)	-19.8(-24;-14.5)
Distrito Federal	4754.5 (4045.6;5533.3)	521.2 (448.3;599.5)	9476.3 (8096.4;10959.5)	312.1 (270.2;359)	-40.1(-44.5;-35.6)
Espírito Santo	9923.8 (8526.1;11356.7)	553.1 (476.3;631.1)	15127.1 (13005.6;17294.3)	336.8 (291;385.1)	-39.1(-43.1;-34.9)
Goiás	14065.7 (12162.2;16282.3)	513.3 (443.4;589.5)	22525 (19579.7;25806.5)	301 (261.7;345)	-41.4(-45.5;-36.8)
Maranhão	12434.4 (10659.9;14397.9)	406.8 (349.9;471.3)	22610.6 (19506.8;26011.2)	315.1 (271.1;361.5)	-22.6(-27.6;-17.4)
Mato Grosso	5048.2 (4327.5;5880.2)	432.5 (371.8;500.3)	10830.6 (9392.7;12448)	292.1 (254.7;333.1)	-32.5(-36.9;-28.1)
Mato Grosso do Sul	6065.5 (5157.5;7014.4)	515.4 (441.4;596.7)	10049.7 (8640.4;11486.9)	325.5 (281.2;371.4)	-36.8(-41.2;-32.6)
Minas Gerais	63178.6 (54522.8;72902.3)	536.4 (464.4;616.6)	80317.8 (69705.8;91963.5)	310.7 (269.7;355.8)	-42.1(-46.7;-37.9)
Pará	13066 (11192.2;15083.4)	477.6 (408;553.7)	24092.9 (20923.5;27440.9)	296.6 (256.5;336.5)	-37.9(-41.9;-33.6)
Paraíba	9857.1 (8470.4;11300.2)	404 (345;465.3)	15120.2 (13151.5;17216.3)	324.7 (282.5;369.1)	-19.6(-25;-13.7)
Paraná	33661.9 (28718.5;38871.7)	555.3 (471;641.8)	42595 (36576.4;48876.7)	317.7 (273.5;363.3)	-42.8(-47.4;-38)
Pernambuco	26388.4 (22669.9;30514.5)	512.8 (442.7;592.5)	35843.8 (31160.7;41052.3)	342.4 (298.8;390.8)	-33.2(-37.5;-28.7)
Piaui	7154.6 (6136.8;8240.6)	424.7 (362.6;489.1)	11367.7 (9877.1;13067.2)	300.8 (261.7;345.3)	-29.2(-33.2;-24.6)
Rio de Janeiro	67411.9 (57661.1;77654.6)	603.2 (517.9;694.9)	78112.1 (67686.5;89538.8)	357.8 (310.3;408.4)	-40.7(-45.2;-35.4)
Rio Grande do Norte	6989.6 (6011.1;7956.2)	398 (342;455.7)	11368.6 (9897.4;13069.4)	288.4 (250.7;331.2)	-27.5(-31.9;-22.8)
Rio Grande do Sul	40477.3 (34799.8;46418.5)	528.1 (457.3;604.3)	48534.9 (41907.9;55770.7)	331.4 (286.5;380)	-37.3(-41.4;-32.7)
Rondônia	2937.5 (2502.1;3408.2)	508.6 (437;589.8)	5212 (4528.8;5996.9)	295.6 (257.4;339.4)	-41.9(-46;-37.5)
Roraima	451.4 (384.2;525.4)	437.1 (375.7;504.9)	1327.3 (1137.7;1541.9)	273.8 (236.1;316.6)	-37.4(-41.3;-32.9)
Santa Catarina	17321.8 (14777.5;19977.4)	546.2 (467.7;637.7)	25189.2 (21733.7;28815.9)	301.5 (260.5;343.6)	-44.8(-49;-39.9)
São Paulo	123269.3 (105277.2;143037.4)	503.6 (431.8;583)	162501.2 (139844.8;186907.7)	299.1 (258.2;342.1)	-40.6(-45.1;-35.6)
Sergipe	4551.5 (3900.5;5230.3)	481.6 (416.8;554.1)	7971.1 (6945.4;9114.2)	330.2 (288.3;376.9)	-31.5(-36;-26.7)
Tocantins	2355.2 (2010.9;2701.8)	431 (373.5;496.1)	4699.2 (4080.2;5401.4)	300.2 (260.2;345.9)	-30.4(-34.9;-25.1)
**B.2.3.3-Hemorragia subaracnóidea**					(;)
Acre	361.4 (300.3;435.9)	152.2 (126;183.9)	990.2 (823.1;1197.9)	124.4 (104;150.2)	-18.3(-22.6;-13.9)
Alagoas	2547.4 (2107.3;3083.5)	154.5 (127.6;187.9)	4406.8 (3654.6;5267.4)	123.5 (102.4;147.2)	-20.1(-24.5;-15.4)
Amapá	232.1 (191.6;278.8)	155 (127.3;188.1)	902.6 (751;1079.4)	125.5 (104.2;151.7)	-19(-23.5;-14.9)
Amazonas	1787.5 (1480.7;2176.3)	150.7 (123.5;183.8)	4505.5 (3742.5;5421.6)	121.9 (101.6;147.5)	-19.1(-23.2;-14.9)
Bahia	12581 (10473.6;15135.6)	156.6 (129.2;189.2)	21268.5 (17762.4;25776.8)	125.7 (104.9;152.1)	-19.8(-24.2;-15.7)
Brasil	178322.3 (147412.5;215453.5)	158.6 (131.7;192.4)	306334.8 (255287.2;369754.8)	124.8 (104.2;150.1)	-21.3(-24.3;-18.6)
Ceará	7235.2 (5975.9;8843.8)	157.6 (130.3;193.1)	13166.1 (10906.1;15881)	126.6 (104.8;152.9)	-19.7(-24.1;-15.1)
Distrito Federal	1789.8 (1478.1;2170.4)	165.8 (137.2;200.5)	4274.5 (3539.2;5205.6)	127.6 (106.3;155)	-23(-28;-17.1)
Espírito Santo	3071.7 (2545.7;3713.8)	160.5 (131.9;194.5)	5799.8 (4800.4;7005.5)	125.4 (104.1;150.5)	-21.8(-26.2;-17.1)
Goiás	4544.9 (3780.9;5488.7)	152.2 (126;184.1)	9354.7 (7742.9;11331.2)	119.6 (99.4;144.5)	-21.5(-25.8;-16.7)
Maranhão	4907.9 (4058;5876.5)	157.5 (130.2;189.6)	9393.7 (7853.4;11298.1)	129.3 (107.2;156.1)	-17.9(-22.4;-13.8)
Mato Grosso	1932.7 (1599.2;2343.7)	149.6 (123.5;182.8)	4740.3 (3954.5;5734.8)	120.7 (101.2;145.4)	-19.3(-23.6;-14.3)
Mato Grosso do Sul	1977.1 (1624.8;2389.4)	154.9 (127.5;189)	3968.7 (3292.1;4814.3)	124 (103.4;150)	-19.9(-24.3;-15.2)
Minas Gerais	20106.5 (16619.3;24414.9)	163.5 (134.5;198.3)	32921.5 (27309.6;39771.4)	125.7 (104.6;151.1)	-23.1(-28.3;-18.2)
Pará	4512.4 (3724.9;5437.7)	152.3 (125.7;184.8)	10338 (8575.1;12465.8)	122.3 (101.6;147.1)	-19.7(-24;-15.4)
Paraíba	3901.8 (3225.5;4735.4)	163.9 (135.7;200.2)	6045.8 (4997.5;7325.1)	128.7 (106.4;156.2)	-21.5(-25.9;-17.1)
Paraná	10404.5 (8631.1;12629.7)	158 (130.9;191.8)	17179.4 (14139.7;20873.3)	124.7 (103.1;150.9)	-21.1(-25.6;-16.1)
Pernambuco	7989.4 (6624.6;9835.5)	151.4 (124.4;187.4)	13170.6 (10925.4;15923.5)	122.4 (102.2;147.9)	-19.1(-24.1;-14.6)
Piaui	2775.1 (2286;3362.5)	161 (132.6;196.5)	4913.8 (4082.1;5920.7)	129.1 (107.3;155.4)	-19.9(-23.9;-15.1)
Rio de Janeiro	18926.8 (15641.4;22940.3)	161.2 (133.5;195)	27140.4 (22430.8;33138.9)	123.4 (102.3;149.7)	-23.5(-27.8;-18.4)
Rio Grande do Norte	2710.1 (2246.3;3278)	156.1 (129;189.2)	5013.6 (4186.9;6053.4)	124.6 (103.6;150.3)	-20.2(-24.5;-16.2)
Rio Grande do Sul	12748.3 (10540.8;15532.4)	157.7 (130.5;192.4)	18004.8 (14901.9;21850.4)	123.5 (102.9;148.8)	-21.7(-25.9;-17.1)
Rondônia	991.8 (815.5;1209.9)	146 (120.3;177.6)	2239.9 (1839.2;2710.2)	119.4 (98.9;144.4)	-18.3(-22.8;-13.7)
Roraima	168.9 (139.2;204.8)	142.1 (117.1;172.1)	602.9 (497.8;734.5)	115.7 (96.1;140.5)	-18.5(-22.8;-14.3)
Santa Catarina	5514.6 (4565.3;6634.1)	159.2 (131.1;192.1)	10720.7 (8888.1;13027.3)	123.3 (102.8;149)	-22.6(-27.3;-17.5)
São Paulo	42197.3 (34696.2;51220.6)	160.4 (132.4;194.9)	70133.3 (58163.2;84885.3)	126.1 (105.1;151.9)	-21.4(-25.5;-16.9)
Sergipe	1498.7 (1239.1;1808.9)	154 (127.2;186.4)	3089.5 (2565.1;3754.7)	123.2 (102.8;149.3)	-20(-24.3;-15.6)
Tocantins	907.2 (754.4;1098.1)	154.9 (128.2;187.3)	2048.9 (1698.5;2471.5)	126.2 (105.1;151.9)	-18.5(-22.7;-14.1)

*Fonte: Dados derivados do estudo Global Burden of Disease 2019, Institute for Health Metrics and Evaluation, University of Washington. ^46^*

**Tabela 2-2:** – Número de casos e taxas de prevalência padronizadas por idade (por 100 mil) de AVC, AVC isquêmico, hemorragia subaracnóidea e hemorragia intracerebral em 1990 e 2019, com variação percentual das taxas, no Brasil, de acordo com o grupo etário.

Causa de morte e grupo etário	1990		2019		Variação percentual (II 95%)
	Número (II 95%)	Taxa (II 95%)	Número (II 95%)	Taxa (II 95%)	
**B.2.3-AVC**					
Abaixo de 5	4182,3 (2610,7;6524,5)	24,7 (15,4;38,5)	3235,1 (2120,2;4898,9)	20,9 (13,7;31,6)	-15,4 (-33;9,6)
15-49 anos	623994,4 (545014,4;705854,8)	814,1 (711,1;920,9)	786738,6 (690686,9;888810,4)	681,2 (598,1;769,6)	-16,3 (-19;-13,4)
50-49 anos	825075,6 (723316,1;937034,1)	5259,4 (4610,7;5973,1)	1296990 (1151199,7;1476134)	3214,9 (2853,5;3658,9)	-38,9 (-41;-36,8)
5-14 anos	48928 (35610,6;66858,5)	138,5 (100,8;189,2)	40623,7 (30515;54156,4)	126 (94,6;167,9)	-9 (-16,7;-0,1)
70+ anos	410923,1 (347995,9;484341)	9714,3 (8226,7;11449,9)	857424,4 (735950,3;1005011,8)	6551 (5622,9;7678,6)	-32,6 (-35,4;-29,7)
Padronizada por idade	1913103,4 (1735455;2095724,2)	1909,3 (1733,1;2100,1)	2985011,7 (2716616,7;3280843,8)	1256,6 (1142,6;1381,1)	-34,2 (-35,8;-32,5)
Toda as idades	1913103,4 (1735455;2095724,2)	1285,4 (1166;1408,1)	2985011,7 (2716616,7;3280843,8)	1377,7 (1253,8;1514,2)	7,2 (4,4;10,4)
**B.2.3.1-AVC isquêmico**					
Abaixo de 5	6688,4 (4586,3;9999,2)	39,5 (27,1;59)	5640,4 (3913,2;8255,7)	36,4 (25,3;53,3)	-7,8 (-13,1;0,7)
15-49 anos	343498,3 (275888,5;421506,2)	448,2 (360;549,9)	451442,9 (365495;550221,4)	390,9 (316,5;476,4)	-12,8 (-16,3;-9,1)
50-49 anos	539690,6 (446267,3;646791)	3440,2 (2844,7;4122,9)	839214,7 (703174,9;1002009,6)	2080,2 (1743;2483,7)	-39,5 (-42,6;-36,7)
5-14 anos	46198,9 (31663;66393)	130,8 (89,6;187,9)	38107,7 (26846,3;53603,5)	118,2 (83,2;166,2)	-9,6 (-13,9;-4,3)
70+ anos	351893,2 (283271,1;428872)	8318,8 (6696,6;10138,6)	705971,2 (577001,6;861003,9)	5393,8 (4408,5;6578,3)	-35,2 (-38,3;-32)
Padronizada por idade	1287969,4 (1118323,7;1460716)	1327,6 (1151,2;1516)	2040376,9 (1784219,6;2330526)	870,1 (761,1;992,8)	-34,5 (-36,7;-32,2)
Toda as idades	1287969,4 (1118323,7;1460716)	865,4 (751,4;981,4)	2040376,9 (1784219,6;2330526)	941,7 (823,5;1075,6)	8,8 (5;12,8)
**B.2.3.2-Hemorragia intracerebral**					
Abaixo de 5	1161,2 (784,1;1623,7)	6,9 (4,6;9,6)	1147 (789,8;1578,7)	7,4 (5,1;10,2)	8 (1,9;15,4)
15-49 anos	218246,5 (176752,2;264569,5)	284,7 (230,6;345,2)	253649,5 (207356,6;301299,5)	219,6 (179,5;260,9)	-22,9 (-26,2;-19,1)
50-49 anos	240417,8 (196762,5;288416,9)	1532,5 (1254,3;1838,5)	341586,5 (285413,3;400854,7)	846,7 (707,5;993,6)	-44,8 (-47,4;-41,8)
5-14 anos	13416,1 (9141,6;18251,2)	38 (25,9;51,7)	12613,2 (8880,5;17085,2)	39,1 (27,5;53)	3 (-3;10,7)
70+ anos	68203,7 (55601,8;84221,2)	1612,4 (1314,4;1991)	148906,8 (121630,5;183577)	1137,7 (929,3;1402,6)	-29,4 (-34;-23,6)
Padronizada por idade	541445,3 (466619,8;621909,3)	507,5 (438,9;584,1)	757903 (659245,3;867100,5)	315,9 (275;361,4)	-37,7 (-40,5;-34,9)
Toda as idades	541445,3 (466619,8;621909,3)	363,8 (313,5;417,9)	757903 (659245,3;867100,5)	349,8 (304,3;400,2)	-3,8 (-8;0,3)
**B.2.3.3-Hemorragia subaracnóidea**					
Abaixo de 5	253,7 (139,8;383,7)	1,5 (0,8;2,3)	245,7 (137,5;369)	1,6 (0,9;2,4)	5,9 (2,7;10,8)
15-49 anos	92830,8 (74524,7;114996,4)	121,1 (97,2;150)	124691,1 (100254,7;154948,8)	108 (86,8;134,2)	-10,9 (-13,5;-8,4)
50-49 anos	71954,7 (56126,6;92135,2)	458,7 (357,8;587,3)	145941,5 (113631,8;186025,6)	361,7 (281,7;461,1)	-21,1 (-24,4;-17,8)
5-14 anos	2881,6 (1811,9;4260,4)	8,2 (5,1;12,1)	2715,1 (1735,5;4024,5)	8,4 (5,4;12,5)	3,2 (0,4;7,1)
70+ anos	10401,5 (7385,5;13977,2)	245,9 (174,6;330,4)	32741,3 (24045,6;42870,8)	250,2 (183,7;327,5)	1,7 (-3,9;8,6)
Padronizada por idade	178322,3 (147412,5;215453,5)	158,6 (131,7;192,4)	306334,8 (255287,2;369754,8)	124,8 (104,2;150,1)	-21,3 (-24,3;-18,6)
Toda as idades	178322,3 (147412,5;215453,5)	119,8 (99;144,8)	306334,8 (255287,2;369754,8)	141,4 (117,8;170,7)	18 (12,8;23,4)

*Fonte: Dados derivados do estudo Global Burden of Disease 2019, Institute for Health Metrics and Evaluation, University of Washington. ^*46*^*

**Tabela 2-3:** – Número de casos e taxas de incidência padronizadas por idade (por 100 mil) de AVC, AVC isquêmico, hemorragia subaracnóidea e hemorragia intracerebral em 1990 e 2019, com variação percentual das taxas, no Brasil e suas unidades federativas.

Causa de morte e localização	1990		2019		Variação percentual (II 95%)
	Número (II 95%)	Taxa (II 95%)	Número (II 95%)	Taxa (II 95%)	
**B.2.3-AVC**					(;)
Acre	411,9 (369,4;460,7)	210,4 (188,3;236,4)	887,5 (791,6;996,9)	133,5 (118,8;151,1)	-36,5(-38,7;-34)
Alagoas	3525,9 (3156,5;3980,9)	241,9 (215,3;273,6)	5078,4 (4533,7;5720,3)	155,2 (138,5;175,1)	-35,8(-37,9;-33,3)
Amapá	242,1 (217,7;270,6)	200 (178,2;226,3)	759,4 (676,7;858,9)	132 (117,3;149,8)	-34(-36,3;-31,7)
Amazonas	1967,1 (1759,6;2201,3)	208,6 (186,4;234,7)	3964,4 (3537,2;4450)	127,4 (113,7;144,3)	-38,9(-41,3;-36,7)
Bahia	16371,4 (14690,4;18405,9)	223,4 (200,3;252,7)	22215,8 (19841,1;25012,1)	136,9 (122,1;154,2)	-38,7(-40,8;-36,4)
Brasil	216640,6 (193728,5;242758,9)	224,6 (201,6;251,8)	295510,5 (264160,9;331953,6)	127 (113,8;142,1)	-43,5(-44,7;-42,2)
Ceará	8168,8 (7330,2;9148,3)	188,5 (169;212,2)	13059,6 (11671,9;14651,1)	130,8 (116,7;147,2)	-30,6(-33,2;-28)
Distrito Federal	1677,4 (1505;1870,5)	223 (199,4;249,7)	3131,6 (2790,5;3507,5)	116,6 (104;131,1)	-47,7(-49,7;-45,3)
Espírito Santo	3991,3 (3566,8;4491,6)	247,6 (219,9;279,8)	5720,5 (5104,5;6463,7)	132,7 (118,5;149,9)	-46,4(-48,6;-44,3)
Goiás	5462,9 (4882,1;6113,3)	229,2 (204,1;257,5)	8554,8 (7617,5;9655,7)	122,6 (109,5;138)	-46,5(-48,6;-44,1)
Maranhão	5729,8 (5149,3;6435,9)	203,2 (181,4;228,3)	9602,8 (8603,6;10833,2)	139,7 (124,9;157,8)	-31,2(-33,9;-28,4)
Mato Grosso	2066,5 (1850,5;2302,2)	213,6 (190,7;242,1)	4224,3 (3790,6;4757,8)	125,1 (112,3;141,8)	-41,4(-43,6;-38,9)
Mato Grosso do Sul	2349,4 (2107,6;2641,2)	228,7 (204,5;256,2)	3816 (3397,7;4324,3)	130,2 (116,1;146,7)	-43(-45,2;-40,6)
Minas Gerais	25738,5 (23130,3;28837,3)	240,8 (215,7;270,8)	32636,7 (29004,9;36771,1)	126,8 (113;142,6)	-47,3(-49,3;-45,4)
Pará	5334,6 (4779,3;5982,6)	220,2 (197;248,2)	9786,9 (8771,1;11013,3)	133 (118,6;150,4)	-39,6(-42,1;-37,2)
Paraíba	4666,9 (4181;5252)	196,3 (175,9;220,6)	5957,2 (5352,3;6650,6)	126,3 (113,5;141,3)	-35,7(-37,9;-33,3)
Paraná	13267,4 (11814,3;14914,7)	249,3 (222,8;281,2)	17372,2 (15503,4;19691,5)	134 (119,9;151,3)	-46,3(-48,3;-44,2)
Pernambuco	10750,5 (9606;12078,3)	224,3 (200,4;252,3)	13678,5 (12252,1;15426)	136,1 (121,7;153,6)	-39,3(-41,5;-36,9)
Piaui	3149,6 (2829,2;3542)	205,9 (184,8;232)	5037,5 (4505,3;5682,3)	133,4 (119,2;150,7)	-35,2(-37,3;-32,7)
Rio de Janeiro	25814,8 (23106,8;29202,3)	254,6 (228,2;287,6)	28566,5 (25517,7;32324)	131,8 (118,1;148,5)	-48,2(-50,2;-46,2)
Rio Grande do Norte	3299,3 (2948,3;3707)	196,4 (175,2;220,9)	4620,3 (4134,6;5184,7)	118,7 (106;133,6)	-39,6(-41,8;-37,2)
Rio Grande do Sul	16023,3 (14290,6;18026,9)	232 (207,3;260,5)	19270,5 (17107,4;21805,9)	129,9 (115,6;146,2)	-44(-46,1;-41,5)
Rondônia	1083,1 (962,8;1214)	238,5 (212,4;268,3)	2001,6 (1796,8;2250,1)	126,3 (113,1;142,4)	-47,1(-49,1;-45)
Roraima	169,2 (150,9;189,3)	210 (187,1;237,4)	498,4 (443,6;559)	121,8 (108,8;137,9)	-42(-44,2;-39,6)
Santa Catarina	6051,7 (5428,2;6799,7)	217,3 (194;244)	9233,2 (8225;10363,8)	116,9 (104,7;130,7)	-46,2(-48,4;-43,9)
São Paulo	46409,7 (41385,4;52301,6)	211,8 (189;237,8)	60790,5 (54232,4;68365,1)	115,3 (103,1;129,1)	-45,6(-47,6;-43,2)
Sergipe	1934 (1730;2172,7)	223,7 (199,5;252,8)	3130,2 (2788,5;3515,3)	137,5 (121,9;154,9)	-38,5(-41;-36,1)
Tocantins	983,6 (880,8;1103,9)	209,4 (186,8;235,8)	1915,1 (1711,7;2149,6)	130,4 (116,1;146,8)	-37,7(-39,9;-35,4)
**B.2.3.1-AVC isquêmico**					(;)
Acre	234,7 (198,1;276)	129,1 (109,1;153,4)	519,9 (437,3;621,3)	83,1 (69,5;100,1)	-35,7(-39;-32,1)
Alagoas	2126,1 (1797,7;2557,6)	152,1 (127,7;182,7)	3132,2 (2649,1;3741)	98,3 (83,1;117,5)	-35,4(-38,5;-31,9)
Amapá	137,8 (116,4;163,5)	123,5 (104,7;146,9)	443,2 (371,4;527,4)	83 (69,3;100,2)	-32,8(-36,4;-29,4)
Amazonas	1115,4 (942,3;1321,9)	128 (107,5;153)	2320 (1961,8;2766,2)	78,9 (66,2;94,7)	-38,4(-41,8;-35,2)
Bahia	9662,6 (8194;11470,4)	137 (115,2;163,5)	13529,8 (11418,1;16201,2)	84,4 (71,3;101,4)	-38,4(-41,6;-35,1)
Brasil	124392,2 (105330,6;147825,6)	136,6 (115,7;163,1)	179196,5 (151357,9;214373,1)	78,2 (66,1;93)	-42,7(-44,3;-41)
Ceará	4856,7 (4120,9;5778,9)	114,8 (97,5;137,8)	7890,5 (6663,6;9437,6)	80 (67,3;95,9)	-30,4(-34,1;-26,7)
Distrito Federal	868,1 (723,3;1033,8)	134 (112,8;159,3)	1809,8 (1509,1;2188,5)	72,3 (60,7;86,7)	-46,1(-49,1;-42,6)
Espírito Santo	2275,7 (1914,3;2715,3)	150,5 (125,8;179,6)	3467 (2922,3;4169)	82,2 (69,5;98,8)	-45,4(-48,6;-42,1)
Goiás	2992,8 (2527,4;3552,5)	136,8 (114,5;163,6)	5058,9 (4248,6;6087,8)	75,1 (63,2;89,3)	-45,1(-48,1;-41,4)
Maranhão	3396,3 (2881,3;4007,1)	125,3 (105,4;149,2)	5690,4 (4787,9;6828,1)	84,6 (70,6;102,1)	-32,5(-36,1;-28,1)
Mato Grosso	1153,3 (973;1377,4)	132,4 (111,5;158,9)	2517,5 (2120,5;3035,3)	78,1 (65,7;93,6)	-41(-44,3;-37,3)
Mato Grosso do Sul	1292,2 (1089,1;1537,5)	136,7 (115,3;162,6)	2274,4 (1911,5;2740,1)	79,7 (67;95,5)	-41,7(-44,5;-38,3)
Minas Gerais	14540,3 (12317,9;17373,4)	144,4 (122,3;173)	19901,1 (16652,3;23738,5)	77,6 (65,5;92,1)	-46,3(-49;-43,5)
Pará	3096,2 (2613,6;3679,7)	136,9 (115,2;164)	5917,9 (4992,5;7076,2)	84,1 (70,4;101)	-38,6(-42,2;-35,2)
Paraíba	2758,3 (2324,9;3314,4)	116,9 (98,1;139,2)	3468,1 (2933,9;4111,9)	73,2 (61,8;87,2)	-37,3(-40,7;-33,6)
Paraná	7723,4 (6504,8;9218,1)	155,3 (130,9;185,7)	10972 (9206,3;13160,8)	85,9 (72,6;102,6)	-44,7(-47,5;-41,6)
Pernambuco	6284,5 (5253,6;7513,5)	135,6 (114,1;162,7)	8045,9 (6746,4;9649)	81,9 (68,3;98,3)	-39,6(-43;-35,9)
Piaui	1833,4 (1549,5;2202,9)	125,4 (106;151,7)	3104,4 (2627,3;3712,2)	82,4 (69,6;98,3)	-34,3(-37,4;-30,3)
Rio de Janeiro	14572,8 (12240,7;17353,3)	152,1 (128,2;181,2)	17229,2 (14426,9;20533,9)	79,8 (67,5;94,4)	-47,6(-50,4;-44,4)
Rio Grande do Norte	1983,6 (1678,6;2356)	120,1 (101,2;142,8)	2791,3 (2349,1;3316,4)	72,3 (60,9;85,8)	-39,8(-43;-36,4)
Rio Grande do Sul	9402,5 (7929,3;11220)	143,6 (121,4;171,7)	12220,3 (10214;14674,4)	81,9 (68,8;97,5)	-43(-46,1;-39,6)
Rondônia	593,2 (495,3;710,8)	149,9 (126;178,4)	1196,9 (1010,6;1430)	79,4 (66,9;94,5)	-47(-49,9;-44,3)
Roraima	91,8 (76,6;108,4)	130,7 (109,5;155,8)	296,9 (251,5;353,1)	77,9 (66,1;92,9)	-40,4(-43,5;-36,5)
Santa Catarina	3274,2 (2755,5;3906,2)	127,5 (107,2;151,8)	5606,5 (4695,5;6715,2)	73 (61,2;86,8)	-42,8(-46;-39,1)
São Paulo	26425,9 (22386,7;31427,6)	129 (109,4;154,4)	36725,9 (30859,2;43889,2)	70,8 (59,9;83,9)	-45,1(-47,9;-42)
Sergipe	1153,2 (971,2;1386,3)	139,3 (117,5;167,3)	1914,7 (1612,8;2293,7)	86,5 (72,8;104)	-37,9(-41,4;-34,3)
Tocantins	547,4 (457,3;652,8)	126,5 (105,7;150,2)	1151,6 (970,8;1370,7)	81 (68,2;96,7)	-35,9(-39;-32,4)
**B.2.3.2-Hemorragia intracerebral**					(;)
Acre	126,1 (107,3;149,1)	60 (50,8;70,3)	233,4 (196,3;275,5)	33 (27,8;39,1)	-45(-47,6;-42,2)
Alagoas	1026,1 (872,9;1207,1)	67,1 (56,8;78,6)	1319,9 (1108,2;1573,9)	39 (32,8;46,8)	-41,9(-44,8;-38,8)
Amapá	74,2 (62,7;87,1)	56,4 (47,6;66,1)	199,7 (167,3;235,8)	32 (26,9;38)	-43,2(-46;-40,4)
Amazonas	619,5 (524,9;728)	60,9 (51,9;71,2)	1057,5 (883,3;1245,1)	32 (26,9;38,1)	-47,4(-50,1;-44,8)
Bahia	4959,8 (4246;5818,1)	64,9 (55,4;76,4)	5735,4 (4853,1;6730)	34,9 (29,5;41)	-46,1(-48,7;-43,3)
Brasil	67428,6 (57078,1;79099,3)	66 (56,1;77,9)	74671 (62811,6;88635,8)	31,6 (26,6;37,3)	-52,1(-53,5;-50,5)
Ceará	2343,9 (1995,2;2740,3)	52,8 (44,8;62,2)	3351,2 (2826,3;3942,5)	33,2 (28;39)	-37,1(-40,2;-33,7)
Distrito Federal	558,7 (471;663,8)	65,4 (55,8;77,3)	790,4 (654,4;941,1)	27,6 (23,2;32,8)	-57,8(-60,2;-55,4)
Espírito Santo	1270,6 (1075,6;1501,2)	73,9 (62,8;87,5)	1461,3 (1228,6;1746,4)	33,1 (27,9;39,5)	-55,1(-57,3;-52,7)
Goiás	1822,3 (1538,8;2124,8)	70,5 (60,1;82,8)	2248,2 (1886,2;2685,2)	31,1 (26,2;37)	-55,9(-58,2;-53,5)
Maranhão	1641 (1394,3;1921,1)	55,7 (47,3;65,5)	2557,2 (2151,7;3039,5)	36,3 (30,3;43,1)	-34,9(-38,2;-31,4)
Mato Grosso	646,7 (543,7;760,9)	60,2 (50,9;70,9)	1077,9 (903,8;1284,1)	30,4 (25,5;36,1)	-49,6(-52,1;-47)
Mato Grosso do Sul	777,3 (655,6;912,7)	69,8 (59,4;82,2)	1002 (836;1189,7)	33,3 (28;39,4)	-52,3(-54,8;-49,9)
Minas Gerais	8317,6 (7018;9699,9)	73,1 (62,5;85,2)	8217,1 (6891,1;9768,9)	31,9 (27;37,7)	-56,4(-58,8;-54,2)
Pará	1633,3 (1385,5;1919,1)	62,8 (53,3;73,8)	2491 (2103,9;2941,4)	32,1 (27,1;38,1)	-48,8(-51,2;-46,2)
Paraíba	1357 (1140,3;1598,3)	56,6 (47,7;66,5)	1652,8 (1391,8;1948,6)	35,2 (29,6;41,7)	-37,7(-40,7;-34,5)
Paraná	4074,7 (3437,9;4796,7)	71,4 (60,1;84,1)	4081,4 (3406,8;4870)	31 (26,1;36,7)	-56,6(-58,9;-54,1)
Pernambuco	3388,5 (2863;3953,6)	68,2 (57,9;79,9)	3762,9 (3169,8;4455,3)	36,6 (30,9;43,3)	-46,4(-48,8;-43,8)
Piaui	933,5 (789,8;1101,7)	58,4 (49,1;68,8)	1256,6 (1061,5;1482,9)	33,3 (28,1;39,4)	-42,9(-45,6;-40,3)
Rio de Janeiro	8489,5 (7139,9;9988,4)	79 (67,3;92)	7573,4 (6346,9;8980,5)	34,9 (29,5;41)	-55,8(-58,3;-53,3)
Rio Grande do Norte	963,6 (817,6;1128,9)	56,4 (47,7;65,9)	1188,6 (1009,1;1401,6)	30,3 (25,7;35,8)	-46,2(-48,5;-43,6)
Rio Grande do Sul	4943,9 (4168;5839)	67,4 (57,3;79,4)	4659,2 (3902;5531)	31,7 (26,8;37,4)	-53(-55,5;-50,6)
Rondônia	351,8 (294,1;419,3)	67,5 (57,2;79,2)	508,8 (427;605)	30,4 (25,7;36)	-54,9(-57,1;-52,8)
Roraima	54,6 (45,5;64,3)	59,6 (50,5;69,8)	125,4 (105,4;149,5)	28,4 (23,9;33,8)	-52,4(-54,9;-49,7)
Santa Catarina	2023,7 (1711,8;2390,1)	67,8 (57,7;79,4)	2267,2 (1895,1;2697,9)	27,9 (23,5;32,9)	-58,8(-61;-56,4)
São Paulo	14142,1 (11564,1;17144,7)	60,6 (49,9;73,2)	14566,1 (11933,1;17602,4)	27,1 (22,3;32,5)	-55,2(-57,4;-52,4)
Sergipe	577,7 (491,6;674,3)	63,8 (53,9;74,8)	802,6 (679;952,8)	34,2 (28,9;40,7)	-46,4(-49;-43,5)
Tocantins	311 (262,5;365,8)	61,3 (52,1;71,9)	483,8 (409,1;573)	31,8 (26,9;37,8)	-48(-50,7;-45,6)
**B.2.3.3-Hemorragia subaracnóidea**					(;)
Acre	51,1 (43,4;61,2)	21,4 (18;25,6)	134,1 (112,5;160,6)	17,5 (14,7;21,1)	-18,3(-23,6;-13,3)
Alagoas	373,7 (317,3;440,8)	22,6 (19,1;26,8)	626,2 (523,3;750,7)	17,9 (15;21,4)	-20,9(-26;-15,6)
Amapá	30,2 (25,5;35,7)	20,1 (16,9;24)	116,5 (97,9;137,7)	17 (14,2;20,1)	-15,4(-20,4;-10,1)
Amazonas	232,3 (195,8;277,2)	19,7 (16,6;23,5)	586,9 (497;703,8)	16,5 (14;19,9)	-16,2(-21,1;-11,1)
Bahia	1749 (1483,9;2051,6)	21,5 (18,2;25,7)	2950,6 (2469;3519,1)	17,6 (14,7;20,9)	-18,3(-23,3;-13,3)
Brasil	24819,8 (21095;29388,3)	22,1 (18,6;26,3)	41643 (34891,4;50228,6)	17,2 (14,4;20,6)	-22,2(-25,1;-19,3)
Ceará	968,2 (825,4;1146,9)	20,9 (17,8;25)	1817,9 (1524;2194,1)	17,7 (14,8;21,3)	-15,7(-20,8;-10,8)
Distrito Federal	250,6 (211,3;301,2)	23,5 (19,8;28,1)	531,4 (441,6;644)	16,7 (14;20)	-29(-33,2;-24)
Espírito Santo	445 (377,6;531,3)	23,2 (19,6;27,8)	792,1 (654,9;957,8)	17,4 (14,5;20,9)	-25(-30;-19,8)
Goiás	647,8 (545,5;774,8)	22 (18,5;26,3)	1247,7 (1037,7;1523,1)	16,4 (13,7;19,9)	-25,2(-29,7;-20,6)
Maranhão	692,5 (586,5;829,9)	22,1 (18,6;26,7)	1355,2 (1142,2;1621)	18,8 (15,8;22,7)	-14,9(-20,1;-9,2)
Mato Grosso	266,6 (222,9;317,9)	20,9 (17,5;24,9)	628,9 (526,9;754,9)	16,6 (14;19,9)	-20,6(-25,2;-15,5)
Mato Grosso do Sul	279,9 (237;330,7)	22,1 (18,7;26,4)	539,6 (447,2;649)	17,2 (14,4;20,6)	-22,2(-27,2;-17,6)
Minas Gerais	2880,7 (2426,3;3418)	23,2 (19,5;27,6)	4518,6 (3782,4;5427,8)	17,3 (14,6;20,7)	-25,4(-30,7;-20,3)
Pará	605,2 (511,4;718,8)	20,4 (17;24,7)	1378,1 (1167,4;1628,7)	16,8 (14,2;19,8)	-17,8(-22,6;-12,3)
Paraíba	551,5 (469;654,5)	22,8 (19,3;27,4)	836,3 (703;998,7)	17,8 (15;21,3)	-22,1(-27,1;-17,3)
Paraná	1469,3 (1239,3;1759,9)	22,6 (19,1;27,1)	2318,8 (1934,8;2839,6)	17,1 (14,4;20,8)	-24,4(-29,3;-19,7)
Pernambuco	1077,4 (909,4;1293,2)	20,4 (17,1;24,8)	1869,7 (1556;2258,3)	17,7 (14,8;21,2)	-13,5(-18,6;-7,9)
Piaui	382,7 (322,5;456,4)	22,2 (18,6;26,8)	676,5 (564,7;813,9)	17,8 (14,8;21,4)	-19,8(-24,6;-14,5)
Rio de Janeiro	2752,4 (2313,7;3277,1)	23,5 (19,8;28,2)	3763,9 (3113,5;4544,3)	17,2 (14,3;20,6)	-27(-31,6;-21,8)
Rio Grande do Norte	352,1 (296,8;418,4)	20 (16,7;23,9)	640,4 (533,2;768,3)	16,1 (13,4;19,3)	-19,5(-24,2;-14,9)
Rio Grande do Sul	1676,9 (1410,2;2002,7)	20,9 (17,5;24,9)	2390,9 (1975,7;2914,1)	16,3 (13,7;19,7)	-21,8(-26,6;-16,4)
Rondônia	138 (115,7;165,1)	21 (17,7;25,2)	295,9 (246,3;357)	16,4 (13,8;19,7)	-22(-26,6;-17,1)
Roraima	22,8 (18,9;27,4)	19,7 (16,4;23,8)	76,1 (62,9;90,7)	15,5 (13;18,6)	-21,3(-26;-15,8)
Santa Catarina	753,8 (635,9;895)	22 (18,4;26,3)	1359,5 (1133;1634,6)	16 (13,5;19)	-27,2(-31,8;-22,4)
São Paulo	5841,7 (4872,1;7001,2)	22,2 (18,6;26,8)	9498,5 (7897,7;11571,9)	17,3 (14,5;21)	-22,2(-27,1;-16,8)
Sergipe	203,1 (173;240,8)	20,7 (17,6;24,6)	413 (345,2;497,7)	16,8 (14;20,3)	-18,7(-23,5;-13,7)
Tocantins	125,3 (106,8;148,7)	21,7 (18,4;26)	279,8 (234,6;335,7)	17,6 (14,8;21,1)	-19,1(-23,8;-14,4)


Fonte: Dados derivados do estudo Global Burden of Disease 2019, Institute for Health Metrics and Evaluation, University of Washington. *^*46*^*

**Tabela 2-4:** – Número de casos e taxas de incidência padronizadas por idade (por 100 mil) de AVC, AVC isquêmico, hemorragia subaracnóidea e hemorragia intracerebral em 1990 e 2019, com variação percentual das taxas, no Brasil, de acordo com o grupo etário.

Causa de morte e grupo etário		1990				2019			Variação percentual (II 95%)
		Número (II 95%)		Taxa (II 95%)		Número (II 95%)		Taxa (II 95%)	
B.2.3-AVC									
Abaixo de 5		4796,6 (3097,4;7039,4)		28,3 (18,3;41,6)		3972,4 (2556,8;5853)		25,6 (16,5;37,8)	-9,5 (-12,9;-6,5)
15-49 anos		63360,3 (54287,5;74063,7)		82,7 (70,8;96,6)		59083,8 (50210,7;69549,5)		51,2 (43,5;60,2)	-38,1 (-40,9;-35,2)
50-49 anos		87814,2 (73059,9;104528,3)		559,8 (465,7;666,3)		120686,6 (99991,1;144226,9)		299,1 (247,9;357,5)	-46,6 (-48,1;-44,8)
5-14 anos		7322,4 (4825,9;10780,3)		20,7 (13,7;30,5)		6041,7 (3937,9;8992,8)		18,7 (12,2;27,9)	-9,6 (-13;-6,6)
70+ anos		53347,1 (43596,4;65336,4)		1261,1 (1030,6;1544,6)		105726 (87241,1;128305,7)		807,8 (666,5;980,3)	-35,9 (-38,7;-32,6)
Padronizada por idade		216640,6 (193728,5;242758,9)		224,6 (201,6;251,8)		295510,5 (264160,9;331953,6)		127 (113,8;142,1)	-43,5 (-44,7;-42,2)
Toda as idades		216640,6 (193728,5;242758,9)		145,6 (130,2;163,1)		295510,5 (264160,9;331953,6)		136,4 (121,9;153,2)	-6,3 (-9,4;-3,2)
**B.2.3.1-AVC isquêmico**									
Abaixo de 5		4013,6 (2501,9;6255,9)		23,7 (14,8;36,9)		3297 (2025,3;5218)		21,3 (13,1;33,7)	-10,2 (-14,5;-6,8)
15-49 anos		24916,5 (18575,7;33199,8)		32,5 (24,2;43,3)		24363,2 (17402,1;33586,9)		21,1 (15,1;29,1)	-35,1 (-40,1;-30,4)
50-49 anos		51461,8 (38832,1;65653,7)		328 (247,5;418,5)		68841,4 (51313,9;88179,8)		170,6 (127,2;218,6)	-48 (-49,9;-45,9)
5-14 anos		4407,5 (2257,7;7655,6)		12,5 (6,4;21,7)		3662,4 (1838,8;6551,7)		11,4 (5,7;20,3)	-9 (-14,4;-4,6)
70+ anos		39592,8 (30181,9;51380,8)		936 (713,5;1214,7)		79032,5 (61618,1;100736,8)		603,8 (470,8;769,7)	-35,5 (-39;-31,2)
Padronizada por idade		124392,2 (105330,6;147825,6)		136,6 (115,7;163,1)		179196,5 (151357,9;214373,1)		78,2 (66,1;93)	-42,7 (-44,3;-41)
Toda as idades		124392,2 (105330,6;147825,6)		83,6 (70,8;99,3)		179196,5 (151357,9;214373,1)		82,7 (69,9;98,9)	-1 (-5,4;3,1)
**B.2.3.2-Hemorragia intracerebral**									
Abaixo de 5		639,3 (316,8;1088,4)		3,8 (1,9;6,4)		544,8 (268,7;901)		3,5 (1,7;5,8)	-6,9 (-11,6;-2,1)
15-49 anos		25215,1 (19371,2;31655,2)		32,9 (25,3;41,3)		19485,6 (14722,1;24909,6)		16,9 (12,7;21,6)	-48,7 (-51,5;-46,1)
50-49 anos		27557,1 (20992,6;36179,2)		175,7 (133,8;230,6)		33135,5 (24835,4;43786,9)		82,1 (61,6;108,5)	-53,2 (-55,3;-51,5)
5-14 anos		2338,7 (1254,4;3880,9)		6,6 (3,6;11)		1875,7 (994,1;3114,7)		5,8 (3,1;9,7)	-12,1 (-16,1;-7,6)
70+ anos		11678,4 (8940,8;15312,2)		276,1 (211,4;362)		19629,4 (15234,8;25320,2)		150 (116,4;193,5)	-45,7 (-48,6;-42,2)
Padronizada por idade		67428,6 (57078,1;79099,3)		66 (56,1;77,9)		74671 (62811,6;88635,8)		31,6 (26,6;37,3)	-52,1 (-53,5;-50,5)
Toda as idades		67428,6 (57078,1;79099,3)		45,3 (38,3;53,1)		74671 (62811,6;88635,8)		34,5 (29;40,9)	-23,9 (-27,7;-20,4)
**B.2.3.3-Hemorragia subaracnóidea**									
Abaixo de 5		143,6 (68,7;277,7)		0,8 (0,4;1,6)		130,7 (62,9;249,2)		0,8 (0,4;1,6)	-0,5 (-5,1;4,2)
15-49 anos		13228,8 (10504,7;16567,2)		17,3 (13,7;21,6)		15235 (11825;19094,1)		13,2 (10,2;16,5)	-23,6 (-27,8;-19,5)
50-49 anos		8795,2 (6604,7;11436)		56,1 (42,1;72,9)		18709,7 (13924,4;24605,4)		46,4 (34,5;61)	-17,3 (-20,8;-13,5)
5-14 anos		576,3 (321,8;933,3)		1,6 (0,9;2,6)		503,5 (278,8;821,4)		1,6 (0,9;2,5)	-4,3 (-8,3;-0,7)
70+ anos		2075,8 (1530,9;2729,2)		49,1 (36,2;64,5)		7064 (5300,2;9103,7)		54 (40,5;69,6)	10 (3,8;16,6)
Padronizada por idade		24819,8 (21095;29388,3)		22,1 (18,6;26,3)		41643 (34891,4;50228,6)		17,2 (14,4;20,6)	-22,2 (-25,1;-19,3)
Toda as idades		24819,8 (21095;29388,3)		16,7 (14,2;19,7)		41643 (34891,4;50228,6)		19,2 (16,1;23,2)	15,3 (8,2;22,1)

*Fonte: Dados derivados do estudo Global Burden of Disease 2019, Institute for Health Metrics and Evaluation, University of Washington. ^*46*^*

**Tabela 2-5:** – Número de mortes e taxas de mortalidade padronizadas por idade (por 100 mil) por AVC, AVC isquêmico, hemorragia subaracnóidea e hemorragia intracerebral em 1990 e 2019, com variação percentual das taxas, no Brasil e suas unidades federativas.

Causa de morte e localização	1990		2019		Variação percentual (II 95%)
	Número (II 95%)	Taxa (II 95%)	Número (II 95%)	Taxa (II 95%)	
**B.2.3-AVC**					
Acre	143,8 (131,9;155,4)	116,6 (105,3;126,7)	346,6 (310,2;383,2)	65,4 (58;72,4)	-43,9(-50,1;-36,7)
Alagoas	1893,6 (1719,9;2096,1)	159 (142,9;175,9)	2592 (2250,7;2940,1)	85,2 (73,4;96,7)	-46,4(-54,8;-37,4)
Amapá	77,5 (70,7;83,5)	98,6 (88,6;106,2)	271,3 (240,2;300,2)	60,1 (52,3;66,6)	-39,1(-45,1;-32,7)
Amazonas	751,6 (686,8;811,9)	122,6 (111,9;132,7)	1496,8 (1303,8;1681,6)	57,8 (49,9;65,1)	-52,9(-58,2;-46,9)
Bahia	7365,2 (6492,7;8251,6)	118,4 (103,9;132,6)	10378,7 (8704,1;12165,3)	64,5 (54,1;75,7)	-45,5(-55;-34,1)
Brasil	105603,9 (100300,3;109634,9)	137,8 (127,8;144)	131007 (119134,6;139017,7)	58,1 (52,6;61,8)	-57,8(-60,4;-55,5)
Ceará	3804,6 (3224,5;4401,7)	97,6 (82,5;113,2)	6627,7 (5527,6;7761)	67,8 (56,7;79,3)	-30,5(-44,4;-13,9)
Distrito Federal	605,3 (547,2;681,5)	161,8 (148;178,1)	1134,4 (1002,1;1274,2)	64,2 (56,4;71,8)	-60,4(-65,2;-55)
Espírito Santo	2030 (1918,2;2129,2)	169,6 (156,7;178,3)	2591,1 (2257,7;2915,3)	63,8 (55,4;71,9)	-62,4(-66,7;-58,1)
Goiás	2732,6 (2384,6;3201,9)	160 (140,7;186,4)	3477 (2933,1;4024,5)	54,5 (45,9;63,2)	-65,9(-71,8;-59,1)
Maranhão	2727,7 (2300,3;3185,3)	118,4 (99,1;137,9)	5826,6 (4963;6787,8)	91,6 (78,1;106,9)	-22,7(-37,2;-3,1)
Mato Grosso	731,8 (649;812,6)	116,9 (103,3;128,8)	1521,5 (1344;1716,5)	51,5 (45;58,1)	-56(-61,6;-49,2)
Mato Grosso do Sul	1019,2 (949,8;1089,1)	137,6 (125,9;147,5)	1497,5 (1313,8;1684,4)	55,1 (48,2;61,9)	-60(-64,3;-55,1)
Minas Gerais	12354,4 (11491,7;13383,7)	146,6 (135,3;158,3)	13090,3 (11454,5;14614,3)	50,1 (43,9;55,9)	-65,9(-69,9;-61,9)
Pará	2536,9 (2266,4;2816,3)	151,5 (134;167,8)	4274,4 (3680,9;4778)	66,2 (56,9;74,1)	-56,3(-61,8;-49,9)
Paraíba	2238,8 (1946,1;2517,7)	101,8 (88,2;114,8)	2887,6 (2473,2;3298)	58,8 (50,6;67,1)	-42,2(-51,3;-31,1)
Paraná	6868,8 (6515;7188,2)	177,5 (165,7;186,5)	7742,1 (6777;8670,9)	63,5 (55,5;71)	-64,2(-68,2;-60,3)
Pernambuco	5685,9 (5287,8;6033)	143,6 (131,7;153,4)	6749,5 (5954,7;7559,6)	70,9 (62,3;79,5)	-50,6(-56,2;-44,6)
Piaui	1678,5 (1513,5;1851,4)	144,9 (128,4;160,8)	2546,2 (2165,1;2865,1)	66,3 (56,9;74,5)	-54,3(-60,1;-47,9)
Rio de Janeiro	14063,6 (13347,2;14643,9)	168,9 (158,4;176,7)	12722,1 (11239,8;14074,9)	58,4 (51,7;64,6)	-65,5(-68,8;-62)
Rio Grande do Norte	1363,5 (1194;1525,5)	89,9 (78,1;100,7)	1750,4 (1451,8;2056,2)	44,2 (36,8;52)	-50,8(-59,2;-40,6)
Rio Grande do Sul	7690 (7244,8;8075,8)	141 (130,9;148,5)	9167,2 (8020,6;10250,4)	60,1 (52,4;67,3)	-57,4(-61,5;-52,8)
Rondônia	395,6 (350;438)	195,7 (179,1;211,8)	825,4 (709,6;948,7)	60,9 (52,2;70,2)	-68,9(-73,4;-63,5)
Roraima	53,7 (48,3;59,2)	145,2 (131,6;158,1)	170,8 (151,3;189,2)	61,1 (53,4;67,5)	-57,9(-62,6;-52,6)
Santa Catarina	3221,9 (3005,7;3411,7)	161,2 (148,7;171,9)	3786,8 (3322,4;4250,2)	52,6 (45,9;58,9)	-67,4(-71;-63,8)
São Paulo	22207,4 (20875;23529)	133,5 (123,7;141,7)	25162,9 (22011,3;27840,6)	49,3 (42,9;54,6)	-63,1(-66,8;-59,2)
Sergipe	955,9 (866,9;1041,7)	148 (133,8;161,5)	1423,6 (1207;1642,1)	66,6 (56,5;76,8)	-55(-61,9;-47,7)
Tocantins	406,1 (354,8;458,2)	146,8 (128,7;166)	946,9 (817,8;1082,4)	71,5 (61,4;81,5)	-51,3(-58,8;-42,2)
**B.2.3.1-AVC isquêmico**					**(;)**
Acre	70,1 (62,3;77,5)	72,3 (63,5;80,1)	181,6 (158,2;205,7)	38,9 (33,5;44,1)	-46,2(-53,4;-37,2)
Alagoas	1044,8 (903,6;1192,7)	98,3 (84,9;111,7)	1480,4 (1261,6;1693,5)	51 (43,4;58,4)	-48,1(-57,5;-37,6)
Amapá	39,7 (35,4;43,1)	61,6 (53,7;67,3)	139,6 (119,5;156,1)	35,8 (30,3;40,3)	-41,9(-47,8;-35,4)
Amazonas	358,8 (324,9;390,4)	72,2 (64,7;78,8)	791 (664;897,8)	33,6 (28,2;38,2)	-53,4(-59,1;-47,4)
Bahia	3842,8 (3286,8;4392,2)	67,9 (57,8;77,4)	5850,1 (4764,5;6852,8)	36,6 (29,9;43)	-46(-57;-33,6)
Brasil	52583,6 (48875,7;55110,8)	80 (72,5;84,3)	73920,9 (64818,8;79592,8)	33,9 (29,7;36,6)	-57,6(-60,9;-54,4)
Ceará	2118,4 (1732,9;2534,7)	57,3 (46,8;68,4)	3934,3 (3210,7;4689,3)	40,8 (33,4;48,6)	-28,7(-44;-7,8)
Distrito Federal	220,4 (198,2;248,8)	97 (87,6;107,8)	605,3 (526,7;685,6)	42 (36,3;47,5)	-56,7(-62,1;-50,5)
Espírito Santo	1014,7 (936;1080,7)	101,5 (92,4;108)	1430,6 (1220,2;1628,7)	37,2 (31,6;42,5)	-63,3(-67,8;-58,7)
Goiás	1188,5 (1024,2;1385,5)	89,1 (77,8;102,5)	1817 (1514,1;2122,7)	30,9 (25,6;36,3)	-65,3(-71,2;-58,6)
Maranhão	1407,4 (1117,9;1698,7)	71 (56,5;84,4)	3417,1 (2880,3;3967,2)	55,3 (46,7;64,2)	-22,1(-39,3;1)
Mato Grosso	343,7 (302,2;384,8)	70,5 (61,1;78,3)	797,9 (683,6;907,2)	30 (25,4;34,2)	-57,4(-63,1;-51,2)
Mato Grosso do Sul	444,6 (403;481,7)	76 (68,2;82,5)	792,9 (683,4;899,6)	31,2 (26,7;35,4)	-59(-63,8;-53,6)
Minas Gerais	5799 (5307,3;6297,5)	82,7 (74,8;89,5)	7229,9 (6179,5;8168,3)	27,9 (23,8;31,6)	-66,2(-70,2;-62,1)
Pará	1345,9 (1183,7;1495,4)	94,5 (81,9;104,9)	2458,5 (2074,9;2785,8)	40,6 (34,3;46)	-57(-62,7;-50,5)
Paraíba	1316,8 (1091,7;1523)	61,9 (51,1;71,6)	1672,8 (1379,4;1924,3)	33,2 (27,7;38,1)	-46,4(-56,5;-33,8)
Paraná	3462,5 (3204,1;3679,6)	108,1 (98,5;115,3)	4689,7 (4062,5;5269,4)	40,4 (34,7;45,3)	-62,7(-67,1;-58,4)
Pernambuco	2975,1 (2658,4;3245,8)	83,7 (74,2;90,9)	3626,1 (3104,5;4110,4)	39,9 (34,1;45,3)	-52,3(-58,9;-45)
Piaui	901 (780,3;1014,2)	89,9 (77,2;101,3)	1562,6 (1267,6;1778,7)	40,3 (33;45,8)	-55,2(-62;-47,8)
Rio de Janeiro	6587,9 (6142,6;6974,8)	94 (86,6;99,7)	6704,8 (5839;7432,1)	31,5 (27,3;34,9)	-66,5(-70;-63)
Rio Grande do Norte	782,5 (666,9;896,7)	53,8 (45,2;61,6)	1026,1 (825,4;1217,3)	25,7 (20,8;30,4)	-52,1(-60,8;-40,6)
Rio Grande do Sul	4123,2 (3793,8;4390,4)	87,1 (78,9;92,8)	5640,7 (4783,8;6329,9)	37,3 (31,6;41,9)	-57,2(-61,6;-52,3)
Rondônia	159,7 (142,5;177,4)	126,6 (115;137,7)	447,2 (378,1;519,2)	36,6 (30,8;42,4)	-71,1(-75,6;-66,1)
Roraima	21,9 (19,8;23,9)	91,3 (81,4;99,8)	87,3 (76;97,2)	38,5 (32,9;43)	-57,9(-63,3;-52,5)
Santa Catarina	1659,3 (1532,4;1780,8)	99 (90,2;106,5)	2258,1 (1953,1;2547,1)	33,5 (28,7;37,8)	-66,2(-70,2;-62)
São Paulo	10643,9 (9783,4;11370)	77,8 (70,4;83,3)	13918,4 (11936,9;15470,3)	28,5 (24,3;31,7)	-63,4(-67;-59,5)
Sergipe	523 (466,3;577,6)	92,2 (81,9;101,6)	819,2 (679,8;954)	40 (33,2;46,6)	-56,7(-63,7;-48,4)
Tocantins	188,1 (162;215,5)	91,9 (79,3;104,4)	541,5 (457,2;626,5)	43,2 (36,3;50)	-53(-61,6;-43,3)
**B.2.3.2-Hemorragia intracerebral**					(;)
Acre	58,9 (53,1;65,3)	37,4 (33,5;41,3)	125,5 (112,1;140,6)	20,8 (18,6;23,3)	-44,4(-52;-35,1)
Alagoas	702,4 (602,1;808,7)	51,7 (44,2;59,7)	886,7 (770,1;1011,6)	27,6 (23,9;31,6)	-46,6(-58,3;-33,5)
Amapá	31,1 (28,2;33,9)	32,1 (29;34,8)	101,4 (89,3;113,2)	19,4 (17;21,6)	-39,5(-46,4;-31,5)
Amazonas	334 (302,8;367,5)	44,6 (40,3;49)	554,6 (485,6;628,3)	19,5 (17;22,2)	-56,2(-61,9;-49,4)
Bahia	2951,9 (2566,8;3389,6)	43,3 (37,5;49,7)	3553,6 (2961,9;4213,8)	22 (18,3;26,1)	-49,2(-59,8;-35,7)
Brasil	44537,3 (42391,2;46721)	49,8 (47,1;52,2)	43825,9 (40717,3;46438,1)	18,6 (17,3;19,8)	-62,6(-65,3;-59,7)
Ceará	1391 (1155,5;1654,1)	33,9 (28;40,5)	2120,3 (1745,1;2510,6)	21,4 (17,6;25,3)	-37(-52;-18,3)
Distrito Federal	305,4 (272,1;350,2)	55,6 (50;62,3)	382,9 (334,5;434,5)	16,6 (14,4;18,8)	-70,2(-74,4;-65,2)
Espírito Santo	856,4 (808,2;905,6)	59,1 (55,5;62,8)	896,6 (777,8;1015,2)	20,7 (17,9;23,5)	-65(-69,7;-60)
Goiás	1296,7 (1119,5;1552)	61,8 (53,7;73,2)	1269 (1068,7;1485,1)	18,3 (15,5;21,3)	-70,4(-76;-63,8)
Maranhão	1056,1 (839,2;1317,7)	39,2 (30,8;49,2)	1867,8 (1566,3;2245,4)	28,5 (23,8;34,3)	-27,3(-45,5;0)
Mato Grosso	314,1 (273,8;354,4)	39,8 (34,9;44,6)	543,3 (478;616,5)	16,5 (14,5;18,7)	-58,5(-65,1;-50,7)
Mato Grosso do Sul	480,1 (444,8;519,9)	53,4 (49,4;57,7)	540,7 (473,4;616,2)	18,5 (16,2;21,2)	-65,3(-69,7;-60,1)
Minas Gerais	5492,7 (5064,6;6110,2)	55 (50,7;60,6)	4397,3 (3880,7;4919,8)	16,6 (14,7;18,5)	-69,9(-73,9;-65,3)
Pará	1022,3 (907,3;1149)	50,7 (44,9;56,9)	1421,5 (1232,5;1598,4)	20,4 (17,7;23,1)	-59,7(-65,7;-52,5)
Paraíba	736,5 (612,7;889,3)	32 (26,6;38,7)	954,1 (827,2;1097,1)	20,1 (17,4;23,1)	-37,3(-51,4;-20,3)
Paraná	2891,5 (2721,3;3082,7)	60,7 (56,7;64,6)	2314,2 (2019,3;2620,5)	17,6 (15,3;19,9)	-71(-75;-67,1)
Pernambuco	2377,5 (2199,5;2588,7)	53,3 (49,1;58,1)	2491,7 (2197,6;2813,9)	24,9 (22;28,1)	-53,4(-59,8;-46)
Piaui	643,2 (567,5;736,9)	46,8 (41;54)	775,3 (672,3;890,1)	20,5 (17,8;23,6)	-56,1(-63,9;-47,4)
Rio de Janeiro	6351,1 (6010;6736,8)	64,9 (61,3;68,9)	4705,5 (4169,5;5275,8)	20,9 (18,5;23,5)	-67,8(-71,5;-63,4)
Rio Grande do Norte	493,1 (431,4;561,6)	31,1 (27,1;35,4)	570,8 (468,7;677,4)	14,6 (12,1;17,4)	-52,9(-62,4;-41,8)
Rio Grande do Sul	3066,7 (2873,9;3255,1)	47,3 (44,1;50,3)	2801,4 (2461,1;3171,1)	18 (15,8;20,3)	-62(-66,5;-56,9)
Rondônia	196,6 (170,7;221,3)	61,5 (54,9;67,6)	288,5 (246,5;339,4)	19 (16,3;22,4)	-69(-74,1;-62,4)
Roraima	26 (22,9;28,9)	47,3 (43;52)	63,2 (55,7;70,9)	17,8 (15,8;20)	-62,3(-67,5;-56,6)
Santa Catarina	1330,5 (1234,2;1427,7)	54,7 (50,3;58,7)	1160,9 (1008,4;1314,2)	14,6 (12,7;16,5)	-73,2(-76,6;-69,5)
São Paulo	9579,9 (8924,2;10296,7)	47,8 (44,3;51,4)	8239 (7219,3;9239,4)	15,3 (13,4;17,1)	-67,9(-72;-63,7)
Sergipe	372 (329,2;416,2)	49,1 (43,3;55)	483,9 (411,5;563,2)	21,6 (18,4;25,2)	-55,9(-63,9;-46,9)
Tocantins	179,6 (150,7;210,6)	47,4 (39,5;55,7)	316,3 (271,6;365,5)	22,4 (19,3;25,9)	-52,6(-62,9;-40,4)
**B.2.3.3-Hemorragia subaracnóidea**					(;)
Acre	14,9 (13;16,9)	6,9 (6,1;7,8)	39,5 (34,9;44,8)	5,7 (5;6,4)	-18(-30,8;-0,2)
Alagoas	146,4 (123,5;172)	9 (7,6;10,7)	224,8 (191,5;261,7)	6,6 (5,6;7,7)	-27,2(-43,3;-6,1)
Amapá	6,6 (5,8;7,7)	4,9 (4,4;5,8)	30,4 (26,9;35)	4,9 (4,3;5,6)	-1,6(-15,3;14)
Amazonas	58,9 (52;67,4)	5,8 (5,2;6,8)	151,3 (131,4;174,4)	4,6 (4;5,4)	-20,5(-34,1;-4,5)
Bahia	570,5 (494;662,3)	7,2 (6,2;8,5)	975 (798,2;1173,9)	5,9 (4,8;7,1)	-18,8(-36,4;3,4)
Brasil	8483 (7668,1;8870,6)	8 (7,3;8,4)	13260,2 (12016,4;14155,9)	5,5 (5;5,9)	-30,3(-36,2;-23,6)
Ceará	295,3 (243;351,7)	6,5 (5,3;7,7)	573 (473,5;700,2)	5,6 (4,7;6,9)	-12,7(-32,5;18,3)
Distrito Federal	79,5 (70;89,6)	9,2 (8,1;10,4)	146,1 (122,3;168,3)	5,5 (4,4;6,4)	-40,1(-51;-28,6)
Espírito Santo	158,9 (131,5;173)	8,9 (7,5;9,7)	263,9 (222,5;310,3)	5,9 (5;6,9)	-33,7(-43,7;-21)
Goiás	247,4 (214,8;287,7)	9,2 (8;10,7)	391 (327,2;464,2)	5,3 (4,5;6,3)	-41,8(-53,2;-28,4)
Maranhão	264,1 (196,8;343,7)	8,2 (6;10,7)	541,6 (438,5;658)	7,8 (6,3;9,5)	-4,7(-31,9;33,9)
Mato Grosso	74,1 (62,4;86,7)	6,6 (5,7;7,7)	180,3 (156,6;208,1)	5 (4,3;5,8)	-24,7(-38,2;-8,6)
Mato Grosso do Sul	94,5 (85,3;103,3)	8,2 (7,5;9)	163,9 (141,6;187,4)	5,4 (4,6;6,1)	-34,8(-44,5;-24)
Minas Gerais	1062,8 (930,5;1162,6)	8,9 (7,9;9,7)	1463,2 (1232,6;1677,6)	5,6 (4,7;6,4)	-37,7(-47,1;-27,8)
Pará	168,7 (146,9;194,8)	6,3 (5,5;7,3)	394,4 (341,7;451,6)	5,1 (4,4;5,8)	-19,2(-33;-2,1)
Paraíba	185,5 (158,3;217,9)	7,8 (6,6;9,1)	260,6 (220,5;302,1)	5,5 (4,6;6,4)	-29,3(-45,3;-10,4)
Paraná	514,8 (457,6;557,9)	8,7 (7,8;9,4)	738,2 (620,2;856,4)	5,5 (4,6;6,4)	-36,7(-46,4;-26,8)
Pernambuco	333,2 (297,6;385,1)	6,6 (5,9;7,8)	631,7 (546,6;728,1)	6,1 (5,3;7)	-7,2(-24,3;11,1)
Piaui	134,3 (116,3;153)	8,1 (7;9,3)	208,3 (177,5;242,9)	5,5 (4,7;6,4)	-32,8(-46,4;-16,8)
Rio de Janeiro	1124,6 (903,7;1234,4)	10 (8,2;10,9)	1311,7 (1110,4;1494,2)	5,9 (5;6,8)	-40,4(-48,8;-28,9)
Rio Grande do Norte	87,8 (74,9;111,2)	5,1 (4,3;6,6)	153,4 (124,8;196,3)	3,9 (3,1;4,9)	-24,4(-40,4;-4,1)
Rio Grande do Sul	500 (459;551,6)	6,6 (6,1;7,3)	725,1 (623,3;839,4)	4,8 (4,1;5,5)	-27,5(-38,3;-15,9)
Rondônia	39,2 (32;45,6)	7,7 (6,6;8,7)	89,6 (76,9;105,3)	5,3 (4,5;6,1)	-31,4(-43,5;-15,5)
Roraima	5,8 (4,9;6,7)	6,6 (5,8;7,5)	20,3 (17,7;24,1)	4,8 (4,2;5,6)	-27(-37,8;-13,1)
Santa Catarina	232,2 (211,8;254)	7,6 (6,9;8,3)	367,8 (315,9;426)	4,5 (3,8;5,1)	-40,9(-49,2;-30,5)
São Paulo	1983,7 (1772,4;2164,2)	8 (7,3;8,7)	3005,4 (2542,4;3434,8)	5,5 (4,7;6,3)	-30,7(-40,8;-20,3)
Sergipe	60,9 (52,9;70,3)	6,7 (5,8;7,8)	120,5 (101,5;144,9)	5 (4,3;6,1)	-24,6(-40,5;-5,1)
Tocantins	38,4 (32;44,7)	7,5 (6,3;8,8)	89,1 (75,5;104,6)	5,8 (5;6,9)	-22,1(-37,2;-1,3)

*Fonte: Dados derivados do estudo Global Burden of Disease 2019, Institute for Health Metrics and Evaluation, University of Washington. ^*46*^*

**Tabela 2-6:** – Número de mortes e taxas de mortalidade padronizadas por idade (por 100 mil) por AVC, AVC isquêmico, hemorragia subaracnóidea e hemorragia intracerebral em 1990 e 2019, com variação percentual das taxas, no Brasil, de acordo com o grupo etário.

Causa de morte e grupo etário	1990		2019		Variação percentual (II 95%)
	Número (II 95%)	Taxa (II 95%)	Número (II 95%)	Taxa (II 95%)	
**B.2.3-AVC**					
Abaixo de 5	416,1 (303;567,7)	2,5 (1,8;3,4)	68,2 (49,6;93,1)	0,4 (0,3;0,6)	-82,1 (-89,3;-70,1)
15-49 anos	14553,1 (14010,9;15182,8)	19 (18,3;19,8)	9310,8 (8821,4;9798,8)	8,1 (7,6;8,5)	-57,5 (-60,4;-54,7)
50-49 anos	37192,4 (35828,1;38681)	237,1 (228,4;246,6)	37277,4 (35281,9;39071,1)	92,4 (87,5;96,8)	-61 (-63,6;-58,6)
5-14 anos	245,7 (219,2;270,1)	0,7 (0,6;0,8)	119,2 (100,6;138,7)	0,4 (0,3;0,4)	-46,8 (-55,2;-37,2)
70+ anos	53196,6 (48776,9;55833,8)	1257,6 (1153,1;1319,9)	84231,4 (73239,5;90754,4)	643,6 (559,6;693,4)	-48,8 (-53;-45,4)
Padronizada por idade	105603,9 (100300,3;109634,9)	137,8 (127,8;144)	131007 (119134,6;139017,7)	58,1 (52,6;61,8)	-57,8 (-60,4;-55,5)
Toda as idades	105603,9 (100300,3;109634,9)	71 (67,4;73,7)	131007 (119134,6;139017,7)	60,5 (55;64,2)	-14,8 (-21;-9,8)
**B.2.3.1-AVC isquêmico**					
Abaixo de 5	47,4 (34,3;62,7)	0,3 (0,2;0,4)	4,4 (3,3;5,9)	0 (0;0)	-89,8 (-93,7;-83,7)
15-49 anos	1810,7 (1667,6;1970,2)	2,4 (2,2;2,6)	925,6 (846,9;1018,1)	0,8 (0,7;0,9)	-66,1 (-70,5;-60,5)
50-49 anos	12625,4 (11913,9;13321,4)	80,5 (75,9;84,9)	11086,3 (10302,3;11863,9)	27,5 (25,5;29,4)	-65,9 (-69,1;-62,2)
5-14 anos	20,5 (17,4;23,6)	0,1 (0;0,1)	5,6 (4,7;6,6)	0 (0;0)	-70 (-75,7;-62,8)
70+ anos	38079,6 (34747,2;40122,2)	900,2 (821,4;948,5)	61898,9 (53255,8;67163,8)	472,9 (406,9;513,2)	-47,5 (-52,1;-43,6)
Padronizada por idade	52583,6 (48875,7;55110,8)	80 (72,5;84,3)	73920,9 (64818,8;79592,8)	33,9 (29,7;36,6)	-57,6 (-60,9;-54,4)
Toda as idades	52583,6 (48875,7;55110,8)	35,3 (32,8;37)	73920,9 (64818,8;79592,8)	34,1 (29,9;36,7)	-3,4 (-12,1;4,4)
**B.2.3.2-Hemorragia intracerebral**					
Abaixo de 5	196,8 (133,4;285,8)	1,2 (0,8;1,7)	19,7 (13,7;27,7)	0,1 (0,1;0,2)	-89,1 (-93,8;-80,6)
15-49 anos	8947,6 (8497,2;9696,9)	11,7 (11,1;12,7)	4999,6 (4652,9;5309,3)	4,3 (4;4,6)	-62,9 (-67,4;-59,5)
50-49 anos	21158,6 (20191,1;22218,4)	134,9 (128,7;141,6)	20099,5 (19034,2;21157,5)	49,8 (47,2;52,4)	-63,1 (-65,7;-60,4)
5-14 anos	99,6 (87,6;112,2)	0,3 (0,2;0,3)	35,7 (29,7;42,1)	0,1 (0,1;0,1)	-60,7 (-67,9;-52,1)
70+ anos	14134,8 (13036,6;15040,8)	334,1 (308,2;355,6)	18671,4 (16453,6;20277,9)	142,7 (125,7;154,9)	-57,3 (-61,8;-53,1)
Padronizada por idade	44537,3 (42391,2;46721)	49,8 (47,1;52,2)	43825,9 (40717,3;46438,1)	18,6 (17,3;19,8)	-62,6 (-65,3;-59,7)
Toda as idades	44537,3 (42391,2;46721)	29,9 (28,5;31,4)	43825,9 (40717,3;46438,1)	20,2 (18,8;21,4)	-32,4 (-37,5;-27,2)
**B.2.3.3-Hemorragia subaracnóidea**					
Abaixo de 5	171,9 (106,6;232,7)	1 (0,6;1,4)	44,1 (32;60,8)	0,3 (0,2;0,4)	-72 (-83,2;-45,1)
15-49 anos	3794,8 (3298;3999,6)	5 (4,3;5,2)	3385,5 (3176,9;3702)	2,9 (2,8;3,2)	-40,8 (-46;-27,3)
50-49 anos	3408,5 (3110,6;3603,8)	21,7 (19,8;23)	6091,5 (5557,2;6562,1)	15,1 (13,8;16,3)	-30,5 (-36,6;-22,3)
5-14 anos	125,6 (110,2;138,6)	0,4 (0,3;0,4)	78 (65,7;91,2)	0,2 (0,2;0,3)	-32 (-43,4;-18)
70+ anos	982,3 (881,6;1178,5)	23,2 (20,8;27,9)	3661,1 (2977,6;4083,9)	28 (22,7;31,2)	20,5 (-16,5;40,4)
Padronizada por idade	8483 (7668,1;8870,6)	8 (7,3;8,4)	13260,2 (12016,4;14155,9)	5,5 (5;5,9)	-30,3 (-36,2;-23,6)
Toda as idades	8483 (7668,1;8870,6)	5,7 (5,2;6)	13260,2 (12016,4;14155,9)	6,1 (5,5;6,5)	7,4 (-1,4;18,8)

*Fonte: Dados derivados do estudo Global Burden of Disease 2019, Institute for Health Metrics and Evaluation, University of Washington. ^46^*

**Tabela 2-7:** – Número de YLLs e taxas de YLLs padronizadas por idade (por 100 mil) por AVC, AVC isquêmico, hemorragia subaracnóidea e hemorragia intracerebral em 1990 e 2019, com variação percentual das taxas, no Brasil e suas unidades federativas.

Causa de morte e grupo etário	1990		2019		Variação percentual (II 95%)
	Número (II 95%)	Taxa (II 95%)	Número (II 95%)	Taxa (II 95%)	
**B.2.3-AVC**					
Acre	3638,2 (3310,9;3958,3)	2093,3 (1918,9;2257,8)	7523,5 (6798,3;8291,5)	1189,9 (1074,3;1316,2)	-43,2(-49,5;-35,9)
Alagoas	45113 (41108,8;49962,3)	3155,6 (2866,2;3497,6)	54270,7 (47737,5;61452,7)	1686,1 (1482,2;1904)	-46,6(-55,1;-37,1)
Amapá	1871,7 (1693,8;2033,1)	1747,8 (1601,3;1882,8)	6259,1 (5602,4;6893,9)	1145,2 (1018,4;1264,8)	-34,5(-41,4;-27,1)
Amazonas	18486,3 (16733,1;20143,4)	2261,8 (2062,1;2447,6)	31259,9 (27577,8;34979,3)	1063,1 (935,3;1192,8)	-53(-58,6;-46,5)
Bahia	173368,1 (154540,2;193719)	2433,5 (2163,9;2731,4)	207268,2 (174444,1;242726,8)	1281 (1077,7;1498,2)	-47,4(-56,9;-36)
Brasil	2590844,4 (2504589,7;2679788,7)	2778,6 (2659,5;2879,2)	2578166 (2413609,9;2702585,5)	1098,7 (1025,8;1153,7)	-60,5(-62,7;-58,4)
Ceará	82644,5 (70969;95343,2)	1924,7 (1649,6;2235,2)	122875,8 (102778,9;144353,5)	1232,7 (1030,7;1448,8)	-36(-49,2;-19,8)
Distrito Federal	18102,9 (16265,1;20466,9)	2863 (2605,7;3189,8)	23254,6 (20647,9;26147,6)	970,7 (860;1088,3)	-66,1(-70,6;-61,2)
Espírito Santo	49253,6 (47109,2;51447,6)	3243 (3085,3;3392,1)	51706,5 (45154,4;58160,6)	1192,4 (1042,7;1341,7)	-63,2(-67,7;-58,7)
Goiás	73040,8 (63335,6;85932,2)	3229,4 (2820,1;3792)	72966,1 (61930,4;85238,7)	1039,6 (884,3;1207,4)	-67,8(-73,8;-60,8)
Maranhão	72614,6 (60933,2;85134,1)	2510,2 (2113,8;2938,1)	113620,3 (96360,6;135605,8)	1703,6 (1445,6;2028,8)	-32,1(-46,4;-12,5)
Mato Grosso	19638,7 (16988,9;22162,1)	2261,8 (2005,3;2508,2)	32952,9 (29423,3;36884,6)	983 (874,9;1104)	-56,5(-62,7;-49,4)
Mato Grosso do Sul	26812,2 (24991,9;28638,5)	2766 (2577,2;2952,7)	30540,2 (27071,5;34205,2)	1036,9 (917,8;1162,7)	-62,5(-66,8;-57,5)
Minas Gerais	319806,1 (297709,9;348085,7)	3061,2 (2848,1;3318,7)	258108,8 (230977,9;288521,3)	982,5 (879,9;1097,6)	-67,9(-72,1;-63,8)
Pará	59420,4 (52481,8;66484,2)	2749 (2447,2;3053,1)	88399,9 (77815,3;98126,2)	1248 (1088,7;1385,5)	-54,6(-60,7;-47,6)
Paraíba	47314,8 (41644,2;53264,8)	2018,2 (1773,1;2272,7)	53969,6 (46911;61530,8)	1141,1 (993,1;1300,9)	-43,5(-52,6;-32,1)
Paraná	165936,3 (158219,6;173304,8)	3360,2 (3187,9;3518,8)	146319,8 (128976,3;164812,7)	1119,8 (985,9;1255,3)	-66,7(-70,7;-62,6)
Pernambuco	128500,4 (121005,2;135907,7)	2776,5 (2609,3;2936)	137705 (122806;154533,5)	1369,2 (1220,7;1532,8)	-50,7(-56,6;-44,7)
Piaui	38717 (35046;42639,9)	2686,6 (2441,7;2953,8)	46373,2 (40842,1;52052,7)	1229,9 (1082,6;1381,8)	-54,2(-60,2;-47,1)
Rio de Janeiro	356654 (340788,2;372187,5)	3545 (3366,4;3689,2)	257751,3 (231425,8;285548,3)	1160,2 (1041;1285)	-67,3(-70,8;-63,3)
Rio Grande do Norte	28185,2 (25144,4;31483,9)	1719,5 (1525,9;1925,1)	32774,7 (27520,4;38814,4)	842,2 (707,4;996,9)	-51(-59,9;-40,6)
Rio Grande do Sul	177413,9 (168848,5;185603,9)	2676,3 (2534;2802,4)	162756,9 (144795,8;181241,8)	1062,5 (943,6;1183,1)	-60,3(-64,5;-55,8)
Rondônia	11663,2 (9984,6;13075,5)	3402,2 (3073;3718,5)	17514,3 (15097,2;20285,1)	1130,3 (975,8;1308,2)	-66,8(-72;-60,6)
Roraima	1557,3 (1365,4;1731,4)	2508,3 (2278,1;2745,1)	3888,6 (3473,1;4323,7)	1039,6 (924,2;1150,6)	-58,6(-63,8;-53)
Santa Catarina	74479,5 (69924,8;78924,5)	2941,9 (2754,1;3115,6)	70469,1 (62180,6;79237)	891,5 (787,2;997,1)	-69,7(-73,2;-66)
São Paulo	565019,3 (533070,5;599374,7)	2670,9 (2511,6;2833,3)	500606,4 (446874;554035,6)	931,3 (830,3;1030,4)	-65,1(-69;-61,1)
Sergipe	21056,2 (19196,7;23080,6)	2649,1 (2408,6;2894,5)	28150,9 (24094,5;32370,1)	1249,3 (1068,6;1436,4)	-52,8(-60,5;-44,7)
Tocantins	10536,2 (9172,6;11978)	2567,4 (2248;2886,8)	18879,7 (16302,6;21715,4)	1312,8 (1135;1504,7)	-48,9(-57,5;-38,5)
**B.2.3.1-AVC isquêmico**					(;)
Acre	1241,7 (1095,2;1383,9)	981,6 (873,1;1086,6)	2796,4 (2477,3;3173,2)	532 (469,4;602,9)	-45,8(-53,4;-35,6)
Alagoas	18153,8 (15408,7;20916,9)	1484,1 (1266,6;1704,1)	23104,8 (19903,4;26376,3)	775,5 (666,8;885,6)	-47,7(-58,6;-35,5)
Amapá	666,5 (605,1;727,3)	829,8 (747,4;902,6)	2305,2 (2010,4;2562,1)	522,6 (452,5;581,9)	-37(-43,8;-29,6)
Amazonas	6476 (5852,2;7075,5)	1033,9 (933,3;1123,7)	11701,7 (10136,3;13174,3)	468,4 (404,9;529)	-54,7(-60,2;-48,5)
Bahia	65136,9 (55706,7;74484,5)	1040,2 (891,1;1186,3)	85232 (70691;100091,3)	543,1 (450,7;638,6)	-47,8(-59;-33,8)
Brasil	947035,1 (895299,9;992583,8)	1211,1 (1133,8;1268,9)	1079632,1 (982065;1148602,5)	479,7 (435,1;510,8)	-60,4(-63,5;-57,3)
Ceará	32503 (26515,3;39027,4)	836,2 (682;1004,7)	53233,6 (43945,9;63548,2)	548,9 (452,4;654,6)	-34,4(-49,3;-12,5)
Distrito Federal	4730,7 (4219,7;5398,6)	1294 (1172,8;1442,7)	8959,4 (7866,4;10165)	484,9 (422,2;546,7)	-62,5(-67,4;-57)
Espírito Santo	17951 (16791,8;19054,5)	1443,5 (1335,2;1530,5)	21006,8 (18047,5;23863,7)	517,2 (444,6;586,4)	-64,2(-69;-59,3)
Goiás	23231,1 (19865,8;27417,6)	1336,2 (1158,6;1557,9)	27989,3 (23581,2;32754,8)	440,5 (370,5;514,7)	-67(-73,1;-59,9)
Maranhão	25999 (19603,3;32038,4)	1095,2 (848,6;1331,4)	48031,8 (40676,2;56801,9)	763,5 (646,1;902,8)	-30,3(-46,8;-2,9)
Mato Grosso	6760 (5891,9;7661,8)	1065,6 (935,9;1193,3)	12646,5 (11001,1;14370,4)	432,8 (375,9;491,6)	-59,4(-65,2;-52,5)
Mato Grosso do Sul	8498,8 (7759,2;9203,4)	1137,4 (1029,6;1227,4)	11876,8 (10349,4;13471,1)	436,9 (379,4;494,6)	-61,6(-66,2;-56)
Minas Gerais	108699,4 (99915,8;118951,1)	1257,1 (1151,5;1368,4)	105525,7 (91920,7;118346,2)	404,8 (352,7;453,6)	-67,8(-71,9;-63,2)
Pará	23740,3 (20920,8;26550,4)	1352,4 (1192,7;1506,7)	37477,3 (32192,9;42111,1)	593,8 (509,4;667,4)	-56,1(-62,3;-49)
Paraíba	20370,9 (16680,5;24037,9)	906,2 (745,9;1063,7)	22708,5 (19399,8;26237,8)	472,5 (404,1;546,5)	-47,9(-58,3;-33,6)
Paraná	64464,8 (60374,7;68432,4)	1598,9 (1481,9;1695,9)	69504,9 (60465,7;78336,2)	559,2 (486,1;627,3)	-65(-69,4;-60,4)
Pernambuco	50355,8 (45411,8;55130,7)	1224,2 (1104,8;1333,5)	53525 (46384,9;60987,6)	566,7 (492,7;645,2)	-53,7(-60,7;-46,2)
Piaui	14999,3 (12981,2;16969)	1249,7 (1089,5;1408,4)	20827 (17690,4;23559,6)	552,8 (470,1;625,8)	-55,8(-63;-47,4)
Rio de Janeiro	122527,2 (114879,3;129568,4)	1443 (1347,1;1525,5)	101343,1 (89470,7;112513)	460,6 (406,7;511,1)	-68,1(-71,6;-64,3)
Rio Grande do Norte	12068 (10468,4;13932,6)	783,6 (678,3;903)	14096,8 (11589,3;16762)	367,4 (303;437,1)	-53,1(-62,2;-40,7)
Rio Grande do Sul	71869,3 (66775;76379,1)	1270,7 (1174,6;1352,1)	78403,3 (67989,1;88418,6)	508,7 (440,8;573,8)	-60(-64,5;-55,2)
Rondônia	3766,2 (3297,1;4198,3)	1782,2 (1621,8;1942,6)	7001 (5912,5;8182,5)	521,9 (441,4;607,5)	-70,7(-75,5;-65,5)
Roraima	468,7 (419,6;519,8)	1237,8 (1123,4;1350,9)	1442,3 (1258,1;1619,2)	500 (436,7;556,5)	-59,6(-64,9;-53,9)
Santa Catarina	29055,8 (27032,4;31053,7)	1417,3 (1310,5;1520,9)	32271,5 (28229,2;36649,5)	440,8 (384,6;499,4)	-68,9(-72,7;-65)
São Paulo	201067 (186754,2;216302,7)	1168,1 (1074,4;1246,4)	206713,7 (181100,9;230259,8)	399,1 (348,7;444,2)	-65,8(-69,3;-61,8)
Sergipe	8730,4 (7749,7;9702,2)	1292,3 (1151,2;1427,3)	11940,9 (10057,6;13906,4)	570,2 (478;664,6)	-55,9(-63,6;-46,9)
Tocantins	3503,7 (2933,1;4088,3)	1216,2 (1047,7;1386,3)	7967 (6754,7;9233,1)	603,5 (512;697,2)	-50,4(-60,1;-39,2)
**B.2.3.2-Hemorragia intracerebral**					(;)
Acre	1757,7 (1577,9;1961,3)	877 (793,1;970,4)	3337,8 (2971,3;3756)	484,3 (431,3;543,1)	-44,8(-52,6;-35,3)
Alagoas	20756,3 (18072,7;23944,2)	1342,9 (1164,5;1545,8)	23503,2 (20270,5;26917,1)	696,8 (602,7;797,2)	-48,1(-59;-36,1)
Amapá	925,7 (822,9;1016,6)	752,8 (682,5;819,5)	2845,7 (2495,7;3187,9)	469,8 (414,2;525,2)	-37,6(-45,2;-28,4)
Amazonas	9619,8 (8639,1;10610,1)	1039,8 (942,8;1143,7)	14370,5 (12524,1;16340,8)	453,4 (396,4;515,7)	-56,4(-62,3;-49,4)
Bahia	84677,6 (74285,2;96506,6)	1129,1 (987,5;1292,9)	90291,3 (74543,4;107505,2)	549,9 (454,1;654,9)	-51,3(-61,8;-38,9)
Brasil	1303555,9 (1249976,5;1374489,1)	1283,5 (1227,7;1351)	1086463 (1025413,3;1141541,4)	449,2 (423,3;472,5)	-65(-67,6;-62,5)
Ceará	38358,7 (32448,7;45304,9)	858,6 (718;1018,3)	51669 (42481,5;61768,7)	510,3 (419,3;609,2)	-40,6(-54,8;-23,4)
Distrito Federal	10017,7 (8886,2;11645,6)	1273,3 (1137,5;1448,2)	9644,7 (8461,1;10976,3)	339,8 (296,7;385,6)	-73,3(-77,2;-68,4)
Espírito Santo	24966,1 (23680,2;26430,9)	1488,7 (1411,4;1572,5)	22397,8 (19379;25441)	493,6 (428;560,4)	-66,8(-71,4;-62,2)
Goiás	39798,3 (34140,5;47575,2)	1575,2 (1358,2;1885)	32574,2 (27320,6;38124)	438,6 (369,2;511,5)	-72,2(-77,8;-65,5)
Maranhão	34728,9 (27796,6;43087,2)	1107,8 (881;1382,6)	47718,5 (39527,8;58098,6)	697,6 (577,8;850,1)	-37(-52,3;-15,5)
Mato Grosso	9773,4 (8348,4;11190)	971,6 (847,6;1097,2)	14273,3 (12554,1;16136,1)	395,1 (348;447,4)	-59,3(-66;-51,2)
Mato Grosso do Sul	14514,1 (13377,8;15746,3)	1346,4 (1249,4;1458,7)	13450,7 (11797,6;15347,6)	436 (383,3;495,6)	-67,6(-72;-62,7)
Minas Gerais	167435,9 (153866,2;189273,2)	1474,3 (1357,6;1655,4)	108433,6 (96309;122277,7)	407,8 (362,1;459,1)	-72,3(-76,6;-68)
Pará	28737 (25168,4;32327,3)	1183,6 (1047,4;1327)	37239,6 (32252,1;41938)	492,7 (426,7;553,4)	-58,4(-64,9;-51)
Paraíba	19904,5 (16932,5;23582,1)	834,9 (707,5;992,8)	22961,4 (19839,1;26504,6)	491,2 (424,2;567,4)	-41,2(-53,8;-25,7)
Paraná	81687,9 (76990,4;86999,7)	1468,5 (1382,2;1562,7)	54832,8 (47517,7;62195,1)	399,4 (347,3;452,6)	-72,8(-76,5;-68,9)
Pernambuco	65139,7 (60440,4;70286)	1322,6 (1228;1428,4)	63855 (56085;72426,3)	612,4 (539,9;694)	-53,7(-60,1;-46,5)
Piaui	18213,3 (16144,9;20912,9)	1151,7 (1020;1313,5)	18767,4 (16413,5;21459,4)	498,8 (437,3;570,2)	-56,7(-63,9;-48,1)
Rio de Janeiro	189847,8 (178940,3;202216,9)	1738,9 (1643,7;1847,2)	117082,3 (103795,5;131957,4)	517,9 (459,5;584,3)	-70,2(-73,7;-66)
Rio Grande do Norte	12755 (11224,4;14401)	759 (666,5;857,1)	13759,1 (11229,2;16571,9)	351,7 (287,4;423,3)	-53,7(-63,2;-42,8)
Rio Grande do Sul	86550,1 (81293,4;91677,5)	1177,9 (1106,3;1248,7)	63875,4 (55424,1;72522)	412,8 (357,6;470,1)	-65(-69,4;-60)
Rondônia	6250,9 (5307;7125,5)	1379,4 (1218,2;1535,9)	7486,2 (6402,6;8872,1)	445,2 (380,7;522,7)	-67,7(-73,5;-60)
Roraima	841,1 (724,7;946,2)	1067,6 (952,2;1181,9)	1728 (1517,2;1949,5)	399,6 (352,9;448,2)	-62,6(-68;-56,5)
Santa Catarina	36527,9 (33998,7;39097,7)	1274,8 (1186,5;1365,9)	27210,5 (23570;31005,4)	322,1 (279,3;365,9)	-74,7(-78;-71)
São Paulo	284456,9 (263629,8;309048,7)	1216,8 (1131,4;1310,4)	202992,4 (179780,6;228623,2)	366 (324,4;411,6)	-69,9(-73,9;-65,7)
Sergipe	9901 (8860,4;11012,8)	1129,8 (1004,7;1258,2)	12224,9 (10331,9;14328,5)	519,8 (440;608,4)	-54(-62,4;-44,6)
Tocantins	5412,9 (4570,5;6344,5)	1100,5 (928;1289,6)	7937,7 (6755,7;9253,1)	526,6 (449,1;612,8)	-52,1(-62,5;-39,9)
**B.2.3.3-Hemorragia subaracnóidea**					(;)
Acre	638,8 (553,3;736,4)	234,8 (205;268)	1389,3 (1226,4;1577,6)	173,7 (153,4;196,9)	-26(-38,1;-10,9)
Alagoas	6202,9 (5172,1;7328,1)	328,6 (273,4;385,4)	7662,7 (6534,3;8941,3)	213,9 (182,7;248,4)	-34,9(-49;-16,4)
Amapá	279,6 (239,4;323,5)	165,2 (145;192,4)	1108,2 (988,3;1270,6)	152,8 (136;175,3)	-7,5(-21;8,9)
Amazonas	2390,6 (2083,9;2722,8)	188,1 (166;215,7)	5187,7 (4523,3;6016,3)	141,4 (123,3;163,9)	-24,9(-37,3;-10,1)
Bahia	23553,6 (20282,4;27063,9)	264,2 (227,7;306,7)	31744,9 (25977,8;38214,4)	188 (154,6;225,7)	-28,8(-44,6;-9)
Brasil	340253,3 (301621,6;356874,5)	284 (253,8;297,2)	412071 (384850,3;438730)	169,8 (158,3;180,8)	-40,2(-44,9;-32,6)
Ceará	11782,8 (9708;14149,8)	229,9 (189,8;273,3)	17973,3 (14699,9;22114)	173,5 (142;212,6)	-24,5(-41,6;1,1)
Distrito Federal	3354,5 (2951,9;3778,2)	295,7 (260,6;333,6)	4650,5 (3988,1;5365)	145,9 (124,6;167,3)	-50,6(-58,4;-41)
Espírito Santo	6336,6 (5170,6;6918,2)	310,8 (254,6;338,4)	8301,9 (7019,7;9774,7)	181,6 (154,2;212,4)	-41,6(-50,6;-29,5)
Goiás	10011,5 (8705,3;11624,9)	318 (275,8;370)	12402,6 (10301,2;14840,8)	160,5 (133,8;190,7)	-49,5(-59,9;-37,5)
Maranhão	11886,7 (8770,9;15728,3)	307,3 (228,6;399,9)	17869,9 (14341;21910,3)	242,5 (195,1;298,1)	-21,1(-43,6;9,7)
Mato Grosso	3105,3 (2559,7;3680)	224,6 (190,2;262,6)	6033,1 (5267,1;6920,4)	155,1 (135,6;178,1)	-30,9(-43,3;-14,6)
Mato Grosso do Sul	3799,2 (3420,5;4170,8)	282,1 (254,3;309,2)	5212,8 (4497,3;5959,5)	164 (141,9;187,1)	-41,9(-50,3;-31,9)
Minas Gerais	43670,8 (37136,2;47942,6)	329,8 (283,9;362)	44149,6 (38291,3;50524,6)	170 (147,6;194)	-48,5(-55,6;-39,3)
Pará	6943,1 (5981,5;7928,9)	213 (184,9;245)	13683 (11958,7;15648,5)	161,6 (141,3;184,9)	-24,1(-37;-8)
Paraíba	7039,5 (6024;8166,2)	277,2 (236,8;324)	8299,7 (7006;9682)	177,4 (149,9;206,9)	-36(-49,6;-19,4)
Paraná	19783,6 (17429,1;21454,1)	292,8 (259,2;317,4)	21982,1 (18625,7;25543,5)	161,2 (137,6;187,1)	-44,9(-53,3;-35,9)
Pernambuco	13004,9 (11647,3;14757,9)	229,7 (205,5;263,4)	20325 (17670,8;23381)	190,1 (165,4;218,8)	-17,3(-31;-1,2)
Piaui	5504,3 (4717;6353,5)	285,2 (246,2;325,6)	6778,8 (5793,9;7860,2)	178,2 (152,6;206,3)	-37,5(-49,6;-22,1)
Rio de Janeiro	44279 (33962,9;48808,5)	363,1 (283,3;399,4)	39325,9 (34177,3;44894,3)	181,7 (158,8;207,1)	-49,9(-57,8;-35)
Rio Grande do Norte	3362,3 (2890,3;4087,5)	176,8 (150,3;218,8)	4918,8 (3993,1;6328,9)	123,1 (100;157,3)	-30,4(-45,1;-11,2)
Rio Grande do Sul	18994,6 (17368,3;20855,9)	227,8 (208,6;250,4)	20478,2 (17663,2;23770,2)	141 (122,3;162,6)	-38,1(-46,9;-27,7)
Rondônia	1646,1 (1305,3;1948,2)	240,6 (199,8;277,8)	3027,1 (2591,3;3565,5)	163,2 (140,1;191,8)	-32,2(-45;-13,4)
Roraima	247,5 (203,2;289,8)	202,9 (172,7;233,4)	718,3 (628,4;855,1)	140 (122,8;165,9)	-31(-42,6;-16,3)
Santa Catarina	8895,8 (8096,2;9718,4)	249,7 (227,1;273,2)	10987,1 (9521;12815,9)	128,6 (111,7;149,5)	-48,5(-55,9;-39)
São Paulo	79495,5 (68778,8;86635,1)	286 (251,5;311,9)	90900,3 (79393,9;103394,2)	166,1 (145,3;188,7)	-41,9(-49,9;-31,7)
Sergipe	2424,8 (2108,6;2782,6)	227 (197;261,6)	3985,1 (3284,1;4788,7)	159,3 (131,8;191,9)	-29,8(-44,6;-11,8)
Tocantins	1619,6 (1328,1;1906,4)	250,7 (210,1;292,7)	2975 (2491,9;3503)	182,6 (153,7;215,9)	-27,1(-41,6;-7,4)


*Fonte: Dados derivados do estudo Global Burden of Disease 2019, Institute for Health Metrics and Evaluation, University of Washington. ^46^*

**Tabela 2-8:** – Número de YLLs e taxas de YLLs padronizadas por idade (por 100 mil) por AVC, AVC isquêmico, hemorragia subaracnóidea e hemorragia intracerebral em 1990 e 2019, com variação percentual das taxas, no Brasil, de acordo com o grupo etário.

Causa de morte e grupo etário	1990		2019		Percent change (95% UI)
	Número (II 95%)	Taxa (II 95%)	Número (II 95%)	Taxa (II 95%)	
**B.2.3-AVC**					
Abaixo de 5	36373,4 (26471,3;49662)	214,7 (156,3;293,2)	5970,3 (4335,4;8153,1)	38,5 (28;52,6)	-82,1 (-89,2;-70)
15-49 anos	716076,9 (689520,8;745933,4)	934,3 (899,6;973,2)	448555,9 (425616,6;472166,9)	388,4 (368,5;408,8)	-58,4 (-61,2;-55,7)
50-49 anos	1084139,7 (1044839,3;1128620,8)	6910,8 (6660,3;7194,3)	1067928,7 (1013004,9;1118810)	2647,1 (2511;2773,2)	-61,7 (-64,3;-59,3)
5-14 anos	19376,5 (17280,8;21317,5)	54,8 (48,9;60,3)	9330,4 (7859,9;10858,3)	28,9 (24,4;33,7)	-47,2 (-55,7;-37,6)
70+ anos	734877,8 (682090,6;768788,5)	17372,7 (16124,8;18174,4)	1046380,8 (932190,5;1117926,7)	7994,7 (7122,2;8541,3)	-54 (-57,4;-51)
Padronizada por idade	2590844,4 (2504589,7;2679788,7)	2778,6 (2659,5;2879,2)	2578166 (2413609,9;2702585,5)	1098,7 (1025,8;1153,7)	-60,5 (-62,7;-58,4)
Toda as idades	2590844,4 (2504589,7;2679788,7)	1740,7 (1682,8;1800,5)	2578166 (2413609,9;2702585,5)	1189,9 (1114;1247,4)	-31,6 (-35,8;-28)
**B.2.3.1-AVC isquêmico**					
Abaixo de 5	4135,2 (2990,3;5467,5)	24,4 (17,7;32,3)	384,4 (283,6;515,9)	2,5 (1,8;3,3)	-89,8 (-93,7;-83,7)
15-49 anos	86173,7 (79496,8;93439,3)	112,4 (103,7;121,9)	43383,5 (39668,9;47636,4)	37,6 (34,3;41,2)	-66,6 (-71,1;-61,1)
50-49 anos	349029,3 (329328,7;368931,8)	2224,9 (2099,3;2351,7)	299595,2 (278841,5;320496,5)	742,6 (691,2;794,4)	-66,6 (-69,8;-62,9)
5-14 anos	1620,3 (1376,7;1865,2)	4,6 (3,9;5,3)	440,2 (365;521,5)	1,4 (1,1;1,6)	-70,2 (-75,9;-63,1)
70+ anos	506076,5 (466579,2;530504)	11963,8 (11030,1;12541,3)	735828,9 (648142,4;789381,6)	5621,9 (4952;6031,1)	-53 (-56,8;-49,5)
Padronizada por idade	947035,1 (895299,9;992583,8)	1211,1 (1133,8;1268,9)	1079632,1 (982065;1148602,5)	479,7 (435,1;510,8)	-60,4 (-63,5;-57,3)
Toda as idades	947035,1 (895299,9;992583,8)	636,3 (601,5;666,9)	1079632,1 (982065;1148602,5)	498,3 (453,3;530,1)	-21,7 (-28,4;-15,1)
**B.2.3.2-Hemorragia intracerebral**					
Abaixo de 5	17235,2 (11675,5;25057,4)	101,8 (68,9;147,9)	1730,8 (1199,6;2437,8)	11,2 (7,7;15,7)	-89 (-93,8;-80,6)
15-49 anos	436522,1 (414806,9;474485,7)	569,5 (541,2;619,1)	238357,1 (221826,9;252852,4)	206,4 (192,1;218,9)	-63,8 (-68,4;-60,4)
50-49 anos	627767,2 (600065,4;659989,2)	4001,7 (3825,1;4207,1)	582538,8 (551911,4;614231,7)	1444 (1368;1522,5)	-63,9 (-66,5;-61,2)
5-14 anos	7857,3 (6901,7;8868,3)	22,2 (19,5;25,1)	2786,3 (2314,7;3296,6)	8,6 (7,2;10,2)	-61,1 (-68,2;-52,6)
70+ anos	214174,1 (199342,6;227529)	5063,1 (4712,5;5378,8)	261050 (235043,8;282383)	1994,5 (1795,8;2157,5)	-60,6 (-64,5;-56,9)
Padronizada por idade	1303555,9 (1249976,5;1374489,1)	1283,5 (1227,7;1351)	1086463 (1025413,3;1141541,4)	449,2 (423,3;472,5)	-65 (-67,6;-62,5)
Toda as idades	1303555,9 (1249976,5;1374489,1)	875,8 (839,8;923,5)	1086463 (1025413,3;1141541,4)	501,4 (473,3;526,9)	-42,7 (-47,2;-38,7)
**B.2.3.3-Hemorragia subaracnóidea**					
Abaixo de 5	15002,9 (9308;20320,5)	88,6 (55;120)	3855,1 (2796,2;5324,8)	24,9 (18;34,4)	-71,9 (-83,2;-44,9)
15-49 anos	193381,1 (168039,5;203641,4)	252,3 (219,2;265,7)	166815,3 (156642,9;182531,3)	144,4 (135,6;158,1)	-42,8 (-47,7;-29,9)
50-49 anos	107343,3 (97585,4;113734,5)	684,3 (622,1;725)	185794,7 (169749,3;200067,8)	460,5 (420,8;495,9)	-32,7 (-38,4;-24,3)
5-14 anos	9898,9 (8666,6;10917,5)	28 (24,5;30,9)	6104 (5139,8;7158,2)	18,9 (15,9;22,2)	-32,4 (-43,7;-18,4)
70+ anos	14627,2 (13237,5;17395,9)	345,8 (312,9;411,2)	49501,9 (40527,8;55106,2)	378,2 (309,6;421)	9,4 (-23,2;27,2)
Padronizada por idade	340253,3 (301621,6;356874,5)	284 (253,8;297,2)	412071 (384850,3;438730)	169,8 (158,3;180,8)	-40,2 (-44,9;-32,6)
Toda as idades	340253,3 (301621,6;356874,5)	228,6 (202,7;239,8)	412071 (384850,3;438730)	190,2 (177,6;202,5)	-16,8 (-23,5;-5,2)

*Fonte: Dados derivados do estudo Global Burden of Disease 2019, Institute for Health Metrics and Evaluation, University of Washington.* ^*46*^

**Tabela 2-9:** – Número de YLDs e taxas de YLDs padronizadas por idade (por 100 mil) por AVC, AVC isquêmico, hemorragia subaracnóidea ehemorragia intracerebral em 1990 e 2019, com variação percentual das taxas, no Brasil e suas unidades federativas,

Causa de morte e localização	1990		2019		Variação percentual (II 95%)
	Número (II 95%)	Taxa (II 95%)	Número (II 95%)	Taxa (II 95%)	
**B,2,3-AVC**					
Acre	315,6 (232,9;404,1)	159,2 (118;201,2)	825,1 (606,5;1042,6)	120,9 (88,8;153,7)	-24(-28,3;-19,3)
Alagoas	2754,3 (2009,9;3460,9)	186,9 (136,7;237,4)	4490,7 (3288,4;5698,7)	136,8 (100,5;172,9)	-26,8(-31,5;-22)
Amapá	191,1 (139,4;243,8)	154,5 (112,9;196,4)	708,2 (515,4;897,8)	119 (86,8;151,2)	-23(-27,3;-18,1)
Amazonas	1623 (1187,4;2072,9)	171,2 (126,4;216,2)	3713,3 (2706,4;4687,6)	116,6 (84,2;147,1)	-31,9(-36,2;-27,3)
Bahia	12715,9 (9333,4;16054,2)	171,5 (125,5;218,1)	19723,7 (14433,8;24962,7)	121,4 (88,8;153,9)	-29,2(-33,3;-24,7)
Brasil	175647 (130408,3;222000,4)	180,4 (133,2;228,8)	283557,2 (208451,8;357622,1)	120,9 (88,7;152,6)	-33(-34,8;-31,1)
Ceará	6314,9 (4619,7;8000,2)	144,1 (104,7;182,6)	12030,7 (8775,1;15287,7)	119,9 (87,3;152,6)	-16,8(-21,8;-11,3)
Distrito Federal	1358,4 (1002,7;1722)	182,6 (135,3;232)	3400,5 (2488,7;4353,9)	124,4 (90,4;159,5)	-31,9(-36,4;-27,5)
Espírito Santo	3144,4 (2307,5;3997,4)	193,5 (140,7;246)	5509,3 (4060,9;6993,3)	126,9 (93,2;161,1)	-34,4(-38,7;-30,1)
Goiás	4211,8 (3090,3;5372,3)	173,5 (126,6;222)	8042,5 (5907,7;10184,9)	113 (82,9;143,8)	-34,8(-39,2;-30,8)
Maranhão	4463,2 (3280,2;5679,7)	157,7 (115,9;201)	8115,7 (5942,9;10311,5)	117,5 (86;148,7)	-25,5(-31,1;-19)
Mato Grosso	1584,1 (1154,8;2003,3)	160,2 (117;202,9)	3944,7 (2881,2;5013,1)	114,7 (84;145,6)	-28,4(-33,5;-23,4)
Mato Grosso do Sul	1798 (1331,6;2289,7)	172,1 (127,6;217,7)	3590,7 (2643,5;4545,9)	121,1 (89,7;153,8)	-29,6(-34,4;-25,2)
Minas Gerais	19927,3 (14714,4;25139,3)	185,2 (135,1;232,7)	30355,4 (22430,4;38566,6)	117,6 (86,8;149,7)	-36,5(-40,5;-32)
Pará	4349,2 (3209,4;5487,1)	178,9 (130,2;227)	8858,9 (6493,4;11120,3)	118,3 (87;150)	-33,9(-38;-29,6)
Paraíba	3654,6 (2656,5;4661,7)	151,8 (110,6;193,6)	5438,7 (4011,3;6852,3)	116,1 (85,5;145,9)	-23,5(-28,4;-18,1)
Paraná	10907,4 (7979;13836)	204,2 (149,8;259,5)	16967,1 (12436,9;21666,3)	129,6 (95,5;165,4)	-36,5(-40,6;-32,1)
Pernambuco	8704,7 (6396,6;11162)	179,1 (131,2;230,4)	12587,5 (9228,9;16057,8)	124,4 (91,2;158,4)	-30,5(-34,8;-25,9)
Piaui	2463,3 (1807,4;3116,9)	158,1 (115,7;201,3)	4431,3 (3229,6;5622,5)	117,8 (85,9;149,4)	-25,5(-30,1;-20,6)
Rio de Janeiro	20883 (15402,8;26611,4)	204,7 (151,8;260,7)	27912,2 (20540,5;35371,1)	128,1 (94,1;162,2)	-37,4(-41,5;-32,9)
Rio Grande do Norte	2501,1 (1834,2;3194,4)	146,5 (107,4;186,3)	4227,7 (3103,5;5405,8)	108,7 (79,8;138,8)	-25,8(-30,3;-21,4)
Rio Grande do Sul	13338,6 (9727,6;16924)	191,3 (140,5;242,4)	19074,6 (13941,1;24256,3)	128,3 (94;162,9)	-33(-37,3;-28,8)
Rondônia	859,9 (621,9;1087)	192,8 (141,5;246,6)	1890,5 (1368;2423,8)	116,7 (84,3;148,8)	-39,5(-43,8;-35,2)
Roraima	132,7 (96,3;169,1)	159,1 (117,9;202,8)	474,1 (344,7;602,1)	111,9 (81,1;141,8)	-29,6(-34;-24,8)
Santa Catarina	5369,6 (3950,9;6821,2)	192,6 (140,3;244,3)	9505,9 (6956,5;12073,4)	118,1 (86,4;150,3)	-38,7(-42,9;-34,4)
São Paulo	39775,5 (29243,7;50394,2)	180,5 (132,3;225,6)	63114,8 (45994,9;80990,2)	118,2 (86,7;151)	-34,5(-38,4;-30,2)
Sergipe	1556 (1134,5;1963,8)	177 (130;222,6)	2917,1 (2147,5;3706)	126,9 (93,1;161,1)	-28,3(-32,9;-24,1)
Tocantins	749,2 (545,2;950,1)	157,4 (114,9;199,7)	1706,2 (1240,4;2167,6)	114,7 (82,9;145,9)	-27,1(-31,7;-21,9)
**B,2,3,1-AVC isquêmico**					***(;)***
Acre	203,3 (145;263,7)	108,6 (77,6;140,6)	533,3 (382,4;685,6)	81,8 (59,2;105,5)	-24,7(-30,2;-18,4)
Alagoas	1867,4 (1326,7;2404,7)	130,8 (92,5;168,9)	3026,7 (2167,3;3859,2)	94,3 (68;121,7)	-27,9(-33,9;-21,5)
Amapá	121,4 (87,9;157,1)	104,3 (75,8;134,5)	451,6 (323,1;583,8)	80,5 (57,6;102,8)	-22,9(-28,5;-16,1)
Amazonas	1026,6 (733,4;1328)	115,4 (83,5;149,6)	2372,4 (1692,6;3054,5)	77,9 (56,5;100,8)	-32,5(-38,2;-26)
Bahia	8358,5 (5907,2;10899,7)	115,6 (82,5;150,5)	13021,4 (9286,5;16835)	81,1 (57,7;104,9)	-29,9(-35,1;-24)
Brasil	114029,9 (82236,3;147025)	122,2 (88;156,8)	188474,4 (134840,6;241680,6)	81,2 (58,3;104)	-33,5(-35,9;-31)
Ceará	4119,6 (2917,8;5309,2)	95,6 (67,7;124,3)	7982,2 (5730,1;10296)	80,3 (57,6;103,6)	-16(-22,6;-8,9)
Distrito Federal	806,4 (570,5;1047,1)	121,6 (86,5;157,4)	2191,9 (1539,4;2856,1)	84,2 (59,7;110)	-30,7(-36,8;-24,3)
Espírito Santo	2031,2 (1453,3;2615,8)	131,1 (93,4;168,8)	3649,1 (2606,6;4719,9)	85,3 (60,9;110,5)	-34,9(-40,2;-29,5)
Goiás	2635,3 (1878,6;3385,1)	115,6 (83;149,8)	5236,5 (3763,7;6709,3)	75,4 (53,8;97,2)	-34,8(-40,4;-29,2)
Maranhão	2973,5 (2130,2;3851,3)	108,3 (77,7;141,6)	5283,4 (3803,4;6818,8)	77,8 (56,2;100,3)	-28,2(-35,4;-19,5)
Mato Grosso	997,1 (707;1288,4)	109,7 (78;141,4)	2578,9 (1824,5;3344,9)	77,7 (55,5;100,7)	-29,2(-35,5;-22,2)
Mato Grosso do Sul	1118,4 (811,3;1444,4)	113,9 (81,9;146,4)	2350,4 (1699,6;3034)	80,8 (58,3;104,2)	-29,1(-35,7;-22,9)
Minas Gerais	12831,1 (9202,7;16648,4)	124,4 (89,1;161,7)	20194 (14245,6;25876,1)	78,4 (55,4;100,7)	-37(-42,4;-31,2)
Pará	2842,7 (2038,8;3656,9)	123,4 (88,1;159,1)	5840 (4174,8;7513,5)	80,8 (57,8;103,8)	-34,5(-39,9;-28,5)
Paraíba	2447,1 (1739,1;3198,3)	101,8 (72,6;133,1)	3526,2 (2527;4564,1)	75,2 (54;97,3)	-26,1(-32,7;-19,2)
Paraná	7155,3 (5075,6;9228,7)	141,8 (99,8;184,4)	11638,4 (8284,6;14990,1)	89,8 (64;116,2)	-36,7(-42,2;-30,9)
Pernambuco	5723,7 (4088,5;7503,4)	120,7 (86,7;157,3)	8240,2 (5882,1;10590)	82,7 (59,1;106,8)	-31,4(-36,8;-25,1)
Piaui	1603,7 (1141;2086,3)	106,5 (75,5;139,2)	2968,6 (2131,1;3859,3)	79,2 (57;103)	-25,7(-31,8;-18,7)
Rio de Janeiro	13465,6 (9762,2;17325,1)	137,6 (99,8;177,1)	18411,2 (13185;23879,7)	84,6 (60,4;109,4)	-38,5(-43,9;-33,1)
Rio Grande do Norte	1649,9 (1192,7;2141,4)	97,6 (70,8;127)	2751,9 (1978,8;3570,7)	71,4 (51,5;92,5)	-26,8(-32,2;-21,2)
Rio Grande do Sul	8764,4 (6296,6;11278,3)	131 (94,6;167,5)	13036 (9252,5;16922,7)	87,1 (62;112,8)	-33,5(-38,6;-28,1)
Rondônia	533,7 (375;691,7)	135,9 (96,9;177,6)	1240,2 (865,9;1593,7)	79,6 (55,7;102,9)	-41,4(-46,6;-36)
Roraima	81,3 (57,6;105,5)	109 (79,2;140,9)	308,1 (218,6;395,9)	77,2 (54,4;99,9)	-29,2(-34,9;-22,6)
Santa Catarina	3412,5 (2447,8;4395,7)	130,3 (93,1;169,5)	6305,7 (4518,2;8157)	79,7 (57;103)	-38,8(-44,7;-33,1)
São Paulo	25756,5 (18589,5;33126,7)	122,8 (89;158)	42288,6 (30369,4;54894,6)	79,9 (57,1;103,6)	-34,9(-40,2;-29,3)
Sergipe	1032 (735,6;1325,6)	121 (85,9;156,1)	1936,1 (1385,7;2511,1)	86,1 (61,6;111,4)	-28,8(-34,9;-23,1)
Tocantins	471,9 (336;606,2)	106 (75,9;137,7)	1111,6 (790,3;1438,5)	76,6 (54,3;98,8)	-27,7(-33,9;-20,4)
**B,2,3,2-Hemorragia intracerebral**					***(;)***
Acre	81,3 (57,8;105,6)	37,2 (26,8;48,1)	205 (147,8;267,5)	28 (20,2;36,5)	-24,7(-32,6;-16,8)
Alagoas	666 (479,2;863,2)	42,6 (30,7;55,1)	1072,4 (773,5;1397,4)	31,4 (22,6;40,8)	-26,3(-33,2;-18,6)
Amapá	49,7 (35,7;64,6)	36,5 (26,6;46,8)	177,4 (127,5;229,8)	27,2 (19,7;35,4)	-25,4(-32,1;-17,3)
Amazonas	442,4 (314,1;573,9)	42,5 (30,3;54,5)	944,7 (676,1;1221,9)	27,8 (19,8;36,1)	-34,6(-41;-27,6)
Bahia	3261 (2355,6;4233,4)	42,1 (30,3;54,4)	4796,7 (3456,7;6283,3)	29 (20,8;38,1)	-31(-38;-23,9)
Brasil	46180,6 (33564,1;59463,8)	44,3 (32,3;56,6)	67702,4 (49062,3;87760,6)	28,5 (20,7;36,9)	-35,7(-38,8;-32,2)
Ceará	1560,2 (1120,4;2029,6)	34,5 (24,7;44,8)	2863,7 (2053,1;3744,4)	28,2 (20,2;36,9)	-18,3(-26,3;-9,3)
Distrito Federal	398,7 (288,3;520,4)	46,4 (33,8;59,9)	832,9 (596;1089,9)	28,6 (20,5;37,5)	-38,2(-44,6;-31,4)
Espírito Santo	846 (606,7;1104,9)	48,3 (34,4;62,6)	1342,8 (974,8;1759,2)	30,3 (22,2;39,6)	-37,2(-44;-29,9)
Goiás	1186,2 (840,5;1549,1)	44,6 (32,1;58,1)	1980,3 (1412,5;2581,1)	27 (19,4;35,1)	-39,5(-45,7;-32,8)
Maranhão	1061,3 (763,1;1378,4)	35,4 (25,6;46,3)	1993,1 (1432,7;2590,5)	28 (20,1;36,4)	-20,8(-28,9;-12,2)
Mato Grosso	422,2 (301,3;553,9)	37,5 (27,2;49,5)	948,5 (689,3;1237,3)	26,2 (19,1;34,2)	-30,1(-36,6;-23,1)
Mato Grosso do Sul	510,5 (362,3;665,6)	44,6 (31,9;58,2)	887,3 (629,4;1156,2)	29,2 (20,8;37,9)	-34,6(-41,3;-27)
Minas Gerais	5358,3 (3909,3;6943,6)	46,5 (34,1;60)	7206,4 (5120,7;9398,5)	27,9 (19,9;36,4)	-40(-46,2;-33)
Pará	1117,7 (800,9;1461,5)	42,1 (30,4;54,7)	2108 (1507,6;2730,8)	26,5 (19,1;34)	-37,1(-43,3;-30,6)
Paraíba	863,4 (616,9;1116,4)	35,5 (25,5;46)	1368,4 (982,5;1785,8)	29,3 (21;38,1)	-17,6(-26,4;-8)
Paraná	2855,1 (2053,2;3711,4)	48,5 (35,1;63,2)	3796,4 (2709;4981,8)	28,6 (20,5;37,4)	-41(-47,5;-34,3)
Pernambuco	2285,5 (1644,5;2972,6)	45,1 (32,6;58,6)	3170,9 (2293,2;4123,8)	30,6 (22,1;39,8)	-32,1(-38,3;-25,3)
Piaui	617,3 (443,9;801,9)	37,4 (26,9;48,8)	1020,9 (725,4;1325,9)	27 (19,3;35,1)	-27,8(-34,9;-20,7)
Rio de Janeiro	5769,1 (4169,7;7533,4)	52,8 (38,4;68,4)	7054,1 (5057,4;9121,2)	32,4 (23,3;41,9)	-38,6(-45;-31)
Rio Grande do Norte	613,5 (442,4;798,3)	35,2 (25,2;45,7)	1025,4 (733,4;1317,3)	26,1 (18,5;33,5)	-25,9(-33,7;-17,2)
Rio Grande do Sul	3471,6 (2512,5;4482,4)	46,5 (33,7;60,1)	4412,9 (3174,2;5808,1)	30 (21,6;39,5)	-35,4(-41,6;-28,5)
Rondônia	242 (171,6;314)	44,1 (31,5;56,7)	453,7 (325,9;593)	26,4 (19;34,3)	-40,1(-46,2;-34,2)
Roraima	37,2 (26,4;48,9)	37,7 (27,2;49,3)	113,7 (80,6;149,1)	24,4 (17,6;31,7)	-35,2(-41,5;-27,8)
Santa Catarina	1479,9 (1065,9;1929,2)	48,2 (34,7;63,2)	2246,6 (1606,6;2960)	27,3 (19,6;35,9)	-43,3(-48,9;-36,8)
São Paulo	10391,4 (7440,7;13527)	43,7 (31,6;56,5)	14560,9 (10443,5;18974,8)	27 (19,4;35,2)	-38,1(-44,4;-30,6)
Sergipe	393,6 (287,3;508,3)	42,5 (31,1;54,9)	705,9 (509,7;909,4)	29,7 (21,5;38,1)	-30,3(-36,9;-23,2)
Tocantins	199,6 (142,4;259,6)	37,9 (27,3;48,7)	413,4 (296,3;532,6)	26,8 (19,2;34,5)	-29,1(-36,8;-20,8)
**B,2,3,3-Hemorragia subaracnóidea**					***(;)***
Acre	31,1 (21,8;41,5)	13,3 (9,3;17,8)	86,9 (60,8;117,1)	11,1 (7,8;15)	-16,4(-26,3;-5)
Alagoas	220,9 (153;298,4)	13,6 (9,4;18,4)	391,6 (277;521,1)	11,1 (7,8;14,8)	-18,3(-29,1;-5,6)
Amapá	20 (14;27,2)	13,6 (9,7;18,4)	79,2 (56,1;108,1)	11,3 (8;15,2)	-17,2(-27,8;-6,2)
Amazonas	154 (108,4;211,1)	13,3 (9,4;18,1)	396,2 (280;541,1)	10,9 (7,8;14,8)	-17,5(-27,3;-6,1)
Bahia	1096,5 (769,6;1497,5)	13,8 (9,7;18,7)	1905,6 (1334,1;2552,4)	11,3 (7,9;15,1)	-18(-28,8;-6,8)
Brasil	15436,6 (10801,8;20590,3)	13,9 (9,7;18,6)	27380,3 (19490,9;36579,7)	11,2 (7,9;15)	-19,5(-23,7;-15,4)
Ceará	635,1 (448,2;859,3)	13,9 (9,8;18,9)	1184,8 (841,8;1609,4)	11,4 (8,1;15,4)	-17,8(-28;-5)
Distrito Federal	153,4 (106,7;206,6)	14,6 (10,2;19,6)	375,8 (263;514,2)	11,5 (8,1;15,6)	-21,5(-31,6;-10,1)
Espírito Santo	267,2 (187,3;364,6)	14,2 (9,8;19)	517,4 (366;694,2)	11,3 (8;15,1)	-20,3(-30,2;-9,5)
Goiás	390,3 (275,4;527,4)	13,3 (9,5;17,9)	825,7 (573,1;1116,1)	10,7 (7,5;14,4)	-19,8(-29,7;-8,2)
Maranhão	428,5 (295,9;580,8)	14 (9,6;18,9)	839,1 (587,1;1128,2)	11,6 (8,2;15,6)	-16,6(-27,6;-4,6)
Mato Grosso	164,7 (113,5;223,8)	13,1 (9;17,8)	417,3 (290,3;562,3)	10,8 (7,6;14,5)	-17,2(-27,2;-6,4)
Mato Grosso do Sul	169,1 (116,8;228,9)	13,5 (9,3;18,1)	352,9 (245,4;473,9)	11,1 (7,7;14,9)	-17,5(-27,2;-5,8)
Minas Gerais	1738 (1220,8;2333,7)	14,3 (10,1;19,2)	2955 (2086,3;3961,8)	11,3 (8;15,1)	-21,2(-31,6;-10,1)
Pará	388,8 (271,9;523,7)	13,4 (9,3;17,9)	911 (642,8;1240,9)	11 (7,7;14,9)	-18,2(-28,5;-6,6)
Paraíba	344,1 (240,1;462,5)	14,5 (10,1;19,4)	544,1 (387,2;728,3)	11,6 (8,2;15,5)	-20,1(-30,1;-9,6)
Paraná	897 (622,7;1212,7)	13,9 (9,7;18,6)	1532,3 (1056,3;2071,6)	11,2 (7,8;15,3)	-19,2(-29,7;-8,6)
Pernambuco	695,5 (480,5;944,5)	13,3 (9,3;18)	1176,4 (816,4;1591,5)	11 (7,7;14,9)	-17,1(-27,7;-4,5)
Piaui	242,4 (170,3;330,5)	14,3 (9,9;19,5)	441,9 (307,3;600,4)	11,6 (8,1;15,8)	-18,5(-28,9;-7,2)
Rio de Janeiro	1648,3 (1148,6;2261,6)	14,2 (10;19,5)	2446,9 (1721,6;3330,8)	11,1 (7,8;15,2)	-21,8(-32,2;-9,4)
Rio Grande do Norte	237,7 (168,4;319)	13,7 (9,6;18,6)	450,4 (318,6;614)	11,2 (7,9;15,3)	-18,3(-28,5;-6,6)
Rio Grande do Sul	1102,7 (770,5;1503,8)	13,8 (9,7;18,8)	1625,7 (1140,6;2203,7)	11,1 (7,8;15)	-19,7(-29,5;-7,9)
Rondônia	84,2 (57,8;114,5)	12,8 (8,9;17,2)	196,6 (139,1;265,5)	10,7 (7,5;14,2)	-16,4(-26,8;-4,3)
Roraima	14,2 (9,9;19,4)	12,3 (8,7;16,6)	52,3 (36,6;71,7)	10,3 (7,3;14)	-16,3(-26,3;-5,7)
Santa Catarina	477,1 (333,1;650)	14 (9,9;18,9)	953,6 (666,5;1286,3)	11,1 (7,7;14,8)	-21,1(-30,6;-9,4)
São Paulo	3627,6 (2524,6;4868,5)	14 (9,7;18,8)	6265,3 (4404,9;8442,8)	11,3 (8;15,2)	-19,2(-29,3;-7,5)
Sergipe	130,4 (92,3;177)	13,5 (9,5;18,6)	275,1 (193,3;373,7)	11,1 (7,8;15)	-18,1(-28,4;-6,7)
Tocantins	77,8 (54,6;106,2)	13,6 (9,5;18,5)	181,2 (127,9;246,5)	11,3 (8;15,3)	-16,9(-26,7;-6,3)

*Fonte: Dados derivados do estudo Global Burden of Disease 2019, Institute for Health Metrics and Evaluation, University of Washington, ^*46*^*

**Tabela 2-10:** – Número de YLDs e taxas de YLDs padronizadas por idade (por 100 mil) por AVC, AVC isquêmico, hemorragia subaracnóidea e hemorragia intracerebral em 1990 e 2019, com variação percentual das taxas, no Brasil, de acordo com o grup

Causa de morte e grupo etário	1990		2019		Variação percentual (II 95%)
	Número (II 95%)	Taxa (II 95%)	Número (II 95%)	Taxa (II 95%)	
**B.2.3-AVC**					
Abaixo de 5	698,9 (446,2;1034,8)	4,1 (2,6;6,1)	605,5 (398;883,7)	3,9 (2,6;5,7)	-5,3 (-10,4;1)
15-49 anos	53038,7 (38137,5;68539,1)	69,2 (49,8;89,4)	67619,4 (48183,5;87875,3)	58,6 (41,7;76,1)	-15,4 (-18,6;-12,1)
50-49 anos	70829,2 (51187,2;90461,9)	451,5 (326,3;576,6)	111713,8 (80775,1;143720,1)	276,9 (200,2;356,2)	-38,7 (-40,9;-36,1)
5-14 anos	5159,6 (3356,4;7554,5)	14,6 (9,5;21,4)	4425,6 (2954,9;6303)	13,7 (9,2;19,5)	-6 (-12,1;1,5)
70+ anos	45920,6 (32727,2;59923)	1085,6 (773,7;1416,6)	99192,9 (70400,8;129782,2)	757,9 (537,9;991,6)	-30,2 (-33,5;-26,8)
Padronizada por idade	175647 (130408,3;222000,4)	180,4 (133,2;228,8)	283557,2 (208451,8;357622,1)	120,9 (88,7;152,6)	-33 (-34,8;-31,1)
Toda as idades	175647 (130408,3;222000,4)	118 (87,6;149,2)	283557,2 (208451,8;357622,1)	130,9 (96,2;165,1)	10,9 (7,7;14,4)
**B.2.3.1-AVC isquêmico**					
Abaixo de 5	575 (352,9;900,4)	3,4 (2,1;5,3)	484,4 (305,1;735,3)	3,1 (2;4,7)	-7,9 (-13,8;-0,3)
15-49 anos	27491,8 (18751,4;37159,1)	35,9 (24,5;48,5)	36232,1 (24894,2;49392,8)	31,4 (21,6;42,8)	-12,5 (-17,6;-7,1)
50-49 anos	44745,5 (31538,3;58754,1)	285,2 (201;374,5)	70202,8 (49237;92286,4)	174 (122;228,8)	-39 (-42,1;-35,7)
5-14 anos	3772,3 (2249;5772,3)	10,7 (6,4;16,3)	3123,3 (1924,8;4738,2)	9,7 (6;14,7)	-9,3 (-17,3;0,4)
70+ anos	37445,3 (26156,1;50128,5)	885,2 (618,3;1185,1)	78431,8 (55156,5;105572,2)	599,2 (421,4;806,6)	-32,3 (-35,9;-28,5)
Padronizada por idade	114029,9 (82236,3;147025)	122,2 (88;156,8)	188474,4 (134840,6;241680,6)	81,2 (58,3;104)	-33,5 (-35,9;-31)
Toda as idades	114029,9 (82236,3;147025)	76,6 (55,3;98,8)	188474,4 (134840,6;241680,6)	87 (62,2;111,5)	13,5 (9,2;18,1)
**B.2.3.2-Hemorragia intracerebral**					
Abaixo de 5	101,8 (64,4;149,7)	0,6 (0,4;0,9)	99,8 (64,8;145,8)	0,6 (0,4;0,9)	7,1 (1,6;13,9)
15-49 anos	17744,7 (12329,9;23735,5)	23,2 (16,1;31)	20847,3 (14380,3;27914,4)	18,1 (12,5;24,2)	-22 (-27,3;-16,4)
50-49 anos	19893,5 (14366,8;26543,6)	126,8 (91,6;169,2)	28759,2 (20440;38067,7)	71,3 (50,7;94,4)	-43,8 (-47,2;-39,7)
5-14 anos	1143,1 (714,8;1658,3)	3,2 (2;4,7)	1072,9 (668,4;1551,9)	3,3 (2,1;4,8)	2,8 (-3,2;11,1)
70+ anos	7297,4 (5111,2;9866)	172,5 (120,8;233,2)	16923,3 (11862;22603,3)	129,3 (90,6;172,7)	-25 (-31,1;-17,9)
Padronizada por idade	46180,6 (33564,1;59463,8)	44,3 (32,3;56,6)	67702,4 (49062,3;87760,6)	28,5 (20,7;36,9)	-35,7 (-38,8;-32,2)
Toda as idades	46180,6 (33564,1;59463,8)	31 (22,6;40)	67702,4 (49062,3;87760,6)	31,2 (22,6;40,5)	0,7 (-4,8;6,3)
**B.2.3.3-Hemorragia subaracnóidea**					
Abaixo de 5	22 (11,8;34,9)	0,1 (0,1;0,2)	21,2 (11,4;33,7)	0,1 (0,1;0,2)	5,3 (2,2;9,7)
15-49 anos	7802,2 (5194,1;10669,3)	10,2 (6,8;13,9)	10540,1 (7231,3;14584)	9,1 (6,3;12,6)	-10,3 (-16,3;-4,4)
50-49 anos	6190,3 (4238,1;8554,9)	39,5 (27;54,5)	12751,8 (8762,5;17660,6)	31,6 (21,7;43,8)	-19,9 (-26,1;-13,3)
5-14 anos	244,2 (137,9;385,3)	0,7 (0,4;1,1)	229,4 (133,9;356,3)	0,7 (0,4;1,1)	2,9 (0,1;6,9)
70+ anos	1177,9 (762,8;1668,6)	27,8 (18;39,4)	3837,8 (2504,8;5430,3)	29,3 (19,1;41,5)	5,3 (-4,4;17,1)
Padronizada por idade	15436,6 (10801,8;20590,3)	13,9 (9,7;18,6)	27380,3 (19490,9;36579,7)	11,2 (7,9;15)	-19,5 (-23,7;-15,4)
Toda as idades	15436,6 (10801,8;20590,3)	10,4 (7,3;13,8)	27380,3 (19490,9;36579,7)	12,6 (9;16,9)	21,8 (14,2;29,3)

*Fonte: Dados derivados do estudo Global Burden of Disease 2019, Institute for Health Metrics and Evaluation, University of Washington. ^*46*^*

**Tabela 2-11:** – Número de DALYs e taxas de DALYs padronizadas por idade (por 100 mil) por AVC, AVC isquêmico, hemorragia subaracnóidea e hemorragia intracerebral em 1990 e 2019, com variação percentual das taxas, no Brasil e suas unidades federativas.

Causa de morte e localização	1990		2019		Variação percentual (II 95%)
	Número (II 95%)	Taxa (II 95%)	Variação percentual (II 95%)	Taxa (II 95%)	
**B.2.3-AVC**					
Acre	3953,9 (3619,8;4276,6)	2252,5 (2074;2424,4)	8348,6 (7568,7;9153,4)	1310,9 (1188,3;1433,8)	-41,8(-47,8;-34,9)
Alagoas	47867,3 (43901,6;52819,4)	3342,5 (3046,7;3689,9)	58761,4 (51951,5;66173,7)	1822,9 (1616,1;2048,1)	-45,5(-53,6;-36,4)
Amapá	2062,9 (1882,6;2227,6)	1902,2 (1747,3;2044,2)	6967,3 (6287,8;7620,7)	1264,2 (1135,5;1381,4)	-33,5(-39,9;-26,7)
Amazonas	20109,3 (18325,7;21820,6)	2433,1 (2221,2;2622,4)	34973,2 (31325,3;39025,7)	1179,7 (1054,5;1317,2)	-51,5(-56,6;-45,4)
Bahia	186084,1 (166927,6;207069,4)	2605 (2332,8;2895,8)	226991,9 (194303,9;262385)	1402,4 (1202,3;1619,2)	-46,2(-55,2;-35,4)
Brasil	2766491,4 (2670978,2;2865452,2)	2959 (2829,6;3063)	2861723,2 (2683069,9;3012805,9)	1219,6 (1142;1285,5)	-58,8(-61;-56,8)
Ceará	88959,4 (77643,6;101337,9)	2068,8 (1789,3;2379,4)	134906,6 (114270,4;156553,5)	1352,6 (1144,6;1568,4)	-34,6(-47;-19,6)
Distrito Federal	19461,3 (17575,3;21841,7)	3045,6 (2783,3;3373)	26655,1 (23860,8;29674,3)	1095 (979,1;1216,4)	-64(-68,4;-59,2)
Espírito Santo	52398 (50083,8;54649,4)	3436,5 (3269,6;3588,3)	57215,8 (50636,1;63791,6)	1319,3 (1168,5;1468,8)	-61,6(-65,9;-57,3)
Goiás	77252,6 (67347,7;90376,8)	3403 (2985,6;3978,2)	81008,6 (69615,5;93585,6)	1152,6 (989,1;1328,7)	-66,1(-72;-59,4)
Maranhão	77077,8 (65445,9;90039,2)	2667,9 (2263;3100,3)	121736 (103916,8;143735,3)	1821,1 (1552,9;2145,6)	-31,7(-45,2;-13,3)
Mato Grosso	21222,8 (18529,1;23676,4)	2422,1 (2158,6;2674,9)	36897,6 (33242,4;40972,5)	1097,7 (988,4;1220,4)	-54,7(-60,5;-47,8)
Mato Grosso do Sul	28610,2 (26745,4;30493,9)	2938 (2748;3130,6)	34130,9 (30562,2;38068,1)	1158 (1038,9;1289,2)	-60,6(-64,7;-55,8)
Minas Gerais	339733,4 (316523,6;369110,1)	3246,5 (3019;3518,1)	288464,3 (260517,4;320004,4)	1100,1 (993,6;1219,9)	-66,1(-70,2;-62,1)
Pará	63769,5 (56956,4;70739)	2927,8 (2631,3;3237,3)	97258,8 (86061,6;107948,3)	1366,3 (1208,8;1517)	-53,3(-59,1;-46,8)
Paraíba	50969,4 (45276,3;56936,1)	2170 (1923,3;2422,3)	59408,3 (52413,8;67229,9)	1257,2 (1110,2;1421,9)	-42,1(-50,7;-31,3)
Paraná	176843,7 (168958;184595,9)	3564,4 (3392,5;3730,9)	163286,9 (145233,7;181362,6)	1249,4 (1109,3;1379,5)	-64,9(-68,8;-61)
Pernambuco	137205,1 (129269;145238,6)	2955,7 (2776,9;3127,6)	150292,6 (134781,7;166740,6)	1493,6 (1341;1658)	-49,5(-55;-43,8)
Piaui	41180,3 (37581,7;45051,1)	2844,7 (2600,7;3108,1)	50804,6 (45057,7;56665,2)	1347,6 (1196,3;1503,9)	-52,6(-58,5;-45,8)
Rio de Janeiro	377537 (359753,7;393981,6)	3749,7 (3570,9;3913,2)	285663,5 (256426,8;314757,9)	1288,3 (1157,8;1419,9)	-65,6(-68,9;-62)
Rio Grande do Norte	30686,3 (27616,3;34007,4)	1866 (1666,5;2076,7)	37002,4 (31613,2;43051,1)	950,9 (813,7;1105,4)	-49(-57,4;-39,5)
Rio Grande do Sul	190752,5 (181635,6;200277,2)	2867,6 (2718;3009,7)	181831,5 (162001,9;201216,5)	1190,7 (1061,7;1314,9)	-58,5(-62,4;-54)
Rondônia	12523,1 (10831,5;13950,3)	3595 (3267,5;3918,2)	19404,8 (16963,8;22202,1)	1247 (1091;1418,6)	-65,3(-70,3;-59,4)
Roraima	1690 (1492,5;1869)	2667,4 (2430,1;2899,2)	4362,7 (3925,8;4818,5)	1151,5 (1032,8;1263,9)	-56,8(-61,8;-51,5)
Santa Catarina	79849,1 (75128,2;84605,6)	3134,4 (2945,7;3316,1)	79975 (71270,1;89055)	1009,6 (897,6;1124,2)	-67,8(-71,1;-64,3)
São Paulo	604794,8 (569838,8;640063,1)	2851,4 (2681,9;3015,5)	563721,2 (507325,6;621571,8)	1049,5 (946,3;1153,5)	-63,2(-66,7;-59,5)
Sergipe	22612,3 (20686,7;24655,8)	2826,1 (2577,3;3076,2)	31067,9 (26888,4;35427)	1376,1 (1192,8;1570,2)	-51,3(-58,5;-43,8)
Tocantins	11285,4 (9918;12704,6)	2724,8 (2408,6;3041,8)	20585,9 (17987,9;23457,2)	1427,5 (1246,1;1620)	-47,6(-55,8;-37,6)
**B.2.3.1-AVC isquêmico**					(;)
Acre	1445 (1296,1;1599,1)	1090,2 (977,5;1196,9)	3329,6 (2969,8;3695,2)	613,7 (545,3;681,5)	-43,7(-50,8;-34,4)
Alagoas	20021,2 (17255,8;22924,7)	1614,9 (1401,1;1835,8)	26131,5 (22660;29606,5)	869,8 (753,3;987,1)	-46,1(-56,3;-34,9)
Amapá	787,9 (717;856,8)	934,1 (846;1013,2)	2756,8 (2429,7;3053,3)	603 (528,6;667)	-35,4(-41,7;-28,9)
Amazonas	7502,6 (6829,2;8203,1)	1149,3 (1045,3;1247,4)	14074,1 (12487,8;15775,3)	546,2 (483;611,7)	-52,5(-57,5;-46,9)
Bahia	73495,4 (63810,7;83067,2)	1155,9 (1004,3;1304,2)	98253,4 (83330,9;113726,8)	624,2 (529,5;723,8)	-46(-56,1;-33,2)
Brasil	1061065 (999618,4;1116287,5)	1333,3 (1244,5;1403,6)	1268106,5 (1157551,8;1356041,6)	561 (510,4;599,8)	-57,9(-61;-55)
Ceará	36622,7 (30420,4;43447,9)	931,9 (774,3;1101,8)	61215,8 (51448,4;71614,2)	629,2 (529,2;737,1)	-32,5(-46,6;-13,1)
Distrito Federal	5537 (4963,6;6234,1)	1415,6 (1290,1;1562,9)	11151,2 (9927,9;12430,9)	569,1 (500,4;632,3)	-59,8(-64,4;-54,7)
Espírito Santo	19982,1 (18630,5;21190,4)	1574,6 (1462,7;1670,2)	24655,8 (21593;27722,9)	602,5 (526,3;677)	-61,7(-66,2;-57,1)
Goiás	25866,3 (22357,1;30295,8)	1451,8 (1263,5;1680,1)	33225,9 (28498,2;38313,9)	515,9 (443,4;592,1)	-64,5(-70,3;-57,5)
Maranhão	28972,4 (22485,8;34775,6)	1203,5 (957,9;1438)	53315,2 (45693,2;61949,2)	841,3 (720,3;979,7)	-30,1(-45,6;-6)
Mato Grosso	7757,1 (6804,9;8655,7)	1175,3 (1037,2;1298,1)	15225,4 (13520,5;17052,5)	510,5 (451;571,7)	-56,6(-62;-50,3)
Mato Grosso do Sul	9617,3 (8855,7;10378,2)	1251,4 (1148,9;1348,5)	14227,2 (12650,7;15990)	517,7 (459,4;579,7)	-58,6(-63,1;-53,4)
Minas Gerais	121530,5 (111693,4;132739,1)	1381,5 (1265,7;1498,9)	125719,7 (111263,1;139550,3)	483,1 (427,3;536,1)	-65(-68,9;-60,8)
Pará	26583 (23751,3;29461,2)	1475,7 (1316,4;1624,1)	43317,3 (38006;48704,4)	674,6 (589;758,1)	-54,3(-60,1;-47,8)
Paraíba	22818 (19197,4;26497,7)	1008 (850,9;1164,9)	26234,7 (22655,5;29851,3)	547,8 (474;624,8)	-45,7(-55,8;-32,7)
Paraná	71620,1 (67166,7;76010)	1740,7 (1623,4;1845)	81143,3 (71177,3;90509,3)	649 (570;721,8)	-62,7(-66,7;-58,4)
Pernambuco	56079,4 (50916,4;61089,4)	1344,9 (1218,5;1461,6)	61765,2 (54385;69585)	649,5 (572,4;731,4)	-51,7(-58,2;-44,8)
Piaui	16603 (14591;18612,8)	1356,2 (1194,6;1513,5)	23795,6 (20662,8;26814)	632 (550,7;712,3)	-53,4(-60,4;-45,4)
Rio de Janeiro	135992,8 (127706,6;144128,1)	1580,7 (1479,5;1669,4)	119754,3 (106880,1;131983,3)	545,1 (484,7;600,2)	-65,5(-68,8;-62)
Rio Grande do Norte	13717,9 (11997,9;15557,9)	881,2 (767,8;996,2)	16848,6 (14243,9;19722)	438,8 (371;513,3)	-50,2(-58,5;-39)
Rio Grande do Sul	80633,6 (75289,1;85992,1)	1401,7 (1302,2;1490)	91439,3 (80768,7;101588,9)	595,8 (526,8;661,3)	-57,5(-61,5;-53,1)
Rondônia	4299,9 (3810,6;4771,5)	1918,2 (1743,4;2081,6)	8241,2 (7143,1;9486,9)	601,6 (518,8;692,2)	-68,6(-73,1;-63,5)
Roraima	550 (496,1;608,2)	1346,9 (1226,1;1465,1)	1750,4 (1551,2;1947,6)	577,2 (511,8;639,3)	-57,1(-62,2;-51,7)
Santa Catarina	32468,4 (30272,1;34691,7)	1547,6 (1440;1650,7)	38577,2 (33969,3;43198,7)	520,5 (460,4;581,1)	-66,4(-70;-62,6)
São Paulo	226823,4 (210500,4;242557,8)	1290,9 (1189;1375,2)	249002,3 (221220;276006,2)	479 (424,5;530,4)	-62,9(-66,2;-59,1)
Sergipe	9762,5 (8615;10829,7)	1413,3 (1257,8;1555,8)	13877 (11998,2;15919,3)	656,3 (566,1;753,4)	-53,6(-60,8;-45,2)
Tocantins	3975,5 (3418,4;4557,8)	1322,2 (1158,6;1492,4)	9078,6 (7754,4;10411,6)	680,1 (579,8;780,2)	-48,6(-57,9;-38,2)
**B.2.3.2-Hemorragia intracerebral**					(;)
Acre	1839 (1657,2;2040,1)	914,2 (827,4;1008,7)	3542,8 (3164,8;3962,9)	512,3 (459,6;572)	-44(-51,5;-34,8)
Alagoas	21422,3 (18719,7;24577,7)	1385,5 (1204,8;1595,7)	24575,6 (21311,8;27913)	728,1 (634,3;828,1)	-47,4(-58,2;-35,7)
Amapá	975,4 (873,5;1064,7)	789,3 (718,9;857,2)	3023,1 (2676,9;3387,4)	497,1 (440;554,1)	-37(-44,2;-28,4)
Amazonas	10062,1 (9057,6;11050,1)	1082,4 (983,6;1184,9)	15315,2 (13500,3;17329,6)	481,2 (426,5;545,3)	-55,5(-61,2;-48,8)
Bahia	87938,6 (77288,1;99639,1)	1171,2 (1030,7;1328,1)	95088 (79241,5;112242,3)	578,9 (483,3;682,9)	-50,6(-60,7;-38,7)
Brasil	1349736,5 (1296807,8;1423036,1)	1327,8 (1274;1397,3)	1154165,4 (1091357,5;1217146,3)	477,6 (450,9;503,8)	-64(-66,6;-61,6)
Ceará	39918,9 (33988,8;46866,7)	893,1 (750,3;1054,3)	54532,7 (45115,4;64606,6)	538,5 (446,4;637,3)	-39,7(-53,7;-23,3)
Distrito Federal	10416,4 (9283,2;12008,7)	1319,7 (1186,3;1497,7)	10477,5 (9230,5;11872,3)	368,5 (324;415,9)	-72,1(-75,8;-67,3)
Espírito Santo	25812,1 (24512,2;27292,3)	1537 (1456,4;1620,6)	23740,7 (20717,1;26779,4)	523,9 (458,6;590,2)	-65,9(-70,3;-61,4)
Goiás	40984,5 (35216,1;48923,1)	1619,8 (1399,7;1933,9)	34554,4 (29125,6;40074,6)	465,6 (393,3;540,9)	-71,3(-76,9;-64,6)
Maranhão	35790,2 (28841,3;44237,6)	1143,2 (915,9;1417,9)	49711,7 (41406;59969,3)	725,6 (604;877)	-36,5(-51,4;-15,9)
Mato Grosso	10195,6 (8768,9;11594,7)	1009,1 (882,3;1135,1)	15221,9 (13452,7;17149,6)	421,3 (373,4;474,5)	-58,3(-64,9;-50,3)
Mato Grosso do Sul	15024,7 (13886,9;16246,9)	1391 (1290,8;1500,3)	14338 (12701,5;16270,4)	465,1 (412,8;527,4)	-66,6(-70,8;-61,7)
Minas Gerais	172794,2 (158931,3;194463,9)	1520,8 (1400,4;1698,4)	115640 (103297,7;129456,7)	435,7 (389;487,8)	-71,4(-75,6;-67,2)
Pará	29854,6 (26395,2;33517,5)	1225,7 (1088;1368,5)	39347,5 (34472;44066,4)	519,2 (455,8;581,9)	-57,6(-64;-50,5)
Paraíba	20767,9 (17738,6;24596,1)	870,4 (742,4;1026,4)	24329,8 (21197,1;27942,4)	520,5 (453,2;597,8)	-40,2(-52,6;-25,2)
Paraná	84543 (79771,8;89664)	1517 (1432,2;1613,9)	58629,2 (51418,8;66053,2)	428,1 (376,1;481,6)	-71,8(-75,5;-67,8)
Pernambuco	67425,3 (62725,9;72662,2)	1367,7 (1271,3;1473,7)	67025,9 (59208,3;75471,8)	643,1 (568,2;724)	-53(-59,3;-46)
Piaui	18830,6 (16752,3;21434,6)	1189,1 (1056,4;1349,7)	19788,3 (17389,9;22475,7)	525,8 (463,1;597,1)	-55,8(-62,9;-47,4)
Rio de Janeiro	195616,9 (184503,4;208076,4)	1791,7 (1692,6;1901,7)	124136,4 (110522,6;139387,9)	550,3 (490,7;617,8)	-69,3(-72,7;-65,2)
Rio Grande do Norte	13368,5 (11836,1;15038,3)	794,2 (700,4;893,9)	14784,6 (12178,2;17578,3)	377,7 (311,1;448,6)	-52,4(-61,8;-41,9)
Rio Grande do Sul	90021,7 (84641,8;95298,5)	1224,3 (1151,1;1296,8)	68288,3 (59641,3;76831,2)	442,9 (386,3;499,9)	-63,8(-68,2;-59)
Rondônia	6492,8 (5543,7;7393)	1423,5 (1256,5;1588,5)	7939,9 (6835,3;9291,1)	471,6 (406,7;549,8)	-66,9(-72,6;-59,1)
Roraima	878,3 (761,3;987,6)	1105,3 (988,9;1218,4)	1841,7 (1630,9;2065,2)	424,1 (375,7;473,5)	-61,6(-67;-55,7)
Santa Catarina	38007,8 (35410,7;40721,5)	1323,1 (1232,3;1419,8)	29457 (25702,3;33414,7)	349,4 (304,4;395,8)	-73,6(-76,8;-70)
São Paulo	294848,3 (273533,7;320151,5)	1260,5 (1173,1;1353,1)	217553,4 (194543,2;243976,7)	393,1 (351,9;440,7)	-68,8(-72,7;-64,7)
Sergipe	10294,6 (9239,9;11405,8)	1172,3 (1047,6;1298,5)	12930,8 (11015,5;15067,4)	549,5 (468,4;639,3)	-53,1(-61,2;-44,1)
Tocantins	5612,4 (4778,8;6561,7)	1138,4 (966,8;1324,8)	8351,1 (7162,9;9670,2)	553,5 (477,1;640,4)	-51,4(-61,4;-39,4)
**B.2.3.3-Hemorragia subaracnóidea**					(;)
Acre	669,8 (588,4;766,7)	248,1 (218,2;282)	1476,2 (1313,2;1664,4)	184,8 (165;208,1)	-25,5(-37,1;-11,4)
Alagoas	6423,8 (5403,3;7533,8)	342,2 (288;399,9)	8054,3 (6938,2;9342,8)	225 (193,7;260,5)	-34,3(-47,9;-16,6)
Amapá	299,6 (258,9;343,2)	178,9 (158,7;206,4)	1187,4 (1062,8;1353,1)	164,1 (146,3;186,4)	-8,3(-20,6;7,2)
Amazonas	2544,6 (2247;2874)	201,4 (179;228,9)	5583,9 (4913,9;6445,9)	152,3 (134,3;175,8)	-24,4(-36;-10,6)
Bahia	24650,1 (21393,8;28224,4)	278 (241,4;320,6)	33650,5 (27808,9;40181,7)	199,3 (165;237,9)	-28,3(-43,4;-9,5)
Brasil	355689,9 (317037;373740,1)	297,9 (267,5;312,7)	439451,3 (411002,3;468439,5)	181 (169,4;192,8)	-39,2(-43,8;-31,8)
Ceará	12417,9 (10343,3;14787,4)	243,8 (204,2;287,9)	19158,1 (15794,9;23207,8)	184,9 (153,1;223,6)	-24,2(-40,5;0,1)
Distrito Federal	3507,9 (3110;3938,7)	310,3 (274,7;347,1)	5026,3 (4331,4;5790)	157,4 (135,3;179,7)	-49,3(-56,7;-39,9)
Espírito Santo	6603,8 (5412,6;7189)	324,9 (268,7;352,5)	8819,3 (7521,5;10292,6)	192,9 (164,8;224,2)	-40,6(-49,6;-29)
Goiás	10401,8 (9100,6;12049,3)	331,4 (289;383,9)	13228,3 (11078,7;15754,8)	171,2 (143,6;202,2)	-48,3(-58,3;-36,4)
Maranhão	12315,2 (9170,6;16162,5)	321,2 (242,9;414,9)	18709,1 (15184,3;22615,2)	254,1 (205,3;308,8)	-20,9(-42,8;8,5)
Mato Grosso	3270 (2710,8;3823,3)	237,6 (202,4;274,7)	6450,3 (5684,7;7409,4)	165,9 (146,5;189,9)	-30,2(-42;-14,8)
Mato Grosso do Sul	3968,3 (3590;4343,7)	295,6 (268,3;323,2)	5565,7 (4859,9;6325)	175,2 (153,2;199)	-40,7(-48,8;-31,2)
Minas Gerais	45408,7 (39218,4;49588,4)	344,1 (298,9;375,4)	47104,6 (41067,1;53672,3)	181,2 (158,9;206)	-47,3(-54,1;-38,5)
Pará	7331,9 (6385;8336)	226,4 (197;258,7)	14594 (12863,3;16610,3)	172,5 (152,2;196,2)	-23,8(-36,1;-8,5)
Paraíba	7383,6 (6372,6;8509,5)	291,7 (250,6;337,7)	8843,8 (7549,8;10263,5)	188,9 (161,6;219,4)	-35,2(-48,3;-19,5)
Paraná	20680,6 (18344,4;22387,3)	306,7 (272,9;330,9)	23514,4 (20164,4;27053,9)	172,4 (148,7;197,6)	-43,8(-51,9;-35,2)
Pernambuco	13700,4 (12356,5;15479,4)	243 (218,8;276,6)	21501,4 (18756,1;24644,5)	201,1 (176;229,8)	-17,2(-30,4;-2,2)
Piaui	5746,7 (4929,9;6600,1)	299,4 (259,7;340,8)	7220,7 (6236,6;8335)	189,8 (164;218,6)	-36,6(-48,3;-21,8)
Rio de Janeiro	45927,3 (35485,7;50624,3)	377,3 (297,1;415,1)	41772,9 (36465,3;47362,4)	192,9 (169,3;218,2)	-48,9(-56,4;-34,3)
Rio Grande do Norte	3599,9 (3104,5;4304,3)	190,6 (163,1;232,6)	5369,2 (4435,8;6786,1)	134,4 (111,1;169,7)	-29,5(-43,4;-11,8)
Rio Grande do Sul	20097,2 (18365,6;21848)	241,6 (221,4;263,4)	22103,9 (19284,4;25482,8)	152,1 (132,9;174,4)	-37,1(-45,4;-27,1)
Rondônia	1730,4 (1389,1;2030,2)	253,4 (212,8;290,4)	3223,7 (2788,9;3765,4)	173,8 (150,7;202,1)	-31,4(-43,6;-13,7)
Roraima	261,7 (217,1;304,3)	215,2 (185,3;246,4)	770,6 (680,9;905,9)	150,3 (133,5;176,3)	-30,2(-41;-16,4)
Santa Catarina	9372,9 (8539,8;10192,6)	263,7 (240,5;287,5)	11940,7 (10397,6;13772,2)	139,7 (122,3;160,2)	-47(-54;-37,9)
São Paulo	83123 (72075,6;90420,8)	300 (263,7;326,2)	97165,6 (85347,2;110658,3)	177,4 (156,3;200,9)	-40,9(-48,5;-31,1)
Sergipe	2555,2 (2239,9;2912,1)	240,5 (210,7;275,3)	4260,2 (3562,3;5058,4)	170,4 (142,7;202)	-29,2(-43,1;-12,2)
Tocantins	1697,4 (1407;1987,1)	264,2 (222,3;307,1)	3156,2 (2681,1;3702,9)	193,9 (165,3;227,7)	-26,6(-40,7;-8,3)

*Fonte: Dados derivados do estudo Global Burden of Disease 2019, Institute for Health Metrics and Evaluation, University of Washington. ^*46*^*

**Tabela 2-12:** – Número de DALYs e taxas de DALYs padronizadas por idade (por 100 mil) por AVC, AVC isquêmico, hemorragia subaracnóidea e hemorragia intracerebral em 1990 e 2019, com variação percentual das taxas, no Brasil, de acordo com o grupo etário.

Causa de morte e grupo etário	1990		2019		Variação percentual (II 95%)
	Número (II 95%)	Taxa (II 95%)	Número (II 95%)	Taxa (II 95%)	
**B.2.3-AVC**					
Abaixo de 5	37072,3 (27007,4;50450,1)	218,9 (159,4;297,8)	6575,8 (4847,9;8823,2)	42,4 (31,3;56,9)	-80,6 (-88,1;-68,6)
15-49 anos	769115,6 (738502,6;803387,2)	1003,5 (963,5;1048,2)	516175,3 (483925,6;549638,4)	446,9 (419;475,9)	-55,5 (-58,2;-52,7)
50-69 anos	1154968,9 (1110879,7;1201696,6)	7362,3 (7081,2;7660,1)	1179642,5 (1118241,2;1239653,5)	2924 (2771,8;3072,8)	-60,3 (-62,8;-57,9)
5-14 anos	24536,1 (21707,5;27385,7)	69,4 (61,4;77,5)	13756 (11528,4;16101,8)	42,7 (35,7;49,9)	-38,6 (-46;-30,7)
70+ anos	780798,4 (727457,3;814613,1)	18458,3 (17197,3;19257,7)	1145573,7 (1032837,8;1220898)	8752,5 (7891,2;9328)	-52,6 (-55,8;-49,7)
Padronizada por idade	2766491,4 (2670978,2;2865452,2)	2959 (2829,6;3063)	2861723,2 (2683069,9;3012805,9)	1219,6 (1142;1285,5)	-58,8 (-61;-56,8)
Todas as idades	2766491,4 (2670978,2;2865452,2)	1858,8 (1794,6;1925,2)	2861723,2 (2683069,9;3012805,9)	1320,8 (1238,4;1390,5)	-28,9 (-32,9;-25,3)
**B.2.3.1-AVC isquêmico**					
Abaixo de 5	4710,2 (3531,7;6087,8)	27,8 (20,9;35,9)	868,8 (655,6;1164,7)	5,6 (4,2;7,5)	-79,8 (-86,2;-70,6)
15-49 anos	113665,5 (102596,5;125599)	148,3 (133,9;163,9)	79615,6 (67096;93825,9)	68,9 (58,1;81,2)	-53,5 (-58,8;-47,3)
50-69 anos	393774,7 (369506,2;418040,5)	2510,1 (2355,4;2664,8)	369798 (343143,9;399308)	916,6 (850,6;989,8)	-63,5 (-66,5;-59,9)
5-14 anos	5392,6 (3886,2;7362,5)	15,3 (11;20,8)	3563,5 (2394,7;5201,8)	11,1 (7,4;16,1)	-27,6 (-35,6;-20,1)
70+ anos	543521,9 (503524,5;570910,5)	12849 (11903,5;13496,5)	814260,7 (727889,1;874704,1)	6221,2 (5561,3;6683)	-51,6 (-55,1;-48,3)
Padronizada por idade	1061065 (999618,4;1116287,5)	1333,3 (1244,5;1403,6)	1268106,5 (1157551,8;1356041,6)	561 (510,4;599,8)	-57,9 (-61;-55)
Todas as idades	1061065 (999618,4;1116287,5)	712,9 (671,6;750)	1268106,5 (1157551,8;1356041,6)	585,3 (534,3;625,9)	-17,9 (-24,2;-11,9)
**B.2.3.2-Hemorragia intracerebral**					
Abaixo de 5	17337,1 (11773,4;25176,4)	102,4 (69,5;148,6)	1830,6 (1306,1;2522,5)	11,8 (8,4;16,3)	-88,5 (-93,4;-80)
15-49 anos	454266,8 (431721,6;491490,4)	592,7 (563,3;641,2)	259204,3 (240714,6;276923,9)	224,4 (208,4;239,8)	-62,1 (-66,6;-58,7)
50-69 anos	647660,7 (618581,9;681800,7)	4128,5 (3943,1;4346,1)	611298 (579754,5;643680)	1515,2 (1437,1;1595,5)	-63,3 (-65,9;-60,6)
5-14 anos	9000,5 (7931,1;10146,9)	25,5 (22,4;28,7)	3859,1 (3235,5;4576,9)	12 (10;14,2)	-53 (-60,8;-44,5)
70+ anos	221471,5 (206213,9;234624,6)	5235,6 (4874,9;5546,6)	277973,3 (249008,1;299692,6)	2123,8 (1902,5;2289,7)	-59,4 (-63,2;-55,7)
Padronizada por idade	1349736,5 (1296807,8;1423036,1)	1327,8 (1274;1397,3)	1154165,4 (1091357,5;1217146,3)	477,6 (450,9;503,8)	-64 (-66,6;-61,6)
Todas as idades	1349736,5 (1296807,8;1423036,1)	906,9 (871,3;956,1)	1154165,4 (1091357,5;1217146,3)	532,7 (503,7;561,8)	-41,3 (-45,6;-37,2)
**B.2.3.3-Hemorragia subaracnóidea**					
Abaixo de 5	15024,9 (9326,8;20352,4)	88,7 (55,1;120,2)	3876,3 (2819,3;5345,7)	25 (18,2;34,5)	-71,8 (-83,1;-44,7)
15-49 anos	201183,3 (176381,8;212269,3)	262,5 (230,1;276,9)	177355,4 (166669,5;193612,9)	153,6 (144,3;167,6)	-41,5 (-46,4;-29)
50-69 anos	113533,5 (103789,1;120381)	723,7 (661,6;767,4)	198546,5 (182109,3;213801,4)	492,1 (451,4;530)	-32 (-37,5;-24)
5-14 anos	10143,1 (8918;11181)	28,7 (25,2;31,6)	6333,4 (5338,3;7382,5)	19,6 (16,6;22,9)	-31,6 (-42,5;-17,8)
70+ anos	15805,1 (14294,1;18534,2)	373,6 (337,9;438,2)	53339,7 (44065,9;59439)	407,5 (336,7;454,1)	9,1 (-21,6;25,3)
Padronizada por idade	355689,9 (317037;373740,1)	297,9 (267,5;312,7)	439451,3 (411002,3;468439,5)	181 (169,4;192,8)	-39,2 (-43,8;-31,8)
Todas as idades	355689,9 (317037;373740,1)	239 (213;251,1)	439451,3 (411002,3;468439,5)	202,8 (189,7;216,2)	-15,1 (-21,6;-4)

*Fonte: Dados derivados do estudo Global Burden of Disease 2019, Institute for Health Metrics and Evaluation, University of Washington. ^*46*^*

**Figura 2-1:**
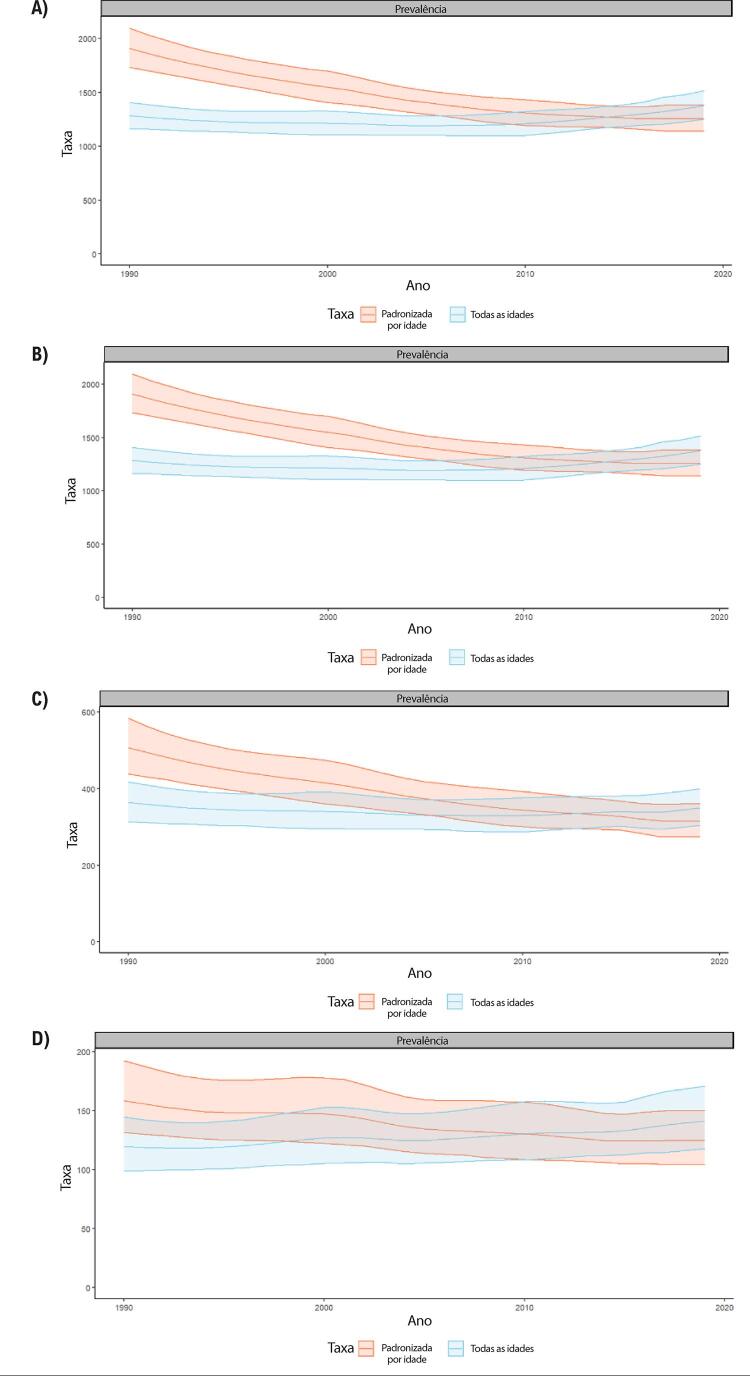
– Taxas de prevalência (por 100 mil habitantes) para todas as idades e padronizadas por idade de AVC (A), AVC isquêmico (B), hemorragia intracerebral (C) e hemorragia subaracnóidea (D), 1990-2019.

Fonte: Dados derivados do estudo Global Burden of Disease 2019, Institute for Health Metrics and Evaluation, University of Washington. ^46^

**Figura 2-2:**
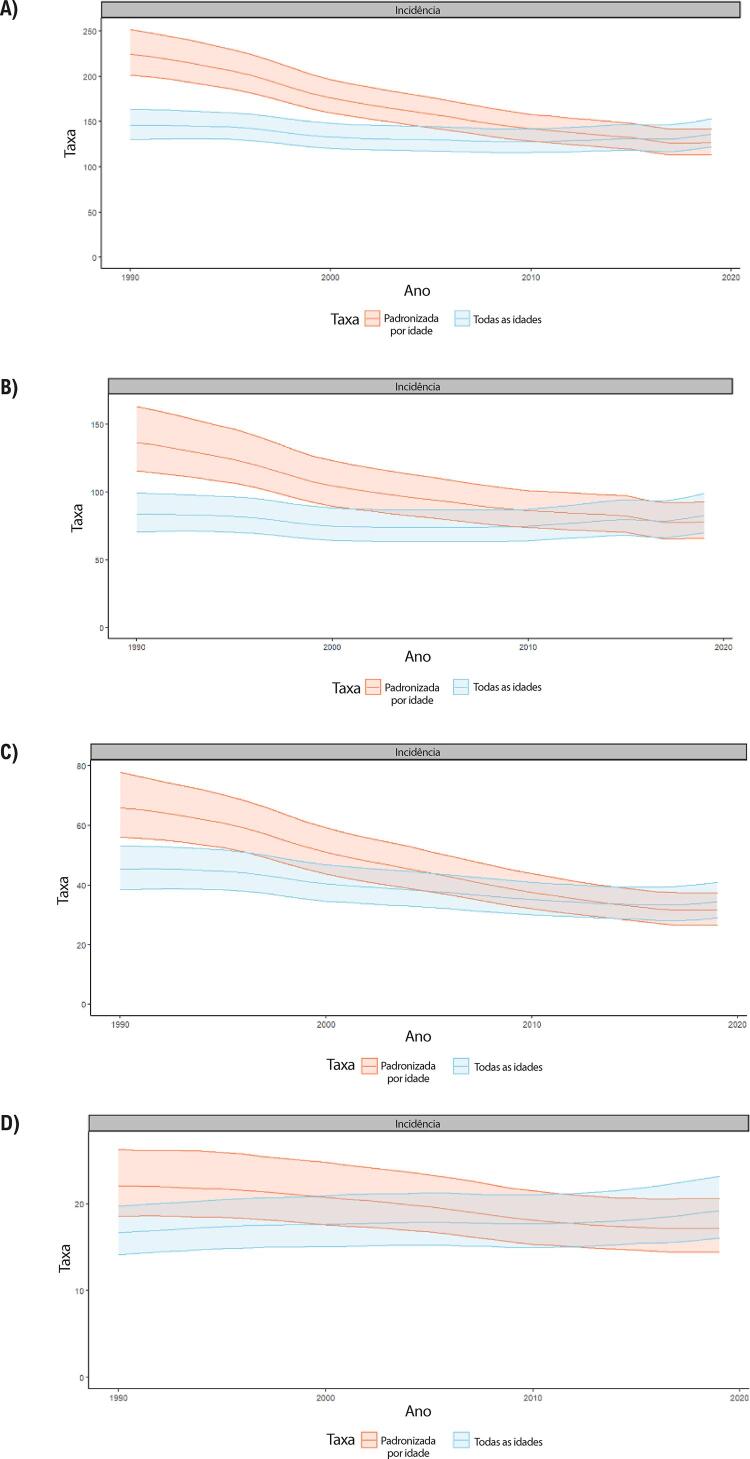
-Taxas de incidência (por 100 mil habitantes) para todas as idades e padronizadas por idade de AVC, (A), AVC isquêmico (B), hemorragia intracerebral (C) e hemorragia subaracnóidea (D), 1990-2019.

Fonte: Dados derivados do estudo Global Burden of Disease 2019, Institute for Health Metrics and Evaluation, University of Washington. ^46^

**Figura 2-3:**
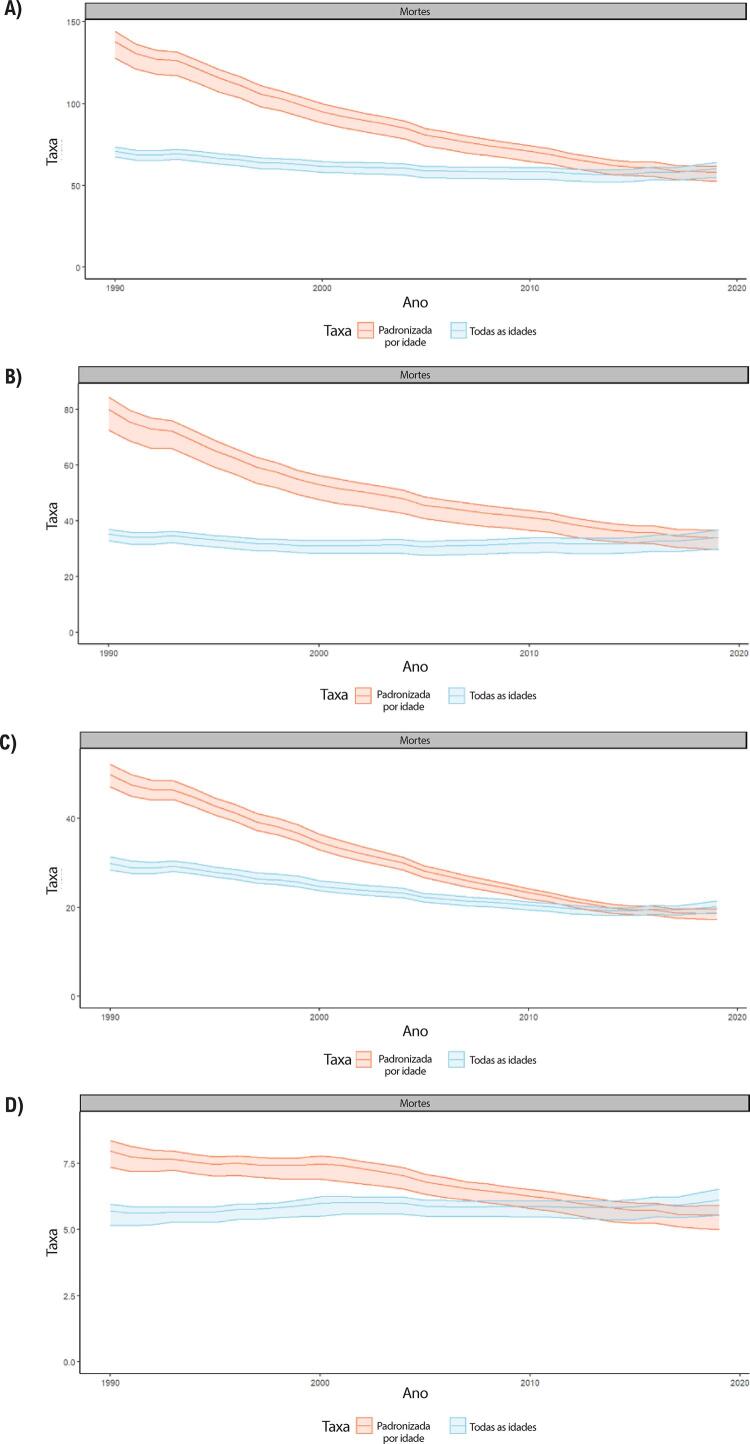
-Taxas de mortalidade (por 100 mil habitantes) para todas as idades e padronizadas por idade por AVC (A), AVC isquêmico (B), hemorragia intracerebral (C) e hemorragia subaracnóidea (D), 1990-2019.

Fonte: Dados derivados do estudo Global Burden of Disease 2019, Institute for Health Metrics and Evaluation, University of Washington. ^46^

### Prevalência

•As estimativas de prevalência de AVC podem diferir levemente entre os estudos, pois cada um seleciona e recruta uma amostra de participantes para representar sua população-alvo (estado, região ou país).

•Em um estudo de base comunitária no Brasil, que aplicou um questionário para 4.496 indivíduos com idade superior a 35 anos, residentes de uma área carente da cidade de São Paulo em 2011, Abe *et al* . identificaram, em uma triagem inicial, 243 questionários positivos para AVC. A taxa de prevalência ajustada para idade para homens foi 4,6% (IC 95%, 3,5 - 5,7) e, para mulheres, 6,5% (IC 95%, 5,5 - 7,5). ^47^

•Usando uma ferramenta de rastreio, um questionário de sintomas de AVC, Fernees *et al* . estudaram a prevalência de AVC na cidade de Coari, na Bacia Amazônica Brasileira, e compararam essa prevalência entre ribeirinhos e a população urbana do mesmo município. Em 4.897 respondentes da área urbana e 1.028 da rural, os autores encontraram uma prevalência bruta de AVC de 6,3% na área rural e de 3,7% na urbana, com diferenças mantidas após ajuste para sexo e idade. ^48^

•Utilizando o *Stepwise Approach to Stroke Surveillance* da OMS, Goulart *et al* . realizaram um estudo para verificar as taxas de mortalidade e morbidade por AVC em uma área de São Paulo. O questionário para determinar a prevalência de AVC foi aplicado de porta em porta em uma vizinhança do PSF (etapa 3). Dos 3.577 indivíduos com mais de 35 anos avaliados em casa, foram identificados 244 (6,8%) sobreviventes de AVC através do questionário validado por um neurologista certificado. ^49^

•Benseñor *et al* ., analisando um inquérito epidemiológico de base domiciliar (PNS - 2013) com uma amostra representativa nacional, avaliaram o número absoluto de indivíduos com AVC e com incapacidade por AVC, com as respectivas prevalências. Foram estimados 2.231.000 indivíduos com AVC e 568.000 com incapacidade grave por AVC. As estimativas das prevalências pontuais de AVC foram 1,6% e 1,4% para homens e mulheres, respectivamente. ^50^

•De acordo com dados do Grupo GBD Brasil, as taxas de prevalência padronizadas por idade de AVCI por 100 mil foram 1.327,6 (1.151,2 a 1.516) em 1990 e 870,1 (761,1 a 992,8) em 2019, representando uma variação percentual de -34,5 (-36,7 a -0,3) (Tabela 2-1 e Figura 2 -1). A maior variação percentual ocorreu em Rondônia, -41,7 (-46,2 a -0,4), e a menor, no Amapá, -23,4 (-28,6 a -0,2) (Tabela 2-1). Para os adultos, a maior variação percentual foi observada no grupo etário de 50-69 anos, -39,5 (-42,6 a -0,4) (Tabela 2-2).

•As taxas de prevalência padronizadas por idade de HIC por 100 mil foram 507,5 (438,9 a 584,1) em 1990 e 315,9 (275 a 361,4) em 2019, representando uma variação percentual de -37,7 (-40,5 a -0,3) (Tabela 2-1 e Figura 2 -1). Para os adultos, a maior variação percentual foi observada no grupo etário de 50-69 anos, -44,8 (-47,4 a -0,4) (Tabela 2-2). ^46^

•As taxas de prevalência padronizadas por idade de HSA por 100 mil foram 158,6 (131,7 a 192,4) em 1990 e 124,8 (104,2 a 150,1) em 2019, representando uma variação percentual de -21,3 (-24,3 a -0,2) ( Figura 2 -1 e Tabela 2-1). Para os adultos, a maior variação percentual foi observada no grupo etário de 50-69 anos, -21,1 (-24,4 a -0,2) Tabela 2-2). ^46^

### Incidência

#### Subtipos de AVC

•Dados do estudo de base comunitária de Joinville mostraram que, ao comparar diferentes períodos (1995, 2005-2006, 2010-2011 e 2012-2013), a incidência de AVC diminuiu. Nos últimos 18 anos, a incidência de AVC geral (todos os tipos principais de AVC) em Joinville diminuiu em 37% (IC 95%, 32 - 42). ^51^

•A incidência de primeiro episódio de AVC ajustada para a população brasileira foi 86,6 por 100 mil (IC 95%, 80,5 - 93,0) em 2005-2006 e 113,46 por 100 mil (IC 95%, 101,5 - 126,8) em 1995. ^52^ A incidência geral ajustada por idade e para a população mundial por 100 mil pessoas-ano foi 143,7 (IC 95%, 128,4 - 160,3) em 1995, caindo para 105,4 (IC 95%, 98,0 - 113,2) em 2005-2006 e para 90,9 (IC 95%, 85,1 - 96,9) em 2012-2013. A incidência padronizada por idade de primeiro episódio de AVC estratificada por gênero e idade também caiu significativamente ao longo do tempo. A redução foi 11% maior nos homens (42%; IC 95%, 35 - 49) do que nas mulheres (31%; IC 95%, 23 - 39) e 16% maior nos jovens (≤ 44 anos: 54%; IC 95%, 41–66) do que nos mais idosos (>44 anos: 38%; IC 95%, 33–43). ^51^

•De 1995 a 2013, a proporção de AVCI aumentou 12%, enquanto a de AVCH diminuiu 16%. Entretanto, a proporção de HSA permaneceu relativamente estável, variando de 7,5% em 1995 a 6% em 2012-2013. O peso da diminuição na incidência de AVC ajustada por idade foi proporcionalmente maior para AVCH do que para AVCI, enquanto o de HSA permaneceu estável. Nos últimos 8 anos, as incidências de AVCI e de AVCH apresentaram reduções absolutas significativas de 15% (IC 95%, 1-28) e de 60% (IC 95%, 13-86), respectivamente. Entretanto, a incidência de HSA apresentou redução absoluta não significativa de 29% (IC 95%, 15-92). ^46^

•No segundo estudo do registro de AVC de Matão, todos os eventos de AVC incidente (81 casos) que ocorreram entre 1 de agosto de 2015 e 31 de julho de 2016 foram registrados. A idade média aumentou em 9%, passando de 65,2 (IC 95%, 62,6–67,8) para 71,0 (IC 95%, 68,1–73,8) anos. Entre os períodos 2003-2004 e 2015-2016, a incidência ajustada por idade diminuiu em 39% (IRR 0,61; IC 95%, 0,46–0,79) e a mortalidade, em 50% (IRR 0,50; IC 95%, 0,31– 0,94). A taxa de letalidade de 1 ano foi 26%. Aproximadamente 56% dos pacientes foram funcionalmente independentes, enquanto 7% tiveram um AVC recorrente. ^53^ Comparando com os resultados do estudo anterior do registro de AVC de Matão, ^54^ não houve diferença significativa entre os desfechos.

•Dados do Grupo GBD Brasil mostram que as taxas de incidência padronizadas por idade de AVC por 100 mil foram 224,6 (201,6 a 251,8) em 1990 e 127 (113,8 a 142,1) em 2019, representando uma variação percentual de -43,5 (-44,7 a -0,4) (Tabela 2-3 e Figura 2 -2). A maior variação percentual ocorreu no Distrito Federal, -47,7 (-49,7 a -0,5), e a menor, no Ceará, -30,6 (-33,2 a -0,3) (Tabela 2-3). A maior variação percentual foi observada no grupo etário de 15-49 anos, -38,1 (-40,9 a -0,4) (Tabela 2-4). ^46^

•As taxas de incidência padronizadas por idade de AVCI por 100 mil foram 136,6 (115,7 a 163,1) em 1990 e 78,2 (66,1 a 93) em 2019, representando uma variação percentual de -42,7 (-44,3 a -0,4) (Tabela 2-3 e Figura 2 -2). Para adultos, a maior variação percentual foi observada no grupo etário de 50-69 anos, -48 (-49,9 a -0,5) (Tabela 2-4). ^46^

•As taxas de incidência padronizadas por idade de HIC por 100 mil foram 66 (56,1 a 77,9) em 1990 e 31,6 (26,6 a 37,3) em 2019, representando uma variação percentual de -52,1 (-53,5 a -0,5) (Tabela 2-3 e Figura 2 -2). Para adultos, a maior variação percentual foi observada no grupo etário de 50-69 anos, -53,2 (-55,3 a -0,5) (Tabela 2-4). ^46^

•As taxas de incidência padronizadas por idade de HSA por 100 mil foram 22,1 (18,6 a 26,3) em 1990 e 17,2 (14,4 a 20,6) em 2019, representando uma variação percentual de -22,2 (-25,1 a -0,2) (Tabela 2-3 e Figura 2 -2). Para adultos, a maior variação percentual foi observada no grupo etário de 15-49 anos, -23,6 (-27,8 a -0,2) (Tabela 2-4). ^46^

#### Mortalidade

•No estudo de base populacional sobre AVC conduzido em Matão, conhecido como MAPS, entre 2003-2004 e 2015-2016, a mortalidade aumentou em 50% (IRR 0,50; IC 95%, 0,31–0,94). A taxa de letalidade de 1 ano foi 26%. Aproximadamente 56% dos pacientes foram funcionalmente independentes, enquanto 7% tiveram um AVC recorrente. ^53^ Comparando com os resultados do primeiro estudo do registro de AVC de Matão, ^54^ não houve diferença significativa entre essas taxas.

•Um estudo avaliou a associação entre mortalidade por doenças cerebrovasculares, o índice de desenvolvimento humano municipal e a extensão da cobertura da saúde suplementar nas unidades federativas brasileiras entre 2004 e 2013. Os resultados mostraram que a porcentagem de cobertura da saúde suplementar no Brasil aumentou naquele período e apresentou uma relação inversa com mortalidade. Além disso, o coeficiente de correlação entre o índice de desenvolvimento humano municipal e as taxas de mortalidade ponderadas por causas mal definidas e padronizadas por idade mostrou associação inversa. Portanto, tanto o índice de desenvolvimento humano municipal quanto a cobertura de saúde foram associados com redução de mortalidade. ^37^

•Dados do Grupo GBD Brasil mostraram taxas de mortalidade por AVC padronizadas por idade por 100 mil de 137,8 (127,8 a 144) em 1990 e 58,1 (52,6 a 61,8) em 2019, representando variação percentual de -57,8 (-60,4 a -0,6) (Tabela 2-5 e Figura 2 -3). A maior variação percentual ocorreu em Goiás, -65,9 (-71,8 a -0,6), e a menor, no Maranhão, -22,7 (-37,2 a 0) (Tabela 2-5). Para os adultos, a maior variação percentual foi observada no grupo etário de 50-69 anos, -61 (-63,6 a -0,6) (Tabela 2-6). ^46^

•As taxas de mortalidade por AVCI padronizadas por idade por 100 mil foram 80 (72,5 a 84,3) em 1990 e 33,9 (29,7 a 36,6) em 2019, representando uma variação percentual de -57,6 (-60,9 a -0,5) (Tabela 2-5 e Figura 2 -3). Para adultos, a maior variação percentual foi observada no grupo etário de 15-49 anos, -66,1 (-70,5 a -0,6) (Tabela 2-6). ^46^

•As taxas de mortalidade por HIC padronizadas por idade por 100 mil foram 49,8 (47,1 a 52,2) em 1990 e 18,6 (17,3 a 19,8) em 2019, representando uma variação percentual de -62,6 (-65,3 a -0,6) (Tabela 2-5 e Figura 2 -3). Para adultos, a maior variação percentual foi observada no grupo etário de 50-69 anos, -63,1 (-65,7 a -0,6) (Tabela 2-6). ^46^

•As taxas de mortalidade por HSA padronizadas por idade por 100 mil foram 8 (7,3 a 8,4) em 1990 e 5,5 (5 a 5,9) em 2019, representando uma variação percentual de -30,3 (-36,2 a -0,2) (Tabela 2-6 e Figura 2 -3). Para adultos, a maior variação percentual foi observada no grupo etário de 15-49 anos, -40,8 (-46 a -0,3) (Tabela 2-6). ^46^

## Tendências Temporais

•As estimativas do GBD 2015 usadas para analisar a magnitude e as tendências das taxas de mortalidade e DALYs por doença cerebrovascular (CID-10: I60-I69) nas 27 unidades federativas do Brasil entre 1990 e 2015 mostraram que, a despeito do aumento no número absoluto de mortes por doença cerebrovascular, a proporção de mortes antes dos 70 anos de idade foi reduzida à metade entre 1990 e 2015. Nesse período, o risco de morte atribuível a AVC decresceu tanto para homens (-2,41% por ano) quanto para mulheres (-2,51% por ano). No entanto, a redução anual nas taxas de mortalidade ajustadas para idade, para ambos os sexos, desacelerou entre 2005 e 2015 quando comparada à do período de 1990-2005. Estados com SDI no tercil inferior apresentaram reduções anuais menos significativas para homens e mulheres (-1,23% e -1,84%, respectivamente) quando comparados àqueles com SDI no tercil médio (-1,94 e -2,22%, respectivamente) e àqueles com SDI no tercil superior (-2,85 e -2,82%, respectivamente). Além disso, houve diminuição nos YLDs nos estados, mas de maneira menos expressiva. ^55^

•Um estudo sobre as tendências de mortalidade por AVC ajustados por idade em adultos (30-69 anos) de regiões brasileiras entre 1996 e 2011 avaliou a influência dos métodos usados para corrigir as taxas de mortalidade nas estimativas finais.

As correções foram implementadas pela realocação de mortes com sexo ou idade não registrados, redistribuição de códigos de lixo e redistribuição de causas mal definidas de mortes, e alterou as taxas de mortalidade por AVC ajustadas por idade observadas significativamente em 1996 e 2011, pré e pós correção, respectivamente: 1) para homens: em 1996, 82,9 e 113,6; e, em 2011, 49,6 e 60,9; e 2) para mulheres: em 1996, 58,2 e 84,4; e, em 2011, 34,7 e 42.3.12. ^56^

•Um estudo avaliando diferenças regionais na transição de mortalidade e utilizando dados do SIM de 1990 a 2012 mostrou uma variação de -48,05% no coeficiente de mortalidade por AVC. A maioria das regiões apresentou redução nas taxas de mortalidade padronizadas por idade: -62% no Sudeste; -55,5% no Sul; -26,91% no Centro-Oeste; e -20,8% no Norte. Apenas no Nordeste ocorreu aumento (13,77%). ^57^

•Na cidade de São Paulo, de 1996 a 2011, 77.848 óbitos por AVC foram confirmados, 51,4% dos quais entre indivíduos de 35-74 anos de idade. Naquele período, taxas de mortalidade ajustadas por idade por doenças cerebrovasculares diminuíram 46,6% nos homens e 47,8% nas mulheres. Para os homens nas áreas de maior renda, a tendência decrescente foi constante; na área de renda média, houve um declínio marcado de 1996 a 2000, seguido por um de menor velocidade entre 2000 e 2011. Nas áreas de renda mais baixa, a variação percentual anual foi maior entre 1996 e 2002, com discreto declínio entre 2002 e 2011. Para as mulheres nas áreas de alta renda, houve um declínio marcado de 1996 a 2003, que foi menor na segunda metade do período; nas áreas de renda baixa e média, o declínio foi constante em todos os períodos. Para o período total, ambos os sexos e grupo etário de 35-74 anos, a diminuição nas taxas ajustadas por idade foi mais pronunciada entre os residentes da área de maior renda em comparação àqueles da área de menor renda. Esse mesmo padrão, mas com diferente magnitude de declínio, foi observado nos indivíduos com idade ≥75 anos em todas as áreas ao se comparar aos outros grupos etários, para os dois sexos. Além disso, a evolução temporal das razões entre as taxas ajustadas por idade de indivíduos de 35-74 anos vivendo em áreas de renda baixa e alta foi: para homens, de 1996 a 1998, a razão das taxas foi 2,03 e, de 2009 a 2011, 2,34. Para as mulheres, de 1996 a 1998, a razão das taxas foi 2,09 e, de 2009 a 2011, 2,58. A tendência das razões entre as taxas ajustadas por idade dessas áreas mostrou um crescimento da variação percentual anual de 1,4 (0,5 - 2,4) para os homens e de 1,1 (0,1 - 2,0) para as mulheres. ^58^

## Carga Global das Doenças Cerebrovasculares

### YLL

•Dados do Grupo GBD Brasil mostraram que as taxas de YLL por AVC padronizadas por idade por 100 mil foram 2.778,6 (2.659,5 a 2.879,2) em 1990 e 1.098,7 (1.025,8 a 1.153,7) em 2019, representando uma variação percentual de -60,5 (-62,7 a -0,6) (Tabela 2-7). Para adultos, a maior variação percentual foi observada no grupo etário de 50-69 anos, -61,7 (-64,3 a -0,6) (Tabela 2-8). ^46^

•As taxas de YLL por AVCI padronizadas por idade por 100 mil foram 1.211,1 (1.133,8 a 1.268,9) em 1990 e 479,7 (435,1 a 510,8) em 2019, representando uma variação percentual de -60,4 (-63,5 a -0,6) (Tabelas 2-7 e 2-8). Para adultos, a maior variação percentual foi observada no grupo etário de 15-49 anos, -66,6 (-71,1 a -0,6) (Tabela 2-8). ^46^

•As taxas de YLL por HIC padronizadas por idade por 100 mil foram 1.283,5 (1.227,7 a 1.351) em 1990 e 449,2 (423,3 a 472,5) em 2019, representando uma variação percentual de -65 (-67,6 a -0,6) (Tabelas 2-7 e 2-8). Para adultos, a maior variação percentual foi observada no grupo etário de 50-69 anos, -63,9 (-66,5 a -0,6) (Tabela 2-8). ^46^

•As taxas de YLL por HSA padronizadas por idade por 100 mil foram 284 (253,8 a 297,2) em 1990 e 169,8 (158,3 a 180,8) em 2019, representando uma variação percentual de -40,2 (-44,9 a -0,3) (Tabela 2-7 e 2-8). Para adultos, a maior variação percentual foi observada no grupo etário de 15-49 anos, -42,8 (-47,7 a -0,3) (Tabela 2-8). ^46^

### YLD

•Dados do Grupo GBD Brasil mostraram que as taxas de YLD por AVC padronizadas por idade por 100 mil foram 180,4 (133,2 a 228,8) em 1990 e 120,9 (88,7 a 152,6) em 2019, representando uma variação percentual de -33 (-34,8 a -0,3) (Tabelas 2-9 e 2-10). A maior variação percentual ocorreu em Rondônia, -39,5 (-43,8 a -0,4), e a menor, no Amapá, -23 (-27,3 a -0,2) (Tabela 2-9). Para adultos, a maior variação percentual foi observada no grupo etário de 50-69 anos, -38,7 (-40,9 a -0,4) (Tabela 2-10). ^46^

•As taxas de YLD por AVCI padronizadas por idade por 100 mil foram 122,2 (88 a 156,8) em 1990 e 81,2 (58,3 a 104) em 2019, representando uma variação percentual de -33,5 (-35,9 a -0,3) (Tabelas 2-9 e 2-10). Para adultos, a maior variação percentual foi observada no grupo etário de 50-69 anos, -39 (-42,1 a -0,4) (Tabela 2-10). ^46^

•As taxas de YLD por HIC padronizadas por idade por 100 mil foram 44,3 (32,3 a 56,6) em 1990 e 28,5 (20,7 a 36,9) em 2019, representando uma variação percentual de -35,7 (-38,8 a -0,3) (Tabelas 2-9 e 2-10). Para adultos, a maior variação percentual foi observada no grupo etário de 50-69 anos, -43,8 (-47,2 a -0,4) (Tabela 2-10). ^46^

•As taxas de YLD por HSA padronizadas por idade por 100 mil foram 13,9 (9,7 a 18,6) em 1990 e 11,2 (7,9 a 15) em 2019, representando uma variação percentual de -19,5 (-23,7 a -0,2) (Tabelas 2-9 e 2-10). Para adultos, a maior variação percentual foi observada no grupo etário de 50-69 anos, -19,9 (-26,1 a -0,1) (Tabela 2-10). ^46^

### DALY

•Dados do Grupo GBD Brasil mostraram que as taxas de DALYs por AVC padronizadas por idade por 100 mil foram 2.959 (2.829,6 a 3.063) em 1990 e 1.219,6 (1.142 a 1.285,5) em 2019, representando uma variação percentual de -58,8 (-61 a -0,6) (Tabelas 2-11 e 2-12). A maior variação percentual ocorreu em Santa Catarina, -67,8 (-71,1 a -0,6), e a menor, no Maranhão, -31,7 (-45,2 a -0,1) (Tabela 2-11). Para os adultos, a maior variação percentual foi observada no grupo etário de 50-69 anos, -60,3 (-62,8 a -0,6) (Tabela 2-12). ^46^

•As taxas de DALYs por AVCI padronizadas por idade por 100 mil foram 1.333,3 (1.244,5 a 1.403,6) em 1990 e 561 (510,4 a 599,8) em 2019, representando uma variação percentual de -57,9 (-61 a -0,6) (Tabelas 2-11 e 2-12). Para adultos, a maior variação percentual foi observada no grupo etário de 50-69 anos, -63,5 (-66,5 a -0,6) (Tabela 2-12). ^46^

•As taxas de DALYs por HIC padronizadas por idade por 100 mil foram 1.327,8 (1.274 a 1.397,3) em 1990 e 477,6 (450,9 a 503,8) em 2019, representando uma variação percentual de -64 (-66,6 a -0,6) (Tabelas 2-11 e 2-12). Para adultos, a maior variação percentual foi observada no grupo etário de 50-69 anos, -63,3 (-65,9 a -0,6) (Tabela 2-12). ^46^

•As taxas de DALYs por HSA padronizadas por idade por 100 mil foram 297,9 (267,5 a 312,7) em 1990 e 181 (169,4 a 192,8) em 2019, representando uma variação percentual de -39,2 (-43,8 a -0,3) (Tabelas 2-11 e 2-12). Para adultos, a maior variação percentual foi observada no grupo etário de 15-49 anos, -41,5 (-46,4 a -0,3) (Tabela 2-12). ^46^

## Utilização da Atenção à Saúde

### Admissões Hospitalares

•Em uma análise de série temporal, Katz *et al* . avaliaram a relação entre taxa de desemprego relacionada a AVC e internação hospitalar no Brasil em um período recente de 11 anos. Dados sobre hospitalizações mensais por AVC de março de 2002 a dezembro de 2013 foram extraídos da base de dados do Sistema Público de Saúde Brasileiro, revelando 1.581.675 internações por AVC no período. A taxa de desemprego diminuiu de 12,9% em 2002 para 4,3% em 2013, enquanto as internações por AVC aumentaram. Entretanto, o modelo ajustado não mostrou associação positiva entre taxa de desemprego e internação por AVC (coeficiente estimado = 2,40 ± 4,34; p=0,58). ^59^

•Utilizando dados do SIH, do SIM e do IBGE, Adami *et al* . analisaram taxas de mortalidade e incidência de hospitalizações relacionadas a AVC em brasileiros de 15-49 anos, por região e grupo etário, entre 2008 e 2012. Definiu-se AVC de acordo com a CID-10 (I60-I64). Mortalidade bruta e padronizada (OMS) e incidência de hospitalizações por 100 mil habitantes, estratificadas por região e grupo etário, foram estimadas. Os autores relataram 131.344 internações por AVC em brasileiros de 15-49 anos entre 2008 e 2012. No mesmo período, a taxa de hospitalizações estabilizou: 24,67 (IC 95%, 24,66 - 24,67) em 2008 e 25,11 (IC 95%, 25,10 - 25,11) em 2012 (β = 0,09, p = 0,692, r2 = 0,05). ^60^

•Dantas *et al* . realizaram um estudo para avaliar hospitalizações no sistema público brasileiro relacionadas a AVC de 2009 a 2016. Os autores selecionaram registros de hospitalização de acordo com os códigos de diagnóstico de AVC da CID-10. De 2009 a 2016, o número de internações subiu de 131.122 para 146.950, tendo o número absoluto de mortes hospitalares aumentado de 28.731 para 31.937. Idade mais jovem e sexo masculino mostraram associação significativa com a sobrevida do paciente. As taxas anuais de hospitalização e de mortalidade hospitalar ajustadas por idade caíram 11,8% e 12,6%, respectivamente, mas a taxa de letalidade aumentou para pacientes acima de 70 anos. ^61^

•Em estudo retrospectivo utilizando dados da base de dados do Sistema Público de Saúde Brasileiro e avaliando as seis principais causas de hospitalização de idosos de 2005 a 2015, AVC foi a terceira causa em 2015 para os dois gêneros e o grupo etário de 60-79 anos, com uma variação de -2,6. ^62^

## Indicadores de Atenção à Saúde e de Qualidade

•Uma análise da tendência de expansão da cobertura do PSF e internação por condições sensíveis à atenção primária no Rio de Janeiro entre 1998 e 2015 mostrou uma queda de 7,6% nas hospitalizações por doenças cerebrovasculares. ^63^

•Um estudo avaliando fatores sociodemográficos relacionados à falta de assistência hospitalar nas mortes por doença cerebrovascular no estado de São Paulo nos períodos de 1996-1998 e 2013-2015 mostrou que, dos 127.319 indivíduos que morreram por AVC naqueles períodos, 19.362 (15,2%) não receberam assistência hospitalar. No segundo período, maior risco de morte sem assistência foi identificado em indivíduos de cor amarela (RR = 1,48; IC 95%, 1,25-1,77) e menor risco naqueles de cor preta (RR = 0,86; IC 95%, 0,76-0,95), casados (RR = 0,70; IC 95%, 0,64-0,75) e residentes na cidade de São Paulo (RR = 0,92; IC 95%, 0,86-0,98). ^64^

•Um estudo de base hospitalar, avaliando 2.407 pacientes consecutivos (idade média, 67,7 ± 14,4 anos; 51,8% mulheres) admitidos em 19 hospitais de Fortaleza, mostrou que AVCI foi o subtipo mais frequente (72,9%), sendo seguido por hemorragia intraparenquimatosa (15,2%), HSA (6,0%), AIT (3%) e AVC indeterminado (2,9%). O tempo médio entre o aparecimento dos sintomas e a internação foi de 12,9 (3,8 - 32,5) horas. Hipertensão foi o fator de risco mais comum. Apenas 1,1% dos pacientes com AVCI receberam trombólise. O tempo médio entre a internação e a obtenção de neuroimagem foi de 3,4 (1,2 - 26,5) horas. ^65^

•Um estudo analisou os fatores que influenciam as tendências temporais dos indicadores de qualidade para manejo de AVCI em hospital terciário, certificado pela *Joint Commission International* como centro primário para AVC. Para tal, 551 pacientes com AVCI, que receberam alta de janeiro de 2009 a dezembro de 2013, foram avaliados. A mediana da idade foi 77,0 anos (intervalo interquartil, 64,0-84,0), sendo 58,4% homens. Dez indicadores de desempenho predefinidos, selecionados do programa *Get With the Guidelines-Stroke* , foram avaliados. Os indicadores de qualidade que melhoraram com o tempo foram o uso de terapia hipolipemiante (P = 0,02) e a orientação sobre AVC (P = 0,04). A mediana do desfecho composto cuidado ideal não melhorou consistentemente no período (P = 0,13). Após ajuste multivariável, apenas tratamento trombolítico (OR 2,06; P < 0,01), dislipidemia (OR 2,03; P < 0,01) e alta em ano de visita da *Joint Commission International* (OR 1.8, P < 0.01) (OR 1,8; P < 0,01) permaneceram como preditores de um índice de cuidado ideal igual ou superior a 85%. Os indicadores de qualidade com pior desempenho (anticoagulação para fibrilação atrial e redução de colesterol) foram semelhantes nos hospitais comunitários terciários e secundários. A medida geral de cuidado ideal não melhorou e foi influenciada por: receber alta em ano de visita da *Joint Commission International* , ter dislipidemia e ter recebido tratamento trombolítico. ^66^

•Outro estudo avaliou indicadores-chave de desempenho do Ministério da Saúde para unidades de AVC de dois centros de Curitiba e Botucatu, incluindo a porcentagem de pacientes admitidos nessas unidades, profilaxia de tromboembolismo venoso nas primeiras 48 horas de internação, pneumonia e mortalidade hospitalar por AVC e alta hospitalar em uso de antitrombóticos para pacientes sem mecanismo cardioembólico. O estudo mostrou que os dois centros internaram mais de 80% dos pacientes em suas unidades para AVC. A incidência de profilaxia de tromboembolismo venoso foi superior a 85% e a de pneumonia hospitalar foi inferior a 13%. A taxa de mortalidade hospitalar por AVC foi inferior a 15% e a de alta hospitalar em uso de antitrombóticos foi superior a 70%. ^67^

•Um estudo avaliou as taxas de mortalidade antes e depois da implementação de uma unidade cardiovascular dedicada a AVC no setor de emergência de um hospital público terciário em Porto Alegre. No período anterior à implementação da unidade vascular (2002 a 2005), foram incluídos 4.164 pacientes e, no período posterior à implementação (2007 a 2010), foram incluídos 6.280 pacientes. A taxa de letalidade geral por condições vasculares agudas diminuiu de 9% para 7,3% com a implementação (p = 0,002). As taxas de mortalidade hospitalar por síndrome coronariana aguda e por embolia pulmonar aguda caíram de 6% para 3,8% (p = 0,003) e de 32,1% para 10,8% (p < 0,001), respectivamente. Entretanto, a taxa de letalidade por AVC não diminuiu a despeito das melhorias nos indicadores de qualidade do cuidado para AVC. ^68^

•Um ensaio clínico randomizado por *cluster* avaliou o efeito de uma intervenção multifacetada de melhoria da qualidade referente à adesão às terapias baseadas em evidência para o cuidado de pacientes com AVCI agudo e AIT (incluindo manejo de caso, lembretes, *roadmap* e *checklist* para o plano terapêutico, materiais educativos, auditorias periódicas e relatórios de *feedback* para cada *cluster* da intervenção).

O estudo avaliou 1.624 pacientes de 36 hospitais, cobrindo todas as regiões brasileiras. O desfecho primário foi um desfecho composto de adesão ao escore para AVCI agudo e às medidas de desempenho para AIT, e os desfechos secundários incluíram um desfecho composto ‘tudo-ou-nada’ de adesão às medidas de desempenho. A idade média geral dos pacientes arrolados no estudo foi 69,4 ± 13,5 anos, e 913 (56,2%) eram homens. As médias do desfecho composto de adesão ao escore e às 10 medidas de desempenho dos hospitais do grupo ‘intervenção’ e do grupo controle foram 85,3% ± 20,1% e 77,8% ± 18,4%, respectivamente, com diferença média de 4,2% (IC 95%, -3,8% a 12,2%). Como desfecho secundário, 402 de 817 pacientes (49,2%) dos hospitais do grupo ‘intervenção’ receberam todas as terapias para as quais eram elegíveis, enquanto, nos do grupo controle, 203 de 807 (25,2%) receberam aquelas terapias (OR, 2,59; IC 95%, 1,22 - 5,53; P = 0,01). A intervenção não resultou em significativo aumento de adesão às terapias baseadas em evidência em pacientes com AVCI agudo ou AIT. Entretanto, ao usar a abordagem ‘tudo-ou-nada’, a intervenção resultou em melhoria da adesão às terapias baseadas em evidência e melhores taxas de trombólise. ^69^

## Genética/História Familiar

### Incapacidade

•Benseñor *et al* . analisaram um inquérito epidemiológico de base domiciliar (PNS - 2013), com uma amostra representativa nacional, para avaliar o número absoluto e as taxas de prevalência de AVC e de incapacidade por AVC. Foram estimados 2.231.000 indivíduos com AVC e 568.000 com incapacidade grave por AVC. As estimativas de prevalências pontuais de AVC foram 1,6% e 1,4% para homens e mulheres, respectivamente. A prevalência de incapacidade por AVC foi 29,5% para homens e 21,5% para mulheres. As taxas de prevalência de AVC aumentaram com a idade, o baixo nível educacional e nos residentes de áreas urbanas, mas não apresentaram diferença de acordo com a cor da pele autodeclarada. O grau de incapacidade por AVC não diferiu estatisticamente de acordo com sexo, raça, nível educacional ou local de residência. ^50^

•Em estudo subsequente baseado na PNS - 2013, o acesso a reabilitação foi deficiente: apenas 0,27% dos indivíduos foram submetidos a fisioterapia para AVC e 0,12% realizaram algum tipo de tratamento de reabilitação, o que compromete o *status* funcional do usuário. ^70^

•Carvalho-Pinto *et al* ., conduzindo estudo observacional retrospectivo, coletaram dados dos prontuários médicos e de visitas domiciliares após AVC de pacientes seguidos em uma unidade de atenção primária em Belo Horizonte, entre maio de 2013 e maio de 2014. Os dados incluíram condição de saúde, assistência recebida após AVC, fatores pessoais e ambientais, funcionalidade e incapacidade organizados de acordo com a estrutura conceitual da Classificação Internacional de Funcionalidade, Incapacidade e Saúde. A maioria dos participantes apresentou boa percepção da própria habilidade manual (2,39 ± 2,29 logits) e limitada habilidade para caminhar (88%), sendo capaz de melhorar a velocidade da marcha natural, apresentou mudança de equilíbrio (51,43%) e mobilidade funcional (54,16%) com risco de queda, e teve percepção negativa da própria qualidade de vida (escore médio de 164,21 ± 35,16 pontos na SSQOL-Brasil). ^71^

### Custo

•Um estudo de custo-efetividade avaliando os trombolíticos no Brasil relatou que, para homens e em 1 ano, o custo do tratamento com rt-PA foi maior do que o custo do tratamento conservador. Esse resultado deve-se principalmente ao custo da medicação. Parte desse custo adicional é compensado pelo menor custo da reabilitação e menor perda de produtividade nos primeiros 2 anos, pois os pacientes tratados com rt-PA apresentaram menos sequelas do que aqueles que receberam tratamento conservador. Depois do segundo ano que se segue a um AVC, para os dois sexos, o tratamento com rt-PA (alteplase), considerando-se os custos diretos e indiretos, começou a ter menor custo em comparação ao conservador. Daí para a frente, o custo adicional da medicação começa a ser mais do que compensado pela menor perda de produtividade e menores custos com seguridade social e reabilitação do paciente. ^72^

## Genética/História Familiar

•Distúrbios mitocondriais, como síndrome MELAS, podem ser responsáveis por até um terço dos AVC criptogênicos em pacientes jovens. Episódios *stroke-like* podem ocorrer em pacientes de qualquer idade com síndrome MELAS e em cerca de 50% daqueles com mutação A3243G do DNA mitocondrial. Essa mutação foi relatada em aproximadamente 80% dos casos de MELAS. Outras mutações do DNA mitocondrial, como T3271C, foram descritas. Em um estudo conduzido por Conforto *et al.* , as duas mutações foram avaliadas em três grupos de pacientes com idade inferior a 46 anos (grupo 1: 15 pacientes com AVC criptogênico; grupo 2: 3 pacientes com diagnóstico de síndrome MELAS, incluindo episódios *stroke-like* ; grupo 3: 20 indivíduos saudáveis).

A mutação A3243G estava ausente em todos os indivíduos dos grupos 1 e 3, mas presente em todos do grupo 2. Esses resultados, portanto, não corroboram o rastreio dessas mutações para o diagnóstico de formas oligossintomáticas de MELAS em casos de AVC criptogênico na ausência de outras características da síndrome. ^73^

## Prevenção

•O Estudo PURE examinou as taxas e preditores do uso de medicação de prevenção secundária baseada em evidência (IECA/BRA, antiagregantes plaquetários, estatinas e betabloqueadores) em pacientes com doenças cardiovasculares, incluindo DAC e AVC, em países da América do Sul, entre os quais o Brasil. O estudo mostrou que um menor número de pacientes com AVC recebeu antiagregantes plaquetários (24,3%), IECA/BRA (37,6%) e estatinas (9,8%) em comparação àqueles com DAC (30,1%, 36,0% e 18,0%, respectivamente). Essa subutilização de terapias em pacientes com AVC variou substancialmente entre os países, tendo a Colômbia o menor uso (sem prescrição de estatinas). Quando pacientes com DAC e AVC foram combinados, a proporção de uso de antiagregantes plaquetários foi mais alta no Chile (38,1%) e mais baixa na Argentina (23,0%). O uso de IECA/BRA e estatinas foi maior no Brasil (46,4% e 26,4%, respectivamente) e menor na Colômbia (26,4% e 1,4%, respectivamente). Entre os participantes com DAC e AVC, o uso foi maior naqueles com maior nível educacional se comparados àqueles sem nenhuma educação, educação primária ou desconhecida [35,6% vs. 23,6%, respectivamente, para antiagregantes plaquetários (p = 0,002); 20,6% vs. 10,9%, respectivamente, para estatinas (p = 0,0007)]. Ex-fumantes com DAC ou AVC tiveram maior probabilidade de receber terapias comprovadas do que fumantes atuais ou aqueles que nunca fumaram [35,2% vs. 26,6% e 27,7%, respectivamente, para antiagregantes plaquetários (p = 0,039); 19,9% vs. 10,6% e 13,0%, respectivamente, para estatinas (p = 0,004)]. Apenas 4,1% dos pacientes receberam todas as 4 terapias (IECA/BRA, antiagregantes plaquetários, estatinas e betabloqueadores), sendo a maior taxa do Brasil (5,5%) e a menor, da Colômbia (0,5%) (p = 0,02). Além disso, observou-se que 30% dos brasileiros com AVC não usam qualquer medicação. ^74^

•O IMPACT-AF, um estudo randomizado com *cluster* para melhorar o tratamento com anticoagulantes em pacientes com fibrilação atrial, uma importante causa de AVC, conduzido na Argentina, no Brasil, na China, na Índia e na Romênia, mostrou que dois-terços dos pacientes estavam em uso de anticoagulação oral na linha de base, 83% usavam um antagonista da vitamina K e 15% usavam um NOAC. Pacientes do Brasil mais frequentemente usavam anticoagulação oral na linha de base (91%), enquanto apenas 38% dos pacientes da China o faziam. De todos os pacientes em uso de antagonistas da vitamina K no Brasil, 40,3% apresentavam INR entre 2 e 3 anteriormente à visita de base. ^75^

## Conscientização, Tratamento e Controle

•Vários estudos mostraram alarmante falta de conhecimento sobre os fatores de risco para AVC, seu tratamento e reconhecimento dos seus sintomas como uma emergência médica. Em um estudo de base comunitária, Pontes-Neto *et al* . entrevistaram indivíduos em locais públicos de quatro importantes cidades no Brasil entre julho de 2004 e dezembro de 2005, usando um questionário estruturado aberto em português, baseado na apresentação de um caso típico de AVC agudo domiciliar. Os autores identificaram 28 termos diferentes em português para denominar AVC. Quanto aos entrevistados, 22% deles não reconheceram qualquer sinal de alarme de AVC. Apenas 34,6% dos entrevistados responderam corretamente quando perguntados sobre o número de telefone de emergência no Brasil (#192). Apenas 51,4% dos entrevistados relataram que chamariam uma ambulância para um familiar com sintomas de AVC. ^76^

•Falavigna *et al* . usaram um questionário fechado e autoadministrado para avaliar o conhecimento sobre AVC entre 952 residentes da cidade de Caxias do Sul. Baixa renda e baixo nível educacional foram preditores independentes da incapacidade de reconhecer que o AVC afeta o cérebro. Renda mais baixa e idade < 50 anos foram preditores independentes da falta de conhecimento sobre os fatores de risco para AVC. ^77^

•Em estudo transversal de base comunitária, Pitton Rissardo *et al* . aplicaram um inquérito de conhecimento sobre AVC em uma amostra de conveniência composta por 633 passantes de uma praça pública na cidade de Santa Maria, Rio Grande do Sul, de dezembro de 2015 a outubro de 2016. Dos respondentes, 33% informaram corretamente o significado do acrônimo ‘AVC’, o termo mais recomendado para designar ‘acidente vascular cerebral’ pela Sociedade Brasileira de Doenças Cerebrovasculares. Cerca de 30% dos respondentes localizaram incorretamente o AVC no coração. Apenas 50% dos respondentes identificaram corretamente um sinal de alarme de AVC. Indivíduos de nível educacional mais alto apresentaram maior probabilidade de chamar uma ambulância para um familiar com sintomas de AVC. ^78^

•Recentemente, tem havido várias iniciativas para promover a conscientização do público sobre AVC no Brasil, em especial através de campanhas anuais por ocasião do Dia Mundial do AVC (29 de outubro), conduzidas pela Organização Mundial do AVC. A despeito desses esforços, apenas 30-40% dos pacientes com AVC são hospitalizados nas primeiras 4 horas após início dos sintomas. ^79^

## Pesquisa Futura

•O portfólio de pesquisa brasileira em neurologia vascular evoluiu muito nos últimos anos, como ilustra a fundação da Rede Brasil AVC. No entanto, ainda há inúmeras oportunidades para melhoria. Os estudos comunitários mais expressivos sobre prevalência e incidência de AVC são provenientes principalmente de duas cidades. Embora representem uma importante realização para a epidemiologia do AVC, avaliação mais abrangente se faz necessária, compreendendo a representação de todas as regiões geográficas brasileiras, das diversas culturas, dos níveis de renda e das etnias.

•Além disso, há limitações para os estudos relacionadas à identificação do AVC usando os códigos da CID. Não é incomum que um código mais amplo seja utilizado na admissão e, por não ser ajustado durante a hospitalização, esse código não representa o verdadeiro subtipo de AVC (e.g., um AVCI pode ser codificado como AVC não específico ou até AIT). Com o advento das tecnologias *big data* (e.g., mineração de dados textuais), informação clínica adicional proveniente dos registros de admissão ou alta pode ser uma fonte confiável de contrarreferência, confirmando ou corrigindo um certo código.

## 3. DOENÇA ARTERIAL CORONARIANA AGUDA E CRÔNICA

### CID-9-CM 410 a 414; CID-10 I10 a I25

**Ver Tabelas 3-1 até 3-3 e Figuras 3-1 até 3-20**

**Table:** Abreviaturas usadas no Capítulo 3

ACCEPT/SBC	Registro Brasileiro da Prática Clínica nas Síndromes Coronarianas Agudas da Sociedade Brasileira de Cardiologia
AAS	Ácido Acetilsalicílico
BRACE	Registro Brasileiro de Síndromes Coronarianas Agudas (do inglês, *Brazilian Registry in Acute Coronary Syndromes* )
BRIDGE-ACS	Brazilian Intervention to Increase Evidence Usage in Acute Coronary Syndromes
BYPASS	Registro Brasileiro de Pacientes Adultos Submetidos a Cirurgia Cardiovascular
CENIC	Central Nacional de Intervenções Cardiovasculares
CRVM	Cirurgia de Revascularização do Miocárdio
DAC	Doença Arterial Coronariana
DALYs	Anos de vida perdidos ajustados por incapacidade (do inglês, *Disability-Adjusted Life-Years* )
DATASUS	Departamento de Informática do Sistema Único de Saúde
DCV	Doença Cardiovascular
DIC	Doença Isquêmica do Coração
ERICO	Estudo de Registro de Insuficiência Coronariana
GBD	Global Burden of Disease
IAM	Infarto Agudo do Miocárdio
IAMCSST	Infarto Agudo do Miocárdio com Supradesnível do Segmento ST
IAMSSST	Infarto Agudo do Miocárdio sem Supradesnível do Segmento ST
IC	Intervalo de Confiança
ICP	Intervenção Coronária Percutânea
II	Intervalo de Incerteza
MASS	Medicine, Angioplasty, or Surgery Study
OR	Odds Ratio
PNS	Pesquisa Nacional de Saúde
RBSCA	Registro Brasileiro de Síndrome Coronariana Aguda
REPLICCAR-I	Registro Paulista de Cirurgia Cardiovascular
RESISST	Registro Soteropolitano de Infarto Agudo do Miocárdio com Supra de ST
SCA	Síndrome Coronariana Aguda
SCC	Síndrome Coronariana Crônica
SDI	Índice Sociodemográfico (do inglês, *Sociodemographic Index* )
SIH	Sistema de Informações Hospitalares
SUS	Sistema Único de Saúde
VICTIM	*Via Crucis* para o Tratamento do Infarto do Miocárdio
YLDs	Anos vividos com incapacidade (do inglês, *Years Lived with Disability* )
YLLs	Anos potenciais de vida perdidos (do inglês, *Years of Life Lost* )

**Tabela 3-1:** – Número de casos, taxas de prevalência de doença arterial coronariana padronizadas por idade (por 100 mil) em 1990 e 2019, e variação percentual das taxas no período, no Brasil e nas unidades federativas.

Local	1990		2019		Variação percentual (II 95%)
	Número (II 95%)	Taxa (II 95%)	Número (II 95%)	Taxa (II 95%)	
Acre	2744 (2323,2;3238,3)	1698,7 (1440,1;2001,1)	10165 (8757,7;11875,3)	1669,7 (1423,7;1950,1)	-1,7(-5,2;1,9)
Alagoas	22487,5 (18952,7;26631,9)	1728,3 (1461,3;2055)	51311,8 (43528,7;60808,3)	1632 (1382,7;1929,3)	-5,6(-9;-1,9)
Amapá	1435,5 (1221,4;1689,8)	1490,3 (1263,4;1756)	7640,2 (6545,1;9014,1)	1495,6 (1275,1;1755,7)	0,4(-3,2;4,2)
Amazonas	11671,4 (9935,2;13793,1)	1553,6 (1317,5;1820,5)	44391,2 (38294,2;52043,1)	1568,5 (1347,3;1835,6)	1(-2,5;4,5)
Bahia	108605,1 (92013,7;128033,5)	1657,4 (1405,6;1953,6)	276650,7 (236718;324478,5)	1729,9 (1472,1;2035,1)	4,4(0,4;8,6)
Brasil	1480208,9 (1271844,8;1725493,1)	1727,7 (1482,4;2017,9)	4003895,6 (3449807,8;4672110,4)	1708,7 (1465,5;1994,4)	-1,1(-2,6;0,5)
Ceará	63391,6 (54165,9;74916,2)	1587,7 (1354,7;1877,1)	152544,4 (130121,9;178195,9)	1539,8 (1311;1803,3)	-3(-6,6;0,3)
Distrito Federal	8632,3 (7371,6;10260,4)	1490 (1278;1748,5)	38394,4 (32946,6;44953,8)	1470,1 (1260,8;1707,5)	-1,3(-4,5;1,9)
Espírito Santo	23779,7 (20442,8;28015,9)	1678,2 (1438,7;1965,6)	72337,9 (62145,1;84311,7)	1669,9 (1435,9;1948,4)	-0,5(-3,8;3)
Goiás	31650 (26872,3;37325,5)	1580,9 (1340,4;1867,5)	112766,7 (95362,9;132919,9)	1634,4 (1385,1;1919,7)	3,4(-0,3;7)
Maranhão	39708,7 (33413,1;47193,9)	1581,3 (1332,7;1880,6)	100754,5 (85612,3;119041,2)	1549,1 (1312,2;1833)	-2(-5,7;1,7)
Mato Grosso	12613,1 (10808,1;14884,5)	1681,9 (1435,6;1982,7)	53895 (46476,4;63120,2)	1662,6 (1429,2;1939,3)	-1,1(-4,7;3)
Mato Grosso do Sul	14979,8 (12842,7;17679,7)	1752,6 (1499,2;2061,5)	53091,8 (45889,2;62249,5)	1820,7 (1570,5;2125)	3,9(0,3;7,6)
Minas Gerais	176140,3 (151248,2;205339,7)	1867,1 (1607;2172,4)	496702,3 (431006,5;576498,2)	1878,2 (1630,8;2178,4)	0,6(-2,4;4)
Pará	30506 (25805,7;36277,5)	1499,4 (1265,2;1775,8)	104725,1 (88987,6;123975,2)	1528,7 (1298;1810)	2(-1,6;5,7)
Paraíba	39766,3 (33685,5;46817,8)	1746,8 (1479,4;2053,9)	75707,5 (64303,6;89064,2)	1602,8 (1358,5;1890,4)	-8,2(-11,3;-5)
Paraná	82034,4 (70312,4;96269)	1763,5 (1512,1;2066,3)	232096 (199173,2;272919)	1749,9 (1503,9;2050,1)	-0,8(-4,1;2,6)
Pernambuco	73242,6 (62254,4;86784,7)	1652,2 (1410,2;1949,7)	165021,6 (141125,5;193595,4)	1660 (1410,8;1940,3)	0,5(-3,1;4,2)
Piauí	22358,8 (18937,4;26398,4)	1636,6 (1394,8;1927,9)	59627,3 (50948,3;69838)	1591,7 (1360,5;1865,5)	-2,7(-6,2;1,3)
Rio de Janeiro	158350,6 (135303,3;186081,8)	1709,7 (1466,3;1999,9)	370396,8 (317368,2;433395,5)	1649,7 (1415,8;1922,9)	-3,5(-6,7;0)
Rio Grande do Norte	25565,4 (21696,6;30114)	1625,3 (1379,4;1913,1)	61672,6 (52687,4;72269,3)	1603,6 (1369,5;1884,2)	-1,3(-5,1;2,2)
Rio Grande do Sul	112630,6 (96716,3;132207,6)	1771,6 (1523;2061)	271808,2 (233542,4;319107,2)	1740,4 (1502,8;2036,2)	-1,8(-4,7;1,5)
Rondônia	5685,1 (4817,2;6785,4)	1660,6 (1412;1967,5)	23529,2 (19995,2;27786,7)	1557,3 (1319,6;1833,5)	-6,2(-10,2;-2,2)
Roraima	976,7 (827,5;1160,6)	1682,3 (1423,7;1991,1)	5879,7 (5000,2;6970,1)	1583,5 (1344,1;1873,9)	-5,9(-9;-2,6)
Santa Catarina	42265,2 (36505,9;49567,5)	1727,3 (1479,7;2012,7)	142261,9 (121996,8;165916,3)	1752,8 (1509,2;2031,4)	1,5(-2;4,9)
São Paulo	349725,3 (301314,8;407755,5)	1818,8 (1563;2117,4)	961471 (831096;1119060,5)	1772,4 (1535,4;2056,1)	-2,6(-5,9;0,8)
Sergipe	13056,6 (11064,9;15423,9)	1688,7 (1435;1987,3)	37283,3 (32043,6;43768,5)	1683,7 (1442,6;1980,3)	-0,3(-3,7;3,3)
Tocantins	6206,4 (5271,1;7415,6)	1552,9 (1320,7;1839,1)	21769,6 (18644,8;25664,3)	1546,3 (1323,8;1824,2)	-0,4(-4;3,1)

*Fonte: Dados derivados do estudo Global Burden of Disease 2019, Institute for Health Metrics and Evaluation, University of Washington. ^46^*

**Tabela 3-2:** – Número de mortes, taxas de mortalidade por doença arterial coronariana padronizadas por idade (por 100 mil) em 1990 e 2019, e variação percentual das taxas no período, no Brasil e nas unidades federativas.

Local	1990		2019		Variação percentual (II 95%)
	Número (II 95%)	Taxa (II 95%)	Número (II 95%)	Taxa (II 95%)	
Acre	138,6 (128,8;147,8)	113,1 (103,5;121,1)	349,4 (313,2;385,3)	65,3 (57,7;71,8)	-42,3(-48;-35,7)
Alagoas	1502,2 (1377,7;1611,1)	128,7 (116,9;138,6)	2785,4 (2455,8;3121,3)	89,1 (78,6;99,8)	-30,7(-39,3;-20,4)
Amapá	78,5 (72,4;84,2)	101 (91,1;108,3)	276,2 (247,5;301,5)	59,9 (52,6;65,5)	-40,7(-46;-35,6)
Amazonas	695,4 (633,5;755,7)	124,3 (112,2;134,8)	1468,3 (1276,5;1651,5)	55,7 (47,9;62,8)	-55,2(-60,4;-49,4)
Bahia	6853,8 (6096,3;7577,2)	113,9 (100,4;125,6)	11441,8 (9797,9;13115,2)	70,2 (60,4;80,3)	-38,3(-47,5;-27)
Brasil	117247 (111650,1;121246,9)	157,9 (146,9;164)	171246,3 (156180;180511,2)	74,9 (67,9;79,1)	-52,6(-54,9;-50,3)
Ceará	3607,4 (3080,1;4121,2)	94,1 (80;107,2)	7663,8 (6453,5;8924,8)	78 (65,6;90,8)	-17,1(-31;1,4)
Distrito Federal	651,4 (589,4;727,4)	189,6 (171,6;207,4)	1273,5 (1126,9;1425,3)	67,3 (58,9;75,3)	-64,5(-68,7;-59,7)
Espírito Santo	1673,8 (1588,5;1743,2)	147,3 (136,8;154,5)	3276,1 (2878,7;3654)	79,1 (69,7;88,2)	-46,3(-51,9;-40,7)
Goiás	2416,9 (2113,1;2844,4)	149,9 (131,3;174,4)	4936,2 (4136;5760)	75 (62,8;87,1)	-50(-58,6;-40,2)
Maranhão	3075,1 (2579,4;3583,6)	131,4 (109,2;152,5)	6670,2 (5691,2;7767,9)	104 (88,9;121)	-20,9(-35,2;-0,8)
Mato Grosso	915,1 (813;1018,8)	151,1 (134,5;166)	1963,2 (1759,7;2180,3)	64,5 (57,2;71,8)	-57,3(-61,9;-51,2)
Mato Grosso do Sul	1149,3 (1076,6;1218)	165,2 (151,3;175,7)	2165,9 (1920,3;2425,8)	78 (68,8;87,1)	-52,8(-57,8;-47,3)
Minas Gerais	12398,7 (11565,9;13348)	161 (147,4;173,3)	15629,4 (13736,3;17428,7)	59,2 (52;66)	-63,2(-67,3;-59,4)
Pará	2242 (1988,9;2500,7)	138,7 (122,8;153,4)	4412,1 (3894,1;4912,4)	66,5 (58,4;74)	-52,1(-58,1;-45,1)
Paraíba	2460,3 (2223,5;2681,6)	113 (101,3;123,2)	4020,9 (3487,9;4554,8)	81,7 (71,4;92,4)	-27,7(-37,2;-17,2)
Paraná	6738 (6451,3;7004,8)	180,6 (168,5;188,5)	9253,8 (8185,6;10317,7)	73,7 (65;82,2)	-59,2(-63,4;-54,6)
Pernambuco	5902,4 (5528,3;6218,4)	150,7 (139;159,2)	9852,3 (8776,8;10963,6)	101,7 (90,2;113,1)	-32,5(-39,8;-24,8)
Piauí	1420,5 (1287,7;1539,2)	125,4 (111,9;137,3)	2681,6 (2321,6;2993,7)	69,6 (60,6;77,8)	-44,5(-51,1;-37,4)
Rio de Janeiro	16553 (15826,5;17095,4)	207 (193,3;215)	19404,4 (17334,6;21411,9)	88,3 (78,9;97,4)	-57,3(-61,1;-53,1)
Rio Grande do Norte	1692,1 (1503,1;1855,9)	113,3 (100,1;124,4)	2945,9 (2469,4;3436,9)	74,1 (62,2;86,5)	-34,6(-44,6;-23)
Rio Grande do Sul	9356,6 (8842,8;9711,2)	170,1 (157,9;177,7)	10964,6 (9615,9;12195,2)	71,2 (62,4;79,1)	-58,2(-62,6;-53,9)
Rondônia	382,6 (340,8;423,2)	192,2 (175,4;207,2)	1071,8 (936,3;1224,9)	76,7 (66,9;87,5)	-60,1(-65,5;-54,3)
Roraima	59,8 (54,7;64,9)	156,8 (144,6;167,7)	205,3 (186,3;224,3)	70,6 (63,1;77)	-55(-59,2;-50,7)
Santa Catarina	3357,7 (3167,7;3535,1)	167,7 (156;177,4)	5223,5 (4616,9;5823,5)	69,7 (61,4;77,6)	-58,4(-62,7;-53,5)
São Paulo	30795 (29080,1;32357,1)	198,6 (183,3;209,7)	38670,9 (34008,3;42716)	74,8 (65,4;82,4)	-62,4(-65,9;-58,3)
Sergipe	744,1 (678;812,9)	121,6 (110,4;132,7)	1550,8 (1322,4;1775,1)	71 (60,4;81,2)	-41,6(-49,6;-32,6)
Tocantins	386,8 (342,1;434,8)	140,7 (125;156,7)	1088,6 (945,9;1247,1)	80,7 (70,2;92)	-42,7(-50,7;-33,9)

*Fonte: Dados derivados do estudo Global Burden of Disease 2019, Institute for Health Metrics and Evaluation, University of Washington. ^46^*

**Tabela 3-3:** – Número de DALYs, taxas de DALYs por doença arterial coronariana padronizadas por idade (por 100 mil) por doença arterial coronariana em 1990 e 2019, e variação percentual das taxas no período, no Brasil e nas unidades federativas.

Local	1990		2019		Variação percentual (II 95%)
	Número (II 95%)	Taxa (II 95%)	Número (II 95%)	Taxa (II 95%)	
Acre	3404,1 (3139,6;3645,5)	2093,7 (1937,9;2235,9)	8056 (7281,6;8875,9)	1271,5 (1148,8;1398,9)	-39,3(-45,8;-31,6)
Alagoas	35988 (33355,1;38794,7)	2642,2 (2445,9;2836,5)	64183,2 (57031,8;72062,4)	1957,6 (1742,2;2194,8)	-25,9(-35,5;-15,1)
Amapá	1872,1 (1729,6;2006,7)	1865,3 (1728,2;1990,5)	6757,6 (6131;7326,5)	1234,7 (1114,1;1343,3)	-33,8(-39,7;-27,6)
Amazonas	16169,9 (14743,4;17642,5)	2166,7 (1982,4;2353)	32551,7 (28674,8;36563,6)	1106,3 (973,8;1241,8)	-48,9(-55,3;-42)
Bahia	157006,3 (140109,9;174887)	2308,3 (2064,6;2565,5)	248479,4 (212225,9;284940,9)	1523,6 (1300,4;1747,9)	-34(-44,6;-20,8)
Brasil	2793361,6 (2696368,8;2875301,9)	3117,1 (2989,1;3214)	3721023,5 (3507748;3892657,2)	1563,3 (1472,1;1636,1)	-49,8(-52,2;-47,6)
Ceará	75826 (65771,4;86754,3)	1860,5 (1616,9;2128,9)	155657,8 (131957,2;182735,2)	1556,1 (1322,1;1827,7)	-16,4(-31,5;4,2)
Distrito Federal	18350,9 (16471,6;20617,2)	3247,2 (2939,1;3588,9)	28941,1 (25769,2;32405,2)	1147,9 (1017,5;1278,2)	-64,6(-69,1;-59,5)
Espírito Santo	39315,3 (37642,8;40842,8)	2723,4 (2595,9;2826,1)	71428,3 (63301,5;80094,9)	1616,2 (1434,6;1806,8)	-40,7(-47,6;-33,7)
Goiás	63256,2 (54931,7;74559,1)	2936,4 (2571,5;3439,6)	115723,9 (98226,5;135146,5)	1606,9 (1366,6;1870,7)	-45,3(-55,3;-32,9)
Maranhão	81890,1 (68565,6;95781,4)	3036,4 (2553,7;3520,6)	144195,8 (121818,6;171277)	2156,5 (1826,3;2545,6)	-29(-42,8;-9,2)
Mato Grosso	24136,6 (21151,7;27140,8)	2916,9 (2600,3;3235,1)	46201,2 (41637,4;51223,9)	1349,3 (1217,7;1495,9)	-53,7(-59,4;-45,9)
Mato Grosso do Sul	29159,4 (27471,5;30999,4)	3186,1 (2989,1;3385,4)	48763,9 (43588,8;54656,4)	1625,7 (1454,1;1821,7)	-49(-54,8;-42,4)
Minas Gerais	294064,9 (275086,6;318894,3)	3036,3 (2834,2;3277,7)	332544,7 (299215,8;369571)	1250,8 (1124,8;1388,1)	-58,8(-63,6;-54,1)
Pará	52443,4 (46628,1;58642,3)	2533,3 (2253,3;2811,5)	100474,6 (89523,2;111319,9)	1396,9 (1245,5;1548,5)	-44,9(-52,4;-35,9)
Paraíba	52628,5 (47840,1;57117,1)	2310,8 (2096;2507,7)	82129,3 (72301,8;93181,2)	1736,4 (1528,3;1969,4)	-24,9(-35,2;-13,2)
Paraná	161480,7 (156250,5;167183)	3367,8 (3231,3;3489)	200724,6 (177943,7;223894)	1495,3 (1327,8;1670,7)	-55,6(-60,4;-50,2)
Pernambuco	135058,1 (128395,1;141504,1)	2984,4 (2825,6;3128,2)	220562,4 (197651;245799)	2163,2 (1936,7;2404,8)	-27,5(-35,5;-18,7)
Piauí	33451,5 (30836,8;36206,4)	2409,3 (2204,1;2608,6)	55492,6 (49570,2;61446,8)	1468,9 (1312,9;1626,4)	-39(-46,4;-31)
Rio de Janeiro	407746,1 (394302,6;419720,2)	4171,5 (4011,9;4299,1)	427010,3 (384691,2;474730,2)	1899,1 (1712,3;2108,5)	-54,5(-58,9;-49,4)
Rio Grande do Norte	34665,5 (31015,3;38186,2)	2189,7 (1958;2411,2)	61578,7 (52237,5;71777,6)	1573 (1335,7;1833,8)	-28,2(-39,9;-14,1)
Rio Grande do Sul	218591,9 (209049,2;225505,3)	3319,3 (3158,8;3432,1)	220881,2 (195868,6;245433,1)	1424,1 (1263,9;1583,3)	-57,1(-61,7;-52,5)
Rondônia	11257,2 (9744,8;12593,1)	3362,8 (3058,3;3657,7)	24715,8 (21437,5;28409,9)	1558,4 (1359,9;1783,2)	-53,7(-60,3;-45,8)
Roraima	1722,5 (1541,1;1885,8)	2850,2 (2648,8;3067)	5071 (4637,9;5541,6)	1319,7 (1203;1436,8)	-53,7(-58,3;-48,3)
Santa Catarina	78531,6 (74508,7;82564,6)	3139,7 (2968,8;3300,9)	112745,8 (100343,6;125606,6)	1372,9 (1220,7;1526,7)	-56,3(-61;-51,2)
São Paulo	739272,4 (701242,6;775952,1)	3716,7 (3515,9;3898)	848171,4 (760424,2;937315,5)	1552,8 (1387,5;1711,6)	-58,2(-62,4;-53,7)
Sergipe	16140,7 (14704,3;17710,1)	2146,4 (1961,4;2348)	33976,8 (29494,2;39095,9)	1483,8 (1289,8;1703,2)	-30,9(-40,9;-19,3)
Tocantins	9931,6 (8722,7;11149,1)	2511,8 (2230,3;2811,7)	24004,5 (20790;27711,9)	1649,1 (1433,6;1900)	-34,3(-44,4;-22,7)

*Fonte: Dados derivados do estudo Global Burden of Disease 2019, Institute for Health Metrics and Evaluation, University of Washington. ^46^*

*Fonte: Ministério da Saúde do Brasil – Sistema de Informações Hospitalares do Sistema Único de Saúde (SIH/SUS). ^95^*

**Figura 3-1:**
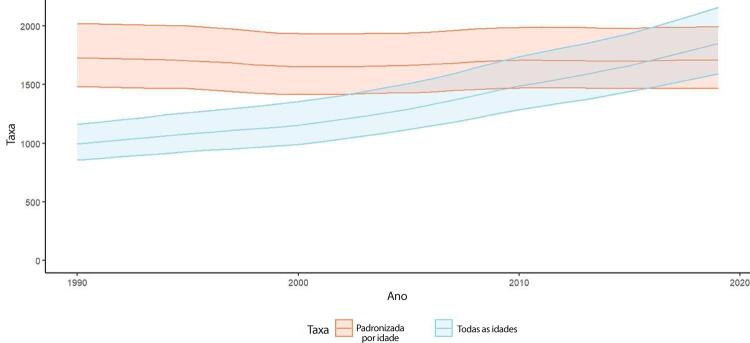
-Taxas de prevalência bruta e padronizada por idade (por 100 mil) de doença arterial coronariana no Brasil, 1990-2019. As áreas sombreadas mostram os intervalos de incerteza 95%.

Fonte: Dados derivados do estudo Global Burden of Disease 2019, Institute for Health Metrics and Evaluation, University of Washington. ^46^

**Figura 3-2:**
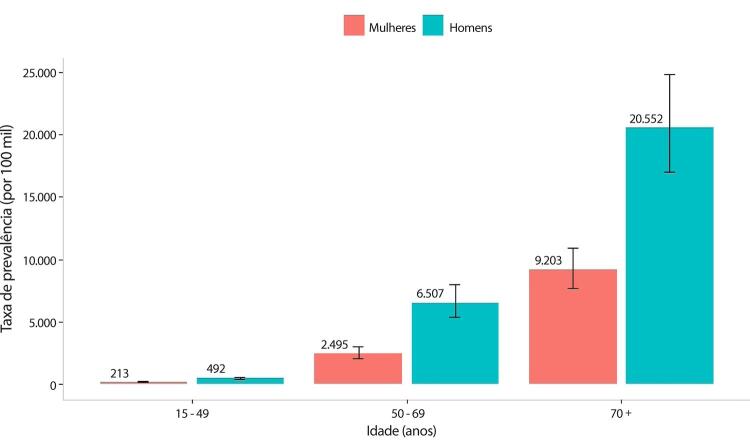
-Taxa de prevalência (por 100 mil) de doença arterial coronariana de acordo com sexo e idade no Brasil, 2019. As barras de erro representam intervalos de incerteza 95%.

Fonte: Dados derivados do estudo Global Burden of Disease 2019, Institute for Health Metrics and Evaluation, University of Washington. ^46^

**Figura 3-3:**
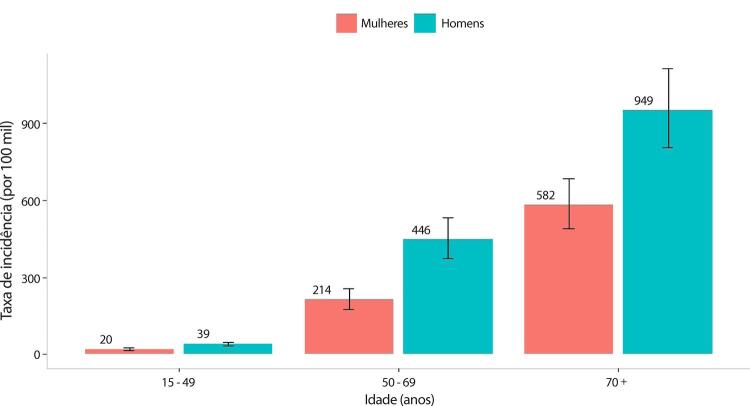
-Taxa de incidência (por 100 mil) de doença arterial coronariana de acordo com sexo e idade no Brasil, 2019. As barras de erro representam intervalos de incerteza 95%.

Fonte: Dados derivados do estudo Global Burden of Disease 2019, Institute for Health Metrics and Evaluation, University of Washington. ^46^

**Figura 3-4:**
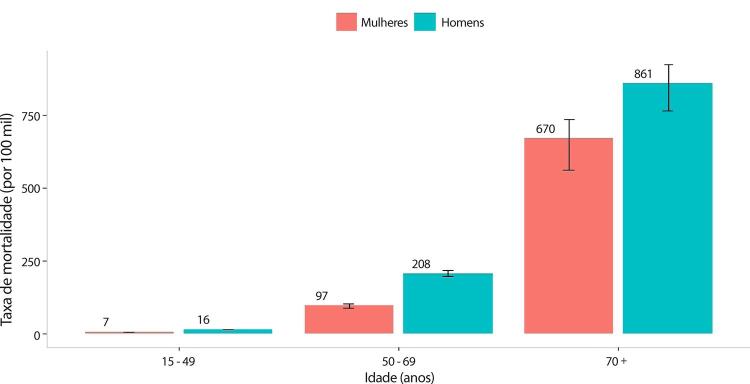
-Taxa de mortalidade (por 100 mil) por doença arterial coronariana de acordo com sexo e idade no Brasil, 2019. As barras de erro representam intervalos de incerteza 95%.

Fonte: Dados derivados do estudo Global Burden of Disease 2019, Institute for Health Metrics and Evaluation, University of Washington. ^46^

**Figura 3-5:**
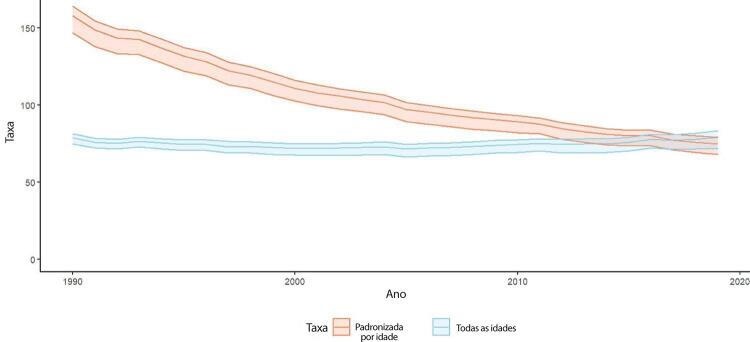
-Taxas de mortalidade bruta e padronizada por idade (por 100 mil) por doença arterial coronariana no Brasil, 1990-2019. As áreas sombreadas mostram os intervalos de incerteza 95%.

Fonte: Dados derivados do estudo Global Burden of Disease 2019, Institute for Health Metrics and Evaluation, University of Washington. ^46^

**Figura 3-6:**
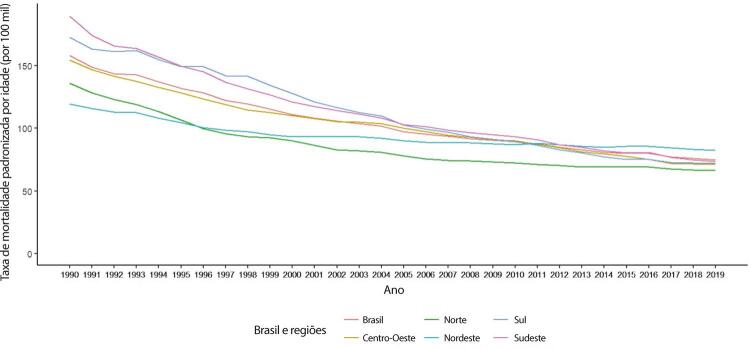
-Taxas de mortalidade padronizadas por idade (por 100 mil) por doença arterial coronariana nas regiões brasileiras, 1990-2019.

Fonte: Dados derivados do estudo Global Burden of Disease 2019, Institute for Health Metrics and Evaluation, University of Washington. ^46^

**Figura 3-7:**
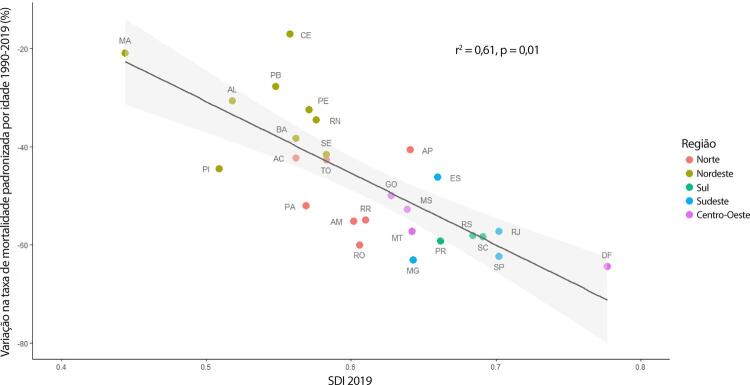
-Correlação entre o Índice Sociodemográfico (SDI) de 2019 e a variação percentual nas taxas de mortalidade padronizadas por idade por doença arterial coronariana de 1990 a 2019.

Fonte: Dados derivados do estudo Global Burden of Disease 2019, Institute for Health Metrics and Evaluation, University of Washington. ^46^ ^,^ ^95^

**Figura 3-8:**
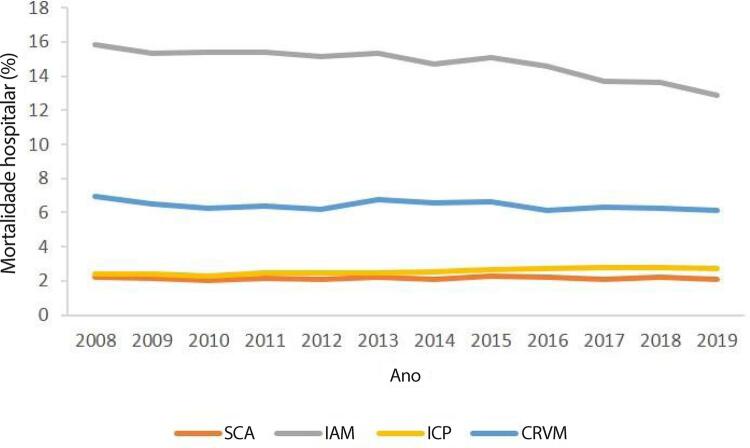
-Taxa de mortalidade hospitalar por síndrome coronariana aguda (SCA), infarto agudo do miocárdio (IAM), intervenção coronária percutânea (ICP) e cirurgia de revascularização do miocárdio (CRVM) no sistema público de saúde do Brasil, 2008-2019.

Fonte: Ministério da Saúde do Brasil – Sistema de Informações Hospitalares do Sistema Único de Saúde (SIH/SUS). ^95^

**Figura 3-9:**
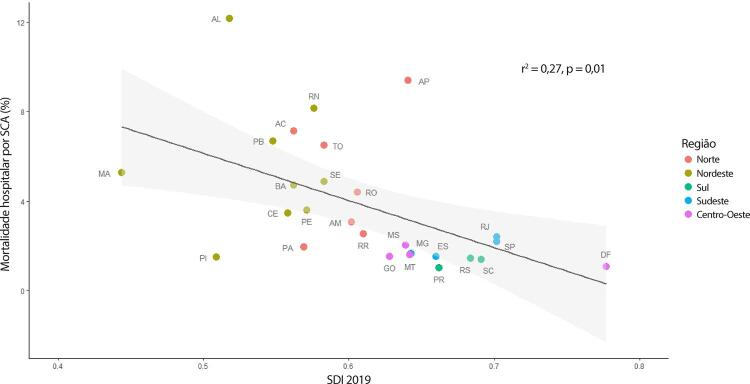
-Correlação entre o Índice Sociodemográfico (SDI) de 2019 e as taxas de mortalidade hospitalar por síndrome coronariana aguda (SCA) no sistema público de saúde do Brasil em 2019.

Fonte: Ministério da Saúde do Brasil – Sistema de Informações Hospitalares do Sistema Único de Saúde (SIH/SUS) ^95^ e Estudo Global Burden of Disease 2019, Institute for Health Metrics and Evaluation, University of Washington. ^46^

**Figura 3-10:**
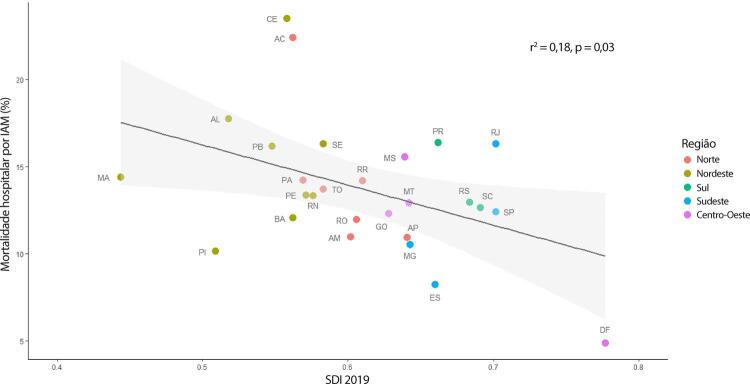
-Correlação entre o Índice Sociodemográfico (SDI) de 2019 e as taxas de mortalidade hospitalar por infarto agudo do miocárdio (IAM) no sistema público de saúde do Brasil em 2019.

Fonte: Ministério da Saúde do Brasil – Sistema de Informações Hospitalares do Sistema Único de Saúde (SIH/SUS) ^95^ e Estudo Global Burden of Disease 2019, Institute for Health Metrics and Evaluation, University of Washington. ^46^

**Figura 3-11:**
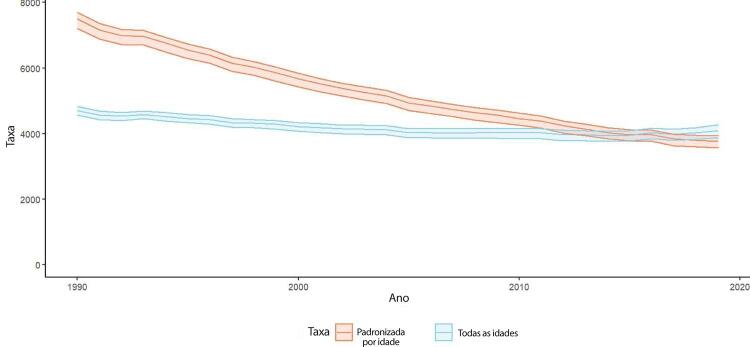
-Taxas de DALYs bruta e padronizada por idade (por 100 mil) por doença arterial coronariana no Brasil, 1990-2019. As áreas sombreadas mostram os intervalos de incerteza 95%.

Fonte: Dados derivados do estudo Global Burden of Disease 2019, Institute for Health Metrics and Evaluation, University of Washington. ^46^

**Figura 3-12:**
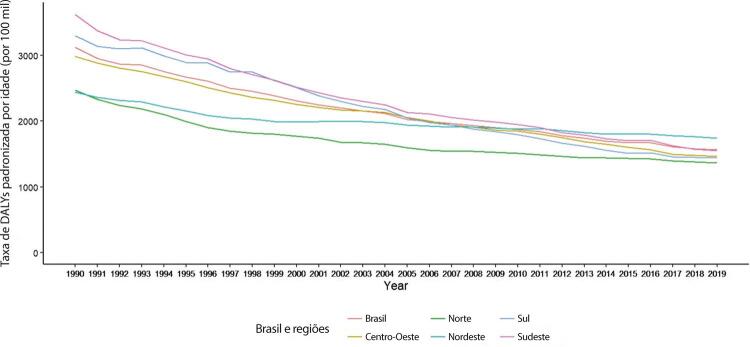
-Taxas de DALYs padronizadas por idade (por 100 mil) por doença arterial coronariana nas regiões brasileiras, 1990-2019.

Fonte: Dados derivados do estudo Global Burden of Disease 2019, Institute for Health Metrics and Evaluation, University of Washington. ^46^

**Figura 3-13:**
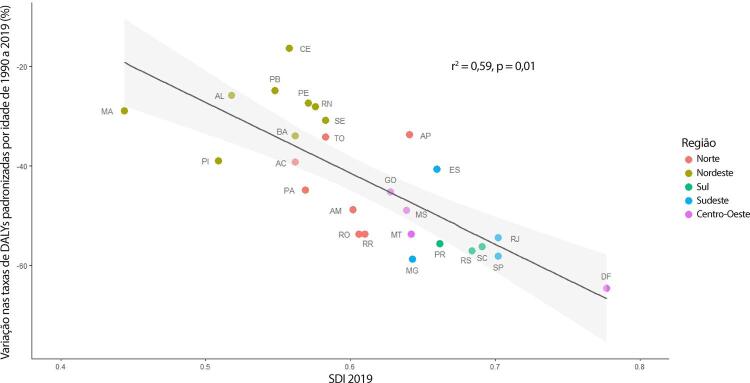
-Correlação entre o Índice Sociodemográfico (SDI) de 2019 e a variação percentual de taxas de DALYs padronizadas por idade por doença arterial coronariana de 1990 a 2019.

Fonte: Dados derivados do estudo Global Burden of Disease 2019, Institute for Health Metrics and Evaluation, University of Washington. ^46^ ^,^ ^95^

**Figura 3-14:**
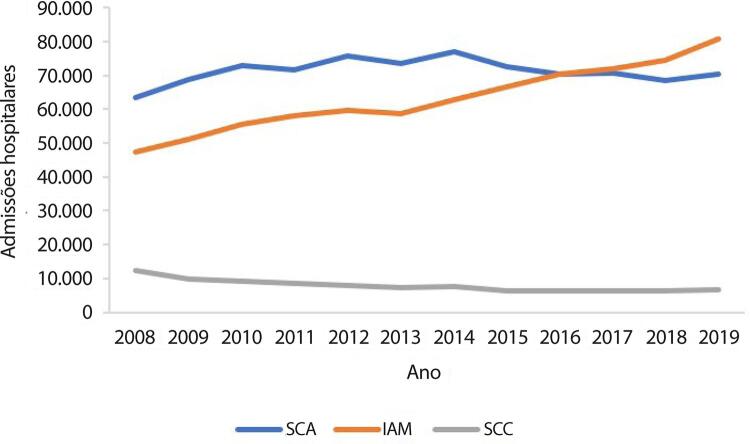
-Número de admissões hospitalares por síndrome coronariana aguda (SCA), infarto agudo do miocárdio (IAM) e síndrome coronariana crônica (SCC) no sistema público de saúde do Brasil, 2008-2019.

Fonte: Ministério da Saúde do Brasil – Sistema de Informações Hospitalares do Sistema Único de Saúde (SIH/SUS). ^95^

**Figura 3-15:**
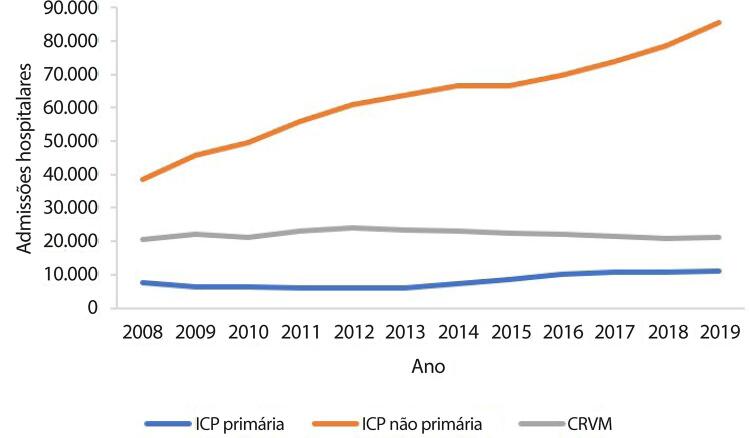
-Número de admissões hospitalares por intervenção coronária percutânea (ICP) primária, ICP não primária e cirurgia de revascularização do miocárdio (CRVM) no sistema público de saúde do Brasil, 2008-2019.

Fonte: Ministério da Saúde do Brasil – Sistema de Informações Hospitalares do Sistema Único de Saúde (SIH/SUS). ^95^

**Figura 3-16:**
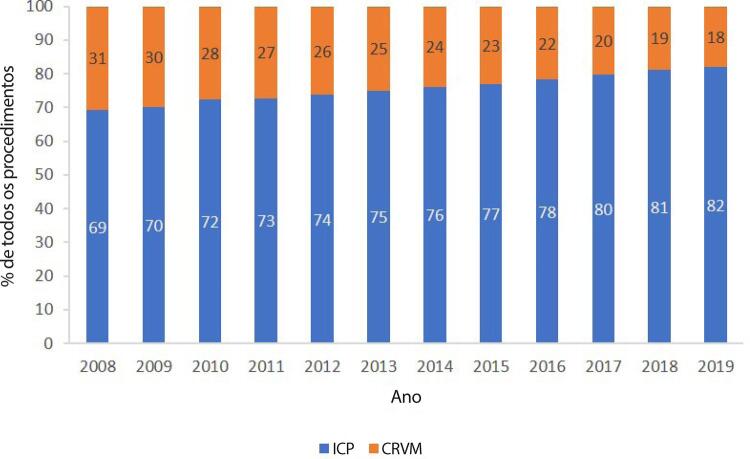
-Porcentagens de intervenção coronária percutânea (ICP) e de cirurgia de revascularização do miocárdio (CRVM) em relação ao total de procedimentos de revascularização miocárdica no sistema público de saúde do Brasil, 2008-2019.

Fonte: Ministério da Saúde do Brasil – Sistema de Informações Hospitalares do Sistema Único de Saúde (SIH/SUS). ^95^

**Figura 3-18:**
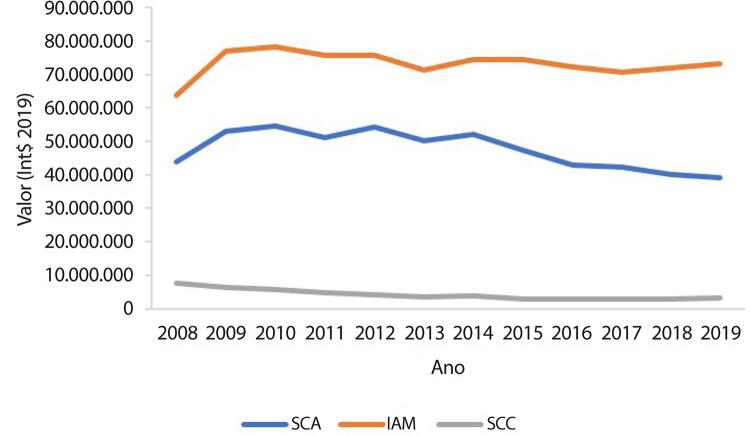
-Valores médios em dólares internacionais (Int$ 2019) reembolsados por admissões hospitalares devidas a síndrome coronariana aguda (SCA), infarto agudo do miocárdio (IAM) e síndrome coronariana crônica (SCC) no sistema público de saúde do Brasil, 2008-2019.

Fonte: Ministério da Saúde do Brasil – Sistema de Informações Hospitalares do Sistema Único de Saúde (SIH/SUS). ^95^

**Figura 3-17:**
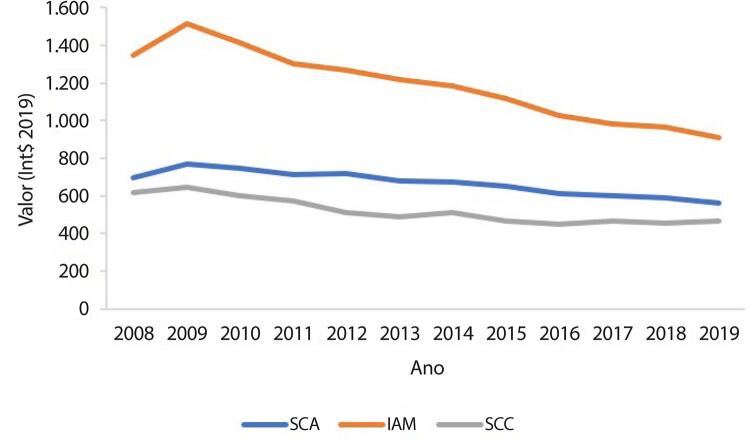
-Valores totais em dólares internacionais (Int$ 2019) reembolsados por admissões hospitalares devidas a síndrome coronariana aguda (SCA), infarto agudo do miocárdio (IAM) e síndrome coronariana crônica (SCC) no sistema público de saúde do Brasil, 2008-2019.

Fonte: Ministério da Saúde do Brasil – Sistema de Informações Hospitalares do Sistema Único de Saúde (SIH/SUS). ^95^

**Figura 3-19:**
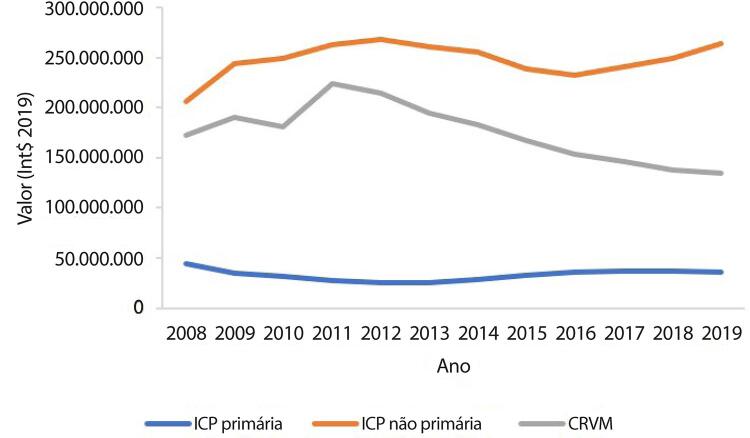
-Valores totais em dólares internacionais (Int$ 2019) reembolsados por admissões hospitalares devidas a intervenção coronária percutânea (ICP) primária, ICP não primária e cirurgia de revascularização do miocárdio (CRVM) no sistema público de saúde do Brasil, 2008-2019.

Fonte: Ministério da Saúde do Brasil – Sistema de Informações Hospitalares do Sistema Único de Saúde (SIH/SUS). ^95^

**Figura 3-20:**
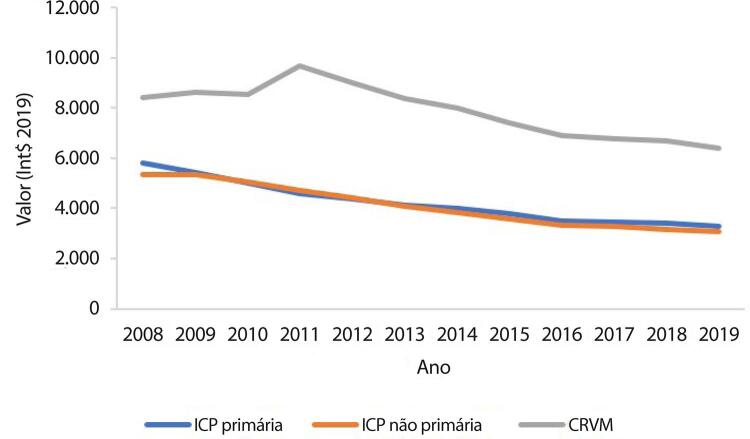
-Valores médios em dólares internacionais (Int$ 2019) reembolsados por admissões hospitalares devidas a intervenção coronária percutânea (ICP) primária, ICP não primária e cirurgia de revascularização do miocárdio (CRVM) no sistema público de saúde do Brasil, 2008-2019.

Fonte: Ministério da Saúde do Brasil – Sistema de Informações Hospitalares do Sistema Único de Saúde (SIH/SUS). ^95^

### Panorama e Prevalência

•A DAC, também conhecida como DIC, compreende um espectro de condições clínicas sintomáticas e assintomáticas tipicamente relacionadas à redução do fluxo sanguíneo para o músculo cardíaco. A causa mais comum é a doença aterosclerótica das coronárias, uma condição crônica de apresentação variável, que progride desde uma longa fase assintomática até angina estável, IAM e angina instável. A DAC é uma causa comum de insuficiência cardíaca, com fração de ejeção ventricular esquerda reduzida ou preservada, arritmias ventriculares e morte súbita cardíaca.

•A DAC foi a principal causa de morte no Brasil na última década, para homens e mulheres. Devido ao seu amplo espectro de apresentação clínica, a prevalência, a incidência e a mortalidade da DAC relatadas variam muito, dependendo da população e do contexto da atenção à saúde estudados.

#### Doença Arterial Coronariana

•De acordo com dados do estudo GBD, no Brasil, o número de portadores de DAC, definidos como indivíduos com IAM prévio, angina estável ou insuficiência cardíaca isquêmica, aumentou de 1,48 milhão em 1990 para mais de 4 milhões em 2019 ( Tabela 3-1 ), e a prevalência bruta de DAC passou de 0,99% para 1,85% no período ( Figura 3-1 ). Essas taxas subiram vertiginosamente com o envelhecimento, sendo de 0,4%, 4,4% e 14,0% entre indivíduos com 15-49 anos, 50-69 anos e a partir dos 70 anos, respectivamente, em 2019. Em todos os grupos etários, a DAC foi mais frequente nos homens do que nas mulheres ( Figura 3-2 ). ^46^

•A elevação contínua no número de casos de DAC pode ser explicada pelo crescimento e envelhecimento da população, pois a taxa de prevalência padronizada por idade permaneceu estável de 1990 a 2019 (variação percentual: -1%, Tabela 3-1 e Figura 3-1 ). Em 2019, a taxa de prevalência de DAC padronizada por idade foi de 1.709 (II 95%, 1.466-1.994) por 100 mil habitantes na população total ( Tabela 3-1 ), 1.046 (II 95%, 905-1.209) por 100 mil mulheres e 2.534 (II 95%, 2.170-2.975) por 100 mil homens. ^46^

•Houve uma diferença, entre as regiões brasileiras, na prevalência de DAC padronizada por idade, que foi maior nas regiões Sudeste e Sul (Minas Gerais: 1.878 por 100 mil habitantes; Paraná e Santa Catarina: ~1.750 por 100 mil) e menor na região Norte (Amapá: 1.496 por 100 mil, Tabela 3-1 ). ^46^

•Na avaliação basal da coorte ELSA-Brasil no período 2008-2010, a prevalência de DAC autorrelatada foi 2,7%. Essa coorte foi composta por mais de 15 mil servidores públicos com idade entre 35 e 74 anos de seis cidades (Salvador, Vitória, Belo Horizonte, Rio de Janeiro, São Paulo e Porto Alegre). A prevalência foi mais alta entre os homens e indivíduos com nível socioeconômico mais baixo. ^80^

#### Infarto do Miocárdio

•A prevalência de IAM foi de 4,0% na pesquisa transversal *May Measurement Month 2017* envolvendo 7.260 indivíduos de diferentes etnias e regiões do Brasil. Os participantes (56% mulheres; idade média, 51,6±16,1 anos) foram recrutados principalmente em hospitais e clínicas indexadas. ^81^

#### Tipos de Síndrome Coronariana Aguda

•O grande registro prospectivo ACCEPT, idealizado pela Sociedade Brasileira de Cardiologia, arrolou 4.782 pacientes com SCA em 53 instituições (hospitais públicos, organizações de saúde e serviços privados) das cinco regiões brasileiras. Em sua maioria, os serviços eram terciários e disponibilizavam ICP e cirurgia cardíaca. Os participantes foram incluídos entre 2010 e 2014. As porcentagens de pacientes com angina instável, IAMSSST e IAMCSST foram 30%, 34% e 36%, respectivamente. ^82^

•O estudo BRACE, um registro epidemiológico observacional transversal de pacientes com SCA, utilizou a metodologia de amostra por conglomerados para selecionar serviços representativos de todos os hospitais com unidades de terapia intensiva ou cardiologia no Brasil. Dos 1.150 pacientes de 72 hospitais incluídos no estudo de 2007 a 2009, 54% apresentavam IAMCSST. Dos outros pacientes com SCA sem elevação do segmento ST, 67% apresentavam IAMSSST e 33%, angina instável. ^83^ ^,^ ^84^

•No estudo ERICO, uma coorte prospectiva de pacientes com SCA admitidos em hospital universitário na cidade de São Paulo, dos 964 participantes recrutados de 2009 a 2012, o diagnóstico inicial foi angina instável em 33%, IAMSSST em 39% e IAMCSST em 28% deles. ^85^

•No BRIDGE-ACS, um estudo randomizado por *cluster* envolvendo 1.150 pacientes com SCA recrutados em hospitais públicos gerais nas principais áreas urbanas brasileiras em 2011, as taxas de diagnóstico de angina instável, IAMSSST e IAMCSST foram 24%, 36% e 40%, respectivamente. ^86^

#### Angina Estável

•Inquéritos populacionais regionais conduzidos em 2007, aplicando o questionário de angina de Rose, mostraram prevalência de angina de 12,3% em brasileiros com idade ≥30 anos em Ribeirão Preto e de 8,2% naqueles ≥40 anos na cidade de Pelotas. ^87^ ^,^ ^88^

•De acordo com a PNS 2013, um inquérito epidemiológico de base domiciliar e com representatividade nacional, as taxas de prevalência geral de angina classe I e de angina classe II foram 7,6% (IC 95%, 7,2%-8,0%) e 4,2% (IC 95%, 3,9%-4,5%), respectivamente. ^89^

•A angina *pectoris* autorreferida foi mais prevalente em mulheres do que em homens em todos os estudos descritos.

•É importante ressaltar a ocorrência de maiores taxas de prevalência nos inquéritos prospectivos em comparação às estatísticas nacionais. Avaliações autorreferidas de angina são muito sensíveis, mas não específicas para DAC, pois não requerem exames confirmatórios nem relatórios de saúde. Além disso, considerando-se a natureza assintomática da DAC, a sua verdadeira epidemiologia pode estar sub-representada nas estatísticas nacionais.

## Mortalidade

•De acordo com as estimativas do GBD, houve 171.246 mortes atribuídas a DAC no Brasil em 2019 ( Tabela 3-2 ), correspondendo a 12% (11%-13%) do total de mortes no país e a 43% de todas as mortes por DCV. ^46^

•A taxa de mortalidade bruta atribuída a DAC foi 79 (II 95%, 72-83) por 100 mil habitantes em 2019. As taxas aumentaram com o envelhecimento: 11 (II 95%, 11-12), 149 (II 95%, 142-156) e 751 (II 95%, 651-807) por 100 mil indivíduos de 15-49 anos, 50-69 anos e a partir dos 70 anos, respectivamente. Em todos os grupos etários, a taxa de mortalidade foi mais alta nos homens do que nas mulheres ( Figura 3-4 ). ^46^

•Em 2019, a taxa de mortalidade por DAC e padronizada por idade foi 75 (II 95%, 68-79) por 100 mil habitantes na população total ( Tabela 3-2 ), 58 (II 95%, 51-63) por 100 mil mulheres e 96 (II 95%, 88-101) por 100 mil homens. ^46^

•Em 2019, a DAC foi a primeira causa de morte no Brasil em todas as suas unidades federativas, exceto no Amazonas e Amapá, onde a DAC foi a segunda mais frequente causa de morte. Além disso, a DAC foi a principal causa de morte tanto em mulheres quanto homens acima de 50 anos e a terceira causa de fatalidade entre homens de 15-49 anos (atrás de violência interpessoal e acidentes nas estradas). ^46^

•De acordo com o estudo GBD, a taxa de mortalidade por DAC não ajustada aumentou discretamente de ~2005 a 2019, enquanto a taxa padronizada por idade diminuiu continuamente de 1990 a 2019 (variação percentual acumulada: -53%, Tabela 3-2 e Figura 3-5 ). ^46^

•A redução na taxa de mortalidade de 1990 a 2019 foi menos pronunciada nos indivíduos de 15-49 anos (-34,9%, II 95%, -38,5% a -31,1%) em comparação àqueles de 50-69 anos (-47,8%, II 95%, -50,6% a -45,0%) ou a partir dos 70 anos (-46,5%, II 95%, -50,1% a -43,7%). ^46^

•Diferenças regionais nas taxas de mortalidade ajustadas por idade e tendências ao longo do tempo foram notadas. Em 1990, as taxas foram mais altas nas regiões Sul e Sudeste e mais baixas nos estados do Norte e Nordeste. De 1990 a 2019, observou-se redução na taxa de mortalidade ajustada por idade em todos os estados, embora menos significativa no Nordeste, tendo a heterogeneidade entre as regiões sido progressivamente atenuada ( Figura 3-6 ). As variações mais discretas ocorreram no Ceará (-17%) e Maranhão (-21%), enquanto as reduções mais pronunciadas foram detectadas no Distrito Federal (-65%), Minas Gerais (-63%) e São Paulo (-62%, Tabela 3-2 ). Observou-se correlação negativa entre a variação na taxa de mortalidade por DAC padronizada por idade por DAC no período e o SDI 2019 ( *r* ^2^ 0,61, p-valor < 0,01, Figura 3-7 ). ^46^ ^,^ ^90^ Em 2019, as mais baixas taxas de mortalidade foram observadas no Amazonas (56 por 100 mil) e Minas Gerais (59 por 100 mil), enquanto as mais altas foram registradas no Maranhão (104 por 100 mil) e em Pernambuco (102 por 100 mil, Tabela 3-2 ). ^46^

•Em análise temporal de dados do Ministério da Saúde do Brasil, o coeficiente de morte relacionada a DIC permaneceu estável para mulheres nas regiões Norte e Centro-Oeste entre 1981 e 2001, enquanto diminuiu no Sul e Sudeste e aumentou no Nordeste. Para os homens, houve tendência decrescente nos eventos nas regiões Sul e Sudeste. ^91^

•Análise conduzida a partir de dados do DATASUS, de 1990 a 2009, mostrou uma redução na taxa de mortalidade por DIC ajustada por idade no Brasil, que passou de 195 a 149 por 100 mil homens (variação: -23,4%) e de 120 a 84 por 100 mil mulheres (variação: -29,5%). ^92^

•A proporção de mortes causadas por DCV permaneceu estável nas últimas décadas, com relatos variando de 26% a 32%, de acordo com o ano. Um estudo ecológico realizado em Porto Alegre, incluindo indivíduos com 45-64 anos, mostrou que a DCV foi responsável por 28,5% de todos os óbitos em 2009. Desses, 40% estavam relacionados a DAC, cuja proporção foi maior entre indivíduos com nível socioeconômico mais baixo (42,7%) do que entre aqueles com nível socioeconômico mais alto (26,3%). ^93^

•Em um estudo ecológico nacional incluindo indivíduos com 35-64 anos, de 1999 a 2001, a taxa de morte relacionada a DAC foi 84 ± 30 por 100 mil habitantes. A incidência de eventos relacionou-se diretamente à taxa de pobreza e ao menor nível educacional. É importante notar que houve grande variação nos resultados entre as 98 cidades participantes, provavelmente em razão da qualidade dos dados. ^94^

### Mortalidade Relacionada à Síndrome Coronariana Aguda

•De acordo com o SIH/SUS, a taxa de mortalidade hospitalar entre pacientes tratados para SCA no sistema público permaneceu estável de 2008 a 2019 [2,2% (1.404 mortes em 63.913 admissões) e 2,1% (1.449 em 70.013 admissões), respectivamente] ( Figura 3-8 ). Houve modesta correlação negativa entre a taxa de mortalidade hospitalar durante tratamento para SCA em 2019 e o SDI ( *r* ^2^ 0,27, p-valor = 0,01), com detecção de taxas mais altas nos estados do Nordeste e do Norte ( Figura 3-9 ). ^90^ ^,^ ^95^

•Entre indivíduos admitidos com IAM, a taxa de mortalidade hospitalar diminuiu de 15,9% (7.627 mortes em 48.114 admissões) em 2008 para 12,9% (10.445 mortes em 80.944 admissões) em 2019, uma variação percentual de -19% ( Figura 3-8 ). Observou-se discreta correlação negativa entre a taxa de mortalidade hospitalar durante tratamento de IAM em 2019 e o SDI ( *r* ^2^ 0,18, p-valor = 0,03, Figura 3-10 ). ^90^ ^,^ ^95^

•Vários registros brasileiros de SCA relataram os desfechos de indivíduos admitidos com SCA. Em geral, a taxa de mortalidade nos registros é mais baixa do que a relatada no SIH/SUS. Vários estudos salientaram as diferenças regionais no tratamento e na mortalidade, assim como os piores desfechos em pacientes tratados no sistema público em comparação aos tratados nos hospitais privados. ^82 – 84 , 96^

•No registro ACCEPT, entre 2.485 pacientes com SCA recrutados em 47 hospitais brasileiros em 2010/2011, a mortalidade por todas as causas em 30 dias foi 1,8% para angina instável, 3,0% para IAMSSST e 3,4% para IAMCSST. ^97^

•Em relato subsequente do registro ACCEPT analisando um total de 4.782 pacientes recrutados até 2014, a taxa de eventos cardiovasculares maiores foi 13,6% após 1 ano de seguimento. Os eventos foram mais frequentes entre pacientes do SUS (16,6 por 100 pacientes/ano) do que entre aqueles do sistema privado ou com plano de saúde (9,1 por 100 pacientes/ano). Além disso, observou-se discrepância regional na taxa de mortalidade em 1 ano, que foi maior na região Norte (19,8%; IC 95%, 12,6%-27,0%) e menor na região Nordeste (5,6%; IC 95%, 3,7%-7,5%). ^82^

•No registro transversal BRACE, a mortalidade hospitalar geral foi 5,2% entre 1.150 pacientes com SCA recrutados de 2007 a 2009 em 72 hospitais representativos de todos os serviços com Cardiologia e unidade de terapia intensiva. ^83^ ^,^ ^84^

•Entre 2003 e 2008, o Registro RBSCA arrolou 2.693 pacientes, 45% dos quais com IAM. A taxa de mortalidade hospitalar para aqueles com angina instável foi 3,1%, enquanto para aqueles com IAM foi 7,7%, levando a uma mortalidade geral de 5,5%. ^98^

•Em estudo retrospectivo multicêntrico de 3.745 pacientes admitidos com SCA entre 2010 e 2015, a mortalidade hospitalar por todas as causas foi 3,3%, sendo que 454 (12,2%) pacientes experienciado pelo menos um evento adverso maior (reinfarto, choque, sangramento, acidente vascular cerebral ou morte). ^99^

•No estudo ERICO, a taxa de mortalidade foi 4,4% aos 30 dias e 12% em 1 ano entre 964 pacientes admitidos com SCA de 2009 a 2012. ^85^

•Em estudo observacional longitudinal realizado de 2011 a 2014 em hospital de alta complexidade em Belo Horizonte, a mortalidade hospitalar foi 9,1% entre 788 pacientes com IAMCSST e 7,6% entre 341 pacientes com IAMSSST. ^100^

•Em estudo do Projeto Minas Telecardio 2, conduzido em 2013 e 2014 em seis unidades de emergência da cidade de Montes Claros, Minas Gerais, entre 593 pacientes com SCA, a mortalidade hospitalar foi 9,4%, variando de 4,9% para angina instável a 17% para os casos de IAMCSST. ^101^

•No registro RESISST, 520 pacientes com IAMCSST foram admitidos em unidades públicas de saúde interconectadas através de uma rede regional de atendimento ao IAM, de janeiro de 2011 a junho de 2013. Apenas 41% dos pacientes receberam terapia de reperfusão e a taxa de mortalidade em 30 dias foi 15,3%. ^102^

•No registro VICTIM, dados de todo o estado de Sergipe coletados de 2014 a 2017 identificaram 707 casos de IAMCSST com mortalidade hospitalar de 10,9%. A taxa de mortalidade foi significativamente maior para aqueles admitidos em hospitais públicos em comparação aos admitidos nos privados (11,9% *versus* 5,9%, respectivamente). ^96^

•Entre 1.263 pacientes com IAMSSST admitidos em um hospital privado da cidade de São Paulo de 2014 a 2018, a taxa de mortalidade hospitalar foi 1,3%. ^103^

•Entre 1.852 pacientes com IAMCSST admitidos em unidades de pronto-atendimento municipal da cidade de São Paulo de 2010 a 2016 e submetidos a tratamento fármaco-invasivo, a mortalidade hospitalar foi 4,0% entre aqueles com idade inferior a 75 anos e 18,2% entre aqueles com idade ≥75 anos. ^104^

•As taxas de mortalidade foram relatadas em um registro de 542 pacientes consecutivos admitidos com IAMCSST e submetidos a ICP primária entre março de 2011 e fevereiro de 2017 em hospital universitário terciário de Porto Alegre. Observou-se morte hospitalar em 10,7% durante o período estudado, que permaneceu estável de 2011 a 2016.

A mortalidade de 1 ano foi 16,6%, com tendência decrescente de 2011 a 2016. ^105^

•Um estudo de série de 21 anos explorou as tendências da mortalidade relacionada a IAM de acordo com sexo, regiões do Brasil e tipo de cidade (capital *versus* não capital). Os dados de mortalidade foram obtidos do Sistema de Informações sobre Mortalidade do Ministério da Saúde brasileiro e os autores utilizaram procedimentos para corrigir as taxas de mortalidade por causas mal definidas, uso de códigos ‘ *garbage* ’ e subnotificação. De 1996 a 2016, a taxa de mortalidade por IAM padronizada por idade diminuiu 44% no país, com substanciais diferenças regionais (variações percentuais: +5% no Norte, +11% no Nordeste, -35% no Centro-Oeste, -68% no Sudeste e -85% no Sul). Além disso, variações temporais foram mais pronunciadas nas mulheres do que nos homens, e nas capitais do que nas cidades não capitais. A taxa de mortalidade por IAM padronizada por idade corrigida diminuiu 49% e 23% entre as mulheres que viviam nas capitais e em outras municipalidades, respectivamente, de 1996 a 2016. Entre os homens, os respectivos declínios foram 43% e 17%. Importante notar a ocorrência de melhorias na qualidade dos dados (por exemplo, redução da subnotificação) ao longo dos anos; esse fenômeno é mais recente no Norte e Nordeste e nas cidades que não são capitais. ^106^

### Mortalidade Relacionada a Intervenção Coronária Percutânea

•De acordo com o SIH/SUS, a taxa de mortalidade hospitalar entre pacientes submetidos a ICP em hospitais públicos permaneceu estável de 2008 a 2019 [2,4% (1.112 mortes em 46.683 admissões) e 2,7% (2.625 em 96.930), respectivamente], com variação percentual de 14% ( Figura 3-8 ). Em 2019, essas taxas variaram de 2,4% no Sudeste a 3,5% no Nordeste. Entre os pacientes submetidos a ICP primária, a mortalidade hospitalar foi 6,8% (765 mortes em 11.270 procedimentos), variando de 5,9% nas regiões Norte, Sul e Centro-Oeste a 7,3% na região Sudeste. ^95^

•Em um estudo de coorte realizado de 2009 a 2013 (Registro ICP-BR multicêntrico), avaliando 4.806 pacientes submetidos a ICP (69% com IAM recente) em 8 centros médicos terciários de referência, a taxa de mortalidade hospitalar foi 2,6%. ^107^

•Outro registro de ICP, que incluiu 1.249 pacientes consecutivos em 2009, encontrou taxa de mortalidade total de 2,3%, variando de 0,2% para angina estável a 6,1% para IAMCSST. ^108^

•Em outro estudo avaliando ICP em hospitais públicos, 166.514 procedimentos foram realizados em 180 hospitais de 2005 a 2008. A mortalidade hospitalar média foi 2,3%, variando de 0% a 11,4%. Essa taxa foi mais baixa na região Sudeste (2,0%) e mais alta na região Norte (3,6%). A taxa de mortalidade hospitalar foi 2,3% nos hospitais de alto volume, responsáveis por 101.218 (60,8%) ICP, tendo sido 2,3% nos hospitais de médio volume e 2,5% nos de baixo volume.

A taxa de mortalidade foi maior entre mulheres e nos pacientes acima de 65 anos. ^109^

•A maioria dos relatos é proveniente de instituições públicas, sendo os dados dos hospitais privados limitados. Uma análise de 440 procedimentos realizados entre 2013 e 2014 em um hospital público e outro privado na cidade do Rio de Janeiro mostrou baixa mortalidade (0,5%), com taxas semelhantes nas duas instituições. ^110^

•Diferenças na mortalidade hospitalar após ICP de acordo com o acesso femoral ou radial foram retrospectivamente analisadas em 158.363 pacientes arrolados no Registro CENIC entre 2006 e 2016 (52% com DAC estável). A utilização do acesso radial aumentou progressivamente de 12% em 2006 para 50% em 2016, tendo sido associada com mortalidade hospitalar mais baixa em comparação à utilização do acesso femoral em uma análise com correspondência por escore de propensão (0,4% *versus* 0,7%; OR, 0,59; IC 95%, 0,47-0,74; p < 0,001; n = 54.242 pacientes). ^111^

•Em 847 pacientes com idade superior a 90 anos, submetidos a ICP (68% com SCA) e incluídos no Registro CENIC entre 2006 e 2016, a taxa de mortalidade hospitalar foi 4,8%. ^112^

•Há escassez de dados sobre as taxas de sobrevida de longo prazo de pacientes submetidos a ICP. Em uma análise de procedimentos realizados no estado do Rio de Janeiro entre 1999 e 2000 em todos os hospitais públicos incluindo 19.263 indivíduos, a sobrevida de 1 ano foi 93% e a de 15 anos foi 57%. Nesse estudo, mulheres, em comparação a homens, apresentaram maior taxa de sobrevida em 15 anos após ICP. ^113^

### Mortalidade Relacionada à Cirurgia de Revascularização do Miocárdio

•De acordo com o SIH/SUS, a taxa de mortalidade hospitalar entre pacientes submetidos a CRVM em instituições públicas foi 7,0% (1.566 mortes em 22.537 procedimentos) em 2008 e 6,1% (1.432 em 23.488 admissões) em 2019, uma variação percentual de -12% ( Figura 3-8 ). Em 2019, a taxa mais baixa foi observada na região Nordeste (4,4%), enquanto a mais alta foi registrada na região Centro-Oeste (10,0%). ^95^

•O registro BYPASS é um banco de dados em andamento estabelecido em 2015 pela Sociedade Brasileira de Cirurgia Cardiovascular, envolvendo 17 instituições representativas de todas as regiões brasileiras. Entre 2.292 pacientes arrolados até novembro de 2018 e submetidos a CRVM isolada ou combinada, a taxa de mortalidade hospitalar foi 2,8%, tendo 5,3% deles permanecido em ventilação mecânica por mais de 24 horas e 1,2% deles apresentado acidente vascular cerebral durante a hospitalização. ^114^ ^,^ ^115^

•O MASS II foi um ensaio clínico randomizado unicêntrico, desenhado para comparar os efeitos de longo prazo de terapia medicamentosa, angioplastia ou estratégias cirúrgicas para tratar DAC multivascular com angina estável e função ventricular preservada, conduzido antes de 2007. As taxas de mortalidade hospitalar para ICP e CRVM foram 2,4% e 2,5%, respectivamente. ^116^ As taxas de sobrevida de 10 anos não diferiram significativamente entre os grupos: 75% para CRVM, 75% para ICP e 69% para terapia medicamentosa (p=0,089). ^117^ Em outro ensaio do mesmo grupo, MASS III, taxas de sobrevida de 10 anos similares foram descritas. ^118^

•No registro REPLICCAR-I, 2.961 pacientes foram submetidos a CRVM isolada em dez hospitais entre 2013 e 2016. A taxa de mortalidade por todas as causas foi 3,4% em 30 dias e 5,3% em seguimento de 4 anos. ^119^

•Várias outras experiências unicêntricas, com análise tanto retrospectiva quanto prospectiva, descreveram mortalidade de curto prazo para pacientes submetidos a CRVM variando de 1,9% a 11,7%. ^120–123^

## Carga de Doença

•O GBD 2019 estimou taxa de 1.563 (II 95%, 1.472-1.636) DALYs por DAC por 100 mil indivíduos ( Tabela 3-3 ). Essa taxa de DALYs correspondeu a 5,7% (II 95%, 5,1%-6,3%) de todos os DALYs, sendo a DAC a segunda causa mais comum de DALYs no Brasil entre mulheres (após distúrbios neonatais) e homens (após violência interpessoal) em 2019. ^46^

•De 1990 a 2019, o número total de DALYs atribuídos a DAC aumentou continuamente, a taxa bruta de DALYs por 100 mil permaneceu estável e a taxa de DALYs padronizada por idade por 100 mil diminuiu gradualmente 50% ( Tabela 3-3 , Figura 3-11 ). ^46^

•Ao se considerar as regiões brasileiras, a tendência das taxas de DALYs padronizadas por idade de 1990 a 2019 se assemelha à das taxas de mortalidade. Em 1990, as mais altas taxas foram observadas no Sul e Sudeste, enquanto as mais baixas, no Norte e Nordeste. Reduções na taxa de DALYs ajustada para idade de 1990 a 2019 foram observadas em todos os estados, com variações de menor magnitude nas regiões Norte e Nordeste, de modo que a heterogeneidade entre as regiões diminuiu ( Figura 3-12 ). Os estados com as menores quedas foram Ceará (-16%), Paraíba (-25%) e Alagoas (-26%, Tabela 3-3 ). Houve correlação negativa entre a variação na taxa de DALYs padronizada por idade por DAC no período e o SDI ( *r* ^2^ 0,59, p-valor < 0,01, Figura 3-13 ). ^46^ ^,^ ^90^ Em 2019, a mais baixa taxa de DALYs foi relatada no Amazonas (1.106 por 100 mil), enquanto as mais altas foram observadas no Maranhão (2.157 por 100 mil) e em Pernambuco (2.163 por 100 mil, Tabela 3-3 ). ^46^

•A maioria dos DALYs associados com DAC foi devida a YLLs. A taxa de YLLs padronizada por idade foi 1.501 (II 95%, 1.408-1.574) por 100 mil, equivalendo a 9,1% (II 95%, 8,6%-9,6%) de todos os YLLs em 2019. Essa taxa caiu pela metade de 1990 a 2019, com variação percentual de -50,8% (II 95%, -53,1% a -48,5%). ^46^

•A taxa de YLDs ajustada por idade atribuída a DAC foi 63 (II 95%, 41-89) por 100 mil. De 1990 a 2019, essa taxa apresentou redução de 5% (II 95%, -7% a -3%). ^46^

## Utilização e Custo da Atenção à Saúde

•De acordo com os dados administrativos do SIH/SUS, o número absoluto de hospitalizações por SCA em instituições públicas permaneceu estável de 2008 a 2019 ( Figura 3-14 ). Em 2019, houve 70.204 (33,4 por 100 mil habitantes) hospitalizações por SCA no país. O número de hospitalizações por IAM aumentou de 47.358 em 2008 a 80.614 em 2019, uma variação percentual de 70%, ou de 25,0 a 38,4 por 100 mil habitantes, uma variação percentual de 54% ( Figura 3-14 ). As hospitalizações por SCC diminuíram de 12.393 (6,5 por 100 mil) em 2008 para 6.703 (3,2 por 100 mil) em 2019 ( Figura 3-14 ). ^95^

•As hospitalizações no serviço público para realização de ICP não primária mais do que dobraram de 2008 (n=38.635) a 2019 (n=85.518, Figura 3-15 ), enquanto a ICP primária para tratamento do IAM aumentou 45% (de 7.648 para 11.099). Considerando as taxas por 100 mil habitantes, os valores de ICP não primária foram 20,4 em 2008 e 40,7 em 2019 (variação percentual: 100%) e os de ICP primária foram 4,0 em 2008 e 5,3 em 2019 (variação percentual: 31%). Enquanto isso, o número total de CRVM permaneceu estável no período ( Figura 3-15 ), totalizando 21.018 procedimentos (10,0 por 100 mil habitantes) em 2019. Em consequência, a ICP em comparação à CRVM aumentou, passando de 69% de todos os procedimentos em 2008 para 82% em 2019 ( Figura 3-16 ). ^95^

•A maioria das ICP realizadas em hospitais públicos nos últimos anos foi categorizada como ICP não primária.

A porcentagem de ICP primária entre todas as ICP permaneceu estável de 2009 a 2019 (entre 9% e 13%). ^95^

•Em 2019, as médias de permanência das hospitalizações por SCA, IAM, ICP e CRVM em hospitais públicos foram 5,4, 8,7, 3,8 e 12,0 dias, respectivamente, permanecendo estáveis desde 2008. ^95^

•De acordo com o SIH/SUS, o custo anual não ajustado associado com todas as admissões por SCA em hospitais públicos aumentou de R$ 44.710.681 em 2008 para R$ 81.167.005 em 2019. No mesmo período, o custo anual relacionado a hospitalização por IAM aumentou de R$ 65.019.331 para R$ 151.123.021, enquanto o custo associado a SCC diminuiu de R$ 7.798.578 para R$ 6.475.644. Convertendo em Int$ 2019 ajustado para paridade do poder de compra, o valor associado ao tratamento para SCA diminuiu discretamente nos últimos anos, chegando a Int$ 39.230.065 em 2019; esse gasto permaneceu estável no contexto do IAM (Int$ 73.041.576 em 2019) e diminuiu para a SCC (Int$ 3.129.842 em 2019, Figura 3-17 ). ^95^

•Em dólares internacionais, o valor médio reembolsado por hospitalização por SCA, IAM ou SCC diminuiu de 2008 a 2019 ( Figura 3 -18). Em 2019, os gastos médios por admissão para SCA, IAM e SCC foram R$ 1.156 (Int$ 559), R$ 1.875 (Int$ 906) e R$ 966 (Int$ 467), respectivamente. ^95^

•O banco de dados administrativo do SUS mostrou que o total reembolsado por ICP não primária foi R$ 546.132.199 (Int$ 263.959.497) em 2019. O custo correspondente para ICP primária foi R$ 74.907.756 (Int$ 36.204.812). O gasto médio pago por paciente foi R$ 6.386 (Int$ 3.087) para ICP não primária e R$ 6.749 (Int$ 3.262) para ICP primária. Com relação à CRVM, o total reembolsado foi R$ 278.544.224 (Int$ 134.627.464), correspondendo a um valor médio de R$ 13.253 (Int$ 6.406) por procedimento cirúrgico. ^95^

•Em dólares internacionais, o custo anual total relacionado a ICP não primária aumentou nos últimos anos, enquanto o relacionado a ICP primária permaneceu estável e o gasto com CRVM caiu ( Figura 3-19 ). O valor médio reembolsado por hospitalização diminuiu tanto para ICP quanto para CRVM desde 2008 ( Figura 3-20 ). ^95^

•Em 2015, utilizou-se uma abordagem de modelagem global para avaliar o impacto econômico (sistema de saúde e produtividade) de quatro condições cardíacas no Brasil (hipertensão, insuficiência cardíaca, IAM e fibrilação atrial), com estimativas de custo anual para 2015. Estimou-se que as quatro condições cardíacas afetassem ~45,7 milhões de pessoas no Brasil, correspondendo a 32% da população adulta. O IAM representou o maior custo financeiro, com prevalência estimada de 0,2% (334.978 casos), sendo o custo para o sistema de saúde por caso de US$ 48.118 e o custo de produtividade de US$ 18.678. ^124^

•O custo anualizado para um indivíduo com DAC crônica foi estimado em R$ 2.733 ± 2.307 pelo SUS, sendo que o custo para o paciente ambulatorial representou 54% do total. Para os planos de saúde privados, o custo foi estimado em R$ 6.788 ± 7.842, dos quais, 69% estavam relacionados a pacientes internados. Quanto ao custo dos pacientes ambulatoriais, os medicamentos foram responsáveis por R$ 1.154, representando, para os pagadores públicos e privados, 77% e 55% dos custos com os pacientes ambulatoriais e 42% e 17% do custo total, respectivamente. ^125^

•Um relato de uma clínica de DAC de um hospital público mostrou um custo anual médio para manejo ambulatorial de US$ 1.521 por paciente (valores de 2015). O custo médio por hospitalização foi US$ 1.976 e os gastos foram maiores no primeiro e último ano de seguimento. Angina instável, procedimentos de revascularização, diabetes, hipertensão e obesidade foram preditores de maior custo de hospitalização. ^126^

•Dados de 2008 a 2014 estimaram que 4.653.884 procedimentos cardíacos diagnósticos foram realizados no Brasil, incluindo 3.015.993 eletrocardiogramas, 862.627 angiografias invasivas e 669.969 exames nucleares, levando a um custo geral de US$ 271 milhões. Nessa avaliação geoespacial nacional do acesso à saúde, mortalidade por SCA foi associada a menor renda, assim como à realização de menor número de exames nucleares e maior número de testes de esforço e de cateterismos cardíacos. ^127^

•Um estudo usando metodologia de microcusto avaliou os custos associados com ICP em 40 pacientes de dois hospitais públicos de ensino em 2017. O custo mediano de ICP foi R$ 4.579 em um hospital e R$ 3.156 em outro. A maior parte da despesa deveu-se ao custo da prótese (72% e 81% do custo total). ^128^

•Um estudo quantitativo, descritivo e transversal realizado em um hospital filantrópico da cidade de São Paulo, avaliando 1.913 pacientes consecutivos submetidos a CRVM em 2012, relatou um custo médio por paciente de US$ 7.993 (mediana, US$ 6.463). O valor pago pelo sistema de saúde público foi US$ 3.450 (mediana, US$ 3.159), resultando em déficit de 51% do custo total para os provedores. ^129^

•Uma análise retrospectiva das solicitações médicas de beneficiários de planos de saúde foi realizada considerando os custos de hospitalização para pacientes admitidos com SCA entre 2010 e 2012. O custo médio por paciente em terapia medicamentosa apenas foi de R$ 18.262, o custo médio por paciente submetido a ICP, de R$ 30.611, e o custo médio por paciente submetido a CRVM, de R$ 37.455. ^130^

•Uma análise de 240 pacientes submetidos a CRVM isolada em hospital de referência em 2013 mostrou um custo médio de hospitalização de R$ 22.647 ± 28.106 (R$ 35.400 ± 40.509 para aqueles com alguma complicação e de R$ 13.997 ± 5.801 para aqueles sem complicação). ^131^

•Uma análise de custo de 101 pacientes submetidos a ICP no SUS em 2014/2015 mostrou um custo mediano de R$ 6.705 ± 3.116 por paciente. Os custos foram menores para ICP eletiva (R$ 5.085 ± 16) do que para ICP por SCA (R$ 6.854 ± 3.396). ^132^

## Qualidade da Atenção à Saúde

•Várias publicações avaliaram a qualidade do cuidado para SCA no Brasil. ^82,83,85,86,96,100–102,133,134^ Esses estudos salientam as oportunidades de melhoria no cuidado, além das diferenças regionais e heterogeneidade dos serviços público e privado que podem impactar os desfechos. Ademais, algumas publicações abordaram a implementação de estratégias para otimizar a qualidade do cuidado para SCA. ^86^ ^,^ ^102^ ^,^ ^133^ ^,^ ^134^

•No registro prospectivo ACCEPT, a taxa de adesão total a medicamentos recomendados pelas diretrizes (terapia antiplaquetária dupla, anticoagulantes parenterais, estatinas e betabloqueadores) foi 62% logo após admissão por SCA. Entre os pacientes com IAMCSST (n=1.714), 82% foram tratados com fibrinólise ou ICP primária. As taxas de reperfusão para IAM diferiram de acordo com a região do país: 87%, 85%, 73%, 67% e 66% no Sul, Sudeste, Nordeste, Centro-Oeste e Norte, respectivamente. Prescrição de AAS foi observada em 95% dos casos por ocasião da alta e em 86% no seguimento de 12 meses. Inibidores do receptor P2Y ^12^ foram prescritos em 92% dos casos na admissão, 79% na alta e 47% após 12 meses. Estatinas foram recomendadas para 93% dos pacientes na alta e 83% após 12 meses. Terapia considerada incompleta e hospitalização em instituição pública, entre outros fatores, foram associadas com eventos cardiovasculares maiores. ^82^

•Na análise do registro transversal BRACE, a qualidade do cuidado na SCA foi medida por um escore de desempenho que incluiu prescrição de AAS na admissão hospitalar, prescrição de AAS/betabloqueador/estatina na alta e terapia de reperfusão para pacientes com IAMCSST em até 12 horas do início dos sintomas, entre outros fatores. Escores mais baixos foram independentemente associados com maior risco de desfechos duros e morte hospitalar.

O escore foi mais baixo nas regiões Norte e Nordeste do que no restante do país tanto nas primeiras 24 horas quanto na alta hospitalar. Escores mais altos foram observados em hospitais de ensino *versus* não de ensino. As instituições públicas e privadas não diferiram significativamente quanto aos escores, embora a terapia de reperfusão para IAMCSST tenha sido mais frequente nos hospitais privados (86% *versus* 75%), enquanto a prescrição de AAS/estatina na alta tenha sido mais comum nos públicos. No geral, as porcentagens de pacientes que receberam prescrição de AAS, estatina e betabloqueador na alta foram 86%, 82% e 69%, respectivamente. ^83^

•Entre participantes incluídos no estudo ERICO entre 2009 e 2012, terapia de reperfusão foi realizada em 72% dos casos de IAMCSST. As taxas de tratamento medicamentoso durante hospitalização foram: 98% para AAS, 96% para clopidogrel e heparina, 92% para estatinas e 84% para betabloqueadores. ^85^

•No estudo VICTIM, o tempo médio entre o início dos sintomas e a admissão hospitalar foi mais longo nos hospitais públicos em comparação aos privados (25 ± 37 *versus* 9 ± 21 horas; respectivamente, P <0,001). A taxa de ICP primária foi mais baixa nos hospitais públicos em comparação aos privados (45% *versus* 78%, respectivamente, P <0,001). ^96^

•Em um hospital universitário de Belo Horizonte, avaliou-se a adesão a 13 medidas de desempenho pré-especificadas em 1.129 pacientes com IAMCSST ou IAMSSST hospitalizados entre 2011 e 2014. A adesão mediana foi 83% e o tratamento de 67% dos pacientes cumpriu pelo menos 80% das medidas de qualidade. A taxa de terapia de reperfusão nos casos de IAMCSST foi 56%. ^100^

•Em um relato do Projeto Minas Telecardio 2 incluindo 593 pacientes com SCA de 2013 a 2014, entre os indivíduos com IAMCSST, 46% receberam terapia de reperfusão, principalmente ICP primária. O tempo porta-balão foi maior do que 90 minutos em 37,5% desses pacientes. No geral, as taxas de prescrição de AAS, inibidores da P2Y _12_ e estatinas foram 97%, 86% e 81% em 24 horas e 93%, 69% e 86% na alta, respectivamente. ^101^

•Em outra publicação do Projeto Minas Telecardio 2, a qualidade do cuidado ao paciente com IAMCSST foi avaliada antes (n = 214) e após (n = 143) a implementação de um protocolo de tratamento coordenado entre 2014 e 2015. A taxa de terapia de reperfusão aumentou de 71% para 81% (P = 0,045), enquanto a porcentagem de pacientes recebendo prescrição de AAS e inibidores da P2Y _12_ aumentou de 94% e 88%, respectivamente, para 100% nos dois casos. Além disso, uma redução não significativa na chance de morte hospitalar foi relatada (OR, 0,73; IC 95%, 0,34–1,60). ^133^

•Em um estudo observacional retrospectivo de hospitais públicos em Belo Horizonte, a taxa de mortalidade hospitalar caiu após implementação de um sistema para tratamento de IAM, que incluiu tele-eletrocardiografia (12% em 2009 e 7% em 2011, P < 0,001). ^134^

•O estudo RESISST relatou desfechos no IAMCSST antes e após a implementação de uma rede regional integrada com suporte de telemedicina na cidade de Salvador. Os autores relataram um aumento nas taxas de reperfusão primária (de 29% para 54%, P <0,001), uma redução na taxa de mortalidade de 30 dias (de 20% para 5%, P <0,001) e um aumento no uso de terapia antiplaquetária dupla e de estatinas. ^102^

•O BRIDGE-ACS, um estudo randomizado por conglomerado, avaliou um programa multifacetado de melhoria da qualidade do tratamento de pacientes com SCA. Em comparação à prática rotineira, a intervenção, que incluiu material educacional para clínicos, lembretes, algoritmos e treinamento de manejo de caso, aumentou a chance de receber medicamentos elegíveis. Além disso, as taxas de evento cardiovascular hospitalar foram 5,5% no grupo intervenção e 7,0% no grupo controle [OR populacional médio (ORPA), 0,72 (IC 95%, 0,36-1,43)], enquanto as taxas de mortalidade por todas as causas em 30 dias foram 7,0% e 8,4% nos grupos intervenção e controle, respectivamente [ORPA, 0,79 (IC 95%, 0,46-1,34)]. ^86^

## Pesquisa Futura

•Dados adicionais são necessários para a melhor compreensão da distribuição epidemiológica da DAC no Brasil, em particular:

•Desenvolvimento de bases de dados nacionais para coletar informação precisa em tempo real sobre a epidemiologia das diferentes apresentações clínicas da DAC, incluindo medidas de prestação do cuidado, desempenho e desfecho;

•Revisões sistemáticas de taxas de prevalência e de mortalidade por SCA, angina estável, ICP e CRVM, incluindo amostras representativas de todas as áreas geográficas do país;

•Avaliação da efetividade de programas estruturados em âmbito nacional para medir a qualidade e o desempenho dos diferentes manejo (público, com e sem fins lucrativos) para entender a atual situação, além de desenhar estratégias visando à redução da morbimortalidade por DCV;

•Análises adicionais econômica e de custo-efetividade do impacto da DAC e de suas intervenções diagnóstica e terapêutica são necessárias, a partir de um nível macro e utilizando métodos de microcusteio para os sistemas de saúde público e privado;

•Desenvolvimento de programas estruturados para avaliar a prevalência, a incidência e o impacto clínico e econômico da DAC crônica no cenário ambulatorial.

## Incidência

•O estudo GBD estimou uma incidência de 260.661 (II 95%, 230.100-293.617) eventos de DAC (principalmente infarto do miocárdio) no Brasil em 2019. Como esperado, a taxa de incidência mostrou-se fortemente associada ao envelhecimento: 29 (II 95%, 23-36), 323 (II 95%, 268-386) e 737 (II 95%, 625-868) por 100 mil indivíduos com idade de 15-49 anos, de 50-69 anos e a partir de 70 anos, respectivamente. Em todos os grupos etários, a DAC foi mais incidente nos homens do que nas mulheres ( Figura 3-3 ). ^46^

•Em 2019, a taxa de incidência de DAC padronizada por idade foi 110 (II 95%, 97-124) por 100 mil habitantes na população total, 78 (II 95%, 69-88) por 100 mil mulheres e 148 (II 95%, 130-166) por 100 mil homens. ^46^

•Enquanto a taxa bruta de incidência de DAC aumentou continuamente de 1990 a 2019, a taxa padronizada por idade diminuiu levemente (-15%) de 1990 a 2000, permanecendo estável desde então (variação percentual: 3% de 2000 a 2019). De 1990 a 2019, a variação percentual na taxa de incidência de DAC ajustada por idade foi -12% (II 95%, -15% a -10%) na população total e 7% (95% UI, 1% a 12%), 2% (95% UI, -1% a 6%) e -19% (II 95%, -22% a -16%) entre indivíduos de 15-49 anos, 50-69 anos e a partir dos 70 anos, respectivamente. ^46^

•Em revisão sistemática de dados de saúde pública em 2012, as taxas de incidência de IAM e SCA por 100 mil habitantes foram 29,8 e 38, respectivamente. ^8^

## 4. CARDIOMIOPATIA E INSUFICIÊNCIA CARDÍACA

### CID-10 I42; I50; B57.2.

**Ver Tabelas 4-1 até 4-12 e Figuras 4-1 até 4-10**

**Table:** Abreviaturas usadas no Capítulo 4

BREATHE	I Registro Brasileiro de Insuficiência Cardíaca
CID-10	Classificação Estatística Internacional de Doenças e Problemas Relacionados à Saúde, 10 ^a^ Revisão
CMCh	Cardiomiopatia Chagásica
CMH	Cardiomiopatia Hipertrófica
CMNCh	Cardiomiopatia Não Chagásica
DALYs	Anos de vida perdidos ajustados por incapacidade (do inglês, *Disability-Adjusted Life-Years* )
DCh	Doença de Chagas
GBD	Global Burden of Disease
HR	Hazard Ratio
IC	Intervalo de Confiança
II	Intervalo de Incerteza
IIQ	Intervalo Interquartil
OR	Odds Ratio
RAP	Risco Atribuível na População
REMADHE	Estudo Educação Repetitiva e Monitoramento para Adesão para Insuficiência Cardíaca (do inglês, *Repetitive Education at Six-Month Intervals and Monitoring for ADherence in Heart Failure Outpatients* )
SDI	Índice Sociodemográfico (do inglês, *Sociodemographic Index* )
SEADE	Fundação Sistema Estadual de Análise de Dados
SIM	Sistema de Informações sobre Mortalidade
SUS	Sistema Único de Saúde
UF	Unidade Federativa
YLDs	Anos vividos com incapacidade (do inglês, *Years Lived with Disability* )
YLLs	Anos de vida perdidos por morte prematura (do inglês, *Years of Life Lost)*

**Tabela 4-1:** – Número de casos prevalentes e taxas de prevalência (por 100 mil) padronizadas por idade de cardiomiopatia e miocardite, e variação percentual das taxas, no Brasil e suas unidades federativas, 1990 e 2019.

Local	1990		2019		Variação percentual (II 95%)
	Número (II 95%)	Taxa (II 95%)	n (II 95%)	Taxa (II 95%)	
Brasil	55954,8 (38550,4;80474,4)	76,6 (53,4;107,2)	164904,6 (115339,9;226269,4)	73 (51,1;100,1)	-4,7(-9,5;0,8)
Acre	77,6 (51,6;113)	59,8 (41,8;84,7)	292,2 (203,7;411,2)	55,7 (39,2;77,4)	-6,9(-20,7;9,3)
Alagoas	893 (594,6;1319,1)	78,1 (54;111,5)	2230,7 (1576,7;3140,2)	75 (52,4;105,5)	-4,1(-17,2;11,5)
Amapá	53,5 (36,2;77,7)	68,9 (48,4;97)	273,7 (192,5;385,7)	64,4 (45,2;89,3)	-6,5(-19,7;8,1)
Amazonas	350,6 (236,1;515,3)	60,3 (42,1;85,6)	1454,6 (1014,7;2010,9)	58,1 (40,5;81)	-3,6(-17;11,8)
Bahia	3814,2 (2517,2;5533,9)	64,4 (43,6;91,4)	10094 (7121;14112,7)	63,7 (44,6;90,4)	-1,1(-16,6;16,1)
Ceará	3069,9 (2131,5;4409,4)	80,8 (56,5;114,9)	7835,9 (5565,8;10891,2)	80,5 (56,9;111,8)	-0,4(-13,8;15,1)
Distrito Federal	272,9 (165,7;416,5)	78,5 (52,8;112,3)	1545,6 (995;2290,9)	72,7 (48,6;102,9)	-7,3(-20;6,7)
Espírito Santo	791,4 (533;1142,4)	66,3 (46;94,8)	2501,4 (1761,3;3478,5)	61 (43;84,1)	-8(-20,4;6,7)
Goiás	1075,7 (651,2;1638)	74,8 (47,6;108,2)	4744,9 (3123,1;6832)	77,9 (51,8;110,8)	4,2(-9,8;20,3)
Maranhão	1059,9 (715,7;1564,1)	48,1 (33,1;68,5)	3064,9 (2111,4;4342)	49,1 (33,7;69,3)	2,2(-13,4;21,1)
Mato Grosso	351,2 (234,6;519,3)	65,6 (46;93)	1782,3 (1254;2514)	62,2 (44,1;87,1)	-5,1(-18,9;11,9)
Mato Grosso do Sul	518,2 (340,8;741,9)	77,6 (53,1;110,1)	2082 (1444,7;2891,3)	76,7 (53,4;105,2)	-1,1(-15,4;14,6)
Minas Gerais	6511,9 (4300,2;9654,8)	82,8 (56,3;117,4)	20996,2 (14121,1;29608,6)	80,2 (53,7;113,4)	-3,1(-15,6;11,8)
Pará	984,4 (668;1427,3)	59,5 (41,7;83,5)	3612,3 (2515,8;5033)	57,8 (40,4;80,2)	-3(-17,1;12,7)
Paraíba	2080,3 (1423,6;2989,5)	94 (65;132)	4211,6 (2938,5;5780,8)	86,6 (60;119,7)	-7,9(-19,8;5,9)
Paraná	2664,2 (1754,8;3847,1)	71,5 (49,2;100,3)	8066,4 (5604,5;11456,8)	64,4 (45,1;90,7)	-9,9(-21,9;4,4)
Pernambuco	2580,1 (1749,8;3669,6)	64,6 (44,8;90,5)	5776,7 (4013,6;8097,6)	61,1 (42,4;85,4)	-5,5(-19,1;9,3)
Piauí	636,4 (430,3;916,8)	54,2 (37,3;76,4)	1883,9 (1312;2665,4)	49,8 (34,7;70,9)	-8,2(-21,6;8,2)
Rio de Janeiro	6696,9 (4702,4;9614,9)	83,2 (58,6;115,6)	16891,5 (11678,1;23485,5)	76,5 (53;105,9)	-8,1(-20;5,6)
Rio Grande do Norte	902,7 (620,2;1261,7)	59,7 (41,7;82,6)	2195,4 (1530,2;3005,3)	56,9 (39,5;79,2)	-4,8(-18,6;11,8)
Rio Grande do Sul	3317,1 (2244,1;4733,2)	60,5 (42,4;85,4)	8432,1 (5886,6;11731,4)	53,8 (37,8;74,4)	-11,1(-23,5;3,9)
Rondônia	111,4 (68,3;174,7)	57 (39,6;80,7)	705,9 (490,3;1010,1)	53,2 (37,2;74,8)	-6,6(-20,8;10,9)
Roraima	34 (22;50)	94,5 (65,8;133,1)	254,9 (173,2;359,1)	84,8 (57,9;118,4)	-10,3(-22,8;3,5)
Santa Catarina	1501,3 (1036,2;2151,8)	74,6 (52,4;103,4)	5218,7 (3607,9;7226,4)	68,7 (47,8;95,2)	-7,9(-19,6;6,2)
São Paulo	14932,1 (10004;21456,5)	94,1 (64,2;132,4)	46530 (32481,2;63983,6)	89,2 (63,1;122,9)	-5,2(-17,1;9,4)
Sergipe	513,3 (343,1;744,4)	75,3 (52,4;104,8)	1526,3 (1062,2;2129,8)	72,8 (51;102,2)	-3,2(-15,8;11,3)
Tocantins	160,6 (102,1;248,9)	54,4 (37;78,4)	700,4 (467,8;1009)	53,9 (36;77,6)	-0,9(-15,2;15,5)

*Fonte: Dados derivados do estudo Global Burden of Disease 2019, Institute for Health Metrics and Evaluation, University of Washington. ^46^*

**Tabela 4-2:** – Número de casos incidentes e taxas de incidência (por 100 mil) padronizadas por idade de cardiomiopatia e miocardite, e variação percentual das taxas, no Brasil e suas unidades federativas, 1990 e 2017.

Local	1990		2017		Variação percentual (II 95%)
	Número (II 95%)	Taxa (II 95%)	n (II 95%)	Taxa (II 95%)	
Brasil	54520,4 (48574,3;61320,7)	46,3 (41,5;52,1)	103879,4 (92495,6;117294,5)	46,7 (41,8;52,6)	0,8 (-0,3;1,8)
Acre	125,8 (111;143)	44,8 (40;50,7)	340,7 (303,3;383,1)	46 (41,1;52)	2,8 (-0,5;6,4)
Alagoas	826,5 (732,4;929,4)	43,6 (38,8;49,3)	1551,4 (1378,8;1749)	46,5 (41,5;52,4)	6,8 (3,5;10,2)
Amapá	85,4 (75,2;97,2)	47,5 (42,5;53,4)	311,1 (274,3;352,6)	47,6 (42,5;53,6)	0,3 (-2,8;3,9)
Amazonas	645,3 (565,6;732,6)	46,4 (41,5;52,6)	1586,5 (1410,7;1793,3)	46,6 (41,6;52,8)	0,5 (-2,9;4,3)
Bahia	4183 (3713,7;4708,9)	45,3 (40,3;50,9)	7177 (6366,6;8059,8)	45,1 (40;50,7)	-0,3 (-3,4;2,7)
Ceará	2232,8 (1990,7;2511,3)	43,2 (38,7;48,7)	4589,2 (4084,6;5197,2)	46,4 (41,4;52,7)	7,5 (3,4;11)
Distrito Federal	502,7 (439,4;574,4)	45,2 (40,3;51,3)	1258,3 (1107,5;1428,3)	45,3 (40,4;51)	0,2 (-3,3;3,7)
Espírito Santo	976,1 (856,5;1104,3)	48,1 (42,8;54,4)	1992,5 (1757;2263,2)	48,2 (42,9;54,4)	0,2 (-3,2;3,8)
Goiás	1391,1 (1220,9;1574,2)	46,3 (41,4;52,2)	3233,1 (2869,8;3671,6)	47,3 (42,2;53,5)	2,3 (-0,8;5,4)
Maranhão	1568,5 (1390,4;1771,6)	43,3 (38,6;48,9)	3314,6 (2958,6;3716,8)	46,7 (41,7;52,6)	8 (4,5;12)
Mato Grosso	628,1 (549,8;715,5)	45,6 (40,7;51,7)	1567,2 (1394,8;1780,2)	46,8 (41,9;52,9)	2,7 (-1;6,3)
Mato Grosso do Sul	648,6 (572,4;728)	48,6 (43,4;54,8)	1426,5 (1264,1;1616,6)	50,6 (45,2;57)	4,1 (0,8;7,7)
Minas Gerais	5880,7 (5207,1;6646,3)	46 (41;52)	11181 (10003,5;12646,6)	47 (42,2;53,2)	2,2 (-1;5,5)
Pará	1548,2 (1365,8;1749,4)	45,4 (40,6;51,3)	3561 (3155,4;4003,7)	45,5 (40,5;51,4)	0,2 (-3,2;3,9)
Paraíba	1181,3 (1051,4;1331,4)	43,2 (38,6;48,9)	2068,4 (1841,2;2347,2)	46,3 (41,2;52,5)	7,1 (3,6;10,5)
Paraná	3140,9 (2777,5;3572,6)	47,1 (41,9;53,3)	5728 (5060,1;6535,4)	47 (41,9;53,2)	-0,2 (-3,8;3)
Pernambuco	2639 (2333,6;2961,8)	45,6 (40,5;51,3)	4609,7 (4098,5;5160,5)	46,1 (41,1;51,6)	1,1 (-2,4;4,5)
Piauí	865,4 (765,7;974,5)	43,8 (39;49,4)	1652,7 (1471;1863,8)	45,8 (40,7;51,6)	4,5 (0,8;8,3)
Rio de Janeiro	5321,9 (4706,8;6019,7)	46,7 (41,6;52,5)	9236,1 (8183,4;10524,9)	46,4 (41,3;52,5)	-0,5 (-4,1;3,1)
Rio Grande do Norte	886,2 (790,4;995,2)	44,3 (39,7;49,8)	1723,7 (1535,1;1947,9)	46,2 (41,2;52,1)	4,2 (1;7,7)
Rio Grande do Sul	3927,6 (3482,4;4424,5)	49,5 (44,1;55,8)	6475,7 (5749;7325,7)	48,5 (43,2;54,5)	-2,1 (-5,5;1,4)
Rondônia	330,5 (289,3;379,1)	45,6 (40,6;51,4)	746,3 (660,9;847,2)	46 (41,2;52,2)	0,9 (-2,6;4,4)
Roraima	61,2 (53,3;70,9)	46,1 (41,2;52)	212,3 (187,3;240,4)	46,4 (41,3;52,4)	0,9 (-2,4;4,2)
Santa Catarina	1694 (1503,4;1912,2)	47,8 (42,9;54,2)	3554,7 (3148,4;4033,5)	47,5 (42,4;53,4)	-0,7 (-4,2;3,5)
São Paulo	12421,8 (10980,5;13984,7)	47,6 (42,4;53,5)	22999,9 (20352,4;26154,9)	46,6 (41,6;52,7)	-1,9 (-5,5;1,4)
Sergipe	520,8 (463,6;584,1)	45,3 (40,5;51,4)	1075,4 (957,1;1218,6)	47,3 (42,1;53,4)	4,5 (1;7,8)
Tocantins	287 (253,9;325,8)	44,8 (40;50,5)	706,6 (629;798)	47,1 (42,1;53,1)	5,1 (1,6;8,9)

*Fonte: Dados derivados do estudo Global Burden of Disease 2017, Institute for Health Metrics and Evaluation, University of Washington. ^46^*

**Tabela 4-3:** – Taxas de incidência de cardiomiopatia e miocardite (por 100 mil) e variação percentual das taxas, por idade e sexo, Brasil, 1990 e 2019.

Sexo e grupo etário	1990	2019	Percent Change (95% UI)
**Ambos**			
15-49 anos	10.9 (7.7;14.8)	11.5 (8.1;15.9)	5.3 (-1.3;12.9)
50-69 anos	26 (17.2;35.7)	26.1 (17.1;36)	0.4 (-0.4;1)
5-14 anos	6.8 (3.8;10.8)	6.8 (3.8;10.8)	0.3 (-0.1;0.9)
70+ anos	65.5 (43.8;94.9)	68.7 (47.3;96.9)	4.9 (-0.4;11.8)
Padronizada por idade	15.8 (12.7;19.2)	15.8 (12.7;19.2)	-0.2 (-0.3;-0.2)
Todas as idades	12.5 (10;15.3)	16.6 (13.4;20.2)	32.6 (22.7;42.9)
Abaixo de 5	5.9 (3.6;8.5)	5.9 (3.6;8.5)	0.1 (0.1;0.2)
**Feminino**			
15-49 anos	9.1 (6.4;12.6)	9.6 (6.8;13.3)	5.4 (-1.5;13.3)
50-69 anos	21.3 (14;29.4)	21.5 (14.1;29.8)	0.9 (0;1.8)
5-14 anos	5.6 (3.2;9.1)	5.6 (3.2;9.1)	0.2 (-0.2;0.7)
70+ anos	56.8 (38.4;82.6)	60.5 (41.7;83.6)	6.6 (0.6;14.1)
Padronizada por idade	13.2 (10.6;16.1)	13.2 (10.6;16.1)	-
Todas as idades	10.6 (8.4;13.1)	14.5 (11.6;17.8)	36.1 (25.6;47.3)
Abaixo de 5	4.8 (2.9;7.1)	4.8 (2.9;7.1)	-
**Masculino**			
15-49 anos	12.7 (9;17.2)	13.4 (9.5;18.3)	5.1 (-1.2;12.2)
50-69 anos	31 (20.3;42.9)	31.2 (20.5;43.2)	0.6 (0;1.1)
5-14 anos	7.9 (4.5;12.5)	7.9 (4.5;12.5)	0.2 (-0.2;0.8)
70+ anos	76.3 (50.4;111)	80 (54.3;114.6)	4.7 (0;10.8)
Padronizada por idade	18.6 (15.1;22.7)	18.6 (15.1;22.7)	0 (0;0)
Todas as idades	14.4 (11.5;17.6)	18.7 (15;22.9)	30.2 (20.7;40.1)
Abaixo de 5	7 (4.2;10)	7 (4.2;10)	-

*Fonte: Dados derivados do estudo Global Burden of Disease 2019, Institute for Health Metrics and Evaluation, University of Washington. ^*46*^*

**Tabela 4-4:** – Número de mortes e taxas de mortalidade padronizadas por idade (por 100 mil) por cardiomiopatia e miocardite, e variação percentual das taxas, no Brasil e suas unidades federativas, 1990 e 2019.

Local	1990		2019		Variação percentual (II 95%)
	Número (II 95%)	Taxa (II 95%)	n (II 95%)	Taxa (II 95%)	
Brasil	13408.9 (10490.2;14283.2)	15.9 (12.4;17.1)	21425.7 (18940;25167.1)	9.4 (8.3;11.1)	-40.8 (-46.4;-25.4)
Acre	17.8 (15.6;22.3)	11.1 (9.7;14.5)	40.6 (32.9;66)	7 (5.6;11.5)	-37.3 (-50.5;-10.7)
Alagoas	252 (186.1;296.6)	17.2 (12.7;20.2)	338.7 (289.2;405.6)	10.6 (8.9;12.8)	-38.7 (-51.1;-10.7)
Amapá	8.7 (7.6;11.1)	9.2 (8;11.7)	35 (29.5;49.9)	7 (5.8;10.1)	-24.3 (-36.3;-6.6)
Amazonas	76.9 (68.3;96.6)	10.8 (9.5;13.3)	160.4 (126.1;276.9)	5.6 (4.3;9.9)	-48 (-59.3;-20.6)
Bahia	945.3 (757.8;1076.4)	13.8 (11;15.9)	1209.7 (947.1;1895)	7.5 (5.8;11.7)	-45.9 (-59.6;-6.3)
Ceará	544.6 (443.7;645.1)	11.9 (9.6;14.2)	862 (699.5;1105.8)	8.7 (7.1;11.2)	-26.8 (-44.4;0.5)
Distrito Federal	115.1 (71.8;134.1)	23.8 (13.8;27.9)	262.2 (177.5;309.1)	13 (8.6;15.5)	-45.2 (-54.4;-33.3)
Espírito Santo	186.7 (162.6;203.2)	14.1 (12.2;15.4)	329.7 (270;483.2)	7.9 (6.4;11.7)	-43.9 (-54.6;-15.5)
Goiás	533 (287.6;636.2)	29.8 (15;35.7)	824.6 (614.7;999.2)	12.7 (9.4;15.4)	-57.2 (-66.1;-34.2)
Maranhão	262.1 (178.7;422)	7.3 (5;13.4)	472.7 (356.7;908.9)	7 (5.2;13.8)	-3.9 (-31.6;35.7)
Mato Grosso	103.7 (83.5;119.2)	14.3 (11.3;16.4)	234.8 (199.8;318.2)	7.7 (6.5;10.4)	-46.3 (-56.3;-21.7)
Mato Grosso do Sul	157.5 (107.9;175.7)	19.9 (13.3;22.4)	264.2 (223.8;343.1)	9.6 (8.1;12.2)	-51.9 (-61;-22.6)
Minas Gerais	1742.7 (1144.9;1958.6)	19.3 (12.8;21.8)	2284.8 (1929.8;2701.6)	8.9 (7.5;10.5)	-54.1 (-62;-33)
Pará	201.4 (175.6;259.7)	10.4 (9.1;13.2)	407.4 (322.7;677.1)	5.9 (4.6;9.8)	-43.5 (-54.9;-19.1)
Paraíba	363.4 (244.6;426.7)	15.4 (10.3;18.2)	522.5 (421.5;611.6)	10.7 (8.7;12.5)	-30.2 (-43.9;-7.1)
Paraná	753.1 (572.7;822.2)	17.7 (13.1;19.5)	965.5 (791.3;1393.5)	7.9 (6.5;11.2)	-55.3 (-64.4;-27.5)
Pernambuco	563.7 (492;634.8)	12.9 (11.2;14.6)	906.9 (766.5;1203.7)	9.3 (7.8;12.5)	-28.2 (-39.6;-6.6)
Piaui	169.9 (145.7;201.3)	11.7 (9.8;14.5)	237.8 (187.3;387.2)	6.2 (4.9;10)	-46.9 (-59.7;-22.1)
Rio de Janeiro	1214.1 (1020.8;1481.7)	13.9 (11.9;16.8)	2858.5 (1949.9;3341.5)	13.2 (9.1;15.4)	-4.8 (-38.5;13)
Rio Grande do Norte	153.2 (132.1;193.4)	9.2 (7.8;11.9)	224.3 (167;392)	5.7 (4.2;9.8)	-38.6 (-53.8;-9.5)
Rio Grande do Sul	586.9 (507.9;899.2)	10 (8.6;15.1)	931.9 (733;1581.7)	6.2 (4.9;10.5)	-37.6 (-47.5;-21.5)
Rondônia	40.6 (33.5;51.5)	15.5 (12.9;18.2)	105.9 (84.9;156.8)	7.4 (6;11)	-52.3 (-62.9;-30.9)
Roraima	11.4 (7.5;13.3)	24.2 (15.4;28.1)	43.6 (32.8;49.3)	13.9 (10.6;16)	-42.4 (-51.6;-23.7)
Santa Catarina	351.2 (292.7;387.2)	16.2 (12.8;18)	598.5 (506;823.3)	8.1 (6.8;10.9)	-50 (-58.9;-30.3)
São Paulo	3890.4 (2446.4;4363.8)	22 (13.2;24.9)	6025.5 (4305.2;7018)	11.9 (8.5;13.8)	-46 (-53.6;-31)
Sergipe	109 (93.2;123)	15 (12.6;17.2)	163.4 (130.9;243.4)	7.4 (5.9;11.1)	-50.7 (-62.1;-21.5)
Tocantins	54.6 (43;63.8)	15.2 (11.6;18)	114.6 (94.2;159.5)	8.3 (6.8;11.7)	-45.2 (-57.4;-12.6)

*Fonte: Dados derivados do estudo Global Burden of Disease 2019, Institute for Health Metrics and Evaluation, University of Washington.* ^*46*^

**Tabela 4-5:** – Taxas de mortalidade por cardiomiopatia e miocardite (por 100 mil) e variação percentual das taxas, por idade e sexo, Brasil, 1990 e 2019.

Sexo e grupo etário	1990	2019	Variação percentual (II 95%)
**Ambos**			
Padronizada por idade	15,9 (12,4;17,1)	9,4 (8,3;11,1)	-40,8 (-46,4;-25,4)
Abaixo de 5	5,7 (4,5;7,2)	2,5 (2;3,1)	-55,5 (-68,3;-37,2)
5-14 anos	0,5 (0,4;0,5)	0,3 (0,3;0,4)	-24,9 (-39,7;-9,3)
15-49 anos	3 (2,5;3,2)	2,5 (2;2,8)	-17,3 (-25,6;-7,7)
50-69 anos	29,2 (22;31,4)	17,2 (15,1;20)	-41 (-47,2;-25,7)
70+ anos	127,2 (96,5;138,3)	84,6 (72,1;104)	-33,5 (-42;-10)
Todas as idades	9 (7;9,6)	9,9 (8,7;11,6)	9,8 (-1,4;37,4)
**Feminino**			
Padronizada por idade	13,5 (9,9;14,7)	7,2 (6,2;9)	-46,6 (-54,8;-17,5)
Abaixo de 5	5,8 (3,9;7,3)	2,3 (1,8;2,8)	-60,5 (-71,2;-36,2)
5-14 anos	0,4 (0,4;0,5)	0,3 (0,2;0,4)	-30,9 (-42,1;-9,8)
15-49 anos	2 (1,5;2,1)	1,4 (1,2;1,5)	-30,6 (-39,3;-11)
50-69 anos	21,9 (14,8;24,2)	10,9 (9,5;13,6)	-50,1 (-58,5;-20,8)
70+ anos	118,7 (85,2;131,5)	76,9 (63;97,4)	-35,2 (-46;4,4)
Todas as idades	7,8 (5,7;8,4)	8,3 (7,1;10,3)	5,6 (-10,9;64,1)
**Masculino**			
Padronizada por idade	18,7 (13,2;20,4)	12,1 (10;15,1)	-35,4 (-43,3;-5,8)
Abaixo de 5	5,6 (3,8;7,7)	2,8 (2;3,5)	-50,6 (-68,7;-20,8)
5-14 anos	0,5 (0,4;0,6)	0,4 (0,3;0,5)	-19,9 (-41,6;4)
15-49 anos	4,2 (3,2;4,6)	3,7 (2,7;4,2)	-11 (-26,7;2,7)
50-69 anos	37 (25,8;40,4)	24,3 (19,5;28,9)	-34,3 (-42,7;-7,6)
70+ anos	137,9 (94;152,9)	95,2 (78,7;127,8)	-31 (-42;9,4)
Todas as idades	10,2 (7,3;11,1)	11,6 (9,5;14,3)	13,4 (-0,7;63,1)

*Fonte: Fonte: Dados derivados do estudo Global Burden of Disease 2019, Institute for Health Metrics and Evaluation, University of Washington. ^*46*^*

**Tabela 4-6:** – Número de DALYs e taxas de DALYs padronizadas por idade (por 100 mil) por cardiomiopatia e miocardite, e variação percentual das taxas, no Brasil e suas unidades federativas, 1990 e 2019.

Local	1990		2019		Variação percentual (II 95%)
	Número (II 95%)	Taxa (II 95%)	n (II 95%)	Taxa (II 95%)	
Brasil	431381,3 (349546,5;465068,6)	399,4 (319,6;426,4)	545772,4 (484988,7;621356,8)	238,6 (213;272,3)	-40,3(-45,8;-27,9)
Acre	722,8 (619,6;863,1)	272,1 (238,3;338,1)	1229,2 (1012,3;1861,1)	172,3 (141,3;266,4)	-36,7(-48,7;-14)
Alagoas	10120,9 (7613,2;12457,1)	491,5 (368,6;575,8)	9795 (8429,4;11297,2)	287,2 (248,3;330,5)	-41,6(-53,2;-22,6)
Amapá	297,3 (252,1;377,5)	209,2 (183,3;265)	1082,7 (939,5;1485,3)	174,1 (150,6;240,6)	-16,8(-28,8;-0,8)
Amazonas	2624,3 (2276,7;3320,4)	238,6 (212,3;300,2)	4833,5 (3967;7682,5)	144,2 (117,3;234,9)	-39,6(-50,3;-16,2)
Bahia	31147,8 (25438,5;35521,1)	360,6 (285,8;411,6)	33834,2 (27283,1;46340)	211,1 (169,7;287,7)	-41,5(-55,2;-10,8)
Ceará	16413,7 (13000,4;20157,3)	260,6 (210,1;309,8)	18369,4 (16682,7;20144,2)	188,2 (170,6;206,9)	-27,8 (-41,3;-10,1)
Distrito Federal	22092,3 (17022,6;28315,1)	375,8 (300,5;461,9)	23436,2 (19481,4;28927,2)	232,7 (193,4;287)	-38,1(-53,2;-18,4)
Espírito Santo	4337,8 (2752,8;5019,5)	513,1 (321;593,1)	7071,6 (4676,3;8364,2)	264,3 (180,3;309,9)	-48,5(-56,3;-37,4)
Goiás	5815,7 (5216;6419,7)	326,8 (289,2;356)	8951,2 (7601,8;12062,7)	209 (177,8;281,7)	-36(-46,3;-14,9)
Maranhão	16299,6 (9819,3;19412,6)	652,4 (364,1;778,8)	20523,4 (15577,5;24450)	294,8 (224,3;350,1)	-54,8(-64,3;-32,3)
Mato Grosso	14600,5 (9341,4;21713)	279,7 (193,3;432,5)	13913,1 (10913,9;22240,9)	185,5 (144,9;308)	-33,7(-56,5;-3,8)
Mato Grosso do Sul	3653,5 (2937,1;4296,1)	329,9 (267,5;377,3)	6406,5 (5543;8476,1)	188,5 (163,6;246,6)	-42,9(-53,3;-20,3)
Minas Gerais	4885,5 (3587,2;5423,8)	446,6 (313,4;496,6)	6646 (5713;8721,8)	227 (196,5;295,2)	-49,2(-58;-23,3)
Pará	6504,4 (5594,8;8556,8)	234,7 (203,8;304,6)	11934,4 (9785,4;18602,1)	154,4 (126,4;242)	-34,2(-46,7;-11,4)
Paraíba	12024,3 (8553,7;14204,9)	431,4 (300,4;502,6)	12785,5 (10190,5;14695,4)	275,2 (220,1;316,3)	-36,2(-47,4;-20,6)
Paraná	22264,3 (17934,9;23976,9)	397,2 (309,5;430,3)	22812,7 (18990,6;32682,4)	182,6 (152,5;255,5)	-54(-62,8;-27,9)
Pernambuco	18399,6 (15769,6;21268,1)	329,9 (284,7;372)	25233 (21518,1;30713,1)	247,5 (212,1;302,5)	-25(-36,1;-11,2)
Piauí	6969,2 (5802,9;8446,6)	324,4 (276,8;385,1)	6207,9 (5130,4;9242,6)	165,6 (137;246,5)	-49(-61,3;-27,4)
Rio de Janeiro	38312,2 (31811,2;46419,8)	357,1 (300,2;427,9)	71716 (46879,8;84553,9)	339,7 (225,5;397,7)	-4,9(-40,6;13,4)
Rio Grande do Norte	5015,5 (4362,7;6077,7)	245,3 (213,5;305,8)	5882,6 (4598,4;9395,7)	152,6 (119,7;243,4)	-37,8(-51,4;-15,2)
Rio Grande do Sul	16956,7 (14758;25507,2)	235,3 (205,2;353,1)	20924,4 (16731,2;34591,4)	148,7 (120,1;241)	-36,8(-45,4;-23,2)
Rondônia	1570 (1255,2;2025)	320,2 (271,4;395,1)	2888,2 (2353,2;4117,1)	178,6 (146,2;255,3)	-44,2(-55,8;-22,1)
Roraima	426,7 (289,1;506,9)	490,8 (321,1;566,9)	1273,9 (964,2;1442)	295,1 (225;332,4)	-39,9(-49;-21,5)
Santa Catarina	10131 (9082,5;11640,1)	350,2 (302,4;387,2)	14421,3 (12268,6;20029,6)	185,8 (158,6;254,9)	-47(-55,1;-30,5)
São Paulo	116500,2 (79828,7;128970,3)	509,1 (334,8;567,6)	149627 (103165,1;174050,1)	294,5 (205,5;340,4)	-42,1(-49,3;-30,3)
Sergipe	3629 (3125,9;4094)	351,7 (303,7;394,9)	4389,6 (3599,6;6196,4)	190,2 (156,5;267,7)	-45,9(-57,3;-21)
Tocantins	2297,9 (1812;2752)	362,6 (285,6;424,6)	3071,7 (2577,8;3989,1)	204,4 (171,7;268,2)	-43,6(-55,4;-16,6)

*Fonte: Fonte: Dados derivados do estudo Global Burden of Disease 2019, Institute for Health Metrics and Evaluation, University of Washington. ^46^*

**Table 4-7:** – Rates of DALYs due to cardiomyopathy and myocarditis (per 100 000) and percent change of rates, by age and sex, Brazil, 1990 and 2019.

Grupo etário e sexo	1990	2019	Variação percentual (II 95%)
**Ambos **			
Padronizada por idade	399,4 (319,6;426,4)	238,6 (213;272,3)	-40,3 (-45,8;-27,9)
Abaixo de 5	498,4 (398,1;630,1)	222,3 (171,8;275,3)	-55,4 (-68,1;-37)
5-14 anos	37,1 (31,7;41,3)	27,9 (22,7;32,8)	-24,7 (-39,2;-9,3)
15-49 anos	156,7 (127,9;166,9)	127,2 (102;139,6)	-18,8 (-26,7;-9,9)
50-69 anos	865,7 (657,8;930,7)	516,7 (455,5;593,5)	-40,3 (-46,6;-25,6)
70+ anos	1841,8 (1402,3;2004,5)	1122,6 (969,8;1383,9)	-39,1 (-46,4;-18,4)
Todas as idades	289,8 (234,9;312,5)	251,9 (223,8;286,8)	-13,1 (-22,1;5,3)
**Feminino**			
Padronizada por idade	321,1 (237,7;346,2)	165,7 (147,3;198)	-48,4 (-55;-21,6)
Abaixo de 5	504,1 (343,7;638,6)	199,6 (160;250)	-60,4 (-71,2;-36)
5-14 anos	34,7 (29,5;39,3)	24,1 (20,1;29)	-30,6 (-41,7;-9,8)
15-49 anos	101,3 (78,6;109,6)	68,8 (61,5;77,4)	-32,1 (-40,4;-13,7)
50-69 anos	644,9 (441,8;712,1)	324,8 (283,4;403,2)	-49,6 (-57,7;-21,2)
70+ anos	1651,5 (1165,2;1824,5)	953,5 (799;1213,2)	-42,3 (-51,4;-8,7)
Todas as idades	237,7 (176,4;258,6)	181,1 (160,3;216,3)	-23,8 (-34,2;14,6)
**Masculino**			
Padronizada por idade	484,8 (353,6;528,5)	320,4 (257,7;371,8)	-33,9 (-41,8;-9,6)
Abaixo de 5	492,9 (334,4;675,3)	244,1 (181;309,8)	-50,5 (-68,6;-20,5)
5-14 anos	39,5 (31,2;46,4)	31,6 (23;38,5)	-19,9 (-41,1;3,6)
15-49 anos	213,9 (163,3;233,8)	187,1 (137,3;210,2)	-12,5 (-27,6;0)
50-69 anos	1105,3 (785,4;1210,3)	733,9 (585,9;861,6)	-33,6 (-41,9;-8)
70+ anos	2078,5 (1430,3;2306,7)	1353,1 (1144,9;1801,6)	-34,9 (-44,9;1)
Todas as idades	343,1 (255,5;380,3)	326,1 (260,4;376,9)	-5 (-17,6;28,2)

*Fonte: Fonte: Dados derivados do estudo Global Burden of Disease 2019, Institute for Health Metrics and Evaluation, University of Washington. ^*46*^*

**Tabela 4-8:** – Número de mortes e taxas de mortalidade padronizadas por idade (por 100 mil) por doença de Chagas, e variação percentual das taxas, no Brasil e suas unidades federativas, 1990 e 2019.

Local	1990		2019		Variação percentual (II 95%)
	Número de mortes (II 95%)	Taxa de mortalidade (II 95%)	Número de mortes (II 95%)	Taxa de mortalidade (II 95%)	
Brasil	7903,9 (2438,4;10073)	8,6 (2,8;10,9)	6523,3 (3350,2;11226,8)	2,8 (1,4;4,8)	-67,5(-76,5;-0,4)
Acre	6,4 (3,6;18,3)	4 (2,3;11,4)	10,9 (5,1;31,4)	1,8 (0,8;5,1)	-55,1(-75,4;2,3)
Alagoas	92,9 (38,9;165,2)	6,9 (2,9;12,5)	112,1 (73,1;189,6)	3,5 (2,3;5,9)	-49,4(-68;0,7)
Amapá	2,5 (1,6;6,1)	2,5 (1,6;6,3)	6,9 (2,6;25,2)	1,3 (0,5;4,8)	-48(-73;4,4)
Amazonas	22,3 (13,7;57,6)	2,8 (1,7;7,4)	38,1 (12,7;139,8)	1,3 (0,4;4,8)	-53,7(-77,5;0)
Bahia	716 (174,3;1023,7)	10,4 (2,6;14,8)	750,6 (287,4;1055,3)	4,6 (1,8;6,5)	-55,2(-68,1;2,8)
Ceará	120,7 (57,8;351,9)	3 (1,4;8,7)	177,6 (83,3;507,5)	1,8 (0,8;5,1)	-39,9(-67,7;15,5)
Distrito Federal	198 (14,4;307,3)	35,1 (2,5;53,8)	172 (18,3;271)	7,7 (0,7;12,3)	-78,1(-83,6;-5,2)
Espírito Santo	46,9 (33,7;102,7)	3,3 (2,3;7,3)	59,2 (24,3;205,2)	1,4 (0,6;4,7)	-58,1(-78,4;2,1)
Goiás	1096,7 (67,1;1729,9)	52,3 (3,3;82,4)	705,6 (105,8;1137,9)	10,7 (1,5;17,3)	-79,6(-84,3;-5,8)
Maranhão	89,2 (20,7;388,3)	3,5 (0,8;15,6)	121,2 (42,7;415)	1,8 (0,7;6,3)	-47,6(-71,8;23,7)
Mato Grosso	52,8 (21,6;70,1)	6,4 (2,7;8,6)	77 (45,1;152,2)	2,3 (1,4;4,6)	-63,6(-76,5;1,2)
Mato Grosso do Sul	67,7 (24,9;87)	7,4 (2,9;9,4)	66,9 (40,1;135,5)	2,3 (1,4;4,6)	-69,1(-80,4;-5,3)
Minas Gerais	1976,9 (279,4;2880,5)	19,4 (2,9;28,1)	1215,5 (329,2;1749)	4,6 (1,2;6,6)	-76,2(-82,1;-3,9)
Pará	86,3 (56,7;182,4)	4,1 (2,7;9)	110,3 (49,2;334,7)	1,6 (0,7;4,7)	-61,9(-80,3;-1,1)
Paraíba	83,9 (42,3;228,1)	3,7 (1,9;10,2)	95,9 (54,3;236,1)	2 (1,1;4,9)	-45,8(-68,2;2,2)
Paraná	415,3 (132,6;537)	8,4 (2,8;10,7)	316,7 (167,2;632,6)	2,4 (1,3;4,8)	-70,7(-80,7;1)
Pernambuco	243,5 (128,6;419,2)	5,4 (2,9;9,5)	237,6 (150;541,9)	2,4 (1,5;5,4)	-56(-73,2;2,4)
Piauí	86,7 (36,4;173,1)	6,4 (2,7;12,9)	101,6 (67,2;192,6)	2,7 (1,8;5,1)	-58(-74,1;-0,2)
Rio de Janeiro	345,3 (224,4;698,8)	3,6 (2,4;7,2)	297,8 (135,9;1073)	1,3 (0,6;4,8)	-62,9(-81,2;0)
Rio Grande do Norte	47,4 (27,1;134,3)	3 (1,7;8,5)	57,6 (24,3;176,4)	1,5 (0,6;4,5)	-50,9(-73,2;-1,2)
Rio Grande do Sul	211 (149;418,7)	3,3 (2,3;6,5)	202 (91,2;715,3)	1,3 (0,6;4,7)	-60,1(-79,8;0,7)
Rondônia	31,3 (10,4;42,3)	9,7 (3,3;12,9)	38,5 (24,9;79,8)	2,5 (1,6;5,1)	-73,9(-84,2;-1,7)
Roraima	2,1 (1,3;5,7)	3,6 (2,2;9,9)	5,4 (1,9;19,7)	1,5 (0,5;5,3)	-58,8(-79,3;4,7)
Santa Catarina	76,3 (55;170,7)	3,1 (2,2;7)	96,2 (38,7;352,7)	1,2 (0,5;4,3)	-61,2(-80,7;0)
São Paulo	1718,8 (479,1;2275,6)	8,3 (2,5;10,8)	1352,9 (542,9;2400,9)	2,5 (1;4,5)	-69,4(-78,6;-1,4)
Sergipe	29,8 (18,4;71,4)	3,9 (2,4;9,6)	39,2 (19,3;110,3)	1,7 (0,9;4,9)	-56,1(-75;-8,1)
Tocantins	37,4 (10,8;60,2)	9,9 (2,8;16)	58,1 (22,9;82,6)	4,1 (1,6;5,8)	-58,3(-75,5;2,4)

*Fonte: Fonte: Dados derivados do estudo Global Burden of Disease 2019, Institute for Health Metrics and Evaluation, University of Washington. ^46^*

**Tabela 4-9:** – Número de casos prevalentes e taxas de prevalência (por 100 mil) padronizadas por idade de insuficiência cardíaca por todas as causas, e variação percentual das taxas, no Brasil e suas unidades federativas, 1990 e 2017.

Local	1990		2017		Variação percentual (II 95%)
	Número (II 95%)	Taxa (II 95%)	n (II 95%)	Taxa (II 95%)	
Brasil	670194,8 (589952,6;753672,6)	818,1 (718,1;922,8)	1686320,1 (1478563,8;1890537,3)	777,2 (680;874,8)	-5 (-7,1;-3)
Acre	1235,9 (1083,5;1395,6)	764,3 (668,5;869)	4025,6 (3559,4;4534,9)	728,8 (638,1;830,1)	-4,6 (-10,1;1,8)
Alagoas	9783 (8509,2;11210,5)	752,5 (654,5;861,9)	22691,5 (19784,3;25922)	764,8 (664,4;879,3)	1,6 (-5,7;8,3)
Amapá	748,2 (662;841,1)	779,1 (680,7;889,6)	3278,9 (2865,5;3672,3)	749,3 (651,9;845,2)	-3,8 (-9,5;2,6)
Amazonas	6097,6 (5376,6;6855,3)	809,8 (709,1;919)	19459,2 (17131;21872,1)	775,9 (678,8;884,1)	-4,2 (-10,2;1,9)
Bahia	52840,3 (46323,4;60082)	783,5 (685,2;893)	118062,7 (103361,3;134066,1)	753,3 (656,9;857,9)	-3,8 (-9,6;2,5)
Ceará	30093,8 (26385,1;34137,4)	739,5 (648;842,1)	77144,8 (67097,6;87800,2)	785,5 (682,6;896)	6,2 (-0,8;14,2)
Distrito Federal	4256,7 (3710,8;4838,9)	813,3 (701,7;932)	16100,7 (13996,8;18333,4)	753,5 (654,5;850,2)	-7,4 (-13,1;-0,6)
Espírito Santo	11320,9 (9847,7;12942,2)	841,5 (730,3;961,6)	31391,5 (27390,7;35566,7)	782,7 (680;889,7)	-7 (-13,2;-0,6)
Goiás	14142 (12371,7;16150,6)	800,7 (703,5;912,7)	46168,1 (40298,2;52244,1)	753 (655,9;854)	-6 (-12,9;1)
Maranhão	18235,7 (15857,1;20802,4)	747,2 (650,9;852,8)	49180,9 (43277;55993,9)	795,1 (697,6;907,8)	6,4 (0,2;13,5)
Mato Grosso	5774,8 (5067;6502,3)	819,3 (712,6;938)	21845,3 (19017,4;24622)	789,4 (688;895,4)	-3,7 (-9,6;3)
Mato Grosso do Sul	6795,1 (5934,8;7652)	846,4 (740,2;961,2)	21183 (18418,4;24002)	816 (710,9;922,6)	-3,6 (-9,3;3,4)
Minas Gerais	74411,2 (64608;84527,1)	826,9 (722;940,5)	187809,8 (163412,5;214570,5)	759,5 (659,4;867)	-8,1 (-14,2;-1,6)
Pará	16002,3 (14153,8;18014,5)	789,4 (694,3;893,2)	46324,1 (40809,4;52186,1)	746,6 (652,1;844,8)	-5,4 (-11,3;0,9)
Paraíba	17922,4 (15619,1;20442,3)	772 (675,4;881)	36827,2 (32186,6;41911,1)	794,6 (693,4;902,5)	2,9 (-3,6;10,2)
Paraná	35843,5 (31360,3;40661,2)	834,4 (726,6;948,5)	93386,5 (81689;106563,6)	779,7 (684;892,6)	-6,6 (-12,2;0,1)
Pernambuco	34084,1 (29826,4;39017,2)	793,9 (695;908,1)	72004 (62756,3;81953,9)	753,9 (655,5;862,9)	-5 (-11,2;1,3)
Piauí	11016,2 (9596,9;12503,6)	803,5 (698,9;912,6)	29097,2 (25425,9;33105,2)	812,7 (708,9;927)	1,1 (-5,6;8,5)
Rio de Janeiro	72976,8 (63619,5;83192)	850,4 (744;970,5)	162697,6 (140265,7;185508,6)	778,9 (674,2;887,1)	-8,4 (-14,1;-1,7)
Rio Grande do Norte	13462,3 (11756,9;15396,2)	827 (720,5;948,1)	31332,8 (27529,6;35470,6)	839 (734;955,6)	1,5 (-4,9;8,1)
Rio Grande do Sul	51590,7 (45263,1;58166,6)	862,8 (754,1;980)	115132,9 (100064,2;130860)	787,4 (685,2;894,6)	-8,7 (-14,7;-2,9)
Rondônia	2451,8 (2150,6;2766,5)	813,4 (710,9;925,4)	9980,6 (8742,3;11325)	766 (669,2;872,1)	-5,8 (-11,9;1)
Roraima	419,1 (368,5;471,8)	809,3 (706,3;925)	2297,9 (2000,3;2611)	774,6 (674,4;884,7)	-4,3 (-10,5;2)
Santa Catarina	19387,5 (16978;21842,4)	847 (741,4;957,7)	55662,9 (48893,1;63470,8)	779,6 (685,4;894,9)	-8 (-13,7;-2)
São Paulo	150009 (131202;169778,7)	842,8 (734,6;959,2)	387169,5 (336629;442688,8)	787,9 (685;899,2)	-6,5 (-12,8;-0,4)
Sergipe	6409,8 (5592,5;7302,6)	754,4 (657,2;860,7)	15587,4 (13661,6;17664,6)	763,8 (668,4;870,4)	1,2 (-4,7;8,8)
Tocantins	2884,2 (2501,8;3284,4)	789,5 (691,7;906,1)	10477,4 (9147,4;11899)	796,5 (692,4;907,9)	0,9 (-5,9;8,5)

*Fonte: Fonte: Dados derivados do estudo Global Burden of Disease 2019, Institute for Health Metrics and Evaluation, University of Washington. ^46^*

**Tabela 4-10:** – Taxas de prevalência de insuficiência cardíaca (por 100 mil) e variação percentual das taxas, por grupo etário e sexo, Brasil, 1990 e 2017.

Grupo etário e sexo	1990	2017	Variação percentual (II 95%)
Padronizada por idade	818,1 (718,1;922,8)	777,2 (680;874,8)	-5 (-7,1;-3)
Abaixo de 5	46,3 (32;63,8)	45 (30,8;61,9)	-2,9 (-5,5;-0,1)
5-14 anos	34,7 (24,2;47,2)	34,1 (23,6;46,7)	-1,6 (-4,5;1,3)
15-49 anos	107,1 (90,7;124,8)	119 (100,2;139,3)	11,1 (5,5;15,6)
50-69 anos	1391,6 (1172,1;1627,5)	1330,4 (1125,6;1570,4)	-4,4 (-6,9;-1,8)
70+ anos	8249,1 (6918,9;9752,5)	8530,2 (7265,9;9922,9)	3,4 (-1;8)
Todas as idades	448,5 (394,8;504,4)	796,1 (698,1;892,6)	77,5 (72,3;82,4)
**Masculino**			
Padronizada por idade	811,8 (714;916,9)	750,6 (656,2;845)	-7,5 (-10,2;-4,8)
Abaixo de 5	46,8 (32,2;64,5)	45,2 (31;62,1)	-3,6 (-7,2;0,4)
5-14 anos	34,3 (23,7;46,9)	33,4 (23,1;45,9)	-2,5 (-6,6;1,7)
15-49 anos	105,3 (89;122,4)	114,2 (95,6;134,7)	8,5 (0,5;14,2)
50-69 anos	1386,9 (1164,2;1643,5)	1311,3 (1102,4;1555,5)	-5,4 (-9,1;-1,5)
70+ anos	8083,9 (6784,9;9549,3)	7926,1 (6721,2;9286,2)	-2 (-6,4;2,9)
Todas as idades	415,6 (367,7;466,8)	685 (602,9;770)	64,8 (59,3;70,4)
**Feminino**			
Padronizada por idade	820,9 (721;933,2)	794,7 (694,4;900,6)	-3,2 (-6,5;-0,1)
Abaixo de 5	45,8 (31,6;63,3)	44,8 (30,7;63,3)	-2,1 (-5,7;2,2)
5-14 anos	35,1 (24,7;47,9)	34,9 (24,3;47,2)	-0,7 (-4,6;3,1)
15-49 anos	109 (91,7;126,6)	123,7 (103,9;144)	13,5 (8,8;18,3)
50-69 anos	1395,9 (1183,9;1632,2)	1347,3 (1137,1;1586,4)	-3,5 (-6,9;0,3)
70+ anos	8381,9 (7012,1;9982,4)	8968,9 (7622,9;10482,3)	7 (1,1;12,8)
Todas as idades	480,8 (422,3;544,4)	902,3 (790,2;1020,9)	87,7 (81;94,4)

*Fonte: Fonte: Dados derivados do estudo Global Burden of Disease 2017, Institute for Health Metrics and Evaluation, University of Washington. ^46^*

**Tabela 4-12:** – Taxas de YLDs por insuficiência cardíaca (por 100 mil) e variação percentual das taxas, por grupo etário e sexo, Brasil, 1990 e 2017.

Grupo etário e sexo	1990	2017	Variação percentual (II 95%)
**Ambos**			
Padronizada por idade	112,2 (82,8;141,2)	108,8 (81,4;134,5)	-3 (-6,7;0,3)
Abaixo de 5	4,5 (2,7;7)	4,3 (2,6;6,8)	-3,4 (-6;-0,6)
5-14 anos	3,2 (1,9;5,1)	3,2 (1,9;5)	-1,4 (-4,4;1,5)
15-49 anos	8,7 (5,7;12,6)	10,4 (6,8;14,8)	18,7 (12,3;25,5)
50-69 anos	165,8 (112,7;226,8)	166,4 (115,3;228,9)	0,3 (-3,8;5,5)
70+ anos	1263,1 (919,3;1599,5)	1308,3 (988,6;1586)	3,6 (-3,2;10,2)
Todas as idades	59 (42,9;75,4)	110,6 (82,3;137,5)	87,5 (78,8;96,2)
**Masculino**			
Padronizada por idade	112,9 (86,9;137,4)	105,2 (81,6;127,1)	-6,8 (-10,9;-2,6)
Abaixo de 5	4,5 (2,7;7,1)	4,3 (2,6;6,8)	-4,2 (-7,8;-0,2)
5-14 anos	3,2 (1,9;5)	3,1 (1,8;4,9)	-2,2 (-6,4;2)
15-49 anos	7,9 (5,1;11,6)	9,5 (6,1;13,8)	19,8 (10,2;29,3)
50-69 anos	165,5 (111,9;230,9)	165,8 (113,5;229,4)	0,2 (-5,9;6,8)
70+ anos	1282,5 (973,9;1555,8)	1225,7 (980;1439,6)	-4,4 (-11,1;3,4)
Todas as idades	55 (40,6;68,5)	94,5 (72,6;115,7)	71,9 (63,1;81,5)
**Feminino**			
Padronizada por idade	111,2 (79,9;145)	110,9 (80,1;140,6)	-0,3 (-4,9;4,2)
Abaixo de 5	4,4 (2,7;7)	4,3 (2,6;6,7)	-2,6 (-6,3;1,7)
5-14 anos	3,2 (1,9;5,2)	3,2 (1,9;5,1)	-0,6 (-4,5;3,2)
15-49 anos	9,5 (6,3;13,4)	11,2 (7,4;15,9)	17,9 (10,5;26,4)
50-69 anos	166,1 (112,5;225,8)	166,9 (116,6;228,9)	0,5 (-5;6,7)
70+ anos	1247,5 (876,2;1659,2)	1368,3 (992,5;1714,5)	9,7 (0,6;17,6)
Todas as idades	62,9 (44,1;82,5)	125,9 (90,9;159,5)	100,2 (88,8;111,5)

*Fonte: Fonte: Dados derivados do estudo Global Burden of Disease 2017, Institute for Health Metrics and Evaluation, University of Washington. ^46^*

**Figura 4-1:**
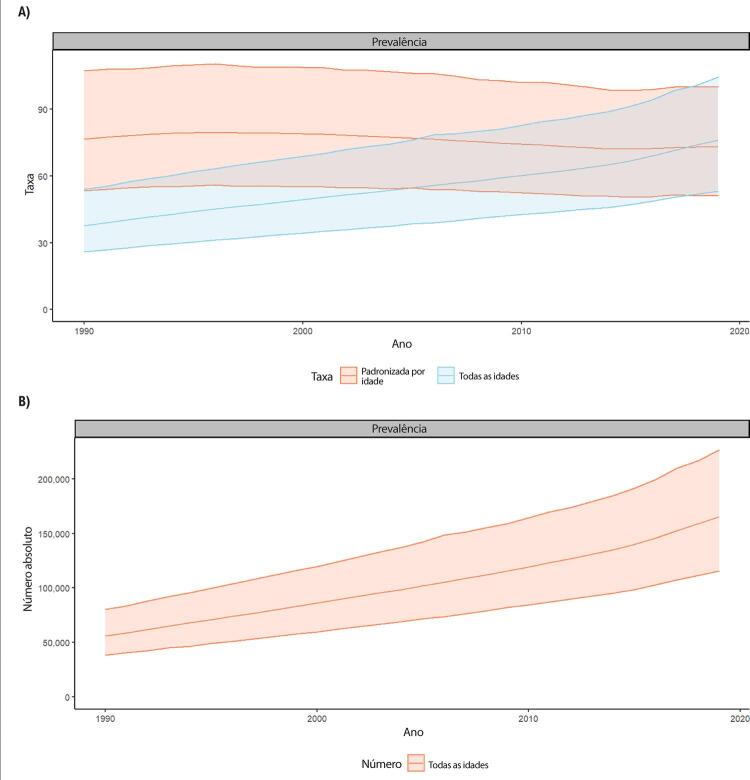
-Taxas de prevalência de cardiomiopatia e miocardite padronizadas por idade (A) e taxas de prevalência brutas de cardiomiopatia e miocardite (B), por 100 mil habitantes, Brasil, 1990-2019.

Fonte: Dados derivados do estudo Global Burden of Disease 2019, Institute for Health Metrics and Evaluation, University of Washington. ^46^

**Figura 4-2:**
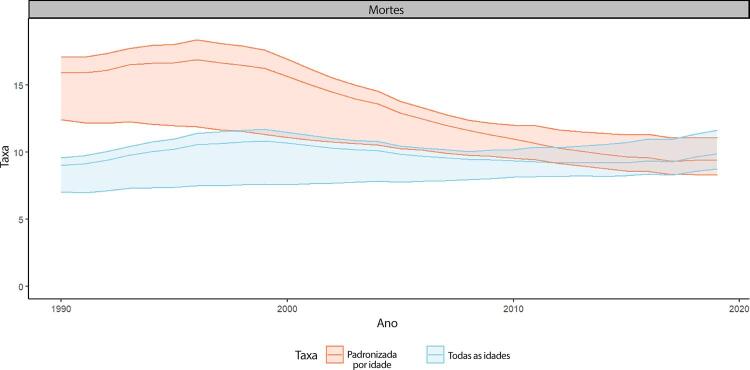
Taxas de mortalidade por cardiomiopatia e miocardite padronizadas por idade e para todas as idades, por 100 mil habitantes, Brasil, 1990-2019.

Fonte: Dados derivados do estudo Global Burden of Disease 2019, Institute for Health Metrics and Evaluation, University of Washington. ^46^

**Figura 4-3:**
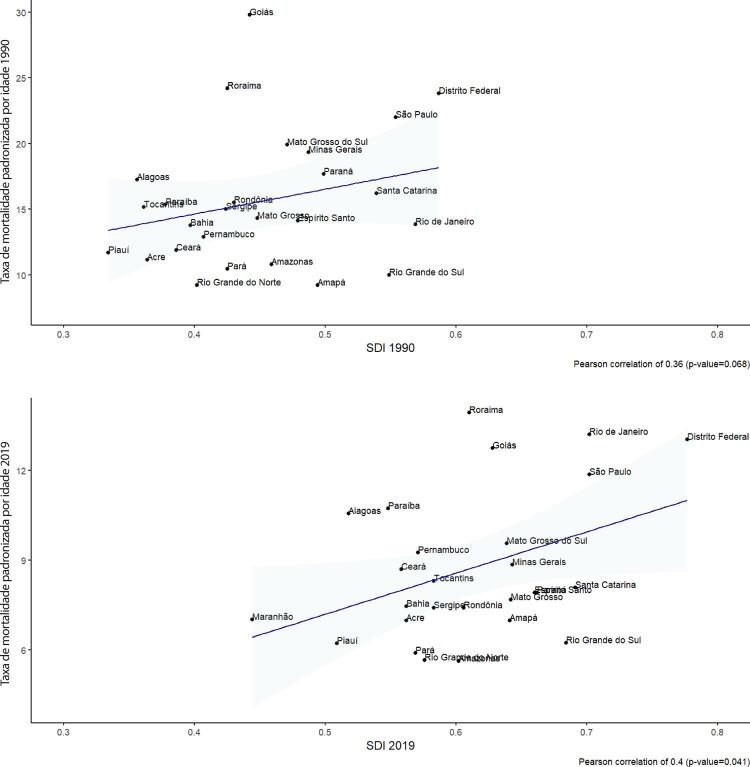
-Correlação entre taxa de mortalidade por cardiomiopatia e miocardite padronizada por idade, por 100 mil habitantes, e o índice sociodemográfico (SDI) de 1990 e 2019.

Fonte: Dados derivados do estudo Global Burden of Disease 2019, Institute for Health Metrics and Evaluation, University of Washington. ^46^

**Figura 4-4:**
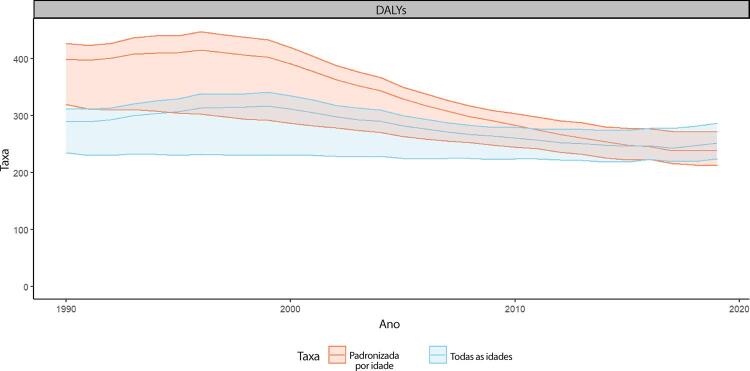
-Taxas de DALYs atribuídos a cardiomiopatia e miocardite padronizadas por idade e para todas as idades, por 100 mil habitantes, no Brasil de 1990 a 2017.

Fonte: Dados derivados do estudo Global Burden of Disease 2017, Institute for Health Metrics and Evaluation, University of Washington. ^152^

**Figura 4-5:**
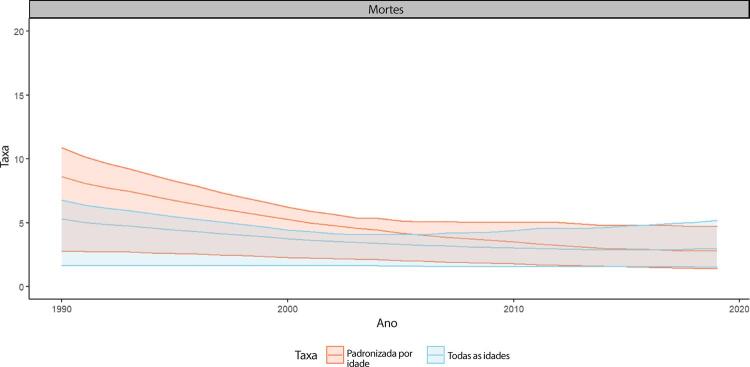
-Taxas de mortalidade por doença de Chagas padronizadas por idade e para todas as idades, por 100 mil habitantes, Brasil, 1990-2019.

Fonte: Dados derivados do estudo Global Burden of Disease 2019, Institute for Health Metrics and Evaluation, University of Washington. ^46^

**Figura 4-6:**
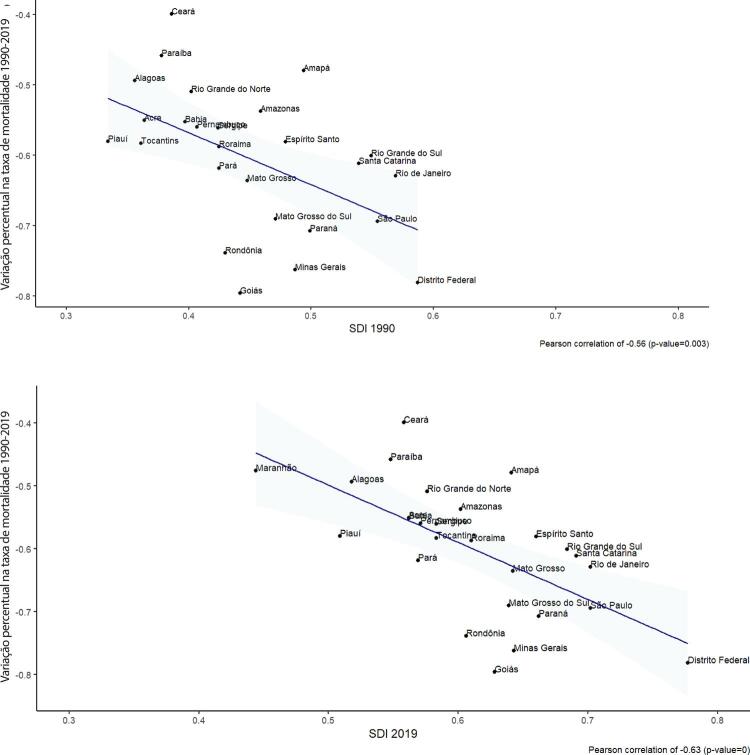
-Correlação entre a variação percentual das taxas de mortalidade por doença de Chagas padronizadas por idade, por 100 mil habitantes, de 1990 a 2019, e o índice sociodemográfico (SDI) em 1990 e em 2019.

Fonte: Dados derivados do estudo Global Burden of Disease 2019, Institute for Health Metrics and Evaluation, University of Washington. ^46^

**Figura 4-7:**
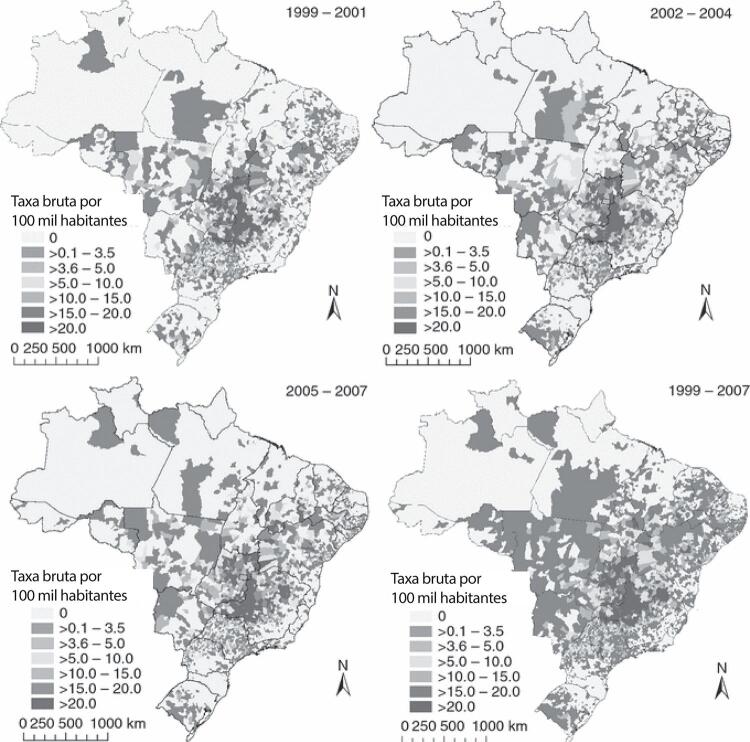
-Distribuição espacial das taxas médias de mortalidade relacionada à doença de Chagas, por 100 mil habitantes, com base em múltiplas causas de morte, por municipalidade, Brasil, 1999–2007.

Fonte: Martins-Melo et al. ^141^

**Figura 4-8:**
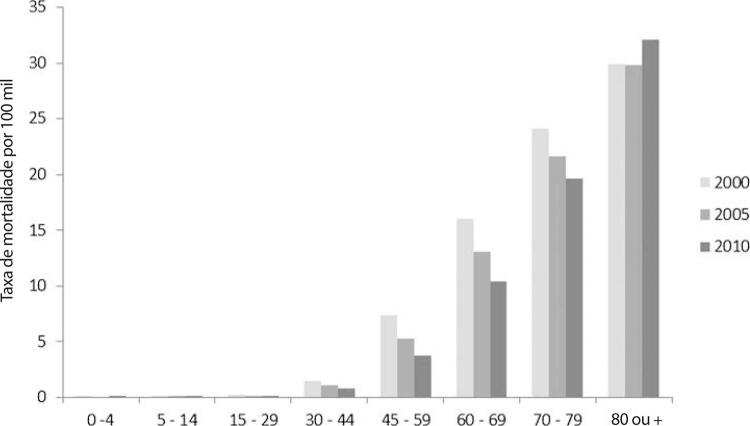
-Taxas padronizadas de mortalidade por doença de Chagas no Brasil de acordo com o grupo etário (em anos) e o ano de ocorrência, de 2000 a 2010.

Fonte: Nóbrega et al. ^142^

**Figura 4-9:**
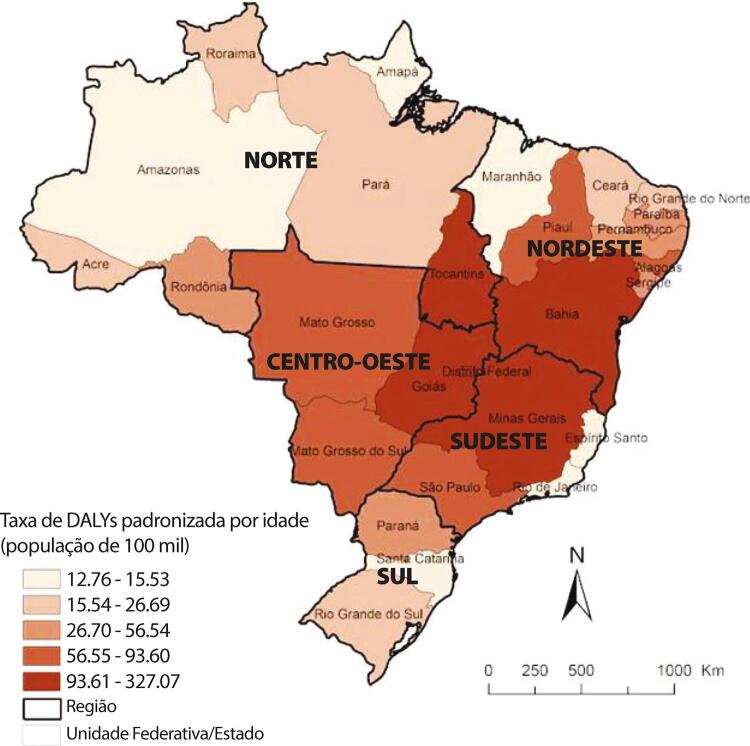
-Taxas de DALYs por doença de Chagas padronizadas por idade, por 100 mil habitantes, em 2016.

Fonte: Martins-Melo et al. ^144^

**Figura 4-10:**
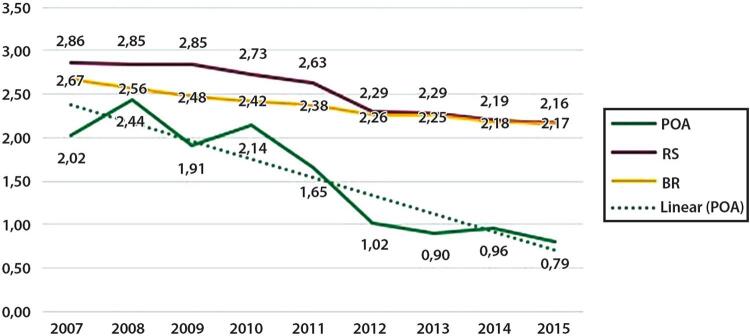
-Tendências da mortalidade por insuficiência cardíaca de 2007 a 2016 no Brasil (BR), no Rio Grande do Sul (RS) e em Porto Alegre (POA).

Fonte: Nicolao et al. ^169^

### Cardiomiopatia e Miocardite

#### Prevalência e Incidência

•De acordo com as estimativas do Estudo GBD 2019, a prevalência padronizada por idade de cardiomiopatia e miocardite diminuiu no Brasil, passando de 76,6 (II 95%, 53,4-107,2) em 1990 para 73,0 (II 95%, 51,1-100,1) em 2019, uma redução de 4,7% (II 95%, - 9,5 a 0,8) no período ( Figura 4-1 .A e Tabela 4-1). Em números absolutos, as estimativas de prevalência de cardiomiopatia e miocardite no Brasil passaram de menos de 60 mil em 1990 para mais de 160 mil em 2019, principalmente devido ao crescimento e envelhecimento da população ( Figura 4-1 .B). A prevalência de cardiomiopatia e miocardite foi maior nos homens (98,9; II 95%, 69,5-137,2) do que nas mulheres (54,1; II 95%, 38,4-73,8) em 2019, embora tenha havido uma diminuição da prevalência de 5% (II 95%, -11,6 - 0) para mulheres e de 2,9% (II 95%, -9,1 - 0) para homens no período. ^46^

•De acordo com as estimativas do Estudo GBD 2019, a prevalência de cardiomiopatia e miocardite varia bastante entre as UF brasileiras, não tendo a variação percentual sido homogênea entre 1990 e 2019 (Tabela 4-1). Em 2019, as maiores taxas foram observadas nos estados de São Paulo, Paraíba e Roraima. De 1990 a 2019, houve aumento das taxas de prevalência padronizadas por idade em Goiás e no Maranhão, e diminuição nas demais UF. ^46^

•Segundo as estimativas do Estudo GBD 2019, as taxas de incidência padronizadas por idade por 100 mil por ano foram 15,8 (II 95%, 12,7-19,2) em 1990 e 15,8 (II 95%, 12,7-19,2) em 2019, com uma pequena variação de -0,2% (II 95%, -0,3 a -0,2) no período (Tabela 4-2). Os números absolutos de casos incidentes foram 18.583 (II 95%, 14.825-22.718) em 1990 e 35.863 (II 95%, 28.946-43.756) em 2019. Esse aumento acha-se relacionado ao crescimento e envelhecimento da população. A Tabela 4-3 mostra as taxas de incidência de cardiomiopatia e miocardite por 100 mil habitantes, por idade, para ambos os sexos, em 1990 e 2019, e a variação percentual das taxas. Em 1990 e 2019, as mais altas taxas foram observadas no grupo ‘70+ anos’. De 1990 a 2019, a incidência aumentou nos grupos etários de ‘15-49 anos’ e ‘70+ anos’ para mulheres e homens. ^46^

#### Mortalidade

•De acordo com as estimativas do Estudo GBD 2019, as taxas de mortalidade por cardiomiopatia e miocardite pareceram aumentar na década de 1990, mas diminuíram nas duas décadas seguintes ( Figura 4-2 ). Como mostra a Tabela 4-4, as taxas de mortalidade foram 15,9 (II 95%, 12,4-17,1) em 1990 e 9,4 (II 95%, 8,3-11,1) em 2019 por 100 mil habitantes, uma redução de 40,8% (II 95%, -46,6 a -25,4). Apesar dessa diminuição nas taxas de mortalidade, o número de mortes por cardiomiopatia e miocardite aumentou naquele período devido ao crescimento e envelhecimento da população. Cardiomiopatia e miocardite foram responsáveis por 13.408 (II 95%, 8417-10.163) mortes em 1990, número que se elevou para 21.425 (II 95%, 17.885-21.745) mortes em 2019. As estimativas do Estudo GBD 2019 das taxas de mortalidade por cardiomiopatia referem-se a casos com cardiomiopatia listada como causa básica de morte. As mortes por insuficiência cardíaca que resulta de outras causas específicas são atribuídas à doença de base, i.e., mortes relacionadas a cardiomiopatia isquêmica são codificadas como devidas a doença isquêmica do coração. Além disso, para o projeto GBD, a insuficiência cardíaca não é considerada uma causa de morte primária e, portanto, todas as mortes codificadas como relacionadas a insuficiência cardíaca são recodificadas para a condição de base (ver adiante). ^46^

•A Tabela 4-4 também mostra o número total de mortes e as taxas de mortalidade padronizadas por idade (por 100 mil habitantes) por cardiomiopatia e miocardite, além da variação percentual das taxas, por UF e no Brasil, em 1990 e 2019. Todas as UF apresentaram diminuição das taxas de mortalidade, tendo as maiores reduções percentuais sido observadas entre 1990 e 2019 em Goiás e no Paraná. Em 2019, as UF com as mais baixas taxas de mortalidade (abaixo de 6,0) foram Amazonas, Rio Grande do Norte e Pará.

•A Tabela 4-5 mostra as taxas de mortalidade por cardiomiopatia e miocardite de acordo com o sexo e os grupos etários, com base nas estimativas do Estudo GBD 2019. As mulheres apresentaram as mais baixas taxas de mortalidade padronizadas por idade, assim como uma redução mais pronunciada de 1990 a 2019. As taxas de mortalidade por cardiomiopatia e miocardite em mulheres foram 13,5 (II 95%, 9,9-14,7) em 1990 e 7,2 (II 95%, 6,2 - 9) em 2019, uma redução de 46,6% (II 95%, -54,8 a -0,2). As taxas nos homens foram 18,7 (II 95%, 13,2 - 20,4) em 1990 e 12,1 (II 95%, 10 - 15,1) em 2019, uma variação de -35,4% (II 95%, -43,3 a -0,1). Como esperado, as mais altas taxas de mortalidade foram observadas no grupo de 70+ anos, sendo, em 1990, 127,2 (II 95%, 96,5-138,3) e, em 2019, 84,6 (II 95%, 72,1-104) por 100 mil. Para o grupo de 50-69 anos, as taxas foram 29,2 (II 95%, 22 – 31,4) em 1990 e 17,2 (II 95%, 15,1 - 20) em 2019 por 100 mil. No geral, as taxas de mortalidade diminuíram de 1990 a 2019 em todos os grupos etários. ^46^

•O Estudo GBD 2019 usa o SDI como uma estimativa do nível socioeconômico de uma localidade. A Figura 4-3 mostra a correlação entre o SDI e a taxa de mortalidade padronizada por idade por cardiomiopatia e miocardite, por 100 mil habitantes, em 1990 e e 2019. Não houve correlação entre a taxa de mortalidade padronizada por idade e o SDI em 1990 (R = - 0,36, p=0,068). Entretanto, houve significativa correlação entre a taxa de mortalidade padronizada por idade e o SDI em 2019 (R = 0,4, p=0,041). ^90^

•Em estudo relatando dados da Fundação SEADE, do estado de São Paulo, as cardiomiopatias foram responsáveis por um total de 3.571 óbitos, correspondendo a 23,3% das mortes relacionadas a insuficiência cardíaca em 2006, a saber: cardiomiopatia dilatada, responsável por 17,2% das mortes; cardiomiopatia alcoólica, por 0,45%; e cardiomiopatias restritivas, por 0,37%. A CMCh e a cardiomiopatia alcoólica foram responsáveis por 7,8% e 0,45% das mortes relacionadas a insuficiência cardíaca, respectivamente. ^135^

•Dados sobre cardiomiopatias específicas são escassos. Em estudo de coorte de 214 pacientes com CMH, acompanhados por 7 anos em um hospital terciário de São Paulo, a idade média foi 37±16 anos, sendo 52% mulheres. Houve 22 mortes (10%), 15 diretamente relacionadas à CMH (11 mortes súbitas). As taxas de sobrevida acumulada foram 94,5% em 5 anos, 91% em 10 anos e 87,9% em 15 anos, com taxa de mortalidade anual de 1%, que é baixa, considerando que o estudo foi realizado em um centro de referência. ^136^

#### Carga de Doença

•De acordo com as estimativas do GBD 2019, as tendências das taxas de DALYs padronizadas por idade por cardiomiopatia e miocardite foram similares àquelas de mortalidade, com pequeno aumento na década de 1990 e diminuição nas décadas seguintes. Como mostra a Tabela 4-6, as taxas de DALYs padronizadas por idade foram 399,4 (II 95%, 319,6-426,4) em 1990 e 238,6 (II 95%, 213-272,3) em 2019, por 100 mil habitantes, uma diminuição de 40,3% (II 95%, -45,8 a -27,9). Tais mudanças são similares àquelas observadas nas taxas de mortalidade. A despeito dessa diminuição nas taxas de DALYs, cardiomiopatia e miocardite foram responsáveis por 431.381 (II 95%, 349.546-465.068) DALYs no Brasil em 1990 e por 545.772 (II 95%, 484.988-621.356) em 2019, o que representa 0,62% de todos os DALYs. ^46^

•A Tabela 4-7 mostra as taxas de DALYs por cardiomiopatia e miocardite de acordo com sexo e grupo etário, a partir das estimativas do Estudo GBD 2019. As taxas de DALYs padronizadas por idade foram menores nas mulheres, que também apresentaram a redução mais pronunciada de 1990 a 2017. As taxas de DALYs para mulheres foram 321,1 (II 95%, 237,7-346,2) em 1990 e 165,7 (II 95%, 147,3 - 198) em 2019, uma redução de 48,4% (II 95%, -55 a – 0,2). As taxas de DALYs para homens foram 484,8 (II 95%, 353,6 – 528,5) em 1990 e 320,4 (II 95%, 257,7 – 371,8) em 2019, uma redução de 33,9% (II 95%, -41,8 a -0,1). Como esperado, as mais altas taxas de DALYs foram observadas no grupo de 70+ anos, seguido pelo grupo de 50-69 anos. No geral, as taxas de DALYs diminuíram de 1990 a 2019 em todos os grupos etários (Tabela 4-4). ^46^

## Doença de Chagas Crônica e Cardiomiopatia Chagásica

### Prevalência e Incidência

•A prevalência de DCh no Brasil em 2010 foi estimada em 1.156.821 pela Organização Mundial da Saúde, ^137^ sendo essa a última estimativa oficial disponível, publicada em 2015. De acordo com tal estimativa, o número de indivíduos com CMCh no Brasil era 231.364. ^137^ Esses números revelam uma tendência significativa de diminuição de casos humanos de DCh no Brasil em relação a estimativas anteriores, sendo isso atribuído a vários fatores, mas principalmente à quase completa interrupção da transmissão vetorial e transfusional no Brasil.

•De acordo com as estimativas do Estudo GBD 2019, a prevalência padronizada por idade de DCh diminuiu significativamente no Brasil, 37,7% (II 95%, -40,2 a -35,2) de 1990 a 2019, passando de 1.463 (II 95%, 1.240-1.711) em 1990 para 912 (II 95%, 788-1.048) em 2019 por 100 mil habitantes. A prevalência de DCh no Brasil em 2019 foi maior entre os homens [987 (II 95%, 856,4 – 1.141,3)] do que entre as mulheres [841 (II 95%, 723,2-962)]. ^46^

•Em uma revisão sistemática de estudos de base populacional sobre a prevalência de DCh no Brasil realizados de 1980 a setembro de 2012, 42 artigos com dados relevantes de prevalência foram identificados a partir de um total de 4.985 referências. ^138^ A estimativa combinada de prevalência de DCh a partir dos estudos para todo o período foi 4,2% (IC 95%, 3,1-5,7), variando de 4,4% (IC 95%, 2,3-8,3) na década de 1980 a 2,4% (IC 95%, 1,5-3,8) após 2000. A prevalência estimada de DCh para homens e mulheres foi similar (4,1% [IC 95%, 2,6-6,6], 4,2% [IC 95%, 2,6-6,8], respectivamente). A maior estimativa combinada de prevalência foi observada em indivíduos com idade >60 anos (17,7%; IC 95%, 11,4-26,5), nas regiões Nordeste (5,0%; IC 95%, 3,1-8,1) e Sudeste (5,0%; IC 95%, 2,4-9,9) e em áreas mistas (urbana/rural) (6,4%; IC 95%, 4,2-9,4). Estima-se que cerca de 4,6 milhões (IC 95%, 2,9-7,2 milhões) de pessoas tenham sido infectadas pelo *T. cruzi* em 2010. Essas estimativas são bem maiores do que as da Organização Mundial da Saúde para 2010. ^137^

Os autores observaram grande heterogeneidade na maioria das estimativas combinadas (I(2)>75%; p<0,001).

•No estudo de coorte retrospectivo sobre Chagas dos *National Institutes of Health* , REDS-II, doadores de sangue inicialmente saudáveis com uma doação-índice soropositiva para *T. cruzi* pareados por idade, sexo e período com doadores soronegativos foram acompanhados por 10 anos. ^139^

A incidência diferencial de cardiomiopatia atribuível à infecção por *T. cruzi* foi 1,85 por 100 pessoas-ano. ^140^

### Mortalidade

•De acordo com o Estudo GBD 2019, o número de mortes por DCh no Brasil diminuiu nas últimas décadas ( Figura 4-5 ). Na década de 1990, a DCh foi responsável por 7903 (II 95%, 2438-10.073) mortes, que diminuíram para 6523 (II 95%, 3350-11.226) em 2019. A taxa de mortalidade padronizada por idade apresentou redução mais marcante (variação de -67,5%), passando de 8,6 (II 95%, 2,8-10,9) mortes por 100 mil habitantes em 1990 para 2,8 (II 95%, 1,8-4,8) por 100 mil habitantes em 2019, correspondendo a 1,6% de todas as mortes cardiovasculares no país. Em 2019, os homens apresentaram maiores taxas de mortalidade padronizadas por idade do que as mulheres (3,5, II 95%, 1,4-6,2; e 2,2, II 95%, 0,9-4,3, respectivamente). ^46^

•A Tabela 4-8 apresenta o número total de mortes por DCh, as taxas de mortalidade padronizadas por idade (por 100 mil habitantes), para ambos os sexos, além da variação percentual das taxas, por UF e no Brasil, em 1990 e 2019. O número de mortes e as taxas de mortalidade variaram significativamente entre as UF nos dois anos. Em 1990, taxas de mortalidade mais altas (> 10 por 100 mil habitantes) foram observadas nas UF centrais brasileiras (Goiás, Distrito Federal, Minas Gerais e Bahia), com pico em Goiás (52,3 por 100 mil, II 95%, 3,3-82,4). Todas as UF apresentaram redução nas taxas de mortalidade, que variaram de 39,9% (II 95%, 67,7 a 15,5) no Ceará a 79,6% (II 95%, -84,3 a -5,8) em Goiás.

•A mortalidade estimada em crianças com menos de 5 anos é praticamente zero. Para os outros grupos etários, a redução nas taxas de mortalidade foi mais acentuada (variação de 76,2%, UI -86,5 a 29,5) no grupo etário de 5-49 anos, passando de 2,6 (UI 0,6-3,5) a 0,6 (UI 0,4-1,3) por 100 mil habitantes. A maioria das mortes ocorreu em indivíduos com 70+ anos, que apresentaram a menor redução percentual (-54,7%, UI -65-6,3) no período de 1990-2019: de 53 (UI 19,2-66,3) a 24 (10,4-36,1) por 100 mil habitantes. A diminuição na taxa de mortalidade padronizada por idade por 100 mil habitantes correlaciona-se com o SDI das UF brasileiras em 1990 (R = -0,56, p=0,003) e em 2019 (R = -0,63, p<0,001) ( Figura 4-6 ).

•Vários estudos de base populacional mostraram uma redução na mortalidade por DCh no Brasil nas últimas décadas. Martins-Melo *et al* . ^141^ relataram uma redução gradual das taxas de mortalidade padronizadas em todo o país, de 3,78 (1999) para 2,78 (2007) mortes/ano por 100 mil habitantes (-26,4%). Nobrega *et al* . ^142^ mostraram que as taxas de mortalidade padronizadas diminuíram em todo o país em 32,4%, passando de 3,4% em 2000 para 2,3% em 2010. A taxa de mortalidade por envolvimento cardíaco diminuiu em todas as regiões do Brasil, exceto na região Norte, onde aumentou em 1,6%. A região Nordeste apresentou a menor redução, enquanto a região Centro-Oeste, a maior. Simões *et al* . ^143^ estudando a evolução da mortalidade por DCh no Brasil de 1980 a 2014, fizeram uma previsão para a mortalidade de 2015 a 2034. Esses autores estimaram um declínio progressivo na mortalidade por DCh, que seria maior entre os jovens.

A redução média esperada foi de 76,1% em comparação ao último período observado (2010-2014) e ao último período previsto (2030-2034). As regiões Centro-Oeste, Sudeste e Sul apresentaram uma redução na taxa de mortes por DCh entre 2000 e 2014. A taxa de mortalidade na região Nordeste não diferiu estatisticamente em nenhum período analisado, mas, na região Norte, apresentou tendência a aumento.

•Tendo por base o SIM, cuja abrangência é nacional, um estudo analisou todas as declarações de óbito emitidas entre 1999 e 2007 no Brasil ^141^ e concluiu que a DCh foi mencionada em 53.930 (0,6%) declarações de óbito: como causa básica em 44.537 (82,6%) declarações de óbito, e como uma causa de morte associada em 9.387 (17,4%) declarações de óbito. A DCh aguda foi responsável por 2,8% das mortes. A taxa de mortalidade padronizada média foi 3,36 por 100 mil habitantes/ano, que é 21% mais alta do que a taxa de mortalidade quando se considera apenas a causa básica de morte (2,78 mortes por 100 mil habitantes/ano).

A mortalidade proporcional, considerando-se múltiplas causas de morte foi 0,6%. Os indivíduos que morreram por DCh foram predominantemente do sexo masculino (57%), com idade superior a 60 anos (62,8%) e residentes do Sudeste (53,6%). O Centro-Oeste apresentou a maior mortalidade proporcional de todas as regiões (2,17%). ^141^

•Na mesma base de dados, calculando a taxa de mortalidade média para cada município de residência e usando *Empirical Bayesian Smoothing* , uma análise espacial identificou um grande *cluster* com alto risco de mortalidade por DCh, envolvendo nove estados na região central do Brasil ( Figura 4-7 ). ^144^

•Nobrega *et al* ., em estudo descritivo usando dados do SIM sobre todas as mortes por DCh no Brasil entre 2000 e 2010, observaram que, nesse período, a maioria (85,9%) ocorreu em homens com mais de 60 anos, tendo sido causadas por comprometimento cardíaco. No período estudado, a taxa de mortalidade diminuiu em todas as faixas etárias, exceto naquela a partir de 80 anos ( Figura 4-8 ). ^142^

•Um estudo de coorte retrospectivo utilizou ligação probabilística para identificar entre doadores de sangue de 1996 a 2000 (2.842 soropositivos e 5.684 soronegativos para DCh) aqueles que morreram até 2010. ^145^ Os autores identificaram 159 mortes entre os doadores soropositivos (5,6%) e 103 mortes entre os soronegativos (1,8%).

Os doadores soropositivos apresentaram um risco 2,3 vezes maior de morte por todas as causas (IC 95%, 1,8-3,0) em comparação aos soronegativos. Entre os doadores soropositivos, apenas 26 tiveram, como causa básica de morte, o código da CID-10 indicativo de DCh (B57.0/B57.5). ^145^ Os autores concluíram que a DCh é uma causa de morte subnotificada na base de dados brasileira de mortalidade.

•Ayub-Ferreira *et al* . compararam o mecanismo de morte na insuficiência cardíaca por CMCh com aquele de outras etiologias em um ensaio clínico prospectivo, o REMADHE, que incluiu pacientes a partir de 18 anos, com insuficiência cardíaca crônica irreversível por pelo menos 6 meses e fração de ejeção ventricular esquerda inferior a 50%. Dos 342 pacientes analisados, 185 (54,1%) morreram, sendo que desses, 56,4% eram portadores de DCh e 53,7% não. De todas as mortes no grupo com DCh, 48,4% foram relacionadas a piora da insuficiência cardíaca, 25,7% a morte súbita e 6,4% a acidente vascular cerebral. A incidência acumulada de mortalidade por todas as causas e mortalidade por insuficiência cardíaca foi significativamente maior em pacientes com DCh do que naqueles sem DCh. ^146^ Não houve diferença na incidência acumulada de mortalidade por morte súbita entre os dois grupos. Na cardiopatia chagásica grave, insuficiência cardíaca progressiva é o principal mecanismo de morte.

•Na Coorte de Idosos de Bambuí, um grande estudo de base populacional de idosos residentes em uma área endêmica para DCh, 1.479 indivíduos com idade igual ou superior a 60 anos (38,1% com teste positivo para *T. cruzi* ) foram acompanhados de 1997 a 2007. Durante o acompanhamento médio de 8,72 anos, 567 participantes morreram. A infecção por *T. cruzi* foi um preditor de mortalidade entre os membros da coorte e essa associação permaneceu altamente significativa após ajustes para idade, sexo e fatores de risco cardiovascular convencionais (HR = 1,56; IC 95%, 1,32-1,85). No geral, o RAP de mortalidade por infecção por *T* . *cruzi* foi 13,2% (IC 95%, 9,8-16,4). ^147^

•Nadruz *et al* . estudaram as tendências temporais no RAP de CMCh para mortalidade em 2 anos entre pacientes com insuficiência cardíaca arrolados nos períodos 2002-2004 (era 1) e 2012-2014 (era 2) em um hospital universitário brasileiro. Foram estudados prospectivamente 362 (15% com CMCh) e 582 (18% com CMCh) pacientes com insuficiência cardíaca e fração de ejeção ≤ 50% nos períodos 1 e 2, respectivamente, tendo-se estimado o RAP de CMCh para mortalidade em 2 anos. Embora os números absolutos de morte tenham diminuído com o tempo nos grupos de CMCh e CMNCh, o RAP de CMCh para mortalidade aumentou entre os pacientes com insuficiência cardíaca [RAP _(era 1)_ = 11,0 (IC 95%: 2,8-18,5%); RAP _(era 2)_ = 21,9 (IC 95%: 16,5-26,9); p=0.023 *vs.* era 1], devido a aumento no HR associado com DCh. ^148^

•Em um estudo de coorte, 298 pacientes com DCh foram acompanhados de março de 1995 a setembro de 2019 no município de Virgem da Lapa, em Minas Gerais. No período de 24 anos estudado, 113 (37,9%) mortes foram registradas na coorte, 107 (35,9%) das quais foram atribuídas a doença do coração: apenas 10 (11,6%) ocorreram em 86 pacientes sem cardiomiopatia, 49 (31,4%) em 156 pacientes com cardiomiopatia e 48 (85,7%) em 56 pacientes com aneurisma de ventrículo esquerdo. O risco de morte foi significativamente maior (7,4 vezes) em pacientes com aneurisma de ventrículo esquerdo. ^149^

•Em um estudo de coorte que acompanhou por dois anos 1.637 pacientes com DCh vivendo em 21 municípios onde a DCh é endêmica, 205 (12,5%) deles apresentaram novos eventos cardiovasculares, 134 dos quais (8,2%) morreram, 28 (1,7%) desenvolveram fibrilação atrial e 43 (2,6%) necessitaram de implantação de marca-passo. Os residentes dos municípios com maior população rural apresentaram proteção contra eventos cardiovasculares (OR = 0,5; IC 95%, 0,4-0,7), enquanto os moradores dos municípios com menor número de médicos por 1.000 habitantes (OR = 1,6; IC 95%, 1,2-2,5) e aqueles residindo em municípios com menor cobertura da atenção primária à saúde (OR = 1,4; IC 95%, 1,1-2,1) apresentaram maiores chances de experienciar eventos cardiovasculares. ^150^

•Em um estudo de coorte incluindo 1.551 pacientes com CMCh em Minas Gerais, foi desenvolvido um escore preditivo de mortalidade em 2 anos. O escore incluiu variáveis simples, como idade, classe funcional da *New York Heart Association* , frequência cardíaca, duração do QRS e níveis anormais de NT-proBNP ajustado para idade. As taxas de mortalidade observadas nos grupos de risco baixo, médio e alto foram 0%, 3,6% e 32,7%, respectivamente, na coorte de derivação e 3,2%, 8,7% e 19,1%, respectivamente, na coorte de validação, com estatística C de 0,82 e 0,71, respectivamente. Esse parece ser um escore útil e simples para ser usado em áreas remotas com limitados recursos tecnológicos. ^151^

### Carga de Doença

•De acordo com o Estudo GBD 2019, foram estimados 174.194 DALYs (II 95%, 109.039-302.974) devidos a DCh no Brasil, com uma redução relativa de 32,1% em comparação ao valor de 1990 (256.380 DALYs, II 95%, 81.697-328.720). As taxas de DALYs padronizadas por idade diminuíram em nível nacional (-70,5%) e em todas as UF brasileiras entre 1990 e 2019, mas com diferentes padrões regionais ( Figura 4-9 ). A diminuição nas taxas de DALYs foi primariamente devida a uma redução consistente nas taxas de YLLs, o principal componente do total de DALYs por DCh. A maior carga fatal e não fatal por DCh foi observada entre os homens e os idosos e nas UF brasileiras com importantes áreas endêmicas de transmissão vetorial no passado, como Goiás, Tocantins, Minas Gerais, Bahia e Distrito Federal. ^46^

## Insuficiência cardíaca

•Como a insuficiência cardíaca não é considerada uma causa básica de morte (i.e., código *garbage* ) no Estudo GBD, todas as mortes atribuídas a insuficiência cardíaca nas declarações de óbito são reclassificadas e/ou redistribuídas para outras causas, de acordo com o método do GBD. Assim, não há dados do GBD sobre mortalidade por insuficiência cardíaca. A insuficiência cardíaca é classificada pelo GBD como um “comprometimento”, portanto, os únicos indicadores do GBD para insuficiência cardíaca são prevalência e YLDs, que é o componente de morbidade do DALYs.

### Prevalência e Incidência

•De acordo com as estimativas do Estudo GBD 2017, a prevalência padronizada por idade de insuficiência cardíaca no Brasil passou de 818 (II 95%, 718-923) em 1990 para 772 (II 95%, 680-875) em 2017, uma diminuição de 5% (95 UI, -7,1 a -3) no período (Tabela 4-9). Em números absolutos, as estimativas de prevalência de insuficiência cardíaca no Brasil subiram de 0,67 milhão em 1990 para quase 1,7 milhão em 2017, principalmente devido a crescimento e envelhecimento da população. A prevalência de insuficiência cardíaca variou entre as UF brasileiras e a variação percentual não foi uniforme entre 1990 e 2017 (Tabela 4-9). Em 2017, as mais altas taxas foram observadas no Rio Grande do Norte e as mais baixas, no Acre. De 1990 a 2017, taxas de prevalência padronizadas por idade decrescentes foram observadas na maioria das UF, tendo aumento nas taxas ocorrido em 8 UF, principalmente na região Nordeste. ^152^

•A Tabela 4-10 mostra a prevalência de insuficiência cardíaca de acordo com sexo e grupo etário, a partir das estimativas do Estudo GBD 2017. A prevalência de insuficiência cardíaca foi maior em mulheres (795; II 95%, 694-901) do que em homens (751; II 95%, 656-845) em 2017, e a redução na prevalência de 1990 a 2017 foi mais pronunciada nos homens, sendo a porcentagem de diminuição 7,5 (II 95%, -10,2 a -4,8) para homens e 3,2 (II 95%, -6,5 a -0,1) para mulheres. Quanto aos grupos etários, as taxas de incidência aumentaram 10 vezes do grupo de 15-49 anos ao de 50-69 anos, e 6 vezes do último grupo ao de 70+ anos, tendo esses aumentos sido similares para mulheres e homens. De 1990 a 2017, a prevalência aumentou apenas no grupo de 15-49 anos, enquanto diminuiu nos demais, provavelmente em associação com a elevação de eventos isquêmicos naquele grupo etário. ^152^

•Uma revisão sistemática, avaliando a carga de insuficiência cardíaca na América Latina, incluiu 143 artigos publicados entre janeiro de 1994 e junho de 2014, com pelo menos 50 participantes com idade ≥ 18 anos; a maioria dos estudos incluídos (64%) foi do Brasil. ^153^ A idade média dos pacientes foi 60±9 anos, a fração de ejeção média, 36±9%, e a prevalência de insuficiência cardíaca, 1% (IC 95%, 0,1-2,7). Dos estudos incluídos, apenas um avaliou incidência, com 1.091 indivíduos identificados através de amostragem probabilística em múltiplas etapas na cidade de Porto Alegre. A idade média foi 42,8±16,9 anos e 55% eram mulheres. A incidência de insuficiência cardíaca em um estudo com apenas uma população fornecendo essa informação foi de 199 casos por 100 mil pessoas-ano. ^154^

•Em estudo de base populacional em atenção primária de uma cidade brasileira de tamanho médio, 633 indivíduos com idade ≥45 anos foram selecionados aleatoriamente e registrados em um programa de atenção primária.

A idade média foi 59,6±10,4 anos e 62% eram mulheres.

A prevalência de insuficiência cardíaca sintomática (estágio C) foi 9,3% e a de insuficiência cardíaca estágio B (anormalidades estruturais) foi 42,7%. Dos pacientes com insuficiência cardíaca, 59% apresentavam fração de ejeção preservada e 41% apresentavam fração de ejeção reduzida. ^155^

•Um estudo baseado na Pesquisa Nacional de Saúde de 2013, com dados de 59.655 adultos (≥ 18 anos), encontrou prevalência autorrelatada de insuficiência cardíaca de 1,1%, representando cerca de 1,7 milhão de indivíduos. Naqueles acima de 60 anos, a prevalência foi de 3,3%. ^156^

•Outro estudo de base populacional com residentes da Zona da Mata, Minas Gerais, envolveu 7.113 idosos frágeis. A idade média foi 72,4 ± 8,0 anos, 67,6% eram mulheres e a prevalência de insuficiência cardíaca foi de 7,9%. ^157^

•Em estudo que incluiu 166 pacientes da área rural de Valença, Rio de Janeiro, a idade média foi 61± *14 anos e 51% eram homens. As principais etiologias foram hipertensiva e isquêmica, sendo 51% portadores de* insuficiência cardíaca com fração de ejeção reduzida, *com características similares àquelas de coortes de centros terciários não rurais* . ^158^

### Mortalidade

•Em estudo avaliando dados do SIM de 2008 a 2012, insuficiência cardíaca foi um código *garbage* usado com frequência no Brasil. Foi listada como causa básica de morte em 123.268 (3,7%) daqueles registros e como causa múltipla de morte em 233.197 (7%). Utilizando 2 métodos de redistribuição para causas específicas de morte, apenas 38,7-44,8% puderam ser reclassificadas para uma causa definida de morte com o diagnóstico principal, dependendo do método de reclassificação. ^159^ A insuficiência cardíaca não deve ser considerada uma causa básica de morte, mas constar da cadeia de eventos que levam à morte. Portanto, qualquer análise de dados do SIM que use insuficiência cardíaca como causa básica de morte a partir de declarações de óbito deve ser interpretada com cautela, pois pode estar estimando de maneira errada a verdadeira carga de insuficiência cardíaca.

•Dados obtidos da Fundação SEADE para mortalidade no estado de São Paulo em 2006 avaliaram 242.832 mortes em estimativa de 41.654.020 habitantes. ^135^ Insuficiência cardíaca e etiologias a ela associadas (exceto doença valvar primária) foram responsáveis por 6,3% do total de mortes. Para esses dados, não houve distribuição nem reclassificação das causas básicas de morte, tendo todas as etiologias associadas com insuficiência cardíaca sido incluídas ao se considerar o impacto da insuficiência cardíaca na mortalidade total.

•Um estudo sobre mortalidade por insuficiência cardíaca nos estados do Rio de Janeiro, São Paulo e Rio Grande do Sul incluiu dados de 2.960.857 declarações de óbito de 1999 a 2005. As porcentagens de morte por insuficiência cardíaca foram 3,0% no formulário restrito (insuficiência cardíaca como causa básica de morte) e 9,0% no formulário abrangente (insuficiência cardíaca mencionada em qualquer linha da declaração de óbito) em 1999. As porcentagens diminuíram com o tempo, passando para 2,4% e 8,6%, respectivamente, em 2005. As taxas de mortalidade decresceram na maioria dos grupos etários, exceto naquele a partir de 80 anos. As taxas aumentaram com a idade e foram claramente mais elevadas entre homens até os 80 anos de idade. ^160^

•Um estudo de coorte brasileiro mostrou dados de 1.220 pacientes ambulatoriais de uma clínica especializada em insuficiência cardíaca, acompanhados por 26±26 meses, de 1991 a 2000. Os pacientes encontravam-se em classe funcional III e IV, tinham idade média de 45,5±11 anos e 78% deles eram homens. As principais etiologias foram cardiomiopatia dilatada (37%), DCh (20%) e cardiomiopatia isquêmica (17%). Durante o período de acompanhamento, 415 (34%) pacientes morreram e 71 (6%) foram submetidos a transplante cardíaco. A DCh foi preditor de mau prognóstico. ^161^

•Dados mais recentes de 700 pacientes consecutivos com insuficiência cardíaca com fração de ejeção reduzida de uma clínica ambulatorial de um centro de saúde terciário em São Paulo mostraram mortalidade de 1 ano de 6,8% (47 pacientes). O desfecho composto de morte e hospitalização foi observado em 123 pacientes (17,7%) e 7 pacientes (1%) foram submetidos a transplante cardíaco. A idade média dos pacientes foi 55,4±12,2 anos e 67% eram homens. As principais etiologias foram cardiomiopatia hipertensiva (26,0%), isquêmica (21,9%) e chagásica (17,0%). Níveis séricos elevados de ureia e de peptídeo natriurético cerebral, assim como pressão arterial sistólica baixa, foram preditores independentes de mortalidade geral em 1 ano na amostra. ^162^

•Em estudo relatando dados do Banco Nacional de Marcapasso Multissítio, incluindo 3.526 pacientes de 2002 a 2007 atendidos no SUS, a idade média dos pacientes foi 59,8±13,3 anos e 66% eram homens. A sobrevida geral dos pacientes submetidos à terapia de ressincronização cardíaca no Brasil foi 80,1% (IC 95%, 79,4-80,8) em 1 ano e 55,6% (IC 95%, 54,6-56,6) em 5 anos, enquanto a mediana da sobrevida geral foi 30,3 meses (IIQ, 16,1-50,9). Observou-se ainda melhora da sobrevida na coorte estudada de 2002 a 2007 (p=0,055). ^163^

### Hospitalizações

•As hospitalizações são a principal consequência de insuficiência cardíaca descompensada, resultando em pior prognóstico e elevando os custos. O Estudo BREATHE avaliou uma amostra de pacientes admitidos por insuficiência cardíaca descompensada aguda. No total, 1.263 pacientes foram incluídos de 51 centros de diferentes regiões brasileiras em 2011 e 2012. A mortalidade hospitalar foi 12,6% e os indicadores de qualidade assistencial baseados nas recomendações de alta hospitalar foram alcançados em menos de 65% dos pacientes. ^164^

•Outros estudos sobre taxa de mortalidade, anteriores ao Estudo BREATHE, ^165^ ^,^ ^166^ mostraram taxas de mortalidade hospitalar similares, variando de 9% a 17%. ^165^

•Em uma comparação de pacientes com insuficiência cardíaca descompensada entre hospitais universitários terciários no Brasil e nos Estados Unidos, os pacientes dos Estados Unidos eram mais velhos (p < 0,01) e apresentaram maior prevalência da etiologia isquêmica (p < 0,01).

A permanência hospitalar foi significativamente mais curta (5 [IIQ, 3-9] vs. 11 [IIQ, 6-19] dias; p < 0,001) e a mortalidade hospitalar, menor (2,4% vs. 13%; p < 0,001) na coorte dos Estados Unidos, mas menos eventos clínicos nos 3 meses que se seguiram à alta foram observados nos pacientes brasileiros (42% vs. 54%; p = 0,02). Esse estudo ressalta a importância de se melhorar o conhecimento sobre insuficiência cardíaca em pacientes brasileiros para que se melhorem a assistência e os desfechos. ^167^

•Na revisão sistemática citada anteriormente, que avalia a carga de insuficiência cardíaca na América Latina, com 64% dos estudos incluídos provenientes do Brasil, ^153^ as taxas de hospitalização foram 33%, 28%, 31% e 35% em acompanhamentos de 3, 6, 12 e 24 a 60 meses, respectivamente. A mediana de permanência hospitalar foi 7,0 [IIQ, 5,20-11,00] dias. A mortalidade hospitalar foi 11,7% (IC 95%, 10,4%-13,0%), sendo as taxas maiores nos pacientes com fração de ejeção reduzida, doença isquêmica do coração ou DCh. A taxa de mortalidade em 1 ano foi 24,5% (IC 95%, 19,4-30,0).

•A partir de dados do SUS, foram descritos os números de hospitalizações e mortes por insuficiência cardíaca em São Paulo de 1992 a 2010. A taxa de mortalidade hospitalar por insuficiência cardíaca foi 15%. Ao se compararem os períodos de 1992-1993 e 2008-2009, houve diminuição de 32% no número de hospitalizações por insuficiência cardíaca (p = 0,002), aumento de 15% na mortalidade (p = 0,004) e aumento na permanência hospitalar por insuficiência cardíaca de 8,8 para 11,3 dias (p = 0,001). ^168^

•Outro estudo com dados do DATASUS avaliou as admissões por insuficiência cardíaca no Brasil no período de 2007 a 2016, comparando-as com as do Rio Grande do Sul e as de Porto Alegre, uma cidade com vários centros de referência. Como ilustra a Figura 4-10 , o estudo mostrou declínio nas taxas de mortalidade intra-hospitalar de 2007 a 2016 no Brasil (19% de redução) e no Rio Grande do Sul (25% de redução), e declínio ainda mais pronunciado em Porto Alegre (65%). ^169^

•Em 2020, um estudo mais recente com dados do DATASUS descreveu, no Brasil de 2008 a 2017, 51.172 hospitalizações por insuficiência cardíaca, que representou a principal causa de admissão por doença cardiovascular (29,4%). À semelhança do mencionado acima, esse estudo mostrou redução nas hospitalizações no período (34%; p = 0,004). Quando estratificados por idade, os indivíduos acima de 60 anos representaram 73% de todas as hospitalizações por insuficiência cardíaca no Brasil. A taxa de mortalidade por insuficiência cardíaca entre 2008 e 2015 foi 14,0 por 100 mil (± 0,53), com redução de 7,7% no período observado. ^170^

### Carga de Doença

•De acordo com estimativas do GBD 2017 ( Tabela 4-11 ), as taxas de YLDs padronizadas por idade por insuficiência cardíaca foram 112 (II 95%, 83-141) em 1990 e 109 (II 95%, 81-134) em 2017 por 100 mil habitantes, correspondendo a diminuição de 3% (II 95%, -6,7 a 0,3). Tais variações são similares às observadas nas taxas de prevalência de insuficiência cardíaca. A despeito dessa diminuição nas taxas de YLDs, a insuficiência cardíaca resultou em 88.114 (II 95%, 64.078-112.624) DALYs no Brasil em 1990 e em 234.169 (II 95%, 174.338-291.188) DALYs em 2017, devido ao crescimento e envelhecimento da população. ^152^

**Tabela 4-11:** – Número de YLDs e taxas de YLDs padronizadas por idade (por 100 mil) por insuficiência cardíaca por todas as causas, e variação percentual das taxas, no Brasil e suas unidades federativas, 1990 e 2017.

Local	1990		2017		Variação percentual (II 95%)
	Número (II 95%)	Taxa (II 95%)	n (II 95%)	Taxa (II 95%)	
Brasil	88114,2 (64078,1;112623,9)	112,2 (82,8;141,2)	234168,9 (174338,9;291187,7)	108,8 (81,4;134,5)	-3 (-6,7;0,3)
Acre	188,7 (140,7;234,7)	123,8 (95,3;148,9)	636,8 (490,6;767,6)	119,8 (93,4;142,4)	-3,2 (-9,6;5,2)
Alagoas	1230,4 (880,5;1587,3)	97,7 (70,9;124,1)	2951 (2189,4;3685,1)	101 (75;125,8)	3,3 (-5,8;12)
Amapá	100,2 (73,7;127,2)	112,2 (84,5;139,2)	451,5 (337,3;558,9)	108,9 (83,4;132,6)	-3 (-9,5;5)
Amazonas	810,4 (592,2;1020,8)	115,9 (87,9;141,5)	2702,1 (2050;3305,1)	112,2 (86,1;135,8)	-3,1 (-10;3,9)
Bahia	6684,8 (4893,5;8540,9)	101,7 (75,5;129)	15736,9 (11812,7;19456)	100,8 (75,3;125,3)	-0,8 (-7,7;7,9)
Ceará	3969,6 (2897,9;5084,5)	99,2 (72,8;126,2)	10451,4 (7838,4;12882,6)	106,6 (79,8;132)	7,4 (-1,3;17)
Distrito Federal	431,1 (298,6;581,8)	97,6 (70,8;128,8)	1940,3 (1366,1;2560)	96,9 (69,9;125,4)	-0,8 (-8,1;9,2)
Espírito Santo	1462,1 (1054,4;1878,9)	113,4 (84,3;143,6)	4162,7 (3063,9;5243,2)	104,9 (77,5;131,9)	-7,5 (-14,4;0,1)
Goiás	1592,7 (1128;2094,6)	103,8 (76,7;132,4)	6291,2 (4685;7868,1)	106,1 (79,5;131,7)	2,2 (-6,5;11,5)
Maranhão	2190,7 (1560,1;2873,3)	92 (66,4;119,5)	5992,2 (4402,4;7553,5)	97,8 (72;122,9)	6,3 (-1,6;15,9)
Mato Grosso	725,4 (526,7;941)	113,1 (83,9;142,3)	3003 (2248,2;3728,7)	112 (84,7;137,3)	-0,9 (-8,1;7,7)
Mato Grosso do Sul	850,1 (609,1;1106,8)	113,3 (83,8;142,8)	2829,1 (2072,2;3549,4)	110,8 (82,1;137,7)	-2,2 (-9,5;6,5)
Minas Gerais	9477,1 (6808,4;12335,3)	110,9 (82,3;141,3)	25557 (18974,8;31992,6)	103,6 (77,2;129,4)	-6,5 (-14,4;1,6)
Pará	2221,3 (1651,1;2795,8)	115,7 (88,1;142,8)	6624,9 (5036,2;8052,8)	109,8 (84,8;132,1)	-5,2 (-12,2;2,3)
Paraíba	2298,3 (1666,9;2947,6)	99,5 (72,4;126,3)	4808 (3560,3;5995,7)	103,4 (76,3;129,4)	4 (-4,2;12,5)
Paraná	4964,3 (3563,3;6413,6)	123,5 (92,3;154,2)	13883,5 (10128,6;17249,1)	117 (86,8;144,4)	-5,2 (-12,7;3,3)
Pernambuco	4627,4 (3326;5865,3)	110,9 (81,9;137,8)	10375,1 (7650,4;12784)	109,6 (81;134,6)	-1,2 (-9;7,3)
Piauí	1230,3 (866,5;1647,8)	92,1 (65,6;122)	3402,3 (2478,9;4365,3)	95,1 (69,4;122,2)	3,3 (-5;11,9)
Rio de Janeiro	9922,7 (7229,9;12662,8)	119,7 (88,6;150,3)	22953,2 (16743,8;28786)	110 (81,3;137,7)	-8,1 (-14,7;-0,4)
Rio Grande do Norte	1606,1 (1145,6;2057,5)	99,5 (71,8;126,6)	3856,8 (2838,4;4879,3)	103,5 (75,7;132)	4 (-3,9;12)
Rio Grande do Sul	8134 (5994,6;10280,5)	140,5 (106,7;171,9)	18696,6 (14241,9;22612,8)	126,9 (97;152,5)	-9,7 (-16,6;-2,5)
Rondônia	300,3 (209,4;397,4)	117,4 (86,9;145,5)	1408,5 (1062,2;1750,7)	111,9 (85,8;137)	-4,7 (-11,7;4,4)
Roraima	46,3 (32,4;62,3)	101,5 (74,8;130,8)	276,8 (197,7;358,6)	98,7 (72,2;125,2)	-2,8 (-9,7;5)
Santa Catarina	2880 (2095,3;3688,1)	133,3 (99,4;165,6)	8433,4 (6334,3;10482,5)	119,7 (90,8;147,4)	-10,2 (-16,8;-3,4)
São Paulo	18988,5 (13523,4;24849,5)	112,8 (81,9;144,5)	53360,6 (38622,8;68602,6)	109,6 (80,1;140,1)	-2,9 (-10,6;5,1)
Sergipe	853,9 (627,3;1082,5)	102,4 (75,6;129)	2085,5 (1551,2;2578,3)	103,7 (77,4;127,2)	1,3 (-6,3;9,9)
Tocantins	327,5 (226,3;438,8)	96,4 (69,6;126,2)	1298,6 (952,1;1635,1)	100,3 (73,9;125,9)	4 (-4,3;14,5)

*Fonte: Fonte: Dados derivados do estudo Global Burden of Disease 2017, Institute for Health Metrics and Evaluation, University of Washington. ^46^*

•A Tabela 4-12 mostra as taxas de YLDs por insuficiência cardíaca de acordo com o sexo e os grupos etários, a partir de estimativas do Estudo GBD 2017. As taxas de YLDs padronizadas por idade foram similares para mulheres e homens em 1990, mas as de 2017 foram 105 (II 95%, 82-127) para homens e 111 (II 95%, 80-141) para mulheres, devido a redução de 6,8% (II 95%, -10,9 a -2,6) para homens e quase nenhuma redução para mulheres (-0,3%, IC 95%, -4,9 a 4,2). Como esperado, as mais altas taxas de YLDs foram observadas no grupo de 70+ anos, seguido pelo grupo de 50-69 anos.

À semelhança das variações observadas na prevalência, de 1990 a 2017, os maiores aumentos de YLDs foram verificados no grupo de 15-49 anos. ^152^

### Utilização e Custo da Atenção à Saúde

**(Ver Tabelas 1-6 a 1-9 e Figuras 1-15 e 1-16 )**

•De acordo com dados do SUS, houve 3.085.359 hospitalizações por insuficiência cardíaca de 2008 a 2019. Esse número representa um terço do total de hospitalizações clínicas relacionadas às condições cardiovasculares no período estudado. Os custos não ajustados foram R$ 3.957.126.308. Em dólares internacionais, valores convertidos para paridade do poder de compra e ajustados para US$ 2019, os custos foram Int$ 2.651.479.951. ^95^

•No período observado, houve redução no número de hospitalizações clínicas por insuficiência cardíaca, que passaram de 298.474 (157 por 100 mil) em 2008 para 222.620 (105 por 100 mil) em 2019, sendo a redução uniforme ao longo dos anos. A despeito dessa redução no número de admissões, os gastos em saúde não ajustados estimados a partir do pagamento direto por assistência a pacientes com insuficiência cardíaca aumentaram de 2008 para 2019 em quase 32%, passando de R$ 272.280.662 (2019 Int$ 267.102.469) em 2008 para R$ 359.301.691 (2019 Int$ 173.659.589) em 2019. O número decrescente de hospitalizações e o gasto crescente representam maiores custos por admissão no período observado (R$ 912 em 2008 para R$ 1.568 em 2018). Insuficiência cardíaca foi responsável pela maioria dos custos relacionados às hospitalizações clínicas por doenças cardiovasculares.

•A carga econômica de insuficiência cardíaca no Brasil foi avaliada usando-se o custo padrão da estrutura da doença para considerar os custos em 2015. Foram analisados a prevalência e os gastos associados ao tratamento, à perda de produtividade por redução do emprego, aos custos da provisão de cuidado formal e informal e à perda de bem-estar. O estudo relatou que a insuficiência cardíaca determina um custo financeiro de R$ 22,1 bilhões/US$ 6,8 bilhões, o segundo dentre as quatro principais condições cardíacas no Brasil: infarto do miocárdio, insuficiência cardíaca, hipertensão e fibrilação atrial. ^124^

•Em estudo utilizando dados do DATASUS sobre admissões por insuficiência cardíaca em 10 anos, Nicolao Nicolao *et al* . ^169^ mostraram um aumento de 97% no custo médio por paciente das hospitalizações relacionadas com insuficiência cardíaca de 2007 a 2016. Dados de Porto Alegre, uma cidade com vários hospitais de referência, mostraram um aumento ainda mais pronunciado (135%), como também uma diminuição mais pronunciada na mortalidade, em comparação aos dados do Brasil (ver acima).

### Transplante Cardíaco Aberto e Implantação de Dispositivo de Assistência Ventricular

•O número de transplantes cardíacos realizados no Brasil aumentou de 149 em 2006 para 357 em 2016 e, embora esse aumento tenha sido significativo naquele período, representa cerca de um quinto da necessidade estimada da população. A sobrevida de 1 ano foi 73%. A sobrevida de 1 ano foi 73% (dados de sobrevida de 2010). ^171^

•Uma análise de custo de transplante cardíaco no Brasil, com todos os receptores consecutivos de transplante cardíaco em um único centro de julho de 2015 a junho de 2017, mostrou, para os 27 pacientes incluídos, uma média de custo total de US$ 74.341, que é mais baixa do que as relatadas para países desenvolvidos, mas excede em 60% o valor de reembolso por paciente. ^172^

•Em estudo descritivo de hospital público de referência em cardiologia localizado em Fortaleza, 16 pacientes foram submetidos a implantação de dispositivo de assistência ventricular de 2008 a 2015. A idade média foi 40,1±3,4 anos e 87,5% eram homens. Cardiopatia chagásica foi a principal etiologia (37,5%). Todos os pacientes apresentaram complicações durante o uso do dispositivo, sendo sangramento a mais frequente [11 (68,8%)]. Quanto ao desfecho clínico, 10 pacientes (62,5%) foram submetidos a transplante cardíaco e 5 (31,3%) morreram. ^173^

## Pesquisa Futura

•Por ser a insuficiência cardíaca considerada um código *garbage* quando designada como causa básica de morte, são necessários estudos que investiguem o melhor método para reclassificar e redistribuir essa causa de modo a reduzir viés e propiciar melhor comparabilidade de dados para o aperfeiçoamento das políticas de saúde.

•Estudos brasileiros de coorte sobre cardiomiopatias são raros, tendo alguns estudos clínicos no Brasil informado dados de insuficiência cardíaca, havendo, no entanto, poucos estudos multicêntricos com dados da população brasileira. Vale ressaltar a importância de se poder contar com dados tanto de insuficiência cardíaca quanto de cardiomiopatia, assim como de pacientes ambulatoriais e hospitalizados, além de se compreender de maneira ampla a carga crescente da insuficiência cardíaca nas doenças cardiovasculares. São necessários mais estudos multicêntricos de larga escala para melhor descrever a carga, os desfechos e os custos da insuficiência cardíaca na população brasileira.

•Além disso, estudos que explorem a qualidade e os custos da assistência na insuficiência cardíaca auxiliariam no desenvolvimento de políticas de saúde para melhorar a conscientização, o acesso a intervenções que salvam vidas, a doação de órgãos, assim como o uso de recursos nesta doença tão complexa.

•As taxas de mortalidade por DCh diminuíram substancialmente nas últimas décadas e espera-se que continuem diminuindo nos próximos anos. Há, na verdade, evidência de que a DCh seja uma causa de morte subnotificada, assim como, provavelmente, de hospitalização. São necessários mais dados sobre taxas de hospitalização e desfechos de pacientes com CMCh.

## 5. DOENÇA VALVAR DO CORAÇÃO

### CID-9 424; CID-10 I34 a I38

**Ver Tabelas 5-1 a 5-5 e Figuras 5-1 a 5-11**

**Table:** Abreviaturas usadas no Capítulo 5

CID	Classificação Estatística Internacional de Doenças e Problemas Relacionados à Saúde
CRVM	Cirurgia de Revascularização do Miocárdio
DAC	Doença Arterial Coronariana
DALYs	Anos de vida perdidos ajustados por incapacidade (do inglês, *Disability-Adjusted Life-Years* )
DCR	Doença Cardíaca Reumática
DVNR	Doença Valvar do Coração Não Reumática
ECG	Eletrocardiograma
FA	Fibrilação Atrial
FRA	Febre Reumática Aguda
GBD	Global Burden of Disease
IC	Intervalo de Confiança
II	Intervalo de Incerteza
IL	Interleucina
SUS	Sistema Único de Saúde
TAVI	Implantação Percutânea de Valva Aórtica (do inglês, *Transcatheter Aortic Valve Implantation* )
UF	Unidade Federativa

**Tabela 5-1:** – Taxas de prevalência padronizadas por idade de doença valvar do coração por 100 mil, em 1990 e 2019, e variação percentual das taxas, no Brasil e suas unidades federativas.

Causa de morte e localização	1990		2019		Variação percentual (II 95%)
	Número (II 95%)	Taxa (II 95%)	Número (II 95%)	Taxa (II 95%)	
**B.2.1-Doença cardíaca reumática **					**(;)**
Acre	3450,4 (2590,5;4467,5)	871,3 (675,4;1091,9)	8765,8 (6801,6;11017,2)	889,9 (698,1;1114,6)	2,1(-4,3;8,5)
Alagoas	21422,5 (16168,7;27275,5)	870,4 (671,7;1095,4)	34480,6 (26684,6;43431,6)	911,8 (708,5;1147,6)	4,8(-1,4;12,1)
Amapá	2291,4 (1738,2;2949,2)	877,6 (686,6;1102)	8204,3 (6320,1;10401,1)	893,7 (695,8;1127,2)	1,8(-3,6;8,2)
Amazonas	17173,8 (12858,6;21944,2)	859,3 (662,8;1072,3)	39188,9 (30084;49139)	877,9 (681,7;1093,7)	2,2(-4,5;8,7)
Bahia	102924,7 (78855,6;131748,4)	883,1 (691,6;1106,3)	153263,4 (118427;190547,8)	923,2 (714,7;1146,6)	4,5(-1,6;10,7)
Brasil	1352613,2 (1040490,6;1695888,8)	899,6 (699,8;1119,1)	2102091,3 (1639303,8;2606302,5)	918,5 (716;1142,5)	2,1(0,2;4)
Ceará	54893,6 (41770,6;69929,9)	879,9 (684,7;1108,8)	94345,5 (73759;117854,4)	907,6 (712,2;1133,8)	3,1(-3;9,6)
Distrito Federal	14943,3 (11322,7;18780,2)	882,4 (685,5;1098,2)	29679,7 (23036,6;37007,4)	884,6 (683;1107,7)	0,3(-5,5;6)
Espírito Santo	23622,8 (18038;29882,3)	883,7 (685,2;1105,3)	37781 (29560,4;47163,4)	895,2 (696,3;1123,7)	1,3(-4,7;8)
Goiás	38342,4 (29158;48881,8)	894,2 (695;1119,9)	68040,7 (52578,2;84812,4)	911,1 (703,6;1137,2)	1,9(-3,8;9,3)
Maranhão	40195 (30705,5;51338,9)	862,1 (667,6;1078,8)	74568,2 (57721,4;93956,1)	894 (691,7;1118,2)	3,7(-2,4;11)
Mato Grosso	17655,9 (13403,8;22630,5)	866,2 (670,9;1092,4)	34380,9 (26531,8;42865,4)	881,9 (679;1099,6)	1,8(-4,1;7,9)
Mato Grosso do Sul	16461,5 (12566,8;20841,5)	900,8 (701,7;1128,6)	27971,6 (21773,1;34975)	923,4 (716,7;1154,5)	2,5(-3,6;9,2)
Minas Gerais	145136,4 (111832;182475)	893,7 (700,5;1114)	210129,5 (164841,3;261917,1)	917,3 (715,3;1146)	2,6(-3,5;8,1)
Pará	41348,9 (31068,7;52497,1)	871,9 (674,1;1090,2)	87391,4 (67117,7;110404)	890,7 (690,9;1115,6)	2,2(-3,4;8,6)
Paraíba	27841,4 (21209,4;35301,7)	888,2 (685,4;1106,3)	40854,4 (31738,7;51294)	912,9 (708,4;1149,3)	2,8(-3,3;8,8)
Paraná	81156 (62014,8;102568,9)	919,2 (710,1;1145,5)	113818,3 (88747,4;140998)	939,4 (730,5;1177,7)	2,2(-3,3;7,8)
Pernambuco	63259,3 (48155,2;80253)	886 (681,7;1107,1)	96588,1 (74881,1;120052)	913,6 (707,8;1139,5)	3,1(-2,5;10,4)
Piauí	22494,3 (16958,5;28886,4)	890,4 (687,3;1117,5)	34073,1 (26412,8;42807,5)	906,3 (700,6;1139,5)	1,8(-4,7;8,5)
Rio de Janeiro	124439,1 (95279,2;157326,7)	902,8 (694,5;1137,9)	172188,9 (135252,5;212372,6)	920,6 (712,8;1140,7)	2(-4,1;7,9)
Rio Grande do Norte	21271,9 (16243;26755,5)	895,1 (696,6;1119,8)	35469,3 (27439,4;44530,2)	910 (704;1144,4)	1,7(-4,1;8,4)
Rio Grande do Sul	89482,7 (68907,8;113899,5)	930,3 (722,5;1165,4)	111269 (87372;139471)	937,4 (724,5;1173)	0,8(-5,2;6,6)
Rondônia	9709,1 (7330,4;12319,3)	862 (671,8;1077,8)	16995,1 (13142,4;21253,2)	879,4 (681;1097,4)	2(-4;8,1)
Roraima	1816,1 (1377,7;2311,1)	854,8 (666,6;1069,7)	5547,7 (4230,2;6961,8)	878,9 (678,9;1101,6)	2,8(-3,3;8,7)
Santa Catarina	44246,8 (34096,9;55614,1)	936,1 (728;1169,1)	72819,1 (57156,6;90488,1)	942,9 (735,4;1169,9)	0,7(-5,1;6,9)
São Paulo	306459,4 (235629,4;380385,5)	917,2 (710,2;1138)	455434,6 (356653,1;565502,4)	934,7 (731,5;1171,7)	1,9(-3,4;7,7)
Sergipe	12997,3 (9939,7;16567,3)	889,4 (692,3;1115,1)	23773,4 (18367,2;29453,9)	925,7 (715,4;1147,8)	4,1(-2,9;11,1)
Tocantins	7577,3 (5704,2;9694,6)	859,4 (660,2;1078,4)	15068,9 (11720,2;18871,1)	868,5 (675,1;1087,3)	1,1(-4,9;8)
**B.2.5-Doença valvar do coração não reumática **					**(;)**
Acre	45,9 (37,3;55)	21,8 (18,2;25,8)	237,7 (200,3;276,7)	33,6 (28,4;39)	53,9(29,2;85,5)
Alagoas	317,7 (260,6;374,9)	20,8 (17,1;24,3)	1007,7 (843,5;1167,9)	29,5 (24,7;33,9)	41,6(15,4;74,6)
Amapá	31,6 (26,1;37,8)	24,8 (20,8;29)	236,1 (197;275,5)	38,4 (32,5;44,8)	54,7(27,4;87,3)
Amazonas	247,3 (204;295,5)	24,3 (20,5;28,5)	1237,1 (1037,9;1449,9)	37,7 (31,7;43,8)	55,2(30,6;84,6)
Bahia	1717,8 (1432,8;2026,8)	23 (19,5;27,1)	4952,1 (4171,7;5747,9)	29,8 (25,1;34,6)	29,2(6,4;55,7)
Brasil	26255,7 (23385;28824,5)	25,3 (22,4;27,8)	95300,5 (82828,7;108921,1)	39 (33,9;44,6)	54,3(41,1;68,3)
Ceará	938,5 (780,3;1092,3)	21,5 (18;25)	3319,7 (2799,3;3846,7)	32,5 (27,4;37,8)	51(25;84)
Distrito Federal	277,5 (231,5;328,5)	30,8 (26,2;35,6)	3271,6 (2650,9;3935,7)	110 (89,2;132)	256,6(194,9;333,7)
Espírito Santo	421,7 (344,2;499,2)	24,1 (20,2;28,2)	1776,4 (1477;2076,8)	38,5 (32,2;44,9)	59,9(32,5;91,1)
Goiás	626,9 (524,7;740,6)	23,6 (20,1;27,7)	2652,7 (2242,2;3112,3)	35 (29,6;40,9)	47,9(23,9;78,7)
Maranhão	583,9 (482,4;690,1)	20,1 (16,8;23,5)	1807,7 (1525;2100,4)	26,1 (22;30,2)	29,9(4,9;59,3)
Mato Grosso	252,7 (209,6;298,6)	23 (19,5;26,7)	1498,6 (1253,1;1755,6)	40,7 (34,2;47,7)	77,2(48,5;112)
Mato Grosso do Sul	288,6 (237,7;341,2)	25,5 (21,3;29,8)	1356,1 (1126;1592)	43,4 (36;51)	70,2(40,9;104,5)
Minas Gerais	2731,8 (2280;3218,2)	23,9 (20,2;28)	8965,3 (7605,9;10440,9)	33,4 (28,3;38,9)	39,9(17,1;69,5)
Pará	577,7 (476,5;682,4)	22 (18,4;25,6)	2437,5 (2051,8;2854,5)	31,4 (26,6;36,5)	42,9(19,1;71,9)
Paraíba	505,8 (426,6;591)	21,8 (18,4;25,4)	1401,1 (1193;1612,9)	29,8 (25,4;34,3)	36,7(13,3;66,1)
Paraná	1569,6 (1320,1;1846,9)	26,2 (22,3;30,5)	5786,1 (4865,1;6888,9)	41,5 (34,8;49,4)	58,4(32,8;87,6)
Pernambuco	1120,9 (946,9;1304,1)	22,4 (19,1;26)	3402,5 (2879,5;3968,3)	32,3 (27,2;37,6)	44(17,7;72,8)
Piauí	333,2 (271,4;393,2)	20,7 (17,2;24,2)	1013,3 (857,1;1175,3)	26,7 (22,6;31)	29,3(6,9;57,3)
Rio de Janeiro	2932 (2439;3452,2)	26,4 (22,3;31)	8945,1 (7447;10466,6)	39,3 (32,8;46)	48,6(23,7;76,8)
Rio Grande do Norte	384,4 (318,7;451,3)	23,2 (19,4;27,1)	1382,3 (1144,1;1600,7)	35 (29;40,5)	50,8(25,6;82,7)
Rio Grande do Sul	2261,2 (1899,8;2643,1)	29,7 (25,1;34,4)	6749,2 (5633,2;7857,4)	43,5 (36,4;50,6)	46,3(23,8;72,5)
Rondônia	130 (107,2;154,2)	23,1 (19,2;26,7)	648,6 (544,5;759,1)	37,4 (31,3;43,7)	61,6(34,5;95,9)
Roraima	21,3 (17;25,7)	22,2 (18,6;25,9)	162,4 (134,6;189,9)	35,6 (29,9;41,3)	60,2(30,6;93,9)
Santa Catarina	841,9 (702,4;975,8)	26,9 (22,6;31,1)	3775,8 (3163,4;4442,1)	43,5 (36,6;51)	61,7(36,3;93,4)
São Paulo	6786,6 (5675;7889)	28,1 (23,7;32,6)	26025,6 (21778,6;30883,2)	46,2 (38,8;54,9)	64,2(38,6;94,8)
Sergipe	196,5 (162,9;230,2)	21,7 (18,1;25,3)	738,2 (628,7;864,3)	30,6 (26;35,7)	41,2(16,6;72,3)
Tocantins	112,9 (93,1;134,4)	21,3 (18;25,1)	514 (434;596,7)	33,4 (28,2;38,7)	56,3(29,1;89,9)
**B.2.5.1-Doença valvar** **aórtica calcífica não reumática**					**(;)**
Acre	10,1 (8;12,6)	5 (4;6,2)	123,4 (98;151,9)	18,5 (14,6;22,9)	266,7(224,6;314,6)
Alagoas	56,9 (45,1;70,2)	3,8 (3,1;4,7)	444,1 (354,3;546,4)	13,4 (10,7;16,5)	249,8(212;294,5)
Amapá	9,2 (7,3;11,3)	7,6 (6;9,4)	133,4 (106,8;163,6)	23,2 (18,7;28,5)	204,1(173,5;241,7)
Amazonas	70,2 (55,6;86,4)	7,3 (5,8;9)	705,8 (562,7;866,4)	22,6 (18;28)	210,8(179,8;251,5)
Bahia	404,7 (324,5;495,1)	5,5 (4,4;6,8)	2294,2 (1832,8;2814,7)	14,1 (11,2;17,3)	154,2(129,8;184,4)
Brasil	7905,4 (6333;9659,2)	7,9 (6,3;9,6)	57152,9 (45926,6;70348,4)	23,7 (19,1;29)	201,8(177,5;231,8)
Ceará	196,4 (154,7;244,3)	4,6 (3,6;5,6)	1730 (1381,8;2137)	17,2 (13,7;21,3)	276,5(231,9;332)
Distrito Federal	108,8 (85,4;132,8)	13,2 (10,5;16,3)	2764 (2173,3;3401,8)	94,8 (75;117,1)	618,3(520,2;732,3)
Espírito Santo	111,5 (88,6;136,7)	6,6 (5,3;8,2)	1049,8 (835,9;1303,9)	23,2 (18,5;28,6)	251,4(211,7;295)
Goiás	164,1 (130,9;201,4)	6,5 (5,2;8)	1421,1 (1139,4;1754,3)	19,4 (15,6;23,8)	198,4(168;237,7)
Maranhão	95,1 (74,8;119,1)	3,3 (2,6;4,1)	695,4 (554,8;852,2)	10,3 (8,2;12,6)	209,2(176,7;247,1)
Mato Grosso	63,4 (49,8;78,4)	6,2 (4,9;7,6)	919,2 (726;1132,8)	26 (20,7;32)	321(274,6;376,5)
Mato Grosso do Sul	86,4 (68,8;106)	8,1 (6,4;10)	867,8 (687,5;1078,8)	28,3 (22,6;34,8)	249,9(214,5;294)
Minas Gerais	727,1 (568,6;898,5)	6,5 (5,1;8)	4835 (3834,5;5958,1)	18,1 (14,4;22,2)	177,8(150,7;214,2)
Pará	129,4 (102,4;159,9)	5,2 (4,1;6,3)	1199,8 (951,4;1476,5)	16,2 (12,8;20)	214,6(179,7;254,2)
Paraíba	106 (83,3;131,7)	4,5 (3,6;5,7)	669,2 (532,7;821,2)	14,3 (11,4;17,6)	215(182,9;255,2)
Paraná	500,8 (399;618,4)	8,8 (7;10,8)	3677,2 (2950,2;4581,6)	26,7 (21,5;33,1)	204,3(171,8;239,2)
Pernambuco	243,8 (191,2;302,7)	5 (3,9;6,2)	1696 (1349,5;2086,2)	16,5 (13,1;20,2)	230,5(196;271,1)
Piauí	61,4 (48,4;75,5)	3,9 (3,1;4,8)	410,2 (324,2;499,1)	10,9 (8,6;13,3)	180(152,3;219,4)
Rio de Janeiro	907,3 (714,4;1122,4)	8,5 (6,8;10,5)	5421,6 (4290,2;6733,4)	23,8 (18,9;29,4)	181(153,8;214,6)
Rio Grande do Norte	95,8 (76,2;118,1)	5,8 (4,6;7,2)	762,9 (606,7;931,8)	19,6 (15,6;24)	238,6(204,2;283,4)
Rio Grande do Sul	894,7 (716,2;1105)	12,2 (9,7;15)	4459,5 (3551,3;5500,3)	28,5 (22,9;34,9)	133,9(112,6;159,4)
Rondônia	32,2 (25,2;39,9)	6,3 (5;7,8)	369,3 (291;456,9)	22,4 (17,7;27,6)	252,9(213,9;300,7)
Roraima	5,4 (4,2;6,8)	6,1 (4,8;7,5)	88,3 (69,6;109,8)	20,8 (16,6;25,8)	242(206,2;283,6)
Santa Catarina	288,2 (232,8;354)	9,6 (7,7;11,8)	2440,1 (1939,3;3004,9)	28,6 (22,9;35)	198(167,5;231)
São Paulo	2472,7 (1981,2;3024,5)	10,6 (8,5;13)	17366,6 (13732,1;21578,8)	31,1 (24,7;38,6)	193(162;227,9)
Sergipe	39,7 (31,1;49,7)	4,5 (3,6;5,6)	346,4 (275,9;428,6)	14,9 (11,8;18,5)	232(196,5;272,4)
Tocantins	24,3 (19,1;30,2)	4,8 (3,8;6)	262,7 (209,8;323,3)	17,7 (14,1;21,7)	265,3(224,9;313,9)
**B.2.5.2-Doença valvar mitral** **degenerativa não reumática**					**(;)**
Acre	47,4 (43,9;50,9)	21,7 (20,2;23,3)	163,5 (151,5;176,9)	21,6 (20;23,2)	-0,5(-4,7;4,2)
Alagoas	346 (320;372,4)	22,2 (20,6;23,9)	761,9 (707,8;817,9)	21,9 (20,3;23,5)	-1,6(-6;2,8)
Amapá	30,3 (28;32,5)	22,2 (20,6;23,8)	147,5 (135,3;160)	21,8 (20,1;23,6)	-1,8(-5,6;2,1)
Amazonas	239,6 (222,1;258,5)	22,1 (20,5;23,8)	760,6 (700,6;827)	21,6 (20;23,4)	-2,4(-6,9;2,3)
Bahia	1701,1 (1585,3;1824,1)	22,3 (20,7;23,9)	3654,4 (3395,5;3938,8)	21,8 (20,2;23,4)	-2,3(-6,1;1,9)
Brasil	24034,9 (22437,7;25715,6)	22,5 (21;24)	53918,2 (50205,1;57756,5)	22 (20,5;23,5)	-2,3(-4;-0,4)
Ceará	979,8 (914;1051,6)	22,1 (20,6;23,7)	2248,8 (2085,5;2425,1)	21,8 (20,2;23,4)	-1,6(-6,1;2,6)
Distrito Federal	224,3 (207,5;242,2)	23,1 (21,5;24,7)	731,7 (675,1;789,5)	22,5 (20,9;24,2)	-2,4(-7,1;2)
Espírito Santo	409,4 (381;439,6)	22,6 (21,1;24,2)	1025 (949;1104,5)	22,2 (20,6;23,9)	-1,7(-5,8;2,5)
Goiás	608,1 (564,3;652,9)	22,1 (20,5;23,7)	1678,8 (1548,4;1815,2)	21,8 (20,1;23,5)	-1,3(-5,6;3)
Maranhão	641,7 (595,4;691,4)	21,7 (20,2;23,4)	1520,8 (1402,1;1649,9)	21,4 (19,8;23,2)	-1,5(-6,6;2,8)
Mato Grosso	251,2 (232,1;271,5)	21,6 (20,1;23,1)	828,9 (766,4;894,9)	21,7 (20,1;23,3)	0,4(-4;4,9)
Mato Grosso do Sul	261,3 (241,6;281,7)	22,1 (20,5;23,7)	692,5 (640,2;746,3)	21,9 (20,3;23,5)	-1,1(-5,7;3,5)
Minas Gerais	2629,8 (2444,6;2826,8)	22,5 (21;24,1)	5799,3 (5351,7;6249,1)	21,9 (20,3;23,6)	-2,6(-7;1,7)
Pará	599,1 (557;645,2)	22 (20,4;23,5)	1752,1 (1625,8;1905,2)	21,5 (20;23,3)	-1,9(-6,1;2,7)
Paraíba	519,3 (482,7;555,7)	22,2 (20,6;23,7)	1015,4 (940,1;1094,2)	21,6 (20;23,3)	-2,6(-6,7;1,8)
Paraná	1395,4 (1296,6;1498,3)	22,6 (21;24,2)	3058,4 (2831,7;3304,5)	22,1 (20,5;23,9)	-1,9(-6,4;2,6)
Pernambuco	1150 (1065,2;1234,1)	22,6 (20,9;24,3)	2351 (2177,2;2534,8)	22,1 (20,5;23,7)	-2,5(-6,6;2,2)
Piauí	357,2 (330,8;383,5)	21,9 (20,2;23,4)	819,1 (761,2;882,8)	21,5 (20;23,2)	-1,4(-6,2;3,2)
Rio de Janeiro	2586,9 (2403,3;2775)	22,9 (21,3;24,6)	4954,2 (4572,2;5335,5)	22,2 (20,5;23,9)	-3,2(-7,4;1,5)
Rio Grande do Norte	376,6 (350,1;404)	22,2 (20,7;23,9)	869 (804,6;936,1)	21,7 (20,1;23,4)	-2,3(-6,4;1,7)
Rio Grande do Sul	1774,1 (1650,6;1908,7)	22,8 (21,3;24,5)	3317,6 (3070,6;3580,6)	22,2 (20,5;23,9)	-2,9(-7;1,2)
Rondônia	128,5 (118,7;139,3)	21,5 (20;23)	389,8 (359,5;421,6)	21,5 (19,9;23,3)	0,2(-3,9;5,1)
Roraima	21,8 (20,1;23,6)	20,9 (19,4;22,5)	104,3 (96;112,7)	21,2 (19,5;22,8)	1,5(-2,6;5,8)
Santa Catarina	740,9 (687,2;794,5)	22,7 (21,1;24,3)	1915,3 (1772,8;2077,6)	22,1 (20,5;23,9)	-2,9(-6,9;1,5)
São Paulo	5691,2 (5272,3;6125,5)	22,8 (21,2;24,5)	12477,6 (11520,7;13408)	22,3 (20,6;23,9)	-2,4(-6,8;2)
Sergipe	207,1 (192,7;221,7)	22,4 (20,9;23,9)	536,6 (497,6;577,2)	21,8 (20,3;23,5)	-2,4(-6,4;1,9)
Tocantins	117 (108,8;126,2)	21,6 (20,2;23,3)	344,2 (318,6;371,1)	21,7 (20,1;23,4)	0,4(-4;4,5)

*Fonte: Dados derivados do estudo Global Burden of Disease 2019, Institute for Health Metrics and Evaluation, University of Washington. ^46^*

**Tabela 5-2:** – Taxas de mortalidade e de DALYs por 100 mil em 1990 e 2019 e variação percentual das taxas, por idade, sexo e causa de morte no Brasil.

Causa de morte e localização	Taxa mortalidade 1990	Taxa mortalidade 2019	Variação percentual da taxa mortalidade (II 95%)	Taxa de DALYs 1990	Taxa de DALYs 2019	Variação percentual da taxa de DALYs (II 95%)
	(II 95%)	(II 95%)		(II 95%)	(II 95%)	
**B.2.1-Doença cardíaca reumática **						
Ambos						
15-49 anos	1,7 (1,6;1,8)	0,6 (0,6;0,7)	-64,6 (-68,3;-60,4)	159,1 (132,1;193,1)	**(II 95%** 95,5 (68,7;130,8)	-40 (-48,4;-32,1)
50-69 anos	6 (5,5;6,5)	2,6 (2,4;2,9)	-55,9 (-61,1;-50,4)	214,1 (194,3;236,2)	113,4 (97,5;132,9)	-47 (-53,2;-40,7)
5-14 anos	0,7 (0,6;0,7)	0,1 (0,1;0,2)	-78,1 (-81,9;-73,6)	78,4 (64,6;99)	39 (25,8;60,5)	-50,3 (-62;-38,6)
70+ anos	12,5 (11,4;13,6)	6,8 (6;7,6)	-45,4 (-52,5;-38,4)	202,1 (184,6;218,4)	111,1 (98,3;123,3)	-45,1 (-51,7;-39,1)
Padronizada por idade	2,8 (2,7;3)	1,2 (1,1;1,2)	-59,4 (-63,1;-55,4)	144,6 (126,8;167,3)	79,3 (61,6;102,6)	-45,1 (-52,1;-38,2)
Todas as idades	2,1 (2;2,2)	1,3 (1,2;1,3)	-39,6 (-45,2;-33,9)	132,8 (114,5;156,3)	85 (66,3;109,9)	-36 (-43,2;-29,2)
Abaixo de 5	0,4 (0,3;0,6)	0 (0;0,1)	-88,2 (-93,4;-79,6)	35 (24,5;50,2)	6,6 (4,6;9,2)	-81,3 (-89,1;-69,5)
Feminino						
15-49 anos	2 (1,9;2,1)	0,7 (0,7;0,8)	-63,3 (-67,4;-58,2)	179,2 (149,4;216,1)	109,5 (79,7;149,9)	-38,9 (-47;-30,7)
50-69 anos	6,9 (6,3;7,6)	3,1 (2,8;3,4)	-55,2 (-60,7;-49)	250,3 (225,4;278,8)	134,8 (115,2;158,4)	-46,2 (-52,5;-39)
5-14 anos	0,7 (0,6;0,7)	0,1 (0,1;0,2)	-77,6 (-81,5;-73,3)	80,8 (65,9;102,2)	42 (27,5;64,6)	-48,1 (-59,8;-36,8)
70+ anos	12,7 (11,5;13,9)	7,6 (6,5;8,4)	-40,5 (-48,8;-32)	207,1 (187,7;226)	121,4 (106,1;136)	-41,4 (-48,5;-34,2)
Padronizada por idade	3,2 (3;3,4)	1,3 (1,2;1,4)	-58,1 (-62,5;-53,3)	162,2 (142,3;187)	90,5 (70,7;116,5)	-44,2 (-51,1;-36,9)
Todas as idades	2,4 (2,3;2,5)	1,5 (1,4;1,7)	-35,8 (-42,3;-28,6)	149,3 (129,2;174,9)	98,6 (78,1;126)	-34 (-40,9;-26,9)
Abaixo de 5	0,4 (0,3;0,6)	0,1 (0;0,1)	-87,3 (-93,2;-77,3)	37,8 (24,7;56,3)	7,4 (5,1;10,3)	-80,5 (-89,1;-66,6)
Masculino						
15-49 anos	1,5 (1,4;1,6)	0,5 (0,4;0,5)	-66,5 (-70,6;-61,4)	138,3 (113,9;169,9)	81,2 (57,8;112,1)	-41,3 (-50,3;-33,4)
50-69 anos	4,9 (4,5;5,4)	2,1 (1,9;2,3)	-57,6 (-63,4;-50,7)	174,8 (157,8;193,8)	89,3 (76;105,2)	-48,9 (-55,5;-41,7)
5-14 anos	0,7 (0,6;0,7)	0,1 (0,1;0,2)	-78,6 (-82,9;-73,3)	76 (62,4;95,1)	36,1 (23,5;56,5)	-52,5 (-64,3;-40,3)
70+ anos	12,3 (11,2;13,6)	5,8 (5,1;6,5)	-52,5 (-60,3;-44,5)	196 (179;215,5)	96,9 (86,2;108,4)	-50,5 (-57,7;-43,1)
Padronizada por idade	2,5 (2,4;2,7)	1 (0,9;1)	-62 (-66,7;-56,7)	126,1 (109,6;146,8)	67,3 (51,7;87,7)	-46,7 (-54,4;-39,3)
Todas as idades	1,8 (1,7;1,9)	1 (0,9;1,1)	-45,3 (-51,7;-37,8)	116 (99,4;137)	70,9 (54,3;92,9)	-38,9 (-46,7;-31,5)
Abaixo de 5	0,3 (0,2;0,5)	0 (0;0,1)	-89,2 (-94,4;-77,5)	32,3 (20,7;47,8)	5,8 (3,9;8,5)	-82,1 (-90,2;-68)
**B.2.5.1-Doença valvar aórtica** **calcífica não reumática**						
Ambos						
15-49 anos	0,4 (0,4;0,5)	0,2 (0,2;0,3)	-49,3 (-55,6;-38,9)	23,1 (20,5;26,7)	11,2 (9,6;12,7)	-51,5 (-57,4;-41,8)
50-69 anos	3,5 (3,1;3,8)	2,3 (2,1;2,7)	-32,7 (-39,1;-23)	102 (90;111,9)	67 (60,3;78,1)	-34,3 (-40,4;-25,1)
70+ anos	14,8 (12,7;16)	17,3 (14,4;20,2)	17 (2;38,5)	209,2 (179,7;226,3)	216,7 (184,1;251,7)	3,6 (-9,5;22,6)
Padronizada por idade	1,8 (1,6;2)	1,5 (1,3;1,8)	-15,6 (-24;-4)	41,9 (37;45,9)	29,2 (26,3;33,4)	-30,3 (-36,3;-21,3)
Todas as idades	1 (0,9;1,1)	1,6 (1,4;1,8)	58 (42,2;78,7)	28,6 (25,4;31,8)	31,5 (28,4;36,2)	10,3 (0,7;25)
Feminino						
15-49 anos	0,3 (0,2;0,4)	0,2 (0,1;0,2)	-42,2 (-55,2;-23,5)	14,5 (10,9;19,6)	8 (5,7;10,7)	-44,7 (-57,3;-26,8)
50-69 anos	2,3 (1,8;2,8)	1,8 (1,5;2,3)	-23,2 (-32,8;-7,1)	66 (51,1;81,1)	49,4 (40,9;64,3)	-25,1 (-34,2;-9,7)
70+ anos	14,6 (11,9;16,2)	17,3 (14;21,1)	18,3 (0,1;45,7)	194,7 (158;215,7)	203 (167;248,8)	4,2 (-12,2;29,4)
Padronizada por idade	1,5 (1,3;1,7)	1,4 (1,1;1,7)	-10,9 (-21,9;7,4)	30,8 (24,5;36,6)	23,8 (20,3;29,5)	-22,8 (-31,2;-6,5)
Todas as idades	0,8 (0,7;1)	1,6 (1,3;2)	89,3 (65,8;129,5)	20,7 (16,3;25,4)	27,6 (23,6;34,3)	33,2 (18,6;62,1)
Masculino						
15-49 anos	0,6 (0,5;0,7)	0,3 (0,2;0,3)	-52,8 (-58,3;-43,6)	32,1 (28,3;35,7)	14,5 (12,4;17,1)	-54,7 (-60,2;-46)
50-69 anos	4,8 (4,3;5,2)	3 (2,7;3,4)	-37,1 (-44;-29,1)	141 (126,3;151,6)	86,8 (77,9;97,5)	-38,4 (-45,1;-30,9)
70+ anos	15,1 (12,9;16,3)	17,4 (14,9;19,8)	15,5 (0,7;34,7)	227,2 (195,3;246,1)	235,4 (203,9;267,6)	3,6 (-9,8;20,7)
Padronizada por idade	2,1 (1,9;2,3)	1,7 (1,5;2)	-18,1 (-27;-8,8)	53,6 (47,6;57,5)	35,3 (31,7;40)	-34,1 (-40,3;-26,6)
Todas as idades	1,2 (1;1,3)	1,6 (1,4;1,8)	35,2 (21,4;50,8)	36,7 (32,8;39,8)	35,7 (32,2;40,4)	-2,7 (-11,7;8,4)
**B.2.5.2-Doença valvar mitral** **degenerativa não reumática**						
Ambos						
15-49 anos	0,3 (0,2;0,4)	0,2 (0,2;0,2)	-42,1 (-50,5;-30,1)	17,1 (13,2;19,4)	9,4 (8;11,3)	-45 (-52,9;-33,2)
50-69 anos	1,6 (1,3;2)	1,2 (0,9;1,4)	-23,6 (-37,4;-13,3)	48,2 (38,8;58,8)	36 (27,5;41,3)	-25,4 (-38,6;-15,6)
70+ anos	4 (3,3;5,7)	4,6 (2,9;5,5)	16,5 (-18,5;48,7)	59 (49,4;84)	64,3 (40,8;75,7)	9 (-23,4;39,2)
Padronizada por idade	0,7 (0,6;0,9)	0,6 (0,4;0,7)	-19 (-34,8;-5,8)	20,4 (16,3;24,6)	14,1 (10,8;16,3)	-30,7 (-41,6;-22,6)
Todas as idades	0,4 (0,4;0,6)	0,6 (0,4;0,7)	36,2 (11,4;56,6)	15,6 (12,4;18,4)	15,6 (11,9;18)	0,2 (-13,5;11,1)
Feminino						
15-49 anos	0,4 (0,3;0,5)	0,2 (0,2;0,3)	-46,6 (-56,6;-32,4)	21,5 (15,4;25)	10,9 (8,8;13,7)	-49,3 (-58,5;-35,8)
50-69 anos	2 (1,5;2,5)	1,4 (1;1,7)	-28,5 (-43,6;-17,8)	59,8 (44,2;75,6)	41,8 (28,8;51)	-30,1 (-44,9;-19,8)
70+ anos	4,3 (3,2;6,7)	5 (2,6;6,3)	15,1 (-26,4;55,5)	63,8 (48,6;98,2)	68 (36,8;85)	6,7 (-31,3;43,3)
Padronizada por idade	0,8 (0,6;1,1)	0,6 (0,4;0,8)	-24,4 (-43,5;-10)	24,9 (18,5;31,3)	15,9 (11,1;19,4)	-36,1 (-48,5;-27,9)
Todas as idades	0,6 (0,4;0,7)	0,7 (0,5;0,9)	30,4 (-1,6;52,8)	19,6 (14,5;24)	18,5 (12,9;22,4)	-5,8 (-23,4;5,7)
Masculino						
15-49 anos	0,2 (0,2;0,3)	0,2 (0,1;0,2)	-33,9 (-45,9;-17,2)	12,5 (8,9;15,6)	7,9 (5,9;10,1)	-37,3 (-48,4;-19,9)
50-69 anos	1,2 (0,9;1,6)	1 (0,7;1,2)	-15,6 (-30,5;0,7)	35,7 (27,1;47,2)	29,4 (19,6;35,8)	-17,5 (-31,5;-1,8)
70+ anos	3,5 (2,8;5,2)	4,2 (2,6;5)	17,6 (-18;45,5)	53,1 (42,5;78)	59,2 (37,3;71,1)	11,5 (-22,3;39)
Padronizada por idade	0,6 (0,4;0,7)	0,5 (0,3;0,6)	-11 (-28,8;4,2)	15,5 (11,7;20,1)	12,1 (8,6;14,5)	-22 (-33,3;-10,2)
Todas as idades	0,3 (0,2;0,4)	0,5 (0,3;0,6)	45,4 (19,8;69,1)	11,4 (8,4;14,6)	12,6 (8,8;15,2)	10,2 (-5,1;26,4)
**B.2.5-Doença valvar do** **coração não reumática**						
Ambos						
15-49 anos	0,8 (0,7;0,8)	0,4 (0,4;0,4)	-45,5 (-50;-40,3)	40,8 (39,1;42,5)	21,2 (19,8;22,7)	-47,9 (-52;-42,9)
50-69 anos	5,1 (4,9;5,4)	3,6 (3,4;3,9)	-29,4 (-34,7;-24,3)	151,3 (145,2;158,3)	104,4 (97,1;111,5)	-31 (-36;-25,9)
70+ anos	18,9 (17,3;20,1)	22,2 (18,8;24,3)	17,2 (5,4;27,4)	270,2 (250,3;285,3)	283,7 (247;309,3)	5 (-5,3;13,8)
Padronizada por idade	2,5 (2,4;2,7)	2,1 (1,9;2,3)	-16,2 (-22,5;-10,3)	62,8 (60,3;65,2)	44 (40,7;47)	-30 (-34,5;-25)
Todas as idades	1,5 (1,4;1,5)	2,2 (2;2,4)	51,9 (39,8;62,7)	44,6 (43,1;46,2)	47,9 (44,4;51,1)	7,3 (0;15,1)
Feminino						
15-49 anos	0,7 (0,6;0,7)	0,4 (0,3;0,4)	-43,9 (-49,8;-36,2)	36,5 (34,5;38,7)	19,5 (17,7;21,4)	-46,5 (-52;-39,4)
50-69 anos	4,3 (4;4,6)	3,2 (2,9;3,5)	-25,1 (-32,7;-16,5)	126,9 (119,5;134,6)	92,7 (84,7;101,1)	-26,9 (-34,1;-18,9)
70+ anos	19 (17,1;20,5)	22,5 (18,7;25,3)	17,9 (4,6;31,3)	260,1 (235,5;279,4)	273,4 (233;304,6)	5,1 (-6;17,2)
Padronizada por idade	2,4 (2,2;2,5)	2 (1,8;2,2)	-15,2 (-23,2;-7,1)	56,2 (53,1;59)	40,3 (36,6;43,8)	-28,2 (-34,5;-21,2)
Todas as idades	1,4 (1,3;1,5)	2,4 (2,1;2,6)	66,5 (49,8;83)	40,7 (38,8;42,8)	46,8 (42,5;50,9)	14,9 (4,5;26,7)
Masculino						
15-49 anos	0,8 (0,8;0,9)	0,4 (0,4;0,5)	-46,9 (-52,1;-40,8)	45,2 (42,7;48,1)	23 (21,1;25,1)	-49,1 (-54,1;-43,3)
50-69 anos	6 (5,7;6,4)	4,1 (3,7;4,4)	-32,5 (-38,7;-25,7)	177,9 (168,9;188,4)	117,6 (108;127,5)	-33,9 (-39,8;-27,2)
70+ anos	18,8 (17,2;20)	21,8 (19;23,8)	16,1 (2,6;27,5)	282,7 (260,4;301,7)	297,6 (262,9;325)	5,3 (-6,1;16)
Padronizada por idade	2,7 (2,5;2,8)	2,3 (2;2,4)	-16,4 (-23,9;-9,5)	69,8 (66,7;73,1)	48,1 (44,5;51,7)	-31 (-36,5;-25)
Todas as idades	1,5 (1,5;1,6)	2,1 (1,9;2,3)	38 (25,8;50,3)	48,6 (46,5;51,1)	49 (45,4;52,7)	0,9 (-7,3;9,9)

*Fonte: Dados derivados do estudo Global Burden of Disease 2019, Institute for Health Metrics and Evaluation, University of Washington. ^46^*

**Tabela 5-3:** – Número de mortes, taxas de mortalidade padronizadas por idade (por 100 mil) em 1990 e 2019 e variação percentual das taxas, por grupo cardiovascular de causa de morte, no Brasil e suas unidades federativas.

Causa de morte e localização	1990		2019		Variação percentual das taxas (II 95%)
	Número (II 95%)	Taxa (II 95%)	Número (II 95%)	Taxa (II 95%)	
**B.2.1-Doença cardíaca reumática **					(;)
Acre	6,4 (5,3;7,6)	2,7 (2,3;3,1)	9 (7,3;10,8)	1,3 (1;1,5)	-52,1(-63,3;-39,3)
Alagoas	50,6 (40,4;65,3)	2,8 (2,2;3,6)	43,4 (35,3;53,2)	1,3 (1;1,6)	-54,9(-68,5;-37)
Amapá	1,9 (1,5;2,2)	1,3 (1,1;1,4)	4,7 (3,8;5,7)	0,7 (0,6;0,9)	-41,1(-53,3;-24,9)
Amazonas	18,5 (15,8;21,2)	1,6 (1,4;1,9)	23,6 (19,4;28,7)	0,7 (0,6;0,9)	-56,2(-65,2;-44,6)
Bahia	243,8 (210,7;286,5)	2,7 (2,3;3,2)	208,7 (169,5;258,2)	1,3 (1;1,6)	-53,5(-65,1;-39,3)
Brasil	3088,2 (2938,9;3256)	2,8 (2,7;3)	2715,2 (2505,1;2913,1)	1,2 (1,1;1,2)	-59,4(-63,1;-55,4)
Ceará	78,8 (64,9;94,7)	1,6 (1,3;1,9)	81,7 (67,6;98,7)	0,8 (0,7;1)	-49(-60;-35,2)
Distrito Federal	43,1 (37,3;50,1)	5,1 (4,4;5,9)	40,7 (32,8;49,6)	1,6 (1,3;1,9)	-68,5(-75,1;-60,2)
Espírito Santo	47,6 (42,9;52,4)	2,6 (2,3;2,9)	56,1 (45,2;68,8)	1,3 (1;1,6)	-50,4(-60,3;-38,2)
Goiás	112,9 (95,8;136,2)	4,1 (3,4;4,9)	114,7 (92,7;139,8)	1,6 (1,3;1,9)	-60,6(-69,3;-50,6)
Maranhão	78,1 (60,4;101,6)	2,2 (1,7;2,9)	69,1 (55,6;84,5)	1 (0,8;1,2)	-56(-69,1;-37)
Mato Grosso	25,9 (20,9;31,2)	2,2 (1,9;2,6)	33 (26,5;39,8)	0,9 (0,8;1,1)	-57,5(-66,7;-45)
Mato Grosso do Sul	28,5 (25,2;32,3)	2,4 (2,1;2,7)	29 (23,8;34,8)	1 (0,8;1,2)	-59,7(-67,5;-50,6)
Minas Gerais	419,6 (379,7;465,1)	3,5 (3,2;3,9)	336,6 (280,4;394,5)	1,3 (1,1;1,5)	-63,1(-69,9;-55,1)
Pará	58 (49,7;68)	2 (1,7;2,3)	65,4 (55,3;77)	0,8 (0,7;1)	-57,8(-66;-47,2)
Paraíba	60,7 (49,1;76,6)	2,4 (1,9;3)	50,3 (40,1;62)	1,1 (0,8;1,3)	-54,9(-68,1;-37,8)
Paraná	197,9 (178,1;217,7)	3,3 (3;3,7)	192,9 (161,2;227,3)	1,5 (1,2;1,7)	-56,2(-63,6;-47,6)
Pernambuco	149,6 (128,8;175,6)	2,7 (2,3;3,2)	148,4 (122,5;179,2)	1,4 (1,2;1,7)	-46,4(-58,4;-33,3)
Piauí	31,3 (26,1;36,7)	1,8 (1,5;2,1)	28,4 (23,5;33,8)	0,8 (0,6;0,9)	-58,3(-67;-47,1)
Rio de Janeiro	295,5 (273,1;320,9)	2,7 (2,5;2,9)	232,3 (193,1;276,8)	1,1 (0,9;1,3)	-59,6(-66,7;-50,8)
Rio Grande do Norte	40,1 (34;46,9)	2,1 (1,8;2,5)	42,3 (33,7;52,1)	1,1 (0,9;1,3)	-49,9(-61,8;-36,2)
Rio Grande do Sul	149,9 (135,7;165,7)	2 (1,8;2,2)	126 (104,6;150)	0,9 (0,7;1)	-58,3(-65,3;-49,8)
Rondônia	13,9 (10,7;16,9)	2,7 (2,3;3,2)	17,7 (14,6;21,1)	1,1 (0,9;1,3)	-60,7(-68,6;-50,3)
Roraima	1,7 (1,3;2)	1,8 (1,6;2,1)	3,5 (2,9;4,3)	0,8 (0,7;1)	-53,1(-62;-41,8)
Santa Catarina	79,4 (70,8;89,1)	2,6 (2,3;2,9)	82,7 (67,8;99,3)	1 (0,9;1,2)	-60,5(-67,8;-51,4)
São Paulo	815,4 (739,4;897,2)	3,5 (3,1;3,9)	629,3 (531,7;737,8)	1,2 (1;1,4)	-65,9(-71,7;-59)
Sergipe	26,5 (22,1;31,4)	2,7 (2,2;3,2)	30,1 (24,1;37,3)	1,3 (1;1,6)	-53(-64,1;-38,5)
Tocantins	12,6 (10,1;15,4)	2,4 (2;2,9)	15,9 (12,6;19,7)	1,1 (0,8;1,3)	-56,4(-67,1;-41,3)
**B.2.5-Doença valvar do coração não reumática**					(;)
Acre	2,7 (2,4;3,1)	1,8 (1,6;2,1)	10,2 (8,9;11,6)	1,8 (1,6;2,1)	-1,6(-17,9;18,5)
Alagoas	23 (20;26,8)	1,8 (1,5;2,1)	61,5 (52,3;70,5)	2 (1,7;2,2)	9,3(-12,2;33,3)
Amapá	2 (1,8;2,2)	2,1 (1,9;2,4)	9,7 (8,6;10,8)	2 (1,7;2,2)	-7,2(-18,6;6,7)
Amazonas	17,1 (15,5;19)	2,3 (2,1;2,6)	46,7 (40,1;53,4)	1,7 (1,4;1,9)	-27,7(-37,5;-16,4)
Bahia	143,8 (124,5;162,7)	2,2 (1,9;2,5)	275,3 (228,2;324,4)	1,7 (1,4;2)	-22(-37,2;-4)
Brasil	2189,8 (2092,3;2275,8)	2,5 (2,4;2,7)	4842,8 (4326;5225,5)	2,1 (1,9;2,3)	-16,2(-22,5;-10,3)
Ceará	48,5 (39,4;59,8)	1,2 (1;1,5)	158 (130,5;188,1)	1,6 (1,3;1,9)	33,8(1,7;75,5)
Distrito Federal	19,6 (17,5;22)	4 (3,5;4,5)	47,9 (41,1;54,8)	2,5 (2,1;2,9)	-38,1(-47,5;-27,5)
Espírito Santo	41,1 (38;44,3)	3 (2,8;3,2)	109,3 (92,8;124,7)	2,7 (2,2;3)	-11,7(-23,5;0,9)
Goiás	62,7 (54,1;74,3)	3,2 (2,8;3,7)	138,3 (114,9;164,7)	2,1 (1,7;2,5)	-34,2(-45,8;-20,5)
Maranhão	29,8 (22,6;39,3)	1,2 (0,9;1,6)	97,4 (82,5;114,8)	1,5 (1,3;1,8)	24,4(-11,1;71,3)
Mato Grosso	18,4 (15,6;21)	2,4 (2,1;2,7)	56,2 (48,7;64)	1,8 (1,6;2,1)	-24,3(-36,1;-9,3)
Mato Grosso do Sul	23,7 (21,7;25,8)	2,8 (2,6;3,1)	56,5 (48,7;65,2)	2 (1,7;2,3)	-28,4(-38,2;-16,9)
Minas Gerais	256,2 (235,7;280,3)	2,7 (2,5;3)	511,4 (442,8;577,5)	2 (1,7;2,2)	-28,2(-37,5;-18,6)
Pará	44,3 (39;50)	2,2 (2;2,5)	110 (95,1;124,9)	1,6 (1,4;1,8)	-27,9(-39,5;-14,1)
Paraíba	25,9 (21,9;31,3)	1,2 (1;1,4)	57 (48,1;66,6)	1,2 (1;1,4)	1,2(-21,4;26,7)
Paraná	152 (143,6;161,2)	3,4 (3,1;3,6)	350,6 (301,7;397,4)	2,8 (2,4;3,2)	-16,2(-27,2;-5,2)
Pernambuco	97,7 (89,1;106,5)	2,3 (2;2,5)	217,6 (189,9;246,3)	2,3 (2;2,6)	-0,4(-14,9;16)
Piauí	18,3 (16;21,3)	1,5 (1,2;1,7)	46,5 (38,9;53,4)	1,2 (1;1,4)	-17,1(-32,8;1,3)
Rio de Janeiro	218,6 (205,1;230,2)	2,4 (2,2;2,5)	418,7 (367;475,5)	1,9 (1,7;2,2)	-19,8(-29,3;-8,9)
Rio Grande do Norte	23,2 (19,7;27)	1,5 (1,2;1,7)	59,8 (49,2;71,6)	1,5 (1,2;1,8)	2,1(-19,5;28,7)
Rio Grande do Sul	189,2 (176,5;200,4)	3,1 (2,8;3,3)	447 (383,4;510,1)	2,9 (2,5;3,3)	-5,1(-17,6;8,1)
Rondônia	8,4 (7;9,6)	2,9 (2,6;3,3)	25,1 (21,4;29,2)	1,8 (1,5;2)	-39,9(-49,5;-28,5)
Roraima	1,2 (1;1,3)	2,3 (2,1;2,6)	5,3 (4,7;6)	1,8 (1,5;2)	-24,8(-34,5;-13,2)
Santa Catarina	84,9 (78,8;91,3)	3,7 (3,4;4)	216,9 (187,3;247,6)	3 (2,5;3,4)	-20,8(-31,6;-9,4)
São Paulo	619,5 (580,7;656,9)	3,2 (3;3,4)	1251,4 (1078,1;1408,6)	2,4 (2,1;2,8)	-23,4(-33,1;-14,5)
Sergipe	11,2 (9,9;12,6)	1,6 (1,4;1,8)	29,3 (24,8;34,2)	1,3 (1,1;1,6)	-15,7(-29,9;2,7)
Tocantins	6,9 (5,8;8,2)	2,2 (1,8;2,6)	29,4 (24,7;34,1)	2,2 (1,8;2,5)	-2,8(-22,3;22)
**B.2.5.1-Doença valvar** **aórtica calcífica não reumática**					(;)
Acre	1,8 (1,4;2,1)	1,3 (1;1,6)	7 (6;8,1)	1,3 (1,1;1,5)	-2,1(-20,8;27)
Alagoas	14 (11;16,6)	1,1 (0,9;1,4)	40,3 (33,9;49,1)	1,3 (1,1;1,6)	13,9(-9,6;53,2)
Amapá	1,4 (1,2;1,5)	1,6 (1,3;1,8)	6,4 (5,6;7,6)	1,4 (1,2;1,6)	-11(-24,5;7,9)
Amazonas	12,2 (9,9;13,8)	1,8 (1,4;2)	33,7 (28,5;39,4)	1,2 (1;1,5)	-29,2(-40,3;-14,3)
Bahia	102,5 (82;119,3)	1,6 (1,3;1,9)	190,6 (155,8;234)	1,2 (1;1,4)	-26,6(-42,6;-2,3)
Brasil	1507,5 (1326;1636,7)	1,8 (1,6;2)	3467,3 (3000,8;4002,6)	1,5 (1,3;1,8)	-15,6(-24;-4)
Ceará	29,4 (21,7;37,7)	0,7 (0,6;1)	103,9 (84,3;130,5)	1,1 (0,9;1,3)	42,1(5,4;107)
Distrito Federal	12,7 (11;15,4)	3 (2,5;3,4)	32,7 (27,3;40)	1,8 (1,5;2,2)	-38,4(-48,2;-26,4)
Espírito Santo	26,7 (23,8;30,6)	2,1 (1,8;2,4)	75,4 (62,3;92,7)	1,9 (1,5;2,3)	-10,8(-24,5;4,8)
Goiás	42,8 (36,5;52,2)	2,3 (2;2,8)	97,5 (79,5;121,2)	1,5 (1,2;1,9)	-35,4(-46,9;-21,7)
Maranhão	19,2 (13,4;26,8)	0,8 (0,6;1,1)	67,3 (53,9;81,9)	1 (0,8;1,3)	30,3(-9,6;85,7)
Mato Grosso	12,5 (10,3;14,3)	1,8 (1,5;2)	38,3 (32,6;45,5)	1,3 (1,1;1,5)	-27,6(-40,7;-10,1)
Mato Grosso do Sul	16,3 (14,2;18,4)	2,1 (1,8;2,3)	39,7 (33,7;47,3)	1,5 (1,2;1,7)	-30,3(-40,8;-17,4)
Minas Gerais	177,8 (159,3;198,2)	2 (1,7;2,2)	370,4 (313;436,4)	1,4 (1,2;1,7)	-28,2(-38,2;-17,2)
Pará	28,5 (23,9;33,1)	1,5 (1,3;1,8)	70,5 (58,8;85,4)	1,1 (0,9;1,3)	-31,4(-44;-14,3)
Paraíba	16,8 (12,2;20,8)	0,8 (0,6;0,9)	39,5 (32,4;46,8)	0,8 (0,7;0,9)	5,5(-18,6;41,8)
Paraná	104,6 (91,7;117,3)	2,5 (2,1;2,7)	257,8 (215,7;308,6)	2,1 (1,8;2,5)	-14,4(-26,4;-1,4)
Pernambuco	60,3 (52,8;68,1)	1,5 (1,3;1,6)	143,8 (120;178,2)	1,5 (1,3;1,9)	3,4(-14,6;27,3)
Piauí	11,3 (9,1;13,6)	1 (0,8;1,2)	30,3 (24,7;37,8)	0,8 (0,6;1)	-17,5(-35,8;4,5)
Rio de Janeiro	143,6 (120,7;157,7)	1,7 (1,4;1,8)	301,5 (259,8;349)	1,4 (1,2;1,6)	-15,9(-27,3;-1,2)
Rio Grande do Norte	16,1 (12,4;19,2)	1 (0,8;1,3)	42,1 (34;51,4)	1,1 (0,9;1,3)	1,6(-21,9;37)
Rio Grande do Sul	134,9 (116,1;149,5)	2,3 (1,9;2,5)	343 (283,6;408)	2,3 (1,9;2,7)	-1,7(-15,5;15,7)
Rondônia	5,6 (4,6;6,5)	2,2 (1,9;2,5)	17,5 (14,7;20,6)	1,3 (1,1;1,5)	-42,8(-52,4;-29,8)
Roraima	0,9 (0,6;1)	1,9 (1,5;2,1)	3,9 (3,3;4,5)	1,4 (1,2;1,6)	-25,9(-36,6;-11,7)
Santa Catarina	61 (53,1;69)	2,8 (2,4;3,2)	166,5 (137;196,3)	2,3 (1,9;2,7)	-18,6(-30,6;-6,3)
São Paulo	443,5 (387,3;491,7)	2,4 (2,1;2,6)	910,2 (757,6;1083,5)	1,8 (1,5;2,1)	-24,2(-35,1;-10,3)
Sergipe	6,8 (5,2;8)	1 (0,8;1,2)	18,4 (15,1;22,4)	0,8 (0,7;1)	-16,9(-33,6;7,9)
Tocantins	4,1 (3,3;4,9)	1,5 (1,2;1,8)	18,9 (14,9;24,7)	1,4 (1,1;1,8)	-3,8(-25,5;25,5)
** B.2.5.2-Doença valvar mitral degenerativa não reumática **					(;)
Acre	0,8 (0,7;1,1)	0,5 (0,4;0,7)	2,9 (2,3;3,9)	0,5 (0,4;0,6)	-2,8(-22,4;27,2)
Alagoas	8,7 (6,7;10,9)	0,6 (0,5;0,8)	20,2 (15,5;25)	0,6 (0,5;0,8)	0,3(-25,3;38)
Amapá	0,6 (0,5;0,8)	0,6 (0,5;0,7)	3 (2,2;3,6)	0,6 (0,4;0,7)	0,4(-18,2;21,6)
Amazonas	4,8 (3,9;6,6)	0,5 (0,4;0,8)	12,2 (9,8;16)	0,4 (0,3;0,5)	-24,5(-40,7;-5,6)
Bahia	40 (31,9;52,4)	0,6 (0,4;0,7)	81,1 (61,1;101,9)	0,5 (0,4;0,6)	-10(-33,1;20,8)
Brasil	663,1 (535,3;818,9)	0,7 (0,6;0,9)	1315,1 (943,8;1507)	0,6 (0,4;0,7)	-19(-34,8;-5,8)
Ceará	17,9 (12,6;23,3)	0,4 (0,3;0,6)	50,6 (36,3;63,9)	0,5 (0,4;0,6)	19,3(-13,5;64,8)
Distrito Federal	6,7 (5;8,1)	1 (0,7;1,4)	14,4 (9,8;17,7)	0,6 (0,4;0,8)	-39(-57,4;-24)
Espírito Santo	14 (9,6;16,4)	0,9 (0,6;1)	32,3 (20,7;40,6)	0,8 (0,5;1)	-15,2(-30;2,6)
Goiás	19,3 (14,4;27,3)	0,8 (0,6;1,2)	38,9 (28,3;50,8)	0,6 (0,4;0,7)	-32,3(-50,2;-13,3)
Maranhão	9,8 (6,6;12,9)	0,4 (0,2;0,5)	27,6 (21,3;39,4)	0,4 (0,3;0,6)	11,3(-23,4;59,6)
Mato Grosso	5,7 (4,4;7,2)	0,6 (0,5;0,8)	17 (12,9;20,5)	0,5 (0,4;0,6)	-16,6(-33,9;4,6)
Mato Grosso do Sul	7,3 (5,6;8,8)	0,7 (0,6;0,9)	16,3 (11,7;19,9)	0,6 (0,4;0,7)	-23,9(-39,7;-6,4)
Minas Gerais	76,3 (63;100,7)	0,7 (0,6;1)	135 (99,2;162,8)	0,5 (0,4;0,6)	-29,4(-48,9;-13)
Pará	15,1 (11,8;18,4)	0,7 (0,5;0,8)	36,2 (26,8;44,4)	0,5 (0,4;0,6)	-22,9(-39;-3,7)
Paraíba	8,5 (6,1;11,5)	0,4 (0,3;0,5)	16 (12,3;23)	0,3 (0,3;0,5)	-8,3(-33,3;25,8)
Paraná	46,1 (33,9;56,6)	0,9 (0,6;1,1)	88,4 (56,8;110,5)	0,7 (0,4;0,9)	-22,7(-40,5;-4,3)
Pernambuco	36,4 (27,1;41,8)	0,8 (0,6;0,9)	70,7 (47,2;85,4)	0,7 (0,5;0,9)	-8,6(-27;13,4)
Piauí	6,7 (5,1;8)	0,5 (0,4;0,6)	15,1 (11,2;19)	0,4 (0,3;0,5)	-17,5(-34,4;3,1)
Rio de Janeiro	73,5 (62,9;94,3)	0,7 (0,6;1)	113,6 (88,7;145,7)	0,5 (0,4;0,7)	-29,3(-42,5;-16,2)
Rio Grande do Norte	6,7 (5,2;9,2)	0,4 (0,3;0,6)	16,2 (12,3;21)	0,4 (0,3;0,5)	1,1(-24,9;35,4)
Rio Grande do Sul	52,8 (39;64,3)	0,8 (0,6;1)	99,5 (60,4;126,5)	0,6 (0,4;0,8)	-16,5(-35,5;3,2)
Rondônia	2,7 (2,1;3,5)	0,7 (0,6;1)	7,2 (5,7;9,4)	0,5 (0,4;0,6)	-32,7(-51;-16)
Roraima	0,3 (0,2;0,5)	0,4 (0,3;0,8)	1,1 (0,8;2,1)	0,3 (0,2;0,6)	-27(-39,1;-11,7)
Santa Catarina	23,4 (16;30,1)	0,9 (0,6;1,2)	48,7 (30,4;61,4)	0,6 (0,4;0,8)	-28,5(-44,4;-12)
São Paulo	172,3 (133,2;223,6)	0,8 (0,6;1,1)	330,3 (211,8;399,1)	0,6 (0,4;0,8)	-21,7(-44,6;-2,1)
Sergipe	4,2 (3,3;5,4)	0,5 (0,4;0,7)	10,4 (7,6;13,1)	0,5 (0,3;0,6)	-14,7(-33,1;13)
Tocantins	2,7 (1,8;3,4)	0,7 (0,5;0,9)	9,9 (6,4;12,5)	0,7 (0,4;0,9)	-1,6(-23,1;29,2)

*Fonte: Dados derivados do estudo Global Burden of Disease 2019, Institute for Health Metrics and Evaluation, University of Washington. ^46^*

**Tabela 5-4:** – Número de DALYs, taxas de DALYs padronizadas por idade (por 100 mil) em 1990 e 2019 e variação percentual das taxas, por grupo cardiovascular de causa de morte, no Brasil e suas unidades federativas.

Causa de morte e localização	1990		2019		Variação percentual das taxas (II 95%)
	Número (II 95%)	Taxa (II 95%)	Número (II 95%)	Taxa (II 95%)	
**B.2.1-Doença cardíaca** **reumática **					**(;)**
Acre	478,6 (384,9;587,3)	134,1 (109,9;161,8)	753,3 (566,2;993,1)	82,6 (62,9;106,6)	-38,4(-49,7;-27,2)
Alagoas	3473,1 (2746,4;4365,8)	149,5 (120;186)	3192,6 (2447,9;4092,5)	86,5 (66,7;110,6)	-42,1(-56;-27,5)
Amapá	197,9 (146,9;261,3)	83,6 (64,4;107)	573,8 (413;795,1)	66,6 (49,3;90,8)	-20,4(-31,2;-9,4)
Amazonas	1696,9 (1318,6;2188,4)	95,4 (77,2;119,6)	2745,7 (1929,8;3783,2)	64,7 (46,3;87,8)	-32,1(-43,3;-21,6)
Bahia	16674,5 (13969;19833,8)	148 (124,8;175,3)	14372 (11188,8;18390,2)	86,4 (67,4;111)	-41,6(-52,4;-30,7)
Brasil	197716,1 (170430,6;232611,3)	144,6 (126,8;167,3)	184224,8 (143687,3;238145,9)	79,3 (61,6;102,6)	-45,1(-52,1;-38,2)
Ceará	6153,3 (4892,5;7694,2)	101,7 (81,3;125,6)	7192 (5239,3;9577,4)	69,5 (50,6;92,6)	-31,6(-43,4;-20,6)
Distrito Federal	2733 (2325,3;3194,9)	202,3 (175,2;232,6)	2748,9 (2101,9;3590,4)	85,6 (65,8;109,5)	-57,7(-65,7;-49,1)
Espírito Santo	3228,8 (2710,2;3880,5)	133,5 (113,7;157,1)	3625,4 (2794,7;4689,5)	84,1 (64,7;109)	-37(-47,1;-27,4)
Goiás	6941,8 (5867,3;8245,7)	186,2 (158,6;219,8)	6955 (5375,9;8927,3)	92,7 (71,8;118,7)	-50,2(-59,9;-39,7)
Maranhão	5793,2 (4511,6;7327)	128,3 (100,7;161,8)	5937,4 (4413,8;8026,1)	74 (55,7;99,1)	-42,3(-55,9;-27,7)
Mato Grosso	2061,6 (1608,5;2580,3)	117,3 (95,7;140,7)	2777,9 (2060,8;3705,6)	72,4 (53,8;96,2)	-38,3(-49,5;-26,2)
Mato Grosso do Sul	2052,3 (1689,3;2521,1)	126,6 (106,3;152)	2259,9 (1708,7;2978,9)	74,2 (56,2;98,2)	-41,4(-51,5;-32,2)
Minas Gerais	25212,5 (21921,1;29506,4)	170,8 (149,3;197,7)	19945,3 (15436,6;25464,7)	83 (63,9;107,9)	-51,4(-59,8;-42,7)
Pará	4685,7 (3688,2;5883)	110,3 (89,1;134,6)	6570,4 (4804,8;8902,7)	70,1 (52;93,5)	-36,5(-46,9;-26,3)
Paraíba	3869,2 (3097,1;4891,2)	130,2 (104,2;163,2)	3595,1 (2709,1;4709,2)	79,5 (59,9;104,8)	-39(-51,9;-25,6)
Paraná	12144,3 (10386,1;14396,5)	156,5 (135,8;181,9)	11030,6 (8623;14136,8)	87,1 (67,7;113,1)	-44,3(-53;-35,7)
Pernambuco	9944,8 (8279,9;11968,4)	144,5 (121,6;173,2)	9819,1 (7641,7;12313,8)	92,9 (72,5;116,8)	-35,7(-47,6;-24,6)
Piauí	2500,4 (1977,2;3115,3)	105,7 (85,2;129,1)	2580,9 (1896,6;3474,4)	68,7 (50,3;92,8)	-35(-46,1;-23,5)
Rio de Janeiro	18562 (15876,8;22061,5)	142,9 (123,1;167,2)	15385,3 (11850;19928,5)	78,6 (59,8;103,2)	-45(-53,8;-35,9)
Rio Grande do Norte	2723,6 (2217,2;3328,6)	120,4 (99,1;146,3)	3111,1 (2330,5;4079)	79,7 (59,7;104,4)	-33,8(-45,4;-23,4)
Rio Grande do Sul	10128,1 (8278,3;12485,4)	113,1 (93,9;138,1)	8733,1 (6508,8;11731,9)	69,5 (50,3;94,7)	-38,5(-48,3;-29,6)
Rondônia	1122,1 (847,6;1419,2)	124,4 (99,6;149,5)	1430,6 (1081,9;1885,8)	76,1 (58;99,1)	-38,8(-49,7;-26,2)
Roraima	165,6 (124,9;220,2)	95,7 (76,1;120,8)	398 (288,9;538,5)	67,2 (50,1;89,6)	-29,7(-41;-18,6)
Santa Catarina	5344,1 (4385,8;6550,2)	128,9 (108,3;153,5)	5883,4 (4408,2;7698,3)	74,6 (55,6;98,4)	-42,1(-51,6;-33,2)
São Paulo	47070,4 (40270,9;55070,9)	159,3 (138,6;182,7)	39172,3 (30110,2;51125,7)	77,3 (58,5;101,5)	-51,5(-59,2;-43)
Sergipe	1807,3 (1481,5;2208,5)	135,6 (111,5;164,9)	2176,7 (1655,5;2835)	86,2 (66;111,8)	-36,4(-48,6;-23,1)
Tocantins	950,7 (747,3;1192,2)	120,7 (97,2;147,6)	1258,9 (933;1679,4)	74,7 (55,7;98,9)	-38,1(-49,6;-24,9)
**B.2.5-Doença valvar do coração não reumática **					**(;)**
Acre	85 (73,5;98,7)	40 (34,9;45,5)	260,4 (229,9;292,8)	37,9 (33,4;42,6)	-5,4(-22,2;15)
Alagoas	663,4 (578,5;772,7)	42,2 (36,8;49,3)	1538,5 (1313,5;1768,2)	45,8 (39,1;52,5)	8,5(-12,8;33,5)
Amapá	64,3 (55,6;72)	47,3 (42;52,1)	262,1 (234,9;291,7)	43,2 (38,4;47,9)	-8,7(-20,5;5,9)
Amazonas	542,6 (482,1;605,8)	52,2 (47,2;57,7)	1191,1 (1029,4;1356,5)	36,9 (31,9;42,1)	-29,2(-38,9;-17,1)
Bahia	4048,8 (3511,8;4582)	51,8 (45,1;58,6)	6278,5 (5226,1;7459,1)	38,3 (31,9;45,4)	-26,1(-41;-8,1)
Brasil	66419,8 (64127,5;68749,3)	62,8 (60,3;65,2)	103773,8 (96149,3;110720)	44 (40,7;47)	-30(-34,5;-25)
Ceará	1261,9 (1037,7;1575,1)	28 (23;34,8)	3539,9 (2933,6;4257,3)	35 (29;42)	25,3(-5,1;65,1)
Distrito Federal	704 (622,6;794,5)	84,4 (74,9;94,3)	1148,6 (990,1;1325,1)	44,2 (38,2;50,5)	-47,7(-55,2;-38,2)
Espírito Santo	1268,1 (1174,9;1371,7)	70,7 (65,4;75,9)	2457,5 (2119,5;2803,4)	56,2 (48,5;64)	-20,5(-31,2;-8,9)
Goiás	2042 (1745,9;2416,1)	76 (65,4;90,2)	3337,7 (2797;3975,1)	46,2 (38,6;54,9)	-39,2(-50,8;-25,1)
Maranhão	893,7 (677,8;1170,3)	30,1 (22,7;39,8)	2260,7 (1897,2;2699,7)	32,7 (27,5;39,1)	8,6(-22,6;51,1)
Mato Grosso	619 (510,5;721,6)	55,9 (47,8;63,7)	1407,6 (1228,7;1606,9)	40,1 (35,1;45,8)	-28,1(-40,1;-12,5)
Mato Grosso do Sul	755 (693,1;822,6)	66,2 (60,9;72,1)	1317,8 (1132,8;1518,3)	43,9 (37,7;50,6)	-33,8(-43,3;-23)
Minas Gerais	8073,1 (7457,7;8872)	68,9 (63,6;75,5)	10888,8 (9597;12210,7)	41,6 (36,7;46,7)	-39,5(-47,1;-31,2)
Pará	1375,1 (1191,3;1561)	51 (44,7;57,4)	2787,7 (2449,2;3195,9)	36,5 (32;41,7)	-28,4(-40,7;-14,4)
Paraíba	643,9 (553,1;788,6)	26,7 (22,9;32,7)	1264,3 (1090,6;1484,8)	26,9 (23,2;31,5)	0,9(-22,1;27)
Paraná	4629,9 (4386,7;4909,4)	79,1 (75;83,9)	7300 (6383,8;8258,8)	55,3 (48,3;62,5)	-30(-39;-20,5)
Pernambuco	2816,5 (2575,4;3061,6)	54,9 (50,3;59,6)	5002 (4359,4;5687,4)	49,1 (42,7;55,6)	-10,6(-24,1;5,1)
Piauí	503,5 (441,6;578)	31,9 (27,9;36,9)	1047,2 (909,9;1190,8)	27,7 (24,1;31,5)	-13,2(-28,9;6)
Rio de Janeiro	6715,9 (6311,1;7069,2)	60,6 (57;63,8)	8742,8 (7701,7;9962,7)	39,9 (35,2;45,2)	-34,2(-42,3;-24,9)
Rio Grande do Norte	596,3 (510,5;694,2)	34,2 (29,3;39,8)	1331,5 (1102,2;1581,2)	33,9 (28,1;40,3)	-0,8(-23;25,9)
Rio Grande do Sul	5430,1 (5055,9;5757,8)	73,2 (68,5;77,5)	8355,5 (7203,2;9441,7)	55,1 (47,6;62,2)	-24,7(-33,8;-14,7)
Rondônia	307,5 (249,9;356,2)	61,6 (53,6;69,4)	627,6 (533,9;731,3)	38,1 (32,6;44,2)	-38,1(-48,8;-24,8)
Roraima	42,8 (36;48,6)	48,3 (43,2;53,2)	139,9 (122,9;158,7)	33,9 (29,9;38,5)	-29,8(-39,2;-18,5)
Santa Catarina	2458,1 (2272,3;2657,3)	81,9 (75,9;88,1)	4423,2 (3874,6;5006,2)	55,2 (48,5;62,4)	-32,5(-41,1;-22,9)
São Paulo	19362,4 (18240;20529,6)	79,7 (74,8;84,5)	25466,7 (22329,9;28597,2)	47,4 (41,5;53,1)	-40,6(-47,7;-33,3)
Sergipe	305,9 (269,7;348,4)	34,1 (30,1;38,4)	705,3 (592,8;831,7)	30 (25,3;35,2)	-12(-28,6;7,7)
Tocantins	211,1 (176,1;249,7)	44,4 (37,2;52,3)	691 (578,1;805,1)	46,1 (38,7;53,5)	3,8(-17,6;31)
** B.2.5.1-Doença valvar aórtica calcífica não reumática **					**(;)**
Acre	51,9 (39;62)	26,5 (20,1;30,9)	163,7 (139;188,2)	25 (21,3;28,7)	-5,7(-24,3;23,4)
Alagoas	363,6 (285,3;425,5)	24,4 (19,2;28,7)	917,3 (762,5;1134,3)	27,8 (23,2;34,4)	14,3(-9,7;53,2)
Amapá	39,3 (32,3;44,9)	31,5 (26,4;35,4)	158,8 (137,4;187,6)	27,7 (24;32,5)	-11,9(-25,8;6,8)
Amazonas	359,5 (291,9;408,9)	36,9 (29,8;41,7)	795,3 (671,5;926,9)	25,5 (21,5;29,8)	-30,7(-41,3;-15,5)
Bahia	2641,7 (2109,8;3085,6)	35,1 (28,2;40,9)	3989,3 (3247;4860,7)	24,5 (19,9;29,9)	-30,3(-45,8;-6,7)
Brasil	42579 (37870,3;47363,9)	41,9 (37;45,9)	68342,2 (61550;78453,3)	29,2 (26,3;33,4)	-30,3(-36,3;-21,3)
Ceará	681,1 (502,2;869,2)	15,6 (11,6;20,1)	2097,8 (1692,1;2650,6)	20,9 (16,9;26,5)	33,7(-1,7;94,9)
Distrito Federal	427,6 (364;525,3)	57 (49,2;67,7)	727,9 (616;894,4)	29,6 (25;36,1)	-48(-55,7;-37,2)
Espírito Santo	751,9 (666,4;894,9)	44,4 (39,5;51,6)	1547,9 (1278,5;1956,8)	35,8 (29,6;45,1)	-19,5(-31,6;-6,3)
Goiás	1300,3 (1084,7;1633,2)	51,4 (43,6;63,1)	2186,1 (1774;2711,5)	30,8 (25,2;38,2)	-40,1(-51,9;-26)
Maranhão	537,9 (372,1;749,1)	18,8 (13,1;26,1)	1430,7 (1131,3;1752,5)	21,1 (16,7;25,8)	12,2(-22,4;60,8)
Mato Grosso	389,5 (311,5;457,7)	37,9 (31,6;43,6)	886,3 (752;1040,4)	26 (22,2;30,6)	-31,6(-44;-14,5)
Mato Grosso do Sul	480 (417,5;547,4)	44,8 (39,3;50,6)	861 (731,5;1024)	29 (24,7;34,5)	-35,2(-45;-22,5)
Minas Gerais	5239 (4676,4;5973,4)	46,4 (41,5;52,2)	7257,9 (6282,1;8566,6)	27,7 (24;32,7)	-40,3(-48,2;-31,3)
Pará	799,5 (667,7;934,9)	32 (26,8;37,2)	1621,9 (1376,2;1975)	22 (18,6;26,6)	-31,3(-44,2;-14,2)
Paraíba	374,9 (262,1;468,5)	15,8 (11,2;19,7)	796,6 (649,7;956)	16,9 (13,8;20,3)	6,6(-17,4;43)
Paraná	2959,8 (2640,8;3386,2)	53,3 (46,9;60,2)	5001,5 (4227,4;5984,7)	38,2 (32,3;45,7)	-28,2(-38,2;-17,4)
Pernambuco	1574,6 (1396;1798,1)	31,9 (28,2;36,2)	3016,7 (2526,4;3737,7)	30 (25,1;37,2)	-6,1(-22,4;14,8)
Piauí	280,7 (223,7;336,3)	18,8 (15,2;22,5)	616,2 (516,4;769,6)	16,3 (13,6;20,3)	-13,3(-31,2;9,6)
Rio de Janeiro	4090,8 (3468;4549,8)	38,3 (32,4;42,2)	5758,8 (4921,1;6639,2)	26,2 (22,4;30,2)	-31,6(-40,4;-20,5)
Rio Grande do Norte	375 (285,6;450,5)	22,1 (16,9;26,5)	853,2 (693,3;1040,7)	21,8 (17,7;26,7)	-1,2(-24,8;32,9)
Rio Grande do Sul	3619,1 (3136,5;4083,7)	50,4 (43,6;56,2)	5938 (4979,2;7063,4)	38,9 (32,7;46,1)	-22,8(-33,1;-10,4)
Rondônia	195,6 (154,3;229,2)	43,3 (36,2;49,3)	403,1 (335,6;477,7)	25,2 (21,1;29,8)	-41,8(-52,8;-26,8)
Roraima	29,8 (21,7;35,6)	36,3 (27,8;41,9)	95,9 (77;112,8)	24,5 (20,4;28,5)	-32,5(-42,3;-18,5)
Santa Catarina	1638,4 (1417,6;1889,5)	57,6 (50,3;65,5)	3162,9 (2677;3733,4)	40 (34;47,1)	-30,5(-40;-19,9)
São Paulo	13095,7 (11482,2;14641,3)	55,8 (48,6;62,1)	17256,1 (14768,7;20498,9)	32,3 (27,6;38,3)	-42,1(-49,8;-30,8)
Sergipe	167,8 (127,4;201,3)	19,8 (15,2;23,5)	398,4 (324,4;492,7)	17,3 (14,1;21,3)	-12,8(-31,7;13,9)
Tocantins	113,9 (91,9;138,5)	26,4 (21,3;31,6)	402,9 (317;537,4)	27,5 (21,8;36,7)	4,2(-20,2;39)
** B.2.5.2-Doença valvar mitral degenerativa não reumática **					**(;)**
Acre	30,8 (24,8;40,5)	12,7 (10,2;17,1)	87,5 (70,5;117,6)	11,7 (9,4;15,7)	-7,5(-26,1;15,3)
Alagoas	289 (228,4;353,5)	17,2 (13,4;21,2)	594,5 (467,7;729,3)	17,2 (13,4;21)	-0,4(-23,6;32,1)
Amapá	23,9 (18,7;29,1)	15,1 (12;18,8)	95,5 (74,7;115,9)	14,3 (10,9;17,4)	-5,1(-20,3;12,4)
Amazonas	176,4 (144,8;232,6)	14,8 (12,2;20,3)	370,2 (300,2;480,1)	10,7 (8,7;13,8)	-27,5(-40,3;-11,6)
Bahia	1363,3 (1095,7;1693,3)	16,2 (13;20,5)	2193,7 (1725,5;2681,5)	13,2 (10,3;16,2)	-18,4(-36,8;5,3)
Brasil	23163,9 (18446,7;27428,1)	20,4 (16,3;24,6)	33793,5 (25861,5;39014,6)	14,1 (10,8;16,3)	-30,7(-41,6;-22,6)
Ceará	545 (408,1;692,2)	11,6 (8,6;14,9)	1346,8 (1010,7;1698,5)	13,2 (9,9;16,6)	13,7(-17,1;54)
Distrito Federal	268,7 (201,2;319,7)	26,6 (19,4;32,9)	398,9 (287,1;482,7)	13,8 (9,8;16,7)	-48,3(-61,4;-37,1)
Espírito Santo	502,3 (340,2;588,9)	25,5 (17,4;29,8)	867,1 (599,1;1068,3)	19,4 (13,3;23,9)	-23,9(-36,2;-9,6)
Goiás	720,9 (530,3;979,3)	24 (17,8;33,5)	1095,9 (824,4;1416,4)	14,7 (11;18,9)	-38,8(-53,3;-21,5)
Maranhão	326,5 (222,8;439)	10,5 (7,2;13,9)	762,9 (582,9;1040,8)	10,7 (8,2;14,7)	2,2(-30,8;47,5)
Mato Grosso	222,5 (166,6;275,9)	17,4 (13,4;21,6)	495,7 (391,1;597,1)	13,5 (10,5;16,3)	-22,4(-37,3;-4,2)
Mato Grosso do Sul	269,3 (203,6;315,4)	21 (16,1;25)	443,9 (337,5;538,1)	14,4 (10,9;17,5)	-31,4(-43,9;-17,7)
Minas Gerais	2759,7 (2198,7;3466,3)	21,9 (17,7;28)	3473,5 (2676;4153,8)	13,3 (10,3;15,9)	-39,1(-53,7;-27,4)
Pará	547,6 (420,2;655,9)	18,1 (14,1;21,9)	1063,7 (825,2;1291,4)	13,2 (10;16,1)	-26,7(-40,6;-9,5)
Paraíba	248,7 (184,3;329)	10,1 (7,4;13,2)	427,3 (334,8;609,2)	9,2 (7,1;13)	-9(-35,2;22,2)
Paraná	1622,1 (1205,7;1926,7)	25,1 (18,5;30,1)	2183,5 (1520,6;2709,5)	16,2 (11,3;20)	-35,3(-47,5;-22,5)
Pernambuco	1209,1 (901,4;1393,7)	22,4 (16,7;25,8)	1901,1 (1367,5;2278,3)	18,3 (13,1;21,9)	-18,3(-32,7;-1)
Piauí	211,4 (164,6;250,5)	12,5 (9,6;14,9)	404 (321,8;497,4)	10,7 (8,5;13,2)	-14,5(-31,4;4,9)
Rio de Janeiro	2576,8 (2159,7;3166,9)	21,9 (18,4;27,3)	2889,1 (2339,7;3691,9)	13,2 (10,7;16,8)	-39,6(-49,2;-29,6)
Rio Grande do Norte	207,9 (165,8;270,9)	11,4 (9,1;15)	439,4 (341,2;567,7)	11,1 (8,7;14,4)	-2,5(-27,6;27,6)
Rio Grande do Sul	1762,1 (1287,4;2066,8)	22,2 (16,4;26,5)	2310,3 (1546,4;2837,7)	15,4 (10,5;18,9)	-30,5(-42,4;-17)
Rondônia	108,1 (81,9;139,1)	17,6 (14;23,7)	210,4 (169,3;274,4)	12,1 (9,7;15,7)	-31,4(-47,3;-13,3)
Roraima	11,7 (7,8;20,6)	10,8 (7,4;19,5)	36,3 (24;64,6)	7,8 (5,2;14)	-28,4(-41,2;-12,2)
Santa Catarina	801,5 (548,8;987,9)	23,8 (16,2;29,7)	1215,4 (818,2;1494,1)	14,7 (9,8;18,1)	-38,3(-49,6;-26,3)
São Paulo	6133,9 (4630,3;7686,7)	23,4 (17,9;29,9)	7925,7 (5513,9;9414)	14,5 (10,2;17,2)	-37,9(-52,3;-26,2)
Sergipe	132,8 (103,6;165,3)	13,7 (10,8;17,5)	290,8 (222,2;361,9)	12 (9,2;15)	-12,3(-30,2;13,9)
Tocantins	91,9 (63,5;114,4)	17,1 (11,4;21,3)	270,5 (184,9;338)	17,4 (11,8;21,9)	2,1(-20,4;32,7)

*Fonte: Dados derivados do estudo Global Burden of Disease 2019, Institute for Health Metrics and Evaluation, University of Washington. ^46^*

**Tabela 5-5:** – Número total de admissões e custos associados em Reais (R$) e Dólares Internacionais 2019 (Int$) para procedimentos clínicos e cirúrgicos para tratar doença valvar do coração no Brasil, de 2008 a 2019.

Procedimento:	2008	2009	2010	2011	2012	2013	2014	2015	2016	2017	2018	2019	Total
Cirurgias valvares (N)	12.201	12.664	12.169	13.181	13.435	13.067	12.993	12.624	12.432	12.277	12.088	12.771	151.902
Cirurgias valvares (R$)	R$ 125,954,499	R$ 140,683,968	R$ 142,383,177	R$ 179,111,011	R$ 183,271,263	R$ 178,563,635	R$ 180,088,277	R$ 176,813,774	R$ 175,318,559	R$ 176,135,832	R$ 177,583,849	R$ 187,382,032	R$ 2,023,289,882
Cirurgias valvares (Int$)	$123,559,115	$128,654,775	$120,060,127	$139,380,403	$132,111,420	$119,760,766	$111,985,310	$102,217,742	$93,772,533	$91,038,538	$89,090,521	$90,566,473	$1,342,197,727
Outras valvoplastias (N)	451	477	445	486	456	527	515	513	399	427	391	450	5,537
Outras valvoplastias (R$)	R$ 1,518,843	R$ 1,661,544	R$ 1,717,544	R$ 1,918,678	R$ 1,870,621	R$ 2,051,540	R$ 2,128,294	R$ 2,085,967	R$ 1,594,213	R$ 1,888,744	R$ 1,689,593	R$ 1,959,571	R$ 22,085,157
Outras valvoplastias (Int$)	$1,489,958	$1,519,473	$1,448,265	$1,493,075	$1,348,440	$1,375,947	$1,323,449	$1,205,917	$852,695	$976,227	$847,637	$947,110	$14,828,198
Valvoplastia mitral (N)	477	551	478	473	403	431	408	341	206	236	200	195	4,399
Valvoplastia mitral (R$)	R$ 3,115,254	R$ 3,585,355	R$ 3,147,310	R$ 3,227,816	R$ 2,718,391	R$ 2,970,343	R$ 2,808,556	R$ 2,392,670	R$ 1,377,571	R$ 1,720,524	R$ 1,461,666	R$ 1,430,166	R$ 29,955,628
Valvoplastia mitral (Int$)	$3,056,009	$3,278,789	$2,653,870	$2,511,818	$1,959,557	$1,992,178	$1,746,460	$1,383,225	$736,820	$889,279	$733,290	$691,235	$21,632,536
Total cirúrgico (N)	13,129	13,692	13,092	14,140	14,294	14,025	13,916	13,478	13,037	12,940	12,679	13,416	161,838
Total cirúrgico (R$)	R$ 130,588,598	R$ 145,930,868	R$ 147,248,032	R$ 184,257,507	R$ 187,860,275	R$ 183,585,519	R$ 185,025,128	R$ 181,292,412	R$ 178,290,344,05	R$ 179,745,101	R$ 180,735,108	R$ 190,771,770	R$ 2,075,330,668
Total cirúrgico (Int$)	$128,105,083	$133,453,038	$124,162,262	$143,385,297	$135,419,418	$123,128,891	$115,055,220	$104,806,885	$95,362,050,10	$92,904,045	$90,671,449	$92,204,819	$1,378,658,462
Doença valvar (clínico) (N)	3,237	4,156	3,526	3,637	3,285	2,996	2,753	2,400	2,244	2,231	2,330	2,289	35,084
Doença valvar (clínico) (R$)	R$ 1,051,959	R$ 1,589,247	R$ 1,439,424	R$ 1,606,640	R$ 1,509,338	R$ 1,509,785	R$ 1,584,222	R$ 1,672,410	R$ 1,675,284	R$ 1,678,874	R$ 2,043,385	R$ 1,999,540	R$ 19,360,112
Doença valvar (clínico) (Int$)	$1,031,953	$1,453,358	$1,213,749	$1,250,253	$1,088,009	$1,012,597	$985,126	$966,836	$896,058	$867,752	$1,025,128	$966,428	$12,757,251

**Figura 5-1:**
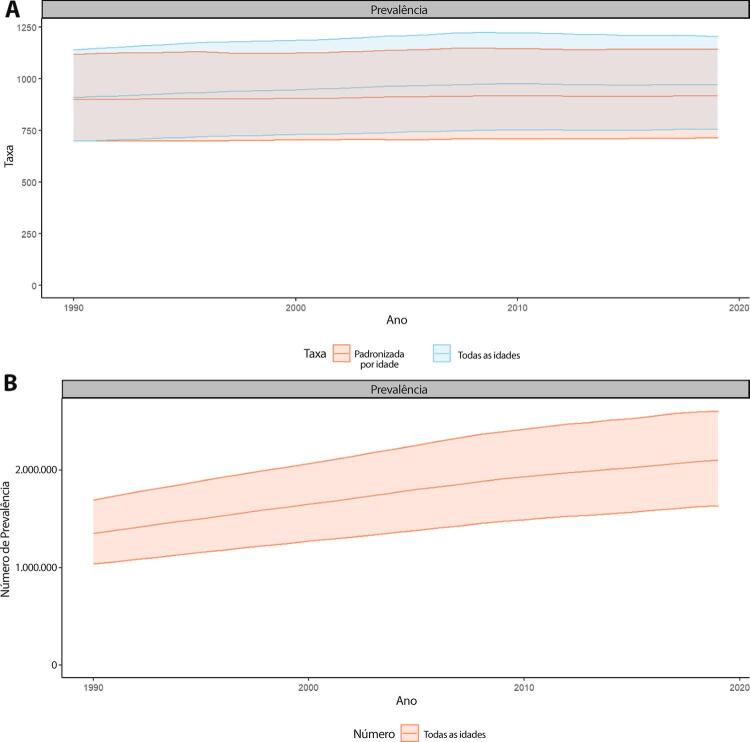
-A: Taxa de prevalência bruta e padronizada por idade de doença cardíaca reumática no Brasil de 1990 a 2019. B: Total de casos prevalentes de doença cardíaca reumática no Brasil de 1990 a 2019.

Fonte: Dados derivados do estudo Global Burden of Disease 2019, Institute for Health Metrics and Evaluation, University of Washington. ^46^

**Figura 5-2:**
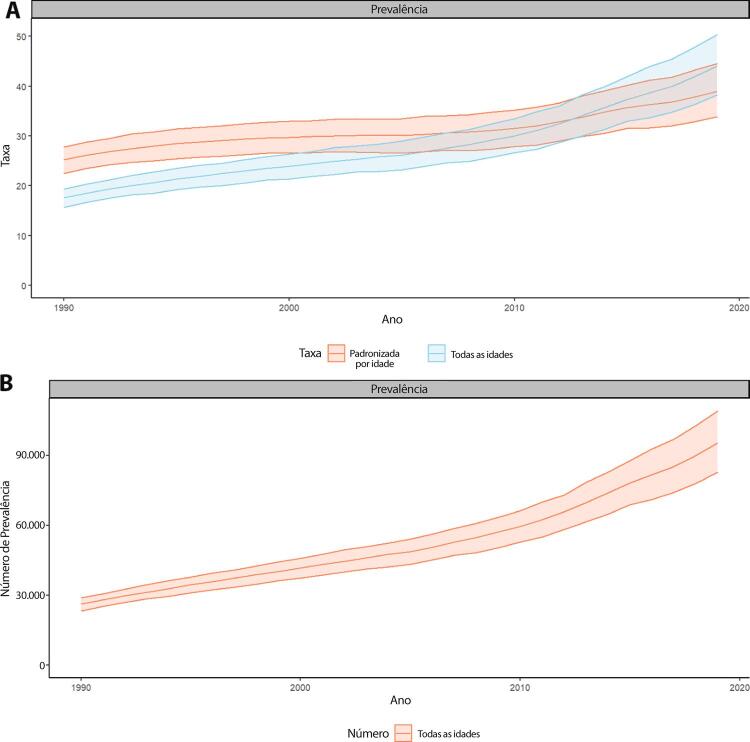
-A: Taxa de prevalência bruta e padronizada por idade de doença valvar do coração não reumática no Brasil de 1990 a 2019. B: Número de casos prevalentes de doença valvar do coração não reumática no Brasil de 1990 a 2019.

Fonte: Dados derivados do estudo Global Burden of Disease 2019, Institute for Health Metrics and Evaluation, University of Washington. ^46^

**Figura 5-3:**
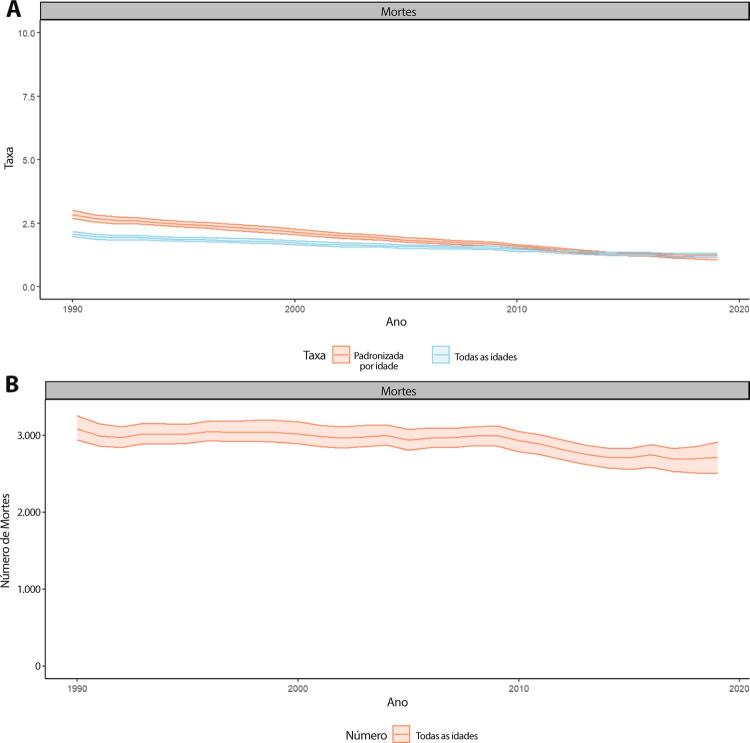
-A: Taxa de mortalidade bruta e padronizada por idade de doença cardíaca reumática no Brasil de 1990 a 2019. B: Número total de mortes atribuíveis a doença cardíaca reumática no Brasil de 1990 a 2019.

Fonte: Dados derivados do estudo Global Burden of Disease 2019, Institute for Health Metrics and Evaluation, University of Washington. ^46^

**Figura 5-4:**
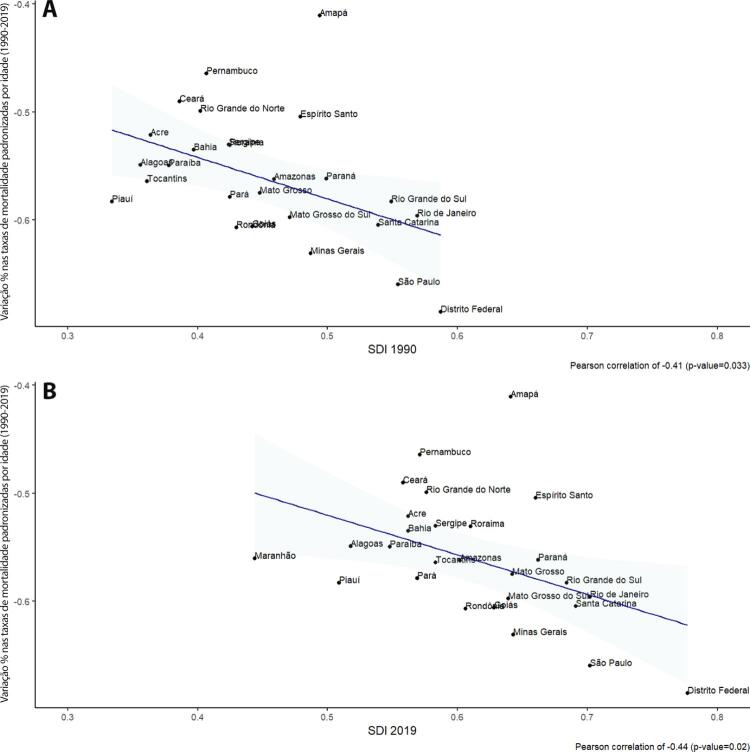
-Correlação entre o Índice Sociodemográfico (SDI) e a variação percentual nas taxas de mortalidade padronizadas por idade atribuíveis a doença cardíaca reumática nas unidades federativas brasileiras em 1990 (A) e 2019 (B).

Fonte: Dados derivados do estudo Global Burden of Disease 2019, Institute for Health Metrics and Evaluation, University of Washington. ^46^

**Figura 5-5:**
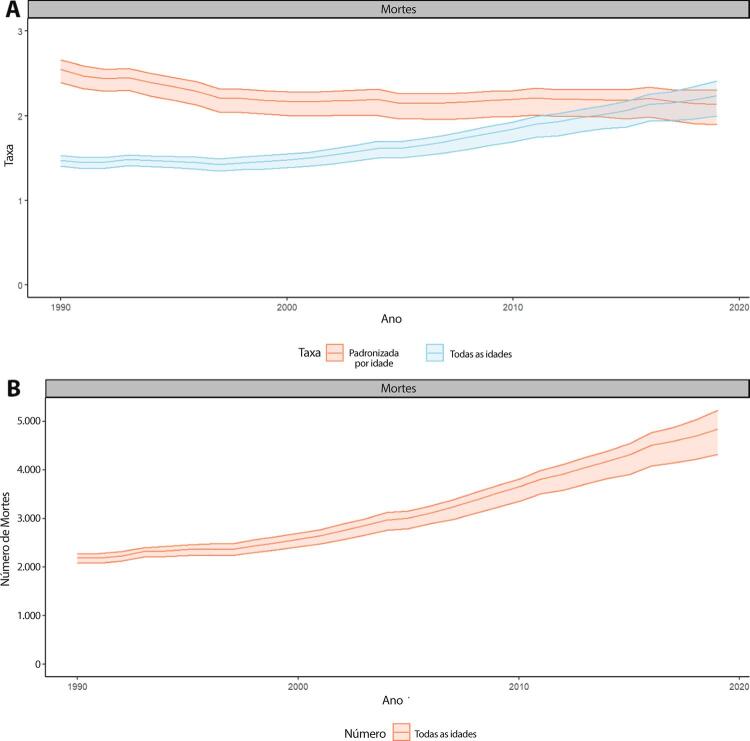
-A: Taxa de mortalidade bruta e padronizada por idade atribuível a doença valvar do coração não reumática no Brasil de 1990 a 2019. B: Número total de mortes atribuíveis a doença valvar do coração não reumática no Brasil de 1990 a 2019.

Fonte: Dados derivados do estudo Global Burden of Disease 2019, Institute for Health Metrics and Evaluation, University of Washington. ^46^

**Figura 5-6:**
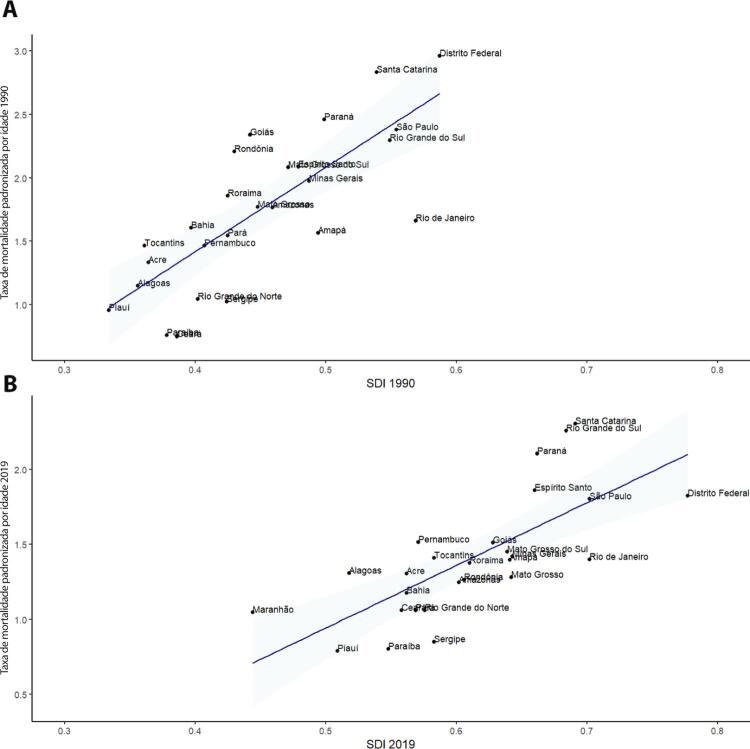
-Correlação entre as taxas de mortalidade padronizadas por idade atribuíveis a doença valvar aórtica calcífica e o Índice Sociodemográfico (SDI) nas unidades federativas brasileiras em 1990 (A) e 2019 (B).

Fonte: Dados derivados do estudo Global Burden of Disease 2019, Institute for Health Metrics and Evaluation, University of Washington.46

**Figura 5-7:**
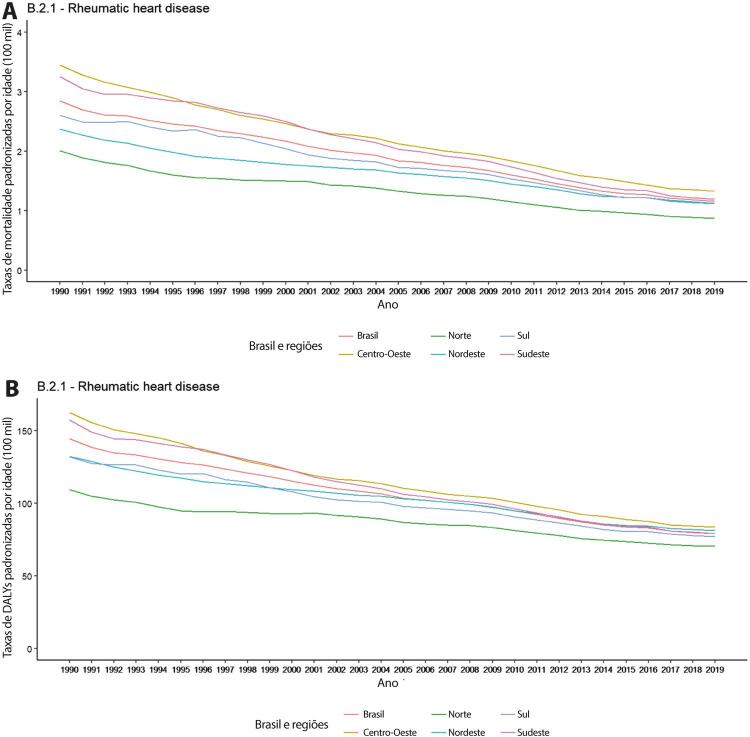
-A: Taxas de mortalidade padronizadas por idade atribuíveis a doença cardíaca reumática no Brasil e em cada região de 1990 a 2019. B: Taxas de DALYs padronizadas por idade atribuíveis a doença cardíaca reumática no Brasil e em cada região de 1990 a 2019.

Fonte: Dados derivados do estudo Global Burden of Disease 2019, Institute for Health Metrics and Evaluation, University of Washington. ^46^

**Figura 5-8:**
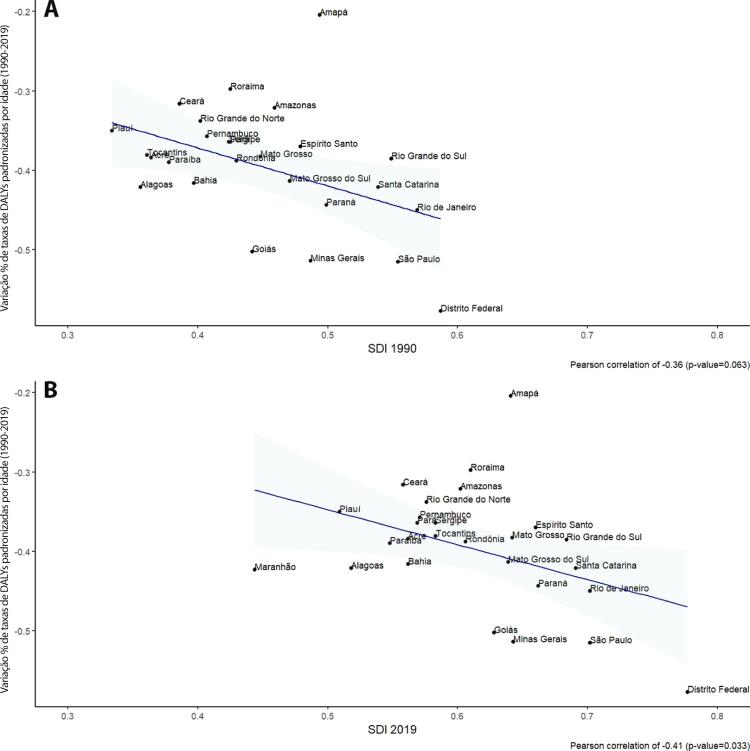
-Correlação entre a variação percentual nas taxas de DALYs atribuíveis a doença cardíaca reumática de 1990 a 2019 e o Índice Sociodemográfico (SDI) nas unidades federativas brasileiras em 1990 (A) e 2019 (B).

Fonte: Dados derivados do estudo Global Burden of Disease 2019, Institute for Health Metrics and Evaluation, University of Washington. ^46^

**Figura 5-9:**
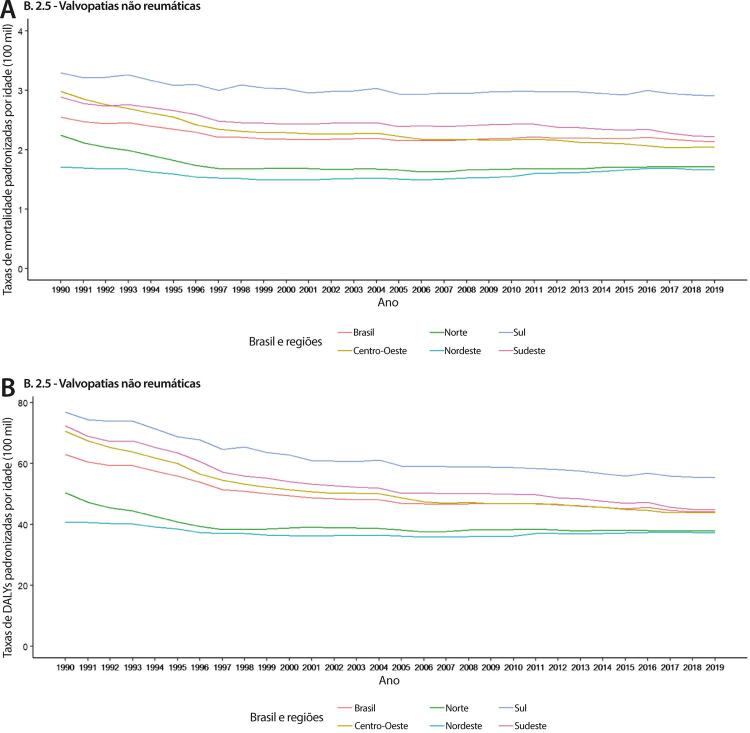
-A: Taxas de mortalidade padronizadas por idade atribuíveis a doença valvar do coração não reumática no Brasil e em cada região de 1990 a 2019. B: Taxas de DALYs padronizadas por idade atribuíveis a doença valvar do coração não reumática no Brasil e em cada região de 1990 a 2019.

Fonte: Dados derivados do estudo Global Burden of Disease 2019, Institute for Health Metrics and Evaluation, University of Washington. ^46^

**Figura 5-10:**
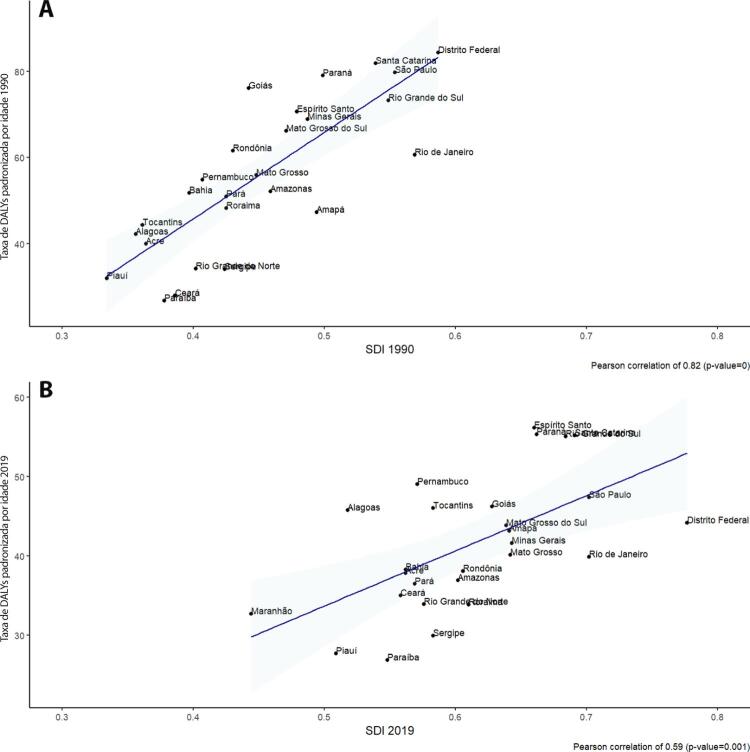
-Correlação entre a variação percentual nas taxas de DALYs atribuíveis a doença valvar do coração não reumática de 1990 a 2019 e o Índice Sociodemográfico (SDI) nas unidades federativas brasileiras em 1990 (A) e 2019 (B).

Fonte: Dados derivados do estudo Global Burden of Disease 2019, Institute for Health Metrics and Evaluation, University of Washington. ^46^

**Figura 5-11:**
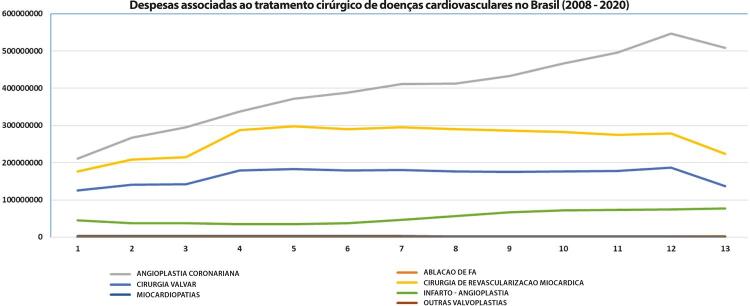
-Despesas associadas ao tratamento cirúrgico de doenças cardiovasculares no Brasil, de acordo com o tipo no SUS, de 2008 a 2020 (Moeda: International Dollars). ^95^

### Prevalência

#### Doença Cardíaca Reumática

•De acordo com o *Global Atlas on Cardiovascular Disease Prevention and Control* , atualizado pelo GBD 2019, estima-se que a DCR afete atualmente cerca de 40,5 milhões de pessoas em todo o mundo, sendo responsável por 1% a 1,5% de todas as mortes cardiovasculares (306 mil mortes). ^1^ ^,^ ^2^ Até a metade do século 20, a DCR foi a principal causa de doença valvar cardíaca no mundo. Melhores condições de saúde, identificação precoce das infecções por *Streptococcus pyogenes* , além do uso de antibióticos, reduziram significativamente a prevalência de DCR em países de alta renda. Dados publicados em 2016 estimaram que a DCR fosse a causa primária de 2,5% das doenças valvares do coração nos Estados Unidos e no Canadá, chegando a 22% na Europa. ^175^ Taxas ainda mais altas foram relatadas no Brasil, sendo a DCR responsável por cerca de 50% das cirurgias valvares no SUS. ^176–178^

•Em países de renda baixa e média, a prevalência de DCR permanece em torno de 444 por 100 mil habitantes. ^4^ ^,^ ^179^ No Brasil, a DCR persiste como a principal etiologia das doenças valvares cardíacas, em especial em pacientes do SUS. Avaliações anteriores mostraram prevalência de 360 por 100 mil no Brasil. ^180^ Outras avaliações encontraram prevalência variando de 100 a 700 por 100 mil crianças em idade escolar. ^181^

•No Brasil, dos 174 pacientes com doença valvar cardíaca aguda que se apresentaram na emergência do Instituto do Coração de São Paulo, observou-se envolvimento reumático em 60%, seguindo-se doença valvar aórtica degenerativa (15%) e prolapso de valva mitral (13%). No total, 27,5% dos pacientes apresentavam regurgitação mitral isolada e 11% apresentavam estenose mitral, estando a doença valvar aórtica presente nos demais. ^182^

•Um estudo recente no Brasil mostrou que as taxas de resolução da doença valvar cardíaca, em especial em pacientes com FRA moderada/grave, podem ser menores do que anteriormente descrito. Apenas 22/69 pacientes (31,9%) apresentaram resolução total da regurgitação mitral após cardite reumática. A resolução total da regurgitação aórtica também foi menos frequente do que a relatada em estudos anteriores à ‘era da ecocardiografia’ (18%). A maioria dos casos persistiu com regurgitação mitral leve ou aórtica residual. ^183^ Em outro estudo envolvendo 258 crianças e adolescentes com FRA acompanhados por 2 a 15 anos, involução das lesões valvares ocorreu em 25% dos pacientes com cardite leve, em 2,5% daqueles com cardite moderada e em nenhum daqueles com cardite grave. ^184^

•Com relação à progressão da doença, um escore ecocardiográfico de risco para predição de desfecho em médio prazo de crianças com DCR detectada à ecocardiografia foi derivado de uma coorte brasileira, ^185^ com boa discriminação em uma segunda amostra de rastreamento no país ^186^ (estatística C=0,71, IC 95%, 0,63 - 0,80) e em um *pool* de coortes de três outros países (estatística C=0,70, IC 95%, 0,64 - 0,76). Sua aplicação pode ser útil para detectar indivíduos com maior risco de sequelas tardias. ^187^

•Além do escore ecocardiográfico de risco, outros marcadores de desfecho desfavorável de DCR latente e clínica (em especial, progressão para doença clínica e necessidade de intervenção valvar, respectivamente) foram avaliados no Brasil. Entre indivíduos com DCR latente, IL-4, IL-8 e IL-1RA parecem ser os melhores preditores de doença clínica. Ademais, a expressão corregulada de IL-6 e fator de necrose tumoral-α está associada a disfunção valvar grave, enquanto altos níveis de IL-10 e IL-4 foram, subsequentemente, preditores de desfecho adverso em indivíduos com doença estabelecida. ^188^

•Em outro estudo genético comparando amostras de indivíduos com DCR latente e clínica a controles, doença clínica associou-se a níveis mais elevados de todas as citocinas, sendo IL-4, CXCL8 e IL-1RA seus mais fortes preditores em comparação à DCR latente. Observou-se uma associação de DCR clínica com polimorfismo nos genes IL-2, IL-4, IL-6 e IL-10, sendo que polimorfismo genético e expressão fenotípica de IL-4 discriminam com precisão DCR latente *versus* DCR clínica, podendo orientar o manejo clínico no futuro. ^189^

•De acordo com o GBD 2019, de 1990 a 2019, a prevalência padronizada por idade de DCR apresentou discreto aumento de 2,1% (II 95%, 0,2 – 4,0), passando de 899,6 (II 95%, 699,8 – 1119,1) para 918,5 (II 95%, 716 – 1142,5) por 100 mil, permanecendo mais alta em mulheres do que em homens em todo o período (Tabela 5-1 e Figura 5 -1 .A). Ainda que pequeno em ambos os sexos, o aumento percentual foi numericamente mais pronunciado em mulheres (3,5%) do que em homens, que apresentaram tendência à estabilidade (0,1%, II 95%, -2,4 – 2,8). Os aumentos percentuais foram mais altos nos estados de Alagoas, Bahia e Sergipe, da região Nordeste, que apresenta a mais baixa renda. Embora as estimativas centrais tenham sido mais elevadas nesses estados, os II 95% foram amplos e se sobrepuseram àqueles de outras UF (Tabela 5-1). ^190^ Entretanto, pode-se levantar a hipótese de que o pequeno aumento na prevalência de DCR observado no período reflita o avanço ocorrido na coleta de dados epidemiológicos e nas estatísticas de saúde, assim como a inclusão sistemática de DCR definitiva subclínica. ^4^ ^,^ ^18^

•A prevalência bruta de DCR, entretanto, aumentou 6,8% (II 95%, 1,7 – 12,5%) de 1990 a 2019, passando de 908,8 (II 95%, 699,1 - 1139,4) para 970,2 (II 95%, 756,6 - 1202,9) por 100 mil, permanecendo também maior nas mulheres no período ( Figura 5 -1 .B). À semelhança da tendência das taxas de prevalência padronizadas por idade, o aumento na prevalência bruta foi mais pronunciado entre as mulheres do que entre os homens (8,4% vs. 4,2%). ^4^ ^,^ ^18^

•Mesmo com as tendências relativamente estáveis retratadas pela modelagem do GBD 2019, a DCR é a causa mais prevalente de doença valvar mitral no Brasil conforme dados publicados, quando tanto estenose mitral (> 90%) quanto regurgitação mitral (55-60%) são consideradas. ^182^

•A estenose mitral ocorre muito mais frequentemente em mulheres do que em homens, na razão de 3 para 2. Trata-se de sequela frequente da FRA, afetando mais de 85% dos casos mesmo em países de alta renda, como os europeus, ^191^ com padrão similar ao observado no Brasil. ^178^ ^,^ ^182^ Mais raramente, a estenose mitral está associada com outras doenças, como calcificação do anel mitral, mucopolissacaridose, artrite reumatoide e síndrome carcinoide congênita. ^175^ ^,^ ^182^

•Estudo mais recente de rastreamento em larga escala, avaliando DCR subclínica, mostrou prevalência de 42 por 1.000 crianças em idade escolar (idade média de 11 anos) em Minas Gerais: 37 por 1.000 para DCR *borderline* e 5 por 1.000 para DCR definitiva. Nesse estudo, observou-se maior prevalência em meninas (48 por 1.000 vs. 35 por 1.000) e em crianças acima dos 12 anos. ^176^ O mesmo projeto concluiu que os centros de atenção primária são o cenário ideal para rastreio de DCR, considerando-se as maiores taxas de participação e envolvimento da população. ^192^

•O rastreamento ecocardiográfico no cenário da atenção primária foi testada como ferramenta adicional, em adição às variáveis clínicas, para predizer alterações maiores no ecocardiograma convencional (incluindo doença valvar significativa e DCR) e priorizar o referenciamento no Brasil. O escore combinado – derivado de 603 pacientes na lista de espera por ecocardiografia e validado em uma amostra similar de 1.526 indivíduos – apresentou boa discriminação, com estatística C = 0,72, sensibilidade = 99% e valor preditivo negativo = 97%, emergindo como ferramenta promissora para o diagnóstico precoce de doença valvar/DCR, avaliação da carga de doença e a priorização de referenciamento. ^193^

•Uma ferramenta adicional para se estimar a prevalência de DCR subclínica é a inteligência artificial. O sistema de *machine learning* ( *Convolutional Neural Network: Redes Neurais Convolucionais* ), atualmente sendo desenvolvido no Brasil para estudos de rastreamento, mostrou boa acurácia (normal=72,8±10,2; DCR *borderline* =64,3±12,2; e DCR definitiva=85,8±11,3), podendo auxiliar a ampliar a disponibilidade do diagnóstico ecocardiográfico precoce no futuro. ^194^

#### Doença Valvar do Coração Não Reumática

•De acordo com o GBD 2019, a prevalência padronizada por idade de DVNR amentou de maneira significativa no Brasil de 1990 a 2019 (aumento de 54,3%), quando passou de 25,3 (II 95%, 22,4 - 27,8) para 39 (II 95%, 33,9 - 44,6), por 100 mil, respectivamente. A variação percentual foi consideravelmente maior para os homens do que para as mulheres (105,9% vs. 20,9%) ( Figura 5 -2 .A). A tendência de elevação foi devida principalmente à doença valvar aórtica calcífica (201,8%), que passou de 7,9 (II 95%, 6,3 - 9,6) por 100 mil em 1990 para 23,7 (II 95%, 19,1 - 29) por 100 mil em 2019, tanto para homens (218,8%) quanto para mulheres (182,2%). Entretanto, para a doença valvar mitral degenerativa, a prevalência padronizada por idade permaneceu estável, com uma discreta variação percentual: -2,3% (II 95%, -4 a -0,4) (Tabela 5-1). ^4^ ^,^ ^18^

•Alinhada às taxas padronizadas por idade, a prevalência bruta de DVNR mostrou aumento significativo de 149,3% (II 95%, 126,7 - 173,3) de 1990 a 2019, passando de 17,6 (II 95%, 15,7 - 19,4) a 44 (II 95%, 38,2 - 50,3) por 100 mil, respectivamente (Tabela 5-1 e Figura 5 -2 .B). Mais uma vez, o aumento foi mais pronunciado para os homens do que para as mulheres. A maior inclinação em comparação às taxas padronizadas por idade sugere que a prevalência esteja aumentando desproporcionalmente nas faixas de idade mais avançada ( Figura 5 -2 .B). ^4^ ^,^ ^18^

•De modo diferente da doença valvar mitral, a doença valvar aórtica é predominantemente degenerativa ou calcífica. Estudos observacionais mostraram que a estenose aórtica é observada em 4,5% da população com idade superior a 75 anos em países de alta renda, como os Estados Unidos. ^195^ De acordo com estudos observacionais ^182^ ^,^ ^196^ e dados do GBD 2019, ^18^ no Brasil, assim como no resto do mundo, tem-se observado uma tendência para aumento da doença valvar aórtica degenerativa em comparação a de outras etiologias, como DCR.

•Portanto, o aumento na prevalência de DVNR em todas as idades tem sido devido principalmente à doença valvar aórtica calcífica [396,6% (II 95%, 353,1 – 450,0)], em especial para os grupos mais idosos (>70 anos) (Tabela 5-2). Entretanto, tem-se observado também tendência a aumento significativo na doença valvar mitral degenerativa [54,1% (II 95%, 50,4 – 58,0)] e outras DVNR, sendo a qualidade de dados sobre DVNR limitada a despeito da melhora em suas fontes (Tabela 5-1). ^4^ ^,^ ^18^

•Contrário ao observado para a etiologia reumática, houve aumento do prolapso de valva mitral como causa de regurgitação mitral primária no Brasil: embora na população geral ele atinja taxas de 1% a 2,5% e tenha bom prognóstico na maioria dos casos, entre os pacientes admitidos com doença valvar cardíaca em uma emergência brasileira em 2009 (56±17 anos, 54% mulheres), 13% apresentavam prolapso de valva mitral como etiologia. ^181^ Inversamente, em um registro hospitalar de cirurgia cardíaca de uma das maiores capitais do país (Salvador), de 2002 a 2005, apenas uma pequena proporção de casos foi associada a prolapso mitral. ^178^ Esse resultado é semelhante ao de um estudo com 78.808 pacientes usando duas grandes bases de dados nacionais (o Sistema Brasileiro de Informações Hospitalares e o Sistema Brasileiro de Informações sobre Mortalidade) de 2001 a 2007, em que apenas 0,24% (187) dos casos informavam prolapso mitral como causa básica. ^197^ Entretanto, os dados podem apresentar viés em razão da ausência de atribuição de código para as etiologias de doença valvar cardíaca no sistema público e na maior parte do sistema privado de saúde.

## Incidência

•De acordo com um estudo baseado em dados hospitalares no Nordeste do Brasil, de 2002 a 2005 (1.320 cirurgias), a incidência anual média de cirurgia valvar cardíaca foi 4,75 por 100 mil residentes e positivamente correlacionada com a idade. As incidências anuais médias de DCR e doença valvar degenerativa foram 2,86 e 0,73 por 100 mil de população, respectivamente. ^178^

### Doença Cardíaca Reumática

•Para a DCR, a incidência específica por idade seguiu uma distribuição bimodal de acordo com a fonte do reembolso da cirurgia, aumentando quase que linearmente em 1 caso por 100 mil de população para cada década de vida até a idade de 40–49 anos, com pico em 4,85 casos por 100 mil de população. Após um declínio, um segundo pico ocorreu no grupo etário de 60-69 anos (6,54 casos por 100 mil de população). ^178^

•A incidência de DCR permaneceu estável no Brasil [aumento percentual: 0,5% (II 95%, -1,2 - 2,6)], variando de 53,9 (II 95%, 40,4 - 67,5) por 100 mil (95.299 casos) em 1990 a 54,2 (II 95%, 40,7 - 68,5) por 100 mil (108.204 casos) em 2019, de acordo com dados do GBD 2019. Essa tendência à estabilidade foi relativamente homogênea em todo o país, com superposição de II 95% mesmo nos estados mais pobres das regiões Norte e Nordeste. ^4^ ^,^ ^18^

•Em geral, observou-se significativa redução na incidência de DCR no grupo etário de 15-49 anos [-12,7% (II 95%, -17,2 a -6,8)], enquanto houve tendência à estabilidade nos demais grupos etários. Tal padrão pode estar associado à melhora no diagnóstico da doença e no controle nos grupos mais jovens (principalmente, 5-18 anos, em que ocorre o pico da incidência de FRA) nas últimas décadas, resultando em menor incidência nos adolescentes e adultos. Entretanto, a diminuição no grupo etário 5-14 anos não foi capturada pelo modelo, presumivelmente influenciado pela falta de dados sobre DCR subclínica antes de 2014. ^4^ ^,^ ^18^

### Doença Valvar do Coração Não Reumática

•Em um padrão diferente daquele da DCR, as taxas de incidência de DVNR padronizadas por idade apresentaram aumento significativo de 11,1% (II 95%, 6,5 - 16,4), passando de 3,6 (II 95%, 3,3 – 4,0) por 100 mil em 1990 para 4,0 (II 95%, 3,6 - 4,5) por 100 mil em 2019, de acordo com estimativas do GBD 2019. Essa elevação deveu-se principalmente ao aumento de 20,1% (II 95%, 12,9 - 28,4) na doença valvar aórtica calcífica, em especial em indivíduos com idade de 50-69 anos [56,9% (II 95%, 39,8 - 75,6)]. ^4^ ^,^ ^18^

•Entretanto, a já mencionada crescente incidência da doença valvar aórtica calcífica em indivíduos de meia-idade [15-49 anos: 56,9% (II 95%, 39,8 - 75,6)] é atípica, considerando-se a epidemiologia da doença, devendo ser interpretada com cautela como uma possível limitação da modelagem do GBD, ^18^ ^,^ ^46^ pois dados primários para essa causa são escassos no Brasil. Ademais, um padrão estável não esperado em indivíduos >70 anos foi modelado. ^4^

Mortalidade

•Doença valvar do coração é uma das principais causas de morte cardiovascular no Brasil, em particular em regiões economicamente desfavorecidas, e a DCR – a etiologia com maior componente social – ocupou a 8 ^a^ /9 ^a^ posição entre as principais causas nas últimas décadas. ^18^ No cenário menos favorecido economicamente, a DCR tem desempenhado importante papel por décadas, com tendência decrescente – nem sempre adequadamente capturada pela modelagem estatística – seguindo-se à melhora socioeconômica. ^174^ ^,^ ^179^ ^,^ ^197^

•Apesar da escassez dos dados em âmbito nacional, a sub-análise de uma coorte multicêntrica com 920 pacientes submetidos a cirurgia valvar (substituição isolada da valva aórtica, 34%; substituição isolada da valva mitral, 25%; 81% cobertas pelo sistema público de saúde) mostrou uma taxa de mortalidade cirúrgica aceitável (7,3%) ^198^ quando comparada à de séries anteriores (11,9%). ^178^

### Doença Cardíaca Reumática

•Contrastando com a tendência crescente de prevalência, as taxas de mortalidade padronizadas por idade atribuíveis à DCR diminuíram significativamente em 59,4% no Brasil, passando de 2,8 (II 95%, 2,7 – 3,0) para 1,2 (II 95%, 1,1 - 1,2) por 100 mil, de acordo com o estudo GBD 2019. A redução percentual foi similar para homens (62,0%) e mulheres (58,1%) (Tabela 5-3 e Figura 5 -3 .A). Observou-se tendência similar para as taxas brutas de mortalidade ( Figura 5 -3 .B). No período, o número total de mortes diminuiu de 3.088 (II 95%, 2.939 – 3.256) em 1990 para 2.715 (II 95%, 2.505 – 2.913) em 2019, apesar do crescimento da população (Tabela 5-3). Essas tendências podem refletir uma melhora das condições de saúde, além de melhor e mais precoce acesso à assistência em saúde. ^4^ ^,^ ^18^

•Em 1990, a DCR ocupou a 10 ^a^ posição entre as causas de morte no Brasil (9 ^a^ a 12 ^a^ em diferentes estados), tendo passado para a 12 ^a^ posição em 2019 (10 ^a^ a 13 ^a^ na maioria dos estados, e 14 ^a^ apenas no Mato Grosso do Sul, na região Centro-Oeste). ^4^ ^,^ ^18^

•A redução mais significativa nas taxas de mortalidade foi observada nas idades menores, especialmente nos grupos etários de ‘5-14 anos’ e ‘15-49 anos’: -78,1% (II 95%, -81,9 a -73,6) e -64,6% (II 95%, -68,3 a -60,4) por 100 mil, respectivamente (Tabela 5-2). ^4^ ^,^ ^18^ Isso pode estar associado ao melhor tratamento de faringite, da FRA e das apresentações precoces da DCR, ainda que as sequelas crônicas persistam como um desafio. ^199^ ^,^ ^200^

•De acordo com dados do GBD 2019, houve significativa correlação negativa entre a variação percentual nas taxas de mortalidade padronizadas por idade e o SDI em 1990 ( Figura 5 -4 ) (r=-0,41, p=0,03) e em 2019 (r=-0,44, p=0,02). ^4^ ^,^ ^18^ Considerando-se a DCR como a etiologia com maior componente social entre as doenças valvares cardíacas, esse padrão difere das estimativas do GBD 2017, não tendo alcançado significância estatística em 2017. Isso pode sugerir que, a despeito das inegáveis melhorias socioeconômicas observadas em todas as regiões brasileiras desde 1990, que impactaram diferentes aspectos de prevenção de doença e atenção à saúde e reduziram significativamente o hiato sociodemográfico, a desigualdade ainda desempenha importante papel na mortalidade por DCR. ^4^ ^,^ ^18^

### Doença Valvar do Coração Não Reumática

•De acordo com o estudo GBD 2019, as taxas de mortalidade padronizadas por idade atribuíveis a DVNR apresentaram uma redução de 16,2% (II 95%, 10,3 - 22,5) de 1990 a 2019 (Tabela 5-3 e Figura 5 -5 .A). Entretanto, para as taxas de mortalidade brutas, observou-se aumento significativo de 51,9% (II 95%, 39,8 - 62,7), com uma considerável contribuição das idades mais avançadas, em especial acima de 70 anos [17,2% (II 95%, 5,4 - 27,4)] (Tabela 5-3 e Figura 5 -5 .B). Os padrões foram similares para homens e mulheres. Tendências semelhantes foram observadas para as taxas de mortalidade por doença valvar aórtica calcífica, com acentuado aumento de 17% (II 95%, 2,0 - 38,5) nos idosos (≥70 anos), refletindo associação com envelhecimento populacional e prevalência de fatores de risco cardiovascular (Tabela 5-2). Para a doença valvar mitral degenerativa, as taxas de mortalidade padronizadas por idade diminuíram 19,0% (II 95%, 5,8 - 34,8), em oposição ao aumento de 36,2% na prevalência bruta (Tabelas 5-1 e 5-3), como resultado das taxas crescentes [16,5% (II 95%, -18,5 - 48,7)] nos septuagenários e mais idosos (Tabela 5-2), embora com II 95% mais amplos. ^4^ ^,^ ^18^ ^,^ ^19^

•As taxas crescentes de mortalidade por DVNR nas idades mais avançadas contrastam bastante com as tendências observadas para DCR, podendo refletir uma maior prevalência e, consequentemente, mortalidade nos grupos etários >70 anos, para DVNR tanto aórtica quanto mitral (Tabela 5-2). De 1990 a 2019, uma carga crescente de doença valvar aórtica calcífica, em homens e mulheres, associou-se com um aumento na mortalidade naquele grupo etário. Os II 95% são amplos em geral para as estimativas de mortalidade por DVNR, em especial para cada envolvimento valvar específico em separado. ^4^ ^,^ ^18^

•Em 1990, a DVNR ocupou a 10 ^a^ posição entre as causas de morte no Brasil (8 ^a^ a 11 ^a^ em diferentes estados), tendo aumentado proporcionalmente para a 9 ^a^ em 2019 (8 ^a^ a 10 ^a^ na maioria dos estados), tendência oposta à observada para a DCR ( Figura 5 -4 ). ^18^

•Os dados do GBD 2019 demonstraram correlações significativas entre as variações nas taxas de mortalidade padronizadas por idade por DVNR em geral e o SDI em 1990 (r= -0,55, p=0,003) e em 2019 (r= -0,58, p=0,001), assim como padrão similar para a doença valvar aórtica calcífica (1990: r= -0,51, p=0,007; 2019: r= -0,54, p=0,003). Fortes correlações positivas foram observadas entre a mortalidade padronizada por idade e o SDI tanto de 1990 (r=0,80, p<0,001) quanto de 2019 (r=0,70, p<0,001) ( Figura 5 -6 ). Como o desenvolvimento socioeconômico se correlaciona com transição epidemiológica e expectativa de vida, um maior SDI está associado a indivíduos mais idosos com risco para condições valvares degenerativas e menos propensos a etiologias infecciosas, como a DCR. Entretanto, no Brasil, as condições socioeconômicas – e possivelmente acesso ótimizado à atenção à saúde – ainda desempenharam um paper importante nas variações de mortalidade por DVNR ao longo do tempo. ^18^

•Correlações similares foram observadas para a doença valvar mitral degenerativa. Por outro lado, para as outras DVNR, não se observou correlação significativa entre o SDI e as taxas de mortalidade padronizadas por idade – e tampouco com suas variações percentuais ao longo do tempo. ^18^

## Carga de Doença

### Doença cardíaca reumática

•De acordo com dados do GBD 2019, a taxa de DALYs padronizada por idade atribuível a DCR diminuiu significativamente em 45,1% no Brasil, passando de 144,6 (II 95%, 126,8 - 167,3) por 100 mil em 1990 para 79,3 (II 95%, 61,6 - 102,6) por 100 mil em 2019 ( Figura 5 -7 .A). As taxas decrescentes observadas no período foram similares para homens e mulheres, -46,7% (II 95%, -54,4 a -39,3) e -44,2% (II 95%, -51,1 a -36,9), respectivamente (Tabela 5-4). ^201^

•As taxas de DALYs padronizadas por idade diminuíram em todos os estados brasileiros, com tendência mais significativa nas regiões com as mais altas taxas em 1990: Centro-Oeste e Sudeste (Tabela 5-2). As regiões Sudeste e Centro-Oeste apresentaram as mais altas taxas de DALYs padronizadas por idade e DALYs proporcionais em 1990, enquanto quatro estados do Nordeste (Sergipe, Bahia, Alagoas e Pernambuco), um do Sul (Paraná) e um do Centro-Oeste (Goiás) ocuparam o topo da lista em 2019. ^4^ ^,^ ^18^

•Um padrão decrescente similar foi observado para as taxas de YLLs padronizadas por idade por DCR, que variaram de 102,1 (II 95%, 97,5 - 107,3) por 100 mil em 1990 a 35,8 (II 95%, 33,5 - 38,4) por 100 mil em 2019, uma redução de 64,9% (II 95%, 61,6 - 67,9). ^4^ ^,^ ^18^

•As estimativas do GBD 2019 não mostraram correlação entre as taxas de DALYs padronizadas por idade e o SDI em 1990 nem em 2019. Da mesma maneira, não houve correlação entre o SDI e a variação percentual nas taxas de DALYs padronizadas por idade em 1990 (r= -0,36, p=0,06), e apenas uma fraca correlação em 2019 (r= -0,41, p=0,03) ( Figura 5 -8 ), sugerindo que os efeitos do desenvolvimento socioeconômico foram menos pronunciados na morbidade em comparação à mortalidade.

### Doença Valvar do Coração Não Reumática

•De acordo com o GBD 2019, as taxas de DALYs padronizadas por idade atribuíveis a DVNR diminuíram (30%) no Brasil, passando de 62,8 (II 95%, 60,3 – 65,2) por 100 mil em 1990 para 44 (II 95%, 40,7 - 47) por 100 mil em 2019 (Tabela 5-4 e Figura 5 -9 .B). O padrão de redução observado no período foi similar para homens (-31%) e mulheres (-28%). Com relação às doenças específicas, as taxas diminuíram igualmente para a doença valvar mitral degenerativa [-30,7% (II 95%, -41,6 a -22,6)] em comparação à doença valvar aórtica calcífica [-30,3% (II 95%, -36,3 a -21,3)]. Para outras DVNR, as taxas padronizadas por idade tenderam ao aumento [19,1% (II 95%, -21,1 – 68,5)], embora os II fossem consideravelmente amplos, incluindo 0. As tendências observadas para os YLLs foram similares. ^4^ ^,^ ^18^

•A tendência decrescente foi relativamente homogênea em todos os estados brasileiros e as taxas de DALYs padronizadas por idade permaneceram mais altas nas regiões Sul e Sudeste em todo o período, assim como em Pernambuco, estado da região Nordeste (5 ^o^ lugar em 2019) ( Figura 5 -9 .B. A redução mais significativa foi observada nas regiões Sudeste e Centro-Oeste (Distrito Federal e estados de São Paulo, Minas Gerais, Goiás e Rio de Janeiro), assim como em Rondônia, na região Norte, que não tem dados primários para a maioria das estimativas. ^4^ ^,^ ^18^

•À semelhança do observado para mortalidade, as taxas decrescentes de DALYs padronizadas por idade devidas a DVNR contrastam com o discreto aumento nas taxas brutas [7,3% (II 95%, 0 - 15,1)] no período (1990 – 2019), influenciadas pelo grupo etário >70 anos (+5%), e mais uma vez sugerindo que a morbidade associada com DVNR esteja se deslocando para os idosos, possivelmente em resposta às mudanças na composição etária da população. ^4^ ^,^ ^18^

•As taxas de DALYs proporcionais no Brasil aumentaram e, de 1990 a 2019, as regiões Sul e Sudeste foram responsáveis pelas maiores proporções de DALYs no período, de acordo com as estimativas do GBD. ^201^ ^,^ ^202^

•Ainda segundo dados do GBD 2019, houve significativas correlações positivas entre as taxas de DALYs padronizadas por idade por DVNR em geral e o SDI em 1990 (r=0,80, p<0,001) e em 2019 (r=0,55, p<0,001) ( Figura 5 -10 ). As variações percentuais nas taxas de DALYs padronizadas por idade (1990 – 2019) também se correlacionaram com o SDI em 1990 (r= -0,72, p<0,001) e em 2019 (r= -0,72, p<0,001). Para a doença valvar aórtica calcífica, foram observadas correlações significativas entre DALYs e SDI em 1990 (r^2^=0,80, p<0,001) e em 2019 (r^2^=0,62, p<0,001), assim como entre as variações percentuais nas taxas de DALYs e o SDI nos dois anos, sugerindo que o desenvolvimento socioeconômico também seja um determinante de DVNR degenerativa, ligado ao envelhecimento e aos fatores de risco.

•Para a doença valvar mitral degenerativa, observou-se correlação positiva entre as taxas de DALYs padronizadas por idade e o SDI em 1990, mas não em 2019, enquanto as variações percentuais nas taxas de DALYs correlacionaram-se negativamente com o SDI em 1990 e em 2019, sugerindo algum impacto dos marcadores socioeconômicos também sobre esta condição.

## Complicações e Doenças Associadas

### Arritmias Associadas com Doença Valvar

•Para pacientes com doença cardíaca valvar, a FA é um fator complicador, ocorrendo, em geral, naqueles com a história natural da doença mais avançada. Acha-se mais comumente associada à doença valvar mitral, em especial estenose mitral. A FA foi observada em 34% de uma coorte de 427 pacientes (idade média, 50±16 anos; 84% mulheres) com estenose mitral grave, tendo sido mais frequente naqueles que morreram durante o acompanhamento (27 de 41; 66%) em comparação aos que sobreviveram (114 de 378; 30%), reforçando seu papel de marcador de prognóstico na doença valvar cardíaca. ^203^

•Além disso, a FA pode se desenvolver na doença valvar aórtica grave, especialmente em pacientes mais idosos e no pós-operatório. Em uma coorte retrospectiva de 348 pacientes (idade média, 76,8±4,6 anos), FA pós-operatória foi observada em 114 (32,8%), mas as taxas foram mais altas a partir de 80 anos em comparação ao grupo etário de 70-79 anos (42,9% vs. 28,8%, p=0,017). ^204^

•Em outra avaliação retrospectiva conduzida em Pernambuco (Nordeste do Brasil), envolvendo 491 pacientes consecutivos após cirurgia por doença valvar do coração, a incidência de FA foi 31,2% e associada com idade >70 anos (OR=6,82; IC 95%, 3,34 - 14,10, p <0,001), doença valvar mitral (OR=3,18; IC 95%, 1,83 - 5,20, p<0,001) e a não utilização de betabloqueadores no período pós-operatório, entre outros fatores. ^205^

•Doença valvar do coração (17,5%) e arritmias (FA e *flutter* atrial – 50,7%) foram as principais fontes cardioembólicas para acidente vascular cerebral em estudo que envolveu 256 pacientes (60,2 ± 6,9 anos, 132 homens) na região Sul do Brasil. ^206^

•No registro BYPASS, uma coorte multicêntrica de pacientes submetidos a cirurgia valvar cardíaca, as complicações pós-operatórias mais frequentes foram arritmias (22,6%), infecções (5,7%) e síndrome de baixo débito (5,1%). ^198^

### Associação entre Doença Valvar do Coração e Doença Arterial Coronariana

•Devido ao elevado risco cirúrgico da combinação de procedimentos valvares e revascularização coronária, é essencial que se reconheça a prevalência de DAC obstrutiva em associação com doença valvar do coração. Estudos mostraram menor prevalência de DAC em pacientes com DCR em comparação àqueles com DVNR, possivelmente como reflexo da menor mediana de idade dos pacientes com DCR e da maior prevalência de fatores de risco coronariano na DVNR. ^207^

•Em estudo no Rio de Janeiro incluindo 1.412 candidatos a cirurgia cardíaca por qualquer indicação, 294 portadores de doença cardíaca valvar primária de etiologia reumática e não reumática foram selecionados. Todos os 294 pacientes tinham idade ≥40 anos e foram submetidos a angiografia coronária. As prevalências de DAC em pacientes com DCR e DVNR foram 4% e 33,6% (p <0,0001), respectivamente. Características e fatores de risco, como idade, dor precordial típica, hipertensão, diabetes mellitus e dislipidemia, foram significativamente associados com DAC obstrutiva. ^208^

•Em outro estudo no Brasil que avaliou 712 pacientes com doença valvar do coração e idade média de 58±13 anos, a incidência de DAC obstrutiva foi 20%. Entretanto, nos mais jovens (<50 anos), a prevalência foi mais baixa (3,3%). ^209^ Esses dados são similares aos observados em outro estudo que incluiu 3.736 pacientes (idade média de 43,7 anos), nos quais a prevalência de DAC obstrutiva combinada com doença valvar do coração foi 3,42%. ^207^

## Utilização e Custo da Atenção à Saúde

•De acordo com a base de dados administrativos do SUS, o total de despesas brutas (reembolso) com hospitalização para tratamento clínico de doença valvar do coração no Brasil aumentou significativamente em 90%, passando de R$ 1.051.959 em 2008 para R$ 1.999.540 em 2019, em um padrão quase linear. Ajustando e convertendo esses valores para dólares internacionais de 2019, o total de custos no sistema público de saúde para hospitalização por condições valvares foi de $ 1.031. 953, em 2008, e $ 966.428, em 2019, uma redução de 6,3%. ^210^

•Da mesma forma, os custos não ajustados associados com procedimentos cirúrgicos/intervencionistas valvares (códigos relacionados com cirurgia valvar, comissurotomia mitral percutânea e outros tipos de valvuloplastia) também aumentaram de 2008 a 2019, passando de R$ 130.588.598 (2019 Int$ 128.105.083) para R$ 190.771.771 (2019 Int$ 92.204.819), embora com menor magnitude em comparação àqueles de internações clínicas (46% vs. 90%). Após ajuste para Int$, observou-se uma significativa redução de 28,0%. O total de despesas com procedimentos cirúrgicos para o SUS nessa série temporal (2008 – 2019) foi R$ 10.524.044.511 (Int$ 6.853.635.725) (Tabela 5-5 e Figura 5 -11 ). ^210^

•O número de internações para cirurgias/intervenções relacionadas às doenças valvares não cresceu muito no Brasil de 2008 a 2019, variando de 12.679 em 2008 a 14.294 em 2019. Isso está provavelmente associado com a complexidade e os custos crescentes das intervenções (em especial, custos hospitalares, dispositivos e próteses) e denota a carga econômica imposta pela incorporação de novos procedimentos e tecnologias, além do efeito marcante da inflação sobre os custos da atenção à saúde – considerando-se os valores mais baixos em 2019 quando ajustados para Int$. Nesse cenário, a futura incorporação de terapias bem estabelecidas ainda não reembolsadas pelo SUS, como a TAVI, contribuirá para aumentar a carga econômica, embora as despesas com demandas judiciais possam facilmente exceder os custos ordinários. ^211^

•O número total de internações por doença valvar (clínica e cirúrgica) nesse período foi de 196.922, tendo a maioria ocorrido na região Sudeste (41,2%), seguida pelas regiões Nordeste (25,7%), Sul (20,2%), Centro-Oeste (7,5%) e Norte (5,4%) (Tabela 5-5). ^210^

•Observou-se queda dramática em alguns tipos de procedimentos, a despeito de suas crescentes indicações, como a comissurotomia mitral percutânea. Especificamente para esse procedimento, os números decrescentes podem estar associados com o atraso nas tabelas de reembolso do SUS, limitando o número de hospitais que realizam essa intervenção. O número absoluto de cirurgias valvares abertas permaneceu estável no período, variando de 12.201 em 2008 a 12.771 em 2019, a despeito do crescente número de casos de doença valvar do coração – em especial DVNR – e da crescente carga para os idosos, à medida que a população envelhece. ^190^ ^,^ ^211^

•Em nenhum dos períodos, o aumento no número de internações esteve em paralelo com as despesas crescentes, sugerindo uma progressiva complexidade – e, consequentemente, custo – dos procedimentos para o tratamento da doença valvar cardíaca, além da inflação dos dispositivos médicos e dos custos hospitalares associados (considerando os valores em Int$) (Tabela 5-5 e Figura 11 ). ^210^

•A partir da base de dados administrativos do SUS, os procedimentos valvares associados com sequelas de DCR não puderam ser diferenciados daqueles associados com outras etiologias, devido à ausência de código específico disponível, além da imprecisão da notificação dos códigos da CID. ^210^

•É interessante notar que estudos observacionais enfatizaram que a DCR permanece sendo a principal etiologia associada com cirurgia cardíaca em jovens no Brasil, atingindo até 60% em um estudo realizado em Salvador, Bahia. ^178^ No Instituto do Coração de São Paulo, o número de cirurgias cardíacas valvares associadas com DCR aumentou substancialmente nos últimos 10 anos, de cerca de 400 cirurgias/ano em 1990 para mais de 600 após 2000. ^121^ Entre 2008 e 2015, houve 26.054 hospitalizações por sequelas de FRA, 45% das quais por doença cardíaca, levando a um custo total possivelmente subestimado de US$ 3,5 milhões ao ano. ^177^ ^,^ ^210^

•Ainda de acordo com estudos observacionais e registros de base hospitalar, as doenças valvares de origem reumática, em geral, são responsáveis por cerca de 90% das cirurgias cardíacas em crianças e por mais de 30% das cirurgias cardíacas em adultos, a maioria jovem. ^135^ Entretanto, poucos estudos epidemiológicos estimaram a carga da doença valvar por causa específica no Brasil.

•O rastreamento ecocardiográfico para DCR latente mostrou-se custo-efetiva em um estudo conduzido no Brasil. Uma estratégia baseada em divisão de tarefas, com aquisição de imagem por não médicos, utilizando dispositivos portáteis, e telemedicina para interpretação remota por especialistas, resultou em uma razão de custo-efetividade incremental de $10.148,38 por DALY evitado, abaixo do limiar estimado de 3 vezes o produto interno bruto per capita, sugerido pela Organização Mundial da Saúde. ^212^

•Informação obtida na base de dados do SUS (DATASUS) mostrou que, de 2008 a 2017, houve 42.720 e 78.966 hospitalizações por FRA e DCR crônica, respectivamente, representando 0,4% e 0,7% das hospitalizações cardiovasculares no país, respectivamente. Essa análise, no entanto, não tem dados específicos para DVNR. ^213^

### Doença Valvar Mitral

•Com base em dados administrativos do SUS de 2001 a 2007 e referentes a cirurgia de valva mitral, em uma série retrospectiva de 78.808 pacientes cirúrgicos consecutivos, a idade média foi de 50,0 anos (35,9 – 62,5) e 40.106 eram mulheres (50,9%). Novamente, a DCR foi a principal etiologia, responsável por 53,7% do total de pacientes submetidos a cirurgia e a mais de 94% daqueles submetidos a procedimentos para estenose mitral, que representou a maior indicação cirúrgica única, correspondendo a 38,9% do total. No geral, a substituição valvar foi realizada em 69,1% das cirurgias. A mortalidade hospitalar foi 7,6%. ^197^

•A mortalidade cirúrgica foi discretamente maior em mulheres do que em homens (7,8% vs. 7,3%; p <0,001) e consideravelmente mais alta nos indivíduos com idade ≥80 anos. Por outro lado, a mais baixa mortalidade foi observada naqueles entre 20 e 39,9 anos (p <0,001). Os pacientes com cirurgias aórtica e mitral combinadas (refletindo etiologia reumática) foram os mais jovens (mediana, 43,3 anos). A cirurgia para estenose aórtica foi mais comum em indivíduos mais idosos (mediana, 58,0 anos) (p <0,001). Reparo valvar apresentou menor mortalidade (3,5%) em comparação a substituição valvar (6,9%), reparo/substituição valvar múltipla (8,2%) e CRVM concomitante (14,6%) (p<0,001). Associação com CRVM ocorreu em 7.147 pacientes (9,1% da amostra). ^197^

•Quanto à comissurotomia percutânea, estudos no Brasil mostraram uma proporção muito maior de mulheres (85%) – coincidente com a epidemiologia da DCR e principalmente da estenose mitral – e de jovens (<40 anos). ^214^ ^,^ ^215^ À medida que se desenvolve *expertise* local nesse procedimento, investigam-se aspectos técnicos, como a utilização rotineira de sedação consciente (baixa dose de midazolam e fentanil), resultando em ansiólise e analgesia, sem efeitos hemodinâmicos. ^216^ Além disso, há esforços contínuos para definir preditores de desfechos clínicos de longo prazo, como alterações na complacência atrioventricular invasiva e não invasiva. ^217^

•Em um estudo retrospectivo visando avaliar o reparo da valva mitral em 54 crianças brasileiras (<16 anos) com DCR crônica precoce, não houve relato de morte peroperatória. Os desfechos tardios mais frequentes (>7 dias) foram lesão mitral residual (n=11) e necessidade de reoperação (n=3). Portanto, o reparo da valva mitral permanece sendo uma estratégia razoável para DCR em idades mais jovens. ^218^

### Doença Valvar Aórtica

•Uma coorte de 724 pacientes consecutivos, submetidos a cirurgia cardíaca no Instituto do Coração de São Paulo, evidenciou, à semelhança de outros estudos, maior taxa de mulheres (55%) e predominância de DCR (60%). Nessa série, entretanto, houve maior proporção de doença valvar aórtica (396 casos) do que de doença valvar mitral (306 casos) em comparação a outras séries. Nos pacientes com doença valvar mitral, observaram-se estenose em 39,9%, regurgitação em 38,4% e disfunção de prótese mitral em 21,7%. Naqueles submetidos a intervenção valvar aórtica, observaram-se estenose em 51,6%, regurgitação em 29,3% e disfunção de prótese em 19,1%. O estudo sugere um aumento de doença valvar aórtica em comparação à doença valvar mitral em um hospital terciário na região Sudeste do Brasil. ^219^

•Outro estudo retrospectivo de coorte foi conduzido em Porto Alegre (Sul do Brasil), com 1.065 pacientes (idade média, 61,4 ± 11,8 anos; 38% mulheres). Substituição de valva aórtica foi realizada em 18,8% e de valva mitral, em 13,4%. Revascularização coronária concomitante foi realizada em 60,3% da amostra e cirurgias valvares, em 32,7%.

A mortalidade hospitalar geral foi 7,8%, sendo menor para CRVM isolada (5,9%), intermediária para cirurgia valvar (aórtica e/ou mitral e/ou tricúspide = 8,6%) e maior para a combinação de procedimentos valvares e CRVM (20,0%). ^220^

### Implante Percutâneo de Valva Aórtica no Brasil

•Como em outros países, a TAVI ganhou importância no Brasil nos últimos 20 anos. Estima-se que mais de 100 mil TAVI já foram realizadas no mundo. ^196^ ^,^ ^211^ A primeira TAVI no Brasil ocorreu em 2008. O registro brasileiro de TAVI informa a realização de 418 TAVI em 18 centros até 2014, tendo esse número crescido exponencialmente desde então. O acesso femoral foi escolhido em 96,2% dos procedimentos e as próteses usadas foram CoreValve ^®^ (86,1%) e Sapien XT ^®^ (13,9%). Nessa experiência inicial, as mortalidades por todas as causas em 30 dias e 1 ano foram 9,1% e 21,5%, respectivamente. ^221^

•Dados do registro de TAVI atualizados em 2017 revelaram um total de 819 pacientes em acompanhamento clínico, demonstrando que o procedimento tem baixa incidência de complicações – em especial desfechos clínicos duros precoces – e ressaltando taxas de disfunção renal pós-procedimento em torno de 18%. ^222^ ^,^ ^223^

•Em outra avaliação realizada na cidade do Rio de Janeiro com 136 pacientes submetidos a TAVI [mediana de idade, 83 (80-87) anos; 51% homens], a mortalidade peroperatória foi 1,5%, a mortalidade de 30 dias, 5,9%, a mortalidade hospitalar, 8,1% e a mortalidade de 1 ano, 15,5%. ^224^

•De 819 valvas aórticas implantadas por via percutânea até 2017, 135 pacientes (20,1%) precisaram de marca-passo permanente. Esses pacientes eram mais velhos (82,5 vs. 81,1 anos; p=0,047), predominantemente homens (59,3% vs 45%; p=0,003) e tinham bloqueio de ramo direito prévio (OR=6,19; IC 95%, 3,56 – 10,75, p≤0,001). O uso da prótese CoreValve ^®^ (OR=3,16; IC 95%, 1,74 – 5,72, p≤0,001) e gradiente transaórtico basal >50 mmHg (OR=1,86; IC 95%, 1,08 – 3,20, p=0,025) foram preditores independentes de implantação de marca-passo permanente. ^223^

### Pesquisa Futura

•Apesar da notável melhora nas últimas décadas, ainda há escassez de dados primários sobre a epidemiologia da doença valvar do coração no Brasil e muito espaço para pesquisa futura.

•Deve-se incluir a coleta de dados administrativos, com o desenvolvimento de codificação específica que permita a discriminação de variáveis, como a valva envolvida, o tipo de disfunção valvar, o tipo de prótese e, principalmente, a etiologia e a associação com doenças sistêmicas. Isso é de especial importância no SUS.

•Além disso, o desenvolvimento em âmbito nacional de registros sobre doença valvar e procedimentos é necessário. O refinamento do sistema de codificação e a implementação mandatória de relatórios clínicos e cirúrgicos – como feito anteriormente para as intervenções coronárias percutâneas – podem ser um passo inicial para melhorar a acurácia na aquisição de dados.

•Como o país tem algumas coortes significativas de pacientes com doença valvar do coração, o acompanhamento de médio e longo prazo dessas amostras deve ser garantido. Importante notar que há iniciativas de pesquisa que requerem incentivos e financiamento para sua continuação, como os estudos em andamento sobre prognóstico de longo prazo de DCR subclínica em crianças e adolescentes, ^176^ ^,^ ^192^ determinantes genéticos e imunológicos da resposta às infecções estreptocócicas que leva a DCR, ^225^ preditores clínicos e relacionados ao procedimento de eventos a curto e longo prazo após comissurotomia mitral percutânea, ^203^ ^,^ ^226^ além de um registro nacional de TAVI. ^221^

•Como um estudo sugere que o rastreamento ecocardiográfico para DCR seja custo-efetivo no Brasil, ^212^ sua aplicação fora do contexto de pesquisa e sua integração no sistema de saúde deveriam ser investigadas em programas de larga escala.

•Além disso, esforços continuados foram direcionados para o desenvolvimento de vacinas contra infecções estreptocócicas, ^225^ devendo-se garantir estudos colaborativos sobre sua eficácia e aplicação clínica para reduzir a carga de DCR.

•Como o reembolso para TAVI acaba de ser aprovado no sistema privado de saúde brasileiro, sua incorporação no SUS parece próxima, ^211^ sendo que a avaliação do seu verdadeiro impacto clínico, orçamentário e social nos desfechos da atenção à saúde pública requer extensa pesquisa e financiamento.

•Finalmente, outras estratégias promissoras para prover diagnóstico precoce e priorizar referenciamentos em áreas com poucos recursos devem ser investigadas no Brasil. Como exemplo, a disponibilidade de modalidades de exames de imagem para o manejo da doença valvar do coração – em especial ecocardiografia – é limitada e distribuída de maneira desigual no país. Nesse cenário, avaliou-se a implementação de tele-ecocardiografia, com a divisão de tarefas e aquisição de imagem por não médicos (ainda não permitida fora do contexto de pesquisa pela regulamentação da atenção à saúde no Brasil) e sua leitura remota. ^227^ Apesar do seu bom desempenho para diagnóstico e discriminação de pacientes com alto risco cardiovascular, ^227^ o impacto sobre os desfechos clínicos e a custo-efetividade da estratégia ainda necessitam avaliação.

•Há espaço para aprimorar o diagnóstico cardíaco remoto no Brasil, através da expansão do tele-ECG, do rastreio de FA, ^228^ das consultas remotas – incluindo aquelas para condições infecciosas, como a COVID-19 ^229^ – e da incorporação das inovações em técnicas de imagem para melhorar o acesso ao cuidado cardiovascular. São necessárias extensas discussões baseadas em robustas evidências científicas.

## Incidência

•De acordo com um estudo baseado em dados hospitalares no Nordeste do Brasil, de 2002 a 2005 (1.320 cirurgias), a incidência anual média de cirurgia valvar cardíaca foi 4,75 por 100 mil residentes e positivamente correlacionada com a idade. As incidências anuais médias de DCR e doença valvar degenerativa foram 2,86 e 0,73 por 100 mil de população, respectivamente. ^178^

### Doença Cardíaca Reumática

•Para a DCR, a incidência específica por idade seguiu uma distribuição bimodal de acordo com a fonte do reembolso da cirurgia, aumentando quase que linearmente em 1 caso por 100 mil de população para cada década de vida até a idade de 40–49 anos, com pico em 4,85 casos por 100 mil de população. Após um declínio, um segundo pico ocorreu no grupo etário de 60-69 anos (6,54 casos por 100 mil de população). ^178^

•A incidência de DCR permaneceu estável no Brasil [aumento percentual: 0,5% (II 95%, -1,2 - 2,6)], variando de 53,9 (II 95%, 40,4 - 67,5) por 100 mil (95.299 casos) em 1990 a 54,2 (II 95%, 40,7 - 68,5) por 100 mil (108.204 casos) em 2019, de acordo com dados do GBD 2019. Essa tendência à estabilidade foi relativamente homogênea em todo o país, com superposição de II 95% mesmo nos estados mais pobres das regiões Norte e Nordeste. ^4^ ^,^ ^18^

•Em geral, observou-se significativa redução na incidência de DCR no grupo etário de 15-49 anos [-12,7% (II 95%, -17,2 a -6,8)], enquanto houve tendência à estabilidade nos demais grupos etários. Tal padrão pode estar associado à melhora no diagnóstico da doença e no controle nos grupos mais jovens (principalmente, 5-18 anos, em que ocorre o pico da incidência de FRA) nas últimas décadas, resultando em menor incidência nos adolescentes e adultos. Entretanto, a diminuição no grupo etário 5-14 anos não foi capturada pelo modelo, presumivelmente influenciado pela falta de dados sobre DCR subclínica antes de 2014. ^4^ ^,^ ^18^

### Doença Valvar do Coração Não Reumática

•Em um padrão diferente daquele da DCR, as taxas de incidência de DVNR padronizadas por idade apresentaram aumento significativo de 11,1% (II 95%, 6,5 - 16,4), passando de 3,6 (II 95%, 3,3 – 4,0) por 100 mil em 1990 para 4,0 (II 95%, 3,6 - 4,5) por 100 mil em 2019, de acordo com estimativas do GBD 2019. Essa elevação deveu-se principalmente ao aumento de 20,1% (II 95%, 12,9 - 28,4) na doença valvar aórtica calcífica, em especial em indivíduos com idade de 50-69 anos [56,9% (II 95%, 39,8 - 75,6)]. ^4^ ^,^ ^18^

•Entretanto, a já mencionada crescente incidência da doença valvar aórtica calcífica em indivíduos de meia-idade [15-49 anos: 56,9% (II 95%, 39,8 - 75,6)] é atípica, considerando-se a epidemiologia da doença, devendo ser interpretada com cautela como uma possível limitação da modelagem do GBD, ^18^ ^,^ ^46^ pois dados primários para essa causa são escassos no Brasil. Ademais, um padrão estável não esperado em indivíduos >70 anos foi modelado. ^4^

Mortalidade

•Doença valvar do coração é uma das principais causas de morte cardiovascular no Brasil, em particular em regiões economicamente desfavorecidas, e a DCR – a etiologia com maior componente social – ocupou a 8 ^a^ /9 ^a^ posição entre as principais causas nas últimas décadas. ^18^ No cenário menos favorecido economicamente, a DCR tem desempenhado importante papel por décadas, com tendência decrescente – nem sempre adequadamente capturada pela modelagem estatística – seguindo-se à melhora socioeconômica. ^174^ ^,^ ^179^ ^,^ ^197^

•Apesar da escassez dos dados em âmbito nacional, a sub-análise de uma coorte multicêntrica com 920 pacientes submetidos a cirurgia valvar (substituição isolada da valva aórtica, 34%; substituição isolada da valva mitral, 25%; 81% cobertas pelo sistema público de saúde) mostrou uma taxa de mortalidade cirúrgica aceitável (7,3%) ^198^ quando comparada à de séries anteriores (11,9%). ^178^

### Doença Cardíaca Reumática

•Contrastando com a tendência crescente de prevalência, as taxas de mortalidade padronizadas por idade atribuíveis à DCR diminuíram significativamente em 59,4% no Brasil, passando de 2,8 (II 95%, 2,7 – 3,0) para 1,2 (II 95%, 1,1 - 1,2) por 100 mil, de acordo com o estudo GBD 2019. A redução percentual foi similar para homens (62,0%) e mulheres (58,1%) (Tabela 5-3 e Figura 5 -3 .A). Observou-se tendência similar para as taxas brutas de mortalidade ( Figura 5 -3 .B). No período, o número total de mortes diminuiu de 3.088 (II 95%, 2.939 – 3.256) em 1990 para 2.715 (II 95%, 2.505 – 2.913) em 2019, apesar do crescimento da população (Tabela 5-3). Essas tendências podem refletir uma melhora das condições de saúde, além de melhor e mais precoce acesso à assistência em saúde. ^4^ ^,^ ^18^

•Em 1990, a DCR ocupou a 10 ^a^ posição entre as causas de morte no Brasil (9 ^a^ a 12 ^a^ em diferentes estados), tendo passado para a 12 ^a^ posição em 2019 (10 ^a^ a 13 ^a^ na maioria dos estados, e 14 ^a^ apenas no Mato Grosso do Sul, na região Centro-Oeste). ^4^ ^,^ ^18^

•A redução mais significativa nas taxas de mortalidade foi observada nas idades menores, especialmente nos grupos etários de ‘5-14 anos’ e ‘15-49 anos’: -78,1% (II 95%, -81,9 a -73,6) e -64,6% (II 95%, -68,3 a -60,4) por 100 mil, respectivamente (Tabela 5-2). ^4^ ^,^ ^18^ Isso pode estar associado ao melhor tratamento de faringite, da FRA e das apresentações precoces da DCR, ainda que as sequelas crônicas persistam como um desafio. ^199^ ^,^ ^200^

•De acordo com dados do GBD 2019, houve significativa correlação negativa entre a variação percentual nas taxas de mortalidade padronizadas por idade e o SDI em 1990 ( Figura 5 -4 ) (r=-0,41, p=0,03) e em 2019 (r=-0,44, p=0,02). ^4^ ^,^ ^18^ Considerando-se a DCR como a etiologia com maior componente social entre as doenças valvares cardíacas, esse padrão difere das estimativas do GBD 2017, não tendo alcançado significância estatística em 2017. Isso pode sugerir que, a despeito das inegáveis melhorias socioeconômicas observadas em todas as regiões brasileiras desde 1990, que impactaram diferentes aspectos de prevenção de doença e atenção à saúde e reduziram significativamente o hiato sociodemográfico, a desigualdade ainda desempenha importante papel na mortalidade por DCR. ^4^ ^,^ ^18^

### Doença Valvar do Coração Não Reumática

•De acordo com o estudo GBD 2019, as taxas de mortalidade padronizadas por idade atribuíveis a DVNR apresentaram uma redução de 16,2% (II 95%, 10,3 - 22,5) de 1990 a 2019 (Tabela 5-3 e Figura 5 -5 .A). Entretanto, para as taxas de mortalidade brutas, observou-se aumento significativo de 51,9% (II 95%, 39,8 - 62,7), com uma considerável contribuição das idades mais avançadas, em especial acima de 70 anos [17,2% (II 95%, 5,4 - 27,4)] (Tabela 5-3 e Figura 5 -5 .B). Os padrões foram similares para homens e mulheres. Tendências semelhantes foram observadas para as taxas de mortalidade por doença valvar aórtica calcífica, com acentuado aumento de 17% (II 95%, 2,0 - 38,5) nos idosos (≥70 anos), refletindo associação com envelhecimento populacional e prevalência de fatores de risco cardiovascular (Tabela 5-2). Para a doença valvar mitral degenerativa, as taxas de mortalidade padronizadas por idade diminuíram 19,0% (II 95%, 5,8 - 34,8), em oposição ao aumento de 36,2% na prevalência bruta (Tabelas 5-1 e 5-3), como resultado das taxas crescentes [16,5% (II 95%, -18,5 - 48,7)] nos septuagenários e mais idosos (Tabela 5-2), embora com II 95% mais amplos. ^4^ ^,^ ^18^ ^,^ ^19^

•As taxas crescentes de mortalidade por DVNR nas idades mais avançadas contrastam bastante com as tendências observadas para DCR, podendo refletir uma maior prevalência e, consequentemente, mortalidade nos grupos etários >70 anos, para DVNR tanto aórtica quanto mitral (Tabela 5-2). De 1990 a 2019, uma carga crescente de doença valvar aórtica calcífica, em homens e mulheres, associou-se com um aumento na mortalidade naquele grupo etário. Os II 95% são amplos em geral para as estimativas de mortalidade por DVNR, em especial para cada envolvimento valvar específico em separado. ^4^ ^,^ ^18^

•Em 1990, a DVNR ocupou a 10 ^a^ posição entre as causas de morte no Brasil (8 ^a^ a 11 ^a^ em diferentes estados), tendo aumentado proporcionalmente para a 9 ^a^ em 2019 (8 ^a^ a 10 ^a^ na maioria dos estados), tendência oposta à observada para a DCR ( Figura 5 -4 ). ^18^

•Os dados do GBD 2019 demonstraram correlações significativas entre as variações nas taxas de mortalidade padronizadas por idade por DVNR em geral e o SDI em 1990 (r= -0,55, p=0,003) e em 2019 (r= -0,58, p=0,001), assim como padrão similar para a doença valvar aórtica calcífica (1990: r= -0,51, p=0,007; 2019: r= -0,54, p=0,003). Fortes correlações positivas foram observadas entre a mortalidade padronizada por idade e o SDI tanto de 1990 (r=0,80, p<0,001) quanto de 2019 (r=0,70, p<0,001) ( Figura 5 -6 ). Como o desenvolvimento socioeconômico se correlaciona com transição epidemiológica e expectativa de vida, um maior SDI está associado a indivíduos mais idosos com risco para condições valvares degenerativas e menos propensos a etiologias infecciosas, como a DCR. Entretanto, no Brasil, as condições socioeconômicas – e possivelmente acesso ótimizado à atenção à saúde – ainda desempenharam um paper importante nas variações de mortalidade por DVNR ao longo do tempo. ^18^

•Correlações similares foram observadas para a doença valvar mitral degenerativa. Por outro lado, para as outras DVNR, não se observou correlação significativa entre o SDI e as taxas de mortalidade padronizadas por idade – e tampouco com suas variações percentuais ao longo do tempo. ^18^

## Carga de Doença

### Doença cardíaca reumática

•De acordo com dados do GBD 2019, a taxa de DALYs padronizada por idade atribuível a DCR diminuiu significativamente em 45,1% no Brasil, passando de 144,6 (II 95%, 126,8 - 167,3) por 100 mil em 1990 para 79,3 (II 95%, 61,6 - 102,6) por 100 mil em 2019 ( Figura 5 -7 .A). As taxas decrescentes observadas no período foram similares para homens e mulheres, -46,7% (II 95%, -54,4 a -39,3) e -44,2% (II 95%, -51,1 a -36,9), respectivamente (Tabela 5-4). ^201^

•As taxas de DALYs padronizadas por idade diminuíram em todos os estados brasileiros, com tendência mais significativa nas regiões com as mais altas taxas em 1990: Centro-Oeste e Sudeste (Tabela 5-2). As regiões Sudeste e Centro-Oeste apresentaram as mais altas taxas de DALYs padronizadas por idade e DALYs proporcionais em 1990, enquanto quatro estados do Nordeste (Sergipe, Bahia, Alagoas e Pernambuco), um do Sul (Paraná) e um do Centro-Oeste (Goiás) ocuparam o topo da lista em 2019. ^4^ ^,^ ^18^

•Um padrão decrescente similar foi observado para as taxas de YLLs padronizadas por idade por DCR, que variaram de 102,1 (II 95%, 97,5 - 107,3) por 100 mil em 1990 a 35,8 (II 95%, 33,5 - 38,4) por 100 mil em 2019, uma redução de 64,9% (II 95%, 61,6 - 67,9). ^4^ ^,^ ^18^

•As estimativas do GBD 2019 não mostraram correlação entre as taxas de DALYs padronizadas por idade e o SDI em 1990 nem em 2019. Da mesma maneira, não houve correlação entre o SDI e a variação percentual nas taxas de DALYs padronizadas por idade em 1990 (r= -0,36, p=0,06), e apenas uma fraca correlação em 2019 (r= -0,41, p=0,03) ( Figura 5 -8 ), sugerindo que os efeitos do desenvolvimento socioeconômico foram menos pronunciados na morbidade em comparação à mortalidade.

### Doença Valvar do Coração Não Reumática

•De acordo com o GBD 2019, as taxas de DALYs padronizadas por idade atribuíveis a DVNR diminuíram (30%) no Brasil, passando de 62,8 (II 95%, 60,3 – 65,2) por 100 mil em 1990 para 44 (II 95%, 40,7 - 47) por 100 mil em 2019 (Tabela 5-4 e Figura 5 -9 .B). O padrão de redução observado no período foi similar para homens (-31%) e mulheres (-28%). Com relação às doenças específicas, as taxas diminuíram igualmente para a doença valvar mitral degenerativa [-30,7% (II 95%, -41,6 a -22,6)] em comparação à doença valvar aórtica calcífica [-30,3% (II 95%, -36,3 a -21,3)]. Para outras DVNR, as taxas padronizadas por idade tenderam ao aumento [19,1% (II 95%, -21,1 – 68,5)], embora os II fossem consideravelmente amplos, incluindo 0. As tendências observadas para os YLLs foram similares. ^4^ ^,^ ^18^

•A tendência decrescente foi relativamente homogênea em todos os estados brasileiros e as taxas de DALYs padronizadas por idade permaneceram mais altas nas regiões Sul e Sudeste em todo o período, assim como em Pernambuco, estado da região Nordeste (5 ^o^ lugar em 2019) ( Figura 5 -9 .B. A redução mais significativa foi observada nas regiões Sudeste e Centro-Oeste (Distrito Federal e estados de São Paulo, Minas Gerais, Goiás e Rio de Janeiro), assim como em Rondônia, na região Norte, que não tem dados primários para a maioria das estimativas. ^4^ ^,^ ^18^

•À semelhança do observado para mortalidade, as taxas decrescentes de DALYs padronizadas por idade devidas a DVNR contrastam com o discreto aumento nas taxas brutas [7,3% (II 95%, 0 - 15,1)] no período (1990 – 2019), influenciadas pelo grupo etário >70 anos (+5%), e mais uma vez sugerindo que a morbidade associada com DVNR esteja se deslocando para os idosos, possivelmente em resposta às mudanças na composição etária da população. ^4^ ^,^ ^18^

•As taxas de DALYs proporcionais no Brasil aumentaram e, de 1990 a 2019, as regiões Sul e Sudeste foram responsáveis pelas maiores proporções de DALYs no período, de acordo com as estimativas do GBD. ^201^ ^,^ ^202^

•Ainda segundo dados do GBD 2019, houve significativas correlações positivas entre as taxas de DALYs padronizadas por idade por DVNR em geral e o SDI em 1990 (r=0,80, p<0,001) e em 2019 (r=0,55, p<0,001) ( Figura 5 -10 ). As variações percentuais nas taxas de DALYs padronizadas por idade (1990 – 2019) também se correlacionaram com o SDI em 1990 (r= -0,72, p<0,001) e em 2019 (r= -0,72, p<0,001). Para a doença valvar aórtica calcífica, foram observadas correlações significativas entre DALYs e SDI em 1990 (r^2^=0,80, p<0,001) e em 2019 (r^2^=0,62, p<0,001), assim como entre as variações percentuais nas taxas de DALYs e o SDI nos dois anos, sugerindo que o desenvolvimento socioeconômico também seja um determinante de DVNR degenerativa, ligado ao envelhecimento e aos fatores de risco.

•Para a doença valvar mitral degenerativa, observou-se correlação positiva entre as taxas de DALYs padronizadas por idade e o SDI em 1990, mas não em 2019, enquanto as variações percentuais nas taxas de DALYs correlacionaram-se negativamente com o SDI em 1990 e em 2019, sugerindo algum impacto dos marcadores socioeconômicos também sobre esta condição.

## Complicações e Doenças Associadas

### Arritmias Associadas com Doença Valvar

•Para pacientes com doença cardíaca valvar, a FA é um fator complicador, ocorrendo, em geral, naqueles com a história natural da doença mais avançada. Acha-se mais comumente associada à doença valvar mitral, em especial estenose mitral. A FA foi observada em 34% de uma coorte de 427 pacientes (idade média, 50±16 anos; 84% mulheres) com estenose mitral grave, tendo sido mais frequente naqueles que morreram durante o acompanhamento (27 de 41; 66%) em comparação aos que sobreviveram (114 de 378; 30%), reforçando seu papel de marcador de prognóstico na doença valvar cardíaca. ^203^

•Além disso, a FA pode se desenvolver na doença valvar aórtica grave, especialmente em pacientes mais idosos e no pós-operatório. Em uma coorte retrospectiva de 348 pacientes (idade média, 76,8±4,6 anos), FA pós-operatória foi observada em 114 (32,8%), mas as taxas foram mais altas a partir de 80 anos em comparação ao grupo etário de 70-79 anos (42,9% vs. 28,8%, p=0,017). ^204^

•Em outra avaliação retrospectiva conduzida em Pernambuco (Nordeste do Brasil), envolvendo 491 pacientes consecutivos após cirurgia por doença valvar do coração, a incidência de FA foi 31,2% e associada com idade >70 anos (OR=6,82; IC 95%, 3,34 - 14,10, p <0,001), doença valvar mitral (OR=3,18; IC 95%, 1,83 - 5,20, p<0,001) e a não utilização de betabloqueadores no período pós-operatório, entre outros fatores. ^205^

•Doença valvar do coração (17,5%) e arritmias (FA e *flutter* atrial – 50,7%) foram as principais fontes cardioembólicas para acidente vascular cerebral em estudo que envolveu 256 pacientes (60,2 ± 6,9 anos, 132 homens) na região Sul do Brasil. ^206^

•No registro BYPASS, uma coorte multicêntrica de pacientes submetidos a cirurgia valvar cardíaca, as complicações pós-operatórias mais frequentes foram arritmias (22,6%), infecções (5,7%) e síndrome de baixo débito (5,1%). ^198^

### Associação entre Doença Valvar do Coração e Doença Arterial Coronariana

•Devido ao elevado risco cirúrgico da combinação de procedimentos valvares e revascularização coronária, é essencial que se reconheça a prevalência de DAC obstrutiva em associação com doença valvar do coração. Estudos mostraram menor prevalência de DAC em pacientes com DCR em comparação àqueles com DVNR, possivelmente como reflexo da menor mediana de idade dos pacientes com DCR e da maior prevalência de fatores de risco coronariano na DVNR. ^207^

•Em estudo no Rio de Janeiro incluindo 1.412 candidatos a cirurgia cardíaca por qualquer indicação, 294 portadores de doença cardíaca valvar primária de etiologia reumática e não reumática foram selecionados. Todos os 294 pacientes tinham idade ≥40 anos e foram submetidos a angiografia coronária. As prevalências de DAC em pacientes com DCR e DVNR foram 4% e 33,6% (p <0,0001), respectivamente. Características e fatores de risco, como idade, dor precordial típica, hipertensão, diabetes mellitus e dislipidemia, foram significativamente associados com DAC obstrutiva. ^208^

•Em outro estudo no Brasil que avaliou 712 pacientes com doença valvar do coração e idade média de 58±13 anos, a incidência de DAC obstrutiva foi 20%. Entretanto, nos mais jovens (<50 anos), a prevalência foi mais baixa (3,3%). ^209^ Esses dados são similares aos observados em outro estudo que incluiu 3.736 pacientes (idade média de 43,7 anos), nos quais a prevalência de DAC obstrutiva combinada com doença valvar do coração foi 3,42%. ^207^

## Utilização e Custo da Atenção à Saúde

•De acordo com a base de dados administrativos do SUS, o total de despesas brutas (reembolso) com hospitalização para tratamento clínico de doença valvar do coração no Brasil aumentou significativamente em 90%, passando de R$ 1.051.959 em 2008 para R$ 1.999.540 em 2019, em um padrão quase linear. Ajustando e convertendo esses valores para dólares internacionais de 2019, o total de custos no sistema público de saúde para hospitalização por condições valvares foi de $ 1.031. 953, em 2008, e $ 966.428, em 2019, uma redução de 6,3%. ^210^

•Da mesma forma, os custos não ajustados associados com procedimentos cirúrgicos/intervencionistas valvares (códigos relacionados com cirurgia valvar, comissurotomia mitral percutânea e outros tipos de valvuloplastia) também aumentaram de 2008 a 2019, passando de R$ 130.588.598 (2019 Int$ 128.105.083) para R$ 190.771.771 (2019 Int$ 92.204.819), embora com menor magnitude em comparação àqueles de internações clínicas (46% vs. 90%). Após ajuste para Int$, observou-se uma significativa redução de 28,0%. O total de despesas com procedimentos cirúrgicos para o SUS nessa série temporal (2008 – 2019) foi R$ 10.524.044.511 (Int$ 6.853.635.725) (Tabela 5-5 e Figura 5 -11 ). ^210^

•O número de internações para cirurgias/intervenções relacionadas às doenças valvares não cresceu muito no Brasil de 2008 a 2019, variando de 12.679 em 2008 a 14.294 em 2019. Isso está provavelmente associado com a complexidade e os custos crescentes das intervenções (em especial, custos hospitalares, dispositivos e próteses) e denota a carga econômica imposta pela incorporação de novos procedimentos e tecnologias, além do efeito marcante da inflação sobre os custos da atenção à saúde – considerando-se os valores mais baixos em 2019 quando ajustados para Int$. Nesse cenário, a futura incorporação de terapias bem estabelecidas ainda não reembolsadas pelo SUS, como a TAVI, contribuirá para aumentar a carga econômica, embora as despesas com demandas judiciais possam facilmente exceder os custos ordinários. ^211^

•O número total de internações por doença valvar (clínica e cirúrgica) nesse período foi de 196.922, tendo a maioria ocorrido na região Sudeste (41,2%), seguida pelas regiões Nordeste (25,7%), Sul (20,2%), Centro-Oeste (7,5%) e Norte (5,4%) (Tabela 5-5). ^210^

•Observou-se queda dramática em alguns tipos de procedimentos, a despeito de suas crescentes indicações, como a comissurotomia mitral percutânea. Especificamente para esse procedimento, os números decrescentes podem estar associados com o atraso nas tabelas de reembolso do SUS, limitando o número de hospitais que realizam essa intervenção. O número absoluto de cirurgias valvares abertas permaneceu estável no período, variando de 12.201 em 2008 a 12.771 em 2019, a despeito do crescente número de casos de doença valvar do coração – em especial DVNR – e da crescente carga para os idosos, à medida que a população envelhece. ^190^ ^,^ ^211^

•Em nenhum dos períodos, o aumento no número de internações esteve em paralelo com as despesas crescentes, sugerindo uma progressiva complexidade – e, consequentemente, custo – dos procedimentos para o tratamento da doença valvar cardíaca, além da inflação dos dispositivos médicos e dos custos hospitalares associados (considerando os valores em Int$) (Tabela 5-5 e Figura 11 ). ^210^

•A partir da base de dados administrativos do SUS, os procedimentos valvares associados com sequelas de DCR não puderam ser diferenciados daqueles associados com outras etiologias, devido à ausência de código específico disponível, além da imprecisão da notificação dos códigos da CID. ^210^

•É interessante notar que estudos observacionais enfatizaram que a DCR permanece sendo a principal etiologia associada com cirurgia cardíaca em jovens no Brasil, atingindo até 60% em um estudo realizado em Salvador, Bahia. ^178^ No Instituto do Coração de São Paulo, o número de cirurgias cardíacas valvares associadas com DCR aumentou substancialmente nos últimos 10 anos, de cerca de 400 cirurgias/ano em 1990 para mais de 600 após 2000. ^121^ Entre 2008 e 2015, houve 26.054 hospitalizações por sequelas de FRA, 45% das quais por doença cardíaca, levando a um custo total possivelmente subestimado de US$ 3,5 milhões ao ano. ^177^ ^,^ ^210^

•Ainda de acordo com estudos observacionais e registros de base hospitalar, as doenças valvares de origem reumática, em geral, são responsáveis por cerca de 90% das cirurgias cardíacas em crianças e por mais de 30% das cirurgias cardíacas em adultos, a maioria jovem. ^135^ Entretanto, poucos estudos epidemiológicos estimaram a carga da doença valvar por causa específica no Brasil.

•O rastreamento ecocardiográfico para DCR latente mostrou-se custo-efetiva em um estudo conduzido no Brasil. Uma estratégia baseada em divisão de tarefas, com aquisição de imagem por não médicos, utilizando dispositivos portáteis, e telemedicina para interpretação remota por especialistas, resultou em uma razão de custo-efetividade incremental de $10.148,38 por DALY evitado, abaixo do limiar estimado de 3 vezes o produto interno bruto per capita, sugerido pela Organização Mundial da Saúde. ^212^

•Informação obtida na base de dados do SUS (DATASUS) mostrou que, de 2008 a 2017, houve 42.720 e 78.966 hospitalizações por FRA e DCR crônica, respectivamente, representando 0,4% e 0,7% das hospitalizações cardiovasculares no país, respectivamente. Essa análise, no entanto, não tem dados específicos para DVNR. ^213^

### Doença Valvar Mitral

•Com base em dados administrativos do SUS de 2001 a 2007 e referentes a cirurgia de valva mitral, em uma série retrospectiva de 78.808 pacientes cirúrgicos consecutivos, a idade média foi de 50,0 anos (35,9 – 62,5) e 40.106 eram mulheres (50,9%). Novamente, a DCR foi a principal etiologia, responsável por 53,7% do total de pacientes submetidos a cirurgia e a mais de 94% daqueles submetidos a procedimentos para estenose mitral, que representou a maior indicação cirúrgica única, correspondendo a 38,9% do total. No geral, a substituição valvar foi realizada em 69,1% das cirurgias. A mortalidade hospitalar foi 7,6%. ^197^

•A mortalidade cirúrgica foi discretamente maior em mulheres do que em homens (7,8% vs. 7,3%; p <0,001) e consideravelmente mais alta nos indivíduos com idade ≥80 anos. Por outro lado, a mais baixa mortalidade foi observada naqueles entre 20 e 39,9 anos (p <0,001). Os pacientes com cirurgias aórtica e mitral combinadas (refletindo etiologia reumática) foram os mais jovens (mediana, 43,3 anos). A cirurgia para estenose aórtica foi mais comum em indivíduos mais idosos (mediana, 58,0 anos) (p <0,001). Reparo valvar apresentou menor mortalidade (3,5%) em comparação a substituição valvar (6,9%), reparo/substituição valvar múltipla (8,2%) e CRVM concomitante (14,6%) (p<0,001). Associação com CRVM ocorreu em 7.147 pacientes (9,1% da amostra). ^197^

•Quanto à comissurotomia percutânea, estudos no Brasil mostraram uma proporção muito maior de mulheres (85%) – coincidente com a epidemiologia da DCR e principalmente da estenose mitral – e de jovens (<40 anos). ^214^ ^,^ ^215^ À medida que se desenvolve *expertise* local nesse procedimento, investigam-se aspectos técnicos, como a utilização rotineira de sedação consciente (baixa dose de midazolam e fentanil), resultando em ansiólise e analgesia, sem efeitos hemodinâmicos. ^216^ Além disso, há esforços contínuos para definir preditores de desfechos clínicos de longo prazo, como alterações na complacência atrioventricular invasiva e não invasiva. ^217^

•Em um estudo retrospectivo visando avaliar o reparo da valva mitral em 54 crianças brasileiras (<16 anos) com DCR crônica precoce, não houve relato de morte peroperatória. Os desfechos tardios mais frequentes (>7 dias) foram lesão mitral residual (n=11) e necessidade de reoperação (n=3). Portanto, o reparo da valva mitral permanece sendo uma estratégia razoável para DCR em idades mais jovens. ^218^

### Doença Valvar Aórtica

•Uma coorte de 724 pacientes consecutivos, submetidos a cirurgia cardíaca no Instituto do Coração de São Paulo, evidenciou, à semelhança de outros estudos, maior taxa de mulheres (55%) e predominância de DCR (60%). Nessa série, entretanto, houve maior proporção de doença valvar aórtica (396 casos) do que de doença valvar mitral (306 casos) em comparação a outras séries. Nos pacientes com doença valvar mitral, observaram-se estenose em 39,9%, regurgitação em 38,4% e disfunção de prótese mitral em 21,7%. Naqueles submetidos a intervenção valvar aórtica, observaram-se estenose em 51,6%, regurgitação em 29,3% e disfunção de prótese em 19,1%. O estudo sugere um aumento de doença valvar aórtica em comparação à doença valvar mitral em um hospital terciário na região Sudeste do Brasil. ^219^

•Outro estudo retrospectivo de coorte foi conduzido em Porto Alegre (Sul do Brasil), com 1.065 pacientes (idade média, 61,4 ± 11,8 anos; 38% mulheres). Substituição de valva aórtica foi realizada em 18,8% e de valva mitral, em 13,4%. Revascularização coronária concomitante foi realizada em 60,3% da amostra e cirurgias valvares, em 32,7%.

A mortalidade hospitalar geral foi 7,8%, sendo menor para CRVM isolada (5,9%), intermediária para cirurgia valvar (aórtica e/ou mitral e/ou tricúspide = 8,6%) e maior para a combinação de procedimentos valvares e CRVM (20,0%). ^220^

### Implante Percutâneo de Valva Aórtica no Brasil

•Como em outros países, a TAVI ganhou importância no Brasil nos últimos 20 anos. Estima-se que mais de 100 mil TAVI já foram realizadas no mundo. ^196^ ^,^ ^211^ A primeira TAVI no Brasil ocorreu em 2008. O registro brasileiro de TAVI informa a realização de 418 TAVI em 18 centros até 2014, tendo esse número crescido exponencialmente desde então. O acesso femoral foi escolhido em 96,2% dos procedimentos e as próteses usadas foram CoreValve ^®^ (86,1%) e Sapien XT ^®^ (13,9%). Nessa experiência inicial, as mortalidades por todas as causas em 30 dias e 1 ano foram 9,1% e 21,5%, respectivamente. ^221^

•Dados do registro de TAVI atualizados em 2017 revelaram um total de 819 pacientes em acompanhamento clínico, demonstrando que o procedimento tem baixa incidência de complicações – em especial desfechos clínicos duros precoces – e ressaltando taxas de disfunção renal pós-procedimento em torno de 18%. ^222^ ^,^ ^223^

•Em outra avaliação realizada na cidade do Rio de Janeiro com 136 pacientes submetidos a TAVI [mediana de idade, 83 (80-87) anos; 51% homens], a mortalidade peroperatória foi 1,5%, a mortalidade de 30 dias, 5,9%, a mortalidade hospitalar, 8,1% e a mortalidade de 1 ano, 15,5%. ^224^

•De 819 valvas aórticas implantadas por via percutânea até 2017, 135 pacientes (20,1%) precisaram de marca-passo permanente. Esses pacientes eram mais velhos (82,5 vs. 81,1 anos; p=0,047), predominantemente homens (59,3% vs 45%; p=0,003) e tinham bloqueio de ramo direito prévio (OR=6,19; IC 95%, 3,56 – 10,75, p≤0,001). O uso da prótese CoreValve ^®^ (OR=3,16; IC 95%, 1,74 – 5,72, p≤0,001) e gradiente transaórtico basal >50 mmHg (OR=1,86; IC 95%, 1,08 – 3,20, p=0,025) foram preditores independentes de implantação de marca-passo permanente. ^223^

### Pesquisa Futura

•Apesar da notável melhora nas últimas décadas, ainda há escassez de dados primários sobre a epidemiologia da doença valvar do coração no Brasil e muito espaço para pesquisa futura.

•Deve-se incluir a coleta de dados administrativos, com o desenvolvimento de codificação específica que permita a discriminação de variáveis, como a valva envolvida, o tipo de disfunção valvar, o tipo de prótese e, principalmente, a etiologia e a associação com doenças sistêmicas. Isso é de especial importância no SUS.

•Além disso, o desenvolvimento em âmbito nacional de registros sobre doença valvar e procedimentos é necessário. O refinamento do sistema de codificação e a implementação mandatória de relatórios clínicos e cirúrgicos – como feito anteriormente para as intervenções coronárias percutâneas – podem ser um passo inicial para melhorar a acurácia na aquisição de dados.

•Como o país tem algumas coortes significativas de pacientes com doença valvar do coração, o acompanhamento de médio e longo prazo dessas amostras deve ser garantido. Importante notar que há iniciativas de pesquisa que requerem incentivos e financiamento para sua continuação, como os estudos em andamento sobre prognóstico de longo prazo de DCR subclínica em crianças e adolescentes, ^176^ ^,^ ^192^ determinantes genéticos e imunológicos da resposta às infecções estreptocócicas que leva a DCR, ^225^ preditores clínicos e relacionados ao procedimento de eventos a curto e longo prazo após comissurotomia mitral percutânea, ^203^ ^,^ ^226^ além de um registro nacional de TAVI. ^221^

•Como um estudo sugere que o rastreamento ecocardiográfico para DCR seja custo-efetivo no Brasil, ^212^ sua aplicação fora do contexto de pesquisa e sua integração no sistema de saúde deveriam ser investigadas em programas de larga escala.

•Além disso, esforços continuados foram direcionados para o desenvolvimento de vacinas contra infecções estreptocócicas, ^225^ devendo-se garantir estudos colaborativos sobre sua eficácia e aplicação clínica para reduzir a carga de DCR.

•Como o reembolso para TAVI acaba de ser aprovado no sistema privado de saúde brasileiro, sua incorporação no SUS parece próxima, ^211^ sendo que a avaliação do seu verdadeiro impacto clínico, orçamentário e social nos desfechos da atenção à saúde pública requer extensa pesquisa e financiamento.

•Finalmente, outras estratégias promissoras para prover diagnóstico precoce e priorizar referenciamentos em áreas com poucos recursos devem ser investigadas no Brasil. Como exemplo, a disponibilidade de modalidades de exames de imagem para o manejo da doença valvar do coração – em especial ecocardiografia – é limitada e distribuída de maneira desigual no país. Nesse cenário, avaliou-se a implementação de tele-ecocardiografia, com a divisão de tarefas e aquisição de imagem por não médicos (ainda não permitida fora do contexto de pesquisa pela regulamentação da atenção à saúde no Brasil) e sua leitura remota. ^227^ Apesar do seu bom desempenho para diagnóstico e discriminação de pacientes com alto risco cardiovascular, ^227^ o impacto sobre os desfechos clínicos e a custo-efetividade da estratégia ainda necessitam avaliação.

•Há espaço para aprimorar o diagnóstico cardíaco remoto no Brasil, através da expansão do tele-ECG, do rastreio de FA, ^228^ das consultas remotas – incluindo aquelas para condições infecciosas, como a COVID-19 ^229^ – e da incorporação das inovações em técnicas de imagem para melhorar o acesso ao cuidado cardiovascular. São necessárias extensas discussões baseadas em robustas evidências científicas.

## 6. FIBRILAÇÃO ATRIAL E *FLUTTER* ATRIAL

### CID-10 I48

**Ver Tabelas 6-1 até 6-5 e Figuras 6-1 até 6-3**

**Table:** Abreviaturas usadas no Capítulo 6

AIT	Ataque Isquêmico Transitório
AVC	Acidente Vascular Cerebral
AVK	Antagonista da Vitamina K
BNP	Peptídeo Natriurético Cerebral
CID-10	Classificação Estatística Internacional de Doenças e Problemas Relacionados à Saúde, 10 ^a^ Revisão
CRVM	Cirurgia de Revascularização Miocárdica
DALYs	Anos de vida perdidos ajustados por incapacidade (do inglês, *Disability-Adjusted Life-Years* )
DCh	Doença de Chagas
ECG	Eletrocardiograma
ELSA-Brasil	Estudo Longitudinal da Saúde do Adulto
FA	Fibrilação Atrial
GARFIELD-AF	Global Anticoagulant Registry in the FIELD-AF
GBD	Global Burden of Disease
HR	Hazard Ratio
IC	Intervalo de Confiança
II	Intervalo de Incerteza
IMPACT-AF	A Multifaceted Intervention to Improve Treatment with Oral Anticoagulants in Atrial Fibrillation
INR	Índice Internacional Normalizado (do inglês, *International Normalized Ratio* )
NOAC	Novos Anticoagulantes Orais
OR	Odds Ratio
PPC	Paridade do Poder de Compra
SDI	Índice Sociodemográfico (do inglês, *Sociodemographic Index* )
SUS	Sistema Único de Saúde
TTR	Tempo na Faixa Terapêutica (do inglês, *Time in Therapeutic Range* )
UF	Unidade Federativa
YLDs	Anos vividos com incapacidade (do inglês, *Years Lived with Disability* )
YLLs	Anos de vida perdidos por morte prematura (do inglês, *Years of Life Lost)*

**Tabela 6-1:** – Número de casos prevalentes e taxas de prevalência padronizadas por idade (por 100 mil habitantes) por fibrilação atrial e flutter em 1990 e 2019, com variação percentual das taxas, por sexo e grupo etário, no Brasil.

Sexo e idade	1990		2019		Variação percentual
	Número (II 95%)	Taxa (II 95%)	Número (II 95%)	Taxa (II 95%)	(II 95%)
**Ambos os sexos**					
15-49 anos	19888 (12331,5;30044)	25,9 (16,1;39,2)	42502,2 (26157,4;64165,2)	36,8 (22,6;55,6)	41,8 (37,2;46,9)
50-69 anos	164660,9 (115683,1;228396,1)	1049,6 (737,4;1455,9)	444122,5 (311101;613445,2)	1100,9 (771,1;1520,6)	4,9 (2,9;6,8)
70+ anos	217157,3 (158121,9;292116,4)	5133,7 (3738;6905,7)	740228,7 (542333,8;984791,4)	5655,6 (4143,6;7524,1)	10,2 (6,8;15)
Padronizada por idade	401706,1 (302349,2;518702,6)	519,4 (393;668,6)	1226853,4 (934018,3;1577999,6)	537,3 (409,2;692,5)	3,5 (1,8;5,1)
Todas as idades	401706,1 (302349,2;518702,6)	269,9 (203,1;348,5)	1226853,4 (934018,3;1577999,6)	566,2 (431,1;728,3)	109,8 (103,4;117,4)
**Mulheres**					
15-49 anos	7612,1 (4581,8;11533,4)	19,6 (11,8;29,6)	16625,9 (10080,7;25447,2)	28,4 (17,2;43,5)	45,4 (38,6;52,3)
50-69 anos	68973,4 (48140,1;96070)	845 (589,8;1177)	191525,6 (133320,6;267053,6)	894,4 (622,6;1247,1)	5,8 (3;8,5)
70+ anos	105220,9 (76305,5;141439,1)	4486,9 (3253,9;6031,4)	379236,7 (275610,2;508021,7)	5022,5 (3650,1;6728,1)	11,9 (7,5;18)
Padronizada por idade	181806,4 (136384,7;235600,5)	437,3 (331,2;566,3)	587388,3 (445578,6;762442,3)	454,9 (345,1;591,9)	4 (1,9;6,5)
Todas as idades	181806,4 (136384,7;235600,5)	241,5 (181,2;313)	587388,3 (445578,6;762442,3)	529,8 (401,9;687,7)	119,4 (111,3;129,4)
**Homens**					
15-49 years	12275,9 (7602,8;18468,4)	32,6 (20,2;49)	25876,3 (16054,7;38681,1)	45,4 (28,2;67,9)	39,4 (33,9;45,1)
50-69 years	95687,5 (67028,6;132303,1)	1271,6 (890,7;1758,1)	252596,9 (176982;344940,7)	1334,5 (935;1822,3)	4,9 (2,3;7,6)
70+ years	111936,4 (81555,8;150612,8)	5938,2 (4326,5;7990)	360992 (264762;481432)	6518,7 (4781;8693,6)	9,8 (6,4;14)
Age-standardized	219899,7 (164842,2;283209,5)	618,6 (468,5;792,3)	639465,2 (486071,9;821088,6)	643,4 (489,2;828,7)	4 (1,9;6)
All Ages	219899,7 (164842,2;283209,5)	298,9 (224,1;385)	639465,2 (486071,9;821088,6)	604,4 (459,4;776,1)	102,2 (96,2;108,8)

*Fonte: Dados derivados do estudo Global Burden of Disease 2019, Institute for Health Metrics and Evaluation, University of Washington. ^*46*^*

**Tabela 6-2:** – Número de casos prevalentes e taxas de prevalência padronizadas por idade (por 100 mil habitantes) por fibrilação atrial e flutter em 1990 e 2019, com variação percentual das taxas, no Brasil e suas unidades federativas

Local	1990		2019		Variação percentual
	Número (II 95%)	Taxa (II 95%)	Número (II 95%)	Taxa (II 95%)	(II 95%)
Acre	658 (494,3;846,9)	477,6 (363,8;609,8)	2719,6 (2059,4;3482,1)	495,6 (375,3;633,2)	3,8(-1,2;8,8)
Alagoas	5737,2 (4311,7;7420,5)	480,6 (364,9;617,3)	14985,3 (11478;19154,2)	496,6 (379,7;636,9)	3,3(-1,9;7,6)
Amapá	382,2 (289,6;491,2)	460 (350,2;590,3)	2167,2 (1660,1;2770,8)	481,3 (367,1;620,4)	4,6(-0,5;9,8)
Amazonas	3032,8 (2269,6;3906,7)	474,9 (360,2;611,7)	12830,4 (9829,7;16439,3)	492,9 (376,6;632,3)	3,8(-1;9)
Bahia	29515,5 (22145,1;37849,7)	481,3 (364,7;615,4)	78718,3 (60254,3;100714,7)	496,7 (377,6;636,5)	3,2(-1,3;7,9)
Ceará	18182,8 (13882,4;23457,1)	473 (361,6;609,1)	47310,1 (35919,5;61015,3)	484,6 (367,2;624,8)	2,4(-1,9;7,3)
Distrito Federal	2005 (1503,1;2616,3)	466,1 (355;598,2)	11277,1 (8448,4;14707,1)	487,8 (371;633,6)	4,6(0;10,2)
Espírito Santo	6046,9 (4508,7;7782,4)	477 (360,7;609,5)	20591,3 (15508,9;26528)	494,1 (373,4;637)	3,6(-1,8;9)
Goiás	8112,6 (6103;10537,3)	473,8 (358,8;618,5)	31759,2 (24091,6;40630,4)	488,7 (373,2;628)	3,1(-2;8,8)
Maranhão	10154,6 (7606,6;13258,2)	445,1 (337,1;576,8)	29373,5 (22463,1;37530)	464,8 (354,9;593)	4,4(-0,6;9,4)
Mato Grosso	2967,4 (2238;3853,5)	481,9 (367,5;627)	14898,3 (11378,3;19217,4)	495,9 (376,2;641,8)	2,9(-2,8;7,8)
Mato Grosso do Sul	3568 (2677,8;4589,9)	484,9 (370,2;623,5)	13928,3 (10490,8;17874,3)	500,6 (375,5;643,5)	3,2(-1,8;7,8)
Minas Gerais	71781,4 (53528,5;93821,9)	831,3 (627,8;1079,1)	223364,2 (168276,2;291716,3)	849,1 (640,5;1103,7)	2,1(-2,2;6,6)
Pará	8234,5 (6281,7;10593,5)	464,3 (354,2;598,1)	31143,2 (23903;39996,1)	485,2 (370,9;626)	4,5(-0,6;9,4)
Paraíba	10701,6 (8052,8;13746,5)	480,4 (363,4;614,6)	23515,3 (17936,4;30396,6)	489,9 (372,3;633,8)	2(-3,8;7,4)
Paraná	19787,5 (14787;25568,1)	487,8 (370,8;626,5)	64224,8 (48888,4;83035,5)	502,7 (383,3;651,8)	3(-1,4;8,1)
Pernambuco	19150,2 (14364,7;24937,2)	463,9 (351,7;600,9)	46115,3 (35058;59389,5)	480,9 (366,5;621,5)	3,7(-1,2;9,9)
Piauí	5728,6 (4318,8;7395,8)	463,3 (353,4;598,2)	18024 (13731,2;23287,2)	479,1 (364,2;620,3)	3,4(-1,1;8,2)
Rio de Janeiro	39769,7 (29930,9;51462,6)	475,4 (358,3;613,4)	109091,6 (82434,2;141021,5)	492 (373,7;634,7)	3,5(-0,9;8,6)
Rio Grande do Norte	7220,3 (5485,8;9275,2)	473,5 (362,3;608,7)	18814,8 (14363,2;24105,1)	489,4 (373,6;630,9)	3,4(-1,1;7,9)
Rio Grande do Sul	27957,6 (20979,2;36240,9)	486,8 (370,7;628,7)	78012,2 (58643;101476,3)	499,6 (376,5;646,6)	2,6(-1,9;7,8)
Rondônia	1196,4 (871,8;1563,9)	477,4 (358,7;611)	6770,9 (5121,3;8697,4)	487,5 (367,9;627)	2,1(-2,6;7)
Roraima	201,9 (151,7;260,8)	476,4 (364,9;615,4)	1589,6 (1203,7;2062,2)	495,4 (377,6;632,2)	4(-0,2;8,6)
Santa Catarina	10427,4 (7849,1;13416,7)	489,4 (370,8;623,4)	38974 (29337,7;50470,5)	504,9 (384,6;648,6)	3,2(-2;8,2)
São Paulo	84287,7 (63124,6;108978,1)	495,8 (379,2;636,4)	269601,2 (204582,8;347991,5)	511,2 (388,9;664)	3,1(-2,8;8,2)
Sergipe	3387,4 (2523,5;4411,4)	480,9 (364,7;627,1)	10672,7 (8098,7;13831,9)	502 (380,7;652,5)	4,4(0,3;9,3)
Tocantins	1510,8 (1130;1956,4)	452,5 (345,4;588)	6381 (4850,7;8197,6)	476,1 (363,6;612,1)	5,2(0,3;10,9)
Brasil	401706,1 (302349,2;518702,6)	519,4 (393;668,6)	1226853,4 (934018,3;1577999,6)	537,3 (409,2;692,5)	3,5(1,8;5,1)

*Fonte: Dados derivados do estudo Global Burden of Disease 2019, Institute for Health Metrics and Evaluation, University of Washington. ^46^*

**Tabela 6-3:** – Número de mortes e taxas de mortalidade padronizadas por idade (por 100 mil habitantes) por fibrilação atrial e flutter em 1990 e 2019, com variação percentual das taxas, por sexo e grupo etário, no Brasil

Sexo e idade	1990		2019		Variação percentual
	Número (II 95%)	Taxa (II 95%)	Número (II 95%)	Taxa (II 95%)	(II 95%)
**Ambos os sexos**					
15-49 anos	57,3 (48,3;76,1)	0,1 (0,1;0,1)	118,1 (91,1;138,6)	0,1 (0,1;0,1)	36,7 (7,7;52,4)
50-69 anos	432,5 (370,3;566,3)	2,8 (2,4;3,6)	1125 (918,5;1355,3)	2,8 (2,3;3,4)	1,1 (-16;10,3)
70+ anos	2169,7 (1822,6;2747)	51,3 (43,1;64,9)	9568,3 (7433,7;11342,6)	73,1 (56,8;86,7)	42,5 (20,2;54,1)
Padronizada por idade	2659,5 (2263,6;3342,6)	4,8 (4;6)	10811,4 (8636,5;12800,8)	5 (4;6)	5,4 (-10,6;13,1)
Todas as idades	2659,5 (2263,6;3342,6)	1,8 (1,5;2,2)	10811,4 (8636,5;12800,8)	5 (4;5,9)	179,3 (133,7;202,2)
**Mulheres**					
15-49 anos	25,6 (23,2;35,2)	0,1 (0,1;0,1)	55,5 (43,6;63,2)	0,1 (0,1;0,1)	44,6 (10,2;65,6)
50-69 anos	202,6 (183,8;264,5)	2,5 (2,3;3,2)	550 (447;619,2)	2,6 (2,1;2,9)	3,5 (-15,5;15,7)
70+ anos	1278,4 (1060,8;1636,8)	54,5 (45,2;69,8)	6122,4 (4570;7074,9)	81,1 (60,5;93,7)	48,7 (21,8;65,3)
Padronizada por idade	1506,6 (1266,7;1935,4)	4,8 (4;6,2)	6727,9 (5082;7750,1)	5,2 (3,9;6)	7,6 (-11,2;19)
Todas as idades	1506,6 (1266,7;1935,4)	2 (1,7;2,6)	6727,9 (5082;7750,1)	6,1 (4,6;7)	203,2 (148,8;236,1)
**Homens**					
15-49 anos	31,8 (23,1;47,1)	0,1 (0,1;0,1)	62,6 (39,9;81,3)	0,1 (0,1;0,1)	30,3 (3,6;51,7)
50-69 anos	229,9 (168,9;336,6)	3,1 (2,2;4,5)	575 (392,4;780,2)	3 (2,1;4,1)	-0,6 (-17,5;11,1)
70+ anos	891,3 (645;1258,4)	47,3 (34,2;66,8)	3445,9 (2286;4594,7)	62,2 (41,3;83)	31,6 (12,7;45,6)
Padronizada por idade	1153 (843,8;1633,3)	4,7 (3,4;6,6)	4083,4 (2781,1;5478,5)	4,8 (3,2;6,4)	1,2 (-13;11)
Todas as idades	1153 (843,8;1633,3)	1,6 (1,1;2,2)	4083,4 (2781,1;5478,5)	3,9 (2,6;5,2)	146,3 (109,6;172,8)

*Fonte: Dados derivados do estudo Global Burden of Disease 2019, Institute for Health Metrics and Evaluation, University of Washington, ^*46*^*

**Tabela 6-4:** – Número de mortes e taxas de mortalidade padronizadas por idade (por 100 mil habitantes) por fibrilação atrial e *flutter* em 1990 e 2019, com variação percentual das taxas, no Brasil e suas unidades federativas

Local	1990		2019		Variação percentual
	Número (II 95%)	Taxa (II 95%)	Número (II 95%)	Taxa (II 95%)	(II 95%)
Acre	4 (3,2;4,7)	5,1 (4;6,1)	24,7 (19,2;28,8)	5,7 (4,4;6,6)	10,7(-5,8;25,5)
Alagoas	37,4 (30,3;46,3)	4 (3,3;5)	137,2 (106,1;169,5)	4,6 (3,5;5,7)	14,7(-3,6;34,2)
Amapá	2,9 (2,2;3,3)	5,2 (4;6)	19,8 (14,1;22,9)	5,4 (3,9;6,3)	3,5(-8,1;13,7)
Amazonas	19,3 (15,9;23,8)	4,8 (3,9;5,9)	95,7 (72,8;119,1)	4 (3,1;5)	-15,9(-26,3;-5,6)
Bahia	219,2 (166,6;261)	4,3 (3,2;5,1)	801,9 (552,5;974,5)	4,8 (3,3;5,8)	11,8(-8,3;33,5)
Ceará	121,8 (89,5;156,9)	3,4 (2,5;4,4)	489,1 (375,1;592,4)	5 (3,9;6,1)	48,5(14,2;83,8)
Distrito Federal	14,3 (12,2;20)	9,2 (7,3;12,5)	104,6 (84;133,8)	8,8 (7;11,2)	-3,8(-22,1;14,1)
Espírito Santo	39,3 (33,4;50,9)	4,9 (4,1;6,5)	193,5 (154,7;236)	5,2 (4,1;6,3)	5(-10,7;17,7)
Goiás	56,1 (47,5;77,4)	5,5 (4,6;7,6)	265,9 (209,7;336,1)	4,8 (3,7;6)	-13,8(-30,5;1,2)
Maranhão	77,7 (42,2;105,3)	4,4 (2,4;6)	445,9 (240,1;554)	7,2 (3,9;9)	65,4(29,7;118)
Mato Grosso	17,7 (14,5;22,4)	4,6 (3,7;5,7)	108,3 (85,9;137,8)	4,3 (3,3;5,4)	-6,7(-18,3;6,1)
Mato Grosso do Sul	23,7 (20,1;30,2)	5,3 (4,3;6,6)	117,1 (94,2;146,4)	4,8 (3,8;6)	-9(-18,7;1)
Minas Gerais	290,7 (249,5;374,6)	5,3 (4,4;6,7)	1193,3 (889,8;1407,2)	4,6 (3,4;5,4)	-13,2(-35,8;-0,4)
Pará	63,2 (48,7;73,1)	5,5 (4,1;6,3)	267 (195,7;317,4)	4,4 (3,2;5,2)	-19,8(-30,7;-7,9)
Paraíba	74,9 (57,3;92,5)	3,8 (2,9;4,7)	221,3 (167,4;273,4)	4,1 (3,2;5,1)	9,5(-10,2;27,9)
Paraná	126,5 (108,6;178,9)	5 (4,2;7,1)	541,3 (444,9;678,3)	4,9 (4;6,1)	-1,6(-18,3;11,1)
Pernambuco	133,9 (113,2;175,8)	4,4 (3,6;5,9)	438,3 (357;561,5)	4,9 (4;6,3)	11,3(-3;25,9)
Piauí	37,9 (29,3;46,4)	4,6 (3,4;5,7)	161,3 (116,8;193,7)	4 (2,9;4,8)	-12,7(-27,6;1,3)
Rio de Janeiro	297,6 (256,7;435,5)	5,2 (4,5;7,6)	1074,4 (853,7;1362,3)	5,2 (4,1;6,6)	-0,7(-21,7;14)
Rio Grande do Norte	54,5 (41,3;66)	4 (3;4,8)	182,9 (136,5;224)	4,3 (3,2;5,3)	8,9(-13,2;29,6)
Rio Grande do Sul	189,9 (162,8;272,6)	4,7 (4;6,7)	733,7 (590,2;905,4)	4,9 (3,9;6,1)	5,3(-17,5;19,9)
Rondônia	5,3 (4,3;7,1)	6,9 (5,4;9)	55,1 (44,3;70,3)	4,7 (3,7;6,1)	-31,6(-42,5;-17)
Roraima	1,2 (0,9;1,5)	7,2 (5,6;8,6)	13,2 (10,5;15,4)	7 (5,6;8,2)	-3(-13,9;8,4)
Santa Catarina	74,8 (64,6;101,5)	5,3 (4,5;7,2)	333,7 (265,1;409,5)	5,2 (4,1;6,4)	-1,4(-19,6;11,7)
São Paulo	642,9 (547,6;820,7)	5,9 (4,9;7,4)	2630,3 (1986,8;3101,9)	5,6 (4,2;6,7)	-3,6(-21,8;9)
Sergipe	24,2 (20,1;30,2)	5,1 (4,3;6,4)	92 (70,5;113,9)	4,4 (3,4;5,5)	-14,2(-27,6;-0,9)
Tocantins	8,7 (6,5;10,6)	6 (4,6;7,3)	69,9 (51,4;84,5)	5,7 (4,2;6,9)	-4,6(-21,2;13,3)
Brasil	2659,5 (2263,6;3342,6)	4,8 (4;6)	10811,4 (8636,5;12800,8)	5 (4;6)	5,4(-10,6;13,1)

*Fonte: Dados derivados do estudo Global Burden of Disease 2019, Institute for Health Metrics and Evaluation, University of Washington. ^46^*

**Tabela 6-5:** – Número de DALYs e taxas de DALYs padronizadas por idade (por 100 mil habitantes) por fibrilação atrial e flutter em 1990 e 2019, com variação percentual das taxas, por sexo e grupo etário, no Brasil

Sexo e idade	1990		2019		Variação percentual
	Número (II 95%)	Taxa (II 95%)	Número (II 95%)	Taxa (II 95%)	(II 95%)
**Ambos os sexos**					
15-49 anos	4251 (3336,7;5517,3)	5,5 (4,4;7,2)	8855,5 (6876;11253,8)	7,7 (6;9,7)	38,3 (16,9;51,2)
50-69 anos	25126,7 (19600,3;32757,4)	160,2 (124,9;208,8)	66612,8 (51964,2;86102,4)	165,1 (128,8;213,4)	3,1 (-6,2;7,9)
70+ anos	42042,9 (34764,9;51839,4)	993,9 (821,9;1225,5)	154647,9 (126905,8;188279,2)	1181,6 (969,6;1438,5)	18,9 (7,2;25,7)
Padronizada por idade	71420,6 (58907,9;88622,2)	98,7 (81,8;121,3)	230116,3 (189167;279885,9)	102,5 (84,3;124,5)	3,9 (-6,4;8,7)
Todas as idades	71420,6 (58907,9;88622,2)	48 (39,6;59,5)	230116,3 (189167;279885,9)	106,2 (87,3;129,2)	121,3 (99,5;133,3)
**Mulheres**					
15-49 anos	1789,8 (1436,2;2324,2)	4,6 (3,7;6)	3878,6 (2980,7;4931,9)	6,6 (5,1;8,4)	44,3 (19,3;62,4)
50-69 anos	11044,7 (8663,5;14294,3)	135,3 (106,1;175,1)	30304,9 (23538,7;39110,9)	141,5 (109,9;182,6)	4,6 (-5,3;11,8)
70+ anos	22605,8 (18670,3;27843,4)	964 (796,2;1187,3)	89454,8 (70991,4;107212,8)	1184,7 (940,2;1419,9)	22,9 (8;32,8)
Padronizada por idade	35440,3 (29438,2;43933,5)	91 (75,8;111,6)	123638,3 (100891,8;148053,7)	95,8 (78;114,7)	5,3 (-7,3;12,5)
Todas as idades	35440,3 (29438,2;43933,5)	47,1 (39,1;58,4)	123638,3 (100891,8;148053,7)	111,5 (91;133,5)	136,9 (109,4;155,1)
**Homens**					
15-49 anos	2461,2 (1840,7;3237)	6,5 (4,9;8,6)	4976,9 (3658,8;6398,8)	8,7 (6,4;11,2)	33,8 (13,8;50,7)
50-69 anos	14082 (10693,3;18706,2)	187,1 (142,1;248,6)	36307,9 (27210,5;47858,4)	191,8 (143,8;252,8)	2,5 (-7,3;8,9)
70+ anos	19437,2 (15002,9;24958,6)	1031,1 (795,9;1324,1)	65193,2 (50511,1;82124,3)	1177,2 (912,1;1483)	14,2 (4;21,4)
Padronizada por idade	35980,3 (28333,3;46350,5)	107,5 (85;136,9)	106478 (82477,5;133352,6)	110,2 (85,5;137,8)	2,6 (-6,4;8,3)
Todas as idades	35980,3 (28333,3;46350,5)	48,9 (38,5;63)	106478 (82477,5;133352,6)	100,6 (78;126)	105,8 (87;117,7)

*Fonte: Dados derivados do estudo Global Burden of Disease 2019, Institute for Health Metrics and Evaluation, University of Washington. ^*46*^*

**Figura 6-1:**
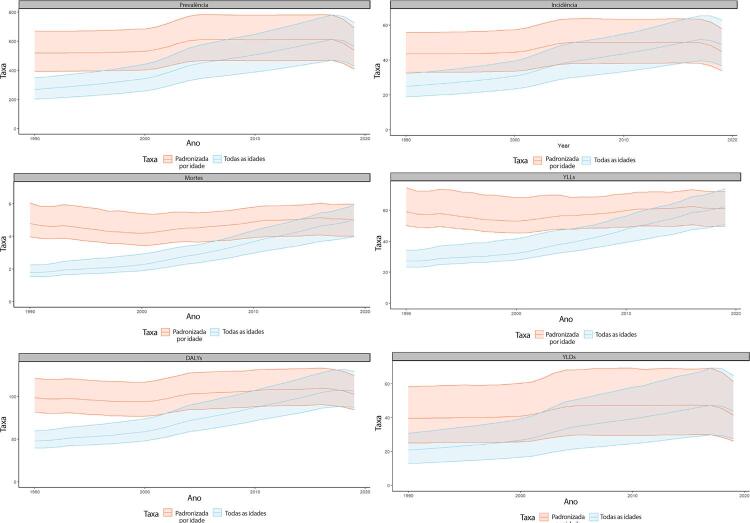
-Taxas de fibrilação/flutter atrial para todas as idades e padronizadas por idade entre 1990 e 2019, no Brasil. A. Prevalência, B. Incidência, C. Mortes, D. YLLs, D. DALYs, E. YLDs. ^46^

**Figura 6-2:**
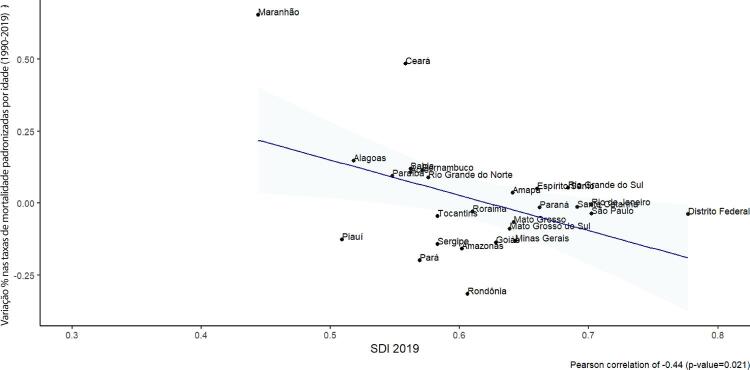
-Correlação do Índice Sociodemográfico (SDI) de 2019 com a variação percentual nas taxas de mortalidade padronizadas por idade por fibrilação atrial e flutter por 100 mil habitantes de 1990 a 2019.

Fonte: Dados derivados do estudo Global Burden of Disease 2019, Institute for Health Metrics and Evaluation, University of Washington. ^46^

**Figura 6-3:**
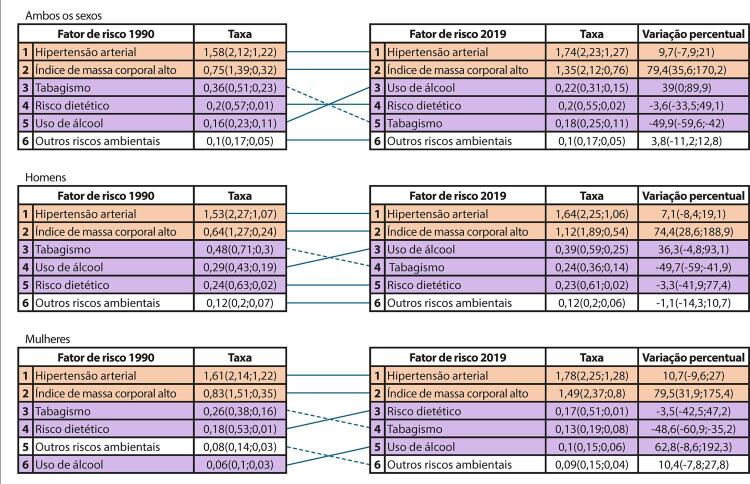
-Mortes (por 100 mil) por fibrilação atrial e flutter atribuíveis a fatores de risco no Brasil em 1990 e 2019 e variação percentual, para ambos os sexos, homens e mulheres.

Fonte: Dados derivados do estudo Global Burden of Disease 2019, Institute for Health Metrics and Evaluation, University of Washington. ^46^

### Prevalência e Incidência

•De acordo com as estimativas do Estudo GBD 2019, as taxas de prevalência de FA e *flutter* atrial padronizadas por idade apresentaram um pequeno aumento no Brasil: de 519 (II 95%, 393-669) em 1990 para 537 (II 95%, 409-692) em 2019, por 100 mil habitantes, para ambos os sexos, com variação de 3,5% (II 95%, 1,8-5,1) no período. A prevalência de FA e *flutter* atrial foi maior nos homens [em 1990, 619 (II 95%, 468-792); em 2019, 643 (II 95%, 489-829)] do que nas mulheres [em 1990, 499 (II 95%, 418-587); em 2019, 522 (II 95%, 440-610)], embora a variação percentual tenha sido similar nos dois sexos (mulheres, 4.0%; II 95%, 1,9-6,5; e homens, 4,0%; II 95%, 1,9-6,0) no período. Em números absolutos, as estimativas de prevalência de FA e *flutter* atrial no Brasil subiram de 0,4 milhão em 1990 para 1,2 milhão em 2019, principalmente devido ao crescimento e envelhecimento da população (Tabela 6-1 e Figura 6-1 ), como sugerido pelas crescentes taxas de prevalência bruta. Em 2019, a proporção de indivíduos com FA prevalente foi de 0,59% (II 95%, 0,45-0,76). Ao estratificar pelas UF brasileiras, a taxa de prevalência de FA e *flutter* atrial é similar na maioria das UF brasileiras, exceto em Minas Gerais, cuja taxa é quase o dobro daquela dos outros estados (Tabela 6-2). Importante ressaltar que Minas Gerais é o único estado para o qual o *Institute for Health Metrics and Evaluation* tem dados primários, o que pode explicar essa diferença e sugerir que a prevalência de FA possa ser ainda maior no Brasil do que estimado pelo GBD. ^46^

•Com relação à taxa de incidência padronizada por idade por 100 mil por-ano, as estimativas do Estudo GBD 2019 foram 44 (II 95%, 33-56) em 1990 e 45 (II 95%, 34-58) em 2019, também maior para os homens nos dois períodos [mulheres, 1990: 37 (II 95%, 28-48) e 2017: 39 (II 95%, 29-50); homens: 1990: 50 (II 95%, 38-64) e 2017: 52 (II 95%, 39-67)].

•Dados de estudos de base populacional mostraram prevalência variando de 0,3% a 2,4%. No estudo de coorte ELSA-Brasil, que incluiu 14.424 adultos com ECG válidos (45,8% homens, faixa etária: 35-74 anos), a prevalência de FA e *flutter* atrial foi 0,3% (homens, 0,5%; mulheres, 0,2%), sendo maior na faixa etária de 65-74 anos (mulheres: OR, 17; 95% CI, 2,1-135,9; homens: OR, 52,3; 95% CI, 3,1-881,8). Não houve diferença na prevalência de acordo com a raça autorreferida, para os dois sexos. ^230^ Em estudo transversal com 1.524 idosos em São Paulo, a prevalência de FA ou *flutter* atrial foi 2,4% (homens 3,9%; mulheres, 2,0%). ^231^

•Os centros de telessaúde no Brasil forneceram informação sobre a prevalência de FA e *flutter* atrial com base em ECG da atenção primária. ^232–235^ No conjunto de dados sobre ECG do sistema de telessaúde de Minas Gerais, que incluiu 1.558.421 indivíduos (idade média, 51±18 anos; 40,2% homens) com ECG realizado entre 2010 e 2017, a prevalência de FA foi 1,33%, maior em homens (1,81% vs. 1,02%) e aumentando com a idade (OR 1,08, CI 95% 1,08-1,08), chegando a 7,0% nos octogenários (8,4% em homens vs. 5,9% em mulheres). ^236^

•Dados de 676.621 ECG (idade média, 51±19 anos; 57,5% mulheres) realizados de 2009 a 2016 foram analisados no serviço de telemedicina da Universidade Federal de São Paulo, revelando uma prevalência de FA em 7 anos de 2,2% e uma prevalência de FA projetada para 2025 no Brasil de 1,7%. ^234^

•O registro de base hospitalar GARFIELD-AF incluiu pacientes ≥18 anos com FA diagnosticada nas 6 semanas anteriores e pelo menos um fator de risco adicional para AVC. No Brasil, 41 *sites* incluíram 1.065 pacientes com FA não valvar entre 2010 e 2014 (idade média, 68±13 anos; 55% homens). A prevalência dos tipos de FA foi a seguinte: primeiro episódio, 52%; paroxística, 25%; persistente, 14%; e permanente, 8%. ^237^

### Mortalidade

•Segundo o Estudo GBD 2019, o número de óbitos por FA no Brasil aumentou nos últimos anos em razão do crescimento e envelhecimento da população. Na década de 1990, a FA foi responsável por 2.659 (II 95%, 2.263-3.342) óbitos, que subiram para 10.811 (II 95%, 8.636-12.801) em 2019. Entretanto, a taxa de mortalidade padronizada por idade por FA permaneceu estável no período, 4,8 (II 95%, 4,0-6,0) óbitos por 100 mil habitantes em 1990 e 5,0 (II 95%, 4,0-6,0) por 100 mil habitantes em 2019, correspondendo a 0,8% (II 95%, 0,6-0,9) de todos os óbitos no país. Embora as taxas de prevalência padronizadas por idade fossem consistentemente mais altas nos homens, as mulheres apresentaram uma maior taxa de mortalidade padronizada por idade em 2019 [(mulheres, 5,2 (II 95%, 3,9-6,0); homens, 4,8 (II 95%, 3,2-6,4)], dados coerentes com os de outros países. ^238^ ^,^ ^239^ Considerando-se os indivíduos com idade > 70 anos, a taxa de mortalidade aumentou de 1990 (51, II 95%, 43-65) a 2019 (73, II 95%, 57-87) (Tabela 6-3). Vale ressaltar que, como a mortalidade baseada apenas em dados de registro vital apresenta um implausível aumento vertiginoso ao longo do tempo, o Estudo GBD 2019 presume, *a priori* , que as taxas de mortalidade específicas para idade e sexo não estejam aumentando nem diminuindo com o tempo. ^4^ Portanto, as pequenas variações com o tempo aqui relatadas são intencionalmente mais baixas do que as reais variações em dados brutos.

•A Figura 6-1 mostra que, embora as taxas de mortalidade padronizadas por idade sejam estáveis, as taxas de mortalidade brutas estão subindo em razão do envelhecimento da população e crescimento do número de indivíduos que vivem mais com doenças crônicas do coração. Além disso, os YLLs, a métrica usada no GBD para óbito prematuro, também estão subindo quando não padronizados por idade.

•A Tabela 6-4 mostra o número total de óbitos e a taxa de mortalidade padronizada por idade por FA por 100 mil habitantes, ambos os sexos, e a variação percentual no Brasil e suas UF no período 1990-2017. As UF com as maiores taxas de mortalidade em 2019 foram Distrito Federal, Maranhão e Roraima, enquanto as menores foram observadas no Piauí, no Amazonas e na Paraíba. Entretanto, devido a problemas de notificação, esses dados podem apresentar inconsistência e devem ser interpretados com cautela.

•O Estudo GBD 2019 utiliza o SDI como uma estimativa do nível socioeconômico de um local. Como demonstrado na Figura 6-2 , houve modesta associação de maior SDI em 2019 com maior variação percentual na taxa de mortalidade padronizada por idade de 1990 a 2019 (r=-0,44, p=0,02), revelando que, nas UF mais desenvolvidas, houve maior redução na mortalidade, possivelmente devido a investimentos em saúde e maior redução na carga de doença cardiovascular como um todo.

•A partir de dados de 1.558.421 ECG de pacientes da atenção primária do sistema de telessaúde de Minas Gerais, ligado ao Sistema de Informação sobre Mortalidade do Brasil, a taxa de mortalidade geral foi 3,34% em um seguimento médio de 3,68 anos. Após ajuste para idade e comorbidades, os portadores de FA apresentaram maior risco de morte geral (HR 2,10; 95% CI, 2,03–2,17) e cardiovascular (HR 2,06; 95% CI, 1,86–2,29), que foi ainda maior nas mulheres, que perderam sua vantagem de sobrevida em relação aos homens na presença de FA. ^236^

•Em seguimento de 10 anos de 1.462 indivíduos com idade ≥ 60 anos (idade média, 69 anos; 61% mulheres) incluídos no estudo de coorte de Bambuí em 1997, FA ou *flutter* mostrou associação independente com um aumento na mortalidade por todas as causas (HR, 2,35; 95% CI, 1,53-3,62) entre pacientes com e sem DCh (HR,1,92; 95% CI, 1,05-3,51). ^240^

•No registro GARFIELD-AF, um estudo mundial de base hospitalar que incluiu 1.061 pacientes ≥18 anos no Brasil com FA diagnosticada nas 6 semanas anteriores e pelo menos um fator de risco adicional para AVC, a taxa de mortalidade por todas as causas por 100-pessoas-ano foi 6,19 (CI 95%, 4,83-7,94), sendo 38,7% óbitos cardiovasculares. ^237^

### Carga de Doença

•De acordo com as estimativas do GBD 2019, a FA resultou em 230.116 (II 95%, 189.167 – 279.885) DALYs no Brasil em 2019, representando 0,35% de todos os DALYs. A taxa de DALYs padronizada por idade foi 103 (II 95%, 84-125) por 100 mil em 2019, maior para os homens (110; II 95% 86-138) do que para as mulheres (96; II 95%, 78-115), embora a proporção de DALYs seja maior para as mulheres (0,42%; II 95%, 0,35-0,48) do que para os homens (0,30%; II 95%, 0,24-0,37) (Tabela 6-5).

•A Figura 6-1 e a Tabela 6-4 revelam o mesmo padrão para as taxas de mortalidade: enquanto a taxa de mortalidade padronizada por idade permaneceu estável de 1990 a 2019, a taxa bruta aumentou.

### Complicações

#### Acidente Vascular Cerebral

•Todos os 429 casos de AVC (87,2% dos quais isquêmicos) que ocorreram na cidade de Joinville em 2015 foram incluídos em um registro, sendo FA detectada em 11,4% deles e em 58% daqueles com AVC cardioembólico. ^241^ Da mesma forma, detectou-se FA em 58% dos 359 pacientes com AVC cardioembólico de uma amostra consecutiva proveniente de um único centro na cidade de Curitiba. ^242^

•Idade (OR=1,04; 95% CI, 1,02-1,08), escala do *National Institutes of Health* para avaliar AVC na admissão (OR=1,10; 95% CI, 1,05-1,16) e aumento do átrio esquerdo (OR=2,5; 95% CI, 1,01-6,29) foram preditores de FA (Estatística C, 0,76; 95% CI, 0,69-0,83) em pacientes hospitalizados por AVC agudo isquêmico ou AIT em uma coorte brasileira. ^243^

•Em uma coorte de 1.121 pacientes com AVC isquêmico em seguimento de 12 anos, FA foi independentemente associada a aumento da mortalidade geral (HR 1,82; 95% CI, 1,43-2,31) e cardiovascular (HR 2,07; 95% CI, 1,36-3,14). ^244^

#### Demência

•Em um estudo transversal com 1.524 participantes com idade >65 anos, diagnosticou-se demência em 11% daqueles com FA e em 4% daqueles sem FA (p=0,07). Os autores reportaram uma razão de chance para demência de 2,8 (95% CI, 1,0-8,1; p = 0,06) entre os indivíduos com FA. ^245^

## Associação de Fatores de Risco para FA/Flutter

•De acordo com o Estudo GBD 2019, as mortes por FA foram atribuídas a seis fatores de risco em 1990 e 2019: hipertensão arterial, índice de massa corporal alto, riscos dietéticos, uso de álcool, tabaco e outros riscos ambientais. Para ambos os sexos, a hipertensão arterial foi o mais importante fator de risco para óbito por FA, representando 35,8% (II 95%, 29,7-42,2) dos óbitos nos homens e 34,4% (II 95%, 27,5-41,7) nas mulheres em 2019.

O índice de massa corporal alto foi o segundo mais importante para os dois sexos, representando 23,9% dos óbitos por FA (II 95%, 12-38) nos homens e 28,8% (II 95%, 16,7-43,2%) nas mulheres, em 2019. É importante notar que enquanto o risco atribuído a hipertensão aumentou levemente de 1990 a 2019 (variação de 7,1% para os homens e de 10,7% para as mulheres), os óbitos por FA atribuíveis a índice de massa corporal alto estão subindo abruptamente (variação de 74,4% para os homens e 79,5% para as mulheres). O risco de óbito por FA atribuível ao uso de álcool também cresce, em particular para as mulheres ( Figura 6-3 ).

•Dados do sistema de telessaúde de Minas Gerais com ECG de 1.558.421 indivíduos (idade média, 51±18 anos; 40,2% homens) realizados entre 2010 e 2017 revelaram, em modelos multivariados ajustados por idade e sexo, a relação das seguintes comorbidades autorrelatadas com a presença de FA: DCh (OR 3,08; 95% CI, 2,91-3,25), infarto do miocárdio prévio (OR 1,74; 95% CI, 1,56-1,93), doença pulmonar obstrutiva crônica (OR 1,48; 95% CI, 1,33-1,66), hipertensão (OR 1,31; 95% CI, 1,27-1,34), dislipidemia (OR 1,09; 95% CI, 1,03-1,16). Tabagismo atual e diabetes não foram associados à prevalência de FA. ^236^

•Um estudo transversal comparando indivíduos com FA a controles saudáveis encontrou maior frequência de apneia do sono no grupo com FA do que no grupo controle (81,6% *versus* 60%, p = 0,03). ^246^

## Comorbidades

### Fibrilação Atrial e Outras Doenças Cardíacas

•A incidência de FA entre 300 idosos (idade média, 75±8 anos; 56% mulheres) monitorados com marca-passo, sem FA na linha de base, foi de 22% em um seguimento de 435 dias, ^247^ atingindo 85% dos pacientes com marca-passo e doença renal crônica em seguimento de 1 ano. ^248^

•No ecocardiograma, a FA foi associada com doença cardíaca (OR = 3,9; 95% CI, 2,1 – 7,2, p <0,001) em 1.518 pacientes (idade média, 58±16 anos, 66% mulheres) de uma lista de espera para ecocardiografia na atenção primária, que também foram rastreados para FA com um dispositivo portátil (prevalência de FA de 6,4%). Os autores sugerem que rastrear FA possa ser útil na atenção primária para estratificar risco e priorizar o ecocardiograma. ^228^

•Insuficiência cardíaca e FA coexistem em muitos pacientes, pois compartilham vias fisiopatológicas similares. Em um estudo retrospectivo de 659 pacientes hospitalizados por insuficiência cardíaca descompensada em 2011, a prevalência de FA foi de 40% (73% FA permanente), estando a FA associada ao avançar da idade (p < 0,0001), etiologia não isquêmica (p = 0,02), disfunção ventricular direita (p = 0,03), pressão sistólica mais baixa (p = 0,02), maior fração de ejeção (p < 0,0001) e aumento do átrio esquerdo (p < 0,0001). Pacientes com FA apresentaram tempo de permanência no hospital mais longo (20,5 ± 16 dias *versus* 16,3 ± 12, p = 0,001). ^249^

•Com relação aos pacientes com doença cardiovascular atendidos na emergência, a prevalência de FA é 40% entre aqueles com insuficiência cardíaca descompensada ^22^ e 44% entre aqueles com doença valvar do coração. ^182^

•Um estudo com pacientes admitidos na unidade de terapia intensiva identificou incidência de FA de 11% durante a internação naquele setor. ^250^

## Fibrilação atrial peroperatória e cirurgia cardiovascular

•No pós-operatório de cirurgia cardíaca, identificou-se FA em 12% a 33% dos pacientes. ^204^ ^,^ ^251^ ^,^ ^252^ As cirurgias de substituição valvar foram associadas a maior ocorrência de FA (31%-33%) durante a hospitalização em comparação à CRVM (12%-16%).

•Idade avançada, doença valvar mitral e não uso de betabloqueadores foram associados com FA no pós-operatório de cirurgia valvar. ^205^ Entre aqueles submetidos a CRVM, a incidência de FA no pós-operatório foi associada com átrio esquerdo > 40,5 mm e idade > 64,5 anos. ^253^

## Fibrilação Atrial e Doença de Chagas

•A FA tem sido consistentemente associada a DCh e aumenta o risco de morte nos pacientes com DCh. ^254–257^ No estudo de coorte de Bambuí, 1.462 participantes com idade ≥ 60 anos (idade média, 69 anos; DCh n=557, 38,1%) e ECG na linha de base foram seguidos por 10 anos. Fibrilação atrial foi mais frequentemente observada nos indivíduos com DCh, 6,1% vs. 3,4% (OR: 3,43; IC 95%, 1,87-6,32, ajustada para idade, sexo e variáveis clínicas), e foi um fator de risco independente para morte (HR: 2,35; IC 95%, 1,53-3,62, ajustada para idade, sexo, variáveis clínicas e níveis de BNP) nos indivíduos com DCh. ^240^

•Em uma grande amostra de 264.324 pacientes submetidos a tele-ECG em unidades de atenção primária à saúde em 2011, DCh foi autorrelatada por 7.590 (2,9%). A idade média dos indivíduos com DCh foi 57,0±13,7 anos e a daqueles sem DCh foi 50,4±19,1 anos, com 5% de octogenários nos dois grupos. Fibrilação atrial foi observada em 5,35% dos indivíduos com DCh e em 1,65% daqueles sem DCh (OR: 3,15; IC 95%, 2,83-3,51, ajustada para idade, sexo e comorbidades autorrelatadas). ^254^

•Em uma revisão sistemática e meta-análise, Rojas *et al* . avaliaram a frequência de anormalidades eletrocardiográficas da DCh na população geral. Foram selecionados 49 estudos, incluindo 34.023 pacientes (12.276 chagásicos e 21.747 não chagásicos). A prevalência de FA foi significativamente mais alta nos chagásicos (OR: 2,11; IC 95%, 1,40-3,19). ^256^

•Em uma amostra de 424 pacientes chagásicos com idade inferior a 70 anos (41,7% mulheres; idade média, 47±11 anos), seguidos por 7,9±3,2 anos, Rassi *et al* . encontraram prevalência de FA de 13,3±3,1%, com forte associação a risco de morte (HR: 5,43; 2,91-10,13) em análise univariada. ^257^

## Utilização e Custo da Atenção à Saúde

**(Ver Tabelas 1-6 a 1-9 e Figuras 1-15 e 1-16)**

•De 2008 a 2019, houve 354.619 hospitalizações por FA e 1.413 procedimentos de ablação para FA e *flutter* atrial realizados pelo SUS, com custos não ajustados de R$ 260.593.600 e R$ 7.912.561, respectivamente. Após ajuste para a inflação brasileira, esses custos foram de R$ 451.530.532 e R$ 13.710.094, respectivamente, e, em dólares internacionais convertidos em PPC ajustados para US$ 2019, $ 169.076.584 e $ 5.047.822, respectivamente.

•Uma análise da carga econômica das condições cardíacas no Brasil estimou uma prevalência de FA de 0,8% (n=1.202.151 casos) em 2015. Os autores estimaram um custo total para FA de R$ 3,921 bilhões (US$ 1,2 bilhão), 94% devidos a custos diretos da atenção à saúde. ^124^

•Um estudo com dados de um ambulatório de anticoagulação privado analisou o custo anual por paciente com FA (n=1.220; idade média, 64 anos) e relatou que 64% do custo total (US$ 10.679) durante seguimento de 1,5 ano foi atribuído a custo com pacientes internados. ^258^

## Conhecimento, Tratamento e Controle

### Anticoagulação

•Houve grande variação no uso de anticoagulação entre os pacientes com FA, de 1,5% a 91%. Estudos com amostras da atenção primária apresentaram maior probabilidade de baixo uso de anticoagulação em comparação a amostras recrutadas de centros terciários ou de cardiologistas, como detalhado a seguir.

•Entre 4.638 indivíduos com FA em centros de atenção primária de 658 municípios em Minas Gerais (idade média, 70±14 anos; 54% homens), submetidos a ECG através de telessaúde em 2011, o uso de AVK foi relatado por 1,5% e o de aspirina, por 3,1%. ^233^

•Em um estudo de pacientes de 125 centros de atenção primária em nove estados de quatro regiões geográficas brasileiras, de janeiro de 2009 a abril de 2016, identificou-se um subconjunto de pacientes com FA (n=301), 189 (63%) dos quais com alto risco de AVC; apenas 28 (15%) faziam uso regular de anticoagulantes orais e 102 (54%), de aspirina. ^234^

•No registro GARFIELD-AF, dos 1.061 pacientes incluídos (82,3% por cardiologistas) no Brasil entre 2010 e 2014 (idade média, 68±13 anos; 55% homens), 86% tinham escore CHA _2_ DS _2_ -VASc ≥2, 19% não usavam anticoagulação na linha de base, 26% estavam recebendo apenas terapia antiplaquetária, 29% estavam usando AVK e 26% estavam usando NOAC. ^237^

•O IMPACT-AF, ^259^ um ensaio randomizado por *cluster* para aperfeiçoar o tratamento com anticoagulantes em pacientes com FA, conduzido na Argentina, Brasil, China, Índia e Romênia, mostrou que dois terços dos pacientes usavam anticoagulação oral na linha de base: 83%, AVK e 15%, NOAC. Dos pacientes do Brasil (n=360), 91% usavam anticoagulação oral na linha de base e 27% usavam NOAC. De todos os pacientes tomando AVK no Brasil, 40,3% apresentavam valores de INR entre 2 e 3 antes da visita na linha de base.

•Um registro de AVC na cidade de Joinville descreveu todos os 429 casos de AVC que ocorreram em 2015, sendo FA detectada em 49 (11,4%) pacientes. Dos 26 pacientes que sabidamente tinham FA prévia, 19 (73%) não estavam anticoagulados, 20 (77%) tinham escore CHA _2_ DS _2_ -VASc ≥ 3 e 21 (81%) tinham escore HAS-BLED < 3. ^241^

•Em uma coorte de 1.121 pacientes com AVC isquêmico, 200 dos quais com FA, anticoagulação para FA foi inversamente associada com mortalidade por todas as causas (efeito tempo-dependente de anticoagulante oral: HR multivariável, 0,47; 95% CI, 0,30–0,50) e mortalidade por AVC (efeito tempo-dependente de anticoagulante oral ≥ 6 meses: OR multivariável, 0,09; 95% CI, 0,01–0,65), mas não com mortalidade cardiovascular. ^244^

•A qualidade da terapia com varfarina foi avaliada usando TTR como parâmetro em diferentes amostras no Brasil. O TTR da anticoagulação para FA variou de 31% a 67% nos estudos. ^258,260–263^ Idade > 65 anos, mas não letramento em saúde, foi associada com um TTR mais longo. ^40^ Em uma análise retrospectiva de 1.220 pacientes de ambulatório privado, aqueles com menor TTR apresentaram sangramentos mais graves e custos de atenção à saúde 40% maiores em seguimento mediano de 1,5 ano. ^258^

### Controle de Ritmo ou Frequência (Medicamento, Cardioversão, Ablação por Cateter)

•Um estudo transversal com 167 pacientes com FA relatou que controle da frequência foi mais comum do que controle do ritmo como estratégia de tratamento (79% vs. 21%; p < 0,001). Entre aqueles tratados com controle de ritmo, as drogas mais prescritas foram amiodarona (43%), sotalol (16%) e propafenona (14%). Os betabloqueadores foram prescritos para 81% dos pacientes em tratamento com controle de frequência. ^264^ Amiodarona foi mencionada por 83% dos médicos como a escolha para controle de ritmo. ^265^

•Dados de 125 centros de atenção primária mostraram que, de 301 pacientes com FA, 91 (30,2%) não recebiam qualquer tipo de tratamento para controle de frequência ou ritmo.

Dos 210 pacientes restantes em tratamento, 147 (70%) usavam agentes para controle de frequência (betabloqueadores, digoxina, diltiazem ou verapamil) e 25 (12%) usavam pelo menos um antiarrítmico (amiodarona ou propafenona). O uso simultâneo de antiarrítmicos e betabloqueadores foi relatado em 36 (17%). ^234^

### Direções Futuras

•Estudos de coorte em andamento têm potencial para preencher os hiatos de informação sobre incidência, fatores de risco, predição de risco e prevenção de FA no Brasil. Até onde se sabe, não há estudo original publicado com informação sobre a incidência de FA no Brasil nem dados longitudinais sobre fatores de risco.

•Estudos desenhados para rastrear FA em uma base populacional ou populações selecionadas através do uso de ECG ou dispositivos de rastreio estão em andamento e deverão fornecer informação sobre a relevância da inclusão dessa estratégia em centros de atenção primária ou especializados.

•O estudo RECALL, o primeiro registro cardiovascular brasileiro de FA, encerrou a inclusão de 4.584 pacientes em 2019 e seus resultados são aguardados. Será o maior registro brasileiro com dados relacionados às características e ao tratamento de pacientes com FA de 73 centros de todas as regiões do Brasil. ^266^

•Estratégias de implementação para aumentar o uso da anticoagulação entre pacientes com FA devem ser encorajadas, em particular no contexto da atenção primária.

Estudos utilizando inteligência artificial para diagnosticar ou prever FA podem ser uma ferramenta para aperfeiçoar o diagnóstico de FA e personalizar estratégias de rastreio.

## 7. HIPERTENSÃO

### CID-10 - I10

**Ver Tabelas 7-1 a 7-6 e Figuras 7-1 a 7-7**

**Table:** Abreviaturas Usadas no Capítulo 7

AVC	Acidente Vascular Cerebral
CID-10	Classificação Estatística Internacional de Doenças e Problemas Relacionados à Saúde, 10 ^a^ Revisão
DALYs	Anos de vida perdidos ajustados por incapacidade (do inglês, *Disability-Adjusted Life-Year* )
DCV	Doenças Cardiovasculares
ELSA	English Longitudinal Study of Aging
ELSA-Brasil	Estudo Longitudinal de Saúde do Adulto - Brasil
ERICA	Estudo de Riscos Cardiovasculares em Adolescentes
GBD	Carga Global de Doenças (do inglês, *Global Burden of Disease* )
HIPERDIA	Programa de Hipertensão Arterial e Diabetes do SUS
HR	Hazard Ratio
IBGE	Instituto Brasileiro de Geografia e Estatística
IC	Intervalo de Confiança
II	Intervalo de Incerteza
IPAQ	Questionário Internacional de Atividade Física (do inglês, *International Physical Activity Questionnaire)*
OR	Odds Ratio
PAD	Pressão Arterial Diastólica
PAS	Pressão Arterial Sistólica
QVRS	Qualidade de Vida Relacionada à Saúde
RP	Razão de Prevalência
SBC	Sociedade Brasileira de Cardiologia
SDI	Índice Sociodemográfico (do inglês, *Sociodemographic Index* )
SUS	Sistema Único de Saúde
UF	Unidade Federativa
YLDs	Anos vividos com incapacidade (do inglês, *Years Lived with Disability* )
YLLs	Anos potenciais de vida perdidos (do inglês, *Years of Life Lost* )

**Tabela 7-1:** – Taxas de hipertensão autorreferida em indivíduos a partir dos 18 anos de idade e intervalos de confiança 95%, de acordo com o sexo, no Brasil, suas regiões, unidades federativas e área de residência (urbana ou rural), 2019.

	Taxas de hipertensão autorreferida (%)								
	Total			Sexo					
				Masculino			Feminino		
	Taxa	IC 95%		Taxa	IC 95%		Taxa	IC 95%	
		Limite inferior	Limite superior		Limite inferior	Limite superior		Limite inferior	Limite superior
Brasil	23,9	23,5	24,4	21,1	20,4	21,7	26,4	25,8	27,1
Urbana	24,0	23,5	24,6	21,4	20,7	22,1	26,3	25,5	27,0
Rural	23,2	22,3	24,1	19,1	18,0	20,2	27,9	26,4	29,4
Norte	16,8	16,0	17,6	14,0	13,1	15,0	19,4	18,2	20,5
Rondônia	18,8	16,9	20,7	16,1	13,7	18,6	21,4	18,3	24,4
Acre	19,2	17,3	21,0	17,7	15,0	20,4	20,5	17,9	23,1
Amazonas	16,0	14,6	17,3	13,9	12,1	15,7	17,9	16,0	19,8
Roraima	15,7	14,0	17,4	12,7	10,5	14,8	18,6	16,0	21,3
Pará	15,3	14,0	16,7	12,0	10,3	13,6	18,4	16,4	20,5
Amapá	18,2	15,7	20,7	15,3	12,5	18,0	20,9	17,4	24,4
Tocantins	22,5	20,2	24,8	20,3	17,3	23,3	24,6	21,6	27,6
Nordeste	23,1	22,5	23,7	19,5	18,6	20,4	26,2	25,4	27,1
Maranhão	19,3	18,0	20,6	16,8	15,1	18,6	21,5	19,8	23,3
Piauí	23,6	21,6	25,6	22,9	19,9	25,9	24,3	21,7	26,8
Ceará	21,3	19,8	22,7	17,1	15,2	19,0	24,9	22,8	26,9
Rio Grande do Norte	21,9	20,3	23,5	18,5	16,6	20,4	24,8	22,5	27,2
Paraíba	25,1	23,1	27,1	22,0	19,1	24,9	27,7	25,3	30,1
Pernambuco	23,4	22,2	24,7	18,4	16,4	20,3	27,5	25,7	29,4
Alagoas	23,9	22,4	25,4	19,1	16,7	21,5	27,9	25,8	30,0
Sergipe	22,5	20,8	24,3	18,6	15,9	21,3	26,0	23,6	28,3
Bahia	25,2	23,6	26,8	21,8	19,4	24,2	28,3	26,0	30,5
Sudeste	25,9	25,0	26,8	23,1	21,9	24,3	28,3	27,0	29,5
Minas Gerais	27,7	25,9	29,5	25,5	23,1	27,9	29,7	27,2	32,1
Espírito Santo	25,5	23,9	27,2	23,7	21,7	25,7	27,1	24,5	29,7
Rio de Janeiro	28,1	26,7	29,4	24,8	22,8	26,7	30,7	28,9	32,5
São Paulo	24,2	22,8	25,6	21,3	19,4	23,2	26,8	24,8	28,8
Sul	24,5	23,5	25,5	22,0	20,8	23,3	26,7	25,3	28,2
Paraná	22,9	21,2	24,7	22,6	20,2	25,0	23,2	20,9	25,6
Santa Catarina	23,6	22,0	25,2	20,5	18,5	22,5	26,6	24,4	28,7
Rio Grande do Sul	26,6	24,8	28,4	22,5	20,4	24,5	30,3	27,6	32,9
Centro-Oeste	21,9	20,9	23,0	20,5	18,9	22,1	23,2	21,8	24,6
Mato Grosso do Sul	24,5	22,7	26,4	23,2	20,6	25,7	25,7	23,3	28,1
Mato Grosso	21,6	19,8	23,5	20,4	17,3	23,4	22,8	20,0	25,5
Goiás	23,4	21,4	25,4	22,0	19,1	25,0	24,7	22,2	27,2
Distrito Federal	16,6	14,7	18,4	14,6	12,3	16,9	18,2	15,5	20,9

*Fonte: IBGE, Diretoria de Pesquisas, Coordenação de Trabalho e Rendimento, Pesquisa Nacional de Saúde 2019. ^306^*

**Tabela 7-2:** – Taxas de hipertensão autorreferida em indivíduos a partir dos 18 anos de idade e intervalos de confiança 95%, de acordo com variáveis sociodemográficas, no Brasil, 2019.

Taxas de hipertensão autorreferida			
Variável	Taxa	IC 95%	
		Limite inferior	Limite superior
**Grupo etário**			
18-29 anos	2,8	2,4	3,3
30-59 anos	20,3	19,6	20,9
60-64 anos	46,9	44,9	48,9
65-74 anos	56,6	54,9	58,2
75+ anos	62,1	60,1	64,1
**Nível educacional**			
Sem instrução ou fundamental incompleto	36,6	35,7	37,4
Fundamental ou médio incompleto	20,4	19,1	21,6
Médio ou superior incompleto	15,4	14,7	16,1
Superior	18,2	17,1	19,3
**Raça/cor da pele**			
Branca	24,4	23,6	25,1
Negra	25,8	24,4	27,2
Parda	22,9	22,2	23,5
***Status* de emprego **			
Empregado	16,9	16,4	17,4
Desempregado	11,9	10,1	13,7
Fora da força de trabalho	38,7	37,8	39,6
Renda mensal (salários mínimos)			
Nenhuma - ¼	16,4	15,1	17,8
1/4 - 1/2	18,7	17,6	19,8
1/2 - 1	25,8	24,9	26,8
1 - 2	25,7	24,9	26,6
2 - 3	25,3	23,5	27,1
3 – 5	25,2	23,2	27,3
Mais de 5	25,0	23,0	27,1
Total	23,9	23,5	24,4

*Fonte: IBGE, Diretoria de Pesquisas, Coordenação de Trabalho e Rendimento, Pesquisa Nacional de Saúde 2019. ^306^*

**Tabela 7-3:** – Número de mortes, taxas de mortalidade padronizadas por idade (por 100 mil) atribuída a pressão arterial sistólica elevada por todas as causas, e variação percentual das taxas, no Brasil, 1990 e 2019.

Grupo etário	1990		2019		Variação percentual (II 95%)
	Número (II 95%)	Taxa (II 95%)	Número (II 95%)	Taxa (II 95%)	
15-49 anos	19166.8 (16242.9;22027.6)	25 (21.2;28.7)	17477.5 (15011.4;19693.6)	15.1 (13;17.1)	-39.5 (-43.9;-34.5)
50-69 anos	62163.9 (55971.7;67686.6)	396.3 (356.8;431.5)	82839 (74580.1;90234.2)	205.3 (184.9;223.7)	-48.2 (-51.1;-45.1)
70+ anos	71875.6 (61751.8;81924.4)	1699.2 (1459.8;1936.7)	139099.3 (116155.4;157920.7)	1062.8 (887.5;1206.6)	-37.5 (-42.3;-33)
Padronizada por idade		197.3 (174.7;218.9)		104.8 (91.2;116.2)	-46.9 (-49.6;-44)
Todas as idades	153206.3 (137435.7;167785.4)	102.9 (92.3;112.7)	239415.9 (209603.5;264681.2)	110.5 (96.7;122.2)	7.3 (0.7;13.6)

**Tabela 7-4:** – Taxas de mortalidade padronizadas por idade (por 100 mil) atribuída a pressão arterial sistólica elevada por todas as causas, e variação percentual das taxas, de acordo com o sexo, no Brasil e suas unidades federativas, 1990 e 2019.

Local	Feminino			Masculino		
	1990 Taxa (II 95%)	2019 Taxa (II 95%)	Variação percentual (II 95%)	1990 Taxa (II 95%)	2019 Taxa (II 95%)	Variação percentual (II 95%)
Acre	126,7 (101,3;148,1)	83,3 (67,6;98,4)	-34,2 (-45;-21,4)	184,6 (152,2;215,6)	131,1 (109,6;153,2)	-29 (-39,8;-15,8)
Alagoas	181,3 (150,6;212,5)	130 (105,7;156,2)	-28,3 (-42,1;-11,6)	212,2 (177,3;247,4)	159,9 (129,1;193,4)	-24,7 (-39,9;-5,6)
Amapá	105 (83,5;128,6)	71,1 (57,5;85,9)	-32,2 (-45;-16,7)	138 (114,7;162,8)	102,3 (85,6;120,5)	-25,9 (-37,3;-11,7)
Amazonas	134,2 (105,1;164)	63,1 (49,8;77,6)	-53 (-63;-39,3)	166,9 (138,9;196,7)	106,1 (86,9;124,8)	-36,4 (-48,2;-23,5)
Bahia	154,2 (120,8;185,7)	87,3 (67,7;109,1)	-43,4 (-55,8;-26,3)	186 (153;220,6)	152,5 (121,1;189,6)	-18 (-36,5;6,2)
Brasil	171,3 (149,6;192,2)	86,3 (73,4;97,2)	-49,6 (-53,2;-45,8)	225,9 (199,7;249,4)	126,9 (111,6;140,4)	-43,8 (-47,2;-40,1)
Ceará	102,6 (77,9;128,7)	82,5 (62,2;104,9)	-19,6 (-39,7;11,6)	128,5 (99,9;159,6)	116,7 (89,8;148,5)	-9,2 (-33,8;24,7)
Distrito Federal	202,9 (162,3;245,4)	89,7 (71,3;107,4)	-55,8 (-64,8;-44,8)	267,3 (216,5;320,7)	110,3 (87,4;133,3)	-58,7 (-66,5;-49,6)
Espírito Santo	176,5 (143;207,5)	93,4 (75,7;112,1)	-47,1 (-57,4;-35,6)	230,4 (201;264)	143,1 (117,4;168,7)	-37,9 (-48,8;-26,8)
Goiás	179 (140,1;224,8)	83,8 (65,6;104,1)	-53,2 (-64;-37,8)	231,5 (184,9;278,1)	119,5 (93,5;147,2)	-48,4 (-60,4;-33,1)
Maranhão	88 (65,4;111,3)	94,7 (73;119,2)	7,6 (-19,2;49)	250,9 (195,5;309,1)	188,8 (151,6;236,6)	-24,8 (-42,6;0,3)
Mato Grosso	148,2 (117,7;179,1)	82,7 (66,3;99,4)	-44,2 (-56,1;-30,5)	170,8 (134,9;207,7)	97 (78,7;117)	-43,2 (-54;-28,9)
Mato Grosso do Sul	175,7 (141,9;204,8)	89,5 (73,8;107,9)	-49,1 (-57,9;-37,1)	213,9 (181,9;244,1)	123,1 (101,6;145,8)	-42,4 (-52,4;-31,3)
Minas Gerais	172,1 (144,1;201,1)	73,2 (58,7;88,1)	-57,5 (-65;-49,3)	226,1 (191,8;260,6)	100,2 (83;118,2)	-55,7 (-62,7;-47,9)
Pará	158,8 (124,9;195,2)	75,6 (60,2;89,5)	-52,4 (-62,9;-38,9)	183,4 (144,7;222,4)	109,8 (89,4;131,2)	-40,1 (-51,9;-23,7)
Paraíba	132,6 (107,2;159,1)	88,2 (70,5;109,8)	-33,5 (-46,9;-15,2)	153,1 (124,2;184,7)	126,9 (102,5;155,4)	-17,1 (-34,9;3,8)
Paraná	213,3 (175;248,8)	92,9 (74,8;110,3)	-56,5 (-65;-47,4)	261,2 (227;296,5)	132,3 (108;156,9)	-49,3 (-57,4;-40,1)
Pernambuco	170,1 (139,5;200,4)	100,2 (81,5;119,8)	-41,1 (-51,9;-27)	206 (176,3;235)	156,7 (127,7;186,6)	-23,9 (-36,7;-7,9)
Piauí	135,9 (107,9;163,4)	83,3 (65,7;101,5)	-38,7 (-50,4;-22)	220,5 (182,2;259,1)	124,1 (102,9;146,2)	-43,7 (-53,1;-32,3)
Rio de Janeiro	212,6 (175,4;247,1)	94,3 (77,2;112,4)	-55,6 (-63,1;-45,6)	278,6 (236,7;316,6)	137,6 (114,4;161,6)	-50,6 (-58;-40,7)
Rio Grande do Norte	113,2 (88,6;138,2)	74,4 (56,7;92,4)	-34,3 (-49,7;-15,2)	151 (123,3;182,8)	114,1 (89,5;143)	-24,4 (-42,3;-1,8)
Rio Grande do Sul	185,7 (158,2;211,6)	84 (68,5;100)	-54,8 (-61,7;-46,4)	231,4 (201;258,6)	122,6 (104;144,7)	-47 (-54,7;-38,7)
Rondônia	245,2 (197,8;292,3)	98,3 (79,4;117,4)	-59,9 (-67,7;-50,1)	225,2 (182,5;268,9)	115,6 (92,7;139,2)	-48,6 (-59,4;-34,8)
Roraima	165 (130,7;198)	89,4 (72,9;106,3)	-45,8 (-55,6;-31,9)	250,1 (213,1;288,5)	136,2 (115,7;156,1)	-45,5 (-53,2;-35,9)
Santa Catarina	200,1 (163,1;235,6)	88,1 (71,1;105)	-56 (-63,7;-46,3)	243,6 (209,6;278,2)	115,6 (96,3;136)	-52,6 (-60,2;-44,3)
São Paulo	196,5 (162,4;231,3)	87,1 (70,3;104)	-55,7 (-63,4;-46)	277,6 (238,9;313,9)	128,2 (107,7;148,6)	-53,8 (-60,5;-46,1)
Sergipe	163,7 (129,8;196,1)	96,4 (77,8;118)	-41,1 (-53,6;-23,7)	197,3 (163,7;231,9)	128,4 (101,1;157,4)	-34,9 (-48,2;-17,2)
Tocantins	145,9 (113,6;178,7)	82,2 (64;100,5)	-43,7 (-56,5;-26,6)	180,1 (141,7;220,5)	137,9 (109,1;169,5)	-23,5 (-40,2;0,1)

*Fonte: Dados derivados do estudo Global Burden of Disease 2019, Institute for Health Metrics and Evaluation, University of Washington. ^46^*

**Tabela 7-5:** – Número de mortes e taxas de mortalidade padronizadas por idade (por 100 mil) atribuída a pressão arterial sistólica elevada por doenças cardiovasculares, e variação percentual das taxas, por grupo etário, no Brasil, 1990 e 2019.

Grupo etário	1990		2019		Variação percentual (II 95%)
	Número (II 95%)	Taxa (II 95%)	Número (II 95%)	Taxa (II 95%)	
15-49 anos	18030.1 (15194.2;20699.9)	23.5 (19.8;27)	16125.8 (13748;18241.4)	14 (11.9;15.8)	-40.6 (-45.2;-35.6)
50-69 anos	58938.4 (52867.6;64283.7)	375.7 (337;409.8)	74600.9 (66631.3;81646.5)	184.9 (165.2;202.4)	-50.8 (-53.6;-47.9)
70+ anos	67682.3 (57758.8;77603.7)	1600 (1365.4;1834.6)	122839.6 (101314.4;141062.5)	938.5 (774.1;1077.8)	-41.3 (-45.9;-37.3)
Padronizada por idade	144650.8 (129424.1;159074)	186.1 (163.8;206.7)	213566.3 (185076;237650.4)	93.4 (80.2;104.2)	-49.8 (-52.5;-47.1)
Todas as idades	144650.8 (129424.1;159074)	97.2 (87;106.9)	213566.3 (185076;237650.4)	98.6 (85.4;109.7)	1.4 (-5.1;7.3)

**Tabela 7-6:** – Taxas de mortalidade padronizadas por idade (por 100 mil) atribuída a pressão arterial sistólica elevada por doenças cardiovasculares, e variação percentual das taxas, de acordo com o sexo, no Brasil e suas unidades federativas, 1990 e 2019.

Local	Feminino			Masculino		
	1990 Taxa (II 95%)	2019 Taxa (II 95%)	Variação percentual (II 95%)	1990 Taxa (II 95%)	2019 Taxa (II 95%)	Variação percentual (II 95%)
Acre	116,1(92;136,8)	71,2(57,1;84,6)	-38,6(-49,2;-25,6)	166,2(135,5;196,1)	109,8(90,3;129,4)	-33,9(-44,7;-21,2)
Alagoas	167,8(138,8;198,4)	117,2(95;141)	-30,1(-43,9;-13,2)	197,2(163,7;232,3)	143,5(115,7;175,4)	-27,2(-42,5;-8,6)
Amapá	95,4(75;118)	59,8(47,9;73,3)	-37,3(-49,7;-22,3)	124,2(102,1;147,7)	86,5(71,9;102,9)	-30,4(-41,6;-16,8)
Amazonas	124,8(96,6;153,6)	53,2(41,7;66,1)	-57,4(-66,4;-44,5)	153,7(126,8;183)	90,4(73,8;107,4)	-41,2(-52,1;-29,4)
Bahia	144,4(112,7;173,9)	77,6(59,9;97,5)	-46,2(-58,1;-30)	172,6(141,2;205,3)	133,3(105,4;166,6)	-22,8(-40,6;0,9)
Brasil	161,6(140,1;182,2)	76,8(64,8;87,4)	-52,5(-56;-49)	212,6(187,4;236)	113(98,4;126,1)	-46,8(-50,3;-43,3)
Ceará	96,4(72,7;121,8)	74,9(56,2;95,5)	-22,3(-41,9;7,6)	121,1(93,5;151,1)	105,1(80,4;135)	-13,2(-36,8;19,1)
Distrito Federal	191,7(152,8;233,2)	80,5(63,2;97,7)	-58(-67,1;-47,4)	246,2(197,3;298,1)	95,3(74,6;116,3)	-61,3(-68,9;-52,5)
Espírito Santo	167,5(135,3;198,4)	83,6(67,4;100,9)	-50,1(-59,9;-39)	218,1(189,1;250,9)	129(105,5;152,6)	-40,9(-51,2;-30,1)
Goiás	168,1(130,5;211,1)	73,2(56,6;91,3)	-56,5(-66,7;-42,1)	214,9(170,3;259,1)	105,1(82;129,8)	-51,1(-62,6;-36,4)
Maranhão	81,4(59,6;104)	85,3(65,2;107,9)	4,8(-21;45,1)	233,6(180,1;289,3)	169,5(134,7;213,8)	-27,5(-44,8;-2,6)
Mato Grosso	138,1(108,7;167,9)	71,7(56,8;86,7)	-48,1(-59,2;-35,2)	158,8(124,8;193,6)	84,3(67,9;102,4)	-46,9(-57,3;-33,4)
Mato Grosso do Sul	166,1(132,8;194,5)	80(65,6;96,7)	-51,8(-60,4;-40,5)	200,8(169,6;229,9)	110,6(91;131,6)	-44,9(-54,4;-33,7)
Minas Gerais	161,1(134,2;189,5)	63,8(50,8;77,3)	-60,4(-67,5;-52,6)	210,2(177,7;242,6)	88,3(72,7;104,7)	-58(-64,7;-50,3)
Pará	148,2(115,8;183,2)	65,9(51,9;78,9)	-55,5(-65,5;-43,1)	169,4(132,3;206,5)	96,3(77,9;116,1)	-43,1(-54,6;-27)
Paraíba	122,2(98,6;147,3)	78,9(62,4;98,4)	-35,5(-48,9;-17,3)	142,5(114,7;174,1)	112,6(90,9;138,4)	-21(-38,4;-0,6)
Paraná	203,1(165,1;237)	82,7(65,9;99)	-59,3(-67,3;-50,7)	248,6(214,7;283,2)	118,6(96,7;141,2)	-52,3(-59,9;-43,4)
Pernambuco	160,4(130,4;189,8)	90,5(73,1;109,2)	-43,6(-54,5;-29,7)	194,3(165,6;222,6)	142,2(115,1;170,4)	-26,8(-39,2;-11,1)
Piauí	128,2(101,6;154,5)	76,2(60,1;93,2)	-40,6(-52,2;-24,4)	208(170,5;246)	112,9(93,3;134,1)	-45,7(-55;-34,3)
Rio de Janeiro	202,8(166,2;236,8)	82,9(67,3;99,5)	-59,1(-66,1;-49,7)	264,8(223,9;301,9)	121,3(99,5;143,2)	-54,2(-61,3;-44,7)
Rio Grande do Norte	106,4(83,3;130,8)	66,2(50,3;82,6)	-37,7(-52,4;-19)	141,8(115,1;172,8)	101,5(79,2;127,3)	-28,4(-45,6;-6,9)
Rio Grande do Sul	176,3(149,6;201,7)	74,8(60,4;90)	-57,6(-64,4;-49,4)	218,6(189;244,8)	109,8(92,9;130,2)	-49,8(-57,1;-41,8)
Rondônia	228,3(183,1;274,5)	84,6(67,3;102,1)	-62,9(-70,3;-53,3)	208,2(167,3;250,2)	99,9(79,6;121,2)	-52(-62,1;-38,7)
Roraima	149,9(116,9;180,6)	75,2(60,5;90,3)	-49,9(-59,2;-36)	228,8(193,7;265,3)	117,3(98,4;135,8)	-48,7(-56,2;-39,3)
Santa Catarina	189,7(154,5;224,4)	78,4(62,5;94,6)	-58,7(-66;-49,6)	230,8(196,7;264,2)	103,7(85,9;122,8)	-55,1(-62,5;-46,9)
São Paulo	186,2(152,7;219,7)	78(62,6;93,7)	-58,1(-65,7;-48,7)	262,4(225,1;297,9)	115,2(96;134,4)	-56,1(-62,3;-48,6)
Sergipe	152,1(119,4;183)	86,2(68,7;106,3)	-43,3(-55,5;-26,5)	180,6(149,3;214,3)	112,2(87,2;137,6)	-37,9(-50,7;-20,7)
Tocantins	135,5(105,4;166,9)	72,7(55,6;89,7)	-46,3(-58,8;-29,5)	165,3(128,4;203,8)	120,2(94,5;148,6)	-27,3(-43,4;-4,1)

*Fonte: Dados derivados do estudo Global Burden of Disease 2019, Institute for Health Metrics and Evaluation, University of Washington. ^46^*

**Figura 7-1:**
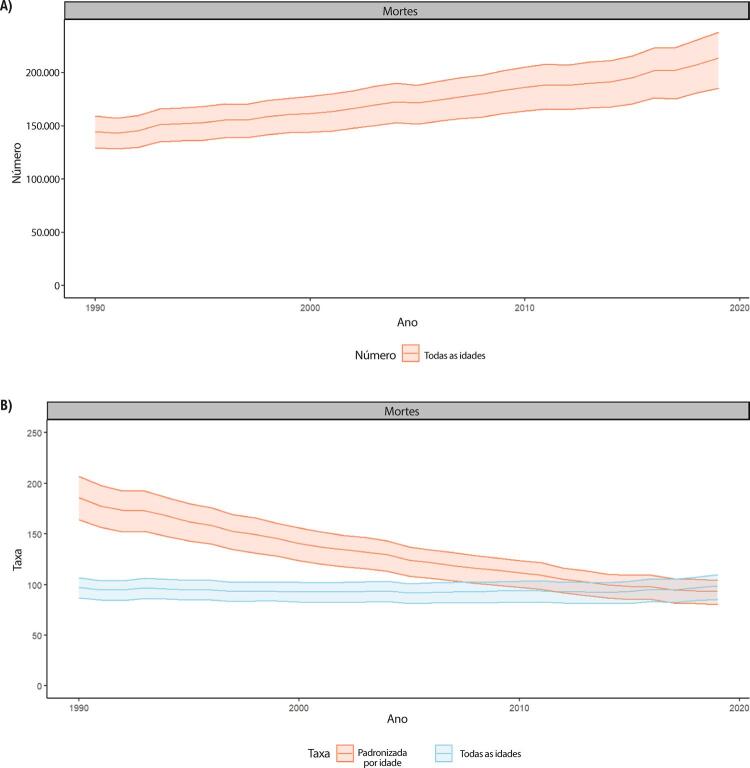
– Número de mortes (A) e taxas de mortalidade (B) atribuída a pressão arterial sistólica elevada no Brasil, 1990-2019.

Fonte: Dados derivados do estudo Global Burden of Disease 2019, Institute for Health Metrics and Evaluation, University of Washington. ^46^

**Figura 7-2:**
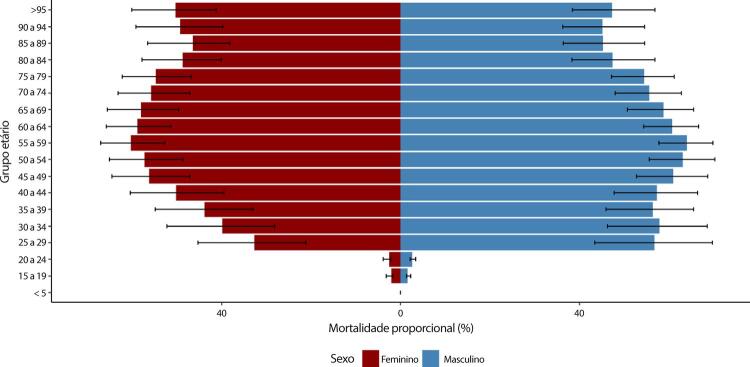
-Mortalidade proporcional por pressão arterial sistólica elevada de acordo com o grupo etário e com o sexo, no Brasil, 2019.

Fonte: Dados derivados do estudo Global Burden of Disease 2019, Institute for Health Metrics and Evaluation, University of Washington. ^46^

**Figura 7-3:**
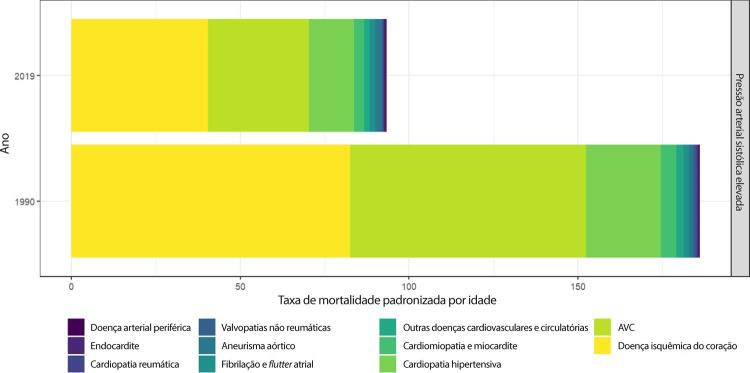
-Taxas de mortalidade por doenças atribuídas a pressão arterial sistólica elevada padronizadas por idade, estratificadas por todas as causas, no Brasil, 1990 e 2019.

Fonte: Dados derivados do estudo Global Burden of Disease 2019, Institute for Health Metrics and Evaluation, University of Washington. ^46^

**Figura 7-4:**
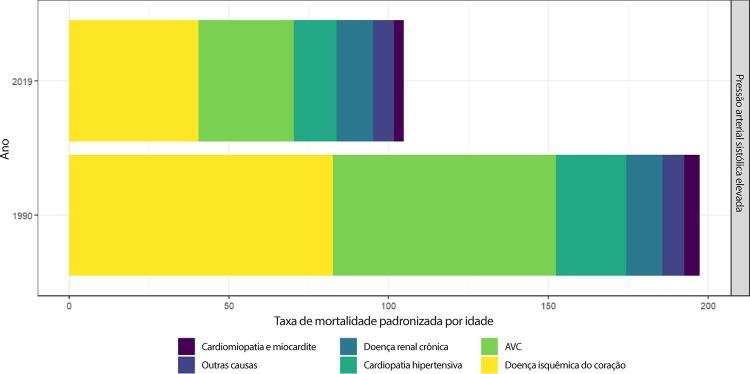
-Taxas de mortalidade por doenças atribuídas a pressão arterial sistólica elevada padronizadas por idade, estratificadas por doenças cardiovasculares, no Brasil, 1990 e 2019.

Fonte: Dados derivados do estudo Global Burden of Disease 2019, Institute for Health Metrics and Evaluation, University of Washington. ^46^

**Figura 7-5:**
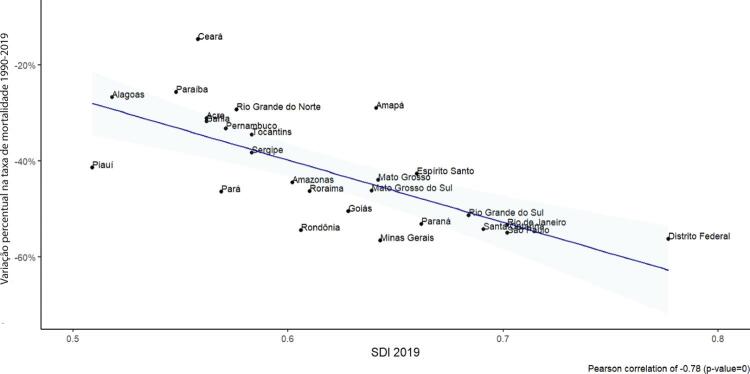
-Correlação entre o Índice Sociodemográfico (SDI) de 2019 e a variação percentual das taxas de mortalidade por doença cardiovascular atribuída a pressão arterial sistólica elevada entre 1990 e 2019, no Brasil.

Fonte: Dados derivados do estudo Global Burden of Disease 2019, Institute for Health Metrics and Evaluation, University of Washington. ^46^

**Figura 7-6:**
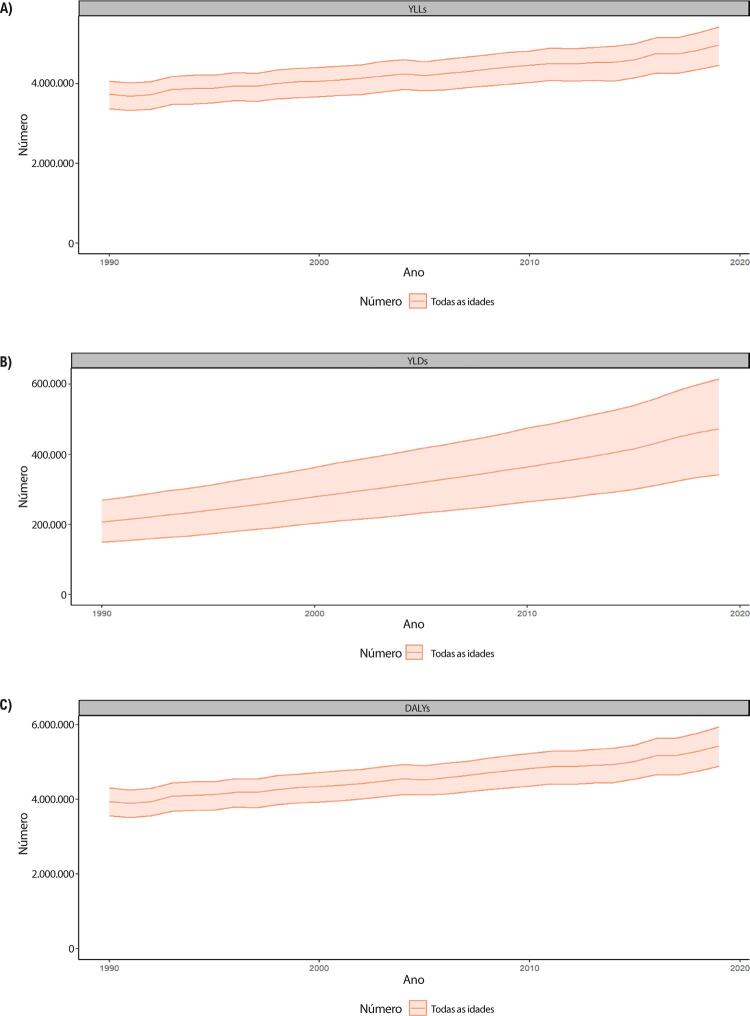
-Número absoluto de YLLs (A), YLDs (B) e DALYs (C) por hipertensão, no Brasil, 1990-2020.

Fonte: Dados derivados do estudo Global Burden of Disease 2019, Institute for Health Metrics and Evaluation, University of Washington. ^46^

**Figura 7-7:**
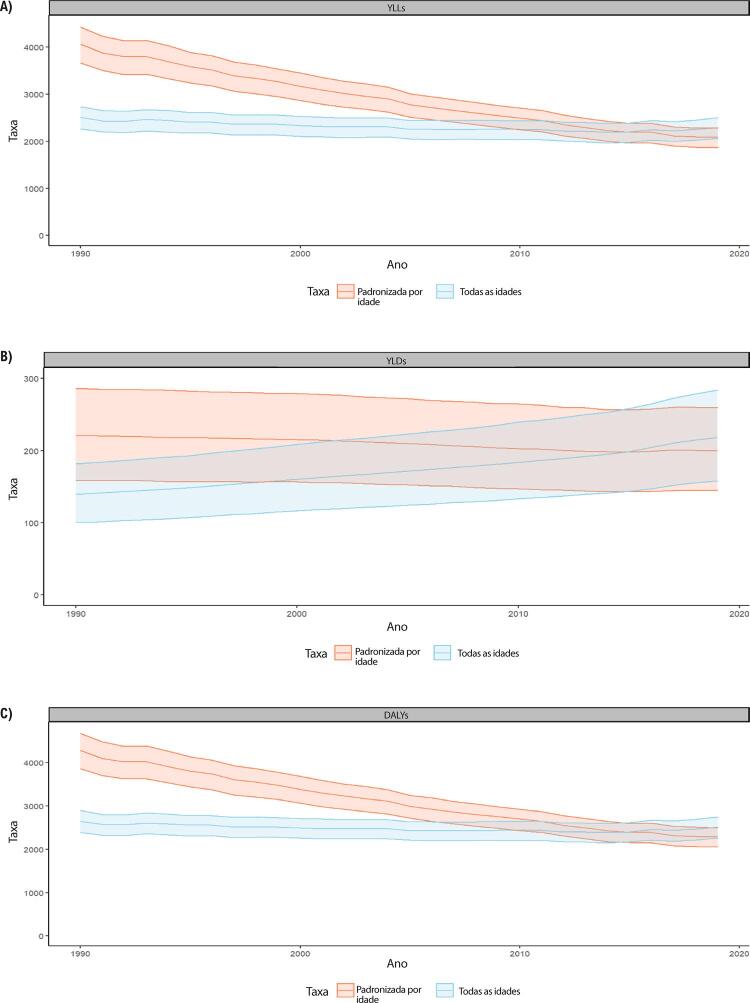
-Taxas de YLLs (A), YLDs (B) e DALYs (C) por hipertensão, no Brasil, 1990-2020.

Fonte: Dados derivados do estudo Global Burden of Disease 2019, Institute for Health Metrics and Evaluation, University of Washington. ^46^

### Panorama

•Com o objetivo de padronização neste documento, hipertensão arterial foi caracterizada como níveis sustentados de PAS igual ou superior a 140 mmHg e/ou PAD igual ou superior a 90 mmHg. ^267^ Os valores de prevalência percentual serão apresentados seguidos por IC 95% ou II 95%, como disponibilizado nos estudos citados.

•Nos estudos populacionais, a hipertensão pode ser aferida ou autorreferida. No primeiro caso, o valor deriva da medida direta da pressão arterial através da utilização de técnicas padronizadas e é descrito em um documento, enquanto a hipertensão autorreferida se caracteriza por uma resposta positiva à pergunta sobre a presença desse diagnóstico médico ou o uso de anti-hipertensivos, ^268^ dependendo, portanto, do acesso do paciente ao diagnóstico e do seu entendimento sobre essa informação.

•Quando o Estudo GBD é a fonte de dados, o risco é atribuído à PAS alta, como descrito em publicação anterior. ^269^

### Prevalência e Incidência

#### Incidência

•Em estudo de 2021, Lopes *et al.* analisaram 8.154 participantes do estudo ELSA-Brasil e relataram uma incidência de hipertensão de 43,2 por 1.000 pessoas-ano, sendo 51,6 nos homens e 37,3 nas mulheres. A incidência por 1.000 pessoas-ano foi maior no grupo etário de 70-74 anos (88,8), nos indivíduos negros (55,9), obesos (79,7) ou diabéticos (91,4) e naqueles de menor nível socioeconômico (58,9). ^270^

#### Prevalência

•Os dados sobre a prevalência de hipertensão podem diferir entre os estudos, dependendo do tipo de pesquisa e da população estudada, especialmente em um país de dimensões continentais e substancial miscigenação, como o Brasil.

#### Hipertensão Aferida

•De acordo com Malta *et al* ., em uma análise dos dados da Pesquisa Nacional de Saúde de 2013, a prevalência de hipertensão aferida a partir dos 18 anos de idade foi 22,8% (IC 95%, 22,1 - 23,4%) em uma amostra de 59.402 indivíduos. ^271^ Naqueles com mais de 75 anos, a prevalência estimada foi de 47,1% (IC 95%, 44,1 - 50,2%) e, em análise estratificada de acordo com o sexo, no grupo etário de 18 a 74 anos, a prevalência foi maior entre os homens, tendo as mulheres apresentado leve predominância apenas no grupo etário ‘acima de 75 anos’ (47,8%, IC 95%, 43,9 -51,7 *versus* 46,2%, IC 95%, 41,3 - 51,3%), revelando uma possível tendência de sobrevivência. A análise por região mostrou que o Sudeste (25%, IC 95%, 23,8 - 26,1%) e o Sul (25%, IC 95%, 23,5 - 26,5%) apresentaram as maiores prevalências em ambos os sexos. ^271^

•No estudo ELSA, Chor *et al* . observaram prevalência de hipertensão de 40,1% nos homens e de 32,2% nas mulheres, com aumento progressivo com a idade, chegando a 63,7% dos indivíduos de 65-74 anos. ^6^ Além disso, houve menor prevalência nos indivíduos pós-graduados (28,4%), em comparação àqueles sem educação secundária completa (44%), e nos indivíduos com renda familiar *per capita* acima de USD $1.000,00 (30,7%), em comparação àqueles com renda inferior a USD $500,00 (40,9%). A avaliação por raça mostrou maior prevalência entre os negros (49,3%) em comparação às outras categorias de raça/cor. ^272^

•Em estudo transversal conduzido em população da região semiárida de Pernambuco, Santiago *et al.* observaram prevalência de hipertensão aferida similar em indivíduos brancos (28,9%, IC 95%, 19,8 - 39,4%) em comparação a negros ou pardos (27%, IC 95%, 22,3 - 32,2%). ^7^ Ao se considerar o nível educacional na análise, a prevalência foi de 44,6% (IC 95%, 36,4 - 53%) entre aqueles que nunca frequentaram a escola, quase duas vezes maior do que entre aqueles com nível educacional elementar ou secundário/superior completo. A avaliação por *status* de emprego mostrou maior prevalência entre indivíduos sem qualquer atividade laboral (30,3%, IC 95%, 24,4 - 36,7%) em comparação àqueles que trabalhavam (23,9%, IC 95%, 18,0 - 30,7%). A prevalência foi maior entre indivíduos dos níveis socioeconômicos superior e intermediário (20,5%, IC 95%, 15,3 - 26,4%) em comparação aos pertencentes ao nível inferior (35,2%, IC 95%, 28,5 - 42,3%). ^273^

•Barbosa *et al* . estimaram a prevalência de hipertensão e seus fatores associados nas regiões brasileiras menos desenvolvidas em estudo transversal realizado com 835 indivíduos com idade >18 anos, que responderam um questionário estruturado domiciliar. ^274^ As medidas de pressão arterial, peso, altura e circunferência abdominal foram tomadas e outros fatores de risco para DCV foram avaliados. A idade variou entre 18 anos e 94 anos (idade média, 39,4 anos), sendo 293 (35,1%) indivíduos normotensos e 313 (37,5%) pré-hipertensos. A prevalência de hipertensão foi 27,4% (IC 95%, 24,4% - 30,6%), sendo maior entre homens (32,1%) do que entre mulheres (24,2%). Na análise ajustada, as seguintes variáveis permaneceram independentemente associadas a hipertensão: sexo masculino (RP 1,52, IC 95%, 1,25 - 1,84), idade ≥ 30 anos (RP 6,65, IC 95%, 4,40 - 10,05 para idade ≥60 anos), sobrepeso (RP 2,09, IC 95%, 1,64 - 2,68), obesidade (RP 2,68, IC 95%, 2,03 - 3,53) e diabetes (RP 1,56, IC 95%, 1,24 - 1,97). ^274^

#### Hipertensão Autorreferida

•O critério mais frequentemente usado para avaliar a prevalência de hipertensão no Brasil é o autorrelato. Picon *et al* ., em meta-análise envolvendo 120.018 indivíduos, demonstrou que, a despeito dos diferentes critérios para o diagnóstico de hipertensão nas décadas de 1980 e 1990, a prevalência de hipertensão caiu de 36,1% (IC 95%, 28,7 - 44,2%) para 28,7% (IC 95%, 26,2 - 31,4%) em 30 anos. ^274^ Entretanto, no início da década de 2000, a prevalência estimada em inquéritos telefônicos ainda foi 20,6% (IC 95%, 19,0 - 22,4%). ^275^

•Uma análise dos dados da Pesquisa Nacional de Saúde de 2013 indicou as seguintes prevalências de hipertensão nos homens: 18,3% (IC 95%, 17,5 - 19,1%) quando autorreferida; 25,8% (IC 95%, 24,8 - 26,7%) quando medida por instrumento; e 33% (IC 95%, 32,1 - 34,0%) quando medida por instrumento e/ou uso de anti-hipertensivo foi relatado. Nas mulheres, as percentagens observadas foram maiores quando se usou o critério autorreferido [24,2% (IC 95%, 23,4 - 24,9%)] e menores quando os demais critérios diagnósticos foram usados: 20% (IC 95%, 19,3 - 20,8%) quando medida por instrumento e 31,7% (IC 95%, 30,9 - 32,5%) quando medida por instrumento e/ou uso de anti-hipertensivo foi relatado. ^268^ Tais diferenças podem ser explicadas pelo fato de que as mulheres buscam os serviços de saúde com maior frequência, apresentando maior probabilidade de serem diagnosticadas com hipertensão.

As regiões Sudeste e Sul e as áreas urbanas do país apresentaram as maiores prevalências quando da análise das regiões geográficas e da área de residência, respectivamente. ^268^

•No estudo VIGITEL, ^276^ baseado em entrevistas telefônicas realizadas em 26 capitais e no Distrito Federal em 2019, a prevalência de hipertensão foi 24,5%, sendo de 27,3% nas mulheres e 21,2% nos homens. As maiores prevalências foram observadas no Distrito Federal (29,6%) e nas cidades de Porto Alegre (27,2%) e Recife (26%) para os homens, e nas cidades do Rio de Janeiro (32,8%), Recife (30,2%) e Salvador (30%) para as mulheres. ^276^ Além disso, esse estudo avaliou a relação com o nível educacional e descobriu que, nos indivíduos com até 8 anos de escolaridade, a prevalência de hipertensão foi cerca de três vezes a daqueles com pelo menos 12 anos de escolaridade, para ambos os sexos. ^276^

•Utilizando estimativas do Estudo GBD 2017, Nascimento *et al* . relataram um aumento na prevalência de PAS elevada padronizada por idade, que passou de 16,9% (II 95%, 16,5 - 17,3%) em 1990 para 18,9% (II 95%, 18,5 - 19,3%) em 2017, tendo esse aumento sido maior entre os homens em todo o período estudado. Além disso, a taxa de crescimento no país foi +0,4% ao ano entre adultos a partir dos 25 anos. ^277^ Ao se analisar de acordo com o sexo, as mais altas prevalências em 2017 foram observadas em Santa Catarina (28,8%, II 95%, 26,6-31%) para o sexo masculino e em Sergipe (19,8%, II 95%, 17,7-21,9%) para o sexo feminino. ^277^

•A Tabela 7-1 mostra a prevalência de hipertensão autorreferida no Brasil de acordo com o sexo em adultos a partir de 18 anos de idade em 2019, como relatado pelo IBGE. A prevalência de hipertensão no país foi 23,9% e maior no sexo feminino do que no masculino (26,4% *versus* 21,1%, respectivamente). A prevalência foi maior nas áreas urbanas do que nas rurais (24,0% *versus* 23,2%, respectivamente) e na região Sudeste para ambos os sexos (23,1% para homens e 28,3% para mulheres).

•A Tabela 7-2 mostra a prevalência de hipertensão autorreferida de acordo com variáveis sociodemográficas, como relatado pelo IBGE. Importante notar que as maiores taxas de prevalência foram observadas em indivíduos a partir dos 75 anos de idade (62,1%), naqueles sem educação formal ou com poucos anos de escolaridade (36,6%) e nos negros (25,8%) e desempregados (38,7%). Em contraste, ao se utilizar renda como critério, indivíduos com renda mais baixa apresentaram menor prevalência de hipertensão (18,7%), provavelmente como reflexo da falta de acesso ao sistema de saúde.

## Mortalidade

### Mortalidade Total Atribuída a Hipertensão

•Oliveira *et al* . compararam a mortalidade por todas as causas atribuída a hipertensão em dois grupos populacionais, sendo um brasileiro e o outro inglês. Os dados usados foram obtidos da coorte ELSA, com 3.205 indivíduos, e da coorte Bambuí, com 1.382 participantes, todos com mais de 60 anos de idade. Importante notar que as características sociodemográficas diferiram nas duas coortes, sendo o nível educacional e a renda familiar maiores na coorte inglesa, enquanto as taxas de tabagismo e diabetes foram maiores na coorte brasileira. Quanto aos riscos relativos, na coorte inglesa, não houve diferença quando hipertensão foi avaliada sozinha, enquanto, na coorte brasileira, hipertensão conferiu um risco relativo de 1,43 (IC 95%, 1,06 - 1,94, p=0,028) para mortalidade em 6 anos. Ademais, as mortes atribuídas a hipertensão na coorte brasileira corresponderam a 25,3% (IC 95%, 8,2 - 39,3%), ultrapassando em muito as da coorte inglesa, na qual o resultado foi limítrofe e sem adequada precisão estatística. ^278^

•A Tabela 7-3 mostra o número de mortes e as taxas de mortalidade atribuída a PAS elevada por todas as causas padronizadas por idade de acordo com os dados do Estudo GBD 2019. Importante notar que nos grupos etários mais velhos, o número de mortes e a taxa de mortalidade são maiores, como esperado para essa doença. Entretanto, a taxa de mortalidade atribuída a PAS elevada em 2019 foi 104,8 por 100 mil habitantes; houve uma redução de 46,9% durante o período estudado, que pode representar uma menor incidência e maior controle terapêutico da hipertensão, assim como uma melhora nas condições socioeconômicas nesse período. ^37^ ^,^ ^279^

•A Tabela 7-4, mostrando as taxas de mortalidade atribuída a PAS elevada por todas as causas padronizadas por idade de acordo com o sexo e as UF brasileiras, indica que essas taxas de mortalidade diminuíram em quase 50% entre 1990 e 2019 para ambos os sexos, tendo sido essa redução maior nas mulheres do que nos homens (49,6% *versus* 43,8%, respectivamente). Entretanto, para as mulheres, exceto em Rondônia, que apresentou a maior redução percentual na taxa de mortalidade atribuída a PAS alta, as maiores quedas foram constatadas nas UF das regiões Sudeste e Sul e no Distrito Federal. O Maranhão foi a única UF a apresentar aumento nas taxas durante o período estudado. Entre os homens, todas as UF apresentaram diminuição na mortalidade atribuída a PAS elevada, com as maiores reduções percentuais registradas nas UF das regiões Sul e Sudeste, como observado para as mulheres (Tabela 7-4).

•A Figura 7-1 mostra o número de mortes (A) e a taxa de mortalidade (B) atribuída a PAS elevada entre 1990 e 2019. Com o aumento na expectativa de vida e consequente envelhecimento da população, além dos dados observados nas tabelas anteriores, esperava-se aumento no número de mortes relacionadas a PAS elevada, como mostra a **F** igura 7-1A. Entretanto, ao se mitigar o efeito da idade nas taxas por padronização por idade, observou-se diminuição na taxa de mortalidade com o tempo (B).

•A Figura 7-2 apresenta a mortalidade proporcional por grupo etário em 2019. Importante notar que houve uma maior proporção de mortes nos grupos entre 55 anos e 74 anos para as mulheres; entretanto, para os homens, a maior proporção foi observada no grupo etário 10 anos mais jovem (45-64 anos). Importante ainda notar que os grupos etários acima de 75 anos para mulheres e acima de 65 anos para os homens podem apresentar outras doenças concorrentes; logo, a proporção de mortes por PAS elevada tende a cair devido às causas competitivas, o que pode, pelo menos em parte, explicar os dados descritos acima.

•A Figura 7-3 apresenta as taxas de mortalidade por doenças atribuídas a PAS elevada padronizadas por idade, estratificadas por todas as causas, entre 1990 e 2019. Como mostrado, a maioria das mortes está relacionada às três principais doenças cardiovasculares, *i.e.,* doença isquêmica do coração, cerebrovascular e hipertensiva, que diminuíram entre 1990 e 2019. A doença hipertensiva, em comparação às outras duas, apresentou a menor redução, o que pode estar relacionado às regras para a escolha da causa básica de morte na declaração de óbito, que, devido a algoritmos específicos, raramente contempla doença hipertensiva como causa básica de morte. ^280^

## Mortalidade Cardiovascular Atribuída a Hipertensão

•Com relação à mortalidade cardiovascular atribuída a PAS elevada, a Tabela 7-5 mostra o número de mortes, as taxas de mortalidade e a variação percentual das taxas entre 1990 e 2019 de acordo com as estimativas do GBD. Houve uma redução na taxa de mortalidade cardiovascular atribuída a hipertensão (por 100 mil habitantes) de quase 50% no período, de 186,1 em 1990 para 93,4 em 2019 (Tabela 7-5) e, à semelhança da mortalidade por todas as causas, as maiores taxas foram observadas nos grupos etários mais idosos.

•A Tabela 7-6 apresenta a mortalidade atribuída a PAS elevada por doenças cardiovasculares de acordo com a UF e o sexo, indicando uma tendência similar à mostrada nas Tabelas 7-3 e 7-4 para mortalidade por todas as causas, que apresentaram avaliação por grupo etário e UF. Portanto, houve uma redução na mortalidade cardiovascular atribuída a hipertensão de 52,5% entre mulheres e de 46,8% entre homens entre 1990 e 2019 no Brasil. Para as mulheres, as UF com maiores reduções foram Rondônia, Minas Gerais, Paraná, Rio de Janeiro e Distrito Federal, com 62,9%, 60,4%, 59,3%, 59,1% e 58%, respectivamente (Tabela 7-6). Para os homens, as maiores reduções foram observadas no Distrito Federal e nos estados de Minas Gerais, São Paulo, Santa Catarina e Rio de Janeiro, 61,3%, 58%, 56,1%, 55,1% e 54,2%, respectivamente.

•A Figura 7-4 mostra as taxas de mortalidade por doenças atribuídas a PAS elevada padronizadas por idade, estratificadas por doenças cardiovasculares, entre 1990 e 2019. As taxas de mortalidade por doença renal crônica permaneceram estáveis, enquanto aquelas por doença isquêmica do coração, cerebrovascular e hipertensiva, além de cardiomiopatias e miocardite, diminuíram no período.

•Por fim, a Figura 7-5 mostra a relação entre o SDI de 2019 e a variação percentual nas taxas de mortalidade por DCV atribuída a PAS elevada de 1990 a 2019, no Brasil e por UF. A Figura 7-5 mostra que as maiores reduções na taxa de mortalidade padronizada por idade ocorreram nas UF com os maiores valores de SDI em uma relação quase que linear, com coeficiente de correlação de 0,78 e p-valor = 0.

## Carga de Doença

•A Figura 7-6 mostra o número absoluto de YLLs (A), YLDs (B) e DALYs (C) relacionados a hipertensão entre 1990 e 2020. Vale notar a tendência a um aumento nos YLLs (A) e, principalmente, YLDs (B), com um consequente impacto na curva de DALYs (A). Essas observações podem ser justificadas, ao menos em parte, por crescimento e envelhecimento populacional.

•Em contraste, a Figura 7-7 mostra uma redução nas taxas padronizadas por idade de YLLs (A) e YLDs (B) com consequente impacto nos DALYs (C). Essas curvas refletem a atenuação do efeito do envelhecimento populacional na carga de doença, com menor influência nos YLDs, o que pode ser parcialmente explicado pela desigualdade de acesso ao sistema de saúde, comprometendo o tratamento das doenças mais graves, com consequente impacto nos YLDs.

## Impacto na Saúde Cardiovascular

•Fuchs *et al.* investigaram a associação entre ‘agrupamento’ de fatores de risco e DCV autorreferida em 1.007 mulheres residentes em Porto Alegre, sul do Brasil. ^281^ A prevalência de hipertensão, diabetes e atividade física e o padrão de dieta foram avaliados quanto à sua associação, em um modo ‘agrupado’, com DCV, que foi definida como autorrelato de história de infarto do miocárdio, insuficiência cardíaca, AVC ou cirurgia de revascularização do miocárdio. Os autores descobriram que hipertensão e diabetes eram o principal ‘agrupamento’ associado com DCV e responsáveis por uma razão de risco independente de 8,5 (IC 95%, 3,0 - 24,5).

•Carvalho *et al.* , avaliando a associação entre hipertensão e qualidade de vida medida pelo SF-36 em uma amostra de 333 indivíduos com hipertensão tratada ou sem hipertensão, mostraram pior qualidade de vida dos pacientes hipertensos em comparação a seus correspondentes normotensos. ^282^ Os autores observaram que os normotensos apresentavam maiores escores do questionário de QVRS em comparação aos hipertensos em todos os domínios, exceto no ‘aspecto emocional’. Com respeito ao ‘componente físico agrupado’, o escore dos homens hipertensos foi 298,4±72,6 em comparação a 333,1±52,1 dos normotensos (P<0,01), e o escore das mulheres hipertensas foi 243,8±84,0 em comparação a 318,7±58,5 das normotensas (P<0,01). Entre os homens, a diferença foi significativa para os domínios ‘capacidade funcional’, ‘aspectos sociais’ e ‘saúde mental’ e, entre as mulheres, houve diferença no domínio ‘aspecto emocional’ do SF-36.

•Para investigar a associação entre hipertensão, pré-hipertensão, idade, tempo do diagnóstico e pressão arterial, Menezes *et al* . usaram dados do ELSA-Brasil, que incluiu 7.063 pacientes com idade média de 58,9 anos na linha de base (2008-2010). Os autores observaram que hipertensão se associou com maiores declínios de memória, fluência e escore cognitivo global. Além disso, pré-hipertensão foi preditor independente de maior declínio no teste de fluência verbal e no escore cognitivo global. Entre indivíduos tratados, o controle da pressão arterial na linha de base associou-se inversamente com o declínio nos escores cognitivo global e teste de memória. ^283^

•A associação de diabetes, hipertensão e diabetes mais hipertensão com DCV (doença arterial coronariana, infarto do miocárdio e AVC) foi investigada por Santos *et al.* em 2.691 pacientes arrolados no programa HIPERDIA em Fortaleza. ^284^ Os autores confirmaram uma significativa associação de hipertensão com doença arterial coronariana, infarto do miocárdio e AVC (P<0,001).

## Utilização e Custo da Atenção à Saúde

•Ao estimar os custos de hipertensão, diabetes e obesidade em pacientes do SUS em 2018, Nilson *et al.* notaram que o custo total de hipertensão, diabetes e obesidade pago pelo SUS chegou a R$ 3,45 bilhões (IC 95%, 3,15 - 3,75), mais de US$ 890 milhões. Desse valor total, 59% foram gastos com hipertensão, 30% com diabetes e 11% com obesidade. Quando se considerou isoladamente obesidade um fator de risco para hipertensão e diabetes, o custo atribuído a essa doença chegou a R$ 1,42 bilhão (IC 95%, 0,98 - 1,87), correspondendo a 41% do custo total. ^285^

•Marinho *et al.* , investigando os custos da atenção à saúde de pacientes com diabetes e hipertensão, compararam os custos diretos de procedimentos ambulatoriais com o valor reembolsado pelo SUS. A principal conclusão do estudo foi que os custos diretos de procedimentos foram maiores do que os reembolsados pelo SUS, caracterizando um subfinanciamento da atenção à saúde pública, o que pode comprometer a qualidade do controle dos fatores de risco cardiovascular. Dos custos, aquele com medicamentos foi o maior, seguido por serviços terceirizados e recursos humanos. ^286^

•Queiroz *et al* . investigaram a associação do número de fatores de risco cardiovascular ou de DCV com internações em 514 usuários do SUS na cidade de Presidente Prudente, estado de São Paulo. Os autores concluíram que hipertensão, arritmias, baixos níveis de atividade física e infarto do miocárdio estavam associados com o número de dias de hospitalização. Além disso, relataram que o número de fatores de risco cardiovascular ou DCV por paciente estava associado com o número de dias de internação. No entanto, as taxas de hospitalização nos últimos 12 meses foram independentemente maiores apenas naqueles com arritmias (OR 3,04; IC 95%, 1,74 - 5,31) e história de infarto do miocárdio (OR 3,07; IC 95%, 1,34 - 7,01). ^287^

## Conhecimento, Tratamento e Controle da Hipertensão

•Utilizando dados da Pesquisa Nacional de Saúde de 2013, Macinko *et al* . estimaram que cerca de 36% da população brasileira (51,4 milhões) tinha diagnóstico prévio e/ou medida da pressão arterial igual ou superior a 140/90 mmHg. Desses, 89% tinham tido contato com o sistema de saúde nos 2 anos anteriores, mas apenas 65% estavam cientes de sua condição. Entre os conhecedores de sua condição de hipertensos, 62% procuravam assistência com regularidade, 92% dos quais haviam recebido prescrição de medicamentos. Daqueles que informaram receber medicamentos, apenas 56% relataram que a assistência à sua condição não apresentava problemas e incluía orientação sobre importantes fatores de risco e comportamento. De toda a população hipertensa, cerca de 33% apresentavam controle de sua pressão arterial. ^288^

•Para a estimativa de prevalência, conscientização, tipos de tratamento anti-hipertensivo e associação de controle de hipertensão com *status* social, coletaram-se os dados de 15.103 indivíduos do estudo ELSA-Brasil na linha de base (2008-2010). ^273^ A pressão arterial foi medida pelo método oscilométrico e 35,8% dos indivíduos foram classificados como hipertensos e 76,8% deles estavam usando medicação anti-hipertensiva. As mulheres estavam mais conscientes do que os homens (84,8% *versus* 75,8%) e usavam medicação com maior frequência (83,1% *versus* 70,7%). Uso de pelo menos uma droga anti-hipertensiva foi relatado por 76,8% (n = 447) dos participantes classificados como hipertensos e foi mais frequente entre as mulheres em todos os grupos etários. Dos usuários de anti-hipertensivos, 69,4% apresentaram níveis controlados de pressão arterial (65,5% dos homens e 72,9% das mulheres). Considerando-se todos os indivíduos hipertensos, cerca de 53% mostraram níveis apropriados de pressão arterial. Entre aqueles em tratamento medicamentoso, maior probabilidade de controle da pressão arterial foi observada no grupo com maior nível educacional do que entre aqueles com nível educacional inferior ao secundário (RP 1,21; IC 95%, 1,14 - 1,28), além de entre indivíduos de ascendência asiática (RP 1,21; IC 95%, 1,12 - 1,32) e brancos (PR 1,19; IC 95%, 1,12 -1,26) em comparação aos negros.

•Em estudo transversal de base populacional, conduzido através de pesquisa domiciliar com amostragem aleatória por agrupamento, sobre a situação de saúde da população idosa de Goiânia, Sousa *et al* . avaliaram 912 idosos (≥ 60 anos) não institucionalizados residentes da área urbana. Os autores relataram uma prevalência de hipertensão de 74,9%, maior (78,6%) entre os homens (OR 1,4; IC 95%, 1,04 - 1,92). A taxa de tratamento foi de 72,6%, sendo maior entre fumantes (OR 2,06; IC 95%, 1,28 - 3,33). A taxa de controle de hipertensão foi de 50,8%, maior entre as mulheres (OR 1,57; IC 95%, 1,19 - 2,08). ^289^

•Em estudo transversal investigando 502 usuários da Estratégia Saúde da Família para controle de hipertensão, Rocha *et al* . compararam três diferentes instrumentos para medir a adesão dos pacientes ao tratamento de hipertensão: o teste Morisky-Green, o questionário de adesão a medicamentos Qualiaids e o questionário Haynes. Os autores relataram as seguintes taxas de prevalência de não adesão: 29,28% pelo teste Morisky-Green; 60,16% pelo questionário Qualiaids; e 13,15% pelo questionário Haynes. Esses achados indicam a existência de oportunidade de melhoria até mesmo em um programa estruturado como a Estratégia Saúde da Família. A despeito da grande variabilidade das taxas de adesão ao tratamento a depender do instrumento usado para medi-la, os autores observaram significativa associação entre adesão ao tratamento e controle da pressão arterial. ^290^

•Para avaliar a evolução de prevalência, conhecimento e controle de hipertensão por 10 anos, dois estudos transversais foram conduzidos em amostras aleatórias em domicílios urbanos e rurais de Pernambuco em 2006 e 2015/2016, envolvendo adultos a partir dos 20 anos de idade. Cerca de um terço da população adulta de Pernambuco tinha hipertensão em 2006 e essa prevalência permaneceu em 2015/2016. Nas áreas rurais, o conhecimento sobre hipertensão aumentou de 44,8% em 2006 para 67,3% em 2015/2016 e o controle de hipertensão passou de 5,3% para 27,1%, de modo que o conhecimento e o controle foram similares nas áreas urbanas e rurais em 2015/2016. Realizou-se análise de regressão logística para estimar a influência de determinantes sociais, comportamentais e antropométricos na hipertensão. A despeito da melhora dos fatores sociais e comportamentais no período de 10 anos, sobrepeso e obesidade abdominal aumentaram. Após ajuste para potenciais fatores de confusão, a probabilidade de apresentar hipertensão mais do que dobrou entre homens (OR 2,03; p < 0,001), adultos jovens (OR 4,41; p < 0,001), idosos (OR 14,44; p < 0,001) e aqueles com obesidade abdominal (OR 2,04; p < 0,001) nas áreas urbanas, assim como nas áreas rurais entre adultos jovens (OR 2,56; p < 0,001) e indivíduos com nível educacional mais baixo (OR 2,21; p = 0,006) e com sobrepeso (OR 2,23; p < 0,001). ^291^

•Em estudo prospectivo, Krieger *et al* . investigaram a prevalência de hipertensão resistente em uma coorte de 1.597 pacientes com hipertensão estágio 2 submetidos a um protocolo de uso de medicamentos em etapas. Dos 1.597 pacientes recrutados, 187 (11,7%) preenchiam os critérios de hipertensão resistente, definida como a falta de controle da hipertensão avaliada por pressão arterial no consultório ≥ 140/90 mmHg e média da pressão arterial no monitoramento ambulatorial de 24 horas ≥ 130/80 mmHg a despeito do tratamento com três medicamentos (enalapril ou losartana, anlodipino e clortalidona) por 12 semanas. Além disso, ao investigarem preditores clínicos de hipertensão, os autores observaram que história de AVC, diabetes mellitus e pressão arterial no consultório ≥ 180/110 mmHg na entrada do estudo foram independentemente associados com hipertensão resistente. ^292^

•No programa *HealthRise* com intervenções de base comunitária para detecção e tratamento de hipertensão e diabetes em comunidades carentes implementado em 2017-2018, mais pacientes haviam alcançado as metas de tratamento de hipertensão [45,9% (43,0%–48,9%)] no final do que na linha de base [35,4% (32,6%–38,6%), p<0,001], na cidade de Vitória da Conquista, na Bahia. Da mesma forma, na cidade de Teófilo Otoni, em Minas Gerais, também incluída no projeto, mais pacientes haviam alcançado as metas de tratamento de hipertensão no final [52,2% (49,3–55%)] em comparação à linha de base [48,3% (45,5–51,2%); p<0,05], sugerindo que as intervenções de base comunitária têm potencial para melhorar o controle da hipertensão. ^293^

## Fatores de Risco e Prevenção

•Em sua dissertação, Treff CA Junior investigou a relação entre hipertensão e níveis de atividade física nos domínios ‘lazer’ e ‘transporte’ do questionário IPAQ, usando o banco de dados do estudo ELSA-Brasil. Aquele autor observou que indivíduos fisicamente ativos durante o tempo de lazer apresentavam níveis mais baixos de PAS (p=0,007) e PAD (p=0,001). Por outro lado, não identificou relação entre hipertensão e níveis de atividade física realizada durante o deslocamento. ^294^

•Ao investigar o papel potencial da dieta na determinação da elevação da pressão arterial e a expressão de outros fatores de risco cardiovascular, Pavan *et al* . estudaram 1.110 indivíduos com 22–89 anos de idade, divididos em três grupos pareados de acordo com sexo e idade (370 da Tanzânia e Uganda, 370 da região Amazônica do Brasil e 370 do norte da Itália), com 111 homens e 259 mulheres em cada grupo. A PAS dos africanos que consumiam uma dieta hipossódica de ‘peixe e vegetais’ foi mais baixa do que a dos brasileiros, cuja dieta teve por base cereais e carne, e ainda do que a dos italianos altamente urbanizados (144,1±21,9; 155,4±26,8; 159,7±22,9 mmHg, respectivamente, < 0,0001). O mesmo ocorreu com a PAD (83,2±11,8; 94,5±15,5; 94,7±11,6 mmHg, respectivamente, < 0,0001). A PAS foi correlacionada com o índice de massa corporal das três populações, mas com a idade apenas dos brasileiros e italianos. O nível de colesterol total e o índice de massa corporal, ambos baixos entre os africanos, aumentaram progressivamente com o aumento do nível socioeconômico. A transição do estilo de vida rural para o urbano parece ser acompanhada pelo aumento nas taxas de fatores de risco cardiovascular. Além disso, fatores ambientais, mais do que raciais, têm crucial impacto no padrão de risco das populações. ^295^

•Em estudo transversal de base populacional, descritivo e observacional, Jardim *et al* . avaliaram 1.739 indivíduos e observaram que hipertensão foi prevalente em 36,4% da população total, maior entre os homens (41,8%) do que entre as mulheres (31,8%). Hipertensão foi positivamente correlacionada a idade ≥60 anos *versus* idade de 18-29 anos (OR 8,92; 5,94 – 14,11; P<0,000), sexo masculino (OR 1,86; 1,47–2,35; P<0,000) e índice de massa corporal (OR 1,44; 1,13 – 1,83; P=0,004). As prevalências de sobrepeso e obesidade foram 30,0% e 13,6%, respectivamente. Sobrepeso foi mais frequente entre as mulheres e obesidade, entre os homens. A prevalência de tabagismo foi 20,1%, mais frequente entre os homens (27,1%) do que entre as mulheres (16,4%). Observou-se estilo de vida sedentário em 62,3% da população, sem diferença entre os sexos. Consumo regular de álcool foi relatado por 44,4% dos indivíduos e mais frequentemente entre os homens. ^296^

•Utilizando dados da pesquisa VIGITEL, Moreira *et al* . tentaram identificar e medir as relações das características sociodemográficas e comportamentais, das características do consumo de alimentos e dos indicadores de saúde relacionados a hipertensão e diabetes. As variáveis independentes analisadas no estudo foram selecionadas tendo por base a sua importância na determinação da carga total de doença, como estimado pela Organização Mundial da Saúde para a região das Américas. ^297^ A análise ajustada relacionada aos dados das mulheres mostrou que idade ≥ 65 anos (RP 1,3; 1,0-1,7), excesso de peso corporal (RP 1,7;1,3-2,2), autoavaliação de mau estado de saúde (RP 1,3; 1,0-1,8) e diagnóstico médico prévio de dislipidemia (RP 1,5; 1,2-1,8) permaneceram independentemente associados com maior prevalência de hipertensão. O consumo de leite integral permaneceu associado com menor prevalência de hipertensão em mulheres (RP 0,7; 0,6-0,9). Na análise ajustada para homens, sobrepeso (RP 1,7; 1,1-2,5) e autoavaliação de mau estado de saúde foram independentemente associados com hipertensão (RP 1,9; 1,4-2,5).

•Ao investigar se a resposta de pressão arterial à ingestão de sal seria específica ao sexo, Mill *et al* . estudaram as variações da pressão arterial de acordo com diferentes ingestões de sal por homens e mulheres. Os autores avaliaram 12.813 indivíduos com uma coleta de urina de 12 horas durante a noite validada, em que a ingestão de sal foi estimada. Em uma única visita, questionários foram aplicados e exames clínico e laboratoriais realizados. A ingestão de sal foi 12,9 ± 5,9 g/d entre os homens e 9,3 ± 4,3 g/d entre as mulheres. Como esperado, os autores concluíram que a pressão arterial aumentou à medida que a ingestão de sal aumentou, independentemente do uso de medicação anti-hipertensiva. No entanto, a inclinação do aumento na pressão arterial provocado pela ingestão de sal foi significativamente maior nas mulheres do que nos homens. Os autores concluíram que a ingestão de sal foi alta nessa grande amostra de brasileiros adultos e apenas poucos participantes atendiam às recomendações das diretrizes. Ademais, a maior responsividade à ingestão de sal observada nas mulheres, mesmo após controle para fatores confundidores, indica maior sensibilidade ao sal e pode ter implicações fisiopatológicas. ^298^

## Crianças e Adolescentes

•Hipertensão, obesidade, dieta pobre e inatividade física na infância e adolescência são preocupações epidemiológicas emergentes. Além disso, identificação e intervenção precoces podem prevenir DCV prematura na vida adulta.

•Para estimar a prevalência de hipertensão e obesidade, além do papel da obesidade na hipertensão, em adolescentes brasileiros, dados dos participantes do Estudo ERICA foram avaliados por Bloch *et al* . A prevalência e o IC 95% de hipertensão arterial e obesidade em âmbito nacional e nas regiões brasileiras foram estimados de acordo com o sexo e o grupo etário, assim como a proporção de hipertensão atribuída a obesidade na população. O estudo avaliou 73.399 estudantes, 55,4% do sexo feminino, idade média de 14,7 ± 1,6 anos. A prevalência de hipertensão foi 9,6% (IC 95%, 9,0 - 10,3), sendo mais baixa nas regiões Norte (8,4%; IC 95%, 7,7 - 9,2) e Nordeste (8,4%; IC 95%, 7,6 - 9,2) e mais alta na região Sul (12,5%; IC 95%, 11,0 - 14,2). A prevalência média de obesidade foi 8,4% (IC 95%, 7,9 - 8,9), mais baixa na região Norte e maior na região Sul. As prevalências de hipertensão e obesidade foram maiores no sexo masculino. Adolescentes obesos apresentaram maior prevalência de hipertensão, 28,4% (IC 95%, 25,5 - 31,2), em comparação aos com sobrepeso, 15,4% (IC 95%, 17,0 - 13,8), ou eutróficos, 6,3% (IC 95%, 5,6 - 7,0), como comumente observado. A proporção de hipertensão atribuída a obesidade foi estimada em 17,8%. Os autores concluíram que o controle da obesidade poderia reduzir a prevalência de hipertensão entre adolescentes brasileiros em cerca de 20%. ^299^

•Christofaro *et al* . analisaram a relação entre a hipertensão de adolescentes e as características sociodemográficas e o estilo de vida de seus pais. Para tal, 1.231 adolescentes, 1.202 mães e 871 pais foram avaliados. A prevalência de hipertensão foi maior entre adolescentes nas seguintes situações: pais mais idosos; ambos os pais relatando hipertensão; e sobrepeso materno. Na análise multivariada, adolescentes com as seguintes características apresentaram maior probabilidade de ter hipertensão: com mães mais idosas (OR 2,36; IC 95%, 1,12 - 4,98); com mães hipertensas (OR 2,22; IC 95%, 1,26 - 3,89); e com pais hipertensos (OR 1,70; IC 95%, 1,03 - 2,81). Em uma análise que considerou os fatores de risco, os maiores riscos de hipertensão foram observados em adolescentes cujas mães tinham quatro ou mais fatores de risco agregados (OR 2,53; IC 95%, 1,11 - 5,74). ^300^

•Para estimar a presença de risco cardiovascular (obesidade e hipertensão) em escolares e suas interações potenciais com aptidão cardiorrespiratória, Burgos *et al* . conduziram estudo transversal em 1.666 escolares com idade entre 7 e 17 anos, sendo 873 (52,4%) do sexo masculino. As seguintes variáveis foram avaliadas: PAS, PAD, índice de massa corporal, percentual de gordura corporal e aptidão cardiorrespiratória. A PAS e a PAD foram correlacionadas com circunferência abdominal, relação cintura-quadril, soma das pregas cutâneas e aptidão cardiorrespiratória. Os autores relataram que 26,7% tinham sobrepeso ou obesidade e 35,9% apresentavam percentual de gordura corporal acima de moderadamente alto. Relataram ainda que 13,9% e 12,1% dos escolares apresentavam níveis limítrofes ou elevados de PAS e PAD, respectivamente. Houve significativa correlação da PAS e PAD com todas as variáveis e uma fraca a moderada correlação com idade, peso, altura, índice de massa corporal e circunferência abdominal. Esses dados indicam um agrupamento de hipertensão, obesidade e falta de aptidão cardiorrespiratória no início da vida e devem determinar o desenvolvimento de programas efetivos de prevenção para reduzir DCV na vida adulta. ^301^

•Schommer *et al* . avaliaram a associação entre variáveis antropométricas e níveis de pressão arterial em escolares do quinto ao oitavo ano para identificar o parâmetro que se correlacionava mais fortemente com os níveis de pressão arterial. Utilizando estudo transversal de base populacional com amostragem probabilística por agrupamento, os autores arrolaram escolares do quinto ao oitavo ano de escolas públicas da cidade de Porto Alegre. A idade média dos participantes foi 12,6 ± 1,6 anos e 55,2% eram do sexo feminino. Níveis anormais de pressão arterial foram identificados em 11,3% da amostra e valores limítrofes, em 16,2%. Das variáveis antropométricas analisadas, circunferência do quadril apresentou a mais forte correlação com pressão arterial elevada (r = 0,462, p < 0,001), sendo seguida por circunferência abdominal (r = 0,404, p < 0,001) e prega cutânea abdominal (r = 0,291, p < 0,001). ^302^

## Pesquisa Futura

•Devido a sua prevalência e seu impacto, a hipertensão é o principal fator de risco cardiovascular para incapacidade e morte no mundo e no Brasil. A despeito do conhecimento atual, faltam dados mais representativos e abrangentes para melhor quantificar a atual incidência de hipertensão, suas tendências, suas trajetórias e determinantes, assim como a morbimortalidade a ela relacionada, estratificadas por região, sexo, idade e nível socioeconômico no Brasil.

•Ao se planejar a redução da carga cardiovascular no Brasil, observa-se um enorme obstáculo ao conhecimento mais profundo e integrado sobre como melhorar a prevenção, o entendimento, o tratamento e o controle da hipertensão, assim como sua relação com outros comportamentos desfavoráveis e fatores de risco cardiovascular, como proposto pela *American Heart Association* com as sete métricas para medir saúde cardiovascular em nível populacional. ^303^ O conhecimento atual aponta para o risco cardiovascular global em nível populacional como sendo o principal determinante da morbimortalidade cardiovascular em âmbito nacional. Portanto, o Brasil necessita urgentemente de melhores dados sobre desfecho e pesquisa em saúde para medir os resultados na população e o desempenho do sistema de saúde, assim como para implementar estratégias de investigação científica de como melhorar esses desfechos. ^304^

•Além disso, devemos evoluir da simples geração de evidência para um modelo de tradução contínua da evidência em boas políticas de atenção à saúde. ^305^ Estratégias nacionais populacionais com campanhas efetivas para a promoção de hábitos saudáveis ( *i.e.* , redução do sal na dieta, taxação dos alimentos pouco saudáveis, aumento de atividade física), aliadas a identificação e tratamento mais efetivos de indivíduos com maior risco cardiovascular, assim como a vigilância objetiva dos resultados, devem ser disseminadas a todos os níveis do nosso sistema de saúde.

•Outro tema que merece ser melhor pesquisado relaciona-se às disparidades de acesso, tempos e desfechos para os hipertensos usuários do SUS em comparação àqueles dos serviços privados de saúde. ^96^ Considerando-se que mais de três quartos dos brasileiros são usuários do SUS, é imperativo que se meçam continuamente os desfechos dos programas de hipertensão implementados pelo SUS, como a Estratégia Saúde da Família, e que se comparem àqueles obtidos no sistema de saúde privado.

## 8. DIABETES MELLITUS

### CID-10 E10 a E14; CID-10-CM E8 a E13

**Ver Tabelas 8-1 a 8-5 e Figuras 8-1 a 8-4**

**Tabela 8-1:** – Prevalência de diabetes *mellitus* (hemoglobina glicada ≥ 6,5% ou uso de antidiabéticos) por sexo, total e regiões.

Variáveis	%	IC 95%
Total	8,4	7,6 – 9,1
Sexo		
Feminino	9,7	8,6-10,7
Masculino	6,9	5,9-7,9
**Região**		
Norte	6,3	5,3 – 7,3
Nordeste	7,6	6,7 – 8,6
Sudeste	9,3	7,9 – 10,7
Sul	7,4	5,9 – 8,9
Centro-Oeste	9,4	7,6 – 11,2

*Fonte: Malta et al. ^345^*

**Tabela 8-2:** – Prevalência de diabetes *mellitus* (hemoglobina glicada ≥ 6,5% ou uso de antidiabéticos) por faixa etária e características sociodemográficas.

Variáveis	%	IC 95%
**Faixa etária**		
18 a 29 anos	1,5	0,7 – 2,2
30 a 44 anos	3,5	2,5 – 4,4
45 a 59 anos	12,6	10,9 – 14,3
60 anos ou mais	20,6	18,2 – 22,9
**Escolaridade**		
Sem instrução	12,3	11,0 – 13,7
Fundamental	7,4	5,6 – 9,2
Médio completo	5,3	4,4 – 6,3
**Raça/cor**		
Branca	8,4	7,3 – 9,6
Preta	10,3	7,5 – 13,0
Parda	7,9	6,9 – 8,9
Outra	7,7	3,3 – 12,1
**Índice de massa corporal**		
Peso baixo/normal	4,0	3,3 – 4,8
Sobrepeso	8,5	7,3 – 9,8
Obesidade	16,9	14,7 – 19,0

Fonte: Malta *et al* . ^345^

**Tabela 8-3:** – Número de mortes e taxa de mortalidade padronizada por idade (por 100 mil) por doença cardiovascular atribuível ao diabetes, para ambos os sexos. Brasil e Estados, 1990 e 2019.

Localidade	1990		2019		Variação percentual da taxa (II 95%)
	Número (II 95%)	Taxa (II 95%)	Número (II 95%)	Taxa (II 95%)	
Acre	64,6 (43,5;94,8)	57,1 (36;92,4)	195,1 (133,9;284,1)	38,3 (25,4;58,4)	-32,9 (-43,8;-16,9)
Alagoas	864,2 (581,3;1270,1)	75,2 (48,9;113)	1708,7 (1203,3;2458,4)	56,7 (39,4;82)	-24,6 (-38,9;-5,4)
Amapá	31,6 (20,7;48,7)	43,9 (27,1;71)	141,6 (94,9;204,8)	33,2 (21,7;49,5)	-24,3 (-39,9;-1,8)
Amazonas	315,8 (206,7;490,7)	58,6 (36,7;96,6)	824,9 (548,9;1222,8)	33 (21,6;49,5)	-43,6 (-54,8;-26,8)
Bahia	2955,3 (1921,1;4621,9)	49,6 (31,6;78,7)	6101,2 (4096,4;9411,7)	38,5 (25,7;59,2)	-22,4 (-41,5;4,7)
Ceará	1422,3 (869,1;2289,7)	37,7 (22,9;62,1)	3981,1 (2517,4;5969,7)	41,1 (25,8;62)	8,9 (-18,9;47,8)
Distrito Federal	327,1 (232,6;446,1)	105,4 (70,3;160,3)	710,8 (488,7;1014,2)	40 (26;62,3)	-62 (-70,9;-50,5)
Espírito Santo	759,3 (500,7;1136,6)	68,2 (43,5;110,6)	1536,2 (1024,7;2294,8)	38 (25,1;57,9)	-44,3 (-56,7;-27,9)
Goiás	990,7 (650,4;1477,2)	65,5 (41,1;104,7)	2109,1 (1393,4;3211,1)	33,3 (21,5;51,5)	-49,1 (-61,8;-30,4)
Maranhão	1388,1 (920,9;2107,1)	62,3 (40,6;96)	3568,4 (2352,1;5390)	56,8 (37,2;86)	-8,9 (-30,1;17,6)
Mato Grosso	319,9 (210,4;476)	59,6 (37,3;92,5)	951,3 (626,1;1401,9)	32,9 (21,2;49,4)	-44,7 (-56,6;-27,6)
Mato Grosso do Sul	489,1 (334,7;696,2)	75,2 (49;114,6)	1050,2 (708,3;1526,9)	38,9 (25,8;57,4)	-48,2 (-58,6;-33,4)
Minas Gerais	4033,5 (2792,7;5941,5)	52,2 (34,4;79,9)	6101,6 (3909,3;9406)	23,3 (14,9;35,9)	-55,3 (-65,7;-41,7)
Pará	1048,9 (680,6;1613,8)	68,1 (42,1;109,9)	2461,9 (1641,9;3553,9)	39 (25,6;57,2)	-42,7 (-54,7;-23,9)
Paraíba	1208,7 (785,1;1848,2)	55 (35,5;85,2)	2106,7 (1395;3164,2)	43,4 (28,9;65)	-21 (-36,7;0,4)
Paraná	2811 (1846,6;4153,8)	79,2 (49,4;126,7)	4756,2 (3146,9;7007,4)	38,6 (25,2;58,5)	-51,3 (-61,1;-36,6)
Pernambuco	2973,6 (1992,5;4385,2)	76,4 (49,9;115,9)	5047,3 (3437,1;7374,5)	53,3 (35,7;78,6)	-30,2 (-42,6;-12,1)
Piaui	655,7 (425,9;1025,4)	59,5 (36,7;96,6)	1481,3 (951,6;2261,5)	39 (25,1;59,3)	-34,5 (-48,3;-12,3)
Rio de Janeiro	7914,2 (5433,6;11183,9)	98,7 (65;142,3)	8936,3 (6041,8;13238,2)	40,6 (27,3;60,1)	-58,9 (-66,3;-49,1)
Rio Grande do Norte	785,8 (515,7;1213,6)	52,5 (33,6;82,2)	1402,4 (894,9;2075,7)	36,1 (23;53,7)	-31,1 (-44,9;-12,2)
Rio Grande do Sul	3206,9 (2141,9;4941,3)	61,2 (39,5;97,9)	4779,8 (3012,6;7502,5)	31 (19,4;49,1)	-49,3 (-61,2;-34)
Rondônia	187,3 (122,5;266,3)	114,4 (73,6;177,2)	547,6 (363,6;815,8)	41,3 (27,1;62,4)	-63,9 (-70,7;-55,7)
Roraima	28,9 (20,2;40,9)	90,6 (59,2;137,9)	116,6 (82,4;166,8)	42,3 (28,1;64,5)	-53,3 (-59,3;-46,1)
Santa Catarina	1462,2 (975,1;2147,5)	78,1 (49,8;119,2)	2392,6 (1603,5;3597,3)	32,9 (21,3;51,3)	-57,9 (-66,4;-46,9)
São Paulo	13914,2 (9600,8;19624,5)	93,2 (59,9;141,8)	16291,4 (10698,7;24578,1)	31,9 (20,5;49,5)	-65,7 (-72,4;-57,6)
Sergipe	483,5 (334;711,7)	76,9 (50,1;119,9)	904,8 (609,4;1323,8)	42,9 (28,8;63,6)	-44,2 (-54,7;-29,4)
Tocantins	169,8 (114,4;250,5)	68,7 (42,5;112,8)	549 (355,4;810,2)	42 (26,6;63,4)	-38,9 (-53,3;-18,9)
Brasil	50812,4 (35649,3;73136,9)	70,4 (47,4;106,1)	80754,1 (55922,4;118175,4)	35,9 (24,5;53)	-49 (-53,4;-43,9)

*Fonte: Dados derivados do estudo Global Burden of Disease 2019, Institute for Health Metrics and Evaluation, University of Washington. ^46^*

**Tabela 8-4:** – Taxa de mortalidade padronizada por idade (por 100 mil) por doença cardiovascular atribuível ao diabetes, para homens e mulheres. Brasil e Estados, 1990 e 2019.

Localidade	Mulheres			Homens		
	1990	2019	Variação percentual da taxa (II 95%)	1990	2019	Variação percentual da taxa (II 95%)
Acre	42.7 (25.5;69.4)	27.8 (17.1;44.3)	-35 (-51.6;-9.2)	76.5 (46.8;125.2)	51.6 (33.8;79.5)	-32.5 (-45.7;-13.3)
Alagoas	65.3 (40.5;102.6)	48.6 (31.7;71.5)	-25.5 (-44.5;3.8)	86.6 (54.3;132.5)	67 (44.9;96.2)	-22.7 (-41.8;6.9)
Amapá	33.9 (19.5;56.2)	23.3 (14.2;37)	-31.3 (-50.9;-2.4)	56.1 (33.4;90.8)	45 (28.8;66.6)	-19.8 (-39.3;12.5)
Amazonas	54.2 (32.8;92.4)	24.8 (15.2;39)	-54.2 (-67.5;-35.7)	62.9 (37.9;103.7)	42 (27.6;62.6)	-33.3 (-49.6;-3.2)
Bahia	43.9 (26.7;70)	26.4 (16.4;42.5)	-40 (-59;-12.2)	56.2 (33.6;95.8)	54.6 (34.4;84.2)	-2.9 (-34.1;51.1)
Ceará	31 (17.4;54.3)	32.2 (18.4;51.1)	4 (-29.2;58.8)	45.6 (25.9;77.9)	52.5 (32.6;80.4)	15.1 (-22.2;77.8)
Distrito Federal	91 (56.3;144.1)	33.8 (20.3;54.2)	-62.9 (-74.3;-48)	137.1 (88.8;210.4)	49.3 (31.2;79.1)	-64 (-73.6;-51.5)
Espírito Santo	58.2 (35;96.8)	28.7 (18;44.9)	-50.7 (-65.1;-28.8)	79.3 (48.3;127.4)	49.8 (31.9;76.7)	-37.1 (-54.7;-13)
Goiás	57.8 (34.7;94.5)	25.9 (15.6;42.7)	-55.1 (-69.7;-31.8)	75.1 (45.4;126.3)	41.8 (25.7;65.1)	-44.3 (-61.8;-16.1)
Maranhão	30 (17.7;49.5)	36.5 (22.2;58)	21.8 (-19;82.1)	123.1 (77.8;194.1)	85.5 (54.6;132.5)	-30.6 (-48;-5.9)
Mato Grosso	53.2 (31.6;84.4)	27 (16.7;42.2)	-49.2 (-64.7;-27.4)	65.1 (37.4;107)	38.7 (24.2;58.8)	-40.5 (-57.6;-12.9)
Mato Grosso do Sul	66.5 (41.6;105.4)	31.1 (19.7;47.9)	-53.2 (-66.4;-34.4)	83.3 (52.6;126.6)	47.8 (30.4;71.3)	-42.6 (-57.2;-22.3)
Minas Gerais	44.7 (28.8;73.4)	18.2 (11.3;29.9)	-59.3 (-72.3;-42)	61.1 (40.5;97.5)	29.6 (17.6;47.8)	-51.6 (-66.4;-30.7)
Pará	59.1 (34;98.7)	28.5 (17.6;44.5)	-51.7 (-65.3;-27.8)	78.4 (45.6;129.7)	50.6 (32.9;76)	-35.4 (-52.2;-7.8)
Paraíba	51.7 (31.8;81.4)	35.5 (21.8;54.7)	-31.3 (-50.2;-5.1)	58.6 (36.9;91.6)	53.5 (35.4;80.3)	-8.7 (-31.2;25.2)
Paraná	67.8 (40.3;113.2)	29.8 (18.2;47.9)	-56 (-68.8;-36.7)	91.1 (54.7;146.6)	49.3 (31.5;75)	-45.9 (-59;-22.2)
Pernambuco	70 (44.3;108.9)	42.1 (26.9;63.4)	-39.9 (-54;-21.3)	84 (52.6;127.2)	68.6 (45.5;101.7)	-18.4 (-38.8;12.1)
Piaui	43.6 (25.9;74.3)	30.1 (18.5;48.5)	-31 (-50;-2.3)	79.6 (47.9;136.7)	49.8 (31.8;77.7)	-37.4 (-53.4;-12.4)
Rio de Janeiro	75.1 (45.5;115.2)	28.9 (17.7;45.2)	-61.6 (-72.4;-46.8)	133.4 (90.5;193.4)	57.3 (38.8;85.3)	-57 (-65.4;-45.8)
Rio Grande do Norte	41.4 (25.9;65.1)	27.1 (16.4;42.4)	-34.4 (-53.4;-8.6)	65.4 (40.9;104.5)	47.8 (29;72.4)	-26.9 (-46.5;2.8)
Rio Grande do Sul	51.5 (30.9;85)	25 (14.7;41.7)	-51.4 (-66.2;-28.2)	74.2 (46.6;120.3)	38.7 (23.8;62.6)	-47.9 (-63.9;-23.2)
Rondônia	115.4 (73.3;180.4)	32.5 (19.9;50.4)	-71.8 (-79.7;-62.9)	112.4 (69.3;172.3)	50 (32.3;75.3)	-55.5 (-66.3;-42.3)
Roraima	74 (47.5;115.5)	34.2 (22.1;53.7)	-53.8 (-61.4;-44.2)	104.5 (65.8;159.2)	49.8 (32.7;75.5)	-52.3 (-59.9;-42.5)
Santa Catarina	69.3 (42.1;112.3)	25.7 (15.4;42.3)	-62.9 (-73.5;-47.7)	88.3 (54.6;139.3)	41.8 (26.5;63.9)	-52.7 (-64.8;-34.7)
São Paulo	75.9 (46.7;120.5)	24.1 (14.7;40.3)	-68.2 (-77.2;-55.4)	115 (75.2;168.4)	42.2 (25.8;64.6)	-63.3 (-72;-53)
Sergipe	67.2 (42.4;106.5)	35.6 (22.4;55.6)	-47 (-61.7;-28.1)	89.2 (56.6;139.4)	52.3 (34.3;78.7)	-41.4 (-54.9;-20.5)
Tocantins	61.7 (38.1;101.7)	31.1 (19.5;48.5)	-49.6 (-63.3;-29.6)	76.7 (44.9;130.4)	55.1 (33.7;86.8)	-28.1 (-50.2;4)
Brasil	58.1 (38.4;88.3)	27.2 (18;41.6)	-53.2 (-59.3;-46.1)	85.4 (58.1;128.3)	47.1 (32.1;68.6)	-44.9 (-50.4;-37.8)

*Fonte: Dados derivados do estudo Global Burden of Disease 2019, Institute for Health Metrics and Evaluation, University of Washington. ^46^*

**Tabela 8-5:** Número de DALYS e taxa de DALYS padronizada por idade (por 100 mil) por doença cardiovascular atribuível ao diabetes, para ambos os sexos. Brasil e Estados, 1990 e 2019.

Localidade	1990		2019		Variação percentual da taxa (II 95%)
	Número (II 95%)	Taxa (II 95%)	Número (II 95%)	Taxa (II 95%)	
Acre	1335.2 (912.8;1888.1)	941.8 (633.3;1371)	3934.6 (2782.2;5669)	683.4 (475.1;986)	-27.4 (-38.8;-10)
Alagoas	17722.1 (12411.8;24790.4)	1387.3 (965.4;1978.1)	35556 (25585.2;48957.8)	1134.8 (819.4;1566.4)	-18.2 (-34.2;3.8)
Amapá	630.7 (433.5;914.9)	723.1 (475.3;1095)	3022.1 (2113.7;4226.6)	623.3 (427.4;894.6)	-13.8 (-31.8;11.6)
Amazonas	6428.5 (4391;9409.2)	965.9 (641.4;1472.8)	16437.5 (11420;23668.2)	608.2 (420.8;881.9)	-37 (-50.4;-17.8)
Bahia	60323 (41070.5;88143.3)	926.4 (622.4;1396.6)	120282.6 (83918;176491.9)	757.4 (525.9;1119.6)	-18.2 (-39.5;10.1)
Ceará	26051.4 (16968.5;39656.1)	662.4 (424.8;1021.6)	71929.9 (49135.3;103619.2)	733.5 (497.6;1060.9)	10.7 (-18.6;51.3)
Distrito Federal	8468 (5993.5;11589.3)	1726.1 (1219.3;2443.4)	14639.6 (10394.9;20195.3)	632 (440.7;895.3)	-63.4 (-71.5;-52.8)
Espírito Santo	15740.9 (11037.6;22572.5)	1165.3 (797.7;1722)	29886.8 (21156.3;42438)	697.7 (488.3;1000.8)	-40.1 (-53.1;-22.7)
Goiás	22199.6 (15278.2;32417.9)	1165 (776.4;1711.4)	43263.8 (29225.9;64727.4)	632.8 (428.1;953.8)	-45.7 (-59.9;-24.9)
Maranhão	30853.4 (20729.6;44978.9)	1241.5 (845.8;1830.5)	68520.9 (47179.2;99823.3)	1062.6 (731.1;1556.5)	-14.4 (-35.9;13.9)
Mato Grosso	7137.7 (4910.7;10348.6)	1035.9 (688.5;1536.7)	19808.7 (13578.4;28204)	622.3 (424.8;897)	-39.9 (-53.1;-20.8)
Mato Grosso do Sul	10808.6 (7735.6;15042.3)	1319.5 (913.6;1871.1)	20964.7 (14543.3;29539.1)	726 (504.9;1024.7)	-45 (-56.3;-29.1)
Minas Gerais	90445.6 (65437.6;127258.5)	961.2 (684.3;1390.3)	119023.5 (80740.1;174655.1)	450.2 (303.6;661.3)	-53.2 (-63.3;-39.1)
Pará	20697.3 (14113;30025.6)	1124.9 (747.8;1683)	49334.3 (34295.7;68456.6)	739.9 (504;1037.5)	-34.2 (-48.3;-14.2)
Paraíba	22665.6 (15429.1;32837.2)	993.8 (674.6;1439.3)	39309.5 (27360.3;56659.5)	837 (582.8;1205.1)	-15.8 (-34.2;10)
Paraná	58887.3 (40717.4;84598.3)	1354.2 (894.9;2009.2)	91772.5 (62792.3;129456.7)	699.4 (477.5;991.9)	-48.4 (-58.9;-32.9)
Pernambuco	59380 (41231.5;83117.2)	1366 (942.4;1923.6)	100595.8 (70745.7;143053.9)	1017.4 (711.7;1452.2)	-25.5 (-39.4;-6.5)
Piaui	12857.4 (8842.4;19094.9)	998.5 (673.1;1525.2)	27097.6 (18663.2;39307.5)	724.6 (499.2;1055.2)	-27.4 (-43.3;-3.5)
Rio de Janeiro	176130.1 (123280.1;244011.1)	1888.9 (1331.8;2618.9)	178623.1 (126398.8;254598.7)	790 (561;1127)	-58.2 (-66.3;-47.6)
Rio Grande do Norte	14427.4 (9841.6;21299.8)	924.2 (630.3;1361.8)	26480.8 (17460.1;37536.8)	693.6 (455.8;984.5)	-25 (-41.7;-2.5)
Rio Grande do Sul	65930.3 (46174.7;94957.8)	1075 (740.8;1584.9)	87421.8 (58608.1;129018)	558.7 (375.7;819)	-48 (-59.9;-32.9)
Rondônia	4693.9 (3130.2;6604.9)	1850.9 (1256.7;2680.8)	11161.5 (7688.3;16108.6)	761.7 (520.9;1110)	-58.8 (-67.1;-48.3)
Roraima	701.7 (486.9;981.2)	1510.3 (1042.4;2179.8)	2550 (1822.3;3524.3)	737.4 (521.9;1032.2)	-51.2 (-58.6;-41.4)
Santa Catarina	29444.9 (20595.6;41977.2)	1315.9 (886.4;1907.9)	45537.1 (31491;64303.7)	577.1 (396.9;820.5)	-56.1 (-65.4;-44.1)
São Paulo	295196.3 (209790.2;409567)	1609.4 (1116.3;2263.4)	315407.1 (218126;449199.4)	586.5 (403.7;842.8)	-63.6 (-71.1;-55.1)
Sergipe	9507.4 (6819.9;13407.5)	1308.1 (916.3;1874.8)	17955.5 (12531.5;25018.5)	821.4 (570.1;1153.4)	-37.2 (-49.4;-21)
Tocantins	3644.4 (2521.7;5217.6)	1085.4 (724.5;1612.6)	10599.2 (7112.5;14982.1)	766.2 (510;1096.3)	-29.4 (-46.1;-5.5)
Brasil	1072308.9 (784276.4;1484959.3)	1279.7 (922.2;1800.5)	1571116.4 (1140912.3;2203187.8)	673.5 (485.1;947.7)	-47.4 (-52.2;-41.9)

*Fonte: Dados derivados do estudo Global Burden of Disease 2019, Institute for Health Metrics and Evaluation, University of Washington. ^46^*

**Figura 8-1:**
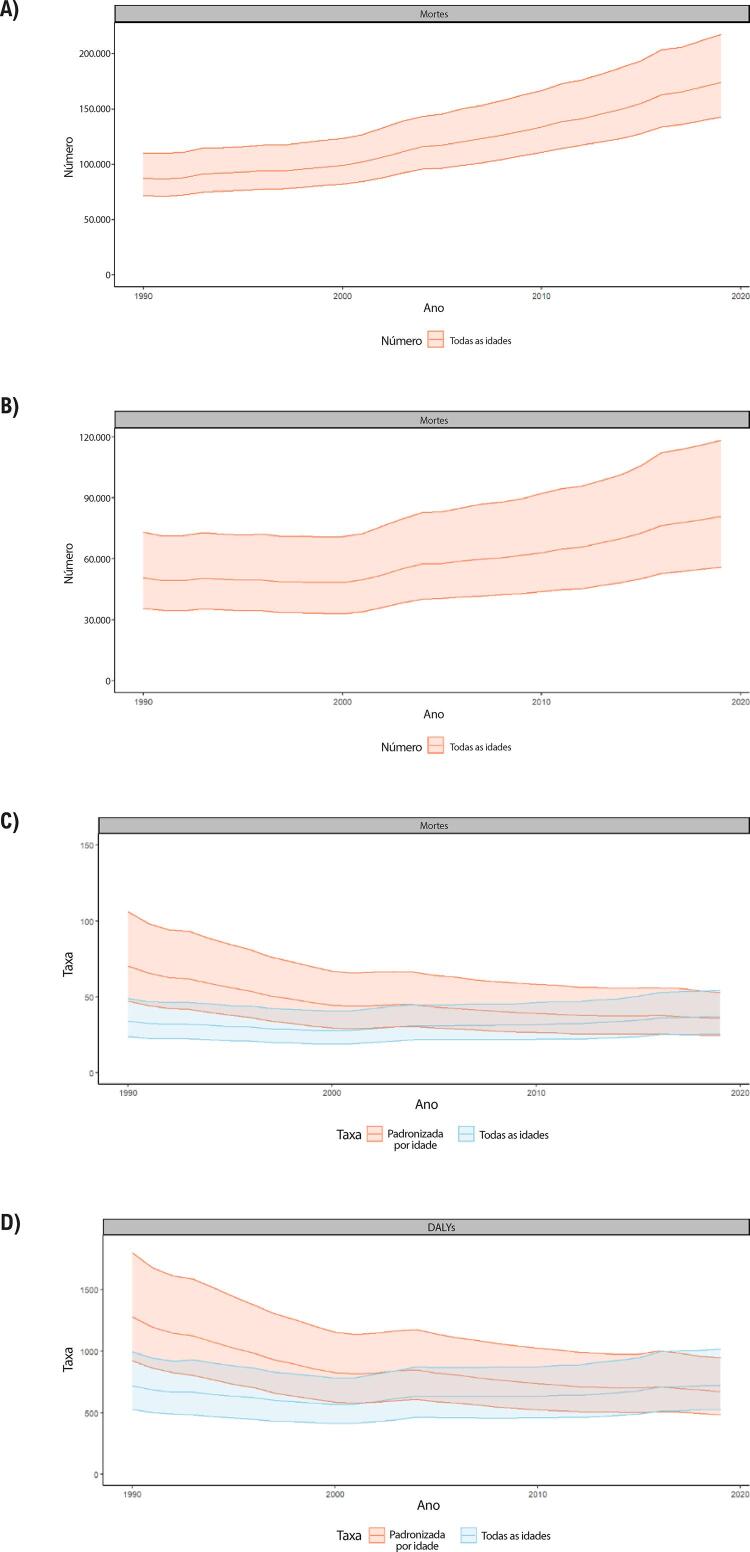
-Número total de mortes por diabetes (A). Por doença cardiovascular atribuível ao diabetes, número de mortes (B), taxa de mortalidade padronizada por idade (por 100 mil) (C) e taxa de DALYs (D). Brasil, 2019.

Fonte: Dados derivados do estudo Global Burden of Disease 2019, Institute for Health Metrics and Evaluation, University of Washington. ^46^

**Figura 8-2:**
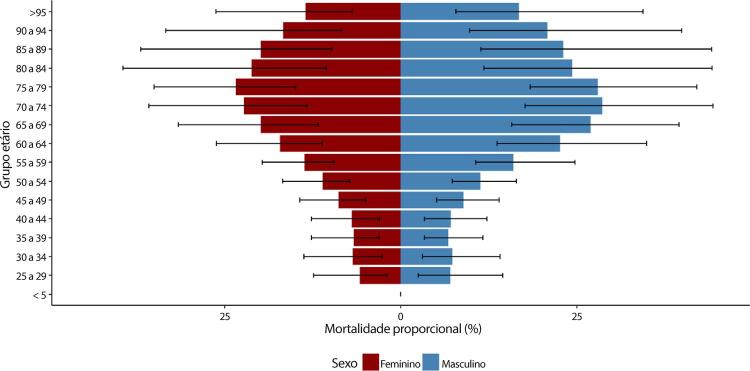
-Mortalidade proporcional por doença cardiovascular atribuível ao diabetes por sexo e grupo etário. Brasil, 2019.

Fonte: Dados derivados do estudo Global Burden of Disease 2019, Institute for Health Metrics and Evaluation, University of Washington. ^46^

**Figura 8-3:**
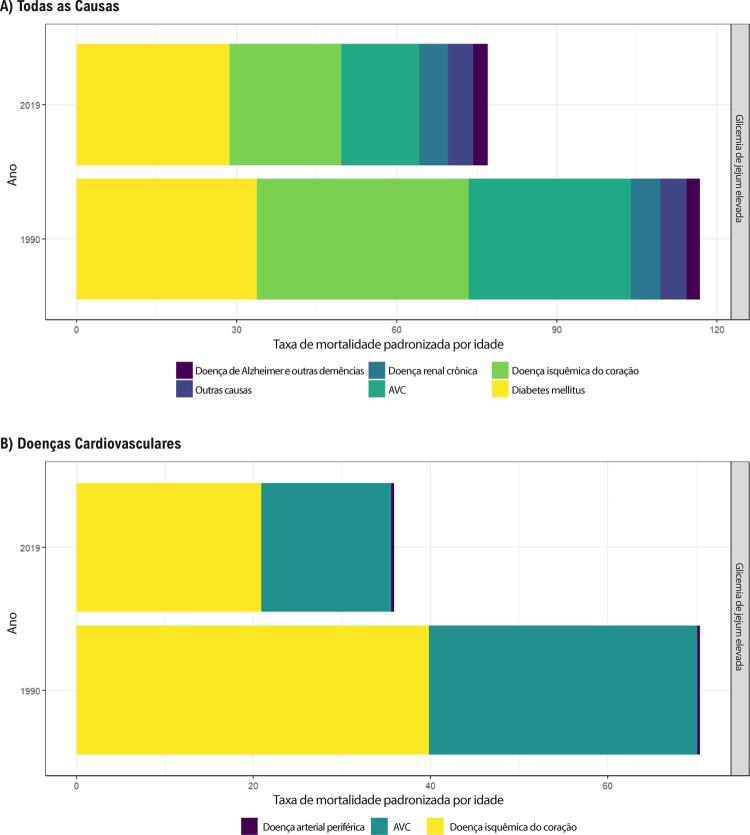
-Mortes atribuíveis ao diabetes estratificadas por todas as causas e por doenças cardiovasculares. Brasil, 2019.

Fonte: Dados derivados do estudo Global Burden of Disease 2019, Institute for Health Metrics and Evaluation, University of Washington. ^46^

**Figura 8-4:**
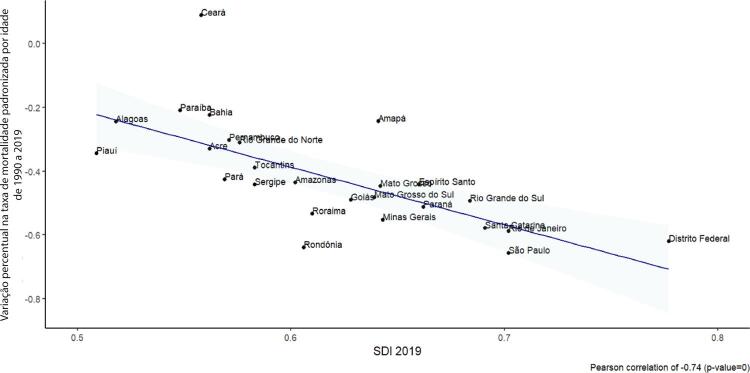
-Relação entre índice sociodemográfico (SDI) de 2019 e variação percentual nas mortes por doença cardiovascular atribuível ao diabetes de 1990 a 2019. Brasil e Estados.

Fonte: Dados derivados do estudo Global Burden of Disease 2019, Institute for Health Metrics and Evaluation, University of Washington. ^46^

**Table:** Abreviaturas usadas no Capítulo 8

CID	Classificação Internacional das Doenças
CID-10	Classificação Internacional das Doenças, 10ª Revisão
DALYs	Anos de vida perdidos ajustados por incapacidade (do inglês, *Disability-Adjusted Life-Years* )
DCV	Doença Cardiovascular
ELSA-Brasil	Estudo Longitudinal de Saúde do Adulto - Brasil
ERICA	Estudo de Riscos Cardiovasculares em Adolescentes
GBD	Carga Global de Doenças (do inglês, *Global Burden of Disease* )
HbA1c	Hemoglobina Glicada
HDL	Lipoproteína de Alta Densidade (do inglês, *High-Density Lipoprotein* )
HR	Hazard Ratio
IBGE	Instituto Brasileiro de Geografia e Estatística
IC	Intervalo de Confiança
IDF	Federação Internacional de Diabetes (do inglês, *International Diabetes Federation* )
II	Intervalo de Incerteza
IMC	Índice de Massa Corporal
LDL	Lipoproteína de Baixa Densidade (do inglês, *Low-Density Lipoprotein* )
MASS	Registro de Medicina, Angioplastia ou Cirurgia (do inglês, *The Medicine, Angioplasty or Surgery Study* )
MEV	Mudanças no Estilo de Vida
OR	Odds Ratio
PNAUM	Pesquisa Nacional sobre o Acesso, Utilização e Promoção do Uso Racional de Medicamentos no Brasil
PR	Prevalence ratio
REACT	Registro do Paciente de Alto Risco Cardiovascular na Prática Clínica
SBC	Sociedade Brasileira de Cardiologia
SDI	Índice Sociodemográfico (do inglês, *Sociodemographic Index)*
SF-36	Questionário de qualidade de vida SF-36 (do inglês, *Short Form 36* )
SIM	Sistema de Informação sobre Mortalidade
SUS	Sistema Único de Saúde
UF	Unidade Federativa
VIGITEL	Sistema de Vigilância de Fatores de Risco e Proteção para Doenças Crônicas por Inquérito Telefônico
YLDs	Anos vividos com incapacidade (do inglês, *Years Lived with Disability)*
YLLs	Anos de vida perdidos por morte prematura (do inglês, *Years of Life Lost)*

### Introdução

•Diabetes melito é uma doença crônica e progressiva, caracterizada por alterações metabólicas decorrentes de hiperglicemia persistente resultante de defeitos na secreção e/ou na ação da insulina produzida pelas células beta pancreáticas. Em longo prazo, a hiperglicemia persistente determina complicações microvasculares (retinopatia, doença renal do diabetes, neuropatia) e macrovasculares (doença arterial coronariana, doença cerebrovascular e doença arterial obstrutiva periférica). Pré-diabetes (tolerância diminuída à glicose e glicemia de jejum alterada) refere-se à condição em que a glicemia está elevada em relação aos parâmetros normais, porém sem atingir critérios para o diagnóstico de diabetes. A identificação do pré-diabetes é importante porque determina maior risco de diabetes no futuro e aumento de risco de DCV, além de que intervenções principalmente relacionadas a MEV comprovadamente reduzem o risco de diabetes nesses indivíduos.

•A classificação do diabetes baseia-se na sua etiologia. Os tipos mais prevalentes são o diabetes tipo 2 (90-95% dos casos) e o diabetes tipo 1 (5-10% dos casos). O diabetes tipo 2 ocorre em geral em adultos a partir dos 30-40 anos, tem etiologia complexa e multifatorial, envolvendo herança poligênica, influência familiar e influência de fatores ambientais (hábitos alimentares inadequados, inatividade física e sedentarismo). A fisiopatologia envolve resistência insulínica acompanhada de aumento inicial na produção de insulina, seguida, ao longo dos anos, de falência pancreática na produção desse hormônio. ^307^

•O diabetes tipo 1, em geral, ocorre em crianças, adolescentes ou adultos jovens, tem etiologia autoimune, poligênica, decorrente da destruição das células beta pancreáticas e deficiência completa de insulina. Pode ocorrer em adultos (antigo LADA – *latent autoimune diabetes in adults,* hoje mais comumente conhecido como diabetes tipo 1 do adulto). Subdivide-se em tipos 1A e 1B a depender da presença de autoanticorpos. No diabetes tipo 1A, autoanticorpos estão presentes, havendo predisposição genética associada a fatores ambientais desencadeantes da resposta imune (infecções virais, componentes da dieta e da microbiota intestinal). No diabetes tipo 1B, autoanticorpos não estão presentes e a etiologia é idiopática. A DCV é a principal causa de morte em pacientes com diabetes, que apresentam o dobro do risco de eventos cardiovasculares maiores quando comparados à população sem a doença, além de piores respostas aos tratamentos e piores desfechos após serem acometidos por algum evento cardiovascular (infarto agudo do miocárdio, acidente vascular cerebral). Os fatores de risco cardiovasculares clássicos (tabagismo, hipertensão arterial, colesterol LDL alto, colesterol HDL baixo) maximizam esse risco, além da duração do diabetes e da coexistência de complicações microvasculares. Estão incluídas nas complicações cardiovasculares do diabetes as doenças arteriais coronariana e periférica e cerebrovascular. ^308^ ^,^ ^309^

•O diabetes compromete diretamente a qualidade de vida, a produtividade e a sobrevida dos indivíduos, além de gerar importante impacto econômico para os sistemas de saúde. Atingir os alvos glicêmicos adequados e controlar os outros fatores de risco cardiovasculares comprovadamente contribuem para a prevenção e o retardo da progressão das complicações crônicas da doença. O acometimento sistêmico de diversos órgãos e a complexidade da doença exigem uma assistência global ao paciente, abrangendo os diversos aspectos que seu tratamento inclui. ^309^

•Neste capítulo, o diabetes mellitus será abordado como fator de risco cardiovascular, uma vez que assim foi identificado desde o clássico estudo de Framingham, ^2^ e sua presença, em associação a tabagismo, hipertensão arterial sistêmica e dislipidemia, aumenta em duas a três vezes o risco de DCV nesses indivíduos. ^310^

### Prevalência

•Teló *et al* ., em revisão sistemática com meta-análise de estudos observacionais brasileiros de 1980 a 2015, incluíram 50 estudos e mostraram uma crescente prevalência de diabetes nas últimas décadas, como se segue: diabetes autorreferido (36 estudos), 5,6% (IC 95% 5,0 - 6,3); diagnosticado por glicemia de jejum (7 estudos), 6,6% (IC 95% 4,8 - 8,9); e diagnosticado por teste oral de tolerância à glicose (7 estudos), 11,9% (IC 95% 7,7 - 17,8). Nas análises de tendência, houve aumento da prevalência de diabetes com o tempo, sendo o maior aumento observado em estudos que utilizaram o teste oral de tolerância à glicose no diagnóstico: 7,4% (IC 95% 7,1 - 7,7) nos anos 1980, 12,1% (IC 95% 10,5 - 13,8) nos anos 1990, 14,5% (IC 95% 13,1 - 16,0) nos anos 2000 e 15,7% (IC 95% 9,8 - 24,3) nos anos 2010. ^311^

•Considerando-se os dados da IDF publicados em 2019, o Brasil era o quinto país no mundo em quantidade de adultos com diabetes, totalizando 16,8 milhões (IC 95% 15,0 - 18,7) de pessoas com essa condição, das quais 46% desconheciam ter a doença. A prevalência de pré-diabetes foi de 9,5% (15,1 milhões de pessoas). ^312^

•Dados obtidos da Pesquisa Nacional de Saúde (2014 a 2015) mostraram as seguintes prevalências de diabetes de acordo com diferentes critérios: 6,6% (IC 95% 5,9 - 7,2) [critério de HbA1c ≥ 6,5%]; de 8,4% (IC 95% 7,6 - 9,1) [critério de HbA1c ≥ 6,5% ou uso de antidiabéticos]; de 9,4% (IC 95% 8,6 - 10,1) [critério de HbA1c ≥ 6,5% ou histórico de diabetes] e de 7,5% (IC 95% 6,7 - a 8,2) [critério de histórico de diabetes]. Essas informações estão apresentadas de acordo com sexo, região (Tabela 8-1), faixa etária, escolaridade, cor da pele autorreferida e IMC (Tabela 8-2), considerando-se o critério mais abrangente (HbA1c ≥ 6,5% ou uso de antidiabéticos). Em qualquer um dos critérios, a prevalência foi maior em mulheres, em indivíduos acima de 30 anos e naqueles com sobrepeso ou obesidade. Maior nível educacional associou-se com menor prevalência de diabetes. A região brasileira com maior prevalência foi a Centro-Oeste. ^28^

•Malta *et al.* , em estudo transversal que incluiu dados autorreferidos de 60.202 brasileiros, analisaram as desigualdades na prevalência autorreferida de doenças não transmissíveis, incluindo o diabetes. A prevalência de diabetes foi maior para indivíduos analfabetos/ensino fundamental incompleto e para aqueles com ensino médio incompleto, alcançando 9,61% (PR 1,42, IC 95% 1,13-1,77) e 5,36% (PR 1,59, IC 95% 1,23-2,06), respectivamente, enquanto para indivíduos com ensino superior completo, a prevalência foi de 4,18%. ^28^

•Iser *et al.* mostraram em dados obtidos da Pesquisa Nacional de Saúde (2014 a 2015) que a prevalência de pré-diabetes pelo critério da Associação Americana de Diabetes (HbA1c 5,7 a 6,4%) foi de 18,5% (IC 95% 17,4 - 19,7) e, pelo critério da Organização Mundial da Saúde (HbA1c 6,0-6,4%), de 7,5% (IC 95% 6,7 - 8,3). ^313^

•Teló *et al.* publicaram em 2019 resultados de grande estudo transversal desenvolvido em amostra representativa de estudantes brasileiros de 12 a 17 anos mostrando que, dentre os 37.854 jovens incluídos, 3,3% (IC 95% 2,9 - 3,7) tinham diabetes tipo 2 (HbA1c ≥ 6.5% ou glicemia de jejum ≥ 126 mg/dl ou histórico de diabetes) e 22,0% (IC 95% 20,6 - 23,4) tinham pré-diabetes (HbA1c entre 5,7% e 6,5% ou glicemia de jejum entre 100 e 126 mg/dl). ^314^

### Incidência

•Estudos de incidência de diabetes reportam em sua maioria dados de países de alta renda e, em geral, de populações adultas, de forma que refletem especialmente a incidência de diabetes tipo 2. O diabetes tipo 2 em crianças e adolescentes tem crescido em muitos países, devido ao aumento na prevalência de sobrepeso, obesidade, inatividade física e sedentarismo. ^312^

•Schmidt *et al.* publicaram análise de seguimento a partir de 2008-2010 por 3,7 ± 0,63 anos de 11.199 funcionários públicos sem diabetes do estudo multicêntrico brasileiro ELSA-Brasil. Diabetes foi diagnosticado no seguimento se glicemia de jejum ≥ 126 mg/dl ou glicemia após sobrecarga oral de glicose ≥ 200 mg/dl ou HbA1c ≥ 6,5%. A incidência cumulativa de diabetes foi de 2,0/100 pessoas-ano (IC 95% 1,8 - 2,1), semelhante entre homens e mulheres, maior nos indivíduos acima de 65 anos [2,8%; IC 95% 2,3 - 3,4], com obesidade (3,8%; IC 95% 3,4 - 4,3) e naqueles com menor nível educacional (3,0%; IC 95% 2,6 - 3,6). ^315^

•Sitnik *et al.* reportaram coorte prospectiva de 1.536 indivíduos sem diabetes, funcionários da Universidade de São Paulo, participantes do Projeto ELSA-Brasil com idade entre 23 anos e 63 anos em 1998 (data da coleta da glicemia de jejum), com o objetivo de avaliar a associação entre glicemia de jejum, incidência de diabetes, aterosclerose subclínica e eventos cardiovasculares. A taxa de incidência ajustada de diabetes foi de 9,8/1.000 pessoas-ano (IC 95% 7,7 - 13,6/1.000 pessoas-ano). ^316^

•O número de crianças e adolescentes com diabetes tipo 1 tem crescido em todo o mundo, aproximadamente 3% ao ano, embora com importantes diferenças regionais. ^6^ ^,^ ^12^ ^,^ ^13^ O Brasil é o terceiro no *ranking* mundial em número de novos casos de crianças e adolescentes de 0 a 14 anos com diabetes tipo 1 (7,3 casos/1000/ano) e também em prevalência da doença na mesma faixa etária (51,1/1000). ^312^

•Negrato *et al.* descreveram a incidência anual de diabetes tipo 1 de 1986 a 2015 na cidade de Bauru (São Paulo) em crianças ≤ 14 anos, usando método de notificação de casos e *capture and recapture* . Foram identificados 302 casos nesse período, uma incidência (por 100 mil) de 12,8 (IC 95% 11,2 - 14,4), variando de 2,8 em 1987 a 25,6 em 2013, sem diferença entre sexos. ^319^

### Mortalidade

#### Mortalidade Geral Atribuível ao Diabetes

•Klafke *et al.* descreveram dados de mortalidade por complicações agudas por diabetes (cetoacidose, estado hiperosmolar, hipoglicemia) no Brasil, para todas as idades, entre 1991 e 2010, período em que ocorreu a implantação do SUS. Usando dados do IBGE e do SIM, foram registradas 694.769 mortes por diabetes nesse período, das quais 81.208 (11,7%) ocorreram por complicações agudas. Em 1991, houve 2.070 mortes em homens por complicações agudas do diabetes, com taxa de mortalidade padronizada por idade por 100 mil habitantes de 7,4 (IC 95% 7,2 - 7,6), e 2.832 mortes em mulheres, com taxa de mortalidade de 9,7 por 100 mil habitantes (IC 95% 9,1 - 9,6), gerando relação de taxa de mortalidade mulher/homem de 1,3. Em comparação com 1991, em 2010 houve 1.600 mortes em homens por complicações agudas do diabetes, com taxa de mortalidade de 2,4 por 100 mil habitantes (IC 95% 2,3 - 2,5), e 2.141 mortes em mulheres, com taxa de mortalidade de 2,5 por 100 mil habitantes (IC 95% 2,4 - 2,6), gerando relação de taxa de mortalidade mulher/homem de 1,0. Desse modo, a mortalidade por complicações do diabetes em 20 anos para ambos os sexos diminuiu 70,9% (IC 95% 67,2 - 74,5), de 8,42 (IC 95% 8,3 - 8,6) mortes por 100 mil habitantes em 1991 para 2,45 por 100 mil habitantes (IC 95% 2,4 - 2,5) em 2010. Essa redução ocorreu em ambos os sexos, em todas as idades e em todas as regiões do Brasil. ^320^

•Malhão *et al.* avaliaram o padrão de mortalidade por diabetes no Brasil, por sexo, em adultos ≥ 20 anos, de 1980 a 2012, usando dados do IBGE e do SIM. De 1980 a 2012, 955.455 pessoas morreram considerando-se diabetes como principal causa. Mulheres representaram 57.7% (551.016) das mortes e homens, 42,3% (404.439). Nesse período, as taxas de mortalidade padronizadas por idade por 100 mil habitantes aumentaram de 20,8 (IC 95% 20,2 - 21,5) para 47,6 (IC 95% 47,0 - 48,2) nos homens e de 28,7 (IC 95% 27,9 - 29,4) para 47,2 (IC 95% 46,7 - 47,7) nas mulheres. Considerando todo o período analisado, a taxa aumentou 2,9% ao ano nos homens (variação percentual média anual de 2,9; IC 95% 2,6 - 3,1) e 1,7% nas mulheres (variação percentual média anual de 1,7; IC 95%: 1,5 - 1,9). Considerando o diabetes como a causa principal ou associada de mortalidade entre 2001 e 2012, o número de mortes foi de 1.076.434 (mulheres 603.686 – 56,1%; homens 472.748 – 43,9%). A taxa de mortalidade padronizada por idade por 100 mil habitantes aumentou nesse período de 76,1 (IC 95% 75,2 - 77,0) para 95,6 (IC 95% 94,8 - 96,5) nos homens, e de 83,7 (IC 95% 82,9 - 84,6) para 93,3 (IC 95% 92,6 - 94,1) nas mulheres. ^321^

•Duncan *et al.* , usando dados sobre diabetes e hiperglicemia do Estudo GBD 2015 para todas as idades, mostraram que no Brasil, em 2015, a taxa de mortalidade por diabetes por 100 mil habitantes foi de 26,8 (IC 95% 25,0 - 28,5) para homens e de 33,2 (IC 95% 31,1 - 35,2) para mulheres. ^322^

•Duncan *et al.* , usando dados sobre diabetes e hiperglicemia do Estudo GBD 2016-2017 para todas as idades, mostraram que, nesse período, para o diabetes tipo 1, a mortalidade padronizada por idade diminuiu mais de 50% para mulheres e cerca de 10% para homens, aproximando assim as taxas específicas de gênero, que eram muito diferentes no início do período. Taxas de mortalidade padronizadas por idade para o diabetes tipo 2, em contraste, estiveram estáveis durante o período, com ligeira diminuição nas mulheres e ligeiro aumento nos homens. Quando traduzida em taxas brutas, no entanto, a mortalidade por diabetes tipo 2 aumentou de forma dramática, basicamente dobrando no período. Os autores também mostraram que as maiores taxas de mortalidade por diabetes em 2017 ocorreram principalmente nos estados do Nordeste, sendo que os maiores aumentos na mortalidade entre 1990 e 2017 foram observados no Norte, Nordeste e Centro-Oeste, com as maiores diminuições observadas no Sudeste. ^323^

•Os dados do Estudo GBD 2019 mostram que, embora as taxas de mortalidade padronizada por idade por diabetes tenham diminuído substancialmente nos últimos anos no Brasil, o número total de mortes pela doença aumentou ( Figura 8-3 A). Houve 87.644 (IC 95% 71.924 -110.625) e 174.198 (IC 95% 142.704 - 217.111) mortes por diabetes no país em 1990 e 2019, respectivamente ( Figura 8-1 A). A taxa de mortalidade padronizada por idade por 100 mil habitantes foi 116,8 (IC 95% 92,8 - 152,0) em 1990 e 77,0 (IC 95% 63,0 - 96,7) em 2019, diminuindo em 34% (IC 95% -40,1 a -28,1) no período. Essa redução não foi homogênea entre as regiões e UF do Brasil, sendo mais pronunciada no Distrito Federal e nas UF do Sudeste, Centro-Oeste e Sul, com redução modesta a pouco significativa em diversas UF do Norte e Nordeste. É importante destacar que a tendência à redução da taxa de mortalidade padronizada por idade por diabetes ocorreu em todos os grupos etários, mas com redução mais significativa na população de 5-14 anos (-45,8%; IC 95% -57,2 a -33,1) e menos significativa para a população de 15-49 anos (-24.8%; IC 95% -30,7 a -18,5), com reduções intermediárias nos grupos etários com mais de 70 anos (-26%; IC 95% -33,2 a -18,2) e 50-69 anos (-33,2%; IC 95% -40,1 a -26,4). ^46^

## Mortalidade Cardiovascular Atribuível ao Diabetes

•A mortalidade por DCV no Brasil atribuível ao diabetes para todas as idades, segundo dados do Estudo GBD 2019, aumentou em termos absolutos de 50.812 mortes (IC 95% 35.649 - 73.137) em 1990 para 80.754 (IC 95% 55.922 - 118.175) em 2019 (Tabela 8-3 e Figura 8-1 B). No entanto, as taxas de mortalidade padronizada por idade por 100 mil habitantes diminuíram de 70,4 (IC 95% 47,4 - 106,1) em 1990 para 35,9 (IC 95% 24,5 - 53,0) em 2019, expressando uma redução de -49,0% (IC 95% -53,4 a -43,9) ( Figura 8-1 C). Essa tendência de redução ocorreu de forma não uniforme nas UF, sendo mais significativa na maioria das UF das regiões Sul, Sudeste, Centro-Oeste e Norte e Distrito Federal, e apenas modesta nas UF da região Nordeste. Na comparação entre sexo, as mulheres apresentaram maiores reduções da taxa de mortalidade em comparação aos homens na maioria das UF do Brasil nos dados de 1990 e 2019, exceto no Maranhão, Piauí e Distrito Federal (Tabela 8-4). Quanto à tendência por grupo etário, a redução da taxa de mortalidade por DCV atribuível ao diabetes ocorreu em todas os grupos etários: 15 a 49 anos (-37,3%; IC 95% -46,8 a -25,3), 50 a 69 anos (-46,0%; IC 95% -54,2 a -35,5) e mais de 70 anos de idade (-43,5%; IC 95% -50,3 a -36,7). É importante destacar que em 2019, na comparação por sexo e grupos etários ( Figura 8-2 ), as mulheres tiveram menores taxas de mortalidade proporcional por DCV atribuível ao diabetes em praticamente todos os grupos etários descritos. ^46^

•Apesar de os dados do Estudo GBD 2019 demonstrarem que o número total de mortes atribuíveis ao diabetes, para todas as idades e estratificadas por todas as causas, aumentou no Brasil entre 1990 e 2019 ( Figura 8-3 A), a taxa de mortalidade padronizada por idade por 100 mil habitantes diminuiu no mesmo período de 116,8 (IC 95% 92,8 - 152,0) para 77 (IC 95% 63,0 - 96,7). É importante destacar que mais de 85% dessa redução ocorreu devido à diminuição das taxas de doença isquêmica do coração, de 39,8% (IC 95% 21,3 -65,3) em 1990 para 20,9% (IC 95% 11,6 - 34,4) em 2019, e de acidente vascular encefálico, de 30,3% (IC 95% 18,2 - 54,8) em 1990 para 14,7% (IC 95% 8,6 - 25,6) em 2019 ( Figura 8-3 B). ^46^

•O SDI é uma estimativa do nível socioeconômico de uma determinada localidade e a Figura 8-4 mostra a relação entre o SDI de 2019 e a variação percentual nas mortes por DCV atribuíveis ao diabetes de 1990 a 2019. Os dados revelam que há correlação entre a maior variação percentual de taxa de mortalidade padronizada pela idade entre 1990 e 2019 e o SDI de 2019, sugerindo que a redução de mortalidade por DCV seguiu a melhora nas condições socioeconômicas. As UF com menores SDI tiveram menores variações percentuais nas mortes (mais mortes), com exceção de Roraima e Rondônia, enquanto as UF com maiores SDI tiveram as maiores variações percentuais nas mortes (menos mortes), como São Paulo, Rio de Janeiro e Distrito Federal.

## Carga de Doença

### Carga de Doença Atribuível ao Diabetes

•Os dados do Estudo GBD 2019 estimaram uma redução da taxa de DALYs atribuíveis ao diabetes padronizada por idade por 100 mil habitantes no Brasil de -26,1% (IC 95% -31,7 a -20,9) entre 1990 e 2019, apesar de ter ocorrido aumento no número total de DALYs de 2.448.714,5 (IC 95% 2.087.403,6 - 2.919.735,6) para 4.778.225,9 (IC 95% 4.017.716,8 -5.709.063,5) no período. A redução da taxa de DALYs, apesar de ter ocorrido em todos os grupos etários, foi mais pronunciada em UF das regiões Sudeste, Sul e Centro-Oeste e no Distrito Federal, sendo pouco expressiva em muitas UF das regiões Norte e Nordeste, incluindo aumento da taxa de DALYs no Amapá, Ceará e Maranhão. Na comparação entre sexos, a redução da taxa foi mais expressiva nas mulheres (-37.7%; IC 95% a 44.2 a -31.1) do que nos homens (-29.9%; IC 95% 36.9 a -22.3) para o mesmo período. ^46^

### Carga de Doença Cardiovascular Atribuível ao Diabetes

•Quanto à carga de DCV, o Estudo GBD 2019 estimou redução de DALYs atribuíveis ao diabetes no Brasil ( Figura 8-1 D e Tabela 8-5), taxa essa padronizada por idade por 100 mil habitantes, de -47,4% (IC 95% - 52,2 a -41,9) entre 1990 e 2019, apesar de ter ocorrido um aumento no número total de DALYs de 1.072.309 (IC 95% 784.276 -1.484.959) para 1.571.116 (IC 95% 1.140.912 - 2.203.188) no período. Essa redução da taxa ocorreu devido à redução dos YLLs, da ordem de -33,4% (IC 95% -42,5 a -15) entre 1990 e 2019. No mesmo período, houve aumento dos YLDs da ordem de 17,6% (IC 95% 0,4 - 50,5). ^46^

•Houve redução heterogênea da taxa de DALYs padronizada por idade por diabetes entre as diversas UF e regiões do Brasil, sendo mais acentuada nas UF das regiões Sudeste, Sul e Centro-Oeste e no Distrito Federal, com redução modesta nas UF na região Norte e ainda mais discreta na maioria das UF do Nordeste.

•Os dados do Estudo GBD 2019 demonstram que a redução da taxa de DALYs por diabetes padronizada por idade de 1990 a 2019 ocorreu em todos os grupos etários: 15 a 49 anos (-36,5%; IC 95% -46,0 a -24,5), 50 a 69 anos (-45,5%; IC 95% -53,4 a -35,2) e mais de 70 anos (-46,2%; IC 95% -52,5 a -39,1). Para homens de todas as idades, a variação foi de -43,1% (IC 95% -49,4 a -35,1) e, para mulheres de todas as idades, a variação foi de -52,0% (IC 95% -58,5 a -44,5). Apenas no Maranhão e no Piauí, os homens tiveram maior redução em comparação às mulheres. ^46^

### Impacto na Saúde Cardiovascular

•Fuchs *et al.* , em estudo transversal realizado em 2005 na cidade de Porto Alegre, Rio Grande do Sul, com adultos de 18-90 anos, avaliou a associação entre o agrupamento de fatores de risco e DCV autorreferida. Os participantes foram entrevistados em casa sobre a presença de diabetes, atividade física e padrão alimentar, além de avaliados quanto à presença de hipertensão arterial sistêmica. Uma amostra de 1.007 mulheres, a maioria branca (73,0%), idade média de 44,8 anos (±0,8) e média de escolaridade de 9,3 anos (±0,3), foi investigada. Hipertensão arterial, diabetes, obesidade, baixa ingestão de frutas e vegetais e falta de atividade física vigorosa ou moderada foram agrupados em uma combinação de fatores de risco, que se associaram de forma independente à DCV autorreferida. O grupo principal dessa associação incluiu a presença de hipertensão arterial e diabetes, representando uma razão de risco independente de 8,5 (IC 95% 3,0 - 24,5). ^281^

•Cardoso *el al.* reportaram os resultados de estudo observacional prospectivo realizado no Rio de Janeiro entre agosto de 2004 e dezembro de 2008 em 620 indivíduos adultos com diabetes tipo 2, seguidos até agosto de 2013 em ambulatório de diabetes de hospital universitário brasileiro. Os autores objetivaram relacionar os níveis de HbA1c com desfechos cardiovasculares. Níveis de HbA1c foram medidos no início do estudo e 3-4 vezes ao ano ao longo do seguimento clínico. Os desfechos primários foram compostos por eventos cardiovasculares totais (fatais e não fatais), eventos cardiovasculares maiores e mortalidade por todas as causas. Mortalidade cardiovascular e não cardiovascular foram os desfechos secundários. A amostra teve idade média de 60,4 anos (± 9,4), 37,1% eram homens, 55% caucasianos. Após acompanhamento mediano de 79 meses (59-93), 125 eventos cardiovasculares totais ocorreram (90 eventos maiores), com um total de 111 óbitos, 64 dos quais por DCV. Após ajustes estatísticos para outros fatores de risco cardiovascular, a linha de base da HbA1c e a HbA1c média do primeiro ano foram preditoras de eventos cardiovasculares totais (HR 1,13; IC 95% 1,04 - 1,23 e HR 1,26; IC 95% 1,12 - 1,41, respectivamente), de eventos cardiovasculares maiores (HR 1,15; IC 95% 1,04 - 1,28 e HR 1,27; IC 95% 1,11 - 1,45, respectivamente), de mortalidade por todas as causas (HR 1,10; IC 95% 1,00 - 1,21 e HR 1,18 e IC 95% 1,04 - 1,35, respectivamente) e mortalidade cardiovascular (HR 1,14; IC 95% 1,01 - 1,27 e HR 1,27; IC 95% 1,08 - 1,50, respectivamente). Cada aumento de 1% na média de HbA1c do primeiro ano aumentou 27,0% (IC 95% 11,0 - 45,0) o risco de eventos cardiovasculares maiores. Proteção cardiovascular foi observada até valores de HbA1c menores que 6,5%. Já HbA1c no segundo ano não foi preditiva para nenhum dos desfechos (HR 1,12; IC 95% 0,98 - 1,28; p=0,09 para eventos cardiovasculares totais; HR 1,09; IC 95% 0,92 - 1,29; p=0,32 para eventos cardiovasculares maiores). ^324^

•Sitnik *et al.* reportaram coorte prospectiva de 1.536 indivíduos sem diabetes, funcionários da Universidade de São Paulo, participantes do Projeto ELSA-Brasil com idade de 23-63 anos em 1998 (data da coleta da glicemia de jejum), com o objetivo de avaliar a associação entre glicemia de jejum e incidência de diabetes com aterosclerose subclínica e eventos cardiovasculares. Níveis de glicemia de jejum de 110-125 mg/dl foram associados a maior espessura da íntima-média da carótida (β 0,028; IC 95% 0,003 - 0,053). Excluindo aqueles indivíduos que desenvolveram diabetes no seguimento, houve associação limítrofe entre maior espessura da íntima-média da carótida e glicemia de jejum de 110-125 mg/dl (β 0,030; IC 95% -0,005 a 0,065). O diabetes foi associado a maior espessura da íntima-média da carótida (β 0,034; IC 95% 0,015 - 0,053), escores de cálcio coronariano ≥ 400 (OR 2,84; IC 95% 1,17 - 6,91) e o resultado combinado de escore de cálcio coronariano ≥ 400 ou evento cardiovascular (OR 3,50; IC 95% 1,60 - 7,65). ^316^

•Schaan *et al.* , através do registro multicêntrico brasileiro, Estudo REACT, realizado de julho de 2010 a maio de 2016, objetivaram estabelecer o risco de longo prazo de eventos clínicos em pacientes de alto risco cardiovascular no Brasil. Esse projeto foi idealizado e coordenado pela SBC com a participação de centros privados e públicos de todas as regiões do Brasil, respeitando a distribuição da população segundo dados do IBGE. Um total de 5.006 indivíduos (≥ 45 anos de idade) foi incluído e analisado em quatro grupos: sem diabetes e nenhum evento cardiovascular anterior (n = 430); com diabetes e nenhum evento cardiovascular anterior (n = 1.138); sem diabetes e com evento cardiovascular anterior (n = 1.747); e com diabetes e com evento cardiovascular anterior (n = 1.691). Evento cardiovascular anterior foi definido como evidência de doença arterial coronariana, acidente vascular encefálico ou acidente isquêmico transitório, doença arterial periférica, presença de três fatores de risco cardiovascular, exceto diabetes (hipertensão arterial, dislipidemia, idade acima de 70 anos, história familiar de doença arterial coronariana, doença carotídea assintomática). Ocorreram eventos clínicos maiores (mortalidade por todas as causas, infarto do miocárdio não fatal, parada cardiorrespiratória não fatal e acidente vascular encefálico não fatal) no acompanhamento de um ano em 332 pacientes. Evento cardiovascular anterior foi associado a maior risco de outro evento no acompanhamento (HR 2,31; IC 95% 1,74 - 3,05, p<0,001), assim como a presença de diabetes (HR 1,28; IC 95% 1,10 - 1,73, p=0,005). Em pacientes com diabetes, a falha em alcançar os alvos de HbA1c foi relacionada a pior sobrevida livre de eventos em comparação a pacientes com bom controle metabólico (HR 1,70; IC 95% 1,01 - 2,84, p=0,044). ^325^

•Rezende *et al.* reportaram os dados retrospectivos de pacientes com diabetes tipo 2 e doença arterial coronariana multivascular inscritos no MASS do Instituto do Coração da Universidade de São Paulo de janeiro de 2003 a dezembro de 2007. Os pacientes foram acompanhados em consultas ambulatoriais a cada seis meses e avaliados prospectivamente para eventos cardiovasculares. De 888 pacientes com diabetes tipo 2 e doença arterial coronariana multiarterial, 725 (81,6%) tinham informações clínicas e de HbA1c completas para análise. A amostra foi caracterizada por idade mediana de 62,4 anos (55,7-68,0), 467 homens (64,4%), período mediano de seguimento de 10,0 anos (8,0-12,3) e média de 9,5 ± 3,8 medidas de HbA1c por paciente. O desfecho composto de morte, infarto do miocárdio ou acidente vascular cerebral isquêmico ocorreu em 262 pacientes (36,1%) da amostra. Aumento de um ponto no valor longitudinal de HbA1c foi significativamente associado a risco 14% maior do desfecho combinado de mortalidade por todas as causas, infarto do miocárdio e acidente vascular cerebral isquêmico (HR 1,14; IC 95% 1,04 - 1,24; p=0,002) na análise não ajustada. Depois de ajustar os fatores de linha de base (idade, sexo, doença arterial coronariana de dois ou três vasos, tratamentos da doença arterial coronariana inicial, fração de ejeção, níveis de creatinina e níveis de colesterol LDL), aumento de um ponto no valor longitudinal de HbA1c foi associado a risco 22% maior do desfecho combinado (HR 1,22; IC 95% 1,12 - 1,35; p<0,001). ^326^

## Conhecimento, Tratamento e Controle de Condição

•O tratamento do diabetes baseia-se em três pilares: dieta, exercício físico e medicamentos (antidiabéticos orais e insulina). Estudo transversal, multicêntrico publicado em 2016 por Gomes e Negrato ^327^ descreveu dados de amostra de conveniência de 1.698 pessoas com diabetes tipo 1 de dez cidades brasileiras quanto à sua adesão ao tratamento (questionário de Morisky), que foi máxima em 9,8% delas, moderada em 42,2% e mínima em 48,0%, associando-se menor adesão a maiores valores de HbA1c (9,2 ± 2,2%, 8,9 ± 2,0% e 8,6 ± 1.9%, respectivamente) para cada grupo avaliado. ^327^

•Ensaio clínico randomizado envolvendo 238 pacientes com diabetes tipo 2 comparou um programa de empoderamento baseado em protocolo de mudança comportamental e seu efeito sobre o controle glicêmico, mostrando que o grupo que foi randomizado para a intervenção obteve menores índices de Hba1c (7,5 ± 1,7% *vs.* 8,1 ± 2,2%). ^328^

•Meiners *et al.* em 2017, a partir de dados do inquérito domiciliar de base populacional PNAUM com delineamento transversal baseado em amostra probabilística da população brasileira, avaliaram 2.624 pessoas com diabetes e idade > 20 anos quanto a acesso e adesão ao tratamento. O acesso total aos medicamentos pesquisados foi de 98%, enquanto a adesão foi provável em 71% (IC 95% 67,2 - 74,5), provavelmente baixa em 9,8% (IC 95% 8,0 - 12,0) ou baixa em 17,2% (IC 95% 14,6 -20,1), com as melhores taxas de adesão na região Centro-Oeste. ^329^

•A aderência ao tratamento (autorreferida) e seus fatores associados foi avaliada por Marinho *et al.* em 2018 em estudo transversal com 476 pacientes diabéticos de um hospital terciário. Boa adesão foi de 93,5% para uso de medicamentos, 59,3% para cuidados com os pés, 56,1% para monitoramento da glicemia, 29,2% para dieta e 22,5% para exercícios físicos. Foram associados à boa adesão menor idade, menor IMC, presença de complicações macrovasculares e melhor desempenho ocupacional e no domínio emocional do SF-36. ^330^

•Silva *et al.* em 2018 realizaram inquérito domiciliar em 63 municípios de Minas Gerais selecionados por conveniência, com o objetivo de analisar o perfil de utilização de medicamentos em pacientes com diabetes naquele estado, avaliando 2.619 pessoas com diabetes (83,7% com diabetes tipo 2 e 10,4% com diabetes tipo 1, idade média de 61,3 ± 16,4 anos). Foi relatado o uso de 13.629 medicamentos, 60% adquiridos em farmácias públicas e 35% genéricos.

Os medicamentos mais frequentes em uso foram metformina, losartan, glibenclamida e sinvastatina; 56,5% (IC 95% 3,4) dos entrevistados estavam em polifarmácia (uso de cinco ou mais medicamentos). Os fatores associados à ocorrência de polifarmácia nesses pacientes foram idade de 40-59 anos (OR 2,46; IC 95% 1,68 - 3,61), idade acima de 60 anos (OR 4,58; IC 95% 3,18 - 6,60), autopercepção de saúde ruim ou muito ruim (OR 1,75; IC 95% 1,26 - 2,38), presença de cinco ou mais comorbidades (OR 3,45; IC 95% 2,84 - 4,19), tempo médio de diagnóstico acima de 10 anos (OR 1,64; IC 95% 1,36 - 1,98), quatro ou mais consultas médicas no último ano (OR 1,79; IC 95% 1,48 - 2,16), ausência de atividade física regular (OR 1,47; IC 95% 1,22 - 1,78), interrupção das atividades habituais nos últimos 15 dias (OR 1,30; IC 95% 1,03 - 1,64) e possuir plano de saúde particular (OR 1,39; IC 95% 1,13 - 1,70). ^331^

•Leitão *et al.,* em estudo transversal de base populacional com indivíduos de 20 anos ou mais que referiram diagnóstico de diabetes, entrevistados pelo Sistema VIGITEL de 2012 a 2018, estimaram a prevalência de uso de antidiabéticos orais e a distribuição das suas fontes de obtenção segundo variáveis sociodemográficas no Brasil. A prevalência de uso de antidiabéticos orais no Brasil passou de 77,4% (IC 95% 74,3 - 80,1) em 2012 para 85,2% (IC 95% 82,8 - 87,2) em 2018. Observou-se aumento de uso na região Sul, crescendo de 73,4% (IC 95% 67,8 - 78,4) em 2012 para 84,9% (IC 95% 79,7 - 88,9) em 2018. Quanto à obtenção de antidiabéticos orais, observou-se diminuição nas farmácias de unidades de saúde do SUS e aumento nas farmácias populares, sem mudança significativa nas farmácias privadas. ^332^

•Estudo transversal com amostra de conveniência a partir de 20 centros médicos brasileiros com dados de atendimentos ocorridos entre 2006 e 2007 descreveu o controle glicêmico de 5.692 pessoas acima de 18 anos com diabetes, 1.904 homens e 3.788 mulheres. A HbA1c estava acima de 7,0% em 72% dos homens e 74% das mulheres. ^333^

•Schneiders *et al* ., em coorte retrospectiva com 488 pacientes em atendimento em nível primário (n=192) e terciário (n=192), avaliaram indicadores de qualidade do cuidado ao diabetes em pessoas com uma HbA1c avaliada no último ano, a saber: avaliação anual de doença renal do diabetes, retinopatia diabética, neuropatia diabética, perfil lipídico, avaliação nutricional e abordagem de cessação de fumo. Apenas 14 pacientes (7,3%) na atenção primária e 52 (27,0%) na terciária tinham avaliados pelo menos 50% desses indicadores de qualidade. As maiores diferenças no cuidado reportado pelos pacientes da atenção primária e terciária foram: avaliação da doença renal do diabetes (84,8% na atenção primária *vs.* 95,8% na terciária), da retinopatia diabética (13,2% na atenção primária *vs.* 35,9% na terciária) e da neuropatia (9,5% na atenção primária *vs.* 58,9% na terciária); e avaliação nutricional (17,2% na atenção primária *vs.* 38,0% na terciária). ^334^

•Alessi *et al* . conduziram estudo transversal multicêntrico na atenção primária e terciária em idosos com diabetes tipo 2 (>65 anos, n=322, 160 da atenção primária e 162 da terciária) buscando avaliar quantitativo de pacientes que teriam controle glicêmico adequado considerando-se a necessidade de alvos glicêmicos customizados em boa parte dessa população. Foram considerados dentro do alvo glicêmico pacientes com HbA1c entre 7,0% e 7,5% quando tivessem expectativa de vida >10 anos; HbA1c entre 7,5% e 8,0% quando tivessem expectativa de vida entre 5 e 10 anos; e HbA1c entre 8,0% e 8,5% quando tivessem expectativa de vida <5 anos. A HbA1c estava fora do alvo em 49,1% e 50,3% dos pacientes na atenção primária e na terciária, respectivamente. Na amostra total, 42,2% dos pacientes estavam acima do alvo de HbA1c, 28,9% estavam no alvo e 28,9% estavam abaixo do alvo. ^335^

## Fatores de Risco e Prevenção

•Obesidade, constituição da dieta, inatividade física e sedentarismo são fatores de risco conhecidos para o desenvolvimento de diabetes tipo 2. A prevalência de diabetes aumenta claramente à medida que aumenta a prevalência de obesidade. ^336^ O diabetes pode ser prevenido ou seu início postergado com MEV, modificações na composição da dieta e medicamentos, na maioria antidiabéticos orais.

•Revisão sistemática com meta-análise publicada por Sbaraini *et al.* em 2021 compilou dados de 151 estudos de prevalência de sobrepeso e obesidade em adolescentes brasileiros de 10-19 anos. Observou-se aumento na prevalência de sobrepeso de 8,2% (IC 95% 7,7 - 8,7) até 2000, de 18,9% (IC 95% 14,7 - 23,2) de 2000 a 2009, e de 25,1% (IC 95% 23,4 - 26,8) de 2010 em diante, padrão que foi similar para a prevalência de obesidade. Regiões Sudeste e Sul tiveram maiores prevalências de sobrepeso e obesidade, sem diferença entre os sexos. ^337^

•Os mesmos autores mostraram, em 37.892 adolescentes do Estudo ERICA, sobrepeso em 17,2%, obesidade em 5,6% e obesidade grave em 1,3%, com aumento da chance de desfechos cardiometabólicos adversos de acordo com o maior IMC, incluindo maior glicemia de jejum [RP 5,30 (IC 95% 1,94 - 14,50)] e HbA1c (2,04, IC 95% 1,29 - 3,25). ^338^

•Flor *et al.* estimaram a relação do diabetes tipo 2 em adultos com mais de 20 anos de idade e sua fração atribuível a sobrepeso e obesidade no Projeto Carga de Doença do Brasil 2008. Os resultados mostraram que 49,2%, 58,3% e 70,6% do diabetes no sexo feminino foi atribuível a sobrepeso, obesidade e excesso de peso, respectivamente. Entre os homens, esses percentuais foram de 40,5%, 45,4% e 60,3%, respectivamente. Diferenças foram observadas nas diferentes regiões brasileiras, sendo que, nas mais desenvolvidas, Sul e Sudeste, houve altas frações de diabetes atribuível a obesidade, enquanto na região Norte, essa relação se deu com o sobrepeso. Esse comportamento pode estar relacionado com uma transição epidemiológica mais tardia nas regiões menos favorecidas do país. ^339^

•Na linha de base do ELSA-Brasil, a análise de 14.912 funcionários públicos brasileiros mostrou prevalência maior de diabetes entre pessoas com IMC de 25-29,9 kg/m^2^ (18,9%; IC 95% 18,0 - 19,9%) e acima de 30 kg/m^2^ (32,1%; IC 95% 30,6 - 33,6%) em comparação com aquelas com IMC ≤ 24,9 kg/m^2^ (11,7; IC 95% 10,9 - 12,6%). ^340^

•Moreira *et al* . avaliaram características sociodemográficas, comportamentais e de hábitos alimentares de 867 adultos de mais de 45 anos de João Pessoa, Paraíba, em pesquisa transversal de base populacional (VIGITEL, 2014) ^297^ e suas associações com a presença de hipertensão arterial sistêmica e diabetes. Em análise ajustada, a prevalência de diabetes foi maior em mulheres com menor nível educacional (0-4 anos de educação, PR 3,3; IC 95% 1,4 - 7,5) e que não consumiam feijões (PR 1,6; IC 95% 1,0 - 2,8). Em homens, as faixas etárias de 55-64 anos (PR 5,1; IC 95% 1,9 - 13,4) e ≥ 65 anos (PR 4,0; IC 95% 1,4 - 10,9) e estado civil ‘casado’ (PR 17,7; IC 95% 2,0 - 153,0) associaram-se a maior prevalência de diabetes. ^297^

•Oliveira *et al* . avaliaram características sociodemográficas, comportamentais e de hábitos alimentares de 572.437 adultos com idade superior a 18 anos de capitais brasileiras e Distrito Federal em pesquisa transversal de base populacional realizada de 2006 a 2016 (VIGITEL, 2014) ^297^ e suas associações com a presença de diabetes autorrelatado. Indivíduos com diabetes relataram maior consumo de frutas e verduras [40,7% (IC 95% 39,7 - 41,8) em diabéticos e 34,0% (IC 95% 33,8 - 34,3) em controles, PR ajustada 1,05] e menor consumo de carne com gordura aparente [24,3% (IC 95% 23,3 - 25,2) *vs.* 32,3% (IC 95% 31,9 - 32,5) PR ajustada 0,95], de leite integral [44,5% (IC 95% 43,5 - 45,5) *vs.* 55,4% (IC 95% 55,1 - 55,7%), PR ajustada 0,87] e de refrigerantes e sucos adoçados [9,5% (IC 95% 8,5 - 10,5) *vs.* 25% (IC 95% 24,6 - 25,4), PR ajustada 0,57]. Indivíduos com diabetes reportaram menor uso abusivo de álcool [15,9% (IC 95% 14,7 - 17,1) *vs.* 26,8% (IC 95% 26,4 - 27,2), PR ajustada 0,86] e menos atividade física no lazer [24,0% (IC 95% 23,1 - 25,0) *vs.* 34,6% (IC 95% 34,3 - 34,9), PR ajustada 0,92] do que pessoas sem diabetes. ^341^

•Em modelo ajustado, a ingestão de pelo menos quatro porções de alimentos lácteos ao dia associou-se com menor ocorrência de diabetes [0,76 (IC 95% 0,59 - 0,97)] em 10.010 adultos de análise transversal do ELSA-Brasil. ^342^

•Considerando a atividade física durante o lazer, o ELSA-Brasil mostrou menor chance de ocorrência de diabetes em homens e mulheres ativos em relação aos inativos: 0,73 (IC 95% 0,61 - 0,87) e 0,83 (IC 95% 0,67 - 1,03), respectivamente. ^343^

•Werneck *et al* . em 2018 avaliaram os níveis e padrões de assistir televisão autorreferidos de brasileiros e sua associação com diabetes tipo 2 em 60.202 adultos a partir de dados da Pesquisa Nacional de Saúde de 2013. Assistir televisão por mais de 4 horas por dia aumentou a chance de desenvolver diabetes em homens (1,64; IC 95% 1,23 - 2,17) e mulheres (1,33; IC 95% 1,09 - 1,63) em comparação a menos de 2 horas por dia. ^344^

•Teló *et al* . mostraram, em estudo transversal com 37.854 adolescentes, maior chance de diabetes tipo 2 naqueles com obesidade (OR 1,59; IC 95% 1,20 - 2,11) e aumento da circunferência abdominal (OR 1,51; IC 95% 1,13 - 2,01), sem associação com inatividade física (< 60 min/dia). ^314^

## Pesquisa Futura

•Ainda há evidências insuficientes quanto à questão de se a prevenção do diabetes através de MEV também preveniria complicações cardiovasculares e microvasculares da doença.

•Estudos de incidência de diabetes tipo 1 e tipo 2 com representatividade nacional, buscando determinantes sociais e comportamentais são necessários.

•Considerando a abrangência do SUS e a possibilidade de atingir boa parte de pacientes com diabetes tipo 1 e 2, estudos focados na avaliação da eficácia e efetividade de cuidado a esses pacientes no Brasil são desejáveis.

•Considerando as várias publicações que mostram aumento de sobrepeso e obesidade na população brasileira de todas as faixas etárias, com preferência para classes sociais mais baixas, políticas públicas eficientes na prevenção da obesidade deveriam ser prioritárias na busca de redução de novos casos de diabetes e suas complicações. Nelas podem ser incluídas: 1. Taxação de alimentos com alto teor calórico; 2. Rotulagem obrigatória de produtos alimentícios; 3. Criação de programas de prevenção e tratamento da obesidade nas comunidades, resgatando pessoas predispostas ao diabetes através de ferramentas simples (questionários); 4. Capacitação de equipes multiprofissionais para que possam se envolver em programas de MEV para prevenção e tratamento do diabetes; 5. Integração de profissionais de educação física aos programas mencionados.

## 9. DISLIPIDEMIA

### CID-10 E78 (E78.0 – E78.9); CID-10-CM E78 (E78.0 – E78.9)

**Ver Tabelas 9-1 a 9-8 e Figuras 9-1 a 9-5**

**Table:** Abreviaturas Usadas no Capítulo 9

CT	Colesterol Total
DALYs	Anos de vida perdidos ajustados por incapacidade (do inglês, *Disability-Adjusted Life-Year* )
DCV	Doenças Cardiovasculares
ELSA-Brasil	Estudo Longitudinal de Saúde do Adulto - Brasil
ERICA	Estudo de Riscos Cardiovasculares em Adolescentes
GBD	Carga Global de Doenças (do inglês, *Global Burden of Disease* )
HDLc	Colesterol da Lipoproteína de Alta Densidade (do inglês, *High-Density Lipoprotein Cholesterol* )
IAM	Infarto Agudo do Miocárdio
IC	Intervalo de Confiança
II	Intervalo de Incerteza
LDLc	Colesterol da Lipoproteína de Baixa Densidade (do inglês, *Low-Density Lipoprotein Cholesterol* )
OR	Odds Ratio
PNAUM	Pesquisa Nacional sobre Acesso, Utilização e Promoção do Uso Racional de Medicamentos no Brasil
PNS	Pesquisa Nacional de Saúde
SDI	Índice Sociodemográfico (do inglês, *Sociodemographic Index* )
SUS	Sistema Único de Saúde
TG	Triglicerídeos
UF	Unidade Federativa
YLDs	Anos vividos com incapacidade (do inglês, *Years Lived with Disability* )
YLLs	Anos potenciais de vida perdidos (do inglês, *Years of Life Lost* )

**Tabela 9-1:** – Níveis médios de lipídios plasmáticos, prevalências de níveis *borderline* e altos, além de população estimada com níveis lipídicos anormais, de acordo com sexo e grupo etário. ERICA-Brasil, 2013-2014.

Lipídios	Médio		Borderline		Alto		População estimada com anormalidade
	mg/dl	IC 95%	%	IC 95%	%	IC 95%	
**Colesterol total**							
População geral	148,1	147,1-149,1	24,2	22,7-25,8	20,1	19,0-21,3	2.940.705
Homens	143,6	142,4-144,8	22,7	20,4-25,2	15,3	13,9-16,9	1.256.102
Mulheres	152,6	151,4-153,9	25,7	24,5-27,0	24,9	23,4-26,5	1.684.602
12-14 anos	149,4	148,0-150,7	25,8	24,3-27,4	20,7	19,1-22,5	937.793
15-17 anos	147,1	145,8-148,3	22,8	20,8-24,9	19,6	18,0-21,2	2.002.911
**LDLc**							
População geral	85,3	84,5-86,1	19,5	18,5-20,5	3,5	3,2-4,0	1.526.733
Homens	83,4	82,2-84,5	17,4	16,0-18,9	2,9	2,3-3,6	669.805
Mulheres	87,2	86,3-88,1	21,5	20,2-22,9	4,3	3,7-4,9	856.928
12-14 anos	86,2	85,1-87,3	20,6	19,0-22,4	3,7	3,1-4,4	467.877
15-17 anos	84,5	83,5-85,5	18,4	17,2-19,7	3,4	2,9-4,1	1058.856
**Triglicerídeos**							
População geral	77,8	76,5-79,2	12,0	11,0-13,0	7,8	7,1-8,6	1.312.329
Homens	76,4	74,7-78,1	10,9	9,8-12,2	7,6	6,5-8,8	610.449
Mulheres	79,3	77,8-80,7	13,0	11,8-14,2	8,1	7,3-9,0	701.880
12-14 anos	78,9	76,7-81,0	12,7	11,0-14,6	8,3	7,2-9,5	434.638
15-17 anos	76,9	75,8-78,1	11,3	10,2-12,4	7,4	6,6-8,4	877.690
**HDLc**	**Médio**		**Baixo**				
População geral	47,3	46,7-47,9	46,8	44,8-48,9	-	-	3.104.161
Homens	44,9	44,4-45,5	55,9	53,7-58,2	-	-	1.256.003
Mulheres	49,6	48,9-50,3	37,8	35,4-40,2	-	-	1.848.158
12-14 anos	47,4	46,7-48,1	45,0	42,3-47,8	-	-	819.980
15-17 anos	47,2	46,4-48,0	48,4	45,9-50,8	-	-	2.284.181

*Modificado de Faria Neto JR et al. ^349^ *a: alteração = níveis borderline + altos.* *b: estimativas populacionais foram obtidas do processamento de microdados do Censo Demográfico do IBGE 2000 e 2010.*

**Tabela 9-2:** – Prevalência de colesterol total ≥ 200 mg/dl de acordo com sexo, grupo etário, nível educacional, cor da pele e região do Brasil, PNS 2014-2015.

	Total			Homens			Mulheres		
	%	IC 95%	p	%	IC 95%	p	%	IC 95%	p
**Total**	**32,7**	**31,5 - 34,1**		**30,1**	**28,2 - 32,1**		**35,1**	**33,4 - 36,8**	**< 0,001**
Grupo etário (anos)									
18 - 29	17,9	15,7 - 20,4	< 0,001	13,9	11,2 - 17,4	< 0,001	21,9	18,7 - 25,5	< 0,001
30 - 44	31,0	28,7 - 33,4		34,9	31,2 - 38,8		27,6	24,9 - 30,5	
45 - 59	43,4	40,8 - 46,0		39,4	35,7 - 43,4		47,0	43,5 - 50,5	
≥ 60	41,9	39,1 - 44,8		33,5	29,5 - 37,9		48,4	44,7 - 52,2	
**Escolaridade (anos)**									
0 - 8	37,1	35,2 - 39,1	< 0,001	31,6	28,9 - 34,5	0,237	42,2	39,6 - 44,8	< 0,001
9 - 11	28,6	25,5 - 32,0		26,6	22,2 - 31,6		30,6	26,4 - 35,2	
≥ 12	30,4	28,4 - 32,5		30,0	26,9 - 33,3		30,8	28,3 - 33,4	
**Cor da pele**									
Branca	33,9	31,9 - 36,0	0,146	30,8	27,8 - 33,9	0,669	36,6	33,9 - 39,4	0,196
Negra	33,2	29,0 - 37,6		30,0	23,9 - 37,0		36,0	30,5 - 41,8	
Parda	31,5	29,8 - 33,3		29,5	26,9 - 32,4		33,4	31,1 - 35,7	
Outras	23,3	14,8 - 34,6		19,6	9,7 - 35,4		25,8	14,2 - 42,2	
**Região**									
Norte	32,5	30,4 - 34,6	0,195	31,0	27,9 - 34,3	0,376	33,9	31,2 - 36,7	0,291
Nordeste	34,0	32,3 - 35,8		30,2	27,7 - 33,0		37,4	35,1 - 39,8	
Sudeste	31,5	29,1 - 34,1		28,7	25,1 - 32,6		34,1	30,9 - 37,4	
Sul	34,7	31,7 - 37,8		33,4	28,9 - 38,3		35,8	32,0 - 39,8	
Centro-Oeste	31,7	28,7 - 34,8		30,1	25,7 - 34,9		33,0	29,1 - 37,2	

*Fonte: Malta et al. 2019 . ^348^*

**Tabela 9-3:** – Prevalência de níveis baixos de HDL-colesterol (< 40 mg/dl) de acordo com sexo, grupo etário, nível educacional, cor da pele e região do Brasil, PNS 2014-2015.

	Total			Homens			Mulheres		
	%	IC 95%	P	%	IC 95%	p	%	IC 95%	p
**Total**	**31,8**	**30,5 - 33,1**		**42,8**	**40,6 - 45,0**		**22,0**	**20,6 - 23,5**	**< 0,001**
Grupo etário (anos)									
18 - 29	29,1	26,2 - 32,2	0,070	39,7	34,9 - 44,7	0,159	18,7	15,9 - 21,9	0,046
30 - 44	31,8	29,4 - 34,2		41,8	37,9 - 45,7		23,0	20,4 - 25,9	
45 - 59	34,1	31,6 - 36,6		44,8	40,9 - 48,8		24,3	21,5 - 27,4	
≥ 60	32,4	29,8 - 35,2		46,5	42,1 - 51,1		21,5	18,7 - 24,6	
**Escolaridade (anos)**									
0 - 8	33,7	31,8 - 35,7	< 0,001	43,3	40,2 - 46,4	0,006	24,9	22,8 - 27,2	< 0,001
9 - 11	38,5	34,9 - 42,2		50,0	44,3 - 55,6		27,0	22,9 - 31,5	
≥ 12	27,8	25,9 - 29,9		39,6	36,2 - 43,2		18,1	16,1 - 20,3	
**Cor da pele**									
Branca	31,0	29,0 - 33,1	0,072	43,0	39,7 - 46,5	0,586	20,6	18,4 - 23,0	0,006
Negra	28,5	24,3 - 33,2		41,8	34,5 - 49,4		16,6	12,6 - 21,6	
Parda	33,5	31,7 - 35,4		43,0	40,0 - 46,1		24,8	22,8 - 27,0	
Outras	24,7	15,8 - 36,5		27,7	15,1 - 45,2		22,7	11,6 - 39,5	
**Região do país**									
Norte	36,6	34,4 - 38,8	< 0,001	47,2	43,7 - 50,7	0,036	26,7	24,2 - 29,4	< 0,001
Nordeste	34,8	33,0 - 36,6		44,3	41,4 - 47,2		26,4	24,3 - 28,6	
Sudeste	30,8	28,3 - 33,4		43,1	38,9 - 47,3		20,0	17,4 - 22,9	
Sul	26,1	23,3 - 29,0		36,3	31,6 - 41,2		16,8	14,1 - 20,0	
Centro-Oeste	34,3	31,1 - 37,6		45,0	39,8 - 50,3		24,7	21,2 - 28,6	

*Fonte: Malta et al. 2019 . ^348^*

**Tabela 9-4:** – Prevalência de níveis altos de LDL-colesterol (> 130 mg/dl) de acordo com sexo, grupo etário, nível educacional, cor da pele e região do Brasil, PNS 2014-2015.

	Total			Homens			Mulheres		
	%	IC 95%	p	%	IC 95%	p	%	IC 95%	p
**Total**	**18,6**	**17,5 - 19,7**		**17,1**	**15,6 - 18,8**		**19,9**	**18,5 - 21,3**	**0,012**
**Grupo etário (anos)**									
18 - 29	8,8	7,2 - 10,7	< 0,001	6,6	4,8 - 9,0	< 0,001	11,0	8,7 - 14,0	< 0,001
30 - 44	17,5	15,7 - 19,5		20,2	17,3 - 23,6		15,2	13,0 - 17,6	
45 - 59	25,6	23,3 - 27,9		23,2	20,0 - 26,7		27,7	24,7 - 30,9	
≥ 60	24,5	22,2 - 27,0		19,5	16,3 - 23,2		28,4	25,1 - 31,9	
**Escolaridade (anos)**									
0 - 8	21,5	20,0 - 23,2	< 0,001	17,8	15,7 - 20,1	0,525	24,9	22,8 - 27,2	< 0,001
9 - 11	16,8	14,3 - 19,7		15,2	11,8 - 19,3		18,5	15,0 - 22,6	
≥ 12	16,7	15,1 - 18,4		17,2	14,8 - 20,0		16,2	14,2 - 18,4	
**Cor da pele**									
Branca	20,1	18,5 - 21,9	0,009	18,8	16,4 - 21,4	0,131	21,3	19,1 - 23,8	0,095
Negra	16,6	13,6 - 20,2		15,2	10,9 - 20,8		17,9	13,9 - 22,7	
Parda	17,4	16,1 - 18,8		15,9	13,9 - 18,1		18,8	17,0 - 20,7	
Outras	10,1	6,0 - 16,6		8,6	3,6 - 19,1		11,2	5,7 - 20,7	
**Região do país**									
Norte	16,2	14,7 - 17,9	0,136	15,5	13,2 - 18,1	0,355	17,0	14,9 - 19,2	0,195
Nordeste	19,8	18,4 - 21,3		17,5	15,5 - 19,8		21,9	19,9 - 23,9	
Sudeste	17,9	16,0 - 19,9		16,1	13,4 - 19,3		19,4	16,8 - 22,2	
Sul	20,0	17,6 - 22,6		19,8	16,2 - 24,0		20,1	17,1 - 23,5	
Centro-Oeste	17,8	15,4 - 20,4		17,8	14,3 - 21,9		17,8	14,8 - 21,3	

*Fonte: Malta et al. 2019 . ^*348*^*

**Tabela 9-5:** – Número de mortes e taxas de mortalidade atribuídas a níveis altos de LDL-colesterol em 1990 e 2019 e variação percentual das taxas, no Brasil e unidades federativas.

Morte por LDLc alto e localização	1990		2019		Variação percentual (II 95%)
	Número (II 95%)	Taxa (II 95%)	Número (II 95%)	Taxa (II 95%)	
Acre	80.9 (63.6;101.6)	64.4 (46.8;87.3)	210.6 (165.1;270.7)	38.3 (28.2;51.5)	-40.5 (-46.4;-32.9)
Alagoas	936.8 (725.7;1212.7)	78.9 (58.7;106.1)	1690.1 (1304.7;2141.2)	53.4 (40.6;69.2)	-32.3 (-40.6;-21.2)
Amapá	47.3 (37.8;59.9)	58.5 (43.4;79.3)	172.7 (139.2;218.4)	36 (27.5;47.8)	-38.5 (-44.6;-31.8)
Amazonas	411.7 (325.7;521.4)	69.7 (51.1;94.9)	883.6 (663.9;1167.2)	32.7 (23.5;44.5)	-53.1 (-59;-46.4)
Bahia	4070.8 (3156.6;5194.6)	66.4 (49.6;87.6)	6786.7 (5077.9;8762.6)	41.3 (30.8;53.8)	-37.8 (-48;-25.8)
Brasil	68327.1 (55096.6;83767.8)	88.6 (67.8;114.8)	99375.3 (78038.6;126142.7)	43.1 (33.4;55.9)	-51.3 (-53.8;-48.6)
Ceará	2062.8 (1514.7;2733.1)	53.3 (38.8;71.8)	4448.1 (3298.5;6074.6)	45.1 (33.2;62)	-15.5 (-29.9;4.8)
Distrito Federal	411.5 (339.1;499.7)	107.1 (77.1;144.1)	791.7 (603.6;1004)	41.7 (29.3;57.5)	-61.1 (-66.1;-55.6)
Espírito Santo	1032.7 (822.4;1303.7)	87.8 (64.6;117.7)	1923.6 (1488.8;2494.9)	45.9 (34.8;60.9)	-47.7 (-53.9;-41)
Goiás	1506.3 (1190.3;1897.7)	87.5 (64.1;116.2)	2894.6 (2210.2;3652.8)	43.1 (32.6;55.7)	-50.7 (-59.5;-40.2)
Maranhão	1836.1 (1417.7;2352.5)	77.1 (57.8;102.6)	3879.6 (2906.2;5171.6)	59.8 (44.1;80.6)	-22.5 (-37;-3.5)
Mato Grosso	548.4 (436.1;671.6)	82.5 (62;107.9)	1170.8 (904.1;1482.8)	37.4 (27.8;49.1)	-54.7 (-60.1;-48.1)
Mato Grosso do Sul	681 (555.3;829.6)	91.6 (70;117.2)	1255.2 (983.2;1596.2)	44.5 (33.9;58)	-51.5 (-56.8;-45.5)
Minas Gerais	7931.1 (6430.8;9675)	98.8 (75.7;126.6)	9517.5 (7373.8;12338.1)	36 (27.7;46.8)	-63.6 (-67.7;-59.3)
Pará	1367.8 (1062.2;1781.8)	81.3 (59.4;112.2)	2690.8 (2065;3506.2)	39.7 (29.8;53)	-51.1 (-57.5;-43.6)
Paraíba	1382.7 (1045.4;1805.4)	64 (48;83.7)	2269.8 (1696.4;3012.5)	46.2 (34.9;60.3)	-27.9 (-37.2;-16.6)
Paraná	4091.5 (3306.9;5040.5)	104.8 (79;139.1)	5530.9 (4235;7223)	43.9 (33.1;58.3)	-58.1 (-62.3;-53.2)
Pernambuco	3428.3 (2704;4329.7)	86.5 (65.4;113.9)	5636 (4347.5;7062.3)	57.6 (44;73.1)	-33.4 (-40.4;-24.4)
Piauí	847.6 (658.5;1106.6)	73.5 (53.9;100.8)	1584.5 (1179.1;2110.5)	41 (30.9;54.7)	-44.2 (-51;-36.5)
Rio de Janeiro	9443.4 (7744.9;11437.7)	112.3 (87.4;141.7)	10806.8 (8418.7;13551.9)	49.1 (37.8;62.2)	-56.3 (-60.6;-51.7)
Rio Grande do Norte	916.4 (688.2;1212)	61.3 (45.6;81.8)	1644.4 (1223.6;2156.1)	41 (30.9;53.6)	-33.1 (-43.6;-20.3)
Rio Grande do Sul	5115.6 (4086.6;6345.7)	90.4 (68.2;117.4)	6199.1 (4632.3;8338.2)	40.5 (30;54.5)	-55.2 (-59.9;-50.7)
Rondônia	242 (196.1;293.9)	108.9 (78.7;147.6)	630.4 (483.6;801.3)	43.7 (32.4;57.5)	-59.9 (-65.6;-52.8)
Roraima	36.7 (30;44.4)	87.6 (64.5;118.2)	123.9 (98.8;152.6)	41 (30.3;54.8)	-53.1 (-57.7;-47.9)
Santa Catarina	1997.2 (1608.5;2492.3)	96.8 (72.5;128.5)	3061.3 (2351.4;3875.8)	40.6 (30.1;52.9)	-58.1 (-62.4;-53.1)
São Paulo	17219.5 (13928.7;21011.3)	104.1 (79.1;133.6)	21988.1 (16961;27558.7)	42.2 (31.9;53.6)	-59.5 (-63.6;-54.9)
Sergipe	450.3 (344;592.6)	72.8 (52.1;100.4)	935.9 (707.3;1229.3)	42.3 (31.4;56.2)	-41.9 (-51;-30)
Tocantins	230.7 (179.4;291)	80.5 (57.9;110.3)	648.6 (491.9;860.9)	47.3 (35.1;63.8)	-41.3 (-50.2;-30.1)

*Fonte: Dados derivados do estudo Global Burden of Disease 2019, Institute for Health Metrics and Evaluation, University of Washington. ^46^*

**Tabela 9-6:** – Taxas de mortalidade atribuídas a altos níveis de LDL-colesterol em 1990 e 2019 e variação percentual das taxas, estratificadas por sexo, no Brasil e unidades federativas.

Taxa de mortalidade atribuída a LDLc alto	Mulheres			Homens		
	1990	2019	Variação percentual (II 95%)	1990	2019	Variação percentual (II 95%)
Acre	49.4(35.1-67.2)	28.8(20.5-39.9)	-41.6(-49.4--31.2)	82.5(59.6-112.2)	49.9(37-66.7)	-39.5(-48--29.1)
Alagoas	67.9(48.6-92.7)	45(32.9-61)	-33.8(-44.2--19.6)	91.3(68.3-122)	63.5(46.7-82.4)	-30.4(-42.8--14.1)
Amapá	47.1(34.3-65.4)	27.1(19.5-37.3)	-42.5(-49.6--34.3)	71(53.5-95.4)	46(34.8-61.1)	-35.2(-42.9--26.7)
Amazonas	63.5(44.2-88.4)	25(17.2-35.2)	-60.6(-66.4--53.3)	75(55.5-100.1)	40.7(29.6-54.8)	-45.8(-54.5--35.8)
Bahia	57.9(41.7-79.8)	30.6(20.8-41.7)	-47.1(-58--32.6)	75.8(56.3-101)	54.7(39.5-72.8)	-27.9(-44.4--6.6)
Brasil	72.9(54.2-97.8)	33.8(25-45.1)	-53.7(-56.9--50.4)	105.7(82.4-133.6)	54.2(42.3-68.4)	-48.8(-52.1--44.9)
Ceará	45.4(31.7-62)	36.8(25.1-51.3)	-18.9(-36.8-7.7)	62.4(44.6-86.9)	54.9(38.8-76.8)	-12(-33.7-19)
Distrito Federal	89.4(63.8-121.1)	34.3(22.9-48.5)	-61.6(-68.4--54.8)	137.9(97.6-187.2)	51.7(36.6-70.3)	-62.5(-67.8--56)
Espírito Santo	76.2(54.7-104.6)	35.9(26.2-48.7)	-52.9(-59.3--45)	100.1(75.8-131.4)	57.7(43.9-76.4)	-42.4(-51.3--32.6)
Goiás	74(52.2-102.8)	34.5(24.7-47.2)	-53.4(-62.3--41.9)	102(75.4-134.7)	52.6(38.9-68.4)	-48.4(-60--32.7)
Maranhão	42.3(29.7-57.3)	41.8(30.2-58.5)	-1(-23.3-31.9)	134.1(95.5-185)	84.3(59.8-116.1)	-37.2(-51.1--18.1)
Mato Grosso	68.8(50.2-91.4)	30.3(21.6-41.1)	-56(-62.7--47.7)	93.8(70.5-124)	44.3(33.6-58.2)	-52.7(-60.3--42.7)
Mato Grosso do Sul	78.6(57.9-103.8)	34.8(24.6-47.5)	-55.7(-61.6--48.8)	102.9(79-131)	54.9(41.8-70.3)	-46.7(-54.6--37.6)
Minas Gerais	83.7(61.4-111.4)	28.9(20.8-39.5)	-65.4(-70.4--60.1)	114.8(89.2-144.7)	43.9(33.5-56.7)	-61.8(-67.5--55.4)
Pará	68.9(47.9-97.3)	29.6(20.9-41.1)	-57(-63.9--48.4)	93.6(68.8-127.3)	50.5(37.6-67.7)	-46(-55.7--33.7)
Paraíba	58.3(42-78.4)	38.1(27.7-51.7)	-34.6(-45.1--20.4)	70.9(52.5-93.2)	55.7(41.7-73)	-21.4(-37--1.5)
Paraná	92.9(67.9-126.1)	35.2(25.2-48.3)	-62.1(-67.2--56.8)	116.5(89.3-151.1)	53.9(39.8-70.7)	-53.7(-60.2--46.2)
Pernambuco	75.6(55.6-100.1)	45(32.8-59.3)	-40.4(-48.7--31.2)	99.1(76.5-127.5)	73.5(55.7-93.5)	-25.8(-37.2--12.3)
Piauí	55.8(39-78.6)	32.7(23.3-46.5)	-41.5(-50.7--30.6)	94.6(70.4-130.5)	50.6(38.7-66.1)	-46.5(-55--35.8)
Rio de Janeiro	88.4(66-117.8)	36.7(27.2-48.4)	-58.4(-63.5--52.5)	142(111.6-176.7)	64.9(50-83.1)	-54.3(-60.1--47.7)
Rio Grande do Norte	49.7(35.3-66.6)	31.1(21.9-41.4)	-37.3(-49.4--22.6)	75.1(55.3-100.5)	52.9(38.4-70.5)	-29.5(-45.8--8.5)
Rio Grande do Sul	76.3(55.9-102.2)	34(24.4-47.2)	-55.4(-60.8--49.6)	105.6(81.6-134.5)	47.5(35-62.7)	-55(-61--48.5)
Rondônia	103.6(71.7-145.3)	35.2(25.2-47.9)	-66.1(-71.6--59.5)	112.1(81.3-152.8)	52.1(37.9-68.5)	-53.5(-62.4--41.9)
Roraima	68.1(48.4-95.3)	31.9(22.5-43.8)	-53.1(-58.4--47.9)	103(76.2-135.5)	49.4(36.7-65.5)	-52.1(-58.1--44.6)
Santa Catarina	84(60.1-115.4)	32.7(22.9-45.3)	-61.1(-66.2--55.1)	110.1(84.9-143.3)	49.2(37.2-63.2)	-55.3(-61.6--48.3)
São Paulo	84.8(61.3-112.9)	32.9(23.6-44.3)	-61.2(-66.3--55.5)	125.3(96.9-159)	53.1(40.3-68.1)	-57.6(-63.1--51)
Sergipe	64.6(45.3-89.8)	35.9(25.6-50)	-44.5(-55.1--30.6)	82.4(58.7-115)	50(36.1-66.6)	-39.3(-52.3--21.8)
Tocantins	67(46.4-94.9)	33.2(23.9-45.5)	-50.5(-59.7--38.3)	93.5(66-129.9)	64.1(46.5-86.8)	-31.5(-44.9--13.7)

*Fonte: Dados derivados do estudo Global Burden of Disease 2019, Institute for Health Metrics and Evaluation, University of Washington. ^46^*

**Tabela 9-7:** – Números e taxas de mortes, DALYs, YLLs e YLDs atribuídas a níveis altos de LDL-colesterol em 1990 e 2019 e variação percentual das taxas, por grupo etário, no Brasil.

Mortes					
	1990		2019		Variação percentual (II 95%)
	Número (II 95%)	Taxa (II 95%)	Número (II 95%)	Taxa (II 95%)	
15-49 anos	11.169,2 (10.105,5-12.136,3)	14,6 (13,2-15,8)	10.693,4 (9.673,6-11.619,4)	9,3 (8,4-10,1)	-36,5 (-40,2 a -32,6)
50-69 anos	28.552,5 (23.642,8-33.735,4)	182 (150,7-215)	37.840,3 (31.377,7-43.975,3)	93,8 (77,8-109)	-48,5 (-51,8 a -45,1)
05-14 anos					
70+ anos	28.605,4 (18.724,9-41.561,4)	676,2 (442,7-982,5)	50.841,6 (32.880,6-75.444,8)	388,4 (251,2-576,4)	-42,6 (-47,5 a -38,6)
Padronizada por idade		88,6 (67,8-114,8)		43,1 (33,4-55,9)	-51,3 (-53,8 a -48,6)
Todas as idades	68.327,1 (55.096,6-83.767,8)	45,9 (37-56,3)	99.375,3 (78.038,6-126.142,7)	45,9 (36-58,2)	-0,1 (-7,4 a -6,7)
Abaixo de 5					
**DALYs**					
	**1990**		**2019**		**Variação percentual (II 95%)**
	**Número (II 95%)**	**Taxa (II 95%)**	**Número (II 95%)**	**Taxa (II 95%)**	
15-49 anos	547.499,7 (493.892-595.058,5)	714,3 (644,4-776,4)	529.727,6 (476.934,4-574.563,7)	458,7 (413-497,5)	-35,8 (-39,3 a -32)
50-69 anos	884.741,5 (742.881,3-1.036.354,8)	5.639,7 (4.735,5-6.606,2)	1.184.341,7 (997.186,1-1.365.623,1)	2.935,7 (2.471,8-3.385)	-47,9 (-51,1 a -44,7)
5-14 anos					
70+ anos	389.558,3 (259.395,2-568.128)	9.209,3 (6.132,2-13.430,7)	649.071,5 (432.523,4-944.262,9)	4.959,1 (3.304,6-7.214,4)	-46,2 (-50,3 a -42,5)
Padronizada por idade		1.940,1 (1.614,4-2.322,9)		981,3 (817,1-1.162,4)	-49,4 (-52 a -46,8)
Todas as idades	1.821.799,5 (1.548.456,3-2.139.062,9)	1.224 (1.040,4-1.437,2)	2.363.140,8 (1.985.655,3-2.781.317,9)	1.090,7 (916,5-1.283,7)	-10,9 (-16 a -6,1)
Abaixo de 5					
YLLs					
	**1990**		**2019**		**Variação percentual (II 95%)**
	**Número (II 95%)**	**Taxa (II 95%)**	**Número (II 95%)**	**Taxa (II 95%)**	
15-49 anos	527.862,7 (477.315,3-573.651,8)	688,7 (622,8-748,4)	498.313,6 (450.959,6-540.989,3)	431,5 (390,5-468,4)	-37,3 (-40,9 a -33,5)
50-69 anos	855.877 (719.469,5-1.001.509,1)	5.455,7 (4.586,2-6.384,1)	1.123.815,9 (947.867-1.297.638,5)	2.785,6 (2.349,5-3.216,5)	-48,9 (-52,2 a -45,7)
5-14 anos					
70+ anos	375.390,1 (246.447,5-550.544,6)	8.874,3 (5.826,1-13.015)	608.617,9 (406.217-887.792,4)	4.650 (3.103,6-6.783)	-47,6 (-51,8 a -44)
Padronizada por idade		1.874,3 (1.560,8-2.234,9)		925,9 (773,1-1.093,9)	-50,6 (-53,2 a -47,9)
Todas as idades	1.759.129,8 (1.501.507,3-2.056.575,1)	1.181,9 (1.008,8-1.381,8)	2.230.747,4 (1.880.846,6-2.617.317)	1.029,6 (868,1-1.208)	-12,9 (-18 a -8)
Abaixo de 5					
YLDs					
	**1990**		**2019**		**Variação percentual (II 95%)**
	**Número (II 95%)**	**Taxa (II 95%)**	**Número (II 95%)**	**Taxa (II 95%)**	
15-49 anos	19.637 (12.830,1-27.604,3)	25,6 (16,7-36)	31.414 (20.934,9-43.414)	27,2 (18,1-37,6)	6,2 (-0,5-13,9)
50-69 anos	28.864,5 (18.492,9-41.136,1)	184 (117,9-262,2)	60.525,8 (38.344,5-87.578,5)	150 (95-217,1)	-18,5 (-24,7 a -12,1)
5-14 anos					
70+ anos	14.168,2 (7.112,4-23.987,6)	334,9 (168,1-567,1)	40.453,6 (21.750,9-67.619,8)	309,1 (166,2-516,6)	-7,7 (-17,4-3)
Padronizada por idade		65,7 (42,2-92,3)		55,4 (36,3-77,7)	-15,8 (-20,6 a -11)
Todas as idades	62.669,7 (41.368,3-87.014,6)	42,1 (27,8-58,5)	132.393,4 (87.120,9-184.088,9)	61,1 (40,2-85)	45,1 (35,5-54,6)
Abaixo de 5					

*Fonte: Dados derivados do estudo Global Burden of Disease 2019, Institute for Health Metrics and Evaluation, University of Washington. ^46^*

**Tabela 9-8:** – Taxas de DALYs atribuídos a níveis altos de LDL-colesterol padronizadas por idade em 1990 e 2019 e variação percentual das taxas, estratificadas por sexo, no Brasil e unidades federativas.

Localização	Mulheres			Homens		
	1990	2019	Variação percentual (II 95%)	1990	2019	Variação percentual (II 95%)
Acre	933.9(730.9-1172.4)	563.1(440-713.7)	-39.7(-48.2--28.8)	1652.5(1305.3-2048.3)	1072.6(861.3-1321)	-35.1(-44.5--23.2)
Alagoas	1400.3(1097.3-1750.5)	983.1(768.6-1227.2)	-29.8(-41.7--14.9)	2166.9(1748.7-2660.8)	1601.9(1262.7-1987.3)	-26.1(-39.5--8.7)
Amapá	863.2(685.6-1082.8)	557.2(444.5-697.8)	-35.5(-43.4--27)	1506.7(1243.3-1837.2)	1055.6(864.5-1291.4)	-29.9(-38.6--20.4)
Amazonas	1084.7(838.7-1405.1)	471.7(360-607.7)	-56.5(-62.8--48.8)	1573.5(1265.9-1945.1)	927.4(722.3-1172.9)	-41.1(-50.9--30.3)
Bahia	1193.9(932.9-1513.8)	677.8(508.4-866.2)	-43.2(-55.6--27.8)	1814(1435.9-2248.1)	1320.7(1009.4-1689)	-27.2(-44--4.8)
Brasil	1425.8(1165.2-1745.1)	692.2(567-842.5)	-51.5(-54.8--47.9)	2496(2090.2-2980.2)	1310.6(1097.4-1543)	-47.5(-50.9--43.8)
Ceará	922(698.3-1185.5)	712.9(526.1-940)	-22.7(-41.2-4.2)	1404.6(1054.1-1827.7)	1263.1(956.6-1661.9)	-10.1(-32.7-23)
Distrito Federal	1520.2(1186.9-1929.7)	544.6(407.7-712.1)	-64.2(-70.5--57.9)	2657(2070.6-3340.4)	1013.4(795.3-1273.3)	-61.9(-67.4--54.6)
Espírito Santo	1382.6(1115.2-1751.2)	725.3(575.8-910.9)	-47.5(-54.9--39.1)	2181.2(1802.6-2663.3)	1347.5(1080.9-1677.9)	-38.2(-48--27.8)
Goiás	1441.1(1105-1866.5)	721.4(555.1-931.4)	-49.9(-60.2--36.6)	2336.7(1833.9-2936.5)	1313.5(1013.1-1659.3)	-43.8(-56.9--26.3)
Maranhão	971.1(720.4-1247.8)	884.4(670.7-1156.5)	-8.9(-30.8-26.9)	3147.1(2354.6-4071.7)	1880.2(1403.5-2520.1)	-40.3(-54.9--19.2)
Mato Grosso	1355.4(1081.9-1688.7)	614.4(480.1-776.8)	-54.7(-61.9--46.1)	2116.8(1663.5-2650)	1083.4(867.6-1325.6)	-48.8(-57.5--37)
Mato Grosso do Sul	1505.9(1226.7-1853.2)	714(558.4-902)	-52.6(-59.1--44.9)	2387.8(1980.9-2867.9)	1338.7(1082.1-1637.9)	-43.9(-52.7--33.9)
Minas Gerais	1549.5(1261.2-1917.9)	601.5(481.6-767.4)	-61.2(-66.7--55.1)	2620.9(2197.5-3117.1)	1075.8(870.3-1303.7)	-59(-65.2--52.1)
Pará	1237.1(959.3-1603.7)	612.2(470.3-777.2)	-50.5(-58.7--40.1)	1992.2(1570.9-2548.5)	1187.9(936-1495)	-40.4(-51.8--26.4)
Paraíba	1196.6(938.8-1467.9)	797.1(621.6-1009)	-33.4(-44.6--18.4)	1724.1(1365.9-2161)	1391.5(1089.9-1748.8)	-19.3(-35.7-1.7)
Paraná	1695.8(1366.5-2120.4)	686.8(532.4-876.2)	-59.5(-65.2--53.6)	2599.9(2167-3137.2)	1268.8(994.8-1596.1)	-51.2(-58--43.1)
Pernambuco	1508.5(1223.5-1864.7)	939(738.7-1166.5)	-37.8(-46.8--28.1)	2311.8(1933.4-2767.6)	1818.3(1446.6-2218.6)	-21.3(-33.9--7.1)
Piauí	1055.1(828.3-1348.6)	662.4(520.5-848)	-37.2(-47.3--25.5)	2056.7(1662.2-2564.9)	1231.8(999.6-1495.1)	-40.1(-49.5--28.7)
Rio de Janeiro	1784.1(1474.1-2152)	792.4(632.7-970.2)	-55.6(-61--49)	3442(2926.8-4060.3)	1622.7(1322-1965.5)	-52.9(-59.2--45.8)
Rio Grande do Norte	996.1(779.7-1245.5)	660.3(494.9-838)	-33.7(-46.7--17.5)	1673.9(1313.8-2102.4)	1309.4(983.6-1656.2)	-21.8(-40.2-1.6)
Rio Grande do Sul	1452(1181.6-1788.3)	656.1(514-845.5)	-54.8(-60.4--48.4)	2481(2041.4-2989.1)	1115.5(880.1-1384.5)	-55(-61.1--48.5)
Rondônia	1767.1(1344.4-2274.7)	702.6(551.9-888.8)	-60.2(-66.9--52.1)	2277.1(1790.4-2862.2)	1224.4(942.4-1536.4)	-46.2(-56.6--32.5)
Roraima	1199.6(947-1536.6)	554.7(430.9-695.3)	-53.8(-59.1--47.9)	2103.1(1689.7-2625.6)	1075.6(865.6-1312.8)	-48.9(-56--40.4)
Santa Catarina	1514.2(1206.4-1898.2)	609.7(474.3-774.7)	-59.7(-65.5--53.8)	2427.5(2005.9-2959.6)	1134.2(907.3-1399.6)	-53.3(-60--45.5)
São Paulo	1547.8(1237-1892.5)	662.1(527.8-827)	-57.2(-62.9--50.7)	2906.3(2421.4-3472.8)	1310.8(1075.3-1602.9)	-54.9(-60.8--47.8)
Sergipe	1149.9(884.9-1477)	745.5(574.6-978.8)	-35.2(-48--18.6)	1693.4(1339.3-2177.8)	1194.7(913.1-1516.8)	-29.4(-44.7--9.5)
Tocantins	1177(907.1-1521.7)	689.2(539.2-869)	-41.4(-52.3--26.8)	1904(1458.8-2435.4)	1415.8(1094.7-1824.6)	-25.6(-41.2--4.2)

*Fonte: Dados derivados do estudo Global Burden of Disease 2019, Institute for Health Metrics and Evaluation, University of Washington. ^46^*

**Figura 9-1:**
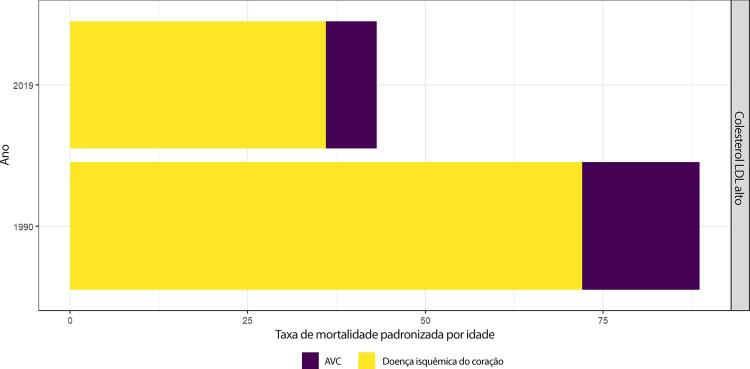
-Variação nas taxas de mortalidade padronizadas por idade por doença isquêmica do coração e acidente vascular cerebral atribuídos a níveis altos de LDL-colesterol, entre 1990 e 2019.

Fonte: Dados derivados do estudo Global Burden of Disease 2019, Institute for Health Metrics and Evaluation, University of Washington. ^46^

**Figura 9-2:**
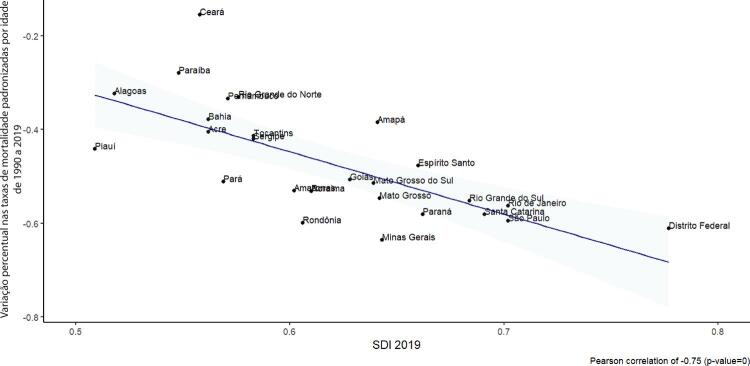
-Correlação entre a variação percentual nas taxas de mortalidade padronizadas por idade entre 1990 e 2019 e o índice sociodemográfico de 2019 (SDI 2019) de cada unidade federativa.

Fonte: Dados derivados do estudo Global Burden of Disease 2019, Institute for Health Metrics and Evaluation, University of Washington. ^46^

**Figura 9-3:**
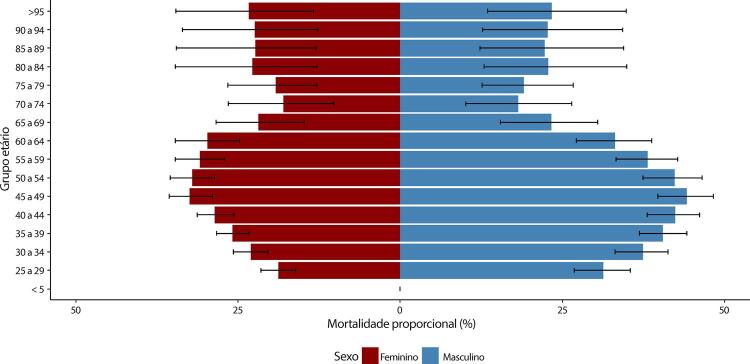
-Mortalidade proporcional atribuída a níveis altos de LDL-colesterol por grupo etário.

Fonte: Dados derivados do estudo Global Burden of Disease 2019, Institute for Health Metrics and Evaluation, University of Washington. ^46^

**Figura 9-4:**
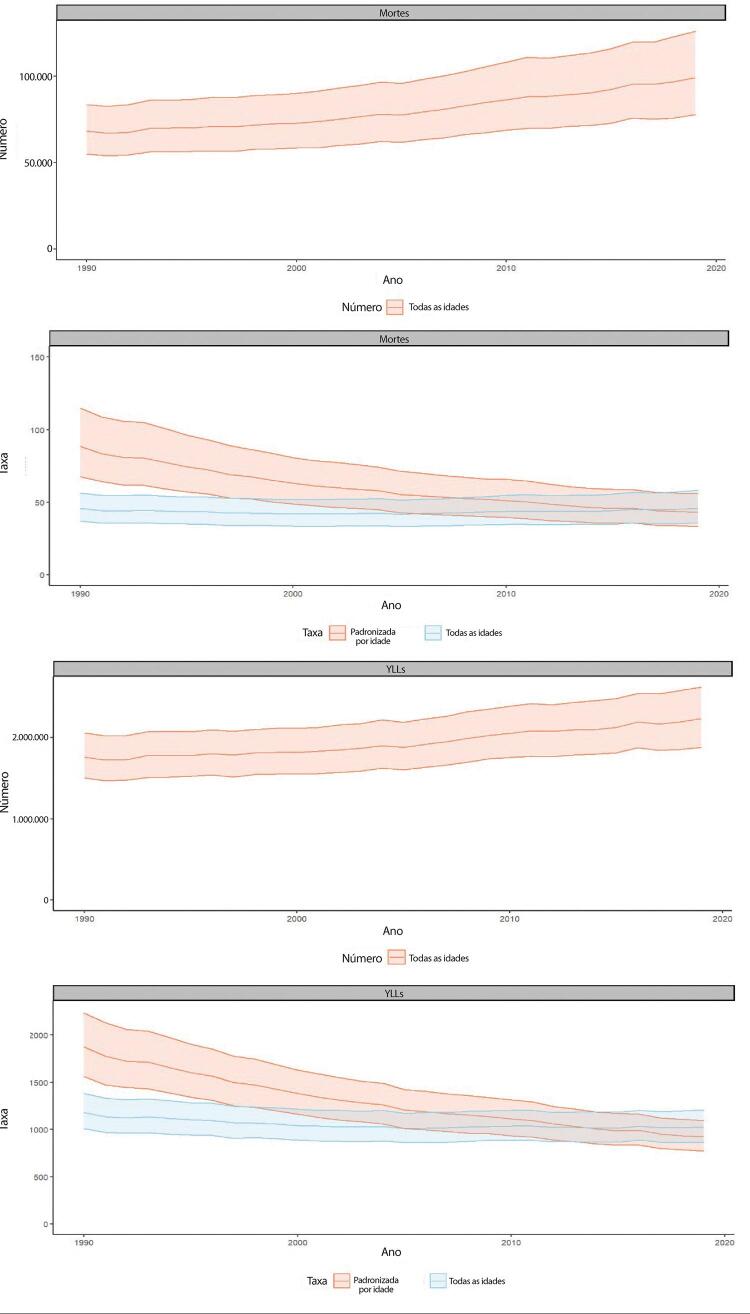
-Números de mortes e de YLLs e taxas de mortalidade e de YLLs atribuídos a níveis elevados de LDL-colesterol entre 1990 e 2019.

Fonte: Dados derivados do estudo Global Burden of Disease 2019, Institute for Health Metrics and Evaluation, University of Washington. ^46^

**Figura 9-5:**
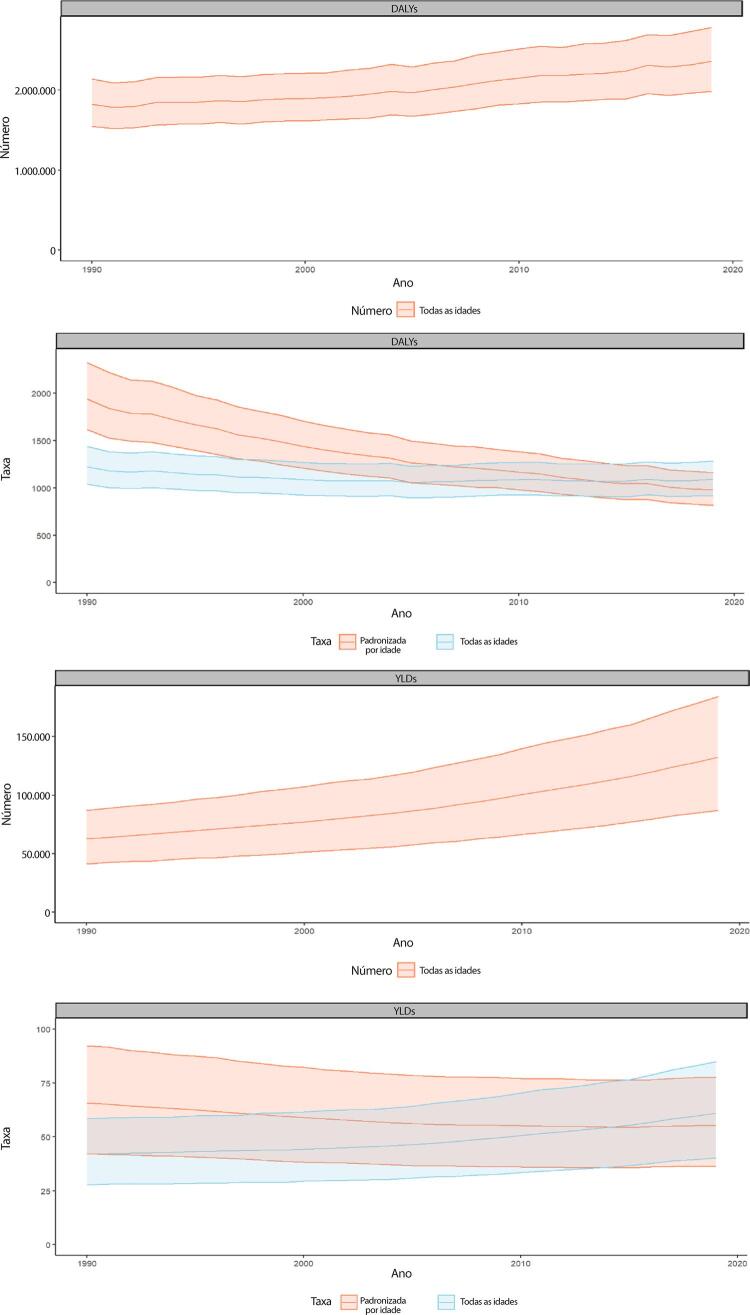
-Números e taxas de DALYs e YLDs atribuídos a níveis altos de LDL-colesterol entre 1990 e 2019.

Fonte: Dados derivados do estudo Global Burden of Disease 2019, Institute for Health Metrics and Evaluation, University of Washington. ^46^

### Introdução

•Define-se dislipidemia como níveis séricos anormais de lipídios, incluindo colesterol, suas frações e/ou TG. A dislipidemia é um conhecido fator de risco para as DCV, nas quais seu papel causal já está inequivocamente estabelecido. ^346^ Já foi demonstrado que o tratamento da dislipidemia, mesmo em prevenção primária, efetivamente reduz DCV. ^347^ Dados sobre os níveis médios de colesterol e a prevalência de dislipidemia foram obtidos primariamente para adultos na PNS 2015 e para adolescentes no Estudo ERICA. Além disso, estudos menores de prevalência (de base regional) foram usados, quando apropriado.

•Este capítulo apresenta dados sobre CT, LDLc, HDLc e TG. As definições de dislipidemia variam historicamente e conforme as posições das sociedades de cardiologia locais. Com o objetivo de classificação e a menos que especificado de outra maneira, neste capítulo, usaremos o termo dislipidemia para os seguintes valores: 1) em adultos: CT ≥ 200 mg/dl, LDLc ≥ 130 mg/dl, HDLc < 40 mg/dl, e TG ≥ 150 mg/dl; ^348^ 2) em crianças e adolescentes: CT ≥ 170 mg/dl, LDLc ≥ 130 mg/dl, HDLc < 45 mg/dl, e TG ≥ 130 mg/dl. ^349^

### Prevalência

#### Jovens

•No Estudo ERICA de abrangência nacional, Faria Neto *et al* . ^349^ avaliaram 38.069 estudantes (60% sexo feminino) com idade variando de 12 a 17 anos, nas capitais das 27 UF brasileiras, além de cinco conjuntos de municípios com mais de 100 mil habitantes, nas cinco regiões geográficas do país. ^349^ Os seguintes valores médios foram encontrados: CT, 148 mg/dl (IC 95%, 147-149 mg/dl); LDLc, 85 mg/dl (IC 95%, 84-86 mg/dl); HDLc, 47 mg/dl (IC 95%, 47-48 mg/dl); e TG, 78 mg/dl (IC 95%, 76-79 mg/dl). Com relação à prevalência de valores anormais, 20,1% (IC 95%, 19-21,3%) mostraram aumento de CT, 3,5% (IC 95%, 3,2-4%) de LDLc e 7,8% (IC 95%, 7,1-8,6%) de TG. A prevalência de HDLc baixo foi de 47% (IC 95%, 45-49%). A Tabela 9-1 mostra dados estratificados por idade e sexo *.*

•Para crianças de 6 a 12 anos de idade, os dados são escassos. Um estudo conduzido em Santa Catarina com 1.011 estudantes de 6 a 14 anos (52,4% meninas) relatou os seguintes níveis médios: CT, 172 (± 27) mg/dl em meninas e 170 (± 28) mg/dl em meninos; LDLc, 104 (± 24) mg/dl em meninas e 104 (± 27) mg/dl em meninos; HDLc, 49 (± 11) mg/dl em meninas e 49 (± 11) mg/dl em meninos; e TG, 80 (24-459) mg/dl em meninas e 77 (14-752) mg/dl em meninos. ^350^

•Um estudo conduzido em Vitória, no estado do Espírito Santo, com 511 crianças (idade, 6 a 9 anos; 46,77% sexo masculino) encontrou níveis altos de CT (em 32,7% delas), de LDLc (em 9,2%) e de TG (em 4,1%) e baixos de HDLc (em 27%). ^351^ Outro estudo conduzido na cidade de Salvador, avaliando 1.131 crianças (idade, 7 a 15 anos; 50,1% sexo masculino), identificou dislipidemia (CT ≥ 170 mg/dl e/ou TG ≥ 130 mg/dl) em 25,5% (IC 95%, 22,7 – 28,3) delas.

A dislipidemia estava associada com excesso de peso corporal (OR: 3,40; IC 95%, 2,07-5,58) e consumo moderado a elevado de alimentos de alto risco (OR: 1,49; IC 95%, 1,01-2,19). ^352^

#### Adultos

•De acordo com estudo de Malta *et al* . ^348^ usando dados da PNS 2014-2015, na população brasileira adulta, a prevalência de níveis altos de CT foi 32,7%, de níveis altos de LDLc, foi 18,6%, e de níveis baixos de HDLc, foi 31,8%. Esse estudo identificou os seguintes níveis médios: CT, 185 mg/dl; HDLc, 46 mg/dl; e LDLc, 105 mg/dl. Enquanto a prevalência de CT elevado foi maior nas mulheres, a prevalência de HDLc baixo foi maior nos homens. Detalhes sobre a prevalência de níveis elevados de CT e LDLc, assim como de níveis baixos de HDLc, estratificada por sexo, para diferentes grupos etários, nível educacional, cor da pele e região do país são apresentados nas Tabelas 9-2 a 9-4. Em geral, níveis educacionais mais altos foram relacionados a menor prevalência de níveis elevados de CT e LDLc, assim como de níveis baixos de HDLc. Os grupos etários mais avançados apresentaram maior prevalência de níveis elevados de CT e LDLc. Residir nas regiões Sul e Sudeste do Brasil esteve relacionado a menor prevalência de níveis baixos de HDLc. Uma relação significativa entre cor de pele autorrelatada e perfil lipídico foi menos clara, mas mulheres negras apresentaram menor prevalência de níveis baixos de HDLc. ^348^ Outros fatores associados com alterações relatadas no perfil lipídico da população brasileira incluem atividade física ^353^ e variações sazonais. ^354^

•O estudo ELSA-Brasil relatou as seguintes porcentagens em mulheres e homens, respectivamente: hipertrigliceridemia, 23,2% e 40,7%; níveis baixos de HDLc, 20,7% e 14,7%; e níveis altos de LDLc, 57,6% e 58,8%. Além disso, o estudo ELSA-Brasil relatou pequenas diferenças, cujo impacto clínico parece limitado, no perfil lipídico de acordo com a cor da pele. ^355^

•Em 2003, Martinez *et al* . avaliaram 81.262 indivíduos (51% sexo masculino; 44,7 ± 15,7 anos) de 13 grandes cidades brasileiras. ^356^ O nível médio de CT foi 199,0 ± 35,0 mg/dl e 13% da amostra apresentava CT acima de 240 mg/dl.

## Risco Atribuído

### Mortalidade

•Os números absolutos de mortes e as taxas de mortalidade nacionais e por UF, incluindo a variação percentual, podem ser vistos na Tabela 9-5. De acordo com as estimativas do Estudo GBD 2019, entre 1990 e 2019, a mortalidade cardiovascular atribuída a altos níveis de LDLc no Brasil aumentou em números absolutos de 68.327 (IC 95%, 55.097 – 83.768) a 99.375 (IC 95%, 78.039 – 126.143), mas a taxa padronizada por idade diminuiu em 51,3%, passando de 88,6 (IC 95%, 67,8 - 114,8) para 43,1 (IC 95%, 33,4 - 55,9) por 100.000, como resultado do envelhecimento da população. Entre os estados, Minas Gerais apresentou a maior redução na taxa de mortalidade (-63%) e o Ceará, a menor (-15%).

•A Tabela 9-6 apresenta as taxas de mortalidade atribuída a níveis elevados de LDLc estratificadas por sexo. Para mulheres, a taxa passou de 72,9 (54,2-97,8) em 1990 para 33,8 (25-45,1) em 2019, uma redução de 53,7% (-56,9 a -50,4) em nível nacional. A maior redução foi observada em Rondônia (-66,1%) e a menor, no Maranhão (-1%). Para homens, a variação percentual das taxas foi de -48,8% (-52,1 a -44,9), que passaram de 105,7 (82,4-133,6) em 1990 para 54,2 (42,3-68,4) em 2019, tendo o Distrito Federal apresentado a maior redução (-61,9%) e o Ceará, a menor (-10,1%).

•As causas específicas de morte atribuídas ao colesterol elevado seguiram a mesma tendência. As mortes por doença isquêmica do coração passaram de 57.020 (IC 95%, 46.252 – 68.541) para 83.759 (IC 95%, 65.742 – 101.543) e por doença cerebrovascular, de 11.306 (IC 95%, 5.270 – 21.619) para 15.615 (IC 95%, 5.522 – 32.805), com uma redução na taxa de mortalidade padronizada por idade para ambas. ^46^ A variação nas taxas de mortalidade padronizadas por idade por doenças isquêmica do coração e cerebrovascular atribuídas a LDLc elevado, entre 1990 e 2019, é representada na Figura 9-1 .

•O SDI é um índice composto que mede renda per capita, fertilidade, educação e desenvolvimento sociodemográfico. O SDI permite a comparação de estados e países de acordo com seu desenvolvimento. A redução na taxa de mortalidade padronizada por idade foi maior nas UF com SDI mais alto (Distrito Federal, São Paulo, Santa Catarina e Rio de Janeiro) e menor naquelas com SDI mais baixo, como as da região Nordeste ( Figura 9-2 ).

•A Tabela 9-7 apresenta os números absolutos de mortes e as taxas de mortalidade por grupo etário. Houve redução na taxa de mortalidade para todos os grupos etários. O grupo etário de 50-69 anos apresentou a maior redução na taxa de mortalidade (-48,5%). A Figura 9-3 ilustra a mortalidade proporcional atribuída a níveis altos de LDLc por grupo etário. Esse fator de risco parece ter maior impacto nos indivíduos com idade entre 40 e 64 anos.

## Anos Potenciais de Vida Perdidos

•O mesmo fenômeno observado para as taxas de mortalidade pode ser evidenciado pelos YLLs, cujos números absolutos variaram de 1.759.130 (IC 95%, 1.501.507 – 2.056.575) a 2.230.747 (IC 95%, 1.880.847 – 2.617.317). A taxa padronizada por idade variou de 1.874,3 (IC 95%, 1.561 – 2.235) a 926 (IC 95%, 773-1.094). ^12^ A Figura 9-4 apresenta o aumento no número de mortes e de YLLs atribuídos ao LDLc, assim como a redução nas taxas de ambos, padronizadas por idade, entre 1990 e 2019.

A ausência de redução na taxa de mortalidade, ao se remover a padronização por idade, é explicada pelo envelhecimento da população.

## Carga de Doença

•Além das complicações fatais das DCV atribuídas à dislipidemia, as complicações não fatais, como IAM e acidente vascular cerebral não fatais, podem ser parcialmente atribuídas à dislipidemia. O impacto dessas condições pode ser medido pelos YLDs e pelos DALYs, esse último sendo a soma dos YLLs e YLDs. Com relação aos YLDs, entre 1990 e 2019, houve aumento em seu número absoluto, que passou de 62.670 (IC 95%, 41.368 – 87.015) a 132.393 (IC 95%, 87.121 – 184.089), e diminuição na sua taxa (por 100.000), que passou de 65,7 (IC 95%, 42,2-92,3) a 55,4 (36,3-77,7), uma variação negativa de 15,8% (Tabela 9-7). ^46^

•Com relação aos DALYs, acompanhando a mesma tendência da mortalidade, houve um aumento em seu número absoluto, que passou de 1.821.799 (IC 95%, 1.548.456 – 2.139.063) para 2.363.141 (IC 95%, 1.985.655 – 2.781.318). Esse aumento foi acompanhado por uma redução na taxa de DALYs padronizada por idade, de 1.940,1 (IC 95%, 1.614,4 – 2.322,9) para 981,3 (IC 95%, 817,1 – 1.162,4), uma variação percentual de -49,4%. Essas variações estão ilustradas na Figura 9-5 .

•A Tabela 9-8 mostra as taxas de DALYs atribuídos aos altos níveis de LDLc padronizadas por idade em 1990 e 2019 e sua variação percentual no período, estratificadas por sexo, no Brasil e suas UF. Para as mulheres, a taxa nacional passou de 1.425,8 (1.165,2 – 1.745,1) em 1990 para 692,2 (567-842,5) em 2019, uma variação percentual de -51,5% (-54,8 a -47,9). A maior variação ocorreu no Distrito Federal, -64,2% (-70,5 a -57,9), e a menor, no Maranhão, -8,9% (-30,8 a -26,9). Para os homens, a taxa de DALYs passou de 2.496 (2.090,2 – 2.980,2) para 1.310,6 (1.097,4 – 1.543) no período, uma variação de -47,5% (-50,9 a -43,8) no Brasil. A maior melhora foi observada no Distrito Federal, -61,9% (-67,4 a -54,6), e a menor variação percentual, no Ceará, -10,1% (-32,7 a -23).

•A Tabela 9-7 mostra os números e as taxas de mortes, DALYs, YLLs e YLDs atribuídos a níveis altos de LDLc em 1990 e 2019, com suas respectivas variações percentuais no período, por grupo etário.

### Dislipidemia Familiar

•A prevalência de dislipidemia familiar foi avaliada no estudo ELSA-Brasil, diagnosticada por Dutch Lipid Clinic Network (DLCN)onde se documentou prevalência de 1 em 263 indivíduos. Essa condição foi mais prevalente nos indivíduos de cor de pele negra (1 em 156) e parda (1 em 204) do que branca (1 em 417). ^357^

•A despeito das controvérsias no uso de triagem em cascata para identificar parentes de indivíduos com dislipidemia familiar, um estudo brasileiro demonstrou que 59% dos familiares de indivíduos com mutações eram portadores das mesmas mutações, sugerindo uma alta prevalência de dislipidemia familiar no subgrupo selecionado. ^358^

### Conscientização e Uso de Estatina no Brasil

•Análise conduzida no estudo ELSA-Brasil, incluindo 15.096 adultos com idade de 35-74 anos, explorou a prevalência de níveis elevados de LDLc (de acordo com os critérios NCEP-ATP-III) e a proporção de participantes conhecedores de seu diagnóstico. ^340^ A frequência de participantes com níveis elevados de LDLc foi 45,5%, dos quais, apenas 58,1% conheciam seu diagnóstico. Dos participantes com níveis elevados de LDLc, 42,3% se tratavam com medicações hipolipemiantes e 58,3% alcançaram o alvo definido pelo painel NCEP-ATP-III.

•Em uma análise baseada na PNAUM entre 2014 e 2015, avaliou-se o uso de estatinas na atenção primária do SUS nas cinco regiões brasileiras. ^359^ Entre os 8.803 respondentes, a prevalência de uso de estatina foi 9,3%, sendo que 81,4% desses usuários relataram ter dislipidemia. Sinvastatina foi a mais usada (90,3%), seguida por atorvastatina (4,7%) e rosuvastatina (1,9%).

•Quanto à conscientização sobre dislipidemia familiar e seu tratamento, Santos *et al* . relataram resultados de um banco de dados com 70.000 indivíduos submetidos a avaliação de saúde rotineira e obrigatória patrocinada pelo empregador em um hospital privado de São Paulo. ^359^ Dos 70.000 indivíduos, 1.987 atendiam aos critérios estabelecidos para dislipidemia familiar (LDLc ≥ 190 mg/dl ou LDLc ≥ 160 mg/dl em uso de estatina). Uma amostra de 200 foi selecionada para completar o questionário. Desses 200 indivíduos, o médico assistente suspeitou de dislipidemia familiar em apenas 29 (14.5%), embora a maioria deles (97%) conhecesse seus altos níveis séricos de colesterol. Apenas 18% tinham a percepção de seu alto risco para DCV, 30% conheciam seus alvos de LDLc e 37% não utilizavam medicação hipolipemiante.

### Dislipidemia e Aterosclerose Subclínica

•Aterosclerose subclínica, incluindo marcadores como escore de cálcio coronariano e espessura médio-intimal da carótida, foi usada como substituto para aterosclerose e, portanto, sua associação com perfil lipídico anormal pode ser de interesse epidemiológico.

•Em um estudo com mais de 3.600 indivíduos, Generoso *et al* . ^19^ demonstraram que HDLc estava associado com cálcio na artéria coronária mesmo após ajuste para os fatores de risco cardiovascular tradicionais em uma população brasileira. Entretanto, essa associação não continuou significativa após ajuste para TG. ^360^ Esse estudo também avaliou as frações de HDLc e mostrou que elas não estavam associadas com calcificação na artéria coronária após ajuste para HDLc total. Além disso, o mesmo grupo demonstrou a associação entre HDLc e a espessura médio-intimal da carótida e ainda que tal associação é modificada pela presença de diabetes. ^361^

•Laurinavicius *et al* . ^362^ estudaram a associação entre níveis muito altos de HDLc e espessura médio-intimal da carótida. Tais níveis muito altos de HDLc podem caracterizar hiperalfalipoproteinemia, uma condição disfuncional do HDLc. A despeito de evidência anterior, esses autores não mostraram associação entre esse perfil e a espessura médio-intimal da carótida. ^362^

•Em uma análise das lipoproteínas ricas em TG no estudo ELSA-Brasil, Bittencourt *et al* . mostraram que essas partículas estão associadas com calcificação na artéria coronária mesmo após ajuste para fatores de risco significativos. ^363^

•Em um estudo com octogenários brasileiros, os autores descobriram que a associação entre LDLc e calcificação na artéria coronária enfraquece com a idade, enquanto a associação com HDLc não. ^364^

•Em conjunto, esses estudos demonstram a robusta associação entre perfil lipídico e aterosclerose subclínica, corroborando achados da associação entre dislipidemia e DCV.

### Pesquisa Futura

•Dados atuais sobre a epidemiologia da dislipidemia na população brasileira contemporânea são limitados. Estudos adicionais sobre a prevalência de dislipidemia na população geral, assim como em grupos específicos de alto risco, como aqueles de nível socioeconômico mais baixo, são necessários.

•A frequência de rastreio de colesterol no Brasil, de acordo com sexo e grupos etários, precisa ser investigada.

•Dados brasileiros locais sobre o impacto da dislipidemia no sistema de saúde, incluindo custos, ainda não foram avaliados.

## 10. OBESIDADE E SOBREPESO

### CID-10 E66

**Ver Tabelas 10-1 a 10-14 e Figuras 10-1 a 10-10**

**Table:** Abreviaturas utilizadas no Capítulo 10

AVC	Acidente Vascular Cerebral
DALYs	Anos de vida perdidos ajustados por incapacidade (do inglês, *Disability-Adjusted Life-Year* )
DCNT	Doenças Crônicas Não Transmissíveis
DCV	Doença Cardiovascular
ELSA-Brasil	Estudo Longitudinal de Saúde do Adulto - Brasil
ERICA	Estudo dos Riscos Cardiovasculares em Adolescentes
GBD	Global Burden of Disease
HR	Hazard Ratio
IC	Intervalo de Confiança
II	Intervalo de Incerteza
IMC	Índice de Massa Corporal
IMC_i	Índice de Massa Corporal Imputado
OMS	Organização Mundial da Saúde
OR	Odds Ratio
PNS	Pesquisa Nacional de Saúde
QALYs	Anos de Vida Ajustados pela Qualidade (do inglês, *Quality-Adjusted Life-Years* )
*RCEI*	Razão de Custo-Efetividade Incremental
RP	Razão de Prevalência
*RR*	Risco Relativo
SABE	Estudo de Saúde, Bem-Estar e Envelhecimento (do espanhol, *Salud, Bienestar y Envejecimiento* )
SDI	Índice Sociodemográfico (do inglês, *Sociodemographic Index* )
SUS	Sistema Único de Saúde
UF	Unidade Federativa
VIGITEL	Pesquisa de Vigilância de Fatores de Risco e Proteção para Doenças Crônicas por Inquérito Telefônico
YLDs	Anos vividos com incapacidade (do inglês, *Years Lived with Disability* )
YLLs	Anos potenciais de vida perdidos (do inglês, *Years of Life Lost* )

**Tabela 10-1:** – Prevalência de excesso de peso e obesidade na população total com idade a partir de 18 anos, por sexo e grupos etários, no Brasil, em 2019.*

Grupos de idade	Prevalência de excesso de peso e obesidade no total de pessoas de 18 anos ou mais de idade								
	Total			Sexo					
				Masculino			Feminino		
	Prevalência %	Intervalo de confiança de 95%		Prevalência %	Intervalo de confiança de 95%		Prevalência %	Intervalo de confiança de 95%	
		Limite inferior	Limite superior		Limite inferior	Limite superior		Limite inferior	Limite superior
**Excesso de peso**									
Total	60,3	58,3	62,1	57,5	54,8	60,2	62,6	59,1	66,0
18 a 24 anos	33,7	27,4	40,6	25,7	19,1	33,7	41,7	31,1	53,1
25 a 39 anos	57,6	53,1	62,1	58,3	52,3	64,1	57,0	50,0	63,8
40 a 59 anos	70,3	67,4	73,1	67,1	62,1	71,8	73,1	68,8	77,0
60 anos e mais	64,4	60,5	68,1	63,3	56,9	69,2	65,3	60,6	69,7
**Obesidade**									
Total	25,9	22,9	29,2	21,8	19,2	24,7	29,5	25,4	34,0
18 a 24 anos	10,7	7,7	14,7	7,9	4,8	12,8	13,5	8,8	20,4
25 a 39 anos	23,7	18,3	30,1	19,3	15,3	24,1	27,9	18,8	39,2
40 a 59 anos	34,4	29,7	39,4	30,2	24,8	36,3	38,0	32,3	44,0
60 anos e mais	24,8	20,9	29,1	21,2	15,6	28,1	27,5	23,0	32,5

*Fonte: IBGE, Diretoria de Pesquisas, Coordenação de Trabalho e Rendimento, Pesquisa Nacional de Saúde 2019.* * Fonte: IBGE, Diretoria de Pesquisas, Coordenação de Trabalho e Rendimento, Pesquisa Nacional de Saúde 2019. ^306^

**Tabela 10-2:** – Percentual de adultos com excesso de peso (IMC_i ≥ 25 kg/m2) com imputação, por sexo, segundo as capitais dos estados brasileiros e Distrito Federal.*

Capitais / DF	Total	IC95%	IC95%	Masculino	IC95%	IC95%	Feminino	IC95%	IC95%
		LimInf	LimSup		LimInf	LimSup		LimInf	LimSup
ARACAJU	53,6	50,5	56,7	56	50,8	61,2	51,7	48	55,3
BELEM	53,3	50,1	56,6	53,8	48,3	59,3	53	49,1	56,8
BELO HORIZONTE	52,5	49,7	55,3	57,1	52,7	61,4	48,6	45,1	52,2
BOA VISTA	54,3	49,5	59,2	60,1	52,7	67,5	49	43	55
CAMPO GRANDE	58	54,7	61,3	63,7	58,6	68,9	52,9	48,8	56,9
CUIABA	55,8	52,7	59	58,1	53	63,1	53,8	49,8	57,8
CURITIBA	53,7	50,6	56,9	59,5	54,6	64,4	48,8	44,8	52,8
FLORIANOPOLIS	53,6	50,3	56,8	58,9	53,9	63,8	48,7	44,6	52,8
FORTALEZA	55,6	52,4	58,7	57,7	52,5	63	53,8	50	57,6
GOIANIA	52,7	49,6	55,8	58,3	53,5	63,2	47,8	43,9	51,7
JOAO PESSOA	54,7	51,5	58	56,6	51,2	62,1	53,1	49,2	57
MACAPA	53,3	48,7	57,9	53	45,6	60,4	53,6	48	59,3
MACEIO	54,4	50,9	58	56,6	50,5	62,8	52,6	48,6	56,7
MANAUS	60,9	57,5	64,4	61,1	55,4	66,8	60,8	56,6	65
NATAL	56,6	53,3	59,8	60,8	55,6	66	52,9	48,9	56,9
PALMAS	49,9	46,2	53,6	56,8	50,8	62,7	43,7	39,2	48,1
PORTO ALEGRE	59,2	56	62,3	63	57,9	68,1	56	52	60
PORTO VELHO	56,6	52,9	60,3	62,2	56,6	67,7	50,6	46,1	55
RECIFE	59,5	56,5	62,5	60,4	55,5	65,2	58,8	55,1	62,5
RIO BRANCO	56,6	52,6	60,7	58	51	64,9	55,4	50,8	59,9
RIO DE JANEIRO	57,1	54	60,2	57,9	52,9	63	56,3	52,5	60,1
SALVADOR	51,8	48,6	54,9	47,2	41,9	52,5	55,5	51,8	59,2
SAO LUIS	50,3	46,9	53,7	57,6	51,9	63,2	44,4	40,5	48,3
SAO PAULO	55,8	53	58,6	56,6	52	61,2	55,1	51,6	58,6
TERESINA	52,7	49,5	55,9	56,3	51,1	61,6	49,7	45,8	53,5
VITORIA	49,1	45,8	52,3	50,6	45,3	55,8	47,8	43,8	51,7
DISTRITO FEDERAL	55	51,2	58,9	55,8	49,2	62,5	54,3	50,1	58,6

(*) Inquéritos 2006 a 2019, com pesos calculados pelo Método de Ponderação Rake. Consulta feita em:06/03/2021. Fonte: VIGITEL 2019. ^276^

**Tabela 10-3:** – Percentual de adultos com obesidade (IMC_i ≥ 30 kg/m^2^ ) com imputação, por sexo, segundo as capitais dos estados brasileiros e Distrito Federal.*

Capitais / DF	Total	IC95%	IC95%	Masculino	IC95%	IC95%	Feminino	IC95%	IC95%
		LimInf	LimSup		LimInf	LimSup		LimInf	LimSup
ARACAJU	20,6	18,1	23	18,7	14,7	22,6	22,1	19	25,2
BELEM	19,6	17,1	22,1	20,1	15,9	24,3	19,1	16,1	22,1
BELO HORIZONTE	19,9	17,7	22,2	20,7	17	24,4	19,2	16,5	22
BOA VISTA	21,2	16,8	25,5	24,6	17,1	32,2	17,9	13,5	22,3
CAMPO GRANDE	22,5	19,8	25,1	23	18,8	27,2	22	18,7	25,4
CUIABA	22,5	19,9	25	21,9	17,9	25,9	23	19,6	26,3
CURITIBA	19,4	17	21,8	21,1	17,2	25	17,9	14,9	20,9
FLORIANOPOLIS	17,8	15,5	20,1	18,8	15,1	22,5	16,8	14	19,7
FORTALEZA	19,9	17,5	22,4	18,9	15,1	22,6	20,9	17,6	24,1
GOIANIA	19,5	17,1	21,8	20,6	16,7	24,4	18,6	15,7	21,4
JOAO PESSOA	20,4	17,6	23,2	18,6	14	23,2	21,8	18,4	25,2
MACAPA	22,9	19	26,7	20,4	14,8	26	25,2	19,9	30,4
MACEIO	20	17,3	22,7	17,5	13,2	21,8	22	18,6	25,5
MANAUS	23,4	20,3	26,5	21	16,4	25,6	25,7	21,6	29,7
NATAL	22,5	19,7	25,4	24,3	19,5	29,1	21	17,8	24,2
PALMAS	15,4	12,8	18	16,6	12,2	21	14,3	11,3	17,3
PORTO ALEGRE	21,6	19	24,3	23,2	18,7	27,7	20,3	17,2	23,4
PORTO VELHO	19,9	16,8	23	21,6	16,5	26,7	18	14,7	21,4
RECIFE	21,7	19,2	24,3	19,7	15,8	23,5	23,4	20	26,8
RIO BRANCO	23,3	19,8	26,8	23,3	17,6	28,9	23,4	19	27,7
RIO DE JANEIRO	21,7	19,2	24,2	20,1	16,1	24,1	23,1	19,9	26,3
SALVADOR	18,1	15,8	20,4	15,5	11,7	19,3	20,3	17,5	23
SAO LUIS	17,2	14,2	20,1	18,8	13,4	24,2	15,8	12,8	18,8
SAO PAULO	19,9	17,7	22	18,5	15,1	21,8	21,1	18,3	23,9
TERESINA	17,6	15,3	19,9	17,1	13,5	20,6	18	15,1	21
VITORIA	17,6	15,3	19,9	16	12,3	19,6	19,1	16,1	22
DISTRITO FEDERAL	19,6	16,3	22,8	18,6	13,1	24	20,4	16,5	24,3

(*) Inquéritos 2006 a 2019, com pesos calculados pelo Método de Ponderação Rake. Consulta feita em:06/03/2021. Fonte: VIGITEL 2019. ^276^

**Tabela 10-4:** – Número de mortes, taxas de mortalidade padronizadas por idade para todas as causas atribuídas a IMC elevado, por 100 mil habitantes, em 1990 e 2019, e variação percentual das taxas no período, no Brasil e unidades federativas.

Causa de morte e Local	1990		2019		Variação percentual (II 95%)
	Número (II 95%)	Taxa (II 95%)	Número (II 95%)	Taxa (II 95%)	
**IMC elevado**					
Acre	80,1 (40,3;128,7)	50,9 (23,5;84,4)	443,6 (305,1;590,6)	75,3 (50,7;102,5)	47,9 (11,7;140,2)
Alagoas	926 (477;1462,1)	69,8 (35,1;111,7)	3244,2 (2234,3;4349,1)	101,1 (69,1;137,2)	44,9 (9;123,8)
Amapá	53,2 (31,5;78,4)	54,7 (30,3;83,8)	365,3 (259,2;478,3)	71,5 (49,3;96,1)	30,6 (6,2;77,4)
Amazonas	525,2 (312,4;765,4)	70,6 (40,5;106,3)	2009,3 (1400,6;2636,9)	71,2 (49;94,7)	0,9 (-17,4;35,7)
Bahia	4395,5 (2318,1;6785,9)	65,3 (33,6;102,3)	13225,3 (8827,3;18489,3)	81,1 (54,1;113,1)	24,1 (-5,4;86,8)
Brasil	74.266,2 (43.491,7;110.056,9)	84,4 (48,1;127,9)	177.939,7 (124.637,7;237.783)	76,2 (52,9;102,1)	-9,7 (-23,1;16,2)
Ceará	1762,6 (831,5;2903,1)	43,8 (20,4;72,5)	7194,6 (4786,1;10036,6)	72,5 (48,2;101,2)	65,4 (19,9;172,1)
Distrito Federal	657,1 (439;908,9)	121,9 (75,5;176,5)	1751,1 (1220,7;2263)	80,6 (54,6;107,6)	-33,8 (-45,2;-14,6)
Espírito Santo	1089 (608,6;1642,8)	75,8 (40,9;117,6)	3588,6 (2472,9;4828,9)	83,7 (57,3;112,7)	10,4 (-13;61,5)
Goiás	1610,1 (830,7;2574,7)	76,8 (38,1;126,3)	5058,6 (3482;6954,6)	73,9 (50,3;101,7)	-3,8 (-29,4;49,2)
Maranhão	1245,2 (489,7;2212,8)	48,2 (18,5;87,4)	5846,2 (3709,8;8423,6)	89 (56;128,6)	84,8 (25,5;250,7)
Mato Grosso	511,1 (276,4;794,5)	63,8 (32,6;101,1)	2471,7 (1734,2;3272,6)	76,8 (52,7;102,4)	20,5 (-9,1;85,8)
Mato Grosso do Sul	746,8 (436,4;1106,5)	83,3 (46,1;128,2)	2223,5 (1521,9;2976,5)	77 (52;103,9)	-7,6 (-26;24,5)
Minas Gerais	7763 (4227,8;11827,3)	79,5 (41,8;123,4)	16660,8 (11377,3;22486,1)	62,7 (42,8;84,8)	-21,1 (-36,5;13,9)
Pará	1320,3 (711,4;2041,6)	65,2 (33,7;103,8)	5128,9 (3498,2;6897,9)	74 (49,8;100,6)	13,5 (-12,8;70,3)
Paraíba	1273,5 (638,4;2048,7)	56,6 (28,1;91,7)	3974,9 (2740,4;5388,3)	82 (56,8;111,2)	45 (9,4;129,3)
Paraná	4468,1 (2563,7;6646,6)	94,2 (52,1;145)	10015,1 (6653;13717,2)	77,1 (50,8;106,2)	-18,2 (-31,9;9,1)
Pernambuco	3345,4 (1772,7;5178,4)	75,4 (38,8;118,8)	9471,3 (6571,7;12641,6)	95,3 (65,8;128,2)	26,3 (0,7;83,6)
Piauí	669,5 (287,7;1133,6)	49,6 (20,4;86,2)	2676,8 (1799,4;3642,1)	70,1 (47,1;94,8)	41,3 (3,1;159,1)
Rio de Janeiro	11.589,1 (7.176,8;16.646,3)	120,7 (72,9;175,6)	19.470,2 (13.465,9;26.105,1)	87,3 (60,2;117,2)	-27,7 (-39,7;-6,8)
Rio Grande do Norte	835,1 (404,9;1341)	53,2 (25,5;85,9)	2990,3 (1997;4137,2)	75,7 (50,4;104,7)	42,2 (4,4;128,1)
Rio Grande do Sul	6158,4 (3741,6;8880,7)	96,3 (56,6;142,5)	11470,3 (7637,2;15714,9)	73,8 (49,1;101,1)	-23,3 (-34,4;-4,2)
Rondônia	306,4 (175,6;451,6)	92,2 (48,4;144,4)	1256,6 (872,4;1680,6)	84,2 (57,6;113,1)	-8,7 (-29,9;33,9)
Roraima	47,4 (27,1;70,9)	84,5 (44,7;131,4)	305,1 (221;394,8)	91 (64,2;119,8)	7,8 (-14,7;58,5)
Santa Catarina	2262,4 (1388,2;3300,1)	93,3 (55,9;139,1)	5617,9 (3838,4;7494)	72,1 (49;96,8)	-22,7 (-35,5;-1,7)
São Paulo	19.864,6 (12.062,3;28.733,9)	102,9 (60,7;151,2)	38.343,9 (26.476,1;51.560,4)	72,4 (49,6;97,8)	-29,6 (-41;-9,3)
Sergipe	547,1 (307,2;815,2)	73,4 (39,6;112,1)	1885,1 (1288,8;2533,3)	84,4 (57,5;114,3)	14,9 (-11,8;65,8)
Tocantins	214 (97,3;356,9)	56,9 (24,3;99,6)	1250,5 (836,2;1702,4)	89,4 (59,9;122,4)	57,1 (11,6;185)

*Fonte: Dados derivados do estudo Global Burden of Disease 2019, Institute for Health Metrics and Evaluation, University of Washington. ^46^*

**Tabela 10-5:** – Taxas de mortalidade padronizadas por idade para todas as causas atribuídas a IMC elevado, por 100 mil habitantes, em 1990 e 2019, por sexo, e variação percentual das taxas no período, no Brasil e unidades federativas.

Causa de morte e Local	Feminino			Masculino		
	1990 Taxa (II 95%)	2019 Taxa (II 95%)	Variação percentual (II 95%)	1990 Taxa (II 95%)	2019 Taxa (II 95%)	Variação percentual (II 95%)
**IMC elevado**						
Acre	52,3 (27,5;83,3)	69,6 (49;92,1)	33 (0,8;108,3)	50,7 (19,9;90,3)	81,5 (50,6;116,5)	60,9 (18;201,5)
Alagoas	75,5 (40,3;117,1)	101,7 (70,8;137,2)	34,7 (-0,2;108,7)	63,4 (27,3;109,8)	99,3 (60,9;145,1)	56,8 (12,2;174,2)
Amapá	54,4 (31,6;80,9)	67,1 (46,8;88)	23,3 (-2;68,1)	54,7 (27,1;88,3)	75,6 (48,2;106,2)	38,3 (7,6;107,4)
Amazonas	76,4 (45,2;113)	66,4 (45,8;87,1)	-13 (-30,5;17,1)	63,8 (32,7;102,1)	75,5 (49,5;105,1)	18,3 (-10;80,3)
Bahia	70,6 (39,7;108,4)	72 (47,6;101,6)	2 (-24,9;50,3)	59,1 (25,6;101,4)	91,4 (56,3;134,8)	54,7 (5,8;173)
Brasil	83,7 (50,6;122,1)	70,1 (50,5;91,3)	-16,3 (-28,8;6)	84,2 (43,8;131,9)	82,4 (54,2;114,6)	-2,1 (-19;35,5)
Ceará	45,5 (22,1;73,7)	69,9 (46,1;98,7)	53,6 (5,5;166,7)	41,8 (17;73,8)	74,2 (44,2;110,6)	77,4 (17,1;234,1)
Distrito Federal	116,2 (74,3;165,2)	71,8 (48,2;96,6)	-38,2 (-49,9;-20,6)	130,6 (75,3;197,1)	92 (60,1;126)	-29,5 (-45,3;-0,6)
Espírito Santo	77,1 (43,8;116,5)	76,1 (52,3;101,2)	-1,3 (-24,9;43,5)	74 (36,2;119,1)	91,5 (57,7;129,2)	23,7 (-8,1;95,6)
Goiás	80,3 (42,5;128,8)	72 (50,2;97,1)	-10,4 (-35,9;42,1)	73 (31,1;126,2)	75,5 (47,6;109,8)	3,3 (-28,9;78)
Maranhão	37,3 (15,6;65,4)	78 (50,1;113)	109 (39,4;294,2)	63 (21,9;121,2)	102,8 (58,2;158,4)	63,1 (4,4;230,1)
Mato Grosso	65,8 (35,4;102,9)	73 (50,5;96,4)	11,1 (-16,8;70,4)	62,2 (28,6;104)	80,4 (53;111,3)	29,2 (-7,9;117,6)
Mato Grosso do Sul	84,6 (49,5;124,6)	72,3 (49,8;97,8)	-14,5 (-32;16,7)	81,7 (42,3;129,2)	81,5 (52,1;114,8)	-0,3 (-22,7;49,6)
Minas Gerais	82,1 (47,2;122,4)	59,5 (41;79,5)	-27,6 (-42,6;1,4)	75,5 (33,6;124,5)	65,7 (41,5;94,2)	-13,1 (-34,7;47,3)
Pará	65,2 (35,5;102,6)	67,6 (46,6;89)	3,6 (-22,3;58,8)	64,3 (30,1;107,6)	80,3 (50,2;113,2)	24,9 (-9,8;103,8)
Paraíba	62,3 (32,2;97,1)	78,6 (55;105,9)	26,2 (-6,2;105,4)	50,5 (21,7;86,3)	85,3 (54;120,7)	69 (18,3;199,6)
Paraná	95,6 (55,4;142,8)	72,2 (49,2;97,3)	-24,5 (-38,5;2,3)	92,5 (46,2;146,6)	81,8 (49,4;117,3)	-11,6 (-29,7;27)
Pernambuco	79,8 (43,8;122)	87 (59,9;115,6)	9 (-16,1;59,4)	69,8 (31,5;115,5)	104,8 (66,6;145,7)	50,1 (12;143,7)
Piauí	49 (21,8;82)	70,4 (48,4;94,3)	43,8 (3,6;160,8)	50,3 (17,7;93,3)	68,7 (43,1;97,9)	36,5 (-6,1;178,4)
Rio de Janeiro	113,2 (71,2;160,1)	77,6 (55,3;101,3)	-31,5 (-43,8;-10)	128,5 (70,8;196,1)	98,7 (63,2;138,8)	-23,2 (-38,8;5,4)
Rio Grande do Norte	53,2 (26,7;84,4)	68,4 (45,7;94)	28,5 (-7,2;103,3)	53,5 (22,8;92,1)	84 (52,4;123,4)	57 (8,2;180,2)
Rio Grande do Sul	89,1 (55,1;129,2)	67,2 (46,7;90,5)	-24,6 (-37,2;-2,6)	103,7 (57,5;157,1)	80,3 (49,6;114,1)	-22,5 (-36,5;1,2)
Rondônia	105,7 (58,6;159,8)	82,4 (57,4;110,3)	-22 (-40,3;15,8)	81,8 (39,2;136,8)	85,9 (54,4;121,8)	5 (-26,4;73,5)
Roraima	86,5 (47,7;130,6)	92,5 (66,8;120,6)	6,9 (-15,6;59)	82,2 (39,5;133,8)	88,4 (57,3;121,5)	7,6 (-17,9;72,3)
Santa Catarina	93,6 (57,7;135,6)	66,9 (46;88,7)	-28,5 (-41,7;-6,6)	91,8 (49,8;143)	76,5 (47,8;107,1)	-16,7 (-33,4;15,5)
São Paulo	99,9 (62;144,6)	65,5 (45,9;86,5)	-34,4 (-46,3;-14,3)	104,1 (57,6;159,8)	79,6 (51,1;113,1)	-23,5 (-38,8;5,9)
Sergipe	80,5 (45,7;119,6)	81,4 (55,2;111,5)	1,2 (-23,9;46,9)	64 (29,9;105,5)	86,9 (53,4;123,4)	35,8 (-4,1;125,1)
Tocantins	58,7 (26,5;98,5)	81,1 (56,5;108,8)	38 (-4,1;153,9)	54,8 (20,9;100,6)	99,3 (60,2;146,9)	81,2 (22,4;268,4)

*Fonte: Dados derivados do estudo Global Burden of Disease 2019, Institute for Health Metrics and Evaluation, University of Washington. ^46^*

**Tabela 10-6:** – Número de mortes, taxas de mortalidade brutas e padronizadas por idade para todas as causas de morte atribuídas a IMC elevado, por grupo etário, no Brasil em 1990 e 2019, e variação percentual das taxas no período.

Grupo etário	1990		2019		Variação percentual (II 95%)
	Número (II 95%)	Taxa (II 95%)	Número (II 95%)	Taxa (II 95%)	
Abaixo de 5	20.3 (8;37.7)	0.1 (0;0.2)	5.8 (2.5;10.8)	0 (0;0.1)	-68.8 (-82.5;-39.7)
5-14 anos	3.9 (1.9;6.3)	0 (0;0)	3.2 (1.6;5.1)	0 (0;0)	-10.4 (-33.3;23.2)
15-49 anos	14678.5 (8877.8;20807.9)	19.2 (11.6;27.1)	19019.1 (14656.4;23409)	16.5 (12.7;20.3)	-14 (-27.5;14.5)
50-69 anos	36499.3 (22180.1;53036.6)	232.7 (141.4;338.1)	76441.7 (54807.4;99553.2)	189.5 (135.9;246.8)	-18.6 (-29.6;2.6)
70+ anos	23064.2 (11761.5;37147.7)	545.2 (278;878.2)	82469.9 (53033.1;116025.8)	630.1 (405.2;886.5)	15.6 (-5.5;59.7)
Padronizada por idade	74266.2 (43491.7;110056.9)	84.4 (48.1;127.9)	177939.7 (124637.7;237783)	76.2 (52.9;102.1)	-9.7 (-23.1;16.2)
Todas as idades	74266.2 (43491.7;110056.9)	49.9 (29.2;73.9)	177939.7 (124637.7;237783)	82.1 (57.5;109.7)	64.6 (42.2;107.4)

**Tabela 10-7:** – Número de mortes e taxas de mortalidade padronizadas por idade por doença cardiovascular atribuída a IMC elevado (por 100 mil), e variação percentual das taxas, no Brasil e suas unidades federativas, 1990 e 2019.

Causa de morte e Local	1990		2019		Variação percentual (II 95%)
	Número (II 95%)	Taxa (II 95%)	Número (II 95%)	Taxa (II 95%)	
**IMC elevado**					
Acre	54,3 (26,3;88,1)	32,9 (15,1;55,2)	240,7 (162;324,9)	39,4 (25,6;54,4)	19,7 (-9,2;92,6)
Alagoas	602,6 (298,9;970)	44,4 (21,4;72,2)	1771,2 (1198,6;2438,8)	54,5 (36,5;75,4)	22,7 (-7,3;89,6)
Amapá	35,8 (20,9;53,5)	35,1 (19,3;54,9)	197,1 (135,5;262,4)	36,8 (24,6;50,4)	4,7 (-14,7;42,6)
Amazonas	352,5 (205,6;520,2)	45,9 (25,7;70,8)	998,7 (674,7;1337,4)	34,4 (22,8;46,8)	-25 (-38,9;-0,1)
Bahia	2885,5 (1491,2;4533,3)	42,2 (21,1;67,7)	6924,3 (4434,5;9803,3)	42,3 (26,9;59,8)	0,1 (-24,1;51,2)
Brasil	52.646,7 (30.085,3;78.950,7)	58,5 (32,7;89,7)	98.506,9 (66.815,9;133.940,7)	41,8 (28,1;56,8)	-28,5 (-38,8;-8,6)
Ceará	1165,6 (535,6;1957)	28,6 (13;48,7)	4027,1 (2576,7;5720,1)	40,3 (25,6;57,4)	40,9 (2,3;132,4)
Distrito Federal	474,2 (311,9;658,5)	82,1 (49,6;120,5)	976,1 (663,5;1284,2)	43,3 (28;58,5)	-47,2 (-56,5;-31,5)
Espírito Santo	806,5 (446,2;1222,5)	54,8 (29;85,3)	2070,4 (1400,5;2823,1)	47,8 (32,1;65,4)	-12,9 (-31;26,8)
Goiás	1188 (602,6;1919,7)	54,8 (26,6;91,9)	2871,2 (1930,6;3989,4)	41 (27,3;57,6)	-25,2 (-45,4;15,5)
Maranhão	835,9 (316,7;1531,8)	31,4 (11,6;58,7)	3369,1 (2093,2;4945,6)	50,7 (31;75)	61,6 (9,6;208,8)
Mato Grosso	368,8 (196,9;581,3)	44,2 (22,1;72,3)	1348,7 (914,3;1828,4)	40,8 (26,9;56,1)	-7,9 (-30,1;42,4)
Mato Grosso do Sul	564 (324,8;844,1)	60,9 (33,8;94,8)	1326,7 (903,8;1798,1)	45,2 (30,2;61,7)	-25,7 (-40,2;0,7)
Minas Gerais	5533 (2941,6;8429,9)	55,5 (28,4;87,4)	8966,4 (6002,5;12276,5)	33,7 (22,5;46,3)	-39,2 (-51,4;-12,9)
Pará	947 (500,7;1486,5)	45,6 (23,1;73,8)	2780,4 (1825,6;3803,1)	39,2 (25,4;54,2)	-14 (-33,6;27,9)
Paraíba	798,3 (387,4;1308,8)	35,2 (17,2;58,2)	2099,6 (1385,9;2931,4)	43,5 (28,8;60,4)	23,5 (-7,9;96)
Paraná	3348,7 (1894,5;4987,2)	69,1 (37,4;105,8)	5507,9 (3564,2;7725,2)	42 (27;59)	-39,2 (-49,7;-18,6)
Pernambuco	2291,3 (1176;3613,6)	50,9 (25,4;81,3)	5351,1 (3592,7;7304,5)	53,1 (35,3;73,1)	4,4 (-16,8;51)
Piauí	475,8 (196,1;816,3)	34,5 (13,7;61)	1548,3 (1017,4;2156,1)	40,5 (26,6;56,2)	17,4 (-14,5;118,3)
Rio de Janeiro	8407 (5110,5;12228,9)	86 (51,1;126,6)	10.753,6 (7.228;14.568,8)	48 (32,2;65,1)	-44,1 (-53,2;-27,8)
Rio Grande do Norte	526,5 (245,4;861,5)	33,3 (15,5;55)	1521,9 (977,9;2172,1)	38,4 (24,7;54,6)	15,4 (-15,8;85,6)
Rio Grande do Sul	4455,3 (2668,3;6541,9)	68,1 (39,9;100,4)	6132,7 (3977,2;8434,1)	39,5 (25,7;54,5)	-41,9 (-50,4;-27,6)
Rondônia	221,7 (124,2;331,6)	63,7 (32,7;102,5)	696,4 (475,8;952,7)	45,3 (30,3;62,8)	-28,9 (-45,6;5,2)
Roraima	31,1 (17,3;47,7)	52,7 (26,9;83,6)	150,4 (104,8;199,1)	43,5 (29,1;59,3)	-17,4 (-34,7;24,4)
Santa Catarina	1628,2 (981,8;2402,5)	65,3 (38,5;100)	3055,7 (2029,3;4095,5)	38,6 (25,3;52,5)	-40,9 (-50,6;-23,7)
São Paulo	14.175,3 (8.410,5;20.750,5)	71,6 (40,9;107,2)	22.149,9 (15.099,6;29.984,3)	41,3 (27,9;56,3)	-42,3 (-51,9;-24,9)
Sergipe	320,9 (173,8;492,9)	41,9 (21,9;66,2)	956,8 (634,6;1320,9)	42,1 (27,9;58,6)	0,6 (-23,4;44,7)
Tocantins	152,9 (67,6;258,2)	38,9 (16,1;68,7)	714,7 (463,1;986,3)	50,3 (32,2;70,2)	29,4 (-7,6;134,9)

*Fonte: Dados derivados do estudo Global Burden of Disease 2019, Institute for Health Metrics and Evaluation, University of Washington. ^46^*

**Tabela 10-8:** – Taxas de mortalidade padronizadas por idade para doença cardiovascular atribuída a IMC elevado, por sexo, no Brasil e unidades federativas, em 1990 e 2019, e variação percentual das taxas no período.

Causa de morte e Local	Feminino			Masculino		
	1990 Taxa (II 95%)	2019 Taxa (II 95%)	Variação percentual (II 95%)	1990 Taxa (II 95%)	2019 Taxa (II 95%)	Variação percentual (II 95%)
**IMC elevado**						
Acre	32,4(17;52)	34,5(23,7;46,3)	6,6(-20;65,5)	34,2(13,3;60,8)	44,8(27,4;64,7)	31,1(-5,3;138,8)
Alagoas	44,9(23,6;70,8)	52,1(35,1;70,8)	16(-15,1;81,4)	43,8(18,5;76,5)	56,9(34,2;83)	30(-8,7;125,4)
Amapá	32,7(18,8;49,3)	31,8(21,4;42,2)	-2,8(-22,6;32,1)	37,6(18,6;60,8)	42(26,3;59,5)	11,8(-11,9;66,4)
Amazonas	47,2(27,3;70,1)	29,5(19,7;39,5)	-37,5(-50,1;-15,8)	44,1(22,4;71,6)	39,3(24,8;56)	-10,8(-31,6;35,7)
Bahia	43,7(23,9;68,7)	36(23,1;51,7)	-17,5(-40;22,8)	40,5(17,3;69,9)	49,6(29,8;74,6)	22,5(-16,1;111,6)
Brasil	54,7(32,2;81,1)	36,2(25,4;48,4)	-33,9(-43,7;-16,7)	62,1(32,4;98,6)	47,9(30,4;66,8)	-22,8(-35,9;6,2)
Ceará	27,7(13,2;45,7)	36,3(23,2;51,8)	31,3(-9;128,3)	29,6(11,8;52,6)	44,5(26,2;66,2)	50,2(-0,7;188,2)
Distrito Federal	74,7(46,2;108,6)	37(23,9;51,4)	-50,4(-60,4;-35,8)	92,7(51,8;141,2)	51,3(32,7;72,2)	-44,6(-57,4;-21,5)
Espírito Santo	52,8(29,2;80,6)	40,7(27,5;55,5)	-22,9(-40,7;9,6)	56,8(27,9;91,3)	55,5(35;79,3)	-2,3(-28;52,5)
Goiás	54,8(28,6;89)	37,5(25,4;51,6)	-31,6(-50,7;8,2)	54,7(23,3;93,9)	44,7(27,3;66,5)	-18,2(-44,3;42)
Maranhão	21,9(9,1;39,4)	41,5(26,1;60,2)	89,9(27,4;262,4)	44(14,8;86,5)	62,2(34,7;98,8)	41,4(-8,9;193,3)
Mato Grosso	42,4(22,4;67,5)	36(24,3;49)	-15,1(-36,9;28,3)	45,8(20,9;77,5)	45,3(28,9;64,1)	-1(-29,5;65,5)
Mato Grosso do Sul	58,7(33,7;87,7)	39,5(26,8;53,7)	-32,6(-46,3;-8,6)	62,7(32,3;99,6)	51,2(32,3;72,4)	-18,3(-36,6;21,6)
Minas Gerais	54,2(30,4;81,5)	30,1(20,6;41,2)	-44,5(-55,9;-21,8)	56,3(24,8;94,4)	37,5(23,3;53,8)	-33,3(-50,3;12,1)
Pará	43,3(23,1;69,4)	32,7(21,7;45)	-24,7(-42,7;16,7)	47,3(21,7;79,9)	45,9(28,1;65,8)	-3(-29,4;56,1)
Paraíba	36(18,3;57,7)	39,4(26,9;54,9)	9,4(-19,9;77,9)	34,6(14,5;59,6)	48,2(29,2;69,8)	39,5(-2,3;147,5)
Paraná	66,9(38,1;100,1)	37,1(24,9;51,3)	-44,5(-55,1;-25,5)	71(35,1;112,4)	47,2(28;69,5)	-33,6(-47,6;-4,3)
Pernambuco	50,8(27,5;78,8)	45(30,2;61,9)	-11,5(-31,4;29,2)	50,6(22,3;84,4)	62,9(39,3;89,1)	24,3(-7,4;100,1)
Piauí	31,7(13,8;54,2)	38,3(25,1;53,6)	20,9(-13,4;119,7)	37,7(13,1;70,7)	42,6(25,9;61,8)	13(-21,6;128,8)
Rio de Janeiro	76,5(47,9;110)	40,1(27,7;53,3)	-47,6(-57,2;-31,5)	96,8(53;148,2)	57,7(36,1;81,9)	-40,4(-52,8;-18,7)
Rio Grande do Norte	30,5(14,8;49,7)	31,8(20,4;45,2)	4,2(-24,7;66,3)	36,5(15,5;63,2)	46,1(27,8;68,6)	26,5(-13,5;124,4)
Rio Grande do Sul	60,5(36,6;87,9)	34,4(23,3;47,1)	-43,2(-52,8;-26,6)	76,3(41,7;116,5)	45(27;64,8)	-41,1(-51,6;-22,1)
Rondônia	68,8(36,8;105,7)	41,2(28,1;56,5)	-40,1(-54,7;-11,5)	59,6(28,2;99,8)	49,3(31;71,5)	-17,3(-42;35,9)
Roraima	48,6(26,2;74,9)	38,8(26,2;52,7)	-20,1(-36,9;20)	55,6(26,4;92,2)	47,1(29,3;65,9)	-15,3(-35,3;34,7)
Santa Catarina	62,1(37,4;91,4)	33,8(22,3;47)	-45,6(-56;-27,5)	68,2(36,7;107,4)	43,4(26,7;61,3)	-36,5(-49,7;-12,6)
São Paulo	65,5(39,5;96,3)	35,3(24,2;47,8)	-46,1(-56,1;-29,6)	77,3(41,6;119,3)	47,9(30,7;68,1)	-38(-50,2;-14,6)
Sergipe	42,8(23,7;65,1)	39,3(25,9;55,1)	-8,1(-32,2;34)	40,3(18,6;68)	45,2(26,7;66,2)	12(-21,5;82,9)
Tocantins	37,9(16,6;65,6)	42,1(28,1;58,2)	11,2(-22,3;105,1)	39,5(14,7;73,3)	59,5(35,5;88)	50,5(1,6;202)

*Fonte: Dados derivados do estudo Global Burden of Disease 2019, Institute for Health Metrics and Evaluation, University of Washington. ^46^*

**Tabela 10-9:** – Número de mortes e taxas de mortalidade brutas e padronizadas por idade por doença cardiovascular atribuída a IMC elevado, por grupo etário, no Brasil, em 1990 e 2019, e variação percentual das taxas no período.

IMC elevado	1990 Número (II 95%)	1990 Taxa (II 95%)	2019 Número (II 95%)	2019 Taxa (II 95%)	Variação percentual (II 95%)
15-49 anos	11.876,9 (7.042;16.974,1)	15,5 (9,2;22,1)	13.729,8 (10.494,6;16.942)	11,9 (9,1;14,7)	-23,3 (-35,3;2,7)
50-69 anos	25.692,7 (15.086,6;37.700,4)	163,8 (96,2;240,3)	43.871,8 (30.231,8;58.205,9)	108,7 (74,9;144,3)	-33,6 (-42,5;-16,5)
5-14 anos					
70+ anos	15.077,1 (7.374,5;24.799,9)	356,4 (174,3;586,3)	40.905,3 (25.123,3;59.857,6)	312,5 (191,9;457,3)	-12,3 (-28,3;20,2)
Padronizada por idade		58,5 (32,7;89,7)		41,8 (28,1;56,8)	-28,5 (-38,8;-8,6)
Todas as idades	52.646,7 (30.085,3;78.950,7)	35,4 (20,2;53)	98.506,9 (66.815,9;133.940,7)	45,5 (30,8;61,8)	28,5 (11,1;63,2)
Abaixo de 5					

*Fonte: Dados derivados do estudo Global Burden of Disease 2019, Institute for Health Metrics and Evaluation, University of Washington. ^46^*

**Tabela 10-10:** – Números de DALYs e taxas de DALYs padronizadas por idade por todas as causas de morte atribuídas a IMC elevado (por 100 mil habitantes), em 1990 e 2019, e variação percentual das taxas no período, no Brasil e unidades federativas.

Causa de morte e Local	1990		2019		Variação percentual (II 95%)
	Número (II 95%)	Taxa (II 95%)	Número (II 95%)	Taxa (II 95%)	
**IMC elevado**					
Acre	80,1 (40,3;128,7)	50,9 (23,5;84,4)	443,6 (305,1;590,6)	75,3 (50,7;102,5)	47,9 (11,7;140,2)
Alagoas	926 (477;1462,1)	69,8 (35,1;111,7)	3244,2 (2234,3;4349,1)	101,1 (69,1;137,2)	44,9 (9;123,8)
Amapá	53,2 (31,5;78,4)	54,7 (30,3;83,8)	365,3 (259,2;478,3)	71,5 (49,3;96,1)	30,6 (6,2;77,4)
Amazonas	525,2 (312,4;765,4)	70,6 (40,5;106,3)	2009,3 (1400,6;2636,9)	71,2 (49;94,7)	0,9 (-17,4;35,7)
Bahia	4395,5 (2318,1;6785,9)	65,3 (33,6;102,3)	13225,3 (8827,3;18489,3)	81,1 (54,1;113,1)	24,1 (-5,4;86,8)
Brasil	74.266,2 (43.491,7;110.056,9)	84,4 (48,1;127,9)	177.939,7 (124.637,7;237.783)	76,2 (52,9;102,1)	-9,7 (-23,1;16,2)
Ceará	1762,6 (831,5;2903,1)	43,8 (20,4;72,5)	7194,6 (4786,1;10036,6)	72,5 (48,2;101,2)	65,4 (19,9;172,1)
Distrito Federal	657,1 (439;908,9)	121,9 (75,5;176,5)	1751,1 (1220,7;2263)	80,6 (54,6;107,6)	-33,8 (-45,2;-14,6)
Espírito Santo	1089 (608,6;1642,8)	75,8 (40,9;117,6)	3588,6 (2472,9;4828,9)	83,7 (57,3;112,7)	10,4 (-13;61,5)
Goiás	1610,1 (830,7;2574,7)	76,8 (38,1;126,3)	5058,6 (3482;6954,6)	73,9 (50,3;101,7)	-3,8 (-29,4;49,2)
Maranhão	1245,2 (489,7;2212,8)	48,2 (18,5;87,4)	5846,2 (3709,8;8423,6)	89 (56;128,6)	84,8 (25,5;250,7)
Mato Grosso	511,1 (276,4;794,5)	63,8 (32,6;101,1)	2471,7 (1734,2;3272,6)	76,8 (52,7;102,4)	20,5 (-9,1;85,8)
Mato Grosso do Sul	746,8 (436,4;1106,5)	83,3 (46,1;128,2)	2223,5 (1521,9;2976,5)	77 (52;103,9)	-7,6 (-26;24,5)
Minas Gerais	7763 (4227,8;11827,3)	79,5 (41,8;123,4)	16660,8 (11377,3;22486,1)	62,7 (42,8;84,8)	-21,1 (-36,5;13,9)
Pará	1320,3 (711,4;2041,6)	65,2 (33,7;103,8)	5128,9 (3498,2;6897,9)	74 (49,8;100,6)	13,5 (-12,8;70,3)
Paraíba	1273,5 (638,4;2048,7)	56,6 (28,1;91,7)	3974,9 (2740,4;5388,3)	82 (56,8;111,2)	45 (9,4;129,3)
Paraná	4468,1 (2563,7;6646,6)	94,2 (52,1;145)	10015,1 (6653;13717,2)	77,1 (50,8;106,2)	-18,2 (-31,9;9,1)
Pernambuco	3345,4 (1772,7;5178,4)	75,4 (38,8;118,8)	9471,3 (6571,7;12641,6)	95,3 (65,8;128,2)	26,3 (0,7;83,6)
Piauí	669,5 (287,7;1133,6)	49,6 (20,4;86,2)	2676,8 (1799,4;3642,1)	70,1 (47,1;94,8)	41,3 (3,1;159,1)
Rio de Janeiro	11589,1 (7176,8;16646,3)	120,7 (72,9;175,6)	19470,2 (13465,9;26105,1)	87,3 (60,2;117,2)	-27,7 (-39,7;-6,8)
Rio Grande do Norte	835,1 (404,9;1341)	53,2 (25,5;85,9)	2990,3 (1997;4137,2)	75,7 (50,4;104,7)	42,2 (4,4;128,1)
Rio Grande do Sul	6158,4 (3741,6;8880,7)	96,3 (56,6;142,5)	11470,3 (7637,2;15714,9)	73,8 (49,1;101,1)	-23,3 (-34,4;-4,2)
Rondônia	306,4 (175,6;451,6)	92,2 (48,4;144,4)	1256,6 (872,4;1680,6)	84,2 (57,6;113,1)	-8,7 (-29,9;33,9)
Roraima	47,4 (27,1;70,9)	84,5 (44,7;131,4)	305,1 (221;394,8)	91 (64,2;119,8)	7,8 (-14,7;58,5)
Santa Catarina	2262,4 (1388,2;3300,1)	93,3 (55,9;139,1)	5617,9 (3838,4;7494)	72,1 (49;96,8)	-22,7 (-35,5;-1,7)
São Paulo	19864,6 (12062,3;28733,9)	102,9 (60,7;151,2)	38343,9 (26476,1;51560,4)	72,4 (49,6;97,8)	-29,6 (-41;-9,3)
Sergipe	547,1 (307,2;815,2)	73,4 (39,6;112,1)	1885,1 (1288,8;2533,3)	84,4 (57,5;114,3)	14,9 (-11,8;65,8)
Tocantins	214 (97,3;356,9)	56,9 (24,3;99,6)	1250,5 (836,2;1702,4)	89,4 (59,9;122,4)	57,1 (11,6;185)

*Fonte: Dados derivados do estudo Global Burden of Disease 2019, Institute for Health Metrics and Evaluation, University of Washington. ^46^*

**Tabela 10-12:** – Número de DALYs, taxas de DALYs padronizadas por idade por doença cardiovascular atribuída a IMC elevado (por 100 mil habitantes), em 1990 e 2019, e variação percentual das taxas no período, no Brasil e unidades federativas.

Causa de morte e Local	1990		2019		Variação percentual (II 95%)
	Número (II 95%)	Taxa (II 95%)	Número (II 95%)	Taxa (II 95%)	
**IMC elevado**					
Acre	1780,2 (889,4;2831,6)	883,9 (440;1418,2)	7206,1 (5100,2;9394,6)	1016,6 (702,7;1344,8)	15 (-11,4;79,5)
Alagoas	18827,9 (9699,9;29802,6)	1258,7 (648,4;1992)	50787,6 (35418;68595,7)	1487,6 (1027,8;2014,5)	18,2 (-10,2;77,2)
Amapá	1192,5 (719,1;1727,5)	957,2 (564,6;1414,6)	6210,3 (4496,9;8037,8)	994,6 (700,1;1313)	3,9 (-14,1;36,2)
Amazonas	11139,6 (6646;16127,6)	1182,8 (693,9;1745,6)	29209,5 (20703,6;38137,7)	898,5 (624,3;1187,5)	-24 (-37,1;-0,6)
Bahia	89200,9 (47500,8;135753)	1206 (642,6;1848,6)	192247,1 (129702,6;260980,6)	1157,8 (781,1;1574,5)	-4 (-27;40,7)
Brasil	1639034,2 (961484,2;2409951,1)	1611,6 (936,5;2376)	2696796,3 (1898493,9;3537093,7)	1108,9 (778,3;1460,7)	-31,2 (-40,5;-12,4)
Ceará	34076,1 (16343,8;55265)	791,3 (381,9;1288,2)	107555,9 (72801,4;147276,2)	1052,9 (706,8;1446,7)	33,1 (-3,5;117,9)
Distrito Federal	16685,1 (11186,2;22749)	2031,2 (1322,9;2849,8)	27936 (19529,6;35968,2)	971 (668,6;1257,6)	-52,2 (-59,9;-39,2)
Espírito Santo	25631,5 (14621,7;37654,9)	1506,1 (843,5;2239,7)	56314,7 (39334,5;74972,3)	1235,5 (864,8;1650,3)	-18 (-34,3;14,2)
Goiás	39404,4 (20135,7;61905,7)	1525,8 (774,6;2442)	81966,4 (56554,9;110604,8)	1089,1 (745,8;1476,4)	-28,6 (-48,1;8,5)
Maranhão	27659,1 (10640,5;49579,6)	950,3 (364,3;1710,5)	95781,1 (62438,9;135543,8)	1378,7 (888,2;1959,2)	45,1 (-3;180,4)
Mato Grosso	12499,7 (6759,1;19413,5)	1201,3 (643,5;1888,1)	39147,3 (27640,9;51142,7)	1069 (743,3;1406,8)	-11 (-31,8;33,7)
Mato Grosso do Sul	18472,6 (10922,8;27010,7)	1676,8 (976,7;2488,9)	36983,9 (26120,2;48494,8)	1188 (836,9;1566,2)	-29,2 (-42,1;-5,4)
Minas Gerais	178307,9 (96726,7;266926,7)	1573,5 (846,8;2377,1)	248509,9 (172433,1;331499,9)	933,9 (649,9;1243,3)	-40,6 (-52,4;-16,3)
Pará	29622,4 (15900,2;45428,6)	1197 (637,6;1854,3)	81046 (55976,2;107682,3)	1049,9 (718;1411,2)	-12,3 (-31,7;28,5)
Paraíba	22695,5 (11404,9;36547,5)	978,7 (497,2;1568)	54880,5 (37966,1;73798,1)	1165,1 (803,2;1567,4)	19 (-10,5;81,8)
Paraná	102453,2 (59672,3;149681,7)	1806,7 (1032,7;2664,8)	146167,1 (99521,6;198031,2)	1063,6 (719,1;1446,9)	-41,1 (-51;-22,6)
Pernambuco	68159,2 (35857,2;104510)	1393,8 (728;2142,4)	149066,6 (103475,9;196806,9)	1418,1 (980,7;1879,4)	1,7 (-18,6;45,6)
Piauí	14484 (6173;24176,3)	930,8 (392,4;1565,3)	40804 (28043,3;54462)	1076,5 (738,6;1442,1)	15,7 (-15,3;109,8)
Rio de Janeiro	265346,7 (163837;376942,1)	2415,5 (1492,4;3462,6)	294309,2 (203915,1;389667,6)	1306,1 (905;1730,1)	-45,9 (-54,6;-32)
Rio Grande do Norte	15080,4 (7424,1;23973,7)	915,4 (450,8;1457,1)	40596,4 (27349,8;55821,9)	1024,7 (691,1;1412,5)	11,9 (-16,1;73,8)
Rio Grande do Sul	134076,3 (82681;189686)	1812,7 (1106,9;2606)	154005,5 (103910,1;205502,3)	1003,2 (683,1;1332,5)	-44,7 (-52,5;-32,3)
Rondônia	7633,3 (4329,7;11286,4)	1575 (856,2;2405,9)	20049,9 (14029,6;26530,5)	1168,3 (810,5;1568)	-25,8 (-42,9;6,3)
Roraima	1085,3 (607,1;1643,6)	1310,2 (715,6;2019)	4525,5 (3246,3;5829,3)	1035,1 (726,4;1359,5)	-21 (-36,8;13,5)
Santa Catarina	48419,9 (29534,7;69616,8)	1659,7 (1004;2418,4)	80459,3 (55267,9;107093,5)	946,7 (647,5;1260,8)	-43 (-52,2;-27,3)
São Paulo	440645,2 (266543;633017,1)	1887,2 (1129,4;2747,7)	604648,5 (420556,1;807155,9)	1086,9 (754,4;1451,7)	-42,4 (-51,5;-25,9)
Sergipe	9519,2 (5300,6;14142,7)	1093,9 (604;1638,1)	26778,2 (18413,5;36053,3)	1120,1 (765,6;1518)	2,4 (-21,7;44,2)
Tocantins	4936 (2229;8219)	996,3 (440,5;1672,6)	19603,7 (13269,9;26463,2)	1283,3 (859,7;1742,7)	28,8 (-7,3;125,2)

*Fonte: Dados derivados do estudo Global Burden of Disease 2019, Institute for Health Metrics and Evaluation, University of Washington. ^46^*

**Tabela 10-11:** – Número de DALYs, taxas de DALYs brutas e padronizadas por idade por todas as causas atribuídas a IMC elevado no Brasil, em 1990 e 2019, por grupo etário, e variação percentual das taxas no período.

Causa de morte e Local	1990		2019		Variação percentual (II 95%)
	Número (II 95%)	Taxa (II 95%)	Número (II 95%)	Taxa (II 95%)	
**IMC elevado**					
15-49 anos	861.762,6 (516.176,5;1.233.120,2)	1124,3 (673,5;1608,8)	1370603,6 (1.050.414,8;1.717.233,6)	1186,8 (909,5;1486,9)	5,6 (-11,6;44,5)
50-69 anos	1.295.145,7 (795.232,8;1.864.296,5)	8255,8 (5069,2;11883,8)	2.994.279,6 (2.154.207,4;3.881.777,3)	7422 (5339,7;9621,9)	-10,1 (-22,2;13,7)
5-14 anos	6200,5 (2476,5;12237,7)	17,5 (7;34,6)	11291 (4692,6;22536,7)	35 (14,6;69,9)	99,5 (53,1;158,3)
70+ anos	412.245,5 (213.001,8;647.980,5)	9745,6 (5035,4;15318,4)	1.437.648,1 (953.535,3;1.955.964,7)	10984,1 (7285,3;14944,1)	12,7 (-7,1;54,6)
Padronizada por idade		2569,4 (1528,8;3742,3)		2404,5 (1733,3;3121,6)	-6,4 (-19,6;19,1)
Todas as idades	2.579.849,9 (1.556.675,2;3.720770,6)	1733,4 (1045,9;2499,9)	5.817.938,7 (4.197.826,2;7.541.630)	2685,2 (1937,5;3480,8)	54,9 (33,7;96,5)
Abaixo de 5	4495,6 (1661,7;8306,5)	26,5 (9,8;49)	4116,5 (1548;8183,5)	26,6 (10;52,8)	0,1 (-37,3;55,2)

*Fonte: Dados derivados do estudo Global Burden of Disease 2019, Institute for Health Metrics and Evaluation, University of Washington. ^46^*

**Tabela 10-13:** – Taxas de DALYs por doença cardiovascular atribuída a IMC elevado, padronizadas por idade (por 100 mil habitantes), por sexo, em 1990 e 2019, e variação percentual das taxas no período, no Brasil e unidades federativas.

Causa de morte e Local	Feminino			Masculino		
	1990 Taxa (II 95%)	2019 Taxa (II 95%)	Variação percentual (II 95%)	1990 Taxa (II 95%)	2019 Taxa (II 95%)	Variação percentual (II 95%)
**IMC elevado**						
Acre	1666,7 (945,5;2533)	2215,7 (1633,1;2831,2)	32,9 (5;90,2)	1462,7 (626,1;2473,2)	2477,1 (1665,7;3353,4)	69,4 (26,3;202,2)
Alagoas	2332,8 (1335,3;3446,4)	3140,8 (2273,2;4072,8)	34,6 (4;96,1)	2000,7 (894,3;3324,9)	3218,7 (2106,4;4491,3)	60,9 (16,5;177,9)
Amapá	1739,8 (1088,9;2470,2)	2201,4 (1625;2869,5)	26,5 (4,4;60,9)	1696,2 (917,9;2578,9)	2439,5 (1674,7;3243,9)	43,8 (15,6;105,3)
Amazonas	2213,9 (1411,8;3085,8)	2146,7 (1574,1;2758,1)	-3 (-19,4;23,3)	1937,2 (1052,2;2967)	2412 (1655,5;3272,9)	24,5 (-1,5;80)
Bahia	2273,3 (1364;3342,5)	2393,3 (1694,4;3160,6)	5,3 (-18,2;48,7)	1872,6 (851,1;3100,8)	2828 (1877,7;3973,6)	51 (6,2;161,3)
Brasil	2522,2 (1618,1;3530,4)	2218,9 (1660,5;2838,6)	-12 (-23,8;8,9)	2607,5 (1429,4;3944,7)	2606,2 (1811,8;3456,8)	-0,1 (-16,5;36,8)
Ceará	1445,3 (749,4;2281,7)	2141,3 (1518,3;2869,9)	48,2 (8,6;135,5)	1311,6 (563,9;2209,6)	2384,6 (1546,8;3346,4)	81,8 (25,1;231,1)
Distrito Federal	3223,9 (2167,8;4339,7)	2101,8 (1524,3;2716,4)	-34,8 (-44,7;-20)	3532,8 (2200,7;5211,7)	2622,5 (1830,6;3414,5)	-25,8 (-39,8;-0,9)
Espírito Santo	2299,2 (1369,2;3332,5)	2367,6 (1715,7;3054,1)	3 (-17,2;39,6)	2302,7 (1204,6;3522,3)	2798 (1890,3;3832)	21,5 (-6;84,9)
Goiás	2409 (1360,3;3691,8)	2230,6 (1605;2906,5)	-7,4 (-30,5;36,5)	2267,1 (1034;3714,1)	2441,9 (1619,5;3440,1)	7,7 (-24,4;83,1)
Maranhão	1294,4 (572,5;2152,6)	2534,5 (1733,4;3481,5)	95,8 (33,9;254,2)	1938,3 (708,5;3561,7)	3090,5 (1889,2;4607,6)	59,4 (3,1;222)
Mato Grosso	2039 (1201,2;3066,6)	2322,2 (1696,7;2988,7)	13,9 (-11,8;63,8)	1887 (929;3042)	2551,9 (1798;3417)	35,2 (-0,9;124,5)
Mato Grosso do Sul	2607,8 (1637,8;3665,6)	2345,2 (1719,7;3027)	-10,1 (-26,4;16,3)	2534,7 (1380,1;3834,3)	2644,9 (1803,1;3572,2)	4,3 (-18,1;49,9)
Minas Gerais	2525,3 (1521,6;3620,3)	1987,2 (1443,5;2594,3)	-21,3 (-35,7;5,1)	2368,8 (1119,3;3800)	2178 (1441,1;2993,5)	-8,1 (-30,2;52,3)
Pará	1934,7 (1111,7;2925,6)	2204,2 (1600,9;2887,9)	13,9 (-10,9;63,4)	1924,2 (965,8;3098,7)	2540,5 (1704,3;3421,4)	32 (-1,7;108,5)
Paraíba	1899,2 (1060,3;2852,9)	2439,5 (1774,9;3210,3)	28,5 (-0,8;96,3)	1594,8 (721,9;2636,9)	2742,3 (1819,5;3764,3)	71,9 (23,7;195,3)
Paraná	2714,9 (1685,9;3894)	2208,6 (1595;2896,2)	-18,7 (-32,2;5,6)	2724,5 (1450,3;4154,4)	2551,3 (1664,5;3540,3)	-6,4 (-24,7;32,5)
Pernambuco	2406 (1411,2;3488,8)	2649,2 (1900,4;3456)	10,1 (-12;52,1)	2159,8 (1023,9;3415,9)	3236,4 (2147,8;4376,2)	49,8 (13,8;135,2)
Piauí	1477,2 (720,6;2368,7)	2204,5 (1603,4;2884,7)	49,2 (10,6;150,6)	1504,4 (573,7;2651,8)	2313,5 (1535,8;3212,2)	53,8 (7,4;209,8)
Rio de Janeiro	3436,9 (2263,2;4669)	2455,3 (1792,8;3163,6)	-28,6 (-39,4;-10,7)	3970,5 (2328,9;5904,3)	3083,7 (2101,7;4138)	-22,3 (-36,9;5,4)
Rio Grande do Norte	1726,8 (947,3;2643,6)	2215,7 (1544,3;2943,3)	28,3 (-0,8;91,8)	1660,8 (767;2717,1)	2688,8 (1800,5;3760,4)	61,9 (16,8;171,5)
Rio Grande do Sul	2588,6 (1691,5;3570,9)	2064,1 (1490,8;2671,4)	-20,3 (-31,6;-0,6)	3051,2 (1777,5;4503,7)	2457,2 (1632,3;3370,7)	-19,5 (-32,3;4,3)
Rondônia	2864,4 (1727,9;4164,1)	2500,5 (1825,5;3218,3)	-12,7 (-29,4;17,4)	2306,9 (1185,4;3619,5)	2676,4 (1809,3;3626,4)	16 (-14,5;82)
Roraima	2485,3 (1513,2;3620,5)	2671,9 (1987,3;3404,1)	7,5 (-12,2;45,5)	2285,2 (1167,1;3580,1)	2652,2 (1828,6;3518,3)	16,1 (-11,2;77,4)
Santa Catarina	2588,5 (1693,7;3628,3)	1998,8 (1455,5;2620,4)	-22,8 (-34,8;-3,5)	2643,6 (1500,7;3972)	2338,3 (1570,6;3175,8)	-11,5 (-28,3;22,1)
São Paulo	2837,6 (1833,2;3917,5)	2053,6 (1507,5;2641,7)	-27,6 (-38,7;-9,2)	3171,7 (1783,1;4669,5)	2531,2 (1733,6;3432,3)	-20,2 (-35;8,7)
Sergipe	2331,9 (1433,6;3333,1)	2538,5 (1822,8;3342,3)	8,9 (-15,2;47,5)	1939,7 (981,7;3024,1)	2777,4 (1831,7;3783,5)	43,2 (5;129,3)
Tocantins	1721,5 (846,4;2717,9)	2523,8 (1842,5;3288,3)	46,6 (7,8;144,4)	1554,5 (636;2746,6)	2856,4 (1884,3;3998,6)	83,7 (27,8;251,3)

*Fonte: Dados derivados do estudo Global Burden of Disease 2019, Institute for Health Metrics and Evaluation, University of Washington. ^46^*

**Tabela 10-14:** – Número de DALYs, taxas de DALYs brutas e padronizadas por idade por doença cardiovascular atribuída a IMC elevado, por grupo etário, em 1990 e 2019, e variação percentual das taxas no período, no Brasil.

IMC elevado	1990 Número (II 95%)	1990 Taxa (II 95%)	2019 Número (II 95%)	2019 Taxa (II 95%)	Variação percentual (II 95%)
15-49 anos	11.876,9 (7.042;16.974,1)	15,5 (9,2;22,1)	13.729,8 (10.494,6;16.942)	11,9 (9,1;14,7)	-23,3 (-35,3;2,7)
50-69 anos	25.692,7 (15.086,6;37.700,4)	163,8 (96,2;240,3)	43.871,8 (30.231,8;58.205,9)	108,7 (74,9;144,3)	-33,6 (-42,5;-16,5)
5-14 anos					
70+ anos	15.077,1 (7.374,5;24.799,9)	356,4 (174,3;586,3)	40.905,3 (25.123,3;59.857,6)	312,5 (191,9;457,3)	-12,3 (-28,3;20,2)
Padronizada por idade		58,5 (32,7;89,7)		41,8 (28,1;56,8)	-28,5 (-38,8;-8,6)
Todas as idades	52.646,7 (30.085,3;78.950,7)	35,4 (20,2;53)	98.506,9 (66.815,9;133.940,7)	45,5 (30,8;61,8)	28,5 (11,1;63,2)
Abaixo de 5					

*Fonte: Dados derivados do estudo Global Burden of Disease 2019, Institute for Health Metrics and Evaluation, University of Washington. ^46^*

**Figura 10-1:**
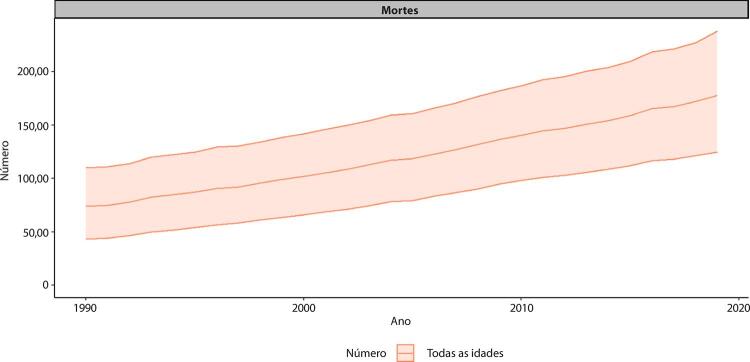
– Número de mortes por todas as causas atribuídas a IMC elevado, todas as idades, de 1990 a 2019.

Fonte: Dados derivados do estudo Global Burden of Disease 2019, Institute for Health Metrics and Evaluation, University of Washington. ^46^

**Figura 10-2:**
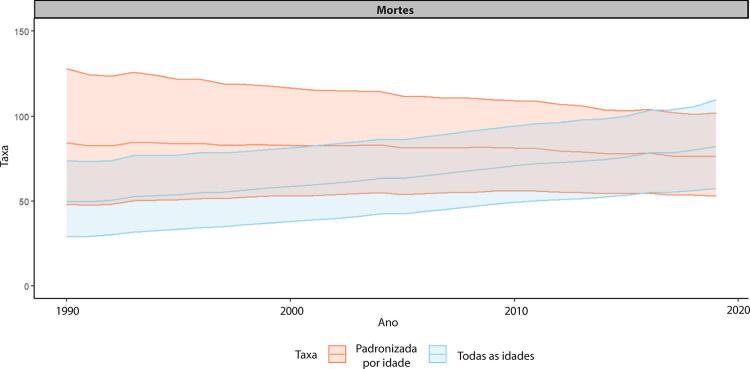
– Taxa de mortalidade por todas as causas atribuídas a IMC elevado, por 100 mil, todas as idades e padronizada por idade, no Brasil, de 1990 a 2019.

Fonte: Dados derivados do estudo Global Burden of Disease 2019, Institute for Health Metrics and Evaluation, University of Washington. ^46^

**Figura 10-3:**
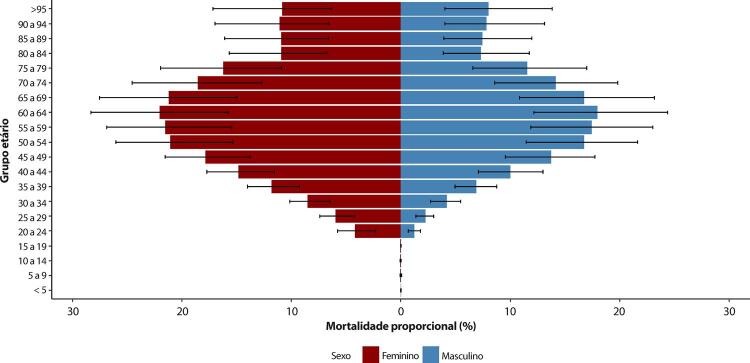
– Mortalidade proporcional por todas as causas atribuídas a IMC elevado, de acordo com grupo etário e sexo, no Brasil, 2019.

Fonte: Dados derivados do estudo Global Burden of Disease 2019, Institute for Health Metrics and Evaluation, University of Washington. ^46^

**Figura 10-4:**
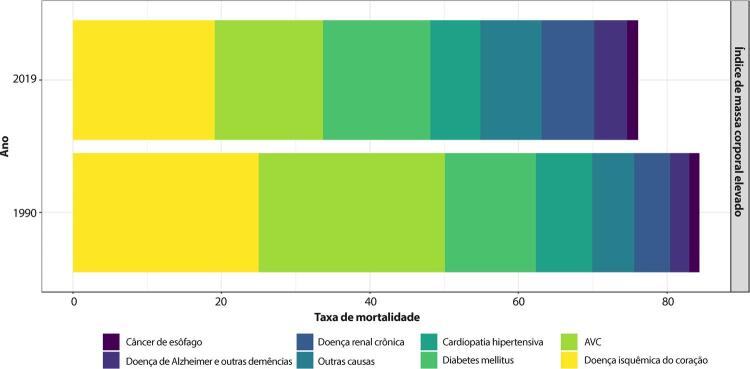
– Taxas de mortalidade por doenças atribuídas a IMC elevado padronizadas por idade, estratificadas por todas as causas, no Brasil, 1990 e 2019.

Fonte: Dados derivados do estudo Global Burden of Disease 2019, Institute for Health Metrics and Evaluation, University of Washington. ^46^

**Figura 10-5:**
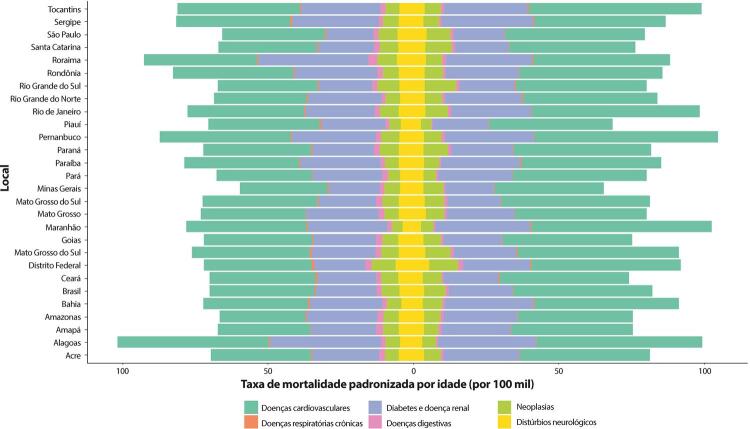
– Taxas de mortalidade padronizadas por idade por causas específicas atribuídas a IMC elevado, por 100 mil habitantes, de acordo com o sexo (mulheres à esquerda e homens à direita), por unidade federativa do Brasil em 2019. As barras coloridas representam as causas específicas de morte conforme a legenda.

Fonte: Dados derivados do estudo Global Burden of Disease 2019, Institute for Health Metrics and Evaluation, University of Washington. ^46^

**Figura 10-6:**
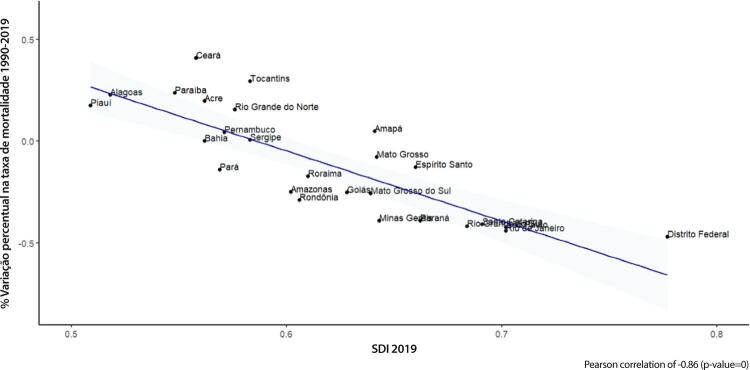
– Correlação entre o Índice Sociodemográfico (SDI) de 2019 e a variação percentual das taxas de mortalidade por doença cardiovascular atribuída a IMC elevado, padronizadas por idade, entre 1990 e 2019.

Fonte: Dados derivados do estudo Global Burden of Disease 2019, Institute for Health Metrics and Evaluation, University of Washington. ^46^

**Figura 10-7:**
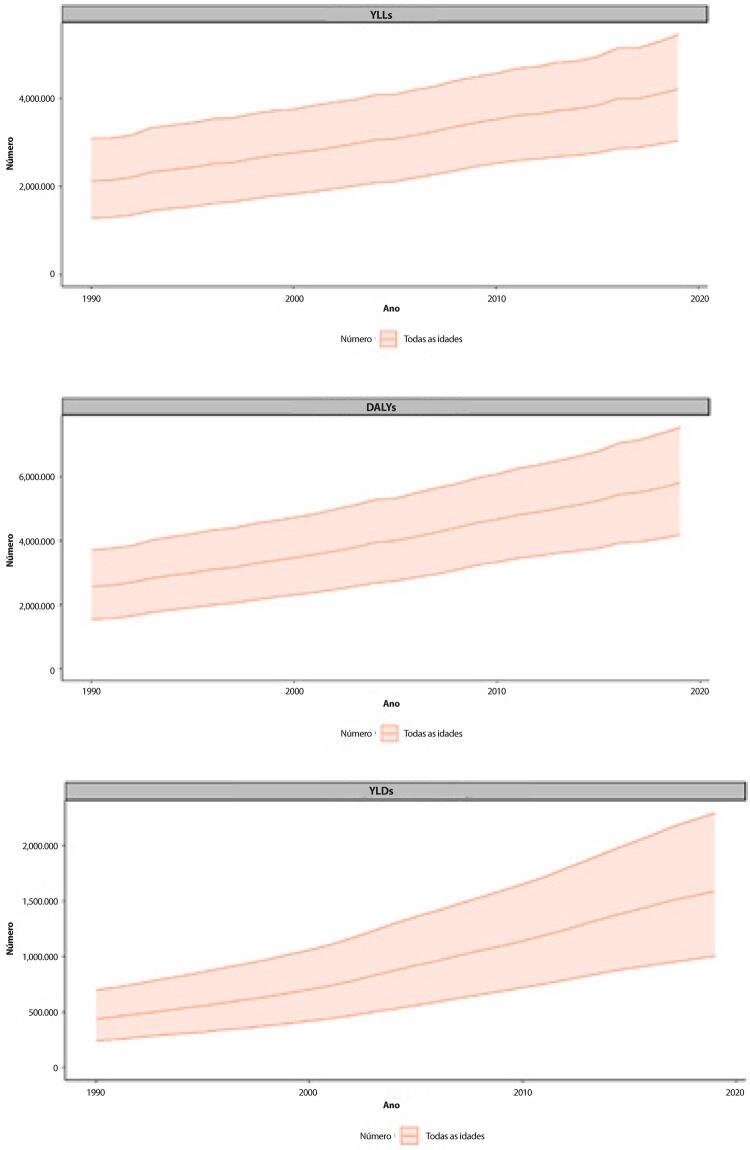
– Número de YLLs (A), YLDs (B) e DALYs (C) por IMC elevado, todas as idades, ambos os sexos, Brasil, de 1990 a 2019.

Fonte: Dados derivados do estudo Global Burden of Disease 2019, Institute for Health Metrics and Evaluation, University of Washington. ^46^

**Figura 10-8:**
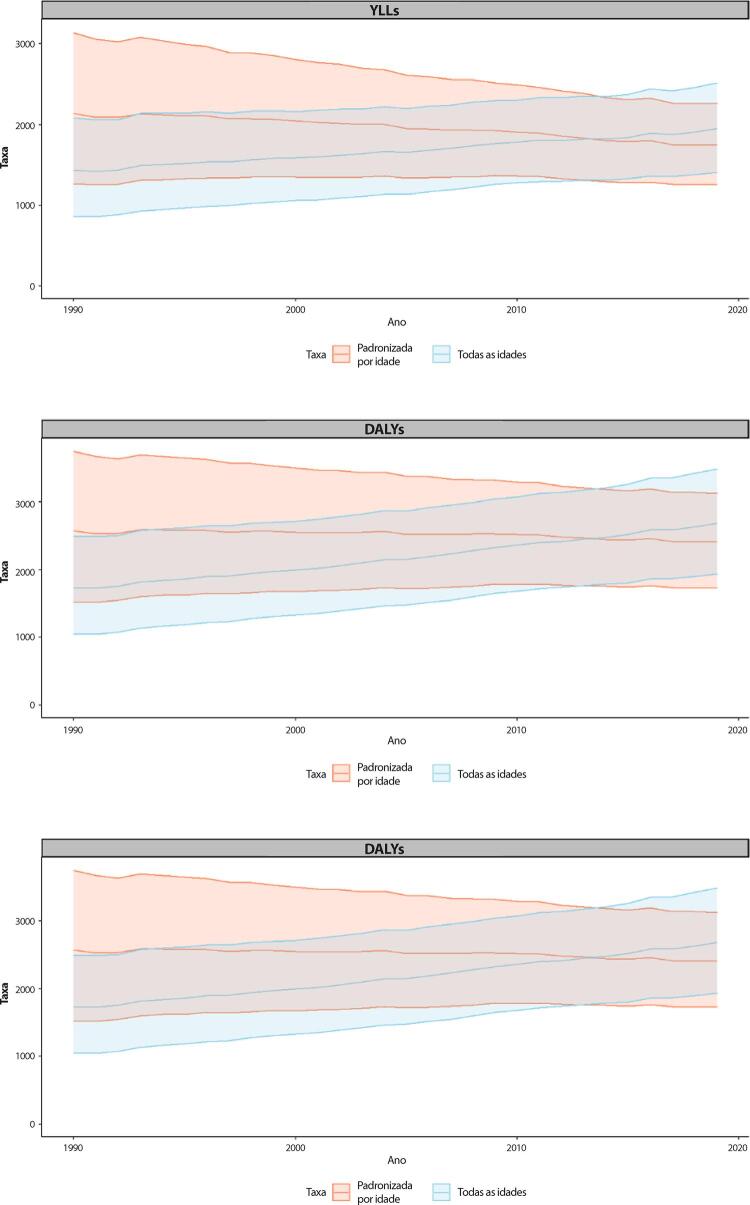
– Taxas de YLLs (A), YLDs (B) e DALYs (C) por IMC elevado para todas as idades e padronizadas por idade, por 100 mil, ambos os sexos, Brasil, de 1990 a 2019.

Fonte: Dados derivados do estudo Global Burden of Disease 2019, Institute for Health Metrics and Evaluation, University of Washington. ^46^

**Figura 10-9:**
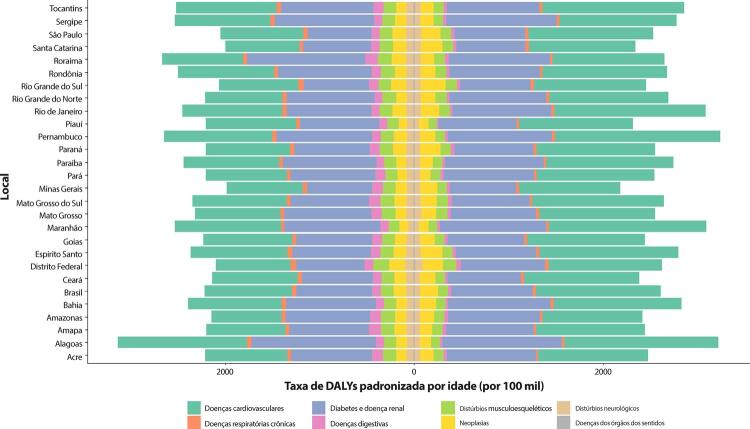
– Taxas de DALYs padronizadas por idade para causas específicas atribuídas a IMC elevado, por 100 mil habitantes, de acordo com o sexo (mulheres à esquerda e homens à direita), por unidade federativa em 2019. As barras coloridas representam as causas específicas de morte conforme a legenda.

Fonte: Dados derivados do estudo Global Burden of Disease 2019, Institute for Health Metrics and Evaluation, University of Washington. ^46^

**Figura 10-10:**
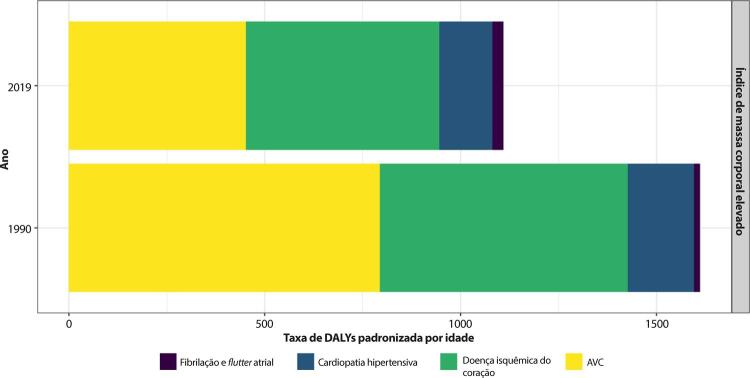
– Taxas de DALYs por doenças cardiovasculares atribuídas a IMC elevado, padronizadas por idade, no Brasil, 1990 e 2019.

Fonte: Dados derivados do estudo Global Burden of Disease 2019, Institute for Health Metrics and Evaluation, University of Washington. ^46^

### Panorama

•Segundo definição da OMS, obesidade é um acúmulo anormal ou excessivo de gordura que representa risco à saúde, sendo considerada presente quando o IMC é ≥ 30 kg/m^2^ . Trata-se de condição multifatorial, ligada não apenas ao desequilíbrio entre consumo e gasto calórico e, portanto, a uma dieta rica em alimentos com alto teor de açúcar e gordura, mas também a fatores genéticos, metabólicos, ambientais, econômicos, socioculturais, dentre outros, que levam, em última análise, ao acúmulo de gordura corporal. Além de inserida no grupo de DCNT, a obesidade é considerada um importante fator de risco para afecções como diabetes mellitus, hipertensão e DCV. A obesidade é atualmente considerada uma pandemia, com impacto tanto em países desenvolvidos quanto naqueles em desenvolvimento e repercussão nos níveis individual, social, familiar e financeiro dos indivíduos. O sobrepeso, definido como IMC acima de 25 kg/m^2^ , também se associa às complicações observadas nos obesos, com aumento crescente de tais complicações ocorrendo em paralelo ao aumento do IMC. ^365^ Apesar de o IMC apresentar limitações para avaliar excesso de peso, como a não definição da quantidade de gordura que contribui para o peso corporal, ^366^ essa variável é atualmente utilizada pela OMS para suas definições.

•As mortes e a carga de doença atribuídas a obesidade aumentaram globalmente entre 1990 e 2019, passando de 2,20 (II 95%, 1,21 - 3,43) para 5,02 (II 95%, 3,22 - 7,11) milhões de mortes e de 67,3 (II 95%, 38 - 104) para 160 (II 95%, 106 - 219) milhões de DALYs em números absolutos. A obesidade contribuiu para mais YLLs, 119 milhões (79,6 - 164), do que YLDs, 40,9 milhões (24,5 - 60,9), no mundo em 2019. Possivelmente a maior parte desse incremento (4,9% {7,3-24,6} mortes, 18% {2,2-42,3} DALYs e 8,3% [-6,6-31,2] YLLs) decorreu do crescimento e envelhecimento da população, demonstrados após padronização pela idade. ^4^

•A obesidade contribui para o agravamento da maioria dos fatores de risco cardiovascular, notadamente para a elevação de pressão arterial, glicemia e lipídios séricos, além de apresentar efeitos adversos decorrentes da inflamação sérica e alteração da estrutura e função cardíacas. Essa associação se manifesta na relação existente entre obesidade e aumento da prevalência de hipertensão, doença arterial coronariana, insuficiência cardíaca e fibrilação atrial, dentre outras. Assim, ainda que não se tenha alcançado consenso sobre a questão de a obesidade ser considerada uma doença ou um fator de risco, este capítulo procura abordá-la como fator de risco cardiovascular. ^367^

•Importante ressaltar que, para a padronização por idade das taxas, foi empregada a população global usada pelo GBD.

### Prevalência

•A Tabela 10 -1 mostra a prevalência de excesso de peso e obesidade na população total com idade a partir de 18 anos, por sexo e grupos etários, no Brasil, em 2019, segundo dados antropométricos aferidos pela PNS. No Brasil, o percentual de adultos (≥18 anos de idade) com excesso de peso e obesidade em 2019 foi de 57,5% (IC 95%, 54,8 - 60,2) e 21,8 % (IC 95%, 19,2 - 24,7) para os homens, e de 62,6% (IC 95%, 59,1 - 66,0) e 29,5% (IC 95%, 25,4 - 34,0) para as mulheres. Observa-se aumento progressivo do excesso de peso com o incremento da idade, variando de 33,7% (IC 95%, 27,4 - 40,6) [masculino: 25,7% (IC 95%, 19,1 - 33,7); feminino: 41,7% (IC 95%, 31,1 - 53,1)] no grupo etário de 18-24 anos a 70,3% (IC 95%, 67,4 - 73,1) [masculino: 67,1% (IC 95%, 62,1 - 71,8); feminino: 73,1% (IC 95%, 68,8 -77,0)] no grupo etário de 40-59 anos. A partir dos 60 anos, há uma pequena redução da prevalência, 64,4% (IC 95%, 60,5 - 68,1) [masculino: 63,3% (IC 95%, 56,9 - 69,2); feminino: 65,3% (IC 95%, 60,3 - 69,7)]. O mesmo aconteceu com a obesidade, com aumento progressivo com a idade, variando de 10,7% (IC 95%, 7,7 - 14,7) [masculino: 7,9% (IC 95%, 4,8 - 12,8); feminino: 13,5% (IC 95%, 8,8 - 20,4)] no grupo etário de 18-24 anos a 34,4% (IC 95%, 29,7 - 39,4) [masculino: 30,2% (IC 95%, 24,8 - 36,3); feminino: 38,0% (IC 95%, 32,3 - 44,0)] no grupo etário de 40-59 anos. A partir dos 60 anos, há uma pequena redução da prevalência, 24,8% (IC 95%, 20,9 - 29,1) [masculino: 21,2% (IC 95%, 15,6 - 28,1); feminino: 27,5 (IC 95%, 23,0 -32,5)]. Nota-se que a prevalência de excesso de peso e obesidade foi maior no sexo feminino em todos os grupos etários.

•A Tabela 10 -2 mostra o percentual de adultos com excesso de peso, segundo método de imputação (IMC_i ≥ 25 kg/m^2^), por sexo, segundo as capitais dos estados brasileiros e Distrito Federal com base na Pesquisa Vigitel 2019. Campo Grande, Cuiabá, Fortaleza, Manaus, Natal, Porto Alegre, Porto Velho, Recife, Rio Branco, Rio de Janeiro e São Paulo apresentaram percentuais superiores aos nacionais para ambos os sexos. Para os homens, as capitais Boa Vista, Campo Grande, Cuiabá, Curitiba, Florianópolis, Fortaleza, Goiana, Manaus, Natal, Porto Alegre, Recife e Rio Branco apresentaram percentuais superiores ao nacional. Nota-se que, para o excesso de peso nas mulheres, houve menor número de capitais acima da média nacional: Manaus, Natal, Recife, Rio Branco, Rio de Janeiro, Salvador, São Paulo e Distrito Federal.

•A Tabela 10 -3 apresenta o percentual de adultos com obesidade, segundo método de imputação (IMC_i ≥ 30 kg/m^2^), por sexo, segundo as capitais dos estados brasileiros e Distrito Federal, a partir da Pesquisa Vigitel 2019. Aracajú, Boa Vista, Campo Grande, Cuiabá, Macapá, Manaus, Natal, Porto Alegre, Recife, Rio de Branco e Rio de Janeiro apresentaram percentuais superiores aos nacionais para ambos os sexos. Para os homens, as capitais Belém, Belo Horizonte, Boa Vista, Campo Grande, Cuiabá, Curitiba, Goiânia, Macapá, Manaus, Natal, Porto Alegre, Porto Velho, Recife, Rio Branco e Rio de Janeiro estavam acima do percentual nacional de obesidade. As mulheres apresentaram percentual superior ao dos homens para obesidade, comportamento oposto ao observado para o excesso de peso. As capitais com percentual de mulheres acima da média nacional foram Aracajú, Campo Grande, Cuiabá, João Pessoa, Macapá, Maceió, Manaus, Porto Alegre, Porto Velho, Recife, Rio Branco, Rio de Janeiro e São Paulo.

•Felisbino-Mendes *et al* ., empregando dados do estudo GBD 2017, demonstraram que a prevalência de obesidade, padronizada para idade, no Brasil foi maior no sexo feminino (29,8%) do que no masculino (24,6%) em 2017. Porém, os homens apresentaram maior aumento da obesidade (244,1%) do que as mulheres (165,7%) no período de 1990 a 2017. Um aumento anual de mais de 300% foi observado na maior parte dos estados do Norte e Nordeste para os homens, provavelmente devido ao atraso da transição epidemiológica nessas regiões geográficas. Mais da metade da população estava acima do peso na maioria dos estados brasileiros, exceto Maranhão e Piauí. ^368^ Esses achados são consistentes com os do estudo que observou maior prevalência de obesidade em populações menos favorecidas, que migraram da desnutrição para a supernutrição, contribuindo para a nutrição inadequada em todo o mundo. ^369^

•Flores-Ortiz *et al* ., estudando dados autorrelatados de peso e altura de 572.437 adultos nas capitais brasileiras e no Distrito Federal, de 2006 a 2016, de ambos os sexos, estimaram a prevalência geral de obesidade e observaram aumento de 11,7% para 18,1% em homens, e de 12,1% para 18,8% em mulheres, que foi mais acentuada também nas regiões Norte, Nordeste e Centro-Oeste. ^370^

•Araújo *et al* ., empregando dados da Pesquisa Vigitel de 2008 a 2015, observaram aumento de sobrepeso e obesidade em mulheres brasileiras em idade reprodutiva, que ocorreu independentemente de idade, educação formal, estado civil, raça/cor e região de residência (exceto para mulheres de 30 a 39 anos, negras e que vivem na região Sul). Os autores salientam que, em comparação aos dados da população geral, observaram maior incremento de obesidade entre mulheres de 18 a 49 anos e aumento na prevalência de sobrepeso entre mulheres de 18 a 29 anos no período, apontando para a ocorrência precoce desse fator de risco para DCV e para DCNT. ^371^ Importante lembrar que a Pesquisa Vigitel utiliza diagnóstico autorrelatado pelo entrevistado.

•Realizou-se estudo transversal de base populacional em 2002 e 2003 com participantes do “Inquérito domiciliar sobre comportamentos de risco e morbidade referida de doenças e agravos não transmissíveis”, composto por 23.457 indivíduos com idade a partir de 15 anos, residentes em 16 capitais (Aracaju, Belém, Belo Horizonte, Brasília, Campo Grande, Curitiba, Florianópolis, Fortaleza, João Pessoa, Manaus, Natal, Porto Alegre, Recife, Rio de Janeiro, São Paulo e Vitória). Esse estudo analisou 3.142 idosos, dos quais 1.868 (59,4%) eram mulheres e 1.274 (40,6%), homens. A média de idade foi 69,5±0,19 anos e 1.742 (55,4%) tinham idade entre 60 e 69 anos. A prevalência de obesidade foi de 17,7% na faixa de 60-69 anos, 22,9% na faixa de 70-79 anos e 17,5% a partir dos 80 anos. A obesidade foi mais frequente entre mulheres (19,3%; IC 95%, 16,6-22,3; χ^2^= 9,5; p = 0,03). ^372^

•Estudo com 157 mulheres pós-menopausa atendidas em dois ambulatórios públicos da cidade de São Paulo observou média de IMC de 28,0 kg/m^2^ e 34,4% de obesidade, que foi classificada como classe I em 26,1%, classe II em 5,7% e classe III em 2,6%. Houve associação estatisticamente significativa da prevalência de obesidade global com o grau de instrução (p=0,006), a prática de atividade física (p<0,001), o uso de terapia hormonal na menopausa (p=0,007) e a paridade (p=0,002). A prevalência de obesidade abdominal foi de 73,8%. A proporção de mulheres com obesidade abdominal foi maior entre aquelas com até 7 anos de escolaridade (p=0,030). ^373^

•Estudo transversal de base populacional em que foram utilizados dados coletados pela PNS de 2013 relatou IMC ideal (<25 kg/m^2^) em 46,8% das mulheres (IC 95%, 45,5 - 48,1), 40,5% dos homens (IC 95%, 39 - 42) e 43,7% do total (IC 95%, 42,7 - 44,7). Na estratificação por faixa etária, a prevalência de IMC ideal foi de 54,2% (IC 95%, 52,4 - 55,8) entre 18 e 35 anos, 36,8% (IC 95%, 35,4 - 38,2) entre 36 e 59 anos e 40,9% (IC 95%, 38,6 - 43,2) a partir dos 60 anos. O IMC ideal foi mais frequente na região Nordeste (47,5%) e menos frequente na região Sul (40,4%) do Brasil. ^374^

•O ELSA-Brasil é uma coorte de 15.105 servidores ativos ou aposentados, voluntários, de universidade ou instituições de pesquisa de 6 cidades do Brasil, inscritos entre agosto de 2008 e dezembro de 2010, com idade entre 35 e 74 anos, majoritariamente composta por mulheres (54%) e adultos de meia-idade (78% com idade <60 anos). Um subestudo que incluiu 14.545 participantes com idades entre 35 e 74 anos, com maioria (54,1%) de mulheres, observou que 22,7% (n = 3.298) eram obesos e 40,8% (n = 5.934) apresentavam sobrepeso. Reportou classificação em “estado metabolicamente saudável”, por múltiplos critérios, em 12,0% (n = 396) de obesos e 25,5% (n = 1514) de sobrepeso, que foi associado a menor idade, sexo feminino, menor IMC e mudança de peso a partir dos 20 anos em todas as categorias de IMC. ^375^ Um subestudo incluindo 6.453 homens e 7.686 mulheres avaliou a associação do IMC e da circunferência da cintura com a classe sócio-ocupacional e observou que, para as mulheres, os efeitos da classe sócio-ocupacional baixa e média foram maiores para aquelas com circunferência da cintura entre 80 e 88 cm ou sobrepeso, e para os homens, a classe sócio-ocupacional baixa e média se associou com circunferência da cintura adequada ou IMC normal. ^376^ Outro subestudo que avaliou toda a coorte reportou que a prevalência de sobrepeso foi maior nos homens e a obesidade foi mais comum nas mulheres. Tempo insuficiente para cuidados pessoais e lazer foi associado com sobrepeso (RP = 1,29; IC 95%, 1,04 - 1,61) e obesidade (RP = 1,65; IC 95%, 1,28 - 2,12) nas mulheres que trabalhavam mais de 40 horas/semana e os autores concluíram que os resultados decorrem das desigualdades de gênero envolvendo as relações entre tempo e saúde. ^377^ Outro estudo empregando modelos de regressão logística multinível ajustados para idade, educação, cor da pele, estado de residência, nível individual, pontuação de coesão social e violência percebida mostrou que as mulheres que vivem em áreas menos coesas socialmente e bairros considerados mais violentos tiveram maiores chances de obesidade em comparação com suas contrapartes (OR 1,25, IC 95%, 1,02 - 1,53; OR 1,28, IC 95%, 1,04 - 1,56, respectivamente). ^378^

#### Crianças e Adolescentes

•O aumento da prevalência de obesidade também foi observado em crianças e adolescentes brasileiros. Meta-análise de 21 estudos com 18.463 crianças/adolescentes brasileiros estimou prevalência de 14,1%, sendo 16,1% nos meninos e 14,95% nas meninas, sem diferença significativa entre os sexos (RP = 1,06; IC 95%, 0,81 - 1,40; p> 0,05). ^379^

•O estudo ERICA avaliou 73.399 alunos, 55,4% do sexo feminino, com média de idade de 14,7±1,6 anos. A prevalência de obesidade foi de 8,4% (IC 95%, 7,9 - 8,9), menor na região Norte e maior na região Sul e, quanto ao sexo, maior no masculino. Adolescentes obesos apresentaram maior prevalência de hipertensão, 28,4% (IC 95%, 25,5 - 31,2), do que adolescentes com sobrepeso, 15,4% (IC 95%, 17,0 - 13,8), ou eutróficos, 6,3% (IC 95%, 5,6 - 7,0). A fração de hipertensão atribuível à obesidade foi de 17,8%. ^299^

•Revisão sistemática com meta-análise publicada por Sbaraini *et al.* em 2021 compilou dados de 151 estudos de prevalência de sobrepeso e obesidade em adolescentes brasileiros de 10 a 19 anos. Observou-se aumento na prevalência de sobrepeso nas últimas décadas: 8,2% (IC 95%, 7,7 - 8,7) até 2000, 18,9% (IC 95%, 14,7 - 23,2) de 2000 a 2009, e 25,1% (IC 95%, 23,4 - 26,8) de 2010 em diante, padrão que foi similar para a prevalência de obesidade. As regiões Sudeste e Sul tiveram maiores prevalências de sobrepeso e obesidade, sem diferença entre os sexos. ^337^

•Os determinantes do aumento da prevalência de obesidade incluem mudanças na alimentação, meio ambiente, maior oferta de alimentos de alto teor energético, *marketing* , urbanização e redução do tempo e espaço para atividades físicas. ^380^

#### Incidência

•Estudo derivado da coorte do ELSA-Brasil com seguimento de 3,8 anos de 13.625 homens e mulheres de 35-74 anos, incluídos no período de 2008-2010, demonstrou incidência global de 7,7% e 10,6% para sobrepeso e obesidade, respectivamente. Os maiores percentuais foram observados entre as mulheres com baixa escolaridade (35,0%) e negras (28,5%), e nos homens jovens (21,1%). Os autores reportaram também aumento de sobrepeso com idade, baixa de renda per capita e menor escolaridade. ^381^

•Empregando dados da Pesquisa Vigitel, foram estimadas a incidência e a persistência da obesidade entre brasileiros adultos no período de 2006 a 2009. Os autores observaram que a incidência do excesso de peso aos 20 anos é estimada em 40% para os homens e 30% para as mulheres. A persistência da obesidade, por sua vez, é estimada em 65% no sexo masculino e 47% no feminino. Os autores apontam a necessidade da elaboração de políticas públicas, particularmente para jovens, que reduzam os riscos dietéticos e promovam a atividade física. ^382^

#### Mortalidade

•Meta-análise de 239 estudos prospectivos demonstrou relação consistente de sobrepeso e obesidade (em todos os graus) com mortalidade por todas as causas em diferentes populações no mundo. Os autores questionaram a hipótese de que houvesse obesos metabolicamente saudáveis. ^383^

•Em 1.450 indivíduos com 60 anos ou mais do Estudo de Coorte de Envelhecimento de Bambuí, observou-se que o IMC contínuo (HR 0,85; IC 95%, 0,80 - 0,90) relacionou-se inversamente à mortalidade, mesmo após ajustes para as variáveis confundidoras. Obesidade ocorreu em 12,5% dos idosos e foi positivamente associada com sexo feminino, maior renda familiar e presença de hipertensão e diabetes, e inversamente com atividade física. Sobrepeso foi inversamente (HR 0,76; IC 95%, 0,61 - 0,93) associado a mortalidade. Indivíduos com IMC entre 25 kg/m^2^ e 35 kg/m^2^ tiveram as menores taxas absolutas de morte em 10 anos de acompanhamento. Os autores concluem que os pontos de corte usuais de IMC não devem ser usados para a elaboração de políticas públicas para idosos no Brasil. ^384^

#### Mortalidade por Todas as Causas Atribuíveis a IMC Elevado

•A Tabela 10 -4 apresenta as taxas de mortalidade por todas as causas atribuídas a IMC elevado, padronizadas por idade, por 100 mil habitantes, em 1990 e 2019, e a variação percentual das taxas no período, no Brasil e UF (GBD 2019). Os maiores decréscimos dos percentuais de mortalidade ocorreram nos estados com maior renda no Brasil. O Distrito Federal apresentou a maior redução -33,8 (-45,2;-14,6), seguido de São Paulo -29,6 (-41;-9,3), Rio de Janeiro -27,7 (-39,7;-6,8), Santa Catarina -22,7 (-35,5;-1,7), Minas Gerais -21,1 (-36,5;13,9), Paraná -18,2 (-31,9;9,1), Bahia -9,7 (-23,1;16,2), Rondônia -8,7 (-29,9;33,9), Mato Grosso do Sul -7,6 (-26;24,5) e Goiás -3,8 (-29,4;49,2). Quanto à obesidade nos homens, segundo a Tabela 10 -5, a maioria das UF apresentou variação percentual positiva nas taxas de morte decorrente de IMC elevado, que variou de 7,6 (-17,9;72,3) em Roraima a 63,1(4,4;230,1) no Maranhão.

•Quanto à obesidade nas mulheres, segundo a Tabela 10 -5, a maioria dos estados apresentou variação percentual positiva das mortes dela decorrentes, com variação de 1,2 (-23,9;46,9) em Sergipe a 109 (39,4;294,2) no Maranhão, exceto o Distrito Federal, que apresentou a maior redução -38,2 (-49,9;-20,6), seguido de São Paulo -34,4 (-46,3;-14,3), Rio de Janeiro -31.5 (-43,8;-10), Santa Catarina -28.5 (-41,7;-6,6), Minas Gerais -27,6 (-42,6;1,4), Paraná -24,5 (-38,5;2,3), Rio Grande do Sul -24,6 (-37,2;-2,6), Mato Grosso do Sul -14,5 (-32;16,7), Amazonas -13 (-30,5;17,1), Goiás -10,4 (-35,9;42,1) e Espírito Santo -1,3 (-24,9;43,5). Os maiores decréscimos dos percentuais de mortalidade ocorreram no Brasil para as mulheres, -16,3 (-28,8;6), em relação aos homens, -2,1 (-19;35,5) ( Tabela 10 -5).

•A Tabela 10 -6 mostra o número de mortes, as taxas de mortalidade brutas e padronizadas por idade para todas as causas de morte atribuídas a IMC elevado no Brasil, por grupo etário, em 1990 e 2019, além da variação percentual no período, segundo dados do GBD 2019. Observa-se redução percentual de -9,7 (-23,1;16,2) se considerarmos as taxas padronizadas por idade no período.

•A Figura 10-1 mostra aumento do número de mortes por todas as causas atribuídas a IMC elevado, em todas as idades no Brasil. Na Figura 10-2 , observam-se as taxas de mortalidade brutas e padronizadas por idade por todas as causas atribuídas a IMC elevado de 1990 a 2019. Enquanto as taxas brutas aumentaram, as padronizadas por idade apresentaram estabilidade no período, sugerindo que um fator determinante do aumento seja o envelhecimento da população. Segundo Brant *et al* ., quanto ao *ranking* das taxas de mortalidade padronizadas por idade por DCV atribuíveis aos fatores de risco, segundo sexo, em 1990 e 2019, houve ascensão do IMC elevado, passando de 6ª posição para 3ª nas mulheres e de 7ª para 4ª nos homens. ^269^

•A distribuição da mortalidade proporcional atribuída ao IMC elevado, por faixa etária e por sexo, pode ser vista na Figura 10-3 . Observamos maior mortalidade proporcional devido a IMC elevado entre 50 e 69 anos em homens e mulheres, com predomínio das mulheres dessas faixas etárias. A mortalidade proporcional padronizada pela idade foi 84,4 (48,1;127,9) em 1990 e 76,2 (52,9;102,1) em 2019, com variação percentual de -9,7 (-23,1;16,2).

•A Figura 10-4 mostra as taxas de mortalidade por doenças atribuídas a IMC elevado, padronizadas por idade, estratificadas por todas as causas, no Brasil, 1990 e 2019, segundo o GBD 2019. O IMC elevado contribuiu predominantemente para mortalidade por: doença isquêmica do coração [número de mortes: 21.732,5 (11.703,8;34.207,7) em 1990 e 45.210,1 (29.102,5;63.084,1) em 2019; mortalidade proporcional: 25,1% (13,2%; 40,2%) em 1990 e 19,1 (12,2%; 27,0%) em 2019]; AVC [número de mortes: 24.398,5 (14.209,8;35.441,2) em 1990 e 35.124,7 (24.073,7;46.859,7) em 2019; mortalidade proporcional: 25% (14,2%;37,1%) em 1990 e 14,6% (9,9%;19,6%) em 2019]; e diabetes [número de mortes: 10.862,5 (6.997,5;15.055,0) em 1990 e 33.811,0 (24.964,7;43.247,8) em 2019; mortalidade proporcional: 12,2% (7,7%;17,3%) em 1990 e 14,5% (10,5%;18,6%) em 2019]. A contribuição para todas as causas de morte foi 74.266,2 (43.491,7;110.056,9) e 177.939,7 (124.637,7;237.783,0) em números de mortes em 1990 e 2019, respectivamente.

• A Figura 10-5 mostra as taxas de mortalidade padronizadas por idade por causas específicas atribuídas a IMC elevado, por 100 mil habitantes, para todas as idades, por sexo, nas UF, segundo o GBD 2019. Observa-se que a mortalidade variou nas UF de forma diferente para os sexos. As maiores taxas de mortalidade padronizadas por idade para DCV e diabetes atribuídas a IMC elevado nas mulheres (por 100 mil habitantes) foram observadas em Alagoas (52,1), Pernambuco (45,0), Tocantins (42,1), Espírito Santo (40,7), Maranhão (40,5), Rondônia (40,2) e Rio de Janeiro (40,1). Nos homens, essas taxas foram maiores em Pernambuco, Maranhão, Rio de Janeiro, Tocantins e Alagoas. Felisbino-Mendes *et al* . reportaram que, em 2017, a obesidade foi responsável por 12,3% das mortes por todas as causas, perfazendo um total de 165.954 mortes. A mortalidade proporcional atribuída a IMC elevado foi mais expressiva nas mulheres (14,6%, II 95%, 10,7 - 18,9) do que nos homens (10,5%, II 95%, 7,2 - 14,1). ^368^

•A Figura 10-6 mostra a correlação entre o SDI de 2019 e a variação percentual das taxas de mortalidade por DCV atribuída ao IMC elevado, padronizadas por idade, entre 1990 e 2019, em todas as idades e ambos os sexos. Nota-se que a diminuição da mortalidade por DCV atribuída a IMC elevado foi correlacionada com melhora nas condições socioeconômicas das UF e provavelmente decorreu da transição epidemiológica tardia com competição de causas de mortes nas regiões com menor renda.

#### Mortalidade por Doenças Cardiovasculares Atribuídas a IMC Elevado

•As Tabelas 10-7 e 10-8 mostram números de mortes e taxas de mortalidade padronizadas por idade por DCV atribuída a IMC elevado, por 100 mil habitantes, e a variação percentual das taxas, no Brasil e suas UF, em 1990 e 2019 (GBD 2019). Com relação aos homens, a maioria das UF apresentou variação percentual negativa das mortes decorrentes de IMC elevado, que variou de -1 (-29,5;65,5) no Mato Grosso a -44,6 (-57,4;-21,5) no Distrito Federal. Os maiores decréscimos dos percentuais de mortalidade ocorreram nas UF com maior renda no Brasil. As maiores variações percentuais positivas nos homens ocorreram nos estados das regiões Norte [Tocantins 50,5 (1,6;202,0)] e Nordeste [Ceará 50,2 (-0,7;188,2)]. No Brasil, ocorreu variação negativa das taxas de mortalidade por DCV atribuíveis a IMC elevado para os homens [-22,8 (-35,9;6,2)].

•Com relação às mulheres, a maioria dos estados apresentou variação percentual negativa das mortes por DCV decorrentes de IMC elevado, que variou de -2,8 (-22,6;32,1) no Amapá a -50,4 (-60,4;-35,8) no Distrito Federal. Os maiores decréscimos nos percentuais de mortalidade ocorreram nas UF com maior renda no Brasil. As maiores variações percentuais positivas nas mulheres ocorreram nos estados da região Nordeste [Maranhão 89,9 (27,4;262,4)]. No Brasil ocorreu variação negativa das taxas de mortalidade por DCV atribuíveis a IMC elevado para as mulheres [-33,9 (-43,7;-16,7)] maior do que para os homens [-22,8 (-35,9;6,2)] ( Tabela 10 -8).

•A Tabela 10 -9 apresenta o número de mortes, as taxas de mortalidade brutas e padronizadas por idade para as DCV atribuídas a IMC elevado no Brasil, em 1990 e 2019, por grupo etário, além da variação percentual das taxas no período, segundo dados do GBD 2019. Observa-se variação percentual de -28,5 (-38,8;-8,6) se considerarmos as taxas padronizadas para todas as idades no período.

## Carga de Doença

### Carga de Doença por Todas as Causas Atribuídas a IMC Elevado

•A Tabela 10 -10 mostra os DALY em números absolutos, suas taxas padronizadas por idade (por 100 mil habitantes) por todas as causas de morte atribuídas a IMC elevado, em 1990 e 2019, e a variação percentual das taxas no Brasil e UF (GBD 2019). Em relação à carga de doença no Brasil em 2019, a obesidade foi responsável por 177.939,7 (124.637,7;237.783) DALYs [76,2 (52,9;102,1) por 100 mil habitantes]. A variação percentual foi de -9,7 (-23,1;16,2) em relação a 1990, para o qual foram estimados 74.266,2 (43.491,7;110.056,9) DALYs [84,4 (48,1;127,9) por 100 mil]. As UF da região Norte [Maranhão 84,8 (25,5;250,7)] e Nordeste [Ceará 65,4 (19,9;172,1)] apresentaram as maiores variações percentuais positivas.

•A Tabela 10 -11 mostra o número de DALYs, suas taxas brutas e padronizadas por idade por todas as causas atribuídas a IMC elevado no Brasil, em 1990 e 2019, por grupo etário, além da variação percentual das taxas no período, segundo dados do GBD 2019. Observa-se que a maior variação percentual positiva ocorreu em crianças e adolescentes, de 5 a 14 anos [99,5 (53,1;158,3)].

•Nas Figuras 10-7 e 10-8, observamos a representação gráfica dos números absolutos e das taxas padronizadas por idade e por todas as idades dos YLLs, YLDs e DALYs atribuídos a IMC elevado, de 1990 a 2019, no Brasil, respectivamente. Nota-se tendência de crescimento de todos os números absolutos e das taxas brutas por 100 mil habitantes. Já as taxas padronizadas por idade dos DALYs e YLLs apresentaram tendência de queda no período, enquanto as de YLDs aumentaram, sugerindo um impacto crescente na morbidade causada pela obesidade mesmo independentemente do envelhecimento da população. Felisbino-Mendes *et al* . estimaram que os DALYs, para ambos os sexos, tiveram aumento de 96% e 42% pelo envelhecimento e crescimento da população, respectivamente, e aumento de 130% pela mudança de exposição ao risco. ^368^

•A Figura 10-9 mostra as taxas de DALYs padronizadas por idade para causas específicas atribuídas a IMC elevado, por 100 mil habitantes, nas UF, para todas as idades, de acordo com o sexo, segundo o GBD 2019. Observa-se que os DALYs variaram nas UF de forma diferente para os sexos. Diabetes e doenças renais foram as segundas maiores causas de DALYs atribuídos a IMC elevado, sendo precedidas pelas DCV.

As maiores taxas de DALYs padronizadas por idade por diabetes e doenças renais atribuídas a IMC elevado nas mulheres (por 100 mil habitantes) foram observadas em Alagoas (1.320,1), Roraima (1.250,8), Sergipe (1.253,6), Pernambuco e Maranhão (1.013,5), Paraíba (990,4), Rondônia (989,3) e Tocantins (981,7). Em relação aos homens, essas taxas (por 100 mil habitantes) foram maiores em Alagoas (1.263,9), Sergipe (1.168,1), Maranhão (1.130,8), Pernambuco (1.104,7), Bahia (1.086,2), Roraima (1.071,7), Paraíba (1.047,4), Rio de Janeiro (1.042,5) e Rio Grande do Norte (1.024,5).

### Carga de Doença Cardiovascular Atribuível a IMC Elevado

•Em relação à carga de doença em 2019, IMC elevado foi responsável por 177.939,7 (II 95%, 124.637 - 237.783) DALYs por DCV [76,2 (II 95%, 52,9 - 102,1) por 100 mil habitantes], com maior relevância para a doença isquêmica do coração [45.210,1 (II 95%, 29.102,5 - 63.084,1)] e AVC [35.124,7 (II 95%, 24.073,7 - 46.859,7)]. Esse fator de risco contribuiu, no Brasil, em 2019, para 5.817.938,7 (II 95%, 4.197.826,2 - 7.541.630) DALYs [2.404,5 (II 95%, 1.733,3 - 3.121,6) por 100 mil habitantes]. O percentual de redução foi de -6,4 (II 95%, -19.6 a 19.1) em relação a 1990, quando foram estimados 2.579.849,9 (II 95%, 1.556.675,2 - 3.720.770,6) DALYs [35.124,7 (II 95%, 24.073,7 - 46.859,7)] ( Figura 10-10 ).

•As Tabelas 10-12 e 10-13 mostram as taxas de DALYs por DCV atribuída a IMC elevado, padronizadas por idade (por 100 mil habitantes) e variação percentual das taxas, por sexo, no Brasil e UF, em 1990 e 2019 (GBD 2019). A maioria das UF apresentou decréscimo dos DALYs para as mulheres no período, que foi mais acentuado no Distrito Federal [-34,8 (-44,7;-20)], Rio de Janeiro [-28,6 (-39,4;-10,7)], São Paulo [-27,6 (-38,7;-9,2)], Santa Catarina [-22,8 (-34,8;-3,5)], Minas Gerais [-21,3 (-35,7;5,1)] e Rio Grande do Sul [-20,3 (-31,6;-0,6)]. O Maranhão apresentou o maior incremento entre 1990 e 2019 [95,8 (33,9;254,2].

•Comportamento semelhante foi observado em relação aos homens, com decréscimo de obesidade, no período de 1990 a 2019. Os maiores decréscimos percentuais foram observados no Distrito Federal [-25,8 (-39,8;-0,9)], Rio de Janeiro [-22,3 (-36,9;5,4)], São Paulo [-20,2 (-35;8,7)], Rio Grande do Sul [-19,5 (-32,3;4,3)], Santa Catarina [-11,5 (-28,3;22,1)] e Minas Gerais [-8,1 (-30,2;52,3)], menores do que os observados para as mulheres. O Ceará apresentou o maior incremento entre 1990 e 2019 [81,8 (25,1;231,1)]. Também os maiores aumentos percentuais foram observados para as mulheres no período ( Tabela 10 -13). Importante observar que, para o Brasil como um todo, observou-se o mesmo padrão, com maior decréscimo percentual para as mulheres [-12 (-23,8;8,9)] em relação aos homens [-0,1 (-16,5;36,8)].

•A Tabela 10 -14 mostra o número de DALYs, as taxas de DALYs brutas e padronizadas por idade por DCV atribuída a IMC elevado no Brasil, em 1990 e 2019, por grupo etário, além da variação percentual das taxas no período, segundo dados do GBD 2019. A maior variação percentual negativa ocorreu na faixa etária de 50 a 69 anos [-33,6 (-42,5;-16,5)]. Houve redução percentual de -31,2 (-40,5;-12,4) se considerarmos as taxas padronizadas por idade no período ( Tabela 10 -12).

## Impacto na Saúde Cardiovascular

•O IMC alto causa principalmente inflamação sistêmica crônica e maior atividade simpática, que pode contribuir para resistência a insulina e hipertensão, levando a disfunção endotelial e aterosclerose e contribuindo para diabetes mellitus. Seu efeito é mediado principalmente por outros fatores de risco intermediários, como hipertensão, hipercolesterolemia e hiperglicemia. ^385^ A obesidade tem natureza multifatorial e é um dos principais fatores para explicar o crescimento das DCNT dada a sua associação frequente com DCV, como hipertensão arterial, AVC, insuficiência cardíaca, dislipidemias, diabetes, fibrilação atrial e morte súbita. As intervenções que reduzem hipertensão, colesterol e glicose poderiam resolver cerca de metade do excesso de risco de doença coronária e três quartos do excesso de risco de AVC associado a alto IMC. ^386^

•Rimes-Dias e Canella aventaram a hipótese de que as DCNT associadas a obesidade geralmente requerem tratamento medicamentoso e realizaram estudo empregando a PNS de 2013, com 59.402 indivíduos com 18 anos ou mais. Avaliaram o número de medicamentos usados para tratar nove DCNT relacionadas a obesidade (hipertensão arterial, DCV, AVC, diabetes, artrite e reumatismo, doença renal crônica, doença pulmonar, lombalgia crônica e depressão). O uso de medicamentos aumentou progressivamente com o aumento do IMC. O risco de tratamento de duas ou mais DCNT com medicamentos foi cerca de 70% maior entre os indivíduos com sobrepeso (RR ajustado = 1,66; IC 95%, 1,46 - 1,89), 170% maior naqueles com obesidade grau I (RR ajustado = 2,68; IC 95%, 2,29 - 3,12), 340% maior na obesidade grau II (RR ajustado = 4,44; IC 95%, 3,54 - 5,56) e 450% maior na obesidade grau III (RR ajustado = 5,53; IC 95%, 3,81 - 8,02) em comparação a indivíduos com peso normal. ^387^

•Foi realizado estudo transversal analisando a presença de fatores de risco cardiovascular de acordo com o padrão de distribuição de gordura corporal em 113 brasileiros com 80 anos ou mais (média de idade, 83,4 anos), de ambos os sexos, recrutados entre 2009 e 2010 na cidade de Presidente Prudente, em São Paulo, como parte do estudo SABE. Os autores observaram a associação de obesidade abdominal e total com fatores de risco para DCV, como maiores níveis de colesterol total e triglicerídeos. Houve importante associação entre hipertensão arterial e obesidade total. ^388^

•Uma coorte com duração de 12 meses, avaliando 89 adolescentes da cidade de Presidente Prudente, com idade entre 11 e 14 anos, demonstrou, após ajustes para gênero, idade, maturação biológica e atividade física, que as mudanças na espessura médio-intimal femoral se associaram com alterações na gordura corporal: para cada aumento percentual na gordura corporal, a espessura médio-intimal femoral aumentou 0,007 mm. ^389^

## Fatores de Risco e Prevenção

•Rabacow *et al* . estimaram a proporção de morte por DCNT que poderia ser reduzida no Brasil através da diminuição do IMC, com dados da PNS de 2013. Redução populacional do IMC para um nível de mínimo risco teórico (22,0 kg/m^2^ ) poderia prevenir aproximadamente 168.431 mortes por ano no Brasil, sendo 106.307 por DCV. Já uma redução para os níveis de IMC de 24,6 kg/m^2^ poderia prevenir 65.721 mortes, representando 10% das mortes por DCNT e 5,8% de todas as mortes. Uma redução de 1,0 kg/m^2^ no IMC populacional poderia prevenir 30.715 mortes, representando 4,6% das mortes por DCNT e 2,7% de todas as mortes. O conjunto dos dados reforça a necessidade da adoção de medidas antiobesidade em nível nacional. ^390^

•Estudo que analisou a Pesquisa de Orçamentos Familiares 2002/2003 e 2008/2009 e a PNS de 2013, com 234.791 adultos de 20-59 anos, demonstrou que os níveis educacionais mais elevados foram associados a obesidade e excesso de peso para homens, enquanto o nível de ensino médio foi associado a aumento de obesidade nas mulheres. Os autores concluíram que os achados revelam a necessidade de educação nutricional e elaboração de campanhas e políticas para conter a epidemia de obesidade. ^391^

## Utilização e Custo da Atenção à Saúde

•Um estudo orçou os custos totais diretos, em um ano, associados com a assistência ambulatorial e hospitalar às doenças relacionadas com excesso de peso e obesidade, na perspectiva do SUS no Brasil, entre 2008 e 2010. Esses valores foram estimados em US$ 2,1 bilhões, sendo US$ 1,4 bilhão (68,4% dos custos totais) devido a internações e US$ 679 milhões devidos a procedimentos ambulatoriais. Usando o fator de risco atribuível à população, os autores avaliaram que aproximadamente 10% desses custos foram atribuíveis a sobrepeso e obesidade. Os custos para as mulheres foram maiores do que para os homens, por causa dos maiores gastos ambulatoriais (73,3% vs. 26,7%). ^392^ Outro estudo avaliou o custo direto total de sobrepeso/obesidade em 3,02% dos custos totais de hospitalização em homens e 5,83% em mulheres, correspondendo a 6,8% e 9,3% de todas as hospitalizações (excluindo gestantes), respectivamente. ^393^

•Um estudo analisou a custo-utilidade da cirurgia de *bypass* gástrico em comparação ao tratamento clínico para obesidade grave com e sem diabetes na perspectiva do SUS. O modelo de Markov considerou um horizonte de tempo de 10 anos e uma taxa de desconto de 5%. Ao longo de 10 anos, a cirurgia de *bypass* gástrico aumentou os QALYs e os custos em comparação ao tratamento clínico, resultando em uma RCEI de Int$ 1.820,17/QALY e Int$ 1.937,73/QALY em indivíduos com e sem diabetes, respectivamente. A análise de sensibilidade mostrou que os valores de utilidade e os custos diretos dos tratamentos foram os parâmetros que mais afetaram a RCEI. Os autores concluíram que a cirurgia de *bypass* gástrico é uma intervenção com boa relação custo-benefício para obesos graves na perspectiva do SUS, com melhor resultado em obesos com diabetes. ^394^

•Um estudo avaliou que os custos totais de hipertensão, diabetes e obesidade no SUS alcançaram R$ 3,45 bilhões (IC 95%, 3,15 - 3,75) em 2018, cerca de US$ 890 milhões. Para obesidade como fator de risco para hipertensão e diabetes, os custos atribuíveis chegaram a R$ 1,42 bilhão (IC 95%, 0,98 - 1,87), ou seja, 41% dos custos totais. ^395^

•Um modelo de micro simulação foi usado para projetar a extensão da obesidade, as doenças a ela relacionadas e os custos de saúde a ela associados no Brasil até 2050. No total, 13 doenças foram consideradas (doença cardíaca coronária, AVC, hipertensão, diabetes, osteoartrite e oito cânceres), simulando três cenários hipotéticos de intervenção: não intervenção, redução de 1% e de 5% no IMC. Os autores estimam que os custos com saúde dobrarão até 2050 (US$ 10,1 bilhões), alcançando US$ 330 bilhões no período de 2010 a 2050. Porém, com intervenções eficazes, como a redução do IMC médio na população em 1% e 5%, os custos podem ser reduzidos para US$ 302 bilhões e US$ 273 bilhões, respectivamente. ^396^

## Pesquisa Futura

•Observam-se lacunas nos dados primários de mortalidade atribuível a IMC elevado no Brasil e suas UF. Registros de âmbito nacional com dados aferidos devem ser realizados para que possamos desenvolver políticas públicas mais efetivas para o controle da obesidade, que vem aumentando no Brasil, em ambos os sexos e nas diversas faixas etárias.

•A maioria das políticas públicas falhou em reduzir a obesidade em adultos e crianças, provavelmente por ser uma condição multifatorial e envolver muitos interesses socioeconômicos. Cabe ressaltar o papel da indústria de alimentos, que inclui a oferta de alimentos ultraprocessados com menores custos, a ausência da comunicação dos riscos associados com o excesso de peso e obesidade nas mídias sociais, além do estilo de vida das grandes cidades, onde as crianças são cada vez mais sedentárias e têm dietas com alto teor calórico. Estudos futuros com intervenções multifatoriais envolvendo toda a família precisarão ser realizados, especialmente para beneficiar crianças e adolescentes. ^397^

•O cuidado centrado no paciente portador de obesidade demandará pesquisas futuras, sendo um componente essencial dos cuidados de saúde de alta qualidade que poderá melhorar os resultados clínicos e a satisfação do paciente, reduzindo os custos de saúde. Um estudo sugere os seguintes itens para os pesquisadores em obesidade: desenvolver agendas que enfatizem a pesquisa de ferramentas e técnicas de cuidado centrado no paciente; priorizar a análise das barreiras que impedem o paciente de alcançar a perda de peso e avaliar ferramentas que podem ser usadas para superar essas barreiras; avaliar os fatores culturais e ambientais que podem afetar a capacidade de perder peso; e estabelecer métricas baseadas em evidências para o paciente obeso. ^398^

•Dados mais recentes destacam a obesidade abdominal, avaliada pela circunferência da cintura, como um marcador de risco de DCV independente do IMC. Estudos que quantificam depósitos de gordura, incluindo gordura ectópica, com métodos de imagem, demonstraram que o excesso de adiposidade visceral foi indicador independente de desfechos cardiovasculares adversos. Estudos que avaliem a obesidade abdominal na população brasileira precisam ser realizados para investigar seu papel incremental na estratificação de risco cardiovascular em ambos os sexos e nos diversos grupos etários. ^399^

## 11. TABAGISMO E USO DE TABACO

### Tabagismo e suas consequências para as doenças cardiovasculares no Brasil e nas Unidades Federativas, 1990 a 2019

CID-10: Z.72.0

**Ver Tabelas 11-1 a 11-13 e Figuras 11-1 a 11-12**

**Table:** Abreviaturas Usadas no Capítulo 11

ANVISA	Agência Nacional de Vigilância Sanitária
AVC	Acidente Vascular Cerebral
DALYs	Anos de vida perdidos ajustados por incapacidade (do inglês, *Disability-Adjusted Life-Year* )
DCNT	Doenças Crônicas Não Transmissíveis
DCV	Doenças Cardiovasculares
ELSA-Brasil	Estudo Longitudinal de Saúde do Adulto - Brasil
ERICA	Estudo dos Riscos Cardiovasculares em Adolescentes
GBD	Global Burden of Disease
PeNSE	Pesquisa Nacional de Saúde do Escolar
PNS	Pesquisa Nacional de Saúde
SDI	Índice Sociodemográfico (do inglês, *Sociodemographic Index* )
UF	Unidade Federativa
Vigitel	Vigilância de Fatores de Risco e Proteção para Doenças Crônicas por Inquérito Telefônico
YLDs	Anos vividos com incapacidade (do inglês, *Years Lived with Disability* )
YLLs	Anos potenciais de vida perdidos (do inglês, *Years of Life Lost* )

**Tabela 11-1:** – Porcentagem de alunos do nono ano que já experimentaram cigarro, de acordo com o sexo e tipo de escola, no Brasil, suas regiões e unidades federativas.

Regiões e unidades federativas																	
	Total			Sexo							Tipo de escola						
				Masculino			Feminino				Pública				Privada		
	Total	II 95%		Total	II 95%		Total	II 95%			Total	II 95%			Total	II 95%	
		Limite inferior	Limite superior		Limite inferior	Limite superior		Limite inferior	Limite superior			Limite inferior		Limite superior		Limite inferior	Limite superior
Brasil	18,4	17,8	19,0	19,4	18,7	20,2	17,4	16,6		18,2	19,4		18,7	20,0	12,6	11,6	13,6
Norte	20,1	18,8	21,3	22,7	21,1	24,3	17,6	16,2		18,9	20,8		19,5	22,2	11,8	8,9	14,6
Rondônia	21,5	18,9	24,2	22,4	18,8	26,0	20,7	18,0		23,4	21,9		19,1	24,7	16,1	11,6	20,7
Acre	26,2	23,7	28,6	28,3	25,6	31,1	24,0	21,0		27,0	27,1		24,5	29,6	12,1	5,8	18,4
Amazonas	21,1	19,1	23,0	23,3	20,8	25,7	18,9	16,7		21,1	21,9		19,9	24,0	9,5	3,2	15,7
Roraima	28,2	25,3	31,2	30,6	27,5	33,7	25,8	21,9		29,6	29,2		26,1	32,3	12,4	1,5	23,3
Pará	18,7	16,3	21,1	22,3	19,1	25,5	15,5	13,0		18,1	19,4		16,9	22,0	12,3	7,7	16,9
Amapá	21,5	19,5	23,6	22,4	20,2	24,7	20,7	18,1		23,2	22,5		20,3	24,6	12,5	7,3	17,8
Tocantins	15,1	12,1	18,1	17,1	13,2	21,1	13,1	10,4		15,8	15,6		12,3	18,9	8,6	5,2	12,1
Nordeste	14,2	13,5	14,9	16,3	15,4	17,3	12,4	11,6		13,3	15,1		14,3	15,9	9,3	8,3	10,4
Maranhão	11,9	10,6	13,2	15,4	13,4	17,5	9,0	7,5		10,4	12,2		10,8	13,6	8,8	6,0	11,6
Piauí	12,8	11,3	14,3	15,5	13,1	17,8	10,5	8,8		12,2	13,3		11,7	15,0	9,4	6,4	12,5
Ceará	18,7	16,5	20,9	19,6	17,1	22,2	17,7	14,9		20,5	19,9		17,4	22,4	11,7	8,8	14,6
Rio Grande do Norte	10,9	9,6	12,1	13,3	11,3	15,3	8,7	7,3		10,2	11,7		10,3	13,2	7,1	5,1	9,1
Paraíba	15,3	13,6	17,1	16,6	14,4	18,8	14,3	12,2		16,4	16,4		14,3	18,5	10,5	8,7	12,4
Pernambuco	14,0	12,4	15,5	14,7	12,6	16,8	13,2	11,2		15,3	15,0		13,1	16,9	9,3	7,4	11,2
Alagoas	13,0	10,9	15,0	13,6	11,5	15,8	12,4	9,7		15,1	13,5		11,1	15,9	11,0	7,6	14,3
Sergipe	9,3	8,1	10,5	11,4	9,5	13,2	7,7	6,5		8,9	9,9		8,5	11,2	7,3	4,8	9,8
Bahia	14,6	12,7	16,4	17,8	15,2	20,5	12,0	9,8		14,2	15,6		13,5	17,7	8,0	4,8	11,2
Sudeste	18,3	17,1	19,4	18,2	16,7	19,6	18,4	16,7		20,0	19,2		17,9	20,5	13,5	11,6	15,4
Minas Gerais	17,6	15,5	19,6	17,4	15,2	19,6	17,7	15,2		20,3	18,5		16,3	20,7	9,1	4,9	13,3
Espírito Santo	17,6	15,5	19,7	18,3	15,7	20,9	16,9	14,5		19,4	18,3		15,9	20,6	13,4	9,9	16,9
Rio de Janeiro	16,6	14,8	18,3	15,3	13,2	17,4	17,7	15,4		20,1	18,0		15,8	20,2	13,0	10,2	15,7
São Paulo	19,2	17,4	21,1	19,5	17,1	21,9	19,0	16,3		21,7	20,0		17,9	22,1	15,1	12,0	18,3
Sul	24,9	23,5	26,3	25,2	23,4	27,0	24,6	22,7		26,4	26,0		24,5	27,5	14,9	12,2	17,7
Paraná	25,5	23,2	27,7	26,4	23,8	29,0	24,4	21,4		27,4	27,4		24,9	30,0	12,0	8,2	15,8
Santa Catarina	22,1	19,5	24,6	24,2	20,9	27,6	20,2	16,9		23,4	22,8		20,0	25,6	16,2	11,9	20,6
Rio Grande do Sul	26,4	24,0	28,8	24,1	20,3	27,9	28,7	25,4		31,9	26,7		24,2	29,1	22,2	15,3	29,2
Centro-Oeste	22,1	20,9	23,2	24,2	22,6	25,7	20,0	18,6		21,4	22,9		21,6	24,1	17,5	15,5	19,5
Mato Grosso do Sul	27,0	24,9	29,2	29,4	26,7	32,1	24,7	21,9		27,6	28,2		25,8	30,6	15,5	13,4	17,6
Mato Grosso	23,2	21,0	25,4	25,6	22,2	29,1	20,7	18,0		23,3	23,9		21,5	26,2	15,5	10,7	20,4
Goiás	18,8	17,1	20,5	20,2	18,2	22,2	17,4	15,0		19,8	19,1		17,2	21,0	17,1	13,9	20,3
Distrito Federal	23,7	20,8	26,6	26,8	22,6	31,0	20,9	18,0		23,9	25,2		21,6	28,7	19,4	15,7	23,0

*Fonte: PeNSE 2015. ^410^*

**Tabela 11-2:** – Porcentagem de alunos do nono ano que fumaram nos 30 dias que antecederam à pesquisa, de acordo com o sexo e tipo de escola, no Brasil, suas regiões e unidades federativas.

Regiões e unidades federativas	Total			Sexo						Tipo de escola					
				Masculino			Feminino			Pública			Privada		
	Total	IC 95%		Total	IC 95%		Total	IC 95%		Total	IC 95%		Total	IC 95%	
		Limite inferior	Limite superior		Limite inferior	Limite superior		Limite inferior	Limite superior		Limite inferior	Limite superior		Limite inferior	Limite superior
Brasil	5,6	5,3	5,9	5,8	5,4	6,3	5,4	4,9	5,8	5,9	5,5	6,3	3,6	3,0	4,3
Norte	6,1	5,4	6,7	7,1	6,2	8,0	5,1	4,4	5,8	6,3	5,6	7,0	3,7	2,0	5,4
Rondônia	5,3	4,2	6,4	5,8	4,1	7,6	4,9	3,7	6,0	5,5	4,3	6,6	3,2	0,7	5,8
Acre	7,1	6,1	8,1	8,2	6,8	9,7	6,0	4,6	7,3	7,4	6,4	8,5	2,4	0,0	4,8
Amazonas	6,6	5,3	7,9	7,7	6,0	9,3	5,5	4,0	7,0	6,8	5,4	8,2	3,6	0,0	7,2
Roraima	10,4	8,4	12,4	12,1	10,0	14,2	8,6	6,3	10,9	10,8	8,7	12,9	3,9	0,0	9,0
Pará	5,5	4,3	6,7	6,5	4,8	8,3	4,6	3,3	5,9	5,7	4,3	7,0	4,2	1,4	7,0
Amapá	7,7	6,6	8,8	8,2	6,9	9,6	7,2	5,8	8,7	8,0	6,9	9,2	4,8	1,1	8,6
Tocantins	4,9	3,5	6,3	5,7	3,8	7,6	4,1	2,8	5,4	5,2	3,7	6,7	1,0	0,4	1,6
Nordeste	4,0	3,6	4,3	4,5	4,0	5,0	3,5	3,1	4,0	4,3	3,8	4,7	2,2	1,8	2,7
Maranhão	3,2	2,5	3,8	3,7	2,6	4,8	2,7	1,9	3,5	3,2	2,5	3,9	2,9	1,4	4,3
Piauí	3,2	2,6	3,9	4,0	2,9	5,1	2,5	1,8	3,2	3,4	2,7	4,2	1,6	0,5	2,8
Ceará	5,9	4,8	7,1	5,8	4,4	7,2	6,1	4,6	7,6	6,4	5,0	7,7	3,5	1,9	5,1
Rio Grande do Norte	1,9	1,4	2,3	2,2	1,5	3,0	1,6	1,1	2,1	2,1	1,6	2,7	0,9	0,3	1,5
Paraíba	4,2	3,3	5,0	5,2	4,0	6,4	3,4	2,4	4,3	4,4	3,4	5,4	3,3	1,8	4,8
Pernambuco	4,2	3,3	5,0	4,4	3,3	5,5	3,9	2,7	5,1	4,6	3,5	5,7	2,1	1,2	3,0
Alagoas	3,6	2,6	4,6	3,3	2,3	4,3	3,9	2,7	5,1	3,6	2,5	4,7	3,6	2,2	5,0
Sergipe	1,6	1,1	2,0	1,8	1,2	2,4	1,4	0,8	2,0	1,7	1,2	2,2	1,1	0,4	1,9
Bahia	4,0	3,0	4,9	5,2	3,6	6,8	3,1	2,0	4,2	4,4	3,3	5,5	1,2	0,4	2,1
Sudeste	6,0	5,3	6,7	5,8	4,9	6,8	6,2	5,2	7,2	6,4	5,6	7,2	4,1	2,9	5,3
Minas Gerais	4,7	3,7	5,8	4,9	3,5	6,3	4,5	3,4	5,7	4,9	3,8	6,1	2,8	0,8	4,8
Espírito Santo	5,0	3,6	6,3	5,2	3,7	6,7	4,7	3,2	6,3	4,9	3,4	6,4	5,4	2,6	8,2
Rio de Janeiro	5,5	4,5	6,5	5,0	3,9	6,0	6,1	4,6	7,5	6,1	4,8	7,4	4,2	3,1	5,3
São Paulo	6,9	5,7	8,1	6,6	5,0	8,2	7,2	5,4	9,0	7,4	6,0	8,7	4,4	2,2	6,5
Sul	7,0	6,2	7,7	7,0	5,9	8,1	6,9	5,9	7,9	7,2	6,3	8,1	4,8	3,1	6,5
Paraná	7,8	6,3	9,2	8,0	6,1	9,8	7,6	5,7	9,5	8,3	6,7	10,0	3,9	1,3	6,6
Santa Catarina	5,4	4,3	6,6	5,4	3,8	6,9	5,4	3,9	7,0	5,4	4,1	6,6	5,8	3,0	8,5
Rio Grande do Sul	7,1	5,8	8,3	6,8	4,8	8,9	7,3	5,7	8,8	7,1	5,8	8,4	5,9	3,9	7,9
Centro-Oeste	6,5	6,0	7,1	7,2	6,5	8,0	5,8	5,2	6,5	6,9	6,3	7,5	4,5	3,4	5,6
Mato Grosso do Sul	8,2	6,9	9,5	8,6	6,7	10,4	7,8	6,1	9,5	8,7	7,3	10,2	3,0	1,1	4,8
Mato Grosso	7,6	6,2	8,9	7,9	5,9	9,9	7,2	5,7	8,8	7,9	6,4	9,3	4,1	0,2	8,1
Goiás	5,6	4,8	6,3	6,4	5,4	7,4	4,7	3,6	5,8	5,8	4,9	6,6	4,3	2,9	5,8
Distrito Federal	6,1	4,9	7,4	7,3	5,2	9,3	5,1	3,9	6,3	6,4	4,9	7,9	5,3	3,4	7,3

*Fonte: PeNSE 2015. ^410^*

**Tabela 11-3:** – Porcentagem de alunos do nono ano que usaram outros derivados do tabaco nos 30 dias que antecederam à pesquisa, de acordo com o sexo e tipo de escola, no Brasil, suas regiões e unidades federativas.

Regiões e unidades federativas	Total			Sexo						Tipo de escola					
				Masculino			Feminino			Pública			Privada		
	Total	IC 95%		Total	IC 95%		Total	IC 95%		Total	IC 95%		Total	IC 95%	
		Limite inferior	Limite superior		Limite inferior	Limite superior		Limite inferior	Limite superior		Limite inferior	Limite superior		Limite inferior	Limite superior
Brasil	6,1	5,7	6,4	6,5	6,1	7,0	5,6	5,1	6,0	6,2	5,8	6,6	5,2	4,5	5,9
Norte	3,0	2,6	3,4	3,7	3,1	4,2	2,4	2,0	2,8	3,0	2,6	3,4	3,1	1,8	4,3
Rondônia	5,4	4,1	6,8	4,7	3,3	6,1	6,1	4,4	7,9	5,4	3,9	6,8	6,3	2,7	10,0
Acre	5,0	4,0	5,9	6,3	4,7	7,9	3,6	2,5	4,7	5,1	4,1	6,1	3,4	1,1	5,8
Amazonas	2,8	2,1	3,5	3,4	2,4	4,4	2,3	1,5	3,0	2,9	2,2	3,6	1,5	0,2	2,7
Roraima	5,2	4,0	6,3	6,4	4,9	7,8	4,0	2,6	5,3	5,1	4,0	6,1	6,8	0,0	15,3
Pará	2,0	1,4	2,7	2,9	1,9	3,9	1,3	0,7	1,9	1,9	1,3	2,6	3,1	0,9	5,2
Amapá	2,7	2,1	3,3	3,3	2,5	4,2	2,2	1,4	2,9	2,7	2,1	3,4	2,6	1,3	3,9
Tocantins	4,1	2,8	5,4	4,8	2,9	6,8	3,4	2,3	4,6	4,2	2,8	5,6	3,2	0,3	6,2
Nordeste	2,3	2,0	2,6	2,9	2,5	3,3	1,8	1,5	2,1	2,3	2,0	2,7	2,1	1,7	2,5
Maranhão	1,7	1,2	2,1	2,1	1,4	2,8	1,4	0,8	1,9	1,6	1,1	2,1	2,6	1,0	4,3
Piauí	1,9	1,4	2,5	2,4	1,6	3,2	1,6	0,9	2,3	2,0	1,4	2,5	1,6	0,0	3,8
Ceará	3,0	1,9	4,0	3,1	2,1	4,2	2,8	1,6	4,1	3,1	1,9	4,3	2,3	1,1	3,5
Rio Grande do Norte	1,5	1,0	1,9	1,8	1,1	2,6	1,2	0,7	1,6	1,5	1,0	2,1	1,2	0,4	2,0
Paraíba	2,4	1,8	2,9	3,7	2,7	4,6	1,3	0,8	1,8	2,3	1,7	3,0	2,6	1,5	3,7
Pernambuco	2,3	1,7	2,9	2,9	2,1	3,7	1,6	1,0	2,3	2,4	1,7	3,1	1,7	1,0	2,3
Alagoas	2,0	1,4	2,6	2,3	1,6	3,0	1,7	0,9	2,5	1,8	1,2	2,4	2,7	0,9	4,5
Sergipe	1,5	1,1	1,9	2,2	1,5	3,0	0,9	0,5	1,3	1,6	1,1	2,0	1,2	0,6	1,8
Bahia	2,6	1,8	3,4	3,5	2,3	4,8	1,9	1,2	2,6	2,7	1,8	3,5	2,3	1,6	3,0
Sudeste	7,5	6,8	8,2	7,8	7,0	8,7	7,2	6,2	8,1	7,8	7,0	8,6	6,0	4,8	7,3
Minas Gerais	4,6	3,8	5,5	5,4	4,2	6,6	3,9	2,7	5,1	4,8	3,9	5,8	2,9	1,6	4,2
Espírito Santo	3,3	2,5	4,1	3,6	2,5	4,6	3,0	2,0	4,0	3,1	2,3	4,0	4,2	2,3	6,0
Rio de Janeiro	4,5	3,7	5,3	4,8	3,9	5,7	4,2	3,1	5,3	4,7	3,7	5,7	3,9	2,6	5,2
São Paulo	10,2	9,0	11,4	10,3	8,9	11,7	10,2	8,6	11,8	10,6	9,3	12,0	8,3	6,0	10,6
Sul	9,6	8,5	10,6	9,6	8,4	10,8	9,6	8,2	11,0	9,8	8,7	11,0	7,7	5,5	9,9
Paraná	13,8	11,9	15,8	13,8	11,6	16,1	13,8	11,3	16,4	14,5	12,3	16,7	9,3	5,6	13,0
Santa Catarina	8,3	6,2	10,5	8,1	6,0	10,1	8,6	5,7	11,5	8,6	6,2	11,0	6,5	3,2	9,8
Rio Grande do Sul	4,6	3,6	5,6	4,6	3,2	6,0	4,6	3,0	6,1	4,6	3,6	5,6	4,6	2,6	6,6
Centro-Oeste	10,0	9,2	10,8	10,4	9,4	11,4	9,6	8,6	10,6	10,0	9,1	10,8	10,2	8,2	12,1
Mato Grosso do Sul	13,9	12,1	15,7	15,1	12,8	17,3	12,8	10,7	14,9	14,1	12,2	16,0	12,1	6,2	18,1
Mato Grosso	9,4	7,9	10,8	8,3	6,3	10,3	10,5	8,6	12,4	9,4	7,9	11,0	8,6	3,7	13,5
Goiás	7,8	6,6	9,0	8,4	7,0	9,8	7,2	5,6	8,7	7,5	6,2	8,9	9,3	7,1	11,5
Distrito Federal	12,2	10,2	14,2	13,6	11,0	16,2	10,9	8,7	13,2	12,5	10,3	14,7	11,4	7,1	15,7

*Fonte: PeNSE 2015. ^410^*

**Tabela 11-4:** – Proporção de usuários atuais de derivados do tabaco a partir dos 18 anos de idade, de acordo com o sexo, no Brasil, suas regiões e unidades federativas, e áreas urbana e rural.

Regiões, unidades federativas e áreas urbana e rural	Total			Sexo					
				Masculino			Feminino		
	Proporção	IC 95%		Proporção	IC 95%		Proporção	IC 95%	
		Limite inferior	Limite superior		Limite inferior	Limite superior		Limite inferior	Limite superior
Brasil	12,8	12,4	13,2	16,2	15,6	16,9	9,8	9,3	10,3
Urbana	12,6	12,1	13,0	15,8	15,0	16,5	9,9	9,4	10,4
Rural	14,3	13,4	15,2	18,6	17,4	19,9	9,3	8,3	10,2
Norte	10,7	9,9	11,5	15,5	14,2	16,8	6,2	5,4	7,1
Rondônia	10,8	9,2	12,4	16,6	14,1	19,1	5,2	3,4	7,1
Acre	15,1	13,2	17,1	19,0	15,7	22,2	11,5	9,2	13,9
Amazonas	10,2	9,0	11,4	16,0	13,6	18,4	4,8	3,6	5,9
Roraima	11,6	9,8	13,3	16,5	13,6	19,4	6,8	5,0	8,6
Pará	10,1	8,6	11,5	14,3	12,0	16,6	6,1	4,5	7,8
Amapá	10,9	8,8	12,9	15,0	11,3	18,6	7,0	4,9	9,2
Tocantins	12,8	10,7	14,9	17,6	14,0	21,1	8,1	5,9	10,3
Nordeste	11,1	10,5	11,7	14,7	13,7	15,6	7,9	7,3	8,6
Maranhão	11,3	10,2	12,4	16,0	14,1	17,9	7,0	5,9	8,2
Piauí	11,7	10,3	13,2	15,9	13,4	18,4	7,9	5,8	10,0
Ceará	12,2	11,1	13,4	15,4	13,3	17,4	9,5	8,1	10,9
Rio Grande do Norte	11,3	9,6	13,1	15,9	12,9	18,8	7,4	5,8	9,1
Paraíba	11,8	10,1	13,4	14,5	11,8	17,2	9,4	7,8	11,0
Pernambuco	11,3	9,6	13,0	14,7	11,3	18,2	8,5	7,1	9,9
Alagoas	10,6	9,3	12,0	13,9	11,6	16,1	7,9	6,6	9,2
Sergipe	9,4	8,2	10,6	11,6	9,5	13,7	7,5	5,9	9,0
Bahia	10,1	8,4	11,7	13,7	11,3	16,0	6,8	5,1	8,5
Sudeste	13,5	12,7	14,3	16,8	15,5	18,1	10,6	9,7	11,5
Minas Gerais	13,2	11,8	14,6	17,1	14,8	19,4	9,7	8,2	11,3
Espírito Santo	10,4	9,1	11,6	14,0	11,6	16,3	7,1	5,7	8,6
Rio de Janeiro	12,1	10,9	13,3	14,6	12,8	16,5	10,0	8,6	11,5
São Paulo	14,4	13,1	15,7	17,7	15,7	19,7	11,5	10,0	12,9
Sul	14,7	13,8	15,7	17,1	15,7	18,5	12,6	11,4	13,8
Paraná	14,7	13,0	16,4	17,3	14,9	19,7	12,3	10,2	14,5
Santa Catarina	13,1	11,6	14,6	15,7	13,5	18,0	10,6	8,8	12,4
Rio Grande do Sul	15,8	14,3	17,4	17,8	15,3	20,2	14,1	12,2	16,1
Centro-Oeste	13,7	12,7	14,7	17,6	15,9	19,2	10,2	9,1	11,3
Mato Grosso do Sul	16,3	14,5	18,2	20,8	17,9	23,7	12,4	10,5	14,3
Mato Grosso	13,0	11,3	14,8	16,3	13,2	19,3	10,0	7,9	12,1
Goiás	13,9	12,1	15,8	18,1	15,0	21,2	10,1	8,1	12,1
Distrito Federal	11,6	9,9	13,4	15,1	12,3	17,9	8,7	6,6	10,8

*Fonte: IBGE, Diretoria de Pesquisas, Coordenação de Trabalho e Rendimento, Pesquisa Nacional de Saúde 2019. ^306^*

**Tabela 11-5:** – Proporção de usuários atuais de tabaco a partir dos 18 anos de idade, de acordo com o sexo, no Brasil, suas regiões e unidades federativas, e áreas urbana e rural.

Regiões, unidades federativas e áreas urbana e rural	Total			Sexo					
				Masculino			Feminino		
	Proporção	IC 95%		Proporção	IC 95%		Proporção	IC 95%	
		Limite inferior	Limite superior		Limite inferior	Limite superior		Limite inferior	Limite superior
Brasil	12,6	12,2	13,0	15,9	15,3	16,6	9,6	9,2	10,1
Urbana	12,4	12,0	12,9	15,6	14,8	16,3	9,8	9,2	10,3
Rural	13,7	12,8	14,6	17,9	16,6	19,1	8,9	7,9	9,9
Norte	10,5	9,7	11,3	15,2	13,9	16,5	6,1	5,2	7,0
Rondônia	10,4	8,8	12,0	15,8	13,3	18,3	5,2	3,4	7,0
Acre	13,9	12,0	15,7	17,0	13,7	20,2	10,9	8,7	13,2
Amazonas	10,2	8,9	11,4	15,9	13,5	18,3	4,7	3,6	5,9
Roraima	11,4	9,7	13,2	16,2	13,3	19,1	6,8	5,0	8,6
Pará	9,8	8,4	11,3	14,1	11,8	16,4	5,9	4,3	7,6
Amapá	10,9	8,8	12,9	15,0	11,3	18,6	7,0	4,9	9,2
Tocantins	12,6	10,4	14,7	17,4	13,8	21,0	7,9	5,6	10,1
Nordeste	10,8	10,2	11,4	14,2	13,3	15,2	7,7	7,1	8,3
Maranhão	11,0	9,9	12,1	15,7	13,9	17,6	6,8	5,6	8,0
Piauí	11,0	9,5	12,5	15,2	12,7	17,7	7,2	5,3	9,1
Ceará	11,6	10,4	12,7	14,5	12,5	16,4	9,0	7,7	10,4
Rio Grande do Norte	11,0	9,2	12,7	15,1	12,2	18,0	7,4	5,8	9,1
Paraíba	11,7	10,0	13,3	14,3	11,6	17,0	9,4	7,8	11,0
Pernambuco	11,2	9,6	12,9	14,7	11,2	18,1	8,5	7,0	9,9
Alagoas	10,6	9,3	11,9	13,8	11,6	16,1	7,9	6,6	9,2
Sergipe	9,2	8,0	10,5	11,4	9,3	13,5	7,3	5,8	8,9
Bahia	9,8	8,2	11,4	13,3	11,0	15,6	6,6	5,0	8,3
Sudeste	13,3	12,5	14,1	16,6	15,3	17,8	10,4	9,5	11,3
Minas Gerais	12,7	11,3	14,1	16,4	14,1	18,7	9,4	7,9	10,9
Espírito Santo	10,2	8,9	11,5	13,9	11,5	16,2	6,9	5,4	8,4
Rio de Janeiro	12,0	10,8	13,2	14,6	12,8	16,5	9,9	8,4	11,4
São Paulo	14,3	13,0	15,6	17,6	15,6	19,6	11,4	10,0	12,8
Sul	14,7	13,7	15,6	17,0	15,6	18,4	12,5	11,4	13,7
Paraná	14,6	12,9	16,3	17,2	14,9	19,6	12,2	10,1	14,3
Santa Catarina	13,0	11,5	14,5	15,6	13,4	17,9	10,6	8,8	12,4
Rio Grande do Sul	15,8	14,2	17,3	17,8	15,3	20,2	14,0	12,1	16,0
Centro-Oeste	13,1	12,1	14,1	16,5	14,9	18,1	10,0	8,9	11,1
Mato Grosso do Sul	14,9	13,2	16,7	18,0	15,4	20,7	12,2	10,3	14,1
Mato Grosso	12,9	11,2	14,6	16,0	13,0	19,0	9,9	7,9	12,0
Goiás	13,4	11,6	15,2	17,3	14,3	20,2	9,9	7,9	11,9
Distrito Federal	11,0	9,3	12,8	14,0	11,2	16,8	8,5	6,4	10,6

*Fonte: IBGE, Diretoria de Pesquisas, Coordenação de Trabalho e Rendimento, Pesquisa Nacional de Saúde 2019. ^306^*

**Tabela 11-6:** – Proporção de usuários atuais de tabaco a partir dos 18 anos de idade, de acordo com o grupo etário, no Brasil, suas regiões e unidades federativas, e áreas urbana e rural.

Regiões, unidades federativas e áreas urbana e rural	Total			Grupos etários											
				18 – 24 anos			25 - 39 anos			40 - 59 anos			≥ 60 anos		
	Proporção	IC 95%		Proporção	IC 95%		Proporção	IC 95%		Proporção	IC 95%		Proporção	IC 95%	
		Limite inferior	Limite superior		Limite inferior	Limite superior		Limite inferior	Limite superior		Limite inferior	Limite superior		Limite inferior	Limite superior
Brasil	12,8	12,4	13,2	10,8	9,6	12,0	12,0	11,2	12,7	14,9	14,2	15,6	11,9	11,2	12,6
Urbana	12,6	12,1	13,0	11,1	9,7	12,4	12,0	11,2	12,9	14,5	13,7	15,3	11,1	10,4	11,9
Rural	14,3	13,4	15,2	9,0	6,9	11,0	11,6	10,1	13,1	17,5	15,9	19,0	16,2	14,6	17,9
Norte	10,7	9,9	11,5	8,0	6,3	9,6	11,1	9,7	12,5	12,2	10,8	13,7	9,9	8,4	11,3
Rondônia	10,8	9,2	12,4	12,6	7,9	17,3	10,5	7,8	13,1	11,7	9,0	14,3	7,9	5,1	10,7
Acre	15,1	13,2	17,1	14,1	9,9	18,3	11,4	8,5	14,3	18,3	15,0	21,7	17,3	13,3	21,3
Amazonas	10,2	9,0	11,4	7,4	4,6	10,1	12,0	9,4	14,5	11,1	8,8	13,4	8,0	5,6	10,3
Roraima	11,6	9,8	13,3	9,1	5,1	13,0	12,8	10,0	15,7	13,2	10,0	16,4	7,9	5,0	10,9
Pará	10,1	8,6	11,5	6,5	3,6	9,5	10,8	8,3	13,4	11,4	8,8	14,0	9,6	7,0	12,2
Amapá	10,9	8,8	12,9	9,2	5,9	12,5	9,2	5,0	13,3	14,1	10,1	18,1	10,1	6,2	14,0
Tocantins	12,8	10,7	14,9	8,0	3,9	12,1	11,7	8,3	15,1	15,1	11,0	19,2	14,1	10,1	18,1
Nordeste	11,1	10,5	11,7	7,5	6,2	8,9	9,0	8,1	9,8	13,4	12,2	14,6	12,8	11,7	13,9
Maranhão	11,3	10,2	12,4	7,9	5,3	10,6	10,2	8,4	12,0	13,3	11,3	15,4	12,6	10,2	15,1
Piauí	11,7	10,3	13,2	7,7	3,3	12,1	10,4	7,6	13,1	12,6	9,7	15,4	14,9	11,3	18,4
Ceará	12,2	11,1	13,4	5,3	2,8	7,9	10,5	8,3	12,6	14,1	12,0	16,2	16,5	13,9	19,2
Rio Grande do Norte	11,3	9,6	13,1	10,2	4,7	15,7	8,0	5,4	10,6	14,3	11,5	17,1	12,3	9,4	15,2
Paraíba	11,8	10,1	13,4	6,9	3,8	10,0	8,8	6,4	11,1	15,7	12,6	18,8	13,0	9,7	16,3
Pernambuco	11,3	9,6	13,0	9,5	6,3	12,7	10,4	7,9	12,8	13,0	9,9	16,2	10,9	8,6	13,2
Alagoas	10,6	9,3	12,0	8,1	4,5	11,7	8,3	6,1	10,4	13,2	10,5	15,8	11,7	8,9	14,6
Sergipe	9,4	8,2	10,6	5,2	1,7	8,7	8,2	6,0	10,4	12,0	9,4	14,6	10,0	7,1	13,0
Bahia	10,1	8,4	11,7	7,2	3,3	11,0	6,8	4,7	8,9	12,9	9,6	16,2	11,8	8,8	14,9
Sudeste	13,5	12,7	14,3	12,8	10,2	15,3	12,8	11,3	14,2	15,6	14,3	16,8	11,5	10,2	12,8
Minas Gerais	13,2	11,8	14,6	11,7	7,1	16,3	11,7	9,6	13,8	15,2	12,8	17,6	12,7	10,3	15,0
Espírito Santo	10,4	9,1	11,6	8,2	3,7	12,7	10,0	7,7	12,4	12,2	10,2	14,1	9,2	6,8	11,6
Rio de Janeiro	12,1	10,9	13,3	10,2	6,7	13,6	12,8	10,4	15,2	13,2	11,4	15,0	10,8	8,8	12,8
São Paulo	14,4	13,1	15,7	14,6	10,4	18,7	13,5	11,0	15,9	16,9	14,8	18,9	11,4	9,3	13,5
Sul	14,7	13,8	15,7	12,6	9,7	15,5	15,2	13,3	17,2	16,5	15,0	18,1	12,4	11,1	13,8
Paraná	14,7	13,0	16,4	14,3	9,4	19,1	16,2	12,6	19,8	15,4	12,4	18,3	11,7	9,3	14,2
Santa Catarina	13,1	11,6	14,6	9,7	5,1	14,3	13,0	10,3	15,7	15,2	12,7	17,7	11,7	9,1	14,2
Rio Grande do Sul	15,8	14,3	17,4	12,5	7,3	17,6	15,7	12,4	19,1	18,6	16,1	21,1	13,5	11,3	15,6
Centro-Oeste	13,7	12,7	14,7	12,7	10,0	15,3	13,3	11,6	15,0	15,4	13,6	17,3	11,7	9,9	13,5
Mato Grosso do Sul	16,3	14,5	18,2	17,3	12,3	22,3	16,6	13,3	19,8	17,8	15,0	20,6	12,8	9,6	16,0
Mato Grosso	13,0	11,3	14,8	13,5	7,6	19,5	14,0	10,6	17,4	14,3	10,9	17,7	7,9	5,2	10,5
Goiás	13,9	12,1	15,8	11,2	6,6	15,8	12,3	9,4	15,2	16,6	13,0	20,2	13,3	10,1	16,6
Distrito Federal	11,6	9,9	13,4	11,3	6,9	15,8	11,9	8,7	15,0	12,1	9,2	15,0	10,5	6,9	14,1

*Fonte: IBGE, Diretoria de Pesquisas, Coordenação de Trabalho e Rendimento, Pesquisa Nacional de Saúde 2019. ^306^*

**Tabela 11-7:** – Proporção de usuários atuais de tabaco a partir dos 18 anos de idade, de acordo com o nível educacional, no Brasil, suas regiões e unidades federativas, e áreas urbana e rural.

Regiões, unidades federativas e áreas urbana e rural	Total			Nível educacional											
				Sem instrução e fundamental incompleto			Fundamental completo e médio incompleto			Médio completo e superior incompleto			Superior completo		
	Proporção	IC 95%		Proporção	IC 95%		Proporção	IC 95%		Proporção	IC 95%		Proporção	IC 95%	
		Limite inferior	Limite superior		Limite inferior	Limite superior		Limite inferior	Limite superior		Limite inferior	Limite superior		Limite inferior	Limite superior
Brasil	12,8	12,4	13,2	17,6	16,8	18,4	15,5	14,3	16,6	9,6	8,9	10,2	7,1	6,3	7,8
Urbana	12,6	12,1	13,0	17,4	16,5	18,3	16,2	14,8	17,5	9,9	9,2	10,5	7,1	6,4	7,9
Rural	14,3	13,4	15,2	18,2	17,0	19,5	11,2	9,3	13,1	5,5	4,5	6,6	4,4	2,4	6,3
Norte	10,7	9,9	11,5	16,7	15,1	18,4	11,0	9,1	12,9	6,4	5,4	7,4	4,0	2,7	5,2
Rondônia	10,8	9,2	12,4	15,3	12,6	18,1	14,0	9,4	18,6	5,6	3,3	7,9	4,7	1,5	7,8
Acre	15,1	13,2	17,1	25,5	21,9	29,1	14,3	9,8	18,7	8,6	5,9	11,4	4,4	1,7	7,0
Amazonas	10,2	9,0	11,4	16,0	13,5	18,6	11,7	8,2	15,1	6,7	4,7	8,6	4,7	2,1	7,3
Roraima	11,6	9,8	13,3	18,9	15,0	22,8	16,5	10,5	22,4	8,2	6,2	10,2	5,2	2,7	7,6
Pará	10,1	8,6	11,5	15,8	12,9	18,7	9,6	6,5	12,6	5,4	3,7	7,2	3,0	0,6	5,4
Amapá	10,9	8,8	12,9	15,1	11,2	19,0	10,0	5,4	14,7	10,1	6,9	13,3	5,3	1,4	9,2
Tocantins	12,8	10,7	14,9	20,4	16,6	24,3	12,4	6,5	18,3	7,3	4,6	10,0	4,0	1,5	6,4
Nordeste	11,1	10,5	11,7	16,8	15,7	17,9	10,3	9,0	11,7	5,6	4,8	6,3	3,6	2,8	4,5
Maranhão	11,3	10,2	12,4	18,1	16,2	20,0	10,3	7,7	12,9	3,8	2,5	5,1	3,6	1,8	5,3
Piauí	11,7	10,3	13,2	17,8	15,2	20,5	8,9	4,1	13,7	5,0	3,3	6,6	6,8	2,9	10,6
Ceará	12,2	11,1	13,4	18,5	16,5	20,6	10,3	7,4	13,3	7,6	5,7	9,4	3,1	0,9	5,4
Rio Grande do Norte	11,3	9,6	13,1	19,5	16,1	22,9	8,2	4,9	11,5	5,2	3,4	6,9	2,6	0,8	4,4
Paraíba	11,8	10,1	13,4	17,4	14,7	20,2	9,3	5,9	12,8	5,9	3,8	8,0	4,7	2,5	6,9
Pernambuco	11,3	9,6	13,0	16,1	13,2	19,0	13,7	9,6	17,7	7,1	5,0	9,2	4,5	2,4	6,6
Alagoas	10,6	9,3	12,0	15,8	13,7	17,9	12,7	8,3	17,0	4,8	2,7	6,8	1,7	0,5	3,0
Sergipe	9,4	8,2	10,6	14,9	12,6	17,3	7,7	4,5	10,9	4,5	2,8	6,2	1,7	0,5	3,0
Bahia	10,1	8,4	11,7	15,2	12,3	18,1	9,5	5,9	13,1	4,3	2,6	6,1	3,3	1,1	5,4
Sudeste	13,5	12,7	14,3	17,4	15,8	19,1	18,0	15,7	20,3	11,2	10,0	12,3	8,6	7,4	9,8
Minas Gerais	13,2	11,8	14,6	17,3	14,9	19,6	16,2	12,4	19,9	10,1	7,9	12,2	6,4	4,4	8,4
Espírito Santo	10,4	9,1	11,6	13,3	10,9	15,7	13,4	9,3	17,5	8,9	6,8	10,9	4,4	2,7	6,2
Rio de Janeiro	12,1	10,9	13,3	14,6	12,2	17,1	16,0	12,3	19,8	10,9	9,2	12,6	8,2	6,3	10,1
São Paulo	14,4	13,1	15,7	19,0	16,0	22,0	20,2	16,2	24,2	11,9	10,1	13,7	9,6	7,7	11,5
Sul	14,7	13,8	15,7	19,0	17,3	20,6	19,3	16,6	22,1	12,1	10,6	13,5	7,6	6,1	9,1
Paraná	14,7	13,0	16,4	20,3	17,3	23,3	21,2	16,3	26,1	11,1	8,3	13,9	6,1	3,9	8,2
Santa Catarina	13,1	11,6	14,6	17,6	14,8	20,5	16,2	12,6	19,9	10,6	8,7	12,6	6,4	4,2	8,7
Rio Grande do Sul	15,8	14,3	17,4	18,5	16,0	21,0	19,9	15,0	24,8	14,0	11,4	16,5	10,0	7,0	13,0
Centro-Oeste	13,7	12,7	14,7	20,9	18,9	23,0	14,8	12,5	17,1	10,5	9,1	11,9	6,0	4,3	7,7
Mato Grosso do Sul	16,3	14,5	18,2	21,1	17,9	24,4	17,4	12,6	22,1	15,9	12,4	19,3	5,8	3,7	7,8
Mato Grosso	13,0	11,3	14,8	17,5	13,5	21,5	20,0	14,6	25,4	9,5	6,6	12,4	3,3	1,5	5,1
Goiás	13,9	12,1	15,8	22,4	18,8	26,0	11,1	7,7	14,4	9,2	6,9	11,4	7,3	2,8	11,8
Distrito Federal	11,6	9,9	13,4	21,1	16,7	25,5	15,8	10,7	20,8	10,5	7,6	13,4	6,3	4,0	8,7

*Fonte: IBGE, Diretoria de Pesquisas, Coordenação de Trabalho e Rendimento, Pesquisa Nacional de Saúde 2019. ^306^*

**Tabela 11-8:** – Proporção de usuários atuais de tabaco a partir dos 18 anos de idade, de acordo com a cor da pele ou raça, no Brasil, suas regiões e unidades federativas, e áreas urbana e rural.

Regiões, unidades federativas e áreas urbana e rural	Total			Cor da pele ou raça								
				Branca			Negra			Parda		
	Proporção	IC 95%		Proporção	IC 95%		Proporção	IC 95%		Proporção	IC 95%	
		Limite inferior	Limite superior		Limite inferior	Limite superior		Limite inferior	Limite superior		Limite inferior	Limite superior
Brasil	12,8	12,4	13,2	11,8	11,2	12,4	13,7	12,5	15,0	13,5	12,9	14,2
Urbana	12,6	12,1	13,0	11,9	11,2	12,5	13,4	12,1	14,7	13,1	12,4	13,8
Rural	14,3	13,4	15,2	11,4	10,3	12,6	15,9	13,2	18,6	15,6	14,3	16,9
Norte	10,7	9,9	11,5	8,5	7,0	9,9	11,0	8,9	13,0	11,2	10,2	12,3
Rondônia	10,8	9,2	12,4	9,3	6,3	12,3	12,2	7,4	16,9	11,1	8,9	13,3
Acre	15,1	13,2	17,1	9,6	5,8	13,4	24,1	16,6	31,5	15,1	12,8	17,4
Amazonas	10,2	9,0	11,4	8,2	5,1	11,3	8,4	4,0	12,9	10,7	9,2	12,1
Roraima	11,6	9,8	13,3	7,6	4,6	10,6	16,9	9,7	24,1	12,0	10,0	13,9
Pará	10,1	8,6	11,5	7,7	5,2	10,3	7,8	4,5	11,0	11,0	9,1	12,8
Amapá	10,9	8,8	12,9	9,4	5,0	13,9	16,7	10,2	23,3	9,8	7,2	12,3
Tocantins	12,8	10,7	14,9	10,4	7,0	13,9	14,6	9,8	19,5	13,0	10,2	15,8
Nordeste	11,1	10,5	11,7	8,8	7,9	9,7	11,9	10,3	13,5	11,9	11,1	12,7
Maranhão	11,3	10,2	12,4	10,0	7,5	12,4	10,4	7,8	13,0	11,9	10,5	13,3
Piauí	11,7	10,3	13,2	8,4	5,5	11,3	9,2	6,0	12,4	13,3	11,4	15,2
Ceará	12,2	11,1	13,4	11,7	9,2	14,1	15,1	10,7	19,4	12,2	10,8	13,6
Rio Grande do Norte	11,3	9,6	13,1	7,5	5,4	9,7	22,1	15,3	28,9	12,4	9,9	14,8
Paraíba	11,8	10,1	13,4	8,5	6,3	10,7	20,8	14,8	26,8	12,2	10,3	14,1
Pernambuco	11,3	9,6	13,0	9,2	7,1	11,3	17,1	11,1	23,1	11,6	9,5	13,8
Alagoas	10,6	9,3	12,0	7,7	5,2	10,2	14,4	9,4	19,4	11,3	9,6	13,0
Sergipe	9,4	8,2	10,6	8,0	5,3	10,7	12,9	8,3	17,5	9,2	7,5	10,9
Bahia	10,1	8,4	11,7	6,5	4,0	8,9	9,0	6,2	11,7	11,9	9,3	14,5
Sudeste	13,5	12,7	14,3	12,3	11,3	13,4	14,3	12,1	16,5	14,9	13,5	16,2
Minas Gerais	13,2	11,8	14,6	11,0	9,0	13,0	13,0	9,8	16,2	15,4	13,3	17,6
Espírito Santo	10,4	9,1	11,6	8,5	6,5	10,6	13,6	9,7	17,5	10,9	8,8	13,0
Rio de Janeiro	12,1	10,9	13,3	11,6	10,0	13,1	12,1	9,2	15,0	12,3	10,3	14,2
São Paulo	14,4	13,1	15,7	13,2	11,7	14,8	17,0	12,2	21,7	16,2	13,7	18,6
Sul	14,7	13,8	15,7	13,2	12,2	14,2	17,3	13,4	21,3	18,9	16,3	21,5
Paraná	14,7	13,0	16,4	12,4	10,6	14,2	18,1	11,6	24,7	18,2	14,4	22,0
Santa Catarina	13,1	11,6	14,6	11,7	10,1	13,3	15,5	7,7	23,3	19,1	14,3	23,9
Rio Grande do Sul	15,8	14,3	17,4	14,7	13,1	16,3	17,3	11,4	23,2	20,2	15,4	25,0
Centro-Oeste	13,7	12,7	14,7	11,7	10,1	13,2	18,0	14,9	21,2	14,2	12,8	15,6
Mato Grosso do Sul	16,3	14,5	18,2	15,2	12,3	18,1	19,5	12,9	26,0	17,4	14,9	19,9
Mato Grosso	13,0	11,3	14,8	11,2	8,0	14,3	18,2	13,0	23,4	13,0	10,8	15,2
Goiás	13,9	12,1	15,8	10,2	7,4	13,0	18,1	12,2	23,9	15,3	12,6	17,9
Distrito Federal	11,6	9,9	13,4	11,6	8,5	14,7	17,2	11,7	22,6	10,2	8,3	12,2

*Fonte: IBGE, Diretoria de Pesquisas, Coordenação de Trabalho e Rendimento, Pesquisa Nacional de Saúde 2019. ^306^*

**Tabela 11-9:** – Prevalência de fumantes com mais de 18 anos de idade nas principais capitais brasileiras, de acordo com o sexo e com base na pesquisa domiciliar Vigitel.

Indicador	Sexo	2006	2007	2008	2009	2010	2011	2012	2013	2014	2015	2016	2017	2018	2019
Fumantes	Masculino	19,3	19,6	18	17,5	16,8	16,5	15,5	14,4	12,8	12,8	12,7	13,2	12,1	12,3
	Feminino	12,4	12,3	12	11,5	11,7	10,7	9,2	8,6	9	8,3	8	7,5	6,9	7,7
	Total	15,6	15,7	14,8	14,3	14,1	13,4	12,1	11,3	10,8	10,4	10,2	10,1	9,3	9,8

*Fonte: Vigitel – 2006 a 2019. ^276^*

**Tabela 11-10:** – Taxas de mortes atribuídas ao tabaco padronizadas por idade (por 100 mil) e variação percentual das taxas, de acordo com o sexo, no Brasil e suas unidades federativas, 1990 e 2019.

Local da morte	Feminino			Masculino		
	1990 (II 95%)	2019 (II 95%)	Variação percentual (II 95%)	1990 (II 95%)	2019 (II 95%)	Variação percentual (II 95%)
Acre	143.9 (122.5;164.7)	79.9 (68.9;90.7)	-44.5 (-52.9; -34.2)	240.9 (221.2;262.2)	123.8 (109.7;138.8)	-48.6 (-55.2; -41.7)
Alagoas	125.3 (105.4;145.5)	58.8 (49.7;69.2)	-53 (-62; -42.4)	216.2 (194.6;237.4)	103.7 (88.1;122.3)	-52 (-60; -42.6)
Amapá	86.3 (73;101.2)	48.5 (41.9;55.6)	-43.8 (-53.6; -31.9)	154.4 (139.7;169.1)	83.4 (74.4;93.4)	-46 (-52.5; -38.4)
Amazonas	101.3 (83.2;121.7)	44.4 (38.1;52.1)	-56.1 (-64.7; -44.8)	183.1 (162.3;204.2)	87.3 (75.6;100.5)	-52.3 (-59.7; -43.4)
Bahia	91.7 (75.5;109.9)	39.9 (32.3;48.9)	-56.5 (-66.7; -43.2)	177.1 (151.9;205.9)	101.1 (82;123.8)	-42.9 (-55.6; -26.8)
Brasil	139.7 (126.3;153.1)	57.1 (52.8;61.5)	-59.1 (-63.1; -54.9)	271.6 (258.6;285)	115.1 (108.1;123.3)	-57.6 (-60.2; -54.8)
Ceará	99.4 (80.3;120.4)	60.8 (49.3;74.3)	-38.8 (-53.3; -18.8)	164.7 (136.9;195.2)	103.3 (82.5;127.2)	-37.3 (-52.5; -16.8)
Distrito Federal	149.4 (122.9;178.2)	52.7 (45.2;62)	-64.7 (-71.1; -56.2)	303.7 (270;339.3)	97.3 (84.2;112)	-68 (-72.7; -61.9)
Espírito Santo	118.7 (101.7;137.1)	48.6 (41.3;56.8)	-59 (-66.3; -49.6)	239.1 (223.2;254.8)	111.2 (94.7;128.1)	-53.5 (-60.6; -46.1)
Goiás	155.7 (126.5;190.4)	60.4 (49.8;72.9)	-61.2 (-69.9;-49.5)	284.3 (242.7;333.5)	115.3 (94.2;139.5)	-59.4 (-68.1;-49.2)
Maranhão	69.8 (55.7;86.8)	46.1 (37.4;57.1)	-34 (-49.7;-11.3)	226 (187.6;269.3)	101.9 (80.7;124.6)	-54.9 (-65.7;-41.5)
Mato Grosso	116 (96.6;137.2)	50.3 (43.3;58.7)	-56.6 (-65;-46.1)	211.5 (182.9;242.8)	94.2 (81.3;108.2)	-55.5 (-63.1;-45.6)
Mato Grosso do Sul	142.4 (122.5;164.1)	55.2 (47.4;63.9)	-61.2 (-67.7;-53.6)	251.9 (230.7;273.9)	108.8 (94.9;125.6)	-56.8 (-63.1;-49.4)
Minas Gerais	139.8 (120.7;159.9)	50 (43.2;57.6)	-64.3 (-70.3;-57)	275 (252.2;298.7)	102.9 (90;117.3)	-62.6 (-67.7;-56.9)
Pará	103.7 (84.8;125.5)	43.1 (36.5;50.9)	-58.5 (-66.9;-48.3)	200.2 (170.7;233.3)	89.2 (76;102.8)	-55.4 (-63.8;-45.8)
Paraíba	102.9 (86.3;120.6)	50.9 (42.4;60.5)	-50.5 (-60.4;-37.7)	162.6 (145.6;180.1)	95.5 (79.7;113.3)	-41.2 (-52.5;-29.3)
Paraná	172.6 (149.9;195.8)	67.2 (57.6;77.6)	-61.1 (-67.7;-53.1)	298.6 (281.7;315.6)	127.2 (109.9;146.8)	-57.4 (-63.5;-50.7)
Pernambuco	140 (122;161)	71.1 (60.5;82.2)	-49.2 (-57.6;-38.9)	221 (205.5;235.5)	133.7 (114.3;154)	-39.5 (-48.9;-29.4)
Piauí	88.5 (74.5;105.1)	41.1 (34.8;48.2)	-53.5 (-62;-42.2)	186.3 (166.6;208)	77.1 (65.4;89.2)	-58.6 (-65.3;-51)
Rio de Janeiro	170 (149.8;190.3)	62 (53.9;71.8)	-63.5 (-69.1;-56.6)	352.4 (334;371.2)	129.1 (112.8;147.9)	-63.4 (-67.9;-57.8)
Rio Grande do Norte	97.7 (80;116.8)	49.5 (40.1;60.3)	-49.4 (-60.8;-35)	160.4 (139;183.6)	86.6 (69.6;106.1)	-46 (-58;-31.3)
Rio Grande do Sul	175.5 (155.4;197)	75.4 (65.5;85.6)	-57 (-63.4;-49.5)	366.9 (346.6;386.8)	144.2 (125.6;165.1)	-60.7 (-65.4;-54.7)
Rondônia	181.8 (154.2;213.4)	63.6 (54.3;74.6)	-65 (-71.6;-57.1)	257.6 (230.2;287)	102.7 (85.9;122.8)	-60.1 (-67.6;-51.2)
Roraima	130.3 (111.6;151)	57.2 (50.2;65.2)	-56.1 (-62.7;-47.9)	252.8 (228.8;278.4)	96 (84.4;107.7)	-62 (-67;-56.1)
Santa Catarina	146.8 (127.3;167.9)	53.5 (46.2;61.2)	-63.5 (-69.9;-55.9)	320.4 (296.4;344.5)	124.3 (109.2;141.8)	-61.2 (-66.4;-55)
São Paulo	164.6 (144.2;185.2)	61 (53;69.1)	-62.9 (-68.5;-56.5)	341.7 (318.2;366.2)	127.3 (111.7;145)	-62.7 (-67.6;-57.3)
Sergipe	89.8 (74;108.2)	40.2 (33;48.8)	-55.2 (-65.2;-41.2)	183.1 (160.2;205.8)	85.2 (69.6;102.8)	-53.5 (-62.5;-42.2)
Tocantins	95.9 (79.5;115.5)	46.7 (38.9;55.9)	-51.3 (-61.4;-38.1)	193.5 (165.8;223.1)	99.1 (83.2;117.7)	-48.8 (-58.8;-36)

*Fonte: Dados derivados do estudo Global Burden of Disease 2019, Institute for Health Metrics and Evaluation, University of Washington. ^46^*

**Tabela 11-11:** – Números de mortes atribuídas ao tabaco e suas taxas padronizadas por idade (por 100 mil), e variação percentual das taxas, no Brasil e suas unidades federativas, 1990 e 2019.

Local da morte	1990		2019		Variação percentual (II 95%)
	Número (II 95%)	Taxa (II 95%)	Número (II 95%)	Taxa (II 95%)	
Acre	268.1 (244.8;290.2)	188.3 (171.9;204.5)	555.2 (499.8;608.6)	99.8 (89.7;109.8)	-47 (-52.6; -40.8)
Alagoas	2251 (2031.3;2493.1)	168.1 (152.9;183.3)	2450.4 (2147.3;2781.5)	78.6 (68.9;89.1)	-53.2 (-59.7; -46.6)
Amapá	105 (95.2;114.6)	118.1 (107.2;129.5)	314.7 (286.5;346.4)	65 (59;71.6)	-45 (-50.8; -38.8)
Amazonas	980.5 (881.8;1086.3)	141.1 (126.2;156.8)	1754.9 (1565;1970)	64.8 (57.7;72.8)	-54 (-60; -47.2)
Bahia	8778 (7710.9;9868.5)	131.7 (115.9;147.5)	10687.3 (9048.9;12468.7)	66.6 (56.3;77.7)	-49.5 (-58.5; -39.1)
Brasil	168443.1 (159638.4;176773.3)	199.9 (189.1;210.6)	191.127.5 (180887.1;202595.5)	82.4 (77.9;87.5)	-58.8 (-61.1; -56.3)
Ceará	5309.1 (4636.5;6033.6)	130.1 (114;148.4)	7831.3 (6685.7;9108.7)	79.6 (68;92.7)	-38.8 (-50.1; -25.6)
Distrito Federal	955.1 (851.9;1072.6)	203.1 (178.6;230)	1524.6 (1362.5;1715)	70.6 (63.2;79.2)	-65.2 (-69.8; -60)
Espírito Santo	2358.2 (2208.3;2512.6)	176.2 (163.1;189.5)	3245.8 (2865.4;3627.9)	76.2 (67.2;85.1)	-56.8 (-62.1; -50.9)
Goiás	4064.4 (3535.8;4726.8)	215.6 (188.9;248.5)	5719 (4862.5;6648.2)	85.9 (73.2;99.7)	-60.2 (-67; -51.9)
Maranhão	3580.9 (2990.5;4188.3)	132.9 (112.6;154.1)	4554 (3835.5;5406)	70.2 (59.2;83.1)	-47.2 (-57.1; -34.3)
Mato Grosso	1196.6 (1045.2;1341.9)	167.7 (148;187)	2265.3 (2030.9;2507.8)	72.4 (64.8;80.2)	-56.8 (-62.7; -49.4)
Mato Grosso do Sul	1602.9 (1488.1;1726.3)	198.8 (183.3;215)	2279.7 (2040;2569)	80.3 (72;90.3)	-59.6 (-64.4; -54.3)
Minas Gerais	18482.8 (17049.2;20105.5)	200.8 (185.2;218.2)	19486.7 (17571.9;21630.8)	73.7 (66.5;81.9)	-63.3 (-67.2; -58.7)
Pará	2922 (2551.9;3292)	149.7 (131.3;168.8)	4359.3 (3833.9;4910.9)	65.1 (57.5;73.3)	-56.5 (-62.7; -49.3)
Paraíba	2969.5 (2689.7;3264.7)	131.1 (118.9;144)	3391.9 (2965.5;3853)	70.3 (61.4;80.1)	-46.4 (-53.8; -38.3)
Paraná	10355.6 (9761.2;10941.2)	234.4 (218.8;249.7)	12142 (10811.1;13558.7)	94 (83.7;104.9)	-59.9 (-64.5; -55.2)
Pernambuco	7749.4 (7180.2;8368.7)	176.8 (164;190.9)	9553.9 (8501;10648.7)	97.7 (87;108.7)	-44.8 (-51.4; -37.8)
Piaui	1774 (1606;1950.4)	133.2 (120.4;146.4)	2162.9 (1901.9;2425.6)	57.4 (50.5;64.3)	-56.9 (-62.7; -51.2)
Rio de Janeiro	22495.9 (21190.9;23737.9)	246.4 (230.1;262.6)	20055.2 (18102.7;22233)	89.7 (80.9;99.3)	-63.6 (-67.3; -59.3)
Rio Grande do Norte	2027.5 (1804.3;2269.4)	127.2 (113.2;141.9)	2549.6 (2176.1;2966.6)	65.7 (56.1;76.6)	-48.3 (-57.1; -38.1)
Rio Grande do Sul	15779.3 (14859.6;16593.5)	257.8 (241.2;273.4)	16325.6 (14743.7;18073.6)	104.6 (94.4;115.9)	-59.4 (-63.6; -54.7)
Rondônia	613.6 (537.2;687.9)	226.1 (205.9;249)	1200.6 (1047.7;1377.8)	83.3 (73.1;95.5)	-63.2 (-68.7; -57.2)
Roraima	94.8 (84.7;103.6)	196.2 (180.3;214.2)	254.2 (230.2;279.4)	76.7 (69.3;84.8)	-60.9 (-65.1; -56.1)
Santa Catarina	5228.8 (4848.3;5603.6)	227 (209.9;244.5)	6668.9 (6011;7389.5)	84.5 (76.1;93.6)	-62.8 (-66.9; -58)
São Paulo	45041.6 (42124.3;47925.1)	243.1 (226.1;260.4)	47506.6 (42991.3;52327.4)	89.1 (80.7;98.4)	-63.3 (-67; -59.3)
Sergipe	958.8 (859.2;1064.6)	130.8 (116.6;146.4)	1314.9 (1124.3;1520.3)	59.7 (51.2;68.7)	-54.4 (-61.8; -46.2)
Tocantins	499.9 (438;568.3)	142.2 (124.8;159.8)	973.1 (847.1;1113.9)	70.7 (61.5;81)	-50.3 (-57.8; -40.9)

*Fonte: Dados derivados do estudo Global Burden of Disease 2019, Institute for Health Metrics and Evaluation, University of Washington. ^46^*

**Tabela 11-12:** – Proporção de não fumantes a partir de 18 anos de idade expostos ao tabagismo passivo em ambiente de trabalho fechado, de acordo com o sexo, no Brasil, suas regiões e unidades federativas, e áreas urbana e rural.

Regiões, unidades federativas e áreas urbana e rural	Total			Sexo					
				Masculino			Feminino		
	Proporção	IC 95%		Proporção	IC 95%		Proporção	IC 95%	
		Limite inferior	Limite superior		Limite inferior	Limite superior		Limite inferior	Limite superior
Brasil	8,4	7,8	9,0	10,4	9,4	11,3	6,7	6,1	7,4
Urbana	8,3	7,7	8,9	10,3	9,3	11,3	6,6	5,9	7,3
Rural	10,2	8,6	11,7	11,6	9,4	13,8	9,0	6,8	11,2
Norte	7,8	6,8	8,8	10,1	8,5	11,7	5,6	4,4	6,8
Rondônia	9,8	6,9	12,8	12,9	7,5	18,3	7,4	3,8	11,0
Acre	5,9	3,9	8,0	6,9	3,2	10,7	5,1	2,7	7,5
Amazonas	7,9	5,7	10,0	10,5	7,1	13,9	5,0	2,8	7,1
Roraima	7,9	5,5	10,3	9,0	5,3	12,7	6,9	4,1	9,7
Pará	6,7	5,0	8,4	8,7	6,1	11,3	4,8	2,8	6,9
Amapá	7,6	4,8	10,3	9,6	5,4	13,8	5,8	2,0	9,5
Tocantins	10,8	7,9	13,6	15,0	10,0	20,0	7,7	4,5	10,9
Nordeste	9,2	8,4	10,0	11,0	9,8	12,3	7,7	6,7	8,8
Maranhão	10,4	8,5	12,3	12,4	9,2	15,5	8,8	6,3	11,3
Piauí	13,2	9,9	16,6	15,4	10,8	20,0	11,4	6,6	16,2
Ceará	8,5	6,6	10,4	9,0	5,9	12,1	8,1	5,7	10,5
Rio Grande do Norte	10,6	7,4	13,9	13,5	8,7	18,3	8,2	4,8	11,7
Paraíba	10,1	7,9	12,3	11,2	8,2	14,2	9,2	6,1	12,2
Pernambuco	8,8	6,8	10,9	9,9	7,2	12,6	7,8	5,2	10,5
Alagoas	8,7	6,4	11,1	10,5	6,7	14,3	7,2	4,5	10,0
Sergipe	8,3	6,3	10,3	9,3	6,4	12,2	7,4	4,4	10,3
Bahia	8,2	6,3	10,1	11,1	7,9	14,3	6,0	3,6	8,3
Sudeste	8,8	7,7	9,8	10,4	8,6	12,2	7,3	6,1	8,6
Minas Gerais	11,5	9,0	13,9	15,8	10,8	20,8	8,2	5,9	10,5
Espírito Santo	9,1	7,2	11,0	12,6	9,3	15,8	6,2	3,9	8,6
Rio de Janeiro	6,8	5,3	8,2	7,8	5,7	10,0	5,8	3,8	7,8
São Paulo	8,3	6,7	9,9	9,1	6,7	11,6	7,6	5,7	9,5
Sul	6,3	5,2	7,4	9,0	7,1	11,0	3,8	2,8	4,9
Paraná	5,8	3,9	7,6	8,5	5,2	11,8	3,2	1,7	4,7
Santa Catarina	5,4	4,1	6,7	7,0	4,9	9,2	3,8	2,3	5,3
Rio Grande do Sul	7,4	5,5	9,3	10,9	7,1	14,7	4,5	2,5	6,5
Centro-Oeste	9,0	7,6	10,4	11,3	8,8	13,8	6,9	5,4	8,4
Mato Grosso do Sul	7,2	5,3	9,0	9,8	6,6	12,9	4,8	3,0	6,7
Mato Grosso	9,2	6,7	11,7	10,3	6,8	13,8	8,2	4,8	11,6
Goiás	11,5	8,5	14,5	14,8	9,3	20,3	8,4	5,4	11,4
Distrito Federal	5,8	4,0	7,6	6,8	4,2	9,3	4,8	2,7	6,9

*Fonte: IBGE, Diretoria de Pesquisas, Coordenação de Trabalho e Rendimento, Pesquisa Nacional de Saúde 2019. ^306^*

**Tabela 11-13:** – Distribuição dos usuários de dispositivos eletrônicos com nicotina líquida ou folha de tabaco triturada, com idade a partir de 15 anos, no Brasil, suas regiões e unidades federativas.

Regiões e unidades federativas	Prevalência de usuários ativos	95% IC	
		Limite inferior	Limite superior
**Brasil**	**0,6**	**0,5**	**0,8**
**Norte **	**0,2**	**0,1**	**0,2**
Rondônia	0,7	0,1	1,4
Acre	0,1	0,0	0,2
Amazonas	0,0	0,0	0,1
Roraima	0,0	0,0	0,1
Pará	0,1	0,0	0,2
Amapá	0,1	0,0	0,3
Tocantins	0,4	0,0	0,7
**Nordeste **	**0,1**	**0,1**	**0,2**
Maranhão	0,1	0,0	0,2
Piauí	0,5	0,1	0,8
Ceará	0,3	0,0	0,5
Rio Grande do Norte	0,0	0,0	0,1
Paraíba	0,0	0,0	0,1
Pernambuco	0,0	0,0	0,1
Alagoas	0,2	0,0	0,4
Sergipe	0,1	0,0	0,2
Bahia	0,1	0,0	0,2
**Sudeste **	**0,7**	**0,5**	**1,0**
Minas Gerais	0,2	0,0	0,3
Espírito Santo	0,4	0,0	0,8
Rio de Janeiro	0,2	0,0	0,3
São Paulo	1,3	0,8	1,7
**Sul **	**1,1**	**0,7**	**1,5**
Paraná	2,1	1,2	3,0
Santa Catarina	1,0	0,5	1,6
Rio Grande do Sul	0,2	0,0	0,3
**Centro-Oeste **	**1,5**	**1,1**	**1,8**
Mato Grosso do Sul	2,1	1,2	3,0
Mato Grosso	0,4	0,2	0,7
Goiás	1,4	0,7	2,0
Distrito Federal	2,2	1,4	2,9

*Source: PNS 2019. ^13^*

**Figura 11-1:**
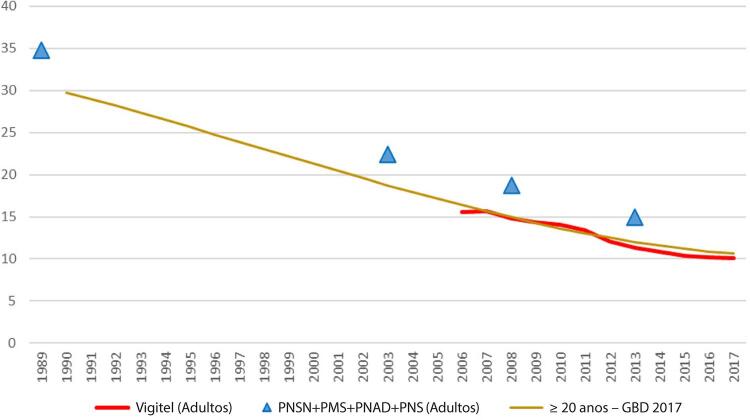
-Tendência da prevalência de tabagismo atual de acordo com as estimativas do GBD 2017 e pesquisas brasileiras (valores brutos) em indivíduos a partir dos 20 anos de idade, Brasil, 1989 a 2017.

Fonte: Malta DC et al. ^400^

**Figura 11-2:**
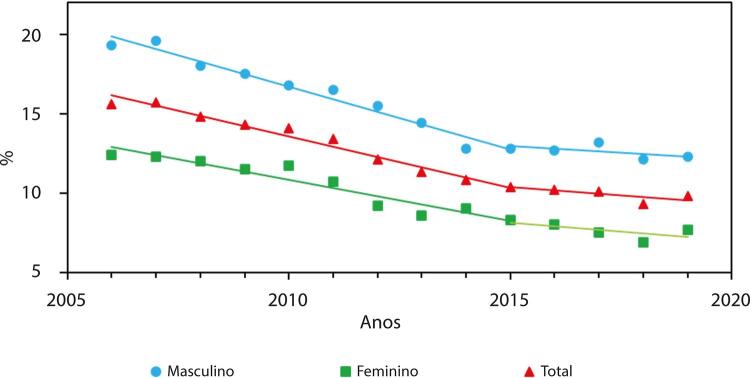
-Tendência da prevalência de tabagismo, de acordo com o sexo e dados da pesquisa Vigitel, nas capitais brasileiras, entre 2006 e 2019.

Fonte: Vigitel – 2006 a 2019. ^276^

**Figura 11-3:**
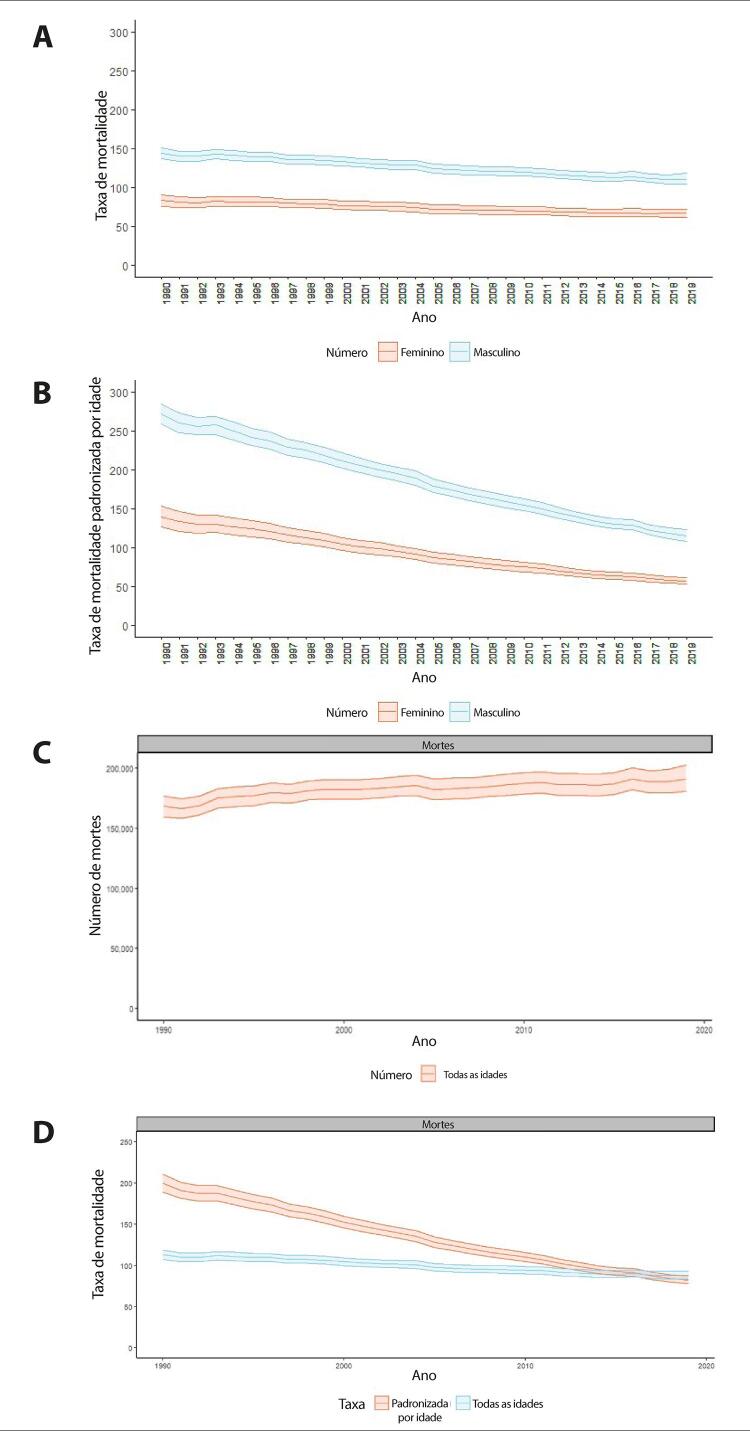
-Taxa de mortalidade (A) e taxa de mortalidade padronizada por idade (B) de acordo com sexo; número absoluto de mortes associadas com o uso de tabaco (C) e taxas de mortalidade associada ao uso de tabaco para todas as idades e padronizadas por idade (D), de 1990 a 2019.

Fonte: Dados derivados do estudo Global Burden of Disease 2019, Institute for Health Metrics and Evaluation, University of Washington. ^46^

**Figura 11-4:**
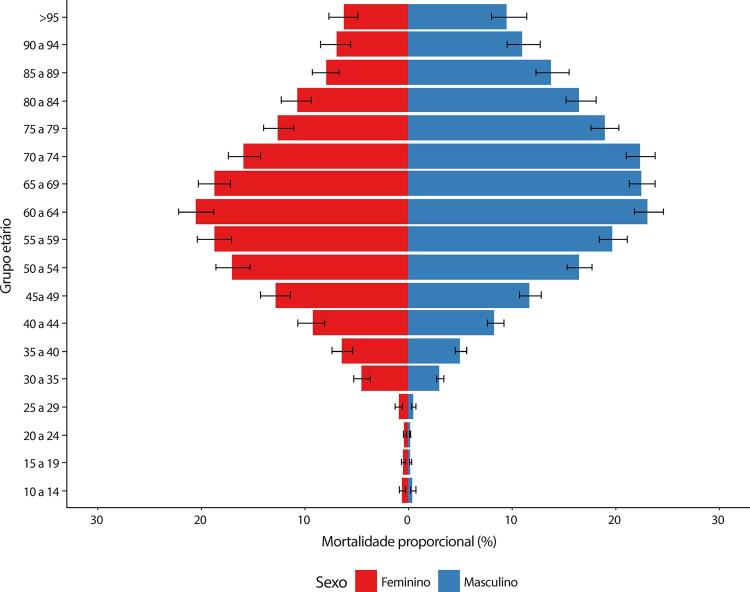
-Proporção de mortalidade de cada grupo etário apresentado no eixo Y de acordo com o sexo, no Brasil em 2019.

Fonte: Dados derivados do estudo Global Burden of Disease 2019, Institute for Health Metrics and Evaluation, University of Washington. ^46^

**Figura 11-5:**
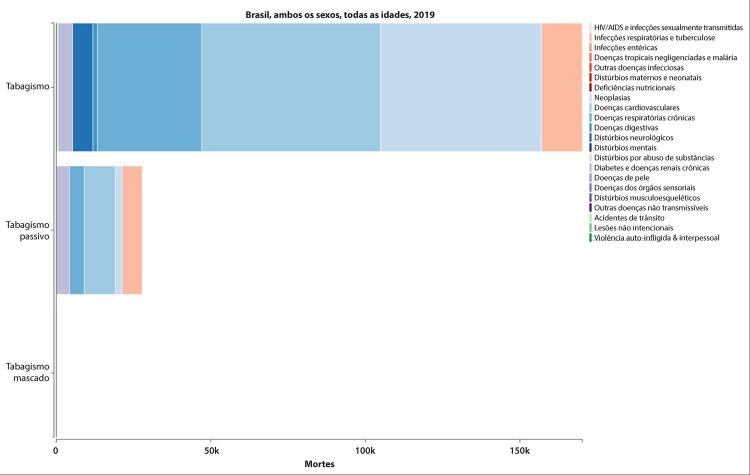
-Número absoluto de mortes atribuídas ao tabaco e ao tabagismo passivo, por todas as causas de morte, para ambos os sexos, 2019. Fonte: Dados derivados do estudo Global Burden of Disease 2019, Institute for Health Metrics and Evaluation, University of Washington. ^46^

**Figura 11-6:**
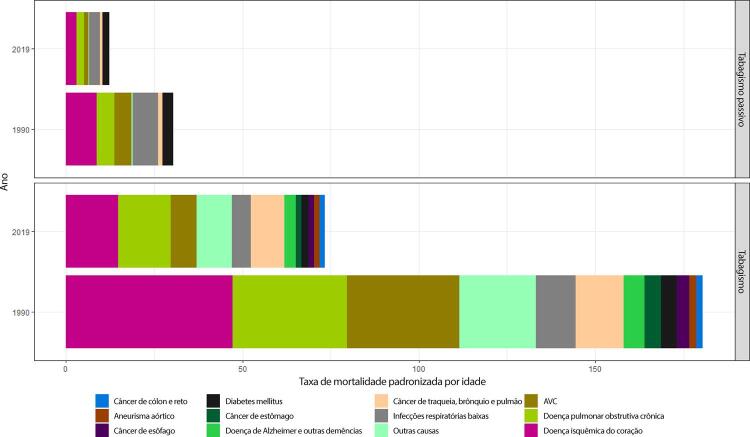
-Taxas de mortalidade por tabagismo e por tabagismo passivo padronizadas por idade para ambos os sexos, por todas as causas de morte, Brasil, 1990 e 2019.

Fonte: Dados derivados do estudo Global Burden of Disease 2019, Institute for Health Metrics and Evaluation, University of Washington. ^46^

**Figura 11-7:**
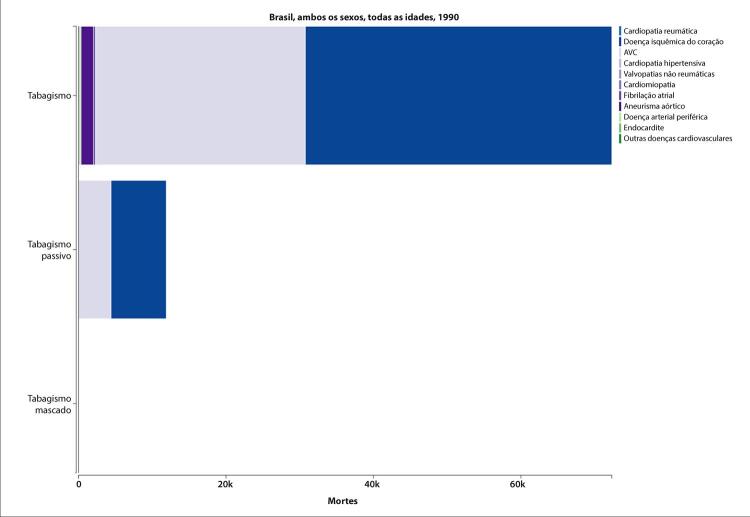
-Número total de mortes por doença cardiovascular atribuída ao tabaco e ao tabagismo passivo, para ambos os sexos. Cada cor representa uma causa específica de morte cardiovascular, 1990.

Fonte: Dados derivados do estudo Global Burden of Disease 2019, Institute for Health Metrics and Evaluation, University of Washington. ^46^

**Figura 11-8:**
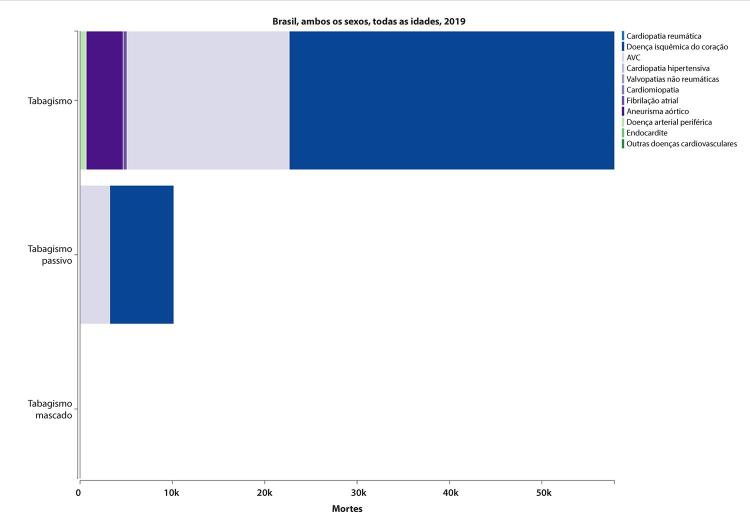
-Número total de mortes por doença cardiovascular atribuída ao tabaco e ao tabagismo passivo, para ambos os sexos. Cada cor representa uma causa específica de morte cardiovascular, 2019.

Fonte: Dados derivados do estudo Global Burden of Disease 2019, Institute for Health Metrics and Evaluation, University of Washington. ^46^

**Figura 11-9:**
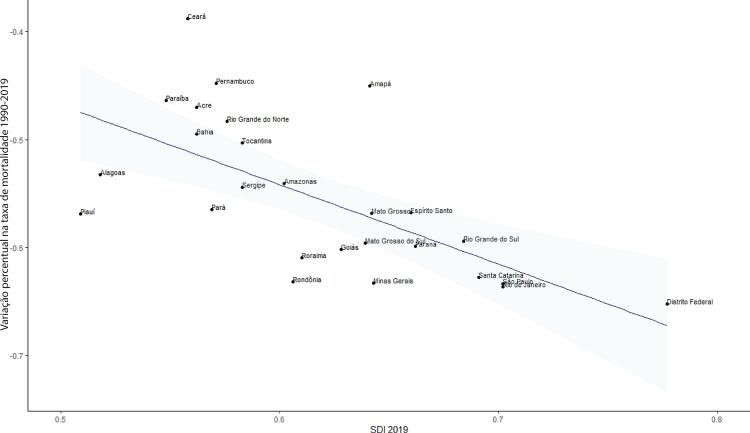
-Correlação entre o Índice Sociodemográfico (SDI) de 2019 e a variação percentual das taxas de mortalidade atribuída ao tabagismo entre 1990 e 2019, no Brasil.

Fonte: Dados derivados do estudo Global Burden of Disease 2019, Institute for Health Metrics and Evaluation, University of Washington. ^46^

**Figura 11-10:**
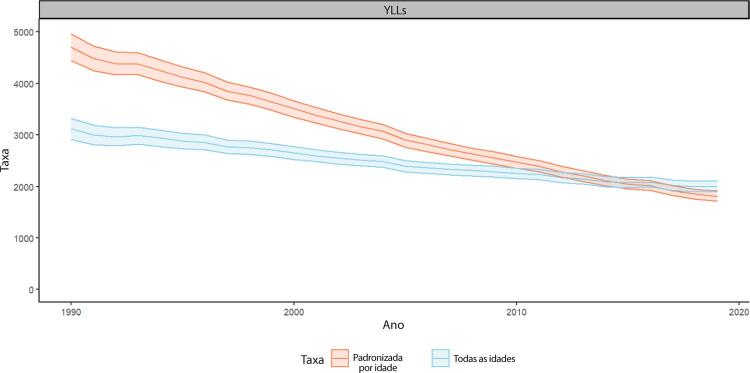
-Tendência temporal das taxas de YLLs por uso de tabaco de 1990 a 2019.

Fonte: Dados derivados do estudo Global Burden of Disease 2019, Institute for Health Metrics and Evaluation, University of Washington. ^46^

**Figura 11-11:**
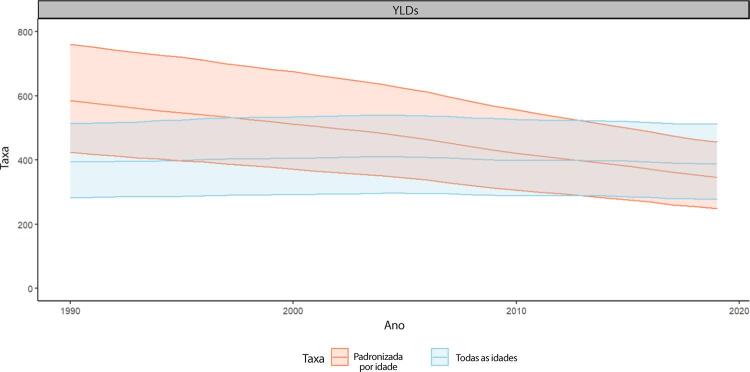
-Tendência temporal das taxas de YLDs por uso de tabaco de 1990 a 2019. Fonte: Dados derivados do estudo Global Burden of Disease 2019, Institute for Health Metrics and Evaluation, University of Washington. ^46^

**Figura 11-12:**
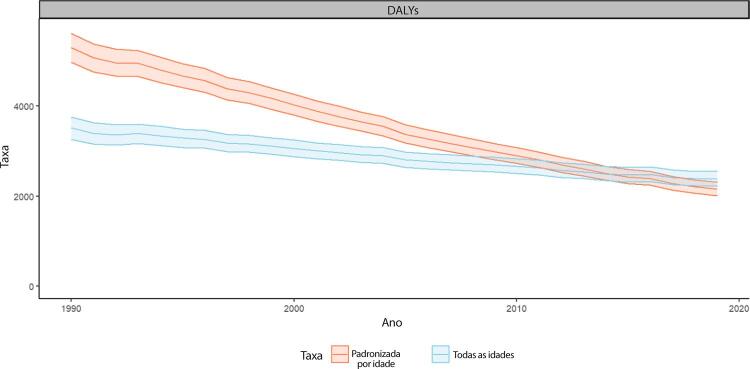
-Tendência temporal das taxas de DALYs por uso de tabaco de 1990 a 2019.

Fonte: Dados derivados do estudo Global Burden of Disease 2019, Institute for Health Metrics and Evaluation, University of Washington. ^46^

### Panorama

#### Definições

•Fumante atual: Adulto que já fumou cigarro em algum momento e ainda fuma. ^19^ ^,^ ^400^ Adolescente que fumou pelo menos um dia nos últimos 30 dias foi considerado fumante atual de cigarro. ^401^

•Tabagismo passivo: Em geral, refere-se à presença de fumaça de cigarro no ambiente de um não fumante. ^400^

•Ex-fumante: Adulto que fumava mas havia cessado o tabagismo por ocasião da entrevista. ^400^

•Experimentação de tabaco: Definido como tendo fumado cigarro pelo menos uma vez na vida. ^401^

•Dispositivos eletrônicos de fumar, mais conhecidos como cigarros eletrônicos, são aqueles operados por bateria que fornecem nicotina, sabores e outras substâncias químicas em aerossol ao usuário. ^3^ ^,^ ^402^ Os líquidos usados nesses dispositivos podem diferir quanto à composição química, concentração de nicotina e aditivos usados, tendo sido descritos mais de 8.000 sabores de cigarros eletrônicos. Discrepância entre a composição do produto declarada na embalagem e a verdadeira já foi mostrada.

•O SDI é um índice composto que mede renda per capita, fertilidade e educação e reflete o desenvolvimento sociodemográfico. O SDI permite que estados e países sejam comparados quanto ao seu desenvolvimento. ^403^

#### Tabaco e Doença Cardiovascular Total

•O uso do tabaco é uma das principais causas de morte prevenível no Brasil e no mundo. ^19^ ^,^ ^400^ O tabaco é um dos principais fatores de risco de DCNT, como DCV, câncer, doenças respiratórias crônicas, restrição ao crescimento intrauterino e predisposição ao parto prematuro. ^3–5^ O impacto negativo do tabaco na saúde resulta tanto do consumo direto de várias formas de produtos derivados do tabaco (fumado, inalado ou mascado) quanto da exposição ao tabagismo passivo. ^400^ ^,^ ^404^

•O tabaco é um fator de risco independente para DCV e multiplica o risco quando associado a outros fatores de risco, como hipertensão, dislipidemia e diabetes mellitus. ^405^ Há um aumento significativo no risco para DCV mesmo em níveis baixos de exposição ao tabaco, incluindo tabagismo passivo e uso de charuto. Além disso, o risco cresce, mas em menor grau, com o aumento no número de cigarros consumidos por dia. ^34^

•Tabagismo atual ou passado está associado com risco aumentado de insuficiência cardíaca com fração de ejeção reduzida ou preservada. ^406^ ^,^ ^407^

•O consumo de tabaco em diferentes formas de tabagismo está associado com risco aumentado de eventos cardiovasculares, com OR de 1,67 (IC 95%, 1,25 – 2,24) para a associação de doença isquêmica do coração e insuficiência cardíaca com consumo de fumo no narguilé. ^408^

•Não fumantes expostos ao tabagismo passivo em casa ou no trabalho apresentam aumento de 25% a 30% no risco de desenvolver DCV. ^4^ ^,^ ^8^ Exposição ao tabagismo passivo eleva o risco de AVC em 20% a 30% e está associada a aumento de mortalidade após o evento. ^3^ ^,^ ^405^

#### Medidas

•A prevalência de tabagismo será abordada com base nas pesquisas populacionais mais recentes no Brasil: o Estudo ELSA-Brasil; a PNS 2019, contendo estimativas para a população a partir de 18 anos; a PeNSE 2015, contendo estimativas para adolescentes de 13 a 15 anos; o Estudo ERICA, que incluiu 74.589 adolescentes de 12 a 17 anos, de municípios com mais de 100 mil habitantes; e as estimativas de tendência para adultos nas capitais brasileiras entre 2006 e 2019. ^401^ ^,^ ^409^ ^,^ ^410^

•As taxas de mortalidade e *hazard ratios* ajustadas da associação entre tabagismo e morte são apresentadas em coortes brasileiras. ^19^ ^,^ ^404^

•As taxas de mortalidade e os números absolutos de mortes atribuídas ao tabaco são apresentados para o Brasil e suas 27 UF em 1990 e 2019, sendo as estimativas extraídas do Estudo GBD 2019. ^19^

## Prevalência

### Prevalência do Uso de Tabaco entre Adolescentes

•As Tabelas 11-1 a 11-3 mostram a prevalência dos indicadores de tabagismo entre adolescentes do nono ano, com idade entre 13 e 15 anos, de acordo com dados da PeNSE 2015. ^410^

•Ainda de acordo com dados da PeNSE 2015, 18,4% (IC 95%, 17,8 - 19) dos adolescentes de 13 a 15 anos já experimentaram cigarro. A prevalência foi maior no sexo masculino (19,4%; IC 95%, 16,6 – 18,2) do que no feminino (17,4%; IC 95%, 18,7 – 20,0). A porcentagem de adolescentes que experimentaram fumar variou de 27,0% no Mato Grosso do Sul a 9,3% em Sergipe, sendo maior na região Sul (24,9%)e menor na região Nordeste. ^410^

•A prevalência de tabagismo atual ou ter fumado nos 30 dias que antecederam a pesquisa foi de 5,6% (IC 95%, 5,3 – 5,9), sem diferença quanto ao sexo (masculino 5,8%; IC 95%, 5,4 – 6,3; feminino 5,4%; IC 95%, 4,9 – 5,8). O uso de outros produtos derivados do tabaco foi de 6,1% (IC 95%, 5,7 – 6,4) entre adolescentes do sexo masculino e 5,6% (IC 95%, 5,1 – 6,0) entre as do sexo feminino. ^410^

•O estudo ERICA, um estudo transversal, de âmbito nacional e base escolar dos riscos cardiovasculares, incluiu 74.589 adolescentes de 12 a 17 anos, de municípios com mais de 100 mil habitantes. Os resultados mostraram que 18,5% (IC 95%, 17.7-19.4) dos adolescentes já haviam fumado pelo menos uma vez, 5,7% (IC 95%, 5,3 – 6,2) fumavam na ocasião da pesquisa e 2,5% (IC 95%, 2,2 – 2,8) fumavam com frequência, sem diferença significativa entre os sexos. ^401^

•A região Sul apresentou maior prevalência de experimentação de tabaco (23,3%; IC 95%, 21,5 – 25,3), de uso atual de tabaco (7,3%; IC 95%, 6,2 - 8.7) e de uso de tabaco por sete dias consecutivos (3,8%; IC 95%, 2,8 – 5,1) em comparação às regiões Norte (19,2%, 5,9% e 2,2%, respectivamente) e Nordeste (15,2%, 4,7% e 1,5%, respectivamente).

•A prevalência do uso de tabaco não diferiu significativamente de acordo com indicadores socioeconômicos (etnia autorrelatada, nível educacional, nível educacional materno, nível educacional paterno) para ambos os sexos.

•A prevalência foi mais alta entre adolescentes que haviam tido empregos remunerados no ano anterior ao estudo, para ambos os sexos [9,3% (IC 95%, 8,1 – 10,5) vs 5,0% (IC 95%, 4,3 - 5,6) para o sexo masculino; 8,8% (IC 95%, 7,5 – 10,1) vs 4,6% (IC 95%, 4,0-5,1) para o sexo feminino]. Além disso, a prevalência foi maior entre aqueles que não moravam com os dois pais em comparação àqueles que moravam [8,0% (IC 95%, 6,8 – 9,2) vs 4,8% (IC 95%, 4,0-5,5) para o sexo masculino; 6,4% (95% CI, 5,7 – 7,1) vs 4,4% (95% CI, 3,8 – 5,1)) para o sexo feminino].

•Estudantes do sexo feminino de escolas públicas relataram mais tabagismo [5.7% (IC 95%, 5.1 - 6.2)] do que as de escolas privadas [3.7% (IC 95%, 2.3 - 5.1)]. Quanto aos adolescentes do sexo masculino, a diferença não foi significativa, 6,1% (IC 95%, 5,6 – 6,9) e 5,2% (IC 95%, 3,5 – 7,0), respectivamente.

•A prevalência de tabagismo é maior entre adolescentes que convivem em casa com fumantes: 8,1% (IC 95%, 6,7 – 9,6) vs 5,4% (IC 95%, 4,7 – 6,1) para o sexo masculino; e 7,1% (IC 95%, 6,2 – 8,0) vs 4,5% (IC 95%, 4,0 – 5,1) para o sexo feminino. Além disso, maior prevalência foi observada entre aqueles em contato com fumantes fora de casa: para o sexo masculino, 9,9% (IC 95%, 8,6 – 11,1) vs 3,6% (IC 95%, 3,0 – 4,3); e para o sexo feminino, 7,6% (IC 95%, 6,8 – 8,4) vs 2,7% (IC 95%, 2,2 – 3,3).

### Prevalência do Uso de Tabaco entre Adultos

•A Tabela 11-4 mostra dados da PNS 2019 indicando que 12,8% (IC 95%, 12,4 – 13,2) dos adultos usam algum derivado do tabaco, sendo o uso maior entre os homens (16,2%; IC 95%, 15,6 – 16,9) do que entre as mulheres (9,9%; IC 95%, 9,3 – 10,3). ^306^

•A prevalência do uso de alguns derivados do tabaco é maior na área rural (14,3%) do que na urbana (12,6%), assim como maior na região Sul (14,7%) do que na Norte (10,7%).

•A proporção de fumantes acima de 18 anos em 2019 foi 12,6% (IC 95%, 12,2 – 13,0) e, de acordo com o sexo, 15,9% (IC 95%, 15,3 – 16,6) dos homens e 9,6% (IC 95%, 9,2 – 10,1) das mulheres ( Tabela 11 -1). ^306^

•Quanto aos grupos etários, a prevalência foi a seguinte em ordem decrescente: 14,9% (IC 95%, 14,2 – 15,5) no grupo de 40-59 anos; 10,8% (IC 95%, 9,6 – 12,0) no grupo de 18-24 anos; 12,0% (IC 95%, 11,2 – 12,7) no grupo de 25-39 anos; e 11,9% (IC 95%, 11.2 - 12.6) no grupo de 60 anos ou mais ( Tabela 11 -5). ^306^

•A prevalência de tabagismo é menor nas populações com níveis mais elevados de educação e renda: 17,6% (IC 95%, 16,8 – 18,4) entre aqueles sem educação formal ou educação fundamental incompleta; 15,5% (IC 95%, 14,3 – 16,6) entre aqueles com educação fundamental completa e nível médio incompleto; 9,6% (IC 95%, 8,9 – 10,2) entre aqueles com nível médio completo e superior incompleto; e 7,1% (IC 95%, 6,3 – 7,8) entre aqueles com nível superior completo ( Tabela 11 -7). ^306^

•A prevalência de tabagismo foi maior entre indivíduos negros (13,7%; IC 95%, 12,5 - 15) em comparação a “pardos” (13,5%; IC 95%, 12,9 – 14,2) e brancos (11,8%; IC 95%, 11,2 – 12,4) ( Tabela 11 -8). ^306^

•A pesquisa Vigitel 2019 mostrou uma frequência de adultos fumantes de 9,8%, maior entre os homens (12,3%) do que entre as mulheres (7,7%). Na população total, a frequência de fumantes tendeu a ser mais baixa entre adultos jovens (menos de 25 anos de idade) e a partir de 65 anos. A frequência de tabagismo diminuiu com o aumento da escolaridade, sendo particularmente alta entre homens com até oito anos de escolaridade (16,8%). ^410^

•De acordo com o Estudo GBD 2019, no Brasil, a prevalência de tabagismo passivo em casa é de 9,2% (II 95%, 8,8 – 9,8), maior entre mulheres (10,2%; IC 95%, 8,7 – 10,8) do que entre homens (7,9%; II 95%, 7,3 – 8,5). ^19^

•A prevalência de tabagismo passivo no trabalho é de 8,4% (IC 95%, 8,8 – 9,8), maior entre homens (10,4%; IC 95%, 9,4 – 11,3) do que entre mulheres (8,7%; IC 95%, 6,1 – 7,4) ( Tabela 11 -12). ^19^

•O uso de cigarros eletrônicos foi medido pela primeira vez na PNS 2019, revelando uma prevalência de 0,6%, sendo o uso maior no Distrito Federal (2,2%) e nos estados do Mato Grosso do Sul (2,1%) e Paraná (2,1%) ( Tabela 11 -13). ^405^

### Tendência de Prevalência

•A prevalência obtida nas principais pesquisas domiciliares nacionais, no inquérito telefônico Vigitel com participantes a partir de 18 anos de idade e na PNS indica uma redução significativa na prevalência de tabagismo na população adulta, de 37,6% de 2006 a 2019. Essa tendência é similar àquela reportada em outros nacionais estudos ( Tabela 11 -9, Figura 11 -1). ^306^ ^,^ ^400^ ^,^ ^410^ ^,^ ^411^ Entretanto, o índice é 0,5% maior do que o calculado em 2018.

•De acordo com os dados do inquérito Vigitel, nas capitais brasileiras, entre 2006 e 2019, houve um declínio no tabagismo para ambos os sexos. Depois de 2015, o declínio foi menor, havendo até estabilidade para os homens ( Tabela 11 -9 e Figura 11 -2). ^400^ ^,^ ^411^

•Entre adolescentes e comparando com a pesquisa PeNSE 2012, a prevalência de tabagismo ativo permaneceu sem alteração: 5,0% em 2012 e 5,6% em 2015. Entretanto, o uso de derivados do tabaco aumentou, passando de 4,8% (IC 95%, 4,6 – 5,0) em 2012 para 6,1% (IC 95%, 5,7 – 6,4) em 2015, sendo a proporção maior para os homens. O uso de cigarro junto com outros derivados do tabaco aumentou em 18%, passando de 7,6% em 2012 para 9,0% em 2015. O narguilé foi o produto mais usado em 2015 (71,6%; IC 95%, 68,8 – 74,2) e com maior frequência entre as mulheres (4% entre adolescentes masculinos e 5,6% entre adolescentes femininas). ^8^ ^,^ ^16^

•De acordo com o Estudo GBD 2019, as mortes relacionadas a tabagismo passivo diminuíram entre 1990 e 2019. As principais causas de morte por DCV associadas a tabagismo passivo foram doença isquêmica do coração e AVC. ^19^ Em 1990, houve 7.489 mortes (5,03 por 100 mil habitantes) por doença isquêmica do coração devida ao tabagismo passivo e, em 2019, 6.934 mortes (3,2 por 100 mil habitantes). ^19^ Em 1990, houve 4.400 mortes (2,96 por 100 mil habitantes) por AVC devido ao tabagismo passivo e, em 2019, 3.219 mortes (1,3 por 100 mil habitantes). ^19^

## Mortalidade

### Tabaco e Mortalidade Total

•A Tabela 11 -10 mostra as taxas de mortalidade atribuída ao tabaco padronizadas por idade (por 100 mil habitantes), além da variação percentual dessas taxas entre 1990 e 2019, de acordo com o sexo, no Brasil e suas UF. A Tabela 11 -11 mostra o número e as taxas de mortes atribuídas ao tabaco padronizadas por idade e variação percentual dessas taxas entre 1990 e 2019. Esses dados foram estimados pelo GBD, considerando a literatura existente e a fração atribuível populacional do tabagismo, que é a proporção de casos atribuídos ao uso de tabaco ( Figura 11 -3). ^19^

•De acordo com dados do GBD 1990, o número absoluto de mortes atribuídas ao tabaco em 1990 foi 168.443 (II 95%, 159.638 – 176.773), tendo aumentado em 2019 para 191.127 (II 95%, 180.887 – 202.595) ( Tabela 11 -11). ^19^

•O número absoluto de mortes atribuídas ao tabaco aumentou principalmente devido ao crescimento e envelhecimento da população; entretanto, houve uma redução de 58,8% (II 95%, 56,3 - 61,1) na taxa de mortalidade atribuída ao tabagismo, que passou de 199,9 (II 95%, 189,1 – 210,6) por 100 mil habitantes em 1990 para 82,4 (II 95%, 77,9 – 87,5) por 100 mil habitantes em 2019 ( Tabela 11 -11). Essa redução resultou da diminuição do risco ou da prevalência de fumantes. ^19^

•As taxas de mortalidade atribuída ao tabaco diminuíram em todas as UF brasileiras e, em 2019, as mais altas foram observadas no Rio Grande do Sul (104,6; II 95%, 94,4 -115,9), Acre (99,8; II 95%, 89,7 – 109,8) e em Pernambuco (97,7; II 95%, 87 – 108,7). ^19^

•A Figura 11 -5 mostra o número absoluto de mortes atribuídas ao tabaco e ao tabagismo passivo. Fumar tabaco contribuiu para mais de 190 mil mortes por DCNT e outras, como DCV (65.693), câncer (53.000: pulmão, cavidade oral, mama), doenças respiratórias crônicas, doenças respiratórias infecciosas e tuberculose, diabetes, doenças digestivas e renais (Figuras 11-5 e 11-6). ^19^

### Tabaco e Mortalidade por DCV

•A análise das causas de morte específicas atribuídas ao tabagismo indica que a mortalidade por DCV por 100 mil habitantes diminuiu de 88,0 (II 95%, 81,3 – 94,3) em 1990 para 26,3 (IC 95%, 23,8 – 28,9) em 2019. ^19^

•Em 2019, o tabagismo foi responsável por 65.696 mortes por DCV. Além disso, houve um decréscimo nas taxas de mortalidade de algumas DCV atribuídas ao tabagismo, como doença isquêmica do coração e AVC, no período estudado (Figuras 11-7 e 11-8). ^2^ Entre as DCV, a doença isquêmica do coração apresentou a maior redução na mortalidade atribuída ao tabagismo, passando de 41.564 mortes [47,2 (II 95%, 43,8 – 50,4) por 100 mil habitantes] em 1990 para 35.218 mortes [14,7 (II 95%, 16,0 – 13,5) por 100 mil habitantes] em 2019. ^19^

•As mortes por AVC passaram de 28.468 [31,8 (II 95%, 29,0 – 34,8) por 100 mil habitantes] em 1990 para 17.577 [7,4 (II 95%, 6,6 – 8,3) por 100 mil habitantes] em 2019. As mortes por aneurisma aórtico aumentaram de 1.678 [7,4 (II 95%, 1,7 – 2,0) por 100 mil habitantes] em 1990 para 3.999 [1,7 (II 95%, 1,5 – 1,9) por 100 mil habitantes] em 2019, enquanto as mortes por doença arterial periférica aumentaram de 343 [0,4 (II 95%, 0,2 – 0,8) por 100 mil habitantes] em 1990 para 674 mortes [0,3 (II 95%, 0,1 – 0,6) por 100 mil habitantes] em 2019. ^19^

•No geral, reduções mais significativas nas taxas de mortalidade atribuída ao tabagismo foram observadas nas UF com SDI mais altos, sendo as maiores reduções observadas naquelas com SDI mais elevados (Distrito Federal, Rio de Janeiro, São Paulo, Santa Catarina, Paraná, Minas Gerais) e as menores reduções observadas nos estados das regiões Norte e Nordeste, que têm os menores SDI (Rio Grande do Norte, Ceará, Bahia, Pará, Paraíba) (correlação de Pearson: 0,637; p < 0,001) ( Figura 11 -9). ^34^

## Carga de Doença

### Carga de Doença total do Tabaco

•As Figuras 11-10, 11-11 e 11-12 mostram as tendências de 1990 a 2019 para as métricas YLL, YLD e DALY. Em todas as situações, as taxas brutas e padronizadas por idade diminuíram. ^19^

•Dados do GBD 2019 estimaram uma redução na taxa de DALYs atribuídos ao tabaco padronizada por idade por 100 mil habitantes no Brasil de 59% (II 95%, 56% - 61%) entre 1990 [4.614,5 (II 95%, 4.372,3 – 4.888,0)] e 2019 [1.893,7 (II 95%, 1.768,6 – 2.028,0)]. As diferenças nas curvas da Figura 11 -12 refletem DALYs ajustados para diferenças na distribuição etária da população, sendo a curva ‘todas as idades’ influenciada por uma combinação de crescimento e envelhecimento populacional.

•A redução nas taxas de DALYs é consequência da diminuição dos YLLs em 61% (II 95%, 58 - 63%) entre 1990 e 2019. A combinação de diminuição da exposição ao tabagismo e taxas de DALYS registradas após remoção do tabagismo como fator de risco contribuiu para a redução geral.

•Houve uma redução heterogênea nas taxas de DALYs atribuídos ao tabaco padronizadas por idade nas diferentes UF e regiões do Brasil, sendo mais pronunciada nos estados do Sudeste, Sul e Centro-Oeste e Distrito Federal, modesta nas UF da região Norte e ainda mais discreta na maioria das UF do Nordeste.

### Carga de Doença Cardiovascular Atribuída ao Tabaco

•Dados do GBD 2019 estimaram uma taxa de DALYs atribuídos ao tabaco padronizada por idade por 100 mil habitantes no Brasil de 650 (II 95%, 604 - 701), uma redução de 69% (II 95%, 67 - 71) em comparação à de 1990, 2.124 (II 95%, 1.993 – 2.254).

•As taxas de YLL padronizadas por idade por 100 mil habitantes foram 2.040 (II 95%, 1.919 – 2.164) em 1990 e 611 (II 95%, 568 - 657) em 2019, uma redução de 70% (II 95%, 68% - 72%) no período.

•As taxas de YLD padronizadas por idade por 100 mil habitantes foram 84 (II 95%, 60 - 108) em 1990 e 39 (II 95%, 28 - 51) em 2019, uma redução de 54% (II 95%, 50-57%).

### Impacto Econômico do Tabaco

•O impacto econômico do tabaco no Brasil, incluindo o custo direto (custos de diagnóstico, tratamento e seguimento), foi recentemente estimado usando um modelo econômico de micro simulação probabilística sobre a história natural, os custos médicos e as perdas de qualidade de vida associados às doenças mais comuns relacionadas ao tabaco. Os dados foram obtidos através de revisão da literatura, estatísticas vitais e bancos de dados hospitalares. Doença pulmonar obstrutiva crônica, DCV, câncer de pulmão e AVC foram responsáveis por 78% daquele custo. Em 2015, o custo direto total do tabaco foi estimado em US$ 11,8 bilhões ao ano, 70% correspondendo ao custo direto associado com a assistência à saúde e o restante associado com o custo indireto por perda de produtividade por morte prematura e incapacidade. O tabaco representou 22% dos custos diretos de DCV no Brasil e 17% dos custos diretos de AVC. O custo atribuído ao tabagismo passivo foi US$ 1,36 bilhão. ^413^ ^,^ ^414^

•Os custos de saúde atribuídos ao uso do tabaco representam uma estimativa de 5,7% de todos os gastos de governo com saúde e 0,7% do produto interno bruto. Estima-se que, no Brasil, 25,6% dos recursos gastos sejam recuperados através dos impostos sobre o tabaco. ^413^ ^,^ ^414^

### Pesquisa Futura

•Devido à falta de dados longitudinais, os riscos de longo prazo de DCV associada ao uso de cigarro eletrônico são desconhecidos. Atualmente, há informação limitada sobre os riscos para a saúde dos produtos derivados do tabaco eletrônico. São necessários pesquisas adicionais e seguimento mais longo, pois o cigarro eletrônico, incluindo o narguilé eletrônico, tornaram-se prevalente entre os jovens.

•Finalmente, a despeito dos resultados positivos do programa de controle do tabagismo, avanços ainda são necessários no país. Isso inclui: medidas regulatórias (elevação de preços/impostos, espaço para advertência na embalagem, adoção de embalagem simples); medidas sociais (incentivo para que pequenos agricultores substituam o cultivo do tabaco por outros cultivos); e medidas legais (supervisão dos produtos de tabaco, controle das fronteiras e combate ao comércio ilegal, políticas de incentivo para criar fundos de controle do tabaco para compensar os custos com assistência à saúde relacionada ao tabagismo em âmbito federal e subnacional).
