# Supplementary material for: Estatística Cardiovascular – Brasil 2021
Source: Arq Bras Cardiol. 2022 Jan 1;118(1):115–373. [Article in Portuguese] doi: 10.36660/abc.20211012 (PMC8959063; doi:10.36660/abc.20211012)
Supplement: Translation

# Translation: Cardiovascular Statistics – Brazil 2021

**Authors:** Gláucia Maria Moraes de Oliveira, Luisa Campos Caldeira Brant, Carisi Anne Polanczyk, Deborah Carvalho Malta, Andreia Biolo, Bruno Ramos Nascimento, Maria de Fatima Marinho de Souza, Andrea Rocha De Lorenzo, Antonio Aurélio de Paiva Fagundes, Beatriz D. Schaan, Fábio Morato de Castilho, Fernando Henpin Yue Cesena, Gabriel Porto Soares, Gesner Francisco Xavier, Jose Augusto Soares Barreto, Luiz Guilherme Passaglia, Marcelo Martins Pinto, M. Julia Machline-Carrion, Marcio Sommer Bittencourt, Octavio M. Pontes, Paolo Blanco Villela, Renato Azeredo Teixeira, Roney Orismar Sampaio, Thomaz A. Gaziano, Pablo Perel, Gregory A. Roth, Antonio Luiz Pinho Ribeiro

**Affiliations:** 1 Brazil Instituto do Coração Edson Saad da Universidade Federal do Rio de Janeiro (UFRJ), Rio de Janeiro, RJ – Brazil. Instituto do Coração Edson Saad da Universidade Federal do Rio de Janeiro (UFRJ), Rio de Janeiro, RJ – Brazil; 2 Brazil Universidade Federal do Rio de Janeiro (UFRJ), Rio de Janeiro, RJ – Brazil Universidade Federal do Rio de Janeiro (UFRJ), Rio de Janeiro, RJ – Brazil; 3 Brazil Universidade Federal de Minas Gerais (UFMG), Belo Horizonte, MG – Brazil Universidade Federal de Minas Gerais (UFMG), Belo Horizonte, MG – Brazil; 4 Brazil Hospital das Clínicas da Universidade Federal de Minas Gerais (UFMG), Belo Horizonte, MG – Brazil Hospital das Clínicas da Universidade Federal de Minas Gerais (UFMG), Belo Horizonte, MG – Brazil; 5 Brazil Universidade Federal do Rio Grande do Sul (UFRS), Porto Alegre, RS – Brazil Universidade Federal do Rio Grande do Sul (UFRS), Porto Alegre, RS – Brazil; 6 Brazil Hospital Moinhos de Vento, Porto Alegre, RS – Brazil Hospital Moinhos de Vento, Porto Alegre, RS – Brazil; 7 Brazil Hospital de Clínicas de Porto Alegre (HCPA), Porto Alegre, RS – Brazil Hospital de Clínicas de Porto Alegre (HCPA), Porto Alegre, RS – Brazil; 8 USA Vital Strategies, New York – USA Vital Strategies, New York – USA; 9 Brazil Instituto Nacional de Cardiologia, Rio de Janeiro, RJ – Brazil Instituto Nacional de Cardiologia, Rio de Janeiro, RJ – Brazil; 10 USA Brigham and Women’s Hospital, Boston – USA Brigham and Women’s Hospital, Boston – USA; 11 Brazil Hospital Israelita Albert Einstein, São Paulo, SP – Brazil Hospital Israelita Albert Einstein, São Paulo, SP – Brazil; 12 Brazil Curso de Medicina da Universidade de Vassouras, Vassouras, RJ – Brazil Curso de Medicina da Universidade de Vassouras, Vassouras, RJ – Brazil; 13 Brazil Universidade Federal de Sergipe, Aracaju, SE – Brazil Universidade Federal de Sergipe, Aracaju, SE – Brazil; 14 Brazil Hospital São Lucas Rede São Luiz D’Or, Aracaju, SE – Brazil Hospital São Lucas Rede São Luiz D’Or, Aracaju, SE – Brazil; 15 Brazil ePHealth Primary Care Solutions, Santo Antônio, SC – Brazil ePHealth Primary Care Solutions, Santo Antônio, SC – Brazil; 16 USA Department of Medicine and Radiology University of Pittsburgh, Pittsburgh – USA Department of Medicine and Radiology University of Pittsburgh, Pittsburgh – USA; 17 Brazil Faculdade de Medicina de Ribeirão Preto da Universidade de São Paulo (USP), São Paulo, SP – Brazil Faculdade de Medicina de Ribeirão Preto da Universidade de São Paulo (USP), São Paulo, SP – Brazil; 18 Brazil Faculdade de Medicina da Universidade de São Paulo (USP), São Paulo, SP – Brazil Faculdade de Medicina da Universidade de São Paulo (USP), São Paulo, SP – Brazil; 19 Brazil Instituto do Coração (Incor) do Hospital das Clínicas da Faculdade de Medicina da Universidade de São Paulo (HCFMUSP), São Paulo, SP – Brazil Instituto do Coração (Incor) do Hospital das Clínicas da Faculdade de Medicina da Universidade de São Paulo (HCFMUSP), São Paulo, SP – Brazil; 20 USA Brigham and Women’s Hospital, Boston – USA Brigham and Women’s Hospital, Boston – USA; 21 USA Department of Medicine, Cardiovascular, Harvard Medical School, Boston – USA Department of Medicine, Cardiovascular, Harvard Medical School, Boston – USA; 22 Switzerland World Heart Federation, Geneva – Switzerland; 23 England Centre for Global Chronic Conditions, London School of Hygiene & Tropical Medicine, London – England Centre for Global Chronic Conditions, London School of Hygiene & Tropical Medicine, London – England; 24 USA Division of Cardiology, Department of Medicine, University of Washington, Washington – USA Division of Cardiology, Department of Medicine, University of Washington, Washington – USA

**Keywords:** Cardiovascular Diseases, Coronary Disease, Cardiomyopathies, Heart Failure, Heart Valve Diseases, Atrial Fibrillation, Atrial Flutter, Statistics, Brazil

## About these statistics

**Table:** Abbreviations Used in ‘About These Statistics’ and Highlights

AF	Atrial Fibrillation
AMI	Acute Myocardial Infarction
CABG	Coronary Artery Bypass Grafting
CI	Confidence Interval
CV	Cardiovascular
CVD	Cardiovascular Disease
DALY	Disability-Adjusted Life Year
FU	Federative Unit
GBD	Global Burden of Disease
GDP	Gross Domestic Product
HR	Hazard Ratio
HS	Hemorrhagic Stroke
IBGE	Brazilian Institute of Geography and Statistics (in Portuguese, *Instituto Brasileiro de Geografia e Estatística* )
ICD	International Statistical Classification of Diseases and Related Health Problems
IHD	Ischemic Heart Disease
IHME	Institute for Health Metrics and Evaluation
Int$	International dollars
IPCA	Brazilian Consumer Price Index (in Portuguese, *Índice de Preços ao Consumidor Amplo* )
IRR	Incidence Rate Ratio
IS	Ischemic Stroke
NRVD	Non-Rheumatic Valvular Heart Disease
OR	Odds Ratio
PCI	Percutaneous Coronary Intervention
PNS	National Health Survey (in Portuguese, *Pesquisa Nacional de Saúde* )
PPP	Purchasing Power Parity
R$	*Reais* , Brazilian currency
RHD	Rheumatic Heart Disease
SAH	Subarachnoid Hemorrhage
SDI	Sociodemographic Index
SIH	Brazilian Hospital Information System (in Portuguese, *Sistema de Informações Hospitalares* )
SIM	Brazilian Mortality Information System (in Portuguese, *Sistema de Informações sobre Mortalidade* )
SUS	Brazilian Unified Health System (in Portuguese, *Sistema Único de Saúde* )
UI	Uncertainty Interval
US$	US dollars
YLD	Year Lived with Disability
YLL	Year of Life Lost

This is the 2021 edition of the **Cardiovascular Statistics - Brazil** , a multi-institutional effort to periodically provide updated information on the epidemiology of heart diseases and stroke in Brazil. The report incorporates official statistics provided by the Brazilian Ministry of Health and other government agencies, by the GBD project led by the IHME of the University of Washington, as well as data generated by other sources and scientific studies, such as cohorts and registries, on CVDs and their risk factors. The document is directed to researchers, clinicians, patients, healthcare policy makers, media professionals, the public, and others who seek comprehensive national data available on heart disease and stroke.

The **Cardiovascular Statistics – Brazil 2021** is an updated and expanded version of the **Cardiovascular Statistics – Brazil 2020** , ^1^ published last year in the *ABC Cardiol.* It includes the most recent data on CVD as a group of conditions and five specific CVDs covered by the 2020 document, in addition to new chapters on CV risk behaviors and factors, specifically hypertension, diabetes, dyslipidemia, obesity, and smoking and tobacco use. The work was conducted by volunteer researchers from several Brazilian Universities and research institutions led by a steering committee of five members (ALPR, CAP, DCM, GMMO, and LCCB), with the support of the Brazilian Society of Cardiology and the collaboration of the GBD Brazil Network ^2^ and of an International Committee (GAR, PP, and TAG). The document follows the methodology used by the American Heart Association to produce the annual **Heart Disease & Stroke Statistics Update** , ^3^ which emphasizes epidemiological and public health data. The **Cardiovascular Statistics – Brazil** neither gives information on pathophysiological mechanisms nor makes treatment recommendations. Unlike guidelines and position papers, it intends to present the newest and best health-related metrics of CVD statistics of the Brazilian population.

Data used in the present document have four different sources: (a) the Brazilian Mortality and Hospital Information Systems, provided by the government; (b) the GBD 2019 estimates; ^4^ (c) the systematic review of the literature with emphasis on the publications of the last 10 years; (d) the health care utilization costs, based on the reimbursement tables from the Public Health System, adjusted for inflation and reported in both original currency units (Reais or US dollars in a specific year) and international dollars. The international dollars were converted to PPP adjusted to US$ 2019 (Int$ 2019) using the Campbell and Cochrane Economics Methods Group and the Evidence for Policy and Practice Information and Coordinating Centre cost converter. ^5^ Better explanation on how mortality rate estimates can vary depending on the source used (mortality information system or GBD datasets) can be seen elsewhere. ^6^

As expected, different or discordant metrics are sometimes presented for a single condition, considering that studies may have distinct methodologies or were conducted in different time periods, locations, and age ranges. These differences are unavoidable, and their possible reasons are always discussed in this document. Since many studies cover a long period of time and life expectancy increased in Brazil in the last decades, we used age-standardized rates, i.e., a weighted average of the age-specific rates per 100 000 persons, in which the weights are the proportions of people in the corresponding age groups of a standard population. The GBD age-standardization uses a global age pattern, although other sources may have used different reference populations. For most studies, race/skin color was used according to the IBGE definition, i.e., white, black, brown, yellow (oriental), and indigenous.

Cardiovascular disease is still responsible for nearly one third of deaths in Brazil and affects disproportionally the most vulnerable stratum of the population, which has marked difficulties in accessing high quality health care. ^7^ ^,^ ^8^ To have representative, reliable and extensive national data on CVD, risk behaviors and factors is an obligatory step towards overcoming these inequalities and providing the best possible CV care to all Brazilians. This study gathers this information, essential to individual care and to plan the next steps of health policy in Brazil. ^9^ In addition, it points out gaps in the knowledge to be filled with further studies. We all aspire for people to live longer and better. Knowing more about CV statistics to help tackle CVD is a good start to this goal.

## Main Brazilian Data Sources

A. For the present version of the **Cardiovascular Statistics - Brazil** document, the main Brazilian data sources were the Brazilian Mortality and Hospital Information Systems, periodic health surveys, such as the National Health Survey, and official population estimates. Brazilian Mortality Information System: In Brazil, the SIM, created in 1975 by the Brazilian Ministry of Health, is responsible for collecting, storing, managing, and disseminating national mortality data. This health information system represented a major advance in the country’s epidemiological surveillance, since its main task is to record all deaths occurring in the Brazilian territory. The Brazilian Ministry of Health implemented a Standard Certificate of Death model, a document for collecting information on death, that uses the ICD to code the causes of death. In addition, a flow of collection, processing, and distribution of death information has been implemented in all 5570 municipalities across the country. ^10^ ^,^ ^11^ The quality of statistics on causes of death in Brazil significantly improved in the last two decades, but data from the beginning of the 2000 decade are still of low quality, specifically in some parts of the country. ^12^ Knowing the heterogeneity of these indicators in Brazil, the **Brazilian Cardiovascular Statistics** report treated data to estimate information closer to real, by correcting for underreporting and redistribution of ill-defined causes of death. More details can be found in the article by Malta *et al* . ^6^

B. Brazilian Hospital Information System: The aim of the SIH database is to register all hospitalizations funded by the SUS. The SIH-SUS compiles the hospitalizations at the municipal level through the “Hospital Admission Authorization”, which has information about the diseases leading to hospitalization (using ICD-10), length of stay, procedures, and costs. ^13^ The SIH-SUS information allows the development of methodologies and the definition of indicators to identify geographical disparities related to hospital resources. ^14^

C. National Health Survey: When the statistics for CV risk factors are cited, a preference for the PNS survey was made. The PNS is a household-based epidemiological survey, representative of Brazil, its large regions, FUs, metropolitan regions, capitals, and other municipalities in each FU. The PNS 2013 sample was composed of 64 348 households. The survey was carried out by the IBGE in partnership with the Ministry of Health. Most health topics were included, such as noncommunicable diseases, risk factors, elderly, women, children, use of health services, health inequalities, anthropometric features, laboratory tests, and blood pressure measurements. ^15^ The PNS data are used by the GBD in its estimates for Brazil.

D. For population estimates, the most updated population estimates generated by the IBGE (www.ibge.gov.br) were used in the denominator. For the hospitalizations and cost analyses, the resident population estimated for the National Audit Office yearly, from 2008 to 2019, was used.

## GBD 2019

The GBD Study ( http://www.healthdata.org/gbd ) is the most comprehensive worldwide observational epidemiological study to date. It describes mortality and morbidity from major diseases, injuries, and risk factors to health at global, national, and regional levels. Examining trends from 1990 to the present and making comparisons across populations enable us to understand the changing health challenges faced by people across the world in the 21st century. The GBD 2019 is the latest publicly available dataset. ^4^ ^,^ ^16^ The GBD Brazil network has been collaborating with the IHME, which leads the project in the world, in the identification and provision of datasets, revision of models and estimates, and validation and publication of the results for Brazil. ^20^ ^,^ ^21^ Details on how the estimates are calculated can be obtained in the capstone papers of the GBD Study and in the IHME website ( http://www.healthdata.org/acting-data/what-we-measure-and-why ). We summarize below the main estimates used in this document:

A. Estimates of deaths and causes of deaths. The main source of information is the SIM, a database from the Brazilian Ministry of Health, adjusted to other national and international sources. The IHME used methods for correcting for underreporting of deaths and “garbage codes” deaths according to previously published algorithms, ^22^ updated in the newer versions of the study ( http://www.healthdata.org/acting-data/determining-causes-death-how-we-reclassify-miscoded-deaths ).

B. The YLLs are years lost due to premature mortality. The YLLs are calculated by subtracting the age at death from the longest possible life expectancy for a person at that age. For example, if the longest life expectancy for men in a given country is 75 years, and a man dies of cancer at 65, this would be 10 years of life lost due to cancer.

C. The YLDs can also be described as years lived in less-than-ideal health. This includes conditions such as influenza, which may last for only a few days, or epilepsy, which can last a lifetime. It is measured by taking the prevalence of the condition multiplied by the disability weight for that condition. Disability weights reflect the severity of different conditions and are developed through surveys with the general population.

D. The DALY is a universal metric that allows researchers and policymakers to compare very different populations and health conditions across time. The DALYs equal the sum of YLLs and YLDs. One DALY equals one lost year of healthy life. The DALYs allow us to estimate the total number of years lost due to specific causes and risk factors at the country, regional, and global levels.

## Systematic Review of the Literature

Descriptors for the elaboration of search strategies were selected in MeSH and DeCS, the controlled vocabularies from MEDLINE and LILACS, respectively. Embase’s plan was designed with Emtree descriptors associated with MeSH. Free terms were also used, that is, significant keywords and their synonyms, spelling variations, and acronyms that are essential for searching in the searched domain, but which are not controlled descriptors (or are not in the synonym list of these descriptors). Importantly, to maintain search uniformity, the same descriptors were used in all search strategies. However, search strategies were customized according to the specifics of each database. In addition, it is worth noting that the terms related to “Brazil” were generally searched in all fields of research (subject, author, title, institutional affiliation, journal name, etc.).

The selected bases for research were MEDLINE/PubMed, Embase, LILACS, CINAHL, Cochrane Library Scopus, and Web of Science. The following bibliographic research filters and limits were used: period of publication (2004-2020); languages (Portuguese, English and Spanish); type of study/publication (Review, Meta-Analysis, Clinical Trial, Randomized Controlled Trial, Comparative Study, Practice Guideline, Guideline, Systematic Review, Evaluation Studies, Government Publications, and Multicenter Study). All references were managed using EndNote Web. From the search, articles were included if the study were population- or community-based; nation- or state-wide studies were preferred. Moreover, articles set at health services or hospitals were included if the study was multicenter and had an adequate sample size (>200 participants was the suggested cut-off), preferably. In addition to the articles identified by the systematic search, the authors could include other studies found in the references of the searched articles or other articles they were aware of in their area of expertise, if the studies fulfilled the criteria above mentioned. Finally, which studies should be described in each chapter was mostly a decision of the experts commissioned to the specific theme.

## Healthcare Utilization

Healthcare costing studies have expressive methodologic variability and need to be carefully interpreted. In the present document, most of the cost data were gathered from the Public Health System reimbursement tables from 2008 to 2019. During this period, adjustment for inflation was performed neither regularly nor homogeneously across the CVD groups or procedures, thus the crude values presented were not adjusted to actual inflation.

To minimize biases in reporting and interpreting cost data, a systematic approach was applied to all chapters. Overall costing studies were described in original units (Reais or US dollars in a specific year) and international dollars. International dollars were converted to PPP adjusted to 2019 US dollars (Int$ 2019) using the Campbell and Cochrane Economics Methods Group and the Evidence for Policy and Practice Information and Coordinating Centre cost converter (https://eppi.ioe.ac.uk/costconversion/default.aspx). A two-stage approach is applied in this method. First, it adjusts the original estimate of cost from the original price-year to a target price-year, using a GDP deflator index (GDPD values). Second, it converts the price-year adjusted cost estimate in the original currency to a target currency, using conversion rates based on PPP for GDP (PPP values). ^5^ For original economic studies, when the base year of the currency was not reported or could not be inferred from the manuscript (e.g. the last year of data collection), the recommendation was to assume the year before the publication of the paper.

## Highlights

### Total Cardiovascular Disease

• According to both the GDB Study 2019 and the SUS database, CVDs are the number 1 cause of death in Brazil. Of the CVDs, IHD was the leading cause of death in the country, followed by stroke in 1990 and 2019.

• According to the GBD Study 2019, CVD prevalence was estimated in 6.1% of the population and has increased from 1990 due to population growth and aging. However, the age-standardized CVD prevalence and incidence rate in Brazil decreased in the same period.

• A reduction in age-adjusted mortality rate from 1990 to 2019 was observed in all FUs, although less impressive in the North and Northeastern as compared to the other regions.

• Age-standardized DALY rates in Brazil decreased from 1990 to 2019, and there was a correlation between the percent decline in DALY rates and the increase in the SDI: the higher the SDI, the greater the decline in DALYs due to CVD.

• Data from the SUS database showed a significant number of clinical and surgical CV procedures paid, led by heart failure, cerebrovascular diseases, and acute coronary syndrome. Hospitalizations for PCI significantly increased in the last decades, while surgical procedures remained stable.

### Stroke

• According to a community-based study performed in the city of Matão in 2003-2004 and 2015-2016, the age-adjusted stroke incidence decreased by 39% (IRR 0.61; 95% CI, 0.46–0.79) and mortality by 50% (IRR 0.50; 95% CI, 0.31– 0.94). The mean age of stroke patients increased by 9%, from 65.2 (95% CI, 62.6–67.8) to 71.0 (95% CI, 68.1–73.8) years. The 1-year case fatality was 26%; approximately 56% of the patients were functionally independent, while 7% had a recurrent stroke.

• Regarding the distribution of stroke subtypes, according to the Joinvasc Registry performed in the city of Joinville, from 1995 to 2013, the proportion of IS increased 12%, whereas that of HS decreased 16%. Meanwhile, the proportion of SAH remained relatively stable, ranging from 7.5% in 1995 to 6% in 2012–2013. In the last 8 years, the incidences of IS and HS showed significant decreases of 15% (95% CI, 1–28) and 60% (95% CI, 13–86), respectively.

• According to data from the GBD Study 2019, the age-standardized mortality rate from stroke per 100 000 in 1990 was 137.8 (127.8 to 144) and, in 2019, 58.1 (52.6 to 61.8), representing a percent change of -57.8 (-60.4 to -0.6). The highest percent change occurred in Goiás, -65.9 (-71.8 to -0.6), and the lowest in Maranhão, -22.7 (-37.2 to 0). For adults, the highest percent change was observed among people aged 50-69 years, -61 (-603.6 to -0.6).

• Considering the burden of stroke in Brazil, the GBD Study 2019 also observed a prominent decrease in YLL: the age-standardized YLL rates due to stroke per 100 000 in 1990 was 2778.6 (2659.5 to 2879.2) and, in 2019, 1098.7 (1025.8 to 1153.7), representing a percent change of -60.5 (-62.7 to -0.6). For adults, the highest percent change was observed among people aged 50-59 years, -61.7 (-64.3 to -0.6).

### Acute and Chronic Coronary Artery Disease

• According to the GBD Study 2019, the number of individuals with IHD (AMI, stable angina, or ischemic heart failure) in Brazil increased from 1.48 million in 1990 to more than 4 million in 2019, and the crude IHD prevalence rate increased from 0.99% to 1.85% in the period, although age-standardized prevalence rates remained stable.

• In 2019, there were 171 246 deaths due to IHD in Brazil, corresponding to 12% of total deaths in the country and 43% of all CVD deaths. IHD was the number one cause of death in Brazil in all but two FUs. A reduction in age-adjusted mortality rate from 1990 to 2019 was observed in all FUs, although less impressive in the Northeastern as compared to the other regions.

• According to the SUS database, the number of hospitalizations due to AMI in the public system increased by 54% from 2008 to 2019, adjusted for the population. Non-primary PCIs per inhabitants doubled, while primary PCIs increased by 31%. Meanwhile, the total number of CABGs remained stable in the period. The in-hospital mortality rate for AMI decreased from 15.9% in 2008 to 12.9% in 2019; for acute coronary syndrome, rates were stable during that period, as well as for PCI and CABG procedures.

### Cardiomyopathy and Heart Failure

• According to GBD Study 2019 estimates, the age-standardized prevalence of cardiomyopathy and myocarditis decreased in Brazil from 76.6 (95% UI, 53.4-107.2) in 1990 to 73.0 (95% UI, 51.1-100.1) in 2019, a decrease of 4.7% (95% UI, - 9.5 to 0.8) in the period. In absolute numbers, estimates of the prevalence of cardiomyopathy and myocarditis in Brazil increased from less than 60 000 in 1990 to over 160 000 in 2019, mainly due to population growth and aging. The cardiomyopathy and myocarditis prevalence was greater in men (98.9; 95% UI, 69.5-137.2) than in women (54.1; 95% UI, 38.4-73.8) in 2019.

• In the NIH REDS-II Chagas retrospective cohort study, initially healthy blood donors with an index *T. cruzi* -seropositive donation and age, sex, and period-matched seronegative donors were followed up for 20 years. The differential incidence of cardiomyopathy was 1.85 per 100 person-years attributable to *T. cruzi* infection in the first 10 years of follow-up, and 0.9 per 100 person-years in the following 10 years. A *T. cruzi* antibody level in the second visit was associated with the development of cardiomyopathy (adjusted OR, 1.4; 95% CI 1.1-1.8) in the last visit.

• According to the GBD Study 2019, the number of deaths due to Chagas disease in Brazil decreased from 7903 (95% UI, 2438-10 073) in 1990 to 6523 (95% UI, 3350-11 226) in 2019. The age-standardized mortality rate showed a more striking decrease (-67.5% change), from 8.6 (95% UI, 2.8-10.9) deaths per 100 000 inhabitants in 1990 to 2.8 (95% UI, 1.8-4.8) per 100 000 inhabitants in 2019, accounting for 1.6% of all CV deaths in the country.

• According to data from the SUS, there were 3 085 359 hospitalizations due to heart failure from 2008 to 2019. This number represents one-third of total clinical admissions related to CV conditions in the period studied, when there was a reduction in the number of clinical admissions due to heart failure from 298 474 (157 per 100 000) in 2008 to 222 620 (105 per 100 000) in 2019, with an even reduction over the years. Despite that reduction in the number of admissions, unadjusted healthcare expenditure estimates from the direct payment for the care of heart failure patients increased from 2008 to 2019 by almost 32%, from R$ 272 280 662 (2019 Int$ 267 102 469) in 2008 to R$ 359 301 691 (2019 Int$ 173 659 589) in 2019. Heart failure accounted for most costs related to clinical admissions due to CVD.

### Valvular Heart Disease

• According to the GBD Study 2019, the pattern of valvular heart disease has been changing in Brazil: age-standardized prevalence was stable from 1990 to 2019 for RHD, but a marked increase of over 50% was observed for NRVD, especially for men and older age groups. Among specific valve diseases, there was a marked 201.8% increase of calcific aortic valve disease, reinforcing the impact of population aging.

• RHD showed a marked decrease in age standardized mortality (-59.4%) from 1990 to 2019, while a milder decrease was observed for NRVD, 16.2% (95% UI, 10.3-22.5). However, crude mortality rates increased significantly for older ages (>70 years), associated with degenerative aortic valve disease, suggesting a growing burden to the health systems and urging specific actions to minimize impacts.

• The burden attributable to valvular heart disease in Brazil remains socioeconomically driven, with significant negative correlations between changes in the age-standardized mortality rates associated with RHD and the SDI in 1990 and 2019, in addition to positive correlations between NRVD mortality rates and the SDI in both years.

• Expenses with valvular heart disease by the Brazilian public health system decreased proportionally from 2008 to 2019 (-6.3% and -28% for clinical and interventional admissions, respectively), due to inflation and monetary correction. This constraint urges discussions towards a budgetary revision, avoiding the deferral or restriction of interventional and surgical procedures and allowing for the incorporation of new technologies and devices.

• Despite the improvement in the past decades, RHD remains as an important cause of morbidity and mortality in Brazil: data from the SUS database show that besides being the etiology of nearly half of the valve surgeries in the public health system – associated with higher hospital mortality – the prevalence of subclinical disease among schoolchildren proved to be high (4.5%), with the implementation of large-scale screening programs in 2014. Thus, coordinated actions for early diagnosis and prophylaxis are needed to avoid disease progression and late sequelae.

### Atrial Fibrillation

• According to the GBD Study 2019, the age-standardized prevalence rates due to AF and atrial flutter increased slightly in Brazil from 519 (95% UI, 393-669) in 1990 to 537 (95% UI, 409-692) in 2019, per 100 000 inhabitants, for both sexes, with 3.5% (95% UI, 1.8-5.1) change in the period.

• In a 10-year follow-up of 1462 individuals aged ≥ 60 years (mean age, 69 years; 61% women) included in the Bambuí Cohort Study in 1997, AF or flutter was independently associated with an increase in all-cause mortality (HR, 2.35; 95% CI, 1.53-3.62) among patients with and without Chagas disease.

• Data from the Telehealth Network of Minas Gerais with ECGs of 1 558 421 individuals (mean age, 51±18 years; 40.2% men) performed between 2010 and 2017 revealed in multivariable models adjusted for age and sex that the following self-reported comorbidities and risk factors related to the presence of AF: Chagas disease (OR 3.08; 95% CI, 2.91-3.25), previous myocardial infarction (OR 1.74; 95% CI, 1.56-1.93), chronic obstructive pulmonary disease (OR 1.48; 95% CI, 1.33-1.66), hypertension (OR 1.31; 95% CI, 1.27-1.34), and dyslipidemia (OR 1.09; 95% CI, 1.03-1.16). Current smoking and diabetes were not associated with prevalent AF.

• Of all 429 cases of stroke (87.2% ischemic strokes) that occurred in the city of Joinville in 2015 and were included in a registry, AF was detected in 11.4% of all patients and in 58% of the cardioembolic strokes. Similarly, AF was detected in 58% of 359 patients with cardioembolic stroke from a single-center, consecutive sample in the city of Curitiba, Brazil.

### Hypertension

• Analysis of the 2013 PNS showed a 22.8% prevalence of measured hypertension in individuals older than 18 years in a sample of 59 402 individuals. In those older than 75 years, the estimated prevalence was 47.1%. In the age groups between 18 and 74 years, the prevalence was higher in men, while women showed a slight predominance only in the age group above 75 years. The analysis by region showed that the Southeastern (25%) and Southern (25%) regions had the highest prevalence for both sexes.

• Using data from the 2013 PNS, 36% of the Brazilians included had a previous diagnosis and/or measured blood pressure ≥140/90 mm Hg. Of these, 89% had contacted the health system in the previous 2 years, but only 65% were aware of their condition. From those aware of their hypertensive condition, 62% regularly sought care, 92% of whom had been prescribed medications. Of those who reported receiving medications, only 56% reported that ongoing care for their condition was free of barriers and included advice about managing important risk factors and behavior. Of the entire hypertensive population, about 33% had their blood pressure under control.

• According to data from participants in the Brazilian Study of Cardiovascular Risks in Adolescents (ERICA), of 73 399 students evaluated, 55.4% were female and the mean age was 14.7 ± 1.6 years. The prevalence of hypertension was 9.6%, with the lowest in the Northern (8.4%) and Northeastern regions (8.4%), and the highest, in the Southern region (12.5%). Obese adolescents had a higher prevalence of hypertension (28.4%) than overweight (15.4%) or eutrophic adolescents (6.3%). The proportion of hypertension due to obesity was estimated in 17.8%.

• In the ELSA-Brasil cohort study, which included 7063 patients with a mean age of 58.9 years at baseline (2008-2010), hypertension was associated with the greatest decline in memory, fluency, and global cognitive score. Prehypertension was also an independent predictor of greater decline in the verbal fluency test and global cognitive score. Moreover, among treated individuals, blood pressure control at baseline was inversely associated with the decline in both global cognitive and memory test scores.

### Diabetes mellitus

• Considering the International Diabetes Federation data published in 2019, Brazil ranked 5th regarding the number of adults with diabetes worldwide, totaling 16.8 million (95% CI, 15.0 – 18.7) people, 46% of whom were not aware of their disease. The prevalence of prediabetes was 9.5% (15.1 million people).

• According to GBD 2019, mortality from CVD attributable to diabetes for all ages in Brazil increased in absolute numbers from 50 812 deaths (95% UI, 35 649 -73 137) in 1990 to 80 754 (95% UI, 55 922 – 118 175) in 2019. However, the age-standardized mortality rates per 100 000 inhabitants decreased from 70.4 (95% UI, 47.4 – 106.1) in 1990 to 35.9 (95% UI, 24.5 – 53.0) in 2019, a reduction of -49.0% (95% UI, -53.4 to -43.9).

• Regarding the burden of CVD attributable to diabetes, the age-standardized DALY rates decreased by -47.4% (95% UI, -52.2 to -41.9) per 100 000 inhabitants from 1990 to 2019, despite the increase in the total number of DALYs from 1 072 309 (95% UI: 784 276 - 1 484 959) to 1 571 116 (95% UI: 1 140 912 – 2 203 188) in the same period. There was a heterogeneous reduction in the age-standardized DALY rates attributable to diabetes among the Brazilian FUs and regions.

### Dyslipidemia

• According to the PNS 2014-2015, the prevalence of dyslipidemia in Brazil is still high: total cholesterol ≥ 200 mg/dL in 32.7% (95% CI, 31.5 - 34.1) of the general population; low HDLc in 31.8% (95% CI, 30.5 – 33.1), and high LDLc in 18.6% (95% CI, 17.5 – 19.7). Greater level of education was related to lower prevalence of high total cholesterol, high LDLc, and low HDLc.

• According to the GBD Study 2019, when analyzing trends from 1990 to 2019, an increase was observed in the absolute numbers of deaths, YLLs, and DALYs, with a decrease in the age-standardized rates for those same metrics in all states and at national level.

• A Brazilian cohort (ELSA-Brasil study) assessed familial hypercholesterolemia and showed a prevalence of 1 in 263 individuals, but data on burden of disease and impact on cost are still lacking.

• Awareness of dyslipidemia according to the ELSA-Brasil is also low (58.1% of individuals with elevated LDLc), with only 42.3% of those individuals receiving medical treatment. Only 58.3% of the individuals on some kind of lipid-lowering medication achieved target serum lipid level.

### Obesity and Overweight

• According to data from IBGE, in Brazil, the percentages of adults (age ≥18 years) with excess weight and obesity in 2019 were, respectively, 57.5% (95% CI, 54.8 – 60.2) and 21.8 % (95% CI, 19.2 – 24.7) for men, and 62.6% (95% CI, 59.1 – 66.0) and 29.5% (95% CI, 25.4 – 34.0) for women. Progressive increase of obesity was observed with age increase, ranging from 10.7% (95% CI, 7.7 – 14.7) [male: 7.9% (95% CI, 4.8 – 12.8); female: 13.5% (95% CI, 8.8 – 20.4)] in the age group of 18-24 years to 34.4% (95% CI, 29.7 – 39.4) [male: 30.2% (95% CI, 24.8 – 36.3); female: 38.0% (95% CI, 32.3 - 44.0)] in the age group of 40-59 years. It is worth noting the higher prevalence of excess weight and obesity in the female sex for all age groups.

• From 1990 to 2019, there was a negative change in the mortality rates from CVD attributable to high body mass index for women [-33.9 (-43.7; -16.7)], which was higher than that for men [-22.8 (-35.9;6.2)]. The highest decreases in the percentage of mortality occurred in the FUs with higher income in Brazil.

• Most FUs had a decrease in the age-standardized rates of DALYs due to CVD attributed to high body mass index for women in the period. Similar behavior was observed in those rates for men, with a percent decrease of obesity from 1990 to 2019.

• Most public policies have failed to reduce obesity in adults and children, probably because obesity is multifactorial and involves many socioeconomic interests. Obesity is a pandemic, with impact on both developed and developing countries and consequences for the individual, social, familial, and financial levels. Nationwide registries of measured obesity should be built to enable the development of more effective public policies to control obesity, which has been increasing in Brazil in both sexes and several age groups.

### Smoking and Tobacco Use

• PNS 2019 data indicate that 12.8% (95% CI, 12.4 - 13.2%) of adults use some tobacco product, being the use higher among males (16.2%; 95% CI, 15.6 - 16.9%) than among females (9.9%; 95% CI, 9.3 - 10.3%). Considering the Vigitel household survey, there was a significant decrease in the prevalence of smoking in the adult population, with a 37.6% reduction from 2006 to 2019. However, there was a 0.5% increase in the prevalence from 2018 to 2019, suggesting a change in the trend and calling for attention.

• According to the GBD Study 2019, there was a reduction of 58.8% (95% UI, 56.2 - 61.1) in the smoking-attributable total mortality rate in Brazil from 1990 to 2019. The same trend was observed for both men and women, and in all Brazilian FUs. Likewise, the smoking-attributable CV mortality reduced by near 70% in the same period.

• The tobacco CV burden of disease decreased from 1990 to 2019, with a 69% (95% UI, 56 - 61) reduction in the age-standardized DALY rate. There was a heterogeneous reduction in the age-standardized DALY rates attributed to tobacco in the different FUs and regions of Brazil, more pronounced in the Southeastern, Southern and West-Central FUs, with a modest reduction in the Northern FUs and an even more discreet reduction in most Northeastern FUs.

• In a study using Markov probabilistic microsimulation economic model, the total direct cost of tobacco was estimated at US$ 11.8 billion per year, 70% corresponding to the direct cost associated with health care and the remainder associated with the indirect cost due to loss of productivity due to premature death and inability. Tobacco represented 22% of the direct costs of CVD in Brazil and 17% of the direct costs of stroke.

## 1. TOTAL CARDIOVASCULAR DISEASES

### ICD-9 390 to 459; ICD-10 I00 to I99.

**See Table 1-1 through 1-13 and Charts 1-1 through 1-16**

**Table:** Abbreviations Used in Chapter 1

AHA	American Heart Association
AMI	Acute Myocardial Infarction
CABG	Coronary artery bypass grafting
CI	Confidence Interval
CVD	Total Cardiovascular Diseases
DALYs	Disability-Adjusted Life Years
DATASUS	Brazilian Unified Health System Database
ELSA-Brasil	The Brazilian Longitudinal Study of Adult Health (in Portuguese *, Estudo Longitudinal de Saúde do Adulto* )
FHP	Family Health Program
FU	Federative Units
GBD	Global Burden of Disease
GDP	Gross Domestic Product
HDI	Human Development Index
HDIm	Municipal Human Development Index
HF	Heart Failure
IBGE	Brazilian Institute of Geography and Statistics
ICD	International Statistical Classification of Diseases and Related Health Problems
IHD	Ischemic Heart Disease
NCD	Noncommunicable Chronic Diseases
NHS	National Health System
OR	Odds Ratio
PAR	Population Attributable Risks
RR	Relative Risk
SDI	Sociodemographic Index
SIDRA	IBGE Automated Retrieval System (in Portuguese, *Sistema IBGE de Recuperação Automática)*
SIM	Brazilian Mortality Information System (in Portuguese, *Sistema de Informações sobre Mortalidade* )
SUS	Brazilian Unified Health System (in Portuguese, *Sistema Único de Saúde* )
UI	Uncertainty Interval

**Table 1-1:** – Number of prevalent cases and age-standardized prevalence rates of cardiovascular diseases, per 100 000 inhabitants, and percent change of rates, according to age group and sex, in Brazil, in 1990 and 2019.

Cause of death and age group	1990		2019		Percent Change (95% UI)
	Number (95% UI)	Rate (95% UI)	Number (95% UI)	Rate (95% UI)	
**Both**					
15-49 years	164612.9 (144806.1;186400)	214.8 (188.9;243.2)	215729.8 (190813.1;243619.6)	186.8 (165.2;210.9)	-13 (-15.8;-10.1)
50-69 years	218837.1 (196759.4;243342.6)	1395 (1254.2;1551.2)	450185.8 (406572.8;498758)	1115.9 (1007.8;1236.3)	-20 (-22.6;-17.2)
5-14 years	50036.9 (33169.3;71176.5)	141.6 (93.9;201.4)	45954.6 (30182.4;65586.3)	142.5 (93.6;203.4)	0.6 (-1.7;3.1)
70+ years	147631.3 (132225.3;164119.3)	3490 (3125.8;3879.8)	372585.5 (336268;412493)	2846.7 (2569.2;3151.6)	-18.4 (-21;-15.7)
Age-standardized	593711.1 (555922.5;636828.7)	593.7 (558.1;636.4)	1095891 (1030085.3;1166807.2)	474.9 (447.1;506.5)	-20 (-21.7;-18.4)
All Ages	593711.1 (555922.5;636828.7)	398.9 (373.5;427.9)	1095891 (1030085.3;1166807.2)	505.8 (475.4;538.5)	26.8 (23.3;30.4)
Under 5	12592.9 (9098.3;17056.8)	74.3 (53.7;100.7)	11435.3 (8280.9;15433.2)	73.8 (53.4;99.6)	-0.7 (-3.6;2.2)
**Female**					
15-49 years	81840.1 (71524.9;92783.6)	210.2 (183.7;238.3)	105700 (92430.3;120336.2)	180.7 (158.1;205.8)	-14 (-16.8;-10.9)
50-69 years	102496.1 (91526.7;114514.7)	1255.7 (1121.3;1402.9)	208399.3 (186607.1;232143.7)	973.2 (871.4;1084)	-22.5 (-25;-19.8)
5-14 years	26514.1 (17557.2;37666.8)	151.7 (100.4;215.5)	24476.1 (16014.6;34617.6)	154.6 (101.1;218.6)	1.9 (-1.4;5.7)
70+ years	77895.9 (69702.8;86822.5)	3321.7 (2972.3;3702.4)	200343.1 (180509.1;222408)	2653.3 (2390.6;2945.5)	-20.1 (-22.9;-17.2)
Age-standardized	294962.9 (275518.3;317426.8)	557.5 (523.8;597.3)	544515.2 (512491.4;581529.1)	437.4 (411;468.6)	-21.5 (-23.3;-20)
All Ages	294962.9 (275518.3;317426.8)	391.9 (366;421.7)	544515.2 (512491.4;581529.1)	491.1 (462.3;524.5)	25.3 (21.7;29.1)
Under 5	6216.7 (4434;8521.4)	74.5 (53.1;102.1)	5596.8 (3974;7644)	73.8 (52.4;100.8)	-1 (-4;2.2)
**Male**					
15-49 years	82772.8 (72882;93235)	219.5 (193.3;247.2)	110029.8 (97839.1;123670.6)	193 (171.6;216.9)	-12.1 (-15.2;-8.8)
50-69 years	116341 (104908;129351.1)	1546 (1394.1;1718.9)	241786.5 (218258.2;268733.5)	1277.4 (1153.1;1419.7)	-17.4 (-20.2;-14.2)
5-14 years	23522.8 (15573.9;33681.3)	131.8 (87.2;188.7)	21478.5 (14174.7;31018.6)	130.9 (86.4;189)	-0.7 (-3.4;2)
70+ years	69735.4 (62169.2;77641.2)	3699.5 (3298.1;4118.9)	172242.4 (155338.1;190212.2)	3110.3 (2805.1;3434.8)	-15.9 (-18.7;-12.9)
Age-standardized	298748.2 (279373.3;320526.9)	635.3 (595.3;681.3)	551375.8 (517313.3;587391.2)	520.8 (489.5;554.7)	-18 (-19.8;-16.3)
All Ages	298748.2 (279373.3;320526.9)	406.1 (379.8;435.7)	551375.8 (517313.3;587391.2)	521.2 (489;555.2)	28.3 (24.6;32.2)
Under 5	6376.2 (4668.2;8561.1)	74.2 (54.3;99.6)	5838.5 (4282.6;7786)	73.8 (54.1;98.4)	-0.5 (-4.1;3.5)
Grand Total	8978586.3 (7687971.2;10466915.3)	46326.6 (38506.4;55504.7)	15575150.5 (13460371.5;18007447.8)	37817.9 (31606;45148.7)	3435.8 (1527.7;5473.2)

*Source: Data derived from Global Burden of Disease Study 2019, Institute for Health Metrics and Evaluation, University of Washington. ^46^*

**Table 1-2:** – Number of incident cases, age-standardized incidence rates of cardiovascular diseases (per 100 000 inhabitants), and percent change of rates in Brazil and its Federative Units, in 1990 and 2019.

Cause of death and Location	1990		2019		Percent Change (95% UI)
	Number (95% UI)	Rate (95% UI)	Number (95% UI)	Rate (95% UI)	
**B.2-Cardiovascular diseases**					
Brazil	593711.1 (555922.5;636828.7)	593.7 (558.1;636.4)	1095891 (1030085.3;1166807.2)	474.9 (447.1;506.5)	-20(-21.7;-18.4)
Acre	1206.9 (1109.3;1324)	549.1 (513.6;588)	3266 (3049.1;3501.1)	471.7 (441.9;504.1)	-14.1(-16.4;-11.8)
Alagoas	9183 (8506.9;9969.2)	581.1 (541.6;625.5)	15952 (14961.2;17073.7)	484.3 (454.2;517.6)	-16.7(-19;-14.3)
Amapá	716.8 (656.5;788.6)	520.5 (486.1;559)	2653.7 (2476.6;2862.4)	440.7 (411.6;473.2)	-15.3(-17.5;-13.2)
Amazonas	5629.9 (5174.7;6164.5)	531.1 (496.3;569.2)	14473.2 (13534.3;15528.2)	451.3 (422.1;482.1)	-15(-17.2;-12.7)
Bahia	44112.1 (41105.1;47717.9)	562.8 (525.9;603.5)	75928.2 (71378.4;80995.6)	473.7 (444.5;506.6)	-15.8(-18.2;-13.8)
Ceará	24076.6 (22398.9;25978.9)	524.8 (491.1;560.8)	45521.1 (42602.7;48610)	458.7 (429.6;490.7)	-12.6(-14.8;-10.4)
Distrito Federal	4505.6 (4168.8;4888.6)	550.4 (515.5;590.1)	11843.1 (11093.6;12666.6)	433.2 (406.9;463.9)	-21.3(-23.6;-19.1)
Espírito Santo	10124.4 (9476.6;10907.6)	597 (558.7;639.6)	20329.8 (19077.6;21677.9)	475.9 (446.4;508.7)	-20.3(-22.7;-17.8)
Goiás	14164.8 (13166.4;15345)	559.3 (522.7;600.2)	30538.9 (28602.7;32638.9)	440.8 (412.6;470.9)	-21.2(-23.5;-19)
Maranhão	16322.6 (15071;17739.8)	528.8 (493.8;568.2)	31679.3 (29575.4;34020.2)	453.8 (424.3;486.7)	-14.2(-16.3;-11.9)
Mato Grosso	5861.4 (5416;6376.9)	560.1 (523.8;603.2)	15572.9 (14640.8;16594.5)	462.1 (433.4;493.7)	-17.5(-19.8;-15.3)
Mato Grosso do Sul	6310.2 (5890.9;6820.4)	582.4 (545;624.2)	14125.2 (13252.9;15040.8)	483.8 (453;515.7)	-16.9(-19.2;-14.7)
Minas Gerais	71837.8 (67116.6;77197)	662.2 (620.6;708.3)	134221.7 (125539.7;143761.7)	526.5 (492.9;563)	-20.5(-22.8;-18.4)
Pará	14537.4 (13368.9;15902.3)	540 (505.1;579.9)	33318.7 (31088.5;35782.7)	443.4 (414;475.7)	-17.9(-20.2;-15.7)
Paraíba	13732.3 (12775.8;14788.3)	549.2 (513.2;590)	20836.7 (19578.6;22302.1)	449.3 (420.7;482.1)	-18.2(-20.8;-16)
Paraná	33688.5 (31469.4;36286)	607.4 (567.4;652)	61307.5 (57604;65450.1)	476.9 (448.7;509.1)	-21.5(-23.7;-19.2)
Pernambuco	29154.2 (27148.1;31474)	575.5 (540.2;616.7)	48609.7 (45664.2;51831.3)	486.1 (455.6;518.8)	-15.5(-17.8;-13.5)
Piaui	9035.6 (8358.6;9805.9)	543.1 (506.4;584.1)	17353.8 (16310.2;18496.1)	464.3 (436.1;495.3)	-14.5(-16.9;-12)
Rio de Janeiro	64591.8 (60558.2;69272.2)	635.4 (597.7;678.7)	100862 (94708.7;107521.3)	473.2 (445;504.7)	-25.5(-27.7;-23.5)
Rio Grande do Norte	9424.3 (8784.4;10171.1)	530.4 (495.3;570.5)	17234 (16146;18374.5)	450.5 (422;481.3)	-15.1(-17.3;-12.6)
Rio Grande do Sul	43143.4 (40538.5;46255.1)	615.4 (577.1;659.5)	70448.5 (66035.9;75279.1)	482.5 (454.4;515.7)	-21.6(-23.8;-19.4)
Rondônia	2951.5 (2701.4;3248.2)	565.1 (526.5;608.3)	7150.6 (6699;7664.9)	449.4 (419.3;481)	-20.5(-23;-17.9)
Roraima	502 (459.5;551.6)	544 (508.2;584.8)	1917.1 (1781.8;2065.3)	445.2 (414.8;476.5)	-18.2(-20.4;-15.9)
Santa Catarina	16812.6 (15702.4;18087.7)	573.7 (537.3;613.9)	37225 (35046.3;39624.9)	470.9 (442.5;502.4)	-17.9(-20.4;-15.2)
São Paulo	133961.2 (125479.2;143345)	604.8 (567.2;644.6)	245976.9 (231441.1;261645.1)	470.8 (442.9;502.5)	-22.2(-24.3;-20.1)
Sergipe	5348.7 (4961.9;5795.4)	572 (534.5;614.3)	11043.8 (10386.9;11787.7)	485.8 (456;518.7)	-15.1(-17.4;-12.5)
Tocantins	2775.6 (2556.8;3025.5)	533.9 (499.9;572.8)	6501.8 (6086.7;6952.9)	440.7 (412.2;470.6)	-17.5(-19.7;-15.2)

*Source: Data derived from Global Burden of Disease Study 2019, Institute for Health Metrics and Evaluation, University of Washington. ^46^*

**Table 1-3:** – Number of deaths, age-standardized mortality rates due to cardiovascular diseases (per 100 000 inhabitants), and percent change of rates in Brazil and its Federative Units, in 1990 and 2019.

Cause of death and Location	1990		2019		Percent Change (95% UI)
	Number (95% UI)	Rate (95% UI)	Number (95% UI)	Rate (95% UI)	
**B.2-Cardiovascular diseases**					
Brazil	269722.7 (257743.7;277272.1)	355.4 (332.5;367.6)	397993 (361776.4;417773.2)	175.7 (159;184.8)	-50.6(-52.7;-48.8)
Acre	349.6 (326.1;369)	279.6 (255.4;295.7)	912.7 (824.2;991.4)	171.2 (151.8;187)	-38.8(-44.1;-33)
Alagoas	4147.2 (3861.4;4448.1)	345.1 (316.4;370.7)	6951.8 (6104.9;7772.3)	224.9 (196.8;250.8)	-34.8(-42.4;-26.7)
Amapá	188.5 (174.2;200.2)	238.8 (214.9;254.2)	704 (637.8;762.4)	154 (137.2;167.9)	-35.5(-40.4;-30.7)
Amazonas	1720.8 (1593.1;1851.7)	289.9 (266.6;311.4)	3750.6 (3301.4;4208.2)	143 (124.9;160.1)	-50.7(-55.9;-44.6)
Bahia	17822.2 (16003.5;19631.3)	288.4 (255.5;317.4)	28572.6 (24486.2;32622.6)	176 (151.4;201.1)	-39(-48.2;-28.5)
Ceará	9061.7 (7840.2;10176.8)	231.2 (198.8;260.9)	17908.4 (15189.3;20616.8)	182.6 (154.9;210.3)	-21(-34.2;-4.9)
Distrito Federal	1643.6 (1503.3;1806.1)	444.2 (408.3;477.6)	3389.8 (3043.6;3756.2)	185.8 (165.1;204.6)	-58.2(-62.4;-53.3)
Espírito Santo	4410.6 (4203.1;4562.4)	374.8 (348.4;389.6)	7850.8 (6919.7;8701.8)	191.8 (167.7;212.9)	-48.8(-54.2;-43.7)
Goiás	6519.1 (5763.1;7447.3)	389.1 (347;440.7)	11384.2 (9683.2;13202.3)	176.2 (149.7;204.1)	-54.7(-62;-46.4)
Maranhão	6817.6 (5871.1;7739.5)	288.3 (245.9;327.4)	15483.5 (13437.5;17842.1)	242.1 (210.4;278.9)	-16(-29.3;1.4)
Mato Grosso	1985.3 (1769.3;2199.9)	319.4 (284.1;349)	4651.3 (4170.1;5171.1)	155.6 (137.5;172.7)	-51.3(-56.6;-44.7)
Mato Grosso do Sul	2631.9 (2474.4;2769.5)	366 (338.5;386.8)	4856.1 (4328.6;5378.6)	177.3 (157.9;196.2)	-51.6(-56.5;-46.5)
Minas Gerais	30599.1 (28786.2;32249.8)	375.8 (347.3;396.3)	38760 (34341.1;42744.9)	147.8 (130.9;163)	-60.7(-64.4;-57)
Pará	5594.9 (5019.4;6163)	337 (300.4;370.7)	10746.4 (9457.6;11880.5)	163.8 (144;181.1)	-51.4(-57;-45)
Paraíba	5822.3 (5293.6;6316)	264.8 (239.1;287.5)	8913.3 (7679.8;10085.3)	180.7 (157.5;204.5)	-31.7(-40.4;-22.8)
Paraná	16189.6 (15581.2;16614.3)	423.1 (399.2;437.6)	22072.6 (19565;24449.1)	179.4 (158.4;198.7)	-57.6(-61.6;-53.4)
Pernambuco	13939.5 (13198.3;14460)	352.1 (327.8;367.1)	21121.2 (18791.5;23336.3)	219.9 (195;243.1)	-37.5(-43.7;-31.4)
Piaui	3829.3 (3497.7;4109.5)	331.2 (296.9;355.1)	6848.1 (5908.3;7614.1)	177.6 (154.4;197.1)	-46.4(-52;-40.6)
Rio de Janeiro	36000.2 (34654.8;36880.4)	441.3 (417.7;454.5)	41989.3 (37764.1;46009)	192.5 (172.8;211.1)	-56.4(-60;-52.4)
Rio Grande do Norte	3713.9 (3297.2;4074.3)	245.4 (216.6;269.9)	6158.7 (5180.9;7181.3)	154.5 (130.7;180.1)	-37(-46.6;-26.1)
Rio Grande do Sul	19771.7 (18934.4;20371)	360.4 (338.1;373.8)	25731.6 (22928.2;28470.1)	168.3 (150.1;186.3)	-53.3(-57.3;-49)
Rondônia	933.5 (827.7;1027.6)	461.4 (425.8;493.9)	2447.7 (2140.3;2781.4)	178.2 (155.3;201.6)	-61.4(-66.2;-55.8)
Roraima	145.1 (132.4;156.2)	382.9 (355.3;404.4)	526.7 (481.9;570.5)	186.5 (166.8;201.7)	-51.3(-55.3;-47.1)
Santa Catarina	7804.4 (7403.4;8174.6)	388.7 (361.6;408.4)	12033.1 (10731.6;13309.4)	164.6 (145.7;181.3)	-57.7(-61.6;-53.6)
São Paulo	65063.6 (61927;67978.8)	403.5 (373.9;423.1)	87751.7 (77569.4;96216.6)	171.5 (150.9;187.8)	-57.5(-61.2;-53.8)
Sergipe	2033.8 (1875.3;2190.6)	319 (292.8;343.3)	3755.8 (3258.8;4282.6)	173.6 (150.1;197.5)	-45.6(-52.8;-37.6)
Tocantins	983.6 (877.3;1090.3)	355.3 (318.1;389.4)	2721 (2382.9;3072)	204 (178.1;230.4)	-42.6(-50.2;-34.5)

*Source: Data derived from Global Burden of Disease Study 2019, Institute for Health Metrics and Evaluation, University of Washington. ^46^*

**Table 1-4:** – Proportional mortality from cardiovascular diseases (CVD), ischemic heart diseases (IHD) and stroke by Brazilian Region and Federative Unit, and in Brazil, in 2019.

Regions/FUs	CVD/Total	IHD/CVD	Stroke/CVD
	%	%	%
North	22.9	30.8	33.1
RO	23.4	28.9	27.0
AC	22.6	36.5	30.0
AM	18.5	28.8	36.9
RR	22.0	27.2	35.7
PA	23.8	31.9	34.2
AP	20.2	30.5	32.8
TO	29.0	30.0	29.3
Northeast	27.3	32.5	29.9
MA	30.5	32.0	32.5
PI	31.3	30.1	33.7
CE	27.4	34.2	28.8
RN	28.2	41.1	24.4
PB	28.3	35.3	25.7
PE	27.8	37.9	28.5
AL	31.0	30.1	31.0
SE	23.3	27.4	32.4
BA	24.0	26.2	31.5
Southeast	27.7	32.6	25.3
MG	25.2	23.9	27.6
ES	28.8	33.7	29.2
RJ	26.9	33.8	24.6
SP	29.2	35.5	24.3
South	26.3	30.9	30.1
PR	27.2	29.4	30.2
SC	27.1	31.0	26.6
RS	25.1	32.1	31.7
West-Central	26.1	33.1	27.6
MS	29.1	39.2	25.8
MT	24.2	30.2	28.0
GO	26.2	32.1	26.6
DF	24.5	31.4	32.9
Brazil	27.0	32.3	27.8

*Source: Brazilian Mortality Information System - SIM/DATASUS. ^43^*

**Table 1-5:** – Number of DALYs, age-standardized DALY rates due to cardiovascular diseases (per 100 000 inhabitants), and percent change of rates in Brazil and its Federative Units, in 1990 and 2019.

Cause of death and Location	1990		2019		Percent Change (95% UI)
	Number (95% UI)	Rate (95% UI)	Number (95% UI)	Rate (95% UI)	
**B.2-Cardiovascular diseases**					
Brazil	7006215.8 (6793265.2;7175766.7)	7496.4 (7214.2;7700.4)	8861401.5 (8394308.2;9258967.5)	3769.7 (3563.7;3941.2)	-49.7(-51.7;-47.9)
Acre	9825.4 (9146.4;10435.1)	5453.4 (5104.7;5755)	22488.6 (20552;24398.9)	3486.4 (3186.5;3773.4)	-36.1(-41.5;-29.9)
Alagoas	110461.2 (103870.2;118711.8)	7466.1 (6979.1;8003.9)	163441.5 (145907.2;181349.4)	5003.3 (4464.2;5545.6)	-33(-40.9;-24.4)
Amapá	5067.3 (4685.6;5415.9)	4630.7 (4327.3;4912.7)	18522.5 (17183.3;19974.3)	3307.7 (3047;3555.6)	-28.6(-34.2;-23)
Amazonas	45754.5 (42072.2;49330.8)	5541.4 (5130.9;5952.4)	89897.2 (81155.2;99494.8)	2991.8 (2679.2;3314.4)	-46(-51.9;-39.4)
Bahia	452812.5 (412140.4;496636)	6276.5 (5696.4;6892.4)	637197.3 (552757.5;722888.3)	3926.1 (3415.5;4453.8)	-37.4(-47;-26.7)
Ceará	220789.6 (195330.6;246721.7)	5001.5 (4427.7;5606.9)	378371.9 (324694.5;435882.1)	3785.3 (3251.5;4360.4)	-24.3(-37.3;-8.7)
Distrito Federal	52059.2 (47758.5;57091.7)	8173.4 (7548.7;8867.2)	81469.6 (73653.4;89826.9)	3262.3 (2942.3;3586.6)	-60.1(-64.4;-55.1)
Espírito Santo	113555.9 (109578.7;117427)	7455 (7140.5;7713.2)	176105.4 (156866.3;194211.4)	4041.5 (3597.1;4452.3)	-45.8(-51.3;-40.3)
Goiás	184416.2 (163600.3;210275.7)	8106 (7232.6;9221.2)	271049.9 (232375.2;312410.9)	3824.8 (3296.7;4398.5)	-52.8(-60.8;-43.6)
Maranhão	201000.2 (172783.1;228799.1)	6794.1 (5859.4;7722.4)	335711.6 (289713.2;391241.8)	4982.5 (4297.2;5816.9)	-26.7(-39.4;-9.2)
Mato Grosso	57476.4 (50382.9;64092.5)	6506.9 (5833.5;7149.3)	113821 (103871.1;125263.6)	3361.9 (3064.4;3696.7)	-48.3(-54.4;-40.7)
Mato Grosso do Sul	72483.1 (68659.5;76315.7)	7521.8 (7098.7;7914.8)	111484.1 (100917.1;123161.6)	3765.4 (3415.8;4150)	-49.9(-55;-44.4)
Minas Gerais	816075.2 (772344.5;861709.5)	7931.8 (7484.4;8365)	861429.4 (783971.7;940221.9)	3293.7 (2995.9;3591.5)	-58.5(-62.3;-54.5)
Pará	142720.7 (128334.6;157462)	6482.5 (5843.6;7126.1)	254515.4 (229761.6;279907.4)	3522.1 (3175.7;3867.1)	-45.7(-52.5;-38.1)
Paraíba	135924.9 (125409;147002)	5725.1 (5271.7;6197.9)	186763.1 (165980.5;210012.9)	3955.7 (3519.7;4448.8)	-30.9(-39.7;-22.1)
Paraná	415852.5 (404061.8;427856.4)	8363.5 (8061.3;8600)	479641.4 (431236.2;529867.9)	3653.3 (3287.2;4026.1)	-56.3(-60.4;-51.9)
Pernambuco	341781.1 (329624.5;354781.3)	7291 (6985.5;7564.9)	482460.4 (433602.6;531932.7)	4764.3 (4292.5;5249.2)	-34.7(-41.2;-28.3)
Piaui	97460.7 (90810.1;104026.2)	6600.2 (6091;7040)	141273 (126999.6;154951.2)	3743.8 (3367.7;4104.8)	-43.3(-49.3;-37)
Rio de Janeiro	945339.2 (918332.6;969826)	9470.4 (9161.4;9714.6)	946417.6 (860018.3;1034911.6)	4280.1 (3886.7;4676.3)	-54.8(-58.6;-50.6)
Rio Grande do Norte	84287 (76592.3;91924.1)	5086.1 (4601.8;5568.9)	132013.6 (113876.1;152147.5)	3382.5 (2917;3896)	-33.5(-43.7;-21.8)
Rio Grande do Sul	487134.2 (472994;500925.1)	7306.3 (7045;7526.8)	521946.9 (472984.7;572058.4)	3430.1 (3115.3;3752.2)	-53.1(-57;-48.6)
Rondônia	29859.8 (25915;33218.3)	8384.3 (7681.5;9083.1)	58381.7 (51876.1;66033.5)	3711.8 (3304;4188)	-55.7(-61.7;-48.5)
Roraima	4589 (4110.6;5005.9)	7068.2 (6561.2;7526.1)	13684.7 (12591.8;14854.5)	3543.2 (3254.2;3826.2)	-49.9(-54.3;-44.8)
Santa Catarina	194673.4 (185924.3;203639.1)	7562 (7195.5;7917.7)	262278.8 (236530;291520.9)	3277.3 (2965.5;3629.3)	-56.7(-60.8;-52.2)
São Paulo	1707145 (1633495.5;1778765.1)	8196.3 (7805.8;8547.4)	1975598 (1798088.4;2156089.9)	3684.9 (3354.2;4015.8)	-55(-58.9;-51.1)
Sergipe	49627.8 (45878.5;53350.1)	6106.6 (5653.9;6589.7)	84931.7 (74523.6;96498.9)	3726.6 (3282.2;4226.8)	-39(-47.1;-29.7)
Tocantins	28043.7 (25082.9;31090.9)	6599.3 (5948.1;7273.5)	60505.7 (53333.3;68340.1)	4170.5 (3667.3;4705.6)	-36.8(-45.7;-27.2)

*Source: Data derived from Global Burden of Disease Study 2019, Institute for Health Metrics and Evaluation, University of Washington. ^46^*

**Table 1-6:** – Number of YLLs, age-standardized YLL rates due to cardiovascular diseases (per 100 000 inhabitants), and percent change of rates, according to age group, in Brazil, in 1990 and 2019.

Cause of death by age group	1990		2019		Percent Change (95% UI)
	Number (95% UI)	Rate (95% UI)	Number (95% UI)	Rate (95% UI)	
**B.2-Cardiovascular diseases**					
Under 5	161337.8 (139792.5;192936.7)	952.5 (825.3;1139.1)	57843.6 (45826.5;71192.8)	373.3 (295.7;459.4)	-60.8(-70.6;-48.9)
5-14 years	62158.5 (57376;66402.3)	175.9 (162.4;187.9)	30871.4 (26559.7;34588.1)	95.7 (82.4;107.3)	-45.6(-52.8;-38.4)
15-49 years	1730356.8 (1689454.9;1774210.6)	2257.6 (2204.2;2314.8)	1478471.7 (1422955.7;1538181.6)	1280.2 (1232.1;1331.9)	-43.3(-45.8;-40.6)
50-69 years	2840819.3 (2775664.6;2909002.7)	18108.6 (17693.3;18543.3)	3565725.8 (3424289;3705718.6)	8838.5 (8487.9;9185.5)	-51.2(-53.3;-49)
70+ years	1841603.2 (1729243.4;1906089.7)	43536 (40879.8;45060.5)	2997320.6 (2653382.7;3166395.5)	22900.4 (20272.6;24192.2)	-47.4(-50.3;-45.3)
All Ages	6636275.6 (6454323.9;6792691)	4458.8 (4336.5;4563.9)	8130233.2 (7701177.6;8447854.9)	3752.4 (3554.4;3899)	-15.8(-19.8;-12.5)
Age-standardized	6636275.6 (6454323.9;6792691)	7122.2 (6860.4;7309.1)	8130233.2 (7701177.6;8447854.9)	3454.4 (3260.8;3591.7)	-51.5(-53.4;-49.7)

*Source: Data derived from Global Burden of Disease Study 2019, Institute for Health Metrics and Evaluation, University of Washington. ^46^*

**Table 1-7:** – Number of YLDs, age-standardized YLD rates due to cardiovascular diseases (per 100 000 inhabitants), and percent change of rates, according to age group, in Brazil, in 1990 and 2019.

Cause of death by age group	1990		2019		Percent Change (95% UI)
	Number (95% UI)	Rate (95% UI)	Number (95% UI)	Rate (95% UI)	
**B.2-Cardiovascular diseases**					
Under 5	1565.1 (1007.5;2281.2)	9.2 (5.9;13.5)	1412.2 (917.3;2069.4)	9.1 (5.9;13.4)	-1.4(-6.5;4.4)
5-14 years	17436.6 (10966.3;26603.3)	49.3 (31;75.3)	16072.8 (9982.1;24508.3)	49.8 (31;76)	1(-2.9;4.9)
15-49 years	124044.8 (86673.5;165579.4)	161.8 (113.1;216)	185532 (128907.5;249696.9)	160.6 (111.6;216.2)	-0.7(-3.9;2.5)
50-69 years	125877.6 (90345.3;162729.8)	802.4 (575.9;1037.3)	255231.2 (183302.9;334295.6)	632.6 (454.4;828.6)	-21.2(-24.4;-18.1)
70+ years	101016.2 (73759.2;129937.3)	2388 (1743.7;3071.8)	272920.1 (198393.9;353367.6)	2085.2 (1515.8;2699.8)	-12.7(-16;-9.2)
All Ages	369940.2 (272305.8;476273.8)	248.6 (183;320)	731168.4 (532797.5;954320.6)	337.5 (245.9;440.5)	35.8(31.6;40.3)
Age-standardized	369940.2 (272305.8;476273.8)	374.3 (276.1;480.5)	731168.4 (532797.5;954320.6)	315.3 (230.1;411.1)	-15.8(-18.2;-13.6)

*Source: Data derived from Global Burden of Disease Study 2019, Institute for Health Metrics and Evaluation, University of Washington. ^46^*

**Table 1-8:** – Total number of hospitalizations for clinical procedures for cardiovascular diseases by competence year, Brazil, 2008 to 2019.

	2008	2009	2010	2011	2012	2013	2014	2015	2016	2017	2018	2019	Total
Chronic ischemic heart disease	12 393	9 743	9 300	8 497	8 000	7 197	7 581	6 403	6 317	6 171	6 292	6 703	94 597
Cerebrovascular disease	159 545	176 047	181 035	184 751	182 065	183 043	187 110	191 678	195 787	198 068	203 066	211 149	2 253 344
Valve disease	3 237	4 156	3 526	3 637	3 285	2 996	2 753	2 400	2 244	2 231	2 330	2 289	35 084
Atrial fibrillation	29 034	28 174	28 382	28 583	28 760	28 268	29 799	29 754	29 889	30 265	30 958	32 753	354 619
Myocardial Infarction (Clinical)	47 358	50 987	55 513	58 194	59 562	58 552	62 809	66 647	70 441	71 835	74 569	80 614	757 081
Heart failure	298 474	297 763	289 110	284 844	264 469	254 285	243 913	240 832	236 358	230 297	222 394	222 620	3 085 359
Cardiomyopathies	2 092	2 363	2 459	2 302	2 357	2 293	2 370	2 230	2 250	1 997	2 251	2 390	27 354
Acute coronary sindrome	63 300	68 833	72 912	71 523	75 734	73 432	76 945	72 686	70 430	70 713	68 413	70 204	855 125
**Total**	**615 433**	**638 066**	**642 237**	**642 331**	**624 232**	**610 066**	**613 280**	**612 630**	**613 716**	**611 577**	**610 273**	**628 722**	**7 462 563**

*Source: Brazilian Mortality Information System – SIM/DATASUS. ^43^*

**Table 1-9:** – Total number of hospitalizations for surgical procedures for cardiovascular diseases by competence year, Brazil, 2008 to 2019.

	2008	2009	2010	2011	2012	2013	2014	2015	2016	2017	2018	2019	Total
Atrial Fibrillation Ablation	68	72	90	85	123	139	143	161	124	120	125	163	1 413
Coronary Angioplasty	38 635	45 648	49 492	55 931	60 959	63 838	66 492	66 550	69 802	73 971	78 575	85 518	755 411
Coronary Artery Bypass Graft Surgery	20 515	22 077	21 225	23 187	23 900	23 249	22 997	22 559	22 248	21 474	20 674	21 018	265 123
Valve surgery	12 201	12 664	12 169	13 181	13 435	13 067	12 993	12 624	12 432	12 277	12 088	12 771	151 902
Infarction - angioplasty	7 648	6 362	6 262	6 033	5 865	6 055	7 135	8 524	10 195	10 774	10 811	11 099	96 763
Cardiomyopathies	15	43	13	21	28	23	20	18	32	29	26	24	292
Other Valvuloplasties	451	477	445	486	456	527	515	513	399	427	391	450	5 537
Mitral Valvuloplasty	477	551	478	473	403	431	408	341	206	236	200	195	4 399
**Total**	**80 010**	**87 894**	**90 174**	**99 397**	**105 169**	**107 329**	**110 703**	**111 290**	**115 438**	**119 308**	**122 890**	**131 238**	**1 280 840**

*Source: Brazilian Mortality Information System – SIM/DATASUS. ^43^*

**Table 1-10:** – Total value (in Reais) of clinical procedures for cardiovascular diseases by competence year, Brazil, 2008 to 2019.

	2008	2009	2010	2011	2012	2013	2014	2015	2016	2017	2018	2019	Total
Chronic ischemic heart disease	7 798 577.82	6 860 711.25	6 593 971.61	6 250 858.75	5 689 946.25	5 248 239.82	6 213 647.76	5 140 625.95	5 326 842.16	5 532 381.71	5 672 327.20	6 475 643.96	72 803 774.24
Cerebrovascular disease	142 061 641.70	188 450 404.06	198 812 522.26	205 447 974.55	218 628 105.94	228 140 748.46	242 663 800.69	252 440 767.47	263 770 567.49	272 140 330.46	286 293 302.84	303 838 674.47	2 802 688 840.39
Valve disease	1 051 959.34	1 589 247.64	1 439 424.14	1 606 640.13	1 509 338.04	1 509 785.49	1 584 222.50	1 672 410.55	1 675 284.06	1 678 874.30	2 043 385.62	1 999 540.54	19 360 112.35
Atrial fibrillation	13 790 984.47	17 396 138.42	18 537 031.69	18 858 448.37	20 371 040.61	19 968 864.00	22 636 728.19	23 329 786.10	23 929 183.91	26 060 564.58	26 971 390.07	28 743 440.36	260 593 600.77
Myocardial Infarction (Clinical)	65 019 330.51	84 308 216.56	92 969 057.67	97 323 922.15	104 897 640.92	106 246 319.66	119 582 977.33	128 723 964.23	134 911 984.37	136 437 974.90	143 349 385.91	151 123 021.25	1 364 893 795.46
Heart failure	272 280 662.78	322 849 486.97	327 913 746.49	330 492 446.60	317 585 920.25	321 711 992.20	326 140 931.65	337 610 340.87	345 565 633.18	346 841 126.90	348 832 330.32	359 301 690.55	3 957 126 308.76
Cardiomyopathies	1 287 646.38	1 901 574.84	2 143 534.84	1 899 610.88	2 110 498.65	2 301 691.59	2 696 303.97	2 681 816.09	3 065 112.69	2 556 468.20	3 119 717.43	3 173 903.30	28 937 878.86
Acute coronary sindrome	44 710 681.49	57 921 695.01	64 611 984.70	65 586 337.38	75 210 291.07	74 619 170.87	83 606 992.07	82 094 816.54	80 185 274.88	82 072 225.98	80 036 822.54	81 167 004.50	871 823 297.03
**Total**	**548 001 484.49**	**681 277 474.75**	**713 021 273.40**	**727 466 238.81**	**746 002 781.73**	**759 746 812.09**	**805 125 604.16**	**833 694 527.80**	**858 429 882.74**	**873 319 947.03**	**896 318 661.93**	**935 822 918.93**	**9 378 227 607.86**

*Source: Brazilian Mortality Information System – SIM/DATASUS. ^43^*

**Table 1-11:** – Total value (in Int$2019) of clinical procedures for cardiovascular diseases by competence year, Brazil, 2008 to 2019.

	Int$2019	Int$2019	Int$2019	Int$2019	Int$2019	Int$2019	Int$2019	Int$2019	Int$2019	Int$2019	Int$2019	Int$2019	Int$2019
**2008**	**2009**	**2010**	**2011**	**2012**	**2013**	**2014**	**2015**	**2016**	**2017**	**2018**	**2019**	**Total**
Chronic Ischemic Heart Disease	$ 7 650 265.61	$ 6 274 085.62	$ 5 560 158.75	$ 4 864 286.14	$ 4 101 608.01	$ 3 519 939.65	$ 3 863 867.68	$ 2 971 845.26	$ 2 849 164.90	$ 2 859 497.35	$ 2 845 701.28	$ 3 129 842.42	$ 50 490 262.67
Cerebrovascular Disease	$ 139 359 934.26	$ 172 336 938.05	$ 167 642 393.83	$ 159 875 270.76	$ 157 598 464.21	$ 153 011 617.97	$ 150 897 001.54	$ 145 938 433.52	$ 141 082 806.42	$ 140 659 953.49	$ 143 628 036.74	$ 146 852 911.78	$ 1 818 883 762.57
Valve Disease	$ 1 031 953.33	$ 1 453 358.90	$ 1 213 749.04	$ 1 250 253.39	$ 1 088 009.05	$ 1 012 597.36	$ 985 126.02	$ 966 836.61	$ 896 058.19	$ 867 752.24	$ 1 025 128.64	$ 966 428.49	$ 12 757 251.26
Atrial Fibrillation	$ 13 528 709.55	$ 15 908 680.29	$ 15 630 767.78	$ 14 675 245.87	$ 14 684 501.34	$ 13 392 908.59	$ 14 076 324.52	$ 13 487 173.53	$ 12 798 988.35	$ 13 469 807.27	$ 13 531 045.80	$ 13 892 431.30	$ 169 076 584.19
Myocardial Infarction (Clinical)	$ 63 782 802.43	$ 77 099 436.14	$ 78 393 227.96	$ 75 735 418.85	$ 75 615 653.52	$ 71 258 297.28	$ 74 360 958.09	$ 74 416 560.70	$ 72 160 292.77	$ 70 520 084.88	$ 71 915 726.50	$ 73 041 576.24	$ 878 300 035.36
Heart Failure	$ 267 102 469.11	$ 295 244 217.24	$ 276 502 932.54	$ 257 182 235.55	$ 228 932 383.01	$ 215 768 874.17	$ 202 806 057.29	$ 195 175 782.35	$ 184 832 484.51	$ 179 270 219.50	$ 175 002 706.17	$ 173 659 589.44	$ 2 651 479 950.88
Cardiomyopathies	$ 1 263 158.11	$ 1 738 980.54	$ 1 807 468.20	$ 1 478 237.03	$ 1 521 356.76	$ 1 543 720.52	$ 1 676 657.92	$ 1 550 383.65	$ 1 639 435.00	$ 1 321 350.27	$ 1 565 104.34	$ 1 534 027.69	$ 18 639 880.03
Acute Coronary Sindrome	$ 43 860 380.31	$ 52 969 096.11	$ 54 482 019.85	$ 51 037 901.30	$ 54 215 473.87	$ 50 046 298.80	$ 51 989 808.02	$ 47 459 802.34	$ 42 888 650.24	$ 42 420 303.78	$ 40 152 988.47	$ 39 230 065.00	$ 570 752 788.09
**Total**	**$537 579 672.71**	**$623 024 792.89**	**$ 601 232 717.95**	**$566 098 848.89**	**$537 757 449.77**	**$509 554 254.34**	**$ 500 655 801.08**	**$ 481 966 817.96**	**$ 459 147 880.38**	**$ 451 388 968.78**	**$ 449 666 437.94**	**$ 452 306 872.36**	**$ 6 170 380 515.05**

*Source: Brazilian Mortality Information System – SIM/DATASUS. ^43^*

**Table 1-12:** – Total value (in Reais) of surgical procedures for cardiovascular diseases by competence year, Brazil, 2008 to 2019.

	2008	2009	2010	2011	2012	2013	2014	2015	2016	2017	2018	019	Total
Atrial Fibrillation Ablation	360 473.27	376 837.24	471 138.73	456 926.51	690 004.54	788 451.61	770 674.50	906 324.09	707 088.34	690 689.60	732 003.78	961 948.99	7 912 561.20
Coronary Angioplasty	210 528 789.87	266 654 421.74	295 641 177.27	337 972 226.29	372 062 717.78	388 919 617.05	411 251 867.70	412 073 165.85	433 590 492.36	466 696 227.86	495 885 258.42	546 132 198.85	4 637 408 161.04
Coronary Artery Bypass Graft Surgery	176 032 311.77	208 585 452.82	214 483 618.24	287 851 733.82	297 843 568.00	290 540 953.96	294 854 279.38	289 638 019.51	286 159 517.75	282 175 032.32	275 110 233.84	278 544 223.76	3 181 818 945.17
Valve Surgery	125 954 499.60	140 683 968.83	142 383 177.39	179 111 011.96	183 271 263.12	178 563 635.59	180 088 277.85	176 813 774.47	175 318 559.92	176 135 832.05	177 583 849.27	187 382 032.83	2 023 289 882.88
Infarction - Angioplasty	45 267 283.32	37 887 853.46	37 112 662.15	35 576 928.51	35 545 437.05	37 287 935.69	45 883 433.02	56 100 757.09	66 515 237.46	71 624 171.14	73 429 322.18	74 907 756.33	617 138 777.40
Cardiomyopathies	168 992.82	544 719.64	192 352.38	325 920.92	436 100.76	353 814.17	305 552.03	298 288.99	526 912.73	451 789.68	426 075.02	404 878.61	4 435 397.75
Other Valvuloplasties	1 518 843.49	1 661 544.32	1 717 544.82	1 918 678.81	1 870 621.37	2 051 540.92	2 128 294.09	2 085 967.07	1 594 213.11	1 888 744.84	1 689 593.28	1 959 571.29	22 085 157.41
Mitral Valvuloplasty	3 115 254.91	3 585 355.62	3 147 310.18	3 227 816.88	2 718 391.46	2 970 343.43	2 808 556.55	2 392 670.47	1 377 571.02	1 720 524.66	1 461 666.40	1 430 166.52	29 955 628.10
Total	562 946 449.05	659 980 153.67	695 148 981.16	846 441 243.70	894 438 104.08	901 476 292.42	938 090 935.12	940 308 967.54	965 789 592.69	1 001 383 012.15	1 026 318 002.19	1 091 722 777.18	10 524 044 510.95

*Source: Brazilian Mortality Information System – SIM/DATASUS. ^43^*

**Table 1-13:** – Total value (in Int$2019) of surgical procedures for cardiovascular diseases by competence year, Brazil, 2008 to 2019.

	Int$ 2019	Int$ 2019	Int$ 2019	Int$ 2019	Int$ 2019	Int$ 2019	Int$ 2019	Int$ 2019	Int$ 2019	Int$ 2019	Int$ 2019	Int$ 2019	Int$ 2019
	**2008**	**2009**	**2010**	**2011**	**2012**	**2013**	**2014**	**2015**	**2016**	**2017**	**2018**	**2019**	**Total**
Atrial Fibrillation Ablation	$ 353 617.84	$ 344 615.74	$ 397 272.89	$ 355 570.55	$ 497 391.02	528 806.26	479 232.88	523 954.67	378 199.92	356 993.60	367 232.71	464 934.26	5 047 822.34
Coronary Angioplasty	$ 206 524 984.25	$ 243 854 115.30	$ 249 290 105.61	$ 263 002 841.99	$ 268 202 080.69	260 844 326.45	255 731 071.29	238 223 457.02	231 914 288.54	241 219 188.62	248 776 431.05	263 959 496.79	2 971 542 387.60
Coronary Artery Bypass Graft Surgery	$ 172 684 555.11	$ 190 750 337.95	$ 180 856 551.64	$ 224 000 134.26	$ 214 701 072.80	194 862 784.28	183 350 901.63	167 442 522.38	153 057 970.91	145 846 544.88	138 017 698.57	134 627 464.36	2 100 198 538.77
Valve Surgery	$ 123 559 115.42	$ 128 654 775.47	$ 120 060 127.13	$ 139 380 403.22	$ 132 111 420.33	119 760 766.01	111 985 310.80	102 217 742.13	93 772 533.78	91 038 538.47	89 090 521.41	90 566 473.09	1 342 197 727.26
Infarction - Angioplasty	$ 44 406 396.77	$ 34 648 249.69	$ 31 294 082.75	$ 27 685 213.69	$ 25 622 992.36	25 008 629.14	28 531 954.26	32 432 386.78	35 576 965.46	37 020 064.48	36 838 128.17	36 204 812.15	395 269 875.70
Cardiomyopathies	$ 165 778.94	$ 498 143.35	$ 162 195.08	$ 253 624.77	$ 314 364.02	237 299.47	190 003.14	172 443.73	281 829.50	233 514.51	213 753.93	195 688.07	2 918 638.51
Other Valvuloplasties	$ 1 489 958.35	$ 1 519 473.85	$ 1 448 265.54	$ 1 493 075.29	$ 1 348 440.70	1 375 947.07	1 323 449.10	1 205 917.61	852 695.82	976 227.08	847 637.59	947 110.34	14 828 198.34
Mitral Valvuloplasty	$ 3 056 009.45	$ 3 278 789.52	$ 2 653 870.12	$ 2 511 818.86	$ 1 959 557.38	1 992 178.33	1 746 460.58	1 383 225.79	736 820.50	889 279.87	733 290.91	691 235.63	21 632 536.94
**Total**	**$ 552 240 416.13**	**$ 603 548 500.87**	**$ 586 162 470.76**	**$ 658 682 682.63**	**$ 644 757 319.30**	**$ 604 610 737.01**	**$ 583 338 383.68**	**$ 543 601 650.11**	**$ 516 571 304.43**	**$ 517 580 351.51**	**$ 514 884 694.34**	**$ 527 657 214.69**	**$ 6 853 635 725.46**

*Source: Brazilian Mortality Information System – SIM/DATASUS.43*

**Chart 1-1:** – Ranking of causes of death in Brazil, 1990 and 2019, according to age-standardized mortality rates per 100 000 inhabitants, both sexes, 1990 and 2019, and percent change of rates.

Source: Data derived from Global Burden of Disease Study 2019, Institute for Health Metrics and Evaluation, University of Washington. ^46^

**Chart 1-2:** – Ranking of causes of cardiovascular death in Brazil, 1990 and 2019, according to age-standardized mortality rate per 100 000 inhabitants, both sexes, 1990 and 2019, and percent change of rates.

Source: Data derived from Global Burden of Disease Study 2019, Institute for Health Metrics and Evaluation, University of Washington. ^46^

**Chart 1-3:** – Ranking of causes of cardiovascular death per Brazilian Federative Unit in 1990, according to age-standardized mortality rates per 100 000 inhabitants, both sexes.

Source: Data derived from Global Burden of Disease Study 2019, Institute for Health Metrics and Evaluation, University of Washington. ^46^

**Chart 1-4:** – Ranking of causes of cardiovascular death per Brazilian Federative Unit in 2019, according to age-standardized mortality rates per 100 000 inhabitants, both sexes.

Source: Data derived from Global Burden of Disease Study 2019, Institute for Health Metrics and Evaluation, University of Washington. ^46^

**Chart 1-5:** – Cardiovascular disease percent prevalence, by sex, in Brazil, 1990-2019.

Source: Data derived from Global Burden of Disease Study 2019, Institute for Health Metrics and Evaluation, University of Washington. ^46^

**Chart 1-6:** – Age-standardized prevalence rate of cardiovascular disease, per 100 000 inhabitants, by sex, Brazil, 1990-2019.

Source: Data derived from Global Burden of Disease Study 2019, Institute for Health Metrics and Evaluation, University of Washington. ^46^

**Chart 1-7:** – Age-standardized mortality rate from cardiovascular disease, per 100 000 inhabitants, by sex, Brazil, 1990-2019.

Source: Data derived from Global Burden of Disease Study 2019, Institute for Health Metrics and Evaluation, University of Washington. ^46^

**Chart 1-8:** – Proportional mortality from cardiovascular diseases, by sex, Brazil, 1990-2019.

Source: Data derived from Global Burden of Disease Study 2019, Institute for Health Metrics and Evaluation, University of Washington. ^46^

**Chart 1-9:** – Geographic distribution of mortality rates per 100 000 inhabitants, standardized by age in the Federative Units of Brazil, according to sex, 2000 and 2019.

Source: Brazilian Mortality Information System (SIM/DATASUS) with redistribution of ill-defined causes and correction for underreporting (according to GBD 2019 estimates) and IBGE population. ^43^

**Chart 1-10:** – Age-standardized mortality rates from cardiovascular disease, per 100 000 inhabitants, by Brazilian region, for females, 1990-2019.

Source: Data derived from Global Burden of Disease Study 2019, Institute for Health Metrics and Evaluation, University of Washington. ^46^

**Chart 1-11:** – Age-standardized mortality rate from cardiovascular disease, per 100 000 inhabitants, by Brazilian regions, for males, 1990-2019.

Source: Data derived from Global Burden of Disease Study 2019, Institute for Health Metrics and Evaluation, University of Washington. ^46^

**Chart 1-12:** – Correlation between percent change of age-standardized mortality rates 2019/1990 and the 2019 sociodemographic index (SDI 2019).

Source: Data derived from Global Burden of Disease Study 2019, Institute for Health Metrics and Evaluation, University of Washington. ^46^

**Chart 1-13:** – Age-standardized rates of DALYs due to cardiovascular diseases, per 100 000 inhabitants, 1990-2019, Brazil and its regions.

Source: Data derived from Global Burden of Disease Study 2019, Institute for Health Metrics and Evaluation, University of Washington. ^46^

**Chart 1-14:** – Correlation between percent change of age-standardized DALY rates 2019/1990 and the 2019 sociodemographic index (SDI 2019).

Source: Data derived from Global Burden of Disease Study 2019, Institute for Health Metrics and Evaluation, University of Washington. ^46^

**Chart 1-15:** – Total number of hospitalizations for clinical procedures due to cardiovascular diseases, by competence year, Brazil, 2008 to 2019.

Source: Brazilian Mortality Information System – SIM/DATASUS. ^43^

**Chart 1-16:** – Total number of hospitalizations for surgical procedures due to cardiovascular diseases, by competence year, Brazil, 2008 to 2019.

Source: Brazilian Mortality Information System – SIM/DATASUS. ^43^

### Overview

• Noncommunicable chronic diseases comprise the world’s leading group of causes of death, responsible for premature deaths, loss of quality of life, and adverse economic and social impacts. The NCD are responsible for approximately 70% of global deaths, equivalent to more than 38 million deaths annually, significantly exceeding deaths from external causes and infectious diseases. ^23^ Of all deaths due to NCD worldwide, nearly 45%, over 17 million, result from CVD. A similar distribution is observed in Brazil, where 72% of deaths are due to NCD, of which 30% are due to CVD, and 16% to neoplasms (Chart 1-1). ^21^ ^,^ ^27^ ^,^ ^28^

• The definition of CVD may vary according to the study, from including all diseases listed in ICD-10 Chapter IX to just grouping together the 3 main causes (IHD, stroke, and HF). For the GBD, the definition of total CVD comprises 10 causes: rheumatic heart disease, IHD, cerebrovascular disease, hypertensive heart disease, cardiomyopathy, myocarditis, atrial fibrillation and flutter, aortic aneurysm, peripheral vascular disease, and endocarditis. ^29^

• Cardiovascular diseases are the number 1 cause of death in Brazil. According to the GBD Study 2019 estimates, of the CVD, IHD was the leading cause of death in the country, followed by stroke in both 1990 and 2019 (Chart 1-2). In fact, in 2019, IHD was the leading cause of death in all Brazilian FUs, except for the state of Amazonas, in the Northern region. Three states in that region, Acre, Amapá, and Pará, showed no significant difference regarding the mortality rates due to IHD and stroke (Charts 1-3 and 1-4).

### Prevalence

• Gonçalves et al. published in 2019 a cross-sectional study from the Brazilian National Health Survey conducted in 2013 on a sample of 60 202 adults aged over 18 years, stratified by sex and age groups, using a hierarchical binary logistic regression model. The self-reported diagnosis of heart disease in Brazil was 4.2% (95% CI, 4.0 - 4.3) and associated with the female sex (OR = 1.1; 95% CI, 1.1 - 1.1), individuals 65 years and older (OR = 4.7; 95% CI, 3.3 – 5), hypertension (OR = 2.4; 95% CI, 2.2 – 2.7), elevated cholesterol (OR = 1.6; 95% CI, 1.5 – 1.8), overweight (OR = 1.5; 95% CI, 1.4 – 1.8) or obesity (OR = 2.0; 95% CI, 1.7 – 2.2), sedentary behavior (OR = 1.5; 95% CI, 1.02 – 2.1), and tobacco use (OR = 1.2; 95% CI, 1.03 – 1.3). ^30^

• In the ELSA-Brasil, a cohort study that included 15 105 civil servants from 6 academic institutions (54% women, 35-74 years, with baseline assessment between 2008 and 2010), the self-reported prevalence was as follows: coronary heart disease, 4.7% (men=5.7%, women=4.0%); HF, 1.5% (men=1.9%, women=1.5%); stroke, 1.3% for both sexes; rheumatic fever, 2.9% (men=2.2%, women=3.4%); and Chagas disease, 0.4%, for both sexes. ^31^

• The prevalence of CVD increases significantly with age. In a longitudinal study in the elderly over 60 years of age, from the state of São Paulo in 2000, 2006 and 2010, the prevalence of CVD was defined as a positive response to the question: “Have you ever been told by a doctor or nurse you had a heart attack, coronary heart disease, angina, congestive disease, or other heart problems?”. The CVD prevalence was 17.9%, 22.2% and 22.9% for 2000, 2006, and 2010, respectively. The presence of CVD was associated with older age, smoking history, diabetes, and hypertension. ^32^

• According to the GBD Study 2019, the CVD prevalence was 6.1% of the population in 2019 and has increased from 1990 due to population growth and aging. However, the age-standardized CVD prevalence rate in Brazil decreased in the same period, from 6138 (95% UI, 5762 - 6519) to 5454 (95% UI, 5082 - 5838) per 100 000 inhabitants (Table 1-1).

• As compared to females, in 2019 males had a higher age-standardized prevalence rate (Charts 1-5 and 1-6) and, from 1990 to 2019, a prevalence rate decline of -8,7 (-10.2 to -7.2), lower than that of females (-12.8, 95% UI, -14.1 to -11.3) in the same period (Chart 1-6 and Table 1-1). Considering the total number in 2019, 12 946 932 (95% UI, 11 899 752 – 13 617 524) individuals had prevalent CVD in Brazil, and 51% of them were men. The prevalence rate declined among the elderly and increased among males and females aged 15-49 years (Table 1-1).

### Incidence

• According to the GBD Study 2019, the age-standardized incidence rate of CVD in Brazil, in 2019, was 475 (95% UI, 447-507) per 100 000 inhabitants. From 1990 to 2019, that rate decreased -20% (-22 to -18) (Table 1-2).

• The age-standardized CVD incidence rate did not differ significantly among the FUs in 2019, varying from 441 in the state of Piauí to 486 in the state of Pernambuco. From 1990 to 2019, all FUs had a reduction in the age-standardized CVD incidence rate, and the state of Rio de Janeiro showed the highest percent change among them (-25.5%; 95% UI, -27.7 to -23.5) (Table 1-2).

### Mortality

• In Brazil, Mansur *et al* . have shown that the age-standardized CVD mortality rate has declined significantly in recent decades. A 2016 study analyzed CVD mortality rates from the age of 30 years and older, by sex, per 100 000 inhabitants. The annual variations in cardiovascular mortality for the periods 1980-2006 and 2007-2012 were, respectively: for both sexes: -1.5% and -0.8%; men: -1.4% and -0.6%; women: -1.7% and -1.0%. ^33^

• Baptista *et al* . investigated how age composition and age-specific mortality rates are related to the observed difference in deaths from CVD in the adult population, by sex, in Brazilian microregions from 1996 to 2015. They suggested, after correcting for underreporting of death counts, that there was a decline in the rates of deaths from CVD in the period studied. However, the main driver of the change in mortality rates was heterogeneous across Brazilian microregions. In general, in the most socioeconomically developed areas, the age structure was more importantly related to the mortality rates, with older populations dying from CVD. Interestingly, there were differences in the main drivers of CVD mortality even within the Brazilian regions and FUs. ^34^

• Data from the GBD Study 2019 reveal that, although mortality rates from CVD in Brazil significantly decreased over the past few years, the total number of deaths due to CVD increased, as a result of population growth and aging. There were 269 722.7 (95% UI, 257 743.7 - 277 272.1) and 397 993 (95% UI, 361 776.4 – 417 773.2) deaths from CVD in the country in 1990 and 2019, respectively. The age-standardized mortality rate, per 100 000 inhabitants, was 355.4 (332.5 - 367.6) in 1990 and 175.7 (159 - 184.8) in 2019, decreasing by -50.6% (-52.7 to -0.5) in the period (Chart 1-7). Although the age-standardized mortality rates were higher for men throughout the whole period, the percent decrease was similar for both sexes, 48% for men and 52% for women (Chart 1-8).

• Table 1-3 shows the number of deaths, the age-standardized mortality rate due to CVD per 100 000 inhabitants, and the percent change of rates, by FU, in Brazil, in 1990-2019. The states of Maranhão and Alagoas had the highest risk for mortality, above the country average. The FUs with the greatest percent reductions in the period were Rondônia, Minas Gerais, Distrito Federal, Paraná, Santa Catarina, and São Paulo, in that order.

• Chart 1-9 shows the geographical distribution of mortality rates per 100 000 inhabitants, standardized by age in the Brazilian FUs, by sex, in 2000 and 2019, according to the SIM, and using the IBGE population. There was a decrease in the standardized mortality rates for both sexes, except for males from the states of Roraima, Piauí and Alagoas, despite the redistribution of ill-defined causes and correction of underreporting according to the GBD 2019 coefficients. Malta *et al.* compared historical series of CVD mortality rates in Brazil using SIM database with and without correction and the GBD 2017 estimates between 2000 and 2017. The authors pointed out that the increase in mortality rates observed in 2017 in some Northern and Northeastern FUs was due to the improvement in death registration and in the definition of underlying causes of death in recent years. ^6^

• Brant *et al.* , analyzing GBD 2015 data, observed a decrease in age-standardized CVD mortality rate from 429.5 (1990) to 256.0 (2015) per 100 000 inhabitants (-40.4%), with marked differences across the FUs. That decrease was more pronounced in the FUs of the Southeastern and Southern regions and the Distrito Federal, regions that concentrate the largest populations and income, and more modest in most Northern and Northeastern states. ^29^

• Importantly, the annual reduction in CVD mortality rates in Brazil was lower in the final years when considering the 1990-2019 period, for males and females.

• Regarding the trend by age group, the largest reductions in the CVD mortality rates per 100 000, between 1990 and 2019, were observed in the ‘under 5’ age group [-60.9 (-70.7 to -48.9)], followed by the 50-69 years age group [-50.6 (-52.7 to -48.4)].

• The FHP coverage was associated with a reduction in hospitalizations and mortality from CVD that were included in the national ambulatory care-sensitive list in Brazil, and its effect increased according to the duration of the FHP implementation in the municipality. Rasella *et al* . observed reductions in the mortalities from cerebrovascular disease and heart disease of 0.82 (95% CI, 0.79 - 0.86) and 0.79 (95% CI, 0.75 - 0.80) respectively, reaching 0.69 (0.66 - 0.73) and 0.64 (0.59 - 0.68), respectively, when the FHP coverage was consolidated during all 8 years studied. ^35^

• According to the SIM database, in 2019, CVD corresponded to 27.0% of total deaths, with the highest proportion in the Southeastern region and lowest in the Northern region. Ischemic heart disease accounted for 32.3% of total deaths from CVD in Brazil, and stroke was responsible for 27.8% of those deaths. The highest proportion of IHD mortality occurred in the states of Mato Grosso do Sul, Pernambuco, and São Paulo, while the highest proportion of stroke deaths occurred in the states of Amazonas, Pará, and Amapá, and the Distrito Federal (Table 1-4).

• The proportion of deaths due to CVD decreased for men (from 30.1% to 27.6%) and for women (from 31.1% to 29.9%) from 2000-2002 to 2015-2017. Moreover, Lotufo noted a constant excess of premature male deaths due to CVD during that period, with a male-to-female ratio of 2:1. ^36^

There is a significant correlation of the SDI, an estimate of the socioeconomic level, with the CVD burden. Chart 1-12 shows the correlation of a greater reduction in the percent change of age-standardized CVD mortality rates, between 1990 and 2019, with a higher 2019 SDI, suggesting that the decrease in mortality from CVD followed the improvement in the local socioeconomic conditions, as observed in other studies. ^5^ ^,^ ^37^

• Lotufo *et al* . compared three different household income levels (high, middle, and low) with mortality rates due to CVD, in the city of São Paulo, from 1996 to 2010. The annual percent change and 95% CI for men living in the high, middle-, and low-income areas were -4.1 (-4.5 to -3.8), -3.0 (-3.5 to -2.6), and -2.5 (-2.8 to -2.1), respectively. The trend rates of women living in the high-income areas were -4.4 (-4.8 to -3.9) in 1996-2005 and -2.6 (-3.8 to -1.4) in 2005-2010. The reduction in deaths due to CVD was more significant for men and women living in the wealthiest neighborhoods, with a greater declining risk of death gradient for those living in the wealthiest areas as compared to the most deprived neighborhoods. ^40^

• An inverse association of the HDIm and the supplementary health coverage with mortality due to CVD was observed, suggesting a relationship between socioeconomic factors and CVD. ^37^ The HDIm increased between 2000 and 2010 in all FUs, in half of which it was 0.7 or higher. Supplementary health coverage increased in the country during the study period and was inversely associated with mortality due to CVD between 2004 and 2013. ^37^

• Soares *et al* . observed a decrease in CVD mortality in the states of Rio de Janeiro, São Paulo, and Rio Grande do Sul that preceded improvement in the socioeconomic index. The GDP per capita evolution, the child mortality decline, a higher educational level (represented by the schooling years of individuals over the age of 25 years), and the HDIm showed a high correlation with the reduction in the CVD mortality rate. A reduction in the mortality rates due to DCV, stroke, and IHD in the state of Rio de Janeiro State in past decades was preceded by an increase in the HDI. An increment of 0.1 in the HDI correlated with the following reductions in the number of deaths per 100 000 inhabitants: 53.5 for DCV; 30.2 for stroke; and 10.0 for IHD. ^38^ ^,^ ^39^

• Baptista *et al* . investigated the relationship between CVD mortality rate and economic development over time and space, measured by GDP per capita, in Brazilian microregions from 2001 to 2015. They used the databases of the SIM (DATASUS) and of the SIDRA (IBGE). The authors observed a rapid decline in CVD mortality in the Southern and Southeastern microregions and a slower decline in the West-Central region. On the other hand, the Northern and Northeastern regions had an increase in CVD mortality over time, maybe due to lower access to healthcare and socioeconomic factors. ^41^

• Silveira *et al* ., studying the effect of ambient temperature on cardiovascular mortality in 27 Brazilian cities, observed a higher number of cardiovascular deaths associated with low and high temperatures in most of the Brazilian cities and the West-Central, Northern, Southern, and Southeastern regions. The overall RR for Brazil was 1.26 (95% CI, 1.17–1.35) for the 1^st^percentile of temperature and 1.07 (95% CI, 1.01–1.13) for the 99^th^percentile of temperature *versus* the 79^th^percentile (27.7 °C), in which RR was the lowest. ^42^

### Burden of Disease

• Age-standardized DALY rates in Brazil were 6907 (95% UI, 6783-7039) per 100 000 inhabitants in 1990 and decreased to 3735 (95% UI, 3621-3849) per 100 000 inhabitants in 2019. The DALY rates declined in all five regions, with different patterns, faster in the South and Southeast and slower in the Northeast (Chart 1-13). There was a correlation between the percent decline in DALY rates and the increase in the SDI: the higher the SDI, the greater the decline in DALYs due to CVD. Distrito Federal, and the states of Rio de Janeiro and Santa Catarina had higher SDI and a great decline in DALY rates, while, the states of Alagoas, Piauí and Ceará had small declines in DALY rates and low SDI (Chart 1-14 and Table 1-5).

• Regarding YLLs, 8 130 233 years of life were lost in 2019 due to CVD mortality. It was higher among individuals aged 50-69 years as compared to the other age groups. The YLL rates have declined since 1990 for all age groups (Table 1-6). Age-standardized YLL rates decreased by 51.5% (95% UI, -53.4 to -49.7) from 1990 to 2019 (Table 1-6).

• Disability caused by CVD did not decline as observed with mortality. The age-standardized YLD rate declined by 15% from 1990 to 2019 (Table 1-7). The ‘50-69 years’ group had the greatest number of YLDs, followed close by the ‘15-49 years’ group. All age groups had a small decline in the age-standardized rates, and the ‘15-49 years’ group had the smallest (-0.7%) (Table 1-7).

### Health Care Utilization and Cost

• In Brazil, from 2008 to 2019, the main groups of clinical and surgical cardiovascular procedures corresponded to 8 743 403 procedures paid by SUS. Of these, 7 462 563 were clinical procedures, led by HF, with 41.3% (3 085 359) of the admissions, followed by cerebrovascular diseases, 30.2% (2 253 344), acute coronary syndrome, 11.5% (855 125), and AMI with clinical approach, 10.1% (757 081) (Tables 1-8 and 1-9).

• Hospital admissions for clinical conditions of CVD decreased by 13 289 from 2008 to 2019 (Table 1-8), although the absolute numbers through the years were stable. In 2008, each clinical admission costed R$890 on average, and, in 2019, that cost was R$1488, a 67% increase (Table 1-10).

• Of the 1 280 840 cardiovascular surgical procedures performed from 2008 to 2019, 755 411 (58.9%) were coronary angioplasties, followed by 265 123 CABG surgeries (20.1%), and 151 902 valve surgeries (11.9%). The angioplasty/CABG ratio in 2008 was 1.8, increasing to 4.1 in 2019.

• Hospitalizations for surgical CVD procedures from 2008 to 2019 increased by 64% (Table 1-9). On average, each surgical procedure was reimbursed R$7036 in 2008, showing an unadjusted increase of 18% in 2019 as compared to 2008, at a cost of R$8319 per procedure (Table 1-12).

• Over the last 12 years, in Brazil, there has been a significant reduction in hospitalizations for HF and an increase in annual hospitalizations for AMI and cerebrovascular diseases, and flat trends in other groups of clinical procedures (Chart 1-15). Regarding the surgical approaches in the same years, there was a great increase in the annual number of coronary angioplasties and a trend towards stability in the number of other surgical procedures (Chart 1-16).

• Tables 1-10 and 1-11 show the amounts in Reais and International Dollars for the year 2019 (Int$2019), respectively, paid by the public health system for clinical cardiovascular admissions, in Brazil, from 2008 to 2019. The total amount spent on those hospitalizations was R$ 9 378 278, corresponding to Int$ 6 170 381 in 2019. Heart failure, cerebrovascular diseases and coronary artery disease syndromes were responsible for most of those values.

• The amounts paid for surgical procedures for CVD are shown in Reais in Table 1-12 and in Int$2019 in Table 1-13. Although they represent a smaller number as compared to clinical procedures, they were responsible for higher expenses, with R$ 10 524 044 spent, equivalent to Int$2019 6 853 635. The procedures used for the treatment of IHD, including coronary angioplasty and CABG, accounted for the largest fraction of those expenses.

### Future Research

• The SIM, implemented in 1975, is an essential tool for monitoring mortality statistics in Brazil, because the registration of all deaths is mandatory in the FUs, with 98% coverage of the national territory in 2017, that coverage being lower in the Northern region than in the Southern region. The Northeastern region has the poorest coverage, still under 95%. ^43^ Although SIM has improved through specific Ministry of Health projects, ^44^ ^,^ ^45^ problems persist, such as ill-defined causes (around 6%), garbage codes and underreporting of deaths, which generate biases that may disrupt the metrics presented. As such, further research is needed to promote methodological adjustments for coverage, redistribution of ill-defined causes, especially in the older years of the historical series. On the other hand, the estimates from the GBD Study need additional research to implement models with better distribution of garbage codes adapted to local realities.

• It is worth mentioning that there is a lack of primary incidence data (cohorts) in Brazil, requiring research that allows us to understand how to face CVD in states and populations with low socioeconomic indices.

• Because of the reduction in the decline trend of age-standardized CVD mortality in the last 5 years, novel strategies to tackle CVD mortality must be studied. Understanding of the drivers of this change is essential to implement effective policies, particularly facing population aging, which will increase the number of individuals with CVD in the country.

## 2. STROKE (CEREBROVASCULAR DISEASES)

### ICD-9 430 to 438; ICD-10 I60 to I69

**See Tables 2-1 through 2-12 and Charts 2-1 through 2-3**

**Table:** Abbreviations Used in Chapter 2

ACEI/ARB	Angiotensin Converting Enzyme Inhibitor/Angiotensin Receptor Blocker
CHD	Coronary Heart Disease
CI	Confidence Interval
DALYs	Disability-Adjusted Life Years
DATASUS	Brazilian Unified Health System Database
GBD	Global Burden of Disease
HS	Hemorrhagic Stroke
ICD	International Statistical Classification of Diseases and Related Health Problems
ICD-9	International Statistical Classification of Diseases and Related Health Problems, 9^th^Revision
ICD-10	International Statistical Classification of Diseases and Related Health Problems, 10^th^Revision
ICH	Intracerebral Hemorrhage
IMPACT-AF	Improve Treatment with Anticoagulants in Patients with Atrial Fibrillation
INR	International Normalized Ratio
IRR	Incidence Rate Ratio
IS	Ischemic Stroke
MAPS	Matão Preventing Stroke Study
MELAS	Mitochondrial Encephalomyopathy, Lactic Acidosis and Stroke-Like Episodes
NOAC	Non-Vitamin K Antagonist Oral Anticoagulant
OR	Odds Ratio
PNS	National Health Survey (in Portuguese, *Pesquisa Nacional de Saúde* )
PURE	Prospective Urban Rural Epidemiological Study
RR	Relative Risk
SAH	Subarachnoid Hemorrhage
SD	Standard Deviation
SDI	Sociodemographic Index
SIM	Brazilian Mortality Information System (in Portuguese, *Sistema de Informações sobre Mortalidade* )
SSQOL	Stroke Specific Quality of Life Scale)
TIA	Transient Ischemic Attack
UI	Uncertainty Interval
VKA	Vitamin K Antagonist
WHO	World Health Organization
YLDs	Years Lived with Disability
YLLs	Years of Life Lost

**Table 2-1:** – Number of cases and age-standardized prevalence rates (per 100 000) of ischemic stroke, subarachnoid hemorrhage, and intracerebral hemorrhage in 1990 and 2019, and percent change of rates, in Brazil and its Federative Units.

Cause of death and location	1990		2019		Percent change (95% UI)
	Number (95% UI)	Rate (95% UI)	Number (95% UI)	Rate (95% UI)	
**B.2.3.1-Ischemic stroke**					
Acre	2337 (2005;2670.8)	1179.3 (1021.6;1368)	5959.2 (5165.9;6781.1)	876.1 (762.2;998)	-25.7(-30.7;-20.7)
Alagoas	20840.6 (17963.5;24003.8)	1415.7 (1222.3;1668.6)	33233.7 (29180.3;38341.4)	1016.1 (895.6;1175.5)	-28.2(-33.5;-22.9)
Amapá	1384 (1196.3;1573.2)	1124.1 (970.4;1298.2)	5077.4 (4424.8;5767.2)	861.2 (746.8;984.5)	-23.4(-28.6;-18)
Amazonas	11728.4 (10152.6;13440.3)	1240 (1069.8;1424.9)	26360.1 (23112.3;29848.5)	832.7 (729.8;950.6)	-32.8(-37.7;-27.4)
Bahia	92983.6 (80433.9;106701.5)	1252.7 (1072.5;1448.2)	140578.2 (122673.2;160762.3)	870.8 (760.2;1001.2)	-30.5(-35.3;-25.6)
Brazil	1287969.4 (1118323.7;1460716)	1327.6 (1151.2;1516)	2040376.9 (1784219.6;2330526)	870.1 (761.1;992.8)	-34.5(-36.7;-32.2)
Ceará	45261 (38660.3;51639)	1031.5 (880.1;1192.4)	85094.6 (74109.4;97715.8)	851.1 (740.3;977.2)	-17.5(-23;-11.4)
Distrito Federal	9491.3 (8108.7;10933.6)	1301.3 (1110.6;1516.4)	24286.9 (20992.9;28153.4)	885.1 (765.4;1030.9)	-32(-36.7;-26.9)
Espírito Santo	22957.7 (19897.4;26104.4)	1423 (1222.2;1642.3)	39703.5 (34433.2;45287.7)	913.4 (795.3;1041.4)	-35.8(-40.2;-31.2)
Goiás	30398.4 (26103.6;34903.4)	1266.7 (1089.5;1464)	57813.2 (50438.7;65757.5)	813.4 (711.9;925.3)	-35.8(-40.6;-31.2)
Maranhão	33415.7 (28606.1;38354.7)	1174.6 (998.4;1372.4)	57772.9 (50605.5;65580.5)	839.2 (735.7;956.6)	-28.6(-35;-20.2)
Mato Grosso	11695.9 (10093.3;13434.1)	1207.4 (1043.7;1397)	28745.8 (25006.5;32932)	838 (727.9;961.7)	-30.6(-36;-25)
Mato Grosso do Sul	12959.8 (11279.9;14803.9)	1255.5 (1093.2;1450.7)	25831.6 (22483.5;29207.6)	870.8 (758.4;984.9)	-30.6(-36.5;-25.4)
Minas Gerais	145398.6 (125793.7;168354.4)	1356.8 (1168.7;1571.8)	217642.4 (189023.2;251547)	843.5 (732.9;971.6)	-37.8(-42.8;-32.7)
Pará	32096.8 (27597.1;36767.6)	1325.8 (1132;1527.9)	64670.6 (56252.5;73679.5)	869 (753.8;991.8)	-34.5(-39.3;-29.4)
Paraíba	26607.1 (22881.2;30854.6)	1096.5 (940.9;1284.1)	37478 (32824.1;42797.6)	804.4 (704;921.6)	-26.6(-32;-21.3)
Paraná	81476.8 (70660.4;93825.4)	1534.4 (1322.5;1786.7)	126272.5 (109037.2;145179.1)	960.2 (830.4;1100)	-37.4(-42;-32.1)
Pernambuco	63426.2 (54597.3;73733.5)	1300.5 (1112.3;1536.8)	89359.9 (77947.2;102237.8)	884.3 (769.8;1015)	-32(-36.4;-26.9)
Piaui	17859.3 (15361.8;20573.6)	1143.9 (986.6;1328.8)	31595 (27482.8;36104)	843.1 (733.5;963)	-26.3(-31.3;-20.4)
Rio de Janeiro	151283.8 (130994.6;173336.9)	1489 (1283.3;1706.2)	197147.1 (170574.1;228003.1)	902.7 (783;1039.9)	-39.4(-44.1;-34.7)
Rio Grande do Norte	18111.6 (15616.7;20695.6)	1056.5 (908.6;1217.1)	29607.2 (25837.3;34049.1)	766.9 (670.6;883.4)	-27.4(-31.6;-22.4)
Rio Grande do Sul	98174.7 (84791.3;112264.8)	1410.7 (1218.8;1619.6)	138340.7 (118660.6;159686.5)	926.6 (799.2;1061.2)	-34.3(-39.1;-29.5)
Rondônia	6438.9 (5505.5;7461.4)	1477.7 (1270.8;1735.5)	13891.4 (11998.5;15994.2)	861.5 (741.1;998.2)	-41.7(-46.2;-36.8)
Roraima	980.8 (844.6;1128.1)	1199.9 (1033.2;1385.3)	3514.8 (3044.9;4034.3)	830.3 (719.9;952.8)	-30.8(-35.5;-25.7)
Santa Catarina	38488 (33075.1;43771.1)	1392.8 (1183.7;1603)	68595.6 (59198.3;78591.2)	848.8 (733.7;970.5)	-39.1(-43.9;-34.1)
São Paulo	295180.2 (255885.5;336598.3)	1344.1 (1162.1;1524.7)	458484.1 (395229.9;529532.9)	857 (741.3;982.9)	-36.2(-40.8;-31.8)
Sergipe	11546.5 (9966.9;13183)	1306.7 (1118.5;1507.3)	21060.5 (18405.7;24150.7)	920.1 (799.5;1060.9)	-29.6(-34.9;-25)
Tocantins	5446.7 (4686.9;6267.8)	1148 (974.7;1327.9)	12260 (10740.9;14034.4)	827.6 (721.1;948.7)	-27.9(-32.9;-21.9)
**B.2.3.2-Intracerebral hemorrhage**					(;)
Acre	954.6 (816;1099.4)	423.3 (363.8;486.7)	2357.5 (2036.2;2708.6)	312.6 (271.5;357.9)	-26.1(-30.5;-21.2)
Alagoas	7759.2 (6563.3;8947.2)	487.7 (412.6;564.5)	12212.5 (10587.5;14019.8)	352.5 (305.3;404.1)	-27.7(-32.4;-23)
Amapá	580.2 (496.8;667.5)	412.4 (356.1;474.8)	2046.9 (1773;2347.9)	303.5 (264;346.5)	-26.4(-30.5;-21.6)
Amazonas	5190.3 (4456.4;5996.5)	481.6 (414.4;554.5)	10793.9 (9317.7;12412.6)	309.3 (268;353.8)	-35.8(-39.8;-31.4)
Bahia	37762.4 (32554.4;43452.4)	480.3 (409.2;551.3)	53634.2 (46593.5;61495.6)	323.4 (281.1;370.3)	-32.7(-37.5;-28)
Brazil	541445.3 (466619.8;621909.3)	507.5 (438.9;584.1)	757903 (659245.3;867100.5)	315.9 (275;361.4)	-37.7(-40.5;-34.9)
Ceará	17834.5 (15383.5;20700.6)	390.6 (335.7;452.2)	31985.9 (27865.3;36677)	313.1 (273.4;358.8)	-19.8(-24;-14.5)
Distrito Federal	4754.5 (4045.6;5533.3)	521.2 (448.3;599.5)	9476.3 (8096.4;10959.5)	312.1 (270.2;359)	-40.1(-44.5;-35.6)
Espírito Santo	9923.8 (8526.1;11356.7)	553.1 (476.3;631.1)	15127.1 (13005.6;17294.3)	336.8 (291;385.1)	-39.1(-43.1;-34.9)
Goiás	14065.7 (12162.2;16282.3)	513.3 (443.4;589.5)	22525 (19579.7;25806.5)	301 (261.7;345)	-41.4(-45.5;-36.8)
Maranhão	12434.4 (10659.9;14397.9)	406.8 (349.9;471.3)	22610.6 (19506.8;26011.2)	315.1 (271.1;361.5)	-22.6(-27.6;-17.4)
Mato Grosso	5048.2 (4327.5;5880.2)	432.5 (371.8;500.3)	10830.6 (9392.7;12448)	292.1 (254.7;333.1)	-32.5(-36.9;-28.1)
Mato Grosso do Sul	6065.5 (5157.5;7014.4)	515.4 (441.4;596.7)	10049.7 (8640.4;11486.9)	325.5 (281.2;371.4)	-36.8(-41.2;-32.6)
Minas Gerais	63178.6 (54522.8;72902.3)	536.4 (464.4;616.6)	80317.8 (69705.8;91963.5)	310.7 (269.7;355.8)	-42.1(-46.7;-37.9)
Pará	13066 (11192.2;15083.4)	477.6 (408;553.7)	24092.9 (20923.5;27440.9)	296.6 (256.5;336.5)	-37.9(-41.9;-33.6)
Paraíba	9857.1 (8470.4;11300.2)	404 (345;465.3)	15120.2 (13151.5;17216.3)	324.7 (282.5;369.1)	-19.6(-25;-13.7)
Paraná	33661.9 (28718.5;38871.7)	555.3 (471;641.8)	42595 (36576.4;48876.7)	317.7 (273.5;363.3)	-42.8(-47.4;-38)
Pernambuco	26388.4 (22669.9;30514.5)	512.8 (442.7;592.5)	35843.8 (31160.7;41052.3)	342.4 (298.8;390.8)	-33.2(-37.5;-28.7)
Piaui	7154.6 (6136.8;8240.6)	424.7 (362.6;489.1)	11367.7 (9877.1;13067.2)	300.8 (261.7;345.3)	-29.2(-33.2;-24.6)
Rio de Janeiro	67411.9 (57661.1;77654.6)	603.2 (517.9;694.9)	78112.1 (67686.5;89538.8)	357.8 (310.3;408.4)	-40.7(-45.2;-35.4)
Rio Grande do Norte	6989.6 (6011.1;7956.2)	398 (342;455.7)	11368.6 (9897.4;13069.4)	288.4 (250.7;331.2)	-27.5(-31.9;-22.8)
Rio Grande do Sul	40477.3 (34799.8;46418.5)	528.1 (457.3;604.3)	48534.9 (41907.9;55770.7)	331.4 (286.5;380)	-37.3(-41.4;-32.7)
Rondônia	2937.5 (2502.1;3408.2)	508.6 (437;589.8)	5212 (4528.8;5996.9)	295.6 (257.4;339.4)	-41.9(-46;-37.5)
Roraima	451.4 (384.2;525.4)	437.1 (375.7;504.9)	1327.3 (1137.7;1541.9)	273.8 (236.1;316.6)	-37.4(-41.3;-32.9)
Santa Catarina	17321.8 (14777.5;19977.4)	546.2 (467.7;637.7)	25189.2 (21733.7;28815.9)	301.5 (260.5;343.6)	-44.8(-49;-39.9)
São Paulo	123269.3 (105277.2;143037.4)	503.6 (431.8;583)	162501.2 (139844.8;186907.7)	299.1 (258.2;342.1)	-40.6(-45.1;-35.6)
Sergipe	4551.5 (3900.5;5230.3)	481.6 (416.8;554.1)	7971.1 (6945.4;9114.2)	330.2 (288.3;376.9)	-31.5(-36;-26.7)
Tocantins	2355.2 (2010.9;2701.8)	431 (373.5;496.1)	4699.2 (4080.2;5401.4)	300.2 (260.2;345.9)	-30.4(-34.9;-25.1)
**B.2.3.3-Subarachnoid hemorrhage**					(;)
Acre	361.4 (300.3;435.9)	152.2 (126;183.9)	990.2 (823.1;1197.9)	124.4 (104;150.2)	-18.3(-22.6;-13.9)
Alagoas	2547.4 (2107.3;3083.5)	154.5 (127.6;187.9)	4406.8 (3654.6;5267.4)	123.5 (102.4;147.2)	-20.1(-24.5;-15.4)
Amapá	232.1 (191.6;278.8)	155 (127.3;188.1)	902.6 (751;1079.4)	125.5 (104.2;151.7)	-19(-23.5;-14.9)
Amazonas	1787.5 (1480.7;2176.3)	150.7 (123.5;183.8)	4505.5 (3742.5;5421.6)	121.9 (101.6;147.5)	-19.1(-23.2;-14.9)
Bahia	12581 (10473.6;15135.6)	156.6 (129.2;189.2)	21268.5 (17762.4;25776.8)	125.7 (104.9;152.1)	-19.8(-24.2;-15.7)
Brazil	178322.3 (147412.5;215453.5)	158.6 (131.7;192.4)	306334.8 (255287.2;369754.8)	124.8 (104.2;150.1)	-21.3(-24.3;-18.6)
Ceará	7235.2 (5975.9;8843.8)	157.6 (130.3;193.1)	13166.1 (10906.1;15881)	126.6 (104.8;152.9)	-19.7(-24.1;-15.1)
Distrito Federal	1789.8 (1478.1;2170.4)	165.8 (137.2;200.5)	4274.5 (3539.2;5205.6)	127.6 (106.3;155)	-23(-28;-17.1)
Espírito Santo	3071.7 (2545.7;3713.8)	160.5 (131.9;194.5)	5799.8 (4800.4;7005.5)	125.4 (104.1;150.5)	-21.8(-26.2;-17.1)
Goiás	4544.9 (3780.9;5488.7)	152.2 (126;184.1)	9354.7 (7742.9;11331.2)	119.6 (99.4;144.5)	-21.5(-25.8;-16.7)
Maranhão	4907.9 (4058;5876.5)	157.5 (130.2;189.6)	9393.7 (7853.4;11298.1)	129.3 (107.2;156.1)	-17.9(-22.4;-13.8)
Mato Grosso	1932.7 (1599.2;2343.7)	149.6 (123.5;182.8)	4740.3 (3954.5;5734.8)	120.7 (101.2;145.4)	-19.3(-23.6;-14.3)
Mato Grosso do Sul	1977.1 (1624.8;2389.4)	154.9 (127.5;189)	3968.7 (3292.1;4814.3)	124 (103.4;150)	-19.9(-24.3;-15.2)
Minas Gerais	20106.5 (16619.3;24414.9)	163.5 (134.5;198.3)	32921.5 (27309.6;39771.4)	125.7 (104.6;151.1)	-23.1(-28.3;-18.2)
Pará	4512.4 (3724.9;5437.7)	152.3 (125.7;184.8)	10338 (8575.1;12465.8)	122.3 (101.6;147.1)	-19.7(-24;-15.4)
Paraíba	3901.8 (3225.5;4735.4)	163.9 (135.7;200.2)	6045.8 (4997.5;7325.1)	128.7 (106.4;156.2)	-21.5(-25.9;-17.1)
Paraná	10404.5 (8631.1;12629.7)	158 (130.9;191.8)	17179.4 (14139.7;20873.3)	124.7 (103.1;150.9)	-21.1(-25.6;-16.1)
Pernambuco	7989.4 (6624.6;9835.5)	151.4 (124.4;187.4)	13170.6 (10925.4;15923.5)	122.4 (102.2;147.9)	-19.1(-24.1;-14.6)
Piaui	2775.1 (2286;3362.5)	161 (132.6;196.5)	4913.8 (4082.1;5920.7)	129.1 (107.3;155.4)	-19.9(-23.9;-15.1)
Rio de Janeiro	18926.8 (15641.4;22940.3)	161.2 (133.5;195)	27140.4 (22430.8;33138.9)	123.4 (102.3;149.7)	-23.5(-27.8;-18.4)
Rio Grande do Norte	2710.1 (2246.3;3278)	156.1 (129;189.2)	5013.6 (4186.9;6053.4)	124.6 (103.6;150.3)	-20.2(-24.5;-16.2)
Rio Grande do Sul	12748.3 (10540.8;15532.4)	157.7 (130.5;192.4)	18004.8 (14901.9;21850.4)	123.5 (102.9;148.8)	-21.7(-25.9;-17.1)
Rondônia	991.8 (815.5;1209.9)	146 (120.3;177.6)	2239.9 (1839.2;2710.2)	119.4 (98.9;144.4)	-18.3(-22.8;-13.7)
Roraima	168.9 (139.2;204.8)	142.1 (117.1;172.1)	602.9 (497.8;734.5)	115.7 (96.1;140.5)	-18.5(-22.8;-14.3)
Santa Catarina	5514.6 (4565.3;6634.1)	159.2 (131.1;192.1)	10720.7 (8888.1;13027.3)	123.3 (102.8;149)	-22.6(-27.3;-17.5)
São Paulo	42197.3 (34696.2;51220.6)	160.4 (132.4;194.9)	70133.3 (58163.2;84885.3)	126.1 (105.1;151.9)	-21.4(-25.5;-16.9)
Sergipe	1498.7 (1239.1;1808.9)	154 (127.2;186.4)	3089.5 (2565.1;3754.7)	123.2 (102.8;149.3)	-20(-24.3;-15.6)
Tocantins	907.2 (754.4;1098.1)	154.9 (128.2;187.3)	2048.9 (1698.5;2471.5)	126.2 (105.1;151.9)	-18.5(-22.7;-14.1)

*Source: Data derived from Global Burden of Disease Study 2019, Institute for Health Metrics and Evaluation, University of Washington.* ^46^

**Table 2-2:** – Number of cases and age-standardized prevalence rates (per 100 000) of stroke, ischemic stroke, subarachnoid hemorrhage, and intracerebral hemorrhage in 1990 and 2019, and percent change of rates, in Brazil, according to age group.

Cause of death and age group	1990		2019		Percent change (95% UI)
	Number (95% UI)	Rate (95% UI)	Number (95% UI)	Rate (95% UI)	
**B.2.3-Stroke**					
*Under 5*	4182.3 (2610.7;6524.5)	24.7 (15.4;38.5)	3235.1 (2120.2;4898.9)	20.9 (13.7;31.6)	-15.4 (-33;9.6)
15-49 years	623994.4 (545014.4;705854.8)	814.1 (711.1;920.9)	786738.6 (690686.9;888810.4)	681.2 (598.1;769.6)	-16.3 (-19;-13.4)
50-69 years	825075.6 (723316.1;937034.1)	5259.4 (4610.7;5973.1)	1296990 (1151199.7;1476134)	3214.9 (2853.5;3658.9)	-38.9 (-41;-36.8)
5-14 years	48928 (35610.6;66858.5)	138.5 (100.8;189.2)	40623.7 (30515;54156.4)	126 (94.6;167.9)	-9 (-16.7;-0.1)
70+ years	410923.1 (347995.9;484341)	9714.3 (8226.7;11449.9)	857424.4 (735950.3;1005011.8)	6551 (5622.9;7678.6)	-32.6 (-35.4;-29.7)
Age-standardized	1913103.4 (1735455;2095724.2)	1909.3 (1733.1;2100.1)	2985011.7 (2716616.7;3280843.8)	1256.6 (1142.6;1381.1)	-34.2 (-35.8;-32.5)
All Ages	1913103.4 (1735455;2095724.2)	1285.4 (1166;1408.1)	2985011.7 (2716616.7;3280843.8)	1377.7 (1253.8;1514.2)	7.2 (4.4;10.4)
**B.2.3.1-Ischemic stroke**					
*Under 5*	6688.4 (4586.3;9999.2)	39.5 (27.1;59)	5640.4 (3913.2;8255.7)	36.4 (25.3;53.3)	-7.8 (-13.1;0.7)
15-49 years	343498.3 (275888.5;421506.2)	448.2 (360;549.9)	451442.9 (365495;550221.4)	390.9 (316.5;476.4)	-12.8 (-16.3;-9.1)
50-69 years	539690.6 (446267.3;646791)	3440.2 (2844.7;4122.9)	839214.7 (703174.9;1002009.6)	2080.2 (1743;2483.7)	-39.5 (-42.6;-36.7)
5-14 years	46198.9 (31663;66393)	130.8 (89.6;187.9)	38107.7 (26846.3;53603.5)	118.2 (83.2;166.2)	-9.6 (-13.9;-4.3)
70+ years	351893.2 (283271.1;428872)	8318.8 (6696.6;10138.6)	705971.2 (577001.6;861003.9)	5393.8 (4408.5;6578.3)	-35.2 (-38.3;-32)
Age-standardized	1287969.4 (1118323.7;1460716)	1327.6 (1151.2;1516)	2040376.9 (1784219.6;2330526)	870.1 (761.1;992.8)	-34.5 (-36.7;-32.2)
All Ages	1287969.4 (1118323.7;1460716)	865.4 (751.4;981.4)	2040376.9 (1784219.6;2330526)	941.7 (823.5;1075.6)	8.8 (5;12.8)
**B.2.3.2-Intracerebral hemorrhage**					
*Under 5*	1161.2 (784.1;1623.7)	6.9 (4.6;9.6)	1147 (789.8;1578.7)	7.4 (5.1;10.2)	8 (1.9;15.4)
15-49 years	218246.5 (176752.2;264569.5)	284.7 (230.6;345.2)	253649.5 (207356.6;301299.5)	219.6 (179.5;260.9)	-22.9 (-26.2;-19.1)
50-69 years	240417.8 (196762.5;288416.9)	1532.5 (1254.3;1838.5)	341586.5 (285413.3;400854.7)	846.7 (707.5;993.6)	-44.8 (-47.4;-41.8)
5-14 years	13416.1 (9141.6;18251.2)	38 (25.9;51.7)	12613.2 (8880.5;17085.2)	39.1 (27.5;53)	3 (-3;10.7)
70+ years	68203.7 (55601.8;84221.2)	1612.4 (1314.4;1991)	148906.8 (121630.5;183577)	1137.7 (929.3;1402.6)	-29.4 (-34;-23.6)
Age-standardized	541445.3 (466619.8;621909.3)	507.5 (438.9;584.1)	757903 (659245.3;867100.5)	315.9 (275;361.4)	-37.7 (-40.5;-34.9)
All Ages	541445.3 (466619.8;621909.3)	363.8 (313.5;417.9)	757903 (659245.3;867100.5)	349.8 (304.3;400.2)	-3.8 (-8;0.3)
**B.2.3.3-Subarachnoid hemorrhage**					
*Under 5*	253.7 (139.8;383.7)	1.5 (0.8;2.3)	245.7 (137.5;369)	1.6 (0.9;2.4)	5.9 (2.7;10.8)
15-49 years	92830.8 (74524.7;114996.4)	121.1 (97.2;150)	124691.1 (100254.7;154948.8)	108 (86.8;134.2)	-10.9 (-13.5;-8.4)
50-69 years	71954.7 (56126.6;92135.2)	458.7 (357.8;587.3)	145941.5 (113631.8;186025.6)	361.7 (281.7;461.1)	-21.1 (-24.4;-17.8)
5-14 years	2881.6 (1811.9;4260.4)	8.2 (5.1;12.1)	2715.1 (1735.5;4024.5)	8.4 (5.4;12.5)	3.2 (0.4;7.1)
70+ years	10401.5 (7385.5;13977.2)	245.9 (174.6;330.4)	32741.3 (24045.6;42870.8)	250.2 (183.7;327.5)	1.7 (-3.9;8.6)
Age-standardized	178322.3 (147412.5;215453.5)	158.6 (131.7;192.4)	306334.8 (255287.2;369754.8)	124.8 (104.2;150.1)	-21.3 (-24.3;-18.6)
All Ages	178322.3 (147412.5;215453.5)	119.8 (99;144.8)	306334.8 (255287.2;369754.8)	141.4 (117.8;170.7)	18 (12.8;23.4)

*Source: Data derived from Global Burden of Disease Study 2019, Institute for Health Metrics and Evaluation, University of Washington.^*46*^*

**Table 2-3:** – Number of cases and age-standardized incidence rates (per 100 000) of stroke, ischemic stroke, subarachnoid hemorrhage, and intracerebral hemorrhage in 1990 and 2019, and percent change of rates, in Brazil and its Federative Units.

Cause of death and location	1990		2019		Percent change (95% UI)
	Number (95% UI)	Rate (95% UI)	Number (95% UI)	Rate (95% UI)	
**B.2.3-Stroke**					**(;)**
Acre	411.9 (369.4;460.7)	210.4 (188.3;236.4)	887.5 (791.6;996.9)	133.5 (118.8;151.1)	-36.5(-38.7;-34)
Alagoas	3525.9 (3156.5;3980.9)	241.9 (215.3;273.6)	5078.4 (4533.7;5720.3)	155.2 (138.5;175.1)	-35.8(-37.9;-33.3)
Amapá	242.1 (217.7;270.6)	200 (178.2;226.3)	759.4 (676.7;858.9)	132 (117.3;149.8)	-34(-36.3;-31.7)
Amazonas	1967.1 (1759.6;2201.3)	208.6 (186.4;234.7)	3964.4 (3537.2;4450)	127.4 (113.7;144.3)	-38.9(-41.3;-36.7)
Bahia	16371.4 (14690.4;18405.9)	223.4 (200.3;252.7)	22215.8 (19841.1;25012.1)	136.9 (122.1;154.2)	-38.7(-40.8;-36.4)
Brazil	216640.6 (193728.5;242758.9)	224.6 (201.6;251.8)	295510.5 (264160.9;331953.6)	127 (113.8;142.1)	-43.5(-44.7;-42.2)
Ceará	8168.8 (7330.2;9148.3)	188.5 (169;212.2)	13059.6 (11671.9;14651.1)	130.8 (116.7;147.2)	-30.6(-33.2;-28)
Distrito Federal	1677.4 (1505;1870.5)	223 (199.4;249.7)	3131.6 (2790.5;3507.5)	116.6 (104;131.1)	-47.7(-49.7;-45.3)
Espírito Santo	3991.3 (3566.8;4491.6)	247.6 (219.9;279.8)	5720.5 (5104.5;6463.7)	132.7 (118.5;149.9)	-46.4(-48.6;-44.3)
Goiás	5462.9 (4882.1;6113.3)	229.2 (204.1;257.5)	8554.8 (7617.5;9655.7)	122.6 (109.5;138)	-46.5(-48.6;-44.1)
Maranhão	5729.8 (5149.3;6435.9)	203.2 (181.4;228.3)	9602.8 (8603.6;10833.2)	139.7 (124.9;157.8)	-31.2(-33.9;-28.4)
Mato Grosso	2066.5 (1850.5;2302.2)	213.6 (190.7;242.1)	4224.3 (3790.6;4757.8)	125.1 (112.3;141.8)	-41.4(-43.6;-38.9)
Mato Grosso do Sul	2349.4 (2107.6;2641.2)	228.7 (204.5;256.2)	3816 (3397.7;4324.3)	130.2 (116.1;146.7)	-43(-45.2;-40.6)
Minas Gerais	25738.5 (23130.3;28837.3)	240.8 (215.7;270.8)	32636.7 (29004.9;36771.1)	126.8 (113;142.6)	-47.3(-49.3;-45.4)
Pará	5334.6 (4779.3;5982.6)	220.2 (197;248.2)	9786.9 (8771.1;11013.3)	133 (118.6;150.4)	-39.6(-42.1;-37.2)
Paraíba	4666.9 (4181;5252)	196.3 (175.9;220.6)	5957.2 (5352.3;6650.6)	126.3 (113.5;141.3)	-35.7(-37.9;-33.3)
Paraná	13267.4 (11814.3;14914.7)	249.3 (222.8;281.2)	17372.2 (15503.4;19691.5)	134 (119.9;151.3)	-46.3(-48.3;-44.2)
Pernambuco	10750.5 (9606;12078.3)	224.3 (200.4;252.3)	13678.5 (12252.1;15426)	136.1 (121.7;153.6)	-39.3(-41.5;-36.9)
Piaui	3149.6 (2829.2;3542)	205.9 (184.8;232)	5037.5 (4505.3;5682.3)	133.4 (119.2;150.7)	-35.2(-37.3;-32.7)
Rio de Janeiro	25814.8 (23106.8;29202.3)	254.6 (228.2;287.6)	28566.5 (25517.7;32324)	131.8 (118.1;148.5)	-48.2(-50.2;-46.2)
Rio Grande do Norte	3299.3 (2948.3;3707)	196.4 (175.2;220.9)	4620.3 (4134.6;5184.7)	118.7 (106;133.6)	-39.6(-41.8;-37.2)
Rio Grande do Sul	16023.3 (14290.6;18026.9)	232 (207.3;260.5)	19270.5 (17107.4;21805.9)	129.9 (115.6;146.2)	-44(-46.1;-41.5)
Rondônia	1083.1 (962.8;1214)	238.5 (212.4;268.3)	2001.6 (1796.8;2250.1)	126.3 (113.1;142.4)	-47.1(-49.1;-45)
Roraima	169.2 (150.9;189.3)	210 (187.1;237.4)	498.4 (443.6;559)	121.8 (108.8;137.9)	-42(-44.2;-39.6)
Santa Catarina	6051.7 (5428.2;6799.7)	217.3 (194;244)	9233.2 (8225;10363.8)	116.9 (104.7;130.7)	-46.2(-48.4;-43.9)
São Paulo	46409.7 (41385.4;52301.6)	211.8 (189;237.8)	60790.5 (54232.4;68365.1)	115.3 (103.1;129.1)	-45.6(-47.6;-43.2)
Sergipe	1934 (1730;2172.7)	223.7 (199.5;252.8)	3130.2 (2788.5;3515.3)	137.5 (121.9;154.9)	-38.5(-41;-36.1)
Tocantins	983.6 (880.8;1103.9)	209.4 (186.8;235.8)	1915.1 (1711.7;2149.6)	130.4 (116.1;146.8)	-37.7(-39.9;-35.4)
**B.2.3.1-Ischemic stroke**					(;)
Acre	234.7 (198.1;276)	129.1 (109.1;153.4)	519.9 (437.3;621.3)	83.1 (69.5;100.1)	-35.7(-39;-32.1)
Alagoas	2126.1 (1797.7;2557.6)	152.1 (127.7;182.7)	3132.2 (2649.1;3741)	98.3 (83.1;117.5)	-35.4(-38.5;-31.9)
Amapá	137.8 (116.4;163.5)	123.5 (104.7;146.9)	443.2 (371.4;527.4)	83 (69.3;100.2)	-32.8(-36.4;-29.4)
Amazonas	1115.4 (942.3;1321.9)	128 (107.5;153)	2320 (1961.8;2766.2)	78.9 (66.2;94.7)	-38.4(-41.8;-35.2)
Bahia	9662.6 (8194;11470.4)	137 (115.2;163.5)	13529.8 (11418.1;16201.2)	84.4 (71.3;101.4)	-38.4(-41.6;-35.1)
Brazil	124392.2 (105330.6;147825.6)	136.6 (115.7;163.1)	179196.5 (151357.9;214373.1)	78.2 (66.1;93)	-42.7(-44.3;-41)
Ceará	4856.7 (4120.9;5778.9)	114.8 (97.5;137.8)	7890.5 (6663.6;9437.6)	80 (67.3;95.9)	-30.4(-34.1;-26.7)
Distrito Federal	868.1 (723.3;1033.8)	134 (112.8;159.3)	1809.8 (1509.1;2188.5)	72.3 (60.7;86.7)	-46.1(-49.1;-42.6)
Espírito Santo	2275.7 (1914.3;2715.3)	150.5 (125.8;179.6)	3467 (2922.3;4169)	82.2 (69.5;98.8)	-45.4(-48.6;-42.1)
Goiás	2992.8 (2527.4;3552.5)	136.8 (114.5;163.6)	5058.9 (4248.6;6087.8)	75.1 (63.2;89.3)	-45.1(-48.1;-41.4)
Maranhão	3396.3 (2881.3;4007.1)	125.3 (105.4;149.2)	5690.4 (4787.9;6828.1)	84.6 (70.6;102.1)	-32.5(-36.1;-28.1)
Mato Grosso	1153.3 (973;1377.4)	132.4 (111.5;158.9)	2517.5 (2120.5;3035.3)	78.1 (65.7;93.6)	-41(-44.3;-37.3)
Mato Grosso do Sul	1292.2 (1089.1;1537.5)	136.7 (115.3;162.6)	2274.4 (1911.5;2740.1)	79.7 (67;95.5)	-41.7(-44.5;-38.3)
Minas Gerais	14540.3 (12317.9;17373.4)	144.4 (122.3;173)	19901.1 (16652.3;23738.5)	77.6 (65.5;92.1)	-46.3(-49;-43.5)
Pará	3096.2 (2613.6;3679.7)	136.9 (115.2;164)	5917.9 (4992.5;7076.2)	84.1 (70.4;101)	-38.6(-42.2;-35.2)
Paraíba	2758.3 (2324.9;3314.4)	116.9 (98.1;139.2)	3468.1 (2933.9;4111.9)	73.2 (61.8;87.2)	-37.3(-40.7;-33.6)
Paraná	7723.4 (6504.8;9218.1)	155.3 (130.9;185.7)	10972 (9206.3;13160.8)	85.9 (72.6;102.6)	-44.7(-47.5;-41.6)
Pernambuco	6284.5 (5253.6;7513.5)	135.6 (114.1;162.7)	8045.9 (6746.4;9649)	81.9 (68.3;98.3)	-39.6(-43;-35.9)
Piaui	1833.4 (1549.5;2202.9)	125.4 (106;151.7)	3104.4 (2627.3;3712.2)	82.4 (69.6;98.3)	-34.3(-37.4;-30.3)
Rio de Janeiro	14572.8 (12240.7;17353.3)	152.1 (128.2;181.2)	17229.2 (14426.9;20533.9)	79.8 (67.5;94.4)	-47.6(-50.4;-44.4)
Rio Grande do Norte	1983.6 (1678.6;2356)	120.1 (101.2;142.8)	2791.3 (2349.1;3316.4)	72.3 (60.9;85.8)	-39.8(-43;-36.4)
Rio Grande do Sul	9402.5 (7929.3;11220)	143.6 (121.4;171.7)	12220.3 (10214;14674.4)	81.9 (68.8;97.5)	-43(-46.1;-39.6)
Rondônia	593.2 (495.3;710.8)	149.9 (126;178.4)	1196.9 (1010.6;1430)	79.4 (66.9;94.5)	-47(-49.9;-44.3)
Roraima	91.8 (76.6;108.4)	130.7 (109.5;155.8)	296.9 (251.5;353.1)	77.9 (66.1;92.9)	-40.4(-43.5;-36.5)
Santa Catarina	3274.2 (2755.5;3906.2)	127.5 (107.2;151.8)	5606.5 (4695.5;6715.2)	73 (61.2;86.8)	-42.8(-46;-39.1)
São Paulo	26425.9 (22386.7;31427.6)	129 (109.4;154.4)	36725.9 (30859.2;43889.2)	70.8 (59.9;83.9)	-45.1(-47.9;-42)
Sergipe	1153.2 (971.2;1386.3)	139.3 (117.5;167.3)	1914.7 (1612.8;2293.7)	86.5 (72.8;104)	-37.9(-41.4;-34.3)
Tocantins	547.4 (457.3;652.8)	126.5 (105.7;150.2)	1151.6 (970.8;1370.7)	81 (68.2;96.7)	-35.9(-39;-32.4)
**B.2.3.2-Intracerebral hemorrhage**					(;)
Acre	126.1 (107.3;149.1)	60 (50.8;70.3)	233.4 (196.3;275.5)	33 (27.8;39.1)	-45(-47.6;-42.2)
Alagoas	1026.1 (872.9;1207.1)	67.1 (56.8;78.6)	1319.9 (1108.2;1573.9)	39 (32.8;46.8)	-41.9(-44.8;-38.8)
Amapá	74.2 (62.7;87.1)	56.4 (47.6;66.1)	199.7 (167.3;235.8)	32 (26.9;38)	-43.2(-46;-40.4)
Amazonas	619.5 (524.9;728)	60.9 (51.9;71.2)	1057.5 (883.3;1245.1)	32 (26.9;38.1)	-47.4(-50.1;-44.8)
Bahia	4959.8 (4246;5818.1)	64.9 (55.4;76.4)	5735.4 (4853.1;6730)	34.9 (29.5;41)	-46.1(-48.7;-43.3)
Brazil	67428.6 (57078.1;79099.3)	66 (56.1;77.9)	74671 (62811.6;88635.8)	31.6 (26.6;37.3)	-52.1(-53.5;-50.5)
Ceará	2343.9 (1995.2;2740.3)	52.8 (44.8;62.2)	3351.2 (2826.3;3942.5)	33.2 (28;39)	-37.1(-40.2;-33.7)
Distrito Federal	558.7 (471;663.8)	65.4 (55.8;77.3)	790.4 (654.4;941.1)	27.6 (23.2;32.8)	-57.8(-60.2;-55.4)
Espírito Santo	1270.6 (1075.6;1501.2)	73.9 (62.8;87.5)	1461.3 (1228.6;1746.4)	33.1 (27.9;39.5)	-55.1(-57.3;-52.7)
Goiás	1822.3 (1538.8;2124.8)	70.5 (60.1;82.8)	2248.2 (1886.2;2685.2)	31.1 (26.2;37)	-55.9(-58.2;-53.5)
Maranhão	1641 (1394.3;1921.1)	55.7 (47.3;65.5)	2557.2 (2151.7;3039.5)	36.3 (30.3;43.1)	-34.9(-38.2;-31.4)
Mato Grosso	646.7 (543.7;760.9)	60.2 (50.9;70.9)	1077.9 (903.8;1284.1)	30.4 (25.5;36.1)	-49.6(-52.1;-47)
Mato Grosso do Sul	777.3 (655.6;912.7)	69.8 (59.4;82.2)	1002 (836;1189.7)	33.3 (28;39.4)	-52.3(-54.8;-49.9)
Minas Gerais	8317.6 (7018;9699.9)	73.1 (62.5;85.2)	8217.1 (6891.1;9768.9)	31.9 (27;37.7)	-56.4(-58.8;-54.2)
Pará	1633.3 (1385.5;1919.1)	62.8 (53.3;73.8)	2491 (2103.9;2941.4)	32.1 (27.1;38.1)	-48.8(-51.2;-46.2)
Paraíba	1357 (1140.3;1598.3)	56.6 (47.7;66.5)	1652.8 (1391.8;1948.6)	35.2 (29.6;41.7)	-37.7(-40.7;-34.5)
Paraná	4074.7 (3437.9;4796.7)	71.4 (60.1;84.1)	4081.4 (3406.8;4870)	31 (26.1;36.7)	-56.6(-58.9;-54.1)
Pernambuco	3388.5 (2863;3953.6)	68.2 (57.9;79.9)	3762.9 (3169.8;4455.3)	36.6 (30.9;43.3)	-46.4(-48.8;-43.8)
Piaui	933.5 (789.8;1101.7)	58.4 (49.1;68.8)	1256.6 (1061.5;1482.9)	33.3 (28.1;39.4)	-42.9(-45.6;-40.3)
Rio de Janeiro	8489.5 (7139.9;9988.4)	79 (67.3;92)	7573.4 (6346.9;8980.5)	34.9 (29.5;41)	-55.8(-58.3;-53.3)
Rio Grande do Norte	963.6 (817.6;1128.9)	56.4 (47.7;65.9)	1188.6 (1009.1;1401.6)	30.3 (25.7;35.8)	-46.2(-48.5;-43.6)
Rio Grande do Sul	4943.9 (4168;5839)	67.4 (57.3;79.4)	4659.2 (3902;5531)	31.7 (26.8;37.4)	-53(-55.5;-50.6)
Rondônia	351.8 (294.1;419.3)	67.5 (57.2;79.2)	508.8 (427;605)	30.4 (25.7;36)	-54.9(-57.1;-52.8)
Roraima	54.6 (45.5;64.3)	59.6 (50.5;69.8)	125.4 (105.4;149.5)	28.4 (23.9;33.8)	-52.4(-54.9;-49.7)
Santa Catarina	2023.7 (1711.8;2390.1)	67.8 (57.7;79.4)	2267.2 (1895.1;2697.9)	27.9 (23.5;32.9)	-58.8(-61;-56.4)
São Paulo	14142.1 (11564.1;17144.7)	60.6 (49.9;73.2)	14566.1 (11933.1;17602.4)	27.1 (22.3;32.5)	-55.2(-57.4;-52.4)
Sergipe	577.7 (491.6;674.3)	63.8 (53.9;74.8)	802.6 (679;952.8)	34.2 (28.9;40.7)	-46.4(-49;-43.5)
Tocantins	311 (262.5;365.8)	61.3 (52.1;71.9)	483.8 (409.1;573)	31.8 (26.9;37.8)	-48(-50.7;-45.6)
**B.2.3.3-Subarachnoid hemorrhage**					(;)
Acre	51.1 (43.4;61.2)	21.4 (18;25.6)	134.1 (112.5;160.6)	17.5 (14.7;21.1)	-18.3(-23.6;-13.3)
Alagoas	373.7 (317.3;440.8)	22.6 (19.1;26.8)	626.2 (523.3;750.7)	17.9 (15;21.4)	-20.9(-26;-15.6)
Amapá	30.2 (25.5;35.7)	20.1 (16.9;24)	116.5 (97.9;137.7)	17 (14.2;20.1)	-15.4(-20.4;-10.1)
Amazonas	232.3 (195.8;277.2)	19.7 (16.6;23.5)	586.9 (497;703.8)	16.5 (14;19.9)	-16.2(-21.1;-11.1)
Bahia	1749 (1483.9;2051.6)	21.5 (18.2;25.7)	2950.6 (2469;3519.1)	17.6 (14.7;20.9)	-18.3(-23.3;-13.3)
Brazil	24819.8 (21095;29388.3)	22.1 (18.6;26.3)	41643 (34891.4;50228.6)	17.2 (14.4;20.6)	-22.2(-25.1;-19.3)
Ceará	968.2 (825.4;1146.9)	20.9 (17.8;25)	1817.9 (1524;2194.1)	17.7 (14.8;21.3)	-15.7(-20.8;-10.8)
Distrito Federal	250.6 (211.3;301.2)	23.5 (19.8;28.1)	531.4 (441.6;644)	16.7 (14;20)	-29(-33.2;-24)
Espírito Santo	445 (377.6;531.3)	23.2 (19.6;27.8)	792.1 (654.9;957.8)	17.4 (14.5;20.9)	-25(-30;-19.8)
Goiás	647.8 (545.5;774.8)	22 (18.5;26.3)	1247.7 (1037.7;1523.1)	16.4 (13.7;19.9)	-25.2(-29.7;-20.6)
Maranhão	692.5 (586.5;829.9)	22.1 (18.6;26.7)	1355.2 (1142.2;1621)	18.8 (15.8;22.7)	-14.9(-20.1;-9.2)
Mato Grosso	266.6 (222.9;317.9)	20.9 (17.5;24.9)	628.9 (526.9;754.9)	16.6 (14;19.9)	-20.6(-25.2;-15.5)
Mato Grosso do Sul	279.9 (237;330.7)	22.1 (18.7;26.4)	539.6 (447.2;649)	17.2 (14.4;20.6)	-22.2(-27.2;-17.6)
Minas Gerais	2880.7 (2426.3;3418)	23.2 (19.5;27.6)	4518.6 (3782.4;5427.8)	17.3 (14.6;20.7)	-25.4(-30.7;-20.3)
Pará	605.2 (511.4;718.8)	20.4 (17;24.7)	1378.1 (1167.4;1628.7)	16.8 (14.2;19.8)	-17.8(-22.6;-12.3)
Paraíba	551.5 (469;654.5)	22.8 (19.3;27.4)	836.3 (703;998.7)	17.8 (15;21.3)	-22.1(-27.1;-17.3)
Paraná	1469.3 (1239.3;1759.9)	22.6 (19.1;27.1)	2318.8 (1934.8;2839.6)	17.1 (14.4;20.8)	-24.4(-29.3;-19.7)
Pernambuco	1077.4 (909.4;1293.2)	20.4 (17.1;24.8)	1869.7 (1556;2258.3)	17.7 (14.8;21.2)	-13.5(-18.6;-7.9)
Piaui	382.7 (322.5;456.4)	22.2 (18.6;26.8)	676.5 (564.7;813.9)	17.8 (14.8;21.4)	-19.8(-24.6;-14.5)
Rio de Janeiro	2752.4 (2313.7;3277.1)	23.5 (19.8;28.2)	3763.9 (3113.5;4544.3)	17.2 (14.3;20.6)	-27(-31.6;-21.8)
Rio Grande do Norte	352.1 (296.8;418.4)	20 (16.7;23.9)	640.4 (533.2;768.3)	16.1 (13.4;19.3)	-19.5(-24.2;-14.9)
Rio Grande do Sul	1676.9 (1410.2;2002.7)	20.9 (17.5;24.9)	2390.9 (1975.7;2914.1)	16.3 (13.7;19.7)	-21.8(-26.6;-16.4)
Rondônia	138 (115.7;165.1)	21 (17.7;25.2)	295.9 (246.3;357)	16.4 (13.8;19.7)	-22(-26.6;-17.1)
Roraima	22.8 (18.9;27.4)	19.7 (16.4;23.8)	76.1 (62.9;90.7)	15.5 (13;18.6)	-21.3(-26;-15.8)
Santa Catarina	753.8 (635.9;895)	22 (18.4;26.3)	1359.5 (1133;1634.6)	16 (13.5;19)	-27.2(-31.8;-22.4)
São Paulo	5841.7 (4872.1;7001.2)	22.2 (18.6;26.8)	9498.5 (7897.7;11571.9)	17.3 (14.5;21)	-22.2(-27.1;-16.8)
Sergipe	203.1 (173;240.8)	20.7 (17.6;24.6)	413 (345.2;497.7)	16.8 (14;20.3)	-18.7(-23.5;-13.7)
Tocantins	125.3 (106.8;148.7)	21.7 (18.4;26)	279.8 (234.6;335.7)	17.6 (14.8;21.1)	-19.1(-23.8;-14.4)

*Source: Data derived from Global Burden of Disease Study 2019, Institute for Health Metrics and Evaluation, University of Washington.^*46*^*

**Table 2-4:** – Number of cases and age-standardized incidence rates (per 100 000) of stroke, ischemic stroke, subarachnoid hemorrhage, and intracerebral hemorrhage in 1990 and 2019, and percent change of rates, in Brazil, according to age group.

Cause of death and age group	1990		2019		Percent change (95% UI)
	Number (95% UI)	Rate (95% UI)	Number (95% UI)	Rate (95% UI)	
**B.2.3-Stroke**					
Under 5	4796.6 (3097.4;7039.4)	28.3 (18.3;41.6)	3972.4 (2556.8;5853)	25.6 (16.5;37.8)	-9.5 (-12.9;-6.5)
15-49 years	63360.3 (54287.5;74063.7)	82.7 (70.8;96.6)	59083.8 (50210.7;69549.5)	51.2 (43.5;60.2)	-38.1 (-40.9;-35.2)
50-69 years	87814.2 (73059.9;104528.3)	559.8 (465.7;666.3)	120686.6 (99991.1;144226.9)	299.1 (247.9;357.5)	-46.6 (-48.1;-44.8)
5-14 years	7322.4 (4825.9;10780.3)	20.7 (13.7;30.5)	6041.7 (3937.9;8992.8)	18.7 (12.2;27.9)	-9.6 (-13;-6.6)
70+ years	53347.1 (43596.4;65336.4)	1261.1 (1030.6;1544.6)	105726 (87241.1;128305.7)	807.8 (666.5;980.3)	-35.9 (-38.7;-32.6)
Age-standardized	216640.6 (193728.5;242758.9)	224.6 (201.6;251.8)	295510.5 (264160.9;331953.6)	127 (113.8;142.1)	-43.5 (-44.7;-42.2)
All Ages	216640.6 (193728.5;242758.9)	145.6 (130.2;163.1)	295510.5 (264160.9;331953.6)	136.4 (121.9;153.2)	-6.3 (-9.4;-3.2)
**B.2.3.1-Ischemic stroke**					
Under 5	4013.6 (2501.9;6255.9)	23.7 (14.8;36.9)	3297 (2025.3;5218)	21.3 (13.1;33.7)	-10.2 (-14.5;-6.8)
15-49 years	24916.5 (18575.7;33199.8)	32.5 (24.2;43.3)	24363.2 (17402.1;33586.9)	21.1 (15.1;29.1)	-35.1 (-40.1;-30.4)
50-69 years	51461.8 (38832.1;65653.7)	328 (247.5;418.5)	68841.4 (51313.9;88179.8)	170.6 (127.2;218.6)	-48 (-49.9;-45.9)
5-14 years	4407.5 (2257.7;7655.6)	12.5 (6.4;21.7)	3662.4 (1838.8;6551.7)	11.4 (5.7;20.3)	-9 (-14.4;-4.6)
70+ years	39592.8 (30181.9;51380.8)	936 (713.5;1214.7)	79032.5 (61618.1;100736.8)	603.8 (470.8;769.7)	-35.5 (-39;-31.2)
Age-standardized	124392.2 (105330.6;147825.6)	136.6 (115.7;163.1)	179196.5 (151357.9;214373.1)	78.2 (66.1;93)	-42.7 (-44.3;-41)
All Ages	124392.2 (105330.6;147825.6)	83.6 (70.8;99.3)	179196.5 (151357.9;214373.1)	82.7 (69.9;98.9)	-1 (-5.4;3.1)
**B.2.3.2-Intracerebral hemorrhage**					
Under 5	639.3 (316.8;1088.4)	3.8 (1.9;6.4)	544.8 (268.7;901)	3.5 (1.7;5.8)	-6.9 (-11.6;-2.1)
15-49 years	25215.1 (19371.2;31655.2)	32.9 (25.3;41.3)	19485.6 (14722.1;24909.6)	16.9 (12.7;21.6)	-48.7 (-51.5;-46.1)
50-69 years	27557.1 (20992.6;36179.2)	175.7 (133.8;230.6)	33135.5 (24835.4;43786.9)	82.1 (61.6;108.5)	-53.2 (-55.3;-51.5)
5-14 years	2338.7 (1254.4;3880.9)	6.6 (3.6;11)	1875.7 (994.1;3114.7)	5.8 (3.1;9.7)	-12.1 (-16.1;-7.6)
70+ years	11678.4 (8940.8;15312.2)	276.1 (211.4;362)	19629.4 (15234.8;25320.2)	150 (116.4;193.5)	-45.7 (-48.6;-42.2)
Age-standardized	67428.6 (57078.1;79099.3)	66 (56.1;77.9)	74671 (62811.6;88635.8)	31.6 (26.6;37.3)	-52.1 (-53.5;-50.5)
All Ages	67428.6 (57078.1;79099.3)	45.3 (38.3;53.1)	74671 (62811.6;88635.8)	34.5 (29;40.9)	-23.9 (-27.7;-20.4)
**B.2.3.3-Subarachnoid hemorrhage**					
Under 5	143.6 (68.7;277.7)	0.8 (0.4;1.6)	130.7 (62.9;249.2)	0.8 (0.4;1.6)	-0.5 (-5.1;4.2)
15-49 years	13228.8 (10504.7;16567.2)	17.3 (13.7;21.6)	15235 (11825;19094.1)	13.2 (10.2;16.5)	-23.6 (-27.8;-19.5)
50-69 years	8795.2 (6604.7;11436)	56.1 (42.1;72.9)	18709.7 (13924.4;24605.4)	46.4 (34.5;61)	-17.3 (-20.8;-13.5)
5-14 years	576.3 (321.8;933.3)	1.6 (0.9;2.6)	503.5 (278.8;821.4)	1.6 (0.9;2.5)	-4.3 (-8.3;-0.7)
70+ years	2075.8 (1530.9;2729.2)	49.1 (36.2;64.5)	7064 (5300.2;9103.7)	54 (40.5;69.6)	10 (3.8;16.6)
Age-standardized	24819.8 (21095;29388.3)	22.1 (18.6;26.3)	41643 (34891.4;50228.6)	17.2 (14.4;20.6)	-22.2 (-25.1;-19.3)
All Ages	24819.8 (21095;29388.3)	16.7 (14.2;19.7)	41643 (34891.4;50228.6)	19.2 (16.1;23.2)	15.3 (8.2;22.1)

*Source: Data derived from Global Burden of Disease Study 2019, Institute for Health Metrics and Evaluation, University of Washington.^*46*^*

**Table 2-5:** – Number of deaths and age-standardized mortality rates (per 100 000) due to stroke, ischemic stroke, subarachnoid hemorrhage, and intracerebral hemorrhage in 1990 and 2019, and percent change of rates, in Brazil and its Federative Units.

Cause of death and location	1990		2019		Percent change (95% UI)
	Number (95% UI)	Rate (95% UI)	Number (95% UI)	Rate (95% UI)	
**B.2.3-Stroke**					
Acre	143.8 (131.9;155.4)	116.6 (105.3;126.7)	346.6 (310.2;383.2)	65.4 (58;72.4)	-43.9(-50.1;-36.7)
Alagoas	1893.6 (1719.9;2096.1)	159 (142.9;175.9)	2592 (2250.7;2940.1)	85.2 (73.4;96.7)	-46.4(-54.8;-37.4)
Amapá	77.5 (70.7;83.5)	98.6 (88.6;106.2)	271.3 (240.2;300.2)	60.1 (52.3;66.6)	-39.1(-45.1;-32.7)
Amazonas	751.6 (686.8;811.9)	122.6 (111.9;132.7)	1496.8 (1303.8;1681.6)	57.8 (49.9;65.1)	-52.9(-58.2;-46.9)
Bahia	7365.2 (6492.7;8251.6)	118.4 (103.9;132.6)	10378.7 (8704.1;12165.3)	64.5 (54.1;75.7)	-45.5(-55;-34.1)
Brazil	105603.9 (100300.3;109634.9)	137.8 (127.8;144)	131007 (119134.6;139017.7)	58.1 (52.6;61.8)	-57.8(-60.4;-55.5)
Ceará	3804.6 (3224.5;4401.7)	97.6 (82.5;113.2)	6627.7 (5527.6;7761)	67.8 (56.7;79.3)	-30.5(-44.4;-13.9)
Distrito Federal	605.3 (547.2;681.5)	161.8 (148;178.1)	1134.4 (1002.1;1274.2)	64.2 (56.4;71.8)	-60.4(-65.2;-55)
Espírito Santo	2030 (1918.2;2129.2)	169.6 (156.7;178.3)	2591.1 (2257.7;2915.3)	63.8 (55.4;71.9)	-62.4(-66.7;-58.1)
Goiás	2732.6 (2384.6;3201.9)	160 (140.7;186.4)	3477 (2933.1;4024.5)	54.5 (45.9;63.2)	-65.9(-71.8;-59.1)
Maranhão	2727.7 (2300.3;3185.3)	118.4 (99.1;137.9)	5826.6 (4963;6787.8)	91.6 (78.1;106.9)	-22.7(-37.2;-3.1)
Mato Grosso	731.8 (649;812.6)	116.9 (103.3;128.8)	1521.5 (1344;1716.5)	51.5 (45;58.1)	-56(-61.6;-49.2)
Mato Grosso do Sul	1019.2 (949.8;1089.1)	137.6 (125.9;147.5)	1497.5 (1313.8;1684.4)	55.1 (48.2;61.9)	-60(-64.3;-55.1)
Minas Gerais	12354.4 (11491.7;13383.7)	146.6 (135.3;158.3)	13090.3 (11454.5;14614.3)	50.1 (43.9;55.9)	-65.9(-69.9;-61.9)
Pará	2536.9 (2266.4;2816.3)	151.5 (134;167.8)	4274.4 (3680.9;4778)	66.2 (56.9;74.1)	-56.3(-61.8;-49.9)
Paraíba	2238.8 (1946.1;2517.7)	101.8 (88.2;114.8)	2887.6 (2473.2;3298)	58.8 (50.6;67.1)	-42.2(-51.3;-31.1)
Paraná	6868.8 (6515;7188.2)	177.5 (165.7;186.5)	7742.1 (6777;8670.9)	63.5 (55.5;71)	-64.2(-68.2;-60.3)
Pernambuco	5685.9 (5287.8;6033)	143.6 (131.7;153.4)	6749.5 (5954.7;7559.6)	70.9 (62.3;79.5)	-50.6(-56.2;-44.6)
Piaui	1678.5 (1513.5;1851.4)	144.9 (128.4;160.8)	2546.2 (2165.1;2865.1)	66.3 (56.9;74.5)	-54.3(-60.1;-47.9)
Rio de Janeiro	14063.6 (13347.2;14643.9)	168.9 (158.4;176.7)	12722.1 (11239.8;14074.9)	58.4 (51.7;64.6)	-65.5(-68.8;-62)
Rio Grande do Norte	1363.5 (1194;1525.5)	89.9 (78.1;100.7)	1750.4 (1451.8;2056.2)	44.2 (36.8;52)	-50.8(-59.2;-40.6)
Rio Grande do Sul	7690 (7244.8;8075.8)	141 (130.9;148.5)	9167.2 (8020.6;10250.4)	60.1 (52.4;67.3)	-57.4(-61.5;-52.8)
Rondônia	395.6 (350;438)	195.7 (179.1;211.8)	825.4 (709.6;948.7)	60.9 (52.2;70.2)	-68.9(-73.4;-63.5)
Roraima	53.7 (48.3;59.2)	145.2 (131.6;158.1)	170.8 (151.3;189.2)	61.1 (53.4;67.5)	-57.9(-62.6;-52.6)
Santa Catarina	3221.9 (3005.7;3411.7)	161.2 (148.7;171.9)	3786.8 (3322.4;4250.2)	52.6 (45.9;58.9)	-67.4(-71;-63.8)
São Paulo	22207.4 (20875;23529)	133.5 (123.7;141.7)	25162.9 (22011.3;27840.6)	49.3 (42.9;54.6)	-63.1(-66.8;-59.2)
Sergipe	955.9 (866.9;1041.7)	148 (133.8;161.5)	1423.6 (1207;1642.1)	66.6 (56.5;76.8)	-55(-61.9;-47.7)
Tocantins	406.1 (354.8;458.2)	146.8 (128.7;166)	946.9 (817.8;1082.4)	71.5 (61.4;81.5)	-51.3(-58.8;-42.2)
**B.2.3.1-Ischemic stroke**					(;)
Acre	70.1 (62.3;77.5)	72.3 (63.5;80.1)	181.6 (158.2;205.7)	38.9 (33.5;44.1)	-46.2(-53.4;-37.2)
Alagoas	1044.8 (903.6;1192.7)	98.3 (84.9;111.7)	1480.4 (1261.6;1693.5)	51 (43.4;58.4)	-48.1(-57.5;-37.6)
Amapá	39.7 (35.4;43.1)	61.6 (53.7;67.3)	139.6 (119.5;156.1)	35.8 (30.3;40.3)	-41.9(-47.8;-35.4)
Amazonas	358.8 (324.9;390.4)	72.2 (64.7;78.8)	791 (664;897.8)	33.6 (28.2;38.2)	-53.4(-59.1;-47.4)
Bahia	3842.8 (3286.8;4392.2)	67.9 (57.8;77.4)	5850.1 (4764.5;6852.8)	36.6 (29.9;43)	-46(-57;-33.6)
Brazil	52583.6 (48875.7;55110.8)	80 (72.5;84.3)	73920.9 (64818.8;79592.8)	33.9 (29.7;36.6)	-57.6(-60.9;-54.4)
Ceará	2118.4 (1732.9;2534.7)	57.3 (46.8;68.4)	3934.3 (3210.7;4689.3)	40.8 (33.4;48.6)	-28.7(-44;-7.8)
Distrito Federal	220.4 (198.2;248.8)	97 (87.6;107.8)	605.3 (526.7;685.6)	42 (36.3;47.5)	-56.7(-62.1;-50.5)
Espírito Santo	1014.7 (936;1080.7)	101.5 (92.4;108)	1430.6 (1220.2;1628.7)	37.2 (31.6;42.5)	-63.3(-67.8;-58.7)
Goiás	1188.5 (1024.2;1385.5)	89.1 (77.8;102.5)	1817 (1514.1;2122.7)	30.9 (25.6;36.3)	-65.3(-71.2;-58.6)
Maranhão	1407.4 (1117.9;1698.7)	71 (56.5;84.4)	3417.1 (2880.3;3967.2)	55.3 (46.7;64.2)	-22.1(-39.3;1)
Mato Grosso	343.7 (302.2;384.8)	70.5 (61.1;78.3)	797.9 (683.6;907.2)	30 (25.4;34.2)	-57.4(-63.1;-51.2)
Mato Grosso do Sul	444.6 (403;481.7)	76 (68.2;82.5)	792.9 (683.4;899.6)	31.2 (26.7;35.4)	-59(-63.8;-53.6)
Minas Gerais	5799 (5307.3;6297.5)	82.7 (74.8;89.5)	7229.9 (6179.5;8168.3)	27.9 (23.8;31.6)	-66.2(-70.2;-62.1)
Pará	1345.9 (1183.7;1495.4)	94.5 (81.9;104.9)	2458.5 (2074.9;2785.8)	40.6 (34.3;46)	-57(-62.7;-50.5)
Paraíba	1316.8 (1091.7;1523)	61.9 (51.1;71.6)	1672.8 (1379.4;1924.3)	33.2 (27.7;38.1)	-46.4(-56.5;-33.8)
Paraná	3462.5 (3204.1;3679.6)	108.1 (98.5;115.3)	4689.7 (4062.5;5269.4)	40.4 (34.7;45.3)	-62.7(-67.1;-58.4)
Pernambuco	2975.1 (2658.4;3245.8)	83.7 (74.2;90.9)	3626.1 (3104.5;4110.4)	39.9 (34.1;45.3)	-52.3(-58.9;-45)
Piaui	901 (780.3;1014.2)	89.9 (77.2;101.3)	1562.6 (1267.6;1778.7)	40.3 (33;45.8)	-55.2(-62;-47.8)
Rio de Janeiro	6587.9 (6142.6;6974.8)	94 (86.6;99.7)	6704.8 (5839;7432.1)	31.5 (27.3;34.9)	-66.5(-70;-63)
Rio Grande do Norte	782.5 (666.9;896.7)	53.8 (45.2;61.6)	1026.1 (825.4;1217.3)	25.7 (20.8;30.4)	-52.1(-60.8;-40.6)
Rio Grande do Sul	4123.2 (3793.8;4390.4)	87.1 (78.9;92.8)	5640.7 (4783.8;6329.9)	37.3 (31.6;41.9)	-57.2(-61.6;-52.3)
Rondônia	159.7 (142.5;177.4)	126.6 (115;137.7)	447.2 (378.1;519.2)	36.6 (30.8;42.4)	-71.1(-75.6;-66.1)
Roraima	21.9 (19.8;23.9)	91.3 (81.4;99.8)	87.3 (76;97.2)	38.5 (32.9;43)	-57.9(-63.3;-52.5)
Santa Catarina	1659.3 (1532.4;1780.8)	99 (90.2;106.5)	2258.1 (1953.1;2547.1)	33.5 (28.7;37.8)	-66.2(-70.2;-62)
São Paulo	10643.9 (9783.4;11370)	77.8 (70.4;83.3)	13918.4 (11936.9;15470.3)	28.5 (24.3;31.7)	-63.4(-67;-59.5)
Sergipe	523 (466.3;577.6)	92.2 (81.9;101.6)	819.2 (679.8;954)	40 (33.2;46.6)	-56.7(-63.7;-48.4)
Tocantins	188.1 (162;215.5)	91.9 (79.3;104.4)	541.5 (457.2;626.5)	43.2 (36.3;50)	-53(-61.6;-43.3)
**B.2.3.2-Intracerebral hemorrhage**					(;)
Acre	58.9 (53.1;65.3)	37.4 (33.5;41.3)	125.5 (112.1;140.6)	20.8 (18.6;23.3)	-44.4(-52;-35.1)
Alagoas	702.4 (602.1;808.7)	51.7 (44.2;59.7)	886.7 (770.1;1011.6)	27.6 (23.9;31.6)	-46.6(-58.3;-33.5)
Amapá	31.1 (28.2;33.9)	32.1 (29;34.8)	101.4 (89.3;113.2)	19.4 (17;21.6)	-39.5(-46.4;-31.5)
Amazonas	334 (302.8;367.5)	44.6 (40.3;49)	554.6 (485.6;628.3)	19.5 (17;22.2)	-56.2(-61.9;-49.4)
Bahia	2951.9 (2566.8;3389.6)	43.3 (37.5;49.7)	3553.6 (2961.9;4213.8)	22 (18.3;26.1)	-49.2(-59.8;-35.7)
Brazil	44537.3 (42391.2;46721)	49.8 (47.1;52.2)	43825.9 (40717.3;46438.1)	18.6 (17.3;19.8)	-62.6(-65.3;-59.7)
Ceará	1391 (1155.5;1654.1)	33.9 (28;40.5)	2120.3 (1745.1;2510.6)	21.4 (17.6;25.3)	-37(-52;-18.3)
Distrito Federal	305.4 (272.1;350.2)	55.6 (50;62.3)	382.9 (334.5;434.5)	16.6 (14.4;18.8)	-70.2(-74.4;-65.2)
Espírito Santo	856.4 (808.2;905.6)	59.1 (55.5;62.8)	896.6 (777.8;1015.2)	20.7 (17.9;23.5)	-65(-69.7;-60)
Goiás	1296.7 (1119.5;1552)	61.8 (53.7;73.2)	1269 (1068.7;1485.1)	18.3 (15.5;21.3)	-70.4(-76;-63.8)
Maranhão	1056.1 (839.2;1317.7)	39.2 (30.8;49.2)	1867.8 (1566.3;2245.4)	28.5 (23.8;34.3)	-27.3(-45.5;0)
Mato Grosso	314.1 (273.8;354.4)	39.8 (34.9;44.6)	543.3 (478;616.5)	16.5 (14.5;18.7)	-58.5(-65.1;-50.7)
Mato Grosso do Sul	480.1 (444.8;519.9)	53.4 (49.4;57.7)	540.7 (473.4;616.2)	18.5 (16.2;21.2)	-65.3(-69.7;-60.1)
Minas Gerais	5492.7 (5064.6;6110.2)	55 (50.7;60.6)	4397.3 (3880.7;4919.8)	16.6 (14.7;18.5)	-69.9(-73.9;-65.3)
Pará	1022.3 (907.3;1149)	50.7 (44.9;56.9)	1421.5 (1232.5;1598.4)	20.4 (17.7;23.1)	-59.7(-65.7;-52.5)
Paraíba	736.5 (612.7;889.3)	32 (26.6;38.7)	954.1 (827.2;1097.1)	20.1 (17.4;23.1)	-37.3(-51.4;-20.3)
Paraná	2891.5 (2721.3;3082.7)	60.7 (56.7;64.6)	2314.2 (2019.3;2620.5)	17.6 (15.3;19.9)	-71(-75;-67.1)
Pernambuco	2377.5 (2199.5;2588.7)	53.3 (49.1;58.1)	2491.7 (2197.6;2813.9)	24.9 (22;28.1)	-53.4(-59.8;-46)
Piaui	643.2 (567.5;736.9)	46.8 (41;54)	775.3 (672.3;890.1)	20.5 (17.8;23.6)	-56.1(-63.9;-47.4)
Rio de Janeiro	6351.1 (6010;6736.8)	64.9 (61.3;68.9)	4705.5 (4169.5;5275.8)	20.9 (18.5;23.5)	-67.8(-71.5;-63.4)
Rio Grande do Norte	493.1 (431.4;561.6)	31.1 (27.1;35.4)	570.8 (468.7;677.4)	14.6 (12.1;17.4)	-52.9(-62.4;-41.8)
Rio Grande do Sul	3066.7 (2873.9;3255.1)	47.3 (44.1;50.3)	2801.4 (2461.1;3171.1)	18 (15.8;20.3)	-62(-66.5;-56.9)
Rondônia	196.6 (170.7;221.3)	61.5 (54.9;67.6)	288.5 (246.5;339.4)	19 (16.3;22.4)	-69(-74.1;-62.4)
Roraima	26 (22.9;28.9)	47.3 (43;52)	63.2 (55.7;70.9)	17.8 (15.8;20)	-62.3(-67.5;-56.6)
Santa Catarina	1330.5 (1234.2;1427.7)	54.7 (50.3;58.7)	1160.9 (1008.4;1314.2)	14.6 (12.7;16.5)	-73.2(-76.6;-69.5)
São Paulo	9579.9 (8924.2;10296.7)	47.8 (44.3;51.4)	8239 (7219.3;9239.4)	15.3 (13.4;17.1)	-67.9(-72;-63.7)
Sergipe	372 (329.2;416.2)	49.1 (43.3;55)	483.9 (411.5;563.2)	21.6 (18.4;25.2)	-55.9(-63.9;-46.9)
Tocantins	179.6 (150.7;210.6)	47.4 (39.5;55.7)	316.3 (271.6;365.5)	22.4 (19.3;25.9)	-52.6(-62.9;-40.4)
**B.2.3.3-Subarachnoid hemorrhage**					(;)
Acre	14.9 (13;16.9)	6.9 (6.1;7.8)	39.5 (34.9;44.8)	5.7 (5;6.4)	-18(-30.8;-0.2)
Alagoas	146.4 (123.5;172)	9 (7.6;10.7)	224.8 (191.5;261.7)	6.6 (5.6;7.7)	-27.2(-43.3;-6.1)
Amapá	6.6 (5.8;7.7)	4.9 (4.4;5.8)	30.4 (26.9;35)	4.9 (4.3;5.6)	-1.6(-15.3;14)
Amazonas	58.9 (52;67.4)	5.8 (5.2;6.8)	151.3 (131.4;174.4)	4.6 (4;5.4)	-20.5(-34.1;-4.5)
Bahia	570.5 (494;662.3)	7.2 (6.2;8.5)	975 (798.2;1173.9)	5.9 (4.8;7.1)	-18.8(-36.4;3.4)
Brazil	8483 (7668.1;8870.6)	8 (7.3;8.4)	13260.2 (12016.4;14155.9)	5.5 (5;5.9)	-30.3(-36.2;-23.6)
Ceará	295.3 (243;351.7)	6.5 (5.3;7.7)	573 (473.5;700.2)	5.6 (4.7;6.9)	-12.7(-32.5;18.3)
Distrito Federal	79.5 (70;89.6)	9.2 (8.1;10.4)	146.1 (122.3;168.3)	5.5 (4.4;6.4)	-40.1(-51;-28.6)
Espírito Santo	158.9 (131.5;173)	8.9 (7.5;9.7)	263.9 (222.5;310.3)	5.9 (5;6.9)	-33.7(-43.7;-21)
Goiás	247.4 (214.8;287.7)	9.2 (8;10.7)	391 (327.2;464.2)	5.3 (4.5;6.3)	-41.8(-53.2;-28.4)
Maranhão	264.1 (196.8;343.7)	8.2 (6;10.7)	541.6 (438.5;658)	7.8 (6.3;9.5)	-4.7(-31.9;33.9)
Mato Grosso	74.1 (62.4;86.7)	6.6 (5.7;7.7)	180.3 (156.6;208.1)	5 (4.3;5.8)	-24.7(-38.2;-8.6)
Mato Grosso do Sul	94.5 (85.3;103.3)	8.2 (7.5;9)	163.9 (141.6;187.4)	5.4 (4.6;6.1)	-34.8(-44.5;-24)
Minas Gerais	1062.8 (930.5;1162.6)	8.9 (7.9;9.7)	1463.2 (1232.6;1677.6)	5.6 (4.7;6.4)	-37.7(-47.1;-27.8)
Pará	168.7 (146.9;194.8)	6.3 (5.5;7.3)	394.4 (341.7;451.6)	5.1 (4.4;5.8)	-19.2(-33;-2.1)
Paraíba	185.5 (158.3;217.9)	7.8 (6.6;9.1)	260.6 (220.5;302.1)	5.5 (4.6;6.4)	-29.3(-45.3;-10.4)
Paraná	514.8 (457.6;557.9)	8.7 (7.8;9.4)	738.2 (620.2;856.4)	5.5 (4.6;6.4)	-36.7(-46.4;-26.8)
Pernambuco	333.2 (297.6;385.1)	6.6 (5.9;7.8)	631.7 (546.6;728.1)	6.1 (5.3;7)	-7.2(-24.3;11.1)
Piaui	134.3 (116.3;153)	8.1 (7;9.3)	208.3 (177.5;242.9)	5.5 (4.7;6.4)	-32.8(-46.4;-16.8)
Rio de Janeiro	1124.6 (903.7;1234.4)	10 (8.2;10.9)	1311.7 (1110.4;1494.2)	5.9 (5;6.8)	-40.4(-48.8;-28.9)
Rio Grande do Norte	87.8 (74.9;111.2)	5.1 (4.3;6.6)	153.4 (124.8;196.3)	3.9 (3.1;4.9)	-24.4(-40.4;-4.1)
Rio Grande do Sul	500 (459;551.6)	6.6 (6.1;7.3)	725.1 (623.3;839.4)	4.8 (4.1;5.5)	-27.5(-38.3;-15.9)
Rondônia	39.2 (32;45.6)	7.7 (6.6;8.7)	89.6 (76.9;105.3)	5.3 (4.5;6.1)	-31.4(-43.5;-15.5)
Roraima	5.8 (4.9;6.7)	6.6 (5.8;7.5)	20.3 (17.7;24.1)	4.8 (4.2;5.6)	-27(-37.8;-13.1)
Santa Catarina	232.2 (211.8;254)	7.6 (6.9;8.3)	367.8 (315.9;426)	4.5 (3.8;5.1)	-40.9(-49.2;-30.5)
São Paulo	1983.7 (1772.4;2164.2)	8 (7.3;8.7)	3005.4 (2542.4;3434.8)	5.5 (4.7;6.3)	-30.7(-40.8;-20.3)
Sergipe	60.9 (52.9;70.3)	6.7 (5.8;7.8)	120.5 (101.5;144.9)	5 (4.3;6.1)	-24.6(-40.5;-5.1)
Tocantins	38.4 (32;44.7)	7.5 (6.3;8.8)	89.1 (75.5;104.6)	5.8 (5;6.9)	-22.1(-37.2;-1.3)

*Source: Data derived from Global Burden of Disease Study 2019, Institute for Health Metrics and Evaluation, University of Washington.^*46*^*

**Table 2-6:** – Number of deaths and age-standardized mortality rates (per 100 000) due to stroke, ischemic stroke, subarachnoid hemorrhage, and intracerebral hemorrhage in 1990 and 2019, and percent change of rates, in Brazil, according to age group.

Cause of death and age group	1990		2019		Percent change (95% UI)
	Number (95% UI)	Rate (95% UI)	Number (95% UI)	Rate (95% UI)	
**B.2.3-Stroke**					
Under 5	416.1 (303;567.7)	2.5 (1.8;3.4)	68.2 (49.6;93.1)	0.4 (0.3;0.6)	-82.1 (-89.3;-70.1)
15-49 years	14553.1 (14010.9;15182.8)	19 (18.3;19.8)	9310.8 (8821.4;9798.8)	8.1 (7.6;8.5)	-57.5 (-60.4;-54.7)
50-69 years	37192.4 (35828.1;38681)	237.1 (228.4;246.6)	37277.4 (35281.9;39071.1)	92.4 (87.5;96.8)	-61 (-63.6;-58.6)
5-14 years	245.7 (219.2;270.1)	0.7 (0.6;0.8)	119.2 (100.6;138.7)	0.4 (0.3;0.4)	-46.8 (-55.2;-37.2)
70+ years	53196.6 (48776.9;55833.8)	1257.6 (1153.1;1319.9)	84231.4 (73239.5;90754.4)	643.6 (559.6;693.4)	-48.8 (-53;-45.4)
Age-standardized	105603.9 (100300.3;109634.9)	137.8 (127.8;144)	131007 (119134.6;139017.7)	58.1 (52.6;61.8)	-57.8 (-60.4;-55.5)
All Ages	105603.9 (100300.3;109634.9)	71 (67.4;73.7)	131007 (119134.6;139017.7)	60.5 (55;64.2)	-14.8 (-21;-9.8)
**B.2.3.1-Ischemic stroke**					
Under 5	47.4 (34.3;62.7)	0.3 (0.2;0.4)	4.4 (3.3;5.9)	0 (0;0)	-89.8 (-93.7;-83.7)
15-49 years	1810.7 (1667.6;1970.2)	2.4 (2.2;2.6)	925.6 (846.9;1018.1)	0.8 (0.7;0.9)	-66.1 (-70.5;-60.5)
50-69 years	12625.4 (11913.9;13321.4)	80.5 (75.9;84.9)	11086.3 (10302.3;11863.9)	27.5 (25.5;29.4)	-65.9 (-69.1;-62.2)
5-14 years	20.5 (17.4;23.6)	0.1 (0;0.1)	5.6 (4.7;6.6)	0 (0;0)	-70 (-75.7;-62.8)
70+ years	38079.6 (34747.2;40122.2)	900.2 (821.4;948.5)	61898.9 (53255.8;67163.8)	472.9 (406.9;513.2)	-47.5 (-52.1;-43.6)
Age-standardized	52583.6 (48875.7;55110.8)	80 (72.5;84.3)	73920.9 (64818.8;79592.8)	33.9 (29.7;36.6)	-57.6 (-60.9;-54.4)
All Ages	52583.6 (48875.7;55110.8)	35.3 (32.8;37)	73920.9 (64818.8;79592.8)	34.1 (29.9;36.7)	-3.4 (-12.1;4.4)
**B.2.3.2-Intracerebral hemorrhage**					
Under 5	196.8 (133.4;285.8)	1.2 (0.8;1.7)	19.7 (13.7;27.7)	0.1 (0.1;0.2)	-89.1 (-93.8;-80.6)
15-49 years	8947.6 (8497.2;9696.9)	11.7 (11.1;12.7)	4999.6 (4652.9;5309.3)	4.3 (4;4.6)	-62.9 (-67.4;-59.5)
50-69 years	21158.6 (20191.1;22218.4)	134.9 (128.7;141.6)	20099.5 (19034.2;21157.5)	49.8 (47.2;52.4)	-63.1 (-65.7;-60.4)
5-14 years	99.6 (87.6;112.2)	0.3 (0.2;0.3)	35.7 (29.7;42.1)	0.1 (0.1;0.1)	-60.7 (-67.9;-52.1)
70+ years	14134.8 (13036.6;15040.8)	334.1 (308.2;355.6)	18671.4 (16453.6;20277.9)	142.7 (125.7;154.9)	-57.3 (-61.8;-53.1)
Age-standardized	44537.3 (42391.2;46721)	49.8 (47.1;52.2)	43825.9 (40717.3;46438.1)	18.6 (17.3;19.8)	-62.6 (-65.3;-59.7)
All Ages	44537.3 (42391.2;46721)	29.9 (28.5;31.4)	43825.9 (40717.3;46438.1)	20.2 (18.8;21.4)	-32.4 (-37.5;-27.2)
**B.2.3.3-Subarachnoid hemorrhage**					
Under 5	171.9 (106.6;232.7)	1 (0.6;1.4)	44.1 (32;60.8)	0.3 (0.2;0.4)	-72 (-83.2;-45.1)
15-49 years	3794.8 (3298;3999.6)	5 (4.3;5.2)	3385.5 (3176.9;3702)	2.9 (2.8;3.2)	-40.8 (-46;-27.3)
50-69 years	3408.5 (3110.6;3603.8)	21.7 (19.8;23)	6091.5 (5557.2;6562.1)	15.1 (13.8;16.3)	-30.5 (-36.6;-22.3)
5-14 years	125.6 (110.2;138.6)	0.4 (0.3;0.4)	78 (65.7;91.2)	0.2 (0.2;0.3)	-32 (-43.4;-18)
70+ years	982.3 (881.6;1178.5)	23.2 (20.8;27.9)	3661.1 (2977.6;4083.9)	28 (22.7;31.2)	20.5 (-16.5;40.4)
Age-standardized	8483 (7668.1;8870.6)	8 (7.3;8.4)	13260.2 (12016.4;14155.9)	5.5 (5;5.9)	-30.3 (-36.2;-23.6)
All Ages	8483 (7668.1;8870.6)	5.7 (5.2;6)	13260.2 (12016.4;14155.9)	6.1 (5.5;6.5)	7.4 (-1.4;18.8)

*Source: Data derived from Global Burden of Disease Study 2019, Institute for Health Metrics and Evaluation, University of Washington.* ^46^

**Table 2-7:** – Number of YLLs and age-standardized YLL rates (per 100 000) due to stroke, ischemic stroke, subarachnoid hemorrhage, and intracerebral hemorrhage in 1990 and 2019, and percent change of rates, in Brazil and its Federative Units.

Cause of death and location	1990		2019		Percent change (95% UI)
	Number (95% UI)	Rate (95% UI)	Number (95% UI)	Rate (95% UI)	
**B.2.3-Stroke**					
Acre	3638.2 (3310.9;3958.3)	2093.3 (1918.9;2257.8)	7523.5 (6798.3;8291.5)	1189.9 (1074.3;1316.2)	-43.2(-49.5;-35.9)
Alagoas	45113 (41108.8;49962.3)	3155.6 (2866.2;3497.6)	54270.7 (47737.5;61452.7)	1686.1 (1482.2;1904)	-46.6(-55.1;-37.1)
Amapá	1871.7 (1693.8;2033.1)	1747.8 (1601.3;1882.8)	6259.1 (5602.4;6893.9)	1145.2 (1018.4;1264.8)	-34.5(-41.4;-27.1)
Amazonas	18486.3 (16733.1;20143.4)	2261.8 (2062.1;2447.6)	31259.9 (27577.8;34979.3)	1063.1 (935.3;1192.8)	-53(-58.6;-46.5)
Bahia	173368.1 (154540.2;193719)	2433.5 (2163.9;2731.4)	207268.2 (174444.1;242726.8)	1281 (1077.7;1498.2)	-47.4(-56.9;-36)
Brazil	2590844.4 (2504589.7;2679788.7)	2778.6 (2659.5;2879.2)	2578166 (2413609.9;2702585.5)	1098.7 (1025.8;1153.7)	-60.5(-62.7;-58.4)
Ceará	82644.5 (70969;95343.2)	1924.7 (1649.6;2235.2)	122875.8 (102778.9;144353.5)	1232.7 (1030.7;1448.8)	-36(-49.2;-19.8)
Distrito Federal	18102.9 (16265.1;20466.9)	2863 (2605.7;3189.8)	23254.6 (20647.9;26147.6)	970.7 (860;1088.3)	-66.1(-70.6;-61.2)
Espírito Santo	49253.6 (47109.2;51447.6)	3243 (3085.3;3392.1)	51706.5 (45154.4;58160.6)	1192.4 (1042.7;1341.7)	-63.2(-67.7;-58.7)
Goiás	73040.8 (63335.6;85932.2)	3229.4 (2820.1;3792)	72966.1 (61930.4;85238.7)	1039.6 (884.3;1207.4)	-67.8(-73.8;-60.8)
Maranhão	72614.6 (60933.2;85134.1)	2510.2 (2113.8;2938.1)	113620.3 (96360.6;135605.8)	1703.6 (1445.6;2028.8)	-32.1(-46.4;-12.5)
Mato Grosso	19638.7 (16988.9;22162.1)	2261.8 (2005.3;2508.2)	32952.9 (29423.3;36884.6)	983 (874.9;1104)	-56.5(-62.7;-49.4)
Mato Grosso do Sul	26812.2 (24991.9;28638.5)	2766 (2577.2;2952.7)	30540.2 (27071.5;34205.2)	1036.9 (917.8;1162.7)	-62.5(-66.8;-57.5)
Minas Gerais	319806.1 (297709.9;348085.7)	3061.2 (2848.1;3318.7)	258108.8 (230977.9;288521.3)	982.5 (879.9;1097.6)	-67.9(-72.1;-63.8)
Pará	59420.4 (52481.8;66484.2)	2749 (2447.2;3053.1)	88399.9 (77815.3;98126.2)	1248 (1088.7;1385.5)	-54.6(-60.7;-47.6)
Paraíba	47314.8 (41644.2;53264.8)	2018.2 (1773.1;2272.7)	53969.6 (46911;61530.8)	1141.1 (993.1;1300.9)	-43.5(-52.6;-32.1)
Paraná	165936.3 (158219.6;173304.8)	3360.2 (3187.9;3518.8)	146319.8 (128976.3;164812.7)	1119.8 (985.9;1255.3)	-66.7(-70.7;-62.6)
Pernambuco	128500.4 (121005.2;135907.7)	2776.5 (2609.3;2936)	137705 (122806;154533.5)	1369.2 (1220.7;1532.8)	-50.7(-56.6;-44.7)
Piaui	38717 (35046;42639.9)	2686.6 (2441.7;2953.8)	46373.2 (40842.1;52052.7)	1229.9 (1082.6;1381.8)	-54.2(-60.2;-47.1)
Rio de Janeiro	356654 (340788.2;372187.5)	3545 (3366.4;3689.2)	257751.3 (231425.8;285548.3)	1160.2 (1041;1285)	-67.3(-70.8;-63.3)
Rio Grande do Norte	28185.2 (25144.4;31483.9)	1719.5 (1525.9;1925.1)	32774.7 (27520.4;38814.4)	842.2 (707.4;996.9)	-51(-59.9;-40.6)
Rio Grande do Sul	177413.9 (168848.5;185603.9)	2676.3 (2534;2802.4)	162756.9 (144795.8;181241.8)	1062.5 (943.6;1183.1)	-60.3(-64.5;-55.8)
Rondônia	11663.2 (9984.6;13075.5)	3402.2 (3073;3718.5)	17514.3 (15097.2;20285.1)	1130.3 (975.8;1308.2)	-66.8(-72;-60.6)
Roraima	1557.3 (1365.4;1731.4)	2508.3 (2278.1;2745.1)	3888.6 (3473.1;4323.7)	1039.6 (924.2;1150.6)	-58.6(-63.8;-53)
Santa Catarina	74479.5 (69924.8;78924.5)	2941.9 (2754.1;3115.6)	70469.1 (62180.6;79237)	891.5 (787.2;997.1)	-69.7(-73.2;-66)
São Paulo	565019.3 (533070.5;599374.7)	2670.9 (2511.6;2833.3)	500606.4 (446874;554035.6)	931.3 (830.3;1030.4)	-65.1(-69;-61.1)
Sergipe	21056.2 (19196.7;23080.6)	2649.1 (2408.6;2894.5)	28150.9 (24094.5;32370.1)	1249.3 (1068.6;1436.4)	-52.8(-60.5;-44.7)
Tocantins	10536.2 (9172.6;11978)	2567.4 (2248;2886.8)	18879.7 (16302.6;21715.4)	1312.8 (1135;1504.7)	-48.9(-57.5;-38.5)
**B.2.3.1-Ischemic stroke**					(;)
Acre	1241.7 (1095.2;1383.9)	981.6 (873.1;1086.6)	2796.4 (2477.3;3173.2)	532 (469.4;602.9)	-45.8(-53.4;-35.6)
Alagoas	18153.8 (15408.7;20916.9)	1484.1 (1266.6;1704.1)	23104.8 (19903.4;26376.3)	775.5 (666.8;885.6)	-47.7(-58.6;-35.5)
Amapá	666.5 (605.1;727.3)	829.8 (747.4;902.6)	2305.2 (2010.4;2562.1)	522.6 (452.5;581.9)	-37(-43.8;-29.6)
Amazonas	6476 (5852.2;7075.5)	1033.9 (933.3;1123.7)	11701.7 (10136.3;13174.3)	468.4 (404.9;529)	-54.7(-60.2;-48.5)
Bahia	65136.9 (55706.7;74484.5)	1040.2 (891.1;1186.3)	85232 (70691;100091.3)	543.1 (450.7;638.6)	-47.8(-59;-33.8)
Brazil	947035.1 (895299.9;992583.8)	1211.1 (1133.8;1268.9)	1079632.1 (982065;1148602.5)	479.7 (435.1;510.8)	-60.4(-63.5;-57.3)
Ceará	32503 (26515.3;39027.4)	836.2 (682;1004.7)	53233.6 (43945.9;63548.2)	548.9 (452.4;654.6)	-34.4(-49.3;-12.5)
Distrito Federal	4730.7 (4219.7;5398.6)	1294 (1172.8;1442.7)	8959.4 (7866.4;10165)	484.9 (422.2;546.7)	-62.5(-67.4;-57)
Espírito Santo	17951 (16791.8;19054.5)	1443.5 (1335.2;1530.5)	21006.8 (18047.5;23863.7)	517.2 (444.6;586.4)	-64.2(-69;-59.3)
Goiás	23231.1 (19865.8;27417.6)	1336.2 (1158.6;1557.9)	27989.3 (23581.2;32754.8)	440.5 (370.5;514.7)	-67(-73.1;-59.9)
Maranhão	25999 (19603.3;32038.4)	1095.2 (848.6;1331.4)	48031.8 (40676.2;56801.9)	763.5 (646.1;902.8)	-30.3(-46.8;-2.9)
Mato Grosso	6760 (5891.9;7661.8)	1065.6 (935.9;1193.3)	12646.5 (11001.1;14370.4)	432.8 (375.9;491.6)	-59.4(-65.2;-52.5)
Mato Grosso do Sul	8498.8 (7759.2;9203.4)	1137.4 (1029.6;1227.4)	11876.8 (10349.4;13471.1)	436.9 (379.4;494.6)	-61.6(-66.2;-56)
Minas Gerais	108699.4 (99915.8;118951.1)	1257.1 (1151.5;1368.4)	105525.7 (91920.7;118346.2)	404.8 (352.7;453.6)	-67.8(-71.9;-63.2)
Pará	23740.3 (20920.8;26550.4)	1352.4 (1192.7;1506.7)	37477.3 (32192.9;42111.1)	593.8 (509.4;667.4)	-56.1(-62.3;-49)
Paraíba	20370.9 (16680.5;24037.9)	906.2 (745.9;1063.7)	22708.5 (19399.8;26237.8)	472.5 (404.1;546.5)	-47.9(-58.3;-33.6)
Paraná	64464.8 (60374.7;68432.4)	1598.9 (1481.9;1695.9)	69504.9 (60465.7;78336.2)	559.2 (486.1;627.3)	-65(-69.4;-60.4)
Pernambuco	50355.8 (45411.8;55130.7)	1224.2 (1104.8;1333.5)	53525 (46384.9;60987.6)	566.7 (492.7;645.2)	-53.7(-60.7;-46.2)
Piaui	14999.3 (12981.2;16969)	1249.7 (1089.5;1408.4)	20827 (17690.4;23559.6)	552.8 (470.1;625.8)	-55.8(-63;-47.4)
Rio de Janeiro	122527.2 (114879.3;129568.4)	1443 (1347.1;1525.5)	101343.1 (89470.7;112513)	460.6 (406.7;511.1)	-68.1(-71.6;-64.3)
Rio Grande do Norte	12068 (10468.4;13932.6)	783.6 (678.3;903)	14096.8 (11589.3;16762)	367.4 (303;437.1)	-53.1(-62.2;-40.7)
Rio Grande do Sul	71869.3 (66775;76379.1)	1270.7 (1174.6;1352.1)	78403.3 (67989.1;88418.6)	508.7 (440.8;573.8)	-60(-64.5;-55.2)
Rondônia	3766.2 (3297.1;4198.3)	1782.2 (1621.8;1942.6)	7001 (5912.5;8182.5)	521.9 (441.4;607.5)	-70.7(-75.5;-65.5)
Roraima	468.7 (419.6;519.8)	1237.8 (1123.4;1350.9)	1442.3 (1258.1;1619.2)	500 (436.7;556.5)	-59.6(-64.9;-53.9)
Santa Catarina	29055.8 (27032.4;31053.7)	1417.3 (1310.5;1520.9)	32271.5 (28229.2;36649.5)	440.8 (384.6;499.4)	-68.9(-72.7;-65)
São Paulo	201067 (186754.2;216302.7)	1168.1 (1074.4;1246.4)	206713.7 (181100.9;230259.8)	399.1 (348.7;444.2)	-65.8(-69.3;-61.8)
Sergipe	8730.4 (7749.7;9702.2)	1292.3 (1151.2;1427.3)	11940.9 (10057.6;13906.4)	570.2 (478;664.6)	-55.9(-63.6;-46.9)
Tocantins	3503.7 (2933.1;4088.3)	1216.2 (1047.7;1386.3)	7967 (6754.7;9233.1)	603.5 (512;697.2)	-50.4(-60.1;-39.2)
**B.2.3.2-Intracerebral hemorrhage**					(;)
Acre	1757.7 (1577.9;1961.3)	877 (793.1;970.4)	3337.8 (2971.3;3756)	484.3 (431.3;543.1)	-44.8(-52.6;-35.3)
Alagoas	20756.3 (18072.7;23944.2)	1342.9 (1164.5;1545.8)	23503.2 (20270.5;26917.1)	696.8 (602.7;797.2)	-48.1(-59;-36.1)
Amapá	925.7 (822.9;1016.6)	752.8 (682.5;819.5)	2845.7 (2495.7;3187.9)	469.8 (414.2;525.2)	-37.6(-45.2;-28.4)
Amazonas	9619.8 (8639.1;10610.1)	1039.8 (942.8;1143.7)	14370.5 (12524.1;16340.8)	453.4 (396.4;515.7)	-56.4(-62.3;-49.4)
Bahia	84677.6 (74285.2;96506.6)	1129.1 (987.5;1292.9)	90291.3 (74543.4;107505.2)	549.9 (454.1;654.9)	-51.3(-61.8;-38.9)
Brazil	1303555.9 (1249976.5;1374489.1)	1283.5 (1227.7;1351)	1086463 (1025413.3;1141541.4)	449.2 (423.3;472.5)	-65(-67.6;-62.5)
Ceará	38358.7 (32448.7;45304.9)	858.6 (718;1018.3)	51669 (42481.5;61768.7)	510.3 (419.3;609.2)	-40.6(-54.8;-23.4)
Distrito Federal	10017.7 (8886.2;11645.6)	1273.3 (1137.5;1448.2)	9644.7 (8461.1;10976.3)	339.8 (296.7;385.6)	-73.3(-77.2;-68.4)
Espírito Santo	24966.1 (23680.2;26430.9)	1488.7 (1411.4;1572.5)	22397.8 (19379;25441)	493.6 (428;560.4)	-66.8(-71.4;-62.2)
Goiás	39798.3 (34140.5;47575.2)	1575.2 (1358.2;1885)	32574.2 (27320.6;38124)	438.6 (369.2;511.5)	-72.2(-77.8;-65.5)
Maranhão	34728.9 (27796.6;43087.2)	1107.8 (881;1382.6)	47718.5 (39527.8;58098.6)	697.6 (577.8;850.1)	-37(-52.3;-15.5)
Mato Grosso	9773.4 (8348.4;11190)	971.6 (847.6;1097.2)	14273.3 (12554.1;16136.1)	395.1 (348;447.4)	-59.3(-66;-51.2)
Mato Grosso do Sul	14514.1 (13377.8;15746.3)	1346.4 (1249.4;1458.7)	13450.7 (11797.6;15347.6)	436 (383.3;495.6)	-67.6(-72;-62.7)
Minas Gerais	167435.9 (153866.2;189273.2)	1474.3 (1357.6;1655.4)	108433.6 (96309;122277.7)	407.8 (362.1;459.1)	-72.3(-76.6;-68)
Pará	28737 (25168.4;32327.3)	1183.6 (1047.4;1327)	37239.6 (32252.1;41938)	492.7 (426.7;553.4)	-58.4(-64.9;-51)
Paraíba	19904.5 (16932.5;23582.1)	834.9 (707.5;992.8)	22961.4 (19839.1;26504.6)	491.2 (424.2;567.4)	-41.2(-53.8;-25.7)
Paraná	81687.9 (76990.4;86999.7)	1468.5 (1382.2;1562.7)	54832.8 (47517.7;62195.1)	399.4 (347.3;452.6)	-72.8(-76.5;-68.9)
Pernambuco	65139.7 (60440.4;70286)	1322.6 (1228;1428.4)	63855 (56085;72426.3)	612.4 (539.9;694)	-53.7(-60.1;-46.5)
Piaui	18213.3 (16144.9;20912.9)	1151.7 (1020;1313.5)	18767.4 (16413.5;21459.4)	498.8 (437.3;570.2)	-56.7(-63.9;-48.1)
Rio de Janeiro	189847.8 (178940.3;202216.9)	1738.9 (1643.7;1847.2)	117082.3 (103795.5;131957.4)	517.9 (459.5;584.3)	-70.2(-73.7;-66)
Rio Grande do Norte	12755 (11224.4;14401)	759 (666.5;857.1)	13759.1 (11229.2;16571.9)	351.7 (287.4;423.3)	-53.7(-63.2;-42.8)
Rio Grande do Sul	86550.1 (81293.4;91677.5)	1177.9 (1106.3;1248.7)	63875.4 (55424.1;72522)	412.8 (357.6;470.1)	-65(-69.4;-60)
Rondônia	6250.9 (5307;7125.5)	1379.4 (1218.2;1535.9)	7486.2 (6402.6;8872.1)	445.2 (380.7;522.7)	-67.7(-73.5;-60)
Roraima	841.1 (724.7;946.2)	1067.6 (952.2;1181.9)	1728 (1517.2;1949.5)	399.6 (352.9;448.2)	-62.6(-68;-56.5)
Santa Catarina	36527.9 (33998.7;39097.7)	1274.8 (1186.5;1365.9)	27210.5 (23570;31005.4)	322.1 (279.3;365.9)	-74.7(-78;-71)
São Paulo	284456.9 (263629.8;309048.7)	1216.8 (1131.4;1310.4)	202992.4 (179780.6;228623.2)	366 (324.4;411.6)	-69.9(-73.9;-65.7)
Sergipe	9901 (8860.4;11012.8)	1129.8 (1004.7;1258.2)	12224.9 (10331.9;14328.5)	519.8 (440;608.4)	-54(-62.4;-44.6)
Tocantins	5412.9 (4570.5;6344.5)	1100.5 (928;1289.6)	7937.7 (6755.7;9253.1)	526.6 (449.1;612.8)	-52.1(-62.5;-39.9)
**B.2.3.3-Subarachnoid hemorrhage**					(;)
Acre	638.8 (553.3;736.4)	234.8 (205;268)	1389.3 (1226.4;1577.6)	173.7 (153.4;196.9)	-26(-38.1;-10.9)
Alagoas	6202.9 (5172.1;7328.1)	328.6 (273.4;385.4)	7662.7 (6534.3;8941.3)	213.9 (182.7;248.4)	-34.9(-49;-16.4)
Amapá	279.6 (239.4;323.5)	165.2 (145;192.4)	1108.2 (988.3;1270.6)	152.8 (136;175.3)	-7.5(-21;8.9)
Amazonas	2390.6 (2083.9;2722.8)	188.1 (166;215.7)	5187.7 (4523.3;6016.3)	141.4 (123.3;163.9)	-24.9(-37.3;-10.1)
Bahia	23553.6 (20282.4;27063.9)	264.2 (227.7;306.7)	31744.9 (25977.8;38214.4)	188 (154.6;225.7)	-28.8(-44.6;-9)
Brazil	340253.3 (301621.6;356874.5)	284 (253.8;297.2)	412071 (384850.3;438730)	169.8 (158.3;180.8)	-40.2(-44.9;-32.6)
Ceará	11782.8 (9708;14149.8)	229.9 (189.8;273.3)	17973.3 (14699.9;22114)	173.5 (142;212.6)	-24.5(-41.6;1.1)
Distrito Federal	3354.5 (2951.9;3778.2)	295.7 (260.6;333.6)	4650.5 (3988.1;5365)	145.9 (124.6;167.3)	-50.6(-58.4;-41)
Espírito Santo	6336.6 (5170.6;6918.2)	310.8 (254.6;338.4)	8301.9 (7019.7;9774.7)	181.6 (154.2;212.4)	-41.6(-50.6;-29.5)
Goiás	10011.5 (8705.3;11624.9)	318 (275.8;370)	12402.6 (10301.2;14840.8)	160.5 (133.8;190.7)	-49.5(-59.9;-37.5)
Maranhão	11886.7 (8770.9;15728.3)	307.3 (228.6;399.9)	17869.9 (14341;21910.3)	242.5 (195.1;298.1)	-21.1(-43.6;9.7)
Mato Grosso	3105.3 (2559.7;3680)	224.6 (190.2;262.6)	6033.1 (5267.1;6920.4)	155.1 (135.6;178.1)	-30.9(-43.3;-14.6)
Mato Grosso do Sul	3799.2 (3420.5;4170.8)	282.1 (254.3;309.2)	5212.8 (4497.3;5959.5)	164 (141.9;187.1)	-41.9(-50.3;-31.9)
Minas Gerais	43670.8 (37136.2;47942.6)	329.8 (283.9;362)	44149.6 (38291.3;50524.6)	170 (147.6;194)	-48.5(-55.6;-39.3)
Pará	6943.1 (5981.5;7928.9)	213 (184.9;245)	13683 (11958.7;15648.5)	161.6 (141.3;184.9)	-24.1(-37;-8)
Paraíba	7039.5 (6024;8166.2)	277.2 (236.8;324)	8299.7 (7006;9682)	177.4 (149.9;206.9)	-36(-49.6;-19.4)
Paraná	19783.6 (17429.1;21454.1)	292.8 (259.2;317.4)	21982.1 (18625.7;25543.5)	161.2 (137.6;187.1)	-44.9(-53.3;-35.9)
Pernambuco	13004.9 (11647.3;14757.9)	229.7 (205.5;263.4)	20325 (17670.8;23381)	190.1 (165.4;218.8)	-17.3(-31;-1.2)
Piaui	5504.3 (4717;6353.5)	285.2 (246.2;325.6)	6778.8 (5793.9;7860.2)	178.2 (152.6;206.3)	-37.5(-49.6;-22.1)
Rio de Janeiro	44279 (33962.9;48808.5)	363.1 (283.3;399.4)	39325.9 (34177.3;44894.3)	181.7 (158.8;207.1)	-49.9(-57.8;-35)
Rio Grande do Norte	3362.3 (2890.3;4087.5)	176.8 (150.3;218.8)	4918.8 (3993.1;6328.9)	123.1 (100;157.3)	-30.4(-45.1;-11.2)
Rio Grande do Sul	18994.6 (17368.3;20855.9)	227.8 (208.6;250.4)	20478.2 (17663.2;23770.2)	141 (122.3;162.6)	-38.1(-46.9;-27.7)
Rondônia	1646.1 (1305.3;1948.2)	240.6 (199.8;277.8)	3027.1 (2591.3;3565.5)	163.2 (140.1;191.8)	-32.2(-45;-13.4)
Roraima	247.5 (203.2;289.8)	202.9 (172.7;233.4)	718.3 (628.4;855.1)	140 (122.8;165.9)	-31(-42.6;-16.3)
Santa Catarina	8895.8 (8096.2;9718.4)	249.7 (227.1;273.2)	10987.1 (9521;12815.9)	128.6 (111.7;149.5)	-48.5(-55.9;-39)
São Paulo	79495.5 (68778.8;86635.1)	286 (251.5;311.9)	90900.3 (79393.9;103394.2)	166.1 (145.3;188.7)	-41.9(-49.9;-31.7)
Sergipe	2424.8 (2108.6;2782.6)	227 (197;261.6)	3985.1 (3284.1;4788.7)	159.3 (131.8;191.9)	-29.8(-44.6;-11.8)
Tocantins	1619.6 (1328.1;1906.4)	250.7 (210.1;292.7)	2975 (2491.9;3503)	182.6 (153.7;215.9)	-27.1(-41.6;-7.4)


*Source: Data derived from Global Burden of Disease Study 2019, Institute for Health Metrics and Evaluation, University of Washington.^*46*^*

**Table 2-8:** – Number of YLLs and age-standardized YLL rates (per 100 000) due to stroke, ischemic stroke, subarachnoid hemorrhage, and intracerebral hemorrhage in 1990 and 2019, and percent change of rates, in Brazil, according to age group.

Cause of death and age group	1990		2019		Percent change (95% UI)
	Number (95% UI)	Rate (95% UI)	Number (95% UI)	Rate (95% UI)	
**B.2.3-Stroke**					
Under 5	36373.4 (26471.3;49662)	214.7 (156.3;293.2)	5970.3 (4335.4;8153.1)	38.5 (28;52.6)	-82.1 (-89.2;-70)
15-49 years	716076.9 (689520.8;745933.4)	934.3 (899.6;973.2)	448555.9 (425616.6;472166.9)	388.4 (368.5;408.8)	-58.4 (-61.2;-55.7)
50-69 years	1084139.7 (1044839.3;1128620.8)	6910.8 (6660.3;7194.3)	1067928.7 (1013004.9;1118810)	2647.1 (2511;2773.2)	-61.7 (-64.3;-59.3)
5-14 years	19376.5 (17280.8;21317.5)	54.8 (48.9;60.3)	9330.4 (7859.9;10858.3)	28.9 (24.4;33.7)	-47.2 (-55.7;-37.6)
70+ years	734877.8 (682090.6;768788.5)	17372.7 (16124.8;18174.4)	1046380.8 (932190.5;1117926.7)	7994.7 (7122.2;8541.3)	-54 (-57.4;-51)
Age-standardized	2590844.4 (2504589.7;2679788.7)	2778.6 (2659.5;2879.2)	2578166 (2413609.9;2702585.5)	1098.7 (1025.8;1153.7)	-60.5 (-62.7;-58.4)
All Ages	2590844.4 (2504589.7;2679788.7)	1740.7 (1682.8;1800.5)	2578166 (2413609.9;2702585.5)	1189.9 (1114;1247.4)	-31.6 (-35.8;-28)
**B.2.3.1-Ischemic stroke**					
Under 5	4135.2 (2990.3;5467.5)	24.4 (17.7;32.3)	384.4 (283.6;515.9)	2.5 (1.8;3.3)	-89.8 (-93.7;-83.7)
15-49 years	86173.7 (79496.8;93439.3)	112.4 (103.7;121.9)	43383.5 (39668.9;47636.4)	37.6 (34.3;41.2)	-66.6 (-71.1;-61.1)
50-69 years	349029.3 (329328.7;368931.8)	2224.9 (2099.3;2351.7)	299595.2 (278841.5;320496.5)	742.6 (691.2;794.4)	-66.6 (-69.8;-62.9)
5-14 years	1620.3 (1376.7;1865.2)	4.6 (3.9;5.3)	440.2 (365;521.5)	1.4 (1.1;1.6)	-70.2 (-75.9;-63.1)
70+ years	506076.5 (466579.2;530504)	11963.8 (11030.1;12541.3)	735828.9 (648142.4;789381.6)	5621.9 (4952;6031.1)	-53 (-56.8;-49.5)
Age-standardized	947035.1 (895299.9;992583.8)	1211.1 (1133.8;1268.9)	1079632.1 (982065;1148602.5)	479.7 (435.1;510.8)	-60.4 (-63.5;-57.3)
All Ages	947035.1 (895299.9;992583.8)	636.3 (601.5;666.9)	1079632.1 (982065;1148602.5)	498.3 (453.3;530.1)	-21.7 (-28.4;-15.1)
**B.2.3.2-Intracerebral hemorrhage**					
Under 5	17235.2 (11675.5;25057.4)	101.8 (68.9;147.9)	1730.8 (1199.6;2437.8)	11.2 (7.7;15.7)	-89 (-93.8;-80.6)
15-49 years	436522.1 (414806.9;474485.7)	569.5 (541.2;619.1)	238357.1 (221826.9;252852.4)	206.4 (192.1;218.9)	-63.8 (-68.4;-60.4)
50-69 years	627767.2 (600065.4;659989.2)	4001.7 (3825.1;4207.1)	582538.8 (551911.4;614231.7)	1444 (1368;1522.5)	-63.9 (-66.5;-61.2)
5-14 years	7857.3 (6901.7;8868.3)	22.2 (19.5;25.1)	2786.3 (2314.7;3296.6)	8.6 (7.2;10.2)	-61.1 (-68.2;-52.6)
70+ years	214174.1 (199342.6;227529)	5063.1 (4712.5;5378.8)	261050 (235043.8;282383)	1994.5 (1795.8;2157.5)	-60.6 (-64.5;-56.9)
Age-standardized	1303555.9 (1249976.5;1374489.1)	1283.5 (1227.7;1351)	1086463 (1025413.3;1141541.4)	449.2 (423.3;472.5)	-65 (-67.6;-62.5)
All Ages	1303555.9 (1249976.5;1374489.1)	875.8 (839.8;923.5)	1086463 (1025413.3;1141541.4)	501.4 (473.3;526.9)	-42.7 (-47.2;-38.7)
**B.2.3.3-Subarachnoid hemorrhage**					
Under 5	15002.9 (9308;20320.5)	88.6 (55;120)	3855.1 (2796.2;5324.8)	24.9 (18;34.4)	-71.9 (-83.2;-44.9)
15-49 years	193381.1 (168039.5;203641.4)	252.3 (219.2;265.7)	166815.3 (156642.9;182531.3)	144.4 (135.6;158.1)	-42.8 (-47.7;-29.9)
50-69 years	107343.3 (97585.4;113734.5)	684.3 (622.1;725)	185794.7 (169749.3;200067.8)	460.5 (420.8;495.9)	-32.7 (-38.4;-24.3)
5-14 years	9898.9 (8666.6;10917.5)	28 (24.5;30.9)	6104 (5139.8;7158.2)	18.9 (15.9;22.2)	-32.4 (-43.7;-18.4)
70+ years	14627.2 (13237.5;17395.9)	345.8 (312.9;411.2)	49501.9 (40527.8;55106.2)	378.2 (309.6;421)	9.4 (-23.2;27.2)
Age-standardized	340253.3 (301621.6;356874.5)	284 (253.8;297.2)	412071 (384850.3;438730)	169.8 (158.3;180.8)	-40.2 (-44.9;-32.6)
All Ages	340253.3 (301621.6;356874.5)	228.6 (202.7;239.8)	412071 (384850.3;438730)	190.2 (177.6;202.5)	-16.8 (-23.5;-5.2)

*Source: Data derived from Global Burden of Disease Study 2019, Institute for Health Metrics and Evaluation, University of Washington.^*46*^*

**Table 2-9:** – Number of YLDs and age-standardized YLD rates (per 100 000) due to stroke, ischemic stroke, subarachnoid hemorrhage, and intracerebral hemorrhage in 1990 and 2019, and percent change of rates, in Brazil and its Federative Units.

Cause of death and location	1990		2019		Percent change (95% UI)
	Number (95% UI)	Rate (95% UI)	Number (95% UI)	Rate (95% UI)	
**B.2.3-Stroke**					
Acre	315.6 (232.9;404.1)	159.2 (118;201.2)	825.1 (606.5;1042.6)	120.9 (88.8;153.7)	-24(-28.3;-19.3)
Alagoas	2754.3 (2009.9;3460.9)	186.9 (136.7;237.4)	4490.7 (3288.4;5698.7)	136.8 (100.5;172.9)	-26.8(-31.5;-22)
Amapá	191.1 (139.4;243.8)	154.5 (112.9;196.4)	708.2 (515.4;897.8)	119 (86.8;151.2)	-23(-27.3;-18.1)
Amazonas	1623 (1187.4;2072.9)	171.2 (126.4;216.2)	3713.3 (2706.4;4687.6)	116.6 (84.2;147.1)	-31.9(-36.2;-27.3)
Bahia	12715.9 (9333.4;16054.2)	171.5 (125.5;218.1)	19723.7 (14433.8;24962.7)	121.4 (88.8;153.9)	-29.2(-33.3;-24.7)
Brazil	175647 (130408.3;222000.4)	180.4 (133.2;228.8)	283557.2 (208451.8;357622.1)	120.9 (88.7;152.6)	-33(-34.8;-31.1)
Ceará	6314.9 (4619.7;8000.2)	144.1 (104.7;182.6)	12030.7 (8775.1;15287.7)	119.9 (87.3;152.6)	-16.8(-21.8;-11.3)
Distrito Federal	1358.4 (1002.7;1722)	182.6 (135.3;232)	3400.5 (2488.7;4353.9)	124.4 (90.4;159.5)	-31.9(-36.4;-27.5)
Espírito Santo	3144.4 (2307.5;3997.4)	193.5 (140.7;246)	5509.3 (4060.9;6993.3)	126.9 (93.2;161.1)	-34.4(-38.7;-30.1)
Goiás	4211.8 (3090.3;5372.3)	173.5 (126.6;222)	8042.5 (5907.7;10184.9)	113 (82.9;143.8)	-34.8(-39.2;-30.8)
Maranhão	4463.2 (3280.2;5679.7)	157.7 (115.9;201)	8115.7 (5942.9;10311.5)	117.5 (86;148.7)	-25.5(-31.1;-19)
Mato Grosso	1584.1 (1154.8;2003.3)	160.2 (117;202.9)	3944.7 (2881.2;5013.1)	114.7 (84;145.6)	-28.4(-33.5;-23.4)
Mato Grosso do Sul	1798 (1331.6;2289.7)	172.1 (127.6;217.7)	3590.7 (2643.5;4545.9)	121.1 (89.7;153.8)	-29.6(-34.4;-25.2)
Minas Gerais	19927.3 (14714.4;25139.3)	185.2 (135.1;232.7)	30355.4 (22430.4;38566.6)	117.6 (86.8;149.7)	-36.5(-40.5;-32)
Pará	4349.2 (3209.4;5487.1)	178.9 (130.2;227)	8858.9 (6493.4;11120.3)	118.3 (87;150)	-33.9(-38;-29.6)
Paraíba	3654.6 (2656.5;4661.7)	151.8 (110.6;193.6)	5438.7 (4011.3;6852.3)	116.1 (85.5;145.9)	-23.5(-28.4;-18.1)
Paraná	10907.4 (7979;13836)	204.2 (149.8;259.5)	16967.1 (12436.9;21666.3)	129.6 (95.5;165.4)	-36.5(-40.6;-32.1)
Pernambuco	8704.7 (6396.6;11162)	179.1 (131.2;230.4)	12587.5 (9228.9;16057.8)	124.4 (91.2;158.4)	-30.5(-34.8;-25.9)
Piaui	2463.3 (1807.4;3116.9)	158.1 (115.7;201.3)	4431.3 (3229.6;5622.5)	117.8 (85.9;149.4)	-25.5(-30.1;-20.6)
Rio de Janeiro	20883 (15402.8;26611.4)	204.7 (151.8;260.7)	27912.2 (20540.5;35371.1)	128.1 (94.1;162.2)	-37.4(-41.5;-32.9)
Rio Grande do Norte	2501.1 (1834.2;3194.4)	146.5 (107.4;186.3)	4227.7 (3103.5;5405.8)	108.7 (79.8;138.8)	-25.8(-30.3;-21.4)
Rio Grande do Sul	13338.6 (9727.6;16924)	191.3 (140.5;242.4)	19074.6 (13941.1;24256.3)	128.3 (94;162.9)	-33(-37.3;-28.8)
Rondônia	859.9 (621.9;1087)	192.8 (141.5;246.6)	1890.5 (1368;2423.8)	116.7 (84.3;148.8)	-39.5(-43.8;-35.2)
Roraima	132.7 (96.3;169.1)	159.1 (117.9;202.8)	474.1 (344.7;602.1)	111.9 (81.1;141.8)	-29.6(-34;-24.8)
Santa Catarina	5369.6 (3950.9;6821.2)	192.6 (140.3;244.3)	9505.9 (6956.5;12073.4)	118.1 (86.4;150.3)	-38.7(-42.9;-34.4)
São Paulo	39775.5 (29243.7;50394.2)	180.5 (132.3;225.6)	63114.8 (45994.9;80990.2)	118.2 (86.7;151)	-34.5(-38.4;-30.2)
Sergipe	1556 (1134.5;1963.8)	177 (130;222.6)	2917.1 (2147.5;3706)	126.9 (93.1;161.1)	-28.3(-32.9;-24.1)
Tocantins	749.2 (545.2;950.1)	157.4 (114.9;199.7)	1706.2 (1240.4;2167.6)	114.7 (82.9;145.9)	-27.1(-31.7;-21.9)
**B.2.3.1-Ischemic stroke**					(;)
Acre	203.3 (145;263.7)	108.6 (77.6;140.6)	533.3 (382.4;685.6)	81.8 (59.2;105.5)	-24.7(-30.2;-18.4)
Alagoas	1867.4 (1326.7;2404.7)	130.8 (92.5;168.9)	3026.7 (2167.3;3859.2)	94.3 (68;121.7)	-27.9(-33.9;-21.5)
Amapá	121.4 (87.9;157.1)	104.3 (75.8;134.5)	451.6 (323.1;583.8)	80.5 (57.6;102.8)	-22.9(-28.5;-16.1)
Amazonas	1026.6 (733.4;1328)	115.4 (83.5;149.6)	2372.4 (1692.6;3054.5)	77.9 (56.5;100.8)	-32.5(-38.2;-26)
Bahia	8358.5 (5907.2;10899.7)	115.6 (82.5;150.5)	13021.4 (9286.5;16835)	81.1 (57.7;104.9)	-29.9(-35.1;-24)
Brazil	114029.9 (82236.3;147025)	122.2 (88;156.8)	188474.4 (134840.6;241680.6)	81.2 (58.3;104)	-33.5(-35.9;-31)
Ceará	4119.6 (2917.8;5309.2)	95.6 (67.7;124.3)	7982.2 (5730.1;10296)	80.3 (57.6;103.6)	-16(-22.6;-8.9)
Distrito Federal	806.4 (570.5;1047.1)	121.6 (86.5;157.4)	2191.9 (1539.4;2856.1)	84.2 (59.7;110)	-30.7(-36.8;-24.3)
Espírito Santo	2031.2 (1453.3;2615.8)	131.1 (93.4;168.8)	3649.1 (2606.6;4719.9)	85.3 (60.9;110.5)	-34.9(-40.2;-29.5)
Goiás	2635.3 (1878.6;3385.1)	115.6 (83;149.8)	5236.5 (3763.7;6709.3)	75.4 (53.8;97.2)	-34.8(-40.4;-29.2)
Maranhão	2973.5 (2130.2;3851.3)	108.3 (77.7;141.6)	5283.4 (3803.4;6818.8)	77.8 (56.2;100.3)	-28.2(-35.4;-19.5)
Mato Grosso	997.1 (707;1288.4)	109.7 (78;141.4)	2578.9 (1824.5;3344.9)	77.7 (55.5;100.7)	-29.2(-35.5;-22.2)
Mato Grosso do Sul	1118.4 (811.3;1444.4)	113.9 (81.9;146.4)	2350.4 (1699.6;3034)	80.8 (58.3;104.2)	-29.1(-35.7;-22.9)
Minas Gerais	12831.1 (9202.7;16648.4)	124.4 (89.1;161.7)	20194 (14245.6;25876.1)	78.4 (55.4;100.7)	-37(-42.4;-31.2)
Pará	2842.7 (2038.8;3656.9)	123.4 (88.1;159.1)	5840 (4174.8;7513.5)	80.8 (57.8;103.8)	-34.5(-39.9;-28.5)
Paraíba	2447.1 (1739.1;3198.3)	101.8 (72.6;133.1)	3526.2 (2527;4564.1)	75.2 (54;97.3)	-26.1(-32.7;-19.2)
Paraná	7155.3 (5075.6;9228.7)	141.8 (99.8;184.4)	11638.4 (8284.6;14990.1)	89.8 (64;116.2)	-36.7(-42.2;-30.9)
Pernambuco	5723.7 (4088.5;7503.4)	120.7 (86.7;157.3)	8240.2 (5882.1;10590)	82.7 (59.1;106.8)	-31.4(-36.8;-25.1)
Piaui	1603.7 (1141;2086.3)	106.5 (75.5;139.2)	2968.6 (2131.1;3859.3)	79.2 (57;103)	-25.7(-31.8;-18.7)
Rio de Janeiro	13465.6 (9762.2;17325.1)	137.6 (99.8;177.1)	18411.2 (13185;23879.7)	84.6 (60.4;109.4)	-38.5(-43.9;-33.1)
Rio Grande do Norte	1649.9 (1192.7;2141.4)	97.6 (70.8;127)	2751.9 (1978.8;3570.7)	71.4 (51.5;92.5)	-26.8(-32.2;-21.2)
Rio Grande do Sul	8764.4 (6296.6;11278.3)	131 (94.6;167.5)	13036 (9252.5;16922.7)	87.1 (62;112.8)	-33.5(-38.6;-28.1)
Rondônia	533.7 (375;691.7)	135.9 (96.9;177.6)	1240.2 (865.9;1593.7)	79.6 (55.7;102.9)	-41.4(-46.6;-36)
Roraima	81.3 (57.6;105.5)	109 (79.2;140.9)	308.1 (218.6;395.9)	77.2 (54.4;99.9)	-29.2(-34.9;-22.6)
Santa Catarina	3412.5 (2447.8;4395.7)	130.3 (93.1;169.5)	6305.7 (4518.2;8157)	79.7 (57;103)	-38.8(-44.7;-33.1)
São Paulo	25756.5 (18589.5;33126.7)	122.8 (89;158)	42288.6 (30369.4;54894.6)	79.9 (57.1;103.6)	-34.9(-40.2;-29.3)
Sergipe	1032 (735.6;1325.6)	121 (85.9;156.1)	1936.1 (1385.7;2511.1)	86.1 (61.6;111.4)	-28.8(-34.9;-23.1)
Tocantins	471.9 (336;606.2)	106 (75.9;137.7)	1111.6 (790.3;1438.5)	76.6 (54.3;98.8)	-27.7(-33.9;-20.4)
**B.2.3.2-Intracerebral hemorrhage**					(;)
Acre	81.3 (57.8;105.6)	37.2 (26.8;48.1)	205 (147.8;267.5)	28 (20.2;36.5)	-24.7(-32.6;-16.8)
Alagoas	666 (479.2;863.2)	42.6 (30.7;55.1)	1072.4 (773.5;1397.4)	31.4 (22.6;40.8)	-26.3(-33.2;-18.6)
Amapá	49.7 (35.7;64.6)	36.5 (26.6;46.8)	177.4 (127.5;229.8)	27.2 (19.7;35.4)	-25.4(-32.1;-17.3)
Amazonas	442.4 (314.1;573.9)	42.5 (30.3;54.5)	944.7 (676.1;1221.9)	27.8 (19.8;36.1)	-34.6(-41;-27.6)
Bahia	3261 (2355.6;4233.4)	42.1 (30.3;54.4)	4796.7 (3456.7;6283.3)	29 (20.8;38.1)	-31(-38;-23.9)
Brazil	46180.6 (33564.1;59463.8)	44.3 (32.3;56.6)	67702.4 (49062.3;87760.6)	28.5 (20.7;36.9)	-35.7(-38.8;-32.2)
Ceará	1560.2 (1120.4;2029.6)	34.5 (24.7;44.8)	2863.7 (2053.1;3744.4)	28.2 (20.2;36.9)	-18.3(-26.3;-9.3)
Distrito Federal	398.7 (288.3;520.4)	46.4 (33.8;59.9)	832.9 (596;1089.9)	28.6 (20.5;37.5)	-38.2(-44.6;-31.4)
Espírito Santo	846 (606.7;1104.9)	48.3 (34.4;62.6)	1342.8 (974.8;1759.2)	30.3 (22.2;39.6)	-37.2(-44;-29.9)
Goiás	1186.2 (840.5;1549.1)	44.6 (32.1;58.1)	1980.3 (1412.5;2581.1)	27 (19.4;35.1)	-39.5(-45.7;-32.8)
Maranhão	1061.3 (763.1;1378.4)	35.4 (25.6;46.3)	1993.1 (1432.7;2590.5)	28 (20.1;36.4)	-20.8(-28.9;-12.2)
Mato Grosso	422.2 (301.3;553.9)	37.5 (27.2;49.5)	948.5 (689.3;1237.3)	26.2 (19.1;34.2)	-30.1(-36.6;-23.1)
Mato Grosso do Sul	510.5 (362.3;665.6)	44.6 (31.9;58.2)	887.3 (629.4;1156.2)	29.2 (20.8;37.9)	-34.6(-41.3;-27)
Minas Gerais	5358.3 (3909.3;6943.6)	46.5 (34.1;60)	7206.4 (5120.7;9398.5)	27.9 (19.9;36.4)	-40(-46.2;-33)
Pará	1117.7 (800.9;1461.5)	42.1 (30.4;54.7)	2108 (1507.6;2730.8)	26.5 (19.1;34)	-37.1(-43.3;-30.6)
Paraíba	863.4 (616.9;1116.4)	35.5 (25.5;46)	1368.4 (982.5;1785.8)	29.3 (21;38.1)	-17.6(-26.4;-8)
Paraná	2855.1 (2053.2;3711.4)	48.5 (35.1;63.2)	3796.4 (2709;4981.8)	28.6 (20.5;37.4)	-41(-47.5;-34.3)
Pernambuco	2285.5 (1644.5;2972.6)	45.1 (32.6;58.6)	3170.9 (2293.2;4123.8)	30.6 (22.1;39.8)	-32.1(-38.3;-25.3)
Piaui	617.3 (443.9;801.9)	37.4 (26.9;48.8)	1020.9 (725.4;1325.9)	27 (19.3;35.1)	-27.8(-34.9;-20.7)
Rio de Janeiro	5769.1 (4169.7;7533.4)	52.8 (38.4;68.4)	7054.1 (5057.4;9121.2)	32.4 (23.3;41.9)	-38.6(-45;-31)
Rio Grande do Norte	613.5 (442.4;798.3)	35.2 (25.2;45.7)	1025.4 (733.4;1317.3)	26.1 (18.5;33.5)	-25.9(-33.7;-17.2)
Rio Grande do Sul	3471.6 (2512.5;4482.4)	46.5 (33.7;60.1)	4412.9 (3174.2;5808.1)	30 (21.6;39.5)	-35.4(-41.6;-28.5)
Rondônia	242 (171.6;314)	44.1 (31.5;56.7)	453.7 (325.9;593)	26.4 (19;34.3)	-40.1(-46.2;-34.2)
Roraima	37.2 (26.4;48.9)	37.7 (27.2;49.3)	113.7 (80.6;149.1)	24.4 (17.6;31.7)	-35.2(-41.5;-27.8)
Santa Catarina	1479.9 (1065.9;1929.2)	48.2 (34.7;63.2)	2246.6 (1606.6;2960)	27.3 (19.6;35.9)	-43.3(-48.9;-36.8)
São Paulo	10391.4 (7440.7;13527)	43.7 (31.6;56.5)	14560.9 (10443.5;18974.8)	27 (19.4;35.2)	-38.1(-44.4;-30.6)
Sergipe	393.6 (287.3;508.3)	42.5 (31.1;54.9)	705.9 (509.7;909.4)	29.7 (21.5;38.1)	-30.3(-36.9;-23.2)
Tocantins	199.6 (142.4;259.6)	37.9 (27.3;48.7)	413.4 (296.3;532.6)	26.8 (19.2;34.5)	-29.1(-36.8;-20.8)
**B.2.3.3-Subarachnoid hemorrhage**					(;)
Acre	31.1 (21.8;41.5)	13.3 (9.3;17.8)	86.9 (60.8;117.1)	11.1 (7.8;15)	-16.4(-26.3;-5)
Alagoas	220.9 (153;298.4)	13.6 (9.4;18.4)	391.6 (277;521.1)	11.1 (7.8;14.8)	-18.3(-29.1;-5.6)
Amapá	20 (14;27.2)	13.6 (9.7;18.4)	79.2 (56.1;108.1)	11.3 (8;15.2)	-17.2(-27.8;-6.2)
Amazonas	154 (108.4;211.1)	13.3 (9.4;18.1)	396.2 (280;541.1)	10.9 (7.8;14.8)	-17.5(-27.3;-6.1)
Bahia	1096.5 (769.6;1497.5)	13.8 (9.7;18.7)	1905.6 (1334.1;2552.4)	11.3 (7.9;15.1)	-18(-28.8;-6.8)
Brazil	15436.6 (10801.8;20590.3)	13.9 (9.7;18.6)	27380.3 (19490.9;36579.7)	11.2 (7.9;15)	-19.5(-23.7;-15.4)
Ceará	635.1 (448.2;859.3)	13.9 (9.8;18.9)	1184.8 (841.8;1609.4)	11.4 (8.1;15.4)	-17.8(-28;-5)
Distrito Federal	153.4 (106.7;206.6)	14.6 (10.2;19.6)	375.8 (263;514.2)	11.5 (8.1;15.6)	-21.5(-31.6;-10.1)
Espírito Santo	267.2 (187.3;364.6)	14.2 (9.8;19)	517.4 (366;694.2)	11.3 (8;15.1)	-20.3(-30.2;-9.5)
Goiás	390.3 (275.4;527.4)	13.3 (9.5;17.9)	825.7 (573.1;1116.1)	10.7 (7.5;14.4)	-19.8(-29.7;-8.2)
Maranhão	428.5 (295.9;580.8)	14 (9.6;18.9)	839.1 (587.1;1128.2)	11.6 (8.2;15.6)	-16.6(-27.6;-4.6)
Mato Grosso	164.7 (113.5;223.8)	13.1 (9;17.8)	417.3 (290.3;562.3)	10.8 (7.6;14.5)	-17.2(-27.2;-6.4)
Mato Grosso do Sul	169.1 (116.8;228.9)	13.5 (9.3;18.1)	352.9 (245.4;473.9)	11.1 (7.7;14.9)	-17.5(-27.2;-5.8)
Minas Gerais	1738 (1220.8;2333.7)	14.3 (10.1;19.2)	2955 (2086.3;3961.8)	11.3 (8;15.1)	-21.2(-31.6;-10.1)
Pará	388.8 (271.9;523.7)	13.4 (9.3;17.9)	911 (642.8;1240.9)	11 (7.7;14.9)	-18.2(-28.5;-6.6)
Paraíba	344.1 (240.1;462.5)	14.5 (10.1;19.4)	544.1 (387.2;728.3)	11.6 (8.2;15.5)	-20.1(-30.1;-9.6)
Paraná	897 (622.7;1212.7)	13.9 (9.7;18.6)	1532.3 (1056.3;2071.6)	11.2 (7.8;15.3)	-19.2(-29.7;-8.6)
Pernambuco	695.5 (480.5;944.5)	13.3 (9.3;18)	1176.4 (816.4;1591.5)	11 (7.7;14.9)	-17.1(-27.7;-4.5)
Piaui	242.4 (170.3;330.5)	14.3 (9.9;19.5)	441.9 (307.3;600.4)	11.6 (8.1;15.8)	-18.5(-28.9;-7.2)
Rio de Janeiro	1648.3 (1148.6;2261.6)	14.2 (10;19.5)	2446.9 (1721.6;3330.8)	11.1 (7.8;15.2)	-21.8(-32.2;-9.4)
Rio Grande do Norte	237.7 (168.4;319)	13.7 (9.6;18.6)	450.4 (318.6;614)	11.2 (7.9;15.3)	-18.3(-28.5;-6.6)
Rio Grande do Sul	1102.7 (770.5;1503.8)	13.8 (9.7;18.8)	1625.7 (1140.6;2203.7)	11.1 (7.8;15)	-19.7(-29.5;-7.9)
Rondônia	84.2 (57.8;114.5)	12.8 (8.9;17.2)	196.6 (139.1;265.5)	10.7 (7.5;14.2)	-16.4(-26.8;-4.3)
Roraima	14.2 (9.9;19.4)	12.3 (8.7;16.6)	52.3 (36.6;71.7)	10.3 (7.3;14)	-16.3(-26.3;-5.7)
Santa Catarina	477.1 (333.1;650)	14 (9.9;18.9)	953.6 (666.5;1286.3)	11.1 (7.7;14.8)	-21.1(-30.6;-9.4)
São Paulo	3627.6 (2524.6;4868.5)	14 (9.7;18.8)	6265.3 (4404.9;8442.8)	11.3 (8;15.2)	-19.2(-29.3;-7.5)
Sergipe	130.4 (92.3;177)	13.5 (9.5;18.6)	275.1 (193.3;373.7)	11.1 (7.8;15)	-18.1(-28.4;-6.7)
Tocantins	77.8 (54.6;106.2)	13.6 (9.5;18.5)	181.2 (127.9;246.5)	11.3 (8;15.3)	-16.9(-26.7;-6.3)

*Source: Data derived from Global Burden of Disease Study 2019, Institute for Health Metrics and Evaluation, University of Washington.^*46*^*

**Table 2-10:** – Number of YLDs and age-standardized YLD rates (per 100 000) due to stroke, ischemic stroke, subarachnoid hemorrhage, and intracerebral hemorrhage in 1990 and 2019, and percent change of rates, in Brazil, according to age group.

Cause of death and age group	1990		2019		Percent change (95% UI)
	Number (95% UI)	Rate (95% UI)	Number (95% UI)	Rate (95% UI)	
**B.2.3-Stroke**					
Under 5	698.9 (446.2;1034.8)	4.1 (2.6;6.1)	605.5 (398;883.7)	3.9 (2.6;5.7)	-5.3 (-10.4;1)
15-49 years	53038.7 (38137.5;68539.1)	69.2 (49.8;89.4)	67619.4 (48183.5;87875.3)	58.6 (41.7;76.1)	-15.4 (-18.6;-12.1)
50-69 years	70829.2 (51187.2;90461.9)	451.5 (326.3;576.6)	111713.8 (80775.1;143720.1)	276.9 (200.2;356.2)	-38.7 (-40.9;-36.1)
5-14 years	5159.6 (3356.4;7554.5)	14.6 (9.5;21.4)	4425.6 (2954.9;6303)	13.7 (9.2;19.5)	-6 (-12.1;1.5)
70+ years	45920.6 (32727.2;59923)	1085.6 (773.7;1416.6)	99192.9 (70400.8;129782.2)	757.9 (537.9;991.6)	-30.2 (-33.5;-26.8)
Age-standardized	175647 (130408.3;222000.4)	180.4 (133.2;228.8)	283557.2 (208451.8;357622.1)	120.9 (88.7;152.6)	-33 (-34.8;-31.1)
All Ages	175647 (130408.3;222000.4)	118 (87.6;149.2)	283557.2 (208451.8;357622.1)	130.9 (96.2;165.1)	10.9 (7.7;14.4)
**B.2.3.1-Ischemic stroke**					
Under 5	575 (352.9;900.4)	3.4 (2.1;5.3)	484.4 (305.1;735.3)	3.1 (2;4.7)	-7.9 (-13.8;-0.3)
15-49 years	27491.8 (18751.4;37159.1)	35.9 (24.5;48.5)	36232.1 (24894.2;49392.8)	31.4 (21.6;42.8)	-12.5 (-17.6;-7.1)
50-69 years	44745.5 (31538.3;58754.1)	285.2 (201;374.5)	70202.8 (49237;92286.4)	174 (122;228.8)	-39 (-42.1;-35.7)
5-14 years	3772.3 (2249;5772.3)	10.7 (6.4;16.3)	3123.3 (1924.8;4738.2)	9.7 (6;14.7)	-9.3 (-17.3;0.4)
70+ years	37445.3 (26156.1;50128.5)	885.2 (618.3;1185.1)	78431.8 (55156.5;105572.2)	599.2 (421.4;806.6)	-32.3 (-35.9;-28.5)
Age-standardized	114029.9 (82236.3;147025)	122.2 (88;156.8)	188474.4 (134840.6;241680.6)	81.2 (58.3;104)	-33.5 (-35.9;-31)
All Ages	114029.9 (82236.3;147025)	76.6 (55.3;98.8)	188474.4 (134840.6;241680.6)	87 (62.2;111.5)	13.5 (9.2;18.1)
**B.2.3.2-Intracerebral hemorrhage**					
Under 5	101.8 (64.4;149.7)	0.6 (0.4;0.9)	99.8 (64.8;145.8)	0.6 (0.4;0.9)	7.1 (1.6;13.9)
15-49 years	17744.7 (12329.9;23735.5)	23.2 (16.1;31)	20847.3 (14380.3;27914.4)	18.1 (12.5;24.2)	-22 (-27.3;-16.4)
50-69 years	19893.5 (14366.8;26543.6)	126.8 (91.6;169.2)	28759.2 (20440;38067.7)	71.3 (50.7;94.4)	-43.8 (-47.2;-39.7)
5-14 years	1143.1 (714.8;1658.3)	3.2 (2;4.7)	1072.9 (668.4;1551.9)	3.3 (2.1;4.8)	2.8 (-3.2;11.1)
70+ years	7297.4 (5111.2;9866)	172.5 (120.8;233.2)	16923.3 (11862;22603.3)	129.3 (90.6;172.7)	-25 (-31.1;-17.9)
Age-standardized	46180.6 (33564.1;59463.8)	44.3 (32.3;56.6)	67702.4 (49062.3;87760.6)	28.5 (20.7;36.9)	-35.7 (-38.8;-32.2)
All Ages	46180.6 (33564.1;59463.8)	31 (22.6;40)	67702.4 (49062.3;87760.6)	31.2 (22.6;40.5)	0.7 (-4.8;6.3)
**B.2.3.3-Subarachnoid hemorrhage**					
Under 5	22 (11.8;34.9)	0.1 (0.1;0.2)	21.2 (11.4;33.7)	0.1 (0.1;0.2)	5.3 (2.2;9.7)
15-49 years	7802.2 (5194.1;10669.3)	10.2 (6.8;13.9)	10540.1 (7231.3;14584)	9.1 (6.3;12.6)	-10.3 (-16.3;-4.4)
50-69 years	6190.3 (4238.1;8554.9)	39.5 (27;54.5)	12751.8 (8762.5;17660.6)	31.6 (21.7;43.8)	-19.9 (-26.1;-13.3)
5-14 years	244.2 (137.9;385.3)	0.7 (0.4;1.1)	229.4 (133.9;356.3)	0.7 (0.4;1.1)	2.9 (0.1;6.9)
70+ years	1177.9 (762.8;1668.6)	27.8 (18;39.4)	3837.8 (2504.8;5430.3)	29.3 (19.1;41.5)	5.3 (-4.4;17.1)
Age-standardized	15436.6 (10801.8;20590.3)	13.9 (9.7;18.6)	27380.3 (19490.9;36579.7)	11.2 (7.9;15)	-19.5 (-23.7;-15.4)
All Ages	15436.6 (10801.8;20590.3)	10.4 (7.3;13.8)	27380.3 (19490.9;36579.7)	12.6 (9;16.9)	21.8 (14.2;29.3)

*Source: Data derived from Global Burden of Disease Study 2019, Institute for Health Metrics and Evaluation, University of Washington.^*46*^*

**Table 2-11:** – Number of DALYs and age-standardized DALY rates (per 100 000) due to stroke, ischemic stroke, subarachnoid hemorrhage, and intracerebral hemorrhage in 1990 and 2019, and percent change of rates, in Brazil and its Federative Units.

Cause of death and location	1990		2019		Percent change (95% UI)
	Number (95% UI)	Rate (95% UI)	Number (95% UI)	Rate (95% UI)	
**B.2.3-Stroke**					
Acre	3953.9 (3619.8;4276.6)	2252.5 (2074;2424.4)	8348.6 (7568.7;9153.4)	1310.9 (1188.3;1433.8)	-41.8(-47.8;-34.9)
Alagoas	47867.3 (43901.6;52819.4)	3342.5 (3046.7;3689.9)	58761.4 (51951.5;66173.7)	1822.9 (1616.1;2048.1)	-45.5(-53.6;-36.4)
Amapá	2062.9 (1882.6;2227.6)	1902.2 (1747.3;2044.2)	6967.3 (6287.8;7620.7)	1264.2 (1135.5;1381.4)	-33.5(-39.9;-26.7)
Amazonas	20109.3 (18325.7;21820.6)	2433.1 (2221.2;2622.4)	34973.2 (31325.3;39025.7)	1179.7 (1054.5;1317.2)	-51.5(-56.6;-45.4)
Bahia	186084.1 (166927.6;207069.4)	2605 (2332.8;2895.8)	226991.9 (194303.9;262385)	1402.4 (1202.3;1619.2)	-46.2(-55.2;-35.4)
Brazil	2766491.4 (2670978.2;2865452.2)	2959 (2829.6;3063)	2861723.2 (2683069.9;3012805.9)	1219.6 (1142;1285.5)	-58.8(-61;-56.8)
Ceará	88959.4 (77643.6;101337.9)	2068.8 (1789.3;2379.4)	134906.6 (114270.4;156553.5)	1352.6 (1144.6;1568.4)	-34.6(-47;-19.6)
Distrito Federal	19461.3 (17575.3;21841.7)	3045.6 (2783.3;3373)	26655.1 (23860.8;29674.3)	1095 (979.1;1216.4)	-64(-68.4;-59.2)
Espírito Santo	52398 (50083.8;54649.4)	3436.5 (3269.6;3588.3)	57215.8 (50636.1;63791.6)	1319.3 (1168.5;1468.8)	-61.6(-65.9;-57.3)
Goiás	77252.6 (67347.7;90376.8)	3403 (2985.6;3978.2)	81008.6 (69615.5;93585.6)	1152.6 (989.1;1328.7)	-66.1(-72;-59.4)
Maranhão	77077.8 (65445.9;90039.2)	2667.9 (2263;3100.3)	121736 (103916.8;143735.3)	1821.1 (1552.9;2145.6)	-31.7(-45.2;-13.3)
Mato Grosso	21222.8 (18529.1;23676.4)	2422.1 (2158.6;2674.9)	36897.6 (33242.4;40972.5)	1097.7 (988.4;1220.4)	-54.7(-60.5;-47.8)
Mato Grosso do Sul	28610.2 (26745.4;30493.9)	2938 (2748;3130.6)	34130.9 (30562.2;38068.1)	1158 (1038.9;1289.2)	-60.6(-64.7;-55.8)
Minas Gerais	339733.4 (316523.6;369110.1)	3246.5 (3019;3518.1)	288464.3 (260517.4;320004.4)	1100.1 (993.6;1219.9)	-66.1(-70.2;-62.1)
Pará	63769.5 (56956.4;70739)	2927.8 (2631.3;3237.3)	97258.8 (86061.6;107948.3)	1366.3 (1208.8;1517)	-53.3(-59.1;-46.8)
Paraíba	50969.4 (45276.3;56936.1)	2170 (1923.3;2422.3)	59408.3 (52413.8;67229.9)	1257.2 (1110.2;1421.9)	-42.1(-50.7;-31.3)
Paraná	176843.7 (168958;184595.9)	3564.4 (3392.5;3730.9)	163286.9 (145233.7;181362.6)	1249.4 (1109.3;1379.5)	-64.9(-68.8;-61)
Pernambuco	137205.1 (129269;145238.6)	2955.7 (2776.9;3127.6)	150292.6 (134781.7;166740.6)	1493.6 (1341;1658)	-49.5(-55;-43.8)
Piaui	41180.3 (37581.7;45051.1)	2844.7 (2600.7;3108.1)	50804.6 (45057.7;56665.2)	1347.6 (1196.3;1503.9)	-52.6(-58.5;-45.8)
Rio de Janeiro	377537 (359753.7;393981.6)	3749.7 (3570.9;3913.2)	285663.5 (256426.8;314757.9)	1288.3 (1157.8;1419.9)	-65.6(-68.9;-62)
Rio Grande do Norte	30686.3 (27616.3;34007.4)	1866 (1666.5;2076.7)	37002.4 (31613.2;43051.1)	950.9 (813.7;1105.4)	-49(-57.4;-39.5)
Rio Grande do Sul	190752.5 (181635.6;200277.2)	2867.6 (2718;3009.7)	181831.5 (162001.9;201216.5)	1190.7 (1061.7;1314.9)	-58.5(-62.4;-54)
Rondônia	12523.1 (10831.5;13950.3)	3595 (3267.5;3918.2)	19404.8 (16963.8;22202.1)	1247 (1091;1418.6)	-65.3(-70.3;-59.4)
Roraima	1690 (1492.5;1869)	2667.4 (2430.1;2899.2)	4362.7 (3925.8;4818.5)	1151.5 (1032.8;1263.9)	-56.8(-61.8;-51.5)
Santa Catarina	79849.1 (75128.2;84605.6)	3134.4 (2945.7;3316.1)	79975 (71270.1;89055)	1009.6 (897.6;1124.2)	-67.8(-71.1;-64.3)
São Paulo	604794.8 (569838.8;640063.1)	2851.4 (2681.9;3015.5)	563721.2 (507325.6;621571.8)	1049.5 (946.3;1153.5)	-63.2(-66.7;-59.5)
Sergipe	22612.3 (20686.7;24655.8)	2826.1 (2577.3;3076.2)	31067.9 (26888.4;35427)	1376.1 (1192.8;1570.2)	-51.3(-58.5;-43.8)
Tocantins	11285.4 (9918;12704.6)	2724.8 (2408.6;3041.8)	20585.9 (17987.9;23457.2)	1427.5 (1246.1;1620)	-47.6(-55.8;-37.6)
**B.2.3.1-Ischemic stroke**					(;)
Acre	1445 (1296.1;1599.1)	1090.2 (977.5;1196.9)	3329.6 (2969.8;3695.2)	613.7 (545.3;681.5)	-43.7(-50.8;-34.4)
Alagoas	20021.2 (17255.8;22924.7)	1614.9 (1401.1;1835.8)	26131.5 (22660;29606.5)	869.8 (753.3;987.1)	-46.1(-56.3;-34.9)
Amapá	787.9 (717;856.8)	934.1 (846;1013.2)	2756.8 (2429.7;3053.3)	603 (528.6;667)	-35.4(-41.7;-28.9)
Amazonas	7502.6 (6829.2;8203.1)	1149.3 (1045.3;1247.4)	14074.1 (12487.8;15775.3)	546.2 (483;611.7)	-52.5(-57.5;-46.9)
Bahia	73495.4 (63810.7;83067.2)	1155.9 (1004.3;1304.2)	98253.4 (83330.9;113726.8)	624.2 (529.5;723.8)	-46(-56.1;-33.2)
Brazil	1061065 (999618.4;1116287.5)	1333.3 (1244.5;1403.6)	1268106.5 (1157551.8;1356041.6)	561 (510.4;599.8)	-57.9(-61;-55)
Ceará	36622.7 (30420.4;43447.9)	931.9 (774.3;1101.8)	61215.8 (51448.4;71614.2)	629.2 (529.2;737.1)	-32.5(-46.6;-13.1)
Distrito Federal	5537 (4963.6;6234.1)	1415.6 (1290.1;1562.9)	11151.2 (9927.9;12430.9)	569.1 (500.4;632.3)	-59.8(-64.4;-54.7)
Espírito Santo	19982.1 (18630.5;21190.4)	1574.6 (1462.7;1670.2)	24655.8 (21593;27722.9)	602.5 (526.3;677)	-61.7(-66.2;-57.1)
Goiás	25866.3 (22357.1;30295.8)	1451.8 (1263.5;1680.1)	33225.9 (28498.2;38313.9)	515.9 (443.4;592.1)	-64.5(-70.3;-57.5)
Maranhão	28972.4 (22485.8;34775.6)	1203.5 (957.9;1438)	53315.2 (45693.2;61949.2)	841.3 (720.3;979.7)	-30.1(-45.6;-6)
Mato Grosso	7757.1 (6804.9;8655.7)	1175.3 (1037.2;1298.1)	15225.4 (13520.5;17052.5)	510.5 (451;571.7)	-56.6(-62;-50.3)
Mato Grosso do Sul	9617.3 (8855.7;10378.2)	1251.4 (1148.9;1348.5)	14227.2 (12650.7;15990)	517.7 (459.4;579.7)	-58.6(-63.1;-53.4)
Minas Gerais	121530.5 (111693.4;132739.1)	1381.5 (1265.7;1498.9)	125719.7 (111263.1;139550.3)	483.1 (427.3;536.1)	-65(-68.9;-60.8)
Pará	26583 (23751.3;29461.2)	1475.7 (1316.4;1624.1)	43317.3 (38006;48704.4)	674.6 (589;758.1)	-54.3(-60.1;-47.8)
Paraíba	22818 (19197.4;26497.7)	1008 (850.9;1164.9)	26234.7 (22655.5;29851.3)	547.8 (474;624.8)	-45.7(-55.8;-32.7)
Paraná	71620.1 (67166.7;76010)	1740.7 (1623.4;1845)	81143.3 (71177.3;90509.3)	649 (570;721.8)	-62.7(-66.7;-58.4)
Pernambuco	56079.4 (50916.4;61089.4)	1344.9 (1218.5;1461.6)	61765.2 (54385;69585)	649.5 (572.4;731.4)	-51.7(-58.2;-44.8)
Piaui	16603 (14591;18612.8)	1356.2 (1194.6;1513.5)	23795.6 (20662.8;26814)	632 (550.7;712.3)	-53.4(-60.4;-45.4)
Rio de Janeiro	135992.8 (127706.6;144128.1)	1580.7 (1479.5;1669.4)	119754.3 (106880.1;131983.3)	545.1 (484.7;600.2)	-65.5(-68.8;-62)
Rio Grande do Norte	13717.9 (11997.9;15557.9)	881.2 (767.8;996.2)	16848.6 (14243.9;19722)	438.8 (371;513.3)	-50.2(-58.5;-39)
Rio Grande do Sul	80633.6 (75289.1;85992.1)	1401.7 (1302.2;1490)	91439.3 (80768.7;101588.9)	595.8 (526.8;661.3)	-57.5(-61.5;-53.1)
Rondônia	4299.9 (3810.6;4771.5)	1918.2 (1743.4;2081.6)	8241.2 (7143.1;9486.9)	601.6 (518.8;692.2)	-68.6(-73.1;-63.5)
Roraima	550 (496.1;608.2)	1346.9 (1226.1;1465.1)	1750.4 (1551.2;1947.6)	577.2 (511.8;639.3)	-57.1(-62.2;-51.7)
Santa Catarina	32468.4 (30272.1;34691.7)	1547.6 (1440;1650.7)	38577.2 (33969.3;43198.7)	520.5 (460.4;581.1)	-66.4(-70;-62.6)
São Paulo	226823.4 (210500.4;242557.8)	1290.9 (1189;1375.2)	249002.3 (221220;276006.2)	479 (424.5;530.4)	-62.9(-66.2;-59.1)
Sergipe	9762.5 (8615;10829.7)	1413.3 (1257.8;1555.8)	13877 (11998.2;15919.3)	656.3 (566.1;753.4)	-53.6(-60.8;-45.2)
Tocantins	3975.5 (3418.4;4557.8)	1322.2 (1158.6;1492.4)	9078.6 (7754.4;10411.6)	680.1 (579.8;780.2)	-48.6(-57.9;-38.2)
**B.2.3.2-Intracerebral hemorrhage**					(;)
Acre	1839 (1657.2;2040.1)	914.2 (827.4;1008.7)	3542.8 (3164.8;3962.9)	512.3 (459.6;572)	-44(-51.5;-34.8)
Alagoas	21422.3 (18719.7;24577.7)	1385.5 (1204.8;1595.7)	24575.6 (21311.8;27913)	728.1 (634.3;828.1)	-47.4(-58.2;-35.7)
Amapá	975.4 (873.5;1064.7)	789.3 (718.9;857.2)	3023.1 (2676.9;3387.4)	497.1 (440;554.1)	-37(-44.2;-28.4)
Amazonas	10062.1 (9057.6;11050.1)	1082.4 (983.6;1184.9)	15315.2 (13500.3;17329.6)	481.2 (426.5;545.3)	-55.5(-61.2;-48.8)
Bahia	87938.6 (77288.1;99639.1)	1171.2 (1030.7;1328.1)	95088 (79241.5;112242.3)	578.9 (483.3;682.9)	-50.6(-60.7;-38.7)
Brazil	1349736.5 (1296807.8;1423036.1)	1327.8 (1274;1397.3)	1154165.4 (1091357.5;1217146.3)	477.6 (450.9;503.8)	-64(-66.6;-61.6)
Ceará	39918.9 (33988.8;46866.7)	893.1 (750.3;1054.3)	54532.7 (45115.4;64606.6)	538.5 (446.4;637.3)	-39.7(-53.7;-23.3)
Distrito Federal	10416.4 (9283.2;12008.7)	1319.7 (1186.3;1497.7)	10477.5 (9230.5;11872.3)	368.5 (324;415.9)	-72.1(-75.8;-67.3)
Espírito Santo	25812.1 (24512.2;27292.3)	1537 (1456.4;1620.6)	23740.7 (20717.1;26779.4)	523.9 (458.6;590.2)	-65.9(-70.3;-61.4)
Goiás	40984.5 (35216.1;48923.1)	1619.8 (1399.7;1933.9)	34554.4 (29125.6;40074.6)	465.6 (393.3;540.9)	-71.3(-76.9;-64.6)
Maranhão	35790.2 (28841.3;44237.6)	1143.2 (915.9;1417.9)	49711.7 (41406;59969.3)	725.6 (604;877)	-36.5(-51.4;-15.9)
Mato Grosso	10195.6 (8768.9;11594.7)	1009.1 (882.3;1135.1)	15221.9 (13452.7;17149.6)	421.3 (373.4;474.5)	-58.3(-64.9;-50.3)
Mato Grosso do Sul	15024.7 (13886.9;16246.9)	1391 (1290.8;1500.3)	14338 (12701.5;16270.4)	465.1 (412.8;527.4)	-66.6(-70.8;-61.7)
Minas Gerais	172794.2 (158931.3;194463.9)	1520.8 (1400.4;1698.4)	115640 (103297.7;129456.7)	435.7 (389;487.8)	-71.4(-75.6;-67.2)
Pará	29854.6 (26395.2;33517.5)	1225.7 (1088;1368.5)	39347.5 (34472;44066.4)	519.2 (455.8;581.9)	-57.6(-64;-50.5)
Paraíba	20767.9 (17738.6;24596.1)	870.4 (742.4;1026.4)	24329.8 (21197.1;27942.4)	520.5 (453.2;597.8)	-40.2(-52.6;-25.2)
Paraná	84543 (79771.8;89664)	1517 (1432.2;1613.9)	58629.2 (51418.8;66053.2)	428.1 (376.1;481.6)	-71.8(-75.5;-67.8)
Pernambuco	67425.3 (62725.9;72662.2)	1367.7 (1271.3;1473.7)	67025.9 (59208.3;75471.8)	643.1 (568.2;724)	-53(-59.3;-46)
Piaui	18830.6 (16752.3;21434.6)	1189.1 (1056.4;1349.7)	19788.3 (17389.9;22475.7)	525.8 (463.1;597.1)	-55.8(-62.9;-47.4)
Rio de Janeiro	195616.9 (184503.4;208076.4)	1791.7 (1692.6;1901.7)	124136.4 (110522.6;139387.9)	550.3 (490.7;617.8)	-69.3(-72.7;-65.2)
Rio Grande do Norte	13368.5 (11836.1;15038.3)	794.2 (700.4;893.9)	14784.6 (12178.2;17578.3)	377.7 (311.1;448.6)	-52.4(-61.8;-41.9)
Rio Grande do Sul	90021.7 (84641.8;95298.5)	1224.3 (1151.1;1296.8)	68288.3 (59641.3;76831.2)	442.9 (386.3;499.9)	-63.8(-68.2;-59)
Rondônia	6492.8 (5543.7;7393)	1423.5 (1256.5;1588.5)	7939.9 (6835.3;9291.1)	471.6 (406.7;549.8)	-66.9(-72.6;-59.1)
Roraima	878.3 (761.3;987.6)	1105.3 (988.9;1218.4)	1841.7 (1630.9;2065.2)	424.1 (375.7;473.5)	-61.6(-67;-55.7)
Santa Catarina	38007.8 (35410.7;40721.5)	1323.1 (1232.3;1419.8)	29457 (25702.3;33414.7)	349.4 (304.4;395.8)	-73.6(-76.8;-70)
São Paulo	294848.3 (273533.7;320151.5)	1260.5 (1173.1;1353.1)	217553.4 (194543.2;243976.7)	393.1 (351.9;440.7)	-68.8(-72.7;-64.7)
Sergipe	10294.6 (9239.9;11405.8)	1172.3 (1047.6;1298.5)	12930.8 (11015.5;15067.4)	549.5 (468.4;639.3)	-53.1(-61.2;-44.1)
Tocantins	5612.4 (4778.8;6561.7)	1138.4 (966.8;1324.8)	8351.1 (7162.9;9670.2)	553.5 (477.1;640.4)	-51.4(-61.4;-39.4)
**B.2.3.3-Subarachnoid hemorrhage**					(;)
Acre	669.8 (588.4;766.7)	248.1 (218.2;282)	1476.2 (1313.2;1664.4)	184.8 (165;208.1)	-25.5(-37.1;-11.4)
Alagoas	6423.8 (5403.3;7533.8)	342.2 (288;399.9)	8054.3 (6938.2;9342.8)	225 (193.7;260.5)	-34.3(-47.9;-16.6)
Amapá	299.6 (258.9;343.2)	178.9 (158.7;206.4)	1187.4 (1062.8;1353.1)	164.1 (146.3;186.4)	-8.3(-20.6;7.2)
Amazonas	2544.6 (2247;2874)	201.4 (179;228.9)	5583.9 (4913.9;6445.9)	152.3 (134.3;175.8)	-24.4(-36;-10.6)
Bahia	24650.1 (21393.8;28224.4)	278 (241.4;320.6)	33650.5 (27808.9;40181.7)	199.3 (165;237.9)	-28.3(-43.4;-9.5)
Brazil	355689.9 (317037;373740.1)	297.9 (267.5;312.7)	439451.3 (411002.3;468439.5)	181 (169.4;192.8)	-39.2(-43.8;-31.8)
Ceará	12417.9 (10343.3;14787.4)	243.8 (204.2;287.9)	19158.1 (15794.9;23207.8)	184.9 (153.1;223.6)	-24.2(-40.5;0.1)
Distrito Federal	3507.9 (3110;3938.7)	310.3 (274.7;347.1)	5026.3 (4331.4;5790)	157.4 (135.3;179.7)	-49.3(-56.7;-39.9)
Espírito Santo	6603.8 (5412.6;7189)	324.9 (268.7;352.5)	8819.3 (7521.5;10292.6)	192.9 (164.8;224.2)	-40.6(-49.6;-29)
Goiás	10401.8 (9100.6;12049.3)	331.4 (289;383.9)	13228.3 (11078.7;15754.8)	171.2 (143.6;202.2)	-48.3(-58.3;-36.4)
Maranhão	12315.2 (9170.6;16162.5)	321.2 (242.9;414.9)	18709.1 (15184.3;22615.2)	254.1 (205.3;308.8)	-20.9(-42.8;8.5)
Mato Grosso	3270 (2710.8;3823.3)	237.6 (202.4;274.7)	6450.3 (5684.7;7409.4)	165.9 (146.5;189.9)	-30.2(-42;-14.8)
Mato Grosso do Sul	3968.3 (3590;4343.7)	295.6 (268.3;323.2)	5565.7 (4859.9;6325)	175.2 (153.2;199)	-40.7(-48.8;-31.2)
Minas Gerais	45408.7 (39218.4;49588.4)	344.1 (298.9;375.4)	47104.6 (41067.1;53672.3)	181.2 (158.9;206)	-47.3(-54.1;-38.5)
Pará	7331.9 (6385;8336)	226.4 (197;258.7)	14594 (12863.3;16610.3)	172.5 (152.2;196.2)	-23.8(-36.1;-8.5)
Paraíba	7383.6 (6372.6;8509.5)	291.7 (250.6;337.7)	8843.8 (7549.8;10263.5)	188.9 (161.6;219.4)	-35.2(-48.3;-19.5)
Paraná	20680.6 (18344.4;22387.3)	306.7 (272.9;330.9)	23514.4 (20164.4;27053.9)	172.4 (148.7;197.6)	-43.8(-51.9;-35.2)
Pernambuco	13700.4 (12356.5;15479.4)	243 (218.8;276.6)	21501.4 (18756.1;24644.5)	201.1 (176;229.8)	-17.2(-30.4;-2.2)
Piaui	5746.7 (4929.9;6600.1)	299.4 (259.7;340.8)	7220.7 (6236.6;8335)	189.8 (164;218.6)	-36.6(-48.3;-21.8)
Rio de Janeiro	45927.3 (35485.7;50624.3)	377.3 (297.1;415.1)	41772.9 (36465.3;47362.4)	192.9 (169.3;218.2)	-48.9(-56.4;-34.3)
Rio Grande do Norte	3599.9 (3104.5;4304.3)	190.6 (163.1;232.6)	5369.2 (4435.8;6786.1)	134.4 (111.1;169.7)	-29.5(-43.4;-11.8)
Rio Grande do Sul	20097.2 (18365.6;21848)	241.6 (221.4;263.4)	22103.9 (19284.4;25482.8)	152.1 (132.9;174.4)	-37.1(-45.4;-27.1)
Rondônia	1730.4 (1389.1;2030.2)	253.4 (212.8;290.4)	3223.7 (2788.9;3765.4)	173.8 (150.7;202.1)	-31.4(-43.6;-13.7)
Roraima	261.7 (217.1;304.3)	215.2 (185.3;246.4)	770.6 (680.9;905.9)	150.3 (133.5;176.3)	-30.2(-41;-16.4)
Santa Catarina	9372.9 (8539.8;10192.6)	263.7 (240.5;287.5)	11940.7 (10397.6;13772.2)	139.7 (122.3;160.2)	-47(-54;-37.9)
São Paulo	83123 (72075.6;90420.8)	300 (263.7;326.2)	97165.6 (85347.2;110658.3)	177.4 (156.3;200.9)	-40.9(-48.5;-31.1)
Sergipe	2555.2 (2239.9;2912.1)	240.5 (210.7;275.3)	4260.2 (3562.3;5058.4)	170.4 (142.7;202)	-29.2(-43.1;-12.2)
Tocantins	1697.4 (1407;1987.1)	264.2 (222.3;307.1)	3156.2 (2681.1;3702.9)	193.9 (165.3;227.7)	-26.6(-40.7;-8.3)

*Source: Data derived from Global Burden of Disease Study 2019, Institute for Health Metrics and Evaluation, University of Washington.^*46*^*

**Table 2-12:** – Number of DALYs and age-standardized DALY rates (per 100 000) due to stroke, ischemic stroke, subarachnoid hemorrhage, and intracerebral hemorrhage in 1990 and 2019, and percent change of rates, in Brazil, according to age group.

Cause of death and age group	1990		2019		Percent change (95% UI)
	Number (95% UI)	Rate (95% UI)	Number (95% UI)	Rate (95% UI)	
**B.2.3-Stroke**					
Under 5	37072.3 (27007.4;50450.1)	218.9 (159.4;297.8)	6575.8 (4847.9;8823.2)	42.4 (31.3;56.9)	-80.6 (-88.1;-68.6)
15-49 years	769115.6 (738502.6;803387.2)	1003.5 (963.5;1048.2)	516175.3 (483925.6;549638.4)	446.9 (419;475.9)	-55.5 (-58.2;-52.7)
50-69 years	1154968.9 (1110879.7;1201696.6)	7362.3 (7081.2;7660.1)	1179642.5 (1118241.2;1239653.5)	2924 (2771.8;3072.8)	-60.3 (-62.8;-57.9)
5-14 years	24536.1 (21707.5;27385.7)	69.4 (61.4;77.5)	13756 (11528.4;16101.8)	42.7 (35.7;49.9)	-38.6 (-46;-30.7)
70+ years	780798.4 (727457.3;814613.1)	18458.3 (17197.3;19257.7)	1145573.7 (1032837.8;1220898)	8752.5 (7891.2;9328)	-52.6 (-55.8;-49.7)
Age-standardized	2766491.4 (2670978.2;2865452.2)	2959 (2829.6;3063)	2861723.2 (2683069.9;3012805.9)	1219.6 (1142;1285.5)	-58.8 (-61;-56.8)
All Ages	2766491.4 (2670978.2;2865452.2)	1858.8 (1794.6;1925.2)	2861723.2 (2683069.9;3012805.9)	1320.8 (1238.4;1390.5)	-28.9 (-32.9;-25.3)
**B.2.3.1-Ischemic stroke**					
Under 5	4710.2 (3531.7;6087.8)	27.8 (20.9;35.9)	868.8 (655.6;1164.7)	5.6 (4.2;7.5)	-79.8 (-86.2;-70.6)
15-49 years	113665.5 (102596.5;125599)	148.3 (133.9;163.9)	79615.6 (67096;93825.9)	68.9 (58.1;81.2)	-53.5 (-58.8;-47.3)
50-69 years	393774.7 (369506.2;418040.5)	2510.1 (2355.4;2664.8)	369798 (343143.9;399308)	916.6 (850.6;989.8)	-63.5 (-66.5;-59.9)
5-14 years	5392.6 (3886.2;7362.5)	15.3 (11;20.8)	3563.5 (2394.7;5201.8)	11.1 (7.4;16.1)	-27.6 (-35.6;-20.1)
70+ years	543521.9 (503524.5;570910.5)	12849 (11903.5;13496.5)	814260.7 (727889.1;874704.1)	6221.2 (5561.3;6683)	-51.6 (-55.1;-48.3)
Age-standardized	1061065 (999618.4;1116287.5)	1333.3 (1244.5;1403.6)	1268106.5 (1157551.8;1356041.6)	561 (510.4;599.8)	-57.9 (-61;-55)
All Ages	1061065 (999618.4;1116287.5)	712.9 (671.6;750)	1268106.5 (1157551.8;1356041.6)	585.3 (534.3;625.9)	-17.9 (-24.2;-11.9)
**B.2.3.2-Intracerebral hemorrhage**					
Under 5	17337.1 (11773.4;25176.4)	102.4 (69.5;148.6)	1830.6 (1306.1;2522.5)	11.8 (8.4;16.3)	-88.5 (-93.4;-80)
15-49 years	454266.8 (431721.6;491490.4)	592.7 (563.3;641.2)	259204.3 (240714.6;276923.9)	224.4 (208.4;239.8)	-62.1 (-66.6;-58.7)
50-69 years	647660.7 (618581.9;681800.7)	4128.5 (3943.1;4346.1)	611298 (579754.5;643680)	1515.2 (1437.1;1595.5)	-63.3 (-65.9;-60.6)
5-14 years	9000.5 (7931.1;10146.9)	25.5 (22.4;28.7)	3859.1 (3235.5;4576.9)	12 (10;14.2)	-53 (-60.8;-44.5)
70+ years	221471.5 (206213.9;234624.6)	5235.6 (4874.9;5546.6)	277973.3 (249008.1;299692.6)	2123.8 (1902.5;2289.7)	-59.4 (-63.2;-55.7)
Age-standardized	1349736.5 (1296807.8;1423036.1)	1327.8 (1274;1397.3)	1154165.4 (1091357.5;1217146.3)	477.6 (450.9;503.8)	-64 (-66.6;-61.6)
All Ages	1349736.5 (1296807.8;1423036.1)	906.9 (871.3;956.1)	1154165.4 (1091357.5;1217146.3)	532.7 (503.7;561.8)	-41.3 (-45.6;-37.2)
**B.2.3.3-Subarachnoid hemorrhage**					
Under 5	15024.9 (9326.8;20352.4)	88.7 (55.1;120.2)	3876.3 (2819.3;5345.7)	25 (18.2;34.5)	-71.8 (-83.1;-44.7)
15-49 years	201183.3 (176381.8;212269.3)	262.5 (230.1;276.9)	177355.4 (166669.5;193612.9)	153.6 (144.3;167.6)	-41.5 (-46.4;-29)
50-69 years	113533.5 (103789.1;120381)	723.7 (661.6;767.4)	198546.5 (182109.3;213801.4)	492.1 (451.4;530)	-32 (-37.5;-24)
5-14 years	10143.1 (8918;11181)	28.7 (25.2;31.6)	6333.4 (5338.3;7382.5)	19.6 (16.6;22.9)	-31.6 (-42.5;-17.8)
70+ years	15805.1 (14294.1;18534.2)	373.6 (337.9;438.2)	53339.7 (44065.9;59439)	407.5 (336.7;454.1)	9.1 (-21.6;25.3)
Age-standardized	355689.9 (317037;373740.1)	297.9 (267.5;312.7)	439451.3 (411002.3;468439.5)	181 (169.4;192.8)	-39.2 (-43.8;-31.8)
All Ages	355689.9 (317037;373740.1)	239 (213;251.1)	439451.3 (411002.3;468439.5)	202.8 (189.7;216.2)	-15.1 (-21.6;-4)

*Source: Data derived from Global Burden of Disease Study 2019, Institute for Health Metrics and Evaluation, University of Washington.^*46*^*

**Chart 2-1:** – Age-standardized prevalence rates of stroke (A), ischemic stroke (B), intracerebral hemorrhage (C), and subarachnoid hemorrhage (D) (per 100 000 inhabitants), 1990-2019. Data derived from Global Burden of Disease Study 2019. ^46^

**Chart 2-2:** – Age-standardized incidence rates of stroke, (A), ischemic stroke (B), intracerebral hemorrhage (C), and subarachnoid hemorrhage (D) (per 100 000 inhabitants), 1990-2019. Data derived from Global Burden of Disease Study 2019. ^46^

**Chart 2-3:** – Age-standardized mortality rates due to stroke (A), ischemic stroke (B), intracerebral hemorrhage (C), and subarachnoid hemorrhage (D) (per 100 000 inhabitants), 1990-2019. Data derived from Global Burden of Disease Study 2019. ^46^

### Prevalence

• Stroke prevalence estimates may differ slightly between studies because each study selects and recruits a sample of participants to represent the target study population (e.g., state, region, or country).

• In a community-based study in Brazil, using a questionnaire applied to 4496 individuals aged over 35 years, living in a deprived neighborhood in the city of São Paulo in 2011, Abe *et al* . found 243 individuals initially screened positive for stroke. The age-adjusted prevalence rate for men was 4.6% (95% CI, 3.5-5.7) and, for women, 6.5% (95% CI, 5.5-7.5). ^47^

• Using a screening tool, the Stroke Symptom Questionnaire, Fernandes et al. studied stroke prevalence in the town of Coari, in the Brazilian Amazon Basin, and compared stroke prevalence in riverside inhabitants to that in the urban population of the same municipality. Out of 4897 respondents in the urban area and 1028 in the rural area, the authors found a 6.3% crude prevalence of stroke in the rural area and 3.7% in the urban area, with differences maintained after sex and age adjustment. ^48^

• Using the WHO Stepwise Approach to Stroke Surveillance, Goulart *et al.* conducted a study to verify stroke mortality and morbidity rates in an area of São Paulo, Brazil. The questionnaire determining stroke prevalence was applied door to door in a family-health-program neighborhood (Step 3). Out of 3577 subjects over the age of 35 years evaluated at home, 244 (6.82%) cases of stroke survivors were identified via the questionnaire validated by a board-certified neurologist. ^49^

• Benseñor *et al.* analyzed a community-based epidemiological survey (PNS - 2013) with a Brazilian representative sample to assess the absolute numbers with respective prevalence rates of stroke and post-stroke disabilities. The authors estimated 2 231 000 strokes and 568 000 stroke cases with severe disabilities. The point prevalence estimates for stroke were 1.6% and 1.4% in men and women, respectively. ^50^

• According to data from the GBD Brazil Group, the age-standardized prevalence rates of IS per 100 000 were 1327.6 (1151.2 to 1516) in 1990 and 870.1 (761.1 to 992.8) in 2019, representing a percent change of -34.5 (-36.7 to -0.3) (Table 2-1 and Chart 2-1). The highest percent change occurred in the state of Rondônia -41.7 (-46.2 to -0.4) and the lowest in the state of Amapá -23.4 (-28.6 to -0.2) (Table 2-1). For adults, the highest percent change was observed among people aged 50-69 years, -39.5 (-42.6 to -0.4) (Table 2-2). ^46^

• The age-standardized prevalence rates of ICH per 100 000 were 507.5 (438.9 to 584.1) in 1990 and 315.9 (275 to 361.4) in 2019, representing a percent change of -37.7 (-40.5 to -0.3) (Table 2-1 and Chart 2-1). For adults, the highest percent change was observed among people aged 50-69 years, -44.8 (-47.4 to -0.4) (Table 2-2). ^46^

• The age-standardized prevalence rates of SAH per 100 000 were 158.6 (131.7 to 192.4) in 1990 and 124.8 (104.2 to 150.1) in 2019, representing a percent change of -21.3 (-24.3 to -0.2) (Chart 2-1 and Table 2-1). For adults, the highest percent change was observed among people aged 50-69 years, -21.1 (-24.4 to -0.2) (Table 2-2). ^46^

### Incidence

#### Stroke Subtypes

• Data from the Joinville community-based study showed that, when comparing different time periods (1995, 2005-2006, 2010-2011, and 2012-2013), the stroke incidence decreased. Over the last 18 years, the overall stroke (e.g., all major stroke types) incidence in Joinville decreased by 37% (95% CI, 32 - 42). ^51^

• The incidence of first ever stroke adjusted to the Brazilian population was 86.6 per 100 000 (95% CI, 80.5 - 93.0) in 2005-2006 and 113.46 per 100 000 (95% CI, 101.5 - 126.8) in 1995. ^52^ The overall incidence, age-adjusted to world population per 100 000 person-years was 143.7 (95% CI, 128.4–160.3) in 1995, fell to 105.4 (95% CI, 98.0–113.2) in 2005–2006, and then to 90.9 (95% CI, 85.1–96.9) in 2012-2013. The age-standardized incidence of first-ever stroke stratified by gender and age also decreased significantly over time. The reduction was 11% greater in men (42%; 95% CI, 35–49) than in women (31%; 95% CI, 23–39), and 16% greater in young people (≤ 44 years: 54%; 95% CI, 41–66) than in older people (>44 years: 38%; 95% CI, 33–43). ^51^

• From 1995 to 2013, the proportion of IS increased by 12%, whereas that of HS decreased by 16%. Meanwhile, the proportion of SAH remained relatively stable, ranging from 7.5% in 1995 to 6% in 2012-2013. The weight of the decrease in age-adjusted stroke incidence was proportionally higher for HS than for IS, whereas that of SAH remained stable. In the last 8 years, the incidences of IS and HS showed significant absolute decreases of 15% (95% CI, 1–28) and 60% (95% CI, 13–86), respectively. Meanwhile, the incidence of SAH showed a 29% nonsignificant absolute decrease (95% CI, 15–92). ^46^

• In the second Matão stroke registry study, all incident stroke events (81 cases) that occurred between August 1, 2015, and July 31, 2016, were registered. The mean age increased by 9%, from 65.2 (95% CI, 62.6–67.8) to 71.0 (95% CI, 68.1–73.8) years. Between 2003-2004 and 2015-2016, the age-adjusted incidence decreased by 39% (IRR 0.61; 95% CI, 0.46–0.79) and mortality by 50% (IRR 0.50; 95% CI, 0.31– 0.94). The 1-year case fatality was 26%; approximately 56% of the patients were functionally independent, while 7% had a recurrent stroke. ^53^ Compared with the results of the previous Matão stroke registry study, ^54^ these outcomes did not differ significantly.

• Data from the GBD Brazil Group show that the age-standardized incidence rates per 100 000 of stroke were 224.6 (201.6 to 251.8) in 1990 and 127 (113.8 to 142.1) in 2019, representing a percent change of -43.5 (-44.7 to -0.4) (Table 2-3 and Chart 2-2). The highest percent change occurred in Distrito Federal, -47.7 (-49.7 to -0.5), and the lowest in the state of Ceará, -30.6 (-33.2 to -0.3) (Table 2-3). The highest percent change was observed among people aged 15-49 years, -38.1 (-40.9 to -0.4) (Table 2-4). ^46^

• The age-standardized incidence rates of IS per 100 000 were 136.6 (115.7 to 163.1) in 1990 and 78.2 (66.1 to 93) in 2019, representing a percent change of -42.7 (-44.3 to -0.4) (Table 2-3 and Chart 2-2). For adults, the highest percent change was observed among people aged 50-69 years, -48 (-49.9 to -0.5) (Table 2-4). ^46^

• The age-standardized incidence rates of ICH per 100 000 were 66 (56.1 to 77.9) in 1990 and 31.6 (26.6 to 37.3) in 2019, representing a percent change of -52.1 (-53.5 to -0.5) (Table 2-3 and Chart 2-2). For adults, the highest percent change was observed among people aged 50-69 years, -53.2 (-55.3 to -0.5) (Table 2-4). ^46^

• The age-standardized incidence rates of SAH per 100 000 were 22.1 (18.6 to 26.3) in 1990 and 17.2 (14.4 to 20.6) in 2019, representing a percent change of -22.2 (-25.1 to -0.2) (Table 2-3 and Chart 2-2). For adults, the highest percent change was observed among people aged 15-49 years, -23.6 (-27.8 to -0.2) (Table 2-4). ^46^

## Mortality

• In the stroke population-based study known as MAPS, between 2003–2004 and 2015–2016, mortality increased by 50% (IRR 0.50; 95% CI, 0.31–0.94). The 1-year case fatality was 26%. Approximately 56% of the patients were functionally independent, while 7% had a recurrent stroke. ^53^ Compared with the results from the first Matão stroke registry study, ^54^ these rates did not differ significantly.

• Results from a study assessing the association between the mortality due to cerebrovascular diseases and the Municipal Human Development Index and extent of supplementary health coverage in the Brazilian Federative Units, between 2004 and 2013, showed that the percentage of supplementary health coverage in Brazil increased in that period and had an inverse relationship with mortality. Additionally, the correlation coefficient between Municipal Human Development Index scores and the mortality rates weighted by ill-defined causes and standardized by age showed and inverse association. Thus, both increased Municipal Human Development Index and health coverages were associated with decreased mortalities. ^37^

• Data from the GBD Brazil Group show that the age-standardized mortality rates from stroke per 100 000 were 137.8 (127.8 to 144) in 1990 and 58.1 (52.6 to 61.8) in 2019, representing a percent change of -57.8 (-60.4 to -0.6) (Table 2-5 and Chart 2-3). The highest percent change occurred in the state of Goiás, -65.9 (-71.8 to -0.6), and the lowest, in the state of Maranhão, -22.7 (-37.2 to 0) (Table 2-5). For adults, the highest percent change was observed among people aged 50-69 years, -61 (-63.6;-0.6) (Table 2-6). ^46^

• The age-standardized mortality rates from IS per 100 000 were 80 (72.5 to 84.3) in 1990 and 33.9 (29.7 to 36.6) in 2019, representing a percent change of -57.6 (-60.9 to -0.5) (Table 2-5 and Chart 2-3). For adults, the highest percent change was observed among people aged 15-49 years, -66.1 (-70.5 to -0.6) (Table 2-6). ^46^

• The age-standardized mortality rates from ICH per 100 000 were 49.8 (47.1 to 52.2) in 1990 and 18.6 (17.3 to 19.8) in 2019, representing a percent change of -62.6 (-65.3 to -0.6) (Table 2-5 and Chart 2-3). For adults, the highest percent change was observed among people aged 50-69 years, -63.1 (-65.7 to -0.6) (Table 2-6). ^46^

• The age-standardized mortality rates from SAH per 100 000 were 8 (7.3 to 8.4) in 1990 and 5.5 (5 to 5.9) in 2019, representing a percent change of -30.3 (-36.2 to -0.2) (Table 2-6 and Chart 2-3). For adults, the highest percent change was observed among people aged 15-49 years, -40.8 (-46 to -0.3) (Table 2-6). ^46^

## Temporal Trends

• The estimates from the GBD 2015 used to analyze the magnitude and trends of mortality rates and DALYs from cerebrovascular disease (ICD-10: I60-I69) in the 27 Brazilian Federative Units between 1990 and 2015, showed that, despite the increase in the absolute number of deaths due to cerebrovascular disease, the proportion of deaths under the age of 70 years was halved between 1990 and 2015. From 1990 to 2015, the risk of death attributable to stroke decreased for both men (-2.41% per year) and women (-2.51% per year). Nevertheless, the annual reduction in mortality rates adjusted for age, for both sexes, slowed between 2005 and 2015 when compared to the previous period of 1990-2005. States in the lower SDI tertile had less significant reductions (-1.23 and -1.84% per year) as compared to those in the middle (-1.94 and - 2.22%) and upper (-2.85 and -2.82%) SDI tertiles for men and women, respectively. In addition, the YLDs decreased among states, but less expressively. ^55^

• A study on age-adjusted stroke mortality trends among adults (30-69 years-old) from Brazilian regions between 1996 and 2011 evaluated the influence of the methods used to correct death rates in the final estimates. Corrections were implemented by reallocation of deaths with non-registered sex or age, redistribution of garbage codes and redistribution of ill-defined causes of deaths, and changed significantly the observed age-adjusted stroke mortality rates in 1996 and 2011, pre- and post-correction, respectively: 1) for men: in 1996, 82.9 and 113.6; and, in 2011, 49.6 and 60.9; and 2) for women: in 1996, 58.2 and 84.4; and, in 2011, 34.7 and 42.3. ^56^

• A study assessing regional differences in mortality transition and using data from the SIM from 1990 to 2012 showed a –48.05% variation in the mortality coefficient for stroke. Most regions showed a reduction in mortality rates: –61.99% in the Southeast, –55.49% in the South, –26.91% in the West-Central, and –20.78% in the North. Only the Northeast showed increased mortality rates (13.77%). ^57^

• In the city of São Paulo, from 1996 to 2011, 77 848 stroke deaths were confirmed with 51.4% of them among persons aged 35–74 years old. In that period, age-adjusted mortality rates from cerebrovascular diseases decreased by 46.6% in men and by 47.8% in women. For men in high-income neighborhoods, the downward trend was constant; in the middle-income area, there was a sharp decline from 1996 to 2000, followed by a slower pace between 2000 and 2011. In the low-income area, the annual percent change was higher between 1996 and 2002, with a mild decline in 2002–2011. For women in high income areas, there was a sharp decline from 1996 to 2003, and a lower decrease in the last half of the period; in the low- and middle-income areas, the decline was constant during all periods. For the full period, both sexes and age group of 35–74 years, the decline in age-adjusted rates were more pronounced among those residing in the wealthiest area as compared to those living in the poorest area. This same pattern, but with a decline in magnitude, was observed in people aged ≥75 years in all areas as compared to other age groups, for both sexes. Additionally, the temporal evolution of the ratios of age-adjusted rates between people aged 35–74 years living in low- and high-income areas was as follows: for men, from 1996 to 1998, the rate ratio was 2.03, and, from 2009 to 2011, 2.34; for women, from 1996 to 1998, the rate ratio was 2.09, and, from 2009 to 2011, 2.58. The trends of the ratios of age-adjusted rates between those areas showed an annual percent change growth of 1.4 (0.5–2.4) for men and 1.1 (0.1–2.0) for women. ^58^

## Global Burden of Cerebrovascular Diseases

### YLL

• Data from the GBD Brazil Group show that the age-standardized YLL rates due to stroke per 100 000 were 2778.6 (2659.5 to 2879.2) in 1990 and 1098.7 (1025.8 to 1153.7) in 2019, representing a percent change of -60.5 (-62.7 to -0.6) (Table 2-7). For adults, the highest percent change was observed among people aged 50-69 years, -61,7 (-64,3 to -0,6) (Table 2-8). ^46^

• The age-standardized YLL rates due to IS per 100 000 were 1211.1 (1133.8 to 1268.9) in 1990 and 479.7 (435.1 to 510.8) in 2019, representing a percent change of -60.4 (-63.5 to -0.6) (Tables 2-7 and 2-8). For adults, the highest percent change was observed among people aged 15-49 years, -66,6 (-71,1 to -0,6) (Table 2-8). ^46^

• The age-standardized YLL rates due to ICH per 100 000 were 1283.5 (1227.7 to 1351) in 1990 and 449.2 (423.3 to 472.5) in 2019, representing a percent change of -65 (-67.6 to -0.6) (Tables 2-7 and 2-8). For adults, the highest percent change was observed among people aged 50-69 years, -63,9 (-66,5 to -0,6) (Table 2-8). ^46^

• The age-standardized YLL rates due to SAH per 100 000 were 284 (253.8 to 297.2) in 1990 and 169.8 (158.3 to 180.8) in 2019, representing a percent change of -40.2 (-44.9 to -0.3) (Tables 2-7 and 2-8). For adults, the highest percent change was observed among people aged 15-49 years, -42,8 (-47,7 to -0,3) (Table 2-8). ^46^

### YLD

• Data from the GBD Brazil Group show that the age-standardized YLD rates due to stroke per 100 000 were 180.4 (133.2 to 228.8) in 1990 and 120.9 (88.7 to 152.6) in 2019, representing a percent change of -33 (-34.8 to -0.3) (Tables 2-9 and 2-10). The highest percent change occurred in the state of Rondônia, -39.5 (-43.8 to -0.4), and the lowest in the state of Amapá, -23 (-27.3 to -0.2) (Table 2-9). For adults, the highest percent change was observed among people aged 50-69 years, -38.7 (-40.9 to -0.4) (Table 2-10). ^46^

• The age-standardized YLD rates due to IS per 100 000 were 122.2 (88 to 156.8) in 1990 and 81.2 (58.3 to 104) in 2019, representing a percent change of -33.5 (-35.9 to -0.3) (Tables 2-9 and 2-10). For adults, the highest percent change was observed among people aged 50-69 years, -39 (-42.1 to -0.4) (Table 2-10). ^46^

• The age-standardized YLD rates due to ICH per 100 000 were 44.3 (32.3 to 56.6) in 1990 and 28.5 (20.7 to 36.9) in 2019, representing a percent change of -35.7 (-38.8 to -0.3) (Tables 2-9 and 2-10). For adults, the highest percent change was observed among people aged 50-69 years, -43.8 (-47.2 to -0.4) (Table 2-10). ^46^

• The age-standardized YLD rates due to SAH per 100 000 were 13.9 (9.7 to 18.6) in 1990 and 11.2 (7.9 to 15) in 2019, representing a percent change of -19.5 (-23.7 to -0.2) (Tables 2-9 and 2-10). For adults, the highest percent change was observed among people aged 50-69 years, -19.9 (-26.1 to -0.1) (Table 2-10). ^46^

### DALY

• Data from the GBD Brazil Group show that the age-standardized DALY rates due to stroke per 100 000 were 2959 (2829.6 to 3063) in 1990 and 1219.6 (1142 to 1285.5) in 2019, representing a percent change of -58.8 (-61 to -0.6) (Tables 2-11 and 2.12). The highest percent change occurred in the state of Santa Catarina -67.8 (-71.1 to -0.6) and the lowest in the state of Maranhão -31.7 (-45.2 to -0.1) (Table 2-11). For adults, the highest percent change was observed among people aged 50-69 years, -60.3 (-62.8 to -0.6) (Table 2-12). ^46^

• The age-standardized DALY rates due to IS per 100 000 were 1333.3 (1244.5 to 1403.6) in 1990 and 561 (510.4 to 599.8) in 2019, representing a percent change of -57.9 (-61 to -0.6) (Tables 2-11 and 2-12). For adults, the highest percent change was observed among people aged 50-69 years, -63.5 (-66.5 to -0.6) (Table 2-12). ^46^

• The age-standardized DALY rates due to ICH per 100 000 were 1327.8 (1274 to 1397.3) in 1990 and 477.6 (450.9 to 503.8) in 2019, representing a percent change of -64 (-66.6 to -0.6) (Tables 2-11 and 2-12). For adults, the highest percent change was observed among people aged 50-69 years, -63.3 (-65.9 to -0.6) (Table 2-12). ^46^

• The age-standardized DALY rates due to SAH per 100 000 were 297.9 (267.5 to 312.7) in 1990 and 181 (169.4 to 192.8) in 2019, representing a percent change of -39.2 (-43.8 to -0.3) (Tables 2-11 and 2-12). For adults, the highest percent change was observed among people aged 15-49 years, -41.5 (-46.4 to -0.3) (Table 2-12). ^46^

## Healthcare Utilization

### Hospital Admissions

• Using time-series analysis, Katz *et al.* evaluated the relationship between stroke-related unemployment rate and hospital admission in Brazil over a recent 11-year span. Data on monthly hospital admissions due to stroke from March 2002 to December 2013 were extracted from the DATASUS, revealing 1 581 675 admissions due to stroke in the period. The unemployment rate decreased from 12.9% in 2002 to 4.3% in 2013, while admissions due to stroke increased. However, the adjusted model showed no positive association between the unemployment rate and admissions due to stroke (estimate coefficient=2.40±4.34; p=0.58). ^59^

• Using data from the SIH, the SIM and the Brazilian Institute of Geography and Statistics, Adami *et al.* analyzed rates of stroke-related mortality and incidence of hospital admissions in Brazilians aged 15-49 years, according to region and age group, between 2008 and 2012. Stroke was defined according to the ICD-10 (I60-I64). Crude and standardized mortality (WHO reference) and incidence of hospital admissions per 100 000 inhabitants, stratified by region and age group, were estimated. The authors found 131 344 hospital admissions due to stroke in Brazilians aged 15-49 years between 2008 and 2012. During the same time, the rate of hospital admissions stabilized: 24.67 (95% CI, 24.66 - 24.67) in 2008 and 25.11 (95% CI, 25.10 - 25.11) in 2012 (β = 0.09, p = 0.692, r2 = 0.05). ^60^

• Dantas *et al.* performed a study to assess stroke-related hospitalizations in the Brazilian Unified Health System from 2009 to 2016. The authors selected hospitalization registries according to the stroke diagnosis codes from the ICD-10. From 2009 to 2016, the number of admissions increased from 131 122 to 146 950, and the absolute number of in-hospital deaths increased from 28 731 to 31 937. Younger age and male sex were significantly associated with patient survival. The annual age-adjusted hospitalization and in-hospital mortality rates decreased by 11.8% and 12.6%, respectively, but the case-fatality rate increased for patients older than 70 years. ^61^

• In a retrospective study using data from DATASUS and assessing the six leading causes of hospital admissions in elders from 2005 and 2015, stroke was the third cause of admissions in 2015 for both genders and the age group of 60-79 years, with a -2.6 variation. ^62^

## Healthcare and Quality Indicators

• An analysis of the expansion trends of the family health strategy and hospitalization for conditions sensitive to primary care in Rio de Janeiro, between 1998 and 2015, showed a 7.6% decrease in hospitalizations for cerebrovascular diseases. ^63^

• A study assessing sociodemographic factors related to lack of hospital care for cerebrovascular disease deaths in the state of São Paulo in the periods of 1996-1998 and 2013-2015 showed that, of the 127 319 people who died due to stroke during the mentioned periods, 19 362 (15.2%) had no hospital care. In the latter period, a higher risk for death without care was identified for individuals of yellow race (RR = 1.48; 95% CI, 1.25-1.77), and a lower risk for black individuals (RR = 0,86; 95% CI, 0.76-0.95), married people (RR = 0.70; 95% CI, 0.64-0.75), and for those living in the city of São Paulo (RR = 0.92; 95% CI, 0.86-0.98). ^64^

• Data from a hospital-based study evaluating 2407 consecutive patients (mean age, 67.7 ± 14.4 years; 51.8% females) admitted to 19 hospitals in the city of Fortaleza showed that IS was the most frequent subtype (72.9%), followed by intraparenchymal hemorrhage (15.2%), SAH (6.0%), TIA (3%), and undetermined stroke (2.9%). The median time from symptom onset to hospital admission was 12.9 (3.8 –32.5) hours. Hypertension was the most common risk factor. Only 1.1% of the patients with IS received thrombolysis. The median time from hospital admission to neuroimaging was 3.4 (1.2–26.5) hours. ^65^

• A study evaluating factors that influence temporal trends in quality indicators for IS in a tertiary hospital, certified by the Joint Commission International as a primary stroke center, assessed 551 patients discharged with IS from January 2009 to December 2013 (median age 77.0 years, interquartile range: 64.0-84.0; 58.4% men). Ten predefined performance measures selected by the *Get With the Guidelines-Stroke* program were assessed. The quality indicators that improved with time were the use of cholesterol lowering therapy (P = 0.02) and stroke education (P = 0.04). The median composite perfect care did not consistently improve throughout the period (P = 0.13). After a multivariable adjustment, only thrombolytic treatment (OR 2.06, P < 0.01), dyslipidemia (OR 2.03, P < .01), and discharge in a Joint Commission International visit year (OR 1.8, P < 0.01) remained as predictors of a perfect care index of 85% or higher. The quality indicators with worse performance (anticoagulation for atrial fibrillation and cholesterol reduction) were similar in the tertiary and secondary community hospitals. The overall perfect care measure did not improve and was influenced by being discharged in a Joint Commission International visit year, having dyslipidemia, and having undergone thrombolytic treatment. ^66^

• An analysis of the stroke units in two centers from the cities of Curitiba and Botucatu, for the key performance indicators required by the Ministry of Health in Brazil, including the percentage of patients admitted to the stroke unit, venous thromboembolism prophylaxis in the first 48 hours after admission, pneumonia and hospital mortality due to stroke, and hospital discharge on antithrombotic therapy in patients without cardioembolic mechanism, showed that both centers admitted over 80% of the patients in their stroke unit. The incidence of venous thromboembolism prophylaxis was > 85% and that of in-hospital pneumonia was < 13%. The rate of in-hospital mortality from stroke was < 15% and that of hospital discharge on antithrombotic therapy was > 70%. ^67^

• A before and after study assessed the effect on mortality rates of the implementation of a dedicated cardiovascular and stroke unit in an emergency department of a tertiary public hospital in the city of Porto Alegre. The period prior to that unit implementation (2002 through 2005) included 4164 patients, and the vascular unit period (2007 to 2010) included 6280 patients. Overall, the case-fatality rate for acute vascular conditions decreased from 9% to 7.3% with the vascular unit implementation (p = 0.002). The in-hospital mortality rates from acute coronary syndrome dropped from 6% to 3.8% (p = 0.003) and from acute pulmonary embolism dropped from 32.1% to 10.8% (p < 0.001). The stroke case-fatality rate did not decrease despite improvements in the quality of stroke healthcare indicators. ^68^

• A cluster randomized trial assessed the effect of a multifaceted quality improvement intervention on adherence to evidence-based therapies for the care of patients with acute IS and TIA (including case management, reminders, a roadmap and checklist for the therapeutic plan, educational materials, and periodic audit and feedback reports to each intervention cluster). The study evaluated 1624 patients from 36 hospitals covering all Brazilian regions. The primary outcome was a composite adherence score for acute IS and TIA performance measures, and the secondary outcomes included an all-or-none composite endpoint of performance measures. The overall mean (SD) age of the patients enrolled in the study was 69.4 (13.5) years, and 913 (56.2%) were men. Overall mean (SD) composite adherence score for the 10 performance measures in the intervention group hospitals as compared to the control group hospitals was 85.3% (20.1%) vs. 77.8% (18.4%) (mean difference, 4.2%; 95% CI, -3.8% to 12.2%). As a secondary endpoint, 402 of 817 patients (49.2%) at the intervention group hospitals received all the therapies they were eligible for vs. 203 of 807 (25.2%) in the control group hospitals (OR, 2.59; 95% CI, 1.22-5.53; P = 0.01). The intervention did not result in a significant increase in the composite adherence score for evidence-based therapies in patients with acute IS or TIA. However, when using an all-or-none approach, the intervention resulted in improved adherence to evidence-based therapies and in improved thrombolysis rates. ^69^

## Complications

### Disability

• Benseñor *et al.* analyzed a community-based epidemiological survey (PNS - 2013) with a Brazilian representative sample to assess the absolute numbers with respective prevalence rates of stroke and post-stroke disabilities. The authors estimated 2 231 000 strokes and 568 000 stroke cases with severe disabilities. The point prevalence estimates for stroke were 1.6% and 1.4% in men and women, respectively. The prevalence of post-stroke disabilities was 29.5% for men and 21.5% for women. Stroke prevalence rates increased with aging, low education level, among people living in urban areas, and showed no difference according to self-reported skin color. The degree of post-stroke disability was not statistically different according to sex, race, education level or living area. ^50^

• In a subsequent assessment based on the PNS - 2013, access to rehabilitation is deficient: only 0.27% of the individuals underwent physical therapy for stroke and 0.12% performed some type of rehabilitation treatment, which impairs the user’s functional status. ^70^

• Carvalho-Pinto *et al.* conducted a retrospective observational study that collected data from medical records and home visits of post-stroke patients followed in a primary healthcare unit in the city of Belo Horizonte, Brazil, between May 2013 and May 2014. Data included health status, care received following stroke, personal and environmental contextual factors, functioning and disability, organized according to the International Classification of Functioning, Disability and Health conceptual framework. Most participants had good self-perception of manual ability (2.39 [SD, 2.29] logits) and limited walking ability (88%), were capable of improving natural gait speed, had a change in balance (51.43%) and functional mobility (54.16%) with risk of falling, and had a negative self-perception of quality of life (average score of 164.21 [SD, 35.16] points in the SSQOL-Brazil). ^71^

### Cost

• A cost-effectiveness study assessing thrombolytic drugs in Brazil reported that, for a 1-year result, for men, the cost of treatment with rt-PA was higher than that of the conservative treatment. This result is mainly directed by the cost of the medication. Part of this additional cost is compensated by the lower cost of rehabilitation and less productivity losses as early as the first 2 years, because the patients treated with rt-PA presented fewer sequelae than those who received conservative treatment. After the second post-stroke year, for both sexes, treatment with rt-PA (alteplase), considering direct and indirect costs, started to have a lower cost when compared to conservative treatment. From this time horizon onward, the additional cost of the medication starts to be more than compensated by the smaller productivity losses and lower social security and patient rehabilitation costs. ^72^

## Genetics/Family History

• Mitochondrial disorders, such as MELAS, may be responsible for up to one third of cryptogenic ISs in young patients. Stroke-like episodes can appear at any age in MELAS and occur in around 50% of the patients with the A3243G mitochondrial DNA mutation. The A3243G mutation has been reported in approximately 80% of the cases of MELAS, and other mitochondrial DNA mutations, such as T3271C, have also been described. In an investigation conducted by Conforto *et al.* both mutations were assessed in three groups of patients aged less than 46 years (Group 1: 15 patients with cryptogenic strokes; Group 2: 3 patients diagnosed with MELAS syndrome, including stroke-like episodes; Group 3: 20 healthy subjects). The A3243G mutation was absent in all subjects in Groups 1 and 3 but was present in all subjects in Group 2. Thus, these results do not support screening for those mutations to diagnose oligosymptomatic forms of MELAS in cryptogenic strokes in the absence of other features of the syndrome. ^73^

## Prevention

• The PURE study examined rates and predictors of use of evidence-based secondary prevention medications (ACEI/ARB, antiplatelets, statins, and beta-blockers) in patients with cardiovascular diseases, including CHD and stroke in South American countries, including Brazil. The study showed that fewer stroke patients received antiplatelets (24.3%), ACEI/ARB (37.6%), and statins (9.8%) as compared with CHD patients (30.1%, 36.0%, and 18.0%, respectively). This underutilization of therapies in stroke patients varied substantially among countries, with the lowest use in Colombia (no prescription of statins). When CHD and stroke patients were combined, the proportion of utilization of antiplatelets was highest in Chile (38.1%) and lowest in Argentina (23.0%). The use of ACEI/ARB and statins was higher in Brazil (46.4% and 26.4%) and lower in Colombia (26.4% and 1.4%), respectively. Among CHD and stroke participants, the use was higher in those with higher education level relative to those with none, primary, or unknown education [35.6% vs. 23.6% for antiplatelets (p = 0.002); 20.6% vs. 10.9% for statins (p = 0.0007)]. Former smokers with CHD or stroke were more likely to receive proven therapies than current smokers or those who had never smoked (35.2% vs. 26.6% and 27.7%, respectively, for antiplatelets [p = 0.039]; 19.9% vs. 10.6% and 13.0% for statins [p = 0.004]). Only 4.1% of the patients received all 4 therapies (antiplatelets, beta-blockers, ACEI/ARB, and statins), with the highest rate in Brazil (5.5%), and the lowest in Colombia (0.5%) (p = 0.02). Moreover, the use of no medication was observed in 30% of Brazilian stroke patients. ^74^

• The IMPACT-AF, a clustered randomized trial to IMProve treatment with AntiCoagulanTs in patients with Atrial Fibrillation, a leading cause of stroke, conducted in Argentina, Brazil, China, India, and Romania, showed that, overall, two-thirds of patients were on oral anticoagulation at baseline, 83% were on a VKA, and 15% were on NOACs. Patients from Brazil were most often on oral anticoagulation at baseline (91%), whereas only 38% of patients from China were on anticoagulation at baseline. Of all patients taking VKAs in Brazil, 40.3% had INR values between 2 and 3 prior to the baseline visit. ^75^

## Awareness, treatment, control

• Several studies have shown alarming lack of knowledge about stroke risk factors, stroke treatment, and recognition of stroke symptoms as a medical emergency. In a community-based study, Pontes-Neto *et al.* interviewed subjects in public places of four major cities in Brazil between July 2004 and December 2005, using a structured, open-ended questionnaire in Portuguese, based on a case presentation of a typical patient with acute stroke at home. The authors found 28 different Portuguese terms to name stroke. Twenty-two percent of the interviewees did not recognize any warning signs of stroke. Only 34.6% of the interviewees answered the correct nationwide emergency telephone number in Brazil (#192). Only 51.4% of the interviewees reported they would call an ambulance for a relative with symptoms of stroke. ^76^

• Falavigna A *et al.* used a closed-ended, self-administered questionnaire to assess the knowledge about stroke among 952 residents of the city of Caxias do Sul, Brazil. Lower income and lower educational level were independent predictors of inability to recognize that stroke affects the brain. Lower income and age < 50 years were independent predictors of lack of knowledge about stroke risk factors. ^77^

• In a community-based and cross-sectional study, Pitton Rissardo *et al.* applied a stroke knowledge survey to a convenience sample of 633 passers-by of a public square from December 2015 to October 2016, in the city of Santa Maria, state of Rio Grande do Sul. Of the respondents, 33% correctly reported the meaning of the acronym “AVC” (in Portuguese, *acidente vascular cerebral* ), the most recommended term to name stroke in Portuguese by the Brazilian Society of Cerebrovascular Diseases. Around 30% of subjects incorrectly localized stroke in the heart. Only 50% of the respondents correctly reported a warning sign of stroke. Individuals with a higher level of education were more likely to call an ambulance for a relative with stroke symptoms. ^78^

• In recent years, there have been several initiatives to improve public stroke awareness in Brazil, mainly around the World Stroke Day (October 29^th^) annual campaigns led by the World Stroke Organization. Despite these efforts, only 30–40% of patients with stroke are hospitalized within 4 hours of symptom onset. ^79^

## Future Research

• The Brazilian research portfolio in vascular neurology has evolved largely in recent years, as illustrated by the foundation of the Brazilian Stroke Research Network. Still, there are several opportunities for improvement. The most expressive community studies on stroke prevalence and incidence derive mostly from two cities. While both examples represent a major achievement in stroke epidemiology, there is still a need for a broader assessment comprising a representation of all geographical regions, diverse cultures, income levels, and ethnicities.

• Additionally, there are inherent constrains related to studies relying on stroke identification using ICD codes. It is not uncommon for users to apply a broader code on admission, that might not be adjusted during hospital stay, thus not representing the actual stroke subtype (e.g., an IS might be coded as non-specified stroke or even as TIA). With the dawn of the big data technologies (e.g., text mining), additional clinical information from admission or discharge records could provide a reliable cross-reference source, thus confirming or correcting a given code.

## 3. ACUTE AND CHRONIC CORONARY HEART DISEASE

### ICD-9-CM 410 to 414; ICD-10 I10 to I25

**See Tables 3-1 through 3-3 and Charts 3-1 through 3-20**

**Table:** Abbreviations Used in Chapter 3

ACCEPT/SBC	Brazilian Registry of Clinical Practice in Acute Coronary Syndromes of the Brazilian Society of Cardiology
ACS	Acute Coronary Syndrome
AMI	Acute Myocardial Infarction
ASA	Acetylsalicylic Acid
BRACE	Brazilian Registry in Acute Coronary Syndromes
BRIDGE-ACS	Brazilian Intervention to Increase Evidence Usage in Acute Coronary Syndromes
BYPASS	Brazilian Registry of Adult Patients Undergoing Cardiovascular Surgery
CABG	Coronary Artery Bypass Grafting
CCS	Chronic Coronary Syndrome
CENIC	Brazilian Nationwide PCI Registry (in Portuguese, *Central Nacional de Intervenções Cardiovasculares* )
CHD	Coronary Heart Disease
CI	Confidence Interval
CVD	Cardiovascular Diseases
DALYs	Disability-Adjusted Life Years
DATASUS	Brazilian Unified Health System Database
ERICO	Strategy of Registry of Acute Coronary Syndrome (in Portuguese, *Estudo de Registro de Insuficiência Coronariana* )
GBD	Global Burden of Disease
IHD	Ischemic Heart Disease
MASS	Medicine, Angioplasty, or Surgery Study
OR	Odds Ratio
PCI	Percutaneous Coronary Intervention
PNS	National Health Survey (in Portuguese, *Pesquisa Nacional de Saúde* )
RBSCA	Brazilian Registry of Acute Coronary Syndrome (in Portuguese, *Registro Brasileiro de Síndrome Coronariana Aguda* )
REPLICCAR-I	São Paulo's Cardiovascular Surgery Registry (in Portuguese, *Registro Paulista de Cirurgia Cardiovascular* )
RESISST	STEMI Registry from Salvador (in Portuguese, *Registro Soteropolitano de Infarto Agudo do Miocárdio com Supra de ST* )
SDI	Sociodemographic Index
SIH	Brazilian Hospital Information System (in Portuguese, *Sistema de Informações Hospitalares* )
STEMI	ST-Elevation Myocardial Infarction
SUS	Brazilian Unified Health System (in Portuguese, *Sistema Único de Saúde* )
UI	Uncertainty Interval
VICTIM	*Via Crucis* for the Treatment of Myocardial Infarction
YLDs	Years Lived with Disability
YLLs	Years of Life Lost

**Table 3-1:** – Number of cases, age-standardized prevalence rates (per 100 000) of ischemic heart disease in 1990 and 2019, and percent change of rates in the period, in Brazil and its Federative Units.

Location	1990		2019		Percent change (95% UI)
	Number (95% UI)	Rate (95% UI)	Number (95% UI)	Rate (95% UI)	
Acre	2744 (2323.2;3238.3)	1698.7 (1440.1;2001.1)	10165 (8757.7;11875.3)	1669.7 (1423.7;1950.1)	-1.7(-5.2;1.9)
Alagoas	22487.5 (18952.7;26631.9)	1728.3 (1461.3;2055)	51311.8 (43528.7;60808.3)	1632 (1382.7;1929.3)	-5.6(-9;-1.9)
Amapá	1435.5 (1221.4;1689.8)	1490.3 (1263.4;1756)	7640.2 (6545.1;9014.1)	1495.6 (1275.1;1755.7)	0.4(-3.2;4.2)
Amazonas	11671.4 (9935.2;13793.1)	1553.6 (1317.5;1820.5)	44391.2 (38294.2;52043.1)	1568.5 (1347.3;1835.6)	1(-2.5;4.5)
Bahia	108605.1 (92013.7;128033.5)	1657.4 (1405.6;1953.6)	276650.7 (236718;324478.5)	1729.9 (1472.1;2035.1)	4.4(0.4;8.6)
Brazil	1480208.9 (1271844.8;1725493.1)	1727.7 (1482.4;2017.9)	4003895.6 (3449807.8;4672110.4)	1708.7 (1465.5;1994.4)	-1.1(-2.6;0.5)
Ceará	63391.6 (54165.9;74916.2)	1587.7 (1354.7;1877.1)	152544.4 (130121.9;178195.9)	1539.8 (1311;1803.3)	-3(-6.6;0.3)
Distrito Federal	8632.3 (7371.6;10260.4)	1490 (1278;1748.5)	38394.4 (32946.6;44953.8)	1470.1 (1260.8;1707.5)	-1.3(-4.5;1.9)
Espírito Santo	23779.7 (20442.8;28015.9)	1678.2 (1438.7;1965.6)	72337.9 (62145.1;84311.7)	1669.9 (1435.9;1948.4)	-0.5(-3.8;3)
Goiás	31650 (26872.3;37325.5)	1580.9 (1340.4;1867.5)	112766.7 (95362.9;132919.9)	1634.4 (1385.1;1919.7)	3.4(-0.3;7)
Maranhão	39708.7 (33413.1;47193.9)	1581.3 (1332.7;1880.6)	100754.5 (85612.3;119041.2)	1549.1 (1312.2;1833)	-2(-5.7;1.7)
Mato Grosso	12613.1 (10808.1;14884.5)	1681.9 (1435.6;1982.7)	53895 (46476.4;63120.2)	1662.6 (1429.2;1939.3)	-1.1(-4.7;3)
Mato Grosso do Sul	14979.8 (12842.7;17679.7)	1752.6 (1499.2;2061.5)	53091.8 (45889.2;62249.5)	1820.7 (1570.5;2125)	3.9(0.3;7.6)
Minas Gerais	176140.3 (151248.2;205339.7)	1867.1 (1607;2172.4)	496702.3 (431006.5;576498.2)	1878.2 (1630.8;2178.4)	0.6(-2.4;4)
Pará	30506 (25805.7;36277.5)	1499.4 (1265.2;1775.8)	104725.1 (88987.6;123975.2)	1528.7 (1298;1810)	2(-1.6;5.7)
Paraíba	39766.3 (33685.5;46817.8)	1746.8 (1479.4;2053.9)	75707.5 (64303.6;89064.2)	1602.8 (1358.5;1890.4)	-8.2(-11.3;-5)
Paraná	82034.4 (70312.4;96269)	1763.5 (1512.1;2066.3)	232096 (199173.2;272919)	1749.9 (1503.9;2050.1)	-0.8(-4.1;2.6)
Pernambuco	73242.6 (62254.4;86784.7)	1652.2 (1410.2;1949.7)	165021.6 (141125.5;193595.4)	1660 (1410.8;1940.3)	0.5(-3.1;4.2)
Piauí	22358.8 (18937.4;26398.4)	1636.6 (1394.8;1927.9)	59627.3 (50948.3;69838)	1591.7 (1360.5;1865.5)	-2.7(-6.2;1.3)
Rio de Janeiro	158350.6 (135303.3;186081.8)	1709.7 (1466.3;1999.9)	370396.8 (317368.2;433395.5)	1649.7 (1415.8;1922.9)	-3.5(-6.7;0)
Rio Grande do Norte	25565.4 (21696.6;30114)	1625.3 (1379.4;1913.1)	61672.6 (52687.4;72269.3)	1603.6 (1369.5;1884.2)	-1.3(-5.1;2.2)
Rio Grande do Sul	112630.6 (96716.3;132207.6)	1771.6 (1523;2061)	271808.2 (233542.4;319107.2)	1740.4 (1502.8;2036.2)	-1.8(-4.7;1.5)
Rondônia	5685.1 (4817.2;6785.4)	1660.6 (1412;1967.5)	23529.2 (19995.2;27786.7)	1557.3 (1319.6;1833.5)	-6.2(-10.2;-2.2)
Roraima	976.7 (827.5;1160.6)	1682.3 (1423.7;1991.1)	5879.7 (5000.2;6970.1)	1583.5 (1344.1;1873.9)	-5.9(-9;-2.6)
Santa Catarina	42265.2 (36505.9;49567.5)	1727.3 (1479.7;2012.7)	142261.9 (121996.8;165916.3)	1752.8 (1509.2;2031.4)	1.5(-2;4.9)
São Paulo	349725.3 (301314.8;407755.5)	1818.8 (1563;2117.4)	961471 (831096;1119060.5)	1772.4 (1535.4;2056.1)	-2.6(-5.9;0.8)
Sergipe	13056.6 (11064.9;15423.9)	1688.7 (1435;1987.3)	37283.3 (32043.6;43768.5)	1683.7 (1442.6;1980.3)	-0.3(-3.7;3.3)
Tocantins	6206.4 (5271.1;7415.6)	1552.9 (1320.7;1839.1)	21769.6 (18644.8;25664.3)	1546.3 (1323.8;1824.2)	-0.4(-4;3.1)

*Source: Data derived from Global Burden of Disease Study 2019, Institute for Health Metrics and Evaluation, University of Washington. ^46^*

**Table 3-2:** – Number of deaths, age-standardized mortality rate (per 100 000) due to ischemic heart disease in 1990 and 2019, and percent change of rates in the period, in Brazil and its Federative Units.

Location	1990		2019		Percent change (95% UI)
	Number (95% UI)	Rate (95% UI)	Number (95% UI)	Rate (95% UI)	
Acre	138.6 (128.8;147.8)	113.1 (103.5;121.1)	349.4 (313.2;385.3)	65.3 (57.7;71.8)	-42.3(-48;-35.7)
Alagoas	1502.2 (1377.7;1611.1)	128.7 (116.9;138.6)	2785.4 (2455.8;3121.3)	89.1 (78.6;99.8)	-30.7(-39.3;-20.4)
Amapá	78.5 (72.4;84.2)	101 (91.1;108.3)	276.2 (247.5;301.5)	59.9 (52.6;65.5)	-40.7(-46;-35.6)
Amazonas	695.4 (633.5;755.7)	124.3 (112.2;134.8)	1468.3 (1276.5;1651.5)	55.7 (47.9;62.8)	-55.2(-60.4;-49.4)
Bahia	6853.8 (6096.3;7577.2)	113.9 (100.4;125.6)	11441.8 (9797.9;13115.2)	70.2 (60.4;80.3)	-38.3(-47.5;-27)
Brazil	117247 (111650.1;121246.9)	157.9 (146.9;164)	171246.3 (156180;180511.2)	74.9 (67.9;79.1)	-52.6(-54.9;-50.3)
Ceará	3607.4 (3080.1;4121.2)	94.1 (80;107.2)	7663.8 (6453.5;8924.8)	78 (65.6;90.8)	-17.1(-31;1.4)
Distrito Federal	651.4 (589.4;727.4)	189.6 (171.6;207.4)	1273.5 (1126.9;1425.3)	67.3 (58.9;75.3)	-64.5(-68.7;-59.7)
Espírito Santo	1673.8 (1588.5;1743.2)	147.3 (136.8;154.5)	3276.1 (2878.7;3654)	79.1 (69.7;88.2)	-46.3(-51.9;-40.7)
Goiás	2416.9 (2113.1;2844.4)	149.9 (131.3;174.4)	4936.2 (4136;5760)	75 (62.8;87.1)	-50(-58.6;-40.2)
Maranhão	3075.1 (2579.4;3583.6)	131.4 (109.2;152.5)	6670.2 (5691.2;7767.9)	104 (88.9;121)	-20.9(-35.2;-0.8)
Mato Grosso	915.1 (813;1018.8)	151.1 (134.5;166)	1963.2 (1759.7;2180.3)	64.5 (57.2;71.8)	-57.3(-61.9;-51.2)
Mato Grosso do Sul	1149.3 (1076.6;1218)	165.2 (151.3;175.7)	2165.9 (1920.3;2425.8)	78 (68.8;87.1)	-52.8(-57.8;-47.3)
Minas Gerais	12398.7 (11565.9;13348)	161 (147.4;173.3)	15629.4 (13736.3;17428.7)	59.2 (52;66)	-63.2(-67.3;-59.4)
Pará	2242 (1988.9;2500.7)	138.7 (122.8;153.4)	4412.1 (3894.1;4912.4)	66.5 (58.4;74)	-52.1(-58.1;-45.1)
Paraíba	2460.3 (2223.5;2681.6)	113 (101.3;123.2)	4020.9 (3487.9;4554.8)	81.7 (71.4;92.4)	-27.7(-37.2;-17.2)
Paraná	6738 (6451.3;7004.8)	180.6 (168.5;188.5)	9253.8 (8185.6;10317.7)	73.7 (65;82.2)	-59.2(-63.4;-54.6)
Pernambuco	5902.4 (5528.3;6218.4)	150.7 (139;159.2)	9852.3 (8776.8;10963.6)	101.7 (90.2;113.1)	-32.5(-39.8;-24.8)
Piauí	1420.5 (1287.7;1539.2)	125.4 (111.9;137.3)	2681.6 (2321.6;2993.7)	69.6 (60.6;77.8)	-44.5(-51.1;-37.4)
Rio de Janeiro	16553 (15826.5;17095.4)	207 (193.3;215)	19404.4 (17334.6;21411.9)	88.3 (78.9;97.4)	-57.3(-61.1;-53.1)
Rio Grande do Norte	1692.1 (1503.1;1855.9)	113.3 (100.1;124.4)	2945.9 (2469.4;3436.9)	74.1 (62.2;86.5)	-34.6(-44.6;-23)
Rio Grande do Sul	9356.6 (8842.8;9711.2)	170.1 (157.9;177.7)	10964.6 (9615.9;12195.2)	71.2 (62.4;79.1)	-58.2(-62.6;-53.9)
Rondônia	382.6 (340.8;423.2)	192.2 (175.4;207.2)	1071.8 (936.3;1224.9)	76.7 (66.9;87.5)	-60.1(-65.5;-54.3)
Roraima	59.8 (54.7;64.9)	156.8 (144.6;167.7)	205.3 (186.3;224.3)	70.6 (63.1;77)	-55(-59.2;-50.7)
Santa Catarina	3357.7 (3167.7;3535.1)	167.7 (156;177.4)	5223.5 (4616.9;5823.5)	69.7 (61.4;77.6)	-58.4(-62.7;-53.5)
São Paulo	30795 (29080.1;32357.1)	198.6 (183.3;209.7)	38670.9 (34008.3;42716)	74.8 (65.4;82.4)	-62.4(-65.9;-58.3)
Sergipe	744.1 (678;812.9)	121.6 (110.4;132.7)	1550.8 (1322.4;1775.1)	71 (60.4;81.2)	-41.6(-49.6;-32.6)
Tocantins	386.8 (342.1;434.8)	140.7 (125;156.7)	1088.6 (945.9;1247.1)	80.7 (70.2;92)	-42.7(-50.7;-33.9)

*Source: Data derived from Global Burden of Disease Study 2019, Institute for Health Metrics and Evaluation, University of Washington. ^46^*

**Table 3-3:** – Number of DALYs, age-standardized DALY rates (per 100 000) due to ischemic heart disease in 1990 and 2019, and percent change of rates in the period, in Brazil and its Federative Units.

Location	1990		2019		Percent change (95% UI)
	Number (95% UI)	Rate (95% UI)	Number (95% UI)	Rate (95% UI)	
Acre	3404.1 (3139.6;3645.5)	2093.7 (1937.9;2235.9)	8056 (7281.6;8875.9)	1271.5 (1148.8;1398.9)	-39.3(-45.8;-31.6)
Alagoas	35988 (33355.1;38794.7)	2642.2 (2445.9;2836.5)	64183.2 (57031.8;72062.4)	1957.6 (1742.2;2194.8)	-25.9(-35.5;-15.1)
Amapá	1872.1 (1729.6;2006.7)	1865.3 (1728.2;1990.5)	6757.6 (6131;7326.5)	1234.7 (1114.1;1343.3)	-33.8(-39.7;-27.6)
Amazonas	16169.9 (14743.4;17642.5)	2166.7 (1982.4;2353)	32551.7 (28674.8;36563.6)	1106.3 (973.8;1241.8)	-48.9(-55.3;-42)
Bahia	157006.3 (140109.9;174887)	2308.3 (2064.6;2565.5)	248479.4 (212225.9;284940.9)	1523.6 (1300.4;1747.9)	-34(-44.6;-20.8)
Brazil	2793361.6 (2696368.8;2875301.9)	3117.1 (2989.1;3214)	3721023.5 (3507748;3892657.2)	1563.3 (1472.1;1636.1)	-49.8(-52.2;-47.6)
Ceará	75826 (65771.4;86754.3)	1860.5 (1616.9;2128.9)	155657.8 (131957.2;182735.2)	1556.1 (1322.1;1827.7)	-16.4(-31.5;4.2)
Distrito Federal	18350.9 (16471.6;20617.2)	3247.2 (2939.1;3588.9)	28941.1 (25769.2;32405.2)	1147.9 (1017.5;1278.2)	-64.6(-69.1;-59.5)
Espírito Santo	39315.3 (37642.8;40842.8)	2723.4 (2595.9;2826.1)	71428.3 (63301.5;80094.9)	1616.2 (1434.6;1806.8)	-40.7(-47.6;-33.7)
Goiás	63256.2 (54931.7;74559.1)	2936.4 (2571.5;3439.6)	115723.9 (98226.5;135146.5)	1606.9 (1366.6;1870.7)	-45.3(-55.3;-32.9)
Maranhão	81890.1 (68565.6;95781.4)	3036.4 (2553.7;3520.6)	144195.8 (121818.6;171277)	2156.5 (1826.3;2545.6)	-29(-42.8;-9.2)
Mato Grosso	24136.6 (21151.7;27140.8)	2916.9 (2600.3;3235.1)	46201.2 (41637.4;51223.9)	1349.3 (1217.7;1495.9)	-53.7(-59.4;-45.9)
Mato Grosso do Sul	29159.4 (27471.5;30999.4)	3186.1 (2989.1;3385.4)	48763.9 (43588.8;54656.4)	1625.7 (1454.1;1821.7)	-49(-54.8;-42.4)
Minas Gerais	294064.9 (275086.6;318894.3)	3036.3 (2834.2;3277.7)	332544.7 (299215.8;369571)	1250.8 (1124.8;1388.1)	-58.8(-63.6;-54.1)
Pará	52443.4 (46628.1;58642.3)	2533.3 (2253.3;2811.5)	100474.6 (89523.2;111319.9)	1396.9 (1245.5;1548.5)	-44.9(-52.4;-35.9)
Paraíba	52628.5 (47840.1;57117.1)	2310.8 (2096;2507.7)	82129.3 (72301.8;93181.2)	1736.4 (1528.3;1969.4)	-24.9(-35.2;-13.2)
Paraná	161480.7 (156250.5;167183)	3367.8 (3231.3;3489)	200724.6 (177943.7;223894)	1495.3 (1327.8;1670.7)	-55.6(-60.4;-50.2)
Pernambuco	135058.1 (128395.1;141504.1)	2984.4 (2825.6;3128.2)	220562.4 (197651;245799)	2163.2 (1936.7;2404.8)	-27.5(-35.5;-18.7)
Piauí	33451.5 (30836.8;36206.4)	2409.3 (2204.1;2608.6)	55492.6 (49570.2;61446.8)	1468.9 (1312.9;1626.4)	-39(-46.4;-31)
Rio de Janeiro	407746.1 (394302.6;419720.2)	4171.5 (4011.9;4299.1)	427010.3 (384691.2;474730.2)	1899.1 (1712.3;2108.5)	-54.5(-58.9;-49.4)
Rio Grande do Norte	34665.5 (31015.3;38186.2)	2189.7 (1958;2411.2)	61578.7 (52237.5;71777.6)	1573 (1335.7;1833.8)	-28.2(-39.9;-14.1)
Rio Grande do Sul	218591.9 (209049.2;225505.3)	3319.3 (3158.8;3432.1)	220881.2 (195868.6;245433.1)	1424.1 (1263.9;1583.3)	-57.1(-61.7;-52.5)
Rondônia	11257.2 (9744.8;12593.1)	3362.8 (3058.3;3657.7)	24715.8 (21437.5;28409.9)	1558.4 (1359.9;1783.2)	-53.7(-60.3;-45.8)
Roraima	1722.5 (1541.1;1885.8)	2850.2 (2648.8;3067)	5071 (4637.9;5541.6)	1319.7 (1203;1436.8)	-53.7(-58.3;-48.3)
Santa Catarina	78531.6 (74508.7;82564.6)	3139.7 (2968.8;3300.9)	112745.8 (100343.6;125606.6)	1372.9 (1220.7;1526.7)	-56.3(-61;-51.2)
São Paulo	739272.4 (701242.6;775952.1)	3716.7 (3515.9;3898)	848171.4 (760424.2;937315.5)	1552.8 (1387.5;1711.6)	-58.2(-62.4;-53.7)
Sergipe	16140.7 (14704.3;17710.1)	2146.4 (1961.4;2348)	33976.8 (29494.2;39095.9)	1483.8 (1289.8;1703.2)	-30.9(-40.9;-19.3)
Tocantins	9931.6 (8722.7;11149.1)	2511.8 (2230.3;2811.7)	24004.5 (20790;27711.9)	1649.1 (1433.6;1900)	-34.3(-44.4;-22.7)

*Source: Data derived from Global Burden of Disease Study 2019, Institute for Health Metrics and Evaluation, University of Washington. ^46^*

**Chart 3-1:** – Crude and age-standardized prevalence rates (per 100 000) of ischemic heart disease in Brazil, 1990-2019. Shaded areas show 95% uncertainty intervals.

Source: Data derived from Global Burden of Disease Study 2019, Institute for Health Metrics and Evaluation, University of Washington. ^46^

**Chart 3-2:** – Prevalence rate (per 100 000) of ischemic heart disease according to sex and age in Brazil, 2019. Error bars show 95% uncertainty intervals.

Source: Data derived from Global Burden of Disease Study 2019, Institute for Health Metrics and Evaluation, University of Washington. ^46^

**Chart 3-3:** – Incidence rate (per 100 000) of ischemic heart disease according to sex and age. Brazil, 2019. Error bars show 95% uncertainty intervals.

Source: Data derived from Global Burden of Disease Study 2019, Institute for Health Metrics and Evaluation, University of Washington. ^46^

**Chart 3-4:** – Mortality rate (per 100 000) due to ischemic heart disease according to sex and age in Brazil, 2019. Error bars show 95% uncertainty intervals.

Source: Data derived from Global Burden of Disease Study 2019, Institute for Health Metrics and Evaluation, University of Washington. ^46^

**Chart 3-5:** – Crude and age-standardized mortality rates (per 100 000) due to ischemic heart disease in Brazil, 1990-2019. Shaded areas show 95% uncertainty intervals.

Source: Data derived from Global Burden of Disease Study 2019, Institute for Health Metrics and Evaluation, University of Washington. ^46^

**Chart 3-6:** – Age-standardized mortality rate (per 100 000) from ischemic heart disease by Brazilian regions, 1990-2019.

Source: Data derived from Global Burden of Disease Study 2019, Institute for Health Metrics and Evaluation, University of Washington. ^46^

**Chart 3-7:** – Correlation between the percent change in age-standardized mortality rates due to ischemic heart disease from 1990 to 2019 and the 2019 Sociodemographic Index (SDI).

Source: Data derived from Global Burden of Disease Study 2019, Institute for Health Metrics and Evaluation, University of Washington. ^46^ ^,^ ^95^

**Chart 3-8:** – In-hospital mortality rate due to acute coronary syndrome (ACS), acute myocardial infarction (AMI), percutaneous coronary intervention (PCI), and coronary artery bypass graft (CABG) surgery in the public health system of Brazil, 2008-2019.

Source: Ministry of Health of Brazil – Hospital Information System of the Unified Health System (SIH/SUS). ^95^

**Chart 3-9:** – Correlation between the in-hospital mortality rate due to acute coronary syndrome (ACS) in the public health system in 2019 and the 2019 Sociodemographic Index (SDI).

Sources: Ministry of Health of Brazil – Hospital Information System of the Unified Health System (SIH/SUS) ^95^ and Global Burden of Disease (GBD) Study 2019. ^46^

**Chart 3-10:** – Correlation between the in-hospital mortality rate due to acute myocardial infarction (AMI) in the public health system in Brazil in 2019 and the 2019 Sociodemographic Index (SDI).

Sources: Ministry of Health of Brazil – Hospital Information System of the Unified Health System (SIH/SUS) ^95^ and Global Burden of Disease (GBD) Study 2019. ^46^

**Chart 3-11:** – Crude and age-standardized DALY rates (per 100 000) due to ischemic heart disease in Brazil, 1990-2019. Shaded areas show 95% uncertainty intervals.

Source: Data derived from Global Burden of Disease Study 2019, Institute for Health Metrics and Evaluation, University of Washington. ^46^

**Chart 3-12:** – Age-standardized DALY rates (per 100 000) due to ischemic heart disease by Brazilian regions, 1990-2019.

Source: Data derived from Global Burden of Disease Study 2019, Institute for Health Metrics and Evaluation, University of Washington. ^46^

**Chart 3-13:** – Correlation between the percent change of age-standardized DALY rates due to ischemic heart disease from 1990 to 2019 and the 2019 Sociodemographic Index (SDI).

Source: Data derived from Global Burden of Disease Study 2019, Institute for Health Metrics and Evaluation, University of Washington. ^46^ ^,^ ^95^

**Chart 3-14:** – Number of hospital admissions for acute coronary syndrome (ACS), acute myocardial infarction (AMI), and chronic coronary syndrome (CCS) to the public health system in Brazil, 2008-2019.

Source: Ministry of Health of Brazil – Hospital Information System of the Unified Health System (SIH/SUS). ^95^

**Chart 3-15:** – Number of hospital admissions for primary percutaneous coronary intervention (PCI), non-primary PCI, and coronary artery bypass graft (CABG) surgery to the public health system in Brazil, 2008-2019.

Source: Ministry of Health of Brazil – Hospital Information System of the Unified Health System (SIH/SUS). ^95^

**Chart 3-16:** – Percentage of percutaneous coronary intervention (PCI) and coronary artery bypass graft surgery (CABG) out of myocardial revascularization procedures in the Brazilian public health system, 2008-2019.

Source: Ministry of Health of Brazil – Hospital Information System of the Unified Health System (SIH/SUS). ^95^

**Chart 3-17:** – Total amount in international dollars (Int$ 2019) reimbursed for hospital admissions due to acute coronary syndrome (ACS), acute myocardial infarction (AMI), and chronic coronary syndrome (CCS) to the public health system in Brazil, 2008-2019.

Source: Ministry of Health of Brazil – Hospital Information System of the Unified Health System (SIH/SUS). ^95^

**Chart 3-18:** – Average amount in international dollars (Int$ 2019) reimbursed per hospital admission due to acute coronary syndrome (ACS), acute myocardial infarction (AMI), and chronic coronary syndrome (CCS) to the public health system in Brazil, 2008-2019.

Source: Ministry of Health of Brazil – Hospital Information System of the Unified Health System (SIH/SUS). ^95^

**Chart 3-19:** – Total amount in international dollars (Int$ 2019) reimbursed for hospital admissions due to primary percutaneous coronary intervention (PCI), non-primary PCI, and coronary artery bypass graft (CABG) surgery to the public health system in Brazil, 2008-2019.

Source: Ministry of Health of Brazil – Hospital Information System of the Unified Health System (SIH/SUS). ^95^

**Chart 3-20:** – Average amount in international dollars (Int$ 2019) reimbursed per hospital admission due to primary percutaneous coronary intervention (PCI), non-primary PCI, and coronary artery bypass graft (CABG) surgery to the public health system in Brazil, 2008-2019.

Source: Ministry of Health of Brazil – Hospital Information System of the Unified Health System (SIH/SUS). ^95^

### Overview and Prevalence

• Ischemic heart disease, also known as CHD, comprises a spectrum of symptomatic and asymptomatic clinical conditions typically related to a reduction in blood flow to the heart muscle. The most common cause is atherosclerotic disease in the coronary arteries, a chronic condition with varied presentations, progressing from a long asymptomatic phase to stable angina, AMI, and unstable angina. Ischemic heart disease is a common cause of heart failure, with reduced or preserved left ventricular ejection fraction, ventricular arrhythmias, and sudden cardiac arrest.

• Ischemic heart disease was the leading cause of death in Brazil in the last decade, for men and women. Because of its wide range of clinical presentations, the reported IHD prevalence, incidence, and mortality vary widely, depending on the population and healthcare setting studied.

#### Ischemic Heart Disease

• According to data from the GBD study, the number of individuals living with IHD, defined as those with previous AMI, stable angina, or ischemic heart failure, in Brazil increased from 1.48 million in 1990 to more than 4 million in 2019 (Table 3-1), and the crude IHD prevalence increased from 0.99% to 1.85% in the period (Chart 3-1). These rates sharply increased with aging: 0.4%, 4.4%, and 14.0% among individuals aged 15-49 years, 50-69 years, and 70 years or older, respectively, in 2019. In all age strata, IHD was more frequent in males than in females (Chart 3-2). ^46^

• The continuous increase in the number of IHD cases can be explained by the population growth and aging, because the age-standardized prevalence rate remained stable from 1990 to 2019 (percent change: -1%, Table 3-1 and Chart 3-1). In 2019, the age-standardized IHD prevalence was 1709 (95% UI, 1466-1994) per 100 000 inhabitants in the total population (Table 3-1), 1046 (95% UI, 905-1209) per 100 000 females and 2534 (95% UI, 2170-2975) per 100 000 males. ^46^

• In general, there was a difference in the age-standardized IHD prevalence among the Brazilian regions in 2019, with the highest prevalence rates observed in the Southeastern and Southern regions (state of Minas Gerais: 1878 per 100 000 inhabitants; states of Paraná and Santa Catarina: ~1750 per 100 000), and the lowest prevalence rates in the Northern region (state of Amapá: 1496 per 100 000, Table 3-1). ^46^

• The self-reported prevalence of IHD was 2.7% in the baseline assessment of the ELSA-Brasil cohort in 2008-2010. This cohort was composed of more than 15 000 civil servants aged 35-74 years from six cities (Salvador, Vitória, Belo Horizonte, Rio de Janeiro, São Paulo, and Porto Alegre). The prevalence was higher in males and individuals in the lower socioeconomic level. ^80^

#### Myocardial Infarction

• The reported prevalence of AMI was 4.0% in the *May Measurement Month 2017* cross-sectional survey of 7260 individuals from different ethnicities and regions of Brazil. Participants (56% women; mean age, 51.6±16.1 years) were mainly recruited from hospitals and indexed clinics. ^81^

#### Types of Acute Coronary Syndrome

• The large prospective ACCEPT registry, planned by the Brazilian Society of Cardiology, enrolled 4782 patients with ACS in 53 institutions (public hospitals, health maintenance organizations, and private services) from the five Brazilian regions. The sites were mostly tertiary, with high availability of PCI and cardiac surgery. Participants were included between 2010 and 2014. The percentages of patients with unstable angina, non-STEMI, and STEMI were 30%, 34%, and 36%, respectively. ^82^

• The BRACE study, a cross-sectional, epidemiological registry of ACS patients, used a stratified “cluster sampling” methodology to select services that were representative of all hospitals with intensive care or cardiology unit in Brazil. Among 1150 patients from 72 hospitals included in the study from 2007 to 2009, 54% had STEMI. Among the remaining patients with non-ST elevation ACS, 67% had non-STEMI, while 33% had unstable angina. ^83^ ^,^ ^84^

• In the ERICO study, a prospective cohort of patients admitted with ACS in a university hospital in the city of São Paulo, among 964 participants recruited from 2009 to 2012, the initial diagnosis was unstable angina in 33%, non-STEMI in 39%, and STEMI in 28% of the cases. ^85^

• The BRIDGE-ACS trial was a cluster-randomized study involving 1150 patients with ACS recruited from general public hospitals in Brazilian major urban areas in 2011. The rates of diagnosis of unstable angina, non-STEMI, and STEMI were 24%, 36%, and 40%, respectively. ^86^

#### Stable Angina

• Regional population surveys conducted in 2007, applying the Angina Rose questionnaire, reported a prevalence of angina of 12.3% among adults aged ≥30 years in the city of Ribeirão Preto, and of 8.2% among individuals aged ≥40 years in the city of Pelotas. ^87^ ^,^ ^88^

• According to the 2013 PNS, an epidemiological home-based survey, country-wide representative, the overall prevalence rates of class I and class II angina were 7.6% (95% CI, 7.2%-8.0%) and 4.2% (95% CI, 3.9%-4.5%), respectively. ^89^

• Self-reported angina *pectoris* was more prevalent in females than in males in all studies described.

• It is important to note the higher prevalence rates observed in prospective surveys as compared to national statistics. Self-reported assessments of angina are very sensitive but not specific to CHD, because they require neither confirmatory exams nor health reports. In addition, national statistics might underrepresent the true epidemiology of CHD, considering its asymptomatic nature.

## Incidence

• The GBD study estimated an incidence of 260 661 (95% UI, 230 100 - 293 617) IHD events (mainly myocardial infarction) in Brazil in 2019. As expected, the incidence rate was strongly associated with aging: 29 (95% UI, 23-36), 323 (95% UI, 268-386), and 737 (95% UI, 625-868) per 100 000 individuals aged 15-49 years, 50-69 years, and 70 years or older, respectively. In all age groups, IHD was more incident in males than in females (Chart 3-3). ^46^

• In 2019, the age-standardized incidence rate was 110 (95% UI, 97-124) per 100 000 inhabitants in the whole population, 78 (95% UI, 69-88) per 100 000 females, and 148 (95% UI, 130-166) per 100 000 males. ^46^

• While the crude IHD incidence rate continuously increased from 1990 to 2019, the age-standardized rate slightly decreased (-15%) from 1990 to 2000, remaining stable thereafter (percent change from 2000 to 2019: 3%). From 1990 to 2019, the percent change in the age-adjusted incidence rate of IHD was -12% (95% UI, -15% to -10%) in the total population, and 7% (95% UI, 1% to 12%), 2% (95% UI, -1% to 6%), and -19% (95% UI, -22% to -16%) among individuals aged 15-49 years, 50-69 years, and 70 years or older, respectively. ^46^

• In a systematic review from public health data in 2012, the incidence rates of AMI and ACS per 100 000 inhabitants were 29.8 and 38, respectively. ^8^

## Mortality

• According to the GBD estimates, there were 171 246 deaths due to IHD in Brazil in 2019 (Table 3-2), corresponding to 12% (11%-13%) of total deaths in the country and 43% of all CVD deaths. ^46^

• The crude mortality rate attributed to IHD was 79 (95% UI, 72-83) per 100 000 inhabitants in 2019. The rates increased with aging: 11 (95% UI, 11-12), 149 (95% UI, 142-156), and 751 (95% UI, 651-807) per 100 000 individuals aged 15-49 years, 50-69 years, and 70 years or older, respectively. In all age groups, the mortality rate was higher in males than in females (Chart 3-4). ^46^

• In 2019, the age-standardized mortality rate due to IHD was 75 (95% UI, 68-79) per 100 000 inhabitants in the total population (Table 3-2), 58 (95% UI, 51-63) per 100 000 females and 96 (95% UI, 88-101) per 100 000 males. ^46^

• In 2019, IHD ranked as the number one cause of death in Brazil in all but two Federative Units (states of Amazonas and Amapá, where IHD was the second most frequent cause of death). In addition, IHD was the main cause of death in both females and males over 50 years of age, and the third cause of fatalities among men aged 15-49 years (after interpersonal violence and road injuries). ^46^

• According to the GBD study, the unadjusted mortality rate due to IHD mildly increased from ~2005 to 2019, while the age-standardized rate continuously decreased from 1990 to 2019 (cumulative percent change: -53%, Table 3-2 and Chart 3-5). ^46^

• The decrease in the mortality rate from 1990 to 2019 was less pronounced in individuals aged 15-49 years (-34.9%, 95% UI, -38.5% to -31,1%) as compared to those aged 50-69 years (-47.8%, 95% UI, -50.6% to -45,0%) or 70 years or older (-46.5%, 95% UI, -50.1% to -43,7%). ^46^

• Regional differences in age-adjusted mortality rates and trends over time were noted. In 1990, rates were higher in the Southern and Southeastern regions, and lower in the Northern and Northeastern states. From 1990 to 2019, a reduction in mortality rates was observed in all states (less impressive in the Northeastern) and the heterogeneity among regions was progressively attenuated (Chart 3-6). The mildest variations occurred in the states of Ceará (-17%) and Maranhão (-21%), whereas the most pronounced decreases were detected in the Distrito Federal (-65%) and the states of Minas Gerais (-63%) and São Paulo (-62%, Table 3-2). A negative correlation was observed between the change in age-standardized mortality rate from IHD in the period and the 2019 SDI ( *r* ^2^0.61, p-value &lt; 0.01, Chart 3-7). ^46^ ^,^ ^90^ In 2019, the lowest death rates were observed in the states of Amazonas (56 per 100 000) and Minas Gerais (59 per 100 000), whereas the highest rates were noted in the states of Maranhão (104 per 100 000) and Pernambuco (102 per 100 000, Table 3-2). ^46^

• In a temporal analysis of data from the Brazilian Ministry of Health, the coefficient of death related to IHD remained stable for women in the Northern and West-Central regions between 1981 and 2001, whereas it decreased in the Southern and Southeastern regions, and increased in the Northeastern. For males, there was a trend towards a decrease in events in the Southern and Southeastern regions. ^91^

• An analysis conducted in the DATASUS from 1990 to 2009 demonstrated a reduction in the age-adjusted mortality rate due to IHD in Brazil. The rate reduced from 195 to 149 per 100 000 males (variation: -23.4%) and from 120 to 84 per 100 000 females (variation: -29.5%). ^92^

• The proportion of deaths caused by CVD has remained stable over the last decades, with reports ranging from 26% to 32%, according to the year. An ecologic study in the city of Porto Alegre, including individuals aged 45-64 years, demonstrated that CVD was responsible for 28.5% of all deaths in 2009. Of those, 40% were related to IHD, whose proportion was higher among those with a lower socioeconomic status (42.7%) than those with a higher socioeconomic status (26.3%). ^93^

• In a national ecologic study including individuals aged 35-64 years, the rate of death related to IHD was 84 ± 30 per 100 000 inhabitants from 1999 to 2001. The incidence of events was directly related to the poverty rate and lower educational attainment. Importantly, there was wide variability in the results across the 98 participating cities, probably due to data quality. ^94^

### Mortality Related to Acute Coronary Syndromes

• According to the SIH/SUS, the in-hospital mortality rate among patients treated for ACS in the public service setting remained stable from 2008 to 2019 [2.2% (1404 deaths out of 63 913 admissions) and 2.1% (1449 out of 70 013), respectively] (Chart 3-8). There was a modest negative correlation between the in-hospital mortality rate during treatment for ACS in 2019 and the SDI ( *r* ^2^0.27, p-value = 0.01), with higher rates detected in the Northeastern and Northern states (Chart 3-9). ^90^ ^,^ ^95^

• Among individuals admitted with AMI, the in-hospital mortality rate decreased from 15.9% (7627 deaths in 48 114 admissions) in 2008 to 12.9% (10 445 deaths in 80 944 admissions) in 2019, a percent change of -19% (Chart 3-8). A mild negative correlation between the in-hospital mortality rate during hospitalization for AMI in 2019 and the SDI was observed ( *r* ^2^0.18, p-value = 0.03, Chart 3-10). ^90^ ^,^ ^95^

• Several Brazilian ACS registries reported the outcomes of individuals admitted with ACS. In general, the mortality rate in registries is lower than that reported in the SIH/SUS. Several studies highlighted regional differences in treatment practices and mortality, as well as poorer outcomes in patients treated in public services as compared to those admitted to private hospitals. ^82^

• In the ACCEPT registry, among 2485 ACS patients recruited from 47 Brazilian hospitals in 2010/2011, the all-cause mortality at 30 days was 1.8% for unstable angina, 3.0% for non-STEMI, and 3.4% for STEMI. ^97^

• In a subsequent report from the ACCEPT registry analyzing a total of 4782 patients recruited up to 2014, the rate of major cardiovascular events was 13.6% after 1 year of follow-up. Events occurred more frequently among patients from the SUS (16.6 per 100 patients/year), as compared to private patients or those with health insurance (9.1 per 100 patients/year). In addition, a regional discrepancy in the rate of death at 1 year was noted, with higher numbers in the Northern region (19.8%; 95% CI, 12.6%-27.0%) and lower rates in the Northeastern region (5.6%; 95% CI, 3.7%-7.5%). ^82^

• In the BRACE cross-sectional registry, the overall in-hospital mortality was 5.2% among 1150 ACS patients recruited from 2007 to 2009 in 72 hospitals representative of all national services with intensive care or cardiology unit. ^83^ ^,^ ^84^

• The RBSCA registry enrolled 2693 patients between 2003 and 2008, including 45% of AMI cases. The in-hospital mortality rate was 3.1% for those with unstable angina and 7.7% for those with AMI, leading to an overall rate of 5.5%. ^98^

• In a retrospective, multicenter study of 3745 patients admitted for ACS between 2010 and 2015, the in-hospital all-cause mortality was 3.3%, and 454 (12.2%) patients experienced at least one major adverse event (reinfarction, shock, bleeding, stroke, or death). ^99^

• In the ERICO study, the mortality rate was 4.4% at 30 days and 12% at 1 year among 964 patients admitted with ACS from 2009 to 2012. ^85^

• In an observational longitudinal study undertaken from 2011 to 2014 in a high-complexity hospital in the city of Belo Horizonte, the in-hospital mortality was 9.1% among 788 patients with STEMI and 7.6% among 341 patients with non-STEMI. ^100^

• In a study from the Minas Telecardio 2 Project, conducted in 2013 and 2014 in six emergency units in the city of Montes Claros, among 593 patients with ACS, the in-hospital mortality was 9.4%, ranging from 4.9% for unstable angina to 17% for STEMI cases. ^101^

• In the RESISST registry, 520 STEMI patients were admitted to public healthcare units interconnected through a Regional Integrated Care Network, from January 2011 to June 2013. Only 41% of the patients underwent reperfusion therapy, and the 30-day mortality rate was 15.3%. ^102^

• In the VICTIM Registry, statewide data from the state of Sergipe collected from 2014 to 2017 identified 707 cases of STEMI with in-hospital mortality of 10.9%. There was a significantly higher mortality rate for those admitted to public hospitals compared to those admitted to private services (11.9% *versus* 5.9%, respectively). ^96^

• Among 1263 non-STEMI patients admitted to a private hospital in the city of São Paulo from 2014 to 2018, the in-hospital mortality rate was 1.3%. ^103^

• Among 1852 STEMI patients admitted to municipal emergency rooms in the city of São Paulo from 2010 to 2016 and undergoing a pharmaco-invasive treatment, the in-hospital mortality was 4.0% among patients aged <75 years and 18.2% among those aged ≥75 years. ^104^

• Mortality rates were reported in a registry of 542 consecutive patients admitted with STEMI and undergoing primary PCI between March 2011 and February 2017 in a tertiary university hospital in the city of Porto Alegre. In-hospital death occurred in 10.7% during the study period and was stable from 2011 to 2016. The 1-year mortality rate was 16.6% with a decreasing trend from 2011 to 2016. ^105^

•A 21-year time series study explored trends in AMI-related mortality according to sex, regions of Brazil, and type of city (state capital *versus* non-capital). Mortality data were retrieved from the Mortality Information System of the Brazilian Ministry of Health and the authors applied procedures to correct mortality rates for ill-defined causes, use of garbage codes, and underreporting. From 1996 to 2016, the age-standardized mortality rate from AMI declined 44% in the country, with substantial regional differences (percent changes: +5% in the North, +11% in the Northeast, -35% in the West-Central, -68% in the Southeast, and -85% in the South). Moreover, temporal changes were more pronounced in females than in males, and in capital cities than in non-capital cities. The corrected age-standardized mortality rate from AMI decreased 49% and 23% in females living in capital cities and in other municipalities, respectively, from 1996 to 2016. Among males, the respective declines were 43% and 17%. Importantly, improvements in the quality of data (e.g., decrease in underreporting) have occurred over the years; this phenomenon is more recent in the Northern and Northeastern regions and in non-capital cities. ^106^

### Mortality Related to Percutaneous Coronary Intervention

• According to the SIH/SUS, the in-hospital mortality rate among patients undergoing any PCI in public hospitals remained fairly stable from 2008 to 2019 [2.4% (1112 deaths out of 46 683 admissions) and 2.7% (2625 out of 96 930), respectively], with a percent change of 14% (Chart 3-8). In 2019, these rates ranged from 2.4% in the Southeastern to 3.5% in the Northeastern region. Among those who underwent primary PCI, the in-hospital mortality was 6.8% (765 fatalities out of 11 270 procedures), ranging from 5.9% in the Northern, Southern, and West-Central regions to 7.3% in the Southeastern region. ^95^

• In a cohort study undertaken from 2009 to 2013 (Brazilian PCI multicenter registry), among 4806 patients undergoing PCI (69% with recent AMI) in eight tertiary referral medical centers, the in-hospital mortality rate was 2.6%. ^107^

• In another PCI registry including 1249 consecutive patients in 2009, the total mortality rate was 2.3%, ranging from 0.2% for patients with stable angina to 6.1% for those with STEMI. ^108^

• In a study that evaluated PCIs in public hospitals from 2005 to 2008, 166 514 procedures were performed in 180 hospitals. The average in-hospital mortality was 2.3%, ranging from 0% to 11.4%. This rate was lower in the Southeastern region (2.0%) and higher in the Northern region (3.6%). The in-hospital mortality rate was 2.3% in high-volume hospitals, accounting for 101 218 (60.8%) PCIs, 2.3% in medium-volume hospitals, and 2.5% in low-volume hospitals. Mortality rate was higher among females and patients older than 65 years. ^109^

• Most reports originate from public institutions, and data from private hospitals are limited. An analysis of 440 procedures performed in a public and another private hospital in the city of Rio de Janeiro between 2013 and 2014 showed low mortality (0.5%), with similar rates in both institutions. ^110^

• Differences in in-hospital mortality after PCI according to femoral or radial access were retrospectively analyzed in 158 363 patients enrolled in the CENIC Registry between 2006 and 2016 (52% with stable CHD). Use of radial access progressively increased from 12% in 2006 to 50% in 2016 and was associated with lower in-hospital mortality rate as compared to femoral access in an analysis with propensity score matching (0.4% *versus* 0.7%; OR, 0.59; 95% CI, 0.47-0.74; p < 0.001; n = 54 242 patients). ^111^

• In 847 patients older than 90 years undergoing PCI (68% with ACS) and included in the CENIC Registry between 2006 and 2016, the in-hospital mortality rate was 4.8%. ^112^

• There are scarce data on long-term survival rates of patients undergoing PCI. In an analysis from procedures performed in the state of Rio de Janeiro between 1999 and 2000 in all public hospitals including 19 263 individuals, 1-year survival was 93% and 15-year survival was 57%. In that study, women, as compared to men, had a higher survival rate within 15 years after PCI. ^113^

### Mortality Related to Coronary Artery Bypass Graft Surgery

• According to the SIH/SUS, the in-hospital mortality rate among patients undergoing CABG surgery in public institutions was 7.0% (1566 deaths in 22 537 procedures) in 2008 and 6.1% (1432 in 23 488 admissions) in 2019, a percent change of -12% (Chart 3-8). In 2019, the lowest rate was observed in the Northeastern region (4.4%), while the highest rate was found in the West-Central region (10.0%). ^95^

• The BYPASS registry is an ongoing database established in 2015 by the Brazilian Society of Cardiovascular Surgery and involves 17 institutions representing all Brazilian regions. Among 2292 patients enrolled until November 2018 who underwent isolated or combined CABG, the in-hospital mortality rate was 2.8%, while 5.3% stayed on mechanical ventilation for more than 24 hours and 1.2% had an in-hospital stroke. ^114^ ^,^ ^115^

• The MASS II trial was a single-center randomized clinical trial designed to compare the long-term effects of medical therapy, angioplasty, or surgical strategies for the treatment of multivessel CAD with stable angina and preserved ventricular function conducted before 2007. The in-hospital mortality rates for PCI and CABG were 2.4% and 2.5%, respectively. ^116^ The 10-year survival rates were not significantly different between the groups: 75% for CABG, 75% for PCI, and 69% for medical therapy (p=0.089). ^117^ In another trial of the same group (MASS III), similar 10-year survival rates were described. ^118^

• In the REPLICCAR-I Registry, 2961 patients underwent isolated CABG in 10 hospitals between 2013 and 2016. The all-cause mortality rate was 3.4% at 30 days and 5.3% at 4 years of follow-up. ^119^

• Several other single-center experiences, with both retrospective and prospective analyses, described short-term mortality for patients who underwent CABG ranging from 1.9% to 11.7%. ^120^

## Burden of Disease

• The GBD 2019 estimated a rate of 1563 (95% UI, 1472-1636) DALYs lost per 100 000 individuals due to IHD (Table 3-3). This DALY rate was equivalent to 5.7% (95% UI, 5.1%-6.3%) of all DALYs, meaning that IHD was the second most common cause of DALYs in Brazil among females (after neonatal disorders) and males (after interpersonal violence) in 2019. ^46^

• From 1990 to 2019, the total number of DALYs attributable to IHD continuously increased, the crude DALY rate per 100 000 remained fairly stable, and the age-standardized DALY rate per 100 000 gradually declined 50% in the period (Table 3-3, Chart 3-11). ^46^

•When considering the Brazilian regions, the trend of age-standardized DALY rate from 1990 to 2019 resembles what was observed for mortality rates. In 1990, the highest rates were in the Southern and Southeastern regions, whereas the lowest rates were in the Northern and Northeastern regions. All states showed reductions in the age-adjusted DALY rate in the period, with variations of smaller magnitude in the Northern and Northeastern regions, so that the heterogeneity among the regions diminished (Chart 3-12). The states with the least pronounced decreases were Ceará (-16%), Paraíba (-25%), and Alagoas (-26%, Table 3-3). There was a negative correlation between the change in age-standardized DALY rate from IHD in the period and the SDI ( *r* ^2^0.59, p-value < 0.01, Chart 3-13). ^46^ ^,^ ^90^ In 2019, the lowest DALY rate was reported in the state of Amazonas (1106 per 100 000), whereas the highest rates were noted in the states of Maranhão (2157 per 100 000) and Pernambuco (2163 per 100 000, Table 3-3). ^46^

• Most of the DALYs associated with IHD were due to YLLs. The age-standardized rate of YLL was 1501 (95% UI, 1408-1574) per 100 000. These YLLs represented 9.1% (95% UI, 8.6%-9.6%) of all YLLs in 2019. This rate halved from 1990 to 2019, with a percent change of -50.8% (95% UI, -53.1% to -48.5%). ^46^

• The age-adjusted rate of YLDs attributable to CHD was 63 (95% UI, 41-89) per 100 000. From 1990 to 2019, this rate reduced by 5% (95% UI, -7% to -3%). ^46^

## Healthcare Utilization and Cost

• According to SIH/SUS administrative data, the absolute number of hospital admissions for ACS to public institutions remained stable from 2008 to 2019 (Chart 3-14). In 2019, there were 70 204 hospital admissions (33.4 per 100 000 inhabitants) due to ACS in the country. The number of hospitalizations due to AMI increased from 47 358 in 2008 to 80 614 in 2019 (percent change: 70%) or from 25.0 to 38.4 per 100 000 inhabitants (percent change: 54%, Chart 3-14). Hospital admissions due to CCSs decreased from 12 393 (6.5 per 100 000) in 2008 to 6703 (3.2 per 100 000) in 2019 (Chart 3-14). ^95^

•Hospital admissions in the public service for non-primary PCIs more than doubled from 2008 (n= 38 635) to 2019 (n= 85 518, Chart 3-15), while primary PCIs for AMI management increased by 45% (from 7 648 to 11 099). Considering rates per 100 000 inhabitants, the values for non-primary PCI were 20.4 in 2008 and 40.7 in 2019 (percent change: 100%) and those for primary PCI were 4.0 in 2008 and 5.3 in 2019 (percent change: 31%). Meanwhile, the total number of CABGs remained stable in the period (Chart 3-15), totalizing 21 018 procedures (10.0 per 100 000 inhabitants) in 2019. As a consequence, percutaneous myocardial revascularization increased as compared to surgery, from 69% of all procedures in 2008 to 82% in 2019 (Chart 3-16). ^95^

• Most PCIs performed in public hospitals in the last years were categorized as non-primary PCIs. The percentage of primary PCIs among all PCIs remained stable from 2009 to 2019 (between 9% and 13%). ^95^

• In 2019, the average lengths of hospital stay for ACS, AMI, PCI and CABG surgery in public hospitals were 5.4, 8.7, 3.8, and 12.0 days, respectively, remaining stable since 2008. ^95^

• According to the SIH/SUS, the unadjusted annual cost associated with all admissions due to ACS to public hospitals increased from R$ 44 710 681 in 2008 to R$ 81 167 005 in 2019. In the same period, the annual cost related to hospitalizations for AMI increased from R$ 65 019 331 to R$ 151 123 021, while the cost associated with CCSs decreased from R$ 7 798 578 to R$ 6 475 644. Converting to purchasing power parity-adjusted Int$ 2019, the amount associated with treatment for ACS has slightly decreased in recent years, reaching Int$ 39 230 065 in 2019; this expenditure has remained stable in the setting of AMI (Int$ 73 041 576 in 2019) and diminished for CCS (Int$ 3 129 842 in 2019, Chart 3-17). ^95^

•In international dollars, the average reimbursed amount per hospital admission due to ACS, AMI, or CCS decreased from 2008 to 2019 (Chart 3-18). In 2019, the average expenditures per admission were R$ 1156 (Int$ 559), R$ 1875 (Int$ 906), and R$ 966 (Int$ 467) for ACS, AMI, and CCS, respectively. ^95^

• The SUS administrative database showed that the total amount reimbursed for non-primary PCIs was R$ 546 132 199 (Int$ 263 959 497) in 2019. The correspondent cost for primary PCIs was R$ 74 907 756 (Int$ 36 204 812). The average expenditure paid per patient was R$ 6386 (Int$ 3087) for non-primary PCI and R$ 6749 (Int$ 3262) for primary PCI. Regarding CABG, the total amount reimbursed was R$ 278 544 224 (Int$ 134 627 464), corresponding to a mean value of R$ 13 253 (Int$ 6406) per surgical procedure. ^95^

•In international dollars, the total annual cost due to non-primary PCI increased in recent years, while the annual cost related to primary PCI remained stable and the CABG expenditure declined (Chart 3-19). The average amount reimbursed per hospital admission has decreased for both PCI and CABG since 2008 (Chart 3-20). ^95^

• A global modeling approach was performed in 2015 to assess the economic impact (health system and productivity) of four heart conditions in Brazil (hypertension, heart failure, AMI, and atrial fibrillation), providing estimates of the annual cost for the year 2015. The four heart conditions were estimated to affect ~45.7 million people in Brazil, corresponding to 32% of the adult population. AMI posed the greatest financial cost, with an estimated prevalence of 0.2% (334 978 cases), a health system cost per case of US$ 48 118, and a productivity cost of US$ 18 678. ^124^

• The annualized cost for an individual with chronic CHD was estimated to be around R$ 2733 ± 2307 by the SUS, with the outpatient cost being responsible for 54% of the total. For private insurance plans, the cost was estimated to be R$ 6788 ± 7842, with 69% of which related to inpatient costs. For outpatient costs, medications were responsible for R$ 1154, representing, for public and private payers, 77% and 55% of the outpatient costs and 42% and 17% of the total cost, respectively. ^125^

• In a report from a CHD clinic of a public hospital, the mean annual cost per outpatient was US$ 1521 (2015 currency). The mean cost per hospitalization was US$ 1976, and the expenses were higher in the first and last years of follow-up. Unstable angina, revascularization procedures, diabetes, hypertension, and obesity were predictors of higher hospitalization costs. ^126^

• Data from 2008 to 2014 estimated that 4 653 884 diagnostic tests were performed for CVD in Brazil, including 3 015 993 electrocardiograms, 862 627 invasive angiographies, and 669 969 nuclear tests, leading to an overall cost of US$ 271 million. In this national geospatial evaluation of health access, ACS mortality was associated with lower income, fewer nuclear tests, and an increase in exercise electrocardiogram tests and cardiac catheterization procedures. ^127^

• A study using micro costing methodology evaluated the costs associated with PCI in 40 patients from two public teaching hospitals in 2017. The median cost of PCI was R$ 4579 in one hospital and R$ 3156 in the other. Most of the expenditure was due to the prosthesis cost (72% and 81% of the total cost). ^128^

• In a quantitative, descriptive, and cross-sectional study carried out in a philanthropic hospital in the city of São Paulo, assessing 1913 consecutive patients who underwent CABG in 2012, the average cost per patient was US$ 7993 (median US$ 6463). The amount paid by the public health system was US$ 3450 (median US$ 3159), resulting in a deficit of 51% of the total cost for the providers. ^129^

• A retrospective analysis of medical claims of beneficiaries of health insurance plans was performed considering hospitalization costs for patients admitted with ACS between 2010 and 2012. The mean cost per patient on medical therapy only was R$ 18 262, for those undergoing PCI, R$ 30 611, and for those undergoing CABG, R$ 37 455. ^130^

• An analysis of 240 patients undergoing isolated CABG in a reference hospital in 2013 showed an average hospitalization cost of R$ 22 647 ± 28 106 (R$ 35 400 ± 40 509 for those with some complication and of R$ 13 997 ± 5 801 for those without complication). ^131^

• A cost analysis of 101 patients undergoing PCI at the SUS in 2014/2015 showed a median cost of R$ 6705 ± 3116 per patient. Costs were lower for elective PCI (R$ 5085 ± 16) than for PCI due to ACS (R$ 6854 ± 3396). ^132^

## Quality of Care

• Several publications addressed the quality of care in ACS in Brazil. ^82^ ^,^ ^83^ ^,^ ^85^ ^,^ ^86^ ^,^ ^96^ ^,^ ^100^ These studies highlight the opportunities for healthcare improvement, as well as the regional differences and heterogeneity of public and private services that may impact the outcomes as described above. Moreover, some publications reported on the implementation of strategies to optimize the quality of care in ACS. ^86^ ^,^ ^102^ ^,^ ^133^ ^,^ ^134^

• In the prospective registry ACCEPT, the rate of full adherence to guideline-recommended medications (dual antiplatelet therapy, parenteral anticoagulants, statins, and beta-blockers) was 62% soon after admission for ACS. Among patients with STEMI (n=1714), 82% were treated with either fibrinolysis or primary PCI. Rates of reperfusion therapies for AMI differed according to the region of the country: 87%, 85%, 73%, 67%, and 66% in the Southern, Southeastern, Northeastern, West-Central, and Northern regions, respectively. Acetylsalicylic acid was prescribed in 95% of the cases on discharge and 86% at 12 months of follow-up. P2Y12 receptor inhibitors were prescribed in 92% of the instances on admission, 79% on discharge, and 47% after 12 months. Statins were recommended to 93% of the patients on discharge and 83% after 12 months. Therapy considered incomplete and hospitalization in a public institution, among other factors, were associated with major cardiovascular events. ^82^

• In the BRACE cross-sectional analysis, the quality of care in ACS was measured by a performance score that included ASA prescription on hospital admission, ASA/beta-blocker/statin prescription on discharge, and reperfusion therapy to STEMI patients within 12 hours from symptom onset, among other factors. Importantly, lower scores were independently associated with a higher risk for hard outcomes and in-hospital death. The score was lower in the Northern and Northeastern regions than in the rest of the country both within the first 24 hours and on hospital discharge. Higher scores were observed in teaching *versus* non-teaching hospitals. No significant difference in the scores was detected between public and private institutions, although reperfusion therapy for STEMI was more frequent in private hospitals (86% *versus* 75%), while ASA/statin prescription on discharge was more common in public hospitals. Overall, the percentages of patients prescribed ASA, statin, and beta-blocker at discharge were 86%, 82%, and 69%, respectively. ^83^

• Among participants included in the ERICO study between 2009 and 2012, reperfusion therapy was performed in 72% of STEMI cases. The rates of medical treatment during hospitalization were as follows: 98% for ASA, 96% for clopidogrel and heparin, 92% for statins, and 84% for beta-blockers. ^85^

• In the VICTIM study, the mean time between symptom onset and hospital admission was longer in the context of the public service as compared to private hospitals (25 ± 37 *versus* 9 ± 21 hours; respectively, P <0.001). The rate of primary PCI was lower in public services than in private hospitals (45% *versus* 78%, respectively, P <0.001). ^96^

• In a university hospital in the city of Belo Horizonte, compliance with 13 pre-specified performance measures was evaluated in 1129 patients with STEMI or non-STEMI hospitalized between 2011 and 2014. The median compliance was 83% and the treatment of 67% of the patients reached at least 80% of the quality measures. The rate of reperfusion therapy in STEMI cases was 56%. ^100^

• In a study from the Minas Telecardio 2 Project, 593 patients with ACS were included between 2013 and 2014. Among individuals with STEMI, 46% received reperfusion therapy, mostly primary PCI. The door-to-balloon time was greater than 90 minutes in 37.5% of those patients. Overall, the rates of ASA, P2Y12 inhibitor, and statin prescriptions were 97%, 86%, and 81% within 24 hours, and 93%, 69%, and 86% on discharge, respectively. ^101^

• In another publication from the Minas Telecardio Project 2, the quality of care for STEMI was evaluated before (n = 214) and after (n = 143) the implementation of a coordinated management protocol that took place between 2014 and 2015. The rate of reperfusion therapy increased from 71% to 81% (P = 0.045), while the percentage of patients being prescribed ASA and P2Y12 inhibitors increased from 94% and 88%, respectively, to 100% in both cases. In addition, a non-significant decrease in the odds of in-hospital death was reported (OR, 0.73; 95% CI, 0.34–1.60). ^133^

• In a retrospective observational study of public services in the city of Belo Horizonte, the in-hospital mortality rate reduced after the implementation of an AMI management system, including tele-electrocardiography (12% in 2009 and 7% in 2011, P < 0.001). ^134^

• The RESISST study reported outcomes in STEMI before and after the implementation of an integrated regional network supported by telemedicine in the city of Salvador, Bahia state. The authors reported an increase in the rates of primary reperfusion (from 29% to 54%, P <0.001), a decrease in the 30-day mortality rate (from 20% to 5%, P <0.001), and an increase in the use of dual antiplatelet therapy and statins. ^102^

• The BRIDGE-ACS trial was a cluster-randomized study that evaluated a multifaceted quality improvement program for the treatment of patients with ACS. Compared to routine practice, the intervention, which included educational materials for clinicians, reminders, algorithms, and case manager training, increased the odds of receiving eligible medications. Moreover, the rates of in-hospital cardiovascular event were 5.5% in the intervention group and 7.0% in the control group (population average OR [ORPA], 0.72 [95% CI, 0.36-1.43]), whereas the 30-day all-cause mortality rates were 7.0% and 8.4% in the intervention and control groups, respectively (ORPA, 0.79 [95% CI, 0.46-1.34]). ^86^

## Future Research

• Additional data are needed for further understanding of the epidemiological distribution of CHD in Brazil, in particular:

1. development of nationwide databases aiming at gathering accurate and real-time information on the epidemiology of distinct clinical presentations of CHD, including delivery of care, performance, and outcome measurements;

2. systematic reviews of prevalence and mortality rates of ACS, stable angina, PCI, and CABG, including representative samples of all geographical areas of the country;

3. assessment of the effectiveness of structured nationwide programs for quality and performance measurement of different providers (public, non-for-profit, and for-profit) to understand the current situation, as well as for designing strategies aimed at reducing CVD morbidity and mortality;

4. additional economic and cost-effectiveness analyses of the impact of CHD and its diagnostic and therapeutic interventions, from a macro level and using micro costing methods for both the public and private healthcare systems;

5. development of structured programs to assess the prevalence, incidence, and clinical and economic impact of chronic CHD in the outpatient setting.

## 4. CARDIOMYOPATHY AND HEART FAILURE

### ICD-10 I42; I50; B57.2

**See Tables 4-1 through 4-12 and Charts 4-1 through 4-10**

**Table:** Abbreviations Used in Chapter 4

BREATHE	Brazilian Registry of Heart Failure
ChCM	Chagas Cardiomyopathy
ChD	Chagas Disease
CI	Confidence Interval
DALY	Disability-Adjusted Life Years
FU	Federative Unit
GBD	Global Burden of Disease
HCM	Hypertrophic Cardiomyopathy
HF	Heart Failure
HF-PEF	Preserved Ejection Fraction Heart Failure
HF-REF	Reduced Ejection Fraction Heart Failure
HR	Hazard Ratio
ICD-10	International Statistical Classification of Diseases and Related Health Problems, 10^th^Revision
IQR	Interquartile Range
NChC	Non-Chagas Cardiomyopathies
OR	Odds Ratio
PAR	Population Attributable Risk
REMADHE	Repetitive Education at Six-Month Intervals and Monitoring for ADherence in Heart Failure Outpatients trial
SDI	Sociodemographic Index
SEADE	Data Analysis State System Foundation (in Portuguese, *Fundação Sistema Estadual de Análise de Dados* )
SIM	Brazilian Mortality Information System (in Portuguese, *Sistema de Informações sobre Mortalidade* )
SUS	Brazilian Unified Health System (in Portuguese, *Sistema Único de Saúde* )
UI	Uncertainty Interval
US	United States of America
YLD	Years Lived with Disability
YLL	Years of Life Lost

**Table 4-1:** – Number of prevalence cases, age-standardized prevalence rates (per 100 000) of cardiomyopathy and myocarditis, and percent change of rates, in Brazil and its Federative Units, 1990 and 2019.

Location	1990		2019		Percent Change (95% UI)
	Number (95% UI)	Rate (95% UI)	n (95% UI)	Rate (95% UI)	
Brazil	55954.8 (38550.4;80474.4)	76.6 (53.4;107.2)	164904.6 (115339.9;226269.4)	73 (51.1;100.1)	-4.7(-9.5;0.8)
Acre	77.6 (51.6;113)	59.8 (41.8;84.7)	292.2 (203.7;411.2)	55.7 (39.2;77.4)	-6.9(-20.7;9.3)
Alagoas	893 (594.6;1319.1)	78.1 (54;111.5)	2230.7 (1576.7;3140.2)	75 (52.4;105.5)	-4.1(-17.2;11.5)
Amapá	53.5 (36.2;77.7)	68.9 (48.4;97)	273.7 (192.5;385.7)	64.4 (45.2;89.3)	-6.5(-19.7;8.1)
Amazonas	350.6 (236.1;515.3)	60.3 (42.1;85.6)	1454.6 (1014.7;2010.9)	58.1 (40.5;81)	-3.6(-17;11.8)
Bahia	3814.2 (2517.2;5533.9)	64.4 (43.6;91.4)	10094 (7121;14112.7)	63.7 (44.6;90.4)	-1.1(-16.6;16.1)
Ceará	3069.9 (2131.5;4409.4)	80.8 (56.5;114.9)	7835.9 (5565.8;10891.2)	80.5 (56.9;111.8)	-0.4(-13.8;15.1)
Distrito Federal	272.9 (165.7;416.5)	78.5 (52.8;112.3)	1545.6 (995;2290.9)	72.7 (48.6;102.9)	-7.3(-20;6.7)
Espírito Santo	791.4 (533;1142.4)	66.3 (46;94.8)	2501.4 (1761.3;3478.5)	61 (43;84.1)	-8(-20.4;6.7)
Goiás	1075.7 (651.2;1638)	74.8 (47.6;108.2)	4744.9 (3123.1;6832)	77.9 (51.8;110.8)	4.2(-9.8;20.3)
Maranhão	1059.9 (715.7;1564.1)	48.1 (33.1;68.5)	3064.9 (2111.4;4342)	49.1 (33.7;69.3)	2.2(-13.4;21.1)
Mato Grosso	351.2 (234.6;519.3)	65.6 (46;93)	1782.3 (1254;2514)	62.2 (44.1;87.1)	-5.1(-18.9;11.9)
Mato Grosso do Sul	518.2 (340.8;741.9)	77.6 (53.1;110.1)	2082 (1444.7;2891.3)	76.7 (53.4;105.2)	-1.1(-15.4;14.6)
Minas Gerais	6511.9 (4300.2;9654.8)	82.8 (56.3;117.4)	20996.2 (14121.1;29608.6)	80.2 (53.7;113.4)	-3.1(-15.6;11.8)
Pará	984.4 (668;1427.3)	59.5 (41.7;83.5)	3612.3 (2515.8;5033)	57.8 (40.4;80.2)	-3(-17.1;12.7)
Paraíba	2080.3 (1423.6;2989.5)	94 (65;132)	4211.6 (2938.5;5780.8)	86.6 (60;119.7)	-7.9(-19.8;5.9)
Paraná	2664.2 (1754.8;3847.1)	71.5 (49.2;100.3)	8066.4 (5604.5;11456.8)	64.4 (45.1;90.7)	-9.9(-21.9;4.4)
Pernambuco	2580.1 (1749.8;3669.6)	64.6 (44.8;90.5)	5776.7 (4013.6;8097.6)	61.1 (42.4;85.4)	-5.5(-19.1;9.3)
Piauí	636.4 (430.3;916.8)	54.2 (37.3;76.4)	1883.9 (1312;2665.4)	49.8 (34.7;70.9)	-8.2(-21.6;8.2)
Rio de Janeiro	6696.9 (4702.4;9614.9)	83.2 (58.6;115.6)	16891.5 (11678.1;23485.5)	76.5 (53;105.9)	-8.1(-20;5.6)
Rio Grande do Norte	902.7 (620.2;1261.7)	59.7 (41.7;82.6)	2195.4 (1530.2;3005.3)	56.9 (39.5;79.2)	-4.8(-18.6;11.8)
Rio Grande do Sul	3317.1 (2244.1;4733.2)	60.5 (42.4;85.4)	8432.1 (5886.6;11731.4)	53.8 (37.8;74.4)	-11.1(-23.5;3.9)
Rondônia	111.4 (68.3;174.7)	57 (39.6;80.7)	705.9 (490.3;1010.1)	53.2 (37.2;74.8)	-6.6(-20.8;10.9)
Roraima	34 (22;50)	94.5 (65.8;133.1)	254.9 (173.2;359.1)	84.8 (57.9;118.4)	-10.3(-22.8;3.5)
Santa Catarina	1501.3 (1036.2;2151.8)	74.6 (52.4;103.4)	5218.7 (3607.9;7226.4)	68.7 (47.8;95.2)	-7.9(-19.6;6.2)
São Paulo	14932.1 (10004;21456.5)	94.1 (64.2;132.4)	46530 (32481.2;63983.6)	89.2 (63.1;122.9)	-5.2(-17.1;9.4)
Sergipe	513.3 (343.1;744.4)	75.3 (52.4;104.8)	1526.3 (1062.2;2129.8)	72.8 (51;102.2)	-3.2(-15.8;11.3)
Tocantins	160.6 (102.1;248.9)	54.4 (37;78.4)	700.4 (467.8;1009)	53.9 (36;77.6)	-0.9(-15.2;15.5)

*Source: Data derived from Global Burden of Disease Study 2019, Institute for Health Metrics and Evaluation, University of Washington. ^46^*

**Table 4-2:** – Number of incidence cases, age-standardized incidence rate (per 100 000) of cardiomyopathy and myocarditis, and percent change of rates, in Brazil and its Federative Units, 1990 and 2017.

Location	1990		2017		Percent Change (95% UI)
	Number (95% UI)	Rate (95% UI)	n (95% UI)	Rate (95% UI)	
Brazil	54520.4 (48574.3;61320.7)	46.3 (41.5;52.1)	103879.4 (92495.6;117294.5)	46.7 (41.8;52.6)	0.8 (-0.3;1.8)
Acre	125.8 (111;143)	44.8 (40;50.7)	340.7 (303.3;383.1)	46 (41.1;52)	2.8 (-0.5;6.4)
Alagoas	826.5 (732.4;929.4)	43.6 (38.8;49.3)	1551.4 (1378.8;1749)	46.5 (41.5;52.4)	6.8 (3.5;10.2)
Amapá	85.4 (75.2;97.2)	47.5 (42.5;53.4)	311.1 (274.3;352.6)	47.6 (42.5;53.6)	0.3 (-2.8;3.9)
Amazonas	645.3 (565.6;732.6)	46.4 (41.5;52.6)	1586.5 (1410.7;1793.3)	46.6 (41.6;52.8)	0.5 (-2.9;4.3)
Bahia	4183 (3713.7;4708.9)	45.3 (40.3;50.9)	7177 (6366.6;8059.8)	45.1 (40;50.7)	-0.3 (-3.4;2.7)
Ceará	2232.8 (1990.7;2511.3)	43.2 (38.7;48.7)	4589.2 (4084.6;5197.2)	46.4 (41.4;52.7)	7.5 (3.4;11)
Distrito Federal	502.7 (439.4;574.4)	45.2 (40.3;51.3)	1258.3 (1107.5;1428.3)	45.3 (40.4;51)	0.2 (-3.3;3.7)
Espírito Santo	976.1 (856.5;1104.3)	48.1 (42.8;54.4)	1992.5 (1757;2263.2)	48.2 (42.9;54.4)	0.2 (-3.2;3.8)
Goiás	1391.1 (1220.9;1574.2)	46.3 (41.4;52.2)	3233.1 (2869.8;3671.6)	47.3 (42.2;53.5)	2.3 (-0.8;5.4)
Maranhão	1568.5 (1390.4;1771.6)	43.3 (38.6;48.9)	3314.6 (2958.6;3716.8)	46.7 (41.7;52.6)	8 (4.5;12)
Mato Grosso	628.1 (549.8;715.5)	45.6 (40.7;51.7)	1567.2 (1394.8;1780.2)	46.8 (41.9;52.9)	2.7 (-1;6.3)
Mato Grosso do Sul	648.6 (572.4;728)	48.6 (43.4;54.8)	1426.5 (1264.1;1616.6)	50.6 (45.2;57)	4.1 (0.8;7.7)
Minas Gerais	5880.7 (5207.1;6646.3)	46 (41;52)	11181 (10003.5;12646.6)	47 (42.2;53.2)	2.2 (-1;5.5)
Pará	1548.2 (1365.8;1749.4)	45.4 (40.6;51.3)	3561 (3155.4;4003.7)	45.5 (40.5;51.4)	0.2 (-3.2;3.9)
Paraíba	1181.3 (1051.4;1331.4)	43.2 (38.6;48.9)	2068.4 (1841.2;2347.2)	46.3 (41.2;52.5)	7.1 (3.6;10.5)
Paraná	3140.9 (2777.5;3572.6)	47.1 (41.9;53.3)	5728 (5060.1;6535.4)	47 (41.9;53.2)	-0.2 (-3.8;3)
Pernambuco	2639 (2333.6;2961.8)	45.6 (40.5;51.3)	4609.7 (4098.5;5160.5)	46.1 (41.1;51.6)	1.1 (-2.4;4.5)
Piauí	865.4 (765.7;974.5)	43.8 (39;49.4)	1652.7 (1471;1863.8)	45.8 (40.7;51.6)	4.5 (0.8;8.3)
Rio de Janeiro	5321.9 (4706.8;6019.7)	46.7 (41.6;52.5)	9236.1 (8183.4;10524.9)	46.4 (41.3;52.5)	-0.5 (-4.1;3.1)
Rio Grande do Norte	886.2 (790.4;995.2)	44.3 (39.7;49.8)	1723.7 (1535.1;1947.9)	46.2 (41.2;52.1)	4.2 (1;7.7)
Rio Grande do Sul	3927.6 (3482.4;4424.5)	49.5 (44.1;55.8)	6475.7 (5749;7325.7)	48.5 (43.2;54.5)	-2.1 (-5.5;1.4)
Rondônia	330.5 (289.3;379.1)	45.6 (40.6;51.4)	746.3 (660.9;847.2)	46 (41.2;52.2)	0.9 (-2.6;4.4)
Roraima	61.2 (53.3;70.9)	46.1 (41.2;52)	212.3 (187.3;240.4)	46.4 (41.3;52.4)	0.9 (-2.4;4.2)
Santa Catarina	1694 (1503.4;1912.2)	47.8 (42.9;54.2)	3554.7 (3148.4;4033.5)	47.5 (42.4;53.4)	-0.7 (-4.2;3.5)
São Paulo	12421.8 (10980.5;13984.7)	47.6 (42.4;53.5)	22999.9 (20352.4;26154.9)	46.6 (41.6;52.7)	-1.9 (-5.5;1.4)
Sergipe	520.8 (463.6;584.1)	45.3 (40.5;51.4)	1075.4 (957.1;1218.6)	47.3 (42.1;53.4)	4.5 (1;7.8)
Tocantins	287 (253.9;325.8)	44.8 (40;50.5)	706.6 (629;798)	47.1 (42.1;53.1)	5.1 (1.6;8.9)

*Source: Data derived from Global Burden of Disease Study 2019, Institute for Health Metrics and Evaluation, University of Washington. ^152^*

**Table 4-3:** – Incidence rates of cardiomyopathy and myocarditis (per 100 000) and percent change of rates, by age and sex, Brazil, 1990 and 2019.

Sex and age group	1990	2019	Percent Change (95% UI)
**Both sexes**			
15-49 years	10,9 (7,7;14,8)	11,5 (8,1;15,9)	5,3 (-1,3;12,9)
50-69 years	26 (17,2;35,7)	26,1 (17,1;36)	0,4 (-0,4;1)
5-14 years	6.8 (3,8;10,8)	6,8 (3,8;10,8)	0,3 (-0,1;0,9)
70+ years	65,5 (43,8;94,9)	68,7 (47,3;96,9)	4,9 (-0,4;11,8)
Age-standardized	15,8 (12,7;19,.2)	15,8 (12,7;19,2)	-0,2 (-0,3;-0,2)
All Ages	12,5 (10;15,3)	16,6 (13,4;20,2)	32,6 (22,7;42,9)
Under 5	5.9 (3,6;8,5)	5,9 (3,6;8,5)	0,1 (0,1;0,2)
**Female**			
15-49 years	9,1 (6,4;12,6)	9,6 (6,8;13,3)	5,4 (-1,5;13,3)
50-69 years	21,3 (14;29,4)	21,5 (14,1;29,.8)	0,9 (0;1,8)
5-14 years	5,6 (3,2;9,1)	5,6 (3,2;9,1)	0,2 (-0,2;0,7)
70+ years	56,8 (38,4;82,6)	60,5 (41,7;83,6)	6,6 (0,6;14,1)
Age-standardized	13,2 (10,6;16,1)	13,2 (10,6;16,1)	-
All Ages	10,6 (8,4;13,1)	14,5 (11,6;17,8)	36,1 (25,6;47,3)
Under 5	4,8 (2,9;7,1)	4,8 (2,9;7,1)	-
**Male**			
15-49 years	12,7 (9;17,2)	13,4 (9,5;18,3)	5,1 (-1,2;12,2)
50-69 years	31 (20,3;42,9)	31,2 (20,5;43,2)	0,6 (0;1,1)
5-14 years	7,9 (4,5;12,5)	7,9 (4,5;12,5)	0,2 (-0,2;0,8)
70+ years	76,3 (50,4;111)	80 (54,3;114,6)	4,7 (0;10,.8)
Age-standardized	18,6 (15,1;22,7)	18,6 (15,1;22,7)	-
All Ages	14,4 (11,5;17,6)	18,7 (15;22,9)	30,2 (20,7;40,1)
Under 5	7 (4,2;10)	7 (4,2;10)	-

So *urce: Data derived from Global Burden of Disease Study 2019, Institute for Health Metrics and Evaluation, University of Washington.* ^*46*^

**Table 4-4:** – Number of deaths, age-standardized mortality rates (per 100 000) of cardiomyopathy and myocarditis, and percent change of rates, in Brazil and its Federative Units, 1990 and 2019.

Location	1990		2019		Percent Change (95% UI)
	Number (95% UI)	Rate (95% UI)	n (95% UI)	Rate (95% UI)	
Brazil	13408.9 (10490.2;14283.2)	15.9 (12.4;17.1)	21425.7 (18940;25167.1)	9.4 (8.3;11.1)	**-40.8 (-46.4;-25.4)**
Acre	17.8 (15.6;22.3)	11.1 (9.7;14.5)	40.6 (32.9;66)	7 (5.6;11.5)	-37.3 (-50.5;-10.7)
Alagoas	252 (186.1;296.6)	17.2 (12.7;20.2)	338.7 (289.2;405.6)	10.6 (8.9;12.8)	-38.7 (-51.1;-10.7)
Amapá	8.7 (7.6;11.1)	9.2 (8;11.7)	35 (29.5;49.9)	7 (5.8;10.1)	-24.3 (-36.3;-6.6)
Amazonas	76.9 (68.3;96.6)	10.8 (9.5;13.3)	160.4 (126.1;276.9)	5.6 (4.3;9.9)	-48 (-59.3;-20.6)
Bahia	945.3 (757.8;1076.4)	13.8 (11;15.9)	1209.7 (947.1;1895)	7.5 (5.8;11.7)	-45.9 (-59.6;-6.3)
Ceará	544.6 (443.7;645.1)	11.9 (9.6;14.2)	862 (699.5;1105.8)	8.7 (7.1;11.2)	-26.8 (-44.4;0.5)
Distrito Federal	115.1 (71.8;134.1)	23.8 (13.8;27.9)	262.2 (177.5;309.1)	13 (8.6;15.5)	-45.2 (-54.4;-33.3)
Espírito Santo	186.7 (162.6;203.2)	14.1 (12.2;15.4)	329.7 (270;483.2)	7.9 (6.4;11.7)	-43.9 (-54.6;-15.5)
Goiás	533 (287.6;636.2)	29.8 (15;35.7)	824.6 (614.7;999.2)	12.7 (9.4;15.4)	-57.2 (-66.1;-34.2)
Maranhão	262.1 (178.7;422)	7.3 (5;13.4)	472.7 (356.7;908.9)	7 (5.2;13.8)	-3.9 (-31.6;35.7)
Mato Grosso	103.7 (83.5;119.2)	14.3 (11.3;16.4)	234.8 (199.8;318.2)	7.7 (6.5;10.4)	-46.3 (-56.3;-21.7)
Mato Grosso do Sul	157.5 (107.9;175.7)	19.9 (13.3;22.4)	264.2 (223.8;343.1)	9.6 (8.1;12.2)	-51.9 (-61;-22.6)
Minas Gerais	1742.7 (1144.9;1958.6)	19.3 (12.8;21.8)	2284.8 (1929.8;2701.6)	8.9 (7.5;10.5)	-54.1 (-62;-33)
Pará	201.4 (175.6;259.7)	10.4 (9.1;13.2)	407.4 (322.7;677.1)	5.9 (4.6;9.8)	-43.5 (-54.9;-19.1)
Paraíba	363.4 (244.6;426.7)	15.4 (10.3;18.2)	522.5 (421.5;611.6)	10.7 (8.7;12.5)	-30.2 (-43.9;-7.1)
Paraná	753.1 (572.7;822.2)	17.7 (13.1;19.5)	965.5 (791.3;1393.5)	7.9 (6.5;11.2)	-55.3 (-64.4;-27.5)
Pernambuco	563.7 (492;634.8)	12.9 (11.2;14.6)	906.9 (766.5;1203.7)	9.3 (7.8;12.5)	-28.2 (-39.6;-6.6)
Piaui	169.9 (145.7;201.3)	11.7 (9.8;14.5)	237.8 (187.3;387.2)	6.2 (4.9;10)	-46.9 (-59.7;-22.1)
Rio de Janeiro	1214.1 (1020.8;1481.7)	13.9 (11.9;16.8)	2858.5 (1949.9;3341.5)	13.2 (9.1;15.4)	-4.8 (-38.5;13)
Rio Grande do Norte	153.2 (132.1;193.4)	9.2 (7.8;11.9)	224.3 (167;392)	5.7 (4.2;9.8)	-38.6 (-53.8;-9.5)
Rio Grande do Sul	586.9 (507.9;899.2)	10 (8.6;15.1)	931.9 (733;1581.7)	6.2 (4.9;10.5)	-37.6 (-47.5;-21.5)
Rondônia	40.6 (33.5;51.5)	15.5 (12.9;18.2)	105.9 (84.9;156.8)	7.4 (6;11)	-52.3 (-62.9;-30.9)
Roraima	11.4 (7.5;13.3)	24.2 (15.4;28.1)	43.6 (32.8;49.3)	13.9 (10.6;16)	-42.4 (-51.6;-23.7)
Santa Catarina	351.2 (292.7;387.2)	16.2 (12.8;18)	598.5 (506;823.3)	8.1 (6.8;10.9)	-50 (-58.9;-30.3)
São Paulo	3890.4 (2446.4;4363.8)	22 (13.2;24.9)	6025.5 (4305.2;7018)	11.9 (8.5;13.8)	-46 (-53.6;-31)
Sergipe	109 (93.2;123)	15 (12.6;17.2)	163.4 (130.9;243.4)	7.4 (5.9;11.1)	-50.7 (-62.1;-21.5)
Tocantins	54.6 (43;63.8)	15.2 (11.6;18)	114.6 (94.2;159.5)	8.3 (6.8;11.7)	-45.2 (-57.4;-12.6)

*Source: Data derived from Global Burden of Disease Study 2019, Institute for Health Metrics and Evaluation, University of Washington. ^46^*

**Table 4-5:** – Mortality rates of cardiomyopathy and myocarditis (per 100 000) and percent change of rates, by age and sex, Brazil, 1990-2019.

Sex and age group	1990	2019	Percent Change (95% UI)
**All**			
Age-standardized	15.9 (12.4;17.1)	9.4 (8.3;11.1)	-40.8 (-46.4;-25.4)
Under 5	5.7 (4.5;7.2)	2.5 (2;3.1)	-55.5 (-68.3;-37.2)
5-14 years	0.5 (0.4;0.5)	0.3 (0.3;0.4)	-24.9 (-39.7;-9.3)
15-49 years	3 (2.5;3.2)	2.5 (2;2.8)	-17.3 (-25.6;-7.7)
50-69 years	29.2 (22;31.4)	17.2 (15.1;20)	-41 (-47.2;-25.7)
70+ years	127.2 (96.5;138.3)	84.6 (72.1;104)	-33.5 (-42;-10)
All Ages	9 (7;9.6)	9.9 (8.7;11.6)	9.8 (-1.4;37.4)
**Female**			
Age-standardized	13.5 (9.9;14.7)	7.2 (6.2;9)	-46.6 (-54.8;-17.5)
Under 5	5.8 (3.9;7.3)	2.3 (1.8;2.8)	-60.5 (-71.2;-36.2)
5-14 years	0.4 (0.4;0.5)	0.3 (0.2;0.4)	-30.9 (-42.1;-9.8)
15-49 years	2 (1.5;2.1)	1.4 (1.2;1.5)	-30.6 (-39.3;-11)
50-69 years	21.9 (14.8;24.2)	10.9 (9.5;13.6)	-50.1 (-58.5;-20.8)
70+ years	118.7 (85.2;131.5)	76.9 (63;97.4)	-35.2 (-46;4.4)
All Ages	7.8 (5.7;8.4)	8.3 (7.1;10.3)	5.6 (-10.9;64.1)
**Male**			
Age-standardized	18.7 (13.2;20.4)	12.1 (10;15.1)	-35.4 (-43.3;-5.8)
Under 5	5.6 (3.8;7.7)	2.8 (2;3.5)	-50.6 (-68.7;-20.8)
5-14 years	0.5 (0.4;0.6)	0.4 (0.3;0.5)	-19.9 (-41.6;4)
15-49 years	4.2 (3.2;4.6)	3.7 (2.7;4.2)	-11 (-26.7;2.7)
50-69 years	37 (25.8;40.4)	24.3 (19.5;28.9)	-34.3 (-42.7;-7.6)
70+ years	137.9 (94;152.9)	95.2 (78.7;127.8)	-31 (-42;9.4)
All Ages	10.2 (7.3;11.1)	11.6 (9.5;14.3)	13.4 (-0.7;63.1)

*Source: Data derived from Global Burden of Disease Study 2019, Institute for Health Metrics and Evaluation, University of Washington.* ^46^

**Table 4-6:** – Number of DALYs, age-standardized DALY rates (per 100 000) due to cardiomyopathy and myocarditis, and percent change of rates, in Brazil and its Federative Units, 1990 and 2019.

Location	1990		2019		Percent Change (95% UI)
	Number (95% UI)	Rate (95% UI)	n (95% UI)	Rate (95% UI)	
Brazil	431381.3 (349546.5;465068.6)	399.4 (319.6;426.4)	545772.4 (484988.7;621356.8)	238.6 (213;272.3)	-40.3(-45.8;-27.9)
Acre	722.8 (619.6;863.1)	272.1 (238.3;338.1)	1229.2 (1012.3;1861.1)	172.3 (141.3;266.4)	-36.7(-48.7;-14)
Alagoas	10120.9 (7613.2;12457.1)	491.5 (368.6;575.8)	9795 (8429.4;11297.2)	287.2 (248.3;330.5)	-41.6(-53.2;-22.6)
Amapá	297.3 (252.1;377.5)	209.2 (183.3;265)	1082.7 (939.5;1485.3)	174.1 (150.6;240.6)	-16.8(-28.8;-0.8)
Amazonas	2624.3 (2276.7;3320.4)	238.6 (212.3;300.2)	4833.5 (3967;7682.5)	144.2 (117.3;234.9)	-39.6(-50.3;-16.2)
Bahia	31147.8 (25438.5;35521.1)	360.6 (285.8;411.6)	33834.2 (27283.1;46340)	211.1 (169.7;287.7)	-41.5(-55.2;-10.8)
Ceará	16413.7 (13000.4;20157.3)	260.6 (210.1;309.8)	18369.4 (16682.7;20144.2)	188.2 (170.6;206.9)	-27.8 (-41.3;-10.1)
Distrito Federal	22092.3 (17022.6;28315.1)	375.8 (300.5;461.9)	23436.2 (19481.4;28927.2)	232.7 (193.4;287)	-38.1(-53.2;-18.4)
Espírito Santo	4337.8 (2752.8;5019.5)	513.1 (321;593.1)	7071.6 (4676.3;8364.2)	264.3 (180.3;309.9)	-48.5(-56.3;-37.4)
Goiás	5815.7 (5216;6419.7)	326.8 (289.2;356)	8951.2 (7601.8;12062.7)	209 (177.8;281.7)	-36(-46.3;-14.9)
Maranhão	16299.6 (9819.3;19412.6)	652.4 (364.1;778.8)	20523.4 (15577.5;24450)	294.8 (224.3;350.1)	-54.8(-64.3;-32.3)
Mato Grosso	14600.5 (9341.4;21713)	279.7 (193.3;432.5)	13913.1 (10913.9;22240.9)	185.5 (144.9;308)	-33.7(-56.5;-3.8)
Mato Grosso do Sul	3653.5 (2937.1;4296.1)	329.9 (267.5;377.3)	6406.5 (5543;8476.1)	188.5 (163.6;246.6)	-42.9(-53.3;-20.3)
Minas Gerais	4885.5 (3587.2;5423.8)	446.6 (313.4;496.6)	6646 (5713;8721.8)	227 (196.5;295.2)	-49.2(-58;-23.3)
Pará	6504.4 (5594.8;8556.8)	234.7 (203.8;304.6)	11934.4 (9785.4;18602.1)	154.4 (126.4;242)	-34.2(-46.7;-11.4)
Paraíba	12024.3 (8553.7;14204.9)	431.4 (300.4;502.6)	12785.5 (10190.5;14695.4)	275.2 (220.1;316.3)	-36.2(-47.4;-20.6)
Paraná	22264.3 (17934.9;23976.9)	397.2 (309.5;430.3)	22812.7 (18990.6;32682.4)	182.6 (152.5;255.5)	-54(-62.8;-27.9)
Pernambuco	18399.6 (15769.6;21268.1)	329.9 (284.7;372)	25233 (21518.1;30713.1)	247.5 (212.1;302.5)	-25(-36.1;-11.2)
Piauí	6969.2 (5802.9;8446.6)	324.4 (276.8;385.1)	6207.9 (5130.4;9242.6)	165.6 (137;246.5)	-49(-61.3;-27.4)
Rio de Janeiro	38312.2 (31811.2;46419.8)	357.1 (300.2;427.9)	71716 (46879.8;84553.9)	339.7 (225.5;397.7)	-4.9(-40.6;13.4)
Rio Grande do Norte	5015.5 (4362.7;6077.7)	245.3 (213.5;305.8)	5882.6 (4598.4;9395.7)	152.6 (119.7;243.4)	-37.8(-51.4;-15.2)
Rio Grande do Sul	16956.7 (14758;25507.2)	235.3 (205.2;353.1)	20924.4 (16731.2;34591.4)	148.7 (120.1;241)	-36.8(-45.4;-23.2)
Rondônia	1570 (1255.2;2025)	320.2 (271.4;395.1)	2888.2 (2353.2;4117.1)	178.6 (146.2;255.3)	-44.2(-55.8;-22.1)
Roraima	426.7 (289.1;506.9)	490.8 (321.1;566.9)	1273.9 (964.2;1442)	295.1 (225;332.4)	-39.9(-49;-21.5)
Santa Catarina	10131 (9082.5;11640.1)	350.2 (302.4;387.2)	14421.3 (12268.6;20029.6)	185.8 (158.6;254.9)	-47(-55.1;-30.5)
São Paulo	116500.2 (79828.7;128970.3)	509.1 (334.8;567.6)	149627 (103165.1;174050.1)	294.5 (205.5;340.4)	-42.1(-49.3;-30.3)
Sergipe	3629 (3125.9;4094)	351.7 (303.7;394.9)	4389.6 (3599.6;6196.4)	190.2 (156.5;267.7)	-45.9(-57.3;-21)
Tocantins	2297.9 (1812;2752)	362.6 (285.6;424.6)	3071.7 (2577.8;3989.1)	204.4 (171.7;268.2)	-43.6(-55.4;-16.6)

*Source: Data derived from Global*

**Table 4-7:** – Rates of DALYs due to cardiomyopathy and myocarditis (per 100 000) and percent change of rates, by age and sex, Brazil, 1990 and 2019.

Age group and sex	1990	2019	Percent Change (95% UI)
**All**			
Age-standardized	399.4 (319.6;426.4)	238.6 (213;272.3)	-40.3 (-45.8;-27.9)
Under 5	498.4 (398.1;630.1)	222.3 (171.8;275.3)	-55.4 (-68.1;-37)
5-14 years	37.1 (31.7;41.3)	27.9 (22.7;32.8)	-24.7 (-39.2;-9.3)
15-49 years	156.7 (127.9;166.9)	127.2 (102;139.6)	-18.8 (-26.7;-9.9)
50-69 years	865.7 (657.8;930.7)	516.7 (455.5;593.5)	-40.3 (-46.6;-25.6)
70+ years	1841.8 (1402.3;2004.5)	1122.6 (969.8;1383.9)	-39.1 (-46.4;-18.4)
All Ages	289.8 (234.9;312.5)	251.9 (223.8;286.8)	-13.1 (-22.1;5.3)
**Female**			
Age-standardized	321.1 (237.7;346.2)	165.7 (147.3;198)	-48.4 (-55;-21.6)
Under 5	504.1 (343.7;638.6)	199.6 (160;250)	-60.4 (-71.2;-36)
5-14 years	34.7 (29.5;39.3)	24.1 (20.1;29)	-30.6 (-41.7;-9.8)
15-49 years	101.3 (78.6;109.6)	68.8 (61.5;77.4)	-32.1 (-40.4;-13.7)
50-69 years	644.9 (441.8;712.1)	324.8 (283.4;403.2)	-49.6 (-57.7;-21.2)
70+ years	1651.5 (1165.2;1824.5)	953.5 (799;1213.2)	-42.3 (-51.4;-8.7)
All Ages	237.7 (176.4;258.6)	181.1 (160.3;216.3)	-23.8 (-34.2;14.6)
**Male**			
Age-standardized	484.8 (353.6;528.5)	320.4 (257.7;371.8)	-33.9 (-41.8;-9.6)
Under 5	492.9 (334.4;675.3)	244.1 (181;309.8)	-50.5 (-68.6;-20.5)
5-14 years	39.5 (31.2;46.4)	31.6 (23;38.5)	-19.9 (-41.1;3.6)
15-49 years	213.9 (163.3;233.8)	187.1 (137.3;210.2)	-12.5 (-27.6;0)
50-69 years	1105.3 (785.4;1210.3)	733.9 (585.9;861.6)	-33.6 (-41.9;-8)
70+ years	2078.5 (1430.3;2306.7)	1353.1 (1144.9;1801.6)	-34.9 (-44.9;1)
All Ages	343.1 (255.5;380.3)	326.1 (260.4;376.9)	-5 (-17.6;28.2)

*Source: Data derived from Global Burden of Disease Study 2019, Institute for Health Metrics and Evaluation, University of Washington.^*46*^*

**Table 4-8:** – Number of deaths, age-standardized mortality rates (per 100 000) of Chagas disease, and percent change of rates, in Brazil and its Federative Units, 1990 and 2019.

Location	1990		2019		Percent change (95% UI)
	Number of deaths (95% UI)	Mortality Rate (95% UI)	Number of deaths (95% UI)	Mortality Rate (95% UI)	
Brazil	7903.9 (2438.4;10073)	8.6 (2.8;10.9)	6523.3 (3350.2;11226.8)	2.8 (1.4;4.8)	-67.5(-76.5;-0.4)
Acre	6.4 (3.6;18.3)	4 (2.3;11.4)	10.9 (5.1;31.4)	1.8 (0.8;5.1)	-55.1(-75.4;2.3)
Alagoas	92.9 (38.9;165.2)	6.9 (2.9;12.5)	112.1 (73.1;189.6)	3.5 (2.3;5.9)	-49.4(-68;0.7)
Amapá	2.5 (1.6;6.1)	2.5 (1.6;6.3)	6.9 (2.6;25.2)	1.3 (0.5;4.8)	-48(-73;4.4)
Amazonas	22.3 (13.7;57.6)	2.8 (1.7;7.4)	38.1 (12.7;139.8)	1.3 (0.4;4.8)	-53.7(-77.5;0)
Bahia	716 (174.3;1023.7)	10.4 (2.6;14.8)	750.6 (287.4;1055.3)	4.6 (1.8;6.5)	-55.2(-68.1;2.8)
Ceará	120.7 (57.8;351.9)	3 (1.4;8.7)	177.6 (83.3;507.5)	1.8 (0.8;5.1)	-39.9(-67.7;15.5)
Distrito Federal	198 (14.4;307.3)	35.1 (2.5;53.8)	172 (18.3;271)	7.7 (0.7;12.3)	-78.1(-83.6;-5.2)
Espírito Santo	46.9 (33.7;102.7)	3.3 (2.3;7.3)	59.2 (24.3;205.2)	1.4 (0.6;4.7)	-58.1(-78.4;2.1)
Goiás	1096.7 (67.1;1729.9)	52.3 (3.3;82.4)	705.6 (105.8;1137.9)	10.7 (1.5;17.3)	-79.6(-84.3;-5.8)
Maranhão	89.2 (20.7;388.3)	3.5 (0.8;15.6)	121.2 (42.7;415)	1.8 (0.7;6.3)	-47.6(-71.8;23.7)
Mato Grosso	52.8 (21.6;70.1)	6.4 (2.7;8.6)	77 (45.1;152.2)	2.3 (1.4;4.6)	-63.6(-76.5;1.2)
Mato Grosso do Sul	67.7 (24.9;87)	7.4 (2.9;9.4)	66.9 (40.1;135.5)	2.3 (1.4;4.6)	-69.1(-80.4;-5.3)
Minas Gerais	1976.9 (279.4;2880.5)	19.4 (2.9;28.1)	1215.5 (329.2;1749)	4.6 (1.2;6.6)	-76.2(-82.1;-3.9)
Pará	86.3 (56.7;182.4)	4.1 (2.7;9)	110.3 (49.2;334.7)	1.6 (0.7;4.7)	-61.9(-80.3;-1.1)
Paraíba	83.9 (42.3;228.1)	3.7 (1.9;10.2)	95.9 (54.3;236.1)	2 (1.1;4.9)	-45.8(-68.2;2.2)
Paraná	415.3 (132.6;537)	8.4 (2.8;10.7)	316.7 (167.2;632.6)	2.4 (1.3;4.8)	-70.7(-80.7;1)
Pernambuco	243.5 (128.6;419.2)	5.4 (2.9;9.5)	237.6 (150;541.9)	2.4 (1.5;5.4)	-56(-73.2;2.4)
Piauí	86.7 (36.4;173.1)	6.4 (2.7;12.9)	101.6 (67.2;192.6)	2.7 (1.8;5.1)	-58(-74.1;-0.2)
Rio de Janeiro	345.3 (224.4;698.8)	3.6 (2.4;7.2)	297.8 (135.9;1073)	1.3 (0.6;4.8)	-62.9(-81.2;0)
Rio Grande do Norte	47.4 (27.1;134.3)	3 (1.7;8.5)	57.6 (24.3;176.4)	1.5 (0.6;4.5)	-50.9(-73.2;-1.2)
Rio Grande do Sul	211 (149;418.7)	3.3 (2.3;6.5)	202 (91.2;715.3)	1.3 (0.6;4.7)	-60.1(-79.8;0.7)
Rondônia	31.3 (10.4;42.3)	9.7 (3.3;12.9)	38.5 (24.9;79.8)	2.5 (1.6;5.1)	-73.9(-84.2;-1.7)
Roraima	2.1 (1.3;5.7)	3.6 (2.2;9.9)	5.4 (1.9;19.7)	1.5 (0.5;5.3)	-58.8(-79.3;4.7)
Santa Catarina	76.3 (55;170.7)	3.1 (2.2;7)	96.2 (38.7;352.7)	1.2 (0.5;4.3)	-61.2(-80.7;0)
São Paulo	1718.8 (479.1;2275.6)	8.3 (2.5;10.8)	1352.9 (542.9;2400.9)	2.5 (1;4.5)	-69.4(-78.6;-1.4)
Sergipe	29.8 (18.4;71.4)	3.9 (2.4;9.6)	39.2 (19.3;110.3)	1.7 (0.9;4.9)	-56.1(-75;-8.1)
Tocantins	37.4 (10.8;60.2)	9.9 (2.8;16)	58.1 (22.9;82.6)	4.1 (1.6;5.8)	-58.3(-75.5;2.4)

*Source: Data derived from Global Burden of Disease Study 2019, Institute for Health Metrics and Evaluation, University of Washington. ^46^*

**Table 4-9:** – Number of prevalence cases, age-standardized prevalence rates (per 100 000) of heart failure from all causes, and percent change of rates, in Brazil and its Federative Units, 1990 and 2017.

Location	1990		2017		Percent Change (95% UI)
	Number (95% UI)	Rate (95% UI)	Number (95% UI)	Rate (95% UI)	
Brazil	670194.8 (589952.6;753672.6)	818.1 (718.1;922.8)	1686320.1 (1478563.8;1890537.3)	777.2 (680;874.8)	-5 (-7.1;-3)
Acre	1235.9 (1083.5;1395.6)	764.3 (668.5;869)	4025.6 (3559.4;4534.9)	728.8 (638.1;830.1)	-4.6 (-10.1;1.8)
Alagoas	9783 (8509.2;11210.5)	752.5 (654.5;861.9)	22691.5 (19784.3;25922)	764.8 (664.4;879.3)	1.6 (-5.7;8.3)
Amapá	748.2 (662;841.1)	779.1 (680.7;889.6)	3278.9 (2865.5;3672.3)	749.3 (651.9;845.2)	-3.8 (-9.5;2.6)
Amazonas	6097.6 (5376.6;6855.3)	809.8 (709.1;919)	19459.2 (17131;21872.1)	775.9 (678.8;884.1)	-4.2 (-10.2;1.9)
Bahia	52840.3 (46323.4;60082)	783.5 (685.2;893)	118062.7 (103361.3;134066.1)	753.3 (656.9;857.9)	-3.8 (-9.6;2.5)
Ceará	30093.8 (26385.1;34137.4)	739.5 (648;842.1)	77144.8 (67097.6;87800.2)	785.5 (682.6;896)	6.2 (-0.8;14.2)
Distrito Federal	4256.7 (3710.8;4838.9)	813.3 (701.7;932)	16100.7 (13996.8;18333.4)	753.5 (654.5;850.2)	-7.4 (-13.1;-0.6)
Espírito Santo	11320.9 (9847.7;12942.2)	841.5 (730.3;961.6)	31391.5 (27390.7;35566.7)	782.7 (680;889.7)	-7 (-13.2;-0.6)
Goiás	14142 (12371.7;16150.6)	800.7 (703.5;912.7)	46168.1 (40298.2;52244.1)	753 (655.9;854)	-6 (-12.9;1)
Maranhão	18235.7 (15857.1;20802.4)	747.2 (650.9;852.8)	49180.9 (43277;55993.9)	795.1 (697.6;907.8)	6.4 (0.2;13.5)
Mato Grosso	5774.8 (5067;6502.3)	819.3 (712.6;938)	21845.3 (19017.4;24622)	789.4 (688;895.4)	-3.7 (-9.6;3)
Mato Grosso do Sul	6795.1 (5934.8;7652)	846.4 (740.2;961.2)	21183 (18418.4;24002)	816 (710.9;922.6)	-3.6 (-9.3;3.4)
Minas Gerais	74411.2 (64608;84527.1)	826.9 (722;940.5)	187809.8 (163412.5;214570.5)	759.5 (659.4;867)	-8.1 (-14.2;-1.6)
Pará	16002.3 (14153.8;18014.5)	789.4 (694.3;893.2)	46324.1 (40809.4;52186.1)	746.6 (652.1;844.8)	-5.4 (-11.3;0.9)
Paraíba	17922.4 (15619.1;20442.3)	772 (675.4;881)	36827.2 (32186.6;41911.1)	794.6 (693.4;902.5)	2.9 (-3.6;10.2)
Paraná	35843.5 (31360.3;40661.2)	834.4 (726.6;948.5)	93386.5 (81689;106563.6)	779.7 (684;892.6)	-6.6 (-12.2;0.1)
Pernambuco	34084.1 (29826.4;39017.2)	793.9 (695;908.1)	72004 (62756.3;81953.9)	753.9 (655.5;862.9)	-5 (-11.2;1.3)
Piauí	11016.2 (9596.9;12503.6)	803.5 (698.9;912.6)	29097.2 (25425.9;33105.2)	812.7 (708.9;927)	1.1 (-5.6;8.5)
Rio de Janeiro	72976.8 (63619.5;83192)	850.4 (744;970.5)	162697.6 (140265.7;185508.6)	778.9 (674.2;887.1)	-8.4 (-14.1;-1.7)
Rio Grande do Norte	13462.3 (11756.9;15396.2)	827 (720.5;948.1)	31332.8 (27529.6;35470.6)	839 (734;955.6)	1.5 (-4.9;8.1)
Rio Grande do Sul	51590.7 (45263.1;58166.6)	862.8 (754.1;980)	115132.9 (100064.2;130860)	787.4 (685.2;894.6)	-8.7 (-14.7;-2.9)
Rondônia	2451.8 (2150.6;2766.5)	813.4 (710.9;925.4)	9980.6 (8742.3;11325)	766 (669.2;872.1)	-5.8 (-11.9;1)
Roraima	419.1 (368.5;471.8)	809.3 (706.3;925)	2297.9 (2000.3;2611)	774.6 (674.4;884.7)	-4.3 (-10.5;2)
Santa Catarina	19387.5 (16978;21842.4)	847 (741.4;957.7)	55662.9 (48893.1;63470.8)	779.6 (685.4;894.9)	-8 (-13.7;-2)
São Paulo	150009 (131202;169778.7)	842.8 (734.6;959.2)	387169.5 (336629;442688.8)	787.9 (685;899.2)	-6.5 (-12.8;-0.4)
Sergipe	6409.8 (5592.5;7302.6)	754.4 (657.2;860.7)	15587.4 (13661.6;17664.6)	763.8 (668.4;870.4)	1.2 (-4.7;8.8)
Tocantins	2884.2 (2501.8;3284.4)	789.5 (691.7;906.1)	10477.4 (9147.4;11899)	796.5 (692.4;907.9)	0.9 (-5.9;8.5)


*Source: Data derived from Global Burden of Disease Study 2019, Institute for Health Metrics and Evaluation, University of Washington.* ^46^

**Table 4-10:** – Prevalence rates of heart failure (per 100 000) and percent change of rates, by age and sex, Brazil, 1990 and 2017.

Age group and sex	1990	2017	Percent Change (95% UI)
Age-standardized	818.1 (718.1;922.8)	777.2 (680;874.8)	-5 (-7.1;-3)
Under 5	46.3 (32;63.8)	45 (30.8;61.9)	-2.9 (-5.5;-0.1)
5-14 years	34.7 (24.2;47.2)	34.1 (23.6;46.7)	-1.6 (-4.5;1.3)
15-49 years	107.1 (90.7;124.8)	119 (100.2;139.3)	11.1 (5.5;15.6)
50-69 years	1391.6 (1172.1;1627.5)	1330.4 (1125.6;1570.4)	-4.4 (-6.9;-1.8)
70+ years	8249.1 (6918.9;9752.5)	8530.2 (7265.9;9922.9)	3.4 (-1;8)
All Ages	448.5 (394.8;504.4)	796.1 (698.1;892.6)	77.5 (72.3;82.4)
**Male**			
Age-standardized	811.8 (714;916.9)	750.6 (656.2;845)	-7.5 (-10.2;-4.8)
Under 5	46.8 (32.2;64.5)	45.2 (31;62.1)	-3.6 (-7.2;0.4)
5-14 years	34.3 (23.7;46.9)	33.4 (23.1;45.9)	-2.5 (-6.6;1.7)
15-49 years	105.3 (89;122.4)	114.2 (95.6;134.7)	8.5 (0.5;14.2)
50-69 years	1386.9 (1164.2;1643.5)	1311.3 (1102.4;1555.5)	-5.4 (-9.1;-1.5)
70+ years	8083.9 (6784.9;9549.3)	7926.1 (6721.2;9286.2)	-2 (-6.4;2.9)
All Ages	415.6 (367.7;466.8)	685 (602.9;770)	64.8 (59.3;70.4)
**Female**			
Age-standardized	820.9 (721;933.2)	794.7 (694.4;900.6)	-3.2 (-6.5;-0.1)
Under 5	45.8 (31.6;63.3)	44.8 (30.7;63.3)	-2.1 (-5.7;2.2)
5-14 years	35.1 (24.7;47.9)	34.9 (24.3;47.2)	-0.7 (-4.6;3.1)
15-49 years	109 (91.7;126.6)	123.7 (103.9;144)	13.5 (8.8;18.3)
50-69 years	1395.9 (1183.9;1632.2)	1347.3 (1137.1;1586.4)	-3.5 (-6.9;0.3)
70+ years	8381.9 (7012.1;9982.4)	8968.9 (7622.9;10482.3)	7 (1.1;12.8)
All Ages	480.8 (422.3;544.4)	902.3 (790.2;1020.9)	87.7 (81;94.4)

*Source: Data derived from Global Burden of Disease Study 2019, Institute for Health Metrics and Evaluation, University of Washington. ^4^*

**Table 4-11:** – Number and age-standardized rates of YLDs (per 100 000) due to heart failure from all causes, and percent change of rates, in Brazil and its Federative Units, 1990 and 2017.

Location	1990		2017		Percent Change (95% UI)
	Number (95% UI)	Rate (95% UI)	Number (95% UI)	Rate (95% UI)	
Brazil	88114.2 (64078.1;112623.9)	112.2 (82.8;141.2)	234168.9 (174338.9;291187.7)	108.8 (81.4;134.5)	-3 (-6.7;0.3)
Acre	188.7 (140.7;234.7)	123.8 (95.3;148.9)	636.8 (490.6;767.6)	119.8 (93.4;142.4)	-3.2 (-9.6;5.2)
Alagoas	1230.4 (880.5;1587.3)	97.7 (70.9;124.1)	2951 (2189.4;3685.1)	101 (75;125.8)	3.3 (-5.8;12)
Amapá	100.2 (73.7;127.2)	112.2 (84.5;139.2)	451.5 (337.3;558.9)	108.9 (83.4;132.6)	-3 (-9.5;5)
Amazonas	810.4 (592.2;1020.8)	115.9 (87.9;141.5)	2702.1 (2050;3305.1)	112.2 (86.1;135.8)	-3.1 (-10;3.9)
Bahia	6684.8 (4893.5;8540.9)	101.7 (75.5;129)	15736.9 (11812.7;19456)	100.8 (75.3;125.3)	-0.8 (-7.7;7.9)
Ceará	3969.6 (2897.9;5084.5)	99.2 (72.8;126.2)	10451.4 (7838.4;12882.6)	106.6 (79.8;132)	7.4 (-1.3;17)
Distrito Federal	431.1 (298.6;581.8)	97.6 (70.8;128.8)	1940.3 (1366.1;2560)	96.9 (69.9;125.4)	-0.8 (-8.1;9.2)
Espírito Santo	1462.1 (1054.4;1878.9)	113.4 (84.3;143.6)	4162.7 (3063.9;5243.2)	104.9 (77.5;131.9)	-7.5 (-14.4;0.1)
Goiás	1592.7 (1128;2094.6)	103.8 (76.7;132.4)	6291.2 (4685;7868.1)	106.1 (79.5;131.7)	2.2 (-6.5;11.5)
Maranhão	2190.7 (1560.1;2873.3)	92 (66.4;119.5)	5992.2 (4402.4;7553.5)	97.8 (72;122.9)	6.3 (-1.6;15.9)
Mato Grosso	725.4 (526.7;941)	113.1 (83.9;142.3)	3003 (2248.2;3728.7)	112 (84.7;137.3)	-0.9 (-8.1;7.7)
Mato Grosso do Sul	850.1 (609.1;1106.8)	113.3 (83.8;142.8)	2829.1 (2072.2;3549.4)	110.8 (82.1;137.7)	-2.2 (-9.5;6.5)
Minas Gerais	9477.1 (6808.4;12335.3)	110.9 (82.3;141.3)	25557 (18974.8;31992.6)	103.6 (77.2;129.4)	-6.5 (-14.4;1.6)
Pará	2221.3 (1651.1;2795.8)	115.7 (88.1;142.8)	6624.9 (5036.2;8052.8)	109.8 (84.8;132.1)	-5.2 (-12.2;2.3)
Paraíba	2298.3 (1666.9;2947.6)	99.5 (72.4;126.3)	4808 (3560.3;5995.7)	103.4 (76.3;129.4)	4 (-4.2;12.5)
Paraná	4964.3 (3563.3;6413.6)	123.5 (92.3;154.2)	13883.5 (10128.6;17249.1)	117 (86.8;144.4)	-5.2 (-12.7;3.3)
Pernambuco	4627.4 (3326;5865.3)	110.9 (81.9;137.8)	10375.1 (7650.4;12784)	109.6 (81;134.6)	-1.2 (-9;7.3)
Piauí	1230.3 (866.5;1647.8)	92.1 (65.6;122)	3402.3 (2478.9;4365.3)	95.1 (69.4;122.2)	3.3 (-5;11.9)
Rio de Janeiro	9922.7 (7229.9;12662.8)	119.7 (88.6;150.3)	22953.2 (16743.8;28786)	110 (81.3;137.7)	-8.1 (-14.7;-0.4)
Rio Grande do Norte	1606.1 (1145.6;2057.5)	99.5 (71.8;126.6)	3856.8 (2838.4;4879.3)	103.5 (75.7;132)	4 (-3.9;12)
Rio Grande do Sul	8134 (5994.6;10280.5)	140.5 (106.7;171.9)	18696.6 (14241.9;22612.8)	126.9 (97;152.5)	-9.7 (-16.6;-2.5)
Rondônia	300.3 (209.4;397.4)	117.4 (86.9;145.5)	1408.5 (1062.2;1750.7)	111.9 (85.8;137)	-4.7 (-11.7;4.4)
Roraima	46.3 (32.4;62.3)	101.5 (74.8;130.8)	276.8 (197.7;358.6)	98.7 (72.2;125.2)	-2.8 (-9.7;5)
Santa Catarina	2880 (2095.3;3688.1)	133.3 (99.4;165.6)	8433.4 (6334.3;10482.5)	119.7 (90.8;147.4)	-10.2 (-16.8;-3.4)
São Paulo	18988.5 (13523.4;24849.5)	112.8 (81.9;144.5)	53360.6 (38622.8;68602.6)	109.6 (80.1;140.1)	-2.9 (-10.6;5.1)
Sergipe	853.9 (627.3;1082.5)	102.4 (75.6;129)	2085.5 (1551.2;2578.3)	103.7 (77.4;127.2)	1.3 (-6.3;9.9)
Tocantins	327.5 (226.3;438.8)	96.4 (69.6;126.2)	1298.6 (952.1;1635.1)	100.3 (73.9;125.9)	4 (-4.3;14.5)

*Source: Data derived from Global Burden of Disease Study 2019, Institute for Health Metrics and Evaluation, University of Washington. ^46^*

**Table 4-12:** – Rates of YLDs due to heart failure (per 100 000) and percent change of rates, by age and sex, Brazil, 1990 and 2017.

Age group and sex	1990	2017	Percent Change (95% UI)
**All**			
Age-standardized	112.2 (82.8;141.2)	108.8 (81.4;134.5)	-3 (-6.7;0.3)
Under 5	4.5 (2.7;7)	4.3 (2.6;6.8)	-3.4 (-6;-0.6)
5-14 years	3.2 (1.9;5.1)	3.2 (1.9;5)	-1.4 (-4.4;1.5)
15-49 years	8.7 (5.7;12.6)	10.4 (6.8;14.8)	18.7 (12.3;25.5)
50-69 years	165.8 (112.7;226.8)	166.4 (115.3;228.9)	0.3 (-3.8;5.5)
70+ years	1263.1 (919.3;1599.5)	1308.3 (988.6;1586)	3.6 (-3.2;10.2)
All Ages	59 (42.9;75.4)	110.6 (82.3;137.5)	87.5 (78.8;96.2)
**Male**			
Age-standardized	112.9 (86.9;137.4)	105.2 (81.6;127.1)	-6.8 (-10.9;-2.6)
Under 5	4.5 (2.7;7.1)	4.3 (2.6;6.8)	-4.2 (-7.8;-0.2)
5-14 years	3.2 (1.9;5)	3.1 (1.8;4.9)	-2.2 (-6.4;2)
15-49 years	7.9 (5.1;11.6)	9.5 (6.1;13.8)	19.8 (10.2;29.3)
50-69 years	165.5 (111.9;230.9)	165.8 (113.5;229.4)	0.2 (-5.9;6.8)
70+ years	1282.5 (973.9;1555.8)	1225.7 (980;1439.6)	-4.4 (-11.1;3.4)
All Ages	55 (40.6;68.5)	94.5 (72.6;115.7)	71.9 (63.1;81.5)
**Female**			
Age-standardized	111.2 (79.9;145)	110.9 (80.1;140.6)	-0.3 (-4.9;4.2)
Under 5	4.4 (2.7;7)	4.3 (2.6;6.7)	-2.6 (-6.3;1.7)
5-14 years	3.2 (1.9;5.2)	3.2 (1.9;5.1)	-0.6 (-4.5;3.2)
15-49 years	9.5 (6.3;13.4)	11.2 (7.4;15.9)	17.9 (10.5;26.4)
50-69 years	166.1 (112.5;225.8)	166.9 (116.6;228.9)	0.5 (-5;6.7)
70+ years	1247.5 (876.2;1659.2)	1368.3 (992.5;1714.5)	9.7 (0.6;17.6)
All Ages	62.9 (44.1;82.5)	125.9 (90.9;159.5)	100.2 (88.8;111.5)

*Source: Data derived from Global Burden of Disease Study 2019, Institute for Health Metrics and Evaluation, University of Washington. ^46^*

**Chart 4-1:** – Prevalence rate (A) and crude prevalence rate (B) of cardiomyopathy and myocarditis, per 100 000 inhabitants, Brazil, 1990-2019.

Source: Data derived from Global Burden of Disease Study 2019, Institute for Health Metrics and Evaluation, University of Washington. ^46^

**Chart 4-2:** – Age-standardized and all ages mortality rate due to cardiomyopathy and myocarditis, per 100 000 inhabitants, Brazil, 1990-2019.

Source: Data derived from Global Burden of Disease Study 2019, Institute for Health Metrics and Evaluation, University of Washington. ^46^

**Chart 4-3:** – Correlation between the sociodemographic index (SDI) and the age-standardized mortality rate due to cardiomyopathy and myocarditis, per 100 000 inhabitants, 1990 and 2019.

Source: Data derived from Global Burden of Disease Study 2019, Institute for Health Metrics and Evaluation, University of Washington. ^46^

**Chart 4-4:** – Age-standardized and all ages rates of DALYs attributable to cardiomyopathy and myocarditis, per 100 000 inhabitants, in Brazil, from 1990 to 2017. Data derived from Global Burden of Disease Study 2017 (GBD 2017). ^152^

**Chart 4-5:** – Age-standardized and all ages mortality rates attributable to Chagas Disease, per 100 000 inhabitants, in Brazil from 1990 to 2019.

Source: Data derived from Global Burden of Disease Study 2019, Institute for Health Metrics and Evaluation, University of Washington. ^46^

**Chart 4-6:** – Correlation between percent change in age-standardized mortality rates due to Chagas disease, per 100 000 inhabitants, from 1990 to 2019 and the Sociodemographic Index (SDI) in 1990 and in 2019.

Source: Data derived from Global Burden of Disease Study 2019, Institute for Health Metrics and Evaluation, University of Washington. ^46^

**Chart 4-7:** – Spatial distribution of mean mortality rates related to Chagas disease, per 100 000 inhabitants, based on multiple causes of death by municipality, Brazil, 1999–2007.

Source: Martins-Melo et al. ^141^

**Chart 4-8:** – Standardized mortality rate due to Chagas disease in Brazil according to age range (in years) and year of occurrence, from 2000 to 2010.

Source: Nóbrega et al. ^142^

**Chart 4-10:** – Trends of mortality from heart failure, from 2007 to 2016, in Brazil (BR), in the state of Rio Grande do Sul (RS), and in the city of Porto Alegre (POA).

Source: Nicolao et al. ^169^

### Cardiomyopathy and Myocarditis

#### Prevalence and Incidence

• According to GBD Study 2019 estimates, the age-standardized prevalence of cardiomyopathy and myocarditis decreased in Brazil, from 76.6 (95% UI, 53.4-107.2) in 1990 to 73.0 (95% UI, 51.1-100.1) in 2019, a decrease of 4.7% (95% UI, - 9.5 to 0.8) in the period (Chart 4-1.A and Table 4-1). In absolute numbers, estimates of the prevalence of cardiomyopathy and myocarditis in Brazil increased from less than 60 000 in 1990 to over 160 000 in 2019, mainly due to population growth and aging (Chart 4-1.B). The cardiomyopathy and myocarditis prevalence was greater in men (98.9; 95% UI, 69.5-137.2) than in women (54.1; 95% UI, 38.4-73.8) in 2019, although there was a prevalence decrease of 5 (95% UI, -11.6 - 0) for women and of 2.9 (95% UI, -9.1 - 0) for men in that period. ^46^

• According to GBD Study 2019 estimates, the prevalence of cardiomyopathy and myocarditis is highly variable amongst Brazilian FUs, and the percent change varied unevenly between 1990 and 2019 (Table 4-1). In 2019, the highest rates were observed in São Paulo, Paraíba, and Roraima. From 1990 to 2019, increased age-standardized prevalence rates were observed in Goiás and Maranhão, while age-standardized prevalence decreased in all other FUs. ^46^

• Regarding age-standardized incidence rates per 100 000 per-year, the GBD Study 2019 estimates were 15.8 (95% UI, 12.7-19.2) in 1990, and 15.8 (95% UI, 12.7-19.2) in 2019, with a small change of -0.2% (95% UI, -0.3 to -0.2) during that period (Table 4-2). Absolute numbers of incident cases were 18 583 (95% UI, 14 825-22 718) in 1990 and 35 863 (95% UI, 28 946-43 756) in 2019; that increase is related to population growth and aging. Table 4-3 depicts the incidence rates of cardiomyopathy and myocarditis per 100 000 inhabitants, by age, for both sexes, in 1990 and 2019, and the percent change of the rates. In 1990 and 2019, the highest rates were observed in the ‘70+ years’ group. From 1990 to 2019, the incidence increased in the ‘15-49 years’ and the ‘70+ years’ age groups for both women and men. ^46^

#### Mortality

• According to the GBD Study 2019 estimates, the mortality rates due to cardiomyopathy and myocarditis seemed to increase in the 1990s but decreased in the following 2 decades (Chart 4-2). As shown in Table 4-4, the mortality rates were 15.9 (95% UI, 12.4-17.1) in 1990 and 9.4 (95% UI, 8.3-11.1) in 2019, per 100 000 inhabitants, a decrease of 40.8% (95% UI, -46.6 to -25.4). Despite this decrease in mortality rates, the number of deaths from cardiomyopathy and myocarditis increased in that period due to population growth and aging. Cardiomyopathy and myocarditis were responsible for 13 408 (95% UI, 8417-10 163) deaths in 1990, rising to 21 425 (95% UI, 17 885-21 745) deaths in 2019. The GBD Study 2019 estimates of mortality rates due to cardiomyopathy refer to cases with cardiomyopathy listed as an underlying cause of death. Death due to HF related to other specific causes are attributed to the underlying disease, i.e., deaths related to ischemic cardiomyopathy are coded as due to ischemic heart disease. Moreover, for the GBD project, HF is not considered a primary cause of death, and all deaths coded as related to HF are recoded to the baseline condition (see below). ^46^

• Table 4-4 also depicts the total number of deaths and age-standardized mortality rate (per 100 000 inhabitants) due to cardiomyopathy and myocarditis, as well as the percent change of rates, by FU, in Brazil, in 1990 and 2019. All FU had decreased mortality rates, with the highest percentages of reductions observed between 1990 and 2019 in the states of Goiás and Paraná. In 2019, the FUs with the lowest mortality rates (below 6.0) were the states of Amazonas, Rio Grande do Norte and Pará.

• Table 4-5 shows mortality rates due to cardiomyopathy and myocarditis according to sex and by age groups, from the GBD Study 2019 estimates. Women had lower age-standardized mortality rates, as well as a more pronounced reduction from 1990 to 2019. Rates in women were 13.5 (95% UI, 9.9-14.7) in 1990 and 7.2 (95% UI, 6.2 - 9) in 2019, a reduction of 46.6% (95% UI, -54.8 to -0.2). Rates in men were 18.7 (95% UI, 13.2 - 20.4) in 1990 and 12.1 (95% UI, 10 - 15.1) in 2019, a variation of -35.4% (95% UI, -43.3 to -0.1). As expected, the highest mortality rate was observed in the 70+ years group, with rates of 127.2 (95% UI, 96.5-138.3) in 1990 and of 84.6 (95% UI, 72.1-104) in 2019 per 100 000. For the 50-69 years group, rates were 29.2 (95% UI, 22 – 31.4) per 100 000 in 1990 and 17.2 (95% UI, 15.1 - 20) in 2019. Overall, mortality rates decreased from 1990 to 2019 in all age groups. ^46^

• The GBD Study 2019 uses the SDI as an estimate of the socioeconomic level of a location. Chart 4-3 depicts the correlation between the SDI and the age-standardized mortality rate due to cardiomyopathy and myocarditis, per 100 000, both in 1990 and 2019. There was no correlation between age-standardized mortality rates and the SDI in 1990 (R = - 0.36, p=0.068). However, there was a significant correlation between age-standardized mortality rates and the SDI in 2019 (R = 0.4, p=0.041). ^90^

• In a study reporting data from the SEADE, from the state of São Paulo, cardiomyopathies were responsible for a total of 3571 deaths, representing 23.3% of HF-related deaths in 2006: dilated cardiomyopathy accounted for 17.2% of the deaths; alcoholic cardiomyopathy, for 0.45%; and restrictive cardiomyopathies, for 0.37%. Chagas disease and alcoholic cardiomyopathy were responsible for 7.8% and 0.45% of the HF-related deaths, respectively. ^135^

• Data on specific cardiomyopathies are scarce. A cohort study of 214 patients with HCM reported data from a 7-year follow-up in a tertiary hospital in São Paulo, Brazil. The mean age was 37±16 years, and 52% were women. There were 22 deaths (10%), 15 directly related to HCM (11 sudden deaths). The cumulative survival rates were 94.5% at 5 years, 91% at 10 years, and 87.9% at 15 years, with an annual mortality rate of 1%, which is low, considering that the study was conducted in a referral center. ^136^

#### Burden of Disease

• According to the GBD 2019 estimates, the trends of age-standardized DALY rates due to cardiomyopathy and myocarditis were similar to those of mortality, with a small increase in the 1990s and a decrease during the following decades. As shown in Table 4-6, age-standardized DALY rates were 399.4 (95% UI, 319.6-426.4) in 1990 and 238.6 (95% UI, 213-272.3) in 2019, per 100 000 inhabitants, a decrease of 40.3% (95% UI, -45.8 to -27.9). These changes are similar to those observed in the mortality rates. Despite that decrease in DALY rates, cardiomyopathy and myocarditis resulted in 431 381 (95% UI, 349,546-465,068) DALYs in Brazil in 1990 and in 545 772 (95% UI, 484 988-621 356) in 2019, which represents 0.62% of all DALYs. ^46^

• Table 4-7 shows DALY rates due to cardiomyopathy and myocarditis according to sex and by age groups, from the GBD Study 2019 estimates. The age-standardized DALY rates were lower in women, and the reduction from 1990 to 2019 was also more pronounced in women. The DALY rates for women were 321.1 (95% UI, 237.7-346.2) in 1990 and 165.7 (95% UI, 147.3 - 198) in 2019, a reduction of 48.4% (95% UI, -55 to – 0.2). The DALY rates for men were 484.8 (95% UI, 353.6 – 528.5) in 1990 and 320.4 (95% UI, 257.7 – 371.8) in 2019, a reduction of 33.9% (95% UI, -41.8 to -0.1). As expected, the highest DALY rate was observed in the 70+ years group, followed by that of the 50-69 years group. Overall, DALY rates decreased from 1990 to 2019 in all age groups (Chart 4-4). ^46^

## Chronic Chagas Disease and Chagas Cardiomyopathy

### Prevalence and Incidence

• The 2010 prevalence of ChD in Brazil was estimated at 1 156 821 by the World Health Organization, ^137^ which is the last official available estimate, published in 2015. According to that statement, the estimated number of individuals with ChCM in Brazil is 231 364 subjects. ^137^ Those numbers reveal a significant decreasing trend of ChD human cases in Brazil in relation to previous estimates, which was attributed to various factors, mainly the almost complete interruption of the vectorial and transfusion-related transmission in Brazil.

• According to the GBD Study 2019 estimates, the age-standardized prevalence of ChD markedly reduced in Brazil, by 37.7% (95% UI, -40.2 to -35.2) from 1990 to 2019: from 1463 (95% UI, 1240-1711) in 1990 to 912 (95% UI, 788-1048) in 2019 per 100 000 inhabitants. The prevalence of ChD in Brazil in 2019 was higher in men [987 (95% UI, 856.4 – 1141.3)] than in women [841 (95% UI, 723.2-962)]. ^46^

• In a systematic review of population-based studies on ChD prevalence in Brazil, performed from 1980 until September 2012, 42 articles with relevant prevalence data were identified from a total of 4985 references. ^138^ The pooled estimate of ChD prevalence across studies for the entire period was 4.2% (95% CI, 3.1-5.7), ranging from 4.4% (95% CI, 2.3-8.3) in the 1980s to 2.4% (95% CI: 1.5-3.8) after 2000. The estimated ChD prevalence for males and females was similar (4.1% [95% CI, 2.6-6.6], 4.2% [95% CI, 2.6-6.8], respectively). The highest pooled prevalence was observed in individuals aged >60 years (17.7%; 95% CI, 11.4-26.5), and in the Northeastern (5.0%; 95% CI, 3.1-8.1) and Southeastern (5.0%; 95% CI, 2.4-9.9) regions and in mixed (urban/rural) areas (6.4%; 95% CI, 4.2-9.4). About 4.6 million (95% CI, 2.9-7.2 million) people are estimated to have been infected with *T. cruzi* in 2010. These estimates are much higher than those from the World Health Organization for 2010. ^137^ The authors observed a high degree of heterogeneity in most pooled estimates (I(2)>75%; p<0.001).

• In the NIH REDS-II Chagas retrospective cohort study, initially healthy blood donors with an index *T. cruzi* -seropositive donation and age, sex, and period-matched seronegative donors were followed up for 10 years. ^139^ The differential incidence of cardiomyopathy was 1.85 per 100 person-years attributable to *T. cruzi* infection in the first 10 years of follow-up, and 0.9 per 100 person-years in the following 10 years. *T. cruzi* antibody level in the second visit was associated with development of cardiomyopathy (adjusted OR of 1.4, 95% CI 1.1-1.8) in the last visit. ^140^

#### Mortality

• According to the GBD Study 2019, the number of deaths due to ChD in Brazil has decreased over the past decades (Chart 4-5). In the 1990s, ChD was responsible for 7903 (95% UI, 2438-10 073) deaths, reducing to 6523 (95% UI, 3350-11 226) deaths in 2019. The age-standardized mortality rate showed more striking decrease (-67.5% change), from 8.6 (95% UI, 2.8-10.9) deaths per 100 000 inhabitants in 1990 to 2.8 (95% UI, 1.8-4.8) per 100 000 inhabitants in 2019, accounting for 1.6% of all cardiovascular deaths in the country. In 2019, men had higher age-standardized mortality rates than women (3.5, 95% UI, 1.4-6.2; and 2.2, 95% UI, 0.9-4.3, respectively). ^46^

• Table 4-8 demonstrates the total number of deaths and age-standardized mortality rates due to ChD (per 100 000 inhabitants), for both sexes, and the percent change of rates, by FU, in Brazil, in 1990 and in 2019. There is striking variability among the FUs regarding the number of deaths and death rates for both years. In 1990, higher mortality rates (> 10 per 100 000 inhabitants) were observed in the Brazilian central FUs of Goiás, Distrito Federal, Minas Gerais and Bahia, with a peak in Goiás (52.3 per 100 000, 95% UI, 3.3-82.4). All FUs showed a reduction in mortality rates, varying from 39.9% (95% UI, 67.7 to 15.5) in the state of Ceará to 79.6% (95% UI, -84.3 to -5.8) in the state of Goiás.

• The estimated mortality in children under 5 years is practically zero. In the other age groups, the reduction in mortality rates was more pronounced (76.2 % change, UI -86.5 to 29.5) in the age group of 15-49 years, from 2.6 (UI 0.6-3.5) to 0.6 (UI 0.4-1.3) per 100 000 inhabitants. Most deaths occurred in individuals aged 70+ years, who presented the lowest percent reduction (-54.7%, UI -65-6.3) during the 1990-2019 period: from 53 (UI 19.2-66.3) to 24 (10.4-36.1) per 100 000 inhabitants. The decrease in age-standardized mortality rate per 100 000 inhabitants correlates with the SDI of Brazilian FUs in 1990 (R = - 0.56, p=0.003) and in 2019 (R = - 0.63, p<0.001) (Chart 4-6).

• Several population-based studies showed a reduction in mortality due to ChD in Brazil in the last decades. Martins-Melo *et al* . ^141^ reported that nationwide standardized mortality rates reduced gradually, from 3.78 (1999) to 2.78 (2007) deaths/year per 100 000 inhabitants (-26.4%). Nobrega *et al* . ^142^ showed that the nationwide standardized mortality rate decreased by 32.4%, from 3.4% in 2000 to 2.3% in 2010. The mortality rate due to cardiac involvement decreased in all regions of Brazil, except for the Northern region, where it increased by 1.6%. The Northeastern region had the smallest and the West-Central had the largest decrease. Simões *et al* . ^143^ studied the evolution of ChD mortality in Brazil from 1980 to 2014 and forecasted the mortality from years 2015 to 2034. Those authors estimated a progressive decline in ChD mortality, which would be highest among the young. The expected average reduction was 76.1% as compared to the last period observed (2010-2014) and the last period predicted (2030-2034). The West-Central, Southeastern, and Southern regions had a reduction in the rate of ChD deaths between 2000 and 2014. The mortality rate in the Northeastern region did not statistically differ in any period analyzed, while, in the Northern region, it showed an increasing trend.

• In a study analyzing all death certificates of individuals who died between 1999 and 2007 in Brazil, ^141^ based on the nationwide SIM, ChD was mentioned in 53 930 (0.6%) death certificates, as an underlying cause in 44 537 (82.6%) death certificates, and as an associated cause of death in 9387 (17.4%) death certificates. Acute ChD was responsible for 2.8% of the deaths. The mean standardized mortality rate was 3.36 per 100 000 inhabitants/year. This is 21% higher than the mortality rate considering merely the underlying cause of death (2.78 deaths per 100 000 inhabitants/year). The proportional mortality considering multiple causes of death was 0.6%. Individuals who died from ChD were predominantly males (57%), aged over 60 years (62.8%), and residing in the Southeastern region (53.6%). The West-Central region showed the highest proportional mortality of all regions (2.17%). ^141^

• In the same database, calculating the mean mortality rate for each municipality of residence and using Empirical Bayesian smoothing, the spatial analysis identified a large cluster of high risk for mortality by ChD, involving nine states in the central region of Brazil (Chart 4-7). ^144^

• Nobrega *et al* ., in a descriptive study using data from the SIM of all individuals who died of ChD in Brazil between 2000 and 2010, observed that, in the 2000-2010 period, most of the deaths due to ChD (85.9%) occurred in males aged > 60 years and were caused by cardiac involvement. During the studied period, the mortality rate decreased in all age groups, except for that of 80 years and over (Chart 4-8). ^142^

• In a retrospective cohort study, probabilistic linkage was used to identify among blood donors from 1996 to 2000 (2842 seropositive and 5684 seronegative for ChD) those who died until 2010. ^145^ The authors identified 159 deaths among the 2842 seropositive blood donors (5.6%) and 103 deaths among the 5684 seronegative blood donors (1.8%). Chagas seropositive donors had a 2.3 times higher risk of death due to all causes (95% CI, 1.8-3.0) than seronegative donors. Among seropositive donors, only 26 had the ICD-10 code indicating ChD as the underlying cause of death (B57.0/B57.5). ^145^ The authors concluded that ChD is an underreported cause of death in the Brazilian mortality database.

• Ayub-Ferreira *et al* . compared the mechanism of death in HF due to ChCM with that of other etiologies in a prospective clinical trial, the REMADHE trial, which included patients aged 18 years or older with irreversible chronic HF of at least 6 months and left ventricular ejection fraction of less than 50%. Of the 342 patients analyzed, 185 (54.1%) died. Death occurred in 56.4% of ChD patients and in 53.7% of non-ChD patients. Of all ChD group deaths, 48.4% were related to HF worsening, 25.7% to sudden death, and 6.4% to stroke. The cumulative incidence of all-cause mortality and HF mortality was significantly higher in ChD patients as compared to non-ChD patients. ^146^ There was no difference in the cumulative incidence of sudden death mortality between the two groups. In severe Chagas heart disease, progressive HF is the most important mechanism of death.

• In the Bambuí Cohort Study, a large population-based cohort study of elderly residing in an area where ChD is endemic, 1479 subjects aged 60 years and over (38.1% positive *T. cruzi* test) were followed up from 1997 to 2007. During a mean 8.72-year follow-up, 567 participants died. *T. cruzi* infection was a predictor of mortality among cohort members, and this association remained largely significant after adjustments for age, sex, and conventional cardiovascular risk factors (HR = 1.56; 95% CI, 1.32-1.85). Overall, the PAR for mortality due to *T. cruzi* infection was 13.2% (95% CI, 9.8-16.4). ^147^

• Nadruz *et al* . studied the temporal trends in PAR of ChCM for 2-year mortality among patients with HF enrolled in 2002-2004 (era 1) and in 2012-2014 (era 2) in a Brazilian university hospital. They prospectively studied 362 (15% with ChCM) and 582 (18% with ChCM) HF patients with ejection fraction ≤50% in eras 1 and 2, respectively, and estimated the PAR of ChCM for 2-year mortality. Although the absolute death rates decreased over time in the ChCM and NChC groups, the PAR of ChCM for mortality increased among patients with HF [PAR_(era 1)_= 11.0 (95% CI, 2.8-18.5%); PAR_(era 2)_= 21.9 (95% CI, 16.5-26.9)%; p=0.023 *versus* era 1], driven by increases in the HR associated with ChD. ^148^

• In a cohort study, 298 ChD patients were followed up from March 1995 to September 2019 in the municipality of Virgem da Lapa, located in the state of Minas Gerais, Brazil. In the 24-year study period, 113 (37.9%) deaths were recorded in the cohort, 107 (35.9%) of which were attributed to heart disease: only 10 (11.6%) occurred in the 86 patients without cardiomyopathy, 49 (31.4%) in the 156 patients with cardiomyopathy, and 48 (85.7%) in the 56 patients with left ventricle aneurysm. The risk of death was 7.4 times significantly higher in patients with left ventricle aneurysms. ^149^

• In a cohort study that followed up 1637 ChD patients residing in 21 municipalities in which ChD is endemic, for two years, 205 (12.5%) patients showed new cardiovascular events, 134 of whom (8.2%) died, 28 (1.7%) developed atrial fibrillation, and 43 (2.6%) required pacemaker implantation. Individuals living in municipalities with a larger rural population had protection against cardiovascular events (OR = 0.5; 95% CI, 0.4-0.7), while those residing in municipalities with fewer physicians per 1000 inhabitants (OR = 1.6; 95% CI, 1.2-2.5) and those living in municipalities with lower primary health care coverage (OR = 1.4; 95% CI, 1.1-2.1) had higher chances of experiencing cardiovascular events. ^150^

• In a cohort study including 1551 patients with ChCM from the state of Minas Gerais, Brazil, a score to predict 2-year mortality was developed. The score included simple variables, such as age, New York Heart Association functional class, heart rate, QRS duration, and abnormal NT-proBNP adjusted by age. The observed mortality rates in the low-, intermediate-, and high-risk groups were 0%, 3.6%, and 32.7%, respectively, in the derivation cohort, and 3.2%, 8.7%, and 19.1%, respectively, in the validation cohort, with C statistics of 0.82 and 0.71, respectively. It seems to be a helpful and simple score that could be used in remote areas with limited technological resources. ^151^

#### Burden of Disease

In the GBD Study 2019, 174 194 DALYs (95% UI, 109 039-302 974) due to ChD were estimated in Brazil, with a relative reduction of 32.1% as compared to that of 1990 (256 380 DALYs, 95% UI, 81 697-328 720). Age-standardized DALY rates declined at national level (-70.5%) and in all Brazilian FUs between 1990 and 2019, but with different regional patterns ( Chart 4-9 ). The decrease in the DALY rates was driven primarily by a consistent reduction in the YLL rates, the main component of total DALYs for ChD. The highest fatal and non-fatal burden due to ChD was observed among males, the elderly, and in the Brazilian FUs encompassing important endemic areas of vectorial transmission in the past, such as the states of Goiás, Tocantins, Minas Gerais, Bahia, and the Distrito Federal. ^46^

**Chart 4-9:** – Age-standardized rates of DALYs due to Chagas disease, per 100 000 population, in Brazil, in 2016. Source: Martins-Melo et al. ^144^

## Heart Failure

• Because HF is not considered an underlying cause of death (i.e., garbage code) in the GBD study, all deaths attributed to HF in death certificates are reclassified and/or redistributed to other causes, according to the GBD method. As such, there is no data from GBD on mortality from HF. Because HF is classified by GBD as an “impairment”, the only indicators we have for HF from the GBD are prevalence and YLDs, which is the morbidity component of DALYs.

### Prevalence and Incidence

• According to the GBD Study 2017 estimates, age-standardized prevalence of HF changed in Brazil from 818 (95% UI, 718-923) in 1990 to 772 (95% UI, 680-875) in 2017, with a decrease of 5% (95% UI, -7.1 to -3) in the period (Table 4-9). In absolute numbers, estimates for the prevalence of HF in Brazil rose from 0.67 million in 1990 to almost 1.7 million in 2017, mainly due to population growth and aging. The prevalence of HF is variable amongst Brazilian FUs, and the percentage of change varied unevenly between 1990 and 2017 (Table 4-9). In 2017, the highest rates were observed in the state of Rio Grande do Norte, and the lowest, in the state of Acre. From 1990 to 2017, decreased age-standardized prevalence rates were observed in most FUs, and increases in rates occurred in 8 FUs, mostly in the Northeastern region. ^152^

• Table 4-10 depicts HF prevalence according to sex and age groups, from the GBD Study 2017 estimates. HF prevalence was higher in women (795; 95% UI, 694-901) than in men (751; 95% UI, 656-845) in 2017, and the reduction in prevalence from 1990 to 2017 was more pronounced in men [the percentage of decrease was 7.5 (95% UI, -10.2 to -4.8) for men and 3.2 (95% UI, -6.5 to -0.1) for women]. Regarding age groups, there is a 10-fold increase in the incidence rates from the ‘15-49 years’ group to the ‘50-69 years’ group, as well a 6-fold increase from the latter to the ‘70+ years’ group, and these increases are similar for women and men. From 1990 to 2017, the prevalence increased only in the ‘15-49 years’ group, while decreased in the others, probably associated with increased ischemic events in that age group. ^152^

• A systematic review evaluated the burden of HF in Latin America and included 143 articles published between January 1994 and June 2014, with at least 50 participants aged ≥ 18 years; most studies included (64%) were from Brazil. ^153^ The patients’ mean age was 60±9 years, and the mean ejection fraction was 36±9%. The prevalence of HF was 1% (95% CI, 0.1-2.7). Of the studies included, only one assessed incidence, with 1091 individuals identified through multistage probability sampling in the city of Porto Alegre, Brazil. The mean age was 42.8±16.9 years, and 55% were women. The incidence of HF in the single population study providing this information was 199 cases per 100 000 person-years. ^154^

• In a population-based study in primary care of a medium-sized city in Brazil, 633 individuals aged ≥45 years were randomly selected and registered in a primary care program of a medium-sized city in Brazil. The mean age was 59.6±10.4 years, and 62% were females; the prevalence of symptomatic HF (stage C) was 9.3%, and the prevalence of stage B HF (structural abnormalities) was 42.7%. Of the patients with HF, 59% presented with HF-PEF and 41%, with HF-REF. ^155^

• A study from the Brazilian National Health Survey held in 2013, with data on 59 655 adults (≥ 18 years), found a prevalence of self-rated HF of 1.1%, which would represent about 1.7 million people. In those aged over 60 years, the prevalence was 3.3%. ^156^

• Another population-based study of residents in Zona da Mata, state of Minas Gerais, involved 7113 frail elderly. The mean age was 72.4 ± 8.0 years, 67.6% were women, and the prevalence of HF was 7.9%. ^157^

• In a study that included 166 patients from the rural area of Valença, state of Rio de Janeiro, the mean age was 61±14 years, and 51% were men. The main etiologies were hypertensive and ischemic, and 51% of the patients had HF-REF, showing characteristics similar to those of cohorts from non-rural tertiary centers. ^158^

### Mortality

• In a study evaluating data from the SIM, from 2008 to 2012, HF was a frequently used garbage code in Brazil. It was listed as the underlying causes of death in 123 268 (3.7%) of those records and as a multiple cause of death in 233 197 (7%). By using two redistribution methods to specific causes of death, only 38.7-44.8% could be reclassify to a defined cause of death with the principal diagnosis, depending on the reclassification method. ^159^ Although HF should not be considered the underlying cause of deaths and rather be in the chain of events that led to death, any analysis of SIM data that uses HF as the underlying cause of death from death certificates must be interpreted with caution, because it may be wrongly estimating the true burden of HF.

• Data obtained from the SEADE for mortality in the state of São Paulo in 2006 evaluated 242 832 deaths in estimated 41 654 020 inhabitants. ^135^ Heart failure and etiologies associated with HF (except primary valvular disease) were responsible for 6.3% of the total deaths. For these data, there was neither distribution nor reclassification of the underlying causes of death, and all etiologies associated with HF were included when considering the impact of HF on total mortality.

• A study of mortality due to HF in three states of Brazil (Rio de Janeiro, São Paulo, and Rio Grande do Sul) included data from 2 960 857 death certificates from 1999 to 2005. The percentages of deaths due to HF were 3.0% in the restricted form (HF as the underlying cause of death) and 9.0% in the comprehensive form (HF mentioned in any line of the death certificate) in 1999. The percentages decreased over time and were 2.4% and 8.6%, respectively, in 2005. The mortality rates decreased in most age groups, except for the group aged 80 years or older. The rates increased with age and were clearly higher among men up to 80 years of age. ^160^

• A Brazilian cohort study showed data of 1220 outpatients in a specialized HF clinic followed up for 26±26 months from 1991 to 2000. Patients were in functional classes III and IV, had a mean age of 45.5±11 years, and 78% were men. The main etiologies were dilated cardiomyopathy (37%), ChD (20%), and ischemic cardiomyopathy (17%). In the follow-up period of 26±26 months, 415 (34%) patients died, and 71 (6%) patients underwent heart transplantation. Chagas disease was a predictor of poor prognosis. ^161^

• More recent data from 700 consecutive patients with HF-REF from the outpatient clinic of a tertiary health center in São Paulo, Brazil, showed 1-year mortality of 6.8% (47 patients). The composite outcome of death and hospitalization was 17.7% (123 patients) and 1% (7 patients) underwent heart transplantation. The patients’ mean age was 55.4±12.2 years, and 67% were men. The main etiologies were hypertensive (26.0%), ischemic (21.9%), and Chagasic (17.0%) forms of cardiomyopathy. High levels of blood urea nitrogen and brain natriuretic peptide, as well as low systolic blood pressure, were independent predictors of 1-year overall mortality in this sample. ^162^

• In a study reporting data from a National Database of Multisite Pacemaker including 3526 patients from 2002 to 2007 cared for at the SUS, the patients’ mean age was 59.8±13.3 years, and 66% were men. The overall survival of patients submitted to cardiac resynchronization therapy in Brazil was 80.1% (95% CI, 79.4–80.8) in 1 year and 55.6% (95% CI, 54.6–56.6) in 5 years, whereas the median overall survival was 30.3 months (IQR, 16.1–50.9). Furthermore, improved survival was observed in the cohort studied, from 2002 throughout 2007 (p=0.055). ^163^

## Hospitalizations

• Hospital admissions are the main consequences of decompensated HF, resulting in worse prognosis and increasing costs. The BREATHE Study evaluated a sample of patients admitted due to acute decompensated HF. A total of 1263 patients were included from 51 centers from different Brazilian regions in 2011 and 2012. In-hospital mortality was 12.6%, and care quality indicators based on hospital discharge recommendations were reached in less than 65% of the patients. ^164^

• Other studies reporting mortality rates before the BREATHE Study ^165^ ^,^ ^166^ showed similar rates of in-hospital mortality, ranging from 9% to 17%. ^165^

• In a comparison of decompensated HF patients in tertiary care teaching hospitals in Brazil and in the US, US patients were older (p < 0.01) and had higher prevalence of the ischemic etiology (p < 0.01). Length-of-stay was significantly shorter (5 [IQR, 3-9] vs. 11 [IIQ, 6-19] days; p < 0.001) and in-hospital mortality was lower (2.4% vs. 13%; p < 0.001) in the US cohort, but fewer clinical events within 3 months after discharge were observed in the Brazilian patients (42% vs. 54%; p = 0.02). That study highlights the importance of improving knowledge about HF in Brazilian patients to improve care and outcomes. ^167^

• In the previously cited systematic review that evaluated the burden of HF in Latin America (64% included studies from Brazil), ^153^ the hospital admission rates were 33%, 28%, 31%, and 35% at 3, 6, 12, and 24 to 60 months of follow-up, respectively. The median hospital length of stay was 7.0 [IQR, 5.20-11.00] days. In-hospital mortality was 11.7% (95% CI, 10.4%-13.0%), and the rate was higher in patients with a reduced ejection fraction, ischemic heart disease, or ChD. The 1-year mortality rate was 24.5% (95% CI, 19.4-30.0).

• Using data from the SUS, the numbers of hospitalizations and deaths due to HF were described in São Paulo, Brazil, from 1992 to 2010. The in-hospital mortality rate due to HF was 15%. Comparing the 1992-1993 and 2008-2009 periods, there was a 32% decrease in the number of hospitalizations due to HF (p = 0.002), a 15% increase in mortality (p = 0.004), and increased hospital length of stay due to HF (from 8.8 to 11.3 days, p = 0.001). ^168^

• Another study with data from the DATASUS evaluated HF admissions over a 10-year period (2007 to 2016) in Brazil as compared to those in the state of Rio Grande do Sul and in the city of Porto Alegre (a city with several referral centers). As depicted in Chart 4-10, that study showed a decline in in-hospital mortality rates from 2007 to 2016, in both Brazil (19% decline) and the state of Rio Grande do Sul (25% decline), and a more pronounced decline in the city of Porto Alegre (65%). ^169^

• In 2020, a more recent study with data from the DATASUS evaluating HF admissions between 2008 and 2017 in Brazil, described 51 172 HF hospitalizations in the period, representing the main cause of hospitalizations for cardiovascular diseases (29.4%). Similarly to the study mentioned above, this study showed a reduction in hospitalizations in the period (34%; p = 0.004). When stratified by age, individuals aged over 60 years accounted for 73% of all cases of HF hospitalization in Brazil. The mortality rate due to HF between 2008 and 2015 was 14.0 per 100 000 (± 0.53), with a 7.7% reduction in the observed period. ^170^

## Burden of Disease

• According to the GBD 2017 estimates (Table 4-11), the age-standardized YLD rates due to HF were 112 (95% UI, 83-141) in 1990 and 109 (95% UI, 81-134) in 2017, per 100 000 inhabitants, corresponding to a decrease of 3% (95% UI, -6.7 to 0.3). These changes are similar to the observed in the HF prevalence rates. Despite this decrease in YLD rates, HF resulted in 88 114 (95% UI, 64 078-112 624) DALYs in Brazil in 1990 and in 234 169 (95% UI, 174 338-291 188) in 2017, due to population growth and aging. ^152^

• Table 4-12 shows YLD rates due to HF according to sex and by age groups, from the GBD Study 2017 estimates. The age-standardized YLD rates were similar in women and men in 1990, but the 2017 rates were 105 (95% UI, 82-127) for men and 111 (95% UI, 80-1416) for women, due to a 6.8% reduction (95% UI, 110.9 to -2.6) for men as compared to almost no reduction for women (-0.3%; 95% CI, -4.9 to 4.2). As expected, the highest YLD rate was observed in the ‘70+ years’ group, followed by the ‘50-69 years’ group. Similarly to the changes observed in prevalence, from 1990 to 2017, the greatest YLD increases were observed in the ‘15-49 years’ group. ^152^

### Health Care Utilization and Cost

**(Refer to Tables 1-6 through 1-9 and Charts 1-15 through 1-1 6)**

• According to data from the SUS, there were 3 085 359 hospitalizations due to HF from 2008 to 2019. This number represents one-third of total clinical admissions related to cardiovascular conditions in the period studied. Unadjusted costs were R$ 3 957 126 308. In international dollars, adjusted total values converted to purchasing power parity for 2019US$ were $ 2 651 479 951. ^95^

• During the period observed, there was a reduction in the number of clinical admissions due to HF from 298 474 (157 per 100 000) in 2008 to 222 620 (105 per 100 000) in 2019, with an even reduction over the years. Despite that reduction in the number of admissions, unadjusted healthcare expenditure estimates from the direct payment for the care of HF patients increased from 2008 to 2019 by almost 32%, from R$ 272 280 662 (2019 Int$ 267 102 469) in 2008 to R$ 359 301 691 (2019 Int$ 173 659 589) in 2019. The decreased number of admissions and increased expenditure represent higher costs *per* admission throughout the observed period (R$ 912 in 2008 to R$ 1568 in 2018). Heart failure accounted for most costs related to clinical admissions due to cardiovascular diseases.

• The economic burden of HF in Brazil was assessed using the standard cost of illness framework in 2015. The analysis assessed the prevalence and associated expenditures on healthcare treatment, productivity losses from reduced employment, costs of providing formal and informal care, and lost wellbeing. The study found that HF imposes a financial cost of R$ 22.1 billion/US$ 6.8 billion, the second out of four major heart conditions in Brazil: myocardial infarction, HF, hypertension, and atrial fibrillation. ^124^

• In the study by Nicolao *et al* ., ^169^ the DATASUS data on HF admissions over a 10-year period (2007 to 2016) showed a 97% increase in the mean per-patient cost of HF-related hospitalizations from 2007 to 2016. Data from the city of Porto Alegre (a city with several referral hospitals) showed an even more pronounced increase (135%), but also a more pronounced decrease in mortality as compared to overall data of Brazil (see above).

### Open Heart Transplantation and Assist Device Placement

• The number of heart transplantations performed in Brazil increased from 149 in 2006 to 357 in 2016. Although the number of heart transplantations increased significantly in that period, it represents approximately one-fifth of the estimated population need. The 1-year survival was 73% (data for survival collected from 2010). ^171^

• An analysis of cost for heart transplantation in Brazil, of all consecutive heart transplant recipients at a single center from July 2015 to June 2017, showed an average total cost for the 27 patients included of US$ 74 341, which is lower than those reported for developed countries, but exceeds the reimbursement value per patient by 60%. ^172^

• In a descriptive study of a public reference hospital in cardiology, located in the city of Fortaleza, Brazil, 16 patients were submitted to ventricular assist device implantation from 2008 to 2015. Mean age was 40.1 ± 3.4 years, and 87.5% were men. Chagas heart disease was the main etiology (37.5%). All patients experienced complications during the use of the device, bleeding being the most frequent (11 [68.8%]). Regarding the clinical outcome, 10 patients (62.5%) underwent cardiac transplantation and 5 patients (31.3%) died. ^173^

## Future Research

• Because HF is considered a garbage code when assigned as the underlying cause of death, studies investigating the better method to reclassify and redistribute this cause are needed to reduce bias and promote better data comparability to enhance health policies.

• Brazilian cohort studies for cardiomyopathies are scarce, and some clinical studies in Brazil reported HF data, but there are few multicenter studies with Brazilian population data. It is worth noting the importance of having data for both HF and cardiomyopathy and for both outpatients and hospitalized ones, in addition to fully understanding the increasing burden of HF on cardiovascular diseases. More multicenter large-scale studies are needed to better describe the burden, outcomes, and costs of HF in the Brazilian population.

• In addition, studies exploring quality of care and costs in HF would help develop health policies to improve awareness, access to life-saving interventions, organ donation, and the better use of resources in this complex and demanding disease.

• Mortality rates due to ChD decreased substantially in the last decades and they are expected to continue to decline in the coming years. Indeed, there is evidence that ChD is an underreported cause of death, and, probably, of hospitalization as well. More data on the hospitalization rates and outcomes in ChCM patients are needed.

## 5. VALVULAR HEART DISEASE

### ICD-9 424; ICD-10 I34 to I38

**See Table 5-1 through 5-5 and Charts 5-1 through 5-11**

**Table:** Abbreviations Used in Chapter 5

AF	Atrial Fibrillation
ARF	Acute Rheumatic Fever
CABG	Coronary Artery Bypass Graft
CAD	Coronary Artery Disease
CI	Confidence Interval
DALYs	Disability-Adjusted Life Years
ECG	Electrocardiogram
FU	Federative Unit
GBD	Global Burden of Disease
HIC	High-Income Countries
ICD	International Statistical Classification of Diseases and Related Health Problems
IL	Interleukine
NRVD	Non-rheumatic Valvular Heart Disease
RHD	Rheumatic Heart Disease
SUS	Brazilian Unified Health System (in Portuguese, *Sistema Único de Saúde* )
TAVI	Transcatheter Aortic Valve Implantation
UI	Uncertainty Interval
YLLs	Years of Life Lost

**Table 5-1:** – Age-standardized prevalence rates of valvular heart disease per 100 000, in 1990 and 2019, and percent change of rates, in Brazil and its Federative Units.

Cause of death and Location	1990		2019		Percent Change (95% UI)
	Number (95% UI)	Rate (95% UI)	Number (95% UI)	Rate (95% UI)	
**B.2.1-Rheumatic heart disease**					**(;)**
Acre	3450.4 (2590.5;4467.5)	871.3 (675.4;1091.9)	8765.8 (6801.6;11017.2)	889.9 (698.1;1114.6)	2.1(-4.3;8.5)
Alagoas	21422.5 (16168.7;27275.5)	870.4 (671.7;1095.4)	34480.6 (26684.6;43431.6)	911.8 (708.5;1147.6)	4.8(-1.4;12.1)
Amapá	2291.4 (1738.2;2949.2)	877.6 (686.6;1102)	8204.3 (6320.1;10401.1)	893.7 (695.8;1127.2)	1.8(-3.6;8.2)
Amazonas	17173.8 (12858.6;21944.2)	859.3 (662.8;1072.3)	39188.9 (30084;49139)	877.9 (681.7;1093.7)	2.2(-4.5;8.7)
Bahia	102924.7 (78855.6;131748.4)	883.1 (691.6;1106.3)	153263.4 (118427;190547.8)	923.2 (714.7;1146.6)	4.5(-1.6;10.7)
Brazil	1352613.2 (1040490.6;1695888.8)	899.6 (699.8;1119.1)	2102091.3 (1639303.8;2606302.5)	918.5 (716;1142.5)	2.1(0.2;4)
Ceará	54893.6 (41770.6;69929.9)	879.9 (684.7;1108.8)	94345.5 (73759;117854.4)	907.6 (712.2;1133.8)	3.1(-3;9.6)
Distrito Federal	14943.3 (11322.7;18780.2)	882.4 (685.5;1098.2)	29679.7 (23036.6;37007.4)	884.6 (683;1107.7)	0.3(-5.5;6)
Espírito Santo	23622.8 (18038;29882.3)	883.7 (685.2;1105.3)	37781 (29560.4;47163.4)	895.2 (696.3;1123.7)	1.3(-4.7;8)
Goiás	38342.4 (29158;48881.8)	894.2 (695;1119.9)	68040.7 (52578.2;84812.4)	911.1 (703.6;1137.2)	1.9(-3.8;9.3)
Maranhão	40195 (30705.5;51338.9)	862.1 (667.6;1078.8)	74568.2 (57721.4;93956.1)	894 (691.7;1118.2)	3.7(-2.4;11)
Mato Grosso	17655.9 (13403.8;22630.5)	866.2 (670.9;1092.4)	34380.9 (26531.8;42865.4)	881.9 (679;1099.6)	1.8(-4.1;7.9)
Mato Grosso do Sul	16461.5 (12566.8;20841.5)	900.8 (701.7;1128.6)	27971.6 (21773.1;34975)	923.4 (716.7;1154.5)	2.5(-3.6;9.2)
Minas Gerais	145136.4 (111832;182475)	893.7 (700.5;1114)	210129.5 (164841.3;261917.1)	917.3 (715.3;1146)	2.6(-3.5;8.1)
Pará	41348.9 (31068.7;52497.1)	871.9 (674.1;1090.2)	87391.4 (67117.7;110404)	890.7 (690.9;1115.6)	2.2(-3.4;8.6)
Paraíba	27841.4 (21209.4;35301.7)	888.2 (685.4;1106.3)	40854.4 (31738.7;51294)	912.9 (708.4;1149.3)	2.8(-3.3;8.8)
Paraná	81156 (62014.8;102568.9)	919.2 (710.1;1145.5)	113818.3 (88747.4;140998)	939.4 (730.5;1177.7)	2.2(-3.3;7.8)
Pernambuco	63259.3 (48155.2;80253)	886 (681.7;1107.1)	96588.1 (74881.1;120052)	913.6 (707.8;1139.5)	3.1(-2.5;10.4)
Piauí	22494.3 (16958.5;28886.4)	890.4 (687.3;1117.5)	34073.1 (26412.8;42807.5)	906.3 (700.6;1139.5)	1.8(-4.7;8.5)
Rio de Janeiro	124439.1 (95279.2;157326.7)	902.8 (694.5;1137.9)	172188.9 (135252.5;212372.6)	920.6 (712.8;1140.7)	2(-4.1;7.9)
Rio Grande do Norte	21271.9 (16243;26755.5)	895.1 (696.6;1119.8)	35469.3 (27439.4;44530.2)	910 (704;1144.4)	1.7(-4.1;8.4)
Rio Grande do Sul	89482.7 (68907.8;113899.5)	930.3 (722.5;1165.4)	111269 (87372;139471)	937.4 (724.5;1173)	0.8(-5.2;6.6)
Rondônia	9709.1 (7330.4;12319.3)	862 (671.8;1077.8)	16995.1 (13142.4;21253.2)	879.4 (681;1097.4)	2(-4;8.1)
Roraima	1816.1 (1377.7;2311.1)	854.8 (666.6;1069.7)	5547.7 (4230.2;6961.8)	878.9 (678.9;1101.6)	2.8(-3.3;8.7)
Santa Catarina	44246.8 (34096.9;55614.1)	936.1 (728;1169.1)	72819.1 (57156.6;90488.1)	942.9 (735.4;1169.9)	0.7(-5.1;6.9)
São Paulo	306459.4 (235629.4;380385.5)	917.2 (710.2;1138)	455434.6 (356653.1;565502.4)	934.7 (731.5;1171.7)	1.9(-3.4;7.7)
Sergipe	12997.3 (9939.7;16567.3)	889.4 (692.3;1115.1)	23773.4 (18367.2;29453.9)	925.7 (715.4;1147.8)	4.1(-2.9;11.1)
Tocantins	7577.3 (5704.2;9694.6)	859.4 (660.2;1078.4)	15068.9 (11720.2;18871.1)	868.5 (675.1;1087.3)	1.1(-4.9;8)
**B.2.5-Non-rheumatic valvular heart disease**					**(;)**
Acre	45.9 (37.3;55)	21.8 (18.2;25.8)	237.7 (200.3;276.7)	33.6 (28.4;39)	53.9(29.2;85.5)
Alagoas	317.7 (260.6;374.9)	20.8 (17.1;24.3)	1007.7 (843.5;1167.9)	29.5 (24.7;33.9)	41.6(15.4;74.6)
Amapá	31.6 (26.1;37.8)	24.8 (20.8;29)	236.1 (197;275.5)	38.4 (32.5;44.8)	54.7(27.4;87.3)
Amazonas	247.3 (204;295.5)	24.3 (20.5;28.5)	1237.1 (1037.9;1449.9)	37.7 (31.7;43.8)	55.2(30.6;84.6)
Bahia	1717.8 (1432.8;2026.8)	23 (19.5;27.1)	4952.1 (4171.7;5747.9)	29.8 (25.1;34.6)	29.2(6.4;55.7)
Brazil	26255.7 (23385;28824.5)	25.3 (22.4;27.8)	95300.5 (82828.7;108921.1)	39 (33.9;44.6)	54.3(41.1;68.3)
Ceará	938.5 (780.3;1092.3)	21.5 (18;25)	3319.7 (2799.3;3846.7)	32.5 (27.4;37.8)	51(25;84)
Distrito Federal	277.5 (231.5;328.5)	30.8 (26.2;35.6)	3271.6 (2650.9;3935.7)	110 (89.2;132)	256.6(194.9;333.7)
Espírito Santo	421.7 (344.2;499.2)	24.1 (20.2;28.2)	1776.4 (1477;2076.8)	38.5 (32.2;44.9)	59.9(32.5;91.1)
Goiás	626.9 (524.7;740.6)	23.6 (20.1;27.7)	2652.7 (2242.2;3112.3)	35 (29.6;40.9)	47.9(23.9;78.7)
Maranhão	583.9 (482.4;690.1)	20.1 (16.8;23.5)	1807.7 (1525;2100.4)	26.1 (22;30.2)	29.9(4.9;59.3)
Mato Grosso	252.7 (209.6;298.6)	23 (19.5;26.7)	1498.6 (1253.1;1755.6)	40.7 (34.2;47.7)	77.2(48.5;112)
Mato Grosso do Sul	288.6 (237.7;341.2)	25.5 (21.3;29.8)	1356.1 (1126;1592)	43.4 (36;51)	70.2(40.9;104.5)
Minas Gerais	2731.8 (2280;3218.2)	23.9 (20.2;28)	8965.3 (7605.9;10440.9)	33.4 (28.3;38.9)	39.9(17.1;69.5)
Pará	577.7 (476.5;682.4)	22 (18.4;25.6)	2437.5 (2051.8;2854.5)	31.4 (26.6;36.5)	42.9(19.1;71.9)
Paraíba	505.8 (426.6;591)	21.8 (18.4;25.4)	1401.1 (1193;1612.9)	29.8 (25.4;34.3)	36.7(13.3;66.1)
Paraná	1569.6 (1320.1;1846.9)	26.2 (22.3;30.5)	5786.1 (4865.1;6888.9)	41.5 (34.8;49.4)	58.4(32.8;87.6)
Pernambuco	1120.9 (946.9;1304.1)	22.4 (19.1;26)	3402.5 (2879.5;3968.3)	32.3 (27.2;37.6)	44(17.7;72.8)
Piauí	333.2 (271.4;393.2)	20.7 (17.2;24.2)	1013.3 (857.1;1175.3)	26.7 (22.6;31)	29.3(6.9;57.3)
Rio de Janeiro	2932 (2439;3452.2)	26.4 (22.3;31)	8945.1 (7447;10466.6)	39.3 (32.8;46)	48.6(23.7;76.8)
Rio Grande do Norte	384.4 (318.7;451.3)	23.2 (19.4;27.1)	1382.3 (1144.1;1600.7)	35 (29;40.5)	50.8(25.6;82.7)
Rio Grande do Sul	2261.2 (1899.8;2643.1)	29.7 (25.1;34.4)	6749.2 (5633.2;7857.4)	43.5 (36.4;50.6)	46.3(23.8;72.5)
Rondônia	130 (107.2;154.2)	23.1 (19.2;26.7)	648.6 (544.5;759.1)	37.4 (31.3;43.7)	61.6(34.5;95.9)
Roraima	21.3 (17;25.7)	22.2 (18.6;25.9)	162.4 (134.6;189.9)	35.6 (29.9;41.3)	60.2(30.6;93.9)
Santa Catarina	841.9 (702.4;975.8)	26.9 (22.6;31.1)	3775.8 (3163.4;4442.1)	43.5 (36.6;51)	61.7(36.3;93.4)
São Paulo	6786.6 (5675;7889)	28.1 (23.7;32.6)	26025.6 (21778.6;30883.2)	46.2 (38.8;54.9)	64.2(38.6;94.8)
Sergipe	196.5 (162.9;230.2)	21.7 (18.1;25.3)	738.2 (628.7;864.3)	30.6 (26;35.7)	41.2(16.6;72.3)
Tocantins	112.9 (93.1;134.4)	21.3 (18;25.1)	514 (434;596.7)	33.4 (28.2;38.7)	56.3(29.1;89.9)
**B.2.5.1-Non-rheumatic calcific aortic valve disease**					**(;)**
Acre	10.1 (8;12.6)	5 (4;6.2)	123.4 (98;151.9)	18.5 (14.6;22.9)	266.7(224.6;314.6)
Alagoas	56.9 (45.1;70.2)	3.8 (3.1;4.7)	444.1 (354.3;546.4)	13.4 (10.7;16.5)	249.8(212;294.5)
Amapá	9.2 (7.3;11.3)	7.6 (6;9.4)	133.4 (106.8;163.6)	23.2 (18.7;28.5)	204.1(173.5;241.7)
Amazonas	70.2 (55.6;86.4)	7.3 (5.8;9)	705.8 (562.7;866.4)	22.6 (18;28)	210.8(179.8;251.5)
Bahia	404.7 (324.5;495.1)	5.5 (4.4;6.8)	2294.2 (1832.8;2814.7)	14.1 (11.2;17.3)	154.2(129.8;184.4)
Brazil	7905.4 (6333;9659.2)	7.9 (6.3;9.6)	57152.9 (45926.6;70348.4)	23.7 (19.1;29)	201.8(177.5;231.8)
Ceará	196.4 (154.7;244.3)	4.6 (3.6;5.6)	1730 (1381.8;2137)	17.2 (13.7;21.3)	276.5(231.9;332)
Distrito Federal	108.8 (85.4;132.8)	13.2 (10.5;16.3)	2764 (2173.3;3401.8)	94.8 (75;117.1)	618.3(520.2;732.3)
Espírito Santo	111.5 (88.6;136.7)	6.6 (5.3;8.2)	1049.8 (835.9;1303.9)	23.2 (18.5;28.6)	251.4(211.7;295)
Goiás	164.1 (130.9;201.4)	6.5 (5.2;8)	1421.1 (1139.4;1754.3)	19.4 (15.6;23.8)	198.4(168;237.7)
Maranhão	95.1 (74.8;119.1)	3.3 (2.6;4.1)	695.4 (554.8;852.2)	10.3 (8.2;12.6)	209.2(176.7;247.1)
Mato Grosso	63.4 (49.8;78.4)	6.2 (4.9;7.6)	919.2 (726;1132.8)	26 (20.7;32)	321(274.6;376.5)
Mato Grosso do Sul	86.4 (68.8;106)	8.1 (6.4;10)	867.8 (687.5;1078.8)	28.3 (22.6;34.8)	249.9(214.5;294)
Minas Gerais	727.1 (568.6;898.5)	6.5 (5.1;8)	4835 (3834.5;5958.1)	18.1 (14.4;22.2)	177.8(150.7;214.2)
Pará	129.4 (102.4;159.9)	5.2 (4.1;6.3)	1199.8 (951.4;1476.5)	16.2 (12.8;20)	214.6(179.7;254.2)
Paraíba	106 (83.3;131.7)	4.5 (3.6;5.7)	669.2 (532.7;821.2)	14.3 (11.4;17.6)	215(182.9;255.2)
Paraná	500.8 (399;618.4)	8.8 (7;10.8)	3677.2 (2950.2;4581.6)	26.7 (21.5;33.1)	204.3(171.8;239.2)
Pernambuco	243.8 (191.2;302.7)	5 (3.9;6.2)	1696 (1349.5;2086.2)	16.5 (13.1;20.2)	230.5(196;271.1)
Piauí	61.4 (48.4;75.5)	3.9 (3.1;4.8)	410.2 (324.2;499.1)	10.9 (8.6;13.3)	180(152.3;219.4)
Rio de Janeiro	907.3 (714.4;1122.4)	8.5 (6.8;10.5)	5421.6 (4290.2;6733.4)	23.8 (18.9;29.4)	181(153.8;214.6)
Rio Grande do Norte	95.8 (76.2;118.1)	5.8 (4.6;7.2)	762.9 (606.7;931.8)	19.6 (15.6;24)	238.6(204.2;283.4)
Rio Grande do Sul	894.7 (716.2;1105)	12.2 (9.7;15)	4459.5 (3551.3;5500.3)	28.5 (22.9;34.9)	133.9(112.6;159.4)
Rondônia	32.2 (25.2;39.9)	6.3 (5;7.8)	369.3 (291;456.9)	22.4 (17.7;27.6)	252.9(213.9;300.7)
Roraima	5.4 (4.2;6.8)	6.1 (4.8;7.5)	88.3 (69.6;109.8)	20.8 (16.6;25.8)	242(206.2;283.6)
Santa Catarina	288.2 (232.8;354)	9.6 (7.7;11.8)	2440.1 (1939.3;3004.9)	28.6 (22.9;35)	198(167.5;231)
São Paulo	2472.7 (1981.2;3024.5)	10.6 (8.5;13)	17366.6 (13732.1;21578.8)	31.1 (24.7;38.6)	193(162;227.9)
Sergipe	39.7 (31.1;49.7)	4.5 (3.6;5.6)	346.4 (275.9;428.6)	14.9 (11.8;18.5)	232(196.5;272.4)
Tocantins	24.3 (19.1;30.2)	4.8 (3.8;6)	262.7 (209.8;323.3)	17.7 (14.1;21.7)	265.3(224.9;313.9)
**B.2.5.2-Non-rheumatic degenerative mitral valve disease**					**(;)**
Acre	47.4 (43.9;50.9)	21.7 (20.2;23.3)	163.5 (151.5;176.9)	21.6 (20;23.2)	-0.5(-4.7;4.2)
Alagoas	346 (320;372.4)	22.2 (20.6;23.9)	761.9 (707.8;817.9)	21.9 (20.3;23.5)	-1.6(-6;2.8)
Amapá	30.3 (28;32.5)	22.2 (20.6;23.8)	147.5 (135.3;160)	21.8 (20.1;23.6)	-1.8(-5.6;2.1)
Amazonas	239.6 (222.1;258.5)	22.1 (20.5;23.8)	760.6 (700.6;827)	21.6 (20;23.4)	-2.4(-6.9;2.3)
Bahia	1701.1 (1585.3;1824.1)	22.3 (20.7;23.9)	3654.4 (3395.5;3938.8)	21.8 (20.2;23.4)	-2.3(-6.1;1.9)
Brazil	24034.9 (22437.7;25715.6)	22.5 (21;24)	53918.2 (50205.1;57756.5)	22 (20.5;23.5)	-2.3(-4;-0.4)
Ceará	979.8 (914;1051.6)	22.1 (20.6;23.7)	2248.8 (2085.5;2425.1)	21.8 (20.2;23.4)	-1.6(-6.1;2.6)
Distrito Federal	224.3 (207.5;242.2)	23.1 (21.5;24.7)	731.7 (675.1;789.5)	22.5 (20.9;24.2)	-2.4(-7.1;2)
Espírito Santo	409.4 (381;439.6)	22.6 (21.1;24.2)	1025 (949;1104.5)	22.2 (20.6;23.9)	-1.7(-5.8;2.5)
Goiás	608.1 (564.3;652.9)	22.1 (20.5;23.7)	1678.8 (1548.4;1815.2)	21.8 (20.1;23.5)	-1.3(-5.6;3)
Maranhão	641.7 (595.4;691.4)	21.7 (20.2;23.4)	1520.8 (1402.1;1649.9)	21.4 (19.8;23.2)	-1.5(-6.6;2.8)
Mato Grosso	251.2 (232.1;271.5)	21.6 (20.1;23.1)	828.9 (766.4;894.9)	21.7 (20.1;23.3)	0.4(-4;4.9)
Mato Grosso do Sul	261.3 (241.6;281.7)	22.1 (20.5;23.7)	692.5 (640.2;746.3)	21.9 (20.3;23.5)	-1.1(-5.7;3.5)
Minas Gerais	2629.8 (2444.6;2826.8)	22.5 (21;24.1)	5799.3 (5351.7;6249.1)	21.9 (20.3;23.6)	-2.6(-7;1.7)
Pará	599.1 (557;645.2)	22 (20.4;23.5)	1752.1 (1625.8;1905.2)	21.5 (20;23.3)	-1.9(-6.1;2.7)
Paraíba	519.3 (482.7;555.7)	22.2 (20.6;23.7)	1015.4 (940.1;1094.2)	21.6 (20;23.3)	-2.6(-6.7;1.8)
Paraná	1395.4 (1296.6;1498.3)	22.6 (21;24.2)	3058.4 (2831.7;3304.5)	22.1 (20.5;23.9)	-1.9(-6.4;2.6)
Pernambuco	1150 (1065.2;1234.1)	22.6 (20.9;24.3)	2351 (2177.2;2534.8)	22.1 (20.5;23.7)	-2.5(-6.6;2.2)
Piauí	357.2 (330.8;383.5)	21.9 (20.2;23.4)	819.1 (761.2;882.8)	21.5 (20;23.2)	-1.4(-6.2;3.2)
Rio de Janeiro	2586.9 (2403.3;2775)	22.9 (21.3;24.6)	4954.2 (4572.2;5335.5)	22.2 (20.5;23.9)	-3.2(-7.4;1.5)
Rio Grande do Norte	376.6 (350.1;404)	22.2 (20.7;23.9)	869 (804.6;936.1)	21.7 (20.1;23.4)	-2.3(-6.4;1.7)
Rio Grande do Sul	1774.1 (1650.6;1908.7)	22.8 (21.3;24.5)	3317.6 (3070.6;3580.6)	22.2 (20.5;23.9)	-2.9(-7;1.2)
Rondônia	128.5 (118.7;139.3)	21.5 (20;23)	389.8 (359.5;421.6)	21.5 (19.9;23.3)	0.2(-3.9;5.1)
Roraima	21.8 (20.1;23.6)	20.9 (19.4;22.5)	104.3 (96;112.7)	21.2 (19.5;22.8)	1.5(-2.6;5.8)
Santa Catarina	740.9 (687.2;794.5)	22.7 (21.1;24.3)	1915.3 (1772.8;2077.6)	22.1 (20.5;23.9)	-2.9(-6.9;1.5)
São Paulo	5691.2 (5272.3;6125.5)	22.8 (21.2;24.5)	12477.6 (11520.7;13408)	22.3 (20.6;23.9)	-2.4(-6.8;2)
Sergipe	207.1 (192.7;221.7)	22.4 (20.9;23.9)	536.6 (497.6;577.2)	21.8 (20.3;23.5)	-2.4(-6.4;1.9)
Tocantins	117 (108.8;126.2)	21.6 (20.2;23.3)	344.2 (318.6;371.1)	21.7 (20.1;23.4)	0.4(-4;4.5)

*Source: Data derived from Global Burden of Disease Study 2019, Institute for Health Metrics and Evaluation, University of Washington. ^46^*

**Table 5-2:** – Death and DALY rates per 100 000 in 1990 and 2019, and percent change of rates, by age, sex, and cause of death, Brazil.

Cause and Location	Death rate 1990	Death rate 2019	Percent change for death rate (95% UI)	DALY rate 1990	DALY rate 2019	Percent change for DALY rate (95% UI)
	(95% UI)	(95% UI)		(95% UI)	(95% UI)	
**B.2.1-Rheumatic heart disease**						
Both						
15-49 years	1.7 (1.6;1.8)	0.6 (0.6;0.7)	-64.6 (-68.3;-60.4)	159.1 (132.1;193.1)	95.5 (68.7;130.8)	-40 (-48.4;-32.1)
50-69 years	6 (5.5;6.5)	2.6 (2.4;2.9)	-55.9 (-61.1;-50.4)	214.1 (194.3;236.2)	113.4 (97.5;132.9)	-47 (-53.2;-40.7)
5-14 years	0.7 (0.6;0.7)	0.1 (0.1;0.2)	-78.1 (-81.9;-73.6)	78.4 (64.6;99)	39 (25.8;60.5)	-50.3 (-62;-38.6)
70+ years	12.5 (11.4;13.6)	6.8 (6;7.6)	-45.4 (-52.5;-38.4)	202.1 (184.6;218.4)	111.1 (98.3;123.3)	-45.1 (-51.7;-39.1)
Age-standardized	2.8 (2.7;3)	1.2 (1.1;1.2)	-59.4 (-63.1;-55.4)	144.6 (126.8;167.3)	79.3 (61.6;102.6)	-45.1 (-52.1;-38.2)
All Ages	2.1 (2;2.2)	1.3 (1.2;1.3)	-39.6 (-45.2;-33.9)	132.8 (114.5;156.3)	85 (66.3;109.9)	-36 (-43.2;-29.2)
Under 5	0.4 (0.3;0.6)	0 (0;0.1)	-88.2 (-93.4;-79.6)	35 (24.5;50.2)	6.6 (4.6;9.2)	-81.3 (-89.1;-69.5)
Female						
15-49 years	2 (1.9;2.1)	0.7 (0.7;0.8)	-63.3 (-67.4;-58.2)	179.2 (149.4;216.1)	109.5 (79.7;149.9)	-38.9 (-47;-30.7)
50-69 years	6.9 (6.3;7.6)	3.1 (2.8;3.4)	-55.2 (-60.7;-49)	250.3 (225.4;278.8)	134.8 (115.2;158.4)	-46.2 (-52.5;-39)
5-14 years	0.7 (0.6;0.7)	0.1 (0.1;0.2)	-77.6 (-81.5;-73.3)	80.8 (65.9;102.2)	42 (27.5;64.6)	-48.1 (-59.8;-36.8)
70+ years	12.7 (11.5;13.9)	7.6 (6.5;8.4)	-40.5 (-48.8;-32)	207.1 (187.7;226)	121.4 (106.1;136)	-41.4 (-48.5;-34.2)
Age-standardized	3.2 (3;3.4)	1.3 (1.2;1.4)	-58.1 (-62.5;-53.3)	162.2 (142.3;187)	90.5 (70.7;116.5)	-44.2 (-51.1;-36.9)
All Ages	2.4 (2.3;2.5)	1.5 (1.4;1.7)	-35.8 (-42.3;-28.6)	149.3 (129.2;174.9)	98.6 (78.1;126)	-34 (-40.9;-26.9)
Under 5	0.4 (0.3;0.6)	0.1 (0;0.1)	-87.3 (-93.2;-77.3)	37.8 (24.7;56.3)	7.4 (5.1;10.3)	-80.5 (-89.1;-66.6)
Male						
15-49 years	1.5 (1.4;1.6)	0.5 (0.4;0.5)	-66.5 (-70.6;-61.4)	138.3 (113.9;169.9)	81.2 (57.8;112.1)	-41.3 (-50.3;-33.4)
50-69 years	4.9 (4.5;5.4)	2.1 (1.9;2.3)	-57.6 (-63.4;-50.7)	174.8 (157.8;193.8)	89.3 (76;105.2)	-48.9 (-55.5;-41.7)
5-14 years	0.7 (0.6;0.7)	0.1 (0.1;0.2)	-78.6 (-82.9;-73.3)	76 (62.4;95.1)	36.1 (23.5;56.5)	-52.5 (-64.3;-40.3)
70+ years	12.3 (11.2;13.6)	5.8 (5.1;6.5)	-52.5 (-60.3;-44.5)	196 (179;215.5)	96.9 (86.2;108.4)	-50.5 (-57.7;-43.1)
Age-standardized	2.5 (2.4;2.7)	1 (0.9;1)	-62 (-66.7;-56.7)	126.1 (109.6;146.8)	67.3 (51.7;87.7)	-46.7 (-54.4;-39.3)
All Ages	1.8 (1.7;1.9)	1 (0.9;1.1)	-45.3 (-51.7;-37.8)	116 (99.4;137)	70.9 (54.3;92.9)	-38.9 (-46.7;-31.5)
Under 5	0.3 (0.2;0.5)	0 (0;0.1)	-89.2 (-94.4;-77.5)	32.3 (20.7;47.8)	5.8 (3.9;8.5)	-82.1 (-90.2;-68)
**B.2.5.1-Non-rheumatic calcific aortic valve disease**						
Both						
15-49 years	0.4 (0.4;0.5)	0.2 (0.2;0.3)	-49.3 (-55.6;-38.9)	23.1 (20.5;26.7)	11.2 (9.6;12.7)	-51.5 (-57.4;-41.8)
50-69 years	3.5 (3.1;3.8)	2.3 (2.1;2.7)	-32.7 (-39.1;-23)	102 (90;111.9)	67 (60.3;78.1)	-34.3 (-40.4;-25.1)
70+ years	14.8 (12.7;16)	17.3 (14.4;20.2)	17 (2;38.5)	209.2 (179.7;226.3)	216.7 (184.1;251.7)	3.6 (-9.5;22.6)
Age-standardized	1.8 (1.6;2)	1.5 (1.3;1.8)	-15.6 (-24;-4)	41.9 (37;45.9)	29.2 (26.3;33.4)	-30.3 (-36.3;-21.3)
All Ages	1 (0.9;1.1)	1.6 (1.4;1.8)	58 (42.2;78.7)	28.6 (25.4;31.8)	31.5 (28.4;36.2)	10.3 (0.7;25)
Female						
15-49 years	0.3 (0.2;0.4)	0.2 (0.1;0.2)	-42.2 (-55.2;-23.5)	14.5 (10.9;19.6)	8 (5.7;10.7)	-44.7 (-57.3;-26.8)
50-69 years	2.3 (1.8;2.8)	1.8 (1.5;2.3)	-23.2 (-32.8;-7.1)	66 (51.1;81.1)	49.4 (40.9;64.3)	-25.1 (-34.2;-9.7)
70+ years	14.6 (11.9;16.2)	17.3 (14;21.1)	18.3 (0.1;45.7)	194.7 (158;215.7)	203 (167;248.8)	4.2 (-12.2;29.4)
Age-standardized	1.5 (1.3;1.7)	1.4 (1.1;1.7)	-10.9 (-21.9;7.4)	30.8 (24.5;36.6)	23.8 (20.3;29.5)	-22.8 (-31.2;-6.5)
All Ages	0.8 (0.7;1)	1.6 (1.3;2)	89.3 (65.8;129.5)	20.7 (16.3;25.4)	27.6 (23.6;34.3)	33.2 (18.6;62.1)
Male						
15-49 years	0.6 (0.5;0.7)	0.3 (0.2;0.3)	-52.8 (-58.3;-43.6)	32.1 (28.3;35.7)	14.5 (12.4;17.1)	-54.7 (-60.2;-46)
50-69 years	4.8 (4.3;5.2)	3 (2.7;3.4)	-37.1 (-44;-29.1)	141 (126.3;151.6)	86.8 (77.9;97.5)	-38.4 (-45.1;-30.9)
70+ years	15.1 (12.9;16.3)	17.4 (14.9;19.8)	15.5 (0.7;34.7)	227.2 (195.3;246.1)	235.4 (203.9;267.6)	3.6 (-9.8;20.7)
Age-standardized	2.1 (1.9;2.3)	1.7 (1.5;2)	-18.1 (-27;-8.8)	53.6 (47.6;57.5)	35.3 (31.7;40)	-34.1 (-40.3;-26.6)
All Ages	1.2 (1;1.3)	1.6 (1.4;1.8)	35.2 (21.4;50.8)	36.7 (32.8;39.8)	35.7 (32.2;40.4)	-2.7 (-11.7;8.4)
**B.2.5.2-Non-rheumatic degenerative mitral valve disease**						
Both						
15-49 years	0.3 (0.2;0.4)	0.2 (0.2;0.2)	-42.1 (-50.5;-30.1)	17.1 (13.2;19.4)	9.4 (8;11.3)	-45 (-52.9;-33.2)
50-69 years	1.6 (1.3;2)	1.2 (0.9;1.4)	-23.6 (-37.4;-13.3)	48.2 (38.8;58.8)	36 (27.5;41.3)	-25.4 (-38.6;-15.6)
70+ years	4 (3.3;5.7)	4.6 (2.9;5.5)	16.5 (-18.5;48.7)	59 (49.4;84)	64.3 (40.8;75.7)	9 (-23.4;39.2)
Age-standardized	0.7 (0.6;0.9)	0.6 (0.4;0.7)	-19 (-34.8;-5.8)	20.4 (16.3;24.6)	14.1 (10.8;16.3)	-30.7 (-41.6;-22.6)
All Ages	0.4 (0.4;0.6)	0.6 (0.4;0.7)	36.2 (11.4;56.6)	15.6 (12.4;18.4)	15.6 (11.9;18)	0.2 (-13.5;11.1)
Female						
15-49 years	0.4 (0.3;0.5)	0.2 (0.2;0.3)	-46.6 (-56.6;-32.4)	21.5 (15.4;25)	10.9 (8.8;13.7)	-49.3 (-58.5;-35.8)
50-69 years	2 (1.5;2.5)	1.4 (1;1.7)	-28.5 (-43.6;-17.8)	59.8 (44.2;75.6)	41.8 (28.8;51)	-30.1 (-44.9;-19.8)
70+ years	4.3 (3.2;6.7)	5 (2.6;6.3)	15.1 (-26.4;55.5)	63.8 (48.6;98.2)	68 (36.8;85)	6.7 (-31.3;43.3)
Age-standardized	0.8 (0.6;1.1)	0.6 (0.4;0.8)	-24.4 (-43.5;-10)	24.9 (18.5;31.3)	15.9 (11.1;19.4)	-36.1 (-48.5;-27.9)
All Ages	0.6 (0.4;0.7)	0.7 (0.5;0.9)	30.4 (-1.6;52.8)	19.6 (14.5;24)	18.5 (12.9;22.4)	-5.8 (-23.4;5.7)
Male						
15-49 years	0.2 (0.2;0.3)	0.2 (0.1;0.2)	-33.9 (-45.9;-17.2)	12.5 (8.9;15.6)	7.9 (5.9;10.1)	-37.3 (-48.4;-19.9)
50-69 years	1.2 (0.9;1.6)	1 (0.7;1.2)	-15.6 (-30.5;0.7)	35.7 (27.1;47.2)	29.4 (19.6;35.8)	-17.5 (-31.5;-1.8)
70+ years	3.5 (2.8;5.2)	4.2 (2.6;5)	17.6 (-18;45.5)	53.1 (42.5;78)	59.2 (37.3;71.1)	11.5 (-22.3;39)
Age-standardized	0.6 (0.4;0.7)	0.5 (0.3;0.6)	-11 (-28.8;4.2)	15.5 (11.7;20.1)	12.1 (8.6;14.5)	-22 (-33.3;-10.2)
All Ages	0.3 (0.2;0.4)	0.5 (0.3;0.6)	45.4 (19.8;69.1)	11.4 (8.4;14.6)	12.6 (8.8;15.2)	10.2 (-5.1;26.4)
**B.2.5-Non-rheumatic valvular heart disease**						
Both						
15-49 years	0.8 (0.7;0.8)	0.4 (0.4;0.4)	-45.5 (-50;-40.3)	40.8 (39.1;42.5)	21.2 (19.8;22.7)	-47.9 (-52;-42.9)
50-69 years	5.1 (4.9;5.4)	3.6 (3.4;3.9)	-29.4 (-34.7;-24.3)	151.3 (145.2;158.3)	104.4 (97.1;111.5)	-31 (-36;-25.9)
70+ years	18.9 (17.3;20.1)	22.2 (18.8;24.3)	17.2 (5.4;27.4)	270.2 (250.3;285.3)	283.7 (247;309.3)	5 (-5.3;13.8)
Age-standardized	2.5 (2.4;2.7)	2.1 (1.9;2.3)	-16.2 (-22.5;-10.3)	62.8 (60.3;65.2)	44 (40.7;47)	-30 (-34.5;-25)
All Ages	1.5 (1.4;1.5)	2.2 (2;2.4)	51.9 (39.8;62.7)	44.6 (43.1;46.2)	47.9 (44.4;51.1)	7.3 (0;15.1)
Female						
15-49 years	0.7 (0.6;0.7)	0.4 (0.3;0.4)	-43.9 (-49.8;-36.2)	36.5 (34.5;38.7)	19.5 (17.7;21.4)	-46.5 (-52;-39.4)
50-69 years	4.3 (4;4.6)	3.2 (2.9;3.5)	-25.1 (-32.7;-16.5)	126.9 (119.5;134.6)	92.7 (84.7;101.1)	-26.9 (-34.1;-18.9)
70+ years	19 (17.1;20.5)	22.5 (18.7;25.3)	17.9 (4.6;31.3)	260.1 (235.5;279.4)	273.4 (233;304.6)	5.1 (-6;17.2)
Age-standardized	2.4 (2.2;2.5)	2 (1.8;2.2)	-15.2 (-23.2;-7.1)	56.2 (53.1;59)	40.3 (36.6;43.8)	-28.2 (-34.5;-21.2)
All Ages	1.4 (1.3;1.5)	2.4 (2.1;2.6)	66.5 (49.8;83)	40.7 (38.8;42.8)	46.8 (42.5;50.9)	14.9 (4.5;26.7)
Male						
15-49 years	0.8 (0.8;0.9)	0.4 (0.4;0.5)	-46.9 (-52.1;-40.8)	45.2 (42.7;48.1)	23 (21.1;25.1)	-49.1 (-54.1;-43.3)
50-69 years	6 (5.7;6.4)	4.1 (3.7;4.4)	-32.5 (-38.7;-25.7)	177.9 (168.9;188.4)	117.6 (108;127.5)	-33.9 (-39.8;-27.2)
70+ years	18.8 (17.2;20)	21.8 (19;23.8)	16.1 (2.6;27.5)	282.7 (260.4;301.7)	297.6 (262.9;325)	5.3 (-6.1;16)
Age-standardized	2.7 (2.5;2.8)	2.3 (2;2.4)	-16.4 (-23.9;-9.5)	69.8 (66.7;73.1)	48.1 (44.5;51.7)	-31 (-36.5;-25)
All Ages	1.5 (1.5;1.6)	2.1 (1.9;2.3)	38 (25.8;50.3)	48.6 (46.5;51.1)	49 (45.4;52.7)	0.9 (-7.3;9.9)

*Source: Data derived from Global Burden of Disease Study 2019, Institute for Health Metrics and Evaluation, University of Washington. ^46^*

**Table 5-3:** – Number of deaths, age-standardized mortality rate (per 100 000) in 1990 and 2019, and percent change of rates, by cardiovascular groups of causes of death, in Brazil and its Federative Units.

Cause of death and Location	1990			2019			Percent Change for rate (95% UI)
	Number (95% UI)	Rate (95% UI)		Number (95% UI)	Rate (95% UI)		
B.2.1-Rheumatic heart disease						(;)	
Acre	6.4 (5.3;7.6)	2.7 (2.3;3.1)	9 (7.3;10.8)		1.3 (1;1.5)	-52.1(-63.3;-39.3)	
Alagoas	50.6 (40.4;65.3)	2.8 (2.2;3.6)	43.4 (35.3;53.2)		1.3 (1;1.6)	-54.9(-68.5;-37)	
Amapá	1.9 (1.5;2.2)	1.3 (1.1;1.4)	4.7 (3.8;5.7)		0.7 (0.6;0.9)	-41.1(-53.3;-24.9)	
Amazonas	18.5 (15.8;21.2)	1.6 (1.4;1.9)	23.6 (19.4;28.7)		0.7 (0.6;0.9)	-56.2(-65.2;-44.6)	
Bahia	243.8 (210.7;286.5)	2.7 (2.3;3.2)	208.7 (169.5;258.2)		1.3 (1;1.6)	-53.5(-65.1;-39.3)	
Brazil	3088.2 (2938.9;3256)	2.8 (2.7;3)	2715.2 (2505.1;2913.1)		1.2 (1.1;1.2)	-59.4(-63.1;-55.4)	
Ceará	78.8 (64.9;94.7)	1.6 (1.3;1.9)	81.7 (67.6;98.7)		0.8 (0.7;1)	-49(-60;-35.2)	
Distrito Federal	43.1 (37.3;50.1)	5.1 (4.4;5.9)	40.7 (32.8;49.6)		1.6 (1.3;1.9)	-68.5(-75.1;-60.2)	
Espírito Santo	47.6 (42.9;52.4)	2.6 (2.3;2.9)	56.1 (45.2;68.8)		1.3 (1;1.6)	-50.4(-60.3;-38.2)	
Goiás	112.9 (95.8;136.2)	4.1 (3.4;4.9)	114.7 (92.7;139.8)		1.6 (1.3;1.9)	-60.6(-69.3;-50.6)	
Maranhão	78.1 (60.4;101.6)	2.2 (1.7;2.9)	69.1 (55.6;84.5)		1 (0.8;1.2)	-56(-69.1;-37)	
Mato Grosso	25.9 (20.9;31.2)	2.2 (1.9;2.6)	33 (26.5;39.8)		0.9 (0.8;1.1)	-57.5(-66.7;-45)	
Mato Grosso do Sul	28.5 (25.2;32.3)	2.4 (2.1;2.7)	29 (23.8;34.8)		1 (0.8;1.2)	-59.7(-67.5;-50.6)	
Minas Gerais	419.6 (379.7;465.1)	3.5 (3.2;3.9)	336.6 (280.4;394.5)		1.3 (1.1;1.5)	-63.1(-69.9;-55.1)	
Pará	58 (49.7;68)	2 (1.7;2.3)	65.4 (55.3;77)		0.8 (0.7;1)	-57.8(-66;-47.2)	
Paraíba	60.7 (49.1;76.6)	2.4 (1.9;3)	50.3 (40.1;62)		1.1 (0.8;1.3)	-54.9(-68.1;-37.8)	
Paraná	197.9 (178.1;217.7)	3.3 (3;3.7)	192.9 (161.2;227.3)		1.5 (1.2;1.7)	-56.2(-63.6;-47.6)	
Pernambuco	149.6 (128.8;175.6)	2.7 (2.3;3.2)	148.4 (122.5;179.2)		1.4 (1.2;1.7)	-46.4(-58.4;-33.3)	
Piauí	31.3 (26.1;36.7)	1.8 (1.5;2.1)	28.4 (23.5;33.8)		0.8 (0.6;0.9)	-58.3(-67;-47.1)	
Rio de Janeiro	295.5 (273.1;320.9)	2.7 (2.5;2.9)	232.3 (193.1;276.8)		1.1 (0.9;1.3)	-59.6(-66.7;-50.8)	
Rio Grande do Norte	40.1 (34;46.9)	2.1 (1.8;2.5)	42.3 (33.7;52.1)		1.1 (0.9;1.3)	-49.9(-61.8;-36.2)	
Rio Grande do Sul	149.9 (135.7;165.7)	2 (1.8;2.2)	126 (104.6;150)		0.9 (0.7;1)	-58.3(-65.3;-49.8)	
Rondônia	13.9 (10.7;16.9)	2.7 (2.3;3.2)	17.7 (14.6;21.1)		1.1 (0.9;1.3)	-60.7(-68.6;-50.3)	
Roraima	1.7 (1.3;2)	1.8 (1.6;2.1)	3.5 (2.9;4.3)		0.8 (0.7;1)	-53.1(-62;-41.8)	
Santa Catarina	79.4 (70.8;89.1)	2.6 (2.3;2.9)	82.7 (67.8;99.3)		1 (0.9;1.2)	-60.5(-67.8;-51.4)	
São Paulo	815.4 (739.4;897.2)	3.5 (3.1;3.9)	629.3 (531.7;737.8)		1.2 (1;1.4)	-65.9(-71.7;-59)	
Sergipe	26.5 (22.1;31.4)	2.7 (2.2;3.2)	30.1 (24.1;37.3)		1.3 (1;1.6)	-53(-64.1;-38.5)	
Tocantins	12.6 (10.1;15.4)	2.4 (2;2.9)	15.9 (12.6;19.7)		1.1 (0.8;1.3)	-56.4(-67.1;-41.3)	
**B.2.5-Non-rheumatic valvular heart disease**						**(;)**	
Acre	2.7 (2.4;3.1)	1.8 (1.6;2.1)	10.2 (8.9;11.6)		1.8 (1.6;2.1)	-1.6(-17.9;18.5)	
Alagoas	23 (20;26.8)	1.8 (1.5;2.1)	61.5 (52.3;70.5)		2 (1.7;2.2)	9.3(-12.2;33.3)	
Amapá	2 (1.8;2.2)	2.1 (1.9;2.4)	9.7 (8.6;10.8)		2 (1.7;2.2)	-7.2(-18.6;6.7)	
Amazonas	17.1 (15.5;19)	2.3 (2.1;2.6)	46.7 (40.1;53.4)		1.7 (1.4;1.9)	-27.7(-37.5;-16.4)	
Bahia	143.8 (124.5;162.7)	2.2 (1.9;2.5)	275.3 (228.2;324.4)		1.7 (1.4;2)	-22(-37.2;-4)	
Brazil	2189.8 (2092.3;2275.8)	2.5 (2.4;2.7)	4842.8 (4326;5225.5)		2.1 (1.9;2.3)	-16.2(-22.5;-10.3)	
Ceará	48.5 (39.4;59.8)	1.2 (1;1.5)	158 (130.5;188.1)		1.6 (1.3;1.9)	33.8(1.7;75.5)	
Distrito Federal	19.6 (17.5;22)	4 (3.5;4.5)	47.9 (41.1;54.8)		2.5 (2.1;2.9)	-38.1(-47.5;-27.5)	
Espírito Santo	41.1 (38;44.3)	3 (2.8;3.2)	109.3 (92.8;124.7)		2.7 (2.2;3)	-11.7(-23.5;0.9)	
Goiás	62.7 (54.1;74.3)	3.2 (2.8;3.7)	138.3 (114.9;164.7)		2.1 (1.7;2.5)	-34.2(-45.8;-20.5)	
Maranhão	29.8 (22.6;39.3)	1.2 (0.9;1.6)	97.4 (82.5;114.8)		1.5 (1.3;1.8)	24.4(-11.1;71.3)	
Mato Grosso	18.4 (15.6;21)	2.4 (2.1;2.7)	56.2 (48.7;64)		1.8 (1.6;2.1)	-24.3(-36.1;-9.3)	
Mato Grosso do Sul	23.7 (21.7;25.8)	2.8 (2.6;3.1)	56.5 (48.7;65.2)		2 (1.7;2.3)	-28.4(-38.2;-16.9)	
Minas Gerais	256.2 (235.7;280.3)	2.7 (2.5;3)	511.4 (442.8;577.5)		2 (1.7;2.2)	-28.2(-37.5;-18.6)	
Pará	44.3 (39;50)	2.2 (2;2.5)	110 (95.1;124.9)		1.6 (1.4;1.8)	-27.9(-39.5;-14.1)	
Paraíba	25.9 (21.9;31.3)	1.2 (1;1.4)	57 (48.1;66.6)		1.2 (1;1.4)	1.2(-21.4;26.7)	
Paraná	152 (143.6;161.2)	3.4 (3.1;3.6)	350.6 (301.7;397.4)		2.8 (2.4;3.2)	-16.2(-27.2;-5.2)	
Pernambuco	97.7 (89.1;106.5)	2.3 (2;2.5)	217.6 (189.9;246.3)		2.3 (2;2.6)	-0.4(-14.9;16)	
Piauí	18.3 (16;21.3)	1.5 (1.2;1.7)	46.5 (38.9;53.4)		1.2 (1;1.4)	-17.1(-32.8;1.3)	
Rio de Janeiro	218.6 (205.1;230.2)	2.4 (2.2;2.5)	418.7 (367;475.5)		1.9 (1.7;2.2)	-19.8(-29.3;-8.9)	
Rio Grande do Norte	23.2 (19.7;27)	1.5 (1.2;1.7)	59.8 (49.2;71.6)		1.5 (1.2;1.8)	2.1(-19.5;28.7)	
Rio Grande do Sul	189.2 (176.5;200.4)	3.1 (2.8;3.3)	447 (383.4;510.1)		2.9 (2.5;3.3)	-5.1(-17.6;8.1)	
Rondônia	8.4 (7;9.6)	2.9 (2.6;3.3)	25.1 (21.4;29.2)		1.8 (1.5;2)	-39.9(-49.5;-28.5)	
Roraima	1.2 (1;1.3)	2.3 (2.1;2.6)	5.3 (4.7;6)		1.8 (1.5;2)	-24.8(-34.5;-13.2)	
Santa Catarina	84.9 (78.8;91.3)	3.7 (3.4;4)	216.9 (187.3;247.6)		3 (2.5;3.4)	-20.8(-31.6;-9.4)	
São Paulo	619.5 (580.7;656.9)	3.2 (3;3.4)	1251.4 (1078.1;1408.6)		2.4 (2.1;2.8)	-23.4(-33.1;-14.5)	
Sergipe	11.2 (9.9;12.6)	1.6 (1.4;1.8)	29.3 (24.8;34.2)		1.3 (1.1;1.6)	-15.7(-29.9;2.7)	
Tocantins	6.9 (5.8;8.2)	2.2 (1.8;2.6)	29.4 (24.7;34.1)		2.2 (1.8;2.5)	-2.8(-22.3;22)	
**B.2.5.1-Non-rheumatic calcific aortic valve disease**						**(;)**	
Acre	1.8 (1.4;2.1)	1.3 (1;1.6)	7 (6;8.1)		1.3 (1.1;1.5)	-2.1(-20.8;27)	
Alagoas	14 (11;16.6)	1.1 (0.9;1.4)	40.3 (33.9;49.1)		1.3 (1.1;1.6)	13.9(-9.6;53.2)	
Amapá	1.4 (1.2;1.5)	1.6 (1.3;1.8)	6.4 (5.6;7.6)		1.4 (1.2;1.6)	-11(-24.5;7.9)	
Amazonas	12.2 (9.9;13.8)	1.8 (1.4;2)	33.7 (28.5;39.4)		1.2 (1;1.5)	-29.2(-40.3;-14.3)	
Bahia	102.5 (82;119.3)	1.6 (1.3;1.9)	190.6 (155.8;234)		1.2 (1;1.4)	-26.6(-42.6;-2.3)	
Brazil	1507.5 (1326;1636.7)	1.8 (1.6;2)	3467.3 (3000.8;4002.6)		1.5 (1.3;1.8)	-15.6(-24;-4)	
Ceará	29.4 (21.7;37.7)	0.7 (0.6;1)	103.9 (84.3;130.5)		1.1 (0.9;1.3)	42.1(5.4;107)	
Distrito Federal	12.7 (11;15.4)	3 (2.5;3.4)	32.7 (27.3;40)		1.8 (1.5;2.2)	-38.4(-48.2;-26.4)	
Espírito Santo	26.7 (23.8;30.6)	2.1 (1.8;2.4)	75.4 (62.3;92.7)		1.9 (1.5;2.3)	-10.8(-24.5;4.8)	
Goiás	42.8 (36.5;52.2)	2.3 (2;2.8)	97.5 (79.5;121.2)		1.5 (1.2;1.9)	-35.4(-46.9;-21.7)	
Maranhão	19.2 (13.4;26.8)	0.8 (0.6;1.1)	67.3 (53.9;81.9)		1 (0.8;1.3)	30.3(-9.6;85.7)	
Mato Grosso	12.5 (10.3;14.3)	1.8 (1.5;2)	38.3 (32.6;45.5)		1.3 (1.1;1.5)	-27.6(-40.7;-10.1)	
Mato Grosso do Sul	16.3 (14.2;18.4)	2.1 (1.8;2.3)	39.7 (33.7;47.3)		1.5 (1.2;1.7)	-30.3(-40.8;-17.4)	
Minas Gerais	177.8 (159.3;198.2)	2 (1.7;2.2)	370.4 (313;436.4)		1.4 (1.2;1.7)	-28.2(-38.2;-17.2)	
Pará	28.5 (23.9;33.1)	1.5 (1.3;1.8)	70.5 (58.8;85.4)		1.1 (0.9;1.3)	-31.4(-44;-14.3)	
Paraíba	16.8 (12.2;20.8)	0.8 (0.6;0.9)	39.5 (32.4;46.8)		0.8 (0.7;0.9)	5.5(-18.6;41.8)	
Paraná	104.6 (91.7;117.3)	2.5 (2.1;2.7)	257.8 (215.7;308.6)		2.1 (1.8;2.5)	-14.4(-26.4;-1.4)	
Pernambuco	60.3 (52.8;68.1)	1.5 (1.3;1.6)	143.8 (120;178.2)		1.5 (1.3;1.9)	3.4(-14.6;27.3)	
Piauí	11.3 (9.1;13.6)	1 (0.8;1.2)	30.3 (24.7;37.8)		0.8 (0.6;1)	-17.5(-35.8;4.5)	
Rio de Janeiro	143.6 (120.7;157.7)	1.7 (1.4;1.8)	301.5 (259.8;349)		1.4 (1.2;1.6)	-15.9(-27.3;-1.2)	
Rio Grande do Norte	16.1 (12.4;19.2)	1 (0.8;1.3)	42.1 (34;51.4)		1.1 (0.9;1.3)	1.6(-21.9;37)	
Rio Grande do Sul	134.9 (116.1;149.5)	2.3 (1.9;2.5)	343 (283.6;408)		2.3 (1.9;2.7)	-1.7(-15.5;15.7)	
Rondônia	5.6 (4.6;6.5)	2.2 (1.9;2.5)	17.5 (14.7;20.6)		1.3 (1.1;1.5)	-42.8(-52.4;-29.8)	
Roraima	0.9 (0.6;1)	1.9 (1.5;2.1)	3.9 (3.3;4.5)		1.4 (1.2;1.6)	-25.9(-36.6;-11.7)	
Santa Catarina	61 (53.1;69)	2.8 (2.4;3.2)	166.5 (137;196.3)		2.3 (1.9;2.7)	-18.6(-30.6;-6.3)	
São Paulo	443.5 (387.3;491.7)	2.4 (2.1;2.6)	910.2 (757.6;1083.5)		1.8 (1.5;2.1)	-24.2(-35.1;-10.3)	
Sergipe	6.8 (5.2;8)	1 (0.8;1.2)	18.4 (15.1;22.4)		0.8 (0.7;1)	-16.9(-33.6;7.9)	
Tocantins	4.1 (3.3;4.9)	1.5 (1.2;1.8)	18.9 (14.9;24.7)		1.4 (1.1;1.8)	-3.8(-25.5;25.5)	
**B.2.5.2-Non-rheumatic degenerative mitral valve disease**						**(;)**	
Acre	0.8 (0.7;1.1)	0.5 (0.4;0.7)	2.9 (2.3;3.9)		0.5 (0.4;0.6)	-2.8(-22.4;27.2)	
Alagoas	8.7 (6.7;10.9)	0.6 (0.5;0.8)	20.2 (15.5;25)		0.6 (0.5;0.8)	0.3(-25.3;38)	
Amapá	0.6 (0.5;0.8)	0.6 (0.5;0.7)	3 (2.2;3.6)		0.6 (0.4;0.7)	0.4(-18.2;21.6)	
Amazonas	4.8 (3.9;6.6)	0.5 (0.4;0.8)	12.2 (9.8;16)		0.4 (0.3;0.5)	-24.5(-40.7;-5.6)	
Bahia	40 (31.9;52.4)	0.6 (0.4;0.7)	81.1 (61.1;101.9)		0.5 (0.4;0.6)	-10(-33.1;20.8)	
Brazil	663.1 (535.3;818.9)	0.7 (0.6;0.9)	1315.1 (943.8;1507)		0.6 (0.4;0.7)	-19(-34.8;-5.8)	
Ceará	17.9 (12.6;23.3)	0.4 (0.3;0.6)	50.6 (36.3;63.9)		0.5 (0.4;0.6)	19.3(-13.5;64.8)	
Distrito Federal	6.7 (5;8.1)	1 (0.7;1.4)	14.4 (9.8;17.7)		0.6 (0.4;0.8)	-39(-57.4;-24)	
Espírito Santo	14 (9.6;16.4)	0.9 (0.6;1)	32.3 (20.7;40.6)		0.8 (0.5;1)	-15.2(-30;2.6)	
Goiás	19.3 (14.4;27.3)	0.8 (0.6;1.2)	38.9 (28.3;50.8)		0.6 (0.4;0.7)	-32.3(-50.2;-13.3)	
Maranhão	9.8 (6.6;12.9)	0.4 (0.2;0.5)	27.6 (21.3;39.4)		0.4 (0.3;0.6)	11.3(-23.4;59.6)	
Mato Grosso	5.7 (4.4;7.2)	0.6 (0.5;0.8)	17 (12.9;20.5)		0.5 (0.4;0.6)	-16.6(-33.9;4.6)	
Mato Grosso do Sul	7.3 (5.6;8.8)	0.7 (0.6;0.9)	16.3 (11.7;19.9)		0.6 (0.4;0.7)	-23.9(-39.7;-6.4)	
Minas Gerais	76.3 (63;100.7)	0.7 (0.6;1)	135 (99.2;162.8)		0.5 (0.4;0.6)	-29.4(-48.9;-13)	
Pará	15.1 (11.8;18.4)	0.7 (0.5;0.8)	36.2 (26.8;44.4)		0.5 (0.4;0.6)	-22.9(-39;-3.7)	
Paraíba	8.5 (6.1;11.5)	0.4 (0.3;0.5)	16 (12.3;23)		0.3 (0.3;0.5)	-8.3(-33.3;25.8)	
Paraná	46.1 (33.9;56.6)	0.9 (0.6;1.1)	88.4 (56.8;110.5)		0.7 (0.4;0.9)	-22.7(-40.5;-4.3)	
Pernambuco	36.4 (27.1;41.8)	0.8 (0.6;0.9)	70.7 (47.2;85.4)		0.7 (0.5;0.9)	-8.6(-27;13.4)	
Piauí	6.7 (5.1;8)	0.5 (0.4;0.6)	15.1 (11.2;19)		0.4 (0.3;0.5)	-17.5(-34.4;3.1)	
Rio de Janeiro	73.5 (62.9;94.3)	0.7 (0.6;1)	113.6 (88.7;145.7)		0.5 (0.4;0.7)	-29.3(-42.5;-16.2)	
Rio Grande do Norte	6.7 (5.2;9.2)	0.4 (0.3;0.6)	16.2 (12.3;21)		0.4 (0.3;0.5)	1.1(-24.9;35.4)	
Rio Grande do Sul	52.8 (39;64.3)	0.8 (0.6;1)	99.5 (60.4;126.5)		0.6 (0.4;0.8)	-16.5(-35.5;3.2)	
Rondônia	2.7 (2.1;3.5)	0.7 (0.6;1)	7.2 (5.7;9.4)		0.5 (0.4;0.6)	-32.7(-51;-16)	
Roraima	0.3 (0.2;0.5)	0.4 (0.3;0.8)	1.1 (0.8;2.1)		0.3 (0.2;0.6)	-27(-39.1;-11.7)	
Santa Catarina	23.4 (16;30.1)	0.9 (0.6;1.2)	48.7 (30.4;61.4)		0.6 (0.4;0.8)	-28.5(-44.4;-12)	
São Paulo	172.3 (133.2;223.6)	0.8 (0.6;1.1)	330.3 (211.8;399.1)		0.6 (0.4;0.8)	-21.7(-44.6;-2.1)	
Sergipe	4.2 (3.3;5.4)	0.5 (0.4;0.7)	10.4 (7.6;13.1)		0.5 (0.3;0.6)	-14.7(-33.1;13)	
Tocantins	2.7 (1.8;3.4)	0.7 (0.5;0.9)	9.9 (6.4;12.5)		0.7 (0.4;0.9)	-1.6(-23.1;29.2)	

*Source: Data derived from Global Burden of Disease Study 2019, Institute for Health Metrics and Evaluation, University of Washington. ^46^*

**Table 5-4:** – Number of DALY, age-standardized DALY rates (per 100 000) in 1990 and 2019, and percent change of rates, by cardiovascular groups of causes of death, in Brazil and its Federative Units.

Cause of death and Location	1990		2019		Percent Change for rate (95% UI)
	Number (95% UI)	Rate (95% UI)	Number (95% UI)	Rate (95% UI)	
**B.2.1-Rheumatic heart disease**					**(;)**
Acre	478.6 (384.9;587.3)	134.1 (109.9;161.8)	753.3 (566.2;993.1)	82.6 (62.9;106.6)	-38.4(-49.7;-27.2)
Alagoas	3473.1 (2746.4;4365.8)	149.5 (120;186)	3192.6 (2447.9;4092.5)	86.5 (66.7;110.6)	-42.1(-56;-27.5)
Amapá	197.9 (146.9;261.3)	83.6 (64.4;107)	573.8 (413;795.1)	66.6 (49.3;90.8)	-20.4(-31.2;-9.4)
Amazonas	1696.9 (1318.6;2188.4)	95.4 (77.2;119.6)	2745.7 (1929.8;3783.2)	64.7 (46.3;87.8)	-32.1(-43.3;-21.6)
Bahia	16674.5 (13969;19833.8)	148 (124.8;175.3)	14372 (11188.8;18390.2)	86.4 (67.4;111)	-41.6(-52.4;-30.7)
Brazil	197716.1 (170430.6;232611.3)	144.6 (126.8;167.3)	184224.8 (143687.3;238145.9)	79.3 (61.6;102.6)	-45.1(-52.1;-38.2)
Ceará	6153.3 (4892.5;7694.2)	101.7 (81.3;125.6)	7192 (5239.3;9577.4)	69.5 (50.6;92.6)	-31.6(-43.4;-20.6)
Distrito Federal	2733 (2325.3;3194.9)	202.3 (175.2;232.6)	2748.9 (2101.9;3590.4)	85.6 (65.8;109.5)	-57.7(-65.7;-49.1)
Espírito Santo	3228.8 (2710.2;3880.5)	133.5 (113.7;157.1)	3625.4 (2794.7;4689.5)	84.1 (64.7;109)	-37(-47.1;-27.4)
Goiás	6941.8 (5867.3;8245.7)	186.2 (158.6;219.8)	6955 (5375.9;8927.3)	92.7 (71.8;118.7)	-50.2(-59.9;-39.7)
Maranhão	5793.2 (4511.6;7327)	128.3 (100.7;161.8)	5937.4 (4413.8;8026.1)	74 (55.7;99.1)	-42.3(-55.9;-27.7)
Mato Grosso	2061.6 (1608.5;2580.3)	117.3 (95.7;140.7)	2777.9 (2060.8;3705.6)	72.4 (53.8;96.2)	-38.3(-49.5;-26.2)
Mato Grosso do Sul	2052.3 (1689.3;2521.1)	126.6 (106.3;152)	2259.9 (1708.7;2978.9)	74.2 (56.2;98.2)	-41.4(-51.5;-32.2)
Minas Gerais	25212.5 (21921.1;29506.4)	170.8 (149.3;197.7)	19945.3 (15436.6;25464.7)	83 (63.9;107.9)	-51.4(-59.8;-42.7)
Pará	4685.7 (3688.2;5883)	110.3 (89.1;134.6)	6570.4 (4804.8;8902.7)	70.1 (52;93.5)	-36.5(-46.9;-26.3)
Paraíba	3869.2 (3097.1;4891.2)	130.2 (104.2;163.2)	3595.1 (2709.1;4709.2)	79.5 (59.9;104.8)	-39(-51.9;-25.6)
Paraná	12144.3 (10386.1;14396.5)	156.5 (135.8;181.9)	11030.6 (8623;14136.8)	87.1 (67.7;113.1)	-44.3(-53;-35.7)
Pernambuco	9944.8 (8279.9;11968.4)	144.5 (121.6;173.2)	9819.1 (7641.7;12313.8)	92.9 (72.5;116.8)	-35.7(-47.6;-24.6)
Piauí	2500.4 (1977.2;3115.3)	105.7 (85.2;129.1)	2580.9 (1896.6;3474.4)	68.7 (50.3;92.8)	-35(-46.1;-23.5)
Rio de Janeiro	18562 (15876.8;22061.5)	142.9 (123.1;167.2)	15385.3 (11850;19928.5)	78.6 (59.8;103.2)	-45(-53.8;-35.9)
Rio Grande do Norte	2723.6 (2217.2;3328.6)	120.4 (99.1;146.3)	3111.1 (2330.5;4079)	79.7 (59.7;104.4)	-33.8(-45.4;-23.4)
Rio Grande do Sul	10128.1 (8278.3;12485.4)	113.1 (93.9;138.1)	8733.1 (6508.8;11731.9)	69.5 (50.3;94.7)	-38.5(-48.3;-29.6)
Rondônia	1122.1 (847.6;1419.2)	124.4 (99.6;149.5)	1430.6 (1081.9;1885.8)	76.1 (58;99.1)	-38.8(-49.7;-26.2)
Roraima	165.6 (124.9;220.2)	95.7 (76.1;120.8)	398 (288.9;538.5)	67.2 (50.1;89.6)	-29.7(-41;-18.6)
Santa Catarina	5344.1 (4385.8;6550.2)	128.9 (108.3;153.5)	5883.4 (4408.2;7698.3)	74.6 (55.6;98.4)	-42.1(-51.6;-33.2)
São Paulo	47070.4 (40270.9;55070.9)	159.3 (138.6;182.7)	39172.3 (30110.2;51125.7)	77.3 (58.5;101.5)	-51.5(-59.2;-43)
Sergipe	1807.3 (1481.5;2208.5)	135.6 (111.5;164.9)	2176.7 (1655.5;2835)	86.2 (66;111.8)	-36.4(-48.6;-23.1)
Tocantins	950.7 (747.3;1192.2)	120.7 (97.2;147.6)	1258.9 (933;1679.4)	74.7 (55.7;98.9)	-38.1(-49.6;-24.9)
**B.2.5-Non-rheumatic valvular heart disease**					**(;)**
Acre	85 (73.5;98.7)	40 (34.9;45.5)	260.4 (229.9;292.8)	37.9 (33.4;42.6)	-5.4(-22.2;15)
Alagoas	663.4 (578.5;772.7)	42.2 (36.8;49.3)	1538.5 (1313.5;1768.2)	45.8 (39.1;52.5)	8.5(-12.8;33.5)
Amapá	64.3 (55.6;72)	47.3 (42;52.1)	262.1 (234.9;291.7)	43.2 (38.4;47.9)	-8.7(-20.5;5.9)
Amazonas	542.6 (482.1;605.8)	52.2 (47.2;57.7)	1191.1 (1029.4;1356.5)	36.9 (31.9;42.1)	-29.2(-38.9;-17.1)
Bahia	4048.8 (3511.8;4582)	51.8 (45.1;58.6)	6278.5 (5226.1;7459.1)	38.3 (31.9;45.4)	-26.1(-41;-8.1)
Brazil	66419.8 (64127.5;68749.3)	62.8 (60.3;65.2)	103773.8 (96149.3;110720)	44 (40.7;47)	-30(-34.5;-25)
Ceará	1261.9 (1037.7;1575.1)	28 (23;34.8)	3539.9 (2933.6;4257.3)	35 (29;42)	25.3(-5.1;65.1)
Distrito Federal	704 (622.6;794.5)	84.4 (74.9;94.3)	1148.6 (990.1;1325.1)	44.2 (38.2;50.5)	-47.7(-55.2;-38.2)
Espírito Santo	1268.1 (1174.9;1371.7)	70.7 (65.4;75.9)	2457.5 (2119.5;2803.4)	56.2 (48.5;64)	-20.5(-31.2;-8.9)
Goiás	2042 (1745.9;2416.1)	76 (65.4;90.2)	3337.7 (2797;3975.1)	46.2 (38.6;54.9)	-39.2(-50.8;-25.1)
Maranhão	893.7 (677.8;1170.3)	30.1 (22.7;39.8)	2260.7 (1897.2;2699.7)	32.7 (27.5;39.1)	8.6(-22.6;51.1)
Mato Grosso	619 (510.5;721.6)	55.9 (47.8;63.7)	1407.6 (1228.7;1606.9)	40.1 (35.1;45.8)	-28.1(-40.1;-12.5)
Mato Grosso do Sul	755 (693.1;822.6)	66.2 (60.9;72.1)	1317.8 (1132.8;1518.3)	43.9 (37.7;50.6)	-33.8(-43.3;-23)
Minas Gerais	8073.1 (7457.7;8872)	68.9 (63.6;75.5)	10888.8 (9597;12210.7)	41.6 (36.7;46.7)	-39.5(-47.1;-31.2)
Pará	1375.1 (1191.3;1561)	51 (44.7;57.4)	2787.7 (2449.2;3195.9)	36.5 (32;41.7)	-28.4(-40.7;-14.4)
Paraíba	643.9 (553.1;788.6)	26.7 (22.9;32.7)	1264.3 (1090.6;1484.8)	26.9 (23.2;31.5)	0.9(-22.1;27)
Paraná	4629.9 (4386.7;4909.4)	79.1 (75;83.9)	7300 (6383.8;8258.8)	55.3 (48.3;62.5)	-30(-39;-20.5)
Pernambuco	2816.5 (2575.4;3061.6)	54.9 (50.3;59.6)	5002 (4359.4;5687.4)	49.1 (42.7;55.6)	-10.6(-24.1;5.1)
Piauí	503.5 (441.6;578)	31.9 (27.9;36.9)	1047.2 (909.9;1190.8)	27.7 (24.1;31.5)	-13.2(-28.9;6)
Rio de Janeiro	6715.9 (6311.1;7069.2)	60.6 (57;63.8)	8742.8 (7701.7;9962.7)	39.9 (35.2;45.2)	-34.2(-42.3;-24.9)
Rio Grande do Norte	596.3 (510.5;694.2)	34.2 (29.3;39.8)	1331.5 (1102.2;1581.2)	33.9 (28.1;40.3)	-0.8(-23;25.9)
Rio Grande do Sul	5430.1 (5055.9;5757.8)	73.2 (68.5;77.5)	8355.5 (7203.2;9441.7)	55.1 (47.6;62.2)	-24.7(-33.8;-14.7)
Rondônia	307.5 (249.9;356.2)	61.6 (53.6;69.4)	627.6 (533.9;731.3)	38.1 (32.6;44.2)	-38.1(-48.8;-24.8)
Roraima	42.8 (36;48.6)	48.3 (43.2;53.2)	139.9 (122.9;158.7)	33.9 (29.9;38.5)	-29.8(-39.2;-18.5)
Santa Catarina	2458.1 (2272.3;2657.3)	81.9 (75.9;88.1)	4423.2 (3874.6;5006.2)	55.2 (48.5;62.4)	-32.5(-41.1;-22.9)
São Paulo	19362.4 (18240;20529.6)	79.7 (74.8;84.5)	25466.7 (22329.9;28597.2)	47.4 (41.5;53.1)	-40.6(-47.7;-33.3)
Sergipe	305.9 (269.7;348.4)	34.1 (30.1;38.4)	705.3 (592.8;831.7)	30 (25.3;35.2)	-12(-28.6;7.7)
Tocantins	211.1 (176.1;249.7)	44.4 (37.2;52.3)	691 (578.1;805.1)	46.1 (38.7;53.5)	3.8(-17.6;31)
**B.2.5.1-Non-rheumatic calcific aortic valve disease**					**(;)**
Acre	51.9 (39;62)	26.5 (20.1;30.9)	163.7 (139;188.2)	25 (21.3;28.7)	-5.7(-24.3;23.4)
Alagoas	363.6 (285.3;425.5)	24.4 (19.2;28.7)	917.3 (762.5;1134.3)	27.8 (23.2;34.4)	14.3(-9.7;53.2)
Amapá	39.3 (32.3;44.9)	31.5 (26.4;35.4)	158.8 (137.4;187.6)	27.7 (24;32.5)	-11.9(-25.8;6.8)
Amazonas	359.5 (291.9;408.9)	36.9 (29.8;41.7)	795.3 (671.5;926.9)	25.5 (21.5;29.8)	-30.7(-41.3;-15.5)
Bahia	2641.7 (2109.8;3085.6)	35.1 (28.2;40.9)	3989.3 (3247;4860.7)	24.5 (19.9;29.9)	-30.3(-45.8;-6.7)
Brazil	42579 (37870.3;47363.9)	41.9 (37;45.9)	68342.2 (61550;78453.3)	29.2 (26.3;33.4)	-30.3(-36.3;-21.3)
Ceará	681.1 (502.2;869.2)	15.6 (11.6;20.1)	2097.8 (1692.1;2650.6)	20.9 (16.9;26.5)	33.7(-1.7;94.9)
Distrito Federal	427.6 (364;525.3)	57 (49.2;67.7)	727.9 (616;894.4)	29.6 (25;36.1)	-48(-55.7;-37.2)
Espírito Santo	751.9 (666.4;894.9)	44.4 (39.5;51.6)	1547.9 (1278.5;1956.8)	35.8 (29.6;45.1)	-19.5(-31.6;-6.3)
Goiás	1300.3 (1084.7;1633.2)	51.4 (43.6;63.1)	2186.1 (1774;2711.5)	30.8 (25.2;38.2)	-40.1(-51.9;-26)
Maranhão	537.9 (372.1;749.1)	18.8 (13.1;26.1)	1430.7 (1131.3;1752.5)	21.1 (16.7;25.8)	12.2(-22.4;60.8)
Mato Grosso	389.5 (311.5;457.7)	37.9 (31.6;43.6)	886.3 (752;1040.4)	26 (22.2;30.6)	-31.6(-44;-14.5)
Mato Grosso do Sul	480 (417.5;547.4)	44.8 (39.3;50.6)	861 (731.5;1024)	29 (24.7;34.5)	-35.2(-45;-22.5)
Minas Gerais	5239 (4676.4;5973.4)	46.4 (41.5;52.2)	7257.9 (6282.1;8566.6)	27.7 (24;32.7)	-40.3(-48.2;-31.3)
Pará	799.5 (667.7;934.9)	32 (26.8;37.2)	1621.9 (1376.2;1975)	22 (18.6;26.6)	-31.3(-44.2;-14.2)
Paraíba	374.9 (262.1;468.5)	15.8 (11.2;19.7)	796.6 (649.7;956)	16.9 (13.8;20.3)	6.6(-17.4;43)
Paraná	2959.8 (2640.8;3386.2)	53.3 (46.9;60.2)	5001.5 (4227.4;5984.7)	38.2 (32.3;45.7)	-28.2(-38.2;-17.4)
Pernambuco	1574.6 (1396;1798.1)	31.9 (28.2;36.2)	3016.7 (2526.4;3737.7)	30 (25.1;37.2)	-6.1(-22.4;14.8)
Piauí	280.7 (223.7;336.3)	18.8 (15.2;22.5)	616.2 (516.4;769.6)	16.3 (13.6;20.3)	-13.3(-31.2;9.6)
Rio de Janeiro	4090.8 (3468;4549.8)	38.3 (32.4;42.2)	5758.8 (4921.1;6639.2)	26.2 (22.4;30.2)	-31.6(-40.4;-20.5)
Rio Grande do Norte	375 (285.6;450.5)	22.1 (16.9;26.5)	853.2 (693.3;1040.7)	21.8 (17.7;26.7)	-1.2(-24.8;32.9)
Rio Grande do Sul	3619.1 (3136.5;4083.7)	50.4 (43.6;56.2)	5938 (4979.2;7063.4)	38.9 (32.7;46.1)	-22.8(-33.1;-10.4)
Rondônia	195.6 (154.3;229.2)	43.3 (36.2;49.3)	403.1 (335.6;477.7)	25.2 (21.1;29.8)	-41.8(-52.8;-26.8)
Roraima	29.8 (21.7;35.6)	36.3 (27.8;41.9)	95.9 (77;112.8)	24.5 (20.4;28.5)	-32.5(-42.3;-18.5)
Santa Catarina	1638.4 (1417.6;1889.5)	57.6 (50.3;65.5)	3162.9 (2677;3733.4)	40 (34;47.1)	-30.5(-40;-19.9)
São Paulo	13095.7 (11482.2;14641.3)	55.8 (48.6;62.1)	17256.1 (14768.7;20498.9)	32.3 (27.6;38.3)	-42.1(-49.8;-30.8)
Sergipe	167.8 (127.4;201.3)	19.8 (15.2;23.5)	398.4 (324.4;492.7)	17.3 (14.1;21.3)	-12.8(-31.7;13.9)
Tocantins	113.9 (91.9;138.5)	26.4 (21.3;31.6)	402.9 (317;537.4)	27.5 (21.8;36.7)	4.2(-20.2;39)
**B.2.5.2-Non-rheumatic degenerative mitral valve disease**					**(;)**
Acre	30.8 (24.8;40.5)	12.7 (10.2;17.1)	87.5 (70.5;117.6)	11.7 (9.4;15.7)	-7.5(-26.1;15.3)
Alagoas	289 (228.4;353.5)	17.2 (13.4;21.2)	594.5 (467.7;729.3)	17.2 (13.4;21)	-0.4(-23.6;32.1)
Amapá	23.9 (18.7;29.1)	15.1 (12;18.8)	95.5 (74.7;115.9)	14.3 (10.9;17.4)	-5.1(-20.3;12.4)
Amazonas	176.4 (144.8;232.6)	14.8 (12.2;20.3)	370.2 (300.2;480.1)	10.7 (8.7;13.8)	-27.5(-40.3;-11.6)
Bahia	1363.3 (1095.7;1693.3)	16.2 (13;20.5)	2193.7 (1725.5;2681.5)	13.2 (10.3;16.2)	-18.4(-36.8;5.3)
Brazil	23163.9 (18446.7;27428.1)	20.4 (16.3;24.6)	33793.5 (25861.5;39014.6)	14.1 (10.8;16.3)	-30.7(-41.6;-22.6)
Ceará	545 (408.1;692.2)	11.6 (8.6;14.9)	1346.8 (1010.7;1698.5)	13.2 (9.9;16.6)	13.7(-17.1;54)
Distrito Federal	268.7 (201.2;319.7)	26.6 (19.4;32.9)	398.9 (287.1;482.7)	13.8 (9.8;16.7)	-48.3(-61.4;-37.1)
Espírito Santo	502.3 (340.2;588.9)	25.5 (17.4;29.8)	867.1 (599.1;1068.3)	19.4 (13.3;23.9)	-23.9(-36.2;-9.6)
Goiás	720.9 (530.3;979.3)	24 (17.8;33.5)	1095.9 (824.4;1416.4)	14.7 (11;18.9)	-38.8(-53.3;-21.5)
Maranhão	326.5 (222.8;439)	10.5 (7.2;13.9)	762.9 (582.9;1040.8)	10.7 (8.2;14.7)	2.2(-30.8;47.5)
Mato Grosso	222.5 (166.6;275.9)	17.4 (13.4;21.6)	495.7 (391.1;597.1)	13.5 (10.5;16.3)	-22.4(-37.3;-4.2)
Mato Grosso do Sul	269.3 (203.6;315.4)	21 (16.1;25)	443.9 (337.5;538.1)	14.4 (10.9;17.5)	-31.4(-43.9;-17.7)
Minas Gerais	2759.7 (2198.7;3466.3)	21.9 (17.7;28)	3473.5 (2676;4153.8)	13.3 (10.3;15.9)	-39.1(-53.7;-27.4)
Pará	547.6 (420.2;655.9)	18.1 (14.1;21.9)	1063.7 (825.2;1291.4)	13.2 (10;16.1)	-26.7(-40.6;-9.5)
Paraíba	248.7 (184.3;329)	10.1 (7.4;13.2)	427.3 (334.8;609.2)	9.2 (7.1;13)	-9(-35.2;22.2)
Paraná	1622.1 (1205.7;1926.7)	25.1 (18.5;30.1)	2183.5 (1520.6;2709.5)	16.2 (11.3;20)	-35.3(-47.5;-22.5)
Pernambuco	1209.1 (901.4;1393.7)	22.4 (16.7;25.8)	1901.1 (1367.5;2278.3)	18.3 (13.1;21.9)	-18.3(-32.7;-1)
Piauí	211.4 (164.6;250.5)	12.5 (9.6;14.9)	404 (321.8;497.4)	10.7 (8.5;13.2)	-14.5(-31.4;4.9)
Rio de Janeiro	2576.8 (2159.7;3166.9)	21.9 (18.4;27.3)	2889.1 (2339.7;3691.9)	13.2 (10.7;16.8)	-39.6(-49.2;-29.6)
Rio Grande do Norte	207.9 (165.8;270.9)	11.4 (9.1;15)	439.4 (341.2;567.7)	11.1 (8.7;14.4)	-2.5(-27.6;27.6)
Rio Grande do Sul	1762.1 (1287.4;2066.8)	22.2 (16.4;26.5)	2310.3 (1546.4;2837.7)	15.4 (10.5;18.9)	-30.5(-42.4;-17)
Rondônia	108.1 (81.9;139.1)	17.6 (14;23.7)	210.4 (169.3;274.4)	12.1 (9.7;15.7)	-31.4(-47.3;-13.3)
Roraima	11.7 (7.8;20.6)	10.8 (7.4;19.5)	36.3 (24;64.6)	7.8 (5.2;14)	-28.4(-41.2;-12.2)
Santa Catarina	801.5 (548.8;987.9)	23.8 (16.2;29.7)	1215.4 (818.2;1494.1)	14.7 (9.8;18.1)	-38.3(-49.6;-26.3)
São Paulo	6133.9 (4630.3;7686.7)	23.4 (17.9;29.9)	7925.7 (5513.9;9414)	14.5 (10.2;17.2)	-37.9(-52.3;-26.2)
Sergipe	132.8 (103.6;165.3)	13.7 (10.8;17.5)	290.8 (222.2;361.9)	12 (9.2;15)	-12.3(-30.2;13.9)
Tocantins	91.9 (63.5;114.4)	17.1 (11.4;21.3)	270.5 (184.9;338)	17.4 (11.8;21.9)	2.1(-20.4;32.7)

*Source: Data derived from Global Burden of Disease Study 2019, Institute for Health Metrics and Evaluation, University of Washington. ^46^*

**Table 5-5:** – Total number of admissions and associated costs in Brazilian Reais (R$) and International Dollars 2019 (Int$) for surgical and clinical procedures to treat valvular heart disease in Brazil, from 2008 to 2019.

Procedure:	2008	2009	2010	2011	2012	2013	2014	2015	2016	2017	2018	2019	Total
Valve surgeries (N)	12,201	12,664	12,169	13,181	13,435	13,067	12,993	12,624	12,432	12,277	12,088	12,771	151,902
Valve surgeries (R$)	R$ 125,954,499	R$ 140,683,968	R$ 142,383,177	R$ 179,111,011	R$ 183,271,263	R$ 178,563,635	R$ 180,088,277	R$ 176,813,774	R$ 175,318,559	R$ 176,135,832	R$ 177,583,849	R$ 187,382,032	R$ 2,023,289,882
Valve surgeries (Int$)	$123.559.115	$128.654.775	$120.060.127	$139.380.403	$132.111.420	$119.760.766	$111.985.310	$102.217.742	$93.772.533	$91.038.538	$89.090.521	$90.566.473	$1.342.197.727
Other valvuloplasties (N)	451	477	445	486	456	527	515	513	399	427	391	450	5,537
Other valvuloplasties (R$)	R$ 1,518,843	R$ 1,661,544	R$ 1,717,544	R$ 1,918,678	R$ 1,870,621	R$ 2,051,540	R$ 2,128,294	R$ 2,085,967	R$ 1,594,213	R$ 1,888,744	R$ 1,689,593	R$ 1,959,571	R$ 22,085,157
Other valvuloplasties (Int$)	$1.489.958	$1.519.473	$1.448.265	$1.493.075	$1.348.440	$1.375.947	$1.323.449	$1.205.917	$852.695	$976.227	$847.637	$947.110	$14.828.198
Mitral valvuloplasty (N)	477	551	478	473	403	431	408	341	206	236	200	195	4,399
Mitral valvuloplasty (R$)	R$ 3,115,254	R$ 3,585,355	R$ 3,147,310	R$ 3,227,816	R$ 2,718,391	R$ 2,970,343	R$ 2,808,556	R$ 2,392,670	R$ 1,377,571	R$ 1,720,524	R$ 1,461,666	R$ 1,430,166	R$ 29,955,628
Mitral valvuloplasty (Int$)	$3.056.009	$3.278.789	$2.653.870	$2.511.818	$1.959.557	$1.992.178	$1.746.460	$1.383.225	$736.820	$889.279	$733.290	$691.235	$21.632.536
Total surgical (N)	13,129	13,692	13,092	14,140	14,294	14,025	13,916	13,478	13,037	12,940	12,679	13,416	161,838
Total surgical (R$)	R$ 130,588,598	R$ 145,930,868	R$ 147,248,032	R$ 184,257,507	R$ 187,860,275	R$ 183,585,519	R$ 185,025,128	R$ 181,292,412	R$ 178,290,344,05	R$ 179,745,101	R$ 180,735,108	R$ 190,771,770	R$ 2,075,330,668
Total surgical (Int$)	$128.105.083	$133.453.038	$124.162.262	$143.385.297	$135.419.418	$123.128.891	$115.055.220	$104.806.885	$95.362.050.10	$92.904.045	$90.671.449	$92.204.819	$1.378.658.462
Valve disease (clinical) (N)	3,237	4,156	3,526	3,637	3,285	2,996	2,753	2,400	2,244	2,231	2,330	2,289	35,084
Valve disease (clinical) (R$)	R$ 1,051,959	R$ 1,589,247	R$ 1,439,424	R$ 1,606,640	R$ 1,509,338	R$ 1,509,785	R$ 1,584,222	R$ 1,672,410	R$ 1,675,284	R$ 1,678,874	R$ 2,043,385	R$ 1,999,540	R$ 19,360,112
Valve disease (clinical) (Int$)	$1.031.953	$1.453.358	$1.213.749	$1.250.253	$1.088.009	$1.012.597	$985.126	$966.836	$896.058	$867.752	$1.025.128	$966.428	$12.757.251

*Source: Data derived from Global Burden of Disease Study 2019, Institute for Health Metrics and Evaluation, University of Washington. ^46^*

**Chart 5-1:** – A: Age-standardized and crude prevalence rates of Rheumatic Heart Disease in Brazil from 1990 to 2019. B: Total prevalent cases of Rheumatic Heart Disease in Brazil from 1990 to 2019.

Source: Data derived from Global Burden of Disease Study 2019, Institute for Health Metrics and Evaluation, University of Washington. ^46^

**Chart 5-2:** – A: Age-standardized and crude prevalence rates of Non-Rheumatic Valvular Heart Disease in Brazil from 1990 to 2019. B: Number of prevalent cases of Non-Rheumatic Valvular Heart Disease in Brazil from 1990 to 2019.

Source: Data derived from Global Burden of Disease Study 2019, Institute for Health Metrics and Evaluation, University of Washington. ^46^

**Chart 5-3:** – A: Age-standardized and crude mortality rates attributable to Rheumatic Heart Disease in Brazil from 1990 to 2019. B: Total number of deaths attributable to Rheumatic Heart Disease in Brazil from 1990 to 2019.

Source: Data derived from Global Burden of Disease Study 2019, Institute for Health Metrics and Evaluation, University of Washington. ^46^

**Chart 5-4:** – Correlation between the percent change in age-standardized mortality rates associated with Rheumatic Heart Disease and the Sociodemographic Index (SDI) in the Brazilian Federative Units in 1990 (A) and 2019 (B).

Source: Data derived from Global Burden of Disease Study 2019, Institute for Health Metrics and Evaluation, University of Washington. ^46^

**Chart 5-5:** – A: Age-standardized and crude mortality rates attributable to Non-Rheumatic Valvular Heart Disease in Brazil from 1990 to 2019. B: Total number of deaths attributable to Non-Rheumatic Valvular Heart Disease in Brazil from 1990 to 2019.

Source: Data derived from Global Burden of Disease Study 2019, Institute for Health Metrics and Evaluation, University of Washington. ^46^

**Chart 5-6:** – Correlation between the age-standardized mortality rates attributable to calcific aortic valve disease and the Sociodemographic Index (SDI) in the Brazilian Federative Units in 1990 (A) and 2019 (B).

Source: Data derived from Global Burden of Disease Study 2019, Institute for Health Metrics and Evaluation, University of Washington. ^46^

**Chart 5-7:** – A: Age-standardized death rates attributable to Rheumatic Heart Disease in Brazil and each region from 1990 to 2019. B: Age-standardized DALY rates attributable to Rheumatic Heart Disease in Brazil and each region from 1990 to 2019.

Source: Data derived from Global Burden of Disease Study 2019, Institute for Health Metrics and Evaluation, University of Washington. ^46^

**Chart 5-8:** – Correlation between the percent change in DALY rates attributable to Rheumatic Heart Disease from 1990 to 2019 and the Sociodemographic Index (SDI) in the Brazilian Federative Units in 1990 (A) and 2019 (B).

Source: Data derived from Global Burden of Disease Study 2019, Institute for Health Metrics and Evaluation, University of Washington. ^46^

**Chart 5-9:** – A: Age-standardized death rates attributable to Non-Rheumatic Valvular Heart Disease in Brazil and each region from 1990 to 2019. B: Age-standardized DALY rates attributable to Non-Rheumatic Valvular Heart Disease in Brazil and each region from 1990 to 2019.

Source: Data derived from Global Burden of Disease Study 2019, Institute for Health Metrics and Evaluation, University of Washington. ^46^

**Chart 5-10:** – Correlation between the age-standardized DALY rates attributable to Non-Rheumatic Valvular Heart Disease and the Sociodemographic Index (SDI) in the Brazilian Federative Units in 1990 (A) and 2019 (B).

Source: Data derived from Global Burden of Disease Study 2019, Institute for Health Metrics and Evaluation, University of Washington. ^46^

**Chart 5-11:** – Expenses associated with the surgical treatment of cardiovascular diseases in Brazil, according to the type in SUS, from 2008 to 2020 (Currency: International Dollars). ^95^

## Prevalence

### Rheumatic Heart Disease

• According to the Global Atlas on Cardiovascular Disease Prevention and Control, updated by the GBD 2019, RHD is estimated to currently affect about 40.5 million people worldwide, accounting for 1-1.5% (306 000 deaths) of all cardiovascular deaths. ^4^ ^,^ ^174^ Until the middle of the 20th century, RHD was the leading cause of heart valve disease in the world. Improved health conditions, early identification of *Streptococcus pyogenes* infections, and antibiotic use have significantly decreased RHD prevalence in HIC. Published data from 2016 estimate that RHD is the primary cause of 2.5% of valvular heart diseases in the United States and Canada, reaching up to 22% in Europe. ^175^ Even higher rates have been reported in Brazil, accounting for about 50% of the heart valve surgeries in SUS. ^176^

• In low- to middle-income countries, the prevalence of RHD remains around 444 per 100 000 inhabitants. ^4^ ^,^ ^179^ In Brazil, it persists as the main etiology of valvular heart disease, especially in patients from the SUS. Previous evaluations have shown prevalence of 360 per 100 000 in our country. ^180^ Other evaluations have found a prevalence ranging from 100 to 700 per 100 000 schoolchildren. ^181^

• In Brazil, of 174 patients presenting with acute valvular heart disease to the emergency room at the São Paulo Heart Institute, rheumatic involvement was observed in 60%, followed by degenerative aortic valve disease (15%), and mitral valve prolapse (13%). At total, 27.5% of the patients had isolated mitral regurgitation and 11% had mitral stenosis, with aortic valve disease present in the remaining patients. ^182^

• A recent study in Brazil has shown that the regression rates of valvular heart disease, especially in patients with moderate/severe ARF, may be lower than previously described. Only 22/69 patients had total regression of mitral regurgitation after rheumatic carditis (31.9%). Aortic regurgitation also has a lower total regression rate than those observed in studies prior to the echocardiographic era (18%). However, most cases persisted with mild mitral or residual aortic regurgitation. ^183^ In another study involving 258 children and adolescents with ARF followed up from 2 to 15 years, valve lesions healed in 25% of the patients with mild carditis, in 2.5% of those with moderate carditis, while no healing was observed in those with severe carditis. ^184^

• Regarding disease progression, an echocardiographic risk score for prediction of mid-term outcomes in children with echo-detected RHD has been derived from a Brazilian cohort, ^185^ with good discrimination in a second screening sample in the country ^186^ (C-statistic=0.71, 95% CI, 0.63 - 0.80) and in pooled cohorts from 3 other countries (C-statistic=0.70, 95% CI, 0.64 - 0.76). Its application may be useful for detecting individuals at higher risk for late sequelae. ^187^

• In addition to the echo score, other markers of unfavorable outcomes of latent and clinical RHD (noticeably progression to clinically prevalent disease and the need for valve intervention, respectively) have been evaluated in Brazil. Among individuals with latent RHD, IL-4, IL-8 and IL-1RA seem to be the best predictors of clinical disease. In addition, the co-regulated expression of IL-6 and TNF-α is associated with severe valve dysfunction, whereas high IL-10 and IL-4 levels predicted subsequently adverse outcomes in individuals with established disease. ^188^

• In another genetic study comparing samples from individuals with latent and clinical RHD and controls, clinical disease associated with higher levels of all cytokines, IL-4, CXCL8 and IL-1RA being its strongest predictors compared to latent RHD. An association of polymorphisms in IL-2, IL-4, IL-6 and IL-10 genes and clinical RHD was observed, and gene polymorphism and phenotypic expression of IL-4 accurately discriminate latent *versus* clinical RHD, and may potentially guide clinical management in the future. ^189^

• According to the GBD 2019, from 1990 to 2019, the age-standardized prevalence of RHD had a slight 2.1% (95% UI, 0.2 – 4.0) increase, from 899.6 (95% UI, 699.8 – 1119.1) to 918.5 (95% UI, 716 – 1142.5) per 100 000, remaining higher in women than in men in the whole period (Table 5-1 and Chart 5-1.A). Although small for both sexes, the percent increase was numerically more pronounced in women (3.5%) than in men, who had a stable trend (0.1%, 95% UI, -2.4 – 2.8). The percent increases were higher in the states of Alagoas, Bahia, and Sergipe, from the lowest-income Northeastern region. Although the central estimates were higher in these states, the 95% UIs were wide, and overlap with those of other FUs (Table 5-1). ^190^ However, it may be hypothesized that the small increase of RHD prevalence observed in the period may reflect the progress of epidemiological data collection and health statistics, as well as the systematic inclusion of definite subclinical RHD. ^4^ ^,^ ^18^

• The crude prevalence of RHD, however, increased 6.8% (95% UI, 1.7 – 12.5%) from 1990 to 2019, from 908.8 (95% UI, 699.1 - 1139.4) to 970.2 (95% UI, 756.6 - 1202.9) per 100 000, also remaining higher in women in the whole period (Chart 5-1.B). Similarly to the trend observed for age-standardized prevalence rates, the increase in crude prevalence was more pronounced for women than for men (8.4% vs. 4.2%). ^4^ ^,^ ^18^

• Even with the relatively stable trends depicted by the GBD 2019 modeling, RHD is the most prevalent cause of mitral valve disease in Brazil according to published data, when both mitral stenosis (over 90%) and mitral regurgitation (about 55-60%) are considered. ^182^

• Mitral stenosis occurs in a much higher frequency in women than in men, in a ratio of 3:2. It is a frequent sequela of ARF, reaching more than 85% of cases even in HIC, such as those in Europe, ^191^ with a similar pattern still observed in Brazil. ^178^ ^,^ ^182^ More rarely, mitral stenosis associates with other diseases, such as mitral annulus calcification, mucopolysaccharidosis, rheumatoid arthritis, and congenital carcinoid syndrome. ^175^ ^,^ ^182^

• Recent large-scale screening study looking at subclinical RHD has shown a prevalence of 42 per 1000 in schoolchildren (mean age of 11 years) in the Southeastern state of Minas Gerais: 37 per 1000 borderline RHD and 5 per 1000 definite RHD. In that study, higher prevalence was observed in females (48 per 1000 vs. 35 per 1000) and in children older than 12 years. ^176^ The same project has concluded that primary care centers are the ideal scenario for RHD screening, considering the higher population participation and involvement rates. ^192^

• Echo screening in the primary care setting has also been tested as an additional tool, on top of clinical variables, to predict major abnormalities in standard echo (including significant valve disease and RHD) and prioritize referrals in Brazil. The combined score – derived from 603 patients in waiting lists for echocardiography, and validated in a similar sample of 1526 individuals – had good discrimination, with C-statistic = 0.72, sensitivity = 99% and negative predictive value = 97%, and emerges as a promising tool for early diagnosis of valve disease/RHD, estimation of disease burden and priority referrals. ^193^

• One additional tool for estimating the prevalence of subclinical RHD is artificial intelligence. The machine learning system (Convolutional Neural Network) currently under development in Brazil from screening studies has shown good accuracy (normal=72.8±10.2; borderline RHD=64.3±12.2; and definite RHD=85.8±11.3) and may help broaden the availability of early echocardiographic diagnosis in the future. ^194^

#### Non-rheumatic Valvular Heart Disease

• According to the GBD 2019, the age-standardized prevalence of NRVD had a marked increase in Brazil from 1990 to 2019, with a significant 54.3% increase from 25.3 (95% UI, 22.4 - 27.8) per 100 000 in 1990 to 39 (95% UI, 33.9 - 44.6) per 100 000 in 2019. The percent change was considerably higher for men compared to women (105.9% vs. 20.9%) (Chart 5-2.A). The increasing trend was mostly driven by calcific aortic valve disease (201.8%), from 7.9 (95% UI, 6.3 - 9.6) per 100 000 in 1990 to 23.7 (95% UI, 19.1 - 29) per 100 000 in 2019, for both men (218.8%) and women (182.2%). Conversely, for mitral degenerative valve disease the age-standardized prevalence was stable, with a slight percent change: -2.3% (95% UI, -4 to -0.4) (Table 5-1). ^4^ ^,^ ^18^

• Aligned with age-standardized rates, the crude prevalence of NRVD showed a marked 149.3% (95% UI, 126.7 - 173.3) increase from 1990 [17.6 (95% UI, 15.7 - 19.4)] to 2019 [44 (95% UI, 38.2 - 50.3)] per 100 000 (Table 5-1 and Chart 5-2.B). Again, the increase was more pronounced for men than for women, and the steeper inclination compared to age-standardized rates suggests that prevalence is increasing disproportionally in the elderly (Chart 5-2.B). ^4^ ^,^ ^18^

• Unlike mitral valve disease, aortic valve disease is predominantly degenerative or calcific. Observational studies show that aortic stenosis is seen in 4.5% of the population >75 years in HIC, such as the United States. ^195^ According to observational studies ^182^ ^,^ ^196^ and the GBD 2019 data, ^18^ in Brazil, as well as in the rest of the world, an increasing trend towards degenerative aortic valve disease has been observed as compared to other etiologies, such as RHD.

• Thus, the increase in all-age prevalence of NRVD has been mostly driven by calcific aortic valve disease [396.6% (95% UI, 353.1 – 450.0)], especially for older age groups (>70 years) (Table 5-2), but increasing trends have been also significant for mitral degenerative valve disease [54.1% (95% UI, 50.4 – 58.0)] and other NRVDs, although data quality remains limited for the latter, despite the improvement of data sources (Table 5-1). ^4^ ^,^ ^18^

• Contrary to that observed for the rheumatic etiology, there has been an increase in mitral valve prolapse as the etiology of primary mitral regurgitation in Brazil: although in the overall population it reaches rates around 1-2.5%, with good prognosis in most cases, of the patients admitted with valve disease in a Brazilian emergency department in 2009 (56±17 years, 54% female), 13% had that etiology. ^181^ Conversely, in a hospital registry of heart surgeries in one of the largest capitals of the country (city of Salvador), from 2002 to 2005, only a small proportion of cases was associated with mitral prolapse, ^178^ similarly to the results of a study with 78 808 patients utilizing 2 large national databases (the Brazilian Hospital Information System and the Mortality Information System) from 2001 to 2007, in which only 0.24% (187) of the cases reported that undelaying cause. ^197^ However, data may be biased by the absence of coding for etiologies of valvular haert disease in the public and most of the private health systems.

## Incidence

• According to a study based on in-hospital medical charts in Northeastern Brazil from 2002 to 2005 (1320 surgeries), the mean annual incidence for cardiac valve surgeries was 4.75 per 100 000 residents and positively associated with age. The mean annual incidence of RHD and degenerative valvular disease were 2.86 and 0.73 per 100 000, respectively. ^178^

### Rheumatic Heart Disease

• For RHD, the age-specific incidence followed a bimodal distribution according to the source of surgery reimbursement, increasing almost linearly by 1 case per 100 000 population for each decade of life until the age of 40–49 years, peaking at 4.85 cases per 100 000 population. Following a decline, a second peak occurred at 60–69 years of age (6.54 cases per 100 000 population). ^178^

• The incidence of RHD remained stable [percent increase: 0.5% (95% UI, -1.2 - 2.6)] in Brazil, ranging from 53.9 (95% UI, 40.4 - 67.5) per 100 000 (95 299 cases) in 1990 to 54.2 (95% UI, 40.7 - 68.5) per 100 000 (108 204 cases) in 2019, according to GBD 2019 data. This stable trend was relatively homogeneous across the country, with overlapping 95% UIs even in the lower resourced states of the Northern and Northeastern regions. ^4^ ^,^ ^18^

• In general, a significant reduction in the incidence of RHD was observed in the age group 15 – 49 years [-12.7% (95% UI, -17.2 to -6.8)], while trends were stable in the other age groups. This pattern may be associated with improved disease diagnosis and prevention in younger groups (noticeably 5 – 18 years, the peak of ARF incidence) in past decades, resulting in a lower incidence in teenagers and adults. However, the decrease in the age group 5 – 14 years was not captured by the model, presumably influenced by the lack of data on subclinical RHD prior to 2014. ^4^ ^,^ ^18^

### Non-Rheumatic Valvular Heart Disease

• In a different pattern as compared to that of RHD, the NRVD age-standardized incidence rates had a significant 11.1% (95% UI, 6.5 - 16.4) increase from 3.6 (95% UI, 3.3 – 4.0) per 100 000 in 1990 to 4.0 (95% UI, 3.6 - 4.5) per 100 000 in 2019, according to the GBD 2019 estimates. This increase was mostly driven by the 20.1% (95% UI, 12.9 - 28.4) increase in calcific aortic valve disease, markedly in individuals aged 50 - 69 years [56.9% (95% UI, 39.8 - 75.6)]. ^4^ ^,^ ^18^

• However, the aforementioned increasing incidence of calcific aortic valve disease in middle-age individuals [15 – 49 years: 56.9% (95% UI, 39.8 - 75.6)] is atypical, considering the disease epidemiology, and may be cautiously interpreted as a possible limitation of the GBD modeling, ^18^ ^,^ ^46^ because primary data for this cause are scarce in Brazil. In addition, a non-expected stable pattern in individuals >70 years was modelled. ^4^

## Mortality

• Valvular heart disease is one of the leading causes of cardiovascular death in Brazil, particularly in economically underserved regions, and RHD – the most socially driven etiology – ranked as the 8^th^/9^th^cause in past decades. ^18^ In the most underserved setting, RHD has been playing an important role for decades, with decreasing trends – not always adequately captured by statistical modeling – following socioeconomic improvement. ^174^ ^,^ ^179^ ^,^ ^197^

• Although nationwide data are still scarce, the sub-analysis of a multicenter cohort with 920 patients who underwent heart valve surgeries (isolated aortic valve replacement, 34%; isolated mitral valve replacement, 25%; 81% covered by the public health system) showed an acceptable surgical mortality rate (7.3%) ^198^ as compared to previous series (11.9%). ^178^

### Rheumatic Heart Disease

• Contrasting with the increasing trend of prevalence, age-standardized mortality rates attributable to RHD significantly decreased by 59.4% in Brazil, from 2.8 (95% UI, 2.7 – 3.0) to 1.2 (95% UI, 1.1 - 1.2) per 100 000, according to the GBD 2019 study. The percent decrease was similar for men (62.0%) and women (58.1%) (Table 5-3 and Chart 5-3.A). A similar trend was observed for the crude mortality rates (Chart 5-3.B). During the period, the total number of deaths decreased from 3088 (95% UI, 2939 - 3256) to 2715 (95% UI, 2505 - 2913) in 1990 and 2019, respectively, despite population growth (Table 5-3). These trends may reflect improvement in health conditions, and better and earlier access to healthcare. ^4^ ^,^ ^18^

• In 1990, RHD ranked 10^th^among the causes of death in Brazil (9^th^to 12^th^in different states), and moved to 12^th^in 2019 (10^th^to 13^th^in most states, and 14^th^only in the West-Central state of Mato Grosso do Sul). ^4^ ^,^ ^18^

• The more compelling decrease in mortality rates was observed in lower ages, especially in the ‘5-14 years’ and ‘15-49 years’ age groups: -78.1% (95% UI, -81.9 to -73.6) and -64.6% (95% UI, -68.3 to -60.4) per 100 000, respectively (Table 5-2). ^4^ ^,^ ^18^ This may be associated with the better treatment of pharyngitis, ARF, and early presentations of RHD, while chronic sequelae still persist as a challenge. ^199^ ^,^ ^200^

• According to the GBD 2019 data, there was a significant negative correlation between the percent change in age-standardized mortality rates and SDI in 1990 (Chart 5-4) (r=-0.41, p=0.03) and in 2019 (r=-0.44, p=0.02). ^4^ ^,^ ^18^ Considering RHD as the most socially-driven etiology of valvular disease, this antecipated pattern differed from the GBD 2017 estimates, which did not reach statistical significance in 2017. This may suggest that, despite the undoubtable socioeconomic improvements observed in all Brazilian regions since 1990, which impacted different aspects of disease prevention and healthcare and significantly reduced the sociodemographic gap, inequality still plays an important role in RHD mortality. ^4^ ^,^ ^18^

### Non-rheumatic Valvular Heart Disease

• According to the GBD 2019 study, age-standardized mortality rates attributable to NRVD showed a 16.2% (95% UI, 10.3 - 22.5) decrease from 1990 to 2019 (Table 5-3 and Chart 5-5.A). However, for crude mortality rates a significant increase was observed [51.9% (95% UI, 39.8 - 62.7)], with a considerable contribution of older ages, markedly over 70 years [17.2% (95% UI, 5.4 - 27.4)] (Table 5-3 and Chart 5-5.B). The patterns were similar for men and women. Similar trends were observed for calcific aortic valve disease mortality rates, with a marked 17% (95% UI, 2.0 - 38.5) increase in the elderly (≥70 years), reflecting the association with population aging and prevalent cardiovascular risk factors (Table 5-2). For mitral degenerative valve disease, the age-standardized mortality rates decreased 19.0% (95% UI, 5.8 - 34.8 ), as opposed to a 36.2% increase in the crude prevalence (Tables 5-1 and 5-3), as a result of the increasing rates [16.5% (95% UI, -18.5 - 48.7)] in septuagenarians and older (Table 5-2), however with wide UIs. ^4^ ^,^ ^18^ ^,^ ^19^

• The increasing mortality rates in older ages due to NRVD noticeably contrasts with the trends observed for RHD, possibly reflecting a higher prevalence and, consequently, mortality in the age groups >70 years, for both aortic and mitral NRVD (Table 5-2). From 1990 to 2019, an increasing burden of calcific aortic valve disease, in both males and females, associated with an increase in mortality in that age group. The 95% UIs are overall wide for NRVD mortality estimates, especially for each specific valvular involvement in separate. ^4^ ^,^ ^18^

• In 1990 NRVD ranked 10^th^among causes of death in Brazil (8^th^to 11^th^in different states), and proportionally increased to 9^th^in 2019 (8^th^to 10^th^in most states), the opposite trend of RHD (Chart 5-4). ^18^

• GBD 2019 data demonstrated significant correlations between the changes in age-standardized mortality rates of NRVD in general and SDI in 1990 (r= -0.55, p=0.003) and 2019 (r= -0.58, p=0.001), and a similar pattern for calcific aortic valve disease (1990: r= -0.51, p=0.007; 2019: r= -0.54, p=0.003). Strong positive correlations were observed between age-standardized mortality and SDI in both 1990 (r=0.80, p<0.001) and 2019 (r=0.70, p<0.001) (Chart 5-6). As socioeconomic development correlates with epidemiological transition and life expectancy, a higher SDI associates with more elderly individuals at risk for degenerative valvular conditions and less prone to infectious etiologies, such as RHD. However, in Brazil, socioeconomic conditions – and possibly access to optimal healthcare – still played an important role on changes in NRVD mortality over time. ^18^

• Similar correlations were observed for degenerative mitral valve disease. On the other hand, for other NRVDs, no significant correlation was observed between age-standardized mortality rates – and their percent changes over time – and SDI. ^18^

## Burden of Disease

### Rheumatic Heart Disease

• According to GBD 2019 data, the age-standardized DALY rate attributable to RHD significantly decreased 45.1% in Brazil, from 144.6 (95% UI, 126.8 - 167.3) per 100 000 in 1990 to 79.3 (95% UI, 61.6 - 102.6) per 100 000 in 2019 (Chart 5-7.A). The decreasind rates observed in the period were similar for men and women, -46.7% (95% UI, -54.4 to -39.3) and -44.2% (95% UI, -51.1 to -36.9), respectively (Table 5-4). ^201^

• Age-standardized DALY rates decreased in all Brazilian states, with a steeper trend in the regions with highest rates in 1990: the West-Central and Southeast (Table 5-2). The Southeastern and West-Central regions had the highest age-standardized DALY rates and proportional DALYs in 1990, while four states of the Northeast (Sergipe, Bahia, Alagoas, and Pernambuco), one from the South (Paraná), and one from the West-Central (Goiás) topped the list in 2019. ^4^ ^,^ ^18^

• A similar downward pattern was observed for the age-standardized YLL rates due to RHD, which ranged from 102.1 (95% UI, 97.5 - 107.3) per 100 000 in 1990 to 35.8 (95% UI, 33.5 - 38.4) per 100 000 in 2019, with a 64.9% (95% UI, 61.6 - 67.9) decrease. ^4^ ^,^ ^18^

• The GBD 2019 estimates showed no correlation between age-standardized DALY rates due to RHD and SDI in 1990 or in 2019. Similarly, the SDI did not correlate with the percent change in age-standardized DALY rates in 1990 (r= -0.36, p=0.06), and a weak correlation was observed in 2019 (r= -0.41, p=0.03) (Chart 5-8), suggesting that the effects of socioeconomic development were less pronounced over morbidity, compared to mortality.

### Non-rheumatic Valvular Heart Disease

• According to the GBD 2019, the age-standardized DALY rates of NRVD decreased (30%) in Brazil, from 62.8 (95% UI, 60.3 - 65.2) per 100 000 in 1990 to 44 (95% UI, 40.7 - 47) per 100 000 in 2019 (Table 5-4 and Chart 5-9.B). The decrease pattern observed in the period was similar for men (-31%) and women (-28%). Regarding specific diseases, rates decreased equally for mitral degenerative valve disease [-30.7% (95% UI, -41.6 to -22.6)] as compared to calcific aortic valve disease [-30.3% (95% UI, -36.3 to -21.3)]. For other NRVD, the age-standardized rates tended to increase [19.1% (95% UI, -21.1 - 68.5)], although UIs were considerably wide, including the 0, in this case. The trends observed for YLLs were similar. ^4^ ^,^ ^18^

• The downward trend was relatively homogeneous accross Brazilian states, and the age-standardized DALY rates remained higher for the Southern and Southeastern regions during the whole period, in addition to the Northeastern state of Pernambuco (5^th^place in 2019) (Chart 5-9.B). The most significant decrease was also observed in the Southeastern and West-Central regions (Distrito Federal and the states of São Paulo, Minas Gerais, Goiás and Rio de Janeiro) in addition to the Northern state of Rondônia (which lacks primary data for most estimates). ^4^ ^,^ ^18^

• Similarly to that observed for mortality, the decreasing age-standardized DALY rates of NRVD contrast with the slight increase of crude rates [7.3% (95% UI, 0 - 15.1)] in the period (1990 – 2019), driven by the >70 age-group (+5%) and again suggesting that morbidity associated with NRVD is shifting to the elderly, presumably following changes in the population age composition. ^4^ ^,^ ^18^

• The proportional DALY rates in Brazil increased, and, from 1990 to 2019, the Southern and Southeastern regions accounted for the highest DALY proportions in the period, according to GBD estimates. ^201^ ^,202201^

• In addition, according to GBD 2019 data, there were significant positive correlations between the age-standardized DALY rates of NRVD in general and the SDI in 1990 (r=0.80, p<0.001) and in 2019 (r=0.55, p<0.001) (Chart 5-10). Percent changes in age-standardized DALY rates (1990 – 2019) also correlated with the SDI in 1990 (r= -0.72, p<0.001) and in 2019 (r= -0.72, p<0.001). For calcific aortic valve disease, significant correlations were observed between DALYs and the SDI in 1990 (r^2^=0.80, p<0.001) and in 2019 (r^2^=0.62, p<0.001), as well as between percent changes in DALY rates and the SDI in both years, suggesting that socioeconomic development is also a determinant of degenerative NRVD, linked to aging and risk factors.

• For degenerative mitral valve disease, a positive correlation between age-standardized DALY rates and the SDI was observed in 1990, but not in 2019, whereas percent changes in DALY rates correlated negatively with the SDI in 1990 and in 2019, suggesting some impact of socioeconomic markers on this condition.

## Complications and Associated Diseases

### Arrhythmias Associated with Valve Disease

• For patients with valvular heart disease, AF is also an aggravating factor and usually occurrs in those with more advanced natural history. It is more commonly associated with mitral valve disease, especially mitral stenosis. In a cohort of 427 patients (mean age 50±16 years, 84% female) with severe mitral stenosis, AF was observed in 34% of them, being more frequent in those who died during follow-up (27 of 41; 66%) as compared to survivors (114 of 378; 30%), reinforcing its role as a prognosis marker in valve disease. ^203^

• In addition, AF can develop in severe aortic valve disease, especially in older and postoperative patients. In a retrospective cohort of 348 patients (mean age, 76.8±4.6 years), postoperative AF was observed in 114 (32.8%), but rates were higher in patients ≥ 80 years as compared to 70-79-year-old patients (42.9% vs. 28.8%, p=0.017). ^204^

• In another retrospective assessment conducted in the state of Pernambuco (Northeastern Brazil), involving 491 consecutive patients after heart valve surgery, the incidence of AF was 31.2% and was associated with age >70 years (OR=6.82; 95% CI, 3.34 - 14.10, p <0.001), mitral valve disease (OR=3.18; 95% CI, 1.83 - 5.20, p<0.001), and no postoperative use of beta-blockers, among other factors. ^205^

• Valvular heart disease (17.5%) and arrhythmias (AF and atrial flutter, 50.7%) were the main cardioembolic sources of stroke in a study involving 256 patients (60.2 ± 6.9 years, 132 males) in the Southern region of Brazil. ^206^

• In the BYPASS registry, a multicenter cohort, of the patients undergoing valve heart surgeries, the most frequent postoperative complications were arrhythmias (22.6%), followed by infections (5.7%), and low-output syndrome (5.1%). ^198^

### Association Between Valvular Heart Disease and Coronary Artery Disease

• Due to the increased surgical risk of combined valve procedures and coronary revascularization, it is essential to recognize the prevalence of obstructive CAD in association with valvular heart disease. Studies have shown a lower prevalence of CAD in patients with RHD as compared to those with NRVD, possibly as a reflection of the lower median age of RHD patients and the higher prevalence of coronary risk factors in NRVD. ^207^

• In a study in Rio de Janeiro (Southeastern Brazil) including 1412 candidates for cardiac surgery of any indication, 294 with primary valvular heart disease of rheumatic and non-rheumatic etiologies were selected. All 294 patients were ≥40 years-old and had coronary angiography performed. The prevalence of obstructuve CAD in RHD and NRVD patients was 4% and 33.6% (p <0.0001), respectively. Characteristics and risk factors, such as age, typical chest pain, hypertension, diabetes mellitus, and dyslipidemia, were significantly associated with obstructive CAD. ^208^

• In another study in Brazil, evaluating 712 patients with valvular heart disease (mean age, 58±13 years), the incidence of obstructive CAD was 20%. However, in younger patients (<50 years) prevalence was much lower (3.3%). ^209^ These data are similar to those observed in another study that included 3736 patients (mean age, 43.7 years), in which prevalence of obstructive CAD combined with valvular heart disease was 3.42%. ^207^

## Healthcare Utilization and Cost

• According to the SUS administrative database, the total crude expenses (reimbursement) with hospital admissions for clinical treatment of valvular heart disease in Brazil showed a significant 90% increase, from R$ 1 051 959 in 2008 to R$ 1 999 540 in 2019 in an almost-linear pattern. Ajusting and converting these values to international dollars in 2019, the total costs for the public system were $ 1 031 953, in 2008, and $ 966 428, in 2019, for the treatment of hospitalization due to valvular conditions, a 6.3% reduction. ^210^

• Similarly, unadjusted costs associated with valvular surgical/interventional procedures (codes related to valve surgery, percutaneous mitral commissurotomy, other types of valvuloplasty) also increased from 2008 to 2019, from R$ 130 588 598 (2019 Int$ 128 105 083) to R$ 190 771 771 (2019 Int$ 92 204 819), although with less magnitude as compared to clinical admissions (46% vs. 90%). After adjustment for Int$, a remarkable 28.0% reduction was observed. The total expense with surgical procedures for the SUS in this time series (2008 – 2019) was R$ 10 524 044 511 (Int$ 6 853 635 725) (Table 5-5 and Chart 5-11). ^210^

• The number of surgical/interventional admissions related to valve diseases remained relatively stable in Brazil from 2008 to 2019, ranging from 12 679 in 2008 to 14 294 in 2019. This is presumably associated with the growing complexity and costs of interventions (markedly, hospital costs, devices and prostheses) and denotes the economic burden posed by the incorporation of new procedures and technologies, but it is also a marked effect of inflation on healthcare costs – considering the lower values in 2019 when adjusted to Int$. In this scenario, the future incorporation of well established therapies not yet reimbused by the SUS, such as TAVI, will contribute to increase the economic burden, although expenses with judicial demands may easily overcome ordinary costs. ^211^

• The total number of hospital admissions due to valve disease (clinical and surgical) in this period was 196 922, and most of them occurred in the Southeastern region (41.2%), followed by the Northeastern (25.7%), Southern (20.2%), West-Central (7.5%), and Northern (5.4%) regions (Table 5-5). ^210^

• A dramatic drop was observed for some types of procedures, despite their growing indications, such as the percutaneous mitral commissurotomy. For this specific procedure, the downward numbers may be associated with the lagged reimbursement tables of the SUS, limiting the number of hospitals that perform this intervention. The absolute number of open heart valve surgeries remained stable, from 12 201 in 2008 to 12 771 in 2019, despite the growing number of cases of valvular heart disease – especially NRVD – and the growing burden in the elderly, as population ages. ^190^ ^,^ ^211^

• In none of the periods, the increase in the number of admissions paralleled the increasing expenses, suggesting not only a progressive complexity – and, consequently, cost – of the procedures to treat heart valve disease, but also inflation over medical devices and associated hospital costs (considering the Int$ values) (Table 5-5 and Chart 11). ^210^

• From the SUS administrative database, valve procedures associated with RHD sequelae cannot be differentiated from those associated with other etiologies, since no specific coding is available, and the reporting of the ICD coding is imprecise. ^210^

• Interestingly, observational studies have reinforced that RHD remains as the main etiology associated with cardiac surgery in young people in Brazil, reaching up to 60% in a study performed in the city of Salvador, Bahia (Northeastern Brazil). ^178^ At the São Paulo Heart Institute (Southeastern region), the number of heart valve surgeries associated with RHD increased substantially over the past 10 years, from around 400 surgeries/year in 1990 to over 600 after 2000. ^121^ Between 2008 and 2015, there were 26 054 hospital admissions due to ARF sequelae, 45% of which due to heart disease, leading to a possibly underestimated total cost of US$ 3.5 million annually. ^177^ ^,^ ^210^

• According to observational studies and hospital-based registries, overall valve diseases of rheumatic origin account for about 90% of the cardiac surgeries in children and for over 30% of the cardiac surgeries in adults, most of them in young ages. ^135^ However, few epidemiological studies estimated the cause-specific burden of valve diseases in Brazil.

• Echocardiographic screening for latent RHD has proven to be cost-effective in one study conducted in Brazil. A strategy based on task-shifting, with imaging acquisition by non-physicians utilizing handheld devices and remote telemedicine interpretation by experts, resulted in an Incremental Cost-Effectiveness Ratio of $10 148.38 per DALY averted, below the estimated threshold of 3 times the gross domestic product per capita, suggested by the World Health Organization. ^212^

• Data from the national database of the SUS (DATASUS) show that from 2008 to 2017 there were 42 720 and 78 966 hospital admissions due to ARF and chronic RHD, respectively, accounting for 0.4% and 0.7% of cardiovascular admissions in the country, respectively. This analysis, however, lacks data from specific NRVD. ^213^

### Mitral Valve Disease

• Based on SUS administrative data from 2001 to 2007 and regarding mitral valve surgery, in a retrospective series of 78 808 consecutive surgical patients, the mean age was 50.0 years (35.9 - 62.5) and 40 106 were females (50.9%). Again, RHD was the main etiology, accounting for 53.7% of the total patients undergoing surgery and for over 94% of those undergoing procedures due to mitral stenosis. Mitral stenosis was the largest single surgical indication, accounting for 38.9% of the total. Overall, valve replacement was done in 69.1% of the surgeries. In-hospital mortality was 7.6%. ^197^

• Surgical mortality was slightly higher in women than in men (7.8% vs. 7.3%; p <0.001), and considerably higher in people ≥80 years. On the other hand, the lowest mortality was observed for those between 20 and 39.9 years (p <0.001). Patients with combined aortic and mitral surgeries (reflecting rheumatic etiology) were the youngest (median, 43.3 years). Surgery for aortic stenosis was more common in older individuals (median, 58.0 years) (p <0.001). Valve repair had lower mortality (3.5%) as compared to valve replacement (6.9%), multiple valve repair and/or replacement (8.2%), and concomitant CABG (14.6%) (p<0.001). Associated CABG occurred in 7147 patients (9.1% of the sample). ^197^

• Regarding percutaneous mitral commissurotomy, studies in Brazil show a much higher proportion of females (85%) – coincident with the epidemiology of RHD and noticeably mitral stenosis – and of young people (<40 years). ^214^ ^,^ ^215^ As local expertise in this procedure develops, technical aspects are being investigated, such as the routine utilization of conscious sedation (low-dose midazolam and fentanyl) resulting in anxiolysis and analgesia, without hemodynamic effects. ^216^ Furthermore, there are continuous efforts to define predictors of long-term clinical outcomes, such as changes in invasive and non-invasive atrioventricular compliance. ^217^

• In a retrospective study aimed at evaluating mitral valve repair in 54 Brazilian children (<16 years) with early chronic RHD, no perioperative death was recorded. The most frequent late (>7 days) outcomes were residual mitral lesion (n=11) and need for reoperation (n=3). Thus, mitral repair remains a reasonable strategy for RHD at younger ages. ^218^

### Aortic Valve Disease

• A cohort of 724 consecutive patients, who underwent cardiac surgery at the São Paulo Heart Institute, has evidenced, similarly to other studies, a higher percentage of women (55%) and predominance of RHD (60%). However, in that series, there was a great proportion of aortic valve disease (396 cases) over mitral valve disease (306 cases) as compared to other series. Of the patients with mitral valve disease, 39.9% had stenosis, 38.4% regurgitation, and 21.7% mitral prosthesis dysfunction. In patients undergoing aortic valve interventions, stenosis was observed in 51.6%, regurgitation in 29.3%, and prosthesis dysfunction in 19.1%. The study suggests an increase in aortic valve disease as compared to mitral valve disease in a tertiary hospital in the Southeastern region of Brazil. ^219^

• Another retrospective cohort study has been conducted in the city of Porto Alegre (Southern Brazil) with 1065 patients (mean age, 61.4 ± 11.8 years; 38% women). Aortic valve replacement was done in 18.8% and mitral valve replacement, in 13.4%. Concomitant coronary revascularization was performed in 60.3% of the sample, and valve surgeries in 32.7%. Overall in-hospital mortality was 7.8%, being lower for isolated CABG (5.9%), intermediate for valve surgery (aortic and/or mitral and/or tricuspid = 8.6%), and higher for combined valve and CABG procedures (20.0%). ^220^

### Transcatheter Aortic Valve Implantation in Brazil

• As in other countries, TAVI gained importance in Brazil in the past 20 years. It is estimated that over 100 000 percutaneous aortic valve implantations have been performed worldwide to date. ^196^ ^,^ ^211^ The first TAVI in Brazil occurred in 2008. The Brazilian TAVI registry reported 418 TAVI in 18 centers until 2014, and this number has grown exponentially since then. Femoral access was the choice in 96.2% of the procedures, and the prostheses used were CoreValve® (86.1%) and Sapien XT® (13.9%). Of that initial experience, all-cause mortality at 30 days and 1 year were 9.1 and 21.5%, respectively. ^221^

• Data from the TAVI registry updated in 2017 revealed a total of 819 patients under clinical follow-up, demonstrating that the procedure has a low incidence of complications – especially early hard cliical outcomes – and highlighting rates of postprocedural renal failure around 18%. ^222^ ^,^ ^223^

• In another assessment performed in the city of Rio de Janeiro, of 136 patients undergoing TAVI [median age, 83 (80-87) years; 51% males], perioperative mortality was 1.5%; 30-day mortality, 5.9%; in-hospital mortality, 8.1%; and 1-year mortality, 15.5%. ^224^

• Of 819 percutaneous aortic valves implanted until 2017, 135 patients (20.1%) required permanent pacemaker implantation. These patients were older (82.5 vs. 81.1 years; p=0.047), predominantly male (59.3% vs 45%; p=0.003), and had previous right bundle-branch block (OR=6.19, 95% CI, 3.56 - 10.75, p≤0.001). The use of CoreValve® prosthesis (OR=3.16, 95% CI, 1.74 - 5.72, p≤0.001) and baseline transaortic gradient >50 mm Hg (OR=1.86, 95% CI, 1.08 - 3.20, p=0.025) were independent predictors of permanent pacemaker implantation. ^223^

### Future Research

• Even considering the noticeable improvement in past decades, there is still paucity of primary data about the epidemiology of valvular heart disease in Brazil, and room for future research.

• Administrative data collection should be included, with the development of specific coding to allow for discrimination of variables, such as the valve involved, type of valvular dysfunction, type of prosthesis, and, especially, etiology and association with systemic diseases. This is especially important in the SUS.

• In addition, the development of nationwide registries on valve disease and procedures is warranted. Refining the coding system and implementing mandatory clinical and surgical reports – as previously done for percutaneous coronary interventions – may be an initial step to improve data acquisition.

• As the country has significant cohorts of patients with valvular heart disease, mid- and long-term follow-up of these samples are warranted. Of note, there are research initiatives that require incentives and funding for their continuation, such as ongoing studies on long-term prognosis of subclinical RHD in children and adolescents, ^176^ ^,^ ^192^ genetic and immune determinants of response to streptococcal infections leading to RHD, ^225^ clinical and procedural predictors of short- and long-term events after percutaneous mitral commissurotomy, ^203^ ^,^ ^226^ and a national TAVI registry. ^221^

• One study suggests that echo screening for RHD is cost-effective in Brazil, ^212^ thus, its application outside research and integration into health systems should be investigated in large-scale programs.

• In addition, continuing efforts have been directed to the development of vaccines for streptococcal infections, ^225^ and collaborative studies on their efficacy and clinical application to reduce RHD burden are warranted.

• As reimbursement for TAVI has just been approved in the Brazilian private health system, its incorporation in the Brazilian SUS seems to be close, ^211^ and a comprehensive evaluation of its actual clinical, budgetary and social impact on public healthcare outcomes requires extensive research and funding.

• Finally, promising strategies to provide early diagnosis and prioritization of referrals in low-resourced areas should be further investigated in Brazil. As an example, the availability of imaging modalities for the management of valvular heart disease – markedly echocardiography – is limited and unequally distributed in the country. In this scenario, the implementation of tele-echocardiography, with task-shifting of imaging acquisition to non-physicians (still not allowed by Brazilian healthcare regulations outside research) and remote reading, has already been evaluated, and implementation should be considered. ^227^ Despite its good overall diagnostic performance and discrimination of patients at higher cardiovascular risk, ^227^ the impact on clinical outcomes and cost-effectiveness of the strategy are yet to be explored.

• There is room for improvement of remote cardiac diagnosis in Brazil, through the expansion of tele-ECG, AF screening, ^228^ remote consultations – including those for infective conditions, such as COVID-19 ^229^ – and the incorporation of imaging innovations to improve access to cardiovascular care. Extensive discussions are required, based on robust scientific evidence.

## 6. ATRIAL FIBRILLATION AND ATRIAL FLUTTER

### ICD-10 I48

**See Tables 6-1 through 6-5 and Charts 6-1 through 6-3**

**Table:** Abbreviations Used in Chapter 6

AF	Atrial Fibrillation
BNP	B-type Natriuretic Peptide
CABG	Coronary Artery Bypass Grafting
ChD	Chagas Disease
CI	Confidence Interval
DALYs	Disability-Adjusted Life Years
ECG	Electrocardiogram
ELSA-Brasil	The Brazilian Longitudinal Study of Adult Health
FU	Federative Unit
GARFIELD-AF	The Global Anticoagulant Registry in the FIELD-AF
GBD	Global Burden of Disease
HF	Heart Failure
HR	Hazard Ratio
ICD-10	International Statistical Classification of Diseases and Related Health Problems, 10^th^Revision
IMPACT-AF	A Multifaceted Intervention to Improve Treatment With Oral Anticoagulants in Atrial Fibrillation
INR	International Normalized Ratio
NOAC	New Oral Anticoagulants
OR	Odds Ratio
PPP	Purchasing Power Parity
SDI	Sociodemographic Index
SUS	Brazilian Unified Health System (in Portuguese, *Sistema Único de Saúde* )
TIA	Transient Ischemic Attack
TTR	Time in Therapeutic Range
UI	Uncertainty Interval
VKA	Vitamin K Antagonists
YLDs	Years Lived with Disability
YLLs	Years of Life Lost

**Table 6-1:** – Number of prevalent cases and age-standardized prevalence rates (per 100 000 inhabitants) of atrial fibrillation and flutter in 1990 and 2019, with percent change of rates, according to sex and age group, in Brazil

Sex and Age	1990		2019		Percent change
	Number (95% UI)	Rate (95% UI)	Number (95% UI)	Rate (95% UI)	(95% UI)
**Both**					
15-49 years	19888 (12331.5;30044)	25.9 (16.1;39.2)	42502.2 (26157.4;64165.2)	36.8 (22.6;55.6)	41.8 (37.2;46.9)
50-69 years	164660.9 (115683.1;228396.1)	1049.6 (737.4;1455.9)	444122.5 (311101;613445.2)	1100.9 (771.1;1520.6)	4.9 (2.9;6.8)
70+ years	217157.3 (158121.9;292116.4)	5133.7 (3738;6905.7)	740228.7 (542333.8;984791.4)	5655.6 (4143.6;7524.1)	10.2 (6.8;15)
Age-standardized	401706.1 (302349.2;518702.6)	519.4 (393;668.6)	1226853.4 (934018.3;1577999.6)	537.3 (409.2;692.5)	3.5 (1.8;5.1)
All Ages	401706.1 (302349.2;518702.6)	269.9 (203.1;348.5)	1226853.4 (934018.3;1577999.6)	566.2 (431.1;728.3)	109.8 (103.4;117.4)
**Female**					
15-49 years	7612.1 (4581.8;11533.4)	19.6 (11.8;29.6)	16625.9 (10080.7;25447.2)	28.4 (17.2;43.5)	45.4 (38.6;52.3)
50-69 years	68973.4 (48140.1;96070)	845 (589.8;1177)	191525.6 (133320.6;267053.6)	894.4 (622.6;1247.1)	5.8 (3;8.5)
70+ years	105220.9 (76305.5;141439.1)	4486.9 (3253.9;6031.4)	379236.7 (275610.2;508021.7)	5022.5 (3650.1;6728.1)	11.9 (7.5;18)
Age-standardized	181806.4 (136384.7;235600.5)	437.3 (331.2;566.3)	587388.3 (445578.6;762442.3)	454.9 (345.1;591.9)	4 (1.9;6.5)
All Ages	181806.4 (136384.7;235600.5)	241.5 (181.2;313)	587388.3 (445578.6;762442.3)	529.8 (401.9;687.7)	119.4 (111.3;129.4)
**Male**					
15-49 years	12275.9 (7602.8;18468.4)	32.6 (20.2;49)	25876.3 (16054.7;38681.1)	45.4 (28.2;67.9)	39.4 (33.9;45.1)
50-69 years	95687.5 (67028.6;132303.1)	1271.6 (890.7;1758.1)	252596.9 (176982;344940.7)	1334.5 (935;1822.3)	4.9 (2.3;7.6)
70+ years	111936.4 (81555.8;150612.8)	5938.2 (4326.5;7990)	360992 (264762;481432)	6518.7 (4781;8693.6)	9.8 (6.4;14)
Age-standardized	219899.7 (164842.2;283209.5)	618.6 (468.5;792.3)	639465.2 (486071.9;821088.6)	643.4 (489.2;828.7)	4 (1.9;6)
All Ages	219899.7 (164842.2;283209.5)	298.9 (224.1;385)	639465.2 (486071.9;821088.6)	604.4 (459.4;776.1)	102.2 (96.2;108.8)

*Source: Data derived from Global Burden of Disease Study 2019, Institute for Health Metrics and Evaluation, University of Washington.^*46*^*

**Table 6-2:** – Number of prevalent cases and age-standardized prevalence rate (per 100 000 inhabitants) of atrial fibrillation and flutter in 1990 and 2019, with percent change of rates, in Brazil and Brazilian Federative Units

Location	1990		2019		Percent change
	Number (95% UI)	Rate (95% UI)	Number (95% UI)	Rate (95% UI)	(95% UI)
Acre	658 (494.3;846.9)	477.6 (363.8;609.8)	2719.6 (2059.4;3482.1)	495.6 (375.3;633.2)	3.8(-1.2;8.8)
Alagoas	5737.2 (4311.7;7420.5)	480.6 (364.9;617.3)	14985.3 (11478;19154.2)	496.6 (379.7;636.9)	3.3(-1.9;7.6)
Amapá	382.2 (289.6;491.2)	460 (350.2;590.3)	2167.2 (1660.1;2770.8)	481.3 (367.1;620.4)	4.6(-0.5;9.8)
Amazonas	3032.8 (2269.6;3906.7)	474.9 (360.2;611.7)	12830.4 (9829.7;16439.3)	492.9 (376.6;632.3)	3.8(-1;9)
Bahia	29515.5 (22145.1;37849.7)	481.3 (364.7;615.4)	78718.3 (60254.3;100714.7)	496.7 (377.6;636.5)	3.2(-1.3;7.9)
Ceará	18182.8 (13882.4;23457.1)	473 (361.6;609.1)	47310.1 (35919.5;61015.3)	484.6 (367.2;624.8)	2.4(-1.9;7.3)
Distrito Federal	2005 (1503.1;2616.3)	466.1 (355;598.2)	11277.1 (8448.4;14707.1)	487.8 (371;633.6)	4.6(0;10.2)
Espírito Santo	6046.9 (4508.7;7782.4)	477 (360.7;609.5)	20591.3 (15508.9;26528)	494.1 (373.4;637)	3.6(-1.8;9)
Goiás	8112.6 (6103;10537.3)	473.8 (358.8;618.5)	31759.2 (24091.6;40630.4)	488.7 (373.2;628)	3.1(-2;8.8)
Maranhão	10154.6 (7606.6;13258.2)	445.1 (337.1;576.8)	29373.5 (22463.1;37530)	464.8 (354.9;593)	4.4(-0.6;9.4)
Mato Grosso	2967.4 (2238;3853.5)	481.9 (367.5;627)	14898.3 (11378.3;19217.4)	495.9 (376.2;641.8)	2.9(-2.8;7.8)
Mato Grosso do Sul	3568 (2677.8;4589.9)	484.9 (370.2;623.5)	13928.3 (10490.8;17874.3)	500.6 (375.5;643.5)	3.2(-1.8;7.8)
Minas Gerais	71781.4 (53528.5;93821.9)	831.3 (627.8;1079.1)	223364.2 (168276.2;291716.3)	849.1 (640.5;1103.7)	2.1(-2.2;6.6)
Pará	8234.5 (6281.7;10593.5)	464.3 (354.2;598.1)	31143.2 (23903;39996.1)	485.2 (370.9;626)	4.5(-0.6;9.4)
Paraíba	10701.6 (8052.8;13746.5)	480.4 (363.4;614.6)	23515.3 (17936.4;30396.6)	489.9 (372.3;633.8)	2(-3.8;7.4)
Paraná	19787.5 (14787;25568.1)	487.8 (370.8;626.5)	64224.8 (48888.4;83035.5)	502.7 (383.3;651.8)	3(-1.4;8.1)
Pernambuco	19150.2 (14364.7;24937.2)	463.9 (351.7;600.9)	46115.3 (35058;59389.5)	480.9 (366.5;621.5)	3.7(-1.2;9.9)
Piauí	5728.6 (4318.8;7395.8)	463.3 (353.4;598.2)	18024 (13731.2;23287.2)	479.1 (364.2;620.3)	3.4(-1.1;8.2)
Rio de Janeiro	39769.7 (29930.9;51462.6)	475.4 (358.3;613.4)	109091.6 (82434.2;141021.5)	492 (373.7;634.7)	3.5(-0.9;8.6)
Rio Grande do Norte	7220.3 (5485.8;9275.2)	473.5 (362.3;608.7)	18814.8 (14363.2;24105.1)	489.4 (373.6;630.9)	3.4(-1.1;7.9)
Rio Grande do Sul	27957.6 (20979.2;36240.9)	486.8 (370.7;628.7)	78012.2 (58643;101476.3)	499.6 (376.5;646.6)	2.6(-1.9;7.8)
Rondônia	1196.4 (871.8;1563.9)	477.4 (358.7;611)	6770.9 (5121.3;8697.4)	487.5 (367.9;627)	2.1(-2.6;7)
Roraima	201.9 (151.7;260.8)	476.4 (364.9;615.4)	1589.6 (1203.7;2062.2)	495.4 (377.6;632.2)	4(-0.2;8.6)
Santa Catarina	10427.4 (7849.1;13416.7)	489.4 (370.8;623.4)	38974 (29337.7;50470.5)	504.9 (384.6;648.6)	3.2(-2;8.2)
São Paulo	84287.7 (63124.6;108978.1)	495.8 (379.2;636.4)	269601.2 (204582.8;347991.5)	511.2 (388.9;664)	3.1(-2.8;8.2)
Sergipe	3387.4 (2523.5;4411.4)	480.9 (364.7;627.1)	10672.7 (8098.7;13831.9)	502 (380.7;652.5)	4.4(0.3;9.3)
Tocantins	1510.8 (1130;1956.4)	452.5 (345.4;588)	6381 (4850.7;8197.6)	476.1 (363.6;612.1)	5.2(0.3;10.9)
Brazil	401706.1 (302349.2;518702.6)	519.4 (393;668.6)	1226853.4 (934018.3;1577999.6)	537.3 (409.2;692.5)	3.5(1.8;5.1)

*Source: Data derived from Global Burden of Disease Study 2019, Institute for Health Metrics and Evaluation, University of Washington. ^46^*

**Table 6-3:** – Number of deaths and age-standardized mortality rates (per 100 000 inhabitants) due to atrial fibrillation and flutter in 1990 and 2019, with percent change of rates, according to sex and age group, in Brazil

Sex and Age	1990		2019		Percent change
	Number (95% UI)	Rate (95% UI)	Number (95% UI)	Rate (95% UI)	(95% UI)
**Both**					
15-49 years	57.3 (48.3;76.1)	0.1 (0.1;0.1)	118.1 (91.1;138.6)	0.1 (0.1;0.1)	36.7 (7.7;52.4)
50-69 years	432.5 (370.3;566.3)	2.8 (2.4;3.6)	1125 (918.5;1355.3)	2.8 (2.3;3.4)	1.1 (-16;10.3)
70+ years	2169.7 (1822.6;2747)	51.3 (43.1;64.9)	9568.3 (7433.7;11342.6)	73.1 (56.8;86.7)	42.5 (20.2;54.1)
Age-standardized	2659.5 (2263.6;3342.6)	4.8 (4;6)	10811.4 (8636.5;12800.8)	5 (4;6)	5.4 (-10.6;13.1)
All Ages	2659.5 (2263.6;3342.6)	1.8 (1.5;2.2)	10811.4 (8636.5;12800.8)	5 (4;5.9)	179.3 (133.7;202.2)
**Female**					
15-49 years	25.6 (23.2;35.2)	0.1 (0.1;0.1)	55.5 (43.6;63.2)	0.1 (0.1;0.1)	44.6 (10.2;65.6)
50-69 years	202.6 (183.8;264.5)	2.5 (2.3;3.2)	550 (447;619.2)	2.6 (2.1;2.9)	3.5 (-15.5;15.7)
70+ years	1278.4 (1060.8;1636.8)	54.5 (45.2;69.8)	6122.4 (4570;7074.9)	81.1 (60.5;93.7)	48.7 (21.8;65.3)
Age-standardized	1506.6 (1266.7;1935.4)	4.8 (4;6.2)	6727.9 (5082;7750.1)	5.2 (3.9;6)	7.6 (-11.2;19)
All Ages	1506.6 (1266.7;1935.4)	2 (1.7;2.6)	6727.9 (5082;7750.1)	6.1 (4.6;7)	203.2 (148.8;236.1)
**Male**					
15-49 years	31.8 (23.1;47.1)	0.1 (0.1;0.1)	62.6 (39.9;81.3)	0.1 (0.1;0.1)	30.3 (3.6;51.7)
50-69 years	229.9 (168.9;336.6)	3.1 (2.2;4.5)	575 (392.4;780.2)	3 (2.1;4.1)	-0.6 (-17.5;11.1)
70+ years	891.3 (645;1258.4)	47.3 (34.2;66.8)	3445.9 (2286;4594.7)	62.2 (41.3;83)	31.6 (12.7;45.6)
Age-standardized	1153 (843.8;1633.3)	4.7 (3.4;6.6)	4083.4 (2781.1;5478.5)	4.8 (3.2;6.4)	1.2 (-13;11)
All Ages	1153 (843.8;1633.3)	1.6 (1.1;2.2)	4083.4 (2781.1;5478.5)	3.9 (2.6;5.2)	146.3 (109.6;172.8)

*Source: Data derived from Global Burden of Disease Study 2019, Institute for Health Metrics and Evaluation, University of Washington.* ^46^

**Table 6-4:** – Number of deaths and age-standardized mortality rate (per 100 000 inhabitants) due to atrial fibrillation and flutter in 1990 and 2019, with percent change of rates, in Brazil and Brazilian Federative Units

Location	1990		2019		Percent change
	Number (95% UI)	Rate (95% UI)	Number (95% UI)	Rate (95% UI)	(95% UI)
Acre	4 (3.2;4.7)	5.1 (4;6.1)	24.7 (19.2;28.8)	5.7 (4.4;6.6)	10.7(-5.8;25.5)
Alagoas	37.4 (30.3;46.3)	4 (3.3;5)	137.2 (106.1;169.5)	4.6 (3.5;5.7)	14.7(-3.6;34.2)
Amapá	2.9 (2.2;3.3)	5.2 (4;6)	19.8 (14.1;22.9)	5.4 (3.9;6.3)	3.5(-8.1;13.7)
Amazonas	19.3 (15.9;23.8)	4.8 (3.9;5.9)	95.7 (72.8;119.1)	4 (3.1;5)	-15.9(-26.3;-5.6)
Bahia	219.2 (166.6;261)	4.3 (3.2;5.1)	801.9 (552.5;974.5)	4.8 (3.3;5.8)	11.8(-8.3;33.5)
Ceará	121.8 (89.5;156.9)	3.4 (2.5;4.4)	489.1 (375.1;592.4)	5 (3.9;6.1)	48.5(14.2;83.8)
Distrito Federal	14.3 (12.2;20)	9.2 (7.3;12.5)	104.6 (84;133.8)	8.8 (7;11.2)	-3.8(-22.1;14.1)
Espírito Santo	39.3 (33.4;50.9)	4.9 (4.1;6.5)	193.5 (154.7;236)	5.2 (4.1;6.3)	5(-10.7;17.7)
Goiás	56.1 (47.5;77.4)	5.5 (4.6;7.6)	265.9 (209.7;336.1)	4.8 (3.7;6)	-13.8(-30.5;1.2)
Maranhão	77.7 (42.2;105.3)	4.4 (2.4;6)	445.9 (240.1;554)	7.2 (3.9;9)	65.4(29.7;118)
Mato Grosso	17.7 (14.5;22.4)	4.6 (3.7;5.7)	108.3 (85.9;137.8)	4.3 (3.3;5.4)	-6.7(-18.3;6.1)
Mato Grosso do Sul	23.7 (20.1;30.2)	5.3 (4.3;6.6)	117.1 (94.2;146.4)	4.8 (3.8;6)	-9(-18.7;1)
Minas Gerais	290.7 (249.5;374.6)	5.3 (4.4;6.7)	1193.3 (889.8;1407.2)	4.6 (3.4;5.4)	-13.2(-35.8;-0.4)
Pará	63.2 (48.7;73.1)	5.5 (4.1;6.3)	267 (195.7;317.4)	4.4 (3.2;5.2)	-19.8(-30.7;-7.9)
Paraíba	74.9 (57.3;92.5)	3.8 (2.9;4.7)	221.3 (167.4;273.4)	4.1 (3.2;5.1)	9.5(-10.2;27.9)
Paraná	126.5 (108.6;178.9)	5 (4.2;7.1)	541.3 (444.9;678.3)	4.9 (4;6.1)	-1.6(-18.3;11.1)
Pernambuco	133.9 (113.2;175.8)	4.4 (3.6;5.9)	438.3 (357;561.5)	4.9 (4;6.3)	11.3(-3;25.9)
Piauí	37.9 (29.3;46.4)	4.6 (3.4;5.7)	161.3 (116.8;193.7)	4 (2.9;4.8)	-12.7(-27.6;1.3)
Rio de Janeiro	297.6 (256.7;435.5)	5.2 (4.5;7.6)	1074.4 (853.7;1362.3)	5.2 (4.1;6.6)	-0.7(-21.7;14)
Rio Grande do Norte	54.5 (41.3;66)	4 (3;4.8)	182.9 (136.5;224)	4.3 (3.2;5.3)	8.9(-13.2;29.6)
Rio Grande do Sul	189.9 (162.8;272.6)	4.7 (4;6.7)	733.7 (590.2;905.4)	4.9 (3.9;6.1)	5.3(-17.5;19.9)
Rondônia	5.3 (4.3;7.1)	6.9 (5.4;9)	55.1 (44.3;70.3)	4.7 (3.7;6.1)	-31.6(-42.5;-17)
Roraima	1.2 (0.9;1.5)	7.2 (5.6;8.6)	13.2 (10.5;15.4)	7 (5.6;8.2)	-3(-13.9;8.4)
Santa Catarina	74.8 (64.6;101.5)	5.3 (4.5;7.2)	333.7 (265.1;409.5)	5.2 (4.1;6.4)	-1.4(-19.6;11.7)
São Paulo	642.9 (547.6;820.7)	5.9 (4.9;7.4)	2630.3 (1986.8;3101.9)	5.6 (4.2;6.7)	-3.6(-21.8;9)
Sergipe	24.2 (20.1;30.2)	5.1 (4.3;6.4)	92 (70.5;113.9)	4.4 (3.4;5.5)	-14.2(-27.6;-0.9)
Tocantins	8.7 (6.5;10.6)	6 (4.6;7.3)	69.9 (51.4;84.5)	5.7 (4.2;6.9)	-4.6(-21.2;13.3)
Brazil	2659.5 (2263.6;3342.6)	4.8 (4;6)	10811.4 (8636.5;12800.8)	5 (4;6)	5.4(-10.6;13.1)

*Source: Data derived from Global Burden of Disease Study 2019, Institute for Health Metrics and Evaluation, University of Washington. ^46^*

**Table 6-5:** – Number of DALYs and age-standardized DALY rates (per 100 000 inhabitants) due to atrial fibrillation and flutter in 1990 and 2019, with percent change of rates, according to sex and age group, in Brazil

Sex and Age	1990		2019		Percent change
	Number (95% UI)	Rate (95% UI)	Number (95% UI)	Rate (95% UI)	(95% UI)
**Both**					
15-49 years	4251 (3336.7;5517.3)	5.5 (4.4;7.2)	8855.5 (6876;11253.8)	7.7 (6;9.7)	38.3 (16.9;51.2)
50-69 years	25126.7 (19600.3;32757.4)	160.2 (124.9;208.8)	66612.8 (51964.2;86102.4)	165.1 (128.8;213.4)	3.1 (-6.2;7.9)
70+ years	42042.9 (34764.9;51839.4)	993.9 (821.9;1225.5)	154647.9 (126905.8;188279.2)	1181.6 (969.6;1438.5)	18.9 (7.2;25.7)
Age-standardized	71420.6 (58907.9;88622.2)	98.7 (81.8;121.3)	230116.3 (189167;279885.9)	102.5 (84.3;124.5)	3.9 (-6.4;8.7)
All Ages	71420.6 (58907.9;88622.2)	48 (39.6;59.5)	230116.3 (189167;279885.9)	106.2 (87.3;129.2)	121.3 (99.5;133.3)
**Female**					
15-49 years	1789.8 (1436.2;2324.2)	4.6 (3.7;6)	3878.6 (2980.7;4931.9)	6.6 (5.1;8.4)	44.3 (19.3;62.4)
50-69 years	11044.7 (8663.5;14294.3)	135.3 (106.1;175.1)	30304.9 (23538.7;39110.9)	141.5 (109.9;182.6)	4.6 (-5.3;11.8)
70+ years	22605.8 (18670.3;27843.4)	964 (796.2;1187.3)	89454.8 (70991.4;107212.8)	1184.7 (940.2;1419.9)	22.9 (8;32.8)
Age-standardized	35440.3 (29438.2;43933.5)	91 (75.8;111.6)	123638.3 (100891.8;148053.7)	95.8 (78;114.7)	5.3 (-7.3;12.5)
All Ages	35440.3 (29438.2;43933.5)	47.1 (39.1;58.4)	123638.3 (100891.8;148053.7)	111.5 (91;133.5)	136.9 (109.4;155.1)
**Male**					
15-49 years	2461.2 (1840.7;3237)	6.5 (4.9;8.6)	4976.9 (3658.8;6398.8)	8.7 (6.4;11.2)	33.8 (13.8;50.7)
50-69 years	14082 (10693.3;18706.2)	187.1 (142.1;248.6)	36307.9 (27210.5;47858.4)	191.8 (143.8;252.8)	2.5 (-7.3;8.9)
70+ years	19437.2 (15002.9;24958.6)	1031.1 (795.9;1324.1)	65193.2 (50511.1;82124.3)	1177.2 (912.1;1483)	14.2 (4;21.4)
Age-standardized	35980.3 (28333.3;46350.5)	107.5 (85;136.9)	106478 (82477.5;133352.6)	110.2 (85.5;137.8)	2.6 (-6.4;8.3)
All Ages	35980.3 (28333.3;46350.5)	48.9 (38.5;63)	106478 (82477.5;133352.6)	100.6 (78;126)	105.8 (87;117.7)

*Source: Data derived from Global Burden of Disease Study 2019, Institute for Health Metrics and Evaluation, University of Washington.^*46*^*

**Chart 6-1:** – All ages and age-standardized rates of atrial fibrillation/flutter between 1990 and 2019, in Brazil. A. Prevalence, B. Incidence, C. Deaths, D. YLLs, D. DALYs, E. YLDs. ^46^

**Chart 6-2:** – Correlation of the 2019 Sociodemographic Index (SDI) and the percent change in age-standardized mortality rates due to atrial fibrillation and flutter per 100 000 inhabitants from 1990 to 2019

Source: Data derived from Global Burden of Disease Study 2019, Institute for Health Metrics and Evaluation, University of Washington. ^46^

**Chart 6-3:** – Deaths (per 100 000) due to atrial fibrillation and flutter attributable to risk factors in Brazil in 1990 and 2019, and percent change, for both sexes, males, and females.

Source: Data derived from Global Burden of Disease Study 2019, Institute for Health Metrics and Evaluation, University of Washington. ^46^

### Prevalence and Incidence

• According to the GBD Study 2019 estimates, the age-standardized prevalence rates due to AF and atrial flutter increased slightly in Brazil from 519 (95% UI, 393-669) in 1990 to 537 (95% UI, 409-692) in 2019, per 100 000 inhabitants, for both sexes, with 3.5% (95% UI, 1.8-5.1) change in this period. The prevalence of AF and atrial flutter was higher in men [in 1990, 619 (95% UI, 468-792); in 2019, 643 (95% UI, 489-829)] than in women [in 1990, 499 (95% UI, 418-587); in 2019, 522 (95% UI, 440-610)], although the percent change was similar for both sexes (women, 4.0%; 95% UI, 1.9-6.5; and men, 4.0%; 95% UI, 1.9-6.0) in this period. In absolute numbers, estimates for the prevalence of AF and atrial flutter in Brazil rose from 0.4 million in 1990 to 1.2 million in 2019, mainly due to population growth and aging (Table 6-1 and Chart 6-1), as suggested by growing crude prevalence rates. In 2019, the proportion of subjects with prevalent AF was 0.59% (95% UI, 0.45-0.76). Stratifying by Brazilian FUs, the prevalence rate of AF and atrial flutter is similar in most Brazilian FUs, except for Minas Gerais, whose rate is nearly twice that of other states (Table 6-2). Importantly, Minas Gerais is the only state for which the Institute for Health Metrics and Evaluation has primary data, what may explain this difference and suggest that the AF prevalence may be even higher in Brazil than that estimated by the GBD. ^46^

• Regarding the age-standardized incidence rate, per 100 000, per-year, the GBD Study 2019 estimates were 44 (95% UI, 33-56) in 1990 and 45 (95% UI, 34-58) in 2019, also higher for men in both time periods [women, 1990: 37 (95% UI, 28-48) and 2017: 39 (95% UI, 29-50); men: 1990: 50 (95% UI, 38-64) and 2017: 52 (95% UI, 39-67)].

• Data from population-based studies showed prevalence varying from 0.3% to 2.4%. In the ELSA-Brasil cohort study, which included 14 424 adults with valid ECG (45.8% men; age range, 35-74 years), the AF and atrial flutter prevalence was 0.3% (men, 0.5%; women, 0.2%), with the highest prevalence in the age range of 65-74 years (women: OR, 17; 95% CI, 2.1-135.9; men: OR, 52.3; 95% CI, 3.1-881.8). There was no difference in prevalence according to self-reported race, for both sexes. ^230^ In a cross-sectional study with 1524 elderly in São Paulo, the prevalence of AF or atrial flutter was 2.4% (men 3.9%; women, 2.0%). ^231^

• Data from telehealth centers across Brazil have provided information on the prevalence of AF and atrial flutter based on ECG from primary care. ^232^ In the ECG dataset of the Telehealth Network of Minas Gerais including 1 558 421 individuals (mean age, 51±18 years; 40.2% men) with ECG performed between 2010 and 2017, the AF prevalence was 1.33%, higher in men (1.81% vs. 1.02%), and increasing with age (OR 1.08, CI 95% 1.08-1.08), reaching 7.0% in octogenarians (8.4% in men vs. 5.9% in women). ^236^

• Data from 676 621 ECG (mean age, 51±19 years; 57.5% women) performed from 2009 to 2016 were analyzed at the Federal University of São Paulo telemedicine center and revealed a 7-year AF prevalence of 2.2% and a countrywide 2025 projected AF prevalence of 1.7%. ^234^

• The GARFIELD-AF is a hospital-based registry that included patients (≥18 years) with AF diagnosed within the previous 6 weeks and at least one additional risk factor for stroke. In Brazil, 41 sites included 1065 patients with non-valvular AF between 2010 and 2014 (mean age, 68±13 years; 55% males). New-onset AF diagnosed within the previous 6 weeks was recorded in 52% of the patients, paroxysmal in 25%, persistent in 14%, and permanent in 8%. ^237^

### Mortality

• In the GBD Study 2019, the number of deaths from AF in Brazil has increased over the past few years, due to population growth and aging. In the 1990s, AF was responsible for 2659 (95% UI, 2263-3342) deaths, which rose to 10 811 (95% UI, 8636-12801) in 2019. However, the age-standardized mortality rate due to AF remained stable in the period, 4.8 (95% UI, 4.0-6.0) deaths per 100 000 inhabitants in 1990 and 5.0 (95% UI, 4.0-6.0) per 100 000 inhabitants in 2019, accounting for 0.8% (95% UI, 0.6-0.9) of all deaths in the country. Although age-standardized prevalence rates were consistently higher in men, women had a higher age-standardized mortality rate in 2019 [(women, 5.2 (95% UI, 3.9-6.0); men, 4.8 (95% UI, 3.2-6.4)], consistent with data from other countries. ^238^ ^,^ ^239^ For individuals > 70 years old, the mortality rate increased from 1990 (51, 95% UI, 43-65) to 2019 (73, 95% UI, 57-87) (Table 6-3). Of note, because mortality based on vital registration data alone provides an implausibly steep increase over time possibly due to changes in ascertainment rather than AF epidemiology, the GBD Study 2019 assumes, a priori, that age and sex-specific mortality rates are neither increasing nor decreasing over time. ^4^ As such, the small changes over time herein reported are intentionally lower than the real changes in raw data.

• Chart 6-1 shows that, although age-standardized mortality rates are stable, crude mortality rates are rising because of population aging and the growing number of individuals living longer with chronic heart diseases. Moreover, the YLL, a metric used in GBD for premature death, is also rising when not standardized to age.

• Table 6-4 shows the total number of deaths and the age-standardized mortality rates (per 100 000 inhabitants) due to AF and flutter, in 1990 and 2019, and percent change, for both sexes, in Brazil and its FUs. The FU with the highest mortality rates in 2019 were Distrito Federal, Maranhão, and Roraima, while those with the lowest were Piauí, Amazonas, and Paraíba. However, due to reporting issues, these data can be inconsistent and should be interpreted with caution.

• The GBD Study 2019 uses the SDI as an estimate of the socioeconomic level of a location. As demonstrated in Chart 6-2, there was a modest association of higher SDI in 2019 and greater percent change in AF age-standardized mortality rate from 1990 to 2019 (r=-0.44, p=0.02), revealing that the most developed FUs had a greater reduction in mortality, possibly due to health investments and a greater reduction in the cardiovascular disease burden as a whole.

• From a dataset of 1 558 421 ECGs from primary care patients (Telehealth Network of Minas Gerais) linked to the Brazilian Mortality Information System, the overall mortality rate was 3.34% in a mean follow-up of 3.68 years. After adjusting for age and comorbidities, patients living with AF had a higher risk of overall (HR 2.10; 95% CI, 2.03–2.17) and cardiovascular (HR 2.06; 95% CI, 1.86–2.29) death, with increased risk in women, who lost their survival advantage over men when AF was present. ^236^

• In a 10-year follow-up of 1462 individuals aged ≥ 60 years (mean age, 69 years; 61% women) included in 1997 in the Bambuí Cohort Study, AF or flutter was independently associated with an increase in all-cause mortality (HR, 2.35; 95% CI, 1.53-3.62) among patients with and without ChD (HR,1.92; 95% CI, 1.05-3.51). ^240^

• In the GARFIELD-AF, a worldwide hospital-based study that included 1061 patients in Brazil (≥18 years) who had AF diagnosed within the previous 6 weeks and at least one additional risk factor for stroke, the all-cause mortality rate per 100-person-years was 6.19 (CI 95%, 4.83-7.94), and 38.7% were cardiovascular deaths. ^237^

### Burden of Disease

• According to GBD 2019 estimates, AF resulted in 230 116 (95% UI, 189 167 – 279 885) DALYs in Brazil in 2019, representing 0.35% of all DALYs. The age-standardized DALY rate was 103 (95% UI, 84-125) per 100 000 in 2019, greater for men (110; 95% UI 86-138) than for women (96; 95% UI, 78-115), although the proportion of DALYs is higher for women (0.42%; 95% UI, 0.35-0.48) than for men (0.30%; 95% UI, 0.24-0.37) (Table 6-5).

• Chart 6-1 and Table 6-4 reveal the same pattern for mortality rates: while the age-standardized mortality rate remained stable from 1990 to 2019, the crude rate increased.

### Complications

#### Stroke

• Of all 429 cases of stroke (87.2% ischemic strokes) that occurred in the city of Joinville in 2015 and were included in a registry, AF was detected in 11.4% of all patients and in 58% of the cardioembolic strokes. ^241^ Similarly, AF was detected in 58% of 359 patients with cardioembolic stroke from a one-center, consecutive sample in the city of Curitiba, Brazil. ^242^

• Age (OR=1.04; 95% CI, 1.02-1.08), National Institutes of Health Stroke Scores on admission (OR=1.10; 95% CI, 1.05-1.16), and the presence of left atrial enlargement (OR=2.5; 95% CI, 1.01-6.29) were predictors of AF (C-statistic, 0.76; 95% CI, 0.69-0.83) among patients hospitalized for acute ischemic stroke or TIA in a Brazilian cohort. ^243^

• In a cohort of 1121 ischemic stroke patients in a 12-year follow-up, AF was independently associated with increased overall (HR 1.82; 95% CI, 1.43-2.31) and cardiovascular (HR 2.07; 95% CI, 1.36-3.14) mortality. ^244^

#### Dementia

• In a cross-sectional study with 1524 participants aged >65 years, dementia was diagnosed in 11% of those with AF *versus* 4% among those without AF (p=0.07); the authors found an odds ratio of dementia of 2.8 (95% CI, 1.0-8.1; p = 0.06) among subjects with AF. ^245^

#### Association of Risk Factors for AF/Flutter

• According to the GBD Study 2019, AF deaths were attributed to six risk factors in 1990 and 2019: high blood pressure, high body mass index, dietary risks, alcohol use, tobacco, and other environmental risks. For both sexes, high systolic blood pressure was the most important risk factor for death due to AF, accounting for 35.8% (95% UI, 29.7-42.2) of deaths for men and 34.4% (95% UI, 27.5-41.7) for women in 2019. High body mass index ranked 2 for both sexes and was responsible for 23.9% of AF deaths (95% UI, 12-38) in men and 28.8% (95% UI, 16.7-43.2%) in women, in 2019. Importantly, while the risk attributed to high blood pressure increased slightly from 1990 to 2019 (7.1% change for men and 10.7% for women), the AF deaths attributable to high body mass index are increasing steeply (74.4% change for men and 79.5% for women). The risk for death from AF attributable to alcohol use is also rising, particularly for women (Chart 6-3).

• Data from the Telehealth Network of Minas Gerais with ECGs of 1 558 421 individuals (mean age, 51±18 years; 40.2% men) performed between 2010 and 2017 revealed in multivariable models adjusted for age and sex that the following self-reported comorbidities related to the presence of AF: ChD (OR 3.08; 95% CI, 2.91-3.25), previous myocardial infarction (OR 1.74; 95% CI, 1.56-1.93), chronic obstructive pulmonary disease (OR 1.48; 95% CI, 1.33-1.66), hypertension (OR 1.31; 95% CI, 1.27-1.34), dyslipidemia (OR 1.09; 95% CI, 1.03-1.16). Current smoking and diabetes were not associated with prevalent AF. ^236^

• A cross-sectional study comparing AF subjects with healthy controls found a higher frequency of sleep apnea in the AF group than in the control group (81.6% *versus* 60%, p = 0.03). ^246^

## Associated Comorbidities

### Atrial Fibrillation and Other Heart Diseases

• The incidence of AF among 300 elderly patients (mean age, 75±8 years; 56% women) monitored with pacemakers, free from AF at baseline, was 22% in a 435-day follow-up ^247^ and reached 85% of the patients with pacemakers and chronic kidney disease in a 1-year follow-up. ^248^

• On echocardiogram, AF was associated with heart disease (OR = 3.9; 95% CI, 2.1 - 7.2, p <0.001) in 1518 patients (mean age, 58±16 years; 66% female) from a waiting list for echocardiogram in primary care, who were also screened for AF with a portable device (AF prevalence of 6.4%). The authors suggest that AF screening could be a useful primary care tool to stratify risk and prioritize echocardiography. ^228^

• Heart failure and AF coexist in many patients as they share similar pathophysiological pathways. In a retrospective study of 659 patients hospitalized for decompensated HF in 2011, the AF prevalence was 40% (73% permanent AF), and AF was associated with increasing age (p < 0.0001), non-ischemic etiology (p = 0.02), right ventricular dysfunction (p = 0.03), lower systolic blood pressure (p = 0.02), higher ejection fraction (p < 0.0001), and enlarged left atrium (p < 0.0001). Patients with AF had longer hospital length of stay (20.5 ± 16 days *versus* 16.3 ± 12, p = 0.001). ^249^

• Of the patients with cardiovascular disease visiting the emergency department, the prevalence of AF was 40% among patients with decompensated HF ^22^ and 44% among those with valvular heart disease. ^182^

• A study including critically ill patients found an AF incidence of 11% during their intensive care unit stay. ^250^

## Perioperative Atrial Fibrillation and Cardiovascular Surgery

• Of patients undergoing cardiac surgery, 12% to 33% had AF in the postoperative period. ^204^ ^,^ ^251^ ^,^ ^252^ Surgeries for valve replacement were associated with a higher occurrence of AF (31-33%) as compared to CABG (12-16%) during hospitalization.

• Advanced age, mitral valve disease, and no beta-blocker use were associated with postoperative AF in valvular surgery. ^205^ Among those who underwent CABG, the postoperative AF incidence was associated with left atrial > 40.5mm and age > 64.5 years. ^253^

## Atrial Fibrillation and Chagas Disease

• AF has been consistently associated with ChD and increases the risk of death in ChD patients. ^254^ In the Bambuí Cohort Study, 1462 participants aged ≥ 60 years (mean age, 69 years; ChD n=557, 38.1%), with baseline ECG, were followed up for 10 years. AF was more frequently observed in ChD subjects [6.1% vs 3.4% (OR: 3.43; 95% CI, 1.87-6.32, adjusted for age, sex, and clinical variables)], in whom it was an independent risk factor for death (HR: 2.35; 95% CI, 1.53-3.62 adjusted for age, sex, clinical variables and BNP levels). ^240^

• In a large sample of 264 324 patients undergoing tele-ECG in primary health care units in 2011, ChD was self-reported by 7590 (2.9%). The mean age of ChD subjects was 57.0 ± 13.7 years, while that of non-ChD subjects was 50.4 ± 19.1 years, with 5% of octogenarians in both groups. AF was observed in 5.35% of the ChD subjects and in 1.65% of non-ChD ones (OR: 3.15; 95% CI, 2.83-3.51, adjusted for age, sex, and self-reported comorbidities). ^254^

• Rojas *et al* . evaluated, in a systematic review and meta-analysis, the frequency of electrocardiographic abnormalities in ChD in the general population. Forty-nine studies were selected, including 34 023 individuals (12 276 ChD and 21 747 non-ChD). The AF prevalence was significantly higher in ChD patients (OR: 2.11; 95% CI, 1.40-3.19). ^256^

• In a sample of 424 ChD patients under the age of 70 years (41.7% female; mean age, 47±11 years), followed up for 7.9 ± 3.2 years, Rassi *et al* . found an AF prevalence of 13.3±3.1% and a strong association with the risk of death [HR: 5.43 (2.91–10.13)] on univariate analysis. ^257^

## Health Care Utilization and Cost

**(Refer to Tables 1-6 through 1-9 and Charts 1-15 and 1-16)**

• From 2008 to 2019, there were 354 619 hospitalizations for AF, and 1413 ablation procedures for AF and atrial flutter were performed by the SUS, with unadjusted costs of R$ 260 593 600 and R$ 7 912 561, respectively. After adjusting for Brazilian inflation, the costs were R$ 451 530 532 and R$ 13 710 094, respectively, and, in international dollars, converted to PPP-adjusted to US$ 2019, $ 169 076 584 and $ 5 047 822, respectively.

• An analysis of the economic burden of heart conditions in Brazil estimated an AF prevalence of 0.8% (n=1 202 151 cases) in 2015. The authors estimated a total cost for AF of R$ 3.921 billion (US$ 1.2 billion), 94% due to direct healthcare costs. ^124^

• A study on data from a private outpatient clinic regarding anticoagulation analyzed the annual cost per AF patient (n=1220; mean age, 64 years) and found that 64% of all costs (US$ 10 679) during a 1.5-year follow-up were attributed to inpatient costs. ^258^

## Awareness, Treatment, and Control

### Anticoagulation

• There was a high variation in the use of anticoagulation in patients with AF, from 1.5% to 91%. Studies with samples from primary care were more likely to have low anticoagulation use as compared to samples recruited from tertiary centers or cardiologists, as detailed below.

• Of the 4638 subjects with AF in primary care centers of 658 municipalities of the Minas Gerais state (mean age, 70±14 years; 54% men), who underwent ECG using telehealth in 2011, VKA use was reported by 1.5% and aspirin, by 3.1%. ^233^

• Of 125 primary care centers from nine states in four Brazilian regions, from January 2009 to April 2016, a subset of 301 patients had AF, 189 (63%) of whom were at high risk for stroke; only 28 (15%) were regular oral anticoagulant users, and 102 (54%) were on aspirin. ^234^

• In the GARFIELD-AF registry, of the 1061 patients included (82.3% by cardiologists) in Brazil between 2010 and 2014 (mean age, 68±13 years; 55% males), 86% had CHA_2_DS_2_-VASc score ≥ 2, 19% were not using anticoagulation therapy at baseline, 26% were only receiving antiplatelet therapy, 29% were using VKA, and 26% were receiving NOAC. ^237^

• The IMPACT-AF, ^259^ a clustered randomized trial to improve treatment with anticoagulants in patients with AF conducted in Argentina, Brazil, China, India, and Romania, showed that two-thirds of the patients were on oral anticoagulation at baseline: 83% were on a VKA and 15% were on NOAC. The patients from Brazil (n=360) were most often on oral anticoagulation at baseline (91%) and 27% were on NOAC. Of all patients taking VKA in Brazil, 40.3% had INR values between 2 and 3 prior to the baseline visit.

• A stroke registry in the city of Joinville described all 429 cases of stroke that occurred in 2015, and AF was detected in 49 (11.4%) patients. Of the 26 patients with known prior AF, 19 (73%) were not anticoagulated, 20 (77%) had a CHA_2_DS_2_-VASc score ≥ 3, and 21 (81%) had a HAS-BLED score < 3. ^241^

• In a cohort of 1121 ischemic stroke patients, 200 of whom had AF, anticoagulation for AF was inversely associated with all-cause mortality (oral anticoagulant time-dependent effect: multivariable HR, 0.47; 95% CI, 0.30–0.50) and stroke mortality (oral anticoagulant time-dependent effect ≥ 6 months: multivariable OR, 0.09; 95% CI, 0.01–0.65), but not with cardiovascular mortality. ^244^

• The quality of warfarin therapy has been evaluated using the parameter TTR in different samples in Brazil. The TTR of anticoagulation for AF has ranged from 31% to 67% in the studies. ^258^ ^,^ ^260^ Anticoagulation Therapy in Patients with Non-valvular Atrial Fibrillation in a Private Setting in Brazil: A Real-World Study ^258^ ^,^ ^260^ Age >65 years, but not health literacy, was associated with a higher TTR value. ^262^ In a retrospective analysis of 1220 patients from the private setting, those with low TTR had more severe bleeding and 40% higher health costs in a median 1.5-year follow-up. ^258^

### Rhythm or Rate Control (Medication, Cardioversion, Catheter Ablation)

• A cross-sectional study with 167 AF patients found that rate control was more common than rhythm control as treatment strategy (79% vs. 21%; p < 0.001). Among those in rhythm control, amiodarone (43%), sotalol (16%), and propafenone (14%) were the most prescribed drugs. Beta-blockers were prescribed in 81% of the patients on rate control. ^264^ Amiodarone was mentioned by 83% of doctors as the choice for the rhythm control strategy. ^265^

• Data from 125 primary care centers showed that of 301 patients with AF, 91 (30.2%) were receiving neither rate nor rhythm control therapy. Of the remaining 210 patients undergoing treatment, 147 (70%) used rate control agents (beta-blockers, digoxin, diltiazem, or verapamil) and 25 (12%) used at least one antiarrhythmic drug (amiodarone or propafenone). The simultaneous use of antiarrhythmic drugs and beta-blockers was reported by 36 (17%). ^234^

## Future Research

• Ongoing cohort studies have the potential to fill information gaps on incidence, risk factors, risk prediction, and prevention of AF in Brazil. To our knowledge, there is no original published study with information on the AF incidence in Brazil or longitudinal data on risk factors.

• Studies designed to screen AF in population-based or selected populations using ECG or screening devices are ongoing and should bring information on the relevance of including this strategy in primary care or specialized centers.

• The First Brazilian Cardiovascular Registry of Atrial Fibrillation, the RECALL study, finished the inclusion of 4584 patients in 2019 and its results are awaited. It will be the largest Brazilian registry regarding AF patient characteristics and treatment from 73 centers from all Brazilian geographic regions. ^266^

• Implementation strategies to enhance anticoagulation use among AF patients should be encouraged, particularly in primary care settings.

• Studies using artificial intelligence to diagnose or predict AF may be a tool to improve AF diagnosis and personalize screening strategies.

## 7. HYPERTENSION

### ICD-10 - I10

**See Tables 7-1 through 7-6 and Charts 7-1 through 7-7**

**Table:** Abbreviations Used in Chapter 7

CI	Confidence Interval
CVD	Cardiovascular Disease
DALYs	Disability-Adjusted Life Years
DBP	Diastolic Blood Pressure
ELSA	English Longitudinal Study of Ageing
ELSA-Brasil	Brazilian Longitudinal Study of Adult Health (in Portuguese *, Estudo Longitudinal de Saúde do Adulto* )
ERICA	Brazilian Study of Cardiovascular Risk in Adolescents (in Portuguese, *Estudo dos Riscos Cardiovasculares em Adolescentes* )
FU	Federative Unit
GBD	Global Burden of Disease
HIPERDIA	SUS’ Program for Arterial Hypertension and Diabetes
HR	Hazard Ratio
HRQOL	Health-Related Quality of Life
IBGE	Brazilian Institute of Geography and Statistics (in Portuguese, *Instituto Brasileiro de Geografia e Estatística* )
ICD-10	International Statistical Classification of Diseases and Related Health Problems, 10^th^Revision
IPAQ	International Physical Activity Questionnaire
OR	Odds Ratio
PR	Prevalence Ratio
SBC	Brazilian Society of Cardiology (in Portuguese, *Sociedade Brasileira de Cardiologia)*
SBP	Systolic Blood Pressure
SDI	Sociodemographic Index
SUS	Brazilian Unified Health System (in Portuguese, *Sistema Único de Saúde* )
UI	Uncertainty Interval
YLD	Years Lived with Disability
YLL	Years of Life Lost

**Table 7-1:** – Rates of self-reported hypertension in individuals aged 18 years or more and 95% confidence intervals, by sex, in Brazil, its regions, Federative Units and residence areas (urban or rural), 2019.

	Self-reported hypertension rates (%)								
	Total			Sex					
				Male			Female		
	Rate	95% CI		Rate	95% CI		Rate	95% CI	
		Lower limit	Upper limit		Lower limit	Upper limit		Lower limit	Upper limit
Brazil	23.9	23.5	24.4	21.1	20.4	21.7	26.4	25.8	27.1
Urban	24.0	23.5	24.6	21.4	20.7	22.1	26.3	25.5	27.0
Rural	23.2	22.3	24.1	19.1	18.0	20.2	27.9	26.4	29.4
North	16.8	16.0	17.6	14.0	13.1	15.0	19.4	18.2	20.5
Rondônia	18.8	16.9	20.7	16.1	13.7	18.6	21.4	18.3	24.4
Acre	19.2	17.3	21.0	17.7	15.0	20.4	20.5	17.9	23.1
Amazonas	16.0	14.6	17.3	13.9	12.1	15.7	17.9	16.0	19.8
Roraima	15.7	14.0	17.4	12.7	10.5	14.8	18.6	16.0	21.3
Pará	15.3	14.0	16.7	12.0	10.3	13.6	18.4	16.4	20.5
Amapá	18.2	15.7	20.7	15.3	12.5	18.0	20.9	17.4	24.4
Tocantins	22.5	20.2	24.8	20.3	17.3	23.3	24.6	21.6	27.6
Northeast	23.1	22.5	23.7	19.5	18.6	20.4	26.2	25.4	27.1
Maranhão	19.3	18.0	20.6	16.8	15.1	18.6	21.5	19.8	23.3
Piauí	23.6	21.6	25.6	22.9	19.9	25.9	24.3	21.7	26.8
Ceará	21.3	19.8	22.7	17.1	15.2	19.0	24.9	22.8	26.9
Rio Grande do Norte	21.9	20.3	23.5	18.5	16.6	20.4	24.8	22.5	27.2
Paraíba	25.1	23.1	27.1	22.0	19.1	24.9	27.7	25.3	30.1
Pernambuco	23.4	22.2	24.7	18.4	16.4	20.3	27.5	25.7	29.4
Alagoas	23.9	22.4	25.4	19.1	16.7	21.5	27.9	25.8	30.0
Sergipe	22.5	20.8	24.3	18.6	15.9	21.3	26.0	23.6	28.3
Bahia	25.2	23.6	26.8	21.8	19.4	24.2	28.3	26.0	30.5
Southeast	25.9	25.0	26.8	23.1	21.9	24.3	28.3	27.0	29.5
Minas Gerais	27.7	25.9	29.5	25.5	23.1	27.9	29.7	27.2	32.1
Espírito Santo	25.5	23.9	27.2	23.7	21.7	25.7	27.1	24.5	29.7
Rio de Janeiro	28.1	26.7	29.4	24.8	22.8	26.7	30.7	28.9	32.5
São Paulo	24.2	22.8	25.6	21.3	19.4	23.2	26.8	24.8	28.8
South	24.5	23.5	25.5	22.0	20.8	23.3	26.7	25.3	28.2
Paraná	22.9	21.2	24.7	22.6	20.2	25.0	23.2	20.9	25.6
Santa Catarina	23.6	22.0	25.2	20.5	18.5	22.5	26.6	24.4	28.7
Rio Grande do Sul	26.6	24.8	28.4	22.5	20.4	24.5	30.3	27.6	32.9
West-Central	21.9	20.9	23.0	20.5	18.9	22.1	23.2	21.8	24.6
Mato Grosso do Sul	24.5	22.7	26.4	23.2	20.6	25.7	25.7	23.3	28.1
Mato Grosso	21.6	19.8	23.5	20.4	17.3	23.4	22.8	20.0	25.5
Goiás	23.4	21.4	25.4	22.0	19.1	25.0	24.7	22.2	27.2
Distrito Federal	16.6	14.7	18.4	14.6	12.3	16.9	18.2	15.5	20.9

*Source: IBGE, Diretoria de Pesquisas, Coordenação de Trabalho e Rendimento, Pesquisa Nacional de Saúde 2019. ^306^*

**Table 7-2:** – Rates of self-reported hypertension in individuals aged 18 years or more and 95% confidence intervals, according to sociodemographic variables, Brazil, 2019.

Self-reported hypertension rates			
Variable	Rate	95% CI	
		Lower limit	Upper limit
**Age group**			
18-29 years	2.8	2.4	3.3
30-59 years	20.3	19.6	20.9
60-64 years	46.9	44.9	48.9
65-74 years	56.6	54.9	58.2
75 or more	62.1	60.1	64.1
**Education level**			
None or incomplete elementary	36.6	35.7	37.4
Elementary or incomplete High School	20.4	19.1	21.6
High School or incomplete College	15.4	14.7	16.1
College	18.2	17.1	19.3
**Race/skin color**			
White	24.4	23.6	25.1
Black	25.8	24.4	27.2
Brown	22.9	22.2	23.5
**Employment status**			
Employed	16.9	16.4	17.4
Unemployed	11.9	10.1	13.7
Not in labor force	38.7	37.8	39.6
**Monthly income (minimum wage)**			
None - ¼	16.4	15.1	17.8
1/4 - 1/2	18.7	17.6	19.8
1/2 - 1	25.8	24.9	26.8
1 - 2	25.7	24.9	26.6
2 - 3	25.3	23.5	27.1
3 – 5	25.2	23.2	27.3
More than 5	25.0	23.0	27.1
Total	23.9	23.5	24.4

*Source: IBGE, Diretoria de Pesquisas, Coordenação de Trabalho e Rendimento, Pesquisa Nacional de Saúde 2019. ^306^*

**Table 7-3:** – Number of deaths, age-standardized mortality rates (per 100 000) due to high systolic blood pressure from all causes, and percent change of rates, Brazil, 1990 and 2019.

Age group	1990		2019		Percent change (95% UI)
	Number (95% UI)	Rate (95% UI)	Number (95% UI)	Rate (95% UI)	
15-49 years	19166.8 (16242.9;22027.6)	25 (21.2;28.7)	17477.5 (15011.4;19693.6)	15.1 (13;17.1)	-39.5 (-43.9;-34.5)
50-69 years	62163.9 (55971.7;67686.6)	396.3 (356.8;431.5)	82839 (74580.1;90234.2)	205.3 (184.9;223.7)	-48.2 (-51.1;-45.1)
70+ years	71875.6 (61751.8;81924.4)	1699.2 (1459.8;1936.7)	139099.3 (116155.4;157920.7)	1062.8 (887.5;1206.6)	-37.5 (-42.3;-33)
Age-standardized		197.3 (174.7;218.9)		104.8 (91.2;116.2)	-46.9 (-49.6;-44)
All Ages	153206.3 (137435.7;167785.4)	102.9 (92.3;112.7)	239415.9 (209603.5;264681.2)	110.5 (96.7;122.2)	7.3 (0.7;13.6)

– Age-standardized mortality rates (per 100 000) due to high systolic blood pressure from all causes, and percent change of rates, by sex, in Brazil and its Federative Units, 1990 and 2019.

**Table 7-4:** – Age-standardized mortality rates (per 100 000) due to high systolic blood pressure from all causes, and percent change of rates, by sex, in Brazil and its Federative Units, 1990 and 2019.

Location	Female			Male		
	1990 Rate (95% UI)	2019 Rate (95% UI)	Percent Change (95% UI)	1990 Rate (95% UI)	2019 Rate (95% UI)	Percent Change (95% UI)
Acre	126.7 (101.3;148.1)	83.3 (67.6;98.4)	-34.2 (-45;-21.4)	184.6 (152.2;215.6)	131.1 (109.6;153.2)	-29 (-39.8;-15.8)
Alagoas	181.3 (150.6;212.5)	130 (105.7;156.2)	-28.3 (-42.1;-11.6)	212.2 (177.3;247.4)	159.9 (129.1;193.4)	-24.7 (-39.9;-5.6)
Amapá	105 (83.5;128.6)	71.1 (57.5;85.9)	-32.2 (-45;-16.7)	138 (114.7;162.8)	102.3 (85.6;120.5)	-25.9 (-37.3;-11.7)
Amazonas	134.2 (105.1;164)	63.1 (49.8;77.6)	-53 (-63;-39.3)	166.9 (138.9;196.7)	106.1 (86.9;124.8)	-36.4 (-48.2;-23.5)
Bahia	154.2 (120.8;185.7)	87.3 (67.7;109.1)	-43.4 (-55.8;-26.3)	186 (153;220.6)	152.5 (121.1;189.6)	-18 (-36.5;6.2)
Brazil	171.3 (149.6;192.2)	86.3 (73.4;97.2)	-49.6 (-53.2;-45.8)	225.9 (199.7;249.4)	126.9 (111.6;140.4)	-43.8 (-47.2;-40.1)
Ceará	102.6 (77.9;128.7)	82.5 (62.2;104.9)	-19.6 (-39.7;11.6)	128.5 (99.9;159.6)	116.7 (89.8;148.5)	-9.2 (-33.8;24.7)
Distrito Federal	202.9 (162.3;245.4)	89.7 (71.3;107.4)	-55.8 (-64.8;-44.8)	267.3 (216.5;320.7)	110.3 (87.4;133.3)	-58.7 (-66.5;-49.6)
Espírito Santo	176.5 (143;207.5)	93.4 (75.7;112.1)	-47.1 (-57.4;-35.6)	230.4 (201;264)	143.1 (117.4;168.7)	-37.9 (-48.8;-26.8)
Goiás	179 (140.1;224.8)	83.8 (65.6;104.1)	-53.2 (-64;-37.8)	231.5 (184.9;278.1)	119.5 (93.5;147.2)	-48.4 (-60.4;-33.1)
Maranhão	88 (65.4;111.3)	94.7 (73;119.2)	7.6 (-19.2;49)	250.9 (195.5;309.1)	188.8 (151.6;236.6)	-24.8 (-42.6;0.3)
Mato Grosso	148.2 (117.7;179.1)	82.7 (66.3;99.4)	-44.2 (-56.1;-30.5)	170.8 (134.9;207.7)	97 (78.7;117)	-43.2 (-54;-28.9)
Mato Grosso do Sul	175.7 (141.9;204.8)	89.5 (73.8;107.9)	-49.1 (-57.9;-37.1)	213.9 (181.9;244.1)	123.1 (101.6;145.8)	-42.4 (-52.4;-31.3)
Minas Gerais	172.1 (144.1;201.1)	73.2 (58.7;88.1)	-57.5 (-65;-49.3)	226.1 (191.8;260.6)	100.2 (83;118.2)	-55.7 (-62.7;-47.9)
Pará	158.8 (124.9;195.2)	75.6 (60.2;89.5)	-52.4 (-62.9;-38.9)	183.4 (144.7;222.4)	109.8 (89.4;131.2)	-40.1 (-51.9;-23.7)
Paraíba	132.6 (107.2;159.1)	88.2 (70.5;109.8)	-33.5 (-46.9;-15.2)	153.1 (124.2;184.7)	126.9 (102.5;155.4)	-17.1 (-34.9;3.8)
Paraná	213.3 (175;248.8)	92.9 (74.8;110.3)	-56.5 (-65;-47.4)	261.2 (227;296.5)	132.3 (108;156.9)	-49.3 (-57.4;-40.1)
Pernambuco	170.1 (139.5;200.4)	100.2 (81.5;119.8)	-41.1 (-51.9;-27)	206 (176.3;235)	156.7 (127.7;186.6)	-23.9 (-36.7;-7.9)
Piauí	135.9 (107.9;163.4)	83.3 (65.7;101.5)	-38.7 (-50.4;-22)	220.5 (182.2;259.1)	124.1 (102.9;146.2)	-43.7 (-53.1;-32.3)
Rio de Janeiro	212.6 (175.4;247.1)	94.3 (77.2;112.4)	-55.6 (-63.1;-45.6)	278.6 (236.7;316.6)	137.6 (114.4;161.6)	-50.6 (-58;-40.7)
Rio Grande do Norte	113.2 (88.6;138.2)	74.4 (56.7;92.4)	-34.3 (-49.7;-15.2)	151 (123.3;182.8)	114.1 (89.5;143)	-24.4 (-42.3;-1.8)
Rio Grande do Sul	185.7 (158.2;211.6)	84 (68.5;100)	-54.8 (-61.7;-46.4)	231.4 (201;258.6)	122.6 (104;144.7)	-47 (-54.7;-38.7)
Rondônia	245.2 (197.8;292.3)	98.3 (79.4;117.4)	-59.9 (-67.7;-50.1)	225.2 (182.5;268.9)	115.6 (92.7;139.2)	-48.6 (-59.4;-34.8)
Roraima	165 (130.7;198)	89.4 (72.9;106.3)	-45.8 (-55.6;-31.9)	250.1 (213.1;288.5)	136.2 (115.7;156.1)	-45.5 (-53.2;-35.9)
Santa Catarina	200.1 (163.1;235.6)	88.1 (71.1;105)	-56 (-63.7;-46.3)	243.6 (209.6;278.2)	115.6 (96.3;136)	-52.6 (-60.2;-44.3)
São Paulo	196.5 (162.4;231.3)	87.1 (70.3;104)	-55.7 (-63.4;-46)	277.6 (238.9;313.9)	128.2 (107.7;148.6)	-53.8 (-60.5;-46.1)
Sergipe	163.7 (129.8;196.1)	96.4 (77.8;118)	-41.1 (-53.6;-23.7)	197.3 (163.7;231.9)	128.4 (101.1;157.4)	-34.9 (-48.2;-17.2)
Tocantins	145.9 (113.6;178.7)	82.2 (64;100.5)	-43.7 (-56.5;-26.6)	180.1 (141.7;220.5)	137.9 (109.1;169.5)	-23.5 (-40.2;0.1)

*Source: Data derived from Global Burden of Disease Study 2019, Institute for Health Metrics and Evaluation, University of Washington. ^46^*

**Table 7-5:** – Number of deaths, age-standardized mortality rates (per 100 000) due to high systolic blood pressure from cardiovascular diseases, and percent change of rates, by age group. Brazil, 1990 and 2019.

Age group	1990		2019		Percent Change (95% UI)
	Number (95% UI)	Rate (95% UI)	Number (95% UI)	Rate (95% UI)	
15-49 years	18030.1 (15194.2;20699.9)	23.5 (19.8;27)	16125.8 (13748;18241.4)	14 (11.9;15.8)	-40.6 (-45.2;-35.6)
50-69 years	58938.4 (52867.6;64283.7)	375.7 (337;409.8)	74600.9 (66631.3;81646.5)	184.9 (165.2;202.4)	-50.8 (-53.6;-47.9)
70+ years	67682.3 (57758.8;77603.7)	1600 (1365.4;1834.6)	122839.6 (101314.4;141062.5)	938.5 (774.1;1077.8)	-41.3 (-45.9;-37.3)
Age-standardized	144650.8 (129424.1;159074)	186.1 (163.8;206.7)	213566.3 (185076;237650.4)	93.4 (80.2;104.2)	-49.8 (-52.5;-47.1)
All Ages	144650.8 (129424.1;159074)	97.2 (87;106.9)	213566.3 (185076;237650.4)	98.6 (85.4;109.7)	1.4 (-5.1;7.3)

**Table 7-6:** – Age-standardized mortality rates (per 100 000) due to high systolic blood pressure from cardiovascular diseases and percent change of rates, by sex, in Brazil and its Federative Units, 1990 and 2019.

Location	Female			Male		
	1990 Rate (95% UI)	2019 Rate (95% UI)	Percent Change (95% UI)	1990 Rate (95% UI)	2019 Rate (95% UI)	Percent Change (95% UI)
Acre	116.1(92;136.8)	71.2(57.1;84.6)	-38.6(-49.2;-25.6)	166.2(135.5;196.1)	109.8(90.3;129.4)	-33.9(-44.7;-21.2)
Alagoas	167.8(138.8;198.4)	117.2(95;141)	-30.1(-43.9;-13.2)	197.2(163.7;232.3)	143.5(115.7;175.4)	-27.2(-42.5;-8.6)
Amapá	95.4(75;118)	59.8(47.9;73.3)	-37.3(-49.7;-22.3)	124.2(102.1;147.7)	86.5(71.9;102.9)	-30.4(-41.6;-16.8)
Amazonas	124.8(96.6;153.6)	53.2(41.7;66.1)	-57.4(-66.4;-44.5)	153.7(126.8;183)	90.4(73.8;107.4)	-41.2(-52.1;-29.4)
Bahia	144.4(112.7;173.9)	77.6(59.9;97.5)	-46.2(-58.1;-30)	172.6(141.2;205.3)	133.3(105.4;166.6)	-22.8(-40.6;0.9)
Brazil	161.6(140.1;182.2)	76.8(64.8;87.4)	-52.5(-56;-49)	212.6(187.4;236)	113(98.4;126.1)	-46.8(-50.3;-43.3)
Ceará	96.4(72.7;121.8)	74.9(56.2;95.5)	-22.3(-41.9;7.6)	121.1(93.5;151.1)	105.1(80.4;135)	-13.2(-36.8;19.1)
Distrito Federal	191.7(152.8;233.2)	80.5(63.2;97.7)	-58(-67.1;-47.4)	246.2(197.3;298.1)	95.3(74.6;116.3)	-61.3(-68.9;-52.5)
Espírito Santo	167.5(135.3;198.4)	83.6(67.4;100.9)	-50.1(-59.9;-39)	218.1(189.1;250.9)	129(105.5;152.6)	-40.9(-51.2;-30.1)
Goiás	168.1(130.5;211.1)	73.2(56.6;91.3)	-56.5(-66.7;-42.1)	214.9(170.3;259.1)	105.1(82;129.8)	-51.1(-62.6;-36.4)
Maranhão	81.4(59.6;104)	85.3(65.2;107.9)	4.8(-21;45.1)	233.6(180.1;289.3)	169.5(134.7;213.8)	-27.5(-44.8;-2.6)
Mato Grosso	138.1(108.7;167.9)	71.7(56.8;86.7)	-48.1(-59.2;-35.2)	158.8(124.8;193.6)	84.3(67.9;102.4)	-46.9(-57.3;-33.4)
Mato Grosso do Sul	166.1(132.8;194.5)	80(65.6;96.7)	-51.8(-60.4;-40.5)	200.8(169.6;229.9)	110.6(91;131.6)	-44.9(-54.4;-33.7)
Minas Gerais	161.1(134.2;189.5)	63.8(50.8;77.3)	-60.4(-67.5;-52.6)	210.2(177.7;242.6)	88.3(72.7;104.7)	-58(-64.7;-50.3)
Pará	148.2(115.8;183.2)	65.9(51.9;78.9)	-55.5(-65.5;-43.1)	169.4(132.3;206.5)	96.3(77.9;116.1)	-43.1(-54.6;-27)
Paraíba	122.2(98.6;147.3)	78.9(62.4;98.4)	-35.5(-48.9;-17.3)	142.5(114.7;174.1)	112.6(90.9;138.4)	-21(-38.4;-0.6)
Paraná	203.1(165.1;237)	82.7(65.9;99)	-59.3(-67.3;-50.7)	248.6(214.7;283.2)	118.6(96.7;141.2)	-52.3(-59.9;-43.4)
Pernambuco	160.4(130.4;189.8)	90.5(73.1;109.2)	-43.6(-54.5;-29.7)	194.3(165.6;222.6)	142.2(115.1;170.4)	-26.8(-39.2;-11.1)
Piauí	128.2(101.6;154.5)	76.2(60.1;93.2)	-40.6(-52.2;-24.4)	208(170.5;246)	112.9(93.3;134.1)	-45.7(-55;-34.3)
Rio de Janeiro	202.8(166.2;236.8)	82.9(67.3;99.5)	-59.1(-66.1;-49.7)	264.8(223.9;301.9)	121.3(99.5;143.2)	-54.2(-61.3;-44.7)
Rio Grande do Norte	106.4(83.3;130.8)	66.2(50.3;82.6)	-37.7(-52.4;-19)	141.8(115.1;172.8)	101.5(79.2;127.3)	-28.4(-45.6;-6.9)
Rio Grande do Sul	176.3(149.6;201.7)	74.8(60.4;90)	-57.6(-64.4;-49.4)	218.6(189;244.8)	109.8(92.9;130.2)	-49.8(-57.1;-41.8)
Rondônia	228.3(183.1;274.5)	84.6(67.3;102.1)	-62.9(-70.3;-53.3)	208.2(167.3;250.2)	99.9(79.6;121.2)	-52(-62.1;-38.7)
Roraima	149.9(116.9;180.6)	75.2(60.5;90.3)	-49.9(-59.2;-36)	228.8(193.7;265.3)	117.3(98.4;135.8)	-48.7(-56.2;-39.3)
Santa Catarina	189.7(154.5;224.4)	78.4(62.5;94.6)	-58.7(-66;-49.6)	230.8(196.7;264.2)	103.7(85.9;122.8)	-55.1(-62.5;-46.9)
São Paulo	186.2(152.7;219.7)	78(62.6;93.7)	-58.1(-65.7;-48.7)	262.4(225.1;297.9)	115.2(96;134.4)	-56.1(-62.3;-48.6)
Sergipe	152.1(119.4;183)	86.2(68.7;106.3)	-43.3(-55.5;-26.5)	180.6(149.3;214.3)	112.2(87.2;137.6)	-37.9(-50.7;-20.7)
Tocantins	135.5(105.4;166.9)	72.7(55.6;89.7)	-46.3(-58.8;-29.5)	165.3(128.4;203.8)	120.2(94.5;148.6)	-27.3(-43.4;-4.1)

*Source: Data derived from Global Burden of Disease Study 2019, Institute for Health Metrics and Evaluation, University of Washington. ^46^*

**Chart 7-1:** – Number of deaths (A) and mortality rates (B) attributable to high systolic blood pressure in Brazil, 1990-2019.

Source: Data derived from Global Burden of Disease Study 2019, Institute for Health Metrics and Evaluation, University of Washington. ^46^

**Chart 7-2:** – Proportional mortality due to high systolic blood pressure according to age groups, by sex, in Brazil, 2019.

Source: Data derived from Global Burden of Disease Study 2019, Institute for Health Metrics and Evaluation, University of Washington. ^46^

**Chart 7-3:** – Age-standardized mortality rates of diseases attributable to high systolic blood pressure, stratified by all causes, in Brazil, 1990 and 2019.

Source: Data derived from Global Burden of Disease Study 2019, Institute for Health Metrics and Evaluation, University of Washington. ^46^

**Chart 7-4:** – Age-standardized mortality rates of diseases attributable to elevated systolic blood pressure, stratified by cardiovascular diseases, in Brazil, 1990 and 2019.

Source: Data derived from Global Burden of Disease Study 2019, Institute for Health Metrics and Evaluation, University of Washington. ^46^

**Chart 7-5:** – Correlation between the 2019 sociodemographic index (SDI) and the percent change of mortality rates from cardiovascular disease attributable to high systolic blood pressure from 1990 to 2019, in Brazil.

Source: Data derived from Global Burden of Disease Study 2019, Institute for Health Metrics and Evaluation, University of Washington. ^46^

**Chart 7-6:** – Absolute number of YLLs (A), YLDs (B), and DALYs (C) due to hypertension, in Brazil, 1990-2020.

Source: Data derived from Global Burden of Disease Study 2019, Institute for Health Metrics and Evaluation, University of Washington. ^46^

**Chart 7-7:** – Rates of YLLs (A), YLDs (B), and DALYs (C) due to hypertension, in Brazil, 1990-2020.

### Overview

• For standardization purposes, in this document arterial hypertension was characterized as sustained blood pressure levels equal to or greater than 140 mm Hg for SBP and/or equal to or greater than 90 mm Hg for DBP. ^267^ The percentage prevalence values will be presented, followed by 95% CIs or 95% UIs, as available in the cited studies.

• In population studies, hypertension can be either measured or self-reported. The former derives from direct blood pressure measurement using standardized techniques and is described in each document, while self-reported hypertension is characterized by a positive answer to a question about the presence of that medical diagnosis or by a positive answer regarding the use of antihypertensive medications, ^268^ depending, therefore, on the patient’s access to the diagnosis and understanding of this information.

• When the GBD Study is the data source, the risk is attributable to high SBP, as described in a previous publication. ^269^

### Prevalence and Incidence

#### Incidence

• In a 2021 study by Lopes *et al.* analyzing 8154 participants from the ELSA-Brasil study, the incidence of hypertension per 1000 person-years was 43.2, 51.6 in men and 37.3 in women. Still, the incidence per 1000 person-years was higher in the ‘70-74 years’ age group (88.8), in black (55.9), obese (79.7), or diabetic (91.4) individuals, and in those with lower socioeconomic status (58.9). ^270^

#### Prevalence

• Data on the prevalence of hypertension may differ between studies, depending on the type of research and the study population, especially in a country with continental dimensions and substantial miscegenation like Brazil.

#### Measured Hypertension

• According to Malta *et al* ., in an analysis of the 2013 National Health Survey data, the prevalence of measured hypertension in individuals older than 18 years was 22.8% (95% CI, 22.1 - 23.4%) in a sample of 59 402 individuals. ^271^ In individuals older than 75 years, the estimated prevalence was 47.1% (95% CI, 44.1 - 50.2%), and, in an analysis stratified by sex, in the age groups between 18 years and 74 years, the prevalence was higher in men, with women showing a slight predominance only in the age group ‘above 75 years’ (47.8%, 95% CI, 43.9 -51.7 *versus* 46.2%, 95% CI, 41.3 - 51.3%), revealing a possible survival bias. The analysis by region showed that the Southeastern (25%, 95% CI, 23.8 - 26.1%) and Southern (25%, 95% CI, 23.5 - 26.5%) regions had the highest prevalence in both sexes. ^271^

• In the ELSA study, Chor *et al* . observed a hypertension prevalence of 40.1% in men and 32.2% in women, with a progressive increase with age, reaching 63.7% in individuals aged 65 years to 74 years. ^6^ In addition, there was a lower prevalence in individuals with postgraduation (28.4%), when compared with those without a complete secondary education (44%), and in those with a *per capita* family income above USD $1000.00 (30.7%), when compared with those with an income below USD $500.00 (40.9%). The evaluation by race showed a higher prevalence among blacks (49.3%) as compared to other race/color categories. ^272^

• In a cross-sectional study carried out in a population from the semiarid region of the state of Pernambuco, Santiago *et al.* observed that the prevalence of measured hypertension was similar among individuals who were white (28.9%, 95% CI, 19.8 - 39.4%) *versus* those who were brown or black (27%, 95% CI, 22.3 - 32.2%). ^7^ When the education level was considered in the analysis, the prevalence was 44.6% (95% CI, 36.4 - 53%) in those who had never attended school, almost twice as high as in those who had completed elementary or secondary/higher education. The assessment by employment status showed a higher prevalence in individuals without any work activity (30.3%, 95% CI, 24.4 - 36.7%) when compared with those who worked (23.9%, 95% CI, 18.0 - 30.7%). Still, the prevalence was higher in individuals belonging to the upper and middle classes (20.5%, 95% CI, 15.3 - 26.4%) compared with those belonging to the lower class (35.2%, 95% CI, 28.5 - 42.3%). ^273^

• Barbosa *et al* . estimated the prevalence of hypertension and its associated factors in the less developed regions of Brazil in a cross-sectional study carried out with 835 individuals >18 years of age, who completed a structured household questionnaire. ^274^ Measurements of blood pressure, weight, height, and waist circumference were taken, and other risk factors for CVD were assessed. Age varied from 18 to 94 years (mean age, 39.4 years), 293 (35.1%) individuals were normotensive and 313 (37.5%) were prehypertensive. The hypertension prevalence was 27.4% (95% CI, 24.4% - 30.6%) and higher among men (32.1%) than among women (24.2%). In the adjusted analysis, the following variables remained independently associated with hypertension: male sex (PR 1.52, 95% CI, 1.25 - 1.84); age ≥ 30 years (PR 6.65, 95% CI, 4.40 - 10.05 for ≥60 years of age); overweight (PR 2.09, 95% CI, 1.64 - 2.68); obesity (PR 2.68, 95% CI, 2.03 - 3.53); and diabetes (PR 1.56, 95% CI, 1.24 - 1.97). ^274^

#### Self-reported Hypertension

• Self-report is the most frequently used criterion to assess the prevalence of hypertension in Brazil. Picon *et al* ., in a meta-analysis involving 120 018 individuals, demonstrated that, despite different criteria for hypertension diagnosis in the 1980s and 1990s, the prevalence of hypertension reduced from 36.1% (95% CI, 28.7 - 44.2%) to 28.7% (95% CI, 26.2 - 31.4%) over 30 years. ^274^ However, in the early 2000s, the prevalence estimate on telephone inquiries was still 20.6% (95% CI, 19.0 - 22.4%). ^275^

• An analysis of data from the 2013 National Health Survey showed a prevalence of hypertension in men of 18.3% (95% CI, 17.5 - 19.1%), according to the self-reported criterion, of 25.8% (95% CI, 24.8 - 26.7%), when measured by an instrument, and of 33% (95% CI, 32.1 - 34.0%), when measured by an instrument and/or use of antihypertensive medication reported. In women, the observed percentages were higher according to the self-reported criterion [24.2% (95% CI, 23.4 - 24.9%)] and lower according to all other diagnostic criteria: 20% (95% CI, 19.3 - 20.8%), when measured by an instrument, and 31.7% (95% CI, 30.9 - 32.5%), when measured by an instrument and/or use of antihypertensive medication reported. ^268^ These differences can be explained by the fact that women seek health services more frequently and are more likely to be diagnosed with hypertension. The Southeastern and Southern regions and the urban areas of the country had the highest prevalence when the geographic regions and the area of residence were analyzed, respectively. ^268^

• In the VIGITEL study, ^276^ based on telephone interviews carried out in 26 capitals and in the Distrito Federal in 2019, the prevalence of hypertension was 24.5%, specifically 27.3% in women and 21.2% in men. The highest prevalence was observed in the Distrito Federal (29.6%) and the cities of Porto Alegre (27.2%) and Recife (26%) for men, and in the cities of Rio de Janeiro (32.8%), Recife (30.2%), and Salvador (30%) for women. ^276^ In addition, that study assessed the relation to level of education and found that, in individuals with up to 8 years of schooling, hypertension prevalence was approximately three times higher than that of individuals with at least 12 years of schooling, for both sexes. ^276^

• Using estimates from the GBD Study 2017, Nascimento *et al* . reported an increased prevalence in age-standardized high SBP from 16.9% (95% UI, 16.5 - 17.3%) in 1990 to 18.9% (95% UI, 18.5 - 19.3%) in 2017, which was higher among men throughout the study period. Furthermore, the growth rate in the country was +0.4% per year among adults older than 25 years. ^11^ When analyzed by sex, the highest prevalence in 2017 was observed in the state of Santa Catarina (28.8%, 95% UI, 26.6-31%) in the male sex and in the state of Sergipe (19.8%, 95% UI, 17.7-21.9%) in the female sex. ^277^

• Table 7-1 shows the prevalence of self-reported hypertension in Brazil according to sex in adults aged 18 years or more in 2019, as reported by the IBGE. The prevalence of hypertension was 23.9% in the country and higher in the female as compared to the male sex (26.4% *versus* 21.1%, respectively). There was a higher prevalence in urban areas as compared to rural ones (24.0% *versus* 23.2%, respectively), and in the Southeastern region for both sexes (23.1% for men and 28.3% for women).

• Table 7-2 shows the prevalence of self-reported hypertension according to sociodemographic variables, as reported by the IBGE. Of note, the highest prevalence rates were observed in individuals older than 75 years (62.1%), in those who had no education or few schooling years (36.6%), and in black (25.8%) and unemployed (38.7%) individuals. In contrast, when the income criterion was used, those with lower income had a lower prevalence of hypertension (18.7%), probably reflecting the lack of access to the health care system.

## Mortality

### Total Mortality Attributable to Hypertension

• Oliveira *et al* . compared the all-cause mortality attributable to hypertension in two population groups, a Brazilian and an English. The data used were obtained from the ELSA cohort, with 3205 individuals, and from the Bambuí cohort, with 1382 participants, all of them older than 60 years. Of note, the sociodemographic characteristics were different between the two cohorts, and the levels of education and family income were higher in the English cohort, while the rates of smoking and diabetes were higher in the Brazilian cohort. Regarding relative risks, in the English cohort, there was no difference when hypertension was assessed alone, while in the Brazilian cohort, hypertension conferred a relative risk of 1.43 (95% CI, 1.06 - 1.94, p=0.028) for mortality in 6 years. Furthermore, deaths attributable to hypertension in the Brazilian cohort were 25.3% (95% CI, 8.2 - 39.3%), far surpassing the English cohort, in which the result was borderline and without adequate statistical precision. ^278^

• Table 7-3 shows the number of deaths and the age-standardized mortality rates attributable to high SBP from all causes according to the GBD Study 2019 data. Note that in older age groups, the number of deaths and the mortality rate are higher, as expected for this disease. However, the mortality rate attributable to high SBP in 2019 was 104.8 per 100 000 inhabitants; there was a 46.9% reduction over the study period, which may represent a lower incidence and greater therapeutic control of hypertension, as well as an improvement in the socioeconomic conditions observed in the period. ^37^ ^,^ ^279^

• Table 7-4, depicting the age-standardized mortality rates attributable to high SBP from all causes according to sex and Brazilian FUs, shows an almost 50% decrease in mortality rates between 1990 and 2019 for both sexes, and the reduction was higher in women as compared to men (49.6% *versus* 43.8%, respectively). However, for women, except for the state of Rondônia — which had the greatest percentage reduction in mortality rate attributed to high SBP — the greatest reductions were found in the FUs of the Southeastern and Southern regions, and in the Distrito Federal. The state of Maranhão was the only FU that showed an increase in the rates during the study period. Among men, all FUs showed a reduction in mortality attributed to high SBP, with the largest percentage decreases recorded in the FUs of the Southern and Southeastern regions, as observed for women (Table 7-4) **.**

• Chart 7-1 shows the number of deaths (A) and mortality rates (B) attributable to high SBP between 1990 and 2019. With increasing life expectancy, the consequent aging of the population, and the data observed in the previous tables, the number of deaths related to high SBP was also expected to increase, as shown in Chart 7-1A. However, when the effect of age was mitigated by age-standardization of the rates, a decrease in mortality rate was observed over time (1B).

• The proportional mortality by age groups in 2019 is shown in Chart 7-2. Of note, there is a greater proportion of deaths in the groups between 55 years and 74 years for women; however, for men, the greatest proportion is observed in the age groups 10 years younger (45 years to 64 years). Importantly, the age groups ‘above 75 years’ for women and ‘above 65 years’ for men may have other concurrent diseases; therefore, the proportion of deaths from high SBP tends to decrease due to competitive causes, which may, at least in part, explain the data described above.

• Chart 7-3 presents the age-standardized mortality rates of diseases attributable to high SBP, stratified for all causes, between 1990 and 2019. As shown, most deaths are related to major CVDs, *i.e.,* ischemic heart disease, cerebrovascular disease, and hypertensive diseases, all three of which decreased between 1990 and 2019. Notably, hypertensive diseases showed the smallest reduction when compared with the other two diseases. This may be related to the rules for the selection of the underlying cause of death in death certificates, in which, because of the use of specific algorithms, hypertensive disease is rarely selected as the underlying cause of death. ^280^

### Cardiovascular Mortality Attributable to Hypertension

• Regarding cardiovascular mortality attributable to high SBP, Table 7-5 shows the number of deaths, mortality rates, and the percent change of rates between 1990 and 2019 according to GBD estimates. There was a decrease by almost 50% in the period, from 186.1 per 100 000 inhabitants in 1990 to 93.4 per 100 000 inhabitants in 2019 (Table 7-5), and, similarly to all-cause mortality, the highest rates were observed in the older age groups.

• Table 7-6 presents the mortality from CVDs attributed to high SBP by FU and sex, indicating a trend similar to that shown in Tables 7-3 and 7-4, both of which show mortality from all causes and include evaluation by age group and FU. Thus, there was a reduction in the cardiovascular mortality attributable to hypertension by 52.5% among women and by 46.8% among men between 1990 and 2019 in Brazil. For females, the FUs with the greatest reductions were the states of Rondônia, Minas Gerais, Paraná, and Rio de Janeiro, and the Distrito Federal, with 62.9%, 60.4%, 59.3%, 59.1%, and 58%, respectively (Table 7-6), while, for males, the greatest reductions were observed in the Distrito Federal, and the states of Minas Gerais, São Paulo, Santa Catarina, and Rio de Janeiro, with 61.3%, 58%, 56.1%, 55.1%, and 54.2%, respectively.

• Chart 7-4 shows the age-standardized mortality rates of diseases attributable to elevated SBP, stratified by CVDs, between 1990 and 2019. The mortality rates from chronic kidney disease remained stable, while those from ischemic heart diseases, stroke, hypertensive diseases, and cardiomyopathies and myocarditis decreased over that period.

• Finally, Chart 7-5 shows the relationship between the 2019 SDI and the percent change in deaths from CVD attributable to high SBP from 1990 to 2019, in Brazil, by FU. Chart 7-5 shows that the greatest reductions in age-standardized mortality rate occurred in those FUs with the highest SDI values in an almost linear relationship, with a correlation coefficient of 0.78 and p-value=0.

### Burden of Disease

• Chart 7-6 shows the absolute number of YLLs (A), YLDs (B), and DALYs (C) related to hypertension between 1990 and 2020. It is worth noting the trend towards an increase in YLLs (A) and mainly in YLDs (B), with a consequent impact on the DALYs curve (A). These observations can be justified, at least partially, by population growth and aging.

• In contrast, the age-standardized rates in Chart 7-7 show a reduction in YLLs (A) and in YLDs (B) with a consequent impact on DALYs (C). These curves reflect the attenuation of the population aging effect on the disease burden, with a smaller influence on YLDs, which may be partially explained by the inequality of access to the healthcare system, hindering the treatment of more severe diseases, with a consequent impact on YLDs.

### Impact on Cardiovascular Health

•Fuchs *et al.* investigated the association between clustering of risk factors and self-reported CVD among 1007 women living in the city of Porto Alegre, Southern Brazil. ^281^ The prevalence of hypertension, diabetes, physical activity, and the diet pattern were evaluated regarding their association, in a clustered fashion, with CVD, which was defined as the self-report of history of myocardial infarction, heart failure, stroke or coronary artery bypass surgery. The investigators found hypertension and diabetes as the main cluster associated with CVD, accounting for an independent risk ratio of 8.5 (95% CI, 3.0 - 24.5).

• Carvalho *et al.* , evaluating, in a sample of 333 individuals with treated hypertension or without hypertension, the association between hypertension and quality of life measured by the SF-36, showed that hypertensive patients had lower quality of life than their normotensive counterparts. ^282^ They found that normotensive individuals have higher HRQOL questionnaire scores as compared to hypertensive patients in all domains except for the ‘Emotional Aspect’. With respect to ‘Grouped Physical Component’, male hypertensive patients scored 298.4±72.6 as compared to 333.1±52.1 of normotensive males (P<0.01), and female hypertensive patients scored 243.8±84.0 as compared to 318.7±58.5 of normotensive females (P<0.01). Among males, the difference was significant for the ‘Functional Capacity’, ‘Social Aspect’, and ‘Mental Health’ domains of the SF-36, and, among females, the difference was observed for the ‘Emotional Aspect’ domain.

• To investigate the association of hypertension, prehypertension, age, duration of diagnosis, and blood pressure, Menezes *et al* . used the ELSA-Brasil data, which included 7063 patients, with a mean age of 58.9 years at baseline (2008-2010). The authors verified that hypertension was associated with the greatest decline in memory, fluency, and global cognitive score. Prehypertension was also an independent predictor of greater decline in the verbal fluency test and global cognitive score. Moreover, among treated individuals, blood pressure control at baseline was inversely associated with the decline in both global cognitive and memory test scores. ^283^

The association of CVD (coronary artery disease, myocardial infarction, and stroke) with diabetes, hypertension, and diabetes plus hypertension was investigated by Santos *et al.* in 2691 patients enrolled in the HIPERDIA program in the city of Fortaleza. ^284^ The authors confirmed a significant association of hypertension with stroke, coronary artery disease, and myocardial infarction (P<0.001).

### Costs and Healthcare Utilization

• Estimating the costs of hypertension, diabetes, and obesity in SUS patients in 2018, Nilson *et al.* noticed that the total cost of hypertension, diabetes, and obesity paid by the SUS reached R$ 3.45 billion (95% CI, 3.15 - 3.75), more than US$890 million. Of that total amount, 59% was spent with hypertension, 30% with diabetes, and 11% with obesity. When obesity was considered separately a risk factor for hypertension and diabetes, the cost attributable to this disease reached R$ 1.42 billion (95% CI, 0.98 - 1.87), corresponding to 41% of the total cost. ^285^

• Marinho *et al.* , investigating healthcare costs of patients with diabetes and hypertension, compared the direct costs of outpatient procedures with the amount reimbursed by the SUS. Their study’s main conclusion was that direct costs of the operation was higher than what SUS had been reimbursing, characterizing a public healthcare underfunding in real life that may compromise the quality of the control of cardiovascular risk factors. Of the costs, that with medications was the highest, followed by outsourced services and human resources. ^286^

• Queiroz *et al* . investigated the association between the number of cardiovascular risk factors or CVD and hospitalizations among 514 users of the SUS in the city of Presidente Prudente, São Paulo state. They concluded that hypertension, arrythmias, lower levels of physical activity, and myocardial infarction were associated with the number of days of hospitalization. In addition, they reported that the number of cardiovascular risk factors or CVD per patient was associated with the number of days of hospitalization. Nevertheless, rates of hospitalization in the last 12 months were independently higher only for those with arrythmias (OR 3.04, 95% CI, 1.74 - 5.31) and history of myocardial infarction (OR 3.07, 95% CI, 1.34 - 7.01). ^287^

### Hypertension Awareness, Treatment, and Control

• Using data from the 2013 National Health Survey, Macinko *et al* . estimated that about 36% of the Brazilian population (51.4 million) had a previous diagnosis and/or measured blood pressure of 140/90 mm Hg or more. Of these, 89% had contacted the health system in the previous 2 years, but only 65% were aware of their condition. From those aware of their hypertensive condition, 62% regularly sought care, 92% of whom had been prescribed medications. Of those who reported receiving medications, only 56% reported that ongoing care for their condition was free of barriers and included advice about managing important risk factors and behavior. Of the entire hypertensive population, about 33% had their blood pressure under control. ^288^

• To estimate prevalence, awareness, types of anti-hypertensive treatment, and the association of hypertension control with social status, data from the ELSA-Brasil baseline (2008-2010) were collected in 15 103 individuals. ^273^ Blood pressure was measured by the oscillometric method, and 35.8% of those individuals were classified as hypertensive, and 76.8% of them were on anti-hypertensive medication. Women were more aware than men (84.8% *versus* 75.8%) and more often on medication (83.1% *versus* 70.7%). Use of at least one anti-hypertensive drug was reported by 76.8% (n = 4147) of the participants classified as having hypertension and was also more frequent among women in all age groups. Of the users of anti-hypertensives, 69.4% showed controlled blood pressure levels (65.5% of the men and 72.9% of the women). Considering all the hypertensive individuals, about 53% showed appropriate blood pressure levels. Among those on drug treatment, controlled blood pressure was more likely in the higher educated group than among participants with less than secondary school education (PR 1.21; 95% CI, 1.14 - 1.28), and among Asian (PR 1.21; 95% CI, 1.12 - 1.32) and Whites (PR 1.19; 95% CI, 1.12 -1.26) as compared to Blacks.

• In a cross-sectional population-based study, carried out through a household survey and randomized cluster sampling, from the Health Status of the Elderly Population of the Municipality of Goiânia, state of Goiás, investigating 912 non-institutionalized elderly (≥ 60 years) living in urban areas, Sousa *et al* . reported a 74.9% prevalence of hypertension, higher (78.6%) among men (OR 1.4, 95% CI, 1.04 - 1.92). The treatment rate was 72.6%, with higher rates observed among smokers (OR 2.06, 95% CI, 1.28 - 3.33). The rate of hypertension control was 50.8%, higher among women (OR 1.57, 95% CI, 1.19 - 2.08). ^289^

• In a cross-sectional study investigating 502 users of the Family Health Strategy to control hypertension, Rocha *et al* . compared three different instruments to measure patient’s adherence to hypertension treatment (the Morisky and Green test, the Qualiaids team’s Medication Adherence Questionnaire, and the Haynes’ Questionnaire). The authors reported the following non-adherence prevalence rates: 29.28%, measured by the Morisky and Green test; 60.16%, by the Qualiaids Questionnaire; and 13.15%, by the Haynes questionnaire. This indicates room for improvement even in a structured program such as the Family Health Strategy. Despite the great variability in the adherence rates depending on the instrument used to measure it, the authors noticed a significant association of adherence to treatment and blood pressure control. ^290^

• To assess the evolution of hypertension prevalence, awareness, and control for over 10 years in the state of Pernambuco, two cross-sectional studies were conducted based on random samples of urban and rural households in 2006 and 2015/2016, involving adults aged 20 years or older. Approximately one third of the adult population of Pernambuco had hypertension in 2006 and this prevalence was maintained in 2015/2016. In rural areas, awareness concerning hypertension rose from 44.8% in 2006 to 67.3% in 2015/2016, and control, from 5.3% to 27.1%, so that awareness and control were similar in urban and rural areas in 2015/2016. A logistic regression analysis was conducted to estimate the influence of the social, behavioral, and anthropometric determinants on hypertension. Although social and behavioral factors improved in the 10-year period, overweight and abdominal obesity increased. After an adjustment for potential confounding factors, the likelihood of having hypertension more than doubled among men (OR 2.03; p < 0.001), young adults (OR 4.41; p < 0.001), the elderly (OR 14.44; p < 0.001), and those with abdominal obesity (OR 2.04; p < 0.001) in urban areas, as well as among young adults (OR 2.56; p < 0.001), and less educated (OR 2.21; p = 0.006) and overweight individuals (OR 2.23; p < 0.001) in rural areas. ^291^

• In a prospective study, Krieger *et al* . investigated the prevalence of resistant hypertension in a cohort of 1597 patients with stage 2 hypertension submitted to a step drug protocol. From 1597 patients recruited, 187 (11.7%) fulfilled the resistant hypertension criteria, defined as the lack of hypertension control evaluated by office blood pressure ≥140/90 mm Hg and mean 24-hour ambulatory blood pressure monitoring ≥ 130/80 mm Hg despite treatment with three drugs (enalapril or losartan, amlodipine and chlorthalidone) for 12 weeks. In addition, investigating the clinical predictors of hypertension, they noticed that history of stroke, diabetes mellitus, and office blood pressure ≥ 180/110 mm Hg at study entry were independently associated with resistant hypertension. ^292^

• In the HealthRise, a program of community-based interventions for the detection and management of hypertension and diabetes in underserved communities implemented in 2017-2018, more patients met treatment targets for hypertension [45.9% (43.0%–48.9%)] at endline than at baseline [35.4% (32.6%–38.6%), p<0.001], in the city of Vitória da Conquista, Bahia state. In the other city included in the project, Teófilo Otoni, Minas Gerais state, more patients met hypertension treatment targets at endline [52.2% (49.3–55%)] as compared to baseline [48.3% (45.5–51.2%); p<0.05], suggesting that these community-based interventions have the potential to improve hypertension control. ^293^

### Risk Factors and Prevention

• Treff CA Jr, in his dissertation, investigated the relationship between hypertension and physical activity levels according to the ‘leisure’ and ‘transportation’ domains of the IPAQ questionnaire. By using the databank of the ELSA-Brasil study, that author observed that leisure-time physically active individuals had lower SBP (p=0.007) and DBP (p=0.001) levels. On the other hand, no relationship was identified between hypertension and the levels of physical activity performed as a form of transportation. ^294^

• Investigating the potential role of diet leading to blood pressure elevation and expression of other cardiovascular risk factors, Pavan *et al* . studied 1110 subjects aged 22–89 years divided into three matched groups by sex and age (370 from Tanzania and Uganda, 370 from the Amazonian region of Brazil, and 370 from northern Italy; 111 men and 259 women in each group). The SBP of Africans eating a low-salt ‘fish and vegetable’ diet was lower than that of Brazilians, whose diet was based on cereals and meat, and that of highly urbanized Italians (144.1±21.9, 155.4±26.8, and 159.7±22.9 mm Hg, respectively, < 0.0001). The same occurred for DBP (83.2±11.8, 94.5±15.5, 94.7±11.6 mm Hg, respectively, < 0.0001). The SBP was correlated to the body mass index of all three populations, but with age only for the Brazilians and Italians. Total cholesterol level and body mass index, both of which are low among Africans, increased progressively with increasing economic level. Transition from a rural to an urbanized lifestyle seems to be accompanied by a rise in rates of cardiovascular risk factors; in addition, environmental, rather than racial factors, have a crucial impact on the risk pattern of populations. ^295^

• In a descriptive, observational, cross-sectional population-based study, Jardim *et al* . evaluated 1739 individuals and observed that hypertension was prevalent in 36.4% of the total population, and higher among males (41.8%) than females (31.8%). Hypertension was positively correlated to age ≥60 years *versus* age of 18-29 years (OR 8.92, 5.94 – 14.11; P<0.000), male gender (OR 1.86, 1.47–2.35; P<0.000), and body mass index (OR 1.44, 1.13 – 1.83; P=0.004). Prevalences of overweight and obesity were 30.0% and 13.6%, respectively. Overweight was higher among females and obesity among males. The prevalence of smoking was 20.1%, higher among males (27.1%) as compared to females (16.4%). A sedentary lifestyle was observed in 62.3% of the population, with no difference between genders. Regular alcohol consumption was reported by 44.4% of the individuals, being more frequent among males. ^296^

• Using data from the VIGITEL survey, Moreira *et al* . tried to identify and measure the relationships of sociodemographic and behavioral characteristics, food consumption characteristics, and health indicators related to hypertension and diabetes. The independent variables analysed in the study were selected based on their importance for determining the total burden of disease, as estimated by the World Health Organization for the Americas region. ^297^ The adjusted analysis relating to the women’s data showed that age ≥ 65 years (PR 1.3; 1.0-1.7), excessive body weight (PR 1.7; 1.3-2.2), self-rated poor health (PR 1.3; 1.0-1.8), and a previous medical diagnosis of dyslipidemia (PR 1.5; 1.2-1.8) remained independently associated with higher prevalence of hypertension. Consuming whole-fat milk remained associated with lower prevalence of hypertension in women (PR 0.7; 0.6-0.9). In the adjusted analysis for men, overweight (PR 1.7; 1.1-2.5) and self-rated poor health were independently associated with hypertension (PR 1.9; 1.4-2.5).

• Investigating whether blood pressure response to salt intake would be sex-specific, Mill *et al* . studied the changes in blood pressure according to different salt intake by men and women in 12 813 individuals with a validated 12-hour overnight urine collection in which salt intake was estimated. A set of questionnaires, clinical examination, and laboratory tests were carried out during a single visit. Salt intake was 12.9 ± 5.9 g/d among men and 9.3 ± 4.3 g/d among women. As expected, the authors concluded that blood pressure increased as salt intake increased, regardless of using blood pressure-lowering medication. Nevertheless, the slope of increase in blood pressure elicited by salt intake was significantly higher in women than in men. They concluded that salt intake was elevated in this large sample of Brazilian adults and only a few participants were compliant with the guidelines’ recommendations. Moreover, the higher responsiveness to salt intake observed in women as compared to men, even after controlling for confounders, indicates higher salt sensitivity and may have pathophysiological implications. ^298^

### Children and Adolescents

• Hypertension, obesity, poor diet, and physical inactivity in childhood and adolescence are an emerging epidemiological concern. Moreover, early identification and intervention may prevent premature CVD in adult life.

• To estimate the prevalence of hypertension and obesity and the role of obesity in hypertension in Brazilian adolescents, data from participants in the ERICA Study were evaluated by Bloch *et al* . Prevalence and 95% CI of arterial hypertension and obesity, both on a national basis and in the Brazilian regions, were estimated by sex and age groups, as was the proportion of hypertension due to obesity in the population. The study assessed 73 399 students, 55.4% female, mean age of 14.7 ± 1.6 years. The prevalence of hypertension was 9.6% (95% CI, 9.0 - 10.3), the lowest being in the Northern (8.4%; 95% CI, 7.7 - 9.2) and Northeastern (8.4%; 95% CI, 7.6 - 9.2) regions, and the highest in the Southern region (12.5%; 95% CI, 11.0 - 14.2). The mean prevalence of obesity was 8.4% (95% CI, 7.9 - 8.9), which was lower in the Northern region and higher in the Southern region. The prevalences of hypertension and obesity were higher among males. Obese adolescents presented a higher prevalence of hypertension, 28.4% (95% CI, 25.5 - 31.2), than overweight adolescents, 15.4% (95% CI, 17.0 - 13.8), or eutrophic adolescents, 6.3% (95% CI, 5.6 - 7.0), as commonly noticed. The proportion of hypertension due to obesity was estimated at 17.8%. The authors concluded that the control of obesity could lower the prevalence of hypertension among Brazilian adolescents by approximately 20%. ^299^

• Christofaro *et al* . analysed the relationship between hypertension of adolescents and their parents’ sociodemographic characteristics and lifestyle. For this task, 1231 adolescents, 1202 mothers, and 871 fathers were investigated. The prevalence of hypertension was higher among adolescents with older parents, with both parents reporting hypertension, and with overweight mothers. In multivariate analysis, adolescents with older mothers (OR 2.36; 95% CI, 1.12 - 4.98), hypertensive mothers (OR 2.22; 95% CI, 1.26 - 3.89), and hypertensive fathers (OR 1.70; 95% CI, 1.03 - 2.81) were more likely to have hypertension. In the analysis that considered clusters of health risk factors, higher risks of hypertension were observed in adolescents whose mothers had four or more aggregated risk factors (OR 2.53; 95% CI, 1.11 - 5.74). ^300^

• To estimate the presence of cardiovascular risk (obesity and hypertension) in schoolchildren and its potential interactions with cardiorespiratory fitness, Burgos *et al* . performed a cross-sectional study conducted in 1666 schoolchildren, aged 7-17 years, 873 (52.4%) of them males. The following variables were evaluated: SBP, DBP, body mass index, body fat percentage, and cardiorespiratory fitness. Systolic blood pressure and DBP were correlated with waist circumference, waist-hip ratio, sum of skin folds, and cardiorespiratory fitness. The authors reported that 26.7% of them were overweight or obese, and 35.9% had body fat percentage over moderately high. They also found that 13.9% and 12.1% of the students were borderline or hypertensive, for SBP and DBP, respectively. There was a significant correlation of SBP and DBP with all variables, and a weak to moderate correlation with age, weight, height, body mass index, and waist circumference. These data indicate a cluster of hypertension, obesity, and lack of cardiorespiratory fitness in early life and must elicit the development of effective prevention programs to reduce CVD in adulthood. ^301^

• Schommer *et al* . investigated the association between anthropometric variables and blood pressure levels in schoolchildren from 5th to 8th grade to identify which parameter more strongly correlated with blood pressure levels. Using a cross-sectional study with probabilistic population-based cluster sampling of schoolchildren, they enrolled schoolchildren from 5th to 8th grade in public elementary schools of the city of Porto Alegre. The participants’ mean age was 12.6 ± 1.6 years, and 55.2% of them were females. Abnormal blood pressure levels were found in 11.3% of the sample and borderline values, in 16.2%. Of the anthropometric variables analyzed, hip circumference showed the strongest correlation with increased blood pressure (r = 0.462, p < 0.001), followed by waist circumference (r = 0.404, p < 0.001) and abdominal skinfold (r = 0.291, p < 0.001). ^302^

### Future Research

• Because of its prevalence and impact, hypertension is the leading cardiovascular risk factor for disability and death around the globe and in Brazil. Despite this current knowledge, more representative and comprehensive data is lacking to better quantify the current incidence, the trends of hypertension, the life course trajectories and their determinants, and the morbidity and mortality related to hypertension, stratified by regions, sex, age, and socioeconomic status in Brazil.

• Planning to decrease cardiovascular burden in Brazil, there is a huge gap regarding a deeper and more integrated knowledge about how to improve hypertension prevention, awareness, treatment, and control, as well as its relationship with other cardiovascular unfavorable behaviors and risk factors, such as proposed by the American Heart Association with the seven metrics to measure cardiovascular health at the population level. ^303^ The current understanding points out to the global cardiovascular risk, at the population level, as the main determinant of cardiovascular morbidity and mortality nationwide. Therefore, better data on outcomes and health service research to measure population end-results and healthcare system performance, and on implementation science investigating strategies of how to improve these outcomes are urgently needed in Brazil. ^304^

• Moreover, we must move on from only generating evidence to a model of continuously translating the evidence into good health care policies. ^305^ National population strategies with effective campaigns to promote healthy habits (i.e.: decrease salt in diet, taxation of unhealthy foods, increase in physical activity), allied to more effective identification and treatment of the individuals at greater cardiovascular risk, and objective surveillance of results must be more disseminated to all levels of our healthcare system.

• Another theme that deserves better research is related to disparities regarding access, timelines, and outcomes of hypertensive patients using the SUS as compared to patients using private healthcare services. ^96^ Considering that more than three-quarters of Brazilians are SUS users, it is imperative to measure continuously the outcomes of hypertension programs implemented by the SUS, such as the Family Health Strategy, and to compare to those reached by the private healthcare system.

## 8. DIABETES MELLITUS

### ICD-10 E10 to E14; ICD-10-CM E8 to E13

**See Tables 8-1 to 8-5 and Charts 8-1 through 8-4**

**Table:** Abbreviations used in Chapter 8

BMI	Body Mass Index
CI	Confidence Interval
CVD	Cardiovascular Diseases
DALYs	Disability-Adjusted Life Years
ELSA-Brazil	Longitudinal Study of Adult Health - Brazil
ERICA	Brazilian Study of Cardiovascular Risk in Adolescents (in Portuguese, *Estudo dos Riscos Cardiovasculares em Adolescentes* )
FU	Federative Unit
GBD	Global Burden of Disease
HbA1c	Glycosylated Hemoglobin
HDL	High Density Lipoprotein
HR	Hazard Ratio
IBGE	Brazilian Institute of Geography and Statistics (in Portuguese, *Instituto Brasileiro de Geografia e Estatística* )
ICD-10	International Statistical Classification of Diseases and Related Health Problems, 10^th^Revision
IDF	International Diabetes Federation
LDL	Low Density Lipoprotein
MASS	The Medicine, Angioplasty or Surgery Registry (in Portuguese, *Registro de Medicina, Angioplastia ou Cirurgia* )
OR	Odds Ratio
PNAUM	Survey on the Access, Utilization and Promotion of Rational Use of Medications in Brazil (in Portuguese, *Pesquisa Nacional sobre o Acesso, Utilização e Promoção do Uso Racional de Medicamentos no Brasi* l)
PNS	National Health Survey (in Portuguese, *Pesquisa Nacional de Saúde* )
PR	Prevalence Ratio
REACT	Registry of Clinical Practice in Patients at High Cardiovascular Risk (in Portuguese, *Registro do Paciente de Alto Risco Cardiovascular na Prática Clínica* )
SBC	Brazilian Society of Cardiology (in Portuguese, *Sociedade Brasileira de Cardiologia* )
SDI	Sociodemographic Index
SF-36	Short Form 36 quality of life questionnaire
SIM	Brazilian Mortality Information System (in Portuguese, *Sistema de Informações sobre Mortalidade* )
SUS	Brazilian Unified Health System (in Portuguese, *Sistema Único de Saúde* )
UI	Uncertainty Interval
VIGITEL	Surveillance System of Risk and Protection Factors for Chronic Diseases by Telephone Survey (in Portuguese, *Sistema de Vigilância de Fatores de Risco e Proteção para Doenças Crônicas por Inquérito Telefônico* )
YLDs	Years Lived with Disability
YLLs	Years of Life Lost

**Table 8-1:** – Prevalence of diabetes mellitus (glycosylated hemoglobin ≥ 6.5% or use of antidiabetic drugs): total, according to sex and Brazilian regions.

Variables	%	95% CI
Total	8.4	7.6 – 9.1
**Sex**		
Female	9.7	8.6-10.7
Male	6.9	5.9-7.9
**Regions**		
North	6.3	5.3 – 7.3
Northeast	7.6	6.7 – 8.6
Southeast	9.3	7.9 – 10.7
South	7.4	5.9 – 8.9
West-Central	9.4	7.6 – 11.2


*Source: Malta et al. ^345^*

**Table 8-2:** – Prevalence of diabetes mellitus (glycosylated hemoglobin ≥ 6.5% or use of antidiabetic drugs) according to age range and sociodemographic characteristics.

Variables	%	95% CI
**Age range (years)**		
18-29	1.5	0.7 – 2.2
30-44	3.5	2.5 – 4.4
45-59	12.6	10.9 – 14.3
≥ 60	20.6	18.2 – 22.9
**Schooling**		
None	12.3	11.0 – 13.7
Elementary	7.4	5.6 – 9.2
Complete middle-school	5.3	4.4 – 6.3
Race/skin color		
White	8.4	7.3 – 9.6
Black	10.3	7.5 – 13.0
Mixed	7.9	6.9 – 8.9
Other	7.7	3.3 – 12.1
**Body mass index**		
Under/normal weight	4.0	3.3 – 4.8
Overweight	8.5	7.3 – 9.8
Obesity	16.9	14.7 – 19.0

Source: Malta *et al* . ^345^

**Table 8-3:** – Number of deaths and age-standardized mortality rate (per 100 000) from cardiovascular disease attributable to diabetes for both sexes, in Brazil and its Federative Units, 1990 and 2019.

Location	1990		2019		Percent change (95% UI)
	Number (95% UI)	Rate (95% UI)	Number (95% UI)	Rate (95% UI)	
Acre	64.6 (43.5;94.8)	57.1 (36;92.4)	195.1 (133.9;284.1)	38.3 (25.4;58.4)	-32.9 (-43.8;-16.9)
Alagoas	864.2 (581.3;1270.1)	75.2 (48.9;113)	1708.7 (1203.3;2458.4)	56.7 (39.4;82)	-24.6 (-38.9;-5.4)
Amapá	31.6 (20.7;48.7)	43.9 (27.1;71)	141.6 (94.9;204.8)	33.2 (21.7;49.5)	-24.3 (-39.9;-1.8)
Amazonas	315.8 (206.7;490.7)	58.6 (36.7;96.6)	824.9 (548.9;1222.8)	33 (21.6;49.5)	-43.6 (-54.8;-26.8)
Bahia	2955.3 (1921.1;4621.9)	49.6 (31.6;78.7)	6101.2 (4096.4;9411.7)	38.5 (25.7;59.2)	-22.4 (-41.5;4.7)
Ceará	1422.3 (869.1;2289.7)	37.7 (22.9;62.1)	3981.1 (2517.4;5969.7)	41.1 (25.8;62)	8.9 (-18.9;47.8)
Distrito Federal	327.1 (232.6;446.1)	105.4 (70.3;160.3)	710.8 (488.7;1014.2)	40 (26;62.3)	-62 (-70.9;-50.5)
Espírito Santo	759.3 (500.7;1136.6)	68.2 (43.5;110.6)	1536.2 (1024.7;2294.8)	38 (25.1;57.9)	-44.3 (-56.7;-27.9)
Goiás	990.7 (650.4;1477.2)	65.5 (41.1;104.7)	2109.1 (1393.4;3211.1)	33.3 (21.5;51.5)	-49.1 (-61.8;-30.4)
Maranhão	1388.1 (920.9;2107.1)	62.3 (40.6;96)	3568.4 (2352.1;5390)	56.8 (37.2;86)	-8.9 (-30.1;17.6)
Mato Grosso	319.9 (210.4;476)	59.6 (37.3;92.5)	951.3 (626.1;1401.9)	32.9 (21.2;49.4)	-44.7 (-56.6;-27.6)
Mato Grosso do Sul	489.1 (334.7;696.2)	75.2 49;114.6)	1050.2 (708.3;1526.9)	38.9 (25.8;57.4)	-48.2 (-58.6;-33.4)
Minas Gerais	4033.5 (2792.7;5941.5)	52.2 (34.4;79.9)	6101.6 (3909.3;9406)	23.3 (14.9;35.9)	-55.3 (-65.7;-41.7)
Pará	1048.9 (680.6;1613.8)	68.1 (42.1;109.9)	2461.9 (1641.9;3553.9)	39 (25.6;57.2)	-42.7 (-54.7;-23.9)
Paraíba	1208.7 (785.1;1848.2)	55 (35.5;85.2)	2106.7 (1395;3164.2)	43.4 (28.9;65)	-21 (-36.7;0.4)
Paraná	2811 (1846.6;4153.8)	79.2 (49.4;126.7)	4756.2 (3146.9;7007.4)	38.6 (25.2;58.5)	-51.3 (-61.1;-36.6)
Pernambuco	2973.6 (1992.5;4385.2)	76.4 (49.9;115.9)	5047.3 (3437.1;7374.5)	53.3 (35.7;78.6)	-30.2 (-42.6;-12.1)
Piaui	655.7 (425.9;1025.4)	59.5 (36.7;96.6)	1481.3 (951.6;2261.5)	39 (25.1;59.3)	-34.5 (-48.3;-12.3)
Rio de Janeiro	7914.2 (5433.6;11183.9)	98.7 (65;142.3)	8936.3 (6041.8;13238.2)	40.6 (27.3;60.1)	-58.9 (-66.3;-49.1)
Rio Grande do North	785.8 (515.7;1213.6)	52.5 (33.6;82.2)	1402.4 (894.9;2075.7)	36.1 (23;53.7)	-31.1 (-44.9;-12.2)
Rio Grande do Sul	3206.9 (2141.9;4941.3)	61.2 (39.5;97.9)	4779.8 (3012.6;7502.5)	31 (19.4;49.1)	-49.3 (-61.2;-34)
Rondônia	187.3 (122.5;266.3)	114.4 (73.6;177.2)	547.6 (363.6;815.8)	41.3 (27.1;62.4)	-63.9 (-70.7;-55.7)
Roraima	28.9 (20.2;40.9)	90.6 (59.2;137.9)	116.6 (82.4;166.8)	42.3 (28.1;64.5)	-53.3 (-59.3;-46.1)
Santa Catarina	1462.2 (975.1;2147.5)	78.1 (49.8;119.2)	2392.6 (1603.5;3597.3)	32.9 (21.3;51.3)	-57.9 (-66.4;-46.9)
São Paulo	13914.2 (9600.8;19624.5)	93.2 (59.9;141.8)	16291.4 (10698.7;24578.1)	31.9 (20.5;49.5)	-65.7 (-72.4;-57.6)
Sergipe	483.5 (334;711.7)	76.9 (50.1;119.9)	904.8 (609.4;1323.8)	42.9 (28.8;63.6)	-44.2 (-54.7;-29.4)
Tocantins	169.8 (114.4;250.5)	68.7 (42.5;112.8)	549 (355.4;810.2)	42 (26.6;63.4)	-38.9 (-53.3;-18.9)
Brazil	50812.4 (35649.3;73136.9)	70.4 (47.4;106.1)	80754.1 (55922.4;118175.4)	35.9 (24.5;53)	-49 (-53.4;-43.9)

*Source: Data derived from Global Burden of Disease Study 2019, Institute for Health Metrics and Evaluation, University of Washington. ^46^*

**Table 8-4:** – Age-standardized mortality rate (per 100 000) from cardiovascular disease attributable to diabetes, for men and women, in Brazil and its Federative Units, 1990 and 2019.

Location	Women			Men		
	1990	2019	Percent change (95% UI)	1990	2019	Percent change (95% UI)
Acre	42.7 (25.5;69.4)	27.8 (17.1;44.3)	-35 (-51.6;-9.2)	76.5 (46.8;125.2)	51.6 (33.8;79.5)	-32.5 (-45.7;-13.3)
Alagoas	65.3 (40.5;102.6)	48.6 (31.7;71.5)	-25.5 (-44.5;3.8)	86.6 (54.3;132.5)	67 (44.9;96.2)	-22.7 (-41.8;6.9)
Amapá	33.9 (19.5;56.2)	23.3 (14.2;37)	-31.3 (-50.9;-2.4)	56.1 (33.4;90.8)	45 (28.8;66.6)	-19.8 (-39.3;12.5)
Amazonas	54.2 (32.8;92.4)	24.8 (15.2;39)	-54.2 (-67.5;-35.7)	62.9 (37.9;103.7)	42 (27.6;62.6)	-33.3 (-49.6;-3.2)
Bahia	43.9 (26.7;70)	26.4 (16.4;42.5)	-40 (-59;-12.2)	56.2 (33.6;95.8)	54.6 (34.4;84.2)	-2.9 (-34.1;51.1)
Ceará	31 (17.4;54.3)	32.2 (18.4;51.1)	4 (-29.2;58.8)	45.6 (25.9;77.9)	52.5 (32.6;80.4)	15.1 (-22.2;77.8)
Distrito Federal	91 (56.3;144.1)	33.8 (20.3;54.2)	-62.9 (-74.3;-48)	137.1 (88.8;210.4)	49.3 (31.2;79.1)	-64 (-73.6;-51.5)
Espírito Santo	58.2 (35;96.8)	28.7 (18;44.9)	-50.7 (-65.1;-28.8)	79.3 (48.3;127.4)	49.8 (31.9;76.7)	-37.1 (-54.7;-13)
Goiás	57.8 (34.7;94.5)	25.9 (15.6;42.7)	-55.1 (-69.7;-31.8)	75.1 (45.4;126.3)	41.8 (25.7;65.1)	-44.3 (-61.8;-16.1)
Maranhão	30 (17.7;49.5)	36.5 (22.2;58)	21.8 (-19;82.1)	123.1 (77.8;194.1)	85.5 (54.6;132.5)	-30.6 (-48;-5.9)
Mato Grosso	53.2 (31.6;84.4)	27 (16.7;42.2)	-49.2 (-64.7;-27.4)	65.1 (37.4;107)	38.7 (24.2;58.8)	-40.5 (-57.6;-12.9)
Mato Grosso do Sul	66.5 (41.6;105.4)	31.1 (19.7;47.9)	-53.2 (-66.4;-34.4)	83.3 (52.6;126.6)	47.8 (30.4;71.3)	-42.6 (-57.2;-22.3)
Minas Gerais	44.7 (28.8;73.4)	18.2 (11.3;29.9)	-59.3 (-72.3;-42)	61.1 (40.5;97.5)	29.6 (17.6;47.8)	-51.6 (-66.4;-30.7)
Pará	59.1 (34;98.7)	28.5 (17.6;44.5)	-51.7 (-65.3;-27.8)	78.4 (45.6;129.7)	50.6 (32.9;76)	-35.4 (-52.2;-7.8)
Paraíba	51.7 (31.8;81.4)	35.5 (21.8;54.7)	-31.3 (-50.2;-5.1)	58.6 (36.9;91.6)	53.5 (35.4;80.3)	-8.7 (-31.2;25.2)
Paraná	67.8 (40.3;113.2)	29.8 (18.2;47.9)	-56 (-68.8;-36.7)	91.1 (54.7;146.6)	49.3 (31.5;75)	-45.9 (-59;-22.2)
Pernambuco	70 (44.3;108.9)	42.1 (26.9;63.4)	-39.9 (-54;-21.3)	84 (52.6;127.2)	68.6 (45.5;101.7)	-18.4 (-38.8;12.1)
Piaui	43.6 (25.9;74.3)	30.1 (18.5;48.5)	-31 (-50;-2.3)	79.6 (47.9;136.7)	49.8 (31.8;77.7)	-37.4 (-53.4;-12.4)
Rio de Janeiro	75.1 (45.5;115.2)	28.9 (17.7;45.2)	-61.6 (-72.4;-46.8)	133.4 (90.5;193.4)	57.3 (38.8;85.3)	-57 (-65.4;-45.8)
Rio Grande do North	41.4 (25.9;65.1)	27.1 (16.4;42.4)	-34.4 (-53.4;-8.6)	65.4 (40.9;104.5)	47.8 (29;72.4)	-26.9 (-46.5;2.8)
Rio Grande do Sul	51.5 (30.9;85)	25 (14.7;41.7)	-51.4 (-66.2;-28.2)	74.2 (46.6;120.3)	38.7 (23.8;62.6)	-47.9 (-63.9;-23.2)
Rondônia	115.4 (73.3;180.4)	32.5 (19.9;50.4)	-71.8 (-79.7;-62.9)	112.4 (69.3;172.3)	50 (32.3;75.3)	-55.5 (-66.3;-42.3)
Roraima	74 (47.5;115.5)	34.2 (22.1;53.7)	-53.8 (-61.4;-44.2)	104.5 (65.8;159.2)	49.8 (32.7;75.5)	-52.3 (-59.9;-42.5)
Santa Catarina	69.3 (42.1;112.3)	25.7 (15.4;42.3)	-62.9 (-73.5;-47.7)	88.3 (54.6;139.3)	41.8 (26.5;63.9)	-52.7 (-64.8;-34.7)
São Paulo	75.9 (46.7;120.5)	24.1 (14.7;40.3)	-68.2 (-77.2;-55.4)	115 (75.2;168.4)	42.2 (25.8;64.6)	-63.3 (-72;-53)
Sergipe	67.2 (42.4;106.5)	35.6 (22.4;55.6)	-47 (-61.7;-28.1)	89.2 (56.6;139.4)	52.3 (34.3;78.7)	-41.4 (-54.9;-20.5)
Tocantins	61.7 (38.1;101.7)	31.1 (19.5;48.5)	-49.6 (-63.3;-29.6)	76.7 (44.9;130.4)	55.1 (33.7;86.8)	-28.1 (-50.2;4)
Brazil	58.1 (38.4;88.3)	27.2 (18;41.6)	-53.2 (-59.3;-46.1)	85.4 (58.1;128.3)	47.1 (32.1;68.6)	-44.9 (-50.4;-37.8)

*Source: Data derived from Global Burden of Disease Study 2019, Institute for Health Metrics and Evaluation, University of Washington. ^46^*

**Table 8-5:** – Number of DALYs and age-standardized DALY rates (per 100 000) from cardiovascular disease attributable to diabetes for both sexes, in Brazil and its Federative Units, 1990 and 2019.

Location	1990		2019		Percent change (95% UI)
	Number (95% UI)	Rate (95% UI)	Number (95% UI)	Rate (95% UI)	
Acre	1335.2 (912.8;1888.1)	941.8 (633.3;1371)	3934.6 (2782.2;5669)	683.4 (475.1;986)	-27.4 (-38.8;-10)
Alagoas	17722.1 (12411.8;24790.4)	1387.3 (965.4;1978.1)	35556 (25585.2;48957.8)	1134.8 (819.4;1566.4)	-18.2 (-34.2;3.8)
Amapá	630.7 (433.5;914.9)	723.1 (475.3;1095)	3022.1 (2113.7;4226.6)	623.3 (427.4;894.6)	-13.8 (-31.8;11.6)
Amazonas	6428.5 (4391;9409.2)	965.9 (641.4;1472.8)	16437.5 (11420;23668.2)	608.2 (420.8;881.9)	-37 (-50.4;-17.8)
Bahia	60323 (41070.5;88143.3)	926.4 (622.4;1396.6)	120282.6 (83918;176491.9)	757.4 (525.9;1119.6)	-18.2 (-39.5;10.1)
Ceará	26051.4 (16968.5;39656.1)	662.4 (424.8;1021.6)	71929.9 (49135.3;103619.2)	733.5 (497.6;1060.9)	10.7 (-18.6;51.3)
Distrito Federal	8468 (5993.5;11589.3)	1726.1 (1219.3;2443.4)	14639.6 (10394.9;20195.3)	632 (440.7;895.3)	-63.4 (-71.5;-52.8)
Espírito Santo	15740.9 (11037.6;22572.5)	1165.3 (797.7;1722)	29886.8 (21156.3;42438)	697.7 (488.3;1000.8)	-40.1 (-53.1;-22.7)
Goiás	22199.6 (15278.2;32417.9)	1165 (776.4;1711.4)	43263.8 (29225.9;64727.4)	632.8 (428.1;953.8)	-45.7 (-59.9;-24.9)
Maranhão	30853.4 (20729.6;44978.9)	1241.5 (845.8;1830.5)	68520.9 (47179.2;99823.3)	1062.6 (731.1;1556.5)	-14.4 (-35.9;13.9)
Mato Grosso	7137.7 (4910.7;10348.6)	1035.9 (688.5;1536.7)	19808.7 (13578.4;28204)	622.3 (424.8;897)	-39.9 (-53.1;-20.8)
Mato Grosso do Sul	10808.6 (7735.6;15042.3)	1319.5 (913.6;1871.1)	20964.7 (14543.3;29539.1)	726 (504.9;1024.7)	-45 (-56.3;-29.1)
Minas Gerais	90445.6 (65437.6;127258.5)	961.2 (684.3;1390.3)	119023.5 (80740.1;174655.1)	450.2 (303.6;661.3)	-53.2 (-63.3;-39.1)
Pará	20697.3 (14113;30025.6)	1124.9 (747.8;1683)	49334.3 (34295.7;68456.6)	739.9 (504;1037.5)	-34.2 (-48.3;-14.2)
Paraíba	22665.6 (15429.1;32837.2)	993.8 (674.6;1439.3)	39309.5 (27360.3;56659.5)	837 (582.8;1205.1)	-15.8 (-34.2;10)
Paraná	58887.3 (40717.4;84598.3)	1354.2 (894.9;2009.2)	91772.5 (62792.3;129456.7)	699.4 (477.5;991.9)	-48.4 (-58.9;-32.9)
Pernambuco	59380 (41231.5;83117.2)	1366 (942.4;1923.6)	100595.8 (70745.7;143053.9)	1017.4 (711.7;1452.2)	-25.5 (-39.4;-6.5)
Piaui	12857.4 (8842.4;19094.9)	998.5 (673.1;1525.2)	27097.6 (18663.2;39307.5)	724.6 (499.2;1055.2)	-27.4 (-43.3;-3.5)
Rio de Janeiro	176130.1 (123280.1;244011.1)	1888.9 (1331.8;2618.9)	178623.1 (126398.8;254598.7)	790 (561;1127)	-58.2 (-66.3;-47.6)
Rio Grande do North	14427.4 (9841.6;21299.8)	924.2 (630.3;1361.8)	26480.8 (17460.1;37536.8)	693.6 (455.8;984.5)	-25 (-41.7;-2.5)
Rio Grande do Sul	65930.3 (46174.7;94957.8)	1075 (740.8;1584.9)	87421.8 (58608.1;129018)	558.7 (375.7;819)	-48 (-59.9;-32.9)
Rondônia	4693.9 (3130.2;6604.9)	1850.9 (1256.7;2680.8)	11161.5 (7688.3;16108.6)	761.7 (520.9;1110)	-58.8 (-67.1;-48.3)
Roraima	701.7 (486.9;981.2)	1510.3 (1042.4;2179.8)	2550 (1822.3;3524.3)	737.4 (521.9;1032.2)	-51.2 (-58.6;-41.4)
Santa Catarina	29444.9 (20595.6;41977.2)	1315.9 (886.4;1907.9)	45537.1 (31491;64303.7)	577.1 (396.9;820.5)	-56.1 (-65.4;-44.1)
São Paulo	295196.3 (209790.2;409567)	1609.4 (1116.3;2263.4)	315407.1 (218126;449199.4)	586.5 (403.7;842.8)	-63.6 (-71.1;-55.1)
Sergipe	9507.4 (6819.9;13407.5)	1308.1 (916.3;1874.8)	17955.5 (12531.5;25018.5)	821.4 (570.1;1153.4)	-37.2 (-49.4;-21)
Tocantins	3644.4 (2521.7;5217.6)	1085.4 (724.5;1612.6)	10599.2 (7112.5;14982.1)	766.2 (510;1096.3)	-29.4 (-46.1;-5.5)
Brazil	1072308.9 (784276.4;1484959.3)	1279.7 (922.2;1800.5)	1571116.4 (1140912.3;2203187.8)	673.5 (485.1;947.7)	-47.4 (-52.2;-41.9)

*Source: Data derived from Global Burden of Disease Study 2019, Institute for Health Metrics and Evaluation, University of Washington. ^46^*

**Chart 8-1:** – Total number of deaths due to diabetes (A). From cardiovascular disease attributable to diabetes: number of deaths (B), age-standardized mortality rate (per 100 000) (C), and DALY rate (D). Brazil, 2019.

Source: Data derived from Global Burden of Disease Study 2019, Institute for Health Metrics and Evaluation, University of Washington. ^46^

**Chart 8-2:** – Proportional mortality from cardiovascular diseases attributable to diabetes according to sex and age group. Brazil, 2019

Source: Data derived from Global Burden of Disease Study 2019, Institute for Health Metrics and Evaluation, University of Washington. ^46^

**Chart 8-3:** – Deaths attributable to diabetes stratified by all causes and by cardiovascular diseases. Brazil, 2019.

Source: Data derived from Global Burden of Disease Study 2019, Institute for Health Metrics and Evaluation, University of Washington. ^46^

**Chart 8-4:** – Correlation between the sociodemographic index (SDI) of 2019 and the percent change in deaths from cardiovascular disease attributable to diabetes from 1990 to 2019, in Brazil and its Federative Units.

Source: Data derived from Global Burden of Disease Study 2019, Institute for Health Metrics and Evaluation, University of Washington. ^46^

### Introduction

• Diabetes mellitus is a chronic and progressive disease, characterized by metabolic changes due to persistent hyperglycemia resulting from defects in the secretion and/or action of the insulin produced by the pancreatic beta cells. In the long run, persistent hyperglycemia causes microvascular (retinopathy, nephropathy, neuropathy) and macrovascular (coronary artery disease, cerebrovascular disease, and peripheral artery occlusive disease) complications. Prediabetes (reduced glucose tolerance and impaired fasting plasma glucose) refers to the condition in which glycemia is elevated as compared to normal parameters, but without meeting the diagnostic criteria for diabetes. The identification of prediabetes is important because it increases the risk for diabetes and CVD; in addition, interventions related to lifestyle changes have proven to reduce the risk of diabetes in those individuals.

• The classification of diabetes is based on its etiology. The most prevalent types are type 2 and type 1 diabetes, representing 90-95% and 5-10% of the cases, respectively. Type 2 diabetes usually occurs in adults over the age of 30-40 years, has a complex and multifactorial etiology, involving polygenic inheritance and influence of familial and environmental factors – unhealthy food habits, physical inactivity, and sedentary lifestyle. Its pathophysiology involves insulin resistance and initial increase in insulin production, followed over the years by pancreatic failure to produce that hormone. ^307^

• Type 1 diabetes usually affects children, adolescents, or young adults, has an autoimmune, polygenic etiology resulting from the destruction of pancreatic beta cells and complete insulin deficiency. It can occur in adults (former LADA – latent autoimmune diabetes in adults, currently known as type 1 adult diabetes). It is subdivided into types 1A and 1B depending on the presence of autoantibodies. Autoantibodies are detected in type 1A diabetes, as well as a genetic predisposition associated with environmental factors that trigger the immune response (viral infections, components of the diet and of the intestinal microbiota). Type 1B diabetes is autoantibody-negative and its etiology is idiopathic. Cardiovascular disease is the major cause of death in patients with diabetes, who have twice the risk of major cardiovascular events as compared to the population without the disease, in addition to worse response to treatment and worse outcomes after a cardiovascular event (acute myocardial infarction, stroke). The classic cardiovascular risk factors [smoking, arterial hypertension, high levels of LDL-cholesterol, low levels of HDL-cholesterol] maximize that risk, the duration of diabetes, and the coexistence of microvascular complications. The cardiovascular complications of diabetes include coronary artery disease, cerebrovascular disease, and peripheral artery disease. ^308^ ^,^ ^309^

• Diabetes directly affects the quality of life, productivity, and survival, in addition to having an important economic impact on health systems. Meeting glycemic targets and control of other cardiovascular risk factors are known to contribute to prevent and delay the progression of the disease’s chronic complications. The systemic impairment of several organs and the disease’s complexity require global care for the patient, comprising several aspects of the treatment. ^309^

• In this chapter, diabetes mellitus is approached as a cardiovascular risk factor, as it has been identified since the classic Framingham study. ^308^ The presence of diabetes mellitus in association with smoking, systemic arterial hypertension, and dyslipidemia increases by two to three times the risk of CVD. ^310^

### Prevalence

• Teló *et al* ., in a systematic review with meta-analysis of Brazilian observational studies from 1980 to 2015, including 50 articles, have shown the increasing prevalence of diabetes in recent decades. The prevalence of diabetes was as follows: 5.6% (95% CI: 5.0 – 6.3) by self-report (36 studies); 6.6% (95% CI: 4.8 – 8.9) by fasting plasma glucose (7 studies); and 11.9% (95% CI: 7.7 – 17.8) by oral glucose tolerance test (7 studies). In trend analyses, increase in the prevalence of diabetes was observed over time, the biggest increase being observed in studies using the oral glucose tolerance test in the diagnosis: 7.4% (95% CI: 7.1 – 7.7) in the 1980s, 12.1% (95% CI: 10.5 – 13.8) in the 1990s, 14.5% (95% CI: 13.1 – 16.0) in the 2000s, and 15.7% (95% CI: 9.8 – 24.3) in the 2010s. ^311^

• Considering the IDF data published in 2019, Brazil ranked 5th regarding the number of adults with diabetes worldwide, totaling 16.8 million (95% CI: 15.0 – 18.7) people with that disease, 46% of whom did not know they had the disease. The prevalence of prediabetes was 9.5% (15.1 million people). ^312^

• Data from the PNS (2014 to 2015) have shown the following prevalence of diabetes according to different criteria: 6.6% (95% CI: 5.9 – 7.2) [HbA1c ≥ 6.5%]; 8.4% (95% CI: 7.6 – 9.1) [HbA1c ≥ 6.5% or use of antidiabetic drugs]; 9.4% (95% CI: 8.6 – 10.1) [HbA1c ≥ 6.5% or history of diabetes], and 7.5% (95% CI: 6.7 – 8.2) [history of diabetes]. That information is presented according to sex, Brazilian geographical regions (Table 8-1), age range, educational level, self-reported skin color, and BMI (Table 8-2), considering the most comprehensive criterion (HbA1c ≥ 6.5% or use of antidiabetic drugs). For all criteria, the prevalence was higher in women, individuals aged over 30 years, and among those with overweight or obesity. Higher educational level was associated with lower prevalence of diabetes, and the West-Central region had the highest prevalence of all Brazilian regions. ^28^

• Malta *et al.* , in a cross-sectional study including self-reported data of 60 202 Brazilians, have analyzed the inequalities in the self-reported prevalence of noncommunicable diseases, including diabetes. The prevalence of diabetes was higher among illiterate individuals or those with incomplete elementary education and those with incomplete secondary education [9.61% (PR 1.42, 95% CI: 1.13-1.77) and 5.36% (PR 1.59, 95% CI: 1.23-2.06), respectively], while those with higher education had a 4.18% prevalence. ^28^

• Iser *et al.* have shown, using data from the PNS (2014 to 2015), that the prevalence of prediabetes was 18.5% (95% CI: 17.4 – 19.7), according to the American Diabetes Association (HbA1c: 5.7-6.4%), and 7.5% (95% CI: 6.7 – 8.3), according to the World Health Organization criterion (HbA1c: 6.0-6.4%). ^313^

• In 2019, Teló *et al.* have published the results of a big cross-sectional study developed in a representative sample of Brazilian students aged 12 to 17 years showing that, of the 37 854 young individuals enrolled, 3.3% (95% CI: 2.9 – 3.7) had type 2 diabetes (HbA1c: ≥6.5% or fasting plasma glucose ≥ 126 mg/dL or history of diabetes) and 22.0% (95% CI: 20.6 – 23.4) had prediabetes (HbA1c: between 5.7% and 6.5% or fasting plasma glucose between 100 and 126 mg/dL). ^314^

### Incidence

• Most studies on the incidence of diabetes have reported data from high-income countries, and usually from the adult population, thus reflecting mainly the incidence of type 2 diabetes. In children and adolescents, type 2 diabetes has been increasing in several countries because of the increase in the prevalence of overweight, obesity, physical inactivity, and sedentary lifstyle. ^312^

• Schmidt *et al.* have recruited from 2008 to 2010 and followed up for 3.7 ± 0.63 years 11 199 civil servants without diabetes from the Brazilian multicenter study, the ELSA-Brazil Study. Diabetes was diagnosed in the follow-up if fasting plasma glucose ≥ 126 mg/dL or glycemia after oral glucose tolerance test ≥ 200 mg/dL or HbA1c ≥ 6.5%. The cumulative incidence of diabetes was 2.0 per 100 person-years (95% CI: 1.8 – 2.1), similar in men and women, higher in individuals over the age of 65 years [2.8%; 95% CI: 2.3 – 3.4], obese (3.8%; 95% CI: 3.4 – 4.3) and those with lower educational level (3.0%; 95% CI: 2.6 – 3.6). ^315^

• Sitnik *et al* ., in a prospective cohort of 1536 individuals without diabetes in 1998 (fasting plasma glucose collection date), civil servants of the University of São Paulo, participants of the ELSA-Brazil Study, aged 23-63 years, have assessed the association of fasting plasma glucose, incidence of diabetes, subclinical atherosclerosis and cardiovascular events. The adjusted diabetes incidence rate was 9.8/1000 person-years (95% CI: 7.7 – 13.6 /1000 person-years). ^316^

• The number of children and adolescents with type 1 diabetes has increased worldwide, approximately 3% per year, although with important regional differences. ^312^ ^,^ ^317^ ^,3186,^ ^12^ ^,^ ^13^ Brazil ranks third in the number of new cases of children and adolescents aged 0-14 years with type 1 diabetes (7.3 cases per 1000/year), in addition to the prevalence of the disease in that same age range (51.1 per 1000). ^312^

• Negrato *et al.* have described the annual incidence of type 1 diabetes from 1986 to 2015 in the city of Bauru, São Paulo state, in children ≤ 14 years of age, using individual case report and capture and recapture method. In that period, 302 cases were identified, an incidence of 12.8 per 100 000 inhabitants (95% CI: 11.2 – 14.4), ranging from 2.8 per 100 000 inhabitants in 1987 to 25.6 per 100 000 inhabitants in 2013, without difference between sexes. ^319^

### Mortality

#### Overall Mortality Attributable to Diabetes

• Klafke *et al.* have described trends in mortality from acute complications of diabetes (ketoacidosis, hyperosmolarity and hypoglycemia) in Brazil, for all ages, from 1991 to 2010, when the SUS was implemented. Using data from the IBGE and the SIM, over that period, 694 769 deaths from diabetes were registered, of which 81 208 (11.7%) were due to acute complications. In 1991, 2070 men died from acute complications of diabetes, with an age-adjusted mortality rate per 100 000 inhabitants of 7.4 (95% CI: 7.2 – 7.6), while 2832 women died, with mortality rate of 9.7 per 100 000 inhabitants (95% CI: 9.1 – 9.6), yielding a woman/man mortality rate of 1.3. As compared to 1991, in 2010, 1600 men died from acute complications of diabetes, with mortality rate of 2.4 per 100 000 inhabitants (95% CI: 2.3 – 2.5), while 2141 women died, with mortality rate of 2.5 per 100 000 inhabitants (95% CI: 2.4 – 2.6), yielding a woman/man mortality rate of 1.0. Thus, mortality from complications of diabetes in 20 years decreased 70.9% (95% CI: 67.2 – 74.5), from 8.42 (95% CI: 8.3 – 8.6) deaths per 100 000 inhabitants in 1991 for both sexes to 2.45 per 100 000 inhabitants (95% CI: 2.4 – 2.5) in 2010. That reduction occurred in both sexes, all ages, and in all Brazilian regions. ^320^

• Malhão *et al.* have assessed the diabetes mortality trends in Brazil, per sex, in adults ≥ 20 years of age, from 1980 to 2012, using data from IBGE and SIM. From 1980 to 2012, 955 455 individuals had diabetes as their underlying cause of death, and 551 016 were women (57.7%) and 404 439, men (42.3%). In that period, the age-standardized mortality rate per 100 000 inhabitants increased from 20.8 (95% CI: 20.2 – 21.5) to 47.6 (95% CI: 47.0 – 48.2) for men, and from 28.7 (95% CI: 27.9 – 29.4) to 47.2 (95% CI: 46.7 – 47.7) for women. Considering the entire period analyzed, that rate increased 2.9% per year for men (mean annual percent change of 2.9; 95% CI: 2.6 – 3.1) and 1.7% for women (mean annual percent change of 1.7; 95% CI: 1.5 – 1.9). Considering diabetes as the underlying or associated cause of death from 2001 to 2012, there were 1 076 434 deaths [603 686 women (56.1%); 472 748 men (43.9%)]. In that period, the age-standardized mortality rate increased from 76.1 per 100 000 inhabitants (95% CI: 75.2 – 77.0) to 95.6 per 100 000 inhabitants (95% CI: 94.8 – 96.5) in men, and from 83.7 per 100 000 inhabitants (95% CI: 82.9 – 84.6) to 93.3 per 100 000 inhabitants (95% CI: 92.6 – 94.1) in women. ^321^

• Duncan *et al.* , using data on diabetes and hyperglycemia from the GBD Study 2015 for all ages, have shown that in Brazil, in 2015, the diabetes mortality rate was 26.8 per 100 000 inhabitants (95% CI: 25.0 – 28.5) for men and 33.2 per 100 000 inhabitants (95% CI: 31.1 – 35.2) for women. ^322^

• Duncan *et al.* , using data on diabetes and hyperglycemia from the GBD Study 2016-2017 for all ages, have shown that, in that period, type 1 diabetes age-standardized mortality rate decreased by more than 50% for women and about 10% for men, thus the sex-specific mortality rates, which were different in the beginning of the period, converged. Age-standardized mortality rates for type 2 diabetes, however, remained stable during the period, with a slight reduction for women and slight increase for men. When considering the crude overall rates, however, mortality from type 2 diabetes increased dramatically, basically doubling in the period. In addition, those authors have shown that the highest mortality rates from diabetes in 2017 occurred mainly in the Northeastern states, and the highest increases in mortality from 1990 to 2017 were observed in the Northern, Northeastern and West-Central regions, and the greatest reductions in the Southeastern region. ^323^

• Data from the GBD Study 2019 have shown that, although the age-standardized mortality rates from diabetes have substantially decreased in recent years in Brazil, the total number of deaths from diabetes has increased (Chart 8-3A). There were 87 644 (95% CI: 71 924 -110 625) and 174 198 (95% CI: 142 704 – 217 111) deaths from diabetes in Brazil in 1990 and 2019, respectively (Chart 8-1A). Age-standardized mortality rate per 100 000 inhabitants was 116.8 (95% CI: 92.8 – 152.0) in 1990 and 77.0 (95% CI: 63.0 – 96.7) in 2019, decreasing by 34% (95% CI: -40.1 to -28.1) in that period. That reduction was not homogeneous in the Brazilian regions and FUs, being more pronounced in the Southeastern, West-Central and Southern regions and the Distrito Federal, with a slight to small reduction in several Northern FUs. It is worth noting that the reduction trend in age-standardized mortality rate from diabetes occurred in all age groups, but more significantly in the age group from 5 to 14 years (-45.8%; 95% CI: -57.2 to -33.1) and less significantly in the age group from 15 to 49 years (-24.8%; 95% CI: -30.7 to -18.5), with intermediate reductions in the age groups > 70 years (-26%; 95% CI: -33.2 to -18.2) and 50-69 years (-33.2%; 95% CI: -40.1 to -26.4). ^46^

## Cardiovascular Mortality Attributable to Diabetes

• Mortality from CVD attributable to diabetes for all ages in Brazil, according to GBD 2019 data, increased in absolute numbers from 50 812 deaths (95% CI: 35 649 -73 137) in 1990 to 80 754 (95% CI: 55 922 – 11 8175) in 2019 (Table 8-3 and Chart 8-1B). However, the age-standardized mortality rates per 100 000 inhabitants decreased from 70.4 (95% CI: 47.4 – 106.1) in 1990 to 35.9 (95% CI: 24.5 – 53.0) in 2019, a reduction of -49.0% (95% CI: -53.4 to -43.9) (Chart 8-1C). This reduction trend occurred in a non-uniform way in the FUs, with a more significant reduction in most FUs of the Southern, Southeastern, West-Central and Northern regions and the Distrito Federal, and only modest reductions in the FUs of the Northeastern region. When comparing between sexes, women had greater mortality rate reductions than men in most Brazilian FUs regarding data from 1990 and 2019, except for the states of Maranhão and Piauí and the Distrito Federal (Table 8-4). Regarding the trend according to age group, the reduction in the mortality rate from CVD attributable to diabetes occurred in all age groups: 15 to 49 years (-37.3%; 95% CI: -46.8 to -25.3), 50 to 69 years (-46.0%; 95% CI: -54.2 to -35.5), and > 70 years (-43.5%; 95% CI: -50.3 a -36.7). It is worth noting that, in 2019, when comparing sex and age groups (Chart 8-2), women had lower proportional mortality rates from CVD attributable to diabetes in almost all age groups described. ^46^

• Although data from the GBD Study 2019 showed that the total number of deaths attributable to diabetes for all ages and stratified for all causes increased in Brazil from 1990 to 2019 (Chart 8-3A), the age-standardized mortality rate per 100 000 inhabitants decreased in the same period from 116.8 (95% CI: 92.8 – 152.0) to 77 (95% CI: 63.0 – 96.7). It is worth noting that more than 85% of that reduction occurred due to the decrease in the rates of ischemic heart disease, from 39.8% (95% CI: 21.3 - 65.3) in 1990 to 20.9% (95% CI: 11.6 – 34.4) in 2019, and of stroke, from 30.3% (95% CI: 18.2 – 54.8) in 1990 to 14.7% (95% CI: 8.6 – 25.6) in 2019 (Chart 8-3B). ^46^

• The SDI is an estimate of the socioeconomic level of a certain place and Chart 8-4 shows the relation of that index in 2019 and the percent change in deaths from CVD attributable to diabetes from 1990 to 2019. The data show the correlation between the greater percent change in the age-standardized mortality rate from 1990 to 2019 and the SDI in 2019, suggesting that the reduction in CVD mortality followed the improvement in socioeconomic conditions. The FUs with lower SDI had smaller percent changes in deaths (more deaths), except for Roraima and Rondônia, while the FUs with higher SDI had higher percent changes in deaths (less deaths), such as São Paulo, Rio de Janeiro, and the Distrito Federal.

## Burden of Disease

### Burden of Disease Attributable to Diabetes

• Data from the GBD Study 2019 estimated a reduction in the age-standardized DALY rates per 100 000 inhabitants attributable to diabetes in Brazil of -26.1% (95% CI: -31.7 to -20.9) from 1990 to 2019, despite the increase in the total number of DALYs from 2 448 714.5 (95% CI: 2 087 403.6 – 2 919 735.6) to 4 778 225.9 (95% CI: 4 017 716.8 – 5 709 063.5) in the period. The reduction in the DALY rate, although occurring in all age groups, was more pronounced in the FUs of the Southeastern, Southern and West-Central regions and the Distrito Federal, being less significant in many FUs of the Northern and Northeastern regions, including the increase in the DALY rate in the states of Amapá, Ceará, and Maranhão. When comparing between sexes, the DALY rate reduction was more significant in women (-37.7%; 95% CI: 44.2 to -31.1) than in men (-29.9%; 95% CI: 36.9 to -22.3) for the same period. ^46^

### Burden of Cardiovascular Disease Attributable to Diabetes

• Data from the GBD Study 2019 regarding the burden of CVD estimated a reduction in the age-standardized DALY rates attributable to diabetes in Brazil (Chart 8-1D and Table 8-5) of -47.4% (95% CI: -52.2 to -41.9) per 100 000 inhabitants from 1990 to 2019, despite the increase in the total number of DALYs from 1 072 309 (95% CI: 784 276 - 1 484 959) to 1 571 116 (95% CI: 1 140 912 – 2 203 188) in the period. This rate reduction occurred due to the reduction in the YLLs of -33.4% (95% CI: -42.5 to -15) from 1990 to 2019. In the same period, there was an increase in the YLDs of 17.6% (95% CI: 0.4 – 50.5). ^46^

• There was a heterogeneous reduction in the age-standardized DALY rates attributable to diabetes among the Brazilian FUs and regions, more marked in the FUs of the Southeastern, Southern, and West-Central regions and the Distrito Federal, with a mild reduction in the FUs of the Northern region and an even milder reduction in most FUs of the Northeastern region.

• Data from the GBD Study 2019 showed that the reduction in the age-standardized DALY rates attributable to diabetes from 1990 to 2019 occurred in all age groups: 15-49 years (-36.5%; 95% CI: -46.0 to -24.5), 50-69 years (-45.5%; 95% CI: -53.4 to -35.2) and > 70 years (-46.2%; 95% CI: -52.5 to -39.1). For men of all ages, the variation was -43.1% (95% CI: -49.4 to -35.1), and for women of all ages, the variation was -52.0% (95% CI: -58.5 to -44.5). Only in the states of Maranhão and Piauí, men had a higher reduction as compared to women. ^46^

### Impact on Cardiovascular Health

• Fuchs *et al.* , in a cross-sectional study carried out in 2005 in the city of Porto Alegre, Rio Grande do Sul state, with adults (18 to 90 years of age), assessed the association of clustering of risk factors and self-reported CVD. The participants were interviewed at home about the presence of diabetes, physical activity, and diet pattern, in addition to assessed regarding the presence of systemic arterial hypertension. The sample consisted of 1007 women, mostly white (73.0%), mean age of 44.8±0.8 years, and mean schooling of 9.3±0.3 years. Arterial hypertension, diabetes, obesity, low fruit and vegetable intake, and lack of vigorous or moderate physical activity were clustered into a combination of risk factors, which independently associated with the self-reported CVD. The major cluster included the presence of arterial hypertension and diabetes, representing an independent risk ratio of 8.5 (95% CI: 3.0 – 24.5). ^281^

• Cardoso *el al.* reported the results of a prospective observational study carried out in Rio de Janeiro from August 2004 to December 2008 with 620 adults with type 2 diabetes, followed up until August 2013 in a diabetes outpatient clinic from a Brazilian university-affiliated hospital. Those authors aimed to relate the HbA1c levels with cardiovascular outcomes. The HbA1c levels were measured at baseline and 3-4 times per year during follow-up. The primary endpoints were a composite of total fatal and nonfatal cardiovascular events, major cardiovascular events, and all-cause mortality. Cardiovascular and noncardiovascular mortalities were the secondary endpoints. The sample’s mean age was 60.4±9.4 years, 37.1% were men, 55% Caucasians. After a median follow-up of 79 [59-93] months, 125 total cardiovascular events occurred (90 major events), with a total of 111 deaths (64 due to CVD). After statistical adjustment to other cardiovascular risk factors, baseline HbA1c and mean HbA1c in the first year were predictors of total cardiovascular events (HR 1.13; 95% CI: 1.04 – 1.23 and HR 1.26; 95% CI: 1.12 – 1.41, respectively), of major cardiovascular events (HR 1.15; 95% CI: 1.04 – 1.28 and HR 1.27; 95% CI: 1.11 –1.45, respectively), of all-cause mortality (HR 1.10; 95% CI: 1.00 – 1.21 and HR 1.18; 95% CI: 1.04 – 1.35, respectively) and cardiovascular mortality (HR 1.14; 95% CI: 1.01 – 1.27 and HR 1.27; 95% CI: 1.08 – 1.50, respectively). Every 1% increase in the mean HbA1c in the first year increased by 27.0% (95% CI: 11.0 – 45.0) the risk of major cardiovascular events. Cardiovascular protection was observed up to HbA1c levels lower than 6.5%. On the second year, however, HbA1c was not a predictor of any endpoint (HR 1.12; 95% CI: 0.98 – 1.28; p=0.09 for total cardiovascular events; HR 1.09; 95% CI: 0.92 – 1.29; p=0.32 for major cardiovascular events). ^324^

• Sitnik *et al.* , in a prospective cohort of 1536 individuals without diabetes in 1998 (fasting plasma glucose collection date), civil servants of the University of São Paulo, participants of the ELSA-Brazil Study, aged 23-63 years, aimed to assess the association of fasting plasma glucose and incidence of diabetes with subclinical atherosclerosis and cardiovascular events. Fasting plasma glucose levels of 110-125mg/dL were associated with higher carotid intima-media thickness (β 0.028; 95% CI: 0.003 – 0.053). Excluding the individuals who developed diabetes in the follow-up, there was a borderline association between higher carotid intima-media thickness and fasting plasma glucose levels of 110-125mg/dL (β 0.030; 95% CI: -0.005 to 0.065). Diabetes was associated with higher carotid intima-media thickness (β 0.034; 95% CI: 0.015 – 0.053), coronary artery calcium scores ≥ 400 (OR 2.84; 95% CI: 1.17 – 6.91) and the combined outcome of coronary artery calcium score ≥ 400 or cardiovascular event (OR 3.50; 95% CI: 1.60 – 7.65). ^316^

• Schaan *et al.* , analyzing data from the REACT Study, a Brazilian multicenter registry, carried out from July 2010 to May 2016, aimed to establish the long-term risk for clinical events of patients at high cardiovascular risk in Brazil. The project was idealized and coordinated by the SBC, with the participation of private and public centers from all Brazilian regions, respecting the population distribution according to IBGE data. A total of 5006 individuals aged ≥ 45 years were included and divided in the following four groups: no diabetes and no previous cardiovascular event (n = 430); diabetes and no previous cardiovascular event (n = 1138); no diabetes and previous cardiovascular event (n = 1747); and diabetes and previous cardiovascular event (n = 1691). Previous cardiovascular event was defined as evidence of coronary artery disease, stroke or transient ischemic accident, peripheral artery disease, presence of three cardiovascular risk factors – except for diabetes (arterial hypertension, dyslipidemia, age > 70 years, family history of coronary artery disease, asymptomatic carotid artery disease). Major clinical events (all-cause mortality, nonfatal myocardial infarction, nonfatal cardiopulmonary arrest, and nonfatal stroke) were observed in 332 patients during the 1-year follow-up. Previous cardiovascular event was associated with a higher risk for another event during follow-up (HR 2.31; 95% CI: 1.74 – 3.05, p<0.001), as was the presence of diabetes (HR 1.28; 95% CI: 1.10 – 1.73, p=0.005). Patients with diabetes, failing to meet the HbA1c target, had worse event-free survival as compared to patients with good metabolic control (HR 1.70; 95% CI: 1.01 – 2.84, p=0.044). ^325^

• Rezende *et al.* , in a retrospective cohort study, included 888 patients with type 2 diabetes and multivessel coronary artery disease in the MASS Registry from the Instituto do Coração of the University of São Paulo from January 2003 to December 2007. The patients were followed-up with outpatient visits every 6 months and prospectively assessed for cardiovascular events. Of the 888 patients, 725 (81.6%) had complete clinical and HbA1c data for analysis. The sample’s characteristics were as follows: median age, 62.4 years (55.7 – 68.0); 467 men (64.4%); median follow-up, 10.0 years (8.0 – 12.3); and a mean amount of 9.5±3.8 HbA1c values for each patient. The composite endpoint of death, myocardial infarction or ischemic stroke occurred in 262 patients (36.1%). An increase of 1 point in the longitudinal HbA1c value was significantly associated with a 14% higher risk of the combined endpoint of all-cause mortality, myocardial infarction, and ischemic stroke (HR 1.14; 95% CI: 1.04 – 1.24; p=0.002) in the unadjusted analysis. After adjusting for baseline factors (age, sex, 2-vessel or 3-vessel coronary artery disease, initial coronary artery disease treatment, ejection fraction, creatinine and LDL-cholesterol levels), a 1-point increase in the longitudinal HbA1c value was associated with a 22% higher risk of the combined endpoint (HR 1.22; 95% CI: 1.12 – 1.35; p<0.001). ^326^

## Knowledge, Treatment and Control Diabetes

• The treatment of diabetes is based on three pillars: diet, physical exercise, and drugs (oral antidiabetic drugs and insulin). A cross-sectional multicenter study published in 2016 by Gomes and Negrato ^327^ with a convenience sample of 1698 patients with type 1 diabetes from 10 Brazilian cities, assessed adherence to treatment (Morisky medication scale questionnaire), which was maximal in 9.8% of them, moderate in 42.2%, and minimal in 48.0%. Lower adherence was associated with higher HbA1c values (9.2 ± 2.2%, 8.9 ± 2.0%, and 8.6 ± 1.9%, respectively) for each group cited. ^327^

• A randomized clinical trial involving 238 patients with type 2 diabetes has compared an empowerment program for selfcare based on a behavior change protocol and its effect on glycemic control, and showed that the group randomized for the intervention had lower Hba1c levels (7.5 ± 1.7% *vs.* 8.1 ± 2.2%). ^328^

• In 2017, Meiners *et al.* , based on data from a household population-based survey (PNAUM) with cross-sectional design and a probabilistic sample of the Brazilian population, have assessed the access and adherence to treatment of 2624 patients with diabetes aged > 20 years. Total access to the medications studied was 98%, while adherence was probable in 71% (95% CI: 67.2 – 74.5), probably low in 9.8% (95% CI: 8.0 – 12.0), and low in 17.2% (95% CI: 14.6 – 20.1), with better adherence rates in the West-Central region. ^329^

• Self-reported adherence to treatment and its associated factors were assessed by Marinho *et al.* in 2018 in a cross-sectional study with 476 patients with diabetes in a tertiary hospital. Good adherence was 93.5% for medication use, 59.3% for foot care, 56.1% for glycemic monitoring, 29.2% for diet, and 22.5% for physical exercise. The following were associated with good adherence: younger age, lower BMI, presence of macrovascular complications, better occupational performance and emotional domain of SF-36. ^330^

• In 2018, Silva *et al.* conducted a household survey in 63 municipalities of Minas Gerais state, selected by convenience, aimed at assessing the use of drugs in patients with diabetes. The study assessed 2619 patients with diabetes (83.7% with type 2 diabetes and 10.4% with type 1 diabetes, mean age of 61.3 ± 16.4 years) and reported the use of 13 629 drugs, 35% were generic drugs and 60% acquired in public drugstores. The most frequently used drugs were metformin, losartan, glybenclamide, and simvastatin. Polypharmacy (use of five or more drugs) was identified in 56.5% (95% CI: 3.4) of the respondents. The factors associated with the occurrence of polypharmacy were age group of 40-59 years (OR 2.46; 95% CI: 1.68 – 3.61), age > 60 years (OR 4.58; 95% CI: 3.18 – 6.60), self-perception of poor or very poor health (OR 1.75; 95% CI: 1.26 – 2.38), presence of five or more comorbidities (OR 3.45; 95% CI: 2.84 – 4.19), mean time of diagnosis > 10 years (OR 1.64; 95% CI: 1.36 – 1.98), having at least four medical visits within the past year (OR 1.79; 95% CI: 1.48 – 2.16), lack of regular physical activity (OR 1.47; 95% CI: 1.22 – 1.78), interruption of the usual activities in the past 15 days (OR 1.30; 95% CI: 1.03 – 1.64), and having private health insurance (OR 1.39; 95% CI: 1.13 – 1.70). ^331^

• Leitão ***et al.*** , in a cross-sectional population-based study with individuals aged ≥ 20 years, reporting a medical diagnosis of diabetes and interviewed over the telephone (VIGITEL System) from 2012 to 2018, estimated the prevalence of use and distribution of sources of oral antidiabetic drugs in Brazil, according to sociodemographic variables. The prevalence of the use of oral antidiabetic drugs in Brazil increased from 77.4% (95% CI: 74.3 – 80.1) in 2012 to 85.2% (95% CI: 82.8 – 87.2) in 2018, and, in the Southern region, that use increased from 73.4% (95% CI: 67.8 – 78.4) in 2012 to 84.9% (95% CI: 79.7 – 88.9) in 2018. There was a decrease in the access to oral antidiabetic drugs in the public health system (SUS) pharmacies and an increase in the access to those drugs in popular pharmacies, without significant changes in that access in private drugstores. ^332^

• A cross-sectional study with convenience sampling from 20 Brazilian medical centers, regarding healthcare provided between 2006 and 2007, described the glycemic control of 5692 patients with diabetes > 18 years (1904 men and 3788 women). Of those, 72% of the men and 74% of the women had HbA1c levels > 7.0%. ^333^

• Schneiders ***et al*** ., in a retrospective cohort with 488 patients of primary (n=192) and tertiary (n=192) healthcare, have assessed the following diabetes care quality indicators in patients whose HbA1c level had been assessed in the past year: annual assessment of diabetic nephropathy, retinopathy and neuropathy, lipid profile, nutritional assessment, and inquiry about tobacco use. From the included patients, only 14 (7.3%) in primary healthcare and 52 (27.0%) in tertiary healthcare had at least 50% of those quality indicators covered. The major differences between the healthcare provided to patients in primary and tertiary healthcare were: assessment of diabetic nephropathy (84.8% *vs.* 95.8%, respectively), retinopathy (13.2% *vs.* 35.9%, respectively) and neuropathy (9.5% *vs.* 58.9%), in addition to nutritional assessment (17.2% *vs.* 38.0%, respectively). ^334^

• Alessi ***et al*** . have conducted a cross-sectional multicenter study on primary and tertiary healthcare to elderly with type 2 diabetes (>65 years, n=322, 160 in primary and 162 in tertiary healthcare) to assess the number of patients with proper glycemic control considering the need for individualized glycemic targets in a good part of that population. Patients meeting the glycemic targets were those with the following characteristics: HbA1c of 7.0-7.5% for an estimated life expectancy >10 years; HbA1c of 7.5-8.0% for an estimated life expectancy of 5-10 years; and HbA1c of 8.0-8.5% for an estimated life expectancy <5 years. In primary and tertiary healthcare, HbA1c level was over the target in 49.1% and 50.3% of the patients, respectively. In the entire sample, 42.2% of the patients were over the HbA1c target, 28.9% met the target, and 28.9% were below the target. ^335^

## Risk Factors and Prevention

• Obesity, diet pattern, physical inactivity and sedentary lifestyle are well-known risk factors for the development of type 2 diabetes. The prevalence of diabetes clearly increases as the prevalence of obesity increases. ^336^ Diabetes can be prevented or its onset postponed with lifestyle changes, diet pattern changes, and use of drugs, mostly oral antidiabetic drugs.

• A systematic review with meta-analysis published by Sbaraini *et al.* in 2021 included 151 studies on the prevalence of overweight and obesity in Brazilian adolescents aged 10-19 years. They reported an increase in overweight prevalence as follows: 8.2% (95% CI: 7.7 – 8.7) up to 2000, 18.9% (95% CI: 14.7 – 23.2) from 2000 to 2009, and 25.1% (95% CI: 23.4 – 26.8) from 2010 onwards, a pattern similar to that of the prevalence of obesity. The Southeastern and Southern regions had higher prevalence of overweight and obesity, without difference between the sexes. ^337^

• Those same authors have shown that of 37 892 adolescents enrolled in the ERICA Study, 17.2% were overweight, 5.6% were obese, and 1.3% were severely obese, increasing the chance of adverse cardiometabolic outcomes according to higher BMI, including higher fasting plasma glucose [RP 5.30 (95% CI: 1.94 – 14.50)] and HbA1c (2.04; 95% CI: 1.29 – 3.25). ^338^

• Flor *et al.* have estimated the relation of type 2 diabetes in adults aged > 20 years and its percentage attributable to overweight and obesity in Brazil (Burden of Disease Project 2008 – Brazil). The results showed that 49.2%, 58.3% and 70.6% of diabetes in women were attributable to overweight, obesity, and excess weight, respectively. Regarding men, those percentages were 40.5%, 45.4%, and 60.3%, respectively. Differences were observed in the different Brazilian regions, and the most developed ones, South and Southeast, showed high percentages of diabetes attributable to obesity, while, in the Northern region, diabetes was related to overweight. This behavior might be related to a late epidemiological transition in less favored regions. ^339^

• In the baseline of the ELSA-Brasil Study, the analysis of 14 912 Brazilian civil servants has shown higher prevalence of diabetes among individuals with BMI of 25-29.9 kg/m^2^(18.9%; 95% CI: 18.0 – 19.9%) and above 30 kg/m^2^(32.1%; 95% CI: 30.6 – 33.6%) as compared to those with BMI ≤ 24.9 kg/m^2^(11.7; 95% CI: 10.9 – 12.6%). ^340^

• In a cross-sectional population-based study (VIGITEL, 2014), Moreira *et al* . have assessed the sociodemographic, behavioral, and dietary characteristics of 867 adults aged > 45 years in the city of João Pessoa, Paraíba state, and their associations with the presence of systemic arterial hypertension and diabetes. In an adjusted analysis, the prevalence of diabetes was higher among women with lower educational level (0-4 years of schooling, PR 3.3; 95% CI: 1.4 – 7.5) and no regular consumption of beans (PR 1.6; 95% CI: 1.0 – 2.8). Among men, the age ranges of 55-64 years (PR 5.1; 95% CI: 1.9 – 13.4) and ≥ 65 years (PR 4.0; 95% CI: 1.4 – 10.9) and being married (PR 17.7; 95% CI: 2.0 – 153.0) were associated with a higher prevalence of diabetes. ^297^

• In a cross-sectional population-based study conducted from 2006 to 2016 (VIGITEL, 2014), ^297^ Oliveira *et al* . assessed the sociodemographic, behavioral, and dietary characteristics of 572 437 adults aged >18 years from the Brazilian capitals and Distrito Federal and their association with self-reported diabetes. Individuals with diabetes had fewer risky and more protective behaviors as follows: higher intake of fruits and vegetables [34.0% (95% CI: 33.8 – 34.3) in controls and 40.7% (95% CI: 39.7 – 41.8) in individuals with diabetes, adjusted PR 1.05]; lower consumption of meat with excess fat [24.3% (95% CI: 23.3 – 25.2) *vs.* 32.3% (95% CI: 31.9 – 32.5), adjusted PR 0.95], of whole milk [44.5% (95% CI: 43.5 – 45.5) *vs.* 55.4% (95% CI: 55.1 – 55.7%), adjusted PR 0.87] and of soft drinks and sugar-sweetened beverages [9.5% (95% CI: 8.5 – 10.5) *vs.* 25% (95% CI: 24.6 – 25.4), adjusted PR 0.57]. Individuals with diabetes reported lower consumption of alcoholic beverages [15.9% (95% CI: 14.7 – 17.1) *vs.* 26.8% (95% CI: 26.4 – 27.2), adjusted PR 0.86] and less leisure-time physical activity [24.0% (95% CI: 23.1 – 25.0) *vs.* 34.6% (95% CI: 34.3 – 34.9), adjusted PR 0.92] than individuals without diabetes. ^341^

• In a cross-sectional analysis of the ELSA-Brazil Study, an adjusted model has shown an inverse association of diabetes and the intake of at least four servings per day of dairy products [0.76 (95% CI: 0.59 – 0.97)] in 10 010 adults. ^342^

• Considering leisure-time physical activity, the ELSA-Brazil Study has shown a lower chance of diabetes in active men and women as compared to inactive ones: 0.73 (95% CI: 0.61 – 0.87) and 0.83 (95% CI: 0.67 – 1.03), respectively. ^343^

• Werneck *et al* ., in 2018, based on data from the PNS-2013, have assessed the self-reported levels and patterns of television watching of 60 202 Brazilian adults and their association with type 2 diabetes. Television watching for more than 4 hours per day increased the likelihood of developing diabetes for men (1.64; 95% CI: 1.23 – 2.17) and women (1.33; 95% CI: 1.09 – 1.63) as compared to those watching television less than 2 hours per day. ^344^

• Teló *et al* ., in a cross-sectional study with 37 854 adolescents, have shown a higher likelihood of type 2 diabetes in those with obesity (OR 1.59; 95% CI: 1.20 – 2.11) and increased waist circumference (OR 1.51; 95% CI: 1.13 – 2.01), with no association with physical inactivity (< 60 min/day). ^314^

## Future Research

• The evidence is still insufficient whether diabetes prevention through lifestyle changes would also prevent cardiovascular and microvascular complications of the disease.

• Studies on the incidence of type 1 and type 2 diabetes with national representativity, aimed at social and behavioral determinants are required.

• Considering the SUS coverage and the possibility of reaching many patients with type 1 and type 2 diabetes, studies focused on assessing the efficacy and effectiveness of the care provided to these patients in Brazil are required.

• Considering the several publications on the increase of the overweight and obesity incidence in the Brazilian population in all age ranges, mostly in the lower socioeconomic levels, efficient public policies to prevent obesity should be prioritized to reduce new cases of diabetes and its complications. Some exemples are: 1. Taxation of high caloric foods; 2. Mandatory labeling of food products; 3. Creation of programs to prevent and treat obesity in communities, rescuing individuals predisposed to diabetes by using simple tools (questionnaires); 4. Training of multiprofessional teams to engage in lifestyle change programs to prevent and treat diabetes; 5. Integration of physical education professionals into those programs.

## 9. DYSLIPIDEMIA

### CID-10 E78 (E78.0 – E78.9); CID-10-CM E78 (E78.0 – E78.9)

**See Tables 9-1 through 9-8 and Charts 9-1 through 9-5**

**Table:** Abbreviations Used in Chapter 9

AMI	Acute Myocardial Infarction
CI	Confidence Interval
CVD	Cardiovascular Diseases
DALYs	Disability-Adjusted Life Years
ELSA-Brasil	The Brazilian Longitudinal Study of Adult Health
ERICA	Brazilian Study of Cardiovascular Risk in Adolescents (in Portuguese, *Estudo dos Riscos Cardiovasculares em Adolescentes* )
FU	Federative Unit
GBD 2019	Global Burden of Disease 2019
HDLc	High-Density-Lipoprotein Cholesterol
LDLc	Low-Density-Lipoprotein Cholesterol
OR	Odds Ratio
PNAUM	Study on the Access to, Use, and Promotion of Rational Use of Medicines in Brazil (in Portuguese, *Pesquisa Nacional sobre Acesso, Utilização e Promoção do Uso Racional de Medicamentos no Brasil* )
PNS	National Health Survey (in Portuguese, *Pesquisa Nacional de Saúde* )
SDI	Sociodemographic Index
SUS	Brazilian Unified Health System (in Portuguese, *Sistema Único de Saúde* )
TC	Total Cholesterol
TG	Triglycerides
UI	Uncertainty Interval
YLDs	Years Lived with Disability
YLLs	Years of Life Lost

**Table 9-1:** – Mean plasma lipid levels, prevalence of borderline and high levels, and estimated population with abnormal levels, by sex and age group. ERICA-Brasil, 2013-2014.

Lipids	Mean		Borderline		High		Estimated population with abnormality
	mg/dL	95% CI	%	95% CI	%	95% CI	
**Total cholesterol**							
General population	148.1	147.1-149.1	24.2	22.7-25.8	20.1	19.0-21.3	2 940 705
Men	143.6	142.4-144.8	22.7	20.4-25.2	15.3	13.9-16.9	1 256 102
Women	152.6	151.4-153.9	25.7	24.5-27.0	24.9	23.4-26.5	1 684 602
12-14 years	149.4	148.0-150.7	25.8	24.3-27.4	20.7	19.1-22.5	937 793
15-17 years	147.1	145.8-148.3	22.8	20.8-24.9	19.6	18.0-21.2	2 002 911
**LDLc**							
General population	85.3	84.5-86.1	19.5	18.5-20.5	3.5	3.2-4.0	1 526 733
Men	83.4	82.2-84.5	17.4	16.0-18.9	2.9	2.3-3.6	669 805
Women	87.2	86.3-88.1	21.5	20.2-22.9	4.3	3.7-4.9	856 928
12-14 years	86.2	85.1-87.3	20.6	19.0-22.4	3.7	3.1-4.4	467 877
15-17 years	84.5	83.5-85.5	18.4	17.2-19.7	3.4	2.9-4.1	1058 856
**Triglycerides**							
General population	77.8	76.5-79.2	12.0	11.0-13.0	7.8	7.1-8.6	1 312 329
Men	76.4	74.7-78.1	10.9	9.8-12.2	7.6	6.5-8.8	610 449
Women	79.3	77.8-80.7	13.0	11.8-14.2	8.1	7.3-9.0	701 880
12-14 years	78.9	76.7-81.0	12.7	11.0-14.6	8.3	7.2-9.5	434 638
15-17 years	76.9	75.8-78.1	11.3	10.2-12.4	7.4	6.6-8.4	877 690
**HDLc**	**Mean**		**Low**				
General population	47.3	46.7-47.9	46.8	44.8-48.9	-	-	3 104 161
Men	44.9	44.4-45.5	55.9	53.7-58.2	-	-	1 256 003
Women	49.6	48.9-50.3	37.8	35.4-40.2	-	-	1 848 158
12-14 years	47.4	46.7-48.1	45.0	42.3-47.8	-	-	819 980
15-17 years	47.2	46.4-48.0	48.4	45.9-50.8	-	-	2 284 181

*Modified from Faria Neto et al. ^*349*^ *LDLc: low-density-lipoprotein cholesterol; HDLc: high-density-lipoprotein cholesterol a: change = borderline + high values. b: Population estimates for the domains were obtained by processing the microdata from the 2000 and 2010 Demographic Census from the Brazilian Institute of Geography and Statistics.*

**Table 9-2:** – Prevalence of total cholesterol ≥ 200 mg/dL according to sex, age group, educational level, skin color, and country region. Brazil, PNS 2014-2015.

	Total			Men			Women		
	%	95% CI	p	%	95% CI	p	%	95% CI	p
**Total**	**32.7**	**31.5 - 34.1**		**30.1**	**28.2 - 32.1**		**35.1**	**33.4 - 36.8**	**< 0.001**
**Age group (years)**									
18 - 29	17.9	15.7 - 20.4	< 0.001	13.9	11.2 - 17.4	< 0.001	21.9	18.7 - 25.5	< 0.001
30 - 44	31.0	28.7 - 33.4		34.9	31.2 - 38.8		27.6	24.9 - 30.5	
45 - 59	43.4	40.8 - 46.0		39.4	35.7 - 43.4		47.0	43.5 - 50.5	
≥ 60	41.9	39.1 - 44.8		33.5	29.5 - 37.9		48.4	44.7 - 52.2	
**Education (school years)**									
0 - 8	37.1	35.2 - 39.1	< 0.001	31.6	28.9 - 34.5	0.237	42.2	39.6 - 44.8	< 0.001
9 - 11	28.6	25.5 - 32.0		26.6	22.2 - 31.6		30.6	26.4 - 35.2	
≥ 12	30.4	28.4 - 32.5		30.0	26.9 - 33.3		30.8	28.3 - 33.4	
**Skin color**									
White	33.9	31.9 - 36.0	0.146	30.8	27.8 - 33.9	0.669	36.6	33.9 - 39.4	0.196
Black	33.2	29.0 - 37.6		30.0	23.9 - 37.0		36.0	30.5 - 41.8	
Mixed	31.5	29.8 - 33.3		29.5	26.9 - 32.4		33.4	31.1 - 35.7	
Others	23.3	14.8 - 34.6		19.6	9.7 - 35.4		25.8	14.2 - 42.2	
**Region**									
North	32.5	30.4 - 34.6	0.195	31.0	27.9 - 34.3	0.376	33.9	31.2 - 36.7	0.291
Northeast	34.0	32.3 - 35.8		30.2	27.7 - 33.0		37.4	35.1 - 39.8	
Southeast	31.5	29.1 - 34.1		28.7	25.1 - 32.6		34.1	30.9 - 37.4	
South	34.7	31.7 - 37.8		33.4	28.9 - 38.3		35.8	32.0 - 39.8	
West-Central	31.7	28.7 - 34.8		30.1	25.7 - 34.9		33.0	29.1 - 37.2	

*Source: Malta et al. ^348^*

**Table 9-3:** – Prevalence of low HDL-cholesterol (< 40 mg/dL) according to sex, age group, educational level, skin color, and Brazilian region, PNS 2014-2015.

	Total			Men			Women		
	%	95% CI	P	%	95% CI	p	%	95% CI	p
**Total**	**31.8**	**30.5 - 33.1**		**42.8**	**40.6 - 45.0**		**22.0**	**20.6 - 23.5**	**< 0.001**
**Age group (years)**									
18 - 29	29.1	26.2 - 32.2	0.070	39.7	34.9 - 44.7	0.159	18.7	15.9 - 21.9	0.046
30 - 44	31.8	29.4 - 34.2		41.8	37.9 - 45.7		23.0	20.4 - 25.9	
45 - 59	34.1	31.6 - 36.6		44.8	40.9 - 48.8		24.3	21.5 - 27.4	
≥ 60	32.4	29.8 - 35.2		46.5	42.1 - 51.1		21.5	18.7 - 24.6	
**Education (school years)**									
0 - 8	33.7	31.8 - 35.7	< 0.001	43.3	40.2 - 46.4	0.006	24.9	22.8 - 27.2	< 0.001
9 - 11	38.5	34.9 - 42.2		50.0	44.3 - 55.6		27.0	22.9 - 31.5	
≥ 12	27.8	25.9 - 29.9		39.6	36.2 - 43.2		18.1	16.1 - 20.3	
**Skin color**									
White	31.0	29.0 - 33.1	0.072	43.0	39.7 - 46.5	0.586	20.6	18.4 - 23.0	0.006
Black	28.5	24.3 - 33.2		41.8	34.5 - 49.4		16.6	12.6 - 21.6	
Mixed	33.5	31.7 - 35.4		43.0	40.0 - 46.1		24.8	22.8 - 27.0	
Others	24.7	15.8 - 36.5		27.7	15.1 - 45.2		22.7	11.6 - 39.5	
**Country region**									
North	36.6	34.4 - 38.8	< 0.001	47.2	43.7 - 50.7	0.036	26.7	24.2 - 29.4	< 0.001
Northeast	34.8	33.0 - 36.6		44.3	41.4 - 47.2		26.4	24.3 - 28.6	
Southeast	30.8	28.3 - 33.4		43.1	38.9 - 47.3		20.0	17.4 - 22.9	
South	26.1	23.3 - 29.0		36.3	31.6 - 41.2		16.8	14.1 - 20.0	
West-Central	34.3	31.1 - 37.6		45.0	39.8 - 50.3		24.7	21.2 - 28.6	

*Source: Malta et al. ^348^*

**Table 9-4:** – Prevalence of high LDL-cholesterol (> 130 mg/dL) according to sex, age group, educational level, skin color, and Brazilian region, PNS 2014-2015.

	Total			Men			Women		
	%	95% CI	p	%	95% CI	p	%	95% CI	p
Total	18.6	17.5 - 19.7		17.1	15.6 - 18.8		19.9	18.5 - 21.3	0.012
**Age group (years)**									
18 - 29	8.8	7.2 - 10.7	< 0.001	6.6	4.8 - 9.0	< 0.001	11.0	8.7 - 14.0	< 0.001
30 - 44	17.5	15.7 - 19.5		20.2	17.3 - 23.6		15.2	13.0 - 17.6	
45 - 59	25.6	23.3 - 27.9		23.2	20.0 - 26.7		27.7	24.7 - 30.9	
≥ 60	24.5	22.2 - 27.0		19.5	16.3 - 23.2		28.4	25.1 - 31.9	
**Education (school years)**									
0 - 8	21.5	20.0 - 23.2	< 0.001	17.8	15.7 - 20.1	0.525	24.9	22.8 - 27.2	< 0.001
9 - 11	16.8	14.3 - 19.7		15.2	11.8 - 19.3		18.5	15.0 - 22.6	
≥ 12	16.7	15.1 - 18.4		17.2	14.8 - 20.0		16.2	14.2 - 18.4	
**Skin color**									
White	20.1	18.5 - 21.9	0.009	18.8	16.4 - 21.4	0.131	21.3	19.1 - 23.8	0.095
Black	16.6	13.6 - 20.2		15.2	10.9 - 20.8		17.9	13.9 - 22.7	
Mixed	17.4	16.1 - 18.8		15.9	13.9 - 18.1		18.8	17.0 - 20.7	
Others	10.1	6.0 - 16.6		8.6	3.6 - 19.1		11.2	5.7 - 20.7	
**Country region**									
North	16.2	14.7 - 17.9	0.136	15.5	13.2 - 18.1	0.355	17.0	14.9 - 19.2	0.195
Northeast	19.8	18.4 - 21.3		17.5	15.5 - 19.8		21.9	19.9 - 23.9	
Southeast	17.9	16.0 - 19.9		16.1	13.4 - 19.3		19.4	16.8 - 22.2	
South	20.0	17.6 - 22.6		19.8	16.2 - 24.0		20.1	17.1 - 23.5	
West-Central	17.8	15.4 - 20.4		17.8	14.3 - 21.9		17.8	14.8 - 21.3	

*Source: Malta et al.348*

**Table 9-5:** – Numbers of deaths and age-standardized mortality rates attributable to high LDL-cholesterol levels in 1990 and 2019, and percent change of rates, in Brazil and Federative Units.

Death due to high LDL cholesterol and location	1990		2019		Percent change (95% UI)
	Number (95% UI)	Rate (95% UI)	Number (95% UI)	Rate (95% UI)	
Acre	80.9 (63.6;101.6)	64.4 (46.8;87.3)	210.6 (165.1;270.7)	38.3 (28.2;51.5)	-40.5 (-46.4;-32.9)
Alagoas	936.8 (725.7;1212.7)	78.9 (58.7;106.1)	1690.1 (1304.7;2141.2)	53.4 (40.6;69.2)	-32.3 (-40.6;-21.2)
Amapá	47.3 (37.8;59.9)	58.5 (43.4;79.3)	172.7 (139.2;218.4)	36 (27.5;47.8)	-38.5 (-44.6;-31.8)
Amazonas	411.7 (325.7;521.4)	69.7 (51.1;94.9)	883.6 (663.9;1167.2)	32.7 (23.5;44.5)	-53.1 (-59;-46.4)
Bahia	4070.8 (3156.6;5194.6)	66.4 (49.6;87.6)	6786.7 (5077.9;8762.6)	41.3 (30.8;53.8)	-37.8 (-48;-25.8)
Brazil	68327.1 (55096.6;83767.8)	88.6 (67.8;114.8)	99375.3 (78038.6;126142.7)	43.1 (33.4;55.9)	-51.3 (-53.8;-48.6)
Ceará	2062.8 (1514.7;2733.1)	53.3 (38.8;71.8)	4448.1 (3298.5;6074.6)	45.1 (33.2;62)	-15.5 (-29.9;4.8)
Distrito Federal	411.5 (339.1;499.7)	107.1 (77.1;144.1)	791.7 (603.6;1004)	41.7 (29.3;57.5)	-61.1 (-66.1;-55.6)
Espírito Santo	1032.7 (822.4;1303.7)	87.8 (64.6;117.7)	1923.6 (1488.8;2494.9)	45.9 (34.8;60.9)	-47.7 (-53.9;-41)
Goiás	1506.3 (1190.3;1897.7)	87.5 (64.1;116.2)	2894.6 (2210.2;3652.8)	43.1 (32.6;55.7)	-50.7 (-59.5;-40.2)
Maranhão	1836.1 (1417.7;2352.5)	77.1 (57.8;102.6)	3879.6 (2906.2;5171.6)	59.8 (44.1;80.6)	-22.5 (-37;-3.5)
Mato Grosso	548.4 (436.1;671.6)	82.5 (62;107.9)	1170.8 (904.1;1482.8)	37.4 (27.8;49.1)	-54.7 (-60.1;-48.1)
Mato Grosso do Sul	681 (555.3;829.6)	91.6 (70;117.2)	1255.2 (983.2;1596.2)	44.5 (33.9;58)	-51.5 (-56.8;-45.5)
Minas Gerais	7931.1 (6430.8;9675)	98.8 (75.7;126.6)	9517.5 (7373.8;12338.1)	36 (27.7;46.8)	-63.6 (-67.7;-59.3)
Pará	1367.8 (1062.2;1781.8)	81.3 (59.4;112.2)	2690.8 (2065;3506.2)	39.7 (29.8;53)	-51.1 (-57.5;-43.6)
Paraíba	1382.7 (1045.4;1805.4)	64 (48;83.7)	2269.8 (1696.4;3012.5)	46.2 (34.9;60.3)	-27.9 (-37.2;-16.6)
Paraná	4091.5 (3306.9;5040.5)	104.8 (79;139.1)	5530.9 (4235;7223)	43.9 (33.1;58.3)	-58.1 (-62.3;-53.2)
Pernambuco	3428.3 (2704;4329.7)	86.5 (65.4;113.9)	5636 (4347.5;7062.3)	57.6 (44;73.1)	-33.4 (-40.4;-24.4)
Piauí	847.6 (658.5;1106.6)	73.5 (53.9;100.8)	1584.5 (1179.1;2110.5)	41 (30.9;54.7)	-44.2 (-51;-36.5)
Rio de Janeiro	9443.4 (7744.9;11437.7)	112.3 (87.4;141.7)	10806.8 (8418.7;13551.9)	49.1 (37.8;62.2)	-56.3 (-60.6;-51.7)
Rio Grande do Norte	916.4 (688.2;1212)	61.3 (45.6;81.8)	1644.4 (1223.6;2156.1)	41 (30.9;53.6)	-33.1 (-43.6;-20.3)
Rio Grande do Sul	5115.6 (4086.6;6345.7)	90.4 (68.2;117.4)	6199.1 (4632.3;8338.2)	40.5 (30;54.5)	-55.2 (-59.9;-50.7)
Rondônia	242 (196.1;293.9)	108.9 (78.7;147.6)	630.4 (483.6;801.3)	43.7 (32.4;57.5)	-59.9 (-65.6;-52.8)
Roraima	36.7 (30;44.4)	87.6 (64.5;118.2)	123.9 (98.8;152.6)	41 (30.3;54.8)	-53.1 (-57.7;-47.9)
Santa Catarina	1997.2 (1608.5;2492.3)	96.8 (72.5;128.5)	3061.3 (2351.4;3875.8)	40.6 (30.1;52.9)	-58.1 (-62.4;-53.1)
São Paulo	17219.5 (13928.7;21011.3)	104.1 (79.1;133.6)	21988.1 (16961;27558.7)	42.2 (31.9;53.6)	-59.5 (-63.6;-54.9)
Sergipe	450.3 (344;592.6)	72.8 (52.1;100.4)	935.9 (707.3;1229.3)	42.3 (31.4;56.2)	-41.9 (-51;-30)
Tocantins	230.7 (179.4;291)	80.5 (57.9;110.3)	648.6 (491.9;860.9)	47.3 (35.1;63.8)	-41.3 (-50.2;-30.1)

*Source: Data derived from Global Burden of Disease Study 2019, Institute for Health Metrics and Evaluation, University of Washington. ^46^ Rates per 100,000 inhabitants.*

**Table 9-6:** – Mortality rate (age-standardized) attributable to high LDL-cholesterol levels in 1990 and 2019, and percent change of rates, stratified by sex, in Brazil and its Federative Units.

Mortality rate attributable to high LDL cholesterol	Women			Men		
	1990	2019	Percent change (95% UI)	1990	2019	Percent change (95% UI)
Acre	49.4(35.1-67.2)	28.8(20.5-39.9)	-41.6(-49.4--31.2)	82.5(59.6-112.2)	49.9(37-66.7)	-39.5(-48--29.1)
Alagoas	67.9(48.6-92.7)	45(32.9-61)	-33.8(-44.2--19.6)	91.3(68.3-122)	63.5(46.7-82.4)	-30.4(-42.8--14.1)
Amapá	47.1(34.3-65.4)	27.1(19.5-37.3)	-42.5(-49.6--34.3)	71(53.5-95.4)	46(34.8-61.1)	-35.2(-42.9--26.7)
Amazonas	63.5(44.2-88.4)	25(17.2-35.2)	-60.6(-66.4--53.3)	75(55.5-100.1)	40.7(29.6-54.8)	-45.8(-54.5--35.8)
Bahia	57.9(41.7-79.8)	30.6(20.8-41.7)	-47.1(-58--32.6)	75.8(56.3-101)	54.7(39.5-72.8)	-27.9(-44.4--6.6)
Brazil	72.9(54.2-97.8)	33.8(25-45.1)	-53.7(-56.9--50.4)	105.7(82.4-133.6)	54.2(42.3-68.4)	-48.8(-52.1--44.9)
Ceará	45.4(31.7-62)	36.8(25.1-51.3)	-18.9(-36.8-7.7)	62.4(44.6-86.9)	54.9(38.8-76.8)	-12(-33.7-19)
Distrito Federal	89.4(63.8-121.1)	34.3(22.9-48.5)	-61.6(-68.4--54.8)	137.9(97.6-187.2)	51.7(36.6-70.3)	-62.5(-67.8--56)
Espírito Santo	76.2(54.7-104.6)	35.9(26.2-48.7)	-52.9(-59.3--45)	100.1(75.8-131.4)	57.7(43.9-76.4)	-42.4(-51.3--32.6)
Goiás	74(52.2-102.8)	34.5(24.7-47.2)	-53.4(-62.3--41.9)	102(75.4-134.7)	52.6(38.9-68.4)	-48.4(-60--32.7)
Maranhão	42.3(29.7-57.3)	41.8(30.2-58.5)	-1(-23.3-31.9)	134.1(95.5-185)	84.3(59.8-116.1)	-37.2(-51.1--18.1)
Mato Grosso	68.8(50.2-91.4)	30.3(21.6-41.1)	-56(-62.7--47.7)	93.8(70.5-124)	44.3(33.6-58.2)	-52.7(-60.3--42.7)
Mato Grosso do Sul	78.6(57.9-103.8)	34.8(24.6-47.5)	-55.7(-61.6--48.8)	102.9(79-131)	54.9(41.8-70.3)	-46.7(-54.6--37.6)
Minas Gerais	83.7(61.4-111.4)	28.9(20.8-39.5)	-65.4(-70.4--60.1)	114.8(89.2-144.7)	43.9(33.5-56.7)	-61.8(-67.5--55.4)
Pará	68.9(47.9-97.3)	29.6(20.9-41.1)	-57(-63.9--48.4)	93.6(68.8-127.3)	50.5(37.6-67.7)	-46(-55.7--33.7)
Paraíba	58.3(42-78.4)	38.1(27.7-51.7)	-34.6(-45.1--20.4)	70.9(52.5-93.2)	55.7(41.7-73)	-21.4(-37--1.5)
Paraná	92.9(67.9-126.1)	35.2(25.2-48.3)	-62.1(-67.2--56.8)	116.5(89.3-151.1)	53.9(39.8-70.7)	-53.7(-60.2--46.2)
Pernambuco	75.6(55.6-100.1)	45(32.8-59.3)	-40.4(-48.7--31.2)	99.1(76.5-127.5)	73.5(55.7-93.5)	-25.8(-37.2--12.3)
Piauí	55.8(39-78.6)	32.7(23.3-46.5)	-41.5(-50.7--30.6)	94.6(70.4-130.5)	50.6(38.7-66.1)	-46.5(-55--35.8)
Rio de Janeiro	88.4(66-117.8)	36.7(27.2-48.4)	-58.4(-63.5--52.5)	142(111.6-176.7)	64.9(50-83.1)	-54.3(-60.1--47.7)
Rio Grande do Norte	49.7(35.3-66.6)	31.1(21.9-41.4)	-37.3(-49.4--22.6)	75.1(55.3-100.5)	52.9(38.4-70.5)	-29.5(-45.8--8.5)
Rio Grande do Sul	76.3(55.9-102.2)	34(24.4-47.2)	-55.4(-60.8--49.6)	105.6(81.6-134.5)	47.5(35-62.7)	-55(-61--48.5)
Rondônia	103.6(71.7-145.3)	35.2(25.2-47.9)	-66.1(-71.6--59.5)	112.1(81.3-152.8)	52.1(37.9-68.5)	-53.5(-62.4--41.9)
Roraima	68.1(48.4-95.3)	31.9(22.5-43.8)	-53.1(-58.4--47.9)	103(76.2-135.5)	49.4(36.7-65.5)	-52.1(-58.1--44.6)
Santa Catarina	84(60.1-115.4)	32.7(22.9-45.3)	-61.1(-66.2--55.1)	110.1(84.9-143.3)	49.2(37.2-63.2)	-55.3(-61.6--48.3)
São Paulo	84.8(61.3-112.9)	32.9(23.6-44.3)	-61.2(-66.3--55.5)	125.3(96.9-159)	53.1(40.3-68.1)	-57.6(-63.1--51)
Sergipe	64.6(45.3-89.8)	35.9(25.6-50)	-44.5(-55.1--30.6)	82.4(58.7-115)	50(36.1-66.6)	-39.3(-52.3--21.8)
Tocantins	67(46.4-94.9)	33.2(23.9-45.5)	-50.5(-59.7--38.3)	93.5(66-129.9)	64.1(46.5-86.8)	-31.5(-44.9--13.7)

*Source: Data derived from Global Burden of Disease Study 2019, Institute for Health Metrics and Evaluation, University of Washington. ^46^ Rates per 100,000 inhabitants.*

**Table 9-7:** – Numbers and rates of deaths, DALYs, YLLs, YLDs attributable to high LDL-cholesterol levels in 1990 and 2019, and percentage change of rates, by age group, in Brazil.

Deaths						
		1990		2019		Percent change (95% UI)
		Number (95% UI)	Rate (95% UI)	Number (95% UI)	Rate (95% UI)	
15-49 years	11169.2 (10105.5-12136.3)		14.6 (13.2-15.8)	10693.4 (9673.6-11619.4)	9.3 (8.4-10.1)	-36.5 (-40.2--32.6)
50-69 years	28552.5 (23642.8-33735.4)		182 (150.7-215)	37840.3 (31377.7-43975.3)	93.8 (77.8-109)	-48.5 (-51.8--45.1)
05-14 years						
70+ years	28605.4 (18724.9-41561.4)		676.2 (442.7-982.5)	50841.6 (32880.6-75444.8)	388.4 (251.2-576.4)	-42.6 (-47.5--38.6)
Age-standardized			88.6 (67.8-114.8)		43.1 (33.4-55.9)	-51.3 (-53.8--48.6)
All ages	68327.1 (55096.6-83767.8)		45.9 (37-56.3)	99375.3 (78038.6-126142.7)	45.9 (36-58.2)	-0.1 (-7.4-6.7)
Under 5						
**DALYs**						
		**1990**		**2019**		**Percent change (95% UI)**
		**Number (95% UI)**	**Rate (95% UI)**	**Number (95% UI)**	**Rate (95% UI)**	
15-49 years		547499.7 (493892-595058.5)	714.3 (644.4-776.4)	529727.6 (476934.4-574563.7)	458.7 (413-497.5)	-35.8 (-39.3--32)
50-69 years		884741.5 (742881.3-1036354.8)	5639.7 (4735.5-6606.2)	1184341.7 (997186.1-1365623.1)	2935.7 (2471.8-3385)	-47.9 (-51.1--44.7)
5-14 years						
70+ years		389558.3 (259395.2-568128)	9209.3 (6132.2-13430.7)	649071.5 (432523.4-944262.9)	4959.1 (3304.6-7214.4)	-46.2 (-50.3--42.5)
Age-standardized			1940.1 (1614.4-2322.9)		981.3 (817.1-1162.4)	-49.4 (-52--46.8)
All ages		1821799.5 (1548456.3-2139062.9)	1224 (1040.4-1437.2)	2363140.8 (1985655.3-2781317.9)	1090.7 (916.5-1283.7)	-10.9 (-16--6.1)
Under 5						
YLLs						
		**1990**		**2019**		**Percent change (95% UI)**
		**Number (95% UI)**	**Rate (95% UI)**	**Number (95% UI)**	**Rate (95% UI)**	
15-49 years		527862.7 (477315.3-573651.8)	688.7 (622.8-748.4)	498313.6 (450959.6-540989.3)	431.5 (390.5-468.4)	-37.3 (-40.9--33.5)
50-69 years		855877 (719469.5-1001509.1)	5455.7 (4586.2-6384.1)	1123815.9 (947867-1297638.5)	2785.6 (2349.5-3216.5)	-48.9 (-52.2--45.7)
5-14 years						
70+ years		375390.1 (246447.5-550544.6)	8874.3 (5826.1-13015)	608617.9 (406217-887792.4)	4650 (3103.6-6783)	-47.6 (-51.8--44)
Age-standardized			1874.3 (1560.8-2234.9)		925.9 (773.1-1093.9)	-50.6 (-53.2--47.9)
All ages		1759129.8 (1501507.3-2056575.1)	1181.9 (1008.8-1381.8)	2230747.4 (1880846.6-2617317)	1029.6 (868.1-1208)	-12.9 (-18--8)
Under 5						
YLDs						
		**1990**		**2019**		**Percent change (95% UI)**
		**Number (95% UI)**	**Rate (95% UI)**	**Number (95% UI)**	**Rate (95% UI)**	
15-49 years		19637 (12830.1-27604.3)	25.6 (16.7-36)	31414 (20934.9-43414)	27.2 (18.1-37.6)	6.2 (-0.5-13.9)
50-69 years		28864.5 (18492.9-41136.1)	184 (117.9-262.2)	60525.8 (38344.5-87578.5)	150 (95-217.1)	-18.5 (-24.7--12.1)
5-14 years						
70+ years		14168.2 (7112.4-23987.6)	334.9 (168.1-567.1)	40453.6 (21750.9-67619.8)	309.1 (166.2-516.6)	-7.7 (-17.4-3)
Age-standardized			65.7 (42.2-92.3)		55.4 (36.3-77.7)	-15.8 (-20.6--11)
All ages		62669.7 (41368.3-87014.6)	42.1 (27.8-58.5)	132393.4 (87120.9-184088.9)	61.1 (40.2-85)	45.1 (35.5-54.6)
Under 5						

*Source: Data derived from Global Burden of Disease Study 2019, Institute for Health Metrics and Evaluation, University of Washington. ^46^ Rates per 100,000 inhabitants.*

**Table 9-8:** – Age-standardized rates of DALYs attributable to high LDL-cholesterol levels in 1990 and 2019, and percent change of rates, stratified by sex, in Brazil and its Federative Units.

Location	Female			Male		
	1990	2019	Percent change (95% UI)	1990	2019	Percent change (95% UI)
Acre	933.9(730.9-1172.4)	563.1(440-713.7)	-39.7(-48.2--28.8)	1652.5(1305.3-2048.3)	1072.6(861.3-1321)	-35.1(-44.5--23.2)
Alagoas	1400.3(1097.3-1750.5)	983.1(768.6-1227.2)	-29.8(-41.7--14.9)	2166.9(1748.7-2660.8)	1601.9(1262.7-1987.3)	-26.1(-39.5--8.7)
Amapá	863.2(685.6-1082.8)	557.2(444.5-697.8)	-35.5(-43.4--27)	1506.7(1243.3-1837.2)	1055.6(864.5-1291.4)	-29.9(-38.6--20.4)
Amazonas	1084.7(838.7-1405.1)	471.7(360-607.7)	-56.5(-62.8--48.8)	1573.5(1265.9-1945.1)	927.4(722.3-1172.9)	-41.1(-50.9--30.3)
Bahia	1193.9(932.9-1513.8)	677.8(508.4-866.2)	-43.2(-55.6--27.8)	1814(1435.9-2248.1)	1320.7(1009.4-1689)	-27.2(-44--4.8)
Brazil	1425.8(1165.2-1745.1)	692.2(567-842.5)	-51.5(-54.8--47.9)	2496(2090.2-2980.2)	1310.6(1097.4-1543)	-47.5(-50.9--43.8)
Ceará	922(698.3-1185.5)	712.9(526.1-940)	-22.7(-41.2-4.2)	1404.6(1054.1-1827.7)	1263.1(956.6-1661.9)	-10.1(-32.7-23)
Distrito Federal	1520.2(1186.9-1929.7)	544.6(407.7-712.1)	-64.2(-70.5--57.9)	2657(2070.6-3340.4)	1013.4(795.3-1273.3)	-61.9(-67.4--54.6)
Espírito Santo	1382.6(1115.2-1751.2)	725.3(575.8-910.9)	-47.5(-54.9--39.1)	2181.2(1802.6-2663.3)	1347.5(1080.9-1677.9)	-38.2(-48--27.8)
Goiás	1441.1(1105-1866.5)	721.4(555.1-931.4)	-49.9(-60.2--36.6)	2336.7(1833.9-2936.5)	1313.5(1013.1-1659.3)	-43.8(-56.9--26.3)
Maranhão	971.1(720.4-1247.8)	884.4(670.7-1156.5)	-8.9(-30.8-26.9)	3147.1(2354.6-4071.7)	1880.2(1403.5-2520.1)	-40.3(-54.9--19.2)
Mato Grosso	1355.4(1081.9-1688.7)	614.4(480.1-776.8)	-54.7(-61.9--46.1)	2116.8(1663.5-2650)	1083.4(867.6-1325.6)	-48.8(-57.5--37)
Mato Grosso do Sul	1505.9(1226.7-1853.2)	714(558.4-902)	-52.6(-59.1--44.9)	2387.8(1980.9-2867.9)	1338.7(1082.1-1637.9)	-43.9(-52.7--33.9)
Minas Gerais	1549.5(1261.2-1917.9)	601.5(481.6-767.4)	-61.2(-66.7--55.1)	2620.9(2197.5-3117.1)	1075.8(870.3-1303.7)	-59(-65.2--52.1)
Pará	1237.1(959.3-1603.7)	612.2(470.3-777.2)	-50.5(-58.7--40.1)	1992.2(1570.9-2548.5)	1187.9(936-1495)	-40.4(-51.8--26.4)
Paraíba	1196.6(938.8-1467.9)	797.1(621.6-1009)	-33.4(-44.6--18.4)	1724.1(1365.9-2161)	1391.5(1089.9-1748.8)	-19.3(-35.7-1.7)
Paraná	1695.8(1366.5-2120.4)	686.8(532.4-876.2)	-59.5(-65.2--53.6)	2599.9(2167-3137.2)	1268.8(994.8-1596.1)	-51.2(-58--43.1)
Pernambuco	1508.5(1223.5-1864.7)	939(738.7-1166.5)	-37.8(-46.8--28.1)	2311.8(1933.4-2767.6)	1818.3(1446.6-2218.6)	-21.3(-33.9--7.1)
Piauí	1055.1(828.3-1348.6)	662.4(520.5-848)	-37.2(-47.3--25.5)	2056.7(1662.2-2564.9)	1231.8(999.6-1495.1)	-40.1(-49.5--28.7)
Rio de Janeiro	1784.1(1474.1-2152)	792.4(632.7-970.2)	-55.6(-61--49)	3442(2926.8-4060.3)	1622.7(1322-1965.5)	-52.9(-59.2--45.8)
Rio Grande do Norte	996.1(779.7-1245.5)	660.3(494.9-838)	-33.7(-46.7--17.5)	1673.9(1313.8-2102.4)	1309.4(983.6-1656.2)	-21.8(-40.2-1.6)
Rio Grande do Sul	1452(1181.6-1788.3)	656.1(514-845.5)	-54.8(-60.4--48.4)	2481(2041.4-2989.1)	1115.5(880.1-1384.5)	-55(-61.1--48.5)
Rondônia	1767.1(1344.4-2274.7)	702.6(551.9-888.8)	-60.2(-66.9--52.1)	2277.1(1790.4-2862.2)	1224.4(942.4-1536.4)	-46.2(-56.6--32.5)
Roraima	1199.6(947-1536.6)	554.7(430.9-695.3)	-53.8(-59.1--47.9)	2103.1(1689.7-2625.6)	1075.6(865.6-1312.8)	-48.9(-56--40.4)
Santa Catarina	1514.2(1206.4-1898.2)	609.7(474.3-774.7)	-59.7(-65.5--53.8)	2427.5(2005.9-2959.6)	1134.2(907.3-1399.6)	-53.3(-60--45.5)
São Paulo	1547.8(1237-1892.5)	662.1(527.8-827)	-57.2(-62.9--50.7)	2906.3(2421.4-3472.8)	1310.8(1075.3-1602.9)	-54.9(-60.8--47.8)
Sergipe	1149.9(884.9-1477)	745.5(574.6-978.8)	-35.2(-48--18.6)	1693.4(1339.3-2177.8)	1194.7(913.1-1516.8)	-29.4(-44.7--9.5)
Tocantins	1177(907.1-1521.7)	689.2(539.2-869)	-41.4(-52.3--26.8)	1904(1458.8-2435.4)	1415.8(1094.7-1824.6)	-25.6(-41.2--4.2)

*Source: Data derived from Global Burden of Disease Study 2019, Institute for Health Metrics and Evaluation, University of Washington. ^46^ Rates per 100,000 inhabitants.*

**Chart 9-1:** – Change in age-standardized mortality rates due to ischemic heart disease and stroke attributable to high LDL-cholesterol levels between 1990 and 2019.

Source: Data derived from Global Burden of Disease Study 2019, Institute for Health Metrics and Evaluation, University of Washington. ^46^ Rates per 100,000 inhabitants.

**Chart 9-2:** – Correlation between the percent change in age-standardized mortality rate from 1990 to 2019 and the 2019 Sociodemographic Index of each federative unit.

Source: Data derived from Global Burden of Disease Study 2019, Institute for Health Metrics and Evaluation, University of Washington. ^12^ Rates per 100,000 inhabitants.

**Chart 9-3:** – Proportional mortality attributable to high LDL-cholesterol levels by age group, Brazil, 2019.

Source: Data derived from Global Burden of Disease Study 2019, Institute for Health Metrics and Evaluation, University of Washington. ^46^

**Chart 9-4:** – Numbers of deaths and YLLs and age-standardized rates of mortality and YLLs attributable to high LDL-cholesterol levels between 1990 and 2019.

Source: Data derived from Global Burden of Disease Study 2019, Institute for Health Metrics and Evaluation, University of Washington. ^46^ Rates per 100,000 inhabitants.

**Chart 9-5:** – Numbers and age-standardized rates of DALYs and YLDs attributable to high LDL-cholesterol levels between 1990 and 2019.

Source: Data derived from Global Burden of Disease Study 2019, Institute for Health Metrics and Evaluation, University of Washington. ^46^ Rates per 100,000 inhabitants.

### Introduction

• Dyslipidemia is defined as abnormal serum lipid levels, including cholesterol, its subfractions and/or TG. Dyslipidemia is a well-defined risk factor for CVD, in which its causal role has been unequivocally established. ^346^ Its treatment, even in primary prevention, has been shown to effectively reduce CVD. ^347^ Data on mean cholesterol levels and prevalence of dyslipidemia were obtained for adults primarily from the PNS 2015 and for adolescents from the ERICA Study. Smaller prevalence studies (regionally based) were also used when appropriate.

• Throughout this chapter we will usually describe data on TC, LDLc, HDLc, and TG when available. Definitions of dyslipidemia vary historically and according to the positions of local cardiology societies. In this chapter, for the purpose of classification and unless stated otherwise, we will use the term dyslipidemia as follows: for adults, TC ≥ 200 mg/dL, LDLc ≥ 130 mg/dL, HDLc < 40 mg/dL, and TG ≥ 150 mg/dL; ^348^ and for children and adolescents, TC ≥ 170 mg/dL, LDLc ≥ 130 mg/dL, HDLc < 45 mg/dL, and TG ≥ 130 mg/dL. ^349^

### Prevalence

#### Youth

• In the nationwide ERICA Study, Faria Neto *et al* . ^349^ evaluated 38 069 schoolchildren (60% girls) aged 12 to 17 years from the capitals of the 27 Brazilian FUs, in addition to five sets of municipalities with more than 100 000 inhabitants, in all five Brazilian geographic regions. ^349^ Mean TC was 148 mg/dL (95% CI, 147-149 mg/dL), LDLc 85 mg/dL (95% CI, 84-86 mg/dL), HDLc 47 mg/dL (95% CI, 47-48 mg/dL) and TG 78 mg/dL (95% CI, 76-79 mg/dL). Regarding the prevalence of abnormal values, 20.1% (95% CI, 19-21.3%) showed an increase in TC, 3.5% (95% CI, 3.2-4%) in LDLc, and 7.8% (95% CI, 7.1-8.6%) in TG. The prevalence of low HDLc was 47% (95% CI, 45-49%). Such data stratified by age and sex can be seen in Table 9-1 *.*

• For children aged 6 to 12 years, there data is scanty. In a study conducted in Santa Catarina with 1011 students aged 6 to 14 years (52.4% girls), the following mean levels were reported: TC, 172 (± 27) mg/dL in girls, 170 (± 28) mg/dL in boys; LDLc, 104 (± 24) mg/dL in girls, 104 (± 27) mg/dL in boys; HDLc, 49 (± 11) mg/dL in girls, 49 (± 11) mg/dL in boys; and TG, 80 (24-459) mg/dL in girls, 77 (14-752) mg/dL in boys. ^350^

• A study conducted in the city of Vitória with 511 children (age, 6 to 9 years; 46.77% boys) found high TC in 32.7% of them, high LDLc in 9.2%, low HDLc in 27%, and elevated TG levels in 4.1%. ^351^ In the city of Salvador, of 1131 children evaluated (age, 7 to 15 years; 50.1% boys), 25.5% (95% CI, 22.7 – 28.3) were found to be dyslipidemic (TC ≥ 170 mg/dL and/or TG ≥ 130 mg/dL). Dyslipidemia was associated with excess body weight (OR: 3.40; 95% CI, 2.07-5.58) and moderate to high consumption of high-risk food (OR: 1.49; 95% CI, 1.01-2.19). ^352^

#### Adults

• According to a study by Malta *et al* . ^348^ using data from the PNS 2014-2015, in the Brazilian adult population, the prevalence of high TC was 32.7%, of high LDLc, 18.6%, and of low HDLc, 31.8%. In that study, the mean levels were as follows: TC, 185 mg/dL; LDLc, 105 mg/dL; and HDLc, 46 mg/dL. While the prevalence of high TC was higher in women, the prevalence of low HDLc was higher in men. Detailed information on the prevalence of elevated TC and LDLc and of low HDLc stratified by sex, for different age groups, educational attainment, skin color and country region are presented in Tables 9-2 to 9-4. In general, higher levels of education were related to lower prevalence of high TC and LDLc and of low HDLc. Older age groups had higher prevalence of elevated TC and LDLc. Residing in the Southern and Southeastern regions of Brazil were related to lower prevalence of low HDLc. An association between self-reported skin color and lipid profile was less clear, but black women had a lower prevalence of low HDLc. ^348^ Other factors associated with lipid profile changes that have been reported in the Brazilian population include physical activity ^353^ and seasonal variations. ^354^

• The ELSA-Brasil study found the following percentages in women and men, respectively: high LDLc, 57.6% and 58.8%; low HDLc, 20.7% and 14.7%; and hypertriglyceridemia, 23.2% and 40.7%. In addition, the ELSA-Brasil study reported small differences, whose clinical impact seem to be limited, in the lipid profile according to skin color. ^355^

• In 2003, Martinez *et al* . reported on the evaluation of 81 262 individuals (51% male; 44.7 ± 15.7 years) from 13 large Brazilian cities. ^356^ Mean TC was 199.0 ± 35.0 mg/dL and 13% of the sample had a TC above 240 mg/dL.

## Attributable Risk

### Mortality

• The absolute number of deaths and mortality rates countrywide and by FU (including percent change) can be seen in Table 9-5. According to the GBD Study 2019 estimates, between 1990 and 2019, the cardiovascular mortality attributable to high levels of LDLc in Brazil increased in absolute numbers, from 68 327 (95% CI, 55 097 - 83 768) to 99 375 (95% CI, 78 039 - 126 143), but had a 51.3% reduction in the age-standardized rate [88.6 (95% CI, 67.8 - 114.8) to 43.1 (95% CI, 33.4 - 55.9) per 100,000], as a result of population aging. Of the states, Minas Gerais had the highest reduction in mortality rate (-63%) and Ceará, the lowest (-15%).

• Table 9-6 describes the mortality rates attributable to elevated LDLc stratified by sex. For women, the rate went from 72.9 (54.2-97.8) in 1990 to 33.8 (25-45.1) in 2019, a reduction of 53.7% (-56.9 to -50.4) at national level. The greatest reduction was observed in the state of Rondônia (-66.1%) and the smallest, in the state of Maranhão (-1%). For men, the overall percent change was -48.8% (-52.1 to -44.9), from 105.7 (82.4-133.6) in 1990 to 54.2 (42.3-68.4) in 2019, and Distrito Federal had the greatest reduction (-61.9%) and Ceará, the lowest (-10.1%).

• The specific causes of death attributable to high LDLc followed the same trend. Mortality from ischemic heart disease went from 57 020 (95% CI, 46 252 - 68 541) to 83 759 (95% CI, 65 742 - 101 543) and cerebrovascular from 11 306 (95% CI, 5 270 - 21 619) to 15 615 (95% CI, 5 522 - 32 805) with a reduction in the age-standardized mortality rate for both. ^46^ The change in the age-standardized mortality rate due to ischemic heart disease and stroke attributable to high LDLc, from 1990 to 2019, is represented in Chart 9-1.

• The SDI is a composite index that measures per capita income, fertility, education, and sociodemographic development. The SDI allows the comparison of states and countries according to their development. The reduction in age-standardized mortality rate was greater in the FUs with higher SDI (Distrito Federal, São Paulo, Santa Catarina, Rio de Janeiro), and smaller in those with lower SDI, such as the FUs in the Northeastern region (Chart 9-2).

• Absolute numbers of deaths and mortality rates by age group are demonstrated in Table 9-7. There was a reduction in the mortality rate for all age groups. The age group of 50-69 years experienced the highest reduction in the mortality rate (-48.5%). Chart 9-3 depicts the proportion of mortality by age group attributable to high LDLc. This risk factor seems to have greater impact on those aged 40 to 64 years.

### Years of Life Lost

• The same phenomenon observed for mortality rates can be evidenced for the metric of YLLs, which varied in absolute numbers from 1 759 130 (95% CI, 1 501 507 – 2 056 575) to 2 230 747 (95% CI, 1 880 847 – 2 617 317). The age-standardized rate varied from 1874.3 (95% CI, 1561 - 2235) to 926 (95% CI, 773-1094). ^12^ The increase in the number of deaths and YLLs attributable to LDLc and the reduction in the age-standardized rates of both between 1990 and 2019 are shown in Chart 9-4. The absence of a reduction in the mortality rate, when removing the age-standardization, is explained by population aging.

### Burden of Disease

• In addition to the fatal complications of CVD attributable to dyslipidemia, non-fatal complications, such as non-fatal AMI and non-fatal stroke, can be partially attributed to dyslipidemia. The impact of these conditions can be measured by YLDs and DALYs, the latter being the sum of YLLs and YLDs. Between 1990 and 2019, the absolute number of YLDs increased from 62 670 (95% CI, 41 368 - 87 015) to 132 393 (95% CI, 87 121 - 184 089), with rate (per 100,000) ranging from 65.7 (95% CI, 42.2-92.3) to 55.4 (36.3-77.7), a negative variation of 15.8% (Table 9-7). ^46^

• Regarding DALYs, following the same trend of that of mortality, there was an increase in the absolute numbers of DALYs, from 1 821 799 (95% CI, 1 548 456 - 2 139 063) to 2 363 141 (95% CI, 1 985 655 - 2 781 318). This increase was accompanied by a reduction in its age-standardized rate [1940.1 (95% CI, 1614.4 - 2322.9) to 981.3 (95% CI, 817.1 - 1162.4)], a percent change of -49.4%. These changes are illustrated in Chart 9-5.

• Table 9-8 shows the age-standardized DALY rates attributable to high LDLc levels in 1990 and 2019, and the percent change in the period, stratified by sex, in Brazil and FUs. In women, the national rate went from 1425.8 (1165.2 - 1745.1) in 1990 to 692.2 (567-842.5) in 2019, a percent change of -51.5% (-54.8 to -47.9). The greatest change was seen in Distrito Federal, -64.2% (-70.5 to -57.9), and the smallest, in the state of Maranhão, -8.9% (-30.8 to -26.9). For men, the rate of DALYs went from 2496 (2090.2 - 2980.2) to 1310.6 (1097.4 - 1543), with a change of -47.5% (-50.9 to -43.8) in Brazil. The FU with the greatest improvement was the Distrito Federal, -61.9% (-67.4 to -54.6), while the state of Ceará showed the smallest percent change, -10.1% (-32.7 to -23).

• Table 9-7 shows the numbers and rates of deaths, DALYs, YLLs, YLDs attributable to high LDLc in 1990 and 2019, with their respective percent changes, by age group.

### Familial Dyslipidemia

• The prevalence of familial dyslipidemia diagnosed by the Dutch Lipid Clinic Network (DLCN) criteria was assessed in the ELSA-Brasil study, with a documented prevalence of 1 in 263 individuals. This condition was more prevalent in blacks (1 in 156) and brown races (mixed ethnicity; 1 in 204) than in white people (1 in 417). ^357^

• Despite controversies on the use of cascade screening to identify relatives of individuals with familial dyslipidemia, a Brazilian study demonstrated that 59% of the relatives of individuals with mutations were also carriers of such mutations, suggesting a high prevalence of familial dyslipidemia in this selected subgroup. ^358^

### Awareness and Statin Use in Brazil

• An analysis conducted in the ELSA-Brasil study, including 15 096 adults aged 35-74 years, explored the prevalence of high LDLc (according to the NCEP-ATP-III criteria) and the proportion of participants aware of this diagnosis. ^340^ The frequency of participants with elevated LDLc was 45.5%, of which only 58.1% were aware of the diagnosis. Among those participants with elevated LDLc, 42.3% were using some lipid-lowering medications as treatment and 58.3% reached the target defined by the NCEP-ATP-III panel.

• In an analysis based on the PNAUM between 2014 and 2015, the use of statins in SUS primary care in the five Brazilian regions was assessed. ^359^ Among the 8803 respondents, the prevalence of statin use was 9.3%, and 81.4% of these users reported having dyslipidemia. Simvastatin was the most widely used statin (90.3%), followed by atorvastatin (4.7%) and rosuvastatin (1.9%).

• Regarding familial dyslipidemia awareness and treatment, Santos *et al* . reported results from a database of 70 000 individuals undergoing a mandatory employer-sponsored routine health evaluation in a private hospital in São Paulo. ^359^ Among 70 000 patients, 1987 met the established criteria for familial dyslipidemia (LDLc ≥ 190 mg/dL or LDLc ≥ 160 mg/dL on statin). A sample of 200 was selected to complete a questionnaire. From the 200 patients, familial dyslipidemia was suspected by the attending physician in only 29 (14.5%), although most of them (97%) were aware of their high blood cholesterol levels. Only 18% had the perception they were at high risk for CVD, only 30% were aware of their LDLc goals, and 37% were not on lipid-lowering medication.

### Dyslipidemia and Subclinical Atherosclerosis

• Subclinical atherosclerosis, including markers like coronary artery calcium score and carotid intima-media thickness, has been used as surrogate for atherosclerosis and CVD. Thus, its association with abnormal lipid profiles can be of epidemiological interest.

• In a study of more than 3600 individuals, Generoso *et al* . demonstrated that HDLc was associated with coronary artery calcium even after adjustment for traditional cardiovascular risk factors in a Brazilian population. However, this association was no longer significant after adjustment for TG. ^360^ In addition, that study evaluated HDLc subfractions and demonstrated they were not associated with coronary artery calcification once adjusted for total HDLc. Furthermore, the same group showed the association between HDLc and carotid intima-media thickness, and its modification by the presence of diabetes. ^361^

• Laurinavicius *et al* . studied the association between very high HDLc levels and carotid intima-media thickness. ^362^ Very high HDLc likely characterizes hyperalphalipoproteinemia, a dysfunctional HDLc condition. Despite prior evidence, their study did not demonstrate an association between such profile and carotid intima-media thickness. ^362^

• In an analysis of TG-rich lipoproteins in the ELSA-Brasil study, Bittencourt *et al* . demonstrated that those particles are associated with coronary artery calcification even after adjusting for significant risk factors. ^363^

• In a study of octogenarian Brazilians, the authors found that the association between LDLc and coronary artery calcification weakens with age, whereas the association of HDLc does not. ^364^

• Collectively, those studies demonstrate a robust association of lipid profile with subclinical atherosclerosis, corroborating findings of the association between dyslipidemia and CVD.

## Future Research

• Current data on the epidemiology of dyslipidemia in the contemporary Brazilian population is limited. Additional studies on its prevalence in the broad population as well as in specific high-risk groups, such as those with a lower socioeconomic status, are needed.

• The frequency of cholesterol screening in Brazil, according to sex and age groups, needs to be investigated.

• Local Brazilian data on dyslipidemia impact on the healthcare system, including costs, are yet to be addressed.

## 10. OBESITY AND OVERWEIGHT

### ICD-10 E66

**See Tables 10-1 through 10-14 and Charts 10-1 through 10-10**

**Table:** Abbreviations Used in Chapter 10

BMI	Body Mass Index
BMI_i	Imputed Body Mass Index
CI	Confidence Interval
CVD	Cardiovascular Diseases
DALYs	Disability-Adjusted Life-Years
ELSA-Brasil	The Brazilian Longitudinal Study of Adult Health (in Portuguese *, Estudo Longitudinal de Saúde do Adulto* )
ERICA	Brazilian Study of Cardiovascular Risk in Adolescents (in Portuguese, *Estudo dos Riscos Cardiovasculares em Adolescentes* )
FU	Federative Unit
GBD	Global Burden of Disease
HR	Hazard Ratio
*ICER*	Incremental Cost-Effectiveness Ratio
NCD	Noncommunicable Chronic Diseases
OR	Odds Ratio
PNS	Brazilian National Health Survey (in Portuguese, *Pesquisa Nacional de Saúde* )
PR	Prevalence Ratio
QALYs	Quality-Adjusted Life-Years
*RR*	Relative Risk
SABE	Health, Well-Being, and Aging survey (in Spanish, *Salud, Bienestar y Envejecimiento* )
SDI	Sociodemographic Index
SUS	Brazilian Unified Health System (in Portuguese, *Sistema Único de Saúde* )
UI	Uncertainty Interval
VIGITEL	Telephone Survey for Surveillance of Non-Communicable Chronic Diseases (in Portuguese, *Vigilância de Fatores de Risco e Proteção para Doenças Crônicas por Inquérito Telefônico* )
WHO	World Health Organization
YLDs	Years Lived with Disability
YLLs	Years of Life Lost

**Table 10-1:** – Prevalence of excess weight and obesity in the total population aged 18 years and over, by sex and age groups, in Brazil, in 2019.*

Age groups	Prevalence of excess weight and obesity in the population aged 18+ years								
	Total			Sex					
				Male			Female		
	Prevalence %	95% confidence interval		Prevalence %	95% confidence interval		Prevalence %	95% confidence interval	
		Lower limit	Upper limit		Lower limit	Upper limit		Lower limit	Upper limit
**Excess weight**									
Total	60.3	58.3	62.1	57.5	54.8	60.2	62.6	59.1	66.0
18-24 years	33.7	27.4	40.6	25.7	19.1	33.7	41.7	31.1	53.1
25-39 years	57.6	53.1	62.1	58.3	52.3	64.1	57.0	50.0	63.8
40-59 years	70.3	67.4	73.1	67.1	62.1	71.8	73.1	68.8	77.0
60+ years	64.4	60.5	68.1	63.3	56.9	69.2	65.3	60.6	69.7
**Obesity**									
Total	25.9	22.9	29.2	21.8	19.2	24.7	29.5	25.4	34.0
18-24 years	10.7	7.7	14.7	7.9	4.8	12.8	13.5	8.8	20.4
25-39 years	23.7	18.3	30.1	19.3	15.3	24.1	27.9	18.8	39.2
40-59 years	34.4	29.7	39.4	30.2	24.8	36.3	38.0	32.3	44.0
60+ years	24.8	20.9	29.1	21.2	15.6	28.1	27.5	23.0	32.5

* Source: IBGE, Diretoria de Pesquisas, Coordenação de Trabalho e Rendimento, Pesquisa Nacional de Saúde 2019. ^306^

**Table 10-2:** – Percentage of overweight adults, according to an imputation method (BMI_i ≥ 25 kg/m2), by sex, in the capitals of the Brazilian states and the Distrito Federal.*

Capitals / Distrito Federal	Total	95% CI	95% CI	Male	95% CI	95% CI	Female	95% CI	95% CI
		Lower limit	Upper limit		Lower limit	Upper limit		Lower limit	Upper limit
ARACAJU	53.6	50.5	56.7	56	50.8	61.2	51.7	48	55.3
BELEM	53.3	50.1	56.6	53.8	48.3	59.3	53	49.1	56.8
BELO HORIZONTE	52.5	49.7	55.3	57.1	52.7	61.4	48.6	45.1	52.2
BOA VISTA	54.3	49.5	59.2	60.1	52.7	67.5	49	43	55
CAMPO GRANDE	58	54.7	61.3	63.7	58.6	68.9	52.9	48.8	56.9
CUIABA	55.8	52.7	59	58.1	53	63.1	53.8	49.8	57.8
CURITIBA	53.7	50.6	56.9	59.5	54.6	64.4	48.8	44.8	52.8
FLORIANOPOLIS	53.6	50.3	56.8	58.9	53.9	63.8	48.7	44.6	52.8
FORTALEZA	55.6	52.4	58.7	57.7	52.5	63	53.8	50	57.6
GOIANIA	52.7	49.6	55.8	58.3	53.5	63.2	47.8	43.9	51.7
JOAO PESSOA	54.7	51.5	58	56.6	51.2	62.1	53.1	49.2	57
MACAPA	53.3	48.7	57.9	53	45.6	60.4	53.6	48	59.3
MACEIO	54.4	50.9	58	56.6	50.5	62.8	52.6	48.6	56.7
MANAUS	60.9	57.5	64.4	61.1	55.4	66.8	60.8	56.6	65
NATAL	56.6	53.3	59.8	60.8	55.6	66	52.9	48.9	56.9
PALMAS	49.9	46.2	53.6	56.8	50.8	62.7	43.7	39.2	48.1
PORTO ALEGRE	59.2	56	62.3	63	57.9	68.1	56	52	60
PORTO VELHO	56.6	52.9	60.3	62.2	56.6	67.7	50.6	46.1	55
RECIFE	59.5	56.5	62.5	60.4	55.5	65.2	58.8	55.1	62.5
RIO BRANCO	56.6	52.6	60.7	58	51	64.9	55.4	50.8	59.9
RIO DE JANEIRO	57.1	54	60.2	57.9	52.9	63	56.3	52.5	60.1
SALVADOR	51.8	48.6	54.9	47.2	41.9	52.5	55.5	51.8	59.2
SAO LUIS	50.3	46.9	53.7	57.6	51.9	63.2	44.4	40.5	48.3
SAO PAULO	55.8	53	58.6	56.6	52	61.2	55.1	51.6	58.6
TERESINA	52.7	49.5	55.9	56.3	51.1	61.6	49.7	45.8	53.5
VITORIA	49.1	45.8	52.3	50.6	45.3	55.8	47.8	43.8	51.7
DISTRITO FEDERAL	55	51.2	58.9	55.8	49.2	62.5	54.3	50.1	58.6

(*) Inquiries from 2006 to 2019, with weights calculated by the Rake Weighting Method. Consultation: 03/06/2021. Source: Vigitel 2019. ^276^

**Table 10-3:** – Percentage of obese adults, according to an imputation method (BMI_i ≥ 30 kg/m2), by sex, in the capitals of the Brazilian states and the Distrito Federal.*

Capitals / Distrito Federal	Total	95% CI	95% CI	Male	95% CI	95% CI	Female	95% CI	95% CI
		Lower limit	Upper limit		Lower limit	Upper limit		Lower limit	Upper limit
ARACAJU	20.6	18.1	23	18.7	14.7	22.6	22.1	19	25.2
BELEM	19.6	17.1	22.1	20.1	15.9	24.3	19.1	16.1	22.1
BELO HORIZONTE	19.9	17.7	22.2	20.7	17	24.4	19.2	16.5	22
BOA VISTA	21.2	16.8	25.5	24.6	17.1	32.2	17.9	13.5	22.3
CAMPO GRANDE	22.5	19.8	25.1	23	18.8	27.2	22	18.7	25.4
CUIABA	22.5	19.9	25	21.9	17.9	25.9	23	19.6	26.3
CURITIBA	19.4	17	21.8	21.1	17.2	25	17.9	14.9	20.9
FLORIANOPOLIS	17.8	15.5	20.1	18.8	15.1	22.5	16.8	14	19.7
FORTALEZA	19.9	17.5	22.4	18.9	15.1	22.6	20.9	17.6	24.1
GOIANIA	19.5	17.1	21.8	20.6	16.7	24.4	18.6	15.7	21.4
JOAO PESSOA	20.4	17.6	23.2	18.6	14	23.2	21.8	18.4	25.2
MACAPA	22.9	19	26.7	20.4	14.8	26	25.2	19.9	30.4
MACEIO	20	17.3	22.7	17.5	13.2	21.8	22	18.6	25.5
MANAUS	23.4	20.3	26.5	21	16.4	25.6	25.7	21.6	29.7
NATAL	22.5	19.7	25.4	24.3	19.5	29.1	21	17.8	24.2
PALMAS	15.4	12.8	18	16.6	12.2	21	14.3	11.3	17.3
PORTO ALEGRE	21.6	19	24.3	23.2	18.7	27.7	20.3	17.2	23.4
PORTO VELHO	19.9	16.8	23	21.6	16.5	26.7	18	14.7	21.4
RECIFE	21.7	19.2	24.3	19.7	15.8	23.5	23.4	20	26.8
RIO BRANCO	23.3	19.8	26.8	23.3	17.6	28.9	23.4	19	27.7
RIO DE JANEIRO	21.7	19.2	24.2	20.1	16.1	24.1	23.1	19.9	26.3
SALVADOR	18.1	15.8	20.4	15.5	11.7	19.3	20.3	17.5	23
SAO LUIS	17.2	14.2	20.1	18.8	13.4	24.2	15.8	12.8	18.8
SAO PAULO	19.9	17.7	22	18.5	15.1	21.8	21.1	18.3	23.9
TERESINA	17.6	15.3	19.9	17.1	13.5	20.6	18	15.1	21
VITORIA	17.6	15.3	19.9	16	12.3	19.6	19.1	16.1	22
DISTRITO FEDERAL	19.6	16.3	22.8	18.6	13.1	24	20.4	16.5	24.3

(*) Inquiries from 2006 to 2019, with weights calculated by the Rake Weighting Method. Consultation: 03/06/2021. Source: Vigitel 2019. ^276^

**Table 10-4:** – Number of deaths, age-standardized mortality rates due to all causes attributable to high BMI, per 100 000 inhabitants, in 1990 and 2019, and percent change of rates in the period, in Brazil and Federative Units.

Cause of death and Location	1990		2019		Percent change (95% UI)
	Number (95% UI)	Rate (95% UI)	Number (95% UI)	Rate (95% UI)	
**High BMI**					
Acre	80.1 (40.3;128.7)	50.9 (23.5;84.4)	443.6 (305.1;590.6)	75.3 (50.7;102.5)	47.9 (11.7;140.2)
Alagoas	926 (477;1462.1)	69.8 (35.1;111.7)	3244.2 (2234.3;4349.1)	101.1 (69.1;137.2)	44.9 (9;123.8)
Amapá	53.2 (31.5;78.4)	54.7 (30.3;83.8)	365.3 (259.2;478.3)	71.5 (49.3;96.1)	30.6 (6.2;77.4)
Amazonas	525.2 (312.4;765.4)	70.6 (40.5;106.3)	2009.3 (1400.6;2636.9)	71.2 (49;94.7)	0.9 (-17.4;35.7)
Bahia	4395.5 (2318.1;6785.9)	65.3 (33.6;102.3)	13225.3 (8827.3;18489.3)	81.1 (54.1;113.1)	24.1 (-5.4;86.8)
Brazil	74266.2 (43491.7;110056.9)	84.4 (48.1;127.9)	177939.7 (124637.7;237783)	76.2 (52.9;102.1)	-9.7 (-23.1;16.2)
Ceará	1762.6 (831.5;2903.1)	43.8 (20.4;72.5)	7194.6 (4786.1;10036.6)	72.5 (48.2;101.2)	65.4 (19.9;172.1)
Distrito Federal	657.1 (439;908.9)	121.9 (75.5;176.5)	1751.1 (1220.7;2263)	80.6 (54.6;107.6)	-33.8 (-45.2;-14.6)
Espírito Santo	1089 (608.6;1642.8)	75.8 (40.9;117.6)	3588.6 (2472.9;4828.9)	83.7 (57.3;112.7)	10.4 (-13;61.5)
Goiás	1610.1 (830.7;2574.7)	76.8 (38.1;126.3)	5058.6 (3482;6954.6)	73.9 (50.3;101.7)	-3.8 (-29.4;49.2)
Maranhão	1245.2 (489.7;2212.8)	48.2 (18.5;87.4)	5846.2 (3709.8;8423.6)	89 (56;128.6)	84.8 (25.5;250.7)
Mato Grosso	511.1 (276.4;794.5)	63.8 (32.6;101.1)	2471.7 (1734.2;3272.6)	76.8 (52.7;102.4)	20.5 (-9.1;85.8)
Mato Grosso do Sul	746.8 (436.4;1106.5)	83.3 (46.1;128.2)	2223.5 (1521.9;2976.5)	77 (52;103.9)	-7.6 (-26;24.5)
Minas Gerais	7763 (4227.8;11827.3)	79.5 (41.8;123.4)	16660.8 (11377.3;22486.1)	62.7 (42.8;84.8)	-21.1 (-36.5;13.9)
Pará	1320.3 (711.4;2041.6)	65.2 (33.7;103.8)	5128.9 (3498.2;6897.9)	74 (49.8;100.6)	13.5 (-12.8;70.3)
Paraíba	1273.5 (638.4;2048.7)	56.6 (28.1;91.7)	3974.9 (2740.4;5388.3)	82 (56.8;111.2)	45 (9.4;129.3)
Paraná	4468.1 (2563.7;6646.6)	94.2 (52.1;145)	10015.1 (6653;13717.2)	77.1 (50.8;106.2)	-18.2 (-31.9;9.1)
Pernambuco	3345.4 (1772.7;5178.4)	75.4 (38.8;118.8)	9471.3 (6571.7;12641.6)	95.3 (65.8;128.2)	26.3 (0.7;83.6)
Piauí	669.5 (287.7;1133.6)	49.6 (20.4;86.2)	2676.8 (1799.4;3642.1)	70.1 (47.1;94.8)	41.3 (3.1;159.1)
Rio de Janeiro	11589.1 (7176.8;16646.3)	120.7 (72.9;175.6)	19470.2 (13465.9;26105.1)	87.3 (60.2;117.2)	-27.7 (-39.7;-6.8)
Rio Grande do Norte	835.1 (404.9;1341)	53.2 (25.5;85.9)	2990.3 (1997;4137.2)	75.7 (50.4;104.7)	42.2 (4.4;128.1)
Rio Grande do Sul	6158.4 (3741.6;8880.7)	96.3 (56.6;142.5)	11470.3 (7637.2;15714.9)	73.8 (49.1;101.1)	-23.3 (-34.4;-4.2)
Rondônia	306.4 (175.6;451.6)	92.2 (48.4;144.4)	1256.6 (872.4;1680.6)	84.2 (57.6;113.1)	-8.7 (-29.9;33.9)
Roraima	47.4 (27.1;70.9)	84.5 (44.7;131.4)	305.1 (221;394.8)	91 (64.2;119.8)	7.8 (-14.7;58.5)
Santa Catarina	2262.4 (1388.2;3300.1)	93.3 (55.9;139.1)	5617.9 (3838.4;7494)	72.1 (49;96.8)	-22.7 (-35.5;-1.7)
São Paulo	19864.6 (12062.3;28733.9)	102.9 (60.7;151.2)	38343.9 (26476.1;51560.4)	72.4 (49.6;97.8)	-29.6 (-41;-9.3)
Sergipe	547.1 (307.2;815.2)	73.4 (39.6;112.1)	1885.1 (1288.8;2533.3)	84.4 (57.5;114.3)	14.9 (-11.8;65.8)
Tocantins	214 (97.3;356.9)	56.9 (24.3;99.6)	1250.5 (836.2;1702.4)	89.4 (59.9;122.4)	57.1 (11.6;185)

*Source: Data derived from Global Burden of Disease Study 2019, Institute for Health Metrics and Evaluation, University of Washington. ^46^*

**Table 10-5:** – Age-standardized mortality rates due to all causes attributable to high BMI, per 100 000 inhabitants, by sex, in 1990 and 2019, and percent change of rates in the period, in Brazil and Federative Units.

Cause of death and Location	Female			Male		
	1990 Rate (95% UI)	2019 Rate (95% UI)	Percent Change (95% UI)	1990 Rate (95% UI)	2019 Rate (95% UI)	Percent Change (95% UI)
**High BMI**						
Acre	52.3 (27.5;83.3)	69.6 (49;92.1)	33 (0.8;108.3)	50.7 (19.9;90.3)	81.5 (50.6;116.5)	60.9 (18;201.5)
Alagoas	75.5 (40.3;117.1)	101.7 (70.8;137.2)	34.7 (-0.2;108.7)	63.4 (27.3;109.8)	99.3 (60.9;145.1)	56.8 (12.2;174.2)
Amapá	54.4 (31.6;80.9)	67.1 (46.8;88)	23.3 (-2;68.1)	54.7 (27.1;88.3)	75.6 (48.2;106.2)	38.3 (7.6;107.4)
Amazonas	76.4 (45.2;113)	66.4 (45.8;87.1)	-13 (-30.5;17.1)	63.8 (32.7;102.1)	75.5 (49.5;105.1)	18.3 (-10;80.3)
Bahia	70.6 (39.7;108.4)	72 (47.6;101.6)	2 (-24.9;50.3)	59.1 (25.6;101.4)	91.4 (56.3;134.8)	54.7 (5.8;173)
Brazil	83.7 (50.6;122.1)	70.1 (50.5;91.3)	-16.3 (-28.8;6)	84.2 (43.8;131.9)	82.4 (54.2;114.6)	-2.1 (-19;35.5)
Ceará	45.5 (22.1;73.7)	69.9 (46.1;98.7)	53.6 (5.5;166.7)	41.8 (17;73.8)	74.2 (44.2;110.6)	77.4 (17.1;234.1)
Distrito Federal	116.2 (74.3;165.2)	71.8 (48.2;96.6)	-38.2 (-49.9;-20.6)	130.6 (75.3;197.1)	92 (60.1;126)	-29.5 (-45.3;-0.6)
Espírito Santo	77.1 (43.8;116.5)	76.1 (52.3;101.2)	-1.3 (-24.9;43.5)	74 (36.2;119.1)	91.5 (57.7;129.2)	23.7 (-8.1;95.6)
Goiás	80.3 (42.5;128.8)	72 (50.2;97.1)	-10.4 (-35.9;42.1)	73 (31.1;126.2)	75.5 (47.6;109.8)	3.3 (-28.9;78)
Maranhão	37.3 (15.6;65.4)	78 (50.1;113)	109 (39.4;294.2)	63 (21.9;121.2)	102.8 (58.2;158.4)	63.1 (4.4;230.1)
Mato Grosso	65.8 (35.4;102.9)	73 (50.5;96.4)	11.1 (-16.8;70.4)	62.2 (28.6;104)	80.4 (53;111.3)	29.2 (-7.9;117.6)
Mato Grosso do Sul	84.6 (49.5;124.6)	72.3 (49.8;97.8)	-14.5 (-32;16.7)	81.7 (42.3;129.2)	81.5 (52.1;114.8)	-0.3 (-22.7;49.6)
Minas Gerais	82.1 (47.2;122.4)	59.5 (41;79.5)	-27.6 (-42.6;1.4)	75.5 (33.6;124.5)	65.7 (41.5;94.2)	-13.1 (-34.7;47.3)
Pará	65.2 (35.5;102.6)	67.6 (46.6;89)	3.6 (-22.3;58.8)	64.3 (30.1;107.6)	80.3 (50.2;113.2)	24.9 (-9.8;103.8)
Paraíba	62.3 (32.2;97.1)	78.6 (55;105.9)	26.2 (-6.2;105.4)	50.5 (21.7;86.3)	85.3 (54;120.7)	69 (18.3;199.6)
Paraná	95.6 (55.4;142.8)	72.2 (49.2;97.3)	-24.5 (-38.5;2.3)	92.5 (46.2;146.6)	81.8 (49.4;117.3)	-11.6 (-29.7;27)
Pernambuco	79.8 (43.8;122)	87 (59.9;115.6)	9 (-16.1;59.4)	69.8 (31.5;115.5)	104.8 (66.6;145.7)	50.1 (12;143.7)
Piauí	49 (21.8;82)	70.4 (48.4;94.3)	43.8 (3.6;160.8)	50.3 (17.7;93.3)	68.7 (43.1;97.9)	36.5 (-6.1;178.4)
Rio de Janeiro	113.2 (71.2;160.1)	77.6 (55.3;101.3)	-31.5 (-43.8;-10)	128.5 (70.8;196.1)	98.7 (63.2;138.8)	-23.2 (-38.8;5.4)
Rio Grande do Norte	53.2 (26.7;84.4)	68.4 (45.7;94)	28.5 (-7.2;103.3)	53.5 (22.8;92.1)	84 (52.4;123.4)	57 (8.2;180.2)
Rio Grande do Sul	89.1 (55.1;129.2)	67.2 (46.7;90.5)	-24.6 (-37.2;-2.6)	103.7 (57.5;157.1)	80.3 (49.6;114.1)	-22.5 (-36.5;1.2)
Rondônia	105.7 (58.6;159.8)	82.4 (57.4;110.3)	-22 (-40.3;15.8)	81.8 (39.2;136.8)	85.9 (54.4;121.8)	5 (-26.4;73.5)
Roraima	86.5 (47.7;130.6)	92.5 (66.8;120.6)	6.9 (-15.6;59)	82.2 (39.5;133.8)	88.4 (57.3;121.5)	7.6 (-17.9;72.3)
Santa Catarina	93.6 (57.7;135.6)	66.9 (46;88.7)	-28.5 (-41.7;-6.6)	91.8 (49.8;143)	76.5 (47.8;107.1)	-16.7 (-33.4;15.5)
São Paulo	99.9 (62;144.6)	65.5 (45.9;86.5)	-34.4 (-46.3;-14.3)	104.1 (57.6;159.8)	79.6 (51.1;113.1)	-23.5 (-38.8;5.9)
Sergipe	80.5 (45.7;119.6)	81.4 (55.2;111.5)	1.2 (-23.9;46.9)	64 (29.9;105.5)	86.9 (53.4;123.4)	35.8 (-4.1;125.1)
Tocantins	58.7 (26.5;98.5)	81.1 (56.5;108.8)	38 (-4.1;153.9)	54.8 (20.9;100.6)	99.3 (60.2;146.9)	81.2 (22.4;268.4)

*Source: Data derived from Global Burden of Disease Study 2019, Institute for Health Metrics and Evaluation, University of Washington. ^46^*

**Table 10-6:** – Number of deaths, crude and age-standardized mortality rates due to all causes attributable to high BMI, by age group, in Brazil in 1990 and 2019, and percent change of rates in the period.

Age group	1990		2019		Percent Change (95% UI)
	Number (95% UI)	Rate (95% UI)	Number (95% UI)	Rate (95% UI)	
Under 5	20.3 (8;37.7)	0.1 (0;0.2)	5.8 (2.5;10.8)	0 (0;0.1)	-68.8 (-82.5;-39.7)
5-14 years	3.9 (1.9;6.3)	0 (0;0)	3.2 (1.6;5.1)	0 (0;0)	-10.4 (-33.3;23.2)
15-49 years	14678.5 (8877.8;20807.9)	19.2 (11.6;27.1)	19019.1 (14656.4;23409)	16.5 (12.7;20.3)	-14 (-27.5;14.5)
50-69 years	36499.3 (22180.1;53036.6)	232.7 (141.4;338.1)	76441.7 (54807.4;99553.2)	189.5 (135.9;246.8)	-18.6 (-29.6;2.6)
70+ years	23064.2 (11761.5;37147.7)	545.2 (278;878.2)	82469.9 (53033.1;116025.8)	630.1 (405.2;886.5)	15.6 (-5.5;59.7)
Age-standardized	74266.2 (43491.7;110056.9)	84.4 (48.1;127.9)	177939.7 (124637.7;237783)	76.2 (52.9;102.1)	-9.7 (-23.1;16.2)
All Ages	74266.2 (43491.7;110056.9)	49.9 (29.2;73.9)	177939.7 (124637.7;237783)	82.1 (57.5;109.7)	64.6 (42.2;107.4)

**Table 10-7:** – Number of deaths, age-standardized mortality rates due to cardiovascular diseases attributed to high BMI (per 100 000 inhabitants), in 1990 and 2019, and percent change of rates in the period, in Brazil and its Federative Units.

Cause of death and Location	1990		2019		Percent change (95% UI)
	Number (95% UI)	Rate (95% UI)	Number (95% UI)	Rate (95% UI)	
**High BMI**					
Acre	54.3 (26.3;88.1)	32.9 (15.1;55.2)	240.7 (162;324.9)	39.4 (25.6;54.4)	19.7 (-9.2;92.6)
Alagoas	602.6 (298.9;970)	44.4 (21.4;72.2)	1771.2 (1198.6;2438.8)	54.5 (36.5;75.4)	22.7 (-7.3;89.6)
Amapá	35.8 (20.9;53.5)	35.1 (19.3;54.9)	197.1 (135.5;262.4)	36.8 (24.6;50.4)	4.7 (-14.7;42.6)
Amazonas	352.5 (205.6;520.2)	45.9 (25.7;70.8)	998.7 (674.7;1337.4)	34.4 (22.8;46.8)	-25 (-38.9;-0.1)
Bahia	2885.5 (1491.2;4533.3)	42.2 (21.1;67.7)	6924.3 (4434.5;9803.3)	42.3 (26.9;59.8)	0.1 (-24.1;51.2)
Brazil	52646.7 (30085.3;78950.7)	58.5 (32.7;89.7)	98506.9 (66815.9;133940.7)	41.8 (28.1;56.8)	-28.5 (-38.8;-8.6)
Ceará	1165.6 (535.6;1957)	28.6 (13;48.7)	4027.1 (2576.7;5720.1)	40.3 (25.6;57.4)	40.9 (2.3;132.4)
Distrito Federal	474.2 (311.9;658.5)	82.1 (49.6;120.5)	976.1 (663.5;1284.2)	43.3 (28;58.5)	-47.2 (-56.5;-31.5)
Espírito Santo	806.5 (446.2;1222.5)	54.8 (29;85.3)	2070.4 (1400.5;2823.1)	47.8 (32.1;65.4)	-12.9 (-31;26.8)
Goiás	1188 (602.6;1919.7)	54.8 (26.6;91.9)	2871.2 (1930.6;3989.4)	41 (27.3;57.6)	-25.2 (-45.4;15.5)
Maranhão	835.9 (316.7;1531.8)	31.4 (11.6;58.7)	3369.1 (2093.2;4945.6)	50.7 (31;75)	61.6 (9.6;208.8)
Mato Grosso	368.8 (196.9;581.3)	44.2 (22.1;72.3)	1348.7 (914.3;1828.4)	40.8 (26.9;56.1)	-7.9 (-30.1;42.4)
Mato Grosso do Sul	564 (324.8;844.1)	60.9 (33.8;94.8)	1326.7 (903.8;1798.1)	45.2 (30.2;61.7)	-25.7 (-40.2;0.7)
Minas Gerais	5533 (2941.6;8429.9)	55.5 (28.4;87.4)	8966.4 (6002.5;12276.5)	33.7 (22.5;46.3)	-39.2 (-51.4;-12.9)
Pará	947 (500.7;1486.5)	45.6 (23.1;73.8)	2780.4 (1825.6;3803.1)	39.2 (25.4;54.2)	-14 (-33.6;27.9)
Paraíba	798.3 (387.4;1308.8)	35.2 (17.2;58.2)	2099.6 (1385.9;2931.4)	43.5 (28.8;60.4)	23.5 (-7.9;96)
Paraná	3348.7 (1894.5;4987.2)	69.1 (37.4;105.8)	5507.9 (3564.2;7725.2)	42 (27;59)	-39.2 (-49.7;-18.6)
Pernambuco	2291.3 (1176;3613.6)	50.9 (25.4;81.3)	5351.1 (3592.7;7304.5)	53.1 (35.3;73.1)	4.4 (-16.8;51)
Piauí	475.8 (196.1;816.3)	34.5 (13.7;61)	1548.3 (1017.4;2156.1)	40.5 (26.6;56.2)	17.4 (-14.5;118.3)
Rio de Janeiro	8407 (5110.5;12228.9)	86 (51.1;126.6)	10753.6 (7228;14568.8)	48 (32.2;65.1)	-44.1 (-53.2;-27.8)
Rio Grande do Norte	526.5 (245.4;861.5)	33.3 (15.5;55)	1521.9 (977.9;2172.1)	38.4 (24.7;54.6)	15.4 (-15.8;85.6)
Rio Grande do Sul	4455.3 (2668.3;6541.9)	68.1 (39.9;100.4)	6132.7 (3977.2;8434.1)	39.5 (25.7;54.5)	-41.9 (-50.4;-27.6)
Rondônia	221.7 (124.2;331.6)	63.7 (32.7;102.5)	696.4 (475.8;952.7)	45.3 (30.3;62.8)	-28.9 (-45.6;5.2)
Roraima	31.1 (17.3;47.7)	52.7 (26.9;83.6)	150.4 (104.8;199.1)	43.5 (29.1;59.3)	-17.4 (-34.7;24.4)
Santa Catarina	1628.2 (981.8;2402.5)	65.3 (38.5;100)	3055.7 (2029.3;4095.5)	38.6 (25.3;52.5)	-40.9 (-50.6;-23.7)
São Paulo	14175.3 (8410.5;20750.5)	71.6 (40.9;107.2)	22149.9 (15099.6;29984.3)	41.3 (27.9;56.3)	-42.3 (-51.9;-24.9)
Sergipe	320.9 (173.8;492.9)	41.9 (21.9;66.2)	956.8 (634.6;1320.9)	42.1 (27.9;58.6)	0.6 (-23.4;44.7)
Tocantins	152.9 (67.6;258.2)	38.9 (16.1;68.7)	714.7 (463.1;986.3)	50.3 (32.2;70.2)	29.4 (-7.6;134.9)

*Source: Data derived from Global Burden of Disease Study 2019, Institute for Health Metrics and Evaluation, University of Washington. ^46^*

**Table 10-8:** – Age-standardized mortality rates due to cardiovascular diseases attributed to high BMI (per 100 000 inhabitants), by sex, in 1990 and 2019, and percent change of rates in the period, in Brazil and its Federative Units.

Cause of death and Location	Female			Male		
	1990 Rate (95% UI)	2019 Rate (95% UI)	Percent change (95% UI)	1990 Rate (95% UI)	2019 Rate (95% UI)	Percent change (95% UI)
**High BMI**						
Acre	32.4(17;52)	34.5(23.7;46.3)	6.6(-20;65.5)	34.2(13.3;60.8)	44.8(27.4;64.7)	31.1(-5.3;138.8)
Alagoas	44.9(23.6;70.8)	52.1(35.1;70.8)	16(-15.1;81.4)	43.8(18.5;76.5)	56.9(34.2;83)	30(-8.7;125.4)
Amapá	32.7(18.8;49.3)	31.8(21.4;42.2)	-2.8(-22.6;32.1)	37.6(18.6;60.8)	42(26.3;59.5)	11.8(-11.9;66.4)
Amazonas	47.2(27.3;70.1)	29.5(19.7;39.5)	-37.5(-50.1;-15.8)	44.1(22.4;71.6)	39.3(24.8;56)	-10.8(-31.6;35.7)
Bahia	43.7(23.9;68.7)	36(23.1;51.7)	-17.5(-40;22.8)	40.5(17.3;69.9)	49.6(29.8;74.6)	22.5(-16.1;111.6)
Brazil	54.7(32.2;81.1)	36.2(25.4;48.4)	-33.9(-43.7;-16.7)	62.1(32.4;98.6)	47.9(30.4;66.8)	-22.8(-35.9;6.2)
Ceará	27.7(13.2;45.7)	36.3(23.2;51.8)	31.3(-9;128.3)	29.6(11.8;52.6)	44.5(26.2;66.2)	50.2(-0.7;188.2)
Distrito Federal	74.7(46.2;108.6)	37(23.9;51.4)	-50.4(-60.4;-35.8)	92.7(51.8;141.2)	51.3(32.7;72.2)	-44.6(-57.4;-21.5)
Espírito Santo	52.8(29.2;80.6)	40.7(27.5;55.5)	-22.9(-40.7;9.6)	56.8(27.9;91.3)	55.5(35;79.3)	-2.3(-28;52.5)
Goiás	54.8(28.6;89)	37.5(25.4;51.6)	-31.6(-50.7;8.2)	54.7(23.3;93.9)	44.7(27.3;66.5)	-18.2(-44.3;42)
Maranhão	21.9(9.1;39.4)	41.5(26.1;60.2)	89.9(27.4;262.4)	44(14.8;86.5)	62.2(34.7;98.8)	41.4(-8.9;193.3)
Mato Grosso	42.4(22.4;67.5)	36(24.3;49)	-15.1(-36.9;28.3)	45.8(20.9;77.5)	45.3(28.9;64.1)	-1(-29.5;65.5)
Mato Grosso do Sul	58.7(33.7;87.7)	39.5(26.8;53.7)	-32.6(-46.3;-8.6)	62.7(32.3;99.6)	51.2(32.3;72.4)	-18.3(-36.6;21.6)
Minas Gerais	54.2(30.4;81.5)	30.1(20.6;41.2)	-44.5(-55.9;-21.8)	56.3(24.8;94.4)	37.5(23.3;53.8)	-33.3(-50.3;12.1)
Pará	43.3(23.1;69.4)	32.7(21.7;45)	-24.7(-42.7;16.7)	47.3(21.7;79.9)	45.9(28.1;65.8)	-3(-29.4;56.1)
Paraíba	36(18.3;57.7)	39.4(26.9;54.9)	9.4(-19.9;77.9)	34.6(14.5;59.6)	48.2(29.2;69.8)	39.5(-2.3;147.5)
Paraná	66.9(38.1;100.1)	37.1(24.9;51.3)	-44.5(-55.1;-25.5)	71(35.1;112.4)	47.2(28;69.5)	-33.6(-47.6;-4.3)
Pernambuco	50.8(27.5;78.8)	45(30.2;61.9)	-11.5(-31.4;29.2)	50.6(22.3;84.4)	62.9(39.3;89.1)	24.3(-7.4;100.1)
Piauí	31.7(13.8;54.2)	38.3(25.1;53.6)	20.9(-13.4;119.7)	37.7(13.1;70.7)	42.6(25.9;61.8)	13(-21.6;128.8)
Rio de Janeiro	76.5(47.9;110)	40.1(27.7;53.3)	-47.6(-57.2;-31.5)	96.8(53;148.2)	57.7(36.1;81.9)	-40.4(-52.8;-18.7)
Rio Grande do Norte	30.5(14.8;49.7)	31.8(20.4;45.2)	4.2(-24.7;66.3)	36.5(15.5;63.2)	46.1(27.8;68.6)	26.5(-13.5;124.4)
Rio Grande do Sul	60.5(36.6;87.9)	34.4(23.3;47.1)	-43.2(-52.8;-26.6)	76.3(41.7;116.5)	45(27;64.8)	-41.1(-51.6;-22.1)
Rondônia	68.8(36.8;105.7)	41.2(28.1;56.5)	-40.1(-54.7;-11.5)	59.6(28.2;99.8)	49.3(31;71.5)	-17.3(-42;35.9)
Roraima	48.6(26.2;74.9)	38.8(26.2;52.7)	-20.1(-36.9;20)	55.6(26.4;92.2)	47.1(29.3;65.9)	-15.3(-35.3;34.7)
Santa Catarina	62.1(37.4;91.4)	33.8(22.3;47)	-45.6(-56;-27.5)	68.2(36.7;107.4)	43.4(26.7;61.3)	-36.5(-49.7;-12.6)
São Paulo	65.5(39.5;96.3)	35.3(24.2;47.8)	-46.1(-56.1;-29.6)	77.3(41.6;119.3)	47.9(30.7;68.1)	-38(-50.2;-14.6)
Sergipe	42.8(23.7;65.1)	39.3(25.9;55.1)	-8.1(-32.2;34)	40.3(18.6;68)	45.2(26.7;66.2)	12(-21.5;82.9)
Tocantins	37.9(16.6;65.6)	42.1(28.1;58.2)	11.2(-22.3;105.1)	39.5(14.7;73.3)	59.5(35.5;88)	50.5(1.6;202)

*Source: Data derived from Global Burden of Disease Study 2019, Institute for Health Metrics and Evaluation, University of Washington.* ^46^

**Table 10-9:** – Number of deaths, crude and age-standardized mortality rates due to cardiovascular disease attributable to high BMI, by age group, in 1990 and 2019, and percent change of rates in the period, in Brazil.

High BMI	1990 Number (95% UI)	1990 Rate (95% UI)	2019 Number (95% UI)	2019 Rate (95% UI)	Percent change (95% UI)
15-49 years	11876.9 (7042;16974.1)	15.5 (9.2;22.1)	13729.8 (10494.6;16942)	11.9 (9.1;14.7)	-23.3 (-35.3;2.7)
50-69 years	25692.7 (15086.6;37700.4)	163.8 (96.2;240.3)	43871.8 (30231.8;58205.9)	108.7 (74.9;144.3)	-33.6 (-42.5;-16.5)
5-14 years					
70+ years	15077.1 (7374.5;24799.9)	356.4 (174.3;586.3)	40905.3 (25123.3;59857.6)	312.5 (191.9;457.3)	-12.3 (-28.3;20.2)
Age-standardized		58.5 (32.7;89.7)		41.8 (28.1;56.8)	-28.5 (-38.8;-8.6)
All ages	52646.7 (30085.3;78950.7)	35.4 (20.2;53)	98506.9 (66815.9;133940.7)	45.5 (30.8;61.8)	28.5 (11.1;63.2)
Under 5	0 (0;0)	0 (0;0)	0 (0;0)	0 (0;0)	0 (0;0)

*Source: Data derived from Global Burden of Disease Study 2019, Institute for Health Metrics and Evaluation, University of Washington. ^46^*

**Table 10-10:** – Number of DALYs and age-standardized rates of DALYs due to all causes attributable to high BMI (per 100 000 inhabitants), in 1990 and 2019, and percent change of rates in the period, in Brazil and Federative Units.

Cause of death and Location	1990		2019		Percent change (95% UI)
	Number (95% UI)	Rate (95% UI)	Number (95% UI)	Rate (95% UI)	
**High BMI**					
Acre	80.1 (40.3;128.7)	50.9 (23.5;84.4)	443.6 (305.1;590.6)	75.3 (50.7;102.5)	47.9 (11.7;140.2)
Alagoas	926 (477;1462.1)	69.8 (35.1;111.7)	3244.2 (2234.3;4349.1)	101.1 (69.1;137.2)	44.9 (9;123.8)
Amapá	53.2 (31.5;78.4)	54.7 (30.3;83.8)	365.3 (259.2;478.3)	71.5 (49.3;96.1)	30.6 (6.2;77.4)
Amazonas	525.2 (312.4;765.4)	70.6 (40.5;106.3)	2009.3 (1400.6;2636.9)	71.2 (49;94.7)	0.9 (-17.4;35.7)
Bahia	4395.5 (2318.1;6785.9)	65.3 (33.6;102.3)	13225.3 (8827.3;18489.3)	81.1 (54.1;113.1)	24.1 (-5.4;86.8)
Brazil	74266.2 (43491.7;110056.9)	84.4 (48.1;127.9)	177939.7 (124637.7;237783)	76.2 (52.9;102.1)	-9.7 (-23.1;16.2)
Ceará	1762.6 (831.5;2903.1)	43.8 (20.4;72.5)	7194.6 (4786.1;10036.6)	72.5 (48.2;101.2)	65.4 (19.9;172.1)
Distrito Federal	657.1 (439;908.9)	121.9 (75.5;176.5)	1751.1 (1220.7;2263)	80.6 (54.6;107.6)	-33.8 (-45.2;-14.6)
Espírito Santo	1089 (608.6;1642.8)	75.8 (40.9;117.6)	3588.6 (2472.9;4828.9)	83.7 (57.3;112.7)	10.4 (-13;61.5)
Goiás	1610.1 (830.7;2574.7)	76.8 (38.1;126.3)	5058.6 (3482;6954.6)	73.9 (50.3;101.7)	-3.8 (-29.4;49.2)
Maranhão	1245.2 (489.7;2212.8)	48.2 (18.5;87.4)	5846.2 (3709.8;8423.6)	89 (56;128.6)	84.8 (25.5;250.7)
Mato Grosso	511.1 (276.4;794.5)	63.8 (32.6;101.1)	2471.7 (1734.2;3272.6)	76.8 (52.7;102.4)	20.5 (-9.1;85.8)
Mato Grosso do Sul	746.8 (436.4;1106.5)	83.3 (46.1;128.2)	2223.5 (1521.9;2976.5)	77 (52;103.9)	-7.6 (-26;24.5)
Minas Gerais	7763 (4227.8;11827.3)	79.5 (41.8;123.4)	16660.8 (11377.3;22486.1)	62.7 (42.8;84.8)	-21.1 (-36.5;13.9)
Pará	1320.3 (711.4;2041.6)	65.2 (33.7;103.8)	5128.9 (3498.2;6897.9)	74 (49.8;100.6)	13.5 (-12.8;70.3)
Paraíba	1273.5 (638.4;2048.7)	56.6 (28.1;91.7)	3974.9 (2740.4;5388.3)	82 (56.8;111.2)	45 (9.4;129.3)
Paraná	4468.1 (2563.7;6646.6)	94.2 (52.1;145)	10015.1 (6653;13717.2)	77.1 (50.8;106.2)	-18.2 (-31.9;9.1)
Pernambuco	3345.4 (1772.7;5178.4)	75.4 (38.8;118.8)	9471.3 (6571.7;12641.6)	95.3 (65.8;128.2)	26.3 (0.7;83.6)
Piauí	669.5 (287.7;1133.6)	49.6 (20.4;86.2)	2676.8 (1799.4;3642.1)	70.1 (47.1;94.8)	41.3 (3.1;159.1)
Rio de Janeiro	11589.1 (7176.8;16646.3)	120.7 (72.9;175.6)	19470.2 (13465.9;26105.1)	87.3 (60.2;117.2)	-27.7 (-39.7;-6.8)
Rio Grande do Norte	835.1 (404.9;1341)	53.2 (25.5;85.9)	2990.3 (1997;4137.2)	75.7 (50.4;104.7)	42.2 (4.4;128.1)
Rio Grande do Sul	6158.4 (3741.6;8880.7)	96.3 (56.6;142.5)	11470.3 (7637.2;15714.9)	73.8 (49.1;101.1)	-23.3 (-34.4;-4.2)
Rondônia	306.4 (175.6;451.6)	92.2 (48.4;144.4)	1256.6 (872.4;1680.6)	84.2 (57.6;113.1)	-8.7 (-29.9;33.9)
Roraima	47.4 (27.1;70.9)	84.5 (44.7;131.4)	305.1 (221;394.8)	91 (64.2;119.8)	7.8 (-14.7;58.5)
Santa Catarina	2262.4 (1388.2;3300.1)	93.3 (55.9;139.1)	5617.9 (3838.4;7494)	72.1 (49;96.8)	-22.7 (-35.5;-1.7)
São Paulo	19864.6 (12062.3;28733.9)	102.9 (60.7;151.2)	38343.9 (26476.1;51560.4)	72.4 (49.6;97.8)	-29.6 (-41;-9.3)
Sergipe	547.1 (307.2;815.2)	73.4 (39.6;112.1)	1885.1 (1288.8;2533.3)	84.4 (57.5;114.3)	14.9 (-11.8;65.8)
Tocantins	214 (97.3;356.9)	56.9 (24.3;99.6)	1250.5 (836.2;1702.4)	89.4 (59.9;122.4)	57.1 (11.6;185)

*Source: Data derived from Global Burden of Disease Study 2019, Institute for Health Metrics and Evaluation, University of Washington. ^46^*

**Table 10-11:** – Number of DALYs, crude and age-standardized rates of DALYs due to all causes attributable to high BMI in Brazil, in 1990 and 2019, by age group, and percent change of rates in the period.

Cause of death and Location	1990		2019		Percent change (95% UI)
	Number (95% UI)	Rate (95% UI)	Number (95% UI)	Rate (95% UI)	
**High BMI**					
15-49 years	861762.6 (516176.5;1233120.2)	1124.3 (673.5;1608.8)	1370603.6 (1050414.8;1717233.6)	1186.8 (909.5;1486.9)	5.6 (-11.6;44.5)
50-69 years	1295145.7 (795232.8;1864296.5)	8255.8 (5069.2;11883.8)	2994279.6 (2154207.4;3881777.3)	7422 (5339.7;9621.9)	-10.1 (-22.2;13.7)
5-14 years	6200.5 (2476.5;12237.7)	17.5 (7;34.6)	11291 (4692.6;22536.7)	35 (14.6;69.9)	99.5 (53.1;158.3)
70+ years	412245.5 (213001.8;647980.5)	9745.6 (5035.4;15318.4)	1437648.1 (953535.3;1955964.7)	10984.1 (7285.3;14944.1)	12.7 (-7.1;54.6)
Age-standardized		2569.4 (1528.8;3742.3)		2404.5 (1733.3;3121.6)	-6.4 (-19.6;19.1)
All ages	2579849.9 (1556675.2;3720770.6)	1733.4 (1045.9;2499.9)	5817938.7 (4197826.2;7541630)	2685.2 (1937.5;3480.8)	54.9 (33.7;96.5)
Under 5	4495.6 (1661.7;8306.5)	26.5 (9.8;49)	4116.5 (1548;8183.5)	26.6 (10;52.8)	0.1 (-37.3;55.2)

*Source: Data derived from Global Burden of Disease Study 2019, Institute for Health Metrics and Evaluation, University of Washington. ^46^*

**Table 10-12:** – Number of DALYs and age-standardized rates of DALYs due to cardiovascular diseases attributed to high BMI (per 100 000 inhabitants), in 1990 and 2019, and percent change of rates in the period, in Brazil and Federative Units.

Cause of death and Location	1990		2019		Percent change (95% UI)
	Number (95% UI)	Rate (95% UI)	Number (95% UI)	Rate (95% UI)	
**High BMI**					
Acre	1780.2 (889.4;2831.6)	883.9 (440;1418.2)	7206.1 (5100.2;9394.6)	1016.6 (702.7;1344.8)	15 (-11.4;79.5)
Alagoas	18827.9 (9699.9;29802.6)	1258.7 (648.4;1992)	50787.6 (35418;68595.7)	1487.6 (1027.8;2014.5)	18.2 (-10.2;77.2)
Amapá	1192.5 (719.1;1727.5)	957.2 (564.6;1414.6)	6210.3 (4496.9;8037.8)	994.6 (700.1;1313)	3.9 (-14.1;36.2)
Amazonas	11139.6 (6646;16127.6)	1182.8 (693.9;1745.6)	29209.5 (20703.6;38137.7)	898.5 (624.3;1187.5)	-24 (-37.1;-0.6)
Bahia	89200.9 (47500.8;135753)	1206 (642.6;1848.6)	192247.1 (129702.6;260980.6)	1157.8 (781.1;1574.5)	-4 (-27;40.7)
Brazil	1639034.2 (961484.2;2409951.1)	1611.6 (936.5;2376)	2696796.3 (1898493.9;3537093.7)	1108.9 (778.3;1460.7)	-31.2 (-40.5;-12.4)
Ceará	34076.1 (16343.8;55265)	791.3 (381.9;1288.2)	107555.9 (72801.4;147276.2)	1052.9 (706.8;1446.7)	33.1 (-3.5;117.9)
Distrito Federal	16685.1 (11186.2;22749)	2031.2 (1322.9;2849.8)	27936 (19529.6;35968.2)	971 (668.6;1257.6)	-52.2 (-59.9;-39.2)
Espírito Santo	25631.5 (14621.7;37654.9)	1506.1 (843.5;2239.7)	56314.7 (39334.5;74972.3)	1235.5 (864.8;1650.3)	-18 (-34.3;14.2)
Goiás	39404.4 (20135.7;61905.7)	1525.8 (774.6;2442)	81966.4 (56554.9;110604.8)	1089.1 (745.8;1476.4)	-28.6 (-48.1;8.5)
Maranhão	27659.1 (10640.5;49579.6)	950.3 (364.3;1710.5)	95781.1 (62438.9;135543.8)	1378.7 (888.2;1959.2)	45.1 (-3;180.4)
Mato Grosso	12499.7 (6759.1;19413.5)	1201.3 (643.5;1888.1)	39147.3 (27640.9;51142.7)	1069 (743.3;1406.8)	-11 (-31.8;33.7)
Mato Grosso do Sul	18472.6 (10922.8;27010.7)	1676.8 (976.7;2488.9)	36983.9 (26120.2;48494.8)	1188 (836.9;1566.2)	-29.2 (-42.1;-5.4)
Minas Gerais	178307.9 (96726.7;266926.7)	1573.5 (846.8;2377.1)	248509.9 (172433.1;331499.9)	933.9 (649.9;1243.3)	-40.6 (-52.4;-16.3)
Pará	29622.4 (15900.2;45428.6)	1197 (637.6;1854.3)	81046 (55976.2;107682.3)	1049.9 (718;1411.2)	-12.3 (-31.7;28.5)
Paraíba	22695.5 (11404.9;36547.5)	978.7 (497.2;1568)	54880.5 (37966.1;73798.1)	1165.1 (803.2;1567.4)	19 (-10.5;81.8)
Paraná	102453.2 (59672.3;149681.7)	1806.7 (1032.7;2664.8)	146167.1 (99521.6;198031.2)	1063.6 (719.1;1446.9)	-41.1 (-51;-22.6)
Pernambuco	68159.2 (35857.2;104510)	1393.8 (728;2142.4)	149066.6 (103475.9;196806.9)	1418.1 (980.7;1879.4)	1.7 (-18.6;45.6)
Piauí	14484 (6173;24176.3)	930.8 (392.4;1565.3)	40804 (28043.3;54462)	1076.5 (738.6;1442.1)	15.7 (-15.3;109.8)
Rio de Janeiro	265346.7 (163837;376942.1)	2415.5 (1492.4;3462.6)	294309.2 (203915.1;389667.6)	1306.1 (905;1730.1)	-45.9 (-54.6;-32)
Rio Grande do Norte	15080.4 (7424.1;23973.7)	915.4 (450.8;1457.1)	40596.4 (27349.8;55821.9)	1024.7 (691.1;1412.5)	11.9 (-16.1;73.8)
Rio Grande do Sul	134076.3 (82681;189686)	1812.7 (1106.9;2606)	154005.5 (103910.1;205502.3)	1003.2 (683.1;1332.5)	-44.7 (-52.5;-32.3)
Rondônia	7633.3 (4329.7;11286.4)	1575 (856.2;2405.9)	20049.9 (14029.6;26530.5)	1168.3 (810.5;1568)	-25.8 (-42.9;6.3)
Roraima	1085.3 (607.1;1643.6)	1310.2 (715.6;2019)	4525.5 (3246.3;5829.3)	1035.1 (726.4;1359.5)	-21 (-36.8;13.5)
Santa Catarina	48419.9 (29534.7;69616.8)	1659.7 (1004;2418.4)	80459.3 (55267.9;107093.5)	946.7 (647.5;1260.8)	-43 (-52.2;-27.3)
São Paulo	440645.2 (266543;633017.1)	1887.2 (1129.4;2747.7)	604648.5 (420556.1;807155.9)	1086.9 (754.4;1451.7)	-42.4 (-51.5;-25.9)
Sergipe	9519.2 (5300.6;14142.7)	1093.9 (604;1638.1)	26778.2 (18413.5;36053.3)	1120.1 (765.6;1518)	2.4 (-21.7;44.2)
Tocantins	4936 (2229;8219)	996.3 (440.5;1672.6)	19603.7 (13269.9;26463.2)	1283.3 (859.7;1742.7)	28.8 (-7.3;125.2)

*Source: Data derived from Global Burden of Disease Study 2019, Institute for Health Metrics and Evaluation, University of Washington. ^46^*

**Table 10-13:** – Age-standardized rates of DALYs due to cardiovascular diseases attributed to high BMI (per 100 000 inhabitants), by sex, in 1990 and 2019, and percent change of rates in the period, in Brazil and Federative Units.

Cause of death and Location	Female			Male		
	1990 Rate (95% UI)	2019 Rate (95% UI)	Percent change (95% UI)	1990 Rate (95% UI)	2019 Rate (95% UI)	Percent change (95% UI)
**High BMI**						
Acre	1666.7 (945.5;2533)	2215.7 (1633.1;2831.2)	32.9 (5;90.2)	1462.7 (626.1;2473.2)	2477.1 (1665.7;3353.4)	69.4 (26.3;202.2)
Alagoas	2332.8 (1335.3;3446.4)	3140.8 (2273.2;4072.8)	34.6 (4;96.1)	2000.7 (894.3;3324.9)	3218.7 (2106.4;4491.3)	60.9 (16.5;177.9)
Amapá	1739.8 (1088.9;2470.2)	2201.4 (1625;2869.5)	26.5 (4.4;60.9)	1696.2 (917.9;2578.9)	2439.5 (1674.7;3243.9)	43.8 (15.6;105.3)
Amazonas	2213.9 (1411.8;3085.8)	2146.7 (1574.1;2758.1)	-3 (-19.4;23.3)	1937.2 (1052.2;2967)	2412 (1655.5;3272.9)	24.5 (-1.5;80)
Bahia	2273.3 (1364;3342.5)	2393.3 (1694.4;3160.6)	5.3 (-18.2;48.7)	1872.6 (851.1;3100.8)	2828 (1877.7;3973.6)	51 (6.2;161.3)
Brazil	2522.2 (1618.1;3530.4)	2218.9 (1660.5;2838.6)	-12 (-23.8;8.9)	2607.5 (1429.4;3944.7)	2606.2 (1811.8;3456.8)	-0.1 (-16.5;36.8)
Ceará	1445.3 (749.4;2281.7)	2141.3 (1518.3;2869.9)	48.2 (8.6;135.5)	1311.6 (563.9;2209.6)	2384.6 (1546.8;3346.4)	81.8 (25.1;231.1)
Distrito Federal	3223.9 (2167.8;4339.7)	2101.8 (1524.3;2716.4)	-34.8 (-44.7;-20)	3532.8 (2200.7;5211.7)	2622.5 (1830.6;3414.5)	-25.8 (-39.8;-0.9)
Espírito Santo	2299.2 (1369.2;3332.5)	2367.6 (1715.7;3054.1)	3 (-17.2;39.6)	2302.7 (1204.6;3522.3)	2798 (1890.3;3832)	21.5 (-6;84.9)
Goiás	2409 (1360.3;3691.8)	2230.6 (1605;2906.5)	-7.4 (-30.5;36.5)	2267.1 (1034;3714.1)	2441.9 (1619.5;3440.1)	7.7 (-24.4;83.1)
Maranhão	1294.4 (572.5;2152.6)	2534.5 (1733.4;3481.5)	95.8 (33.9;254.2)	1938.3 (708.5;3561.7)	3090.5 (1889.2;4607.6)	59.4 (3.1;222)
Mato Grosso	2039 (1201.2;3066.6)	2322.2 (1696.7;2988.7)	13.9 (-11.8;63.8)	1887 (929;3042)	2551.9 (1798;3417)	35.2 (-0.9;124.5)
Mato Grosso do Sul	2607.8 (1637.8;3665.6)	2345.2 (1719.7;3027)	-10.1 (-26.4;16.3)	2534.7 (1380.1;3834.3)	2644.9 (1803.1;3572.2)	4.3 (-18.1;49.9)
Minas Gerais	2525.3 (1521.6;3620.3)	1987.2 (1443.5;2594.3)	-21.3 (-35.7;5.1)	2368.8 (1119.3;3800)	2178 (1441.1;2993.5)	-8.1 (-30.2;52.3)
Pará	1934.7 (1111.7;2925.6)	2204.2 (1600.9;2887.9)	13.9 (-10.9;63.4)	1924.2 (965.8;3098.7)	2540.5 (1704.3;3421.4)	32 (-1.7;108.5)
Paraíba	1899.2 (1060.3;2852.9)	2439.5 (1774.9;3210.3)	28.5 (-0.8;96.3)	1594.8 (721.9;2636.9)	2742.3 (1819.5;3764.3)	71.9 (23.7;195.3)
Paraná	2714.9 (1685.9;3894)	2208.6 (1595;2896.2)	-18.7 (-32.2;5.6)	2724.5 (1450.3;4154.4)	2551.3 (1664.5;3540.3)	-6.4 (-24.7;32.5)
Pernambuco	2406 (1411.2;3488.8)	2649.2 (1900.4;3456)	10.1 (-12;52.1)	2159.8 (1023.9;3415.9)	3236.4 (2147.8;4376.2)	49.8 (13.8;135.2)
Piauí	1477.2 (720.6;2368.7)	2204.5 (1603.4;2884.7)	49.2 (10.6;150.6)	1504.4 (573.7;2651.8)	2313.5 (1535.8;3212.2)	53.8 (7.4;209.8)
Rio de Janeiro	3436.9 (2263.2;4669)	2455.3 (1792.8;3163.6)	-28.6 (-39.4;-10.7)	3970.5 (2328.9;5904.3)	3083.7 (2101.7;4138)	-22.3 (-36.9;5.4)
Rio Grande do Norte	1726.8 (947.3;2643.6)	2215.7 (1544.3;2943.3)	28.3 (-0.8;91.8)	1660.8 (767;2717.1)	2688.8 (1800.5;3760.4)	61.9 (16.8;171.5)
Rio Grande do Sul	2588.6 (1691.5;3570.9)	2064.1 (1490.8;2671.4)	-20.3 (-31.6;-0.6)	3051.2 (1777.5;4503.7)	2457.2 (1632.3;3370.7)	-19.5 (-32.3;4.3)
Rondônia	2864.4 (1727.9;4164.1)	2500.5 (1825.5;3218.3)	-12.7 (-29.4;17.4)	2306.9 (1185.4;3619.5)	2676.4 (1809.3;3626.4)	16 (-14.5;82)
Roraima	2485.3 (1513.2;3620.5)	2671.9 (1987.3;3404.1)	7.5 (-12.2;45.5)	2285.2 (1167.1;3580.1)	2652.2 (1828.6;3518.3)	16.1 (-11.2;77.4)
Santa Catarina	2588.5 (1693.7;3628.3)	1998.8 (1455.5;2620.4)	-22.8 (-34.8;-3.5)	2643.6 (1500.7;3972)	2338.3 (1570.6;3175.8)	-11.5 (-28.3;22.1)
São Paulo	2837.6 (1833.2;3917.5)	2053.6 (1507.5;2641.7)	-27.6 (-38.7;-9.2)	3171.7 (1783.1;4669.5)	2531.2 (1733.6;3432.3)	-20.2 (-35;8.7)
Sergipe	2331.9 (1433.6;3333.1)	2538.5 (1822.8;3342.3)	8.9 (-15.2;47.5)	1939.7 (981.7;3024.1)	2777.4 (1831.7;3783.5)	43.2 (5;129.3)
Tocantins	1721.5 (846.4;2717.9)	2523.8 (1842.5;3288.3)	46.6 (7.8;144.4)	1554.5 (636;2746.6)	2856.4 (1884.3;3998.6)	83.7 (27.8;251.3)

*Source: Data derived from Global Burden of Disease Study 2019, Institute for Health Metrics and Evaluation, University of Washington. ^46^*

**Table 10-14:** – Number of DALYs, crude and age-standardized rates of DALYs due to cardiovascular disease attributable to high BMI, in 1990 and 2019, by age group, and percent change of rates in the period, in Brazil.

High BMI	1990 Number (95% UI)	1990 Rate (95% UI)	2019 Number (95% UI)	2019 Rate (95% UI)	Percent change (95% UI)
15-49 years	11876.9 (7042;16974.1)	15.5 (9.2;22.1)	13729.8 (10494.6;16942)	11.9 (9.1;14.7)	-23.3 (-35.3;2.7)
50-69 years	25692.7 (15086.6;37700.4)	163.8 (96.2;240.3)	43871.8 (30231.8;58205.9)	108.7 (74.9;144.3)	-33.6 (-42.5;-16.5)
5-14 years					
70+ years	15077.1 (7374.5;24799.9)	356.4 (174.3;586.3)	40905.3 (25123.3;59857.6)	312.5 (191.9;457.3)	-12.3 (-28.3;20.2)
Age-standardized		58.5 (32.7;89.7)		41.8 (28.1;56.8)	-28.5 (-38.8;-8.6)
All ages	52646.7 (30085.3;78950.7)	35.4 (20.2;53)	98506.9 (66815.9;133940.7)	45.5 (30.8;61.8)	28.5 (11.1;63.2)
Under 5					

*Source: Data derived from Global Burden of Disease Study 2019, Institute for Health Metrics and Evaluation, University of Washington. ^46^*

**Chart 10-1:** – Number of deaths due to all causes attributed to high BMI, all ages, from 1990 to 2019. Data from the Global Burden of Disease (GBD) 2019 Study. ^46^

**Chart 10-2:** – Crude and age-standardized mortality rate due to all causes attributed to high BMI, per 100 000, in Brazil, from 1990 to 2019. Data from the Global Burden of Disease (GBD) 2019 Study. ^46^

**Chart 10-3:** – Proportional mortality due to all causes attributed to high BMI, by age group and sex, in Brazil, 2019. Data from the Global Burden of Disease (GBD) 2019 Study. ^46^

**Chart 10-4:** – Age-standardized mortality rates due to diseases attributed to high BMI, stratified by all causes in Brazil, 1990 and 2019. Data from the Global Burden of Disease (GBD) 2019 Study. ^46^

**Chart 10-5:** – Age-standardized mortality rates due to specific causes attributed to high BMI, per 100 000 inhabitants, by sex (women to the left and men to the right), in the Brazilian Federative Units, in 2019. The colored bars represent the specific causes of death according to the legend. Data from the Global Burden of Disease (GBD) 2019 Study. ^46^

**Chart 10-6:** – Correlation between the Sociodemographic Index (SDI) 2019 and the percent change of the age-standardized rates of mortality from cardiovascular disease attributed to high BMI, 1990 - 2019. Data from the Global Burden of Disease (GBD) 2019 Study. ^46^

**Chart 10-7:** – Absolute number of YLLs (A), YLDs (B), and DALYs (C) due to high BMI, all ages, both sexes, Brazil, from 1990 to 2019. Data from the Global Burden of Disease (GBD) 2019 Study. ^46^

**Chart 10-8:** – Rates of YLLs (A), YLDs (B), and DALYs (C) attributed to high BMI for all ages and age-standardized, per 100 000, both sexes, Brazil, from 1990 to 2019. Data from the Global Burden of Disease (GBD) 2019 Study. ^46^

**Chart 10-9:** – Age-standardized rates of DALYs due to specific causes attributed to high BMI, per 100 000 inhabitants, by sex (women to the left and men to the right), in the Brazilian Federative Units, in 2019. The colored bars represent the specific causes of death according to the legend. Data from the Global Burden of Disease (GBD) 2019 Study. ^46^

**Chart 10-10:** – Age-standardized rates of DALYs due to cardiovascular diseases attributed to high BMI, in Brazil, 1990 and 2019. Data from the Global Burden of Disease (GBD) 2019 Study. ^46^

### Overview

• According to the WHO, obesity is defined as abnormal or excessive fat accumulation that presents a risk to health, being present when the BMI is equal to or greater than 30 kg/m^2^. Obesity is a multifactorial condition, related not only to the imbalance between calorie intake and expenditure, which results from a diet rich in high-sugar and high-fat foods, but also to genetic, metabolic, environmental, economic, and sociocultural factors, which eventually lead to the accumulation of excess body fat. In addition to being included in the NCD group, obesity is considered an important risk factor for conditions such as diabetes mellitus, hypertension, and CVD. Obesity is currently considered a pandemic, with impact on both developed and developing countries and consequences for the individual, social, familial, and financial levels. Overweight, defined as BMI over 25 kg/m^2^, is also associated with the complications observed in obese individuals, which increase in parallel with BMI increase. ^365^ Despite the limitations of the use of BMI to assess excess weight, such as its inability to define the amount of body fat that contributes to body weight, ^366^ the WHO currently uses that variable for its definitions.

• The deaths and disease burden attributed to obesity increased globally between 1990 and 2019: from 2.20 (95% UI, 1.21 – 3.43) to 5.02 (95% UI, 3.22 – 7.11) million deaths, and from 67.3 (95% UI, 38 - 104) to 160 (95% UI, 106 - 219) million DALYs in absolute numbers. Obesity contributed to more YLLs [119 million (79.6 - 164)] than YLDs [40.9 million (24.5 - 60.9)] worldwide in 2019. Most of that increase might have resulted from population growth and aging, evidenced after age standardization (4,9% {7,3-24,6} deaths, 18% {2,2- 42,3} DALYs, and 8,3% [-6,6–31,2] YLLs). ^4^

• Obesity contributes to compound most cardiovascular risk factors, particularly to increase blood pressure, blood glucose, and serum lipid levels, and has adverse effects due to serum inflammation and changes in heart structure and function. This association manifests in the relationship existing between obesity and the increased prevalence of hypertension, coronary artery disease, heart failure, and atrial fibrillation. Thus, although there is no clear consensus whether obesity is a disease or a risk factor, this chapter approaches obesity as a cardiovascular risk factor. ^367^

• It is worth noting that, for the purpose of age-standardization of the rates, we considered the global population used by the GBD Study.

### Prevalence

• Table 10-1 shows the prevalence of excess weight and obesity among individuals aged 18 years and over, by sex and age group, in Brazil, in 2019, according to anthropometric data from the PNS. In Brazil, the percentages of adults (age ≥18 years) with excess weight and obesity in 2019 were, respectively, 57.5% (95% CI, 54.8 – 60.2) and 21.8 % (95% CI, 19.2 – 24.7) for men, and 62.6% (95% CI, 59.1 – 66.0) and 29.5% (95% CI, 25.4 – 34.0) for women. Progressive increase of excess weight was observed with age increase, ranging from 33.7% (95% CI, 27.4 – 40.6) [male: 25.7% (95% CI, 19.1 – 33.7); female: 41.7% (95% CI, 31.1 – 53.1)] in the age group of 18-24 years to 70.3% (95% CI, 67.4 – 73.1) [male: 67.1% (95% CI, 62.1 – 71.8); female: 73.1% (95% CI, 68.8 -77.0)] in the age group of 40-59 years. For the age group of 60+ years, there was a slight reduction in the excess weight prevalence, 64.4% (95% CI, 60.5 – 68.1) [male: 63.3% (95% CI, 56.9 – 69.2); female: 65.3% (95% CI, 60.3 – 69.7)]. The same occurred with obesity, a progressive increase with age increase, ranging from 10.7% (95% CI, 7.7 – 14.7) [male: 7.9% (95% CI, 4.8 – 12.8); female: 13.5% (95% CI, 8.8 – 20.4)] in the age group of 18-24 years to 34.4% (95% CI, 29.7 – 39.4) [male: 30.2% (95% CI, 24.8 – 36.3); female: 38.0% (95% CI, 32.3 - 44.0)] in the age group of 40-59 years. For the age group of 60+ years, there was a slight reduction in obesity prevalence, 24.8% (95% CI, 20.9 – 29.1) [male: 21.2% (95% CI, 15.6 – 28.1); female: 27.5% (95% CI, 23.0 -32.5)]. It is worth noting the higher prevalence of excess weight and obesity in the female sex for all age groups.

• Table 10-2 shows the percentage of overweight adults, according to a method of imputation (BMI_i ≥ 25 kg/m^2^), by sex, in the Brazilian capitals and the Distrito Federal, according to Vigitel 2019 data. The capitals Campo Grande, Cuiabá, Fortaleza, Manaus, Natal, Porto Alegre, Porto Velho, Recife, Rio Branco, Rio de Janeiro, and São Paulo showed percentages higher than the national values for both sexes. For men, the capitals Boa Vista, Campo Grande, Cuiabá, Curitiba, Florianópolis, Fortaleza, Goiana, Manaus, Natal, Porto Alegre, Recife, and Rio Branco showed percentages higher than the national values. It is worth noting that, for female excess weight, the number of capitals above the national mean was smaller: Manaus, Natal, Recife, Rio Branco, Rio de Janeiro, Salvador, São Paulo, and Distrito Federal.

• Table 10-3 shows the percentages of adults with obesity, according to a method of imputation (BMI_i ≥ 30 kg/m^2^), by sex, in the Brazilian capitals and the Distrito Federal, according to Vigitel 2019 data. The capitals Aracajú, Boa Vista, Campo Grande, Cuiabá, Macapá, Manaus, Natal, Porto Alegre, Recife, Rio de Branco, and Rio de Janeiro showed percentages higher than the national values for both sexes. For men, the capitals Belém, Belo Horizonte, Boa Vista, Campo Grande, Cuiabá, Curitiba, Goiânia, Macapá, Manaus, Natal, Porto Alegre, Porto Velho, Recife, Rio Branco, and Rio de Janeiro showed percentages higher than the national values. Women had higher percentages of obesity compared to men, contrary to that observed for excess weight. The capitals with female obesity percentage above the national mean were Aracajú, Campo Grande, Cuiabá, João Pessoa, Macapá, Maceió, Manaus, Porto Alegre, Porto Velho, Recife, Rio Branco, Rio de Janeiro, and São Paulo.

• Felisbino-Mendes *et al* ., using data from the GBD study 2017, have reported that the age-standardized prevalence of obesity in Brazil was higher in the female sex (29.8%) compared to that in the male sex (24.6%) in 2017. However, men showed a higher increase of obesity (244.1%) compared to women (165.7%) from 1990 to 2017. An annual increase over 300% was observed in most states of the Northern and Northeastern regions for men, probably due to the delay in the epidemiological transition in those regions. More than half of the population had excess weight in most Brazilian states, except for Maranhão and Piauí. ^368^ These findings are consistent with those indicating higher prevalence of obesity in more challenged populations that migrated from under-nutrition to over-nutrition, contributing to inadequate nutrition around the world. ^369^

• Flores-Ortiz *et al* ., assessing self-reported weight and height data of 572 437 adults of both sexes in the Brazilian capitals and the Distrito Federal, from 2006 to 2016, have estimated the overall prevalence of obesity, which increased from 11.7% to 18.1% in men, and from 12.1% to 18.8% in women, being more marked in the Northern, Northeastern, and West-Central regions. ^370^

• Araújo *et al* ., using Vigitel data from 2008 to 2015, reported an increase in overweight and obesity in Brazilian women of reproductive age, which occurred independently of age, formal education, marital status, race/skin color, and household region (except for women aged 30-39 years, black, and living in the Southern region). Those authors emphasized that, compared to the general population, there was a higher increase of obesity in women aged 18-49 years, in addition to an increase in the prevalence of overweight in women aged 18-29 years in the period, indicating the early occurrence of that risk factor for CVD and NCD. ^371^ It is worth noting that the Vigitel uses self-reported diagnosis.

• A cross-sectional population-based study was conducted in 2002 and 2003 with participants from the “Household inquiry on risk behavior and self-reported morbidity from NCDs”. The study assessed 23 457 individuals aged 15 years or older, living in 16 Brazilian capitals (Aracaju, Belém, Belo Horizonte, Brasília, Campo Grande, Curitiba, Florianópolis, Fortaleza, João Pessoa, Manaus, Natal, Porto Alegre, Recife, Rio de Janeiro, São Paulo, and Vitória). There were 3142 elderly, of whom 1868 (59.4%) were women and 1274 (40.6%) were men. The mean age was 69.5±0.19 years, and 1742 (55.4%) were 60-69 years old. The prevalence of obesity was 17.7% in the age group ‘60-69 years’, 22.9% in the age group ‘70-79 years’, and 17.5% in the age group ‘80+ years’. Obesity was more frequent in women (19.3%; 95% CI, 16.6 - 22.3; χ^2^= 9.5; p = 0.03). ^372^

• A study with 157 postmenopausal women from two public outpatient clinics in the city of São Paulo, Brazil, has reported mean BMI of 28.0 kg/m^2^and 34.4% of obesity, which was classified as class I in 26.1%, class II in 5.7%, and class III in 2.6%. There was a statistically significant association of the overall obesity prevalence with education level (p=0.006), physical activity (p<0.001), use of hormone-replacement therapy in menopause (p=0.007), and number of pregnancies (p=0.002). Abdominal obesity prevalence was 73.8%. The proportion of women with abdominal obesity was higher among those with up to 7 years of schooling (p=0.030). ^373^

• A population-based cross-sectional study using data from the PNS 2013 has reported ideal BMI (<25 kg/m^2^) in 46.8% of the women (95% CI, 45.5 - 48.1), 40.5% of the men (95% CI, 39 - 42), and 43.7% of the total sample (95% CI, 42.7 - 44.7). When stratifying by age group, the ideal BMI prevalence was 54.2% (95% CI, 52.4 - 55.8) in the age group ‘18-35 years’, 36.8% (95% CI, 35.4 - 38.2) in the age group ‘36-59 years’, and 40.9% (95% CI, 38.6 - 43.2) in the age group ‘60+ years’. Ideal BMI was more frequent in the Northeastern region (47.5%) and less frequent in the Southern region (40.4%) of Brazil. ^374^

• The ELSA-Brasil is a cohort of 15 105 voluntary active or retired civil servants from universities or research institutions from 6 Brazilian cities, enrolled from August 2008 to December 2010, aged between 35 and 74 years, composed mostly of women (54%) and middle-aged adults (78% aged <60 years). A sub-study including 14 545 participants aged 35-74 years, mostly (54.1%) women, has shown that 22.7% (n = 3298) of the participants were obese and 40.8% (n = 5934), overweight. In addition, a ‘metabolically healthy status’, according to multiple criteria, was identified in 12.0% (n = 396) of the obese and in 25.5% (n = 1514) of the overweight individuals, being associated with younger age, female sex, lower BMI, and weight change in all BMI categories after the age of 20 years. ^375^ Another sub-study, including 6453 men and 7686 women and assessing the association of BMI and waist circumference with the socio-occupational class, has shown that, for women, the effects of low and intermediate socio-occupational class were greater for those with waist circumference between 80 and 88 cm or overweight, while for men, the low and intermediate socio-occupational class associated with adequate waist circumference or normal BMI. ^376^ Another sub-study assessing the entire cohort has reported a higher prevalence of overweight among men, while obesity was more common among women. Insufficient time for self-care and leisure was associated with overweight (PR = 1.29; 95% CI, 1.04 - 1.61) and obesity (PR = 1.65; 95% CI, 1.28 - 2.12) among women working more than 40 hours/week, and the authors concluded that the results were due to gender inequality involving the relation between time use and health. ^377^ Another study using multilevel logistic regression models adjusted to age, education, skin color, state of residence, individual level, and social cohesion and perceived violence scores has shown that women living in less socially cohesive areas and more violent neighborhoods were more likely to be obese as compared to their counterparts (OR 1.25, 95% CI, 1.02 - 1.53; OR 1.28, 95% CI, 1.04 - 1.56, respectively). ^378^

#### Children and Adolescents

• The increase in obesity prevalence has also been observed among Brazilian children and adolescents. A meta-analysis of 21 studies with 18 463 Brazilian children/adolescents has estimated a 14.1% obesity prevalence, 16.1% for boys and 14.95% for girls, with no significant difference between sexes (PR = 1.06; 95% CI, 0.81 - 1.40; p> 0.05). ^379^

• The ERICA study has assessed 73 399 students, 55.4% of the female sex, with mean age of 14.7±1.6 years. The prevalence of obesity was 8.4% (95% CI, 7.9 - 8.9), lower in the Northern region and higher in the Southern region, and, regarding sex, higher among men. Obese adolescents had a higher prevalence of hypertension, 28.4% (95% CI, 25.5 - 31.2), as compared to overweight adolescents, 15.4% (95% CI, 17.0 - 13.8), and eutrophic adolescents, 6.3% (95% CI, 5.6 - 7.0). The fraction of hypertension attributable to obesity was 17.8%. ^299^

• A systematic review with meta-analysis published by Sbaraini *et al.* in 2021 compiled data from 151 studies on overweight and obesity prevalence in Brazilian adolescents aged 10-19 years. An increase in overweight prevalence was observed in recent decades: 8.2% (95% CI, 7.7 - 8.7) up to 2000, 18.9% (95% CI, 14.7 - 23.2) from 2000 to 2009, and 25.1% (95% CI, 23.4 - 26.8) from 2010 onward, a pattern similar to that of obesity prevalence. The Southeastern and Southern regions had the highest overweight and obesity prevalence, with no difference between sexes. ^337^

• The determinants of obesity prevalence increase include diet changes, environment, greater offer of high-energy food, marketing, urbanization, and reduced time and space for physical activities. ^380^

#### Incidence

• A study derived from the ELSA-Brasil cohort with a 3.8-year follow-up of 13 625 men and women aged 35-74 years, included from 2008 to 2010, showed 7.7% and 10.6% global incidence of overweight and obesity, respectively. The highest percentages were observed among low education level (35.0%) and black (28.5%) women and young men (21.1%). The authors reported overweight increase with age, low per capita income, and fewer years of schooling. ^381^

• Using data from the Vigitel, obesity incidence and persistence were estimated among Brazilian adults from 2006 to 2009. The authors observed that the overweight incidence at the age of 20 years is estimated at 40% for men and 30% for women. The persistence of obesity, however, is estimated at 65% for the male sex and 47% for the female sex. The authors highlight the need to elaborate public policies, particularly for the youth, to reduce dietary risks and promote physical activity. ^382^

#### Mortality

• A meta-analysis of 239 prospective studies has shown a consistent relationship of overweight and obesity (all grades) with all-cause mortality in different populations around the world. The authors hypothesized the possibility of metabolically healthy obese individuals. ^383^

• A study assessing 1450 individuals aged 60 years or more from the Bambuí Cohort Study of Aging has reported the inverse relationship of continuous BMI (HR 0.85; 95% CI, 0.80 - 0.90) with mortality, even after adjusting to confounding variables. Obesity occurred in 12.5% of the elderly and was positively associated with the female sex, higher family income, and the presence of hypertension and diabetes, and inversely associated with physical activity. Overweight was inversely (HR 0.76; 95% CI, 0.61 - 0.93) associated with mortality. Individuals with BMI between 25 kg/m^2^and 35 kg/m^2^had the lowest absolute rates of death at 10-year follow-up. The authors concluded that the usual BMI cut-off points should not be used to guide public policies for the elderly in Brazil. ^384^

#### All-cause Mortality Attributable to High BMI

• Table 10-4 shows the age-standardized rates of mortality due to all causes attributable to high BMI, per 100 000 inhabitants, and percent change of the rates, in Brazil and FUs in 1990 and 2019 (GBD 2019). The greatest decreases in the mortality percentages occurred in the Brazilian states with the highest incomes. Distrito Federal showed the greatest reduction -33.8 (-45.2;-14.6), followed by São Paulo -29.6 (-41;-9.3), Rio de Janeiro -27.7 (-39.7;-6.8), Santa Catarina -22.7 (-35.5;-1.7), Minas Gerais -21.1 (-36.5;13.9), Paraná -18.2 (-31.9;9.1, Bahia -9.7 (-23.1;16.2), Rondônia -8.7 (-29.9;33.9), Mato Grosso do Sul -7.6 (-26;24.5), and Goiás -3.8 (-29.4;49.2). Regarding obesity in men, according to Table 10-5, most FUs had a positive percent change in the death rates due to high BMI, which ranged from 7.6 (-17.9;72.3) in the state of Roraima to 63.1(4.4;230.1) in the state of Maranhão.

• Regarding obesity in women, according to Table 10-5, most FUs had a positive percent change in the death rates due to high BMI, ranging from 1.2 (-23.9;46.9) in the state of Sergipe to 109 (39.4;294.2) in the state of Maranhão, except for Distrito Federal, which showed the greatest reduction -38.2 (-49.9;-20.6), followed by São Paulo -34.4 (-46.3;-14.3), Rio de Janeiro -31.5 (-43.8;-10), Santa Catarina -28.5 (-41.7;-6.6), Minas Gerais -27.6 (-42.6;1.4), Paraná -24.5 (-38.5;2.3), Rio Grande do Sul -24.6 (-37.2;-2.6), Mato Grosso do Sul -14.5 (-32;16.7), Amazonas -13 (-30.5;17.1), Goiás -10.4 (-35.9;42.1), and Espírito Santo -1.3 (-24.9;43.5). The greatest decreases in the mortality percentages in Brazil occurred for women -16.3 (-28.8;6) as compared to men, -2.1 (-19;35.5) (Table 10-5).

• Table 10-6 shows the number of deaths and the crude and age-standardized mortality rates due to all causes attributable to high BMI in Brazil, by age group, in 1990 and 2019, in addition to the percent change of the rates in the period, according to GBD 2019 data. Considering age-standardized rates in the period, there was a percent reduction of -9.7 (-23.1;16.2).

• Chart 10-1 shows the increase in the number of deaths due to all causes attributable to high BMI, in Brazil. Chart 10-2 shows the crude and age-standardized mortality rates due to all causes attributable to high BMI from 1990 to 2019. While the crude rates increased, the age-standardized rates showed stability in the period, suggesting that population aging is the determinant of the increase. According to Brant *et al* ., regarding the ranking of the age-standardized mortality rates due to CVD attributable to risk factors, by sex, in 1990 and 2019, there was an increase in the incidence of high BMI, and that ranking passed from 6th to 3rd in women and from 7th to 4th in men. ^269^

• Chart 10-3 shows the distribution of proportional mortality due to all causes attributed to high BMI, by age group and sex. Proportional mortality attributed to high BMI is higher in the age group ‘50-69 years’ in men and women, with a predominance of women. Age-standardized proportional mortality was 84.4 (48.1;127.9) in 1990 and 76.2 (52.9;102.1) in 2019, with a percent change of -9.7 (-23.1;16.2).

• Chart 10-4 shows the age-standardized mortality rates due to diseases attributed to high BMI, stratified by all causes in Brazil, 1990 and 2019, according to GBD 2019. High BMI contributed mainly to mortality from: ischemic heart disease [number of deaths: 21 732.5 (11 703.8;34 207.7) in 1990 and 45 210.1 (29 102.5;63 084.1) in 2019; proportional mortality: 25.1% (13.2%;40.2%) in 1990 and 19.1 (12.2%;27.0%) in 2019]; stroke [number of deaths: 24 398.5 (14 209.8;35 441.2) in 1990 and 35 124.7 (24 073.7;46 859.7) in 2019; proportional mortality: 25% (14.2%;37.1%) in 1990 and 14.6% (9.9%;19.6%) in 2019]; and diabetes [number of deaths: 10 862.5 (6 997.5;15 055.0) in 1990 and 33 811.0 (24 964.7;43 247.8) in 2019; proportional mortality: 12.2% (7.7%;17.3%) in 1990 and 14.5% (10.5%;18.6%) in 2019]. The contribution to all causes of death was 74 266.2 (43 491.7;110 056.9) and 177 939.7 (124 637.7;237 783.0) in numbers of deaths in 1990 and 2019, respectively.

• Chart 10-5 shows the age-standardized mortality rates due to specific causes attributed to high BMI, per 100 000 inhabitants, in the FU, for all ages, by sex, according to the GBD 2019. Mortality varied in the FUs differently by sex. The highest age-standardized mortality rates (per 100 000 inhabitants) due to CVD and diabetes attributed to high BMI in women were observed in Alagoas (52.1), Pernambuco (45.0), Tocantins (42.1), Espírito Santo (40.7), Maranhão (40.5), Rondônia (40.2), and Rio de Janeiro (40.1). For men, those rates were higher in Pernambuco, Maranhão, Rio de Janeiro, Tocantins, and Alagoas. Felisbino-Mendes *et al* . have reported that, in 2017, obesity accounted for 12.3% of all deaths, making up a total of 165 954 deaths. Proportional mortality attributed to high BMI was more expressive in women (14.6%, 95% UI, 10.7 - 18.9) than in men (10.5%, 95% UI, 7.2 - 14.1). ^368^

• Chart 10-6 shows the correlation between the SDI 2019 and the percent change of the age-standardized mortality rates from CVD attributed to high BMI between 1990 and 2019, for all ages and both sexes. The reduction in mortality from CVD attributed to high BMI correlated with the socioeconomic improvement in the FUs and might have resulted from the late epidemiological transition and competing causes of death in the regions with the lowest income.

#### Mortality from Cardiovascular Diseases Attributable to High BMI

• Tables 10-7 and 10-8 show the number of deaths and age-standardized mortality rates from CVD attributed to high BMI, per 100 000 inhabitants, in 1990 and 2019, and the percent change of rates in the period, in Brazil and its FUs (GBD 2019). Most FUs showed a negative percent change of the rates of death due to high BMI in men, which ranged from -1 (-29.5;65.5) in Mato Grosso to -44.6 (-57.4;-21.5) in the Distrito Federal. The highest decreases in the percent of mortality occurred in the FUs with higher income in Brazil. The highest positive percent changes occurred in the FUs of the Northern [Tocantins 50.5 (1.6;202.0)] and Northeastern [Ceará 50.2 (-0.7;188.2)] regions. In Brazil, there was a negative change in the mortality rates from CVD attributable to high BMI for men [-22.8 (-35.9;6.2)].

• Regarding women, most states had a negative percent change of mortality rates from CVD attributable to high BMI, which ranged from -2.8 (-22.6;32.1) in Amapá to -50.4 (-60.4;-35.8) in the Distrito Federal. The highest decreases in the percent of mortality occurred in the FUs with higher income in Brazil. The highest positive percent changes occurred in the FUs of the Northern [Maranhão 89.9 (27.4;262.4)]. In Brazil, there was a negative change in the mortality rates from CVD attributable to high BMI for women [-33.9 (-43.7;-16.7)], which was higher than that for men [-22.8 (-35.9;6.2)] (Table 10-8).

• Table 10-9 shows the number of deaths, the crude and age-standardized mortality rates from CVD attributed to high BMI in Brazil, in 1990 and 2019, by age group, and the percent change of rates in the period, according to the GBD 2019. Considering the age-standardized rates in the period, the percent change was -28.5 (-38.8;-8.6).

## Burden of disease

### Burden of Disease from All Causes Attributable to High BMI

• Table 10-10 shows DALY in absolute numbers, age-standardized DALY rates (per 100 000 inhabitants) due to all causes of death attributed to high BMI, and the percent change of rates in Brazil and FUs in 1990 and 2019 (GBD 2019). Regarding the burden of disease in 2019, high BMI accounted for 177 939.7 (124 637.7;237 783) DALYs [76.2 (52.9;102.1) per 100 000 inhabitants]. The percent change was -9.7 (-23.1;16.2) in relation to 1990, for when 74 266.2 (43 491.7;110 056.9) DALYs [84.4 (48.1;127.9) per 100 000 inhabitants] were estimated. The FUs of the Northern [Maranhão 84.8 (25.5;250.7)] and Northeastern [Ceará 65.4 (19.9;172.1)] regions had the highest positive percent changes.

• Table 10-11 shows the number of DALYs, the crude and age-standardized DALY rates due to all causes attributed to high BMI in Brazil, in 1990 and 2019, by age group, and the percent change of the rates in the period, according to GBD 2019 data. The highest positive percent change occurred in children and adolescents, from 5 to 14 years [99.5 (53.1;158.3)].

• Charts 10-7 and 10-8 show the graphic representation of the absolute numbers and the age-standardized and all age rates of YLLs, YLDs, and DALYs due to high BMI, from 1990 to 2019, in Brazil, respectively. It is worth noting the growth trend of all absolute numbers and crude rates per 100 000 inhabitants. However, the age-standardized rates of DALYs and YLLs showed a trend towards decrease in the period, while those of YLDs increased, suggesting a growing impact on morbidity due to obesity even regardless of population aging. Felisbino-Mendes *et al* . have estimated that the DALYs, for both sexes, increased by 96% and 42% due to population aging and growth, respectively, and by 130% due to change in exposure to risk. ^368^

• Chart 10-9 shows the age-standardized DALYs rates due to specific causes attributed to high BMI, per 100 000 inhabitants, in the FUs, for all ages, by sex, according to GBD 2019. The DALYs varied in the FUs differently by sex. Diabetes and kidney diseases were the second major causes of DALYs attributed to high BMI, being preceded by CVD. The highest age-standardized rates of DALYs due to diabetes and kidney diseases attributed to high BMI in women (per 100 000 inhabitants) were observed in Alagoas (1320.1), Roraima (1250.8), Sergipe (1253.6), Pernambuco and Maranhão (1013.5), Paraíba (990.4), Rondônia (989.3), and Tocantins (981.7). Regarding men, those rates (per 100 000 inhabitants) were higher in Alagoas (1263.9), Sergipe (1168.1), Maranhão (1130.8), Pernambuco (1104.7), Bahia (1086.2), Roraima (1071.7), Paraíba (1047.4), Rio de Janeiro (1042.5), and Rio Grande do Norte (1024.5).

### Burden of Cardiovascular Disease Attributable to High BMI

• Regarding burden of disease in 2019, high BMI accounted for 177 939.7 (95% UI, 124 637 – 237 783) DALYs due to CVD [76.2 (95% UI, 52.9 – 102.1) per 100 000 inhabitants], with higher relevance to ischemic heart disease [45 210.1 (95% UI, 29 102.5 – 63 084.1)] and stroke [35 124.7 (95% UI, 24 073.7 – 46 859.7)]. In Brazil, this risk factor contributed, in 2019, to 5 817 938.7 (95% UI, 4 197 826.2 – 7 541 630) DALYs [2404.5 (95% UI, 1733.3 – 3121.6) per 100 000 inhabitants]. The percent reduction was -6.4 (95% UI, -19.6 to 19.1) in relation to 1990, for when 2 579 849.9 (95% UI, 1 556 675.2 – 3 720 770.6) DALYs [35 124.7 (95% UI, 24 073.7 – 46 859.7)] were estimated (Chart 10-10).

• Tables 10-12 and 10-13 show the age-standardized rates of DALYs due to CVD attributed to high BMI, per 100 000 inhabitants, in 1990 and 2019, and the percent change of rates in the period, by sex, in Brazil and FUs (GBD 2019). Most FUs had a decrease in the DALYs for women in the period, which was more marked in the Distrito Federal [-34.8 (-44.7;-20)], Rio de Janeiro [-28.6 (-39.4;-10.7)], São Paulo -27.6 (-38.7;-9.2)], Santa Catarina [-22.8 (-34.8;-3.5)], Minas Gerais [-21.3 (-35.7;5.1)], and Rio Grande do Sul [-20.3 (-31.6;-0.6)]. The state of Maranhão had the highest increase between 1990 and 2019 [95.8 (33.9;254.2].

• Similar behavior was observed for men, with a percent decrease of obesity from 1990 to 2019. The highest percent decreases were observed in the Distrito Federal [-25.8 (-39.8;-0.9)], Rio de Janeiro [-22.3 (-36.9;5.4)], São Paulo [-20.2 (-35;8.7)], Rio Grande do Sul [-19.5 (-32.3;4.3)], Santa Catarina [-11.5 (-28.3;22.1)], and Minas Gerais [-8.1 (-30.2;52.3)], smaller than those observed for women. Ceará had the highest increase between 1990 and 2019 [81.8 (25.1;231.1)]. In addition, the highest percent increases were observed for women in the period (Table 10-13). It is worth noting that the same pattern was observed for Brazil as a whole, with higher percent decrease for women [-12 (-23.8;8.9)] as compared to men [-0.1 (-16.5;36.8)].

• Table 10-14 shows the number of DALYs, the crude and age-standardized rates of DALYs due to CVD attributed to high BMI in Brazil, in 1990 and 2019, by age group, and the percent change of the rates in the period, according to data from GBD 2019. The highest negative percent change occurred in the age group ‘50-69 years’ [-33.6 (-42.5;-16.5)]. There was a percent reduction of -31.2 (-40.5;-12.4) regarding the age-standardized rates in the period (Table 10-12).

## Impact on Cardiovascular Health

• High BMI causes chronic systemic inflammation and high sympathetic activity, which can contribute to insulin resistance and hypertension, leading to endothelial dysfunction and atherosclerosis, contributing to diabetes mellitus. Its effect is mainly mediated by other intermediate risk factors, such as hypertension, hypercholesterolemia, and hyperglycemia. ^385^ Obesity has a multifactorial nature and is one of the major factors to explain the increase in NCD because of its frequent association with CVD, such as arterial hypertension, stroke, heart failure, dyslipidemia, diabetes, atrial fibrillation, and sudden death. The interventions that reduce hypertension, and cholesterol and glucose levels could solve half of the risk for coronary artery disease and three-quarters of the risk for stroke associated with high BMI. ^386^

• Rimes-Dias and Canella have hypothesized that the NCDs associated with obesity usually require drug treatment and carried out a study with data from the PNS 2013, with 59 402 individuals aged 18 years or older. They assessed the number of medications used to treat nine NCDs related to obesity (arterial hypertension, CVD, stroke, diabetes, arthritis and rheumatism, chronic kidney disease, lung disease, chronic low back pain, and depression). The use of medications increased progressively with BMI increase. The risk of having to use medications to treat two or more NCDs was 70% greater among overweight individuals (adjusted RR = 1.66; 95% CI, 1.46 – 1.89), 170% greater among those with class I obesity (adjusted RR = 2.68; 95% CI, 2.29 – 3.12), 340% greater among those with class II obesity (adjusted RR = 4.44; 95% CI, 3.54 – 5.56), and 450% greater among those with class III obesity (adjusted RR = 5.53; 95% CI, 3.81 – 8.02) as compared to normal-weight individuals. ^387^

• A cross-sectional study, assessing the presence of cardiovascular risk factors according to the pattern of body fat distribution, was conducted with 113 Brazilians aged 80 years or more (mean age, 83.4 years), of both sexes, recruited from 2009 to 2010 in the city of Presidente Prudente, São Paulo state, as part of the SABE survey. The authors reported the association of abdominal and total obesity with risk factors for CVD, such as higher levels of total cholesterol and triglycerides. There was a significant association of arterial hypertension and total obesity. ^388^

• A cohort of 12-month duration, assessing 89 adolescents aged 11-14 years in the city of Presidente Prudente, has shown, after adjusting to gender, age, biological maturation, and physical activity, that the changes in femoral intima-media thickness associate with changes in body fat: for each percent increase in body fat, the femoral intima-media thickness increased by 0.007 mm. ^389^

## Risk Factors and Prevention

• Rabacow *et al* . have estimated the proportion of deaths from NCDs that could be reduced in Brazil by reducing population-wide BMI by using data from the PNS 2013. The population-wide BMI reduction to a theoretical minimum risk exposure level (22.0 kg/m^2^) could prevent approximately 168 431 deaths per year in Brazil, of which 106 307 from CVD. The reduction to a BMI level of 24.6 kg/m^2^could prevent 65 721 deaths, representing 10% of the deaths from NCDs and 5.8% of all deaths. A reduction by 1.0 kg/m^2^in BMI at population level could prevent 30 715 deaths, representing 4.6% of the deaths from NCDs and 2.7% of all deaths. This data set emphasizes the need for adopting anti-obesity measures at national level. ^390^

• A study, analyzing data from the Household Budget Survey 2002/2003 and 2008/2009 and from the PNS 2013, with 234 791 adults aged 20-59 years, has shown that the highest education levels were associated with obesity and excess weight for men, and the middle school level was associated with an increase in obesity for women. The authors concluded that the findings reveal the need for nutritional education, in addition to campaigns and policies to hold the obesity epidemic. ^391^

## Health Care Utilization and Cost

• A study assessed the 1-year total direct costs associated with the outpatient and inpatient care to overweight- and obesity-related diseases, from the perspective of the SUS, in Brazil, between 2008 and 2010. The values were estimated at US$ 2.1 billion, of which US$ 1.4 billion (68.4% of the total costs) due to hospitalizations and US$ 679 million due to outpatient procedures. Using the population attributable risk, the authors reported that approximately 10% of those costs were attributable to overweight and obesity. The costs for women were higher than those for men, because of higher outpatient clinic expenses (73.3% vs. 26.7%). ^392^ Another study has estimated the total direct cost of overweight/obesity at 3.02% of the total costs with hospitalization for men and 5.83% for women, corresponding to 6.8% and 9.3% of all hospitalizations (excluding pregnant women), respectively. ^393^

• A study has assessed the cost-utility of gastric *bypass* surgery as compared to clinical treatment for severe obesity with and without diabetes from the SUS perspective. A 10-year time horizon and a 5% discount rate were considered in the Markov model. Over 10 years, gastric *bypass* surgery increased the QALYs and costs as compared to clinical treatment, resulting in an ICER of Int$ 1820.17/QALY and Int$ 1937.73/QALY for individuals with and without diabetes, respectively. The sensitivity analysis showed that the utility values and direct costs of the treatments were the parameters that affected most the ICER. The authors concluded that gastric *bypass* surgery has a good cost-benefit ratio for severely obese individuals from the SUS perspective, with better results for obese individuals with diabetes. ^394^

• A study concluded that the total costs of hypertension, diabetes, and obesity in the SUS reached R$ 3.45 billion (95% CI, 3.15 – 3.75) in 2018 (US$ 890 million). Regarding obesity as a risk factor for hypertension and diabetes, the attributable costs reached R$ 1.42 billion (95% CI, 0.98 – 1.87), representing 41% of the total costs. ^395^

• A micro-simulation model was used to project the extent of obesity and obesity-related diseases and associated healthcare costs in Brazil by 2050. The model considered 13 diseases (coronary artery disease, stroke, hypertension, diabetes, osteoarthritis, and 8 types of cancer), simulating 3 hypothetical intervention scenarios: no intervention, and 1% and 5% reduction in BMI. The authors estimated that the healthcare costs will double by 2050 (US$ 10.1 billion), reaching US$ 330 billion in the 2010-2050 period. However, effective interventions, such as 1% and 5% reductions in mean BMI across the population, can reduce costs to US$ 302 billion and US$ 273 billion, respectively. ^396^

## Future Research

• Gaps in primary data on mortality attributable to high BMI in Brazil and its FUs have been observed. Nationwide registries with measured data should be built to enable the development of more effective public policies to control obesity, which has been increasing in Brazil in both sexes and several age groups.

• Most public policies have failed to reduce obesity in adults and children, probably because obesity is multifactorial and involves many socioeconomic interests. It is worth emphasizing the role played by the food industry, which includes the offer of ultra-processed foods at lower costs, the absence of communication about the risks associated with overweight and obesity in social medias, in addition to the lifestyle in the big cities, where children are increasingly sedentary and eat high-calorie diets. Future studies with multifactorial interventions involving the whole family need to be conducted, to benefit mainly children and adolescents. ^397^

• The patient-centered care for obesity requires future research and is an essential component of high-quality healthcare that can improve clinical outcomes and patient satisfaction, reducing healthcare costs. A study has suggested the following topics for obesity research: development of research agendas that emphasize the study of tools and techniques of patient-centered healthcare; prioritization of the analysis of patient’s barriers that prevent weight loss and assessment of tools to overcome such barriers; assessment of cultural and environmental factors that can affect one’s ability to lose weight; and implementation of evidence-based metrics for obese patients. ^398^

• More recent data have highlighted abdominal obesity, assessed by waist circumference, as a CVD risk marker that is independent of BMI. Studies quantifying fat depots, including ectopic fat, with imaging methods have shown that excess visceral adiposity is an independent indicator of poor cardiovascular outcomes. Studies assessing abdominal obesity in the Brazilian population need to be performed to investigate its incremental role in cardiovascular risk stratification in both sexes and several age groups. ^399^

## 11. SMOKING AND TOBACCO USE

### Smoking and the consequences for cardiovascular diseases in Brazil and Federative Units, 1990 to 2019

ICD-10: Z.72.0

**See Tables 11-1 through 11-13 and Charts 11-1 through 11-12**

**Table:** Abbreviations Used in Chapter 11

ANVISA	Brazilian National Health Surveillance Agency (in Portuguese, *Agência Nacional de Vigilância Sanitária* )
DALYs	Disability-Adjusted Life Years
NCDs	Noncommunicable Chronic Diseases
CVD	Cardiovascular Diseases
ELSA-Brasil	The Brazilian Longitudinal Study of Adult Health (in Portuguese *, Estudo Longitudinal de Saúde do Adulto* )
ERICA	Brazilian Study of Cardiovascular Risk in Adolescents (in Portuguese, *Estudo dos Riscos Cardiovasculares em Adolescentes* )
FU	Federative Unit
GBD	Global Burden of Disease
PeNSE	National Survey of Schoolchildren Health (in Portuguese: *Pesquisa Nacional de Saúde do Escolar* )
PNS	Brazilian National Health Survey (in Portuguese, *Pesquisa Nacional de Saúde* )
SDI	Sociodemographic Index
Vigitel	Telephone Survey for Surveillance of Non-Communicable Chronic Diseases (in Portuguese, *Vigilância de Fatores de Risco e Proteção para Doenças Crônicas por Inquérito Telefônico* )
YLDs	Years Lived with Disability
YLLs	Years of Life Lost

**Table 11-1:** – Percentage of 9th grade students who experimented with cigarettes, by sex and type of school, in Brazil, its regions and Federative Units.

Major regions and Federative Units	Total			Sex								Type of school						
				Male				Female				Public				Private		
	Total	95% UI		Total	95% UI			Total	95% UI			Total		95% UI		Total	95% UI	
		Lower limit	Upper limit		Lower limit		Upper limit		Lower limit		Upper limit			Lower limit	Upper limit		Lower limit	Upper limit
Brazil	18.4	17.8	19.0	19.4	18.7	20.2	17.4	16.6			18.2	19.4		18.7	20.0	12.6	11.6	13.6
North	20.1	18.8	21.3	22.7	21.1	24.3	17.6	16.2			18.9	20.8		19.5	22.2	11.8	8.9	14.6
Rondônia	21.5	18.9	24.2	22.4	18.8	26.0	20.7	18.0			23.4	21.9		19.1	24.7	16.1	11.6	20.7
Acre	26.2	23.7	28.6	28.3	25.6	31.1	24.0	21.0			27.0	27.1		24.5	29.6	12.1	5.8	18.4
Amazonas	21.1	19.1	23.0	23.3	20.8	25.7	18.9	16.7			21.1	21.9		19.9	24.0	9.5	3.2	15.7
Roraima	28.2	25.3	31.2	30.6	27.5	33.7	25.8	21.9			29.6	29.2		26.1	32.3	12.4	1.5	23.3
Pará	18.7	16.3	21.1	22.3	19.1	25.5	15.5	13.0			18.1	19.4		16.9	22.0	12.3	7.7	16.9
Amapá	21.5	19.5	23.6	22.4	20.2	24.7	20.7	18.1			23.2	22.5		20.3	24.6	12.5	7.3	17.8
Tocantins	15.1	12.1	18.1	17.1	13.2	21.1	13.1	10.4			15.8	15.6		12.3	18.9	8.6	5.2	12.1
Northeast	14.2	13.5	14.9	16.3	15.4	17.3	12.4	11.6			13.3	15.1		14.3	15.9	9.3	8.3	10.4
Maranhão	11.9	10.6	13.2	15.4	13.4	17.5	9.0	7.5			10.4	12.2		10.8	13.6	8.8	6.0	11.6
Piauí	12.8	11.3	14.3	15.5	13.1	17.8	10.5	8.8			12.2	13.3		11.7	15.0	9.4	6.4	12.5
Ceará	18.7	16.5	20.9	19.6	17.1	22.2	17.7	14.9			20.5	19.9		17.4	22.4	11.7	8.8	14.6
Rio Grande do Norte	10.9	9.6	12.1	13.3	11.3	15.3	8.7	7.3			10.2	11.7		10.3	13.2	7.1	5.1	9.1
Paraíba	15.3	13.6	17.1	16.6	14.4	18.8	14.3	12.2			16.4	16.4		14.3	18.5	10.5	8.7	12.4
Pernambuco	14.0	12.4	15.5	14.7	12.6	16.8	13.2	11.2			15.3	15.0		13.1	16.9	9.3	7.4	11.2
Alagoas	13.0	10.9	15.0	13.6	11.5	15.8	12.4	9.7			15.1	13.5		11.1	15.9	11.0	7.6	14.3
Sergipe	9.3	8.1	10.5	11.4	9.5	13.2	7.7	6.5			8.9	9.9		8.5	11.2	7.3	4.8	9.8
Bahia	14.6	12.7	16.4	17.8	15.2	20.5	12.0	9.8			14.2	15.6		13.5	17.7	8.0	4.8	11.2
Southeast	18.3	17.1	19.4	18.2	16.7	19.6	18.4	16.7			20.0	19.2		17.9	20.5	13.5	11.6	15.4
Minas Gerais	17.6	15.5	19.6	17.4	15.2	19.6	17.7	15.2			20.3	18.5		16.3	20.7	9.1	4.9	13.3
Espírito Santo	17.6	15.5	19.7	18.3	15.7	20.9	16.9	14.5			19.4	18.3		15.9	20.6	13.4	9.9	16.9
Rio de Janeiro	16.6	14.8	18.3	15.3	13.2	17.4	17.7	15.4			20.1	18.0		15.8	20.2	13.0	10.2	15.7
São Paulo	19.2	17.4	21.1	19.5	17.1	21.9	19.0	16.3			21.7	20.0		17.9	22.1	15.1	12.0	18.3
South	24.9	23.5	26.3	25.2	23.4	27.0	24.6	22.7			26.4	26.0		24.5	27.5	14.9	12.2	17.7
Paraná	25.5	23.2	27.7	26.4	23.8	29.0	24.4	21.4			27.4	27.4		24.9	30.0	12.0	8.2	15.8
Santa Catarina	22.1	19.5	24.6	24.2	20.9	27.6	20.2	16.9			23.4	22.8		20.0	25.6	16.2	11.9	20.6
Rio Grande do Sul	26.4	24.0	28.8	24.1	20.3	27.9	28.7	25.4			31.9	26.7		24.2	29.1	22.2	15.3	29.2
West-Central	22.1	20.9	23.2	24.2	22.6	25.7	20.0	18.6			21.4	22.9		21.6	24.1	17.5	15.5	19.5
Mato Grosso do Sul	27.0	24.9	29.2	29.4	26.7	32.1	24.7	21.9			27.6	28.2		25.8	30.6	15.5	13.4	17.6
Mato Grosso	23.2	21.0	25.4	25.6	22.2	29.1	20.7	18.0			23.3	23.9		21.5	26.2	15.5	10.7	20.4
Goiás	18.8	17.1	20.5	20.2	18.2	22.2	17.4	15.0			19.8	19.1		17.2	21.0	17.1	13.9	20.3
Distrito Federal	23.7	20.8	26.6	26.8	22.6	31.0	20.9	18.0			23.9	25.2		21.6	28.7	19.4	15.7	23.0

*Source: IBGE, PeNSE 2015. ^410^*

**Table 11-2:** – Percentage of 9th grade students who smoked in the 30 days prior to the survey, by sex and type of school, in Brazil, its regions and Federative Units.

Major regions and Federative Units	Total			Sex						Type of school					
				Male			Female			Public			Private		
	Total	95% CI		Total	95% CI		Total	95% CI		Total	95% CI		Total	95% CI	
		Lower limit	Upper limit		Lower limit	Upper limit		Lower limit	Upper limit		Lower limit	Upper limit		Lower limit	Upper limit
Brazil	5.6	5.3	5.9	5.8	5.4	6.3	5.4	4.9	5.8	5.9	5.5	6.3	3.6	3.0	4.3
North	6.1	5.4	6.7	7.1	6.2	8.0	5.1	4.4	5.8	6.3	5.6	7.0	3.7	2.0	5.4
Rondonia	5.3	4.2	6.4	5.8	4.1	7.6	4.9	3.7	6.0	5.5	4.3	6.6	3.2	0.7	5.8
Acre	7.1	6.1	8.1	8.2	6.8	9.7	6.0	4.6	7.3	7.4	6.4	8.5	2.4	0.0	4.8
Amazonas	6.6	5.3	7.9	7.7	6.0	9.3	5.5	4.0	7.0	6.8	5.4	8.2	3.6	0.0	7.2
Roraima	10.4	8.4	12.4	12.1	10.0	14.2	8.6	6.3	10.9	10.8	8.7	12.9	3.9	0.0	9.0
Pará	5.5	4.3	6.7	6.5	4.8	8.3	4.6	3.3	5.9	5.7	4.3	7.0	4.2	1.4	7.0
Amapá	7.7	6.6	8.8	8.2	6.9	9.6	7.2	5.8	8.7	8.0	6.9	9.2	4.8	1.1	8.6
Tocantins	4.9	3.5	6.3	5.7	3.8	7.6	4.1	2.8	5.4	5.2	3.7	6.7	1.0	0.4	1.6
Northeast	4.0	3.6	4.3	4.5	4.0	5.0	3.5	3.1	4.0	4.3	3.8	4.7	2.2	1.8	2.7
Maranhão	3.2	2.5	3.8	3.7	2.6	4.8	2.7	1.9	3.5	3.2	2.5	3.9	2.9	1.4	4.3
Piauí	3.2	2.6	3.9	4.0	2.9	5.1	2.5	1.8	3.2	3.4	2.7	4.2	1.6	0.5	2.8
Ceará	5.9	4.8	7.1	5.8	4.4	7.2	6.1	4.6	7.6	6.4	5.0	7.7	3.5	1.9	5.1
Rio Grande do Norte	1.9	1.4	2.3	2.2	1.5	3.0	1.6	1.1	2.1	2.1	1.6	2.7	0.9	0.3	1.5
Paraíba	4.2	3.3	5.0	5.2	4.0	6.4	3.4	2.4	4.3	4.4	3.4	5.4	3.3	1.8	4.8
Pernambuco	4.2	3.3	5.0	4.4	3.3	5.5	3.9	2.7	5.1	4.6	3.5	5.7	2.1	1.2	3.0
Alagoas	3.6	2.6	4.6	3.3	2.3	4.3	3.9	2.7	5.1	3.6	2.5	4.7	3.6	2.2	5.0
Sergipe	1.6	1.1	2.0	1.8	1.2	2.4	1.4	0.8	2.0	1.7	1.2	2.2	1.1	0.4	1.9
Bahia	4.0	3.0	4.9	5.2	3.6	6.8	3.1	2.0	4.2	4.4	3.3	5.5	1.2	0.4	2.1
Southeast	6.0	5.3	6.7	5.8	4.9	6.8	6.2	5.2	7.2	6.4	5.6	7.2	4.1	2.9	5.3
Minas Gerais	4.7	3.7	5.8	4.9	3.5	6.3	4.5	3.4	5.7	4.9	3.8	6.1	2.8	0.8	4.8
Espírito Santo	5.0	3.6	6.3	5.2	3.7	6.7	4.7	3.2	6.3	4.9	3.4	6.4	5.4	2.6	8.2
Rio de Janeiro	5.5	4.5	6.5	5.0	3.9	6.0	6.1	4.6	7.5	6.1	4.8	7.4	4.2	3.1	5.3
São Paulo	6.9	5.7	8.1	6.6	5.0	8.2	7.2	5.4	9.0	7.4	6.0	8.7	4.4	2.2	6.5
South	7.0	6.2	7.7	7.0	5.9	8.1	6.9	5.9	7.9	7.2	6.3	8.1	4.8	3.1	6.5
Paraná	7.8	6.3	9.2	8.0	6.1	9.8	7.6	5.7	9.5	8.3	6.7	10.0	3.9	1.3	6.6
Santa Catarina	5.4	4.3	6.6	5.4	3.8	6.9	5.4	3.9	7.0	5.4	4.1	6.6	5.8	3.0	8.5
Rio Grande do Sul	7.1	5.8	8.3	6.8	4.8	8.9	7.3	5.7	8.8	7.1	5.8	8.4	5.9	3.9	7.9
West-Central	6.5	6.0	7.1	7.2	6.5	8.0	5.8	5.2	6.5	6.9	6.3	7.5	4.5	3.4	5.6
Mato Grosso do Sul	8.2	6.9	9.5	8.6	6.7	10.4	7.8	6.1	9.5	8.7	7.3	10.2	3.0	1.1	4.8
Mato Grosso	7.6	6.2	8.9	7.9	5.9	9.9	7.2	5.7	8.8	7.9	6.4	9.3	4.1	0.2	8.1
Goiás	5.6	4.8	6.3	6.4	5.4	7.4	4.7	3.6	5.8	5.8	4.9	6.6	4.3	2.9	5.8
Distrito Federal	6.1	4.9	7.4	7.3	5.2	9.3	5.1	3.9	6.3	6.4	4.9	7.9	5.3	3.4	7.3

*Source: IBGE, PeNSE 2015. ^410^*

**Table 11-3:** – Percentage of 9th grade students who used other tobacco products in the 30 days prior to the survey, by sex and type of school, in Brazil, its regions and Federative Units.

Major regions and Federative Units	Total			Sex						Type of school					
				Male			Female			Public			Private		
	Total	95% CI		Total	95% CI		Total	95% CI		Total	95% CI		Total	95% CI	
		Lower limit	Upper limit		Lower limit	Upper limit		Lower limit	Upper limit		Lower limit	Upper limit		Lower limit	Upper limit
Brazil	6.1	5.7	6.4	6.5	6.1	7.0	5.6	5.1	6.0	6.2	5.8	6.6	5.2	4.5	5.9
North	3.0	2.6	3.4	3.7	3.1	4.2	2.4	2.0	2.8	3.0	2.6	3.4	3.1	1.8	4.3
Rondônia	5.4	4.1	6.8	4.7	3.3	6.1	6.1	4.4	7.9	5.4	3.9	6.8	6.3	2.7	10.0
Acre	5.0	4.0	5.9	6.3	4.7	7.9	3.6	2.5	4.7	5.1	4.1	6.1	3.4	1.1	5.8
Amazonas	2.8	2.1	3.5	3.4	2.4	4.4	2.3	1.5	3.0	2.9	2.2	3.6	1.5	0.2	2.7
Roraima	5.2	4.0	6.3	6.4	4.9	7.8	4.0	2.6	5.3	5.1	4.0	6.1	6.8	0.0	15.3
Pará	2.0	1.4	2.7	2.9	1.9	3.9	1.3	0.7	1.9	1.9	1.3	2.6	3.1	0.9	5.2
Amapá	2.7	2.1	3.3	3.3	2.5	4.2	2.2	1.4	2.9	2.7	2.1	3.4	2.6	1.3	3.9
Tocantins	4.1	2.8	5.4	4.8	2.9	6.8	3.4	2.3	4.6	4.2	2.8	5.6	3.2	0.3	6.2
Northeast	2.3	2.0	2.6	2.9	2.5	3.3	1.8	1.5	2.1	2.3	2.0	2.7	2.1	1.7	2.5
Maranhão	1.7	1.2	2.1	2.1	1.4	2.8	1.4	0.8	1.9	1.6	1.1	2.1	2.6	1.0	4.3
Piauí	1.9	1.4	2.5	2.4	1.6	3.2	1.6	0.9	2.3	2.0	1.4	2.5	1.6	0.0	3.8
Ceará	3.0	1.9	4.0	3.1	2.1	4.2	2.8	1.6	4.1	3.1	1.9	4.3	2.3	1.1	3.5
Rio Grande do Norte	1.5	1.0	1.9	1.8	1.1	2.6	1.2	0.7	1.6	1.5	1.0	2.1	1.2	0.4	2.0
Paraíba	2.4	1.8	2.9	3.7	2.7	4.6	1.3	0.8	1.8	2.3	1.7	3.0	2.6	1.5	3.7
Pernambuco	2.3	1.7	2.9	2.9	2.1	3.7	1.6	1.0	2.3	2.4	1.7	3.1	1.7	1.0	2.3
Alagoas	2.0	1.4	2.6	2.3	1.6	3.0	1.7	0.9	2.5	1.8	1.2	2.4	2.7	0.9	4.5
Sergipe	1.5	1.1	1.9	2.2	1.5	3.0	0.9	0.5	1.3	1.6	1.1	2.0	1.2	0.6	1.8
Bahia	2.6	1.8	3.4	3.5	2.3	4.8	1.9	1.2	2.6	2.7	1.8	3.5	2.3	1.6	3.0
Southeast	7.5	6.8	8.2	7.8	7.0	8.7	7.2	6.2	8.1	7.8	7.0	8.6	6.0	4.8	7.3
Minas Gerais	4.6	3.8	5.5	5.4	4.2	6.6	3.9	2.7	5.1	4.8	3.9	5.8	2.9	1.6	4.2
Espírito Santo	3.3	2.5	4.1	3.6	2.5	4.6	3.0	2.0	4.0	3.1	2.3	4.0	4.2	2.3	6.0
Rio de Janeiro	4.5	3.7	5.3	4.8	3.9	5.7	4.2	3.1	5.3	4.7	3.7	5.7	3.9	2.6	5.2
São Paulo	10.2	9.0	11.4	10.3	8.9	11.7	10.2	8.6	11.8	10.6	9.3	12.0	8.3	6.0	10.6
South	9.6	8.5	10.6	9.6	8.4	10.8	9.6	8.2	11.0	9.8	8.7	11.0	7.7	5.5	9.9
Paraná	13.8	11.9	15.8	13.8	11.6	16.1	13.8	11.3	16.4	14.5	12.3	16.7	9.3	5.6	13.0
Santa Catarina	8.3	6.2	10.5	8.1	6.0	10.1	8.6	5.7	11.5	8.6	6.2	11.0	6.5	3.2	9.8
Rio Grande do Sul	4.6	3.6	5.6	4.6	3.2	6.0	4.6	3.0	6.1	4.6	3.6	5.6	4.6	2.6	6.6
West-Central	10.0	9.2	10.8	10.4	9.4	11.4	9.6	8.6	10.6	10.0	9.1	10.8	10.2	8.2	12.1
Mato Grosso do Sul	13.9	12.1	15.7	15.1	12.8	17.3	12.8	10.7	14.9	14.1	12.2	16.0	12.1	6.2	18.1
Mato Grosso	9.4	7.9	10.8	8.3	6.3	10.3	10.5	8.6	12.4	9.4	7.9	11.0	8.6	3.7	13.5
Goiás	7.8	6.6	9.0	8.4	7.0	9.8	7.2	5.6	8.7	7.5	6.2	8.9	9.3	7.1	11.5
Distrito Federal	12.2	10.2	14.2	13.6	11.0	16.2	10.9	8.7	13.2	12.5	10.3	14.7	11.4	7.1	15.7

*Source: IBGE, PeNSE 2015. ^410^*

**Table 11-4:** – Proportion of current users of tobacco products aged 18 years and over, by sex, in Brazil, its regions, Federative Units, and urban and rural areas.

Major regions, Federative Units, rural and urban area	Total			Sex					
				Male			Female		
	Proportion	95% CI		Proportion	95% CI		Proportion	95% CI	
		Lower limit	Upper limit		Lower limit	Upper limit		Lower limit	Upper limit
Brazil	12.8	12.4	13.2	16.2	15.6	16.9	9.8	9.3	10.3
Urban	12.6	12.1	13.0	15.8	15.0	16.5	9.9	9.4	10.4
Rural	14.3	13.4	15.2	18.6	17.4	19.9	9.3	8.3	10.2
North	10.7	9.9	11.5	15.5	14.2	16.8	6.2	5.4	7.1
Rondônia	10.8	9.2	12.4	16.6	14.1	19.1	5.2	3.4	7.1
Acre	15.1	13.2	17.1	19.0	15.7	22.2	11.5	9.2	13.9
Amazonas	10.2	9.0	11.4	16.0	13.6	18.4	4.8	3.6	5.9
Roraima	11.6	9.8	13.3	16.5	13.6	19.4	6.8	5.0	8.6
Pará	10.1	8.6	11.5	14.3	12.0	16.6	6.1	4.5	7.8
Amapá	10.9	8.8	12.9	15.0	11.3	18.6	7.0	4.9	9.2
Tocantins	12.8	10.7	14.9	17.6	14.0	21.1	8.1	5.9	10.3
Northeast	11.1	10.5	11.7	14.7	13.7	15.6	7.9	7.3	8.6
Maranhão	11.3	10.2	12.4	16.0	14.1	17.9	7.0	5.9	8.2
Piauí	11.7	10.3	13.2	15.9	13.4	18.4	7.9	5.8	10.0
Ceará	12.2	11.1	13.4	15.4	13.3	17.4	9.5	8.1	10.9
Rio Grande do Norte	11.3	9.6	13.1	15.9	12.9	18.8	7.4	5.8	9.1
Paraíba	11.8	10.1	13.4	14.5	11.8	17.2	9.4	7.8	11.0
Pernambuco	11.3	9.6	13.0	14.7	11.3	18.2	8.5	7.1	9.9
Alagoas	10.6	9.3	12.0	13.9	11.6	16.1	7.9	6.6	9.2
Sergipe	9.4	8.2	10.6	11.6	9.5	13.7	7.5	5.9	9.0
Bahia	10.1	8.4	11.7	13.7	11.3	16.0	6.8	5.1	8.5
Southeast	13.5	12.7	14.3	16.8	15.5	18.1	10.6	9.7	11.5
Minas Gerais	13.2	11.8	14.6	17.1	14.8	19.4	9.7	8.2	11.3
Espírito Santo	10.4	9.1	11.6	14.0	11.6	16.3	7.1	5.7	8.6
Rio de Janeiro	12.1	10.9	13.3	14.6	12.8	16.5	10.0	8.6	11.5
São Paulo	14.4	13.1	15.7	17.7	15.7	19.7	11.5	10.0	12.9
South	14.7	13.8	15.7	17.1	15.7	18.5	12.6	11.4	13.8
Paraná	14.7	13.0	16.4	17.3	14.9	19.7	12.3	10.2	14.5
Santa Catarina	13.1	11.6	14.6	15.7	13.5	18.0	10.6	8.8	12.4
Rio Grande do Sul	15.8	14.3	17.4	17.8	15.3	20.2	14.1	12.2	16.1
West-Central	13.7	12.7	14.7	17.6	15.9	19.2	10.2	9.1	11.3
Mato Grosso do Sul	16.3	14.5	18.2	20.8	17.9	23.7	12.4	10.5	14.3
Mato Grosso	13.0	11.3	14.8	16.3	13.2	19.3	10.0	7.9	12.1
Goiás	13.9	12.1	15.8	18.1	15.0	21.2	10.1	8.1	12.1
Distrito Federal	11.6	9.9	13.4	15.1	12.3	17.9	8.7	6.6	10.8

*Source: IBGE, Diretoria de Pesquisas, Coordenação de Trabalho e Rendimento, Pesquisa Nacional de Saúde 2019. ^306^*

**Table 11-5:** – Proportion of current tobacco users aged 18 years and over, by sex, in Brazil, its regions, Federative Units, and urban and rural areas.

Major regions, Federative Units, rural and urban area	Total			Sex					
				Male			Female		
	Proportion	95% CI		Proportion	95% CI		Proportion	95% CI	
		Lower limit	Upper limit		Lower limit	Upper limit		Lower limit	Upper limit
Brazil	12.6	12.2	13.0	15.9	15.3	16.6	9.6	9.2	10.1
Urban	12.4	12.0	12.9	15.6	14.8	16.3	9.8	9.2	10.3
Rural	13.7	12.8	14.6	17.9	16.6	19.1	8.9	7.9	9.9
North	10.5	9.7	11.3	15.2	13.9	16.5	6.1	5.2	7.0
Rondônia	10.4	8.8	12.0	15.8	13.3	18.3	5.2	3.4	7.0
Acre	13.9	12.0	15.7	17.0	13.7	20.2	10.9	8.7	13.2
Amazonas	10.2	8.9	11.4	15.9	13.5	18.3	4.7	3.6	5.9
Roraima	11.4	9.7	13.2	16.2	13.3	19.1	6.8	5.0	8.6
Pará	9.8	8.4	11.3	14.1	11.8	16.4	5.9	4.3	7.6
Amapá	10.9	8.8	12.9	15.0	11.3	18.6	7.0	4.9	9.2
Tocantins	12.6	10.4	14.7	17.4	13.8	21.0	7.9	5.6	10.1
Northeast	10.8	10.2	11.4	14.2	13.3	15.2	7.7	7.1	8.3
Maranhão	11.0	9.9	12.1	15.7	13.9	17.6	6.8	5.6	8.0
Piauí	11.0	9.5	12.5	15.2	12.7	17.7	7.2	5.3	9.1
Ceará	11.6	10.4	12.7	14.5	12.5	16.4	9.0	7.7	10.4
Rio Grande do Norte	11.0	9.2	12.7	15.1	12.2	18.0	7.4	5.8	9.1
Paraíba	11.7	10.0	13.3	14.3	11.6	17.0	9.4	7.8	11.0
Pernambuco	11.2	9.6	12.9	14.7	11.2	18.1	8.5	7.0	9.9
Alagoas	10.6	9.3	11.9	13.8	11.6	16.1	7.9	6.6	9.2
Sergipe	9.2	8.0	10.5	11.4	9.3	13.5	7.3	5.8	8.9
Bahia	9.8	8.2	11.4	13.3	11.0	15.6	6.6	5.0	8.3
Southeast	13.3	12.5	14.1	16.6	15.3	17.8	10.4	9.5	11.3
Minas Gerais	12.7	11.3	14.1	16.4	14.1	18.7	9.4	7.9	10.9
Espírito Santo	10.2	8.9	11.5	13.9	11.5	16.2	6.9	5.4	8.4
Rio de Janeiro	12.0	10.8	13.2	14.6	12.8	16.5	9.9	8.4	11.4
São Paulo	14.3	13.0	15.6	17.6	15.6	19.6	11.4	10.0	12.8
South	14.7	13.7	15.6	17.0	15.6	18.4	12.5	11.4	13.7
Paraná	14.6	12.9	16.3	17.2	14.9	19.6	12.2	10.1	14.3
Santa Catarina	13.0	11.5	14.5	15.6	13.4	17.9	10.6	8.8	12.4
Rio Grande do Sul	15.8	14.2	17.3	17.8	15.3	20.2	14.0	12.1	16.0
West-Central	13.1	12.1	14.1	16.5	14.9	18.1	10.0	8.9	11.1
Mato Grosso do Sul	14.9	13.2	16.7	18.0	15.4	20.7	12.2	10.3	14.1
Mato Grosso	12.9	11.2	14.6	16.0	13.0	19.0	9.9	7.9	12.0
Goiás	13.4	11.6	15.2	17.3	14.3	20.2	9.9	7.9	11.9
Distrito Federal	11.0	9.3	12.8	14.0	11.2	16.8	8.5	6.4	10.6

*Source: IBGE, Diretoria de Pesquisas, Coordenação de Trabalho e Rendimento, Pesquisa Nacional de Saúde 2019. ^306^*

**Table 11-6:** – Proportion of current tobacco users aged 18 years and over, by age group, in Brazil, its regions, Federative Units, and urban and rural areas.

Major regions, Federative Units, rural and urban area	Total			Age groups											
				18 – 24 years			25 - 39 years			40 - 59 years			≥ 60 years		
	Proportion	95% CI		Proportion	95% CI		Proportion	95% CI		Proportion	95% CI		Proportion	95% CI	
		Lower limit	Upper limit		Lower limit	Upper limit		Lower limit	Upper limit		Lower limit	Upper limit		Lower limit	Upper limit
Brazil	12.8	12.4	13.2	10.8	9.6	12.0	12.0	11.2	12.7	14.9	14.2	15.6	11.9	11.2	12.6
Urban	12.6	12.1	13.0	11.1	9.7	12.4	12.0	11.2	12.9	14.5	13.7	15.3	11.1	10.4	11.9
Rural	14.3	13.4	15.2	9.0	6.9	11.0	11.6	10.1	13.1	17.5	15.9	19.0	16.2	14.6	17.9
North	10.7	9.9	11.5	8.0	6.3	9.6	11.1	9.7	12.5	12.2	10.8	13.7	9.9	8.4	11.3
Rondônia	10.8	9.2	12.4	12.6	7.9	17.3	10.5	7.8	13.1	11.7	9.0	14.3	7.9	5.1	10.7
Acre	15.1	13.2	17.1	14.1	9.9	18.3	11.4	8.5	14.3	18.3	15.0	21.7	17.3	13.3	21.3
Amazonas	10.2	9.0	11.4	7.4	4.6	10.1	12.0	9.4	14.5	11.1	8.8	13.4	8.0	5.6	10.3
Roraima	11.6	9.8	13.3	9.1	5.1	13.0	12.8	10.0	15.7	13.2	10.0	16.4	7.9	5.0	10.9
Pará	10.1	8.6	11.5	6.5	3.6	9.5	10.8	8.3	13.4	11.4	8.8	14.0	9.6	7.0	12.2
Amapá	10.9	8.8	12.9	9.2	5.9	12.5	9.2	5.0	13.3	14.1	10.1	18.1	10.1	6.2	14.0
Tocantins	12.8	10.7	14.9	8.0	3.9	12.1	11.7	8.3	15.1	15.1	11.0	19.2	14.1	10.1	18.1
Northeast	11.1	10.5	11.7	7.5	6.2	8.9	9.0	8.1	9.8	13.4	12.2	14.6	12.8	11.7	13.9
Maranhão	11.3	10.2	12.4	7.9	5.3	10.6	10.2	8.4	12.0	13.3	11.3	15.4	12.6	10.2	15.1
Piauí	11.7	10.3	13.2	7.7	3.3	12.1	10.4	7.6	13.1	12.6	9.7	15.4	14.9	11.3	18.4
Ceará	12.2	11.1	13.4	5.3	2.8	7.9	10.5	8.3	12.6	14.1	12.0	16.2	16.5	13.9	19.2
Rio Grande do Norte	11.3	9.6	13.1	10.2	4.7	15.7	8.0	5.4	10.6	14.3	11.5	17.1	12.3	9.4	15.2
Paraíba	11.8	10.1	13.4	6.9	3.8	10.0	8.8	6.4	11.1	15.7	12.6	18.8	13.0	9.7	16.3
Pernambuco	11.3	9.6	13.0	9.5	6.3	12.7	10.4	7.9	12.8	13.0	9.9	16.2	10.9	8.6	13.2
Alagoas	10.6	9.3	12.0	8.1	4.5	11.7	8.3	6.1	10.4	13.2	10.5	15.8	11.7	8.9	14.6
Sergipe	9.4	8.2	10.6	5.2	1.7	8.7	8.2	6.0	10.4	12.0	9.4	14.6	10.0	7.1	13.0
Bahia	10.1	8.4	11.7	7.2	3.3	11.0	6.8	4.7	8.9	12.9	9.6	16.2	11.8	8.8	14.9
Southeast	13.5	12.7	14.3	12.8	10.2	15.3	12.8	11.3	14.2	15.6	14.3	16.8	11.5	10.2	12.8
Minas Gerais	13.2	11.8	14.6	11.7	7.1	16.3	11.7	9.6	13.8	15.2	12.8	17.6	12.7	10.3	15.0
Espírito Santo	10.4	9.1	11.6	8.2	3.7	12.7	10.0	7.7	12.4	12.2	10.2	14.1	9.2	6.8	11.6
Rio de Janeiro	12.1	10.9	13.3	10.2	6.7	13.6	12.8	10.4	15.2	13.2	11.4	15.0	10.8	8.8	12.8
São Paulo	14.4	13.1	15.7	14.6	10.4	18.7	13.5	11.0	15.9	16.9	14.8	18.9	11.4	9.3	13.5
South	14.7	13.8	15.7	12.6	9.7	15.5	15.2	13.3	17.2	16.5	15.0	18.1	12.4	11.1	13.8
Paraná	14.7	13.0	16.4	14.3	9.4	19.1	16.2	12.6	19.8	15.4	12.4	18.3	11.7	9.3	14.2
Santa Catarina	13.1	11.6	14.6	9.7	5.1	14.3	13.0	10.3	15.7	15.2	12.7	17.7	11.7	9.1	14.2
Rio Grande do Sul	15.8	14.3	17.4	12.5	7.3	17.6	15.7	12.4	19.1	18.6	16.1	21.1	13.5	11.3	15.6
West-Central	13.7	12.7	14.7	12.7	10.0	15.3	13.3	11.6	15.0	15.4	13.6	17.3	11.7	9.9	13.5
Mato Grosso do Sul	16.3	14.5	18.2	17.3	12.3	22.3	16.6	13.3	19.8	17.8	15.0	20.6	12.8	9.6	16.0
Mato Grosso	13.0	11.3	14.8	13.5	7.6	19.5	14.0	10.6	17.4	14.3	10.9	17.7	7.9	5.2	10.5
Goiás	13.9	12.1	15.8	11.2	6.6	15.8	12.3	9.4	15.2	16.6	13.0	20.2	13.3	10.1	16.6
Distrito Federal	11.6	9.9	13.4	11.3	6.9	15.8	11.9	8.7	15.0	12.1	9.2	15.0	10.5	6.9	14.1

*Source: IBGE, Diretoria de Pesquisas, Coordenação de Trabalho e Rendimento, Pesquisa Nacional de Saúde 2019. ^306^*

**Table 11-7:** – Proportion of current tobacco users aged 18 years and over, by education level, in Brazil, its regions, Federative Units, and urban and rural areas.

Major regions, Federative Units, rural and urban area	Total			Education level											
				Without instruction and incomplete primary			Complete primary and incomplete secondary			Complete secondary and incomplete college			Complete college		
	Proportion	95% CI		Proportion	95% CI		Proportion	95% CI		Proportion	95% CI		Proportion	95% CI	
		Lower limit	Upper limit		Lower limit	Upper limit		Lower limit	Upper limit		Lower limit	Upper limit		Lower limit	Upper limit
Brazil	12.8	12.4	13.2	17.6	16.8	18.4	15.5	14.3	16.6	9.6	8.9	10.2	7.1	6.3	7.8
Urban	12.6	12.1	13.0	17.4	16.5	18.3	16.2	14.8	17.5	9.9	9.2	10.5	7.1	6.4	7.9
Rural	14.3	13.4	15.2	18.2	17.0	19.5	11.2	9.3	13.1	5.5	4.5	6.6	4.4	2.4	6.3
North	10.7	9.9	11.5	16.7	15.1	18.4	11.0	9.1	12.9	6.4	5.4	7.4	4.0	2.7	5.2
Rondônia	10.8	9.2	12.4	15.3	12.6	18.1	14.0	9.4	18.6	5.6	3.3	7.9	4.7	1.5	7.8
Acre	15.1	13.2	17.1	25.5	21.9	29.1	14.3	9.8	18.7	8.6	5.9	11.4	4.4	1.7	7.0
Amazonas	10.2	9.0	11.4	16.0	13.5	18.6	11.7	8.2	15.1	6.7	4.7	8.6	4.7	2.1	7.3
Roraima	11.6	9.8	13.3	18.9	15.0	22.8	16.5	10.5	22.4	8.2	6.2	10.2	5.2	2.7	7.6
Pará	10.1	8.6	11.5	15.8	12.9	18.7	9.6	6.5	12.6	5.4	3.7	7.2	3.0	0.6	5.4
Amapá	10.9	8.8	12.9	15.1	11.2	19.0	10.0	5.4	14.7	10.1	6.9	13.3	5.3	1.4	9.2
Tocantins	12.8	10.7	14.9	20.4	16.6	24.3	12.4	6.5	18.3	7.3	4.6	10.0	4.0	1.5	6.4
Northeast	11.1	10.5	11.7	16.8	15.7	17.9	10.3	9.0	11.7	5.6	4.8	6.3	3.6	2.8	4.5
Maranhão	11.3	10.2	12.4	18.1	16.2	20.0	10.3	7.7	12.9	3.8	2.5	5.1	3.6	1.8	5.3
Piauí	11.7	10.3	13.2	17.8	15.2	20.5	8.9	4.1	13.7	5.0	3.3	6.6	6.8	2.9	10.6
Ceará	12.2	11.1	13.4	18.5	16.5	20.6	10.3	7.4	13.3	7.6	5.7	9.4	3.1	0.9	5.4
Rio Grande do Norte	11.3	9.6	13.1	19.5	16.1	22.9	8.2	4.9	11.5	5.2	3.4	6.9	2.6	0.8	4.4
Paraíba	11.8	10.1	13.4	17.4	14.7	20.2	9.3	5.9	12.8	5.9	3.8	8.0	4.7	2.5	6.9
Pernambuco	11.3	9.6	13.0	16.1	13.2	19.0	13.7	9.6	17.7	7.1	5.0	9.2	4.5	2.4	6.6
Alagoas	10.6	9.3	12.0	15.8	13.7	17.9	12.7	8.3	17.0	4.8	2.7	6.8	1.7	0.5	3.0
Sergipe	9.4	8.2	10.6	14.9	12.6	17.3	7.7	4.5	10.9	4.5	2.8	6.2	1.7	0.5	3.0
Bahia	10.1	8.4	11.7	15.2	12.3	18.1	9.5	5.9	13.1	4.3	2.6	6.1	3.3	1.1	5.4
Southeast	13.5	12.7	14.3	17.4	15.8	19.1	18.0	15.7	20.3	11.2	10.0	12.3	8.6	7.4	9.8
Minas Gerais	13.2	11.8	14.6	17.3	14.9	19.6	16.2	12.4	19.9	10.1	7.9	12.2	6.4	4.4	8.4
Espírito Santo	10.4	9.1	11.6	13.3	10.9	15.7	13.4	9.3	17.5	8.9	6.8	10.9	4.4	2.7	6.2
Rio de Janeiro	12.1	10.9	13.3	14.6	12.2	17.1	16.0	12.3	19.8	10.9	9.2	12.6	8.2	6.3	10.1
São Paulo	14.4	13.1	15.7	19.0	16.0	22.0	20.2	16.2	24.2	11.9	10.1	13.7	9.6	7.7	11.5
South	14.7	13.8	15.7	19.0	17.3	20.6	19.3	16.6	22.1	12.1	10.6	13.5	7.6	6.1	9.1
Paraná	14.7	13.0	16.4	20.3	17.3	23.3	21.2	16.3	26.1	11.1	8.3	13.9	6.1	3.9	8.2
Santa Catarina	13.1	11.6	14.6	17.6	14.8	20.5	16.2	12.6	19.9	10.6	8.7	12.6	6.4	4.2	8.7
Rio Grande do Sul	15.8	14.3	17.4	18.5	16.0	21.0	19.9	15.0	24.8	14.0	11.4	16.5	10.0	7.0	13.0
West-Central	13.7	12.7	14.7	20.9	18.9	23.0	14.8	12.5	17.1	10.5	9.1	11.9	6.0	4.3	7.7
Mato Grosso do Sul	16.3	14.5	18.2	21.1	17.9	24.4	17.4	12.6	22.1	15.9	12.4	19.3	5.8	3.7	7.8
Mato Grosso	13.0	11.3	14.8	17.5	13.5	21.5	20.0	14.6	25.4	9.5	6.6	12.4	3.3	1.5	5.1
Goiás	13.9	12.1	15.8	22.4	18.8	26.0	11.1	7.7	14.4	9.2	6.9	11.4	7.3	2.8	11.8
Distrito Federal	11.6	9.9	13.4	21.1	16.7	25.5	15.8	10.7	20.8	10.5	7.6	13.4	6.3	4.0	8.7

*Source: IBGE, Diretoria de Pesquisas, Coordenação de Trabalho e Rendimento, Pesquisa Nacional de Saúde 2019. ^306^*

**Table 11-8:** – Proportion of current tobacco users aged 18 years and over, by skin color or race, in Brazil, its regions, Federative Units, and urban and rural areas.

Major regions, Federative Units, rural and urban area	Total			Color or race								
				White			Black			“Pardos” or mixed race involving African ancestry		
	Proportion	95% CI		Proportion	95% CI		Proportion	95% CI		Proportion	95% CI	
		Lower limit	Upper limit		Lower limit	Upper limit		Lower limit	Upper limit		Lower limit	Upper limit
Brazil	12.8	12.4	13.2	11.8	11.2	12.4	13.7	12.5	15.0	13.5	12.9	14.2
Urban	12.6	12.1	13.0	11.9	11.2	12.5	13.4	12.1	14.7	13.1	12.4	13.8
Rural	14.3	13.4	15.2	11.4	10.3	12.6	15.9	13.2	18.6	15.6	14.3	16.9
North	10.7	9.9	11.5	8.5	7.0	9.9	11.0	8.9	13.0	11.2	10.2	12.3
Rondônia	10.8	9.2	12.4	9.3	6.3	12.3	12.2	7.4	16.9	11.1	8.9	13.3
Acre	15.1	13.2	17.1	9.6	5.8	13.4	24.1	16.6	31.5	15.1	12.8	17.4
Amazonas	10.2	9.0	11.4	8.2	5.1	11.3	8.4	4.0	12.9	10.7	9.2	12.1
Roraima	11.6	9.8	13.3	7.6	4.6	10.6	16.9	9.7	24.1	12.0	10.0	13.9
Pará	10.1	8.6	11.5	7.7	5.2	10.3	7.8	4.5	11.0	11.0	9.1	12.8
Amapá	10.9	8.8	12.9	9.4	5.0	13.9	16.7	10.2	23.3	9.8	7.2	12.3
Tocantins	12.8	10.7	14.9	10.4	7.0	13.9	14.6	9.8	19.5	13.0	10.2	15.8
Northeast	11.1	10.5	11.7	8.8	7.9	9.7	11.9	10.3	13.5	11.9	11.1	12.7
Maranhão	11.3	10.2	12.4	10.0	7.5	12.4	10.4	7.8	13.0	11.9	10.5	13.3
Piauí	11.7	10.3	13.2	8.4	5.5	11.3	9.2	6.0	12.4	13.3	11.4	15.2
Ceará	12.2	11.1	13.4	11.7	9.2	14.1	15.1	10.7	19.4	12.2	10.8	13.6
Rio Grande do Norte	11.3	9.6	13.1	7.5	5.4	9.7	22.1	15.3	28.9	12.4	9.9	14.8
Paraíba	11.8	10.1	13.4	8.5	6.3	10.7	20.8	14.8	26.8	12.2	10.3	14.1
Pernambuco	11.3	9.6	13.0	9.2	7.1	11.3	17.1	11.1	23.1	11.6	9.5	13.8
Alagoas	10.6	9.3	12.0	7.7	5.2	10.2	14.4	9.4	19.4	11.3	9.6	13.0
Sergipe	9.4	8.2	10.6	8.0	5.3	10.7	12.9	8.3	17.5	9.2	7.5	10.9
Bahia	10.1	8.4	11.7	6.5	4.0	8.9	9.0	6.2	11.7	11.9	9.3	14.5
Southeast	13.5	12.7	14.3	12.3	11.3	13.4	14.3	12.1	16.5	14.9	13.5	16.2
Minas Gerais	13.2	11.8	14.6	11.0	9.0	13.0	13.0	9.8	16.2	15.4	13.3	17.6
Espírito Santo	10.4	9.1	11.6	8.5	6.5	10.6	13.6	9.7	17.5	10.9	8.8	13.0
Rio de Janeiro	12.1	10.9	13.3	11.6	10.0	13.1	12.1	9.2	15.0	12.3	10.3	14.2
São Paulo	14.4	13.1	15.7	13.2	11.7	14.8	17.0	12.2	21.7	16.2	13.7	18.6
South	14.7	13.8	15.7	13.2	12.2	14.2	17.3	13.4	21.3	18.9	16.3	21.5
Paraná	14.7	13.0	16.4	12.4	10.6	14.2	18.1	11.6	24.7	18.2	14.4	22.0
Santa Catarina	13.1	11.6	14.6	11.7	10.1	13.3	15.5	7.7	23.3	19.1	14.3	23.9
Rio Grande do Sul	15.8	14.3	17.4	14.7	13.1	16.3	17.3	11.4	23.2	20.2	15.4	25.0
West-Central	13.7	12.7	14.7	11.7	10.1	13.2	18.0	14.9	21.2	14.2	12.8	15.6
Mato Grosso do Sul	16.3	14.5	18.2	15.2	12.3	18.1	19.5	12.9	26.0	17.4	14.9	19.9
Mato Grosso	13.0	11.3	14.8	11.2	8.0	14.3	18.2	13.0	23.4	13.0	10.8	15.2
Goiás	13.9	12.1	15.8	10.2	7.4	13.0	18.1	12.2	23.9	15.3	12.6	17.9
Distrito Federal	11.6	9.9	13.4	11.6	8.5	14.7	17.2	11.7	22.6	10.2	8.3	12.2

*Source: IBGE, Diretoria de Pesquisas, Coordenação de Trabalho e Rendimento, Pesquisa Nacional de Saúde 2019. ^306^*

**Table 11-9:** – Prevalence of smokers over 18 years of age in the main Brazilian capitals, by sex, according to the Vigitel household survey.

Indicator	Sex	2006	2007	2008	2009	2010	2011	2012	2013	2014	2015	2016	2017	2018	2019
Smokers	Male	19.3	19.6	18	17.5	16.8	16.5	15.5	14.4	12.8	12.8	12.7	13.2	12.1	12.3
	Female	12.4	12.3	12	11.5	11.7	10.7	9.2	8.6	9	8.3	8	7.5	6.9	7.7
	Total	15.6	15.7	14.8	14.3	14.1	13.4	12.1	11.3	10.8	10.4	10.2	10.1	9.3	9.8

*Source: Vigitel – 2006 to 2019. ^276^*

**Table 11-10:** – Age-standardized rate (per 100 000) of deaths attributed to tobacco, and percent change of rates, by sex, in Brazil and Federative Units, 1990 and 2019.

Location of death	Female			Male		
	1990 (95% UI)	2019 (95% UI)	Percent change (95% UI)	1990 (95% UI)	2019 (95% UI)	Percent change (95% UI)
Acre	143.9 (122.5;164.7)	79.9 (68.9;90.7)	-44.5 (-52.9; -34.2)	240.9 (221.2;262.2)	123.8 (109.7;138.8)	-48.6 (-55.2; -41.7)
Alagoas	125.3 (105.4;145.5)	58.8 (49.7;69.2)	-53 (-62; -42.4)	216.2 (194.6;237.4)	103.7 (88.1;122.3)	-52 (-60; -42.6)
Amapá	86.3 (73;101.2)	48.5 (41.9;55.6)	-43.8 (-53.6; -31.9)	154.4 (139.7;169.1)	83.4 (74.4;93.4)	-46 (-52.5; -38.4)
Amazonas	101.3 (83.2;121.7)	44.4 (38.1;52.1)	-56.1 (-64.7; -44.8)	183.1 (162.3;204.2)	87.3 (75.6;100.5)	-52.3 (-59.7; -43.4)
Bahia	91.7 (75.5;109.9)	39.9 (32.3;48.9)	-56.5 (-66.7; -43.2)	177.1 (151.9;205.9)	101.1 (82;123.8)	-42.9 (-55.6; -26.8)
Brazil	139.7 (126.3;153.1)	57.1 (52.8;61.5)	-59.1 (-63.1; -54.9)	271.6 (258.6;285)	115.1 (108.1;123.3)	-57.6 (-60.2; -54.8)
Ceará	99.4 (80.3;120.4)	60.8 (49.3;74.3)	-38.8 (-53.3; -18.8)	164.7 (136.9;195.2)	103.3 (82.5;127.2)	-37.3 (-52.5; -16.8)
Distrito Federal	149.4 (122.9;178.2)	52.7 (45.2;62)	-64.7 (-71.1; -56.2)	303.7 (270;339.3)	97.3 (84.2;112)	-68 (-72.7; -61.9)
Espírito Santo	118.7 (101.7;137.1)	48.6 (41.3;56.8)	-59 (-66.3; -49.6)	239.1 (223.2;254.8)	111.2 (94.7;128.1)	-53.5 (-60.6; -46.1)
Goiás	155.7 (126.5;190.4)	60.4 (49.8;72.9)	-61.2 (-69.9;-49.5)	284.3 (242.7;333.5)	115.3 (94.2;139.5)	-59.4 (-68.1;-49.2)
Maranhão	69.8 (55.7;86.8)	46.1 (37.4;57.1)	-34 (-49.7;-11.3)	226 (187.6;269.3)	101.9 (80.7;124.6)	-54.9 (-65.7;-41.5)
Mato Grosso	116 (96.6;137.2)	50.3 (43.3;58.7)	-56.6 (-65;-46.1)	211.5 (182.9;242.8)	94.2 (81.3;108.2)	-55.5 (-63.1;-45.6)
Mato Grosso do Sul	142.4 (122.5;164.1)	55.2 (47.4;63.9)	-61.2 (-67.7;-53.6)	251.9 (230.7;273.9)	108.8 (94.9;125.6)	-56.8 (-63.1;-49.4)
Minas Gerais	139.8 (120.7;159.9)	50 (43.2;57.6)	-64.3 (-70.3;-57)	275 (252.2;298.7)	102.9 (90;117.3)	-62.6 (-67.7;-56.9)
Pará	103.7 (84.8;125.5)	43.1 (36.5;50.9)	-58.5 (-66.9;-48.3)	200.2 (170.7;233.3)	89.2 (76;102.8)	-55.4 (-63.8;-45.8)
Paraíba	102.9 (86.3;120.6)	50.9 (42.4;60.5)	-50.5 (-60.4;-37.7)	162.6 (145.6;180.1)	95.5 (79.7;113.3)	-41.2 (-52.5;-29.3)
Paraná	172.6 (149.9;195.8)	67.2 (57.6;77.6)	-61.1 (-67.7;-53.1)	298.6 (281.7;315.6)	127.2 (109.9;146.8)	-57.4 (-63.5;-50.7)
Pernambuco	140 (122;161)	71.1 (60.5;82.2)	-49.2 (-57.6;-38.9)	221 (205.5;235.5)	133.7 (114.3;154)	-39.5 (-48.9;-29.4)
Piauí	88.5 (74.5;105.1)	41.1 (34.8;48.2)	-53.5 (-62;-42.2)	186.3 (166.6;208)	77.1 (65.4;89.2)	-58.6 (-65.3;-51)
Rio de Janeiro	170 (149.8;190.3)	62 (53.9;71.8)	-63.5 (-69.1;-56.6)	352.4 (334;371.2)	129.1 (112.8;147.9)	-63.4 (-67.9;-57.8)
Rio Grande do Norte	97.7 (80;116.8)	49.5 (40.1;60.3)	-49.4 (-60.8;-35)	160.4 (139;183.6)	86.6 (69.6;106.1)	-46 (-58;-31.3)
Rio Grande do Sul	175.5 (155.4;197)	75.4 (65.5;85.6)	-57 (-63.4;-49.5)	366.9 (346.6;386.8)	144.2 (125.6;165.1)	-60.7 (-65.4;-54.7)
Rondônia	181.8 (154.2;213.4)	63.6 (54.3;74.6)	-65 (-71.6;-57.1)	257.6 (230.2;287)	102.7 (85.9;122.8)	-60.1 (-67.6;-51.2)
Roraima	130.3 (111.6;151)	57.2 (50.2;65.2)	-56.1 (-62.7;-47.9)	252.8 (228.8;278.4)	96 (84.4;107.7)	-62 (-67;-56.1)
Santa Catarina	146.8 (127.3;167.9)	53.5 (46.2;61.2)	-63.5 (-69.9;-55.9)	320.4 (296.4;344.5)	124.3 (109.2;141.8)	-61.2 (-66.4;-55)
São Paulo	164.6 (144.2;185.2)	61 (53;69.1)	-62.9 (-68.5;-56.5)	341.7 (318.2;366.2)	127.3 (111.7;145)	-62.7 (-67.6;-57.3)
Sergipe	89.8 (74;108.2)	40.2 (33;48.8)	-55.2 (-65.2;-41.2)	183.1 (160.2;205.8)	85.2 (69.6;102.8)	-53.5 (-62.5;-42.2)
Tocantins	95.9 (79.5;115.5)	46.7 (38.9;55.9)	-51.3 (-61.4;-38.1)	193.5 (165.8;223.1)	99.1 (83.2;117.7)	-48.8 (-58.8;-36)

*Source: Data derived from Global Burden of Disease Study 2019, Institute for Health Metrics and Evaluation, University of Washington. ^46^*

**Table 11-11:** – Number and age-standardized rates (per 100 000) of deaths attributed to tobacco, and percent change of rates, in Brazil and Federative Units, 1990 and 2019.

Location of death	1990		2019		Percent change (95% UI)
	Number (95% UI)	Rate (95% UI)	Number (95% UI)	Rate (CI 95%)	
Acre	268.1 (244.8;290.2)	188.3 (171.9;204.5)	555.2 (499.8;608.6)	99.8 (89.7;109.8)	-47 (-52.6; -40.8)
Alagoas	2251 (2031.3;2493.1)	168.1 (152.9;183.3)	2450.4 (2147.3;2781.5)	78.6 (68.9;89.1)	-53.2 (-59.7; -46.6)
Amapá	105 (95.2;114.6)	118.1 (107.2;129.5)	314.7 (286.5;346.4)	65 (59;71.6)	-45 (-50.8; -38.8)
Amazonas	980.5 (881.8;1086.3)	141.1 (126.2;156.8)	1754.9 (1565;1970)	64.8 (57.7;72.8)	-54 (-60; -47.2)
Bahia	8778 (7710.9;9868.5)	131.7 (115.9;147.5)	10687.3 (9048.9;12468.7)	66.6 (56.3;77.7)	-49.5 (-58.5; -39.1)
Brazil	168443.1 (159638.4;176773.3)	199.9 (189.1;210.6)	191.127.5 (180887.1;202595.5)	82.4 (77.9;87.5)	-58.8 (-61.1; -56.3)
Ceará	5309.1 (4636.5;6033.6)	130.1 (114;148.4)	7831.3 (6685.7;9108.7)	79.6 (68;92.7)	-38.8 (-50.1; -25.6)
Distrito Federal	955.1 (851.9;1072.6)	203.1 (178.6;230)	1524.6 (1362.5;1715)	70.6 (63.2;79.2)	-65.2 (-69.8; -60)
Espírito Santo	2358.2 (2208.3;2512.6)	176.2 (163.1;189.5)	3245.8 (2865.4;3627.9)	76.2 (67.2;85.1)	-56.8 (-62.1; -50.9)
Goiás	4064.4 (3535.8;4726.8)	215.6 (188.9;248.5)	5719 (4862.5;6648.2)	85.9 (73.2;99.7)	-60.2 (-67; -51.9)
Maranhão	3580.9 (2990.5;4188.3)	132.9 (112.6;154.1)	4554 (3835.5;5406)	70.2 (59.2;83.1)	-47.2 (-57.1; -34.3)
Mato Grosso	1196.6 (1045.2;1341.9)	167.7 (148;187)	2265.3 (2030.9;2507.8)	72.4 (64.8;80.2)	-56.8 (-62.7; -49.4)
Mato Grosso do Sul	1602.9 (1488.1;1726.3)	198.8 (183.3;215)	2279.7 (2040;2569)	80.3 (72;90.3)	-59.6 (-64.4; -54.3)
Minas Gerais	18482.8 (17049.2;20105.5)	200.8 (185.2;218.2)	19486.7 (17571.9;21630.8)	73.7 (66.5;81.9)	-63.3 (-67.2; -58.7)
Pará	2922 (2551.9;3292)	149.7 (131.3;168.8)	4359.3 (3833.9;4910.9)	65.1 (57.5;73.3)	-56.5 (-62.7; -49.3)
Paraíba	2969.5 (2689.7;3264.7)	131.1 (118.9;144)	3391.9 (2965.5;3853)	70.3 (61.4;80.1)	-46.4 (-53.8; -38.3)
Paraná	10355.6 (9761.2;10941.2)	234.4 (218.8;249.7)	12142 (10811.1;13558.7)	94 (83.7;104.9)	-59.9 (-64.5; -55.2)
Pernambuco	7749.4 (7180.2;8368.7)	176.8 (164;190.9)	9553.9 (8501;10648.7)	97.7 (87;108.7)	-44.8 (-51.4; -37.8)
Piaui	1774 (1606;1950.4)	133.2 (120.4;146.4)	2162.9 (1901.9;2425.6)	57.4 (50.5;64.3)	-56.9 (-62.7; -51.2)
Rio de Janeiro	22495.9 (21190.9;23737.9)	246.4 (230.1;262.6)	20055.2 (18102.7;22233)	89.7 (80.9;99.3)	-63.6 (-67.3; -59.3)
Rio Grande do Norte	2027.5 (1804.3;2269.4)	127.2 (113.2;141.9)	2549.6 (2176.1;2966.6)	65.7 (56.1;76.6)	-48.3 (-57.1; -38.1)
Rio Grande do Sul	15779.3 (14859.6;16593.5)	257.8 (241.2;273.4)	16325.6 (14743.7;18073.6)	104.6 (94.4;115.9)	-59.4 (-63.6; -54.7)
Rondônia	613.6 (537.2;687.9)	226.1 (205.9;249)	1200.6 (1047.7;1377.8)	83.3 (73.1;95.5)	-63.2 (-68.7; -57.2)
Roraima	94.8 (84.7;103.6)	196.2 (180.3;214.2)	254.2 (230.2;279.4)	76.7 (69.3;84.8)	-60.9 (-65.1; -56.1)
Santa Catarina	5228.8 (4848.3;5603.6)	227 (209.9;244.5)	6668.9 (6011;7389.5)	84.5 (76.1;93.6)	-62.8 (-66.9; -58)
São Paulo	45041.6 (42124.3;47925.1)	243.1 (226.1;260.4)	47506.6 (42991.3;52327.4)	89.1 (80.7;98.4)	-63.3 (-67; -59.3)
Sergipe	958.8 (859.2;1064.6)	130.8 (116.6;146.4)	1314.9 (1124.3;1520.3)	59.7 (51.2;68.7)	-54.4 (-61.8; -46.2)
Tocantins	499.9 (438;568.3)	142.2 (124.8;159.8)	973.1 (847.1;1113.9)	70.7 (61.5;81)	-50.3 (-57.8; -40.9)

*Source: Data derived from Global Burden of Disease Study 2019, Institute for Health Metrics and Evaluation, University of Washington. ^46^*

**Table 11-12:** – Proportion of non-smokers aged 18 years and over exposed to secondhand smoke in the closed workplace, by sex, in Brazil, its regions, Federative Units, and urban and rural areas.

Major regions, Federative Units, rural and urban area	Total			Sex					
				Male			Female		
	Proportion	95% CI		Proportion	95% CI		Proportion	95% CI	
		Lower limit	Upper limit		Lower limit	Upper limit		Lower limit	Upper limit
Brazil	8.4	7.8	9.0	10.4	9.4	11.3	6.7	6.1	7.4
Urban	8.3	7.7	8.9	10.3	9.3	11.3	6.6	5.9	7.3
Rural	10.2	8.6	11.7	11.6	9.4	13.8	9.0	6.8	11.2
North	7.8	6.8	8.8	10.1	8.5	11.7	5.6	4.4	6.8
Rondônia	9.8	6.9	12.8	12.9	7.5	18.3	7.4	3.8	11.0
Acre	5.9	3.9	8.0	6.9	3.2	10.7	5.1	2.7	7.5
Amazonas	7.9	5.7	10.0	10.5	7.1	13.9	5.0	2.8	7.1
Roraima	7.9	5.5	10.3	9.0	5.3	12.7	6.9	4.1	9.7
Pará	6.7	5.0	8.4	8.7	6.1	11.3	4.8	2.8	6.9
Amapá	7.6	4.8	10.3	9.6	5.4	13.8	5.8	2.0	9.5
Tocantins	10.8	7.9	13.6	15.0	10.0	20.0	7.7	4.5	10.9
Northeast	9.2	8.4	10.0	11.0	9.8	12.3	7.7	6.7	8.8
Maranhão	10.4	8.5	12.3	12.4	9.2	15.5	8.8	6.3	11.3
Piauí	13.2	9.9	16.6	15.4	10.8	20.0	11.4	6.6	16.2
Ceará	8.5	6.6	10.4	9.0	5.9	12.1	8.1	5.7	10.5
Rio Grande do Norte	10.6	7.4	13.9	13.5	8.7	18.3	8.2	4.8	11.7
Paraíba	10.1	7.9	12.3	11.2	8.2	14.2	9.2	6.1	12.2
Pernambuco	8.8	6.8	10.9	9.9	7.2	12.6	7.8	5.2	10.5
Alagoas	8.7	6.4	11.1	10.5	6.7	14.3	7.2	4.5	10.0
Sergipe	8.3	6.3	10.3	9.3	6.4	12.2	7.4	4.4	10.3
Bahia	8.2	6.3	10.1	11.1	7.9	14.3	6.0	3.6	8.3
Southeast	8.8	7.7	9.8	10.4	8.6	12.2	7.3	6.1	8.6
Minas Gerais	11.5	9.0	13.9	15.8	10.8	20.8	8.2	5.9	10.5
Espírito Santo	9.1	7.2	11.0	12.6	9.3	15.8	6.2	3.9	8.6
Rio de Janeiro	6.8	5.3	8.2	7.8	5.7	10.0	5.8	3.8	7.8
São Paulo	8.3	6.7	9.9	9.1	6.7	11.6	7.6	5.7	9.5
South	6.3	5.2	7.4	9.0	7.1	11.0	3.8	2.8	4.9
Paraná	5.8	3.9	7.6	8.5	5.2	11.8	3.2	1.7	4.7
Santa Catarina	5.4	4.1	6.7	7.0	4.9	9.2	3.8	2.3	5.3
Rio Grande do Sul	7.4	5.5	9.3	10.9	7.1	14.7	4.5	2.5	6.5
West-Central	9.0	7.6	10.4	11.3	8.8	13.8	6.9	5.4	8.4
Mato Grosso do Sul	7.2	5.3	9.0	9.8	6.6	12.9	4.8	3.0	6.7
Mato Grosso	9.2	6.7	11.7	10.3	6.8	13.8	8.2	4.8	11.6
Goiás	11.5	8.5	14.5	14.8	9.3	20.3	8.4	5.4	11.4
Distrito Federal	5.8	4.0	7.6	6.8	4.2	9.3	4.8	2.7	6.9

*Source: IBGE, Diretoria de Pesquisas, Coordenação de Trabalho e Rendimento, Pesquisa Nacional de Saúde 2019. ^306^*

**Table 11-13:** – Distribution of persons aged 15 and over, using electronic devices with liquid nicotine or chopped tobacco leaf, according to major regions and federation units.

Major regions and federation units	Prevalence of Active users	95% CI	
		Lower limit	Upper limit
**Brazil**	**0.6**	**0.5**	**0.8**
**North**	**0.2**	**0.1**	**0.2**
Rondônia	0.7	0.1	1.4
Acre	0.1	0.0	0.2
Amazonas	0.0	0.0	0.1
Roraima	0.0	0.0	0.1
Pará	0.1	0.0	0.2
Amapá	0.1	0.0	0.3
Tocantins	0.4	0.0	0.7
**Northeast**	**0.1**	**0.1**	**0.2**
Maranhão	0.1	0.0	0.2
Piauí	0.5	0.1	0.8
Ceará	0.3	0.0	0.5
Rio Grande do Norte	0.0	0.0	0.1
Paraíba	0.0	0.0	0.1
Pernambuco	0.0	0.0	0.1
Alagoas	0.2	0.0	0.4
Sergipe	0.1	0.0	0.2
Bahia	0.1	0.0	0.2
**Southeast**	**0.7**	**0.5**	**1.0**
Minas Gerais	0.2	0.0	0.3
Espírito Santo	0.4	0.0	0.8
Rio de Janeiro	0.2	0.0	0.3
São Paulo	1.3	0.8	1.7
South	1.1	0.7	1.5
Paraná	2.1	1.2	3.0
Santa Catarina	1.0	0.5	1.6
Rio Grande do Sul	0.2	0.0	0.3
**West-Central**	**1.5**	**1.1**	**1.8**
Mato Grosso do Sul	2.1	1.2	3.0
Mato Grosso	0.4	0.2	0.7
Goiás	1.4	0.7	2.0
Distrito Federal	2.2	1.4	2.9

*Source: PNS 2019. ^13^*

**Chart 11-1:** – Current smoking prevalence trends according to the GBD 2017 estimates and Brazilian surveys (crude values) of individuals aged 20 years and more, Brazil, 1989 to 2017.

Source: Malta DC et al. ^400^

**Chart 11-2:** – Trend of smoking prevalence, by sex, according to the Vigitel data, in Brazilian capitals, between 2006 and 2019.

Source: Vigitel – 2006 to 2019. ^276^

**Chart 11-3:** – Mortality rate and age-standardized mortality rate, by sex, absolute number of deaths associated with tobacco use, and mortality rates (all ages and age-standardized), from 1990 to 2019.

Source: Data derived from Global Burden of Disease Study 2019, Institute for Health Metrics and Evaluation, University of Washington. ^46^

**Chart 11-4:** – Proportional mortality attributable to tobacco by age group and sex, Brazil, 2019

Source: Data derived from Global Burden of Disease Study 2019, Institute for Health Metrics and Evaluation, University of Washington. ^46^

**Chart 11-5:** – Absolute number of deaths attributed to tobacco and secondhand smoke, by all causes of deaths, for both sexes, 2019.

Source: Data derived from Global Burden of Disease Study 2019, Institute for Health Metrics and Evaluation, University of Washington. ^46^

**Chart 11-6:** – Age-standardized mortality rates due to smoking and secondhand smoke for both sexes, by all causes of death, Brazil, 1990 and 2019.

Source: Data derived from Global Burden of Disease Study 2019, Institute for Health Metrics and Evaluation, University of Washington. ^46^

**Chart 11-7:** – Total number of deaths due to cardiovascular diseases attributed to tobacco and secondhand smoke, for both sexes. Each color represents a specific cause of cardiovascular death, 1990.

Source: Data derived from Global Burden of Disease Study 2019, Institute for Health Metrics and Evaluation, University of Washington. ^46^

**Chart 11-8:** – Total number of deaths due to cardiovascular diseases attributed to tobacco and secondhand smoke, for both sexes. Each color represents a specific cause of cardiovascular death, 2019.

Source: Data derived from Global Burden of Disease Study 2019, Institute for Health Metrics and Evaluation, University of Washington. ^46^

**Chart 11-10:** – Time trend of the rates of YLLs due to tobacco use from 1990 to 2019.

Source: Data derived from Global Burden of Disease Study 2019, Institute for Health Metrics and Evaluation, University of Washington. ^46^

**Chart 11-9:** – Correlation between the 2019 Sociodemographic Index (SDI) and percent change of mortality rates attributable to smoking between 1990 and 2019, in Brazil.

Source: Data derived from Global Burden of Disease Study 2019, Institute for Health Metrics and Evaluation, University of Washington. ^46^

**Chart 11-11:** – Time trend of the rates of YLDs due to tobacco use from 1990 to 2019.

Source: Data derived from Global Burden of Disease Study 2019, Institute for Health Metrics and Evaluation, University of Washington. ^46^

**Chart 11-12:** – Time trend of the rates of DALYs due to tobacco use from 1990 to 2019.

Source: Data derived from Global Burden of Disease Study 2019, Institute for Health Metrics and Evaluation, University of Washington. ^46^

### Overview

#### Definitions

• Current smoker: An adult who has smoked cigarettes in his or her lifetime and who currently smokes cigarettes. ^19^ ^,^ ^400^ Adolescents who smoked at least one day in past 30 days were considered current cigarette smokers. ^401^

• Secondhand smoke: Also called passive smoking. Usually refers to cigarette smoke in the environment of a nonsmoker. ^400^

• Former (past) smoker: An adult who has smoked in his or her lifetime but who had quit smoking at the time of interview. ^400^

• Experimenting with tobacco use: Defined as having tried cigarettes at least once in life. ^401^

• Electronic smoking devices, more commonly called electronic cigarettes or e-cigarettes, are battery-operated devices that provide nicotine, flavors, and other chemicals to the user in an aerosol. ^3^ ^,^ ^402^ The liquids used in these devices can be quite different in terms of chemical composition, nicotine concentration, and additives used, and more than 8000 flavors of electronic cigarettes have been described. Discrepancy between the composition declared on the packaging and the actual composition of the product has been shown.

• The SDI is a composite index that measures per capita income, fertility, and education. It mirrors sociodemographic development. The SDI allows you to compare states and countries according to their development. ^403^

#### Tobacco and total CVD

• Tobacco use is one of the main preventable causes of death in Brazil and worldwide. ^19^ ^,^ ^400^ Tobacco is one of the main risk factors for NCDs, such as CVD, cancer, chronic respiratory diseases, intrauterine growth restriction, and predisposition to premature birth. ^3^ The negative impact of tobacco on health results from both the direct consumption of various forms of tobacco products (smoked, inhaled or chewed) and the exposure to secondhand smoke. ^400^ ^,^ ^404^

• Tobacco is an independent risk factor for CVD and multiplies the risk when associated with other risk factors, such as hypertension, dyslipidemia, and diabetes mellitus. ^405^ There is a marked increase in the risk of CVD even at low levels of tobacco exposure, including secondhand smoke and cigar smoking. In addition, the risk grows, but to a lesser extent, with the increase in the number of cigarettes per day. ^34^

• Current or past smoking is associated with an increased risk of heart failure with reduced and preserved ejection fraction. ^406^ ^,^ ^407^

• Tobacco consumption through different forms of smoking is associated with an increased risk for cardiovascular events, with an OR of 1.67 (95% CI, 1.25 - 2.24) for the association of ischemic heart disease and heart failure with waterpipe smoking. ^408^

• Non-smokers exposed to secondhand smoke at home or at work have their risk of developing CVD increased by 25% to 30%. ^4^ ^,^ ^8^ Exposure to secondhand smoke increases the risk of stroke by 20% to 30% and is associated with increased mortality after the event. ^3^ ^,^ ^405^

#### Measurements

• The prevalence of smoking will be addressed according to the most recent population surveys in Brazil: the ELSA-Brasil Study; the PNS 2019 (containing estimates for the population aged 18 years and over); the PeNSE 2015 Survey, containing estimates for teenagers aged 13 to 15 years; the ERICA Study, which included 74 589 adolescents aged 12 to 17 years, from municipalities with over 100 000 inhabitants; and trend estimates for adults in the Brazilian capitals between 2006 and 2019. ^401^ ^,^ ^409^ ^,^ ^410^

• Mortality rates and adjusted hazard ratios of the association between smoking and death are shown in Brazilian cohorts. ^19^ ^,^ ^404^

• Mortality rates and absolute numbers of deaths attributable to tobacco will be presented for Brazil and its 27 FUs in 1990 and 2019. Estimates are from the GBD Study 2019. ^19^

## Prevalence

### Prevalence of tobacco use among adolescents

• Tables 11-1 to 11-3 show the prevalence of smoking indicators among adolescents of the ninth grade, aged 13 to 15 years, according to data from the PeNSE 2015 survey. ^410^

• In addition, according to data from the PeNSE 2015 survey, 18.4% (95% CI, 17.8 - 19) of the adolescents aged 13 to 15 years have already tried cigarettes in their lifetime. The prevalence was higher among males (19.4%; 95% CI, 16.6 - 18.2) than among females (17.4%; 95% CI, 18.7 - 20.0). The share of adolescents who tried smoking ranged from 27.0% in Mato Grosso do Sul to 9.3% in Sergipe, being highest in the Southern (24.9%) and lowest in the Northeastern region. ^410^

• The prevalence of current smoking or having smoked in the 30 days prior to the survey was 5.6% (95% CI, 5.3 - 5.9), with no difference according to sex (male 5.8%; 95% CI, 5.4 - 6.3; female 5.4%; 95% CI, 4.9 - 5.8). The use of other tobacco products was 6.1% (95% CI, 5.7 - 6.4) among male adolescents and 5.6% (95% CI, 5.1 - 6.0) among females. ^410^

• The ERICA study, a cross-sectional, nationwide, school-based study of cardiovascular risks, included 74 589 adolescents aged 12 to 17 years, from municipalities with over 100 000 inhabitants. The results showed that 18.5% (95% CI, 17.7-19.4) of the adolescents had smoked at least once in life, 5.7% (95% CI, 5.3 - 6.2) smoked at the time of the research, and 2.5% (95% CI, 2.2 - 2.8) smoked often, without significant difference between sexes. ^401^

• In the Southern region, there was a higher prevalence of experimenting with tobacco use (23.3%; 95% CI, 21.5 - 25.3), current tobacco use (7.3%; 95% CI, 6.2 - 8.7), and tobacco use for 7 consecutive days (3.8%; 95% CI, 2.8 - 5.1) as compared to the Northern (19.2%, 5.9%, and 2.2%, respectively) and Northeastern regions (15.2%, 4.7%, and 1.5%, respectively).

• There was no statistically significant difference in the prevalence of tobacco use according to socioeconomic indicators (reported ethnicity, education level, mother’s education level, and father’s education level) for both sexes.

• The prevalence was higher among adolescents who had paid jobs in the year prior to the study, for both sexes [9.3% (95% CI, 8.1 - 10.5) vs 5.0% (95% CI, 4.3-5.6) for males; 8.8% (95% CI, 7.5 - 10.1) vs 4.6% (95% CI, 4.0-5.1) for females]. In addition, there was a higher prevalence among those who did not live with both parents as compared to those who did [8.0% (95% CI, 6.8 - 9.2) vs 4.8% (95% CI, 4.0-5.5) for males; 6.4% (95% CI, 5.7 – 7.1) vs 4.4% (95% CI, 3.8 - 5.1) for females].

• Females attending public schools reported smoking more often [5.7% (95% CI, 5.1 - 6.2)] than those from private schools [3.7% (95% CI, 2.3 - 5.1)]. In the male sex, the difference was not significant, 6.1% (95% CI, 5.6 - 6.9) and 5.2% (95% CI, 3.5 - 7.0), respectively.

• Adolescents having the presence of smokers at home had a higher prevalence of smoking, 8.1% (95% CI, 6.7 - 9.6) vs no presence, 5.4% (95% CI, 4.7 - 6.1), for males, and 7.1% (95% CI, 6.2 - 8.0) vs no presence, 4.5% (95% CI, 4.0 - 5.1), for females. Furthermore, a higher prevalence was seen in those in contact with smokers outside home: males, 9.9% (95% CI, 8.6 - 11.1) vs 3.6% (95% CI, 3.0 - 4.3); and females, 7.6% (95% CI, 6.8 - 8.4) vs 2.7% (95% CI, 2.2 - 3.3).

### Prevalence of tobacco use among adults

• PNS 2019 data, identified in Table 11-4, indicate that 12.8% (95% CI, 12.4 - 13.2) of adults use some tobacco product, being the use higher among males (16.2%; 95% CI, 15.6 - 16.9), than among females (9.9%; 95% CI, 9.3 - 10.3). ^306^

• The prevalence of some tobacco product use is higher in the rural area (14.3%) than in the urban area (12.6%), as well as higher in the Southern (14.7%) than in the Northern (10.7%) region.

• The proportion of tobacco smokers over 18 years of age in 2019 was 12.6% (95% CI, 12.2 - 13.0), and, according to sex, 15.9% (95% CI, 15.3 - 16.6) of males and 9.6% (95% CI, 9.2 - 10.1) of females (Table 11-1). ^306^ Regarding age groups, the prevalence was as follows, from the highest to the lowest: 14.9% (95% CI, 14.2 - 15.5) in the 40-59 years group; 10.8% (95% CI, 9.6 - 12.0) in the 18-24 years group; 12.0% (95% CI, 11.2 - 12.7) in the 25-39 years group; and 11.9% (95% CI, 11.2 - 12.6) in the 60 years or older group (Table 11-5). ^306^

• The prevalence is lower in populations with higher education and income, being 17.6% (95% CI, 16.8 - 18.4) among those without education or with incomplete primary education, 15.5% (95% CI, 14.3 - 16.6) among those with complete primary education and incomplete secondary education, 9.6% (95% CI, 8.9 - 10.2) among individuals with complete secondary education and incomplete college education, and 7.1% (95% CI, 6.3 - 7.8) among those with complete college education (Table 11-7). ^306^

• The prevalence of smoking was higher among black individuals (13.7%; 95% CI, 12.5 - 15) as compared to “pardos” or mixed race involving African ancestry (13.5%; 95% CI, 12.9 - 14.2) and white individuals (11.8%; 95% CI, 11.2 - 12.4) (Table 11-8). ^306^

• The Vigitel 2019 showed a frequency of adult smokers of 9.8%, higher in males (12.3%) than in females (7.7%). In the total population, the frequency of smokers tended to be lower among young adults (less than 25 years of age) and among those aged 65 years and over. The frequency of smoking decreased with increasing schooling, and was particularly high among men with up to 8 years of schooling (16.8%). ^410^

• According to the GBD Study 2019, in Brazil, the prevalence of secondhand smoke at home is 9.2% (95% UI, 8.8 - 9.8%), higher among females (10.2%; 95% CI, 8.7 - 10.8%) than among males (7.9%; 95% UI, 7.3 - 8.5%). ^19^

• The prevalence of secondhand smoke at work is 8.4% (95% CI, 8.8 - 9.8), higher among males (10.4%; 95% CI, 9.4 - 11.3) than among females (8.7%; 95% CI, 6.1 - 7.4) (Table 11-12). ^19^

• The use of electronic cigarettes was measured for the first time in the PNS 2019, revealing a prevalence of 0.6%, with greater electronic cigarette use in the Distrito Federal (2.2%) and in the states of Mato Grosso do Sul (2.1%) and Paraná (2.1%) (Table 11-13). ^405^

### Prevalence trend

• The prevalence obtained in the main national household surveys, in the Vigitel telephone survey for participants aged 18 years or older, and in the PNS points to a significant decrease in the prevalence of smoking in the adult population, with a 37.6% reduction from 2006 to 2019. This trend is similar to that found in other national studies (Table 11-9, Chart 11-1). ^306^ ^,^ ^400^ ^,^ ^410^ ^,^ ^411^ However, the index is 0.5% higher than that calculated in 2018.

• According to Vigitel data, in the Brazilian capitals, between 2006 and 2019, there was a decline in smoking for both sexes. After 2015, the decline was smaller, even with stability for males (Table 11-9 and Chart 11-2). ^400^ ^,^ ^411^

• Among adolescents, in comparison with the PeNSE 2012 survey, the prevalence of current smoking remained unchanged at 5.0% (2012) and 5.6% (2015). However, the use of other tobacco products increased from 4.8% (95% CI, 4.6 - 5.0) in 2012 to 6.1% (95% CI, 5.7 - 6.4) in 2015, with a higher proportion among males. Cigarette smoking together with the use of other tobacco products increased by 18%, from 7.6% (2012) to 9.0% (2015). The hookah was the most used product in 2015 (71.6%; 95% CI, 68.8 - 74.2), being more frequent among females (4% among male adolescents and 5.6% among female adolescents). ^405^ ^,^ ^412^

• According to the GBD Study 2019, passive smoking deaths decreased between 1990 and 2019. The main causes of passive smoking death from CVD were ischemic heart disease and stroke. ^19^ In 1990, there were 7489 (5.03 per 100 000 inhabitants) deaths from ischemic heart disease due to secondhand smoke and, in 2019, there were 6934 deaths (3.2 per 100 000 inhabitants). ^19^ In 1990, there were 4400 (2.96 per 100 000 inhabitants) deaths from stroke due to secondhand smoke and, in 2019, it decreased to 3219 (1.3 per 100 000 inhabitants). ^19^

## Mortality

### Tobacco and total mortality

•Table 11-10 shows the age-standardized rate of deaths (per 100 000 inhabitants) attributed to tobacco, and the percent change of rates by sex between 1990 and 2019, in Brazil and each FU. Table 11-11 shows the number and age-standardized rate of deaths attributed to tobacco and percent change of rates. These data were estimated by the GBD considering the existing literature and the population attributable fraction due to smoking, that is, the proportion of cases attributed to tobacco use (Chart 11-3). ^19^

• According to GBD data in 1990, the absolute number of deaths attributed to tobacco was 168 443 (95% UI, 159 638 – 176 773) and in 2019 it increased to 191 127 (95% UI, 180 887 – 202 595) deaths (Table 11-11). ^19^

• The absolute number of deaths attributed to tobacco increased, mainly due to population aging and growth; however, there was a reduction by 58.8% (95% UI, 56.3 - 61.1) in mortality rates attributable to smoking, from 199.9 per 100 000 inhabitants (95% UI, 189.1 - 210.6) in 1990 to 82.4 per 100 000 inhabitants (95% UI, 77.9 - 87.5) in 2019 (Table 11-11). This reduction occurred due to the reduction in risk or the prevalence of smokers. ^19^

• Mortality rates due to tobacco decreased in all Brazilian FUs and, in 2019, the highest mortality rate attributable to smoking was found in the states of Rio Grande do Sul (104.6; 95% UI, 94.4 -115.9), Acre (99.8; 95% UI, 89.7 - 109.8), and Pernambuco (97.7; 95% UI, 87 - 108.7). ^19^

• Chart 11-4 shows the **Proportional mortality** attributable to tobacco by age group. The proportion is highest among men, aged 60 to 74 years, male.

• Chart 11-5 shows the absolute numbers of deaths attributed to tobacco and secondhand smoke. The use of smoked tobacco has contributed to more than 190 000 deaths from NCDs and others, including CVD (65 693), cancer (53 000: lungs, oral cavity, breast), chronic respiratory diseases, infectious respiratory diseases and tuberculosis, diabetes, digestive diseases, kidney diseases (Charts 11-5 and 11-6). ^19^

### Tobacco and CVD mortality

• Analysis of the specific causes of deaths attributable to smoking indicates that CVD mortality decreased from 88.0 per 100 000 inhabitants (95% UI, 81.3 - 94.3) in 1990 to 26.3 per 100 000 inhabitants (95% CI, 23.8 - 28.9) in 2019. ^19^

• In 2019, smoking was responsible for 65 696 deaths from CVD. In addition, there was a decrease in the mortality rates of some CVDs attributable to smoking, such as ischemic heart disease and stroke, in the studied period (Charts 11-7 and 11-8). ^19^ Among CVDs, the greatest reduction in mortality attributed to smoking was observed for ischemic heart disease. There was a reduction from 41 564 deaths [47.2 (95% UI, 43.8 - 50.4) per 100 000 inhabitants] in 1990 to 35 218 deaths [14.7 (95% UI, 16.0 - 13.5) per 100 000 inhabitants] in 2019. ^19^

• Stroke deaths decreased from 28 468 [31.8 (95% UI, 29.0 - 34.8) per 100 000 inhabitants] in 1990 to 17 577 [7.4 (95% UI, 6.6 - 8.3) per 100 000 inhabitants] in 2019. The deaths from aortic aneurysm increased from 1678 [7.4 (95% UI, 1.7 - 2.0) per 100 000 inhabitants] in 1990 to 3999 [1.7 (95% UI, 1.5 - 1.9) per 100 000 inhabitants] in 2019, and the deaths from peripheral arterial diseases increased from 343 [0.4 (95% UI, 0.2 - 0.8) per 100 000 inhabitants] in 1990 to 674 [0.3 (95% UI, 0.1 - 0.6) per 100 000 inhabitants] in 2019. ^19^

• Overall, larger reductions in smoking-attributable mortality rates were observed in the FUs with higher SDI, with the highest declines observed in those with high SDI (Distrito Federal, Rio de Janeiro, São Paulo, Santa Catarina, Paraná, Minas Gerais) and the smallest reductions in the UFs of the Northern and Northeastern regions of Brazil, with lower SDIs (Rio Grande do Norte, Ceará, Bahia, Pará, Paraíba) (Pearson correlation: 0.637; p < 0.001) (Chart 11-9). ^34^

## Burden of Disease

### Tobacco total burden of disease

• Charts 11-10, 11-11 and 11-12 show the trends from 1990 to 2019 for the metrics YLLs, YLDs and DALYs. In all situations, crude and age-standardized rates declined. ^19^

• GBD 2019 data estimated a reduction in the age-standardized DALY rate attributable to tobacco per 100 000 inhabitants in Brazil by 59% (95% UI, 56% - 61%) between 1990 [4614.5 (95% UI, 4372.3 - 4888.0)] and 2019 [1893.7 (95% UI, 1768.6 - 2028.0)]. The differences in the curves in Chart 11-12 reflect DALYs adjusted for differences in the age distribution of the population, with the ‘all ages’ curve driven mainly by a combination of population growth and aging.

• The reduction in DALY rates is a consequence of the decrease in YLLs by 61% (95% UI, 58 - 63%) between 1990 and 2019. In both instances, a combination of reduced smoking exposure and reduced risk-deleted DALY rates contributed to overall reductions.

• There was a heterogeneous reduction in age-standardized DALYs attributed to tobacco in the different FUs and regions of Brazil, more pronounced in the Southeastern, Southern and West-Central FUs, with a modest reduction in the Northern FUs and an even more discreet reduction in most Northeastern FUs.

### Tobacco cardiovascular burden of disease

• GBD 2019 data estimated age-standardized DALY rates attributable to tobacco per 100 000 inhabitants in Brazil of 650 (95% UI, 604 - 701), a reduction of 69% (95% UI, 67 - 71) as compared to the 1990 age-standardized DALY rate, 2124 (95% UI, 1993 - 2254).

• The age-standardized YLL rates per 100 000 inhabitants were 2040 (95% UI, 1919 - 2164) in 1990 and 611 (95% UI, 568 - 657) in 2019, a reduction of 70% (95% UI, 68% - 72%) in the period.

• The age-standardized YLD rates per 100 000 inhabitants were 84 (95% UI, 60 - 108) in 1990 and 39 (95% UI, 28 - 51) in 2019, a reduction of 54% (95% UI, 50-57%).

### Economic Impact of Tobacco

In Brazil, the economic impact of tobacco, including the direct cost (costs of diagnosis, treatment, and follow-up), was recently estimated using a developed probabilistic microsimulation economic model of the natural history, medical costs, and quality-of-life losses associated with the most common tobacco-related diseases. Data inputs were obtained through literature review, vital statistics, and hospital databases. Chronic obstructive pulmonary diseases, CVD, lung cancer, and stroke accounted for 78% of that cost. In 2015, the total direct cost of tobacco was estimated at US$ 11.8 billion per year, 70% corresponding to the direct cost associated with health care and the remainder associated with the indirect cost due to the loss of productivity due to premature death and inability. Tobacco represented 22% of the direct costs of CVD in Brazil and 17% of the direct costs of stroke. The cost attributed to secondhand smoke was US$ 1.36 billion. ^413^ ^,^ ^414^

The health costs attributed to the use of tobacco represent an estimate of 5.7% of all government spending on health and 0.7% of the gross domestic product. It is estimated that, in Brazil, 25.6% of the resources spent are recovered through taxes on tobacco. ^413^ ^,^ ^414^

### Future Research

Due to the lack of longitudinal data, the long-term CVD risks associated with e-cigarette use are unknown. Limited information on the health risks of electronic tobacco products is currently available. More research and extended follow-up are still necessary because e-cigarette, including electronic hookah use, have become prevalent among youth.

Finally, despite the positive results of the tobacco control program, advances in tobacco control are still necessary in the country. The following steps include regulatory measures (price/taxation increase, warning spaces on the packaging, adoption of plain packaging), social measures (support for small farmers to move forward from replacing tobacco cultivation with other crops), and legal actions (supervision of tobacco products, border control and combat of illegal trade, support policies to create tobacco control funds to compensate for smoking-related healthcare costs at federal and subnational level).
